# Global burden of chronic respiratory diseases and risk factors, 1990–2019: an update from the Global Burden of Disease Study 2019

**DOI:** 10.1016/j.eclinm.2023.101936

**Published:** 2023-05

**Authors:** GBD 2019 Chronic Respiratory Diseases Collaborators, Sara Momtazmanesh, Sahar Saeedi Moghaddam, Seyyed-Hadi Ghamari, Elaheh Malakan Rad, Negar Rezaei, Parnian Shobeiri, Amirali Aali, Mohsen Abbasi-Kangevari, Zeinab Abbasi-Kangevari, Michael Abdelmasseh, Meriem Abdoun, Deldar Morad Abdulah, Abu Yousuf Md Abdullah, Aidin Abedi, Hassan Abolhassani, Zahra Abrehdari-Tafreshi, Basavaprabhu Achappa, Denberu Eshetie Adane Adane, Tigist Demssew Adane, Isaac Yeboah Addo, Mohammad Adnan, Qor-inah Estiningtyas Sakilah Adnani, Sajjad Ahmad, Ali Ahmadi, Keivan Ahmadi, Ali Ahmed, Ayman Ahmed, Tarik Ahmed Rashid, Hanadi Al Hamad, Fares Alahdab, Astawus Alemayehu, Sheikh Mohammad Alif, Syed Mohamed Aljunid, Sami Almustanyir, Khalid A Altirkawi, Nelson Alvis-Guzman, Javad Aminian Dehkordi, Mehrdad Amir-Behghadami, Robert Ancuceanu, Catalina Liliana Andrei, Tudorel Andrei, Catherine M Antony, Anayochukwu Edward Anyasodor, Jalal Arabloo, Judie Aru-lappan, Tahira Ashraf, Seyyed Shamsadin Athari, Engi F Attia, Meshe-sha Tsegazeab Ayele, Sina Azadnajafabad, Abraham Samuel Babu, Sara Bagherieh, Ovidiu Constantin Baltatu, Maciej Banach, Mainak Bardhan, Francesco Barone-Adesi, Amadou Barrow, Saurav Basu, Nebiyou Simegnew Bayileyegn, Isabela M Bensenor, Nikha Bhardwaj, Pankaj Bhardwaj, Ajay Nagesh Bhat, Krittika Bhattacharyya, Souad Bouaoud, Dejana Braithwaite, Michael Brauer, Muhammad Hammad Butt, Zahid A Butt, Daniela Calina, Luis Alberto Cámera, Gashaw Sisay Chanie, Periklis Charalampous, Vijay Kumar Chattu, Odgerel Chimed-Ochir, Dinh-Toi Chu, Aaron J Cohen, Natália Cruz-Martins, Omid Dadras, Aso Mohammad Darwesh, Saswati Das, Sisay Abebe Debela, Laura Delgado-Ortiz, Diriba Dereje, Mostafa Dianatinasab, Nancy Diao, Daniel Diaz, Lankamo Ena Digesa, Gebisa Dirirsa, Paul Narh Doku, Deepa Dongarwar, Abdel Douiri, Haneil Larson Dsouza, Ebrahim Eini, Michael Ekholuenetale, Temitope Cyrus Ekundayo, Ahmed Elabbas Mustafa Elagali, Muhammed Elhadi, Daniel Berhanie Enyew, Ryen-chindorj Erkhembayar, Farshid Etaee, Adeniyi Francis Fagbamigbe, Andre Faro, Ali Fatehizadeh, Ginenus Fekadu, Irina Filip, Florian Fischer, Masoud Foroutan, Richard Charles Franklin, Peter Andras Gaal, Santosh Gaihre, Abduzhappar Gaipov, Mesfin Gebrehiwot, Urge Gerema, Motuma Erena Getachew, Tamiru Getachew, Mansour Gha-fourifard, Reza Ghanbari, Ahmad Ghashghaee, Ali Gholami, Artyom Urievich Gil, Mahaveer Golechha, Pouya Goleij, Davide Golinelli, Habtamu Alganeh Guadie, Bhawna Gupta, Sapna Gupta, Veer Bala Gupta, Vivek Kumar Gupta, Mostafa Hadei, Rabih Halwani, Asif Hanif, Arief Hargono, Mehdi Harorani, Risky Kusuma Hartono, Hamidreza Hasani, Abdiwahab Hashi, Simon I Hay, Mohammad Heidari, Merel E Hellemons, Claudiu Herteliu, Ramesh Holla, Nobuyuki Horita, Mohammad Hoseini, Mehdi Hosseinzadeh, Junjie Huang, Salman Hussain, Bing-Fang Hwang, Ivo Iavicoli, Segun Emmanuel Ibitoye, Sufyan Ibrahim, Olayinka Stephen Ilesanmi, Irena M Ilic, Milena D Ilic, Mustapha Immurana, Nahlah Elkudssiah Ismail, Linda Merin J, Mihajlo Jakovljevic, Elham Jamshidi, Manthan Dilipkumar Janodia, Tahereh Javaheri, Sathish Kumar Jayapal, Shubha Jayaram, Ravi Prakash Jha, Olatunji Johnson, Tamas Joo, Nitin Joseph, Jacek Jerzy Jozwiak, Vaishali K, Billingsley Kaambwa, Zubair Kabir, Laleh R Kalankesh, Rohollah Kalhor, Himal Kandel, Shama D Karanth, Ibraheem M Karaye, Bekalu Getnet Kassa, Gizat M Kassie, Leila Keikavoosi-Arani, Mohammad Keykhaei, Himanshu Khajuria, Imteyaz A Khan, Moien AB Khan, Yusra H Khan, Haneen Khreis, Min Seo Kim, Adnan Kisa, Sezer Kisa, Luke D Knibbs, Pavel Kolkhir, Somayeh Komaki, Farzad Kompani, Hamid Reza Koohestani, Ali Koolivand, Oleksii Korzh, Ai Koyanagi, Kewal Krishan, Kris J Krohn, Naveen Kumar, Nithin Kumar, Om P Kurmi, Ambily Kuttikkattu, Carlo La Vecchia, Judit Lám, Qing Lan, Savita Lasrado, Kamaluddin Latief, Paolo Lauriola, Sang-woong Lee, Yo Han Lee, Samson Mideksa Legesse, Jacopo Lenzi, Ming-Chieh Li, Ro-Ting Lin, Gang Liu, Wei Liu, Chun-Han Lo, László Lorenzovici, Yifei Lu, Soun-darya Mahalingam, Elham Mahmoudi, Narayan B Mahotra, Mohammad-Reza Malekpour, Ahmad Azam Malik, Tauqeer Hussain Mallhi, Deborah Carvalho Malta, Borhan Mansouri, Elezebeth Mathews, Sazan Qadir Maulud, Enkeleint A Mechili, Entezar Mehrabi Nasab, Ritesh G Menezes, Dechasa Adare Mengistu, Alexios-Fotios A Mentis, Mahboo-beh Meshkat, Tomislav Mestrovic, Ana Carolina Micheletti Gomide Nogueira de Sá, Erkin M Mirrakhimov, Awoke Misganaw, Prasanna Mithra, Javad Moghadasi, Esmaeil Mohammadi, Mokhtar Mohammadi, Marita Mohammadshahi, Shafiu Mohammed, Syam Mohan, Nagab-hishek Moka, Lorenzo Monasta, Mohammad Ali Moni, Md Monir-uzzaman, Fateme Montazeri, Maryam Moradi, Ebrahim Mostafavi, Christine Mpundu-Kaambwa, Efrén Murillo-Zamora, Christopher J L Murray, Tapas Sadasivan Nair, Vinay Nangia, Sreenivas Narasimha Swamy, Aparna Ichalangod Narayana, Zuhair S Natto, Biswa Prakash Nayak, Wogene Wogene Negash, Evangelia Nena, Sandhya Neupane Kandel, Robina Khan Niazi, Antonio Tolentino Nogueira de Sá, Ali Nowroozi, Chimezie Igwegbe Nzoputam, Ogochukwu Janet Nzoputam, Bogdan Oancea, Rahman Md Obaidur, Oluwakemi Ololade Odukoya, Hassan Okati-Aliabad, Akinkunmi Paul Okekunle, Osaretin Christabel Okonji, Andrew T Olagunju, Ahmed Omar Bali, Sergej M Ostojic, PA Mahesh, Alicia Padron-Monedero, Jagadish Rao Padubidri, Mohammad Taha Pahlevan Fallahy, Tamás Palicz, Adrian Pana, Eun-Kee Park, Jay Patel, Rajan Paudel, Uttam Paudel, Paolo Pedersini, Marcos Pereira, Renato B Pereira, Ionela-Roxana Petcu, Majid Pirestani, Maarten J Postma, Akila Prashant, Mohammad Rabiee, Amir Radfar, Sima Rafiei, Fakher Rahim, Mohammad Hifz Ur Rahman, Mosiur Rahman, Muhammad Aziz Rahman, Amir Masoud Rahmani, Shayan Rahmani, Vahid Rahmanian, Prashant Rajput, Juwel Rana, Chythra R Rao, Sowmya J Rao, Sina Rashedi, Mohammad-Mahdi Rashidi, Zubair Ahmed Ratan, David Laith Rawaf, Salman Rawaf, Lal Rawal, Reza Rawassizadeh, Mohammad Sadegh Razeghinia, Elrashdy Moustafa Mohamed Redwan, Maryam Rezaei, Nazila Rezaei, Nima Rezaei, Mohsen Rezaeian, Mónica Rodrigues, Jefferson Antonio Buendia Rodriguez, Leonardo Roever, David Rojas-Rueda, Kristina E Rudd, Aly M A Saad, Siamak Sabour, Basema Saddik, Erfan Sadeghi, Masoumeh Sadeghi, Umar Saeed, Maryam Sahebazzamani, Amirhossein Sahebkar, Harihar Sahoo, Mirza Rizwan Sajid, Sateesh Sakhamuri, Sana Salehi, Abdallah M Samy, Milena M Santric-Milicevic, Bruno Piassi Sao Jose, Brijesh Sathian, Maheswar Satpathy, Ganesh Kumar Saya, Subramanian Senthilkumaran, Allen Seylani, Saeed Shahabi, Masood Ali Shaikh, Mohd Shanawaz, Mohammed Shannawaz, Rahim Ali Sheikhi, Sha-shank Shekhar, Migbar Mekonnen Sibhat, Colin R Simpson, Jasvinder A Singh, Paramdeep Singh, Surjit Singh, Valentin Yurievich Skryabin, Anna Aleksandrovna Skryabina, Mohammad Sadegh Soltani-Zangbar, Suhang Song, Ireneous N Soyiri, Paschalis Steiropoulos, Leo Stockfelt, Jing Sun, Ken Takahashi, Iman M Talaat, Ker-Kan Tan, Nathan Y Tat, Vivian Y Tat, Birhan Tsegaw Taye, Pugazhenthan Thangaraju, Rekha Thapar, Friedrich Thienemann, Amir Tiyuri, Mai Thi Ngoc Tran, Jaya Prasad Tripathy, Lorainne Tudor Car, Biruk Shalmeno Tusa, Irfan Ullah, Sana Ullah, Marco Vacante, Pascual R Valdez, Rohollah Valiza-deh, Job F M van Boven, Tommi Juhani Vasankari, Siavash Vaziri, Francesco S Violante, Bay Vo, Ning Wang, Melissa Y Wei, Ronny Westerman, Nuwan Darshana Wickramasinghe, Suowen Xu, Xiaoyue Xu, Lalit Yadav, Yazachew Yismaw, Dong Keon Yon, Naohiro Yone-moto, Chuanhua Yu, Yong Yu, Ismaeel Yunusa, Mazyar Zahir, Moein Zangiabadian, Zahra Zareshahrabadi, Armin Zarrintan, Mikhail Sergeevich Zastrozhin, Zelalem Banjaw Zegeye, Yunquan Zhang, Mohsen Naghavi, Bagher Larijani, Farshad Farzadfar

**Affiliations:** Non-Communicable Diseases Research Center, Endocrinology and Metabolism Population Sciences Institute, Tehran University of Medical Sciences, Tehran, Iran; School of Medicine, Tehran University of Medical Sciences, Tehran, Iran; Kiel Institute for the World Economy, Kiel, Germany; Non-Communicable Diseases Research Center, Endocrinology and Metabolism Population Sciences Institute, Tehran University of Medical Sciences, Tehran, Iran; Non-Communicable Diseases Research Center, Endocrinology and Metabolism Population Sciences Institute, Tehran University of Medical Sciences, Tehran, Iran; Social Determinants of Health Research Center, Shahid Beheshti University of Medical Sciences, Tehran, Iran; Department of Pediatric Cardiology, Tehran University of Medical Sciences, Tehran, Iran; Non-Communicable Diseases Research Center, Endocrinology and Metabolism Population Sciences Institute, Tehran University of Medical Sciences, Tehran, Iran; Endocrinology and Metabolism Research Center, Endocrinology and Metabolism Clinical Sciences Institute, Tehran University of Medical Sciences, Tehran, Iran; Non-Communicable Diseases Research Center, Endocrinology and Metabolism Population Sciences Institute, Tehran University of Medical Sciences, Tehran, Iran; School of Medicine, Tehran University of Medical Sciences, Tehran, Iran; Faculty of Medicine Mashhad University of Medical Sciences, Mashhad, Iran; Non-Communicable Diseases Research Center, Endocrinology and Metabolism Population Sciences Institute, Tehran University of Medical Sciences, Tehran, Iran; Non-Communicable Diseases Research Center, Endocrinology and Metabolism Population Sciences Institute, Tehran University of Medical Sciences, Tehran, Iran; Social Determinants of Health Research Center, Shahid Beheshti University of Medical Sciences, Tehran, Iran; Department of Surgery Marshall University, Huntington, WV, USA; Faculty of Medicine University of Setif Algeria, Sétif, Algeria; Community and Maternity Nursing Unit University of Duhok, Duhok, Iraq; School of Planning, Faculty of Environment University of Waterloo, Waterloo, ON, Canada; Department of Neurosurgery University of Southern California, Los Angeles, CA, USA; Keck School of Medicine University of Southern California, Los Angeles, CA, USA; Research Center for Immunodeficiencies, Tehran University of Medical Sciences, Tehran, Iran Department of Biosciences and Nutrition Karolinska University Hospital, Huddinge, Sweden; Cellular and Molecular Biology Department University of Tehran, Tehran, Iran; Department of Internal Medicine Manipal Academy of Higher Education, Mangalore, India; Department of Anesthesia and Critical Care Debre Tabor University, Debre Tabor, Ethiopia; Clinical and psychosocial epidemiology University of Groningen, Groningen, Netherlands; Clinical and psychosocial Epidemiology University of Groningen, Groningen, Netherlands; Centre for Social Research in Health University of New South Wales, Sydney, NSW, Australia; Quality and Systems Performance Unit Cancer Institute NSW, Sydney, NSW, Australia; Department of Neonatology Indiana University Health Ball Memorial Hospital, Muncie, IN, USA; Faculty of Medicine Universitas Padjadjaran (Padjadjaran University), Bandung, Indonesia; Department of Health and Biological Sciences Abasyn University, Peshawar, Pakistan; Department of Epidemiology, Shahid Beheshti University of Medical Sciences, Tehran, Iran; Department of Epidemiology and Biostatistics Shahrekord University of Medical Sciences, Shahrekord, Iran; School of Public Health, Faculty of Medicine Imperial College London, London, UK; Institute of Endemic Diseases University of Khartoum, Khartoum, Sudan; Swiss Tropical and Public Health Institute University of Basel, Basel, Switzerland; School of Pharmacy Monash University, Bandar Sunway, Malaysia; Department of Pharmacy Quaid I Azam University Islamabad, Islamabad, Pakistan; Department of Computer Science and Engineering University of Kurdistan Hewler, Erbil, Iraq; Geriatric and Long Term Care Department Hamad Medical Corpora-tion, Doha, Qatar; Rumailah Hospital Hamad Medical Corporation, Doha, Qatar; Mayo Evidence-based Practice Center Mayo Clinic Foundation for Medical Education and Research, Rochester, MN, USA; Department of Public Health Harar Health Science College, Harar, Ethiopia; Department of Public Health Rift Valley University, Harar, Ethiopia; School of Public Health and Preventive Medicine Monash University, Melbourne, VIC, Australia; Department of Health Policy and Management Kuwait University, Kuwait, Kuwait; International Centre for Casemix and Clinical Coding National University of Malaysia, Bandar Tun Razak, Malaysia; College of Medicine Alfaisal University, Riyadh, Saudi Arabia; Ministry of Health, Riyadh, Saudi Arabia; Pediatric Intensive Care Unit King Saud University, Riyadh, Saudi Arabia; Research Group in Hospital Management and Health Policies Universidad de la Costa (University of the Coast), Barranquilla, Colombia; Research Group in Health Economics University of Cartagena, Cartagena, Colombia; Applied Science and Technology University of California Berkeley, Berkeley, CA, USA; Chemical Engineering Department-Biotechnology group Tarbiat Modares University, Tehran, Iran; Road Traffic Injury Research Center Tabriz University of Medical Sciences, Tabriz, Iran; Department of Health Service Management Iranian Center of Excellence in Health Management, Tabriz, Iran; Faculty of Pharmacy Carol Davila University of Medicine and Pharmacy, Bucharest, Romania; Cardiology Department Carol Davila University of Medicine and Pharmacy, Bucharest, Romania; Department of Statistics and Econometrics Bucharest University of Economic Studies, Bucharest, Romania; Institute for Health Metrics and Evaluation University of Washington, Seattle, WA, USA; School of Dentistry and Medical Sciences Charles Sturt University, Orange, NSW, Australia; Health Management and Economics Research Center Iran University of Medical Sciences, Tehran, Iran; Department of Maternal and Child Health Sultan Qaboos University, Muscat, Oman; University Institute of Radiological Sciences and Medical Imaging Technology The University of Lahore, Lahore, Pakistan; Department of Immunology Zanjan University of Medical Sciences, Zanjan, Iran; Division of Pulmonary, Critical Care, and Sleep Medicine University of Washington, Seattle, WA, USA; Department of Anatomy Arba Minch University, Arba Minch, Ethiopia; Non-Communicable Diseases Research Center, Endocrinology and Metabolism Population Sciences Institute, Tehran University of Medical Sciences, Tehran, Iran; Department of Physiotherapy Manipal Academy of Higher Education, Manipal, India; Department of Medicine University of Melbourne, Melbourne, VIC, Australia; School of Medicine Isfahan University of Medical Sciences, Isfahan, Iran; Department of Pharmacology & Therapeutics Khalifa University, Abu Dhabi, United Arab Emirates; Center of Innovation, Technology and Education (CITE) Anhembi Morumbi University, Sao Jose dos Campos, Brazil; Department of Hypertension Medical University ofLodz, Lodz, Poland; Polish Mothers’ Memorial Hospital Research Institute, Lodz, Poland; Molecular Microbiology and Bacteriology National Institute of Cholera and Enteric Diseases, Kolkata, India; Department of Molecular Microbiology Indian Council of Medical Research, New Delhi, India; Department of Translational Medicine University of Eastern Piedmont, Novara, Italy; Department of Public & Environmental Health University of the Gambia, Brikama, The Gambia; Epidemiology and Disease Control Unit Ministry of Health, Kotu, The Gambia; Department of Academics Indian Institute ofPublic Health, Gurgaon, India; Surgery Department Jimma University, jimma, Ethiopia; Department of Internal Medicine University of São Paulo, São Paulo, Brazil; Department of Anatomy All India Institute of Medical Sciences, Jodhpur, India; Department of Community Medicine and Family Medicine All India Institute of Medical Sciences, Jodhpur, India; School of Public Health All India Institute of Medical Sciences, Jodhpur, India; Department of General Medicine Manipal Academy of Higher Education, Mangalore, India; Department of Statistical and Computational Genomics National Institute of Biomedical Genomics, Kalyani, India; Department of Statistics University of Calcutta, Kolkata, India; Department of Medicine University Ferhat Abbas of Setif, Setif, Algeria; Department of Epidemiology and Preventive Medicine University Hospital Saadna Abdenour, Setif, Algeria; Department of Epidemiology University of Florida, Gainesville, FL, USA; Cancer Population Sciences Program University of Florida Health Cancer Center, Gainesville, FL, USA; Institute for Health Metrics and Evaluation University of Washington, Seattle, WA, USA; School of Population and Public Health University of British Columbia, Vancouver, BC, Canada; Faculty of Pharmacy University ofCentral Punjab, Lahore, Pakistan; School of Public Health and Health Systems University of Waterloo, Waterloo, ON, Canada; Al Shifa School of Public Health Al Shifa Trust Eye Hospital, Rawalpindi, Pakistan; Clinical Pharmacy University of Medicine and Pharmacy of Craiova, Romania, Craiova, Romania; Internal Medicine Department Hospital Italiano de Buenos Aires (Italian Hospital of Buenos Aires), Buenos Aires, Argentina; Board of Directors Argentine Society of Medicine, Buenos Aires, Argentina; Clinical Pharmacy University of Gondar, Gondar, Ethiopia; Department of Public Health Erasmus University Medical Center, Rotterdam, Netherlands; Department of Community Medicine Datta Meghe Institute of Medical Sciences, Sawangi, India; Saveetha Medical College and Hospitals Saveetha University, Chennai, India; Department of Public Health and Health Policy Hiroshima University, Hiroshima, Japan; Center for Biomedicine and Community Health VNU-International School, Hanoi, Viet Nam; Institute for Health Metrics and Evaluation University of Washington, Seattle, WA, USA; Health Effects Institute, Boston, MA, USA; Therapeutic and Diagnostic Technologies Cooperativa de Ensino Superior Politécnico e Universitário (Polytechnic and University Higher Education Cooperative), Gandra, Portugal; Institute for Research and Innovation in Health University of Porto, Porto, Portugal; Section Global Health and Rehabilitation Western Norway University of Applied Sciences, Bergen, Norway; Department of Global Public Health and Primary Care University of Bergen, Bergen, Norway; Department of Information Technology University of Human Development, Sulaymaniyah, Iraq; Department of Biochemistry Ministry of Health and Welfare, New Delhi, India; School of Public Health Salale University, Fiche, Ethiopia; NCDs and Environment Programme ISGlobal Instituto de Salud Global de Barcelona, Barcelona, Spain; Department of Experimental and Health Sciences Pompeu Fabra University, Barcelona, Spain; Department of Biomedical Science Jimma University, Jimma, Ethiopia; Department of Epidemiology Maastricht University, Maastricht, Netherlands; Department of Epidemiology Shiraz University of Medical Sciences, Shiraz, Iran; Department of Environmental Health Harvard University, Boston, MA, USA; Center of Complexity Sciences National Autonomous University of Mexico, Mexico City, Mexico; Faculty of Veterinary Medicine and Zootechnics Autonomous University of Sinaloa, Culiacán Rosales, Mexico; Department of Comprehensive Nursing Arba Minch University, Arba Minch, Ethiopia; Department of Environmental Health Haramaya University, Harar, Ethiopia; School of Nursing and Midwifery University of Cape Coast, Cape Coast, Ghana; Health Science Center University of Texas, Houston, TX, USA; School of Population Health and Environmental Sciences King’s College London, London, UK; Department of Forensic Medicine and Toxicology Manipal Academy of Higher Education, Mangalore, India; Forensic Medicine and Toxicology Kasturba Medical College Mangalore, Mangalore, Dakshina Kannada District, Karnataka State, India; Department of Or-thodontics Ahvaz Jundishapur University of Medical Sciences, Ahvaz, Iran; Department of Epidemiology and Medical Statistics University of Ibadan, Ibadan, Nigeria; Faculty of Public Health University of Ibadan, Ibadan, Nigeria; Department of Biological Sciences University of Medical Sciences, Ondo, Ondo, Nigeria; Minderoo Foundation, Perth, WA, Australia; School of Biological Sciences The University of Western Australia, Crawley, WA, Australia; Faculty of Medicine University of Tripoli, Tripoli, Liby; Department of Health informatics Haramaya University, Harar, Ethiopia; Department of International Cyber Education Mongolian National University of Medical Sciences, Ulaanbaatar, Mongolia; Department of Internal Medicine Yale University, New Haven, CT, USA; Department of Epidemiology and Medical Statistics University of Ibadan, Ibadan, Nigeria; Institute of Applied Health Sciences University of Aberdeen, Aberdeen, UK; Department of Psychology Federal University of Sergipe, São Cristóvão, Brazil; Department of Environmental Health Engineering Isfahan University of Medical Sciences, Isfahan, Iran; School of Pharmac The Chinese University of Hong Kong, Hong Kong, China; Department of Pharmacy Department of Public Health (M E Getachew MPH), Wollega University, Nekemte, Ethiopia; Psychiatry Department Kaiser Permanente, Fontana, CA, USA; School of Health Sciences A.T. Still University, Mesa, AZ, USA; Institute of Public Health Charité Universitätsmedizin Berlin (Charité Medical University Berlin), Berlin, Germany; Department of Medical Parasitology Abadan University of Medical Sciences, Abadan, Iran; Faculty of Medicine Abadan University of Medical Sciences, Abadan, Iran; School of Public Health, Medical, and Veterinary Sciences James Cook University, Douglas, QLD, Australia; Health Services Management Training Centre Semmelweis University, Budapest, Hungary; Department of Applied Social Sciences Sapientia Hungarian University of Transylvania, Târgu-Mureş, Romania; Institute of Applied Health Sciences (IAHS) University of Aberdeen, Coleraine, UK; Department of Medicine Nazarbayev University School of Medicine, Nur-Sultan, Kazakhstan; Department of Environmental Health Wollo University, Dessie, Ethiopia; Department of Public Health Jimma University, Jimma, Oromia, Ethiopia; Department of Public Health Jimma University, Jimma, Ethiopia; Department of Biomedical Science Arba Minch University, Arba Minch, Ethiopia; Department of Medical Surgical Nursing Tabriz University of Medical Sciences, Tabriz, Iran; Department of Environmental Health Engineering Qazvin University of Medical Sciences, Qazvin, Iran; School of Public Health Qazvin University of Medical Sciences, Qazvin, Iran; Department of Epidemiology and Biostatistics Neyshabur University of Medical Sciences, Neyshabur, Iran; Non-Communicable Diseases Research Center Neyshabur University of Medical Sciences, Neyshabur, Iran; NCD Surveillance Unit World Health Organization (WHO), Moscow, Russia; Institute for Leadership and Health Management Moscow Medical Academy, Moscow, Russia; Health Systems and Policy Research Indian Institute of Public Health, Gandhinagar, India; Department of Genetics Sana Institute of Higher Education, Sari, Iran; Department of Biomedical and Neuromotor Sciences University of Bologna, Bologna, Italy; Department of Health Informatics Bahir Dar University, Bahir Dar, Ethiopia; Department of Public Health Torrens University Australia, Melbourne, VIC, Australia; Toxicology Department Shriram Institute for Industrial Research, Delhi, Delhi, India; School of Medicine Deakin University, Geelong, VIC, Australia; Faculty of Medicine Health and Human Sciences Macquarie University, Sydney, NSW, Australia; Institute for Environmental Research, Tehran University of Medical Sciences, Tehran, Iran; Clinical Sciences Department University of Sharjah, Sharjah, United Arab Emirates; College of Medicine University of Sharjah, Sharjah, United Arab Emirates; University Institute of Public Health The University of Lahore, Lahore, Pakistan; Department of Epidemiology Universitas Airlangga (Airlangga University), Surabaya, Indonesia; Department of Nursing Arak University of Medical Sciences, Arak University of Medical Sciences, Iran; Sekolah Tinggi Ilmu Kesehatan Indonesia Maju (Indonesian Advanced College of Health Sciences) Institution of Public Health Sciences, Jakarta, Indonesia; Department of Ophthalmology Iran University of Medical Sciences, Karaj, Iran; Department of Public Health Jigjiga University, Jigjiga, Ethiopia; Institute for Health Metrics and Evaluation University of Washington, Seattle, WA, USA; Department of Health Metrics Sciences, School of Medicine University of Washington, Seattle, WA, USA; Community-Oriented Nursing Midwifery Research Center Shahrekord University of Medical Sciences, Shahrekord, Iran; Department of Pulmonary Medicine Erasmus University Medical Center, Rotterdam, Netherlands; Department of Statistics and Econometrics Bucharest University of Economic Studies, Bucharest, Romania; School of Business London South Bank University, London, UK; Kasturba Medical College, Mangalore Manipal Academy of Higher Education, Manipal, India; Department of Pulmonology Yokohama City University, Yokohama, Japan; National Human Genome Research Institute (NHGRI) National Institutes of Health, Bethesda, MD, USA; Department of Environmental Health Shiraz University of Medical Sciences, Shiraz, Iran; Research Center for Health Sciences, Institute of Health Shiraz University of Medical Sciences, Shiraz, Iran; Department of Computer Science University of Human Development, Sulaymaniyah, Iraq; Institute of Research and Development Duy Tan University, Da Nang, Viet Nam; Jockey Club School of Public Health and Primary Care The Chinese University of Hong Kong, Hong Kong, China; Czech National Centre for Evidence-Based Healthcare and Knowledge Translation Masaryk University, Brno, Czech Republic; Institute of Biostatistics and Analyses Masaryk University, Brno, Czech Republic; Department of Occupational Safety and Health China Medical University, Taichung, Taiwan; Department of Public Health University of Naples Federico II, Naples, Italy; Department of Health Promotion and Education University of Ibadan, Ibadan, Nigeria; Community Medicine Manipal Academy of Higher Education, Manipal, India; Department of Community Medicine University of Ibadan, Ibadan, Nigeria; Department of Community Medicine University College Hospital, Ibadan, Ibadan, Nigeria; Faculty of Medicine University of Belgrade, Belgrade, Serbia; Department of Epidemiology University of Kragujevac, Kragujevac, Serbia; Institute of Health Research University of Health and Allied Sciences, Ho, Ghana; Department of Clinical Pharmacy MAHSA University, Bandar Saujana Putra, Malaysia; Institute of Advanced Manufacturing Technologies Peter the Great St. Petersburg Polytechnic University, St. Petersburg, Russia; Institute of Comparative Economic Studies Hosei University, Tokyo, Japan; Functional Neurosurgery Research Center, Shahid Beheshti University of Medical Sciences, Tehran, Iran; Division of Pulmonary Medicine Lausanne University Hospital (CHUV), Lausanne, Switzerland; Manipal College of Pharmaceutical Sciences Manipal Academy of Higher Education, Manipal, India; Health Informatic Lab Boston University, Boston, MA, USA; Centre of Studies and Research Ministry ofHealth, Muscat, Oman; Department of Biochemistry Government Medical College, Mysuru, India; Department of Community Medicine Dr. Baba Saheb Ambedkar Medical College & Hospital, Delhi, India; Department of Community Medicine Banaras Hindu University, Varanasi, India; Department of Mathematics University of Manchester, Manchester, UK; Health Services Management Training Centre Semmelweis University, Budapest, Hungary; Hungarian Health Management Association Hungarian Health Management Association, Budapest, Hungary; Department of Community Medicine Manipal Academy of Higher Education, Mangalore, India; Department of Family Medicine and Public Health University of Opole, Opole, Poland; Health Economics Unit Flinders University, Adelaide, SA, Australia; College of Medicine and Public Health Flinders University, Adelaide, SA, Australia; School of Public Health University College Cork, Cork, Ireland; Social Determinants of Health Research Center Gonabad University of Medical Sciences, Gonabad, Iran; Institute for Prevention of Non-communicable Diseases Qazvin University of Medical Sciences, Qazvin, Iran; Health Services Management Department Qazvin University of Medical Sciences, Qazvin, Iran; Save Sight Institute University of Sydney, Sydney, NSW, Australia; Sydney Eye Hospital South Eastern Sydney Local Health District, Sydney, NSW, Australia; UF Health Cancer Center University of Florida, Gainesville, FL, USA; School of Health Professions and Human Services Hofstra University, Hempstead, NY, USA; Department of Midwifery Debre Tabor University, Debre Tabor, Ethiopia; Quality Use of Medicines and Pharmacy Research Centre University of South Australia, Adelaide, SA, Australia; Department of Healthcare Services Management Alborz University of Medical Sciences, Karaj, Iran; Non-Communicable Diseases Research Center, Endocrinology and Metabolism Population Sciences Institute, Tehran University of Medical Sciences, Tehran, Iran; Students’ Scientific Research Center (SSRC), Tehran University of Medical Sciences, Tehran, Iran; Amity Institute of Forensic Sciences Amity University, Noida, India; Department of Pediatrics Rutgers University, New Brunswick, NJ, USA; Family Medicine Department United Arab Emirates University, Al Ain, United Arab Emirates; Primary Care Department NHS North West London, London, UK; Clinical Pharmacy Jouf University, Sakaka, Saudi Arabia; MRC Epidemiology Unit University of Cambridge, Cambridge, UK; Texas A&;M Transportation Institute Texas A&;M University, College Station, Texas, USA; Department of Genomics and Digital Health Samsung Advanced Institute for Health Sciences & Technology (SAIHST), Seoul, South Korea; Public Health Center Ministry of Health and Welfare, Wando, South Korea; School of Health Sciences Kristiania University College, Oslo, Norway; Department of International Health and Sustainable Development Tulane University, New Orleans, LA, USA; Department of Nursing and Health Promotion Oslo Metropolitan University, Oslo, Norway; School of Public Health University of Sydney, Sydney, NSW, Australia; Institute for Allergology Charité Universitätsmedizin Berlin (Charité Medical University Berlin), Berlin, Germany; Division of Immune-mediated Skin Diseases First Moscow State Medical University (Sechenov University), Moscow, Russia; Department of Physiology Hamedan University of Medical Sciences, Hamedan, Iran; Children ‘s Medical Center, Tehran University of Medical Sciences, Tehran, Iran; Social Determinants of Health Research Center Saveh University of Medical Sciences, Saveh, Iran; Department of Environmental Health Engineering Arak University of Medical Sciences, Arak, Iran; Department of General Practice – Family Medicine Kharkiv Medical Academy of Postgraduate Education, Kharkiv, Ukraine; Biomedical Research Networking Center for Mental Health Network (CIBERSAM) San Juan de Dios Sanitary Park, Sant Boi de Llobregat, Spain; Catalan Institution for Research and Advanced Studies (ICREA), Barcelona, Spain; Department of Anthropology Panjab University, Chandigarh, India; Institute for Health Metrics and Evaluation University of Washington, Seattle, WA, USA; Department of Community Medicine Manipal Academy of Higher Education, Mangalore, India; Amity Institute of Biotechnology Amity University Rajasthan, Jaipur, India; Department of Community Medicine Manipal Academy of Higher Education, Mangalore, India; Faculty of Health and Life Sciences Coventry University, Coventry, UK; Department of Medicine McMaster University, Hamilton, ON, Canada; Department of Nephrology Pushpagiri Institute of Medical Sciences and Research Centre, Thiruvalla, India; Department of Clinical Sciences and Community Health University of Milan, Milan, Italy; Health Services Management Training Centre Semmelweis University, Budapest, Hungary; Department of Orthodontics & Dentofacial Orthopedics Dr. D. Y. Patil University, Pune, India; NEVES Society for Patient Safety NEVES Society for Patient Safety, Budapest, Hungary; Division of Cancer Epidemiology and Genetics National Cancer Institute, Rockville, MD, USA; Department of Otorhinolaryngology Father Muller Medical College, Mangalore, India; Centre for Family Welfare University of Indonesia, Depok, Indonesia; Global Health and Health Security Taipei Medical University, Taipei, Taiwan; International Society Doctors for the Environment, Arezzo, Italy; Pattern Recognition and Machine Learning Lab Gachon University, Seongnam, South Korea; Department of Preventive Medicine, College of Medicine Korea University, Seoul, South Korea; Knowledge Translation Directorate Ethiopian Public Health Institute, Addis Ababa, Ethiopia; Department of Biomedical and Neuromotor Sciences University of Bologna, Bologna, Italy; Department of Health Promotion and Health Education National Taiwan Normal University, Taipei, Taiwan; College of Public Health China Medical University, Taichung, Taiwan; Asbestos Diseases Research Institute, Concord, NSW, Australia; School of Life Sciences University of Technology Sydney, Ultimo, NSW, Australia; Centre for Inflammation Centenary Institute, Camperdown, NSW, Australia; Institute for Health and Environment Chongqing University of Science and Technology, Chongqing, China; Department of Internal Medicine Kirk Kerkorian School of Medicine at UNLV, LasVegas, NV, USA; Department of Health Economics Syreon Research Romania, TARGU MURES, Romania; Department of Doctoral Studies George Emil Palade University of Medicine, Pharmacy, Science, and Technology from Targu Mures, Tirgu Mures, Romania; Department of Epidemiology University of North Carolina Chapel Hill, Chapel Hill, NC, USA; Department of Pediatrics Manipal Academy of Higher Education, Mangalore, India; Department of Cardiology, Tehran University of Medical Sciences, Tehran, Iran; Department of Clinical Physiology Tribhuvan University, Kathmandu, Nepal; Non-Communicable Diseases Research Center, Endocrinology and Metabolism Population Sciences Institute, Tehran University of Medical Sciences, Tehran, Iran; Rabigh Faculty of Medicine King Abdulaziz University, Jeddah, Saudi Arabia; Department of Clinical Pharmacy Jouf University, Sakaka, Saudi Arabia; Department of Maternal and Child Nursing and Public Health Federal University of Minas Gerais, Belo Horizonte, Brazil; Substance Abuse Prevention Research Center Kermanshah University of Medical Sciences, Kermanshah, Iran; Department of Public Health and Community Medicine Central University of Kerala, Kasaragod, India; College of Education-Department of Biology Sala-haddin University-Erbil, Erbil, Iraq; Department of Healthcare University of Vlora, Vlora city, Albania; Clinic of Social and Family Medicine University of Crete, Heraklion, Greece; Tehran Heart Center, Tehran University of Medical Sciences, Tehran, Iran; Forensic Medicine Division Imam Abdulrahman Bin Faisal University, Dammam, Saudi Arabia; Department of Environmental Health Science Haramaya University, Harar, Ethiopia; International Dx Department BGI Genomics, Copenhagen, Denmark; Department of Biology Isfahan University of Medical Sciences, Isfahan, Iran; Institute for Health Metrics and Evaluation University of Washington, Seattle, WA, USA; University Centre Varazdin University North, Varazdin, Croatia; Department of Maternal and Child Nursing and Public Health Federal University of Minas Gerais, Belo Horizonte, Brazil; Internal Medicine Programme Kyrgyz State Medical Academy, Bishkek, Kyrgyzstan; Department of Atherosclerosis and Coronary Heart Disease National Center of Cardiology and Internal Disease, Bishkek, Kyrgyzstan; Department of Health Metrics Sciences, School of Medicine University of Washington, Seattle, WA, USA; National Data Management Center for Health Ethiopian Public Health Institute, Addis Ababa, Ethiopia; Department of Community Medicine Manipal Academy of Higher Education, Mangalore, India; Department ofHigher Education Management Islamic Azad University, Tehran, Iran; Non-Communicable Diseases Research Center, Endocrinology and Metabolism Population Sciences Institute, Tehran University of Medical Sciences, Tehran, Iran; Department of Information Technology Lebanese French University, Erbil, Iraq; National Institute for Health Research, Tehran University of Medical Sciences, Tehran, Iran; Health Systems and Policy Research Unit Ahmadu Bello University, Zaria, Nigeria; Substance Abuse and Toxicology Research Center Jazan University, Jazan, Saudi Arabia; Center for Transdisciplinary Research Saveetha Institute of Medical and Technical Science, Chennai, India; Oncology Department Appalachian Regional Healthcare, Hazard, KY, USA; Internal Medicine University ofKentucky, Lexington, KY, USA; Clinical Epidemiology and Public Health Research Unit Burlo Garofolo Institute for Maternal and Child Health, Trieste, Italy; School of Health & Rehabilitation Sciences The University of Queensland, Brisbane, QLD, Australia; Mater Research Institute The University of Queensland, Brisbane, QLD, Australia; Non-Communicable Diseases Research Center, Endocrinology and Metabolism Population Sciences Institute, Tehran University of Medical Sciences, Tehran, Iran; School of Medicine, Shahid Beheshti University of Medical Sciences, Tehran, Iran; Department of Epidemiology and Biostatistics Iran University of Medical Sciences, Tehran, Iran; Department of Medicine Stanford University, Palo Alto, CA, USA; Stanford Cardiovascular Institute Stanford University, Palo Alto, CA, USA; Health and Social Care Economics Group Flinders University, Adelaide, SA, Australia; Family Medicine Unit Mexican Institute of Social Security, Colima, Mexico; Postgraduate in Medical Sciences Universidad de Colima, Colima, Mexico; Institute for Health Metrics and Evaluation University of Washington, Seattle, WA, USA; Department of Health Metrics Sciences, School of Medicine University of Washington, Seattle, WA, USA; Health Workforce Department World Health Organisation, Geneva, Switzerland; Suraj Eye Institute, Nagpur, India; Mysore Medical College and Research Institute Government Medical College, Mysore, India; Manipal College of Dental Sciences, Manipal Manipal Academy of Higher Education, Manipal, India; Department of Health Policy and Oral Epidemiology Harvard University, Boston, MA, USA; Department of Dental Public Health King Abdulaziz University, Jeddah, Saudi Arabia; Amity Institute of Forensic Sciences Amity University, Noida, India; Department of Nursing Madda Walabu University, Ginnir, Ethiopia; School of Nursing Madda Walabu University, Goba, Ethiopia; Department of Medicine Democritus University of Thrace, Alexandroupolis, Greece; Estia Health Blakehurst Estia Health, Sydney, NSW, Australia; International Islamic University Islamabad, Islamabad, Pakistan; Departamento de Clínica Médica (Department of Clinical Medicine) Federal University of Minas Gerais, Belo Horizonte, Brazil; School of Medicine, Tehran University of Medical Sciences, Tehran, Iran; Center of Excellence in Reproductive Health Innovation (CERHI) University of Benin, Benin City, Nigeria; Department of Physiology University of Benin, Edo, Nigeria; Department of Physiology Benson Idahosa University, Benin City, Nigeria; Department of Applied Economics and Quantitative Analysis University of Bucharest, Bucharest, Romania; National Institute of Infectious Diseases Center for Surveillance, Immunization, and Epidemiologic Research, Tokyo, Japan; Center for Evidence-Based Medicine and Clinical Research, Dhaka, Bangladesh; Department of Community Health and Primary University of Lagos, Idi Araba, Nigeria; Department of Family and Preventive Medicine University of Utah, Salt Lake City, UT, USA; Health Promotion Research Center Zahedan University of Medical Sciences, Zahedan, Iran; College of Medicine University of Ibadan, Ibadan, Nigeria; Department of Food and Nutrition Seoul National University, Seoul, South Korea; School of Pharmacy University of the Western Cape, Cape Town, South Africa; Department of Psychiatry and Behavioural Neurosciences McMaster University, Hamilton, ON, Canada; Department of Psychiatry University ofLagos, Lagos, Nigeria; Diplomacy and Public Relations Department University of Human Development, Sulaymaniyah, Iraq; Department of Biomedical Sciences University of Novi Sad, Novi Sad, Serbia; Department of Respiratory Medicine Jagadguru Sri Shivarathreeswara Academy of Health Education and Research, Mysore, India; National School of Public Health Institute of Health Carlos III, Madrid, Spain; Department of Forensic Medicine and Toxicology Kasturba Medical College, Mangalore, Manipal Academy of Higher Education, Manipal, India, Mangalore, India; Department of Medicine, Tehran University of Medical Sciences, Tehran, Iran; Health Services Management Training Centre Semmelweis University, Budapest, Hungary; Hungarian Health Management Association Hungarian Health Management Association, Budapest, Hungary; Department of Public Health Babes Bolyai University, Cluj Napoca, Romania; Department of Health Metrics Center for Health Outcomes & Evaluation, Bucharest, Romania; Department of Medical Humanities and Social Medicine Kosin University, Busan, South Korea; Global Health Governance Programme University of Edinburgh, Edinburgh, UK; School of Dentistry University of Leeds, Leeds, UK; Central Department of Public Health Tribhuvan University, Kathmandu, Nepal; Faculty of Humanities and Social Sciences Tribhuvan University, Kathmandu, Nepal; Research Section Nepal Health Research Council, Kathmandu, Nepal; Clinical Research Department IRCCS Fondazione Don Carlo Gnocchi, Milan, Italy; Institute of Collective Health Federal University of Bahia, Salvador, Brazil; Department of Chemistry University of Porto, Porto, Portugal; Department of Statistics and Econometrics Bucharest University of Economic Studies, Bucharest, Romania; Department of Parasitology and Entomology Tarbiat Modares University, Tehran, Iran; University Medical Center Groningen University of Groningen, Groningen, Netherlands; Center of Excellence in Higher Education for Pharmaceutical Care Innovation Universitas Padjadjaran (Padjadjaran University), Bandung, Indonesia; Department of Biochemistry Jagadguru Sri Shivarathreeswara University, Mysuru, India; Biomedical Engineering Department Amirkabir University of Technology, Tehran, Iran; College of Medicine University of Central Florida, Orlando, FL, USA; Social Determinants of Health Research Center Qazvin University of Medical Sciences, Qazvin, Iran; Department of Anesthesia Cihan University of Sulaimaniya, Sulaimaniya, Iraq; Department of Community Medicine Maharishi Markandeshwar Medical College & Hospital, Solan, India; Department of Population Science and Human Resource Development University of Rajshahi, Rajshahi, Bangladesh; School of Nursing and Healthcare Professions Federation University Australia, Berwick, VIC, Australia; School of Nursing and Midwifery La Trobe University, Melbourne, VIC, Australia; School of Medicine, Shahid Beheshti University of Medical Sciences, Tehran, Iran; Future Technology Research Center National Yunlin University of Science and Technology, Yunlin, Taiwan; Non-Communicable Diseases Research Center, Endocrinology and Metabolism Population Sciences Institute, Tehran University of Medical Sciences, Tehran, Iran; Department of Public Health Torbat Jam Faculty of Medical Sciences, Torbat Jam, Iran; Institute of Environment and Sustainable Development Banaras Hindu University, Varanasi, India; Department of Epidemiology, Biostatistics and Occupational Health McGill University, Montreal, QC, Canada; Research and Innovation Division South Asian Institute for Social Transformation (SAIST), Dhaka, Bangladesh; Department of Community Medicine Manipal Academy of Higher Education, Manipal, India; Department of Oral Pathology Sharavathi Dental College and Hospital, Shimogga, India; Non-Communicable Diseases Research Center, Endocrinology and Metabolism Population Sciences Institute, Tehran University of Medical Sciences, Tehran, Iran; Department of Cardiology, Tehran University of Medical Sciences, Tehran, Iran; Non-Communicable Diseases Research Center, Endocrinology and Metabolism Population Sciences Institute, Tehran University of Medical Sciences, Tehran, Iran; Social Determinants of Health Research Center, Shahid Beheshti University of Medical Sciences, Tehran, Iran; Department of Biomedical Engineering Khulna University of Engineering and Technology, Khulna, Bangladesh; School of Health and Society University of Wollongong, Wollongong, NSW, Australia; WHO Collaborating Centre for Public Health Education and Training Imperial College London, London, UK; Inovus Medical, St Helens, UK; Department of Primary Care and Public Health Imperial College London, London, UK; Academic Public Health England Public Health England, London, UK; School of Health, Medical and Applied Sciences CQ University, Sydney, NSW, Australia; Department of Computer Science Boston University, Boston, MA, USA; Department of Immunology and Laboratory Sciences Sirjan School of Medical Sciences, Sirjan, Iran; Department of Immunology Kerman University of Medical Sciences, Kerman, Iran; Department Biological Sciences King Abdulaziz University, Jeddah, Egypt; Department of Protein Research Research and Academic Institution, Alexandria, Egypt; Non-Communicable Diseases Research Center, Endocrinology and Metabolism Population Sciences Institute, Tehran University of Medical Sciences, Tehran, Iran; Medical Toxicology & Drug Abuse Research Center Birjand University of Medical Sciences, Birjand, Iran; Research Center for Immunodeficiencies, Tehran University of Medical Sciences, Tehran, Iran; Network ofImmunity in Infection, Malignancy and Autoimmunity (NIIMA) Universal Scientific Education and Research Network (USERN), Tehran, Iran; Department of Epidemiology and Biostatistics Rafsanjan University of Medical Sciences, Rafsanjan, Iran; Geography/Demography University of Coimbra, Portugal, Coimbra, Portugal; Department ofPharmacology and Toxicology University of Antioquia, Medellin, Colombia; Department of Clinical Research Federal University of Uberlândia, Uberlândia, Brazil; Department of Environmental and Radiological Health Sciences Colorado State University, Fort Collins, CO, USA; Barcelona Institute for Global Health, Barcelona, Spain; Department of Critical Care Medicine University of Pittsburgh, Pittsburgh, PA, USA; Cardiovascular Department Zagazig University-Egypt, Zagazig, Egypt; Department of Epidemiology, Shahid Beheshti University of Medical Sciences, Tehran, Iran; Sharjah Institute for Medical Research University of Sharjah, Sharjah, United Arab Emirates; Research Consultation Center (RCC) Shiraz University of Medical Sciences, Iran; Cardiac Rehabilitation Research Center Isfahan University of Medical Sciences, Isfahan, Iran; International Center of Medical Sciences Research, Islamabad, Pakistan; Department of Pathology and Microbiology Jinnah Medical College, Peshawar, Pakistan; Medical Laboratory Sciences Sirjan School of Medical Sciences, Sirjan, Iran; Department of Medical Biochemistry Rafsanjan University of Medical Sciences, Rafsanjan, Iran; Applied Biomedical Research Center Mashhad University of Medical Sciences, Mashhad, Iran; Biotechnology Research Center Mashhad University of Medical Sciences, Mashhad, Iran; Department of Development Studies International Institute for Population Sciences, Mumbai, India; Department of Statistics University of Gujrat, Pakistan, Gujrat, Pakistan; Clinical Medical Sciences University of the West Indies, St. Augustine, Trinidad and Tobago; Thoracic Department North Central Regional Health Authority, Champ Fleurs, Trinidad and Tobago; Mark and Mary Stevens Neuroimaging and Informatics Institute University of Southern California, Los Angeles, CA, USA; Department of Entomology Ain Shams University, Cairo, Egypt; Faculty of Medicine University of Belgrade, Belgrade, Serbia; School of Public Health and Health Management University of Belgrade, Belgrade, Serbia; Department of Infectious Diseases and Tropical Medicine Federal University of Minas Gerais, Belo Horizonte, Brazil; Geriatric and Long Term Care Department Hamad Medical Corpora-tion, Doha, Qatar; Faculty of Health & Social Sciences Bournemouth University, Bournemouth, UK; UGC Centre of Advanced Study in Psychology Utkal University, Bhubaneswar, India; Udyam-Global Association for Sustainable Development, Bhubaneswar, India; Department of Preventive and Social Medicine Jawaharlal Institute of Postgraduate Medical Education and Research, Puducherry, India; Emergency Department Manian Medical Centre, Erode, India; National Heart, Lung, and Blood Institute National Institute of Health, Rockville, MD, USA; Health Policy Research Center Shiraz University of Medical Sciences, Shiraz, Iran; Independent Consultant, Karachi, Pakistan; Department of Health Education and Promotion Jazan University, Jazan, Saudi Arabia; Amity Institute of Public Health Amity University, Noida, India; Department of Health Care Management Technical University of Berlin, Berlin, Germany; Department of Health in Disasters and Emergencies Shahrekord University of Medical Sciences, Shahrekord, Iran; Department of Cardiovascular Medicine Cleveland Clinic, Cleveland, OH, USA; Department of Pediatrics and Child Health Nursing Dilla University, Dilla, Ethiopia; Usher Institute University of Edinburgh, Edinburgh, UK; School of Health Victoria University of Wellington, Wellington, New Zealand; School of Medicine University of Alabama at Birmingham, Birmingham, AL, USA; Medicine Service US Department ofVeterans Affairs (VA), Birmingham, AL, USA; Department of Radiodiagnosis All India Institute of Medical Sciences, Bathinda, India; Department of Pharmacology All India Institute of Medical Sciences, Jodhpur, India; Clinical Branch Moscow Research and Practical Centre on Addictions, Moscow, Russia; Addiction Psychiatry Department Russian Medical Academy of Continuous Professional Education, Moscow, Russia; Department of Infectious Diseases and Epidemiology Pirogov Russian National Research Medical University, Moscow, Russia; Department of Immunology Tabriz University of Medical Sciences, Tabriz, Iran; Department of Health Policy and Management University of Georgia College of Public Health, Athens, GA, USA; Hull York Medical School University of Hull, Hull City, UK; Department of Medicine Democritus University of Thrace, Alexandroupolis, Greece; Occupational and Environmental Medicine Department University of Gothenburg, Gothenburg, Sweden; School of Medicine Griffith University, Gold Coast, QLD, Australia; School of Computing Sciences University of Technology Sydney, Sydney, NSW, Australia; University of Western Australia, Sydney, NSW, Australia; University of Occupational and Environmental Health, Japan; Clinical Sciences Department University of Sharjah, Sharjah, United Arab Emirates; Pathology Department Alexandria University, Alexandria, Egypt; Department of Surgery National University of Singapore, Singapore, Singapore; Department of Economics Rice University, Houston, TX, USA; Research and Innovation Enventure Medical Innovation, Houston, TX, USA; Department of Pathology University of Texas, Galveston, TX, USA; School of Nursing and Midwifery Debre Berhan University, Debre Berehan, Ethiopia; Department of Pharmacology All India Institute of Medical Sciences, RAIPUR, India; Department of Community Medicine Manipal Academy of Higher Education, Mangalore, India; Department of Medicine University of Cape Town, Cape Town, South Africa; Department of Internal Medicine University of Zürich, Zurich, Switzerland; Department of Epidemiology and Biostatistics Iran University of Medical Sciences, Tehran, Iran; Department of Epidemiology and Biostatistics Birjand University of Medical Sciences, Birjand, Iran; School of Public Health and Social Work Queensland University of Technology, Brisbane, QLD, Australia; Health Informatics Department Hanoi Medical University, Ha Noi, Viet Nam; Department of Community Medicine All India Institute of Medical Sciences, Nagpur, India; Lee Kong Chian School of Medicine Nanyang Technological University, Singapore, Singapore; Department of Epidemiology and Biostatistics Haramaya University, Haramaya, Ethiopia; Department of Life Sciences University of Management and Technology, Lahore, Pakistan; Department of Zoology University of Education, Lahore, Lahore, Pakistan; Division of Science and Technology University of Education, Lahore, Lahore, Pakistan; Department of General Surgery and Medical-Surgical Specialties University of Catania, Catania, Italy; Board of Directors Argentine Society of Medicine, Buenos Aires, Argentina; Velez Sarsfield Hospital, Buenos Aires, Argentina; Urmia University of Medical Sciences, Urmia, Iran; University Medical Center Groningen University of Groningen, Groningen, Netherlands; UKK Institute, Tampere, Finland; Faculty of Medicine and Health Technology Tampere University, Tampere, Finland; Department of Infectious Disease Kermanshah University of Medical Sciences, Kermanshah, Iran; Department of Medical and Surgical Sciences University of Bologna, Bologna, Italy; Occupational Health Unit Sant’Orsola Malpighi Hospital, Bologna, Italy; Faculty of Information Technology HUTECH University, Ho Chi Minh City, Viet Nam; School of Public Health and Social Work Queensland University of Technology, Brisbane, QLD, Australia; National Center for Chronic and Noncommunicable Disease Control and Prevention Chinese Center for Disease Control and Prevention, Beijing, China; Division of General Internal Medicine and Health Services Research University of California Los Angeles, Los Angeles, CA, USA; Department of Medicine Greater Los Angeles Healthcare System, Los Angeles, CA, USA; Competence Center of Mortality-Follow-Up of the German National Cohort Federal Institute for Population Research, Wiesbaden, Germany; Department of community Medicine Rajarata University of Sri Lanka, Anuradhapura, Sri Lanka; School of Population Health University of New South Wales, Sydney, NSW, Australia; Department of Endocrinology University of Science and Technology of China, Hefei, China; Department of Medicine University of Rochester, Rochester, NY, USA; Cardiovascular Program The George Institute for Global Health, Sydney, NSW, Australia; Caring Futures Institute Flinders University, Adelaide, SA, Australia; Research and Development Division The George Institute for Global Health, New Delhi, India; Department of Pharmacology Bahir Dar University, Bahir Dar, Ethiopia; Pharmacy Department Alkan Health Science, Business and Tech-nology College, Bahir Dar, Ethiopia; Department ofPediatrics Kyung Hee University, Seoul, South Korea; Department of Neuro-psychopharmacology National Center of Neurology and Psychiatry, Kodaira, Japan; Department of Public Health Juntendo University, Tokyo, Japan; Department of Epidemiology and Biostatistics Wuhan University, Wuhan, China; School of Public Health and Management Hubei University ofMedicine, Shiyan, China; Department of Clinical Pharmacy and Outcomes Sciences University of South Carolina, Columbia, SC, USA; Urology and Nephrology Research Center, Shahid Beheshti University of Medical Sciences, Tehran, Iran; School of Medicine, Shahid Beheshti University of Medical Sciences, Tehran, Iran; Department of Medical Mycology and Parasitology Shiraz University of Medical Sciences, Shiraz, Iran; Department of Radiology Tabriz University of Medical Sciences, Tabriz, Iran; Addictology Department Russian Medical Academy of Continuous Professional Education, Moscow, Russia; Department of Bioengineering and Therapeutic Sciences University of California San Francisco, San Francisco, CA, USA; Department of Biomedical Science Jimma University, Jimma, Oromia, Ethiopia, Ethiopia; School of Public Health Hubei Province Key Laboratory of Occupational Hazard Identification and Control Wuhan University of Science and Technology, Wuhan, China; Institute for Health Metrics and Evaluation University of Washington, Seattle, WA, USA; Department of Health Metrics Sciences, School of Medicine University of Washington, Seattle, WA, USA; Endocrinology and Metabolism Research Center, Endocrinology and Metabolism Clinical Sciences Institute, Tehran University of Medical Sciences, Tehran, Iran; Non-Communicable Diseases Research Center, Endocrinology and Metabolism Population Sciences Institute, Tehran University of Medical Sciences, Tehran, Iran; Endocrinology and Metabolism Research Center, Endocrinology and Metabolism Clinical Sciences Institute, Tehran University of Medical Sciences, Tehran, Iran

**Keywords:** Asthma, Chronic obstructive pulmonary disease, Epidemiology, Interstitial lung disease, Lung disease, Morbidity, Mortality, Pneumoconiosis, Pulmonary emphysema

## Abstract

**Background:**

Updated data on chronic respiratory diseases (CRDs) are vital in their prevention, control, and treatment in the path to achieving the third UN Sustainable Development Goals (SDGs), a one-third reduction in premature mortality from non-communicable diseases by 2030. We provided global, regional, and national estimates of the burden of CRDs and their attributable risks from 1990 to 2019.

**Methods:**

Using data from the Global Burden of Diseases, Injuries, and Risk Factors Study (GBD) 2019, we estimated mortality, years lived with disability, years of life lost, disability-adjusted life years (DALYs), prevalence, and incidence of CRDs, i.e. chronic obstructive pulmonary disease (COPD), asthma, pneumoconiosis, interstitial lung disease and pulmonary sarcoidosis, and other CRDs, from 1990 to 2019 by sex, age, region, and Socio-demographic Index (SDI) in 204 countries and territories. Deaths and DALYs from CRDs attributable to each risk factor were estimated according to relative risks, risk exposure, and the theoretical minimum risk exposure level input.

**Findings:**

In 2019, CRDs were the third leading cause of death responsible for 4.0 million deaths (95% uncertainty interval 3.6–4.3) with a prevalence of 454.6 million cases (417.4–499.1) globally. While the total deaths and prevalence of CRDs have increased by 28.5% and 39.8%, the age-standardised rates have dropped by 41.7% and 16.9% from 1990 to 2019, respectively. COPD, with 212.3 million (200.4–225.1) prevalent cases, was the primary cause of deaths from CRDs, accounting for 3.3 million (2.9–3.6) deaths. With 262.4 million (224.1–309.5) prevalent cases, asthma had the highest prevalence among CRDs. The age-standardised rates of all burden measures of COPD, asthma, and pneumoconiosis have reduced globally from 1990 to 2019. Nevertheless, the age-standardised rates of incidence and prevalence of interstitial lung disease and pulmonary sarcoidosis have increased throughout this period. Low- and low-middle SDI countries had the highest age-standardised death and DALYs rates while the high SDI quintile had the highest prevalence rate of CRDs. The highest deaths and DALYs from CRDs were attributed to smoking globally, followed by air pollution and occupational risks. Non-optimal temperature and high body-mass index were additional risk factors for COPD and asthma, respectively.

**Interpretation:**

Albeit the age-standardised prevalence, death, and DALYs rates of CRDs have decreased, they still cause a substantial burden and deaths worldwide. The high death and DALYs rates in low and low-middle SDI countries highlights the urgent need for improved preventive, diagnostic, and therapeutic measures. Global strategies for tobacco control, enhancing air quality, reducing occupational hazards, and fostering clean cooking fuels are crucial steps in reducing the burden of CRDs, especially in low- and lower-middle income countries.

## Introduction

Chronic respiratory disease (CRD) is an umbrella term describing conditions affecting the lungs and airways, including chronic obstructive pulmonary disease (COPD), asthma, pneumoconiosis, interstitial lung disease (ILD), and pulmonary sarcoidosis. CRD, being the third leading cause of mortality globally in 2019, is associated with a substantial burden and cost.^1–3^ The sustainable development goal (SDG) target 3.4, defined by the United Nations (UN), is a one-third reduction of premature mortality from non-communicable diseases (NCDs), including CRDs, by 2030.^4^ The World Health Organization (WHO) is the principal coordinating body for the implementation of health-related SDGs, and its strategy for the period 2019–2023 outlines three key goals: one billion more individuals enjoying better health and well-being, universal health coverage, and enhanced protection against health emergencies.^5^ In addition to its efforts in monitoring health-related indicators,^6^ the WHO has also established the global action plan (GAP) for healthy lives and well-being for all (SDG3 GAP) to improve collaboration between the prominent actors in the multilateral system to accelerate progress towards health-related SDGs targets.^7^ While such programmes aim to promote health in all aspects, mitigating endeavors specific to CRDs have been undertaken as well. The WHO Global Alliance against Chronic Respiratory Diseases (GARD),^8^ in addition to focused Global Initiatives for COPD (GOLD)^9^ and Asthma (GINA),^10^ have been established to reduce the burden of CRDs.

The latest report on the global prevalence and attributable health burden of CRDs has been conducted using the Global Burden of Diseases, Injuries, and Risk Factors Study (GBD) 2017.^11^ Newly available data sources, locations, several risk factors, and some analytical changes lead to more precise estimations in the updated GBD 2019. Environmental and occupational risks and smoking are the leading risk factors of CRDs, with various distributions by geographical location, culture, age, and sex. Understanding the trend of these risk factors and identification of the at-risk populations can help policymakers in developing and efficiently targeting risk modification interventions, which can result in reduced disability and premature mortality.

Using the GBD 2019 study, we described the burden of CRDs and attributable risk factors by sex, age, and Socio-demographic Index (SDI) on global, regional, and national levels as well as their trends from 1990 to 2019. This report aims to picture the overview of the current burden of CRDs. We drafted this manuscript as part of the GBD Collaborator Network under the guidance of the GBD protocol. The ultimate objective is to highlight the most prominent risk factors and at-risk populations to help caregivers and policymakers to develop targeted risk reduction measures effectively. This work updates all past GBD estimates of CRDs.^11,12^

## Methods

### Overview

The GBD is an international collaborative effort determining the burden of 369 diseases and injuries and 87 risk factors in 204 countries and territories, which are categorised into 21 regions and seven super-regions, from 1990. The results are available from the GBD online results tool and can be viewed interactively via the GBD compare tool. The detailed process of burden estimation for CRDs and risk factors is previously reported^1,13^ and included in appendix 1. We obtained the data in this study from GBD 2019 public datasets available from http://ghdx.healthdata. org/gbd-results-tool (accessed on July 1st, 2021).

This study follows the Guidelines for Accurate and Transparent Health Estimates Reporting (GATHER) (appendix 1 pp 138–139).

### Case definition

Standard definitions are used for each cause. According to the GOLD classification, COPD is defined as a measurement of <0.7 one second of forceful exhalation/total forced expiration (FEV1/FVC) on spirometry following bronchodilation. Other alternative definitions, including GOLD pre-bronchodilation, Lower Limit of Normal (LLN) post-bronchodilation, LLN pre-bronchodilation, and European Respiratory Society (ERS) guidelines are also included. Pneumoconiosis is defined as a chronic lung disease marked by lung scarring and other interstitial injuries. Pneumoconiosis includes silicosis, asbestosis, coal worker’s pneumoconiosis, and other pneumoconiosis. Asthma is a chronic lung disease marked by spasms in the bronchi usually resulting from an allergic reaction or hypersensitivity and causing difficulty in breathing. We define asthma as a diagnosis established by a physician in addition to wheezing in the past year. The alternative definitions include selfreported asthma in the past year or ever, only a doctor’s diagnosis, or only wheezing in the past year due to exposure to dust and other containments. The American Thoracic Society criteria are used as the standard definition for ILD. ILD and pulmonary sarcoidosis are CRDs that damage lung function and oxygen uptake via inflammation and/or scarring. The list of other CRDs and relevant International Classification of Diseases (ICD)-10 and ICD-9 codes are available in appendix 1.

### Fatal estimates

Mortality data for CRDs (the parent cause) were retrieved from vital registries, verbal autopsies (household mortality surveys), and surveillance data. Verbal autopsies data were not incorporated in the fatal estimation of child causes. We pooled and standardised the input data based on different coding systems, representativeness, completeness, age and sex aggregation, and misclassification of maternal and HIV/AIDS deaths. Various linear mixed-effect models and spatiotemporal Gaussian process regression models were created using the Cause of Death Ensemble model (CODEm) framework accounting for location-specific covariates.^1,13^ We used CoDCorrect analysis to adjust and ensure the internal consistency of the results from the CODEm model. Multiplication of the estimated number of deaths by the standard life expectancy at the age of death resulted in years of life lost (YLL).

### Nonfatal estimates

Nonfatal estimates include incidence, prevalence, and years lived with disability (YLD). Input data were obtained from hospital claims, literature identified by a systematic review, population-representative surveys, and medical expenditure panel surveys. Hospital inpatient and insurance data were the primary data sources used for pneumoconiosis and ILD and pulmonary sarcoidosis. After data adjustment, estimation of prevalence and incidence by cause and sequela was performed using DisMod-MR 2.1, a Bayesian meta-regression method, and included incorporation of severity distributions, disability weights, and comorbidity adjustment of the sequela. YLD was estimated by combining prevalence and incidence of causes and sequela with levels of severity related to disability using disability weights while adjusting for comorbidity. Modeling other CRDs together in a DisMod- MR model would not generate reliable estimates of outcome due to the variability of these diseases in their underlying causes, risk factors, and associated health outcomes. The YLD from other CRDs was calculated by multiplying the YLDs/YLLs ratio calculated across the specified CRDs by the YLL estimated for other CRDs.

### Risk estimates

We used the comparative risk assessment (CRA) framework to measure attributable burden, which is the quantity of current burden that would have been reduced in case the past population’s exposure had changed to the theoretical minimum risk exposure level (TMREL).^14,15^ We modeled the attributed burden by (1) estimating the relative risk (RR) of the risk–outcome pairs, (2) exposure estimation, (3) establishing the TMREL, (4) calculating population attributable fraction, (5) estimation of RR-weighted prevalence of exposure (summary exposure value), and (6) aggregating risk factors and accounting for their mediation.

GBD risk factors are classified into a risk hierarchy containing four levels, from Level 1, i.e. general categories (behavioural, environmental/occupational, and metabolic), to level 4, i.e. the most specific (such as ambient particulate matter (PM) pollution).^11^

The risk–outcome pairs were included if convincing or probable evidence was available according to the World Cancer Research Fund grading system. Risk factors for COPD include environmental/occupational risks, i.e. ambient PM pollution, ambient ozone pollution, occupational PM, gases, and fumes, household air pollution from solid fuels, and non-optimal temperature, and behavioural risks, i.e. smoking and secondhand smoke. Asthma risk factors include environmental/occupational risks, i.e. occupational asthmagens, behavioral risks, i.e. smoking, and metabolic risks, i.e. high body-mass index (BMI). Pneumoconiosis risk factors comprise environmental/occupational risks, i.e. occupational exposure to silica, asbestos, and occupational PM, gases, and fumes. All risk factors were reported at the most specific level, except for non-optimal temperature, which is a level 2 risk. No risk factors were included for ILD and pulmonary sarcoidosis, and other CRDs.

### Decomposition analysis

Using decomposition analysis, we estimated the contribution of the age-specific CRD incidence rates changes while controlling for population size, sex distribution, and age structure.^16^ In scenario 1, we accounted for population growth by applying the population size of 2019 onto the rate, sex, and age structure of 1990. The difference between the number of incident cases in 1990 and the estimated numbers in this scenario results only from population growth. In scenario 2, we applied the 1990 age-sex specific rates to the 2019 age-sex specific population numbers to account for both population growth and change in age structure. The difference between the number of incident cases in 2019 and the numbers estimated in the second scenario is due to a change in age-sex specific rates of CRD incidence. We reported the contribution of each factor to the overall change of the new cases as the percent of change (appendix 2 Table S3).

### Socio-demographic Index (SDI)

The SDI, ranging from 0 to 100, indicates socio-demographic development by incorporating lagged distributed income per capita, average years of education, and total fertility rate.^17^ We used the SDI to classify the 204 GBD countries and territories into quintiles.

### Statistical analysis

We calculated age-standardised rates (ASRs) by the GBD global standard population.^17^ Point estimates are presented with 95% uncertainty interval (UI), and rates are reported per 100 000 populations. 95% UIs were estimated using the 25th and 975th ordered values among 1000 draws in each computational stage.

### Role of the funding source

The funders of the study had no role in study design, data collection, data analysis, data interpretation, or the writing of the report. The corresponding author had full access to the data in the study and final responsibility for the decision to submit for publication.

## Results

### Total CRDs

**Fig. 1:**
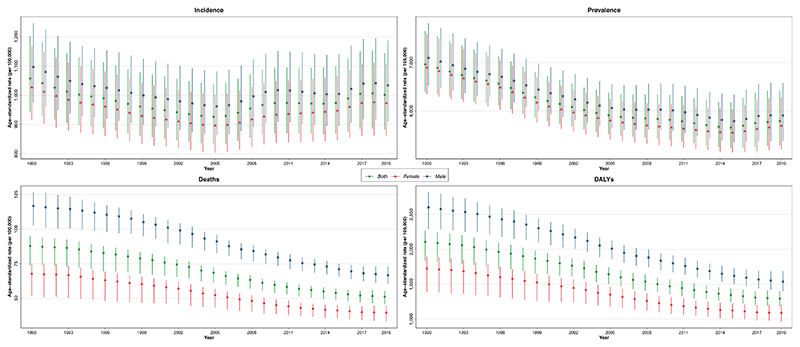
Global age-standardised rates of incidence, prevalence, deaths, and DALYs of chronic respiratory diseases in men, women, and in both sexes combined, 1990–2019. DALYs = Disability-Adjusted Life Years.

**Fig. 2:**
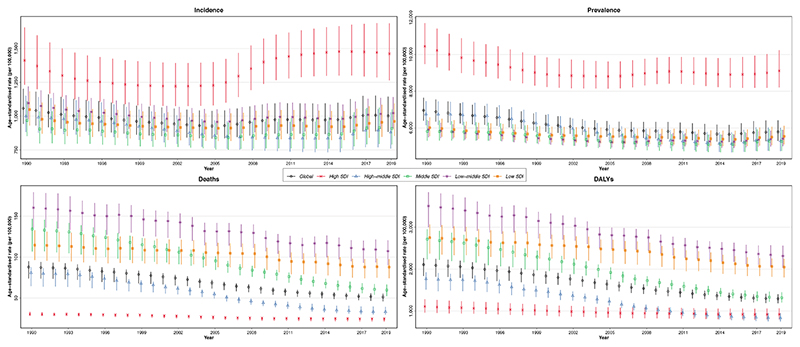
Global age-standardised rates of incidence, prevalence, deaths, and DALYs of chronic respiratory diseases in both sexes combined in each SDI quintile. DALYs = Disability-Adjusted Life Years, SDI = Socio-demographic Index.

**Fig. 3:**
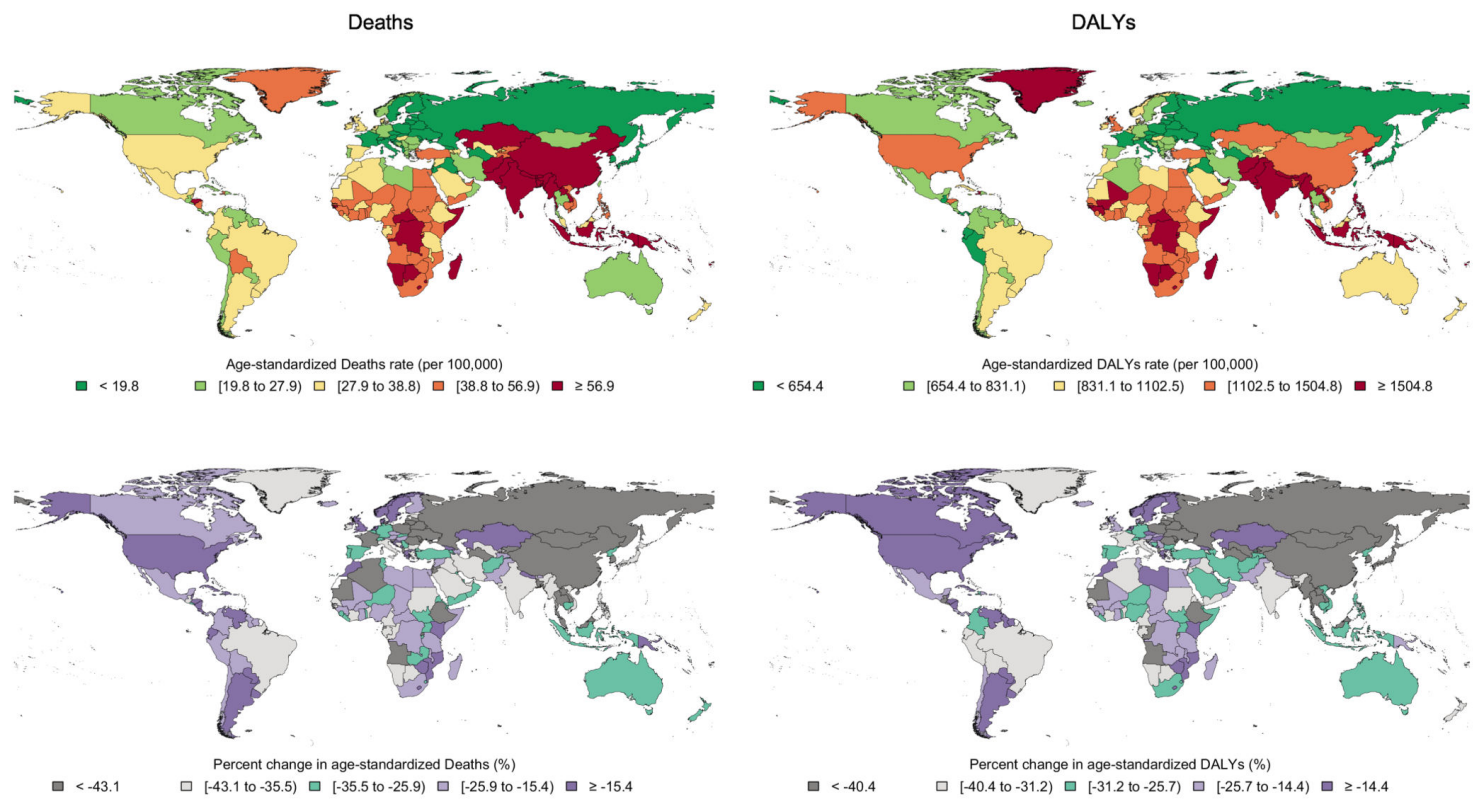
Global age-standardised rate of deaths and DALYs from chronic respiratory diseases in 2019 and their percent change from 1990 in both sexes combined. DALYs = Disability-Adjusted Life Years.

In 2019, the CRDs were the third leading cause of mortality, accounting for 4.0 million (95% UI 3.6–4.3) deaths globally. The ASR of mortality has steadily decreased by 41.7% (32.2%–47.6%) from 1990 to 2019 (Table 1). The ASR of mortality was higher in men throughout the investigated period and 1.7 of that of women in 2019 (Fig. 1). Among 21 GBD regions, Oceania, followed by South Asia, had the highest ASR of mortality, while high-income Asia Pacific, followed by Eastern Europe, had the lowest in 2019 (appendix 2, Table S5). From 1990 until 2019, the ASR of mortality decreased significantly in all SDI quintiles, and high SDI and low-middle SDI countries had the lowest and highest estimates, respectively (Fig. 2). Nepal had the highest ASR of mortality from CRDs in 2019 (231.2 [175.8–270.3]), and Singapore had the largest reduction in this rate from 1990 (80.5% [72.0%–83.4%]) among 204 countries and territories (Fig. 3).

The CRDs were responsible for 103.5 million (94.8–112.3) DALYs constituting 4.1% (3.7%–4.4%) of global DALYs for all causes in 2019 (not shown). YLLs accounted for 68.5% of the ASR of DALYs in 2019 (appendix 2, Fig. S9). The ASR of DALYs has decreased by 38.6% (30.9%–43.3%) globally from 1990 to 2019. Throughout 1990–2019, the ASR of DALYs has been higher in men (Fig. 1). Oceania, followed by South Asia, had the highest ASR of DALYs while high-income Asia Pacific and Eastern Europe had the lowest. The ASR of DALYs decreased in all SDI quintiles from 1990 to 2019, with middle and high-middle SDI countries experiencing the largest decrease (appendix 2, Table S2). Singapore had the largest reduction in the ASR of DALYs due to CRDs from 1990 (68.3% [61.8%–72.0%]) (Fig. 3).

In 2019, 454.6 million (417.4–499.1) people were estimated to suffer from CRD. The ASR of prevalence has decreased by 16.9% (15.1%–18.5%) from 1990 to 2019. No significant difference has been found between the sexes throughout the investigated period in the ASR of prevalence. High-income North America, followed by Australasia, had the highest ASR of prevalence, while East and Central Asia had the lowest. The high SDI quintile had the highest ASR of prevalence throughout the investigated period, while it was comparable among other SDI quintiles. All SDI quintiles had a lower ASR of prevalence in 2019 than in 1990 (Fig. 2).

In 2019, 77.6 million (68.9–87.9) new cases of CRDs were estimated globally, which has increased by 49.0% (42.1%–55.6%) from 1990. Decomposition analysis showed that population growth, responsible for 91.0% of the increased crude incidence number (44.6% out of 49.0%), had been the main driving force (appendix 2, Table S3). However, the ASR of incidence has decreased by 5.3% (3.6%–7.1%) from 1990 to 2019. No significant difference has been found between the sexes throughout the investigated period in the ASR of incidence (Fig. 1). High-income North America had the highest ASR of incidence in 2019, whereas Western Europe and East Asia had the lowest. Similar to prevalence, the high SDI had the highest ASR of incidence from 1990 until 2019, while it was comparable among other SDI quintiles.

**Fig. 4:**
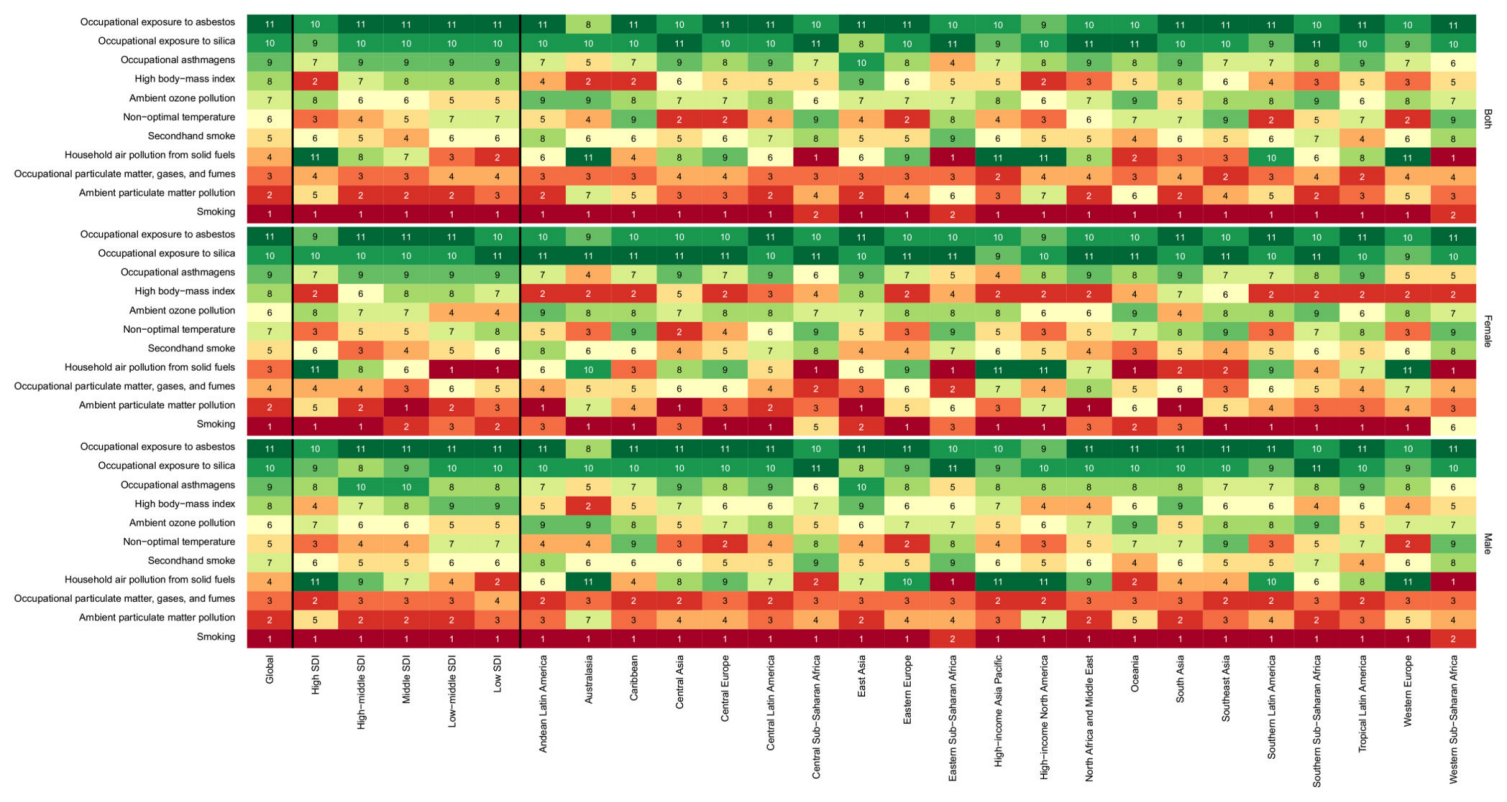
Ranked contribution of risk factors to the age-standardised rate of DALYs from chronic respiratory diseases by region, 2019, for both sexes combined, females, and males. Risk factors are ranked from 1 (the leading risk factor for age-standardised Disability-Adjusted Life Years (DALYs); dark red) to 11 (the lowest risk factor for age-standardised DALYs; dark green). The numbers inside each box indicate the ranking.

From total DALYs and deaths due to CRDs in 2019, 62.0% and 69.6% were attributed to all risk factors (not shown). Globally, smoking was the primary risk factor responsible for the ASR of DALYs from CRDs followed by ambient PM pollution (Table 2). The major risk factors varied in different regions. Household air pollution from solid fuels was the leading risk factor accounting for DALYs and death in Central, Western, and Eastern Sub-Saharan Africa. The burden attributed to ambient PM pollution was the lowest in the high SDI quintile (Fig. 4, appendix 2, Fig. S15).

### COPD

With 212.3 million (200.4–225.1) prevalent cases and 16.2 million (15.2–17.2) new cases, COPD accounted for 3.3 million (2.9–3.6) deaths globally in 2019. Among CRDs, COPD has been the main contributor to the global ASR of DALYs and mortality. The ASR of prevalence, incidence, deaths, and DALYs have significantly decreased from 1990 to 2019 by 8.7% (7.3%–10.2%), 7.4% (5.9%–8.8%), 41.7% (31.1%–48.0%), and 39.8% (30.2%–44.9%), respectively. Men have had higher ASRs of prevalence, deaths, DALYs, and incidence throughout the investigated period (appendix 2, Fig. S17). COPD constituted the majority of new and prevalent cases in the older than 35 and 50 age groups, respectively, and the incidence and prevalence rates increased with aging globally (appendix 2, Figs. S6–S8).

In 2019, high-income North America had the highest ASR of prevalence, but Oceania had the highest ASR of incidence, deaths, and DALYs. The ASR of deaths and DALYs dropped in all SDI quintiles in 2019 than 1990. The low-middle SDI quintile has had the highest ASR of deaths and DALYs throughout the investigated period, while the high SDI quintile has had the lowest. Compared to other SDI quintiles, the ASR of prevalence has been the lowest in low SDI countries from 1990 to 2019. Nevertheless, from 2010, it has been comparable between high-middle SDI and low SDI quintiles. Moreover, the highest ASR of incidence has been observed in low-middle SDI countries from 1990 to 2019 (appendix 2, Fig. S18). The ASR of prevalence has slightly increased in low SDI countries (2.1% [0.6%–3.4%]) while it has decreased in other SDI quintiles from 1990 to 2019.

Globally, smoking was the most prevalent risk factor of COPD and was responsible for 424.0 (380.2–465.7) ASR of DALYs and 20.4 (18.1–22.6) ASR of deaths followed by ambient PM pollution. While these risk factors were common between sexes, the third most prevalent risk factors were occupational PM, gases, and fumes and household air pollution from solid fuels in men and women, respectively. Geographical location and sociodemographic status also affected the distribution of the risk factors. In contrast to other SDI quintiles, where smoking had the highest attributable ASR of DALYs, household air pollution from solid fuels was the leading risk factor in low SDI countries, accounting for 531.8 (343.2–744.9) ASR of DALYs. Interestingly, in high SDI countries, non-optimal temperature was the second most prevalent risk factor following smoking (appendix 2, Figs. S19 and S20).

### Asthma

**Fig. 5:**
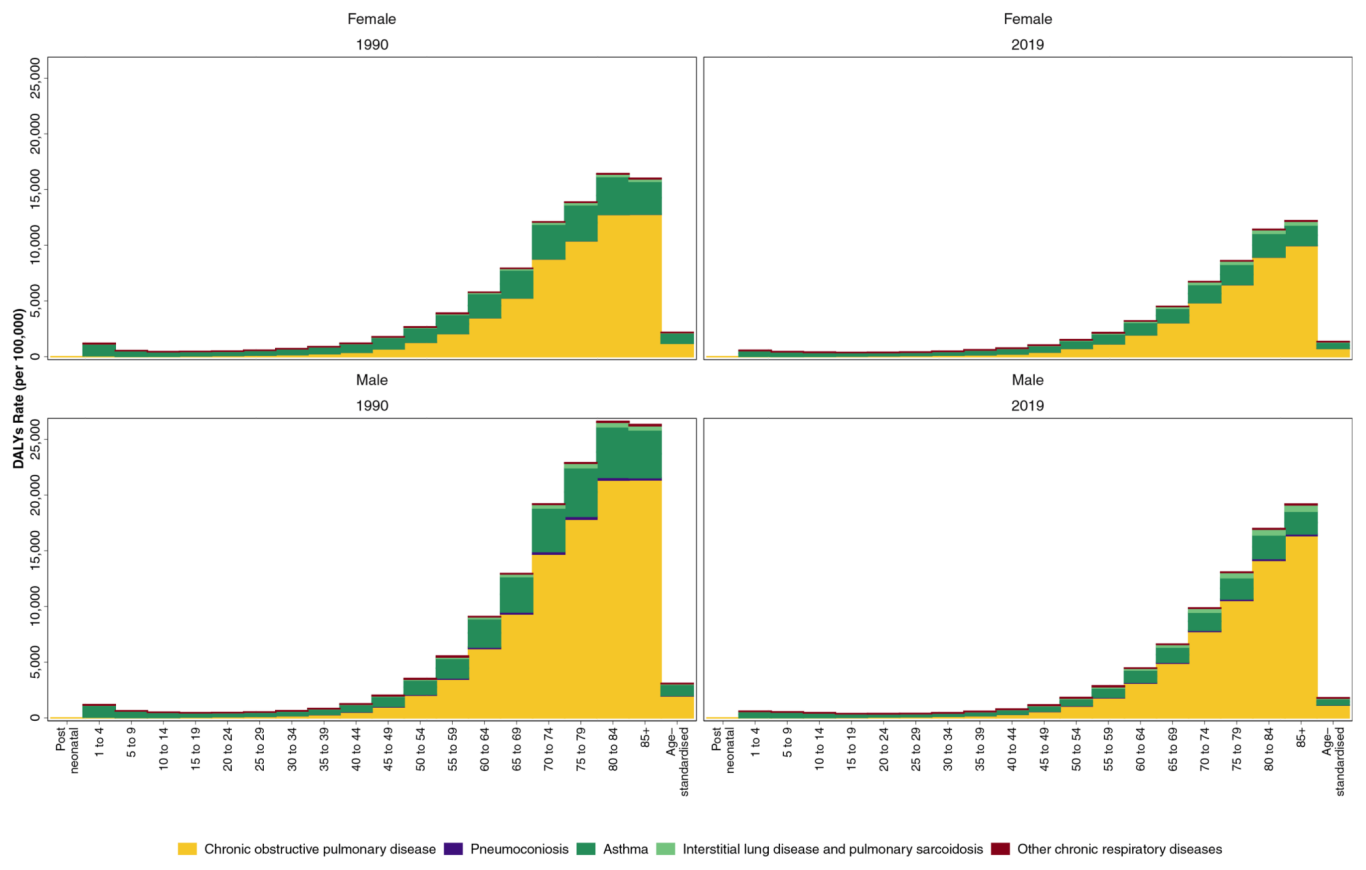
Absolute rate of DALYs from chronic respiratory diseases by age in men and women in 1990 and 2019 with age-standardised rate. DALYs = Disability-Adjusted Life Years.

Asthma accounted for 21.6 million (17.1–27.0) DALYs globally in 2019 with 262.4 million (224.1–309.5) prevalent cases and 37.0 million (29.6–45.9) new cases. Asthma has been the main contributor to the global ASR of prevalence and incidence of CRDs. All measures were closely comparable between the sexes (appendix 2, Fig. S21). The ASR of incidence, prevalence, deaths, and DALYs have significantly decreased from 1990 to 2019 by 13.1% (10.2%–16.3%), 24.1% (20.8%–27.2%), 51.3% (43.7%–59.1%), and 42.5% (36.6%–48.5%), respectively. Asthma constituted the majority of DALYs in the under 35 age group, with the highest incidence rate in the 1–4 years age group (1884.6 [1183.7–2879.0]) in 2019 worldwide (Fig. 5, appendix 2, Figs. S6–S9).

In 2019, high-income North America had the highest ASR of prevalence and incidence, whereas Oceania had the highest ASR of death and DALYs. The lowest ASR of prevalence and incidence were observed in East and South Asia, respectively. East Asia had the lowest ASR of DALYs, while Eastern Europe had the lowest ASR of deaths. From 1990 to 2019, high SDI countries have had the lowest ASR of death and the largest decline in that (73.0% [69.9%–75.4%]), as well as the highest ASR of incidence and prevalence, compared to other quintiles (appendix 2, Fig. S22).

In 2019, worldwide, high BMI was the leading risk factor comprising 44.8 (26.4–68.6) ASR of attributed DALYs in both sexes, followed by smoking. When stratified by sex, smoking was the primary risk factor in men, accounting for 40.3 (21.9–56.1) ASR of DALYs. However, smoking stood as the last risk factor in women (appendix 2, Fig. S24). In all SDI quintiles, smoking was the second most prominent risk factor after high BMI, except for low SDI. Nevertheless, in low SDI countries, occupational asthmagens ranked second (appendix 2, Fig. S23).

### ILD and pulmonary sarcoidosis

ILD and pulmonary sarcoidosis were responsible for 3.8 million (2.9–4.5) DALYs globally in 2019, with 4.7 million (4.0–5.4) prevalent cases and 24.2 million (19.6–29.5) new cases. Throughout the investigated period, the ASR of DALYs and deaths have been slightly lower in women, while the ASR of prevalence and incidence were comparable (appendix 2, Fig. S25). Globally, the ASR of prevalence and incidence have increased from 1990 to 2019 by 9.4% (6.1%–12.9%) and 14.1% (11.1%–17.3%), respectively. Nevertheless, the ASR of deaths and DALYs have remained stable (Table 1).

In 2019, Andean Latin America, followed by South Asia, had the highest, while Eastern Europe, followed by East Asia, had the lowest ASR of death and DALYs. High-income Asia Pacific and high-income North America had the highest ASR of prevalence and incidence, respectively. The high SDI quintile had the highest ASR of prevalence, whereas middle and high-middle SDI countries had the lowest ASR of DALYs and deaths (appendix 2, Fig. S26). In all SDI quintiles, except for low-middle SDI, the ASR of prevalence significantly increased from 1990 to 2019 (appendix 2, Table S2).

### Pneumoconiosis

Globally, silicosis, asbestosis, coal workers, and other pneumoconiosis were estimated to account for 0.9 million collectively (0.8–1.1) DALYs and 3.1 million (2.6–3.6) prevalent cases in 2019. Pneumoconiosis prevalence has remained comparable from 1990 to 2019 while the ASR of DALYs, deaths, and incidence have decreased by 44.4% (31.2%–52.9%), 53.3% (38.6%–60.9%), and 13.7% (6.6%–21.3%), respectively (Table 1). Despite the overall decreasing trend in the ASR of DALYs, deaths, and incidence, the ASR of incidence slightly rose by 5.4% (1.1%–10.2%) from 1990 to 2019 in women globally. Men have had significantly higher ASR of DALYs, deaths, prevalence, and incidence throughout the investigated period (appendix 2, Fig. S27).

Pneumoconiosis ranked third among all causes constituting DALYs from CRDs in East Asia responsible for 29.2 (22.9–37.1) ASR of DALYs in 2019, which is markedly higher than other 21 regions (appendix 2, Fig. S14). This region has had the highest ASR of DALYs, deaths, incidence, and prevalence due to pneumoconiosis from 1990 to 2019. Asbestosis was the primary contributor to the ASR of DALYs due to pneumoconiosis in Australasia, high-income North America, Oceania, Eastern, and Southern Sub-Saharan Africa (appendix 2, Fig. S31). In other 21 regions, mainly silicosis and to a lesser extent, other pneumoconiosis constituted most of the ASR of DALYs (appendix 2, Table S4). Moreover, from 1990 to 2019, middle and high-middle SDI regions had the highest ASR of prevalence, while the low SDI quintile, followed by high SDI, had the lowest ASR of pneumoconiosis prevalence (appendix 2, Fig. S28).

Globally, occupational exposure to silica, PM, gases, fumes, and asbestos were the risk factors of pneumoconiosis in order of attributed ASR of DALYs. Nevertheless, occupational PM, gases, and fumes ranked first, and occupational exposure to asbestos ranked last in women (appendix 2, Fig. S29).

## Discussion

Globally, the total number of deaths, DALYs, incidence, and prevalence of CRDs rose, whereas the ASR of all these indices declined in both sexes combined during the past three decades. The increase in crude numbers is primarily due to population growth. On a global scale, significant progress was achieved in reducing ASR of deaths, DALYs, prevalence, and incidence of COPD, asthma, and pneumoconiosis in both sexes combined. Nevertheless, this trend was variable among different geographical locations and sexes. Among CRDs, the global ASR of deaths and DALYs of ILD and pulmonary sarcoidosis remained stable while the incidence and prevalence grew. Asthma had the highest crude and ASR of incidence and prevalence among CRDs, while COPD accounted for the highest deaths and DALYs.

In the past three decades, a considerable drop was observed in the ASR of DALYs due to CRDs attributable to all risk factors, except for ambient PM pollution, high BMI, and occupational asbestos exposure in both sexes. Smoking, followed by ambient PM, is the major risk of CRDs worldwide in both sexes. The non-optimal temperature is a new risk added in GBD 2019, which is responsible for 8.3% (6.5%–10.1%) of total DALYs due to COPD in 2019 (not shown). This finding highlights the potential consequences of climate change on CRDs, particularly COPD.^18^ Climate change can increase temperature variability and result in extremely cold or warm temperatures, which can directly aggravate COPD exacerbations or increase exposure to environmental risk factors.^18^ Climate change may also result in longer pollen seasons with pollens with increased quantity and potency affecting the burden of asthma.^19^ High BMI, as the only evaluated metabolic risk factor for CRDs, was the leading risk factor of asthma in both sexes combined worldwide, with a more prominent role in women. The steady trend of the ASR of deaths and DALYs from asthma attributed to high BMI worldwide highlights the necessity of global attention for lifestyle modification interventions, which may reduce morbidity in patients with concomitant asthma and obesity.^20^ Given the higher prevalence of obesity in high- and upper-middle-income countries compared to low- and lower-middle-income countries (LMICs), these interventions may be of more value in these nations.^21^

Smoking was the leading risk factor for DALYs from CRDs in all regions except for Sub-Saharan Africa. A significant decrease is observed in the DALYs attributed to smoking in East Asia (66.7% [52.0%–72.6%]), which is accompanied by a marked drop in the ASR of DALYs (67.0% [52.6%–72.0%]). Like this region, the ASR of DALYs attributed to smoking declined in all regions, except for the Caribbean. These findings indicate that measures developed by the WHO Framework Convention on Tobacco Control (WHO FCTC) and in the WHO MPOWER package,^22^ such as demand reduction acts, regulation of advertisement, contents and labeling of tobacco products, and taxation on tobacco, have played a substantial role in lowering smoking globally.^23^ Targeted tobacco control strategies in China, the largest and most populous country in East Asia, namely the Healthy China 2030 strategy, which aims to reduce the smoking prevalence to 20%, could explain the significant reduction of burden due to smoking in this region.^24^

Nevertheless, there is a substantial potential for further reduction of CRDs burden attributed to smoking globally as many countries have not been fully adherent to tobacco control policies.^25^ Specifically, strong policies from the WHO FCTC have been poorly implemented in many LMICs.^23^ The Caribbean is the only region without a considerable change in the ASR of CRDs burden attributed to smoking. Cuba, the most populated country in this region, is among the few countries with growth in the ASR of DALYs from CRDs attributed to smoking from 1990 to 2019 (25.4% [2.1%–51.0%]) (appendix 2, Table S6). Cuba is one of the handful of countries that have not ratified the WHO FCTC programme, with a low cessation rate among Cubans found by previous investigations.^26^ This finding mandates a more careful reconsideration of tobacco control strategies in this region. To reduce smoking prevalence and the associated burden of CRDs, prevention of smoking initiation in adolescents and smoking cessation among current smokers are essential; however, the higher estimated prevalence of tobacco use in high- and upper-middle-income countries compared to LMICs indicates that the latter approach could be more crucial in these nations.^27^ In addition to tobacco smoking, epidemiological evidence suggests that e-cigarettes use is associated with COPD and asthma.^28^ While the GBD 2019 study has not included e-cigarettes use as a risk factor, its potential impact on the burden of CRDs cannot be overlooked.

In Sub-Saharan Africa, household air pollution from solid fuels was the primary risk factor responsible for DALYs from CRDs. While globally, the attributed burden of CRDs due to household air pollution has had the most considerable drop from 1990 to 2019 compared to the other risk factors (79.4% [72.7%–84.4%]), it still accounts for a substantial burden in the LMICs. According to the Energy Sector Management Assistance Program (ESMAP), near four billion people are estimated to lack access to modern energy heating or cooking services, and women and children have a higher exposure enduring a larger impact. Financial, social, and cultural barriers hinder the transition from traditional biomass cooking fuels to modern energy sources, i.e. electricity and gas.^29^

The Clean Cooking Alliance (CCA) is one of the most prominent global initiatives to make clean cooking accessible in the LMICs.^30^ Despite global attempts to improve access to clean energies, traditional solid fuel combustion has increased in the Sub-Saharan region due to the outgrowing pace of population growth.^29^ Improved access to modern energy cooking services is an indispensable step in achieving the SDGs defined by the UN until 2030. Not only can this significantly reduce mortality due to CRD, but it can also improve gender equality, access to affordable and clean energy, climate change, and terrestrial ecosystems.^31^ Importantly, household air pollution has been cited as a risk factor of asthma,^32^ albeit due to the mixed reports, this is not included in the GBD 2019 study, and further research is required to assess the association.

Globally, ambient PM pollution is the second major risk factor of CRDs, with no significant alteration in the attributed ASR of DALYs and deaths from CRDs in the past three decades. The ASR of DALYs from CRDs due to ambient PM pollution has decreased in Central, Eastern, and Western Europe, whereas it has risen in Sub-Saharan Africa and low SDI quintile in both sexes combined from 1990 to 2019. The growing burden in the Sub-Saharan Africa region is chiefly ascribed to increased desert dust due to climate change and rapid urbanisation.^33,34^ The LMICs have shown higher concentrations of PM pollution due to lack of legislation and/or adherence to air quality guidelines, higher prevalence of coal power stations, and not meeting vehicles emission standards.^35,36^ The European region is at the forefront of combatting ambient PM pollution with the European Green Deal, which aims to reduce greenhouse gas emissions by at least 55% by 2030 compared to 1990.^37^

The ASR of DALYs from COPD, asthma, and pneumoconiosis attributed to occupational risks has dropped in the past three decades worldwide in both sexes combined. The major DALYs attributed to the occupational risks are from COPD. The ASR of DALYs from CRDs attributed to these risks are approximately three-fold in men than women globally in 2019, which is justified by the lower employment rate of women in professions involving the relevant exposures. Analysis of the GBD 2016 study showed that the population attributable fraction for occupational risks for COPD, asthma, and pneumoconiosis were 17%, 10%, and 100%, respectively.^38^ The highest DALYs from CRDs due to occupational risks are observed in South Asia, Oceania, and East Asia. In China, pneumoconiosis constituted 90% of occupational diseases.^39^ Allocation of resources and occupational health legislation are critical in these regions to reduce toxic exposures and ensure high-quality health services for susceptible workers.^40^

The highest ASR of deaths and DALYs from CRDs is observed in Oceania and South Asia and the low SDI quintile despite the moderate ASR of prevalence in these regions. On the other hand, the high SDI quintile has the highest ASR of prevalence but the lowest deaths in 2019. These findings accent the variability of management and quality of care among countries with different income levels. Chronic respiratory care is a multi-faceted challenge in LMICs. Lack of preventive measures and increased lifetime exposure to CRDs risks should not be overlooked. CRDs are commonly underdiagnosed in these countries; therefore, patients are frequently only detected when developing severe symptoms. Restricted access to the diagnostic tools, i.e. spirometry and chest imaging, at the primary care level and shortage of trained clinical staff able to accurately perform and interpret the tests are the primary challenges in diagnosing CRDs in the LMICs. A dearth of health professionals with clinical respiratory training and limited access to medications impede the appropriate management of CRDs in such settings.^41^ For instance, inhaled corticosteroids are vital in managing asthma and have been shown to reduce morbidity and mortality.^10^ Nevertheless, they are typically unavailable, unaffordable, or under-prescribed in the LMICs. Improving chronic respiratory care in these regions hinges upon fortified healthcare systems providing high-quality preventive, diagnostic, therapeutic, rehabilitative, and palliative measures.^41^

Multiple global initiatives have been developed over the past few decades to improve respiratory care, undoubtedly contributing to the global decline in the age-standardised burden of CRDs. The Package of Essential Non-communicable (PEN) disease interventions for primary health care was designed to facilitate the provision of acceptable care for patients with NCDs, including CRDs, even in settings with limited resources.^42^ The Practical Approach to Lung health (PAL) was another tool created by the WHO to improve the management of respiratory patients in primary healthcare settings, especially in countries with weak health systems.^43^ Years after the development of the PAL, the GARD was established to improve the prevention, diagnosis, and medical care of CRDs according to local needs worldwide by estimating population needs, advocating for health promotion and prevention, and developing cost-effective strategies for CRDs.^44^ In addition, other global initiatives focusing on COPD (GOLD)^9^ and asthma (GINA)^10^ have been developed to increase awareness, improve prevention, management, and access to effective treatments.

With COVID-19 continuing to spread around the world, the interaction between COVID-19 and CRDs is under the spotlight.^45^ A population cohort study found that while asthma was not associated with a major increased risk of severity, COPD and ILD were independent predictors of severity and higher mortality in patients with COVID-19. However, the death rates from COVID-19 were lower than the ordinary risk of death from any cause.^46^ As ILD can impact the outcomes of COVID-19, COVID-19 may also result in long-lasting fibrotic-like changes in the lungs, which can be detectable even after 6–12 months on imaging in some cases.^47,48^

This study is an updated comprehensive analysis of the global, regional, and national epidemiology of CRDs and their associated risk factors. Previous reports utilising the GBD 2019 data have reported the burden attributable to certain sub-causes or risk factors, but none have focused on all CRDs included in the GBD 2019 study.^36,40,49^ Whilst the GBD 2019 supplies a comprehensive estimation of the burden of most NCDs, it faces several limitations. Lack of reliable primary data sources, particularly in the LMICs, could adversely affect the accuracy of the estimates. The paucity of primary investigations in addition to the under-diagnosis in these regions can lead to underestimation. The GBD addresses this limitation by improving data processing and modeling and adding newly available data sources in each iteration. Nevertheless, further original investigations are incremental in accurately measuring the burden of diseases in such regions. Even when primary data are available, the various case definition of CRDs and lack of using the preferred definition could also affect the precision of the estimates. The GBD 2019 study entailed a wider alternative definition for COPD and asthma than the GBD 2017 and performed a bias mapping from the alternative to reference definitions.

Furthermore, we could not account for genetic susceptibilities in this study, albeit they can play a major role in developing COPD and asthma.^50^ This is beyond the scope of this manuscript and can be addressed in the future cycles of the GBD. Other CRDs were responsible for a considerable burden, although they encompassed various diseases, which hindered the measurement of the nonfatal estimates. Development of cause-specific estimates for sleep apnea and allergic rhinitis and sinusitis can be considered in the next cycles of the GBD, given their high prevalence.^51,52^ Lastly, reconsidering the available evidence for risk–outcome pairs would be crucial in future iterations, especially for ILD and pulmonary sarcoidosis. While currently, no risk factors have been cited for this cause, occupational and environmental risks can increase the risk of developing the disease.^53^

We were also unable to quantitatively account for the effect of climate change on the burden of CRDs due to a lack of sufficient data on environmental indicators within the same time span (1990–2019). Future endeavors are needed for collecting reliable data on climate change indicators enabling a quantitative assessment of their impact. Moreover, the GBD 2019 estimation was conducted before the COVID-19 pandemic.^54^ Therefore, future iterations of the GBD study need to address the impact of the COVID-19 pandemic on the burden of CRDs.

CRDs were the third leading cause of death in 2019. The age-standardised DALYs, death, prevalence, and incidence rates of CRDs have significantly dropped from 1990 to 2019 globally. However, the age-standardised prevalence and incidence rates grew in the high SDI quintile. While COPD primarily contributes to deaths and DALYs from CRDs, asthma has the highest prevalence worldwide. Men have higher age-standardised rates of deaths and DALYs from COPD and pneumoconiosis. The high age-standardised rates of deaths and DALYs from CRDs in the LMICs, particularly East Asia and Oceania, highlight the gaps in prevention, diagnosis, and management and warrant further investigations and respiratory care improvement strategies.

The estimates provided in this study can provide policymakers and healthcare providers with an overview of the burden and risk factors of CRDs to facilitate the path towards achieving the third SDG. Full global adherence to tobacco control measures and air quality improvement strategies are crucial in reducing the burden attributed to CRDs. In the LMICs, where CRDs are responsible for a substantial burden, in addition to these policies, improvement of respiratory care by providing clinical respiratory training for healthcare workers, raising public awareness, and access to diagnostic tools and medications are fundamental. Global attempts to foster clean cooking and heating energies in the LMICs, particularly the Sub-Saharan region, are essential for reducing deaths and DALYs from CRDs burden, especially in women.

## Supplementary Material

Supplementary Materials

## Figures and Tables

**Table 1:** Global incidence, prevalence, deaths, DALYs, YLLs, and YLDs from chronic respiratory diseases.

Measure	Age (metric)	Year	CRDs	Cause specific				
				COPD	Pneumoconiosis	Asthma	ILD &; pulmonary sarcoidosis	Other CRDs
**Incidence**	All ages (number)	% Change^a^	49.0 (42.1 to 55.6)	85.9 (82.3 to 89.2)	61.5 (44.6 to 77.6)	15.0 (11.7 to 18.0)	118.6 (110.2 to 127.0)	
		2019	77,625,300 (68,884,564 to 87,929,749)	16,214,828 (15,224,111 to 17,220,809)	199,125 (172,556 to 228,809)	36,979,267 (29,601,976 to 45,928,112)	24,232,080 (19,609,750 to 29,463,387)	
	Age-standardised (rate per 100,000)	% Change	−5.3 (−7.1 to −3.6)	−7.4 (−8.8 to −5.9)	−13.7 (−21.3 to −6.6)	−13.1 (−16.3 to −10.2)	14.1 (11.1 to 17.3)	
		2019	1001.6 (883.0 to 1144.4)	200.5 (188.6 to 212.6)	2.4 (2.1 to 2.7)	504.3 (400.6 to 633.3)	294.4 (238.5 to 356.6)	
**Prevalence**	All ages (number)	% Change	39.8 (36.3 to 43.2)	84.8 (81.6 to 88.0)	83.9 (62.1 to 102.9)	15.6 (12.7 to 18.9)	114.2 (106.4 to 122.1)	
		2019	454,557,390 (417,354,403 to 499,144,380)	212,335,951 (200,422,146 to 225,097,834)	3,072,550 (2,596,999 to 3,596,518)	262,405,182 (224,047,914 to 309,452,681)	4,710,180 (4,020,397 to 5,401,700)	
	Age-standardised (rate per 100,000)	% Change	−16.9 (−18.5 to −15.1)	−8.7 (−10.2 to −7.3)	−8.4 (−19.1 to 0.3)	−24.0 (−27.2 to −20.8)	9.4 (6.1 to 12.9)	
		2019	5789.2 (5290.7 to 6418.1)	2638.2 (2492.2 to 2796.1)	36.8 (31.1 to 43.1)	3415.5 (2898.9 to 4066.2)	57.6 (49.4 to 65.7)	
**Deaths**	All ages (number)	% Change	28.5 (15.2 to 50.1)	30.2 (15.7 to 55.0)	−3.0 (−19.2 to 29.1)	0.2 (−14.2 to 15.1)	166.6 (93.0 to 241.0)	52.3 (29.1 to 82.9)
		2019	3,974,315 (3,581,757 to 4,303,823)	3,280,636 (2,902,855 to 3,572,367)	23,015 (20,348 to 26,159)	461,069 (366,580 to 559,006)	169,833 (118,756 to 204,802)	39,761 (31,085 to 46,581)
	Age-standardised (rate per 100,000)	% Change	−41.7 (−47.6 to −32.2)	−41.7 (−48.0 to −31.1)	−53.3 (−60.9 to −38.6)	−51.3 (−59.1 to −43.7)	23.4 (−13.1 to 58.6)	−19.5 (−30.2 to −6.0)
		2019	51.3 (45.9 to 55.5)	42.5 (37.6 to 46.3)	0.3 (0.3 to 0.3)	5.8 (4.6 to 7.0)	2.2 (1.5 to 2.6)	0.5 (0.4 to 0.6)
**DALYs**	All ages (number)	% Change	20.8 (12.1 to 36.1)	25.6 (15.1 to 46.0)	11.2 (−6.1 to 38.1)	−3.5 (−10.8 to 4.5)	122.9 (79.4 to 168.6)	76.5 (48.2 to 104.5)
		2019	103,533,107 (94,792,077 to 112,266,452)	74,432,367 (68,204,127 to 80,193,347)	919,077 (761,478 to 1,116,127)	21,550,977 (17,141,587 to 26,971,997)	3,770,894 (2,864,234 to 4,468,319)	2,859,792 (2,461,295 to 3,217,791)
	Age-standardised (rate per 100,000)	% Change	−38.6 (−43.3 to −30.9)	−39.8 (−44.9 to −30.2)	−44.4 (−52.9 to −31.2)	−42.5 (−48.5 to −36.6)	11.7 (−10.8 to 35.1)	13.8 (−2.0 to 31.0)
		2019	1293.7 (1183.0 to 1403.6)	926.1 (848.8 to 997.7)	11.1 (9.2 to 13.5)	273.6 (216.7 to 343.4)	46.4 (35.1 to 55.0)	36.5 (31.4 to 41.1)
**YLLs**	All ages (number)	% Change	8.8 (−1.7 to 27.2)	11.9 (−0.5 to 35.1)	−18.2 (−33.5 to 11.9)	−15.8 (−24.5 to −3.6)	124.8 (75.1 to 178.8)	32.0 (4.1 to 67.0)
		2019	71,145,745 (64,700,056 to 77,011,749)	54,594,898 (48,711,468 to 59,513,367)	479,340 (418,214 to 550,546)	11,354,712 (9,279,939 to 13,372,007)	3,291,056 (2,406,555 to 3,952,188)	1,425,739 (1,135,697 to 1,693,896)
	Age-standardised (rate per 100,000)	% Change	−46.5 (−51.7 to −37.2)	−46.8 (−52.6 to −36.1)	−58.9 (−66.3 to −44.0)	−53.5 (−59.0 to −46.5)	12.3 (−13.1 to 39.7)	−17.7 (−31.9 to 1.7)
		2019	885.9 (805.6 to 959.4)	680.8 (606.4 to 741.6)	5.8 (5.1 to 6.7)	140.6 (115.3 to 165.3)	40.6 (29.7 to 48.8)	18.0 (14.2 to 21.4)
**YLDs**	All ages (number)	% Change	59.4 (51.9 to 67.3)	89.4 (85.4 to 93.6)	82.9 (61.1 to 101.9)	15.4 (12.7 to 18.7)	110.4 (102.4 to 119.0)	165.8 (157.8 to 172.7)
		2019	32,387,362 (26,116,058 to 38,488,142)	19,837,469 (16,596,490 to 22,441,727)	439,737 (292,559 to 625,475)	10,196,265 (6,654,649 to 15,061,355)	479,838 (321,777 to 690,617)	1,434,053 (1,173,488 to 1,649,383)
	Age-standardised (rate per100,000)	% Change	−9.9 (−12.2 to −7.7)	−4.9 (−6.6 to −3.0)	−8.6 (−19.0 to 0.5)	−23.4 (−26.6 to −20.2)	8.1 (4.8 to 11.6)	81.3 (74.5 to 86.7)
		2019	407.9 (327.4 to 486.9)	245.3 (205.2 to 276.8)	5.3 (3.5 to 7.5)	133.0 (86.9 to 197)	5.9 (3.9 to 8.4)	18.5 (15.1 to 21.3)

Data in parentheses are 95% Uncertainty Intervals (95% UIs). CRDs = Chronic Respiratory Diseases; COPD = Chronic Obstructive Pulmonary Disease; ILD = Interstitial Lung Disease; DALYs = Disability-Adjusted Life Years; YLLs = Years of Life Lost; YLDs = Years Lived with Disability.

a % Change (1990–2019).

**Table 2:** Global age-standardised rates (per 100,000) of deaths and DALYs from chronic respiratory diseases attributed to risk factors in both sexes combined with percent change.

Measure	Year	Risk factor	CRDs	Cause specific		
				COPD	Pneumoconiosis	Asthma
**Deaths**	**% Change^a^**	**Environmental/occupational risks**	−52.1 (−58.1 to −42.3)	−52.0 (−58.2 to −42.2)	−53.3 (−60.9 to −38.6)	−55.1 (−62.3 to −45.6)
		Air pollution	−57.4 (−64.3 to −47.8)	−57.4 (−64.3 to −47.8)		
		Particulate matter pollution	−61.8 (−68.4 to −52.2)	−61.8 (−68.4 to −52.2)		
		Ambient particulate matter pollution	−12.1 (−39.9 to 32.0)	−12.1 (−39.9 to 32.0)		
		Household air pollution from solid fuels	−80.9 (−85.8 to −74.1)	−80.9 (−85.8 to −74.1)		
		Ambient ozone pollution	−21.6 (−31.6 to −6.3)	−21.6 (−31.6 to −6.3)		
		Non-optimal temperature	−49.7 (−57.1 to −35.2)	−49.7 (−57.1 to −35.2)		
		Occupational risks	−46.9 (−53.8 to −35.2)	−45.9 (−53.0 to −34.2)	−53.3 (−60.9 to −38.6)	−55.1 (−62.3 to −45.6)
		Occupational carcinogens	−52.0 (−61.1 to −33.5)		−52.0 (−61.1 to −33.5)	
		Occupational exposure to asbestos	15.6 (−8.5 to 33.6)		15.6 (−8.5 to 33.6)	
		Occupational exposure to silica	−58.9 (−67.9 to −39.4)		−58.9 (−67.9 to −39.4)	
		Occupational asthmagens	−55.1 (−62.3 to −45.6)			−55.1 (−62.3 to −45.6)
		Occupational particulate matter, gases, and fumes	−46.1 (−53.1 to −34.2)	−45.9 (−53 to −34.2)	−56.5 (−63.5 to −40.9)	
		**Behavioral risks**	−45.7 (−51.9 to −37.2)	−45.0 (−51.3 to −36.2)		−62.5 (−69.5 to −54.8)
		Tobacco	−45.7 (−51.9 to −37.2)	−45.0 (−51.3 to −36.2)		−62.5 (−69.5 to −54.8)
		Smoking	−44.9 (−51.4 to −36.7)	−44.0 (−50.5 to −35.8)		−62.5 (−69.5 to −54.8)
		Secondhand smoke	−51.8 (−58.7 to −38.0)	−51.8 (−58.7 to −38.0)		
		**Metabolic risks**	−20.1 (−35.6 to 5.2)			−20.1 (−35.6 to 5.2)
		High body-mass index	−20.1 (−35.6 to 5.2)			−20.1 (−35.6 to 5.2)
	**2019**	**Environmental/occupational risks**	24.2 (20.6 to 27.5)	23.5 (20.0 to 26.8)	0.3 (0.3 to 0.3)	0.4 (0.3 to 0.5)
		Air pollution	16.8 (13.3 to 20.3)	16.8 (13.3 to 20.3)		
		Particulate matter pollution	14.0 (10.9 to 17.4)	14.0 (10.9 to 17.4)		
		Ambient particulate matter pollution	9.0 (7.1 to 11.1)	9.0 (7.1 to 11.1)		
		Household air pollution from solid fuels	5.1 (3.0 to 7.8)	5.1 (3.0 to 7.8)		
		Ambient ozone pollution	4.7 (2.2 to 7.3)	4.7 (2.2 to 7.3)		
		Non-optimal temperature	5.1 (4.0 to 6.3)	5.1 (4.0 to 6.3)		
		Occupational risks	7.3 (5.9 to 8.9)	6.6 (5.2 to 8.2)	0.3 (0.3 to 0.3)	0.4 (0.3 to 0.5)
		Occupational carcinogens	0.2 (0.2 to 0.2)		0.2 (0.2 to 0.2)	
		Occupational exposure to asbestos	0 (0 to 0.1)		0 (0 to 0.1)	
		Occupational exposure to silica	0.2 (0.1 to 0.2)		0.2 (0.1 to 0.2)	
		Occupational asthmagens	0.4 (0.3 to 0.5)			0.4 (0.3 to 0.5)
		Occupational particulate matter, gases, and fumes	6.7 (5.3 to 8.3)	6.6 (5.2 to 8.2)	0.1 (0.1 to 0.1)	
		**Behavioral risks**	23.1 (20.3 to 25.8)	22.5 (19.7 to 25.0)		0.7 (0.4 to 1.0)
		Tobacco	23.1 (20.3 to 25.8)	22.5 (19.7 to 25.0)		0.7 (0.4 to 1.0)
		Smoking	21.1 (18.8 to 23.4)	20.4 (18.1 to 22.6)		0.7 (0.4 to 1.0)
		Secondhand smoke	3.6 (1.9 to 5.5)	3.6 (1.9 to 5.5)		
		**Metabolic risks**	0.9 (0.5 to 1.5)			0.9 (0.5 to 1.5)
		High body-mass index	0.9 (0.5 to 1.5)			0.9 (0.5 to 1.5)
**DALYs**	**% Change^a^**	**Environmental/occupational risks**	−51.6 (−57.2 to −42.9)	−52.0 (−57.7 to −43.0)	−44.4 (−52.9 to −31.2)	−45.9 (−52.8 to −38.7)
		Air pollution	−56.9 (−63.3 to −47.6)	−56.9 (−63.3 to −47.6)		
		Particulate matter pollution	−60.0 (−66.2 to −50.7)	−60.0 (−66.2 to −50.7)		
		Ambient particulate matter pollution	−8.8 (−35.8 to 34.2)	−8.8 (−35.8 to 34.2)		
		Household air pollution from solid fuels	−79.4 (−84.4 to −72.6)	−79.4 (−84.4 to −72.6)		
		Ambient ozone pollution	−26.5 (−36.2 to −11.0)	−26.5 (−36.2 to −11.0)		
		Non-optimal temperature	−55.1 (−62.7 to −40.5)	−55.1 (−62.7 to −40.5)		
		Occupational risks	−44.7 (−50.5 to −34.4)	−44.5 (−50.7 to −33.5)	−44.4 (−52.9 to −31.2)	−45.9 (−52.8 to −38.7)
		Occupational carcinogens	−40.9 (−51.3 to −25.9)		−40.9 (−51.3 to −25.9)	
		Occupational exposure to asbestos	−6.1 (−18.3 to 5.9)		−6.1 (−18.3 to 5.9)	
		Occupational exposure to silica	−43.3 (−54.2 to −27.1)		−43.3 (−54.2 to −27.1)	
		Occupational asthmagens	−45.9 (−52.8 to −38.7)			−45.9 (−52.8 to −38.7)
		Occupational particulate matter, gases, and fumes	−44.7 (−50.8 to −33.6)	−44.5 (−50.7 to −33.5)	−54.3 (−61.5 to −39.9)	
		**Behavioral risks**	−45.5 (−50.8 to −37.9)	−44.5 (−49.8 to −36.7)		−59.6 (−65.3 to −54.1)
		Tobacco	−45.5 (−50.8 to −37.9)	−44.5 (−49.8 to −36.7)		−59.6 (−65.3 to −54.1)
		Smoking	−45.2 (−50.8 to −37.8)	−43.9 (−49.7 to −36.4)		−59.6 (−65.3 to −54.1)
		Secondhand smoke	−49.3 (−55.1 to −37.5)	−49.3 (−55.1 to −37.5)		
		**Metabolic risks**	−11.9 (−26.1 to 8.9)			−11.9 (−26.1 to 8.9)
		High body-mass index	−11.9 (−26.1 to 8.9)			−11.9 (−26.1 to 8.9)
	**2019**	**Environmental/occupational risks**	510.9 (446.5 to 574.1)	476.9 (411.7 to 538.6)	11.1 (9.2 to 13.5)	22.9 (18.2 to 28.2)
		Air pollution	349.8 (280.2 to 413.2)	349.8 (280.2 to 413.2)		
		Particulate matter pollution	305.0 (239.7 to 369.2)	305.0 (239.7 to 369.2)		
		Ambient particulate matter pollution	190.8 (153.5 to 234.8)	190.8 (153.5 to 234.8)		
		Household air pollution from solid fuels	114.2 (69.8 to 172.4)	114.2 (69.8 to 172.4)		
		Ambient ozone pollution	77.0 (37.0 to 119.5)	77.0 (37.0 to 119.5)		
		Non-optimal temperature	77.9 (59.9 to 96.8)	77.9 (59.9 to 96.8)		
		Occupational risks	177.0 (151.8 to 203.6)	143.0 (118.6 to 168.7)	11.1 (9.2 to 13.5)	22.9 (18.2 to 28.2)
		Occupational carcinogens	8.8 (7.1 to 10.9)		8.8 (7.1 to 10.9)	
		Occupational exposure to asbestos	0.9 (0.7 to 1.0)		0.9 (0.7 to 1.0)	
		Occupational exposure to silica	7.9 (6.2 to 10.0)		7.9 (6.2 to 10.0)	
		Occupational asthmagens	22.9 (18.2 to 28.2)			22.9 (18.2 to 28.2)
		Occupational particulate matter, gases, and fumes	145.4 (120.9 to 171.1)	143 (118.6 to 168.7)	2.3 (1.9 to 2.9)	
		**Behavioral risks**	495.4 (444.3 to 546.0)	469.9 (418.1 to 519.1)		25.5 (13.6 to 36.2)
		Tobacco	495.4 (444.3 to 546.0)	469.9 (418.1 to 519.1)		25.5 (13.6 to 36.2)
		Smoking	449.5 (403.9 to 493.6)	424.0 (380.2 to 465.7)		25.5 (13.6 to 36.2)
		Secondhand smoke	78.8 (39.2 to 118.7)	78.8 (39.2 to 118.7)		
		**Metabolic risks**	44.8 (26.4 to 68.6)			44.8 (26.4 to 68.6)
		High body-mass index	44.8 (26.4 to 68.6)			44.8 (26.4 to 68.6)

Data in parentheses are 95% Uncertainty Intervals (95% UIs). COPD = Chronic Obstructive Pulmonary Disease, CRDs = Chronic Respiratory Diseases, DALYs = Disability-Adjusted Life Years.

a % Change (1990–2019).

## Data Availability

Data from this study are openly available in the online database of GBD 2019 as described in Methods.

## Research in context

### Evidence before this study

The Global Burden of Diseases, Injuries, and Risk Factors Study (GBD) provides the most comprehensive measurement of epidemiological features of non-communicable diseases (NCDs) to date. Among NCDs, chronic respiratory diseases (CRDs) account for a substantial burden and premature mortality worldwide. We reviewed online medical databases by a structured search with keywords “chronic respiratory disease(s)” or “chronic obstructive pulmonary disease (COPD)” or “asthma” or “pneumoconiosis” or “interstitial lung disease (ILD)” or “pulmonary sarcoidosis” AND “prevalence” or “incidence” or “mortality” or “disability-adjusted life year(s) (DALYs)” or “epidemiology” or “risk factor(s)” or “population attributable fraction (PAF)”. The GBD Collaborator Network has published the most recent paper on the CRDs using GBD 2017. This investigation concluded that CRDs account for substantial deaths and disabilities globally, and updated population measurements are essential for monitoring the progress towards achieving the third Sustainable Development Goal (SDG) of the United Nations (UN), a one-third reduction of premature mortality from NCDs by 2030.

### Added value of this study

As part of GBD 2019 study, this study provides updated estimates of mortality, disability, prevalence, and incidence of CRDs, including COPD, asthma, pneumoconiosis, interstitial lung disease, and pulmonary sarcoidosis from 1990-2019 in 21 GBD regions encompassing 204 countries and territories, by age, sex, and Socio-demographic Index (SDI). The DALYs and deaths attributed to potentially modifiable behavioural, environmental and occupational, and metabolic risk factors are also reported. This is the first report published by the GBD Collaborator Network reporting the global and regional burden of sub-causes of pneumoconiosis, i.e. silicosis, asbestosis, coal worker’s pneumoconiosis, and other pneumoconiosis. It is also the first to describe the attributable burden of CRDs to high body-mass index (BMI). Furthermore, this is the first cycle of GBD investigating the burden due to non-optimal temperature, which accentuates the potential role of climate change in disability and deaths from CRDs.

### Implications of all the available evidence

Results provided in this study reflect the impacts of the so far adopted strategies and shed light on the future location-specific policies that need to be established for reducing the burden resulting from CRDs by identifying the populations with the highest burden and the most influential risk factors. The high burden of deaths and disabilities from CRDs in low-middle income countries emphasises the crucial role of prevention, raising public awareness, specialised respiratory care training for the healthcare providers, and enhancing access to diagnostic tools as well as treatments in these countries. Future primary research is also essential for obtaining a more accurate picture of the current burden of CRDs in this region. Since smoking was the primary risk factor responsible for deaths and DALYs from CRDs, full enforcement of tobacco control programmes, especially in the Caribean region, is imperative in future policies. Given the high burden attributed to household air pollution in the Sub-Saharan region and low SDI countries, particularly in women, increased focus should be directed to promoting clean cooking and heating energies in this region. Ultimately, in addition to the aforementioned measures, global strategies for improving air quality and limiting occupational hazards are key steps in achieving the third UN SDG.
