# Global burden of chronic respiratory diseases and risk factors, 1990–2019: an update from the Global Burden of Disease Study 2019

**DOI:** 10.1016/j.eclinm.2023.101936

**Published:** 2023-04-25

**Authors:** GBD 2019 Chronic Respiratory Diseases Collaborators, Sara Momtazmanesh, Sahar Saeedi Moghaddam, Seyyed-Hadi Ghamari, Elaheh Malakan Rad, Negar Rezaei, Parnian Shobeiri, Amirali Aali, Mohsen Abbasi-Kangevari, Zeinab Abbasi-Kangevari, Michael Abdelmasseh, Meriem Abdoun, Deldar Morad Abdulah, Abu Yousuf Md Abdullah, Aidin Abedi, Hassan Abolhassani, Zahra Abrehdari-Tafreshi, Basavaprabhu Achappa, Denberu Eshetie Adane Adane, Tigist Demssew Adane, Isaac Yeboah Addo, Mohammad Adnan, Qorinah Estiningtyas Sakilah Adnani, Sajjad Ahmad, Ali Ahmadi, Keivan Ahmadi, Ali Ahmed, Ayman Ahmed, Tarik Ahmed Rashid, Hanadi Al Hamad, Fares Alahdab, Astawus Alemayehu, Sheikh Mohammad Alif, Syed Mohamed Aljunid, Sami Almustanyir, Khalid A. Altirkawi, Nelson Alvis-Guzman, Javad Aminian Dehkordi, Mehrdad Amir-Behghadami, Robert Ancuceanu, Catalina Liliana Andrei, Tudorel Andrei, Catherine M. Antony, Anayochukwu Edward Anyasodor, Jalal Arabloo, Judie Arulappan, Tahira Ashraf, Seyyed Shamsadin Athari, Engi F. Attia, Meshesha Tsegazeab Ayele, Sina Azadnajafabad, Abraham Samuel Babu, Sara Bagherieh, Ovidiu Constantin Baltatu, Maciej Banach, Mainak Bardhan, Francesco Barone-Adesi, Amadou Barrow, Saurav Basu, Nebiyou Simegnew Bayileyegn, Isabela M. Bensenor, Nikha Bhardwaj, Pankaj Bhardwaj, Ajay Nagesh Bhat, Krittika Bhattacharyya, Souad Bouaoud, Dejana Braithwaite, Michael Brauer, Muhammad Hammad Butt, Zahid A. Butt, Daniela Calina, Luis Alberto Cámera, Gashaw Sisay Chanie, Periklis Charalampous, Vijay Kumar Chattu, Odgerel Chimed-Ochir, Dinh-Toi Chu, Aaron J. Cohen, Natália Cruz-Martins, Omid Dadras, Aso Mohammad Darwesh, Saswati Das, Sisay Abebe Debela, Laura Delgado-Ortiz, Diriba Dereje, Mostafa Dianatinasab, Nancy Diao, Daniel Diaz, Lankamo Ena Digesa, Gebisa Dirirsa, Paul Narh Doku, Deepa Dongarwar, Abdel Douiri, Haneil Larson Dsouza, Ebrahim Eini, Michael Ekholuenetale, Temitope Cyrus Ekundayo, Ahmed Elabbas Mustafa Elagali, Muhammed Elhadi, Daniel Berhanie Enyew, Ryenchindorj Erkhembayar, Farshid Etaee, Adeniyi Francis Fagbamigbe, Andre Faro, Ali Fatehizadeh, Ginenus Fekadu, Irina Filip, Florian Fischer, Masoud Foroutan, Richard Charles Franklin, Peter Andras Gaal, Santosh Gaihre, Abduzhappar Gaipov, Mesfin Gebrehiwot, Urge Gerema, Motuma Erena Getachew, Tamiru Getachew, Mansour Ghafourifard, Reza Ghanbari, Ahmad Ghashghaee, Ali Gholami, Artyom Urievich Gil, Mahaveer Golechha, Pouya Goleij, Davide Golinelli, Habtamu Alganeh Guadie, Bhawna Gupta, Sapna Gupta, Veer Bala Gupta, Vivek Kumar Gupta, Mostafa Hadei, Rabih Halwani, Asif Hanif, Arief Hargono, Mehdi Harorani, Risky Kusuma Hartono, Hamidreza Hasani, Abdiwahab Hashi, Simon I. Hay, Mohammad Heidari, Merel E. Hellemons, Claudiu Herteliu, Ramesh Holla, Nobuyuki Horita, Mohammad Hoseini, Mehdi Hosseinzadeh, Junjie Huang, Salman Hussain, Bing-Fang Hwang, Ivo Iavicoli, Segun Emmanuel Ibitoye, Sufyan Ibrahim, Olayinka Stephen Ilesanmi, Irena M. Ilic, Milena D. Ilic, Mustapha Immurana, Nahlah Elkudssiah Ismail, Linda Merin J, Mihajlo Jakovljevic, Elham Jamshidi, Manthan Dilipkumar Janodia, Tahereh Javaheri, Sathish Kumar Jayapal, Shubha Jayaram, Ravi Prakash Jha, Olatunji Johnson, Tamas Joo, Nitin Joseph, Jacek Jerzy Jozwiak, Vaishali K, Billingsley Kaambwa, Zubair Kabir, Laleh R. Kalankesh, Rohollah Kalhor, Himal Kandel, Shama D. Karanth, Ibraheem M. Karaye, Bekalu Getnet Kassa, Gizat M. Kassie, Leila Keikavoosi-Arani, Mohammad Keykhaei, Himanshu Khajuria, Imteyaz A. Khan, Moien A.B. Khan, Yusra H. Khan, Haneen Khreis, Min Seo Kim, Adnan Kisa, Sezer Kisa, Luke D. Knibbs, Pavel Kolkhir, Somayeh Komaki, Farzad Kompani, Hamid Reza Koohestani, Ali Koolivand, Oleksii Korzh, Ai Koyanagi, Kewal Krishan, Kris J. Krohn, Naveen Kumar, Nithin Kumar, Om P. Kurmi, Ambily Kuttikkattu, Carlo La Vecchia, Judit Lám, Qing Lan, Savita Lasrado, Kamaluddin Latief, Paolo Lauriola, Sang-woong Lee, Yo Han Lee, Samson Mideksa Legesse, Jacopo Lenzi, Ming-Chieh Li, Ro-Ting Lin, Gang Liu, Wei Liu, Chun-Han Lo, László Lorenzovici, Yifei Lu, Soundarya Mahalingam, Elham Mahmoudi, Narayan B. Mahotra, Mohammad-Reza Malekpour, Ahmad Azam Malik, Tauqeer Hussain Mallhi, Deborah Carvalho Malta, Borhan Mansouri, Elezebeth Mathews, Sazan Qadir Maulud, Enkeleint A. Mechili, Entezar Mehrabi Nasab, Ritesh G. Menezes, Dechasa Adare Mengistu, Alexios-Fotios Mentis, Mahboobeh Meshkat, Tomislav Mestrovic, Ana Carolina Micheletti Gomide Nogueira de Sá, Erkin M. Mirrakhimov, Awoke Misganaw, Prasanna Mithra, Javad Moghadasi, Esmaeil Mohammadi, Mokhtar Mohammadi, Marita Mohammadshahi, Shafiu Mohammed, Syam Mohan, Nagabhishek Moka, Lorenzo Monasta, Mohammad Ali Moni, Md Moniruzzaman, Fateme Montazeri, Maryam Moradi, Ebrahim Mostafavi, Christine Mpundu-Kaambwa, Efrén Murillo-Zamora, Christopher J.L. Murray, Tapas Sadasivan Nair, Vinay Nangia, Sreenivas Narasimha Swamy, Aparna Ichalangod Narayana, Zuhair S. Natto, Biswa Prakash Nayak, Wogene Wogene Negash, Evangelia Nena, Sandhya Neupane Kandel, Robina Khan Niazi, Antonio Tolentino Nogueira de Sá, Ali Nowroozi, Chimezie Igwegbe Nzoputam, Ogochukwu Janet Nzoputam, Bogdan Oancea, Rahman Md Obaidur, Oluwakemi Ololade Odukoya, Hassan Okati-Aliabad, Akinkunmi Paul Okekunle, Osaretin Christabel Okonji, Andrew T. Olagunju, Ahmed Omar Bali, Sergej M. Ostojic, Mahesh P. A, Alicia Padron-Monedero, Jagadish Rao Padubidri, Mohammad Taha Pahlevan Fallahy, Tamás Palicz, Adrian Pana, Eun-Kee Park, Jay Patel, Rajan Paudel, Uttam Paudel, Paolo Pedersini, Marcos Pereira, Renato B. Pereira, Ionela-Roxana Petcu, Majid Pirestani, Maarten J. Postma, Akila Prashant, Mohammad Rabiee, Amir Radfar, Sima Rafiei, Fakher Rahim, Mohammad Hifz Ur Rahman, Mosiur Rahman, Muhammad Aziz Rahman, Amir Masoud Rahmani, Shayan Rahmani, Vahid Rahmanian, Prashant Rajput, Juwel Rana, Chythra R. Rao, Sowmya J. Rao, Sina Rashedi, Mohammad-Mahdi Rashidi, Zubair Ahmed Ratan, David Laith Rawaf, Salman Rawaf, Lal Rawal, Reza Rawassizadeh, Mohammad Sadegh Razeghinia, Elrashdy Moustafa Mohamed Redwan, Maryam Rezaei, Nazila Rezaei, Nima Rezaei, Mohsen Rezaeian, Mónica Rodrigues, Jefferson Antonio Buendia Rodriguez, Leonardo Roever, David Rojas-Rueda, Kristina E. Rudd, Aly M.A. Saad, Siamak Sabour, Basema Saddik, Erfan Sadeghi, Masoumeh Sadeghi, Umar Saeed, Maryam Sahebazzamani, Amirhossein Sahebkar, Harihar Sahoo, Mirza Rizwan Sajid, Sateesh Sakhamuri, Sana Salehi, Abdallah M. Samy, Milena M. Santric-Milicevic, Bruno Piassi Sao Jose, Brijesh Sathian, Maheswar Satpathy, Ganesh Kumar Saya, Subramanian Senthilkumaran, Allen Seylani, Saeed Shahabi, Masood Ali Shaikh, Mohd Shanawaz, Mohammed Shannawaz, Rahim Ali Sheikhi, Shashank Shekhar, Migbar Mekonnen Sibhat, Colin R. Simpson, Jasvinder A. Singh, Paramdeep Singh, Surjit Singh, Valentin Yurievich Skryabin, Anna Aleksandrovna Skryabina, Mohammad Sadegh Soltani-Zangbar, Suhang Song, Ireneous N. Soyiri, Paschalis Steiropoulos, Leo Stockfelt, Jing Sun, Ken Takahashi, Iman M. Talaat, Ker-Kan Tan, Nathan Y. Tat, Vivian Y. Tat, Birhan Tsegaw Taye, Pugazhenthan Thangaraju, Rekha Thapar, Friedrich Thienemann, Amir Tiyuri, Mai Thi Ngoc Tran, Jaya Prasad Tripathy, Lorainne Tudor Car, Biruk Shalmeno Tusa, Irfan Ullah, Sana Ullah, Marco Vacante, Pascual R. Valdez, Rohollah Valizadeh, Job F.M. van Boven, Tommi Juhani Vasankari, Siavash Vaziri, Francesco S. Violante, Bay Vo, Ning Wang, Melissa Y. Wei, Ronny Westerman, Nuwan Darshana Wickramasinghe, Suowen Xu, Xiaoyue Xu, Lalit Yadav, Yazachew Yismaw, Dong Keon Yon, Naohiro Yonemoto, Chuanhua Yu, Yong Yu, Ismaeel Yunusa, Mazyar Zahir, Moein Zangiabadian, Zahra Zareshahrabadi, Armin Zarrintan, Mikhail Sergeevich Zastrozhin, Zelalem Banjaw Zegeye, Yunquan Zhang, Mohsen Naghavi, Bagher Larijani, Farshad Farzadfar

**Keywords:** Asthma, Chronic obstructive pulmonary disease, Epidemiology, Interstitial lung disease, Lung disease, Morbidity, Mortality, Pneumoconiosis, Pulmonary emphysema

## Abstract

**Background:**

Updated data on chronic respiratory diseases (CRDs) are vital in their prevention, control, and treatment in the path to achieving the third UN Sustainable Development Goals (SDGs), a one-third reduction in premature mortality from non-communicable diseases by 2030. We provided global, regional, and national estimates of the burden of CRDs and their attributable risks from 1990 to 2019.

**Methods:**

Using data from the Global Burden of Diseases, Injuries, and Risk Factors Study (GBD) 2019, we estimated mortality, years lived with disability, years of life lost, disability-adjusted life years (DALYs), prevalence, and incidence of CRDs, i.e. chronic obstructive pulmonary disease (COPD), asthma, pneumoconiosis, interstitial lung disease and pulmonary sarcoidosis, and other CRDs, from 1990 to 2019 by sex, age, region, and Socio-demographic Index (SDI) in 204 countries and territories. Deaths and DALYs from CRDs attributable to each risk factor were estimated according to relative risks, risk exposure, and the theoretical minimum risk exposure level input.

**Findings:**

In 2019, CRDs were the third leading cause of death responsible for 4.0 million deaths (95% uncertainty interval 3.6–4.3) with a prevalence of 454.6 million cases (417.4–499.1) globally. While the total deaths and prevalence of CRDs have increased by 28.5% and 39.8%, the age-standardised rates have dropped by 41.7% and 16.9% from 1990 to 2019, respectively. COPD, with 212.3 million (200.4–225.1) prevalent cases, was the primary cause of deaths from CRDs, accounting for 3.3 million (2.9–3.6) deaths. With 262.4 million (224.1–309.5) prevalent cases, asthma had the highest prevalence among CRDs. The age-standardised rates of all burden measures of COPD, asthma, and pneumoconiosis have reduced globally from 1990 to 2019. Nevertheless, the age-standardised rates of incidence and prevalence of interstitial lung disease and pulmonary sarcoidosis have increased throughout this period. Low- and low-middle SDI countries had the highest age-standardised death and DALYs rates while the high SDI quintile had the highest prevalence rate of CRDs. The highest deaths and DALYs from CRDs were attributed to smoking globally, followed by air pollution and occupational risks. Non-optimal temperature and high body-mass index were additional risk factors for COPD and asthma, respectively.

**Interpretation:**

Albeit the age-standardised prevalence, death, and DALYs rates of CRDs have decreased, they still cause a substantial burden and deaths worldwide. The high death and DALYs rates in low and low-middle SDI countries highlights the urgent need for improved preventive, diagnostic, and therapeutic measures. Global strategies for tobacco control, enhancing air quality, reducing occupational hazards, and fostering clean cooking fuels are crucial steps in reducing the burden of CRDs, especially in low- and lower-middle income countries.

**Funding:**

10.13039/100000865Bill & Melinda Gates Foundation.

**Research in context**

**Evidence before this study**

The Global Burden of Diseases, Injuries, and Risk Factors Study (GBD) provides the most comprehensive measurement of epidemiological features of non-communicable diseases (NCDs) to date. Among NCDs, chronic respiratory diseases (CRDs) account for a substantial burden and premature mortality worldwide. We reviewed online medical databases by a structured search with keywords “chronic respiratory disease(s)” or “chronic obstructive pulmonary disease (COPD)” or “asthma” or “pneumoconiosis” or “interstitial lung disease (ILD)” or “pulmonary sarcoidosis” AND “prevalence” or “incidence” or “mortality” or “disability-adjusted life year(s) (DALYs)” or “epidemiology” or “risk factor(s)” or “population attributable fraction (PAF)”. The GBD Collaborator Network has published the most recent paper on the CRDs using GBD 2017. This investigation concluded that CRDs account for substantial deaths and disabilities globally, and updated population measurements are essential for monitoring the progress towards achieving the third Sustainable Development Goal (SDG) of the United Nations (UN), a one-third reduction of premature mortality from NCDs by 2030.

**Added value of this study**

As part of GBD 2019 study, this study provides updated estimates of mortality, disability, prevalence, and incidence of CRDs, including COPD, asthma, pneumoconiosis, interstitial lung disease, and pulmonary sarcoidosis from 1990-2019 in 21 GBD regions encompassing 204 countries and territories, by age, sex, and Socio-demographic Index (SDI). The DALYs and deaths attributed to potentially modifiable behavioural, environmental and occupational, and metabolic risk factors are also reported. This is the first report published by the GBD Collaborator Network reporting the global and regional burden of sub-causes of pneumoconiosis, i.e. silicosis, asbestosis, coal worker's pneumoconiosis, and other pneumoconiosis. It is also the first to describe the attributable burden of CRDs to high body-mass index (BMI). Furthermore, this is the first cycle of GBD investigating the burden due to non-optimal temperature, which accentuates the potential role of climate change in disability and deaths from CRDs.

**Implications of all the available evidence**

Results provided in this study reflect the impacts of the so far adopted strategies and shed light on the future location-specific policies that need to be established for reducing the burden resulting from CRDs by identifying the populations with the highest burden and the most influential risk factors. The high burden of deaths and disabilities from CRDs in low-middle income countries emphasises the crucial role of prevention, raising public awareness, specialised respiratory care training for the healthcare providers, and enhancing access to diagnostic tools as well as treatments in these countries. Future primary research is also essential for obtaining a more accurate picture of the current burden of CRDs in this region. Since smoking was the primary risk factor responsible for deaths and DALYs from CRDs, full enforcement of tobacco control programmes, especially in the Caribean region, is imperative in future policies. Given the high burden attributed to household air pollution in the Sub-Saharan region and low SDI countries, particularly in women, increased focus should be directed to promoting clean cooking and heating energies in this region. Ultimately, in addition to the aforementioned measures, global strategies for improving air quality and limiting occupational hazards are key steps in achieving the third UN SDG.

## Introduction

Chronic respiratory disease (CRD) is an umbrella term describing conditions affecting the lungs and airways, including chronic obstructive pulmonary disease (COPD), asthma, pneumoconiosis, interstitial lung disease (ILD), and pulmonary sarcoidosis. CRD, being the third leading cause of mortality globally in 2019, is associated with a substantial burden and cost.^1^, ^2^, ^3^ The sustainable development goal (SDG) target 3.4, defined by the United Nations (UN), is a one-third reduction of premature mortality from non-communicable diseases (NCDs), including CRDs, by 2030.^4^ The World Health Organization (WHO) is the principal coordinating body for the implementation of health-related SDGs, and its strategy for the period 2019–2023 outlines three key goals: one billion more individuals enjoying better health and well-being, universal health coverage, and enhanced protection against health emergencies.^5^ In addition to its efforts in monitoring health-related indicators,^6^ the WHO has also established the global action plan (GAP) for healthy lives and well-being for all (SDG3 GAP) to improve collaboration between the prominent actors in the multilateral system to accelerate progress towards health-related SDGs targets.^7^ While such programmes aim to promote health in all aspects, mitigating endeavors specific to CRDs have been undertaken as well. The WHO Global Alliance against Chronic Respiratory Diseases (GARD),^8^ in addition to focused Global Initiatives for COPD (GOLD)^9^ and Asthma (GINA),^10^ have been established to reduce the burden of CRDs.

The latest report on the global prevalence and attributable health burden of CRDs has been conducted using the Global Burden of Diseases, Injuries, and Risk Factors Study (GBD) 2017.^11^ Newly available data sources, locations, several risk factors, and some analytical changes lead to more precise estimations in the updated GBD 2019. Environmental and occupational risks and smoking are the leading risk factors of CRDs, with various distributions by geographical location, culture, age, and sex. Understanding the trend of these risk factors and identification of the at-risk populations can help policymakers in developing and efficiently targeting risk modification interventions, which can result in reduced disability and premature mortality.

Using the GBD 2019 study, we described the burden of CRDs and attributable risk factors by sex, age, and Socio-demographic Index (SDI) on global, regional, and national levels as well as their trends from 1990 to 2019. This report aims to picture the overview of the current burden of CRDs. We drafted this manuscript as part of the GBD Collaborator Network under the guidance of the GBD protocol. The ultimate objective is to highlight the most prominent risk factors and at-risk populations to help caregivers and policymakers to develop targeted risk reduction measures effectively. This work updates all past GBD estimates of CRDs.^11^^,^^12^

## Methods

### Overview

The GBD is an international collaborative effort determining the burden of 369 diseases and injuries and 87 risk factors in 204 countries and territories, which are categorised into 21 regions and seven super-regions, from 1990. The results are available from the GBD online results tool and can be viewed interactively via the GBD compare tool. The detailed process of burden estimation for CRDs and risk factors is previously reported^1^^,^^13^ and included in appendix 1. We obtained the data in this study from GBD 2019 public datasets available from http://ghdx.healthdata.org/gbd-results-tool (accessed on July 1st, 2021).

This study follows the Guidelines for Accurate and Transparent Health Estimates Reporting (GATHER) (appendix 1 pp 138–139).

### Case definition

Standard definitions are used for each cause. According to the GOLD classification, COPD is defined as a measurement of <0.7 one second of forceful exhalation/total forced expiration (FEV1/FVC) on spirometry following bronchodilation. Other alternative definitions, including GOLD pre-bronchodilation, Lower Limit of Normal (LLN) post-bronchodilation, LLN pre-bronchodilation, and European Respiratory Society (ERS) guidelines are also included. Pneumoconiosis is defined as a chronic lung disease marked by lung scarring and other interstitial injuries. Pneumoconiosis includes silicosis, asbestosis, coal worker's pneumoconiosis, and other pneumoconiosis. Asthma is a chronic lung disease marked by spasms in the bronchi usually resulting from an allergic reaction or hypersensitivity and causing difficulty in breathing. We define asthma as a diagnosis established by a physician in addition to wheezing in the past year. The alternative definitions include self-reported asthma in the past year or ever, only a doctor's diagnosis, or only wheezing in the past year due to exposure to dust and other containments. The American Thoracic Society criteria are used as the standard definition for ILD. ILD and pulmonary sarcoidosis are CRDs that damage lung function and oxygen uptake via inflammation and/or scarring. The list of other CRDs and relevant International Classification of Diseases (ICD)-10 and ICD-9 codes are available in appendix 1.

### Fatal estimates

Mortality data for CRDs (the parent cause) were retrieved from vital registries, verbal autopsies (household mortality surveys), and surveillance data. Verbal autopsies data were not incorporated in the fatal estimation of child causes. We pooled and standardised the input data based on different coding systems, representativeness, completeness, age and sex aggregation, and misclassification of maternal and HIV/AIDS deaths. Various linear mixed-effect models and spatiotemporal Gaussian process regression models were created using the Cause of Death Ensemble model (CODEm) framework accounting for location-specific covariates.^1^^,^^13^ We used CoDCorrect analysis to adjust and ensure the internal consistency of the results from the CODEm model. Multiplication of the estimated number of deaths by the standard life expectancy at the age of death resulted in years of life lost (YLL).

### Nonfatal estimates

Nonfatal estimates include incidence, prevalence, and years lived with disability (YLD). Input data were obtained from hospital claims, literature identified by a systematic review, population-representative surveys, and medical expenditure panel surveys. Hospital inpatient and insurance data were the primary data sources used for pneumoconiosis and ILD and pulmonary sarcoidosis. After data adjustment, estimation of prevalence and incidence by cause and sequela was performed using DisMod-MR 2.1, a Bayesian meta-regression method, and included incorporation of severity distributions, disability weights, and comorbidity adjustment of the sequela. YLD was estimated by combining prevalence and incidence of causes and sequela with levels of severity related to disability using disability weights while adjusting for comorbidity. Modeling other CRDs together in a DisMod-MR model would not generate reliable estimates of outcome due to the variability of these diseases in their underlying causes, risk factors, and associated health outcomes. The YLD from other CRDs was calculated by multiplying the YLDs/YLLs ratio calculated across the specified CRDs by the YLL estimated for other CRDs.

### Risk estimates

We used the comparative risk assessment (CRA) framework to measure attributable burden, which is the quantity of current burden that would have been reduced in case the past population's exposure had changed to the theoretical minimum risk exposure level (TMREL).^14^^,^^15^ We modeled the attributed burden by (1) estimating the relative risk (RR) of the risk–outcome pairs, (2) exposure estimation, (3) establishing the TMREL, (4) calculating population attributable fraction, (5) estimation of RR-weighted prevalence of exposure (summary exposure value), and (6) aggregating risk factors and accounting for their mediation.

GBD risk factors are classified into a risk hierarchy containing four levels, from Level 1, i.e. general categories (behavioural, environmental/occupational, and metabolic), to level 4, i.e. the most specific (such as ambient particulate matter (PM) pollution).^11^

The risk–outcome pairs were included if convincing or probable evidence was available according to the World Cancer Research Fund grading system. Risk factors for COPD include environmental/occupational risks, i.e. ambient PM pollution, ambient ozone pollution, occupational PM, gases, and fumes, household air pollution from solid fuels, and non-optimal temperature, and behavioural risks, i.e. smoking and secondhand smoke. Asthma risk factors include environmental/occupational risks, i.e. occupational asthmagens, behavioral risks, i.e. smoking, and metabolic risks, i.e. high body-mass index (BMI). Pneumoconiosis risk factors comprise environmental/occupational risks, i.e. occupational exposure to silica, asbestos, and occupational PM, gases, and fumes. All risk factors were reported at the most specific level, except for non-optimal temperature, which is a level 2 risk. No risk factors were included for ILD and pulmonary sarcoidosis, and other CRDs.

### Decomposition analysis

Using decomposition analysis, we estimated the contribution of the age-specific CRD incidence rates changes while controlling for population size, sex distribution, and age structure.^16^ In scenario 1, we accounted for population growth by applying the population size of 2019 onto the rate, sex, and age structure of 1990. The difference between the number of incident cases in 1990 and the estimated numbers in this scenario results only from population growth. In scenario 2, we applied the 1990 age-sex specific rates to the 2019 age-sex specific population numbers to account for both population growth and change in age structure. The difference between the number of incident cases in 2019 and the numbers estimated in the second scenario is due to a change in age-sex specific rates of CRD incidence. We reported the contribution of each factor to the overall change of the new cases as the percent of change (appendix 2 Table S3).

### Socio-demographic Index (SDI)

The SDI, ranging from 0 to 100, indicates socio-demographic development by incorporating lagged distributed income per capita, average years of education, and total fertility rate.^17^ We used the SDI to classify the 204 GBD countries and territories into quintiles.

### Statistical analysis

We calculated age-standardised rates (ASRs) by the GBD global standard population.^17^ Point estimates are presented with 95% uncertainty interval (UI), and rates are reported per 100 000 populations. 95% UIs were estimated using the 25th and 975th ordered values among 1000 draws in each computational stage.

### Role of the funding source

The funders of the study had no role in study design, data collection, data analysis, data interpretation, or the writing of the report. The corresponding author had full access to the data in the study and final responsibility for the decision to submit for publication.

## Results

### Total CRDs

**Fig. 1:**
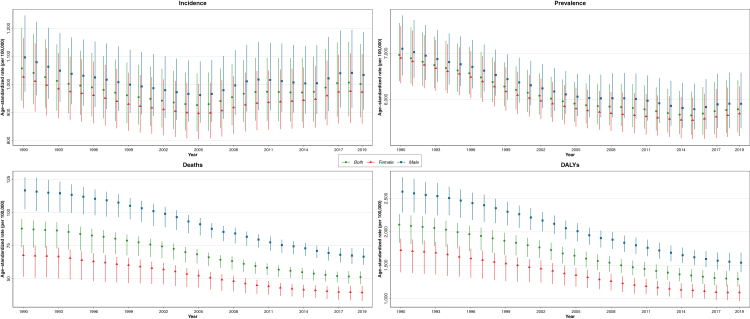
**Global age-standardised rates of incidence, prevalence, deaths, and DALYs of chronic respiratory diseases in men, women, and in both sexes combined, 1990–2019.** DALYs = Disability-Adjusted Life Years.

**Fig. 2:**
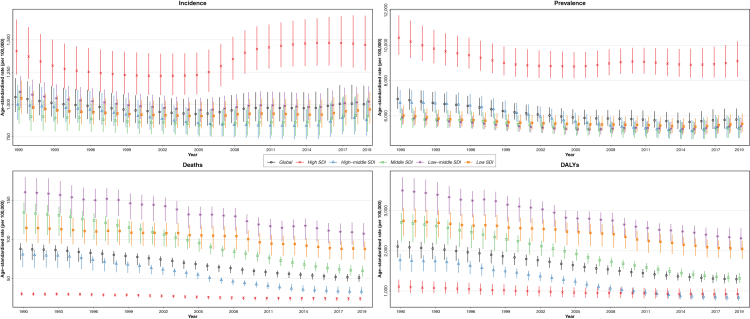
**Global age-standardised rates of incidence, prevalence, deaths, and DALYs of chronic respiratory diseases in both sexes combined in each SDI quintile.** DALYs = Disability-Adjusted Life Years, SDI = Socio-demographic Index.

**Fig. 3:**
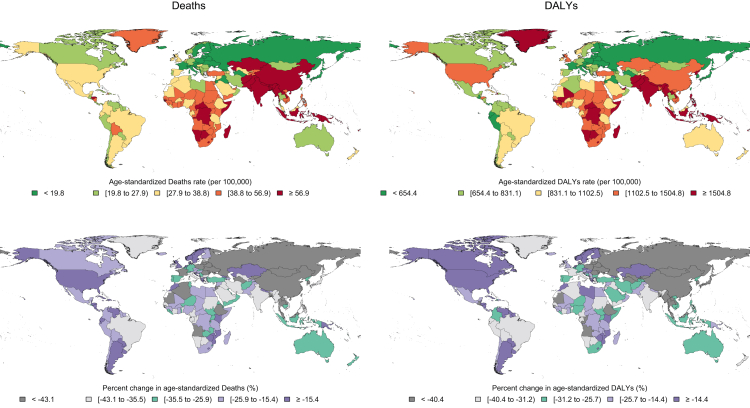
**Global age-standardised rate of deaths and DALYs from chronic respiratory diseases in 2019 and their percent change from 1990 in both sexes combined.** DALYs = Disability-Adjusted Life Years.

In 2019, the CRDs were the third leading cause of mortality, accounting for 4.0 million (95% UI 3.6–4.3) deaths globally. The ASR of mortality has steadily decreased by 41.7% (32.2%–47.6%) from 1990 to 2019 (Table 1). The ASR of mortality was higher in men throughout the investigated period and 1.7 of that of women in 2019 (Fig. 1). Among 21 GBD regions, Oceania, followed by South Asia, had the highest ASR of mortality, while high-income Asia Pacific, followed by Eastern Europe, had the lowest in 2019 (appendix 2, Table S5). From 1990 until 2019, the ASR of mortality decreased significantly in all SDI quintiles, and high SDI and low-middle SDI countries had the lowest and highest estimates, respectively (Fig. 2). Nepal had the highest ASR of mortality from CRDs in 2019 (231.2 [175.8–270.3]), and Singapore had the largest reduction in this rate from 1990 (80.5% [72.0%–83.4%]) among 204 countries and territories (Fig. 3).

**Table 1:** Global incidence, prevalence, deaths, DALYs, YLLs, and YLDs from chronic respiratory diseases.

Measure	Age (metric)	Year	CRDs	Cause specific				
				COPD	Pneumoconiosis	Asthma	ILD & pulmonary sarcoidosis	Other CRDs
**Incidence**	All ages (number)	% Change^a^	49.0 (42.1 to 55.6)	85.9 (82.3 to 89.2)	61.5 (44.6 to 77.6)	15.0 (11.7 to 18.0)	118.6 (110.2 to 127.0)	
		2019	77,625,300 (68,884,564 to 87,929,749)	16,214,828 (15,224,111 to 17,220,809)	199,125 (172,556 to 228,809)	36,979,267 (29,601,976 to 45,928,112)	24,232,080 (19,609,750 to 29,463,387)	
	Age-standardised (rate per 100,000)	% Change	−5.3 (−7.1 to −3.6)	−7.4 (−8.8 to −5.9)	−13.7 (−21.3 to −6.6)	−13.1 (−16.3 to −10.2)	14.1 (11.1 to 17.3)	
		2019	1001.6 (883.0 to 1144.4)	200.5 (188.6 to 212.6)	2.4 (2.1 to 2.7)	504.3 (400.6 to 633.3)	294.4 (238.5 to 356.6)	
**Prevalence**	All ages (number)	% Change	39.8 (36.3 to 43.2)	84.8 (81.6 to 88.0)	83.9 (62.1 to 102.9)	15.6 (12.7 to 18.9)	114.2 (106.4 to 122.1)	
		2019	454,557,390 (417,354,403 to 499,144,380)	212,335,951 (200,422,146 to 225,097,834)	3,072,550 (2,596,999 to 3,596,518)	262,405,182 (224,047,914 to 309,452,681)	4,710,180 (4,020,397 to 5,401,700)	
	Age-standardised (rate per 100,000)	% Change	−16.9 (−18.5 to −15.1)	−8.7 (−10.2 to −7.3)	−8.4 (−19.1 to 0.3)	−24.0 (−27.2 to −20.8)	9.4 (6.1 to 12.9)	
		2019	5789.2 (5290.7 to 6418.1)	2638.2 (2492.2 to 2796.1)	36.8 (31.1 to 43.1)	3415.5 (2898.9 to 4066.2)	57.6 (49.4 to 65.7)	
**Deaths**	All ages (number)	% Change	28.5 (15.2 to 50.1)	30.2 (15.7 to 55.0)	−3.0 (−19.2 to 29.1)	0.2 (−14.2 to 15.1)	166.6 (93.0 to 241.0)	52.3 (29.1 to 82.9)
		2019	3,974,315 (3,581,757 to 4,303,823)	3,280,636 (2,902,855 to 3,572,367)	23,015 (20,348 to 26,159)	461,069 (366,580 to 559,006)	169,833 (118,756 to 204,802)	39,761 (31,085 to 46,581)
	Age-standardised (rate per 100,000)	% Change	−41.7 (−47.6 to −32.2)	−41.7 (−48.0 to −31.1)	−53.3 (−60.9 to −38.6)	−51.3 (−59.1 to −43.7)	23.4 (−13.1 to 58.6)	−19.5 (−30.2 to −6.0)
		2019	51.3 (45.9 to 55.5)	42.5 (37.6 to 46.3)	0.3 (0.3 to 0.3)	5.8 (4.6 to 7.0)	2.2 (1.5 to 2.6)	0.5 (0.4 to 0.6)
**DALYs**	All ages (number)	% Change	20.8 (12.1 to 36.1)	25.6 (15.1 to 46.0)	11.2 (−6.1 to 38.1)	−3.5 (−10.8 to 4.5)	122.9 (79.4 to 168.6)	76.5 (48.2 to 104.5)
		2019	103,533,107 (94,792,077 to 112,266,452)	74,432,367 (68,204,127 to 80,193,347)	919,077 (761,478 to 1,116,127)	21,550,977 (17,141,587 to 26,971,997)	3,770,894 (2,864,234 to 4,468,319)	2,859,792 (2,461,295 to 3,217,791)
	Age-standardised (rate per 100,000)	% Change	−38.6 (−43.3 to −30.9)	−39.8 (−44.9 to −30.2)	−44.4 (−52.9 to −31.2)	−42.5 (−48.5 to −36.6)	11.7 (−10.8 to 35.1)	13.8 (−2.0 to 31.0)
		2019	1293.7 (1183.0 to 1403.6)	926.1 (848.8 to 997.7)	11.1 (9.2 to 13.5)	273.6 (216.7 to 343.4)	46.4 (35.1 to 55.0)	36.5 (31.4 to 41.1)
**YLLs**	All ages (number)	% Change	8.8 (−1.7 to 27.2)	11.9 (−0.5 to 35.1)	−18.2 (−33.5 to 11.9)	−15.8 (−24.5 to −3.6)	124.8 (75.1 to 178.8)	32.0 (4.1 to 67.0)
		2019	71,145,745 (64,700,056 to 77,011,749)	54,594,898 (48,711,468 to 59,513,367)	479,340 (418,214 to 550,546)	11,354,712 (9,279,939 to 13,372,007)	3,291,056 (2,406,555 to 3,952,188)	1,425,739 (1,135,697 to 1,693,896)
	Age-standardised (rate per 100,000)	% Change	−46.5 (−51.7 to −37.2)	−46.8 (−52.6 to −36.1)	−58.9 (−66.3 to −44.0)	−53.5 (−59.0 to −46.5)	12.3 (−13.1 to 39.7)	−17.7 (−31.9 to 1.7)
		2019	885.9 (805.6 to 959.4)	680.8 (606.4 to 741.6)	5.8 (5.1 to 6.7)	140.6 (115.3 to 165.3)	40.6 (29.7 to 48.8)	18.0 (14.2 to 21.4)
**YLDs**	All ages (number)	% Change	59.4 (51.9 to 67.3)	89.4 (85.4 to 93.6)	82.9 (61.1 to 101.9)	15.4 (12.7 to 18.7)	110.4 (102.4 to 119.0)	165.8 (157.8 to 172.7)
		2019	32,387,362 (26,116,058 to 38,488,142)	19,837,469 (16,596,490 to 22,441,727)	439,737 (292,559 to 625,475)	10,196,265 (6,654,649 to 15,061,355)	479,838 (321,777 to 690,617)	1,434,053 (1,173,488 to 1,649,383)
	Age-standardised	% Change	−9.9 (−12.2 to −7.7)	−4.9 (−6.6 to −3.0)	−8.6 (−19.0 to 0.5)	−23.4 (−26.6 to −20.2)	8.1 (4.8 to 11.6)	81.3 (74.5 to 86.7)
	(rate per100,000)	2019	407.9 (327.4 to 486.9)	245.3 (205.2 to 276.8)	5.3 (3.5 to 7.5)	133.0 (86.9 to 197)	5.9 (3.9 to 8.4)	18.5 (15.1 to 21.3)

Data in parentheses are 95% Uncertainty Intervals (95% UIs).

CRDs = Chronic Respiratory Diseases; COPD = Chronic Obstructive Pulmonary Disease; ILD = Interstitial Lung Disease; DALYs = Disability-Adjusted Life Years; YLLs = Years of Life Lost; YLDs = Years Lived with Disability.

a % Change (1990–2019).

The CRDs were responsible for 103.5 million (94.8–112.3) DALYs constituting 4.1% (3.7%–4.4%) of global DALYs for all causes in 2019 (not shown). YLLs accounted for 68.5% of the ASR of DALYs in 2019 (appendix 2, Fig. S9). The ASR of DALYs has decreased by 38.6% (30.9%–43.3%) globally from 1990 to 2019. Throughout 1990–2019, the ASR of DALYs has been higher in men (Fig. 1). Oceania, followed by South Asia, had the highest ASR of DALYs while high-income Asia Pacific and Eastern Europe had the lowest. The ASR of DALYs decreased in all SDI quintiles from 1990 to 2019, with middle and high-middle SDI countries experiencing the largest decrease (appendix 2, Table S2). Singapore had the largest reduction in the ASR of DALYs due to CRDs from 1990 (68.3% [61.8%–72.0%]) (Fig. 3).

In 2019, 454.6 million (417.4–499.1) people were estimated to suffer from CRD. The ASR of prevalence has decreased by 16.9% (15.1%–18.5%) from 1990 to 2019. No significant difference has been found between the sexes throughout the investigated period in the ASR of prevalence. High-income North America, followed by Australasia, had the highest ASR of prevalence, while East and Central Asia had the lowest. The high SDI quintile had the highest ASR of prevalence throughout the investigated period, while it was comparable among other SDI quintiles. All SDI quintiles had a lower ASR of prevalence in 2019 than in 1990 (Fig. 2).

In 2019, 77.6 million (68.9–87.9) new cases of CRDs were estimated globally, which has increased by 49.0% (42.1%–55.6%) from 1990. Decomposition analysis showed that population growth, responsible for 91.0% of the increased crude incidence number (44.6% out of 49.0%), had been the main driving force (appendix 2, Table S3). However, the ASR of incidence has decreased by 5.3% (3.6%–7.1%) from 1990 to 2019. No significant difference has been found between the sexes throughout the investigated period in the ASR of incidence (Fig. 1). High-income North America had the highest ASR of incidence in 2019, whereas Western Europe and East Asia had the lowest. Similar to prevalence, the high SDI had the highest ASR of incidence from 1990 until 2019, while it was comparable among other SDI quintiles.

**Fig. 4:**
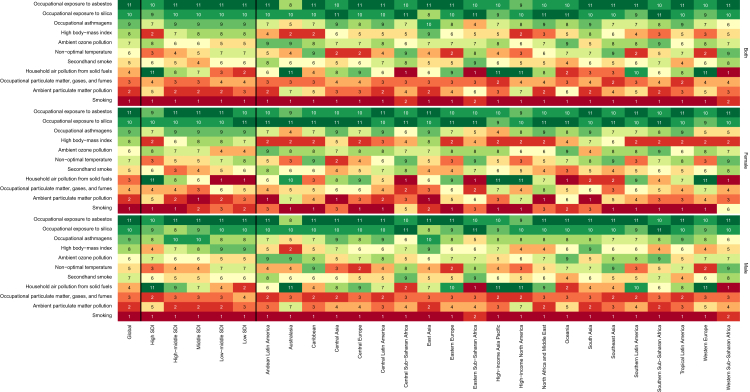
**Ranked contribution of risk factors to the age-standardised rate of DALYs from chronic respiratory diseases by region, 2019, for both sexes combined, females, and males.** Risk factors are ranked from 1 (the leading risk factor for age-standardised Disability-Adjusted Life Years (DALYs); dark red) to 11 (the lowest risk factor for age-standardised DALYs; dark green). The numbers inside each box indicate the ranking.

From total DALYs and deaths due to CRDs in 2019, 62.0% and 69.6% were attributed to all risk factors (not shown). Globally, smoking was the primary risk factor responsible for the ASR of DALYs from CRDs followed by ambient PM pollution (Table 2). The major risk factors varied in different regions. Household air pollution from solid fuels was the leading risk factor accounting for DALYs and death in Central, Western, and Eastern Sub-Saharan Africa. The burden attributed to ambient PM pollution was the lowest in the high SDI quintile (Fig. 4, appendix 2, Fig. S15).

**Table 2:** Global age-standardised rates (per 100,000) of deaths and DALYs from chronic respiratory diseases attributed to risk factors in both sexes combined with percent change.

Measure	Year	Risk factor	CRDs	Cause specific		
				COPD	Pneumoconiosis	Asthma
**Deaths**	**% Change**^a^	**Environmental/occupational risks**	−52.1 (−58.1 to −42.3)	−52.0 (−58.2 to −42.2)	−53.3 (−60.9 to −38.6)	−55.1 (−62.3 to −45.6)
		Air pollution	−57.4 (−64.3 to −47.8)	−57.4 (−64.3 to −47.8)		
		Particulate matter pollution	−61.8 (−68.4 to −52.2)	−61.8 (−68.4 to −52.2)		
		Ambient particulate matter pollution	−12.1 (−39.9 to 32.0)	−12.1 (−39.9 to 32.0)		
		Household air pollution from solid fuels	−80.9 (−85.8 to −74.1)	−80.9 (−85.8 to −74.1)		
		Ambient ozone pollution	−21.6 (−31.6 to −6.3)	−21.6 (−31.6 to −6.3)		
		Non-optimal temperature	−49.7 (−57.1 to −35.2)	−49.7 (−57.1 to −35.2)		
		Occupational risks	−46.9 (−53.8 to −35.2)	−45.9 (−53.0 to −34.2)	−53.3 (−60.9 to −38.6)	−55.1 (−62.3 to −45.6)
		Occupational carcinogens	−52.0 (−61.1 to −33.5)		−52.0 (−61.1 to −33.5)	
		Occupational exposure to asbestos	15.6 (−8.5 to 33.6)		15.6 (−8.5 to 33.6)	
		Occupational exposure to silica	−58.9 (−67.9 to −39.4)		−58.9 (−67.9 to −39.4)	
		Occupational asthmagens	−55.1 (−62.3 to −45.6)			−55.1 (−62.3 to −45.6)
		Occupational particulate matter, gases, and fumes	−46.1 (−53.1 to −34.2)	−45.9 (−53 to −34.2)	−56.5 (−63.5 to −40.9)	
		**Behavioral risks**	−45.7 (−51.9 to −37.2)	−45.0 (−51.3 to −36.2)		−62.5 (−69.5 to −54.8)
		Tobacco	−45.7 (−51.9 to −37.2)	−45.0 (−51.3 to −36.2)		−62.5 (−69.5 to −54.8)
		Smoking	−44.9 (−51.4 to −36.7)	−44.0 (−50.5 to −35.8)		−62.5 (−69.5 to −54.8)
		Secondhand smoke	−51.8 (−58.7 to −38.0)	−51.8 (−58.7 to −38.0)		
		**Metabolic risks**	−20.1 (−35.6 to 5.2)			−20.1 (−35.6 to 5.2)
		High body-mass index	−20.1 (−35.6 to 5.2)			−20.1 (−35.6 to 5.2)
	**2019**	**Environmental/occupational risks**	24.2 (20.6 to 27.5)	23.5 (20.0 to 26.8)	0.3 (0.3 to 0.3)	0.4 (0.3 to 0.5)
		Air pollution	16.8 (13.3 to 20.3)	16.8 (13.3 to 20.3)		
		Particulate matter pollution	14.0 (10.9 to 17.4)	14.0 (10.9 to 17.4)		
		Ambient particulate matter pollution	9.0 (7.1 to 11.1)	9.0 (7.1 to 11.1)		
		Household air pollution from solid fuels	5.1 (3.0 to 7.8)	5.1 (3.0 to 7.8)		
		Ambient ozone pollution	4.7 (2.2 to 7.3)	4.7 (2.2 to 7.3)		
		Non-optimal temperature	5.1 (4.0 to 6.3)	5.1 (4.0 to 6.3)		
		Occupational risks	7.3 (5.9 to 8.9)	6.6 (5.2 to 8.2)	0.3 (0.3 to 0.3)	0.4 (0.3 to 0.5)
		Occupational carcinogens	0.2 (0.2 to 0.2)		0.2 (0.2 to 0.2)	
		Occupational exposure to asbestos	0 (0 to 0.1)		0 (0 to 0.1)	
		Occupational exposure to silica	0.2 (0.1 to 0.2)		0.2 (0.1 to 0.2)	
		Occupational asthmagens	0.4 (0.3 to 0.5)			0.4 (0.3 to 0.5)
		Occupational particulate matter, gases, and fumes	6.7 (5.3 to 8.3)	6.6 (5.2 to 8.2)	0.1 (0.1 to 0.1)	
		**Behavioral risks**	23.1 (20.3 to 25.8)	22.5 (19.7 to 25.0)		0.7 (0.4 to 1.0)
		Tobacco	23.1 (20.3 to 25.8)	22.5 (19.7 to 25.0)		0.7 (0.4 to 1.0)
		Smoking	21.1 (18.8 to 23.4)	20.4 (18.1 to 22.6)		0.7 (0.4 to 1.0)
		Secondhand smoke	3.6 (1.9 to 5.5)	3.6 (1.9 to 5.5)		
		**Metabolic risks**	0.9 (0.5 to 1.5)			0.9 (0.5 to 1.5)
		High body-mass index	0.9 (0.5 to 1.5)			0.9 (0.5 to 1.5)
**DALYs**	**% Change**^a^	**Environmental/occupational risks**	−51.6 (−57.2 to −42.9)	−52.0 (−57.7 to −43.0)	−44.4 (−52.9 to −31.2)	−45.9 (−52.8 to −38.7)
		Air pollution	−56.9 (−63.3 to −47.6)	−56.9 (−63.3 to −47.6)		
		Particulate matter pollution	−60.0 (−66.2 to −50.7)	−60.0 (−66.2 to −50.7)		
		Ambient particulate matter pollution	−8.8 (−35.8 to 34.2)	−8.8 (−35.8 to 34.2)		
		Household air pollution from solid fuels	−79.4 (−84.4 to −72.6)	−79.4 (−84.4 to −72.6)		
		Ambient ozone pollution	−26.5 (−36.2 to −11.0)	−26.5 (−36.2 to −11.0)		
		Non-optimal temperature	−55.1 (−62.7 to −40.5)	−55.1 (−62.7 to −40.5)		
		Occupational risks	−44.7 (−50.5 to −34.4)	−44.5 (−50.7 to −33.5)	−44.4 (−52.9 to −31.2)	−45.9 (−52.8 to −38.7)
		Occupational carcinogens	−40.9 (−51.3 to −25.9)		−40.9 (−51.3 to −25.9)	
		Occupational exposure to asbestos	−6.1 (−18.3 to 5.9)		−6.1 (−18.3 to 5.9)	
		Occupational exposure to silica	−43.3 (−54.2 to −27.1)		−43.3 (−54.2 to −27.1)	
		Occupational asthmagens	−45.9 (−52.8 to −38.7)			−45.9 (−52.8 to −38.7)
		Occupational particulate matter, gases, and fumes	−44.7 (−50.8 to −33.6)	−44.5 (−50.7 to −33.5)	−54.3 (−61.5 to −39.9)	
		**Behavioral risks**	−45.5 (−50.8 to −37.9)	−44.5 (−49.8 to −36.7)		−59.6 (−65.3 to −54.1)
		Tobacco	−45.5 (−50.8 to −37.9)	−44.5 (−49.8 to −36.7)		−59.6 (−65.3 to −54.1)
		Smoking	−45.2 (−50.8 to −37.8)	−43.9 (−49.7 to −36.4)		−59.6 (−65.3 to −54.1)
		Secondhand smoke	−49.3 (−55.1 to −37.5)	−49.3 (−55.1 to −37.5)		
		**Metabolic risks**	−11.9 (−26.1 to 8.9)			−11.9 (−26.1 to 8.9)
		High body-mass index	−11.9 (−26.1 to 8.9)			−11.9 (−26.1 to 8.9)
	**2019**	**Environmental/occupational risks**	510.9 (446.5 to 574.1)	476.9 (411.7 to 538.6)	11.1 (9.2 to 13.5)	22.9 (18.2 to 28.2)
		Air pollution	349.8 (280.2 to 413.2)	349.8 (280.2 to 413.2)		
		Particulate matter pollution	305.0 (239.7 to 369.2)	305.0 (239.7 to 369.2)		
		Ambient particulate matter pollution	190.8 (153.5 to 234.8)	190.8 (153.5 to 234.8)		
		Household air pollution from solid fuels	114.2 (69.8 to 172.4)	114.2 (69.8 to 172.4)		
		Ambient ozone pollution	77.0 (37.0 to 119.5)	77.0 (37.0 to 119.5)		
		Non-optimal temperature	77.9 (59.9 to 96.8)	77.9 (59.9 to 96.8)		
		Occupational risks	177.0 (151.8 to 203.6)	143.0 (118.6 to 168.7)	11.1 (9.2 to 13.5)	22.9 (18.2 to 28.2)
		Occupational carcinogens	8.8 (7.1 to 10.9)		8.8 (7.1 to 10.9)	
		Occupational exposure to asbestos	0.9 (0.7 to 1.0)		0.9 (0.7 to 1.0)	
		Occupational exposure to silica	7.9 (6.2 to 10.0)		7.9 (6.2 to 10.0)	
		Occupational asthmagens	22.9 (18.2 to 28.2)			22.9 (18.2 to 28.2)
		Occupational particulate matter, gases, and fumes	145.4 (120.9 to 171.1)	143 (118.6 to 168.7)	2.3 (1.9 to 2.9)	
		**Behavioral risks**	495.4 (444.3 to 546.0)	469.9 (418.1 to 519.1)		25.5 (13.6 to 36.2)
		Tobacco	495.4 (444.3 to 546.0)	469.9 (418.1 to 519.1)		25.5 (13.6 to 36.2)
		Smoking	449.5 (403.9 to 493.6)	424.0 (380.2 to 465.7)		25.5 (13.6 to 36.2)
		Secondhand smoke	78.8 (39.2 to 118.7)	78.8 (39.2 to 118.7)		
		**Metabolic risks**	44.8 (26.4 to 68.6)			44.8 (26.4 to 68.6)
		High body-mass index	44.8 (26.4 to 68.6)			44.8 (26.4 to 68.6)

Data in parentheses are 95% Uncertainty Intervals (95% UIs).

COPD = Chronic Obstructive Pulmonary Disease, CRDs = Chronic Respiratory Diseases, DALYs = Disability-Adjusted Life Years.

a % Change (1990–2019).

### COPD

With 212.3 million (200.4–225.1) prevalent cases and 16.2 million (15.2–17.2) new cases, COPD accounted for 3.3 million (2.9–3.6) deaths globally in 2019. Among CRDs, COPD has been the main contributor to the global ASR of DALYs and mortality. The ASR of prevalence, incidence, deaths, and DALYs have significantly decreased from 1990 to 2019 by 8.7% (7.3%–10.2%), 7.4% (5.9%–8.8%), 41.7% (31.1%–48.0%), and 39.8% (30.2%–44.9%), respectively. Men have had higher ASRs of prevalence, deaths, DALYs, and incidence throughout the investigated period (appendix 2, Fig. S17). COPD constituted the majority of new and prevalent cases in the older than 35 and 50 age groups, respectively, and the incidence and prevalence rates increased with aging globally (appendix 2, Figs. S6–S8).

In 2019, high-income North America had the highest ASR of prevalence, but Oceania had the highest ASR of incidence, deaths, and DALYs. The ASR of deaths and DALYs dropped in all SDI quintiles in 2019 than 1990. The low-middle SDI quintile has had the highest ASR of deaths and DALYs throughout the investigated period, while the high SDI quintile has had the lowest. Compared to other SDI quintiles, the ASR of prevalence has been the lowest in low SDI countries from 1990 to 2019. Nevertheless, from 2010, it has been comparable between high-middle SDI and low SDI quintiles. Moreover, the highest ASR of incidence has been observed in low-middle SDI countries from 1990 to 2019 (appendix 2, Fig. S18). The ASR of prevalence has slightly increased in low SDI countries (2.1% [0.6%–3.4%]) while it has decreased in other SDI quintiles from 1990 to 2019.

Globally, smoking was the most prevalent risk factor of COPD and was responsible for 424.0 (380.2–465.7) ASR of DALYs and 20.4 (18.1–22.6) ASR of deaths followed by ambient PM pollution. While these risk factors were common between sexes, the third most prevalent risk factors were occupational PM, gases, and fumes and household air pollution from solid fuels in men and women, respectively. Geographical location and socio-demographic status also affected the distribution of the risk factors. In contrast to other SDI quintiles, where smoking had the highest attributable ASR of DALYs, household air pollution from solid fuels was the leading risk factor in low SDI countries, accounting for 531.8 (343.2–744.9) ASR of DALYs. Interestingly, in high SDI countries, non-optimal temperature was the second most prevalent risk factor following smoking (appendix 2, Figs. S19 and S20).

### Asthma

**Fig. 5:**
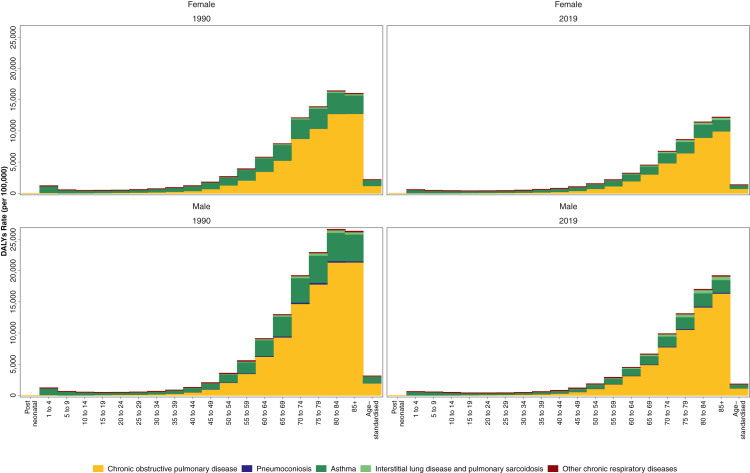
**Absolute rate of DALYs from chronic respiratory diseases by age in men and women in 1990 and 2019 with age-standardised rate.** DALYs = Disability-Adjusted Life Years.

Asthma accounted for 21.6 million (17.1–27.0) DALYs globally in 2019 with 262.4 million (224.1–309.5) prevalent cases and 37.0 million (29.6–45.9) new cases. Asthma has been the main contributor to the global ASR of prevalence and incidence of CRDs. All measures were closely comparable between the sexes (appendix 2, Fig. S21). The ASR of incidence, prevalence, deaths, and DALYs have significantly decreased from 1990 to 2019 by 13.1% (10.2%–16.3%), 24.1% (20.8%–27.2%), 51.3% (43.7%–59.1%), and 42.5% (36.6%–48.5%), respectively. Asthma constituted the majority of DALYs in the under 35 age group, with the highest incidence rate in the 1–4 years age group (1884.6 [1183.7–2879.0]) in 2019 worldwide (Fig. 5, appendix 2, Figs. S6–S9).

In 2019, high-income North America had the highest ASR of prevalence and incidence, whereas Oceania had the highest ASR of death and DALYs. The lowest ASR of prevalence and incidence were observed in East and South Asia, respectively. East Asia had the lowest ASR of DALYs, while Eastern Europe had the lowest ASR of deaths. From 1990 to 2019, high SDI countries have had the lowest ASR of death and the largest decline in that (73.0% [69.9%–75.4%]), as well as the highest ASR of incidence and prevalence, compared to other quintiles (appendix 2, Fig. S22).

In 2019, worldwide, high BMI was the leading risk factor comprising 44.8 (26.4–68.6) ASR of attributed DALYs in both sexes, followed by smoking. When stratified by sex, smoking was the primary risk factor in men, accounting for 40.3 (21.9–56.1) ASR of DALYs. However, smoking stood as the last risk factor in women (appendix 2, Fig. S24). In all SDI quintiles, smoking was the second most prominent risk factor after high BMI, except for low SDI. Nevertheless, in low SDI countries, occupational asthmagens ranked second (appendix 2, Fig. S23).

### ILD and pulmonary sarcoidosis

ILD and pulmonary sarcoidosis were responsible for 3.8 million (2.9–4.5) DALYs globally in 2019, with 4.7 million (4.0–5.4) prevalent cases and 24.2 million (19.6–29.5) new cases. Throughout the investigated period, the ASR of DALYs and deaths have been slightly lower in women, while the ASR of prevalence and incidence were comparable (appendix 2, Fig. S25). Globally, the ASR of prevalence and incidence have increased from 1990 to 2019 by 9.4% (6.1%–12.9%) and 14.1% (11.1%–17.3%), respectively. Nevertheless, the ASR of deaths and DALYs have remained stable (Table 1).

In 2019, Andean Latin America, followed by South Asia, had the highest, while Eastern Europe, followed by East Asia, had the lowest ASR of death and DALYs. High-income Asia Pacific and high-income North America had the highest ASR of prevalence and incidence, respectively. The high SDI quintile had the highest ASR of prevalence, whereas middle and high-middle SDI countries had the lowest ASR of DALYs and deaths (appendix 2, Fig. S26). In all SDI quintiles, except for low-middle SDI, the ASR of prevalence significantly increased from 1990 to 2019 (appendix 2, Table S2).

### Pneumoconiosis

Globally, silicosis, asbestosis, coal workers, and other pneumoconiosis were estimated to account for 0.9 million collectively (0.8–1.1) DALYs and 3.1 million (2.6–3.6) prevalent cases in 2019. Pneumoconiosis prevalence has remained comparable from 1990 to 2019 while the ASR of DALYs, deaths, and incidence have decreased by 44.4% (31.2%–52.9%), 53.3% (38.6%–60.9%), and 13.7% (6.6%–21.3%), respectively (Table 1). Despite the overall decreasing trend in the ASR of DALYs, deaths, and incidence, the ASR of incidence slightly rose by 5.4% (1.1%–10.2%) from 1990 to 2019 in women globally. Men have had significantly higher ASR of DALYs, deaths, prevalence, and incidence throughout the investigated period (appendix 2, Fig. S27).

Pneumoconiosis ranked third among all causes constituting DALYs from CRDs in East Asia responsible for 29.2 (22.9–37.1) ASR of DALYs in 2019, which is markedly higher than other 21 regions (appendix 2, Fig. S14). This region has had the highest ASR of DALYs, deaths, incidence, and prevalence due to pneumoconiosis from 1990 to 2019. Asbestosis was the primary contributor to the ASR of DALYs due to pneumoconiosis in Australasia, high-income North America, Oceania, Eastern, and Southern Sub-Saharan Africa (appendix 2, Fig. S31). In other 21 regions, mainly silicosis and to a lesser extent, other pneumoconiosis constituted most of the ASR of DALYs (appendix 2, Table S4). Moreover, from 1990 to 2019, middle and high-middle SDI regions had the highest ASR of prevalence, while the low SDI quintile, followed by high SDI, had the lowest ASR of pneumoconiosis prevalence (appendix 2, Fig. S28).

Globally, occupational exposure to silica, PM, gases, fumes, and asbestos were the risk factors of pneumoconiosis in order of attributed ASR of DALYs. Nevertheless, occupational PM, gases, and fumes ranked first, and occupational exposure to asbestos ranked last in women (appendix 2, Fig. S29).

## Discussion

Globally, the total number of deaths, DALYs, incidence, and prevalence of CRDs rose, whereas the ASR of all these indices declined in both sexes combined during the past three decades. The increase in crude numbers is primarily due to population growth. On a global scale, significant progress was achieved in reducing ASR of deaths, DALYs, prevalence, and incidence of COPD, asthma, and pneumoconiosis in both sexes combined. Nevertheless, this trend was variable among different geographical locations and sexes. Among CRDs, the global ASR of deaths and DALYs of ILD and pulmonary sarcoidosis remained stable while the incidence and prevalence grew. Asthma had the highest crude and ASR of incidence and prevalence among CRDs, while COPD accounted for the highest deaths and DALYs.

In the past three decades, a considerable drop was observed in the ASR of DALYs due to CRDs attributable to all risk factors, except for ambient PM pollution, high BMI, and occupational asbestos exposure in both sexes. Smoking, followed by ambient PM, is the major risk of CRDs worldwide in both sexes. The non-optimal temperature is a new risk added in GBD 2019, which is responsible for 8.3% (6.5%–10.1%) of total DALYs due to COPD in 2019 (not shown). This finding highlights the potential consequences of climate change on CRDs, particularly COPD.^18^ Climate change can increase temperature variability and result in extremely cold or warm temperatures, which can directly aggravate COPD exacerbations or increase exposure to environmental risk factors.^18^ Climate change may also result in longer pollen seasons with pollens with increased quantity and potency affecting the burden of asthma.^19^ High BMI, as the only evaluated metabolic risk factor for CRDs, was the leading risk factor of asthma in both sexes combined worldwide, with a more prominent role in women. The steady trend of the ASR of deaths and DALYs from asthma attributed to high BMI worldwide highlights the necessity of global attention for lifestyle modification interventions, which may reduce morbidity in patients with concomitant asthma and obesity.^20^ Given the higher prevalence of obesity in high- and upper-middle-income countries compared to low- and lower-middle-income countries (LMICs), these interventions may be of more value in these nations.^21^

Smoking was the leading risk factor for DALYs from CRDs in all regions except for Sub-Saharan Africa. A significant decrease is observed in the DALYs attributed to smoking in East Asia (66.7% [52.0%–72.6%]), which is accompanied by a marked drop in the ASR of DALYs (67.0% [52.6%–72.0%]). Like this region, the ASR of DALYs attributed to smoking declined in all regions, except for the Caribbean. These findings indicate that measures developed by the WHO Framework Convention on Tobacco Control (WHO FCTC) and in the WHO MPOWER package,^22^ such as demand reduction acts, regulation of advertisement, contents and labeling of tobacco products, and taxation on tobacco, have played a substantial role in lowering smoking globally.^23^ Targeted tobacco control strategies in China, the largest and most populous country in East Asia, namely the Healthy China 2030 strategy, which aims to reduce the smoking prevalence to 20%, could explain the significant reduction of burden due to smoking in this region.^24^

Nevertheless, there is a substantial potential for further reduction of CRDs burden attributed to smoking globally as many countries have not been fully adherent to tobacco control policies.^25^ Specifically, strong policies from the WHO FCTC have been poorly implemented in many LMICs.^23^ The Caribbean is the only region without a considerable change in the ASR of CRDs burden attributed to smoking. Cuba, the most populated country in this region, is among the few countries with growth in the ASR of DALYs from CRDs attributed to smoking from 1990 to 2019 (25.4% [2.1%–51.0%]) (appendix 2, Table S6). Cuba is one of the handful of countries that have not ratified the WHO FCTC programme, with a low cessation rate among Cubans found by previous investigations.^26^ This finding mandates a more careful reconsideration of tobacco control strategies in this region. To reduce smoking prevalence and the associated burden of CRDs, prevention of smoking initiation in adolescents and smoking cessation among current smokers are essential; however, the higher estimated prevalence of tobacco use in high- and upper-middle-income countries compared to LMICs indicates that the latter approach could be more crucial in these nations.^27^ In addition to tobacco smoking, epidemiological evidence suggests that e-cigarettes use is associated with COPD and asthma.^28^ While the GBD 2019 study has not included e-cigarettes use as a risk factor, its potential impact on the burden of CRDs cannot be overlooked.

In Sub-Saharan Africa, household air pollution from solid fuels was the primary risk factor responsible for DALYs from CRDs. While globally, the attributed burden of CRDs due to household air pollution has had the most considerable drop from 1990 to 2019 compared to the other risk factors (79.4% [72.7%–84.4%]), it still accounts for a substantial burden in the LMICs. According to the Energy Sector Management Assistance Program (ESMAP), near four billion people are estimated to lack access to modern energy heating or cooking services, and women and children have a higher exposure enduring a larger impact. Financial, social, and cultural barriers hinder the transition from traditional biomass cooking fuels to modern energy sources, i.e. electricity and gas.^29^

The Clean Cooking Alliance (CCA) is one of the most prominent global initiatives to make clean cooking accessible in the LMICs.^30^ Despite global attempts to improve access to clean energies, traditional solid fuel combustion has increased in the Sub-Saharan region due to the outgrowing pace of population growth.^29^ Improved access to modern energy cooking services is an indispensable step in achieving the SDGs defined by the UN until 2030. Not only can this significantly reduce mortality due to CRD, but it can also improve gender equality, access to affordable and clean energy, climate change, and terrestrial ecosystems.^31^ Importantly, household air pollution has been cited as a risk factor of asthma,^32^ albeit due to the mixed reports, this is not included in the GBD 2019 study, and further research is required to assess the association.

Globally, ambient PM pollution is the second major risk factor of CRDs, with no significant alteration in the attributed ASR of DALYs and deaths from CRDs in the past three decades. The ASR of DALYs from CRDs due to ambient PM pollution has decreased in Central, Eastern, and Western Europe, whereas it has risen in Sub-Saharan Africa and low SDI quintile in both sexes combined from 1990 to 2019. The growing burden in the Sub-Saharan Africa region is chiefly ascribed to increased desert dust due to climate change and rapid urbanisation.^33^^,^^34^ The LMICs have shown higher concentrations of PM pollution due to lack of legislation and/or adherence to air quality guidelines, higher prevalence of coal power stations, and not meeting vehicles emission standards.^35^^,^^36^ The European region is at the forefront of combatting ambient PM pollution with the European Green Deal, which aims to reduce greenhouse gas emissions by at least 55% by 2030 compared to 1990.^37^

The ASR of DALYs from COPD, asthma, and pneumoconiosis attributed to occupational risks has dropped in the past three decades worldwide in both sexes combined. The major DALYs attributed to the occupational risks are from COPD. The ASR of DALYs from CRDs attributed to these risks are approximately three-fold in men than women globally in 2019, which is justified by the lower employment rate of women in professions involving the relevant exposures. Analysis of the GBD 2016 study showed that the population attributable fraction for occupational risks for COPD, asthma, and pneumoconiosis were 17%, 10%, and 100%, respectively.^38^ The highest DALYs from CRDs due to occupational risks are observed in South Asia, Oceania, and East Asia. In China, pneumoconiosis constituted 90% of occupational diseases.^39^ Allocation of resources and occupational health legislation are critical in these regions to reduce toxic exposures and ensure high-quality health services for susceptible workers.^40^

The highest ASR of deaths and DALYs from CRDs is observed in Oceania and South Asia and the low SDI quintile despite the moderate ASR of prevalence in these regions. On the other hand, the high SDI quintile has the highest ASR of prevalence but the lowest deaths in 2019. These findings accent the variability of management and quality of care among countries with different income levels. Chronic respiratory care is a multi-faceted challenge in LMICs. Lack of preventive measures and increased lifetime exposure to CRDs risks should not be overlooked. CRDs are commonly underdiagnosed in these countries; therefore, patients are frequently only detected when developing severe symptoms. Restricted access to the diagnostic tools, i.e. spirometry and chest imaging, at the primary care level and shortage of trained clinical staff able to accurately perform and interpret the tests are the primary challenges in diagnosing CRDs in the LMICs. A dearth of health professionals with clinical respiratory training and limited access to medications impede the appropriate management of CRDs in such settings.^41^ For instance, inhaled corticosteroids are vital in managing asthma and have been shown to reduce morbidity and mortality.^10^ Nevertheless, they are typically unavailable, unaffordable, or under-prescribed in the LMICs. Improving chronic respiratory care in these regions hinges upon fortified healthcare systems providing high-quality preventive, diagnostic, therapeutic, rehabilitative, and palliative measures.^41^

Multiple global initiatives have been developed over the past few decades to improve respiratory care, undoubtedly contributing to the global decline in the age-standardised burden of CRDs. The Package of Essential Non-communicable (PEN) disease interventions for primary health care was designed to facilitate the provision of acceptable care for patients with NCDs, including CRDs, even in settings with limited resources.^42^ The Practical Approach to Lung health (PAL) was another tool created by the WHO to improve the management of respiratory patients in primary healthcare settings, especially in countries with weak health systems.^43^ Years after the development of the PAL, the GARD was established to improve the prevention, diagnosis, and medical care of CRDs according to local needs worldwide by estimating population needs, advocating for health promotion and prevention, and developing cost-effective strategies for CRDs.^44^ In addition, other global initiatives focusing on COPD (GOLD)^9^ and asthma (GINA)^10^ have been developed to increase awareness, improve prevention, management, and access to effective treatments.

With COVID-19 continuing to spread around the world, the interaction between COVID-19 and CRDs is under the spotlight.^45^ A population cohort study found that while asthma was not associated with a major increased risk of severity, COPD and ILD were independent predictors of severity and higher mortality in patients with COVID-19. However, the death rates from COVID-19 were lower than the ordinary risk of death from any cause.^46^ As ILD can impact the outcomes of COVID-19, COVID-19 may also result in long-lasting fibrotic-like changes in the lungs, which can be detectable even after 6–12 months on imaging in some cases.^47^^,^^48^

This study is an updated comprehensive analysis of the global, regional, and national epidemiology of CRDs and their associated risk factors. Previous reports utilising the GBD 2019 data have reported the burden attributable to certain sub-causes or risk factors, but none have focused on all CRDs included in the GBD 2019 study.^36^^,^^40^^,^^49^ Whilst the GBD 2019 supplies a comprehensive estimation of the burden of most NCDs, it faces several limitations. Lack of reliable primary data sources, particularly in the LMICs, could adversely affect the accuracy of the estimates. The paucity of primary investigations in addition to the under-diagnosis in these regions can lead to underestimation. The GBD addresses this limitation by improving data processing and modeling and adding newly available data sources in each iteration. Nevertheless, further original investigations are incremental in accurately measuring the burden of diseases in such regions. Even when primary data are available, the various case definition of CRDs and lack of using the preferred definition could also affect the precision of the estimates. The GBD 2019 study entailed a wider alternative definition for COPD and asthma than the GBD 2017 and performed a bias mapping from the alternative to reference definitions.

Furthermore, we could not account for genetic susceptibilities in this study, albeit they can play a major role in developing COPD and asthma.^50^ This is beyond the scope of this manuscript and can be addressed in the future cycles of the GBD. Other CRDs were responsible for a considerable burden, although they encompassed various diseases, which hindered the measurement of the nonfatal estimates. Development of cause-specific estimates for sleep apnea and allergic rhinitis and sinusitis can be considered in the next cycles of the GBD, given their high prevalence.^51^^,^^52^ Lastly, reconsidering the available evidence for risk–outcome pairs would be crucial in future iterations, especially for ILD and pulmonary sarcoidosis. While currently, no risk factors have been cited for this cause, occupational and environmental risks can increase the risk of developing the disease.^53^

We were also unable to quantitatively account for the effect of climate change on the burden of CRDs due to a lack of sufficient data on environmental indicators within the same time span (1990–2019). Future endeavors are needed for collecting reliable data on climate change indicators enabling a quantitative assessment of their impact. Moreover, the GBD 2019 estimation was conducted before the COVID-19 pandemic.^54^ Therefore, future iterations of the GBD study need to address the impact of the COVID-19 pandemic on the burden of CRDs.

CRDs were the third leading cause of death in 2019. The age-standardised DALYs, death, prevalence, and incidence rates of CRDs have significantly dropped from 1990 to 2019 globally. However, the age-standardised prevalence and incidence rates grew in the high SDI quintile. While COPD primarily contributes to deaths and DALYs from CRDs, asthma has the highest prevalence worldwide. Men have higher age-standardised rates of deaths and DALYs from COPD and pneumoconiosis. The high age-standardised rates of deaths and DALYs from CRDs in the LMICs, particularly East Asia and Oceania, highlight the gaps in prevention, diagnosis, and management and warrant further investigations and respiratory care improvement strategies.

The estimates provided in this study can provide policymakers and healthcare providers with an overview of the burden and risk factors of CRDs to facilitate the path towards achieving the third SDG. Full global adherence to tobacco control measures and air quality improvement strategies are crucial in reducing the burden attributed to CRDs. In the LMICs, where CRDs are responsible for a substantial burden, in addition to these policies, improvement of respiratory care by providing clinical respiratory training for healthcare workers, raising public awareness, and access to diagnostic tools and medications are fundamental. Global attempts to foster clean cooking and heating energies in the LMICs, particularly the Sub-Saharan region, are essential for reducing deaths and DALYs from CRDs burden, especially in women.

## Contributors

Please see appendix 3 (pp 341–346) for more detailed information about individual author contributions to the research, divided into the following categories: managing the overall research enterprise; writing the first draft of the manuscript; primary responsibility for applying analytical methods to produce estimates; primary responsibility for seeking, cataloguing, extracting, or cleaning data; designing or coding figures and tables; providing data or critical feedback on data sources; developing methods or computational machinery; providing critical feedback on methods or results; drafting the manuscript or revising it critically for important intellectual content; and managing the estimation or publications process.

## Data sharing statement

Data from this study are openly available in the online database of GBD 2019 as described in Methods.

## Editor note

The Lancet Group takes a neutral position with respect to territorial claims in published maps and institutional affiliations.

## Declaration of interests

R Ancuceanu reports consulting fees from Abbvie; payment or honoraria for lectures, presentations, speakers bureaus, manuscript writing or educational events from Abbvie, B. Braun, Sandoz, and Laropharm all outside the submitted work. NS Bayileyegn reports participation on a Data Safety Monitoring Board or Advisory Board with Jimma University; leadership or fiduciary roles in board, society, committee or advocacy groups, paid or unpaid with Jimma University as part of the disaster response team all outside the submitted work. S Das reports grants or contracts from the Department of Science and Technology, government of India all outside the submitted work. TC Ekundayo other support from the African-German Network of Excellence in Science, the Federal Ministry of Education and Research, and the Alexander von Humboldt Foundation all outside the submitted work. R Erkhembayar reports grants or contracts from World Health Organization, Country Office in Mongolia for disease burden estimates and utilizations, training and capacity building at CHD, MoH, Mongolia all outside the submitted work. A Faro reports support for the present manuscript from CNPq - National Council for Scientific and Technological Development, Brazil all outside the submitted work. I Filip reports other support from Avicenna Medical and Research Institute all outside the submitted work. R Franklin reports grants or contracts for Heatwaves in Queensland Arc Flash, Human Factors from Queensland Government; payment or honoraria for lectures, presentations, speakers bureaus, manuscript writing or educational events from World Safety Conference 2022; support for attending meetings and/or travel from ACTM - Tropical Medicine and Travel Medicine Conference 2022; leadership or fiduciary roles in board, society, committee or advocacy groups, paid or unpaid with Kidsafe as a Director, with Auschem as a Director, with ISASH as part of the Governance Committee, with Farmsafe as a Director, and with PHAA Injury Prevention SIG Convenor all outside the submitted work. C Herteliu reports grants or contracts from Romanian Ministry of Research Innovation and Digitalization, MCID for project number ID-585-CTR-42-PFE-2021 research grant (Jan 2022-Jun 2023) “Enhancing institutional performance through development of infrastructure and transdisciplinary research ecosystem within socio-economic domain - PERFECTIS” all outside the submitted work. NE Ismail reports leadership or fiduciary roles in board, society, committee or advocacy groups, paid or unpaid with Malaysian Academy of Pharmacy as a Council Member. J Jozwiak reports payment or honoraria for lectures, presentations, speakers bureaus, manuscript writing or educational events from Novartis and Adamed all outside the submitted work. K Krishan reports other support from UGC Centre of Advanced Study, CAS II, Department of Anthropology, Panjab University, Chandigarh, India all outside the submitted work. AFA Mentis reports grants or contracts from `MilkSafe: A novel pipeline to enrich formula milk using omics technologies,’ a research co financed by the European Regional Development Fund of the European Union and Greek national funds through the Operational Program Competitiveness, Entrepreneurship and Innovation, under the call RESEARCH - CREATE - INNOVATE (project code: T2EDK-02222), as well as from ELIDEK (Hellenic Foundation for Research and Innovation, MIMS-860); payment for expert testimony having served as external peer-reviewer for Fondazione Cariplo, Italy; participation on a Data Safety Monitoring Board or Advisory Board serving as Editorial Board Member for “Systematic Reviews” journal, for “Annals of Epidemiology” journal, and as Associate Editor for “Translational Psychiatry;” stock or stock options in a family winery; and other support from BGI Group as a scientific officer all outside the submitted work. N Moka reports leadership or fiduciary roles in board, society, committee or advocacy groups, paid or unpaid with Kentucky Society of Clinical Oncology as Treasurer. MJ Postma reports leadership or fiduciary roles in board, society, committee or advocacy groups, paid or unpaid with JCVI (UK) as an unpaid Member; stock or stock options in HealthEcore (Zeist, NL) and PAG BV (Groningen, NL) all outside the submitted work. A Radfar reports other support from Avicenna Medical and Research Institute all outside the submitted work. J Singh reports consulting fees Crealta/Horizon, Medisys, Fidia, PK Med, Two labs Inc., Adept Field Solutions, Clinical Care options, Clearview healthcare partners, Putnam associates, Focus forward, Navigant consulting, Spherix, MedIQ, Jupiter Life Science, UBM LLC, Trio Health, Medscape, WebMD, and Practice Point communications; and the National Institutes of Health and the American College of Rheumatology; payment or honoraria for lectures, presentations, speakers bureaus, manuscript writing or educational events from Simply Speaking on the speaker's bureau; support for attending meetings and/or travel from the steering committee of OMERACT; participation on a Data Safety Monitoring Board or Advisory Board with FDA Arthritis Advisory Committee; leadership or fiduciary roles in board, society, committee or advocacy groups, paid or unpaid with OMERACT as a steering committee member, Veterans Affairs Rheumatology Field Advisory Committee as Chair, and UAB Cochrane Muskuloskelatal Group Satellite Center on Network Meta-analysis as editor and Director; stock or stock options in TPT Global Tech, Vaxart pharmaceuticals, Atyu biopharma, Adaptimmune Therapeutics, GeoVax Labs, Pieris Pharmaceuticals, Enzolytics Inc., Seres Therapeutics, Tonix Pharmaceuticals and Charlotte's Web Holdings, Inc all outside the submitted work. E Upadhyay reports patents planned, issued or pending for “A system and method of reusable filters for anti-pollution mask” (published), “A system and method for electricity generation through crop stubble by using microbial fuel cells” (published), “A system for disposed personal protection equipment (PPE) into biofuel through pyrolysis and method” (published), and “A novel herbal pharmaceutical aid for formulation of gel and method thereof” (filed) all outside the submitted work. M Wei reports support for the present manuscript from NIH National Institute on Aging K23 career development award; grants or contract from NIH National Institute on Aging K23 career development award; support for attending meetings and/or travel from NIH National Institute on Aging K23 career development award to attend national meetings (SGIM, AGS) to present results; leadership or fiduciary roles in board, society, committee or advocacy groups, paid or unpaid with Society of General Internal Medicine (SGIM) as Chair of the Research Committee all outside the submitted work.

## Footnotes

Appendix A Supplementary data related to this article can be found at https://doi.org/10.1016/j.eclinm.2023.101936.
