# Supplementary material for: Global burden of chronic respiratory diseases and risk factors, 1990–2019: an update from the Global Burden of Disease Study 2019
Source: eClinicalMedicine. 2023 Apr 25;59:101936. doi: 10.1016/j.eclinm.2023.101936 (PMC7614570; doi:10.1016/j.eclinm.2023.101936)
Supplement: Supplementary material [file mmc1.pdf]

Appendix 1: Online Methods

List of abbreviations 2

GBD overview 3

Geographic locations of the analysis 3

Time period of the analysis 3

Statement of GATHER compliance 3

Data input sources overview 4

Cause of Death (CoD) modeling descriptions 5

Chronic Respiratory Diseases 5

Chronic Obstructive Pulmonary Disease 8

Pneumoconiosis Diseases 11

Asthma 14

Interstitial Lung Disease and Pulmonary Sarcoidosis 17

Other Chronic Respiratory Diseases 20

Non-fatal modeling descriptions 23

Chronic obstructive pulmonary disease (COPD) 23

Pneumoconiosis 30

Asthma 35

Interstitial lung disease (ILD) and pulmonary sarcoidosis 40

Other chronic respiratory diseases 44

Risk-specific modeling descriptions 45

Non-optimal temperature 45

High body-mass index 51

Smoking 59

Secondhand smoke 64

Ambient particulate matter pollution 67

Ambient ozone pollution 105

Household air pollution 120

Occupational risk factors 127

Calculating the burden of multiple risk factors 136

Inclusion of a risk–outcome pair in the GBD 137

Supplementary Tables and Figure

Table S1. GATHER checklist 138

Table S2. List of International Classification of Diseases (ICD) codes 140

Table S3. Restrictions on age and sex by cause for GBD 2019 141

Table S4. Total number of site years by cause and source type for 2019 142

Table S5. CODEm covariates used 143
Table S6. Comparison of GBD 2017 and GBD 2019 covariates and level of covariates used in cause of death modeling 158
Table S7. SDI quintiles for countries estimated in GBD 2019 167
Figure S1. Classification of GBD regions and super regions 168

Appendix 2: Online Figures and Tables

B.3 Chronic respiratory diseases (CRDs)
Figure S1. Age-standardised rate of deaths and DALYs from CRDs in 2019 and their percent change from 1990 in men 170
Figure S2. Age-standardised rate of deaths and DALYs from CRDs in 2019 and their percent change from 1990 to 2019 in women 171
Figure S3. Age-standardised rate of prevalence and incidence of CRDs in 2019 and their percent change from 1990 to 2019 in both sexes 172
Figure S4. Age-standardised rate of prevalence and incidence of CRDs in 2019 and their percent change from 1990 to 2019 in men 173
Figure S5. Age-standardised rate of prevalence and incidence of CRDs in 2019 and their percent change from 1990 to 2019 in women 174
Figure S6. Absolute death rate due to CRDs by age and sex, 1990 and 2019 175
Figure S7. Absolute prevalence rate due to CRDs by age and sex, 1990 and 2019 176
Figure S8. Absolute incidence rate due to CRDs by age, 2019 and sex, 1990 and 2019 177
Figure S9. Contribution of YLLs and YLDs to age-standardised and age-specific DALYs from CRDs, 2019 178
Figure S10. Correlation between the SDI and age-standardised DALYs rates due to CRDs in 1990, 2000, 2010, and 2019 globally 179
Figure S11. Correlation between the SDI and age-standardised death rates due to CRDs in 1990, 2000, 2010, and 2019 globally 180
Figure S12. Correlation between the SDI and age-standardised prevalence rates due to CRDs in 1990, 2000, 2010, and 2019 globally 181
Figure S13. Correlation between the SDI and age-standardised incidence rates due to CRDs in 1990, 2000, 2010, and 2019 globally 182
Figure S14. Ranked contribution of the specific sub-causes to the age-standardised DALYs from CRDs by region, 2019, for both sexes, females, and males 183
Figure S15. Ranked contribution of risk factors to the age-standardised death from CRDs by region, 2019, for both sexes, females, and males 184

Figure S16. Age-standardised DALYs from CRDs attributed to risk factors by sex and region in 1990 and 2019 ... 185
B.3.1 Chronic obstructive pulmonary diseases (COPD)
Figure S17. Global age-standardised rates of incidence, prevalence, deaths, and DALYs of COPD by sex, 1990-2019 ... 186
Figure S18. Global age-standardised rates of incidence, prevalence, deaths, and DALYs of COPD by SDI, both sexes, 1990-2019 ... 187
Figure S19. Ranked contribution of risk factors to the age-standardised DALYs from COPD by region, 2019, for both sexes, females, and males ... 188
Figure S20. Age-standardised DALYs from COPD attributed to risk factors by sex and region in 1990 and 2019 ... 189
B.3.3 Asthma
Figure S21. Global age-standardised rates of incidence, prevalence, deaths, and DALYs of asthma by sex, 1990-2019 ... 190
Figure S22. Global age-standardised rates of incidence, prevalence, deaths, and DALYs of asthma by SDI, both sexes, 1990-2019 ... 191
Figure S23. Ranked contribution of risk factors to the age-standardised DALYs from asthma by region, 2019, for both sexes, females, and males ... 192
Figure S24. Age-standardised DALYs from asthma attributed to risk factors by sex and region in 1990 and 2019 ... 193
B.3.4 Interstitial Lung Disease (ILD) and pulmonary sarcoidosis
Figure S25. Global age-standardised rates of incidence, prevalence, deaths, and DALYs of ILD and pulmonary sarcoidosis by sex, 1990-2019 ... 194
Figure S26. Global age-standardised rates of incidence, prevalence, deaths, and DALYs of ILD and pulmonary sarcoidosis by SDI, both sexes, 1990-2019 ... 195
B.3.2 Pneumoconiosis (Coal Worker’s Pneumoconiosis, Asbestosis, Silicosis, and Other Pneumoconiosis)
Figure S27. Global age-standardised rates of incidence, prevalence, deaths, and DALYs of pneumoconiosis by sex, 1990-2019 ... 196
Figure S28. Global age-standardised rates of incidence, prevalence, deaths, and DALYs of pneumoconiosis by SDI, both sexes, 1990-2019 ... 197
Figure S29. Ranked contribution of risk factors to the agestandardised DALYs from pneumoconiosis by region, 2019, for both sexes, females, and males ... 198
Figure S30. Age-standardised DALYs from pneumoconiosis attributed to risk factors by sex and region in 1990 and 2019 ... 199

Figure S31. Ranked contribution of the specific sub-causes to the age-standardised DALYs from pneumoconiosis by region, 2019, for both sexes, females, and males 200
Table S1. Global incidence, prevalence, deaths, DALYs, YLLs, and YLDs from CRDs by sex and for both sexes combined 201
Table S2. Rate of incidence, prevalence, deaths, DALYs, YLLs, and YLDs from CRDs by SDI, by sex and for both sexes combined 203
Table S3. Results from decomposition analysis of the total incidence number in both sexes combined, by region and SDI 209
Table S4. Age-standardised rate of incidence, prevalence, deaths, DALYs, YLLs, and YLDs from sub-causes of pneumoconiosis by SDI, by sex 211
Table S5. Age-standardised rates and the total number of incidence, prevalence, deaths, DALYs, YLLs, and YLDs in CRDs and its sub causes (i.e., COPD, pneumoconiosis, asthma, ILD and pulmonary sarcoidosis, and other CRDs) in males, females, and both sexes combined in 7 GBD super regions, 21 GBD regions, and 204 countries and territories in 1990 and 2019 with percent change (1990 to 2019) 217
Table S6. Age-standardised rates and crude figures of deaths, DALYs, YLLs, and YLDs attributed to risk factors in CRDs and its sub causes (i.e., COPD, pneumoconiosis, and asthma) in males, females, and both sexes combined in 7 GBD super regions, 21 GBD regions, and 204 countries and territories in 1990 and 2019 with percent change (1990 to 2019) 281

Appendix 3: Authors' Contributions

Authors' Contributions 341

Appendix 1: Online Methods

Results appendix to "Global Burden of Chronic Respiratory Diseases and Risk Factors, 1990–2019: Update From the GBD 2019 Study"

This appendix provides further methodological detailed results for "Global Burden of Chronic Respiratory Diseases and Risk Factors, 1990–2019: Update From the GBD 2019 Study".

Portions of this appendix have been reproduced or adapted from Vos et al. and Murray et al.

Reference:

Vos T, Lim SS, Abbafati C, et al. Global burden of 369 diseases and injuries in 204 countries and territories, 1990-2019: a systematic analysis for the Global Burden of Disease Study 2019. The Lancet 2020; 396(10258): 1204-22.

Murray CJL, Aravkin AY, Zheng P, et al. Global burden of 87 risk factors in 204 countries and territories, 1990-2019: a systematic analysis for the Global Burden of Disease Study 2019. The Lancet 2020; 396(10258): 1223-49.

List of abbreviations

Abbreviation	Meaning
**BMI**	body-mass index
**CF**	correction factor
**COD**	causes of death
**CODEm**	cause of death ensemble modelling
**COPD**	chronic obstructive pulmonary disease
**CRA**	comparative risk assessment
**CSV**	comma-separated values
**CSMR**	cause-specific mortality rate
**DALY**	disability-adjusted life-year
**DHS**	Demographic and Health Survey
**DisMod-MR**	disease model-Bayesian meta-regression
**DW**	disability weights
**EMR**	excess mortality rate
**FAO**	Food and Agriculture Organization
**GATHER**	Guidelines for Accurate and Transparent Health Estimates Reporting
**GBD**	Global Burden of Diseases, Injuries, and Risk Factors Study
**GHDx**	Global Health Data exchange
**HAP**	household air pollution
**HAQ**	Healthcare Access and Quality
**ICD-**	International Classification of Diseases
**IER**	integrated exposure response
**IHME**	Institute for Health Metrics and Evaluation
**ILO**	International Labour Organization
**LDI**	lag-distributed income
**LOESS**	locally estimated scatterplot smoothing
**MCCD**	Medical Certification of Causes of Death
**MEPS**	Medical Expenditure Panel Surveys
**MICS**	Multiple Indicator Cluster Surveys
**MR-BRT**	meta-regression—Bayesian, regularised, trimmed
**PAF**	population attributable fraction
**PM2.5**	particulate matter <2.5 [illegible]m in diameter
**RMSE**	root mean square error
**RR**	relative risk
**SCD**	Survey of Causes of Death
**SDI**	Socio-demographic Index
**SHS**	secondhand smoke
**SRS**	Sample Registration System
**ST-GPR**	spatiotemporal Gaussian process regression
**TMREL**	theoretical minimum-risk exposure level
**UI**	uncertainty interval
**UN**	United Nations
**USA**	United States
**VA**	verbal autopsy
**VR**	vital registration
**WHO**	World Health Organization
**YLDs**	years lived with disability
**YLLs**	years of life lost

GBD overview

Geographic locations of the analysis

We produced estimates for 204 countries and territories that were grouped into 21 regions and seven super-regions. The seven super-regions are central Europe, eastern Europe, and central Asia; high income; Latin America and the Caribbean; north Africa and the Middle East; south Asia; southeast Asia, east Asia, and Oceania; and sub-Saharan Africa. For GBD 2019, 9 countries and territories (Cook Islands, Monaco, San Marino, Nauru, Niue, Palau, Saint Kitts and Nevis, Tokelau, and Tuvalu) were added, such that the GBD location hierarchy now includes all WHO member states. This year, GBD includes subnational analyses for several new countries and continues to analyse at subnational levels countries that were added in previous cycles. Subnational estimation in GBD 2019 includes five new countries (Italy, Nigeria, Pakistan, the Philippines, and Poland) and 16 countries previously estimated at subnational levels (Brazil, China, Ethiopia, India, Indonesia, Iran, Japan, Kenya, Mexico, New Zealand, Norway, Russia, South Africa, Sweden, the UK, and the USA). All analyses are at the first level of administrative organisation within each country except for New Zealand (by Māori ethnicity), Sweden (by Stockholm and non-Stockholm), the UK (by local government authorities), and the Philippines (by provinces). All subnational estimates for these countries were incorporated into model development and evaluation as part of GBD 2017. To meet data use requirements, in this publication we present subnational estimates for Brazil, India, Indonesia, Japan, Kenya, Mexico, Sweden, the UK, and the USA); given space constraints, these results are presented in appendix 2 instead of the main text. Subnational estimates for China are included in maps but are not reported in appendix tables. Subnational estimates for other countries will be released in separate publications.

For GBD 2019, we have also defined locations as standard locations and non-standard locations. Standard GBD locations are defined as the set of all subnationals belonging to countries where data quality is high and with populations over 200 million, in addition to all other countries. Standard locations include the subnationals for China, India, the United States, and Brazil, but not Indonesia; China, India, the United States, and Brazil are also included at the country level. All other countries with subnational estimates are defined as non-standard locations.

Time period of the analysis

A complete set of risk-specific exposures, relative risks (RRs), theoretical minimum-risk exposure levels (TMRELs), and population attributable fractions (PAFs) were computed for the years 1990–2019.

Statement of GATHER compliance

This study complies with the Guidelines for Accurate and Transparent Health Estimates Reporting (GATHER) recommendations. We have documented the steps involved in our analytical procedures and detailed the data sources used. See table S1 for the GATHER checklist.

The GATHER recommendations may be found here: http://gather-statement.org/
Reference: Stevens GA, Alkema L, Black RE, et al. Guidelines for Accurate and Transparent Health Estimates Reporting: the GATHER statement. The Lancet 2016; 388: e19–23.

Data input sources overview

GBD 2019 incorporated a large number and wide variety of input sources to estimate mortality, causes of death and illness, and risk factors for 204 countries and territories from 1990-2019. These input sources are accessible through an interactive citation tool available in the GHDx.

Users can retrieve citations for a specific GBD component, cause or risk, and location by choosing from the available selection boxes. They can then view and access GHDx records for input sources and export a comma-separated value (CSV) file that includes the GHDx metadata, citations, and information about where the data were used in GBD. Additional metadata for each input source are available through the citation tool as required by the GATHER statement.

The citation tool is accessible through the GHDx at http://ghdx.healthdata.org/gbd-2019/data-input-sources.

Reference: Stanaway JD, Afshin A, Gakidou E, et al. Global, regional, and national comparative risk assessment of 84 behavioural, environmental and occupational, and metabolic risks or clusters of risks for 195 countries and territories, 1990–2017: a systematic analysis for the Global Burden of Disease Study 2017. The Lancet 2018; 392: 1923–94.

Cause of Death (CoD) modeling descriptions

Chronic Respiratory Diseases

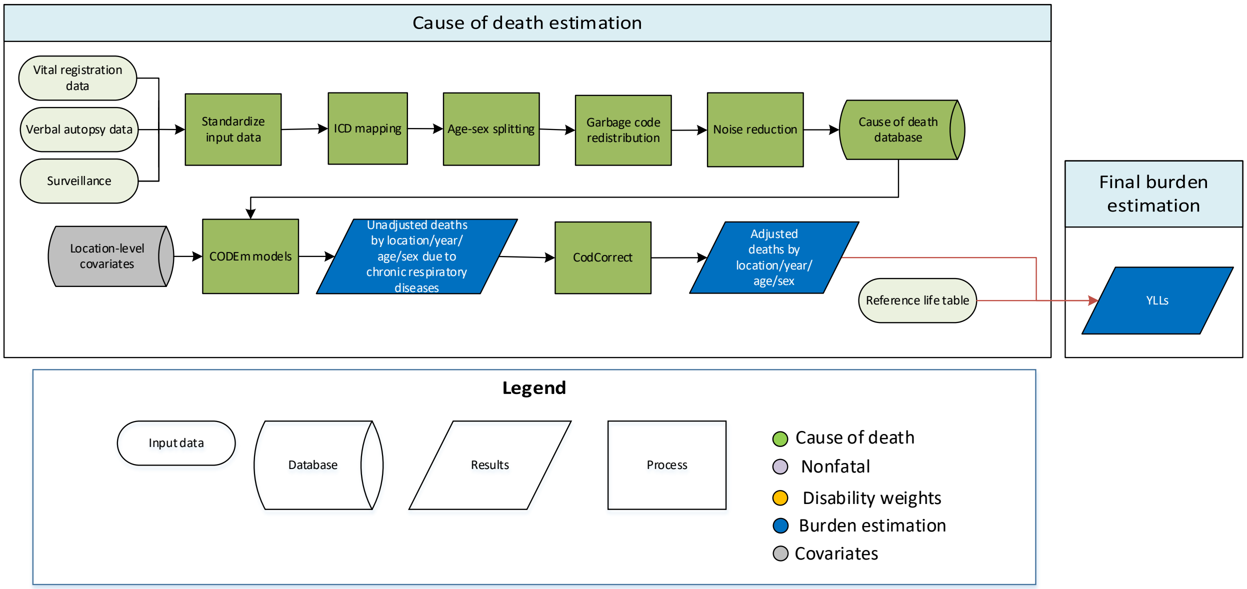

Input data

Sources used to estimate chronic respiratory disease mortality included vital registration, verbal autopsy, and surveillance data from China. Our outlier criteria excluded data points that (1) were implausibly high or low, (2) substantially conflicted with established age or temporal patterns, or (3) significantly conflicted with other data sources conducted from the same locations or locations with similar characteristics (ie, Socio-demographic Index).

Modelling strategy

The standard CODEm modelling approach was applied to estimate deaths due to chronic respiratory diseases. Separate models were conducted for male and female mortality, and the age range for both models was 1 to 95+ years.

Key Changes from GBD 2017

- We added estimates for the following new locations: Monaco, San Marino, Cook Islands, Nauru, Niue, Palau, Tokelau, Tuvalu, Monaco, San Marino, St Kitts and Nevis
- We added subnational location data for the following: Italy, Poland, Pakistan, the Philippines, and Nigeria
- We excluded all MCCD (the very incomplete hospital death data largely from urban areas) and all SCD (earlier verbal autopsy data using lesser quality instruments and analysis) from India, based on discussions with GBD India collaborators. Thus, the estimates are driven by the more recent higher quality SRS verbal autopsy data and covariates.
- Healthcare quality and access index covariate changed to a level 2 covariate from level 1.
- Smoking prevalence and indoor air pollution both moved to a level 1 covariate from level 2.
- We removed the covariate SEV for chronic respiratory disease.
- The SDI covariate was allowed to take a positive or negative direction in GBD 2017, but was specified to only be selected if a negative association was detected in GBD 2019.

The following covariates were used for GBD 2019:

Level	Covariate	Direction
1	indoor air pollution (all cooking fuels)	+
	cumulative cigarettes (10 years)	+
	cumulative cigarettes (5 years)	+
	smoking prevalence	+
2	healthcare quality and access index	-
	outdoor air pollution ($PM_{2.5}$)	+
	population above 1500m elevation (proportion)	+
3	LDI (I$ per capita)	-
	education (years per capita)	-
	socio-demographic index	-
	population between 500 and 1,500m elevation (proportion)	+
	population density over 1,000 people/kilometer^2 (proportion)	+

Chronic respiratory diseases served as a "parent" to the following causes:

- chronic obstructive pulmonary disease
- pneumoconiosis (silicosis, asbestosis, coal worker's pneumoconiosis, other pneumoconiosis)
- asthma
- interstitial lung disease and pulmonary sarcoidosis
- other chronic respiratory diseases

The unadjusted death estimates for all these "child" causes are summed and fit to the distribution of deaths estimated for the "parent" during the CODCorrect adjustment process. This results in deaths recorded using non-specific coding systems, such as verbal autopsy, being included in the parent model and redistributed to the child models proportionately. This approach assumes that deaths reported in non-specific data-sources have the same underlying distribution of specific causes as deaths reported in more specific data-sources.

Covariate Influences:

The following plots show the influence of each covariate on the four CODEm models (male global, male data rich, female global, and female data rich). A positive standardized beta (to the right) means that the covariate was associated with increased death. A negative standardized beta (to the left) means the covariate was associated with decreased death.

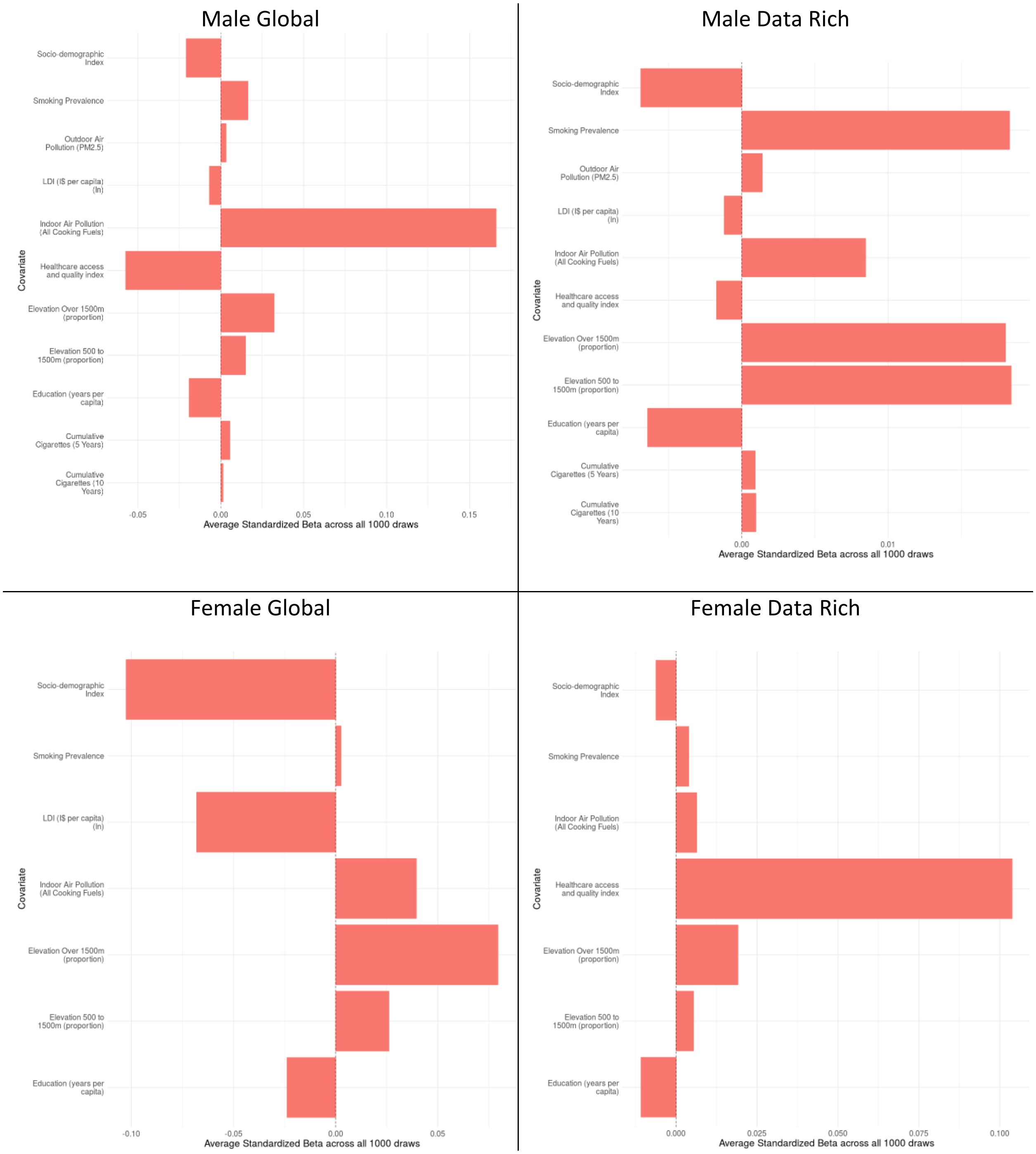

Chronic Obstructive Pulmonary Disease

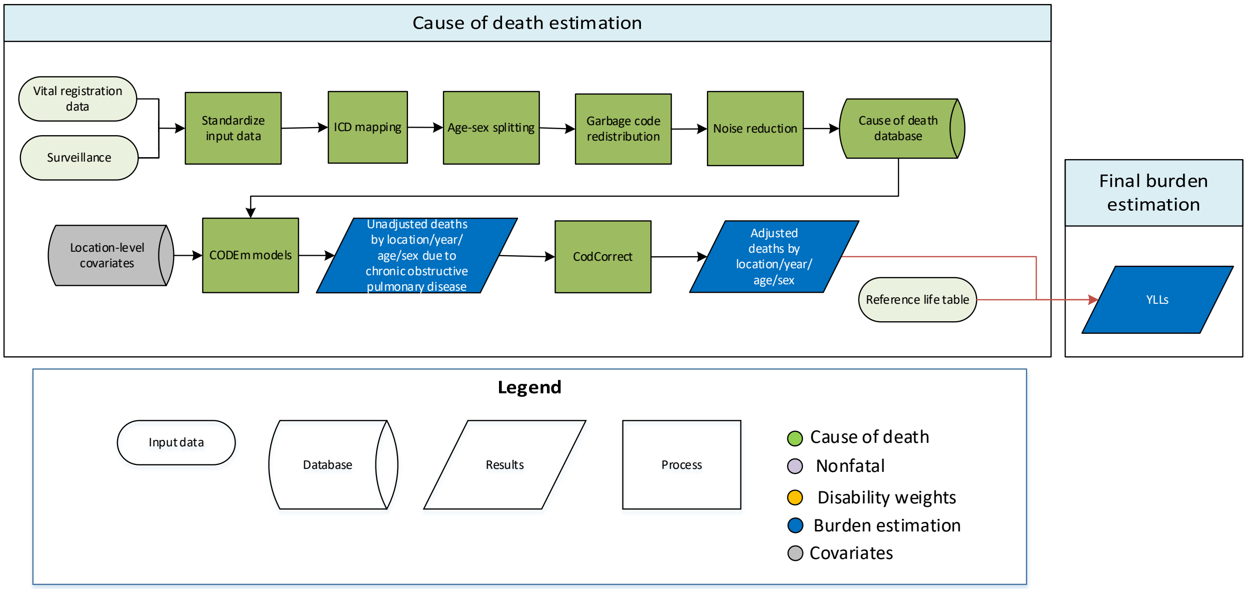

Input data

Data used to estimate chronic obstructive pulmonary disease (COPD) mortality included vital registration and surveillance data from the cause of death (COD) database. Verbal autopsy data were not included and were instead mapped to an overall chronic respiratory disease model. Our outlier criteria excluded data points that (1) were implausibly high or low, (2) substantially conflicted with established age or temporal patterns, or (3) substantially conflicted with other data sources conducted from the same locations or locations with similar characteristics (ie, Socio-demographic Index).

Modelling strategy

The standard CODEm modelling approach (as described in the relevant appendix section) was applied to estimate deaths due to COPD. Separate models were conducted for male and female mortality, and the age range for both models was 1-95+ years.

Key Changes from GBD 2017

- We added estimates for the following new locations: Monaco, San Marino, Cook Islands, Nauru, Niue, Palau, Tokelau, Tuvalu, Monaco, San Marino, St Kitts and Nevis
- We added subnational location data for the following: Italy, Poland, Pakistan, the Philippines, and Nigeria
- We added a covariate for total number of cigarettes smoked in the past 20 years, by age group. We also replaced the covariate for log income per capita with 10-year lagged income per capita.
- Outdoor air pollution covariate was moved to level 1.

The following covariates were used for GBD 2019:

Level	Covariate	Direction
1	log-transformed SEV scalar: COPD	+
	cumulative cigarettes (10 years)	+
	cumulative cigarettes (5 years)	+
	cumulative cigarettes (20 years)	+
	elevation over 1,500m (proportion)	+
	outdoor air pollution ($PM_{2.5}$)	+
2	smoking prevalence	+
	indoor air pollution (all cooking fuels)	+
	healthcare access and quality index	-
3	socio-demographic index	-
	lagged 10 year income per capita (I$ per capita)	-
	education (years per capita)	-

Chronic obstructive pulmonary disease is a “child’ disease that is fit into an overall “parent” chronic respiratory disease model. The unadjusted death estimates from COPD are summed alongside other ”child” causes (asthma, interstitial lung disease and pulmonary sarcoidosis, and pneumoconiosis) and fit to the distribution of deaths in an overall chronic respiratory disease “parent” model as part of the CODCorrect adjustment process. This results in deaths recorded using non-specific coding systems, such as verbal autopsy, being included in the parent model and redistributed to the child models proportionately.

Covariate Influences:

The following plots show the influence of each covariate on the four CODEm models (male global, male data rich, female global, and female data rich). A positive standardized beta (to the right) means that the covariate was associated with increased death. A negative standardized beta (to the left) means the covariate was associated with decreased death.

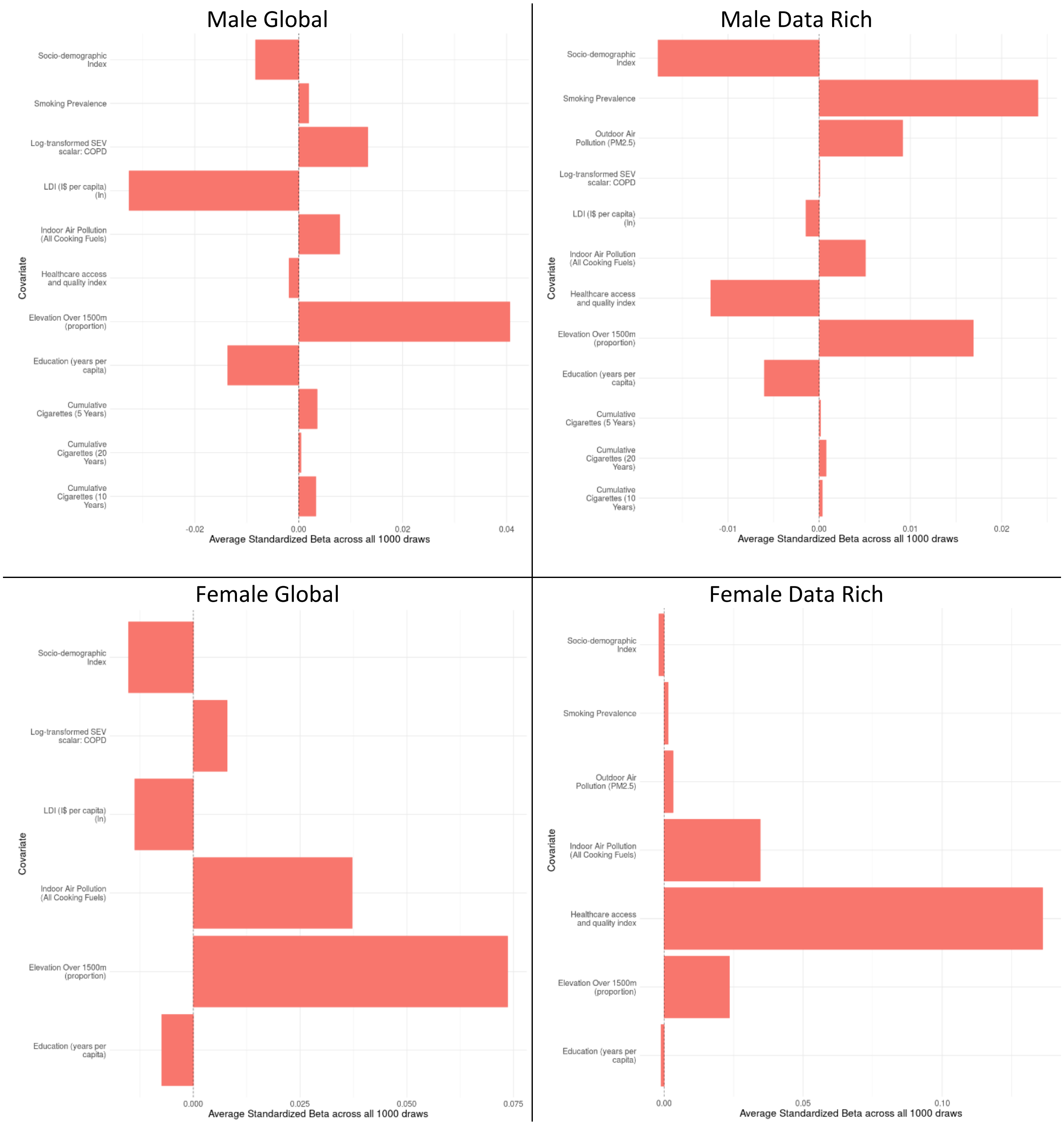

Pneumoconiosis Diseases: Silicosis, Asbestosis, Coal Worker's Pneumoconiosis, and Other Pneumoconiosis

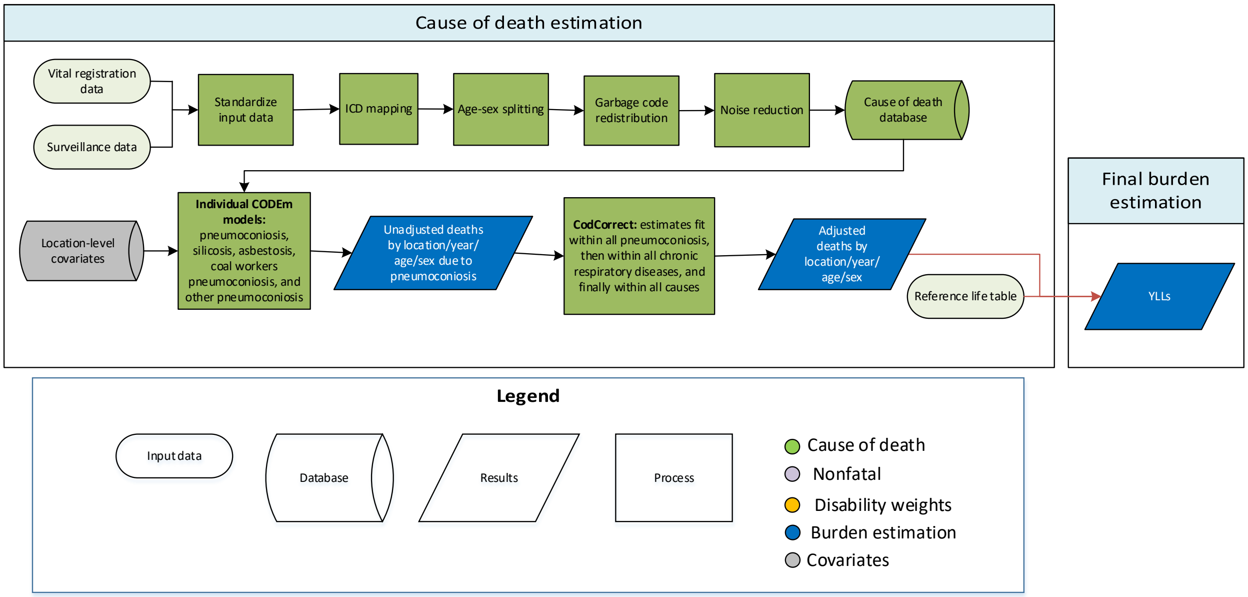

Input data

Data used to estimate pneumoconiosis mortality included vital registration and China mortality surveillance data from the cause of death (COD) database. Our outlier criteria excluded data points that (1) were implausibly high or low, (2) substantially conflicted with established age or temporal patterns, or (3) substantially conflicted with other data sources conducted from the same locations or locations with similar characteristics (ie, socio-demographic index).

Modelling strategy

The standard CODEm modelling approach was applied to estimate deaths due to pneumoconiosis diseases. Separate models were conducted for male and female mortality, and the age range for both models was 15–95+ years. The mortality estimates from pneumoconiosis disease models were ultimately fit into the chronic respiratory envelope, which is the parent cause for pneumoconiosis disease. The pneumoconiosis model serves as an envelope or "parent" model for silicosis, asbestosis, coal worker's pneumoconiosis, and other pneumoconiosis. In CoDCorrect, estimates for each of these "child" models are first fit within all pneumoconiosis, then within all chronic respiratory disease, before being fit to the all-cause mortality envelope.

Key Changes from GBD 2017

- We added estimates for the following new locations: Monaco, San Marino, Cook Islands, Nauru, Niue, Palau, Tokelau, Tuvalu, Monaco, San Marino, St Kitts and Nevis
- We added subnational location data for the following: Italy, Poland, Pakistan, the Philippines, and Nigeria
- We switched the covariate from log income per capita to a 10-year lagged income per capita and removed the elevation covariates that were previously in GBD 2017.

- We added back SEV scalars that were previously dropped in GBD 2017. These are SEVs for occupational asbestos, beryllium, and silica.

The following table indicates covariates used in the pneumoconiosis models, their level, and direction:

Level	Covariate	Direction
1	asbestos consumption per capita*	+
	coal production per capita*	+
	gold production per capita*	+
	age- and sex-specific SEV for occupational asbestos	+
	age- and sex-specific SEV for occupational beryllium	+
	age- and sex-specific SEV for occupational silica	+
2	smoking prevalence	+
	indoor air pollution (all cooking fuels)	+
	cumulative cigarettes (5 years)	+
	healthcare access and quality index	-
3	LDI (I$ per capita)	-
	education (years per capita)	-
	socio-demographic index	-

* asbestos, coal, and gold covariates are each only used in a subset of the pneumoconiosis models, as follows: all three are included in the parent all pneumoconiosis model, asbestos consumption is included in the asbestosis model, coal production is included in the coal worker's pneumoconiosis model, and gold production is included in the silicosis model.

Covariate Influences:

The following plots show the influence of each covariate on the four CODEm models (male global, male data rich, female global, and female data rich). A positive standardized beta (to the right) means that the covariate was associated with increased death. A negative standardized beta (to the left) means the covariate was associated with decreased death.

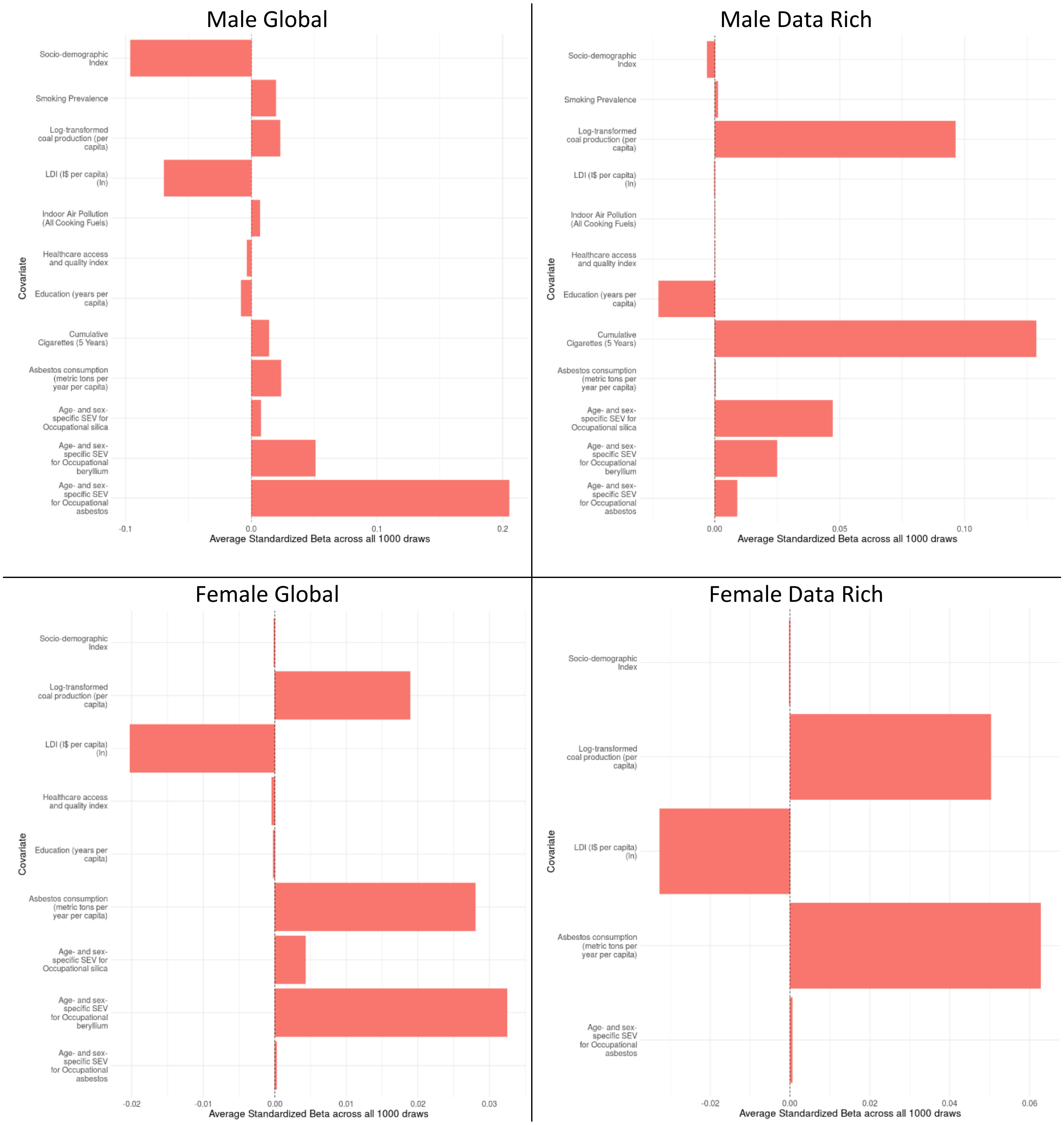

Asthma

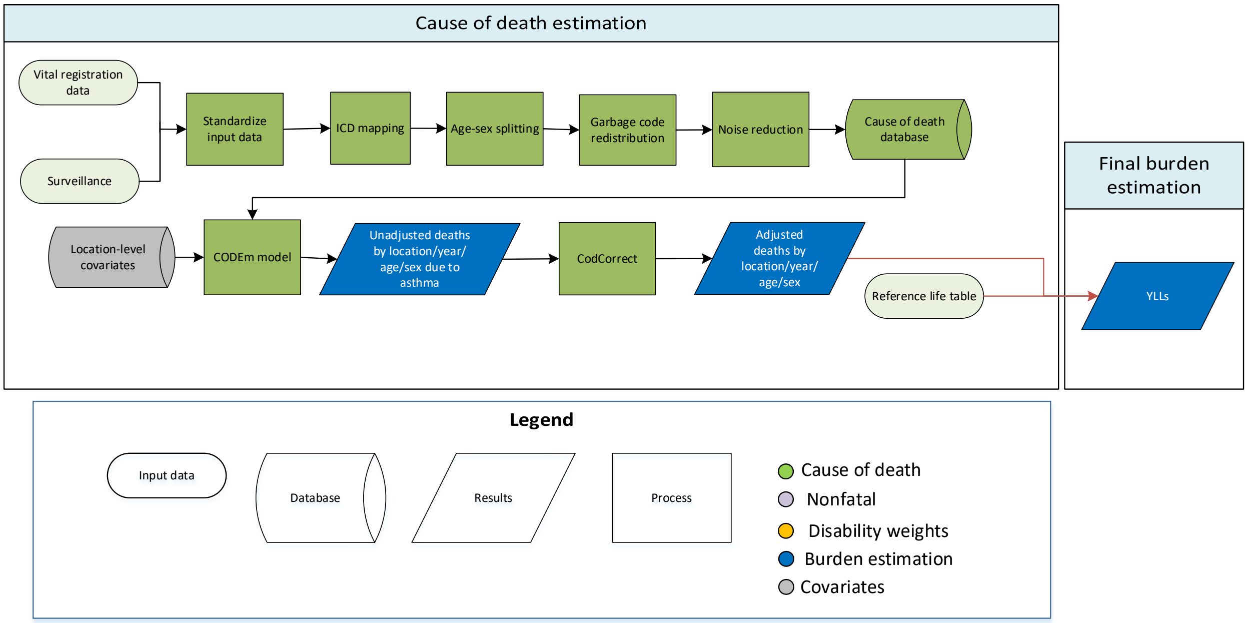

Input data

Data used to estimate asthma mortality included vital registration and surveillance data from the cause of death (COD) database. Verbal autopsy data were not included and were instead mapped to an overall chronic respiratory model. Our outlier criteria excluded data points that (1) were implausibly high or low relative to global or regional patterns, (2) substantially conflicted with established age or temporal patterns, or (3) significantly conflicted with other data sources conducted from the same locations or locations with similar characteristics (ie, Socio-demographic Index).

Modelling strategy

The standard CODEm modelling approach was applied to estimate deaths due to asthma. Separate models were conducted for male and female mortality, and the age range for both models was 1–95+ years.

Key Changes from GBD 2017

- We added estimates for the following new locations: Monaco, San Marino, Cook Islands, Nauru, Niue, Palau, Tokelau, Tuvalu, Monaco, San Marino, St Kitts and Nevis
- We added subnational location data for the following: Italy, Poland, Pakistan, the Philippines, and Nigeria
- We switched the covariate from log income per capita to a 10-year lagged income per capita.

The following table has the full list of covariates used in GBD 2019.

Level	Covariate	Direction
1	log-transformed SEV scalar: asthma	+
	cumulative cigarettes (10 years)	+
	cumulative cigarettes (5 years)	+
	healthcare access and quality index	-
2	smoking prevalence	+
	indoor air pollution (all cooking fuels)	+
	outdoor air pollution ($PM_{2.5}$)	+
3	lagged 10 year LDI (I$ per capita)	-
	education (years per capita)	-
	socio-demographic index	-

Asthma is a “child’ disease that is fit into an overall chronic respiratory disease model. In CODCorrect, the unadjusted death estimates for asthma are combined with those for chronic obstructive pulmonary disease, interstitial lung disease and pulmonary sarcoidosis, pneumoconiosis, and other chronic respiratory diseases and fit to the distribution of deaths in an overall chronic respiratory disease “parent” model. This results in deaths recorded using non-specific coding systems, such as verbal autopsy, being included in the parent model and redistributed to the child models proportionately.

Covariate Influences:

The following plots show the influence of each covariate on the four CODEm models (male global, male data rich, female global, and female data rich). A positive standardized beta (to the right) means that the covariate was associated with increased death. A negative standardized beta (to the left) means the covariate was associated with decreased death.

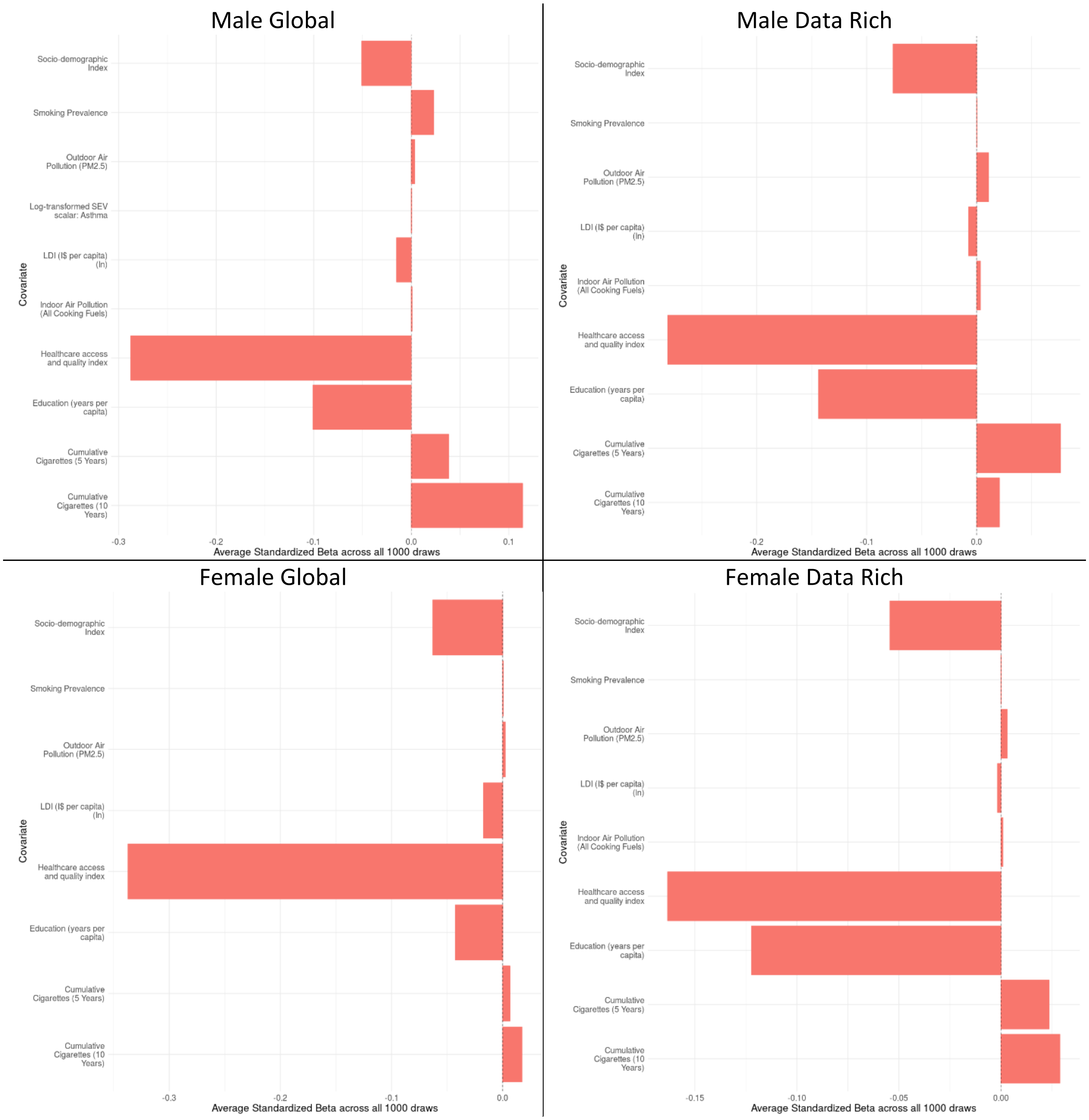

Interstitial Lung Disease and Pulmonary Sarcoidosis

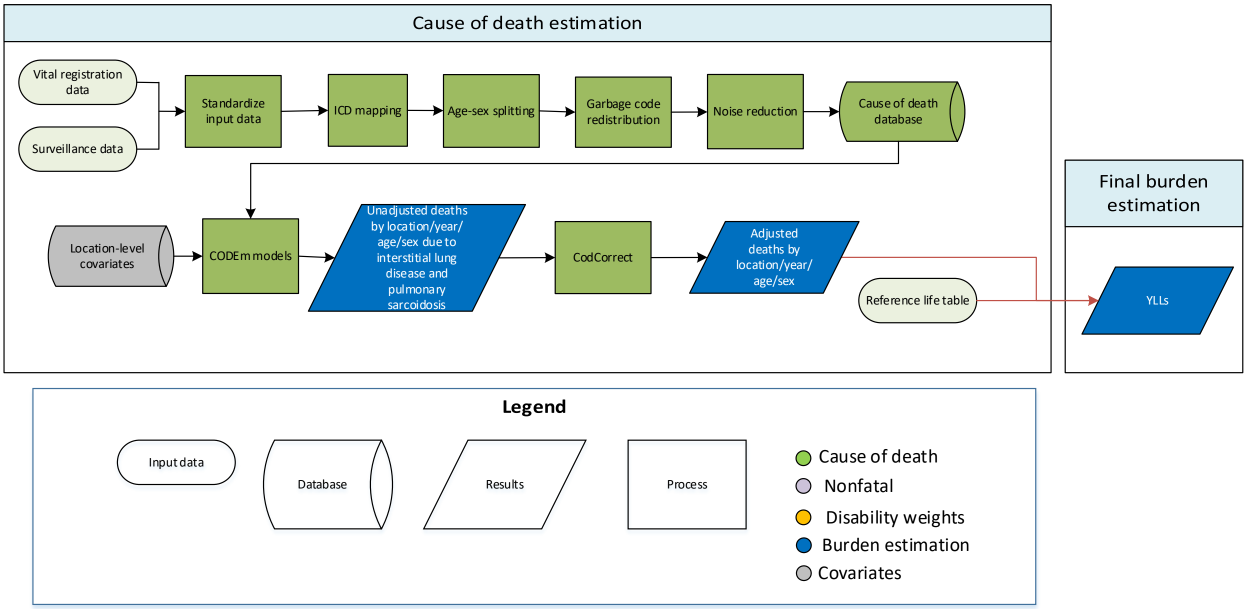

Input data

Data used to estimate interstitial lung disease and pulmonary sarcoidosis mortality included vital registration and surveillance data from the cause of death (COD) database. Our outlier criteria excluded data points that (1) were implausibly high or low, (2) substantially conflicted with established age or temporal patterns, or (3) substantially conflicted with other data sources conducted from the same locations or locations with similar characteristics (ie, Socio-demographic Index).

Modelling strategy

The standard CODEm modelling approach was applied to estimate deaths due to interstitial lung disease and pulmonary sarcoidosis. Separate models were conducted for male and female mortality, and the age range for both models was 1–95+ years.

Key Changes from GBD 2017

- We added estimates for the following new locations: Monaco, San Marino, Cook Islands, Nauru, Niue, Palau, Tokelau, Tuvalu, Monaco, San Marino, St Kitts and Nevis
- We added subnational location data for the following: Italy, Poland, Pakistan, the Philippines, and Nigeria
- We removed the covariate for the population density and added a covariate for the proportion of employed population working in professional occupations.
- The direction for the socio-demographic index covariate was changed from no direction to a negative in 2019.

The following covariates were used for GBD 2019:

Level	Covariate	Direction
1	log-transformed SEV scalar: interstitial lung disease	+
	smoking prevalence	+
	cumulative cigarettes (5 years)	+
	occupational professionals	-
2	elevation over 1,500m (proportion)	+
	elevation between 500 and 1,500m (proportion)	+
	indoor air pollution (all cooking fuels)	+
	outdoor air pollution ($PM_{2.5}$)	+
	healthcare access and quality index	-
3	log LDI (I$ per capita)	-
	education (years per capita)	-
	socio-demographic index	-

Interstitial lung disease and pulmonary sarcoidosis is a “child’ disease that is fit into an overall chronic respiratory disease model. The unadjusted death estimates from interstitial lung disease and pulmonary sarcoidosis are summed alongside other “child” causes (chronic obstructive pulmonary disease, asthma, and pneumoconiosis) and fit to the distribution of deaths in an overall chronic respiratory disease “parent” model as part of the CODCorrect adjustment process. This results in deaths recorded using non-specific coding systems, such as verbal autopsy, being included in the parent model and redistributed to the child models proportionately.

Covariate Influences:

The following plots show the influence of each covariate on the four CODEm models (male global, male data rich, female global, and female data rich). A positive standardized beta (to the right) means that the covariate was associated with increased death. A negative standardized beta (to the left) means the covariate was associated with decreased death.

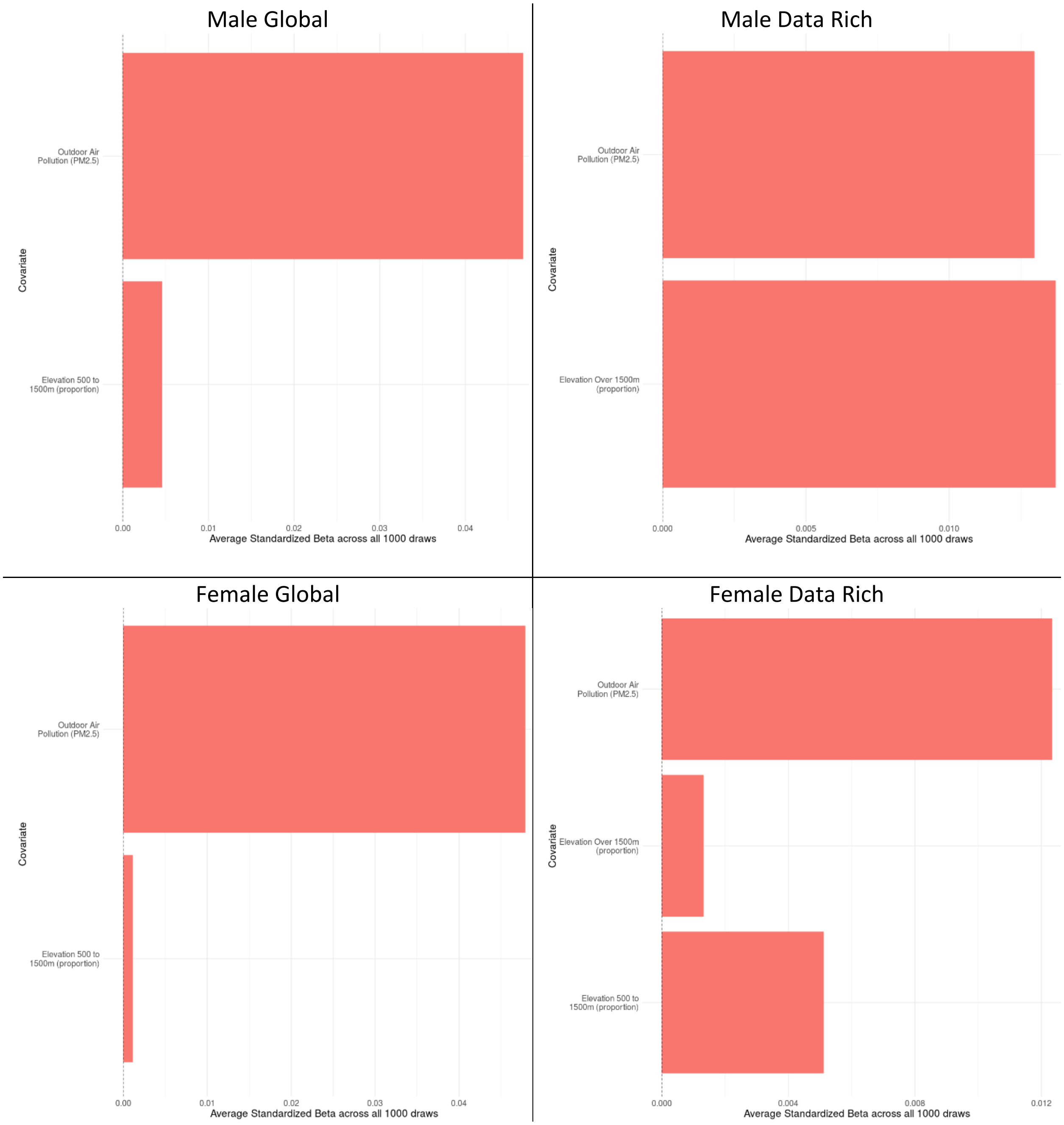

Other Chronic Respiratory Diseases

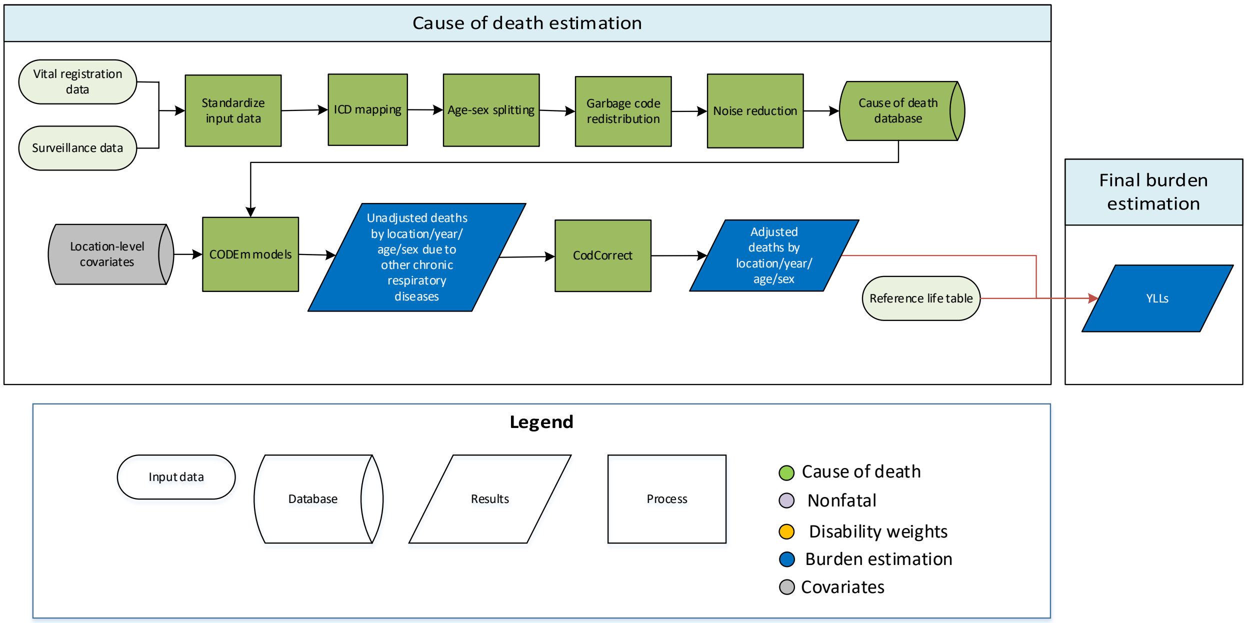

Input data

Data used to estimate other chronic respiratory diseases included vital registration and surveillance data from the cause of death (COD) database. Our outlier criteria excluded data points that (1) were implausibly high or low, (2) substantially conflicted with established age or temporal patterns, or (3) substantially conflicted with other data sources conducted from the same locations or locations with similar characteristics (ie, Socio-demographic Index).

Modelling strategy

The standard CODEm modelling approach was applied to estimate deaths due to other chronic respiratory diseases. Separate models were conducted for male and female mortality, and the age range for both models was 1 year to 95+ years.

Key Changes from GBD 2017

- We removed the log transformed SEV and changed log income per capita into a 10 year-lagged income per capita.

The following covariates were used for GBD 2019:

Level	Covariate	Direction
1	smoking prevalence	+
	cumulative cigarettes (5 years)	+
	indoor air pollution (all cooking fuels)	+

	outdoor air pollution ($PM_{2.5}$)	+
2	elevation over 1,500m (proportion)	+
	elevation between 500 and 1,500m (proportion)	+
	population density over 1,000 ppl/km^2 (proportion)	+
	healthcare access and quality index	-
3	LDI (I$ per capita)	-
	education (years per capita)	-
	socio-demographic Index	-

Other chronic respiratory is a "child' cause that is fit into an overall chronic respiratory disease model. The unadjusted death estimates from Other chronic respiratory are summed alongside unadjusted estimates for other "child" causes (chronic obstructive pulmonary disease, interstitial lung disease and pulmonary sarcoidosis, pneumoconiosis and asthma) and fit to the distribution of deaths in an overall chronic respiratory disease "parent" model. This results in deaths recorded using non-specific coding systems, such as verbal autopsy, being included in the parent model and redistributed to the child models proportionately.

Covariate Influences:

The following plots show the influence of each covariate on the four CODEm models (male global, male data rich, female global, and female data rich). A positive standardized beta (to the right) means that the covariate was associated with increased death. A negative standardized beta (to the left) means the covariate was associated with decreased death.

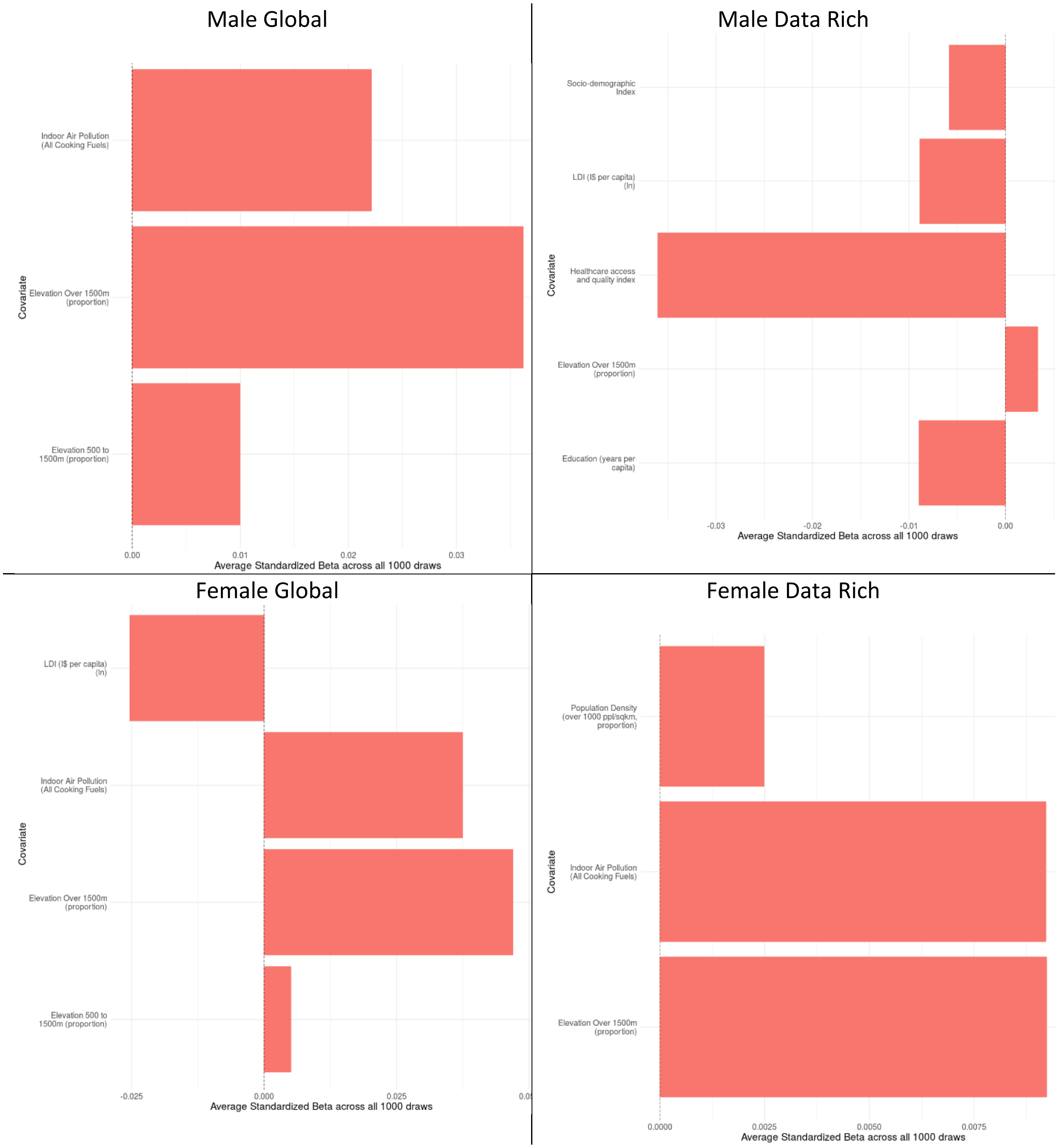

Non-fatal modeling descriptions

Chronic obstructive pulmonary disease (COPD)

Flowchart

Chronic Obstructive Pulmonary Disease (COPD)

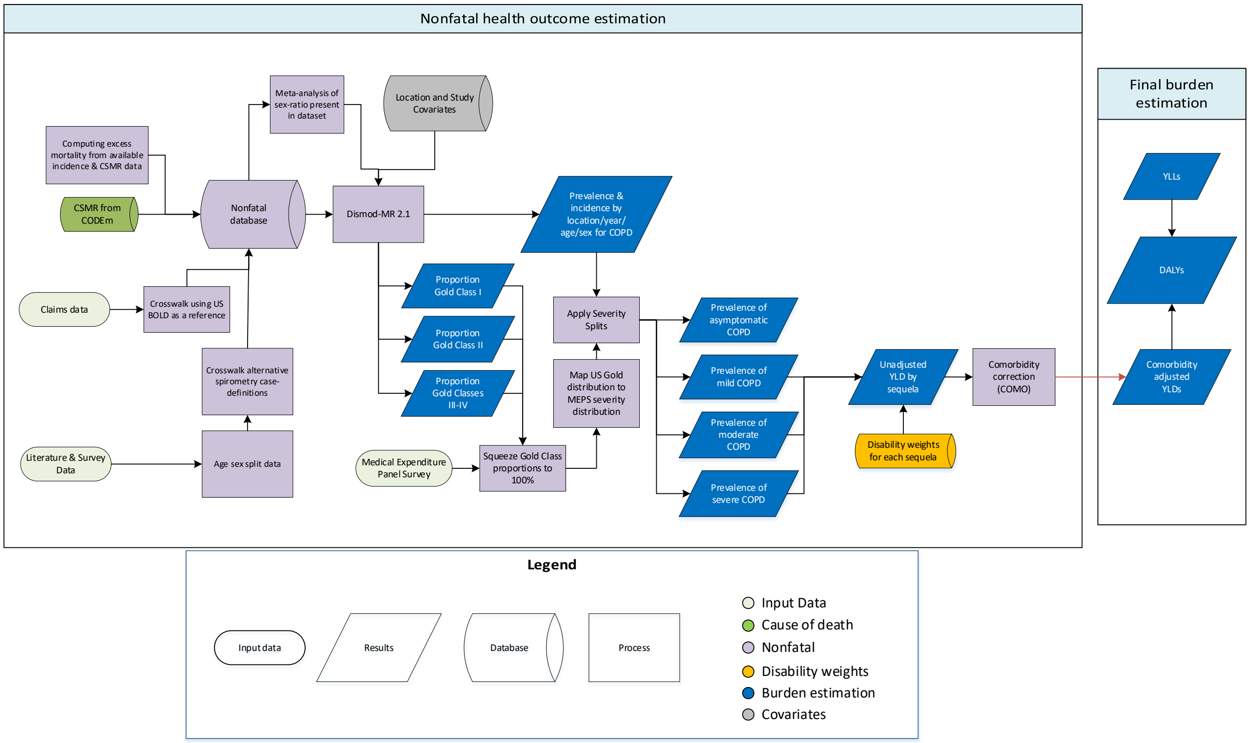

Case definition

COPD is defined as in the Global Initiative for Chronic Obstructive Lung Disease (GOLD) classification: a measurement of <0.7 FEV_1/FVC (one second of forceful exhalation/total forced expiration) on spirometry after bronchodilation. The severity grading of COPD follows this GOLD class definition.

GOLD CLASS	FEV_1 Score
I: Mild	>=80% of normal
II: Moderate	50-79% of normal
IV: Severe	<50% of normal

ICD-10 codes associated with COPD include J41, J42, J43, J44, and J47. The corresponding ICD-9 codes are 491-492, and 496. J40 & 490 (Bronchitis, not specified as acute or chronic) and J47 & 494 (Bronchiectasis) were removed from COPD mapping in GBD 2017.

Alternative case definitions that differ from the GOLD Post-bronchodilation definition are as follows: GOLD Pre-bronchodilation, Lower Limit of Normal (LLN) Post-bronchodilation, LLN Pre-bronchodilation, and European Respiratory Society (ERS) guidelines. These are all different methods of evaluating whether an individual has COPD.

Input data

No systematic review of the literature was completed for GBD 2019; however, for GBD 2016, we updated the systematic review from previous iterations. The full search term was:

(chronic obstructive pulmonary disease[Title/Abstract] AND (prevalence[Title/Abstract] or incidence [Title/Abstract] or mortality [Title/Abstract] or death [Title/Abstract]) AND "Cross-Sectional Studies"[MeSH Terms]) Filters: Publication date from 04/01/2015 to 11/01/2016; Humans

COPD has the following data sources

- Prevalence, incidence, and remission data from literature
- Hospital claims data
- Proportion data of GOLD class severities
- Burden of Obstructive Lung Disease (BOLD) Study data

Prevalence, incidence, and remission data relating to COPD are extracted from literature provided by collaborators or found with a systematic review. All data include spirometry-based measures. Other data come from hospital claims data for nonfatal estimation and vital registrations for cause of death.

GOLD class proportions are extracted from literature when the severity is available. Our models estimate three separate severities:

- Mild COPD: GOLD class I
- Moderate COPD: Gold class II
- Severe COPD: Gold class III & IV

These severities are used in the modelling process to split COPD by severities.

The Burden of Obstructive Lung Disease (BOLD) data is specifically notable because of its use in bias adjustments described in the data processing section.

New data this year include the English Longitudinal Study of Aging (ELSA), and claims data for the United States. Additional information on the claims data collection and pre-corrections are provided elsewhere. Briefly, we determined USA national and state-level estimates of COPD prevalence from a database of individual-level ICD-coded health service encounters. Persons with any inpatient claim or at least two outpatient claims associated with COPD were marked as a prevalent case for that year.

Data Inputs for Chronic Obstructive Pulmonary Disease

Measure	Total sources	Countries with data
All measures	166	57
Prevalence	142	54
Incidence	6	6
Relative risk	2	2
Proportion	36	32

Data Processing

Age-Sex and Sex Split

In some cases, data are reported by only age or only sex, but not both. For example, a study may have included the prevalence of males and females with COPD and then separately reported the prevalence for both sexes in smaller age bins (e.g. age 40-45, 46-50, etc.) that have COPD. In these cases, we perform an age-sex split by utilizing proportions within the study to disaggregate the data.

When data are not disaggregated into male and female categories for a given data source, we instead perform a sex-split on the data by applying sex proportions from other studies that do have male and female specific data. When data are aggregated into age categories larger than 25 years, we split into smaller age bins based on super-regional age patterns in the 2017 COPD model.

Modeled excess mortality data

For GBD 2019, we implemented a new method of modeling excess mortality rate (EMR).

In previous rounds, priors on EMR were estimated in DisMod by matching prevalence data points with their corresponding CSMR values within the same age, sex, year, location (by dividing CSMR by prevalence).

However, for many causes, DisMod estimated a rather unrealistic pattern of EMR compared to an expected pattern of decreasing EMR with greater access to quality health care. Such unexpected patterns often signal inconsistencies between CSMR estimates and the measures of prevalence and/or incidence.

In an effort to provide greater guidance to DisMod on the expected pattern of EMR, EMR data generated in the previous round were used as inputs for modeling in MR-BRT with age, sex, and healthcare access and quality index (HAQi) included as covariates. Results from MR-BRT were then predicted for each location year, sex and for ages 0, 10, 20 ….100.

This method led to improvements in the consistency of EMR relative to health care access. We also included HAQi as a country-level covariate in Dismod to inform EMR with the mean and standard deviation produced from MR-BRT analysis.

Bias Adjustments

In GBD 2019, we improved the bias adjustment methods by utilizing a MR-BRT model outside of DisMod to allow a more direct comparison between different case definitions and/or study designs. In GBD 2017, these adjustments were performed within DisMod.

We made a series of adjustments to data that do not completely match our case definition. Different diagnosis often leads to different estimates of COPD. Similarly, claims data is subject to biases. Claims data are often systemically lower than survey data, probably due to selection bias with regard to socioeconomic status. Adjustments are made to these data to correct these biases.

The adjustment is a logit-transformation method in MR-BRT. The general process is described below:

1. Identify data points with overlapping year, age, sex, and location between reference and alternative definitions.
2. Logit transform overlapping data points of alternative and reference case definitions
3. Convert overlapping data points into a difference in logit space using the following equation: $logit(altnerative) - logit(reference)$
4. Use the delta method to compute standard errors of overlapping data points in logit space, then calculate standard error of logit difference using the following equation: $\sqrt{(variance\ of\ alternative) + (variance\ of\ reference)}$
5. Using MR-BRT, conduct a random effects meta-regression to obtain the pooled logit difference of alternative to reference
6. Apply the pooled logit difference to all data points of alternative case definitions using the following equation:
   $new_{estimate} = inverse.logit((logit(alternative)) - (pooled\ logit\ difference))$
7. Calculate new standard errors using the delta method, accounting for gamma (between-study heterogeneity)

Data derived from claims from commercial health insurance in the United States were also adjusted using a factor estimated in MR-BRT. Claims data, notably US Marketscan was adjusted in relation to the BOLD study data. In this case, the BOLD data serves as the reference definition while the marketscan data are the alternative definition.

MR-BRT Crosswalk Adjustment Factors

Data input	Status	Gamma	Beta Coefficient, Logit (95% CI)	Adjustment factor*
GOLD Post	Ref	0.25	---	---
GOLD Pre	Alt		0.50 (-0.02 - 1.07)	0.62 (0.49 - 0.74)
ERS	Alt		0.70 (0.11 - 1.31)	0.67 (0.53 - 0.79)
LLN Pre	Alt	0.08	0.10 (0.01 - 0.19)	0.52 (0.50 - 0.55)
LLN Post			-0.34 (-0.50 - -0.19)	0.42 (0.38 -0.45)
BOLD	Ref	.19	---	---
Marketscan	Alt		-1.93 (-2.35 - -1.50)	0.13 (0.08 - 0.18)

Adjustment factor is the inverse-logit transformed Beta coefficient; <0.5 represents that alternative is adjusted upward; >0.5 represents alternative is adjusted downward

Modelling strategy

The estimation of COPD burden has two distinct steps.

1. Estimate prevalence and incidence using a DisMod-MR 2.1 model
2. Estimate proportion of COPD severities using GOLD class groupings in DisMod-MR 2.1

After these two steps, the COPD prevalence and incidence is split by age, sex, location for each severity level.

Step 1: Main COPD model – Estimate prevalence and incidence using DisMod-MR 2.1

Model Settings

We set remission to 0 because individuals do not recover once they have COPD. The symptoms are only managed. Incidence ceiling is set at .0002 before age 15 and a ceiling at .0005 before age 30 to avoid a kick-up of estimates in age ranges with few or no primary data.

Each model includes a series of country-level covariates that describe spatiotemporal patterns.

- COPD standardised exposure variables (SEV) aggregates multiple risk factors into a single variable.
- Healthcare Access and Quality (HAQi) index on EMR to capture country-level variation of EMR, assuming a negative coefficient (ie, lower mortality with rising GDP and HAQ). The priors of HAQi came from the EMR MRBRT prediction.
- The proportion of elevation over 1500m was included as a country-level covariate on EMR because of its significance in COPD cause of death models.

Model coefficients for COPD

Model	Variable name	Measure	Beta	Exponentiated
COPD	Elevation over 1500m (proportion)	excess mortality rate	0.60<br>(0.14 — 0.95)	1.81<br>(1.15 — 2.58)
COPD	Healthcare access and quality index	excess mortality rate	-0.022<br>(-0.023 — -0.022)	0.98<br>(0.98 — 0.98)
COPD	Log age-standardised SEV scalar: COPD	prevalence	0.91<br>(0.90 — 0.92)	2.47<br>(2.46 — 2.50)

Step 2: GOLD class models to estimate proportions of severities

The GOLD class models use data from surveys that specified prevalence by GOLD class after expressing the values as a proportion of all COPD cases. For GBD 2016 we used fixed effects from the SEV scalar and the log of lag-distributed income (LDI) per capita to assist estimation. For GBD 2017, we dropped these covariates because they did not produce significant coefficients and also did not use them for GBD 2019. We also restricted random effects to +/-0.5 to control implausible geographical variation.

Severity Splits

The three GOLD class groupings reflect a grading based on a physiological measurement rather than a direct measurement of disease severity. In order to map the epidemiological findings by GOLD class into

the three COPD health states for which we have disability weights (DW), we used the 2001–2011 Medical Expenditure Panel Survey (MEPS) data from the United States. Specifically, we convert the GOLD class designations estimated for the USA in 2005 (the midpoint of MEPS years of analyses) into GBD classifications of asymptomatic, mild, moderate, and severe COPD.

The table below shows the three health states of COPD and the corresponding lay descriptions and disability weights. The graph shows the average proportion by GOLD class (after scaling to 100%) across all ages for USA in 2005. We also show the proportion of MEPS respondents reporting any health service contact in the past year for COPD with a DW value attributable to COPD of 0, mild range (0 to midpoint between DWs for mild and moderate), moderate range (midpoint of DW values mild and moderate to midpoint of DW values for moderate and severe) and severe range (midpoint between DW values moderate and severe or higher). The DW value for COPD was derived from a regression with indicator variables for all health states reported by MEPS respondents and their reported overall level of disability derived from a conversion of 12-Item Short Form Surveys (SF-12) answers to GBD DW values. This analysis gave the severity distribution for each GBD cause reported in MEPS after correcting for any comorbid causes individual respondents reported during a year.

Description of Health States

Health state	Lay description	DW (95% CI)
Mild COPD	This person has cough and shortness of breath after heavy physical activity, but is able to walk long distances and climb stairs.	0.019 (0.011–0.033)
Moderate COPD	This person has cough, wheezing, and shortness of breath, even after light physical activity. The person feels tired and can walk only short distances or climb only a few stairs.	0.225 (0.153–0.31)
Severe COPD	This person has cough, wheezing, and shortness of breath all the time. The person has great difficulty walking even short distances or climbing any stairs, feels tired when at rest, and is anxious.	0.408 (0.273–0.556)

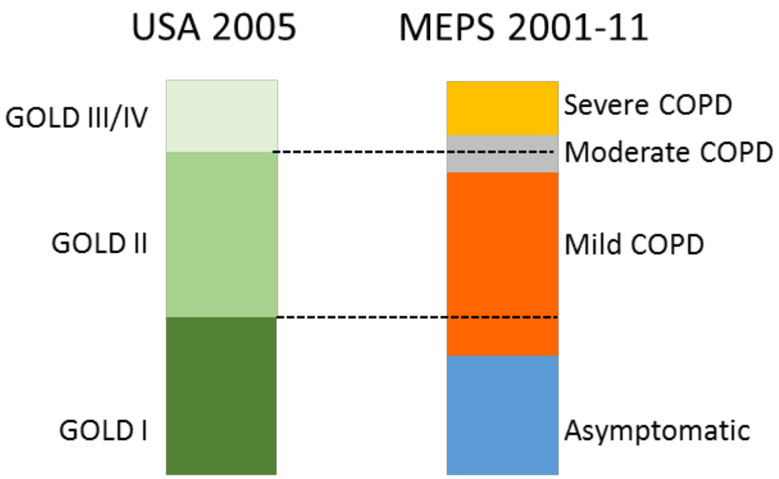

The algorithm to translate GOLD class to COPD DW categories first assigns GOLD III&IV to severe COPD and what remains to moderate. Next, GOLD class I is assigned to the asymptomatic category first and what remains goes to mild COPD. This algorithm is repeated for each age and sex category and for all 1,000 draws from the DisMod models of GOLD classes and the MEPS analyses. We end up with proportions of each of the GOLD class categories that map onto GBD COPD health states with uncertainty bounds determined by the 25th and 975th values of the 1,000 draws. These values are then applied to the estimates of the proportion of cases by GOLD class category, after scaling to 100%, by location, year, age, and sex. This assumes that the relationship between GOLD class and GBD COPD health states in the United States applies everywhere.

Pneumoconiosis

Coal Worker's Pneumoconiosis, Asbestosis, Silicosis, and Other Pneumoconiosis

Flowchart

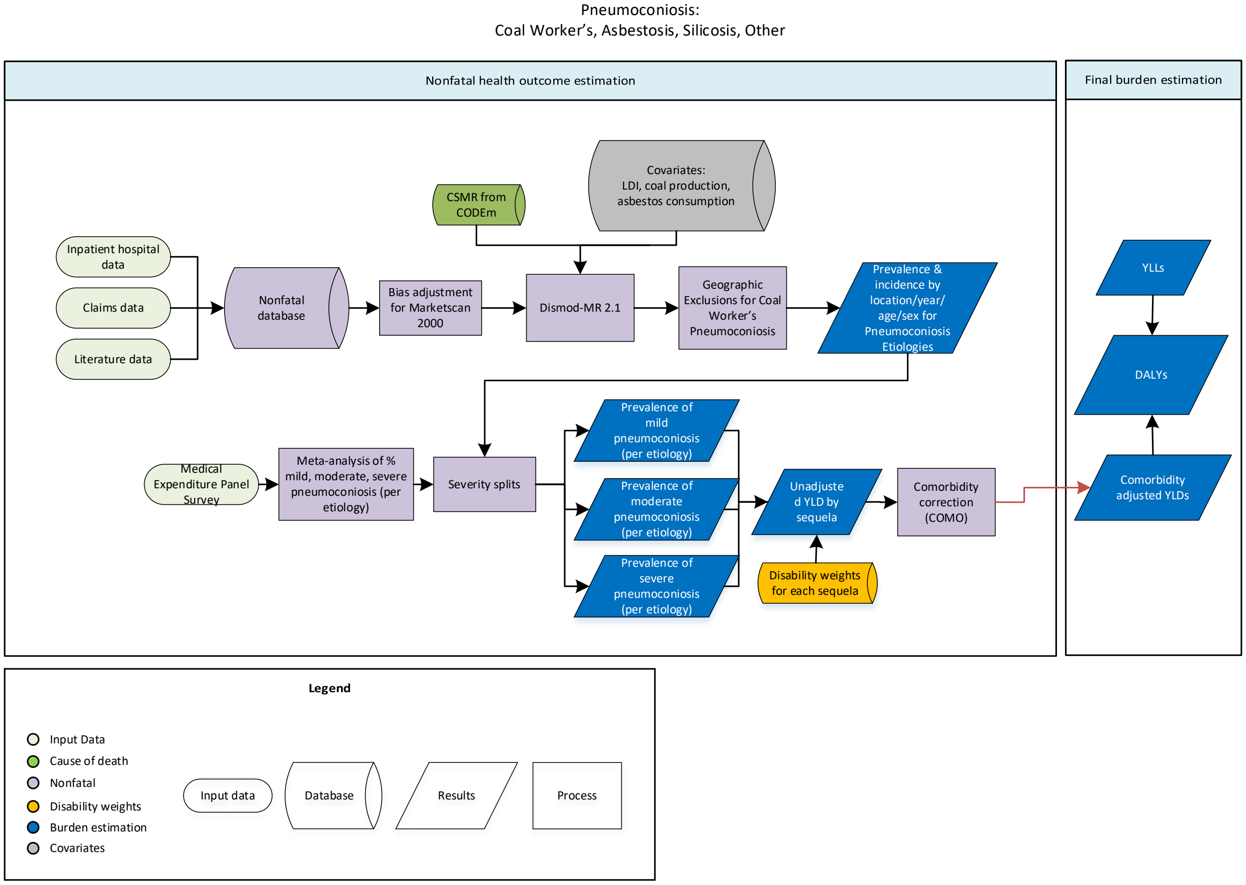

Input data and methodological appendix

Case definition

Pneumoconiosis is a chronic lung disease characterized by lung scarring and other interstitial damage caused by exposure to dust and other containments – usually through occupational exposure. For GBD, we model pneumoconiosis by exposure type: coal, asbestos, silica, and other.

Input data

Data used to make estimates of pneumoconiosis come from two sources: inpatient hospital reports, and hospital claims data. For GBD 2019, new claims data were added for the U.S. for the years 2015 and 2016.

Data Inputs for Pneumoconiosis

Cause/Impairment Name	Measure	Total sources	Countries with data
Pneumoconiosis	All measures	309	44
Pneumoconiosis	Prevalence	294	44
Pneumoconiosis	Proportion	15	1
Asbestosis	All measures	279	37
Asbestosis	Prevalence	279	37
Coal workers pneumoconiosis	All measures	251	35
Coal workers pneumoconiosis	Prevalence	251	35
Other pneumoconiosis	All measures	259	41
Other pneumoconiosis	Prevalence	259	41

Data Processing

Bias Adjustments

In GBD 2019, we improved the bias adjustment methods by utilizing a MR-BRT model outside of DisMod to allow a more direct comparison between different case definitions and/or study designs.

For the pneumoconiosis, adjusted U.S. Marketscan claims data collected in th year 2000 to all other U.S. Marketscan data. To do so, we used the logit difference for data points from reference (non-2000 claims data) and alternative (2000 claims data) matched on age, sex and location as input into MR-BRT.

The adjustment is a logit-transformation method in MR-BRT. The general process is described below:

1. Identify data points with overlapping age, sex, and location between reference and alternative definitions.
2. Logit transform overlapping data points of alternative and reference case definitions
3. Convert overlapping data points into a difference in logit space using the following equation: $logit(altnerative) - logit(reference)$
4. Use the delta method to compute standard errors of overlapping data points in logit space, then calculate standard error of logit difference using the following equation: $\sqrt{(variance\ of\ alternative) + (variance\ of\ reference)}$
5. Using MR-BRT, conduct a random effects meta-regression to obtain the pooled logit difference of alternative to reference
6. Apply the pooled logit difference to all data points of alternative case definitions using the following equation: $new_{estimate} = inverse.logit((logit(alternative)) - (pooled\ logit\ difference))$
7. Calculate new standard errors using the delta method, accounting for gamma (between-study heterogeneity)

The coefficients for bias adjustments are shown below:

MR-BRT Crosswalk Adjustment Factor: Asbestosis

Data input	Status	Gamma	Beta Coefficient, Logit (95% CI)	Adjustment factor*

Marketscan (not 2000)	Ref		---	---
Marketscan 2000	Alt	0.0	-0.25 (-0.36 to -0.15)	0.44

**Adjustment factor is the inverse-logit transformed Beta coefficient; <0.5 represents that alternative is adjusted upward; >0.5 represents alternative is adjusted downward*

MR-BRT Crosswalk Adjustment Factor: Coal Worker's Pneumoconiosis

Data input	Status	Gamma	Beta Coefficient, Logit (95% CI)	Adjustment factor*
Marketscan (not 2000)	Ref		---	---
Marketscan 2000	Alt	0.0	-0.34 (-0.76 to 0.07)	0.42

**Adjustment factor is the inverse-logit transformed Beta coefficient; <0.5 represents that alternative is adjusted upward; >0.5 represents alternative is adjusted downward*

MR-BRT Crosswalk Adjustment Factor: Silicosis

Data input	Status	Gamma	Beta Coefficient, Logit (95% CI)	Adjustment factor*
Marketscan (not 2000)	Ref		---	---
Marketscan 2000	Alt	0.0	-0.48 (-1.91 to 0.96)	0.38

**Adjustment factor is the inverse-logit transformed Beta coefficient; <0.5 represents that alternative is adjusted upward; >0.5 represents alternative is adjusted downward*

MR-BRT Crosswalk Adjustment Factor: Other Pneumoconiosis

Data input	Status	Gamma	Beta Coefficient, Logit (95% CI)	Adjustment factor*
Marketscan (not 2000)	Ref		---	---
Marketscan 2000	Alt	0.0	0.14 (-0.32 to 0.59)	0.53

**Adjustment factor is the inverse-logit transformed Beta coefficient; <0.5 represents that alternative is adjusted upward; >0.5 represents alternative is adjusted downward*

Modeled excess mortality data

As part of iteration of estimates for all pneumoconioses, we tested a new method of modeling excess mortality rate (EMR) that was not used in the final model.

In previous rounds, priors on EMR were estimated in DisMod by matching prevalence data points with their corresponding CSMR values within the same age, sex, year, location (by dividing CSMR by prevalence). For short duration conditions (remission>1), the corresponding prevalence was derived by running an initial model and then applying the same CSMR/prevalence method.

However, for many causes, DisMod estimated a rather unrealistic pattern of EMR compared to an expected pattern of decreasing EMR with greater access to quality health care. Such unexpected patterns often signal inconsistencies between CSMR estimates and the measures of prevalence and/or incidence.

In effort to provide greater guidance to DisMod on the expected pattern of EMR, EMR data generated in the previous round were used as inputs in MR-BRT modeling with age, sex, and healthcare access and quality index (HAQi) included as covariates. Results from MR-BRT were then predicted for each location year, sex and for ages 0, 10, 20 ....100.

While this method led to some improvements in the consistency of EMR relative to health care access, the resulting prevalence estimates were unrealistic. We decided to continue using the DisMod EMR estimations while keeping HAQi as a country-level covariate to inform EMR with a mean and standard deviation produced from MR-BRT.

This method was utilized for all pneumoconiosis: asbestosis, coal worker's pneumoconiosis, silicosis, and other pneumoconiosis.

Modelling strategy

Estimates for the pneumoconioses are produced using a standard DisMod-MR 2.1 approach.

For all aetiologies, we use prior settings of zero remission. Additionally, we assume no incidence and prevalence before the age of 15. We include a predictive covariate on healthcare access and quality. Location random effects are set at -20 to 20 for prevalence and incidence hazard to reflect large location variations.

Covariates on Asbestos, Mesothelioma, and coal production were removed in GBD 2019.

Cause	Measure	Variable name	Beta	Exponentiated
Asbestosis	Prevalence	Asbestos consumption (per capita)	0.47 (0.015–1.70)	1.60 (1.02–5.47)
Asbestosis	Prevalence	Log-transformed age-standardised SEV scalar: Mesothelioma	0.029 (0.000016–0.32)	1.03 (1.00–1.38)
Asbestosis	Excess Mortality	Healthcare access and quality index	-0.025 (-0.025 — -0.024)	0.98 (0.98 — 0.98)
Coal worker's	Prevalence	Coal production (per capita)	0.0017 (-0.00025 to 0.0045)	1.00 (1.00–1.00)
Coal Worker's	Excess Mortality	Healthcare Access and Quality index	-0.07809	0.013502

Severity Split Inputs

Data to inform estimates of the severity gradient due to pneumoconiosis etiologies are derived from previous analyses of the Medical Expenditure Panel Survey (MEPS). The disability weights are shared by all aetiologies.

Severity level	Lay description	DW (95% CI)	Severity Distributions
Asymptomatic			23.0% (20.8 – 25.0)
Mild	Has cough and shortness of breath after heavy physical activity, but is able to walk long distances and climb stairs.	0.019 (0.011–0.033)	34.2% (26.4 – 37.5)
Moderate	Has cough, wheezing, and shortness of breath, even after light physical activity. The person feels tired and can walk only short distances or climb only a few stairs.	0.225 (0.153–0.312)	13.3% (9.7 – 19.4)
Severe	Has cough, wheezing, and shortness of breath all the time. The person has great difficulty walking even short distances or climbing any stairs, feels tired when at rest, and is anxious.	0.408 (0.273–0.556)	29.5 (20.8 – 36.1)

Geographical Exclusions

In GBD 2019, we set estimates for coal worker's pneumoconiosis to zero prevalence for any location with no coal production for all years. This exclusion was applied after running a DisMod model. The assumption here is that coal worker's pneumoconiosis should be near zero in areas where there is no coal production.

Asthma

Flowchart

Asthma

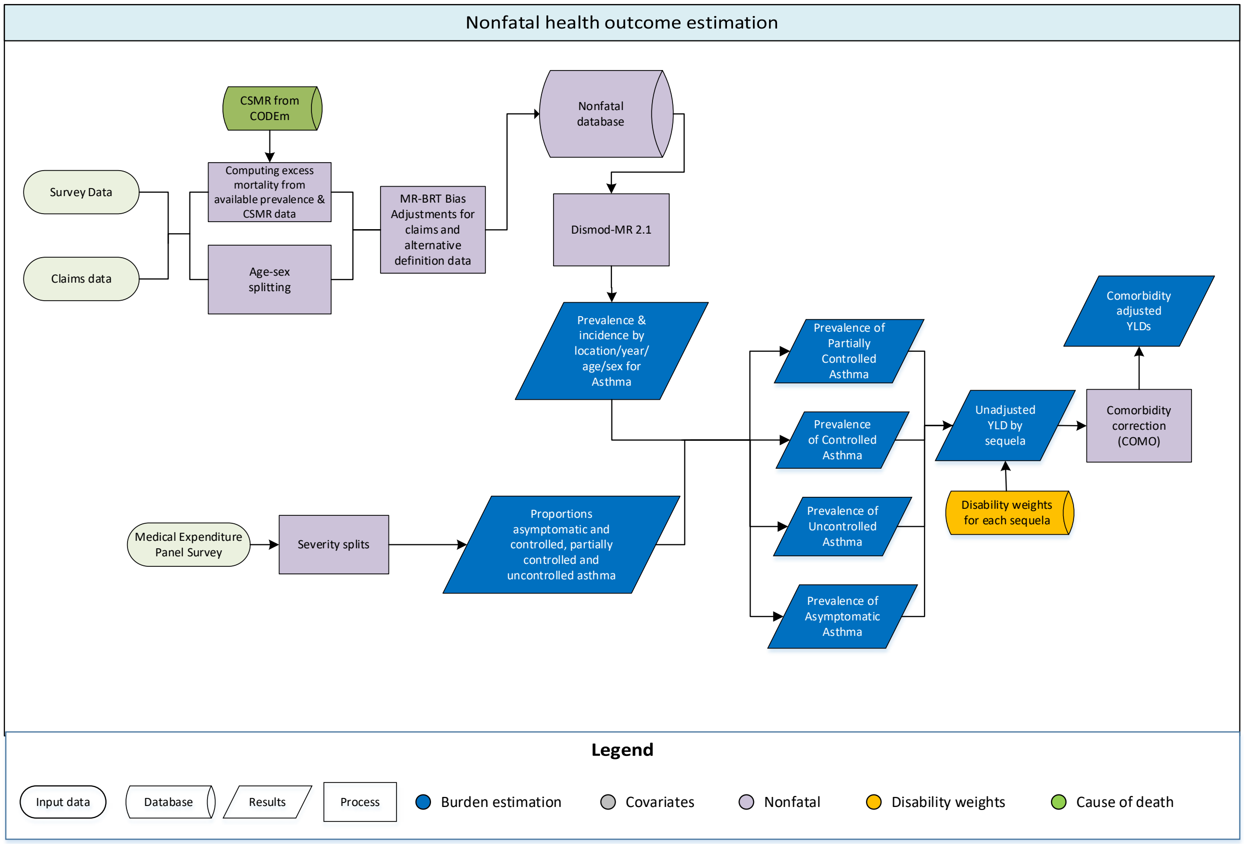

Case definition

Asthma is a chronic lung disease marked by spasms in the bronchi usually resulting from an allergic reaction or hypersensitivity and causing difficulty in breathing. We define asthma as a doctor's diagnosis and wheezing in the past year. The relevant ICD-10 codes are J45 and J46. ICD-9 code is 493.

Alternative case definitions include the following:

- Self-reported Asthma in the past year
- Self-reported Asthma ever
- Only a doctor's diagnosis in the past year
- Only wheezing in the past year

Input data

The last full systemic review of the literature on Asthma was done for GBD 2016. The following search string was used in PubMed and filtered by studies of humans published between January 2012 and November 2016.

(Asthma[Title/Abstract] AND prevalence[Title/Abstract] AND "Cross-Sectional Studies"[MeSH Terms])

Data in literature matching our case definitions were extracted. Those that had definitions outside our alternative case definitions were not included. In addition to claims data used in GBD 2017, we added new USA claims data for the years 2015 and 2016. We also added new data for Wave 7 of the English Longitudinal Study of Ageing (ELSA). Surveys carried out as part of the International Study of Asthma and Allergies in Childhood (ISAAC) collaboration are the most important source of prevalence data in children.

Data Inputs for Asthma

Measure	Total sources	Countries with data
All measures	413	136
Prevalence	374	136
Incidence	11	6
Remission	28	16
Relative risk	5	3
Standardized mortality ratio	1	1
With-condition mortality rate	4	2
Proportion	15	1

Data processing

Age-Sex and Sex Split

In some cases, data are reported by only age or only sex, but not both. For example, a study may have included the prevalence of males and females with Asthma and then separately reported the prevalence of both sexes combined in smaller age bins (e.g. 40-45 years, 46-50, etc.) that have Asthma. In these cases, we perform an age-sex split by utilizing proportions within the study to disaggregate the data.

When data are not disaggregated into male and female categories, we instead perform a sex-split on the data by applying sex proportions from outside studies. The sex split analysis was carried out using MR-BRT and included a cubic spline on age to reflect the higher prevalence of asthma in males at young ages, which then transitions to a higher prevalence of asthma in females during the teenage years.
When data are aggregated into age categories larger than 25 years, we split the data into smaller age bins based on the global age pattern from an initial Dismod model that only included input data with age ranges under 25 years.

Modeled excess mortality data

As part of iteration of estimates for Asthma, we tested a new method of modeling excess mortality rate (EMR) that was not used in the final model.

In previous rounds, priors on EMR were estimated in DisMod by matching prevalence data points with their corresponding CSMR values within the same age, sex, year, location (by dividing CSMR by prevalence).

However, for many causes, DisMod estimated a rather unrealistic pattern of EMR compared to an expected pattern of decreasing EMR with greater access to quality health care. Such unexpected patterns often signal inconsistencies between CSMR estimates and the measures of prevalence and/or incidence.

In effort to provide greater guidance to DisMod on the expected pattern of EMR, EMR data generated in the previous GBD round were used as inputs into aMR-BRT model that includedage, sex and healthcare access and quality index (HAQi) as covariates. Results from MR-BRT were then predicted for each location year, sex and for ages 0, 10, 20 ....100.

While this method led to some improvements in the consistency of EMR relative to health care access, the resulting prevalence estimates were unrealistic. We decided to continue using the DisMod EMR estimations while keeping HAQi as a country-level covariate to inform EMR with a mean and standard deviation produced from MR-BRT.

Bias Adjustments

In GBD 2019, we improved the bias adjustment methods by utilizing a MR-BRT model outside of DisMod to allow a more direct comparison between different case definitions and/or study designs.

We made a series of adjustments to data that don't completely match our case definition, doctor's diagnosis and wheezing in the past year. The estimation of Asthma in a population varies slightly by the case definition used (wheezing and diagnosis, only wheezing, etc). Similarly, claims data is subject to biases. An analysis for GBD 2017 showed that claims data were systemically lower than asthma survey data, probably reflecting selection bias with regard to socioeconomic status. Adjustments are made to these data to correct these biases.

The adjustment is a logit-transformation method in MR-BRT. The general process is described below:

1. Identify data points with overlapping year, age, sex, and location between reference and alternative definitions.
2. Logit transform overlapping data points of alternative and reference case definitions
3. Convert overlapping data points into a difference in logit space using the following equation: $logit(altnerative) - logit(reference)$
4. Use the delta method to compute standard errors of overlapping data points in logit space, then calculate standard error of logit difference using the following equation: $\sqrt{(variance\ of\ alternative) + (variance\ of\ reference)}$
5. Using MR-BRT, conduct a random effects meta-regression to obtain the pooled logit difference of alternative to reference
6. Apply the pooled logit difference to all data points of alternative case definitions using the following equation:
$$new_{estimate} = inverse.logit((logit(alternative)) - (pooled\ logit\ difference))$$
7. Calculate new standard errors using the delta method, accounting for gamma (between-study heterogeneity)

Data derived from claims from commercial health insurance in the United States were also adjusted using a factor estimated in MR-BRT. To account for this, we estimated a MarketScan 2000 coefficient and a separate MarketScan coefficient for the remaining years of Marketscan data, by comparing the national

values in these datasets to national asthma estimates from the USA National Health and Nutrition Examination Survey and National Health Interview Surveys.

The coefficients for bias adjustments are shown:

MR-BRT Crosswalk Adjustment Factors

Data input	Status	Gamma	Beta Coefficient, Logit (95% CI)	Adjustment factor*
Wheezing + Doctor's Diagnosis	Ref	0.26	---	---
Only Wheezing	Alt		1.09<br>(0.61, 1.59)	0.75<br>(.65, 0.83)
Only Diagnosis	Alt		0.99<br>(0.50, 1.48)	0.73<br>(0.62, 0.82)
Self-reported currently have asthma	Alt		.01<br>(-0.48, 0.56)	0.50<br>(0.38, 0.64)
Self-reported ever having asthma	Alt		0.66<br>(0.11, 1.20)	0.66<br>(0.53, 0.77)
Marketscan 2000	Alt	0.00	-1.35<br>(-1.37, -1.33)	0.21<br>(0.20, 0.21)
Marketscan 2010 - 2016	Alt	0.60	-1.60<br>(-2.71, -0.43)	.17<br>(.06, .41)

***Adjustment factor is the inverse-logit transformed Beta coefficient; <0.5 represents that alternative is adjusted upward; >0.5 represents alternative is adjusted downward**

Modelling strategy

We use DisMod-MR 2.1 as the main modelling tool for asthma. Prior settings include a maximum remission of 0.3 (reflecting the upper bound of the highest observed data) and no incidence between the ages of 0 and 0.5 year, as a diagnosis cannot be made in young infants.

Predictive covariates

To assist estimation, particularly in locations with few or no data, we included covariates in our DisMod model that are associated with measures of asthma epidemiology in prior studies and for which estimates of those covariates are available for all GBD year-age-sex-location combinations. Specifically, we use log LDI and the asthma standardised exposure variable (SEV), a scalar that combines exposure of all GBD risks that influence asthma.

We also used HAQi covariate with priors from tests on excess mortality rate in MR-BRT.

Covariate Table	Measure	Beta	Exponentiated
Healthcare Access and Quality Index	EMR	-0.06<br>(-.062 to -.059)	.94<br>(.93 to .94)

Log SEV scalar: asthma	prevalence	0.75 (0.75–0.76)	2.13 (2.12–2.14)
Log LDI (I$ per capita)	excess mortality rate	-0.5 (-0.5 to -0.5)	0.61 (0.61–0.61)

Severity split inputs

Lay descriptions and disability weights for the asthma health states are shown in the table below. The distribution between the three health states is derived from an analysis of the USA Medical Expenditure Panel Surveys (MEPS). The methods are described in full in a separate section of this appendix. Briefly, MEPS is an ongoing survey of health service encounters with as its main objective to collect data on health expenditure. Panels are recruited every year and followed up for a period of two years. Diagnostic information provided by respondents on the reasons for any health care contact are coded into three-digit ICD-9 codes by professional coders.

Twice over the two-year follow-up period, respondents are asked to fill in 12-Item Short Form Surveys (SF-12). From convenience samples asking respondents to fill in SF-12 for 60 of the GBD health states, IHME has created a mapping from SF-12 scores to GBD disability weights (DW). We perform a regression with indicator variables for all GBD causes that we can identify from the ICD codes in MEPS to derive for each individual with a diagnosis the amount of disability that can be attributed to that condition after controlling for any comorbid conditions. Anyone with a diagnosis of asthma in whom the disability assigned to asthma is negative or zero we assume is asymptomatic (at the time of asking SF-12 question relating to their health status in the past four weeks). Non-zero values we bin into the three health states assuming a split between these at the midpoint between DW values. The table below gives the proportions in MEPS in each of the health states and an asymptomatic state.

Severity level	Lay description	DW (95% CI)	Severity distribution
Asymptomatic			36.2% (35.0–37.3%)
Controlled	This person has wheezing and cough once a month, which does not cause difficulty with daily activities.	0.015 (0.007–0.026)	19.9% (13.6–27.8%)
Partially controlled	This person has wheezing and cough once a week, which causes some difficulty with daily activities.	0.036 (0.022–0.055)	20.6% (15.1–25.8%)
Uncontrolled	This person has wheezing, cough, and shortness of breath more than twice a week, which causes difficulty with daily activities and sometimes wakes the person at night.	0.133 (0.086–0.192)	23.3% (18.7–30.3%)

Interstitial lung disease and pulmonary sarcoidosis (ILD)

Flowchart

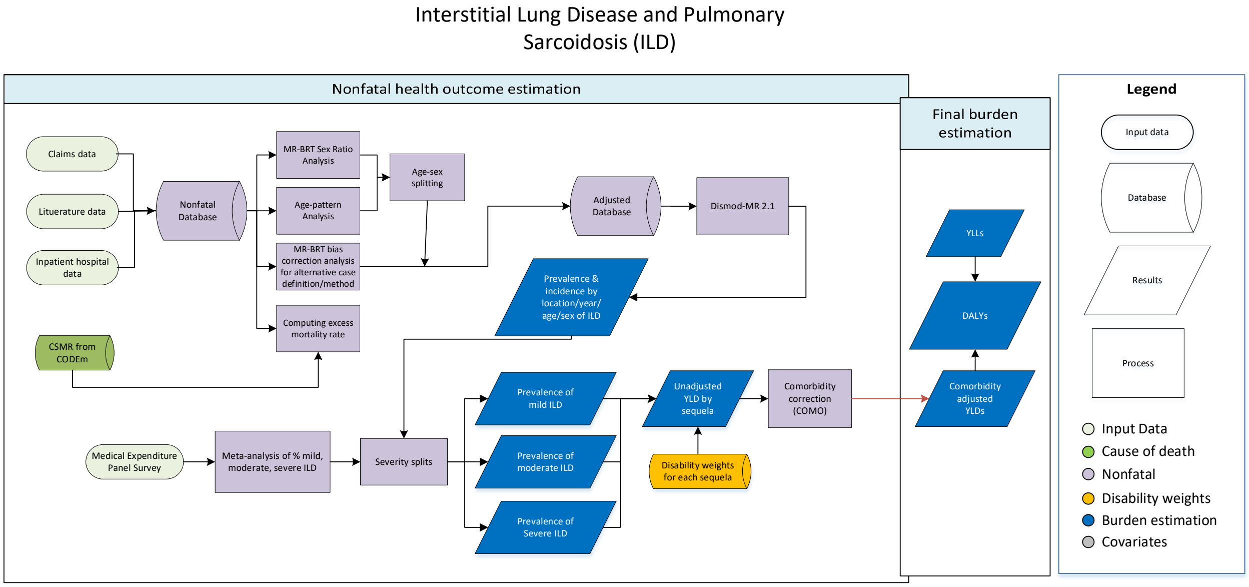

Case definition

Interstitial lung diseases and pulmonary sarcoidosis are a collection of chronic respiratory diseases that impair lung function and oxygen uptake through scarring and/or inflammation. The relevant ICD codes are D86 and J84. For interstitial lung disease, we use the American Thoracic Society as the gold standard definition.

Input data

Model Inputs

No systematic review of the literature was conducted for ILD for this iteration of the Global Burden of Disease. These reviews are done on a rotating basis and updates will be made for a future iteration.

Data used to make estimates of ILD are from three sources. The first is literature data from previous systematic reviews – usually from smaller-scale studies of prevalence or incidence. The second data type is claims data for the United States. The source and preparation of these data is described elsewhere. The third data type is adjusted hospital inpatient records. Because these records only report primary diagnosis, a priori adjustments are made based on location and healthcare access and quality.

Data inputs for interstitial lung disease and pulmonary sarcoidosis

Measure	Total sources	Countries with data
All measures	342	51
Prevalence	306	45
Incidence	27	16
With-condition mortality rate	2	2
Proportion	15	1

Data Processing

Age-Sex and Sex Split

In some cases, data are reported by only age or only sex, but not both. For example, a study may have included the proportion of males and females with ILD and then separately reported the proportion of both sexes in smaller age bins (e.g. age 40-45, 45-50, etc.) that have ILD. In these cases, we perform an age-sex split by utilizing proportions within the study to disaggregate the data.

When no information by sex in a study is present, we instead perform a sex-split on the data by applying separate sex proportions. The sex split analysis was carried out using MR-BRT. When data are aggregated into age categories larger than 25 years, we split the data into smaller age bins based on the global age pattern from an initial DisMod model.

Bias Adjustments

In GBD 2019, we improved the bias adjustment methods by utilizing a MR-BRT model outside of DisMod to allow a more direct comparison between different case definitions and/or study designs.

We made a series of adjustments to data that don't completely match our case definition. Data that only reports IPF or only sarcoidosis tend to vary estimates of ILD in a population. Similarly, claims tends to differ from the population, probably representing selection bias due to socioeconomic status. We make adjustments to these data to reflect these possible variations. The adjustment is a logit-transformation method in MR-BRT. The general process is described below:

1. Identify data points with overlapping year, age, sex, and location between reference and alternative definitions.
2. Logit transform overlapping data points of alternative and reference case definitions
3. Convert overlapping data points into a difference in logit space using the following equation: $logit(altnerative) - logit(reference)$
4. Use the delta method to compute standard errors of overlapping data points in logit space, then calculate standard error of logit difference using the following equation: $\sqrt{(variance\ of\ alternative) + (variance\ of\ reference)}$
5. Using MR-BRT, conduct a random effects meta-regression to obtain the pooled logit difference of alternative to reference
6. Apply the pooled logit difference to all data points of alternative case definitions using the following equation:
$$new_{estimate} = inverse.logit((logit(alternative)) - (pooled\ logit\ difference))$$
7. Calculate new standard errors using the delta method, accounting for gamma (between-study heterogeneity)

MR-BRT Crosswalk Adjustment Factors

Data input	Status	Gamma	Beta Coefficient, Logit (95% CI)	Adjustment factor*
IPF and Sarcoidosis	Ref	0.23	---	---
Only IPF	Alt		-1.46 (-2.09 - -0.79)	0.19 (0.11 - 0.31)
Only Sarcoidosis	Alt		-1.07 (-1.71 - -0.40)	0.26 (0.15—0.40)
Marketscan 2000	Alt	0	-0.31 (-0.32 - -0.29)	0.42 (0.42 - 0.43)

**Adjustment factor is the inverse-logit transformed Beta coefficient; <0.5 represents that alternative is adjusted upward; >0.5 represents alternative is adjusted downward*

Modelling strategy

Estimates for ILD are produced using a standard DisMod-MR 2.1 approach. We use prior settings of zero remission and we constrain the super-region random effects to -0.5 to 0.5 to ensure model stability.

We employed predictive covariates to improve estimation in locations with scarce prevalence data. These were income per capita and the healthcare access and quality index (HAQi). The priors on HAQi were model outputs from the MRBRT modelling on EMR as described in the next section.

Variable name	Measure	Beta	Exponentiated
LDI (I$ per capita)	excess mortality rate	-0.2 (-0.2 to -0.2)	0.82 (0.82–0.82)
Healthcare Access and Quality index	excess mortality rate	-0.014 (-0.014 — -0.014)	0.99 (0.99 — 0.99)

Predicted excess mortality rate with MR-BRT

Similar to other causes, we include estimates of cause-specific mortality rate (CSMR) and Excess Mortality Rate (EMR) as model inputs. In previous rounds, priors on excess mortality rate (EMR) were estimated in DisMod by matching prevalence data points with their corresponding CSMR values within the same age, sex, year, location (by dividing CSMR by prevalence). For short duration conditions (remission>1), the corresponding prevalence was derived by running an initial model and then applying the same CSMR/prevalence method.

However, for many causes, DisMod estimated a rather unrealistic pattern of EMR compared to an expected pattern of decreasing EMR with greater access to quality health care. Such unexpected patterns often signal inconsistencies between CSMR estimates and the measures of prevalence and/or incidence.

To provide greater guidance to DisMod on the expected pattern of EMR, EMR data generated in the previous round were modeled using the MR-BRT approach by age and sex with a prior on healthcare access and quality index (HAQi) having a negative coefficient. Results from MR-BRT were then predicted for each location year, sex and for ages 0, 10, 20 ….100. We included HAQi as a country-level covariate to inform EMR with a mean and standard deviation produced from MR-BRT.

Severity Splits

Data to inform estimates of the severity gradient due to ILD are derived from previously analyses of the Medical Expenditure Panel Survey (MEPS). The table below illustrates the lay descriptions and disability weights associated with different levels of severity of interstitial lung disease.

Severity level	Lay description	DW (95% CI)
Mild	Has cough and shortness of breath after heavy physical activity, but is able to walk long distances and climb stairs.	0.019 (0.011–0.033)
Moderate	Has cough, wheezing, and shortness of breath, even after light physical activity. The person feels tired and can walk only short distances or climb only a few stairs.	0.225 (0.153–0.312)
Severe	Has cough, wheezing, and shortness of breath all the time. The person has great difficulty walking even short distances or climbing any stairs, feels tired when at rest, and is anxious.	0.408 (0.273–0.556)

Other chronic respiratory diseases

In addition to the chronic respiratory diseases described above, there are other types of chronic respiratory diseases with a range of severities and associated sequelae. Because these chronic respiratory diseases are diverse in their underlying causes and risk factors as well as in their associated health outcomes, modelling them together in a DisMod-MR model would not produce reliable estimates of prevalence. Instead, we calculated the YLDs caused by other chronic respiratory diseases directly using a YLD/YLL ratio as a 'place holder'.

We calculated the ratio of YLDs to YLLs across the specified chronic respiratory diseases for which non-fatal outcomes were modelled, using YLL estimates from the GBD 2019 cause of death (CoD) analysis. We then multiplied this YLD/YLL ratio by the YLL estimates for other chronic respiratory diseases.

Risk-specific modeling descriptions

Non-optimal temperature

Flowchart

Non-optimal temperature exposure

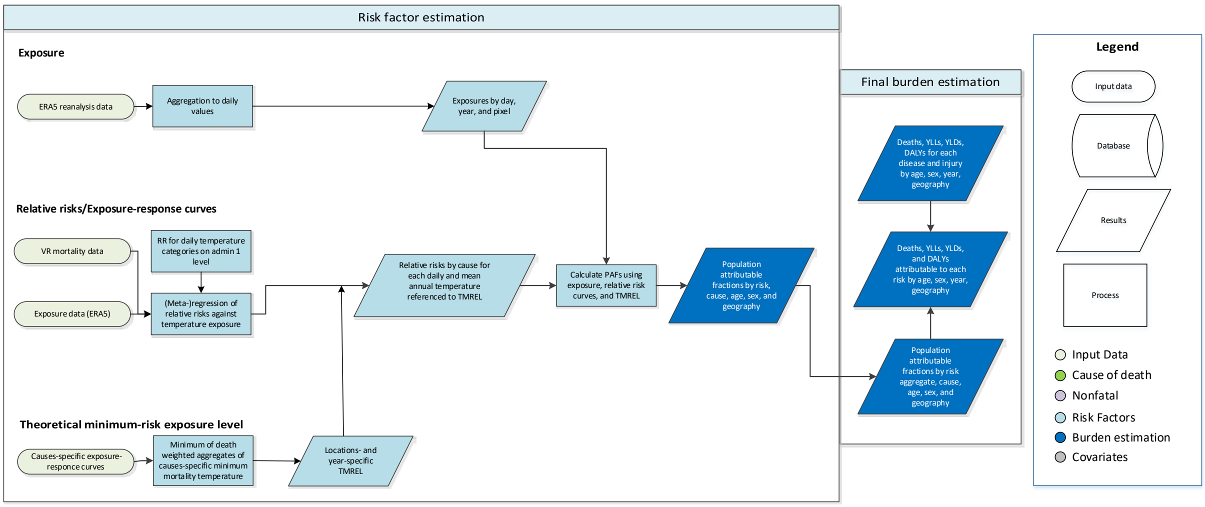

Input data and modelling strategy

Case definition

The exposure of non-optimal temperature is defined as the same day exposure to ambient temperature that is either warmer or colder than the temperature associated with the minimum mortality risk. Specifically, we define the theoretical minimum risk exposure level (TMREL) for temperature as the temperature that is associated with the lowest overall mortality attributable to the risk, in a given location and year. Given varying exposure-response curves for different mean annual temperature zones, as well as spatially and temporally varying cause compositions, we estimate TMRELs by year and location and are not using a globally uniform TMREL. High temperature (heat) exposure is defined as exposure to temperatures warmer than this TMREL and low temperature (cold) is defined as temperatures colder than this TMREL.

Exposure

ERA5 data

We derived exposure estimates from the ERA5 reanalysis dataset from the European Centre for Medium-Range Weather Forecasts (ECMWF). ECMWF produced ERA5 estimates using their Integrated Forecast System (IFS). Hourly values of surface temperature are available for a spatial resolution of 0.25°x0.25°. Uncertainty estimates for these temperature values, ie, the ensemble spread (standard deviation) is available for every 3 hours (00:00, 03:00, 06:00, 09:00, 12:00, 15:00, 18:00, 21:00) for a spatial resolution of 0.5°x0.5°. At the time of analysis, data were available from 1979 to June 2019.[1,2] We calculated daily averages of temperature and spread for each pixel and then assigned an uncertainty value to each daily temperature value. Based on the spread we derived 1,000 draws of each daily temperature pixel.

Population data

Population data for calculating population-weighted location means were derived from WorldPop, which is an open source project initiated in 2013[3]. Multi-temporal, globally consistent, high-resolution human population data at 1 km x 1 km resolution can be downloaded from http://www.worldpop.org.uk/ for 2000, 2005, 2010, 2015, and 2020. For the purpose of our work, we interpolated in-between the 5-year estimation bins to obtain annual data. Further, we extrapolated until 1990 by using the 2000-2005 growth rate for back-casting.

Table 1: Data inputs for exposure for non-optimal temperature.

Input data	Exposure
Source count (total)	203
Number of countries and territories with data	204

Exposure-response modelling

Mortality data

Deaths at the individual-level that included information regarding the cause (ie, ICD code), date, and the location at the second administrative level (admin2) or finer were collected from the GBD cause-of-death (CoD) database for vital registration data sources. We adapted the GBD standard procedure for garbage code redistribution to redistribute daily mortality data rather than annual data and mapped ICD causes to GBD causes for level 3. In total, we analysed 58.9 million deaths from eight different countries and 15,197 administrative units. For Brazil, the data covers a period from 1999 to 2016 for 5,570 municipalities and 19.9 million deaths. For Chile, the data covers the period from 1990 to 1996 and 2009 to 2011 for 15 regions and 2.46 million deaths. For Colombia, the data covers a period from 2001 to 2005 for 1,125 municipalities and 0.95 million deaths. For Guatemala, the data covers a period from 2009 to 2016 for 333 municipalities and 0.49 million deaths. For Mexico, the data covers a period from 1996 to 2015 for 2,438 municipalities and 9.88 million deaths. For New Zealand, the data covers a period from 1988 to 2014 for 20 district health boards and 0.76 million deaths. For the United States, the data covers a period from 1980 to 1988 for 3,124 municipalities and 18.3 million deaths. For China, the data covers the year 2016 for 2,556 counties and 6.1 million deaths.

Exposure-response modelling (MR-BRT)

To estimate cause-specific mortality, based on average daily temperature and temperature zone (defined by mean annual temperature), we used a robust meta-regression framework, implemented through the MR-BRT (Bayesian, regularised, trimmed) tool. The tool allows three features that are essential to the analysis:[5]

- A meta-analytic framework that can handle heterogeneous data sources
- A robust approach to outlier detection and removal (trimming)
- Specification of the functional dependence of outcome vs. average daily temperature and temperature zone as a 2-dimensional surface through a spline interface.

The use of trimming in a vast array of inference and machine learning problems is standard.[6,7,8] The use of high-dimensional splines has been proposed before,[9] but the methods used for estimation go beyond prior work, and we explain them below.

The functional relationship between any outcome y and input variables (t1, t2) models y as a linear combination of 2d spline basis elements. Each spline basis element is a product of individual basis elements for 1D splines for t1 and t2. Therefore, the inference problem looks for a combination of simple curvilinear 2D elements that fit the data while preserving smoothness across element boundaries. The MR-BRT tool also allows prior information to influence the shape of the spline, particularly in areas with sparse data.

For the purpose of modelling the relationship between mortality and mean annual and daily temperature we imposed monotonicity in the direction of daily temperature. For all J-shaped curves that depicted an increase in mortality above and below a threshold, we forced the curve to monotonically decrease at the lower end of the temperature distribution and to monotonically increase at the upper end. For all external causes that displayed a monotonic increase over the entire temperature range, we imposed monotonicity only in the direction of warmer temperatures. We placed 2 knots of degree 3 in the direction of mean annual temperature when fitting the surface. In the direction of daily mean temperature, we placed 3 knots of degree 3 for J-shaped causes and 2 knots of degree 1 for external causes that monotonically increase over temperature range. Figure 1 shows an example of a relative risk (RR) surface along daily and annual mean temperature for drowning.

We estimated uncertainty using a two-step approach. First, we derived the uncertainty of the mean surface from the measurement error using the fit-retrofit error. Second, we added uncertainty from the random effects by sampling it separately from the cold and warm side.

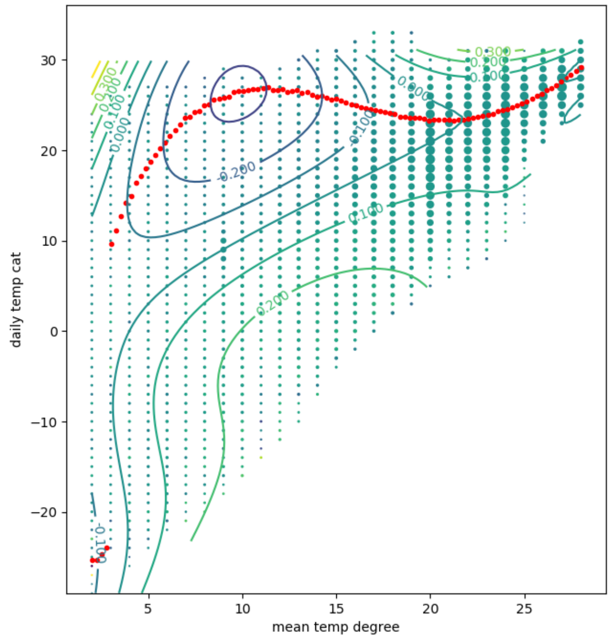

Figure 1: Log relative risk of death from lower respiratory infection along mean annual temperature (mean temp cat) and daily mean temperature (daily temp cat). The red dotted line depicts minimum mortality temperature along mean annual temperature zones. Green and blue lines depict isopleths, ie, lines of equal log RR of mortality

Table 2: Data inputs for relative risks for non-optimal temperature

Input data	Relative risk
Source count (total)	112
Number of countries with data	8

Cause selection

We excluded all causes with fewer than 100,000 deaths as well as causes of death that did not represent a particular entity but rather a summary category (eg, other cardiovascular diseases). Further, dementia and protein energy malnutrition were not considered in this analysis due to inconsistencies in data classification. The remaining causes were selected based on significance. For this, for each cause and each mean temperature zone we determined the widest range of consecutive daily temperatures with statistically significant relative risks, expressed as a percentage of the full range of daily temperatures in that mean temperature zone. Figure 2 gives an example of the temperature-mortality relationship for three selected slices (mean annual temperature of 6 °C, 17 °C and 21 °C). Significant areas along the exposure-response curves are marked in grey. We included all causes where at least 30% of zones had a consecutive significance range that spanned at least 5% of the full range of daily temperatures. Twelve causes met these criteria and were included as outcomes associated with non-optimal temperature: ischaemic heart disease, stroke, hypertensive heart disease, diabetes, chronic kidney disease, lower respiratory infection, chronic obstructive pulmonary disease, homicide, suicide, mechanical injuries, transport-related injuries, and drowning.

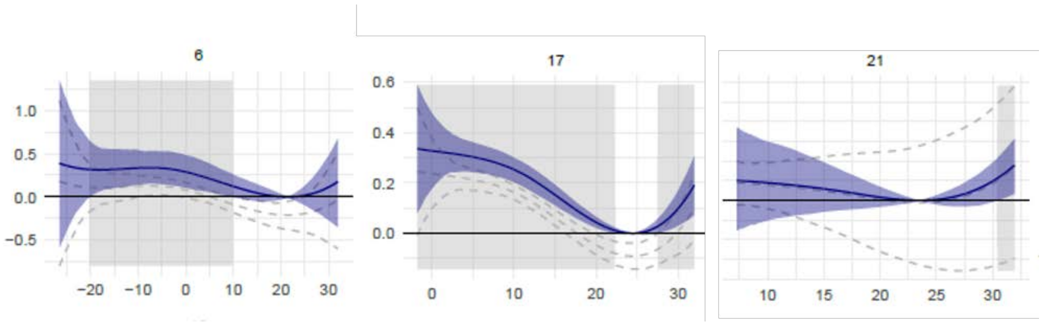

Figure 2: Selected exposure-response curves for the relationship between daily mean temperature and log RR of lower respiratory infection mortality for mean annual temperature categories of 6 °C, 17 °C and 21 °C. Temperatures where associations are significant are displayed in grey.

Theoretical minimum risk exposure level (TMREL)

For the purpose of this analysis, the TMREL was defined as the temperature associated with the lowest mortality for all included causes. We calculated a death-weighted average of the cause-specific exposure-response curves with the minimum of this average curve being the TMREL. This was done for each year and each of the 990 GBD locations using CoD estimates produced for the GBD 2019 study. As climate zones or mean annual temperature can vary within a location, we calculated the TMREL for every mean annual temperature, assuming a consistent cause composition within a location. This approach represents the first use of spatially and temporally varying TMRELs within the GBD study.

Population attributable fractions

The population attributable fraction (PAF) was calculated for each temperature pixel and each day of the year (ie, pixel-day). Subsequently, we population-weighted each pixel using the fraction of the population living in a given pixel relative to the GBD location. Depending on whether the daily mean temperature was below or above the TMREL, the effect was assigned to either low or high temperature. Daily population-weighted high and low temperature PAFs were then aggregated for the location and the year. Temperature effects can be either harmful or protective depending on whether the RR is above or below 1. For harmful temperature effects, ie, effects with a RR above 1, we used the following equation to derive PAFs: PAF=(RR-1)/RR; For temperature effects exhibiting a protective effect the equation was adapted by implementing the reverse RR: PAF=-((1/RR)-1)/(1/RR). The PAF associated with non-optimal temperature exposure is an aggregate of heat and cold effects in each location and year. We estimated the temperature attributable burden as the product of the total burden for that cause and the corresponding PAF for each GBD location, year, age group, and sex.

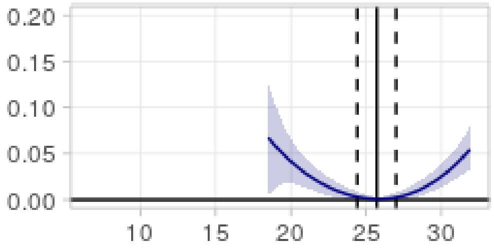

Figure 3: Schematic illustration of the exposure-response relationship between temperature and mortality and associated low temperature (cold) and high temperature effects beyond the theoretical minimum exposure level (TMREL). The blue line depicts the exposure-response curve with blue shaded line showing 95% uncertainty range. The black solid line depicts the TMREL with dashed black lines displaying 95% uncertainty range. Effects left of the TMREL are counted towards cold PAFs and right of the TMREL towards heat PAFS.

References

1 Hersbach H, Bell B, Berrisford P, et al. Global reanalysis : goodbye Global reanalysis : goodbye ERA-Interim , hello. 2019. DOI:10.21957/vf291hehd7.

2 Copernicus Climate Change Service (C3S) (2017): ERA5: Fifth generation of ECMWF atmospheric reanalyses of the global climate. Copernicus Climate Change Service Climate Data Store (CDS), September 2019. .

3 Geography and Environmental Science, University of Southampton. Age and Sex Structures, Global Per Country 2000-2020 - WorldPop. Southampton , United Kingdom: Geography and Environmental Science, University of Southampton, 2018. .

4 Roth GA, Abate D, Hassen Abate K, et al. Global, regional, and national age-sex-specific mortality for 282 causes of death in 195 countries and territories, 1980-2017: a systematic analysis for the Global Burden of Disease Study 2017 GBD 2017 Causes of Death Collaborators*. 2018 DOI:10.1016/S0140-6736(18)32203-7.

5 Zheng P, Aravkin AY, Barber R, Sorensen RJD, Murray CJL. Trimmed Constrained Mixed Effects Models: Formulations and Algorithms. 2019; published online Sept 23. http://arxiv.org/abs/1909.10700 (accessed Nov 13, 2019).

6 Rousseeuw PJ. Least Median of Squares Regression. J Am Stat Assoc 1984; 79: 871–80.

7 Aravkin A, Davis D. Trimmed Statistical Estimation via Variance Reduction. Math Oper Res 2019; : moor.2019.0992.

8 Yang E, Lozano AC, Aravkin A. A general family of trimmed estimators for robust high-dimensional data analysis. Electron J Stat 2018; 12: 3519–53.

9 Pya N, Wood SN. Shape constrained additive models. Stat Comput 2015; 25: 543–59.

High body-mass index

Flowchart

Adult (Ages 20+) High Body-Mass Index: Data and Model Flow Chart

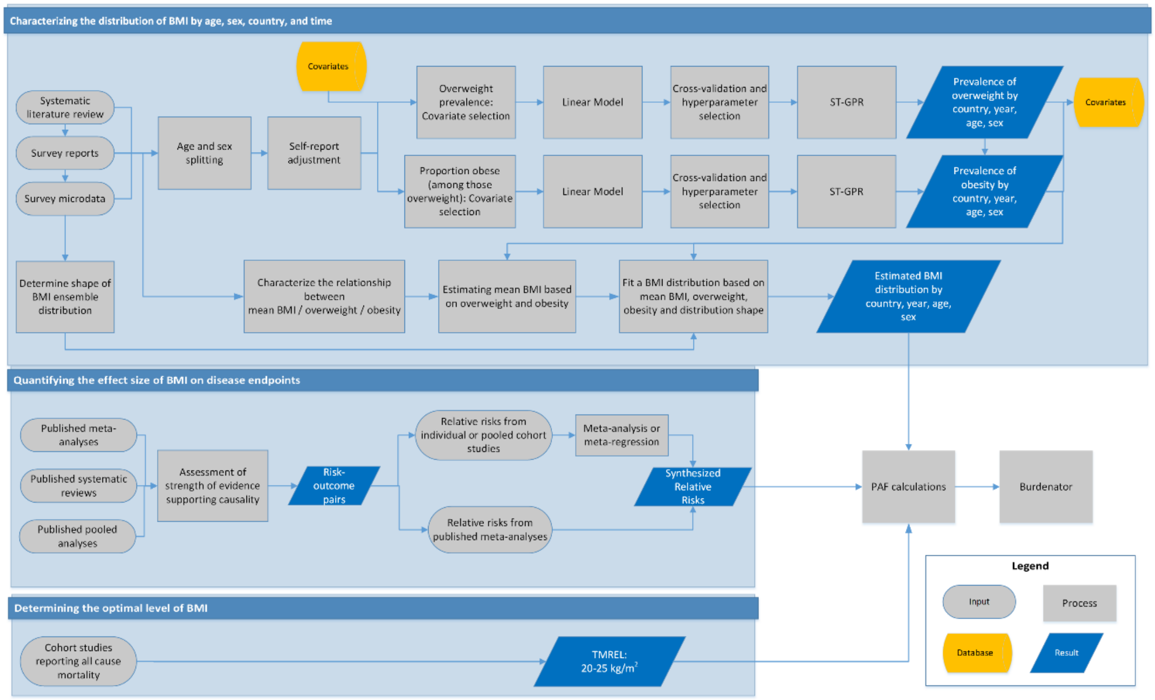

Childhood (Ages 2-19) High Body-Mass Index: Data and Model Flow Chart

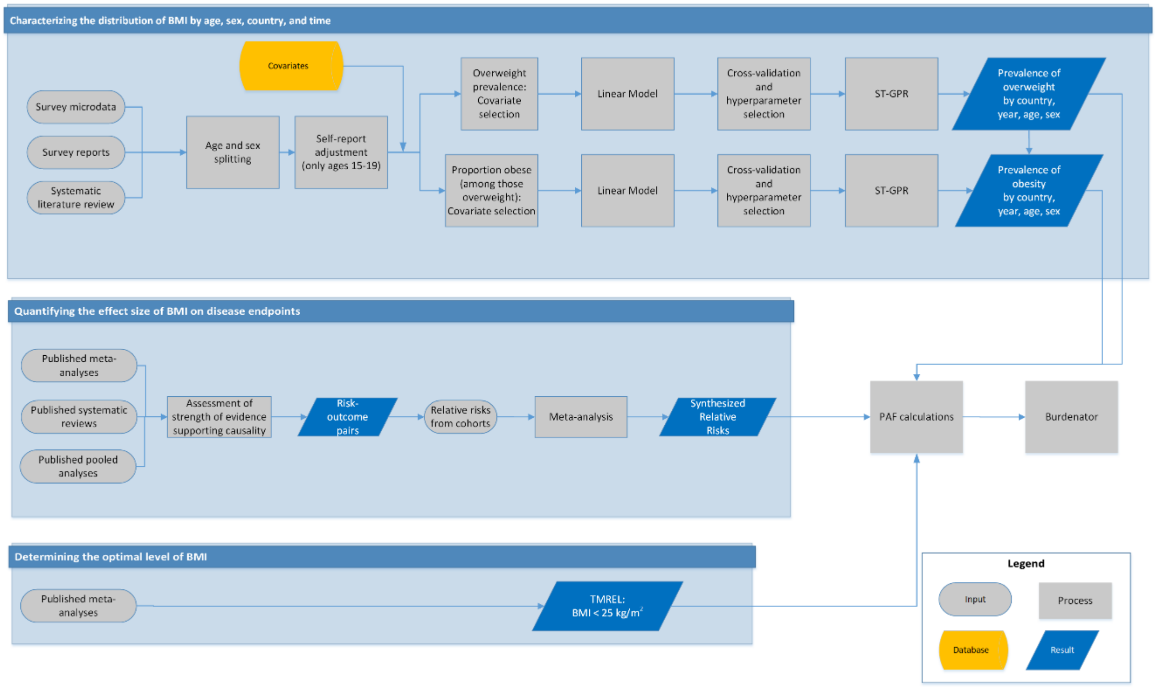

Input data and methodological summary

Case definitions

High body-mass index (BMI) for adults (ages 20+) is defined as BMI greater than 20 to 25 kg/m^2. High BMI for children (ages 1–19) is defined as being overweight or obese based on International Obesity Task Force standards.

Data sources

In GBD 2019, new data were added from sources included in the annual GHDx update of known survey series. We conducted a systematic review in GBD 2017 to identify studies providing nationally or subnationally representative estimates of overweight prevalence, obesity prevalence, or mean body-mass index (BMI). We limited the search to literature published between January 1, 2016, and December 31, 2016, to update the systematic literature search previously performed as part of GBD 2015.

The search for adults was conducted on 4 January 2017, using the following terms:

((("Body Mass Index"[Mesh] OR "Overweight"[Mesh] OR "Obesity"[Mesh]) AND ("Geographic Locations"[Mesh] NOT “United States”[Mesh]) AND ("humans"[Mesh] AND "adult"[MeSH]) AND ("Data Collection"[Mesh] OR "Health Services Research"[Mesh] OR "Population Surveillance"[Mesh] OR "Vital statistics"[Mesh] OR "Population"[Mesh] OR "Epidemiology"[Mesh] OR "surve*"[TiAb]) NOT (Comment[ptyp] OR Case Reports[ptyp] OR "hospital"[TiAb])) AND ("2016/01/01"[Date - Publication] : "2016/12/31"[Date - Publication]))

The search for children was conducted on 4 August 2016, using the following terms:
((("Body Mass Index"[Mesh] OR "Overweight"[Mesh] OR "Obesity"[Mesh]) AND ("Geographic Locations"[Mesh] NOT “United States”[Mesh]) AND ("humans"[Mesh] AND "child"[MeSH]) AND ("Data Collection"[Mesh] OR "Health Services Research"[Mesh] OR "Population Surveillance"[Mesh] OR "Vital statistics"[Mesh] OR "Population"[Mesh] OR "Epidemiology"[Mesh] OR "surve*"[TiAb]) NOT (Comment[ptyp] OR Case Reports[ptyp] OR "hospital"[TiAb])) AND ("2016/01/01"[Date - Publication] : "2016/12/31"[Date - Publication]))

Table 1: Data inputs for exposure for high body-mass index.

Input data	Exposure
Source count (total)	2022
Number of countries with data	190

Table 2: Data inputs for relative risks for high body-mass index.

Input data	Relative risk
Source count (total)	267
Number of countries with data	32

Eligibility criteria

We included representative studies providing data on mean BMI or prevalence of overweight or obesity among adults or children. For adults, studies were included if they defined overweight as BMI≥25 kg/m^2 and obesity as BMI≥30 kg/m^2, or if estimates using those cutoffs could be back-calculated from reported categories. For children (children ages 2–19), studies were included if they used International Obesity Task Force (IOTF) standards to define overweight and obesity thresholds. We only included studies reporting data collected after January 1, 1980. Studies were excluded if they used non-random samples (eg, case-control studies or convenience samples), conducted among specific subpopulations (eg, pregnant women, racial or ethnic minorities, immigrants, or individuals with specific diseases), used alternative methods to assess adiposity (eg, waist-circumference, skin-fold thickness, or hydrodensitometry), had sample sizes of less than 20 per age-sex group, or provided inadequate information on any of the inclusion criteria. We also excluded review articles and non-English-language articles.

Data collection process

Where individual-level survey data were available, we computed mean BMI using weight and height. We then used BMI to determine the prevalence of overweight and obesity. For individuals aged over 19 years, we considered them to be overweight if their BMI was greater than or equal to 25 kg/m^2, and obese if their BMI was greater than or equal to 30 kg/m^2. For individuals aged 2 to 19 years, we used monthly IOTF cutoffs[2] to determine overweight and obese status when age in months was available. When only age in years was available, we used the cutoff for the midpoint of that year. Obese individuals were also considered to be overweight. We excluded studies using the World Health Organization (WHO) standards or country-specific cutoffs to define childhood overweight and obesity. At the individual level, we considered BMI<10 kg/m^2 and BMI>70 kg/m^2 to be biologically implausible and excluded those observations.

The rationale for choosing to use the IOTF cutoffs over the WHO standards has been described elsewhere.[1] Briefly, the IOTF cutoffs provide consistent child-specific standards for ages 2–18 derived from surveys covering multiple countries. By contrast, the WHO growth standards apply to children under age 5, and the WHO growth reference applies to children ages 5–19. The WHO growth reference for children ages 5–19 was derived from United States data, which are less representative than the multinational data used by IOTF. Additionally, the switch between references at age 5 can produce artificial discontinuities. Given that we estimate global childhood overweight and obesity for ages 2–19 (with ages 19 using standard adult cutoffs), the IOTF cutoffs were preferable. Additionally, we found that IOTF cutoffs were more commonly used in scientific literature covering childhood obesity.

From report and literature data, we extracted data on mean BMI, prevalence of overweight, and prevalence of obesity, measures of uncertainty for each, and sample size, by the most granular age and sex groups available. Additionally, we extracted the same study-level covariates as were extracted from microdata (measurement, urbanicity, and representativeness), as well as location and year.

In addition to the primary indicators described above, we extracted relevant survey-design variables, including primary sampling unit, strata, and survey weights, which were used to tabulate individual-level microdata and produce accurate measures of uncertainty. We extracted three study-level covariates: 1) whether height and weight data were measured or self-reported; 2) whether the study was predominantly conducted in an urban area, rural area, or both; and 3) the level of representativeness of the study (national or subnational).

Finally, we extracted relevant demographic indicators, including location, year, age, and sex. We estimated the standard error of the mean from individual-level data, where available, and used the reported standard error of the mean for published data. When multiple data sources were available for the same country, we included all of them in our analysis. If data from the same data source were available in multiple formats such as individual-level data and tabulated data, we used individual-level data.

Modelling strategy

Age and sex splitting

Any report or literature data provided in age groups wider than the standard five-year age groups or as both sexes combined were split using the approach used by Ng and colleagues.[2] Briefly, age-sex patterns were identified using sources with data on multiple age-sex groups and these patterns were applied to split aggregated report and literature data. Uncertainty in the age-sex split was propagated by multiplying the standard error of the data by the square root of the number of splits performed. We did not propagate the uncertainty in the age pattern and sex pattern used to split the data as they seemed to have small effect.

Self-report bias adjustment

We included both measured and self-reported data. We tested for bias in self-report data compared to measured data, which is considered to be the gold-standard. There was no clear direction of bias for children ages 2–14, so for these age groups we only included measured data. For individuals ages 15 and above, we adjusted self-reported data for overweight prevalence and obesity prevalence. In GBD 2017, the self-report bias adjustment used a nested hierarchical mixed-effects regression model. This approach was updated in GBD 2019 to utilise the power of MR-BRT. For both overweight and obesity, we fit sex-specific MR-BRT models on the logit difference between measured and self-reported with a fixed effect on super-region. The bias coefficients derived from these two models are in Table 1 and 2.

Table 1: MR-BRT self-report crosswalk adjustment factors for overweight prevalence

Model	Data input	Reference or alternative case definition	Gamma	Beta coefficient, logit (95% CI)
Females	Measured data	Ref	0.26	---
	Self-reported data (southeast Asia, east Asia, and Oceania)	Alt		-0.53 (-1.03, -0.04)
	Self-reported data (central Europe, eastern Europe, and central Asia)	Alt		-0.20 (-0.69, 0.30)
	Self-reported data (high-income)	Alt		-0.25 (-0.75, 0.24)
	Self-reported data (Latin America and Caribbean)	Alt		-0.19 (-0.69, 0.31)
	Self-report data (north Africa and Middle East)	Alt		-0.38 (-0.89,0.11)
	Self-report data (south Asia)	Alt		0.36 (-0.14, 0.85)
	Self-report data (sub-Saharan Africa)	Alt		-0.26 (-0.76, 0.24)
Males	Measured data	Ref	0.43	---

	Self-reported data (southeast Asia, east Asia, and Oceania)	Alt		-0.36 (-1.17, 0.50)
	Self-reported data (central Europe, eastern Europe, and central Asia)	Alt		-0.03 (-0.84, 0.82)
	Self-reported data (high-income)	Alt		0.05 (-0.77, 0.87)
	Self-reported data (Latin America and Caribbean)	Alt		-0.02 (-0.84, 0.81)
	Self-report data (north Africa and Middle East)	Alt		-0.21 (-1.04, 0.61)
	Self-report data (south Asia)	Alt		0.53 (-0.28, 1.37)
	Self-report data (sub-Saharan Africa)	Alt		-0.27 (-1.09, 0.55)

Table 2: MR-BRT self-report crosswalk adjustment factors for obesity prevalence

Model	Data input	Reference or alternative case definition	Gamma	Beta coefficient, logit (95% CI)
Females	Measured data	Ref	0.38	---
	Self-reported data (southeast Asia, east Asia, and Oceania)	Alt		-0.11 (-0.86, 0.64)
	Self-reported data (central Europe, eastern Europe, and central Asia)	Alt		-0.95 (-1.70, -0.19)
	Self-reported data (high-income)	Alt		-0.42 (-1.16, 0.34)
	Self-reported data (Latin America and Caribbean)	Alt		-0.41 (-1.16, 0.34)
	Self-report data (north Africa and Middle East)	Alt		-0.48 (-1.23, 0.27)
	Self-report data (south Asia)	Alt		0.50 (-0.25, 1.26)
	Self-report data (sub-Saharan Africa)	Alt		-0.41 (-1.16, 0.34)
Males	Measured data	Ref	0.74	
	Self-reported data (southeast Asia, east Asia, and Oceania)	Alt		0.04 (-1.41, 1.53)
	Self-reported data (central Europe, eastern Europe, and central Asia)	Alt		-0.79 (-2.25, 0.71)
	Self-reported data (high-income)	Alt		-0.13 (-1.58, 1.40)
	Self-reported data (Latin America and Caribbean)	Alt		-0.26 (-1.70, 1.21)
	Self-report data (north Africa and Middle East)	Alt		-0.33 (-1.77, 1.16)
	Self-report data (south Asia)	Alt		0.66 (-0.78, 2.15)
	Self-report data (sub-Saharan Africa)	Alt		-0.41 (-1.86, 1.08)

Prevalence estimation for overweight and obesity

After adjusting for self-report bias and splitting aggregated data into five-year age-sex groups, we used spatiotemporal Gaussian process regression (ST-GPR) to estimate the prevalence of overweight and obesity. This modelling approach has been described in detail elsewhere.

The linear model, which when added to the smoothed residuals forms the mean prior for GPR is as follows:

$$\mathrm{logit}(\mathrm{overweight})_{c,a,t} = \beta_0 + \beta_1 \mathrm{energy}_{c,t} + \beta_2 \mathrm{SDI}_{c,t} + \beta_3 \mathrm{vehicles}_{c,t} + \beta_4 \mathrm{agriculture}_{c,t} + \sum_{k=5}^{21} \beta_k \mathrm{I}_{A[a]} + \alpha_s + \alpha_r + \alpha_c$$

$$\mathrm{logit}(\mathrm{obesity/overweight})_{c,a,t} = \beta_0 + \beta_1 \mathrm{energy}_{c,t} + \beta_2 \mathrm{SDI}_{c,t} + \beta_3 \mathrm{vehicles}_{c,t} + \sum_{k=4}^{21} \beta_k \mathrm{I}_{A[a]} + \alpha_s + \alpha_r + \alpha_c$$

where energy is ten-year lag-distributed energy consumption per capita, SDI is a composite index of development including lag-distributed income per capita, education, and fertility, $\mathrm{vehicles}$ is is the number of two- or four-wheel vehicles per capita, and $\mathrm{agriculture}$ is the proportion of the population working in agriculture. $\mathrm{I}_{A[a]}$ is a dummy variable indicating specific age group A that the prevalence point captures, and α_s, α_r, and α_c are super-region, region, and country random intercepts, respectively. Random effects were used in model fitting but were not used in prediction.

We tested all combinations of the following covariates to see which performed best in terms of in-sample AIC for the overweight linear model and the obesity as a proportion of overweight linear model: ten-year lag-distributed energy per capita, proportion of the population living in urban areas, SDI, lag-distributed income per capita, educational attainment (years) per capita, proportion of the population working in agriculture, grams of sugar adjusted for energy per capita, grams of sugar not adjusted for energy per capita, and the number of two- or four-wheeled vehicles per capita. We selected these candidate covariates based on theory as well as reviewing covariates used in other publications. The final linear model was selected based on 1) if the direction of covariates matched what is expected from theory, 2) all the included covariates were significant, and 3) minimising in-sample AIC. The covariate selection process was performed using the dredge package in R.

Estimating mean BMI

To estimate the mean BMI for adults in each country, age, sex, and time period 1980–2019, we first used the following nested hierarchical mixed-effects model, fit using restricted maximum likelihood on data from sources containing estimates of all three indicators (prevalence of overweight, prevalence of obesity, and mean BMI), in order to characterise the relationship between overweight, obesity, and mean BMI:

$$\log(\mathrm{BMI}_{c,a,s,t}) = \beta_0 + \beta_1 \mathrm{ow}_{c,a,s,t} + \beta_2 \mathrm{ob}_{c,a,s,t} + \beta_3 \mathrm{sex} + \sum_{k=4}^{20} \beta_k \mathrm{I}_{A[a]} + \alpha_s(1 + \mathrm{ow}_{c,a,s,t} + \mathrm{ob}_{c,a,s,t}) + \alpha_r(1 + \mathrm{ow}_{c,a,s,t} + \mathrm{ob}_{c,a,s,t}) + \alpha_c(1 + \mathrm{ow}_{c,a,s,t} + \mathrm{ob}_{c,a,s,t}) + \epsilon_{c,a,s,t}$$

where $\mathrm{ow}_{c,a,s,t}$ is the prevalence of overweight in country c, age a, sex s, and year t, $\mathrm{ob}_{c,a,s,t}$ is the prevalence of obesity in country c, age a, sex s, and year t, sex is a fixed effect on sex, $I_{A[a]}$ is an indicator variable for age, and α_s, α_r, and α_c are random effects at the super-region, region, and country, respectively. The model was run in Stata 13.

We applied 1000 draws of the regression coefficients to the 1000 draws of overweight prevalence and obesity prevalence produced through ST-GPR to estimate 1000 draws of mean BMI for each country, year, age, and sex. This approach ensured that overweight prevalence, obesity prevalence, and mean BMI were correlated at the draw level and uncertainty was propagated.

Estimating BMI distribution

We used the ensemble distribution approach described in the manuscript. We fit ensemble weights by source and sex, with source- and sex-specific weights averaged across all sources included to produce the final global weights. The ensemble weights were fit on measured microdata. The final ensemble weights were exponential = 0.002, gamma = 0.028, inverse gamma = 0.085, log-logistic = 0.187, Gumbel = 0.220, Weibull = 0.011, log-normal = 0.058, normal = 0.012, beta = 0.136, mirror gamma = 0.008, and mirror Gumbel = 0.113.

One thousand draws of BMI distributions for each location, year, age group, and sex estimated were produced by fitting an ensemble distribution using 1000 draws of estimated mean BMI, 1000 draws of estimated standard deviation, and the ensemble weights. Estimated standard deviation was produced by optimising a standard deviation to fit estimated overweight prevalence draws and estimated obesity prevalence draws.

Assessment of risk-outcome pairs

Risk-outcome pairs were defined based on strength of available evidence supporting a causal effect. We performed a systematic review of published meta-analyses, pooled analyses, and systematic reviews available through PubMed using the following search string: ("Body Mass Index"[Mesh] OR "Overweight"[Mesh] OR "Obesity"[Mesh]) AND (Meta-Analysis[ptyp] OR "systematic review"[tiab] OR "pooled analysis"[tiab]). Inclusion criteria are 1) the health outcome is included in GBD, 2) at least one prospective cohort is included, and 3) that the summary effect size is statistically significant. For outcomes meeting inclusion criteria we completed causal criteria tables to evaluate the strength of evidence supporting a causal relationship. Gallbladder disease, cataract, multiple myeloma, gout, non-Hodgkin lymphoma, asthma, Alzheimer’s disease, and atrial fibrillation were added as new outcomes in GBD 2016, resulting in a total of 38 outcomes.

Theoretical minimum risk exposure level

For adults (ages 20+), the theoretical minimum risk exposure level (TMREL) of BMI (20–25 kg/m^2) was determined based on the BMI level that was associated with the lowest risk of all-cause mortality in prospective cohort studies.[3]

For children (ages 2–19), the TMREL is “normal weight,” that is, not overweight or obese, based on IOTF cutoffs.

Relative risk

The relative risk per five-unit change in BMI for each disease endpoint was obtained from meta-analyses, and where available, pooled analyses of prospective observational studies. In cases where a relative risk per five-unit change in BMI was not available we computed our own dose-response meta-analysis using two-step generalised least squares for time trends estimation methods.

For childhood outcomes (ages 2–19), we computed categorical relative risks for overweight and obesity using a random effects meta-analysis.

References

1.) Cole, TJ, and T Lobstein. "Extended International (IOTF) Body Mass Index Cut-Offs for Thinness, Overweight and Obesity." Pediatric Obesity 2012; 7(4): 284–94.
2.) Ng M, Fleming T, Robinson M, et al. Global, regional, and national prevalence of overweight and obesity in children and adults during 1980–2013: a systematic analysis for the Global Burden of Disease Study 2013. The Lancet 2014; 384: 766–81.
3.) Angelantonio ED, Bhupathiraju SN, Wormser D, et al. Body-mass index and all-cause mortality: individual-participant-data meta-analysis of 239 prospective studies in four continents. The Lancet 2016; 388: 776–86.

Smoking

Flowchart

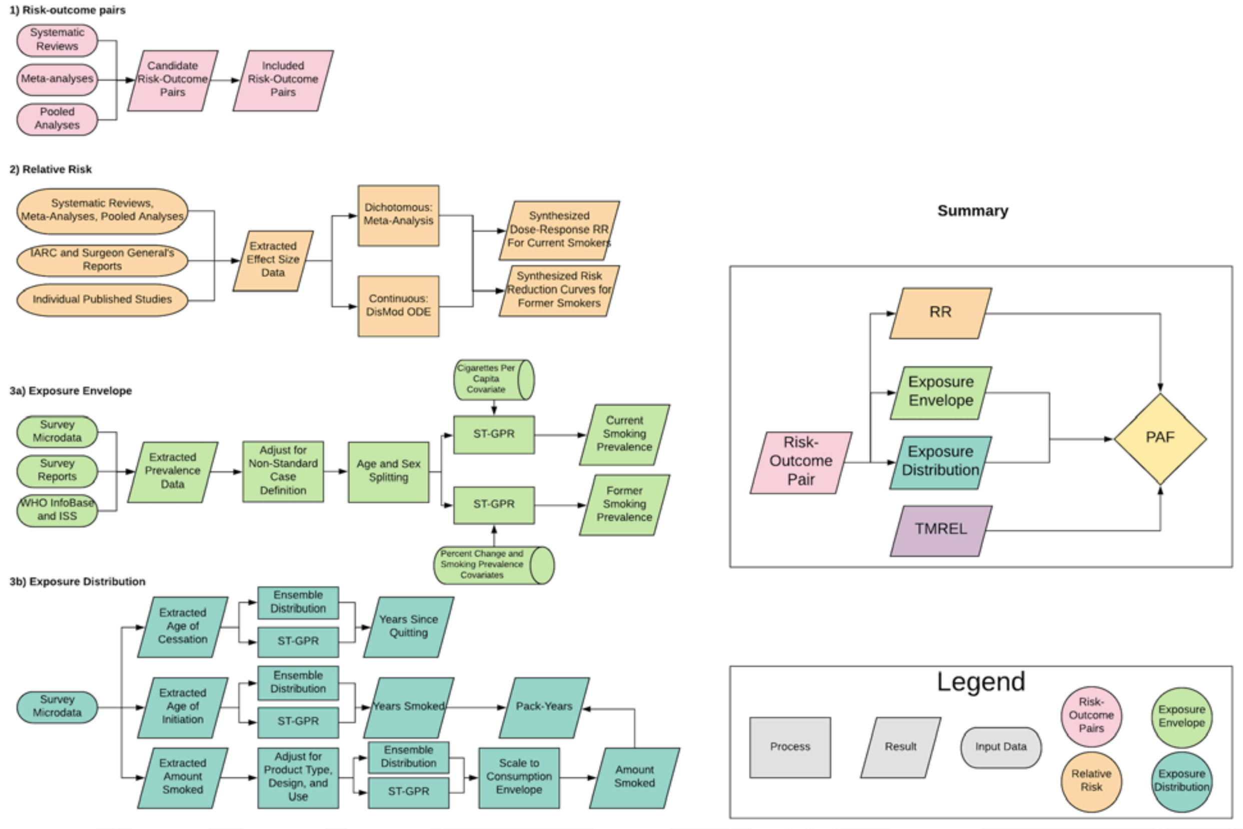

Input data and methodological summary

Definition

Exposure

As in GBD 2017, we estimated the prevalence of current smoking and the prevalence of former smoking using data from cross-sectional nationally representative household surveys. We defined current smokers as individuals who currently use any smoked tobacco product on a daily or occasional basis. We defined former smokers as individuals who quit using all smoked tobacco products for at least six months, where possible, or according to the definition used by the survey.

Input data

Our extraction method has not changed from GBD 2017. We extracted primary data from individual-level microdata and survey report tabulations. We extracted data on current, former, and/or ever smoked tobacco use reported as any combination of frequency of use (daily, occasional, and unspecified, which includes both daily and occasional smokers) and type of smoked tobacco used (all smoked tobacco, cigarettes, hookah, and other smoked tobacco products such as cigars or pipes),

resulting in 36 possible combinations. Other variants of tobacco products, for example hand-rolled cigarettes, were grouped into the four type categories listed above based on product similarities.

For microdata, we extracted relevant demographic information, including age, sex, location, and year, as well as survey metadata, including survey weights, primary sampling units, and strata. This information allowed us to tabulate individual-level data in the standard GBD five-year age-sex groups and produce accurate estimates of uncertainty. For survey report tabulations, we extracted data at the most granular age-sex group provided.

Table 1: Data inputs for exposure for smoking.

Input data	Exposure
Source count (total)	3439
Number of countries with data	201

Table 2: Data inputs for relative risks for smoking.

Input data	Relative risk
Source count (total)	673
Number of countries with data	16

Crosswalk

Our GBD smoking case definitions were current smoking of any tobacco product and former smoking of any tobacco product. All other data points were adjusted to be consistent with either of these definitions. Some sources contained information on more than one case definition and these sources were used to develop the adjustment coefficient to transform alternative case definitions to the GBD case definition. The adjustment coefficient was the beta value derived from a linear model with one predictor and no intercept. We used the same crosswalk adjustment coefficients as in GBD 2017, and thus we have not included a methods explanation in this appendix, as it has been detailed previously.

Age and sex splitting

As in GBD 2017, we split data reported in broader age groups than the GBD 5-year age groups or as both sexes combined by adapting the method reported in Ng et al[1] to split using a sex- geography- time-specific reference age pattern. We separated the data into two sets: a training dataset, with data already falling into GBD sex-specific 5-year age groups, and a split dataset, which reported data in aggregated age or sex groups. We then used spatiotemporal Gaussian process regression (ST-GPR) to estimate sex-geography-time-specific age patterns using data in the training dataset. The estimated age patterns were used to split each source in the split dataset.

The ST-GPR model used to estimate the age patterns for age-sex splitting used an age weight parameter value that minimises the effect of any age smoothing. This parameter choice allowed the estimated age pattern to be driven by data, rather than being enforced by any smoothing parameters of the model. Because these age-sex split data points were to be incorporated in the final ST-GPR exposure model, we

did not want to doubly enforce a modelled age pattern for a given sex-location-year on a given aggregate data point.

Modelling strategy

Smoking prevalence modelling

We used ST-GPR to model current and former smoking prevalence. The model is nearly identical to that in GBD 2017. Full details on the ST-GPR method are reported elsewhere in the appendix. Briefly, the mean function input to GPR is a complete time series of estimates generated from a mixed effects hierarchical linear model plus weighted residuals smoothed across time, space, and age. The linear model formula for current smoking, fit separately by sex using restricted maximum likelihood in R, is:

$$logit(p_{g,a,t}) = \beta_0 + \beta_1 CPC_{g,t} + \sum_{k=2}^{19} \beta_k I_{A[a]} + \alpha_s + \alpha_r + \alpha_g + \epsilon_{g,a,t}$$

Where $CPC_{g,t}$ is the tobacco consumption covariate by geography g and time t, described above, $I_{A[a]}$ is a dummy variable indicating specific age group A that the prevalence point $p_{g,a,t}$ captures, and α_s, α_r, and α_g are super-region, region, and geography random intercepts, respectively. Random effects were used in model fitting but not in prediction.

The linear model formula for former smoking is:

$$logit(p_{g,a,t}) = \beta_0 + \beta_1 PctChange_{A[a],g,t} + \beta_3 CSP_{A[a],g,t} + \sum_{k=3}^{20} \beta_k I_{A[a]} + \alpha_s + \alpha_r + \alpha_g + \epsilon_{g,a,t}$$

Where $PctChange_{A[a],g,t}$ is the percentage change in current smoking prevalence from the previous year, and $CSP_{A[a],g,t}$ is the current smoking prevalence by specific age group A, geography g, and time t that point $p_{g,a,t}$ captures, both derived from the current smoking ST-GPR model defined above.

Supply-side estimation

The methods for modelling supply-side-level data were changed substantially from those used in GBD 2017. The raw data were domestic supply (USDA Global Surveillance Database and UN FAO) and retail supply (Euromonitor) of tobacco. Domestic supply was calculated as production + imports - exports. The data went through three rounds of outliering. First, they were age-sex split using daily smoking prevalence to generate number of cigarettes per smoker per day for a given location-age-sex-year. If more than 12 points for a particular source-location-year (equal to over 1/3 of the split points) were above the given thresholds, that source-location-year was outliered. A point would not be outliered if it was (in cigarettes per smoker): under five (10–14 year olds); under 20 (males, 15–19 year olds); under 18 (females, 15–19 year olds); under 38/35 and over three (males/females, 20+ year olds). These thresholds were chosen by visualising histograms of the data for each age-sex, as well as with expert knowledge about reasonable consumption levels. In the second round of outliering, the mean tobacco per capita value over a 10-year window was calculated. If a point was over 70% of that mean value away

from the mean value, it was outliered. The 70% limit was chosen using histograms of these distances. Additionally, some manual outliering was performed to account for edge cases. Finally, data smoothing was performed by taking a three-year rolling mean over each location-year.

Next, a simple imputation to fill in missing years was performed for all series to remove compositional bias from our final estimates. Since the data from our main sources covered different time periods, by imputing a complete time series for each data series, we reduced the probability that compositional bias of the sources was leading to biased final estimates. To impute the missing years for each series, we modelled the log ratio of each pair of sources as a function of an intercept and nested random effects on super-region, region, and location. The appropriate predicted ratio was multiplied by each source that we did have, and then the predictions were averaged to get the final imputed value. For example, if source A was missing for a particular location-year, but sources B and C were present, then we predicted A twice: once from the modelled ratio of A to B, and again from the modelled ratio of A to C. These two predictions were then averaged. For some locations where there was limited overlap between series, the predicted ratio did not make sense, and a regional ratio was used.

Finally, variance was calculated both across series (within a location-year) as well as across years (within a location-source). Additionally, if a location-year had one imputed point was, the variance was multiplied by 2. If a location-year had two imputed points, the variance was multiplied by 4. The average estimates in each location-year were the input to an ST-GPR model. For this, we used a simple mixed effects model, which was modelled in log space with nested location random effects. Subnational estimates were then further modelled by splitting the country-level estimates using current smoking prevalence.

Theoretical minimum-risk exposure level

The theoretical minimum-risk exposure level is 0.

Exposure among current and former smokers

Identical to GBD 2017, we estimated exposure among current smokers for two continuous indicators: cigarettes per smoker per day and pack-years. Pack-years incorporates aspects of both duration and amount. One pack-year represents the equivalent of smoking one pack of cigarettes (assuming a 20-cigarette pack) per day for one year. Since the pack-years indicator collapses duration and intensity into a single dimension, one pack-year of exposure can reflect smoking 40 cigarettes per day for six months or smoking 10 cigarettes per day for two years.

To produce these indicators, we simulated individual smoking histories based on distributions of age of initiation and amount smoked. We informed the simulation with cross-sectional survey data capturing these indicators, modelled at the mean level for all locations, years, ages, and sexes using ST-GPR. We rescaled estimates of cigarettes per smoker per day to an envelope of cigarette consumption based on supply-side data. We estimated pack-years of exposure by summing samples from age- and time-specific distributions of cigarettes per smoker for a birth cohort in order to capture both age trends and time trends and avoid the common assumption that the amount someone currently smokes is the amount they have smoked since they began smoking. All distributions were age-, sex-, and region- specific ensemble distributions, which were found to outperform any single distribution.

We estimated exposure among former smokers using years since cessation. We utilised ST-GPR to model mean age of cessation using cross-sectional survey data capturing age of cessation. Using these

estimates, we generated ensemble distributions of years since cessation for every location, year, age group, and sex.

Relative risk

The same risk-outcome pairs from GBD 2017 were used: tuberculosis, lower respiratory tract infections, oesophageal cancer, stomach cancer, bladder cancer, liver cancer, laryngeal cancer, lung cancer, breast cancer, cervical cancer, colorectal cancer, lip and oral cancer, nasopharyngeal cancer, other pharyngeal cancer, pancreatic cancer, kidney cancer, leukaemia, ischaemic heart disease, ischaemic stroke, haemorrhagic stroke, subarachnoid haemorrhage, atrial fibrillation and flutter, aortic aneurysm, peripheral arterial disease, chronic obstructive pulmonary disease, other chronic respiratory diseases, asthma, peptic ulcer disease, gallbladder and biliary tract diseases, Alzheimer disease and other dementias, Parkinson disease (protective), multiple sclerosis, type-II diabetes, rheumatoid arthritis, low back pain, cataracts, macular degeneration, and fracture.

Dose-response risk curves

Input data for relative risks were nearly the same as in GBD 2017. The only addition was for chronic obstructive pulmonary disease, for which a few additional studies were included. We synthesised effect sizes by cigarettes per smoker per day, pack-years, and years since quitting from cohort and case-control studies to produce nonlinear dose-response curves using a Bayesian meta-regression model. For outcomes with significant differences in effect size by sex or age, we produced sex- or age-specific risk curves.

We estimated risk curves of former smokers compared to never smokers taking into account the rate of risk reduction among former smokers seen in the cohort and case-control studies, and the cumulative exposure among former smokers within each age, sex, location, and year group.

Population attributable fraction (PAF)

As in GBD 2017, we estimated PAFs based on the following equation:

$$PAF = \frac{p(n) + p(f)\int \exp(x) * rr(x) + p(c)\int \exp(y) * rr(y) - 1}{p(n) + p(f)\int \exp(x) * rr(x) + p(c)\int \exp(y) * rr(y)}$$

where $p(n)$ is the prevalence of never smokers, $p(f)$ is the prevalence of former smokers, $p(c)$ is the prevalence of current smokers, $\exp(x)$ is a distribution of years since quitting among former smokers, $rr(x)$ is the relative risk for years since quitting, $\exp(y)$ is a distribution of cigarettes per smoker per day or pack-years, and $rr(y)$ is the relative risk for cigarettes per smoker per day or pack-years.

We used pack-years as the exposure definition for cancers and chronic respiratory diseases, and cigarettes per smoker per day for cardiovascular diseases and all other health outcomes.

References

1. Ng M, Freeman MK, Fleming TD, Robinson M, Dwyer-Lindgren L, Thomson B, et al. Smoking Prevalence and Cigarette Consumption in 187 Countries, 1980–2012. *JAMA*. 2014 Jan 8;311(2):183–92.

Secondhand smoke

Flowchart

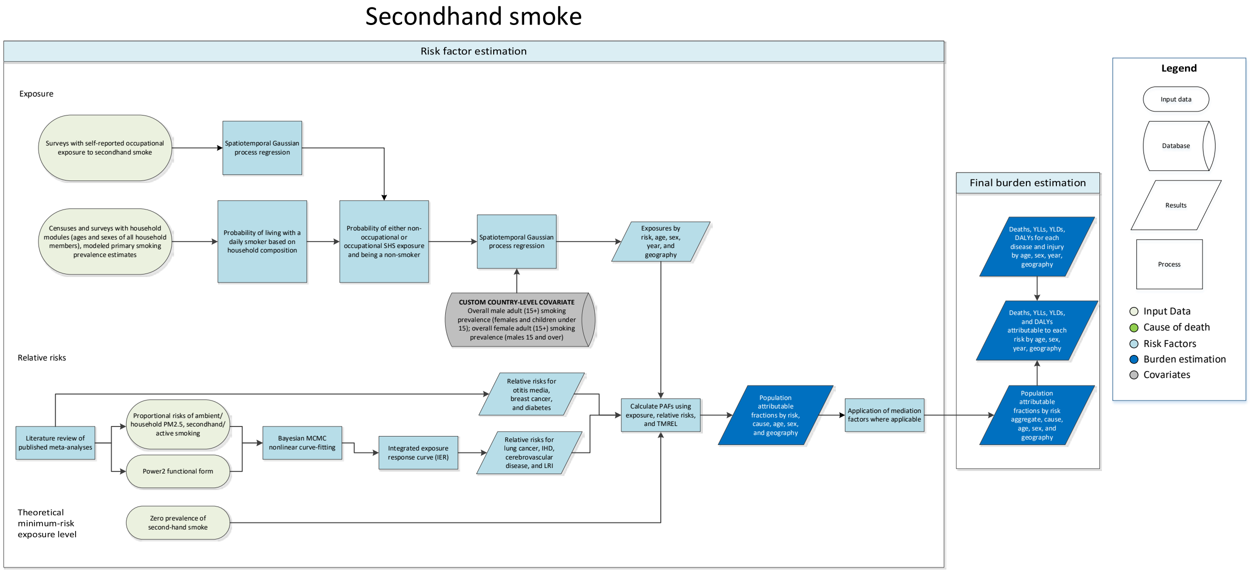

Exposure

Case definition

We define secondhand smoke exposure as current exposure to secondhand tobacco smoke at home, at work, or in other public places. We use household composition as a proxy for non-occupational secondhand smoke exposure and make the assumption that all persons living with a daily smoker are exposed to tobacco smoke. We use surveys to estimate the proportion of individuals exposed to secondhand smoke at work. We only consider non-smokers to be exposed to secondhand smoke. Non-smokers are defined as all persons who are not daily smokers. Ex-smokers and occasional smokers are considered non-smokers in this analysis. Exposure is evaluated for both children and adults.

Input data

To calculate the proportion of non-smokers who live with at least one smoker, we used unit record data on household composition, which included the ages and sexes of all persons living in the same household. Our sources included representative major survey series with a household composition module, including the Demographic Health Surveys (DHS), the Multiple Indicator Cluster Surveys (MICS), and the Living Standards Measurement Surveys (LSMS); and national and subnational censuses, which included those captured in the IPUMS project and identified using the Global Health Data Exchange catalog (GHDx).

To calculate the proportion of individuals exposed to secondhand smoke at work, by age and sex, we used cross-sectional surveys that ask respondents about self-reported occupational secondhand smoke exposure. Sources include the Global Adult Tobacco Surveys, Eurobarometer Surveys, and WHO STEPS Surveys. We identified sources using the GHDx.

No major changes have been introduced to data inputs since 2016. A new systematic review is planned for the next GBD round. Table 1 summarizes exposure input data.

Input data	Exposure
Source count (total)	721
Number of countries with data	153

Given the nature of the data used in our models (microdata), no crosswalk for case definition adjustment or age- and sex-splitting processes were required. Estimates of daily smoking prevalence in each location were also used in our calculations, as described in the modelling strategy section below.

Modelling strategy

Identical to GBD 2017, we estimated the probability that each person is living with a smoker and is also a non-smoker themselves using set theory. First, household composition data were used at the individual level to capture the ages and sexes of each person in the household. Second, we analysed surveys with both household composition data and tobacco use questions and determined that the distribution of household size, mean age of the household members, and the age distribution were not significantly different between households with and without a self-reported smoker. Since we did not find that household composition varied between smokers and non-smokers, we then used the GBD 2019 primary daily smoking prevalence model to calculate the probability that each household member is a daily smoker. Next, we used the probability of the union of sets on each individual household member to calculate the overall probability that at least one of the other household members was a daily smoker. As in GBD 2017, we incorporated occupational exposure by modelling prevalence of current exposure to secondhand smoke at work, by age, sex, location, and year, using ST-GPR. In order to avoid double counting we calculated the probability that an individual is exposed through either non-occupational exposure or occupational exposure, given their age, sex, and household composition. Finally, we multiplied this probability of exposure by the probability that the individual is not a smoker themselves (ie, 1 minus primary daily smoking prevalence for that person's location, year, age, and sex). We then collapsed these individual-level probabilities to produce average probabilities of exposure by location, year, age, and sex.

These probabilities were modelled in the GBD ST-GPR framework, which generates exposure estimates from a mixed effects hierarchical linear model plus weighted residuals smoothed across time, space, and age. The linear model formula was fit separately by sex using restricted maximum likelihood in R.

We used the sex-specific overall daily smoking prevalence for adults (age 15 and older) as a country-level covariate in the model. The overall male adult daily smoking prevalence was used as the covariate for females of all ages and for males under age 15. The overall female adult daily smoking prevalence was used as the covariate for males age 15 and older.

All input datapoints from the probability calculation had a measure of uncertainty (variance and sample size) coming from the uncertainty of the primary smoking prevalence model and the sample size from

the unit record data going into the modelling process. Geographical random effects were used in model fitting but were not used in prediction.

Theoretical minimum-risk exposure level

The theoretical minimum-risk exposure level for secondhand smoke is zero exposure among non-smokers, meaning that non-smokers would not live with any primary smokers.

Relative risks

The same risk-outcome pairs from GBD 2017 were used. For children ages 0-14, we estimated the burden of otitis media attributable to secondhand smoke exposure. For all ages we estimated the burden of lower respiratory infections (LRI), and for adults greater than or equal to 25 years of age we estimated the burden of lung cancer, chronic obstructive pulmonary disease (COPD), ischaemic heart disease, and cerebrovascular disease attributable to secondhand smoke exposure, breast cancer, and type 2 diabetes.

For lung cancer, ischaemic heart disease, cerebrovascular disease, and LRI, we used country-specific relative risks created using integrated exposure response curves (IER) for $PM_{2.5}$ air pollution. IER curve calculation was updated with the GBD 2019 cigarettes per smoker estimates. The relative risks for otitis media[1], breast cancer[2], and diabetes[3] are derived from published meta-analyses and are the same as the ones used in the previous GBD cycle. Table 2 summarizes relative risk input data.

Input data	Exposure
Source count (total)	232
Number of countries with data	34

We used the standard GBD population attributable fraction (PAF) equation to estimate burden based on exposure and relative risks.

References

1. Jones LL, Hassanien A, Cook DG, Britton J, Leonardi-Bee J. Parental smoking and the risk of middle ear disease in children. Arch Pediatr Adolesc Med. 2012; 166: 18–27.
2. Macacu A, Autier P, Boniol M, Boyle P. Active and passive smoking and risk of breast cancer: a meta-analysis. Breast Cancer Res Treat 2015; 154:213–224.
3. Zhu B, Wu X, Wang X, Zheng Q, Sun G. The association between passive smoking and type 2 diabetes: a meta-analysis. Asia-Pacific Journal of Public Health 2014; 26:226-237.

Ambient particulate matter pollution

Flowchart

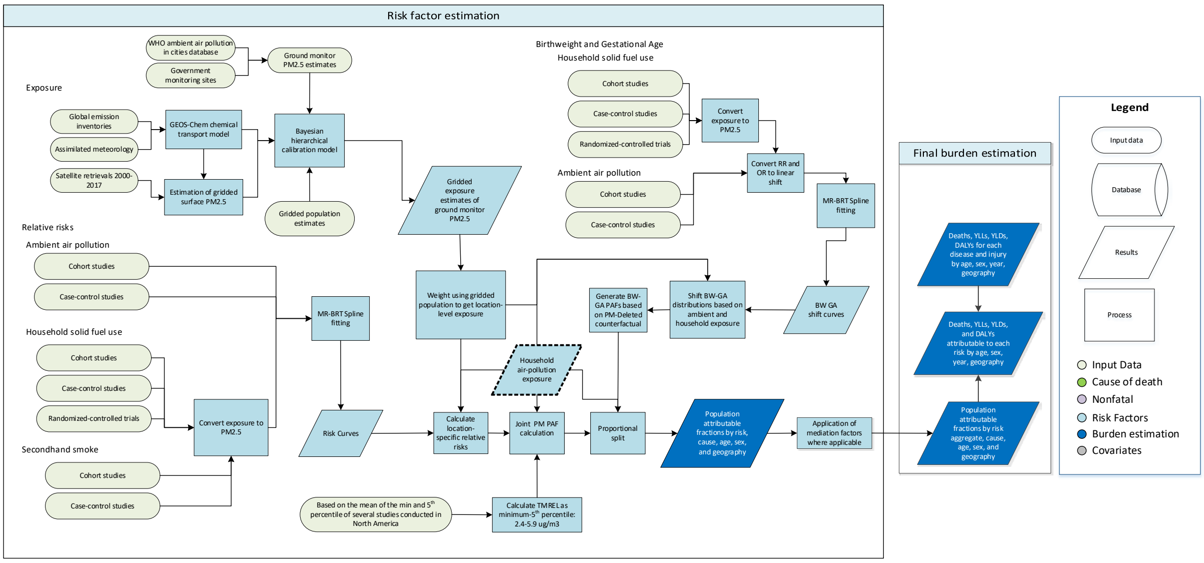

Input data and modelling strategy

Exposure

Definition

Exposure to ambient particulate matter pollution is defined as the population-weighted annual average mass concentration of particles with an aerodynamic diameter less than 2.5 micrometers ($PM_{2.5}$) in a cubic meter of air. This measurement is reported in µg/m^3.

Input data

The data used to estimate exposure to ambient particulate matter pollution comes from multiple sources, including satellite observations of aerosols in the atmosphere, ground measurements, chemical transport model simulations, population estimates, and land-use data. Table 1 summarizes exposure input data.

Table 1: Exposure Input Data

Input data	Exposure
Source count (total)	663
Number of countries with data	114

The following details the updates in methodology and input data used in GBD 2019.

$PM_{2.5}$ ground measurement database

Ground measurements used for GBD 2019 include updated measurements from sites included in 2017 and additional measurements from new locations. New and up-to-date data (mainly from the USA, Canada, EU, Bangladesh, China and USA embassies and consulates), were added to the data

from the 2018 update of the WHO Global Ambient Air Quality Database used in GBD 2017. The updated data included measurements of concentrations of PM_{10} and $PM_{2.5}$ from 10,408 ground monitors from 116 countries from 2010 to 2017. The majority of measurements were recorded in 2016 and 2017 (as there is a lag in reporting measurements, few data from 2018 or newer were available). Annual averages were excluded if they were based on less than 75% coverage within a year. If information on coverage was not available, then data were included unless there were already sufficient data within the same country (monitor density greater than 0.1).

For locations measuring only PM_{10}, $PM_{2.5}$ measurements were estimated from PM_{10}. This was performed using a hierarchy of conversion factors ($PM_{2.5}/PM_{10}$ ratios): (i) for any location a 'local' conversation factor was used, constructed as the ratio of the average measurements (of $PM_{2.5}$ and PM_{10}) from within 50km of the location of the PM_{10} measurement, and within the same country, if such measurements were available; (ii) if there was not sufficient local information to construct a conversion factor then a country-wide conversion factor was used; and (iii) if there was no appropriate information within a country, then a regional factor was used. In each case, to avoid the possible effects of outliers in the measured data (both $PM_{2.5}$ and PM_{10}), extreme values of the ratios were excluded (defined as being greater/lesser than the 95% and 5% quantiles of the empirical distributions of conversion factors). As with GBD 2013, 2015, 2016, and 2017 databases, in addition to values of $PM_{2.5}$ and whether they were direct measurement or converted from PM_{10}, the database also included additional information, where available, related to the ground measurements such as monitor geo-coordinates and monitor site type.

Satellite-based estimates

The global geophysical $PM_{2.5}$ estimates for the years 2000–2017 are from Hammer and colleagues Version V4.GL.03.NoGWR used at 0.1°x0.1° resolution (~11 x 11 km resolution at the equator).[1] The method is based on the algorithms of van Donkelaar and colleagues (2016) as used in GBD 2017,[2] with updated satellite retrievals, chemical transport modelling, and ground-based monitoring. The algorithm uses aerosol optical depth (AOD) from several updated satellite products (MAIAC, MODIS C6.1, and MISR v23), including finer resolution, increased global coverage, and improved long-term stability. Ground-based observations from a global sunphotometer network (AERONET version 3) are used to combine different AOD information sources. This is the first time that data from MAIAC at 1 km resolution was used to estimate $PM_{2.5}$ at the global scale. The GEOS-Chem chemical transport model with updated algorithms was used for geophysical relationships between surface $PM_{2.5}$ and AOD. Updates to the GEOS-Chem simulation included improved representation of mineral dust and secondary organic aerosol, as well as updated emission inventories. The resultant geophysical $PM_{2.5}$ estimates are highly consistent with ground monitors worldwide (R^2=0.81, slope = 1.03, n = 2541).

Population data

A comprehensive set of population data, adjusted to match UN2015 Population Prospectus, on a high-resolution grid was obtained from the Gridded Population of the World (GPW) database. Estimates for 2000, 2005, 2010, 2015, and 2020 were available from GPW version 4, with estimates for 1990 and 1995 obtained from the GPW version 3. These data are provided on a 0.0083°× 0.0083° resolution. Aggregation to each 0.1°×0.1° grid cell was accomplished by summing the central 12 × 12 population cells. Populations estimates for 2001–2004, 2006–2009, 2011–2014 and 2016–2019 were obtained by interpolation using natural splines with knots placed at 2000, 2005, 2010, 2015, and 2020. This was performed for each grid cell.

Chemical transport model simulations

Estimates of the sum of particulate sulfate, nitrate, ammonium, and organic carbon and the compositional concentrations of mineral dust simulated using the GEOS Chem chemical transport model, and a measure combining elevation and the distance to the nearest urban land surface (as described in van Donkelaar and colleagues 2016[2] and Hammer and colleagues (submitted))[1] were available for 2000–2017 for each 0.1°×0.1° grid cell.

Modelling strategy

The following is a summary of the modelling approach, known as the Data Integration Model for Air Quality (DIMAQ) used in GBD 2015, 2016, 2017, and now in GBD 2019.[3,4]

Before the implementation of DIMAQ (ie, in GBD 2010 and GBD 2013), exposure estimates were obtained using a single global function to calibrate available ground measurements to a "fused" estimate of $PM_{2.5}$; the mean of satellite-based estimates and those from the TM5 chemical transport model, calculated for each 0.1°×0.1° grid cell. This was recognised to represent a tradeoff between accuracy and computational efficiency when utilising all the available data sources. In particular, the GBD 2013 exposure estimates were known to underestimate ground measurements in specific locations (see discussion in Brauer and colleagues, 2015).[5] This underestimation was largely due to the use of a single, global calibration function, whereas in reality the relationship between ground measurements and other variables will vary spatially.

In GBD 2015 and GBD 2016, coefficients in the calibration model were estimated for each country. Where data were insufficient within a country, information can be "borrowed" from a higher aggregation (region) and, if enough information is still not available, from an even higher level (super-region). Individual country-level estimates were therefore based on a combination of information from the country, its region, and its super-region. This was implemented within a Bayesian hierarchical modelling (BHM) framework. BHMs provide an extremely useful and flexible framework in which to model complex relationships and dependencies in data. Uncertainty can also be propagated through the model, allowing uncertainty arising from different components, both data sources and models, to be incorporated within estimates of uncertainty associated with the final estimates. The results of the modelling comprise a posterior distribution for each grid cell, rather than just a single point estimate, allowing a variety of summaries to be calculated. The primary outputs here are the median and 95% credible intervals for each grid cell. Based on the availability of ground measurement data, modelling and evaluation were focused on the year 2016.

The model used in GBD 2017 and GBD 2019 also included within-country calibration variation.[6] The model used for GBD 2019, henceforth referred to as DIMAQ2, provides a number of substantial improvements over the initial formulation of DIMAQ. In DIMAQ, ground measurements from different years were all assumed to have been made in the primary year of interest and then regressed against values from other inputs (eg, satellites, etc.) made in that year. In the presence of changes over time, therefore, and particularly in areas where no recent measurements were available, there was the possibility of mismatches between the ground measurements and other variables. In DIMAQ2, ground measurements were matched with other inputs (over time), and the (global-level) coefficients were allowed to vary over time, subject to smoothing that is induced by a first-order random walk process. In addition, the manner in which spatial variation can be incorporated within the model has developed: where there are sufficient data, the calibration equations can now vary (smoothly) both within and between countries, achieved by allowing the coefficients to follow (smooth) Gaussian processes. Where there are insufficient data within a

country, to produce accurate equations, as before, information is borrowed from lower down the hierarchy and it is supplemented with information from the wider region.

DIMAQ2 as described above is used for all regions except for the north Africa and Middle East and sub-Saharan Africa super-regions, where there are insufficient data across years to allow the extra complexities of the new model to be implemented. In these super-regions, a simplified version of DIMAQ2 is used in which the temporal component is dropped.

Model evaluation

Model development and comparison was performed using within- and out-of-sample assessment. In the evaluation, cross-validation was performed using 25 combinations of training (80%) and validation (20%) datasets. Validation sets were obtained by taking a stratified random sample, using sampling probabilities based on the cross-tabulation of $PM_{2.5}$ categories (0-24.9, 25-49.9, 50-74.9, 75-99.9, 100+ μg/m^3) and super-regions, resulting in them having the same distribution of $PM_{2.5}$ concentrations and super-regions as the overall set of sites. The following metrics were calculated for each training/evaluation set combination: for model fit – R^2 and deviance information criteria (DIC, a measure of model fit for Bayesian models); for predictive accuracy – root mean squared error (RMSE) and population weighted root mean squared error (PwRMSE). The median R^2 was 0.9, and the median PwRMSE was 10.1 μg/m^3.

All modelling was performed on the log-scale. The choice of which variables were included in the model was made based on their contribution to model fit and predictive ability. The following is a list of variables and model structures that were included in DIMAQ.

Continuous explanatory variables:

- (SAT) Estimate of $PM_{2.5}$ (in μg/m^3) from satellite remote sensing on the log-scale.
- (POP) Estimate of population for the same year as SAT on the log-scale.
- (SNAOC) Estimate of the sum of sulfate, nitrate, ammonium, and organic carbon simulated using the GEOS Chem chemical transport model.
- (DST) Estimate of compositional concentrations of mineral dust simulated using the GEOS-Chem chemical transport model.
- (EDxDU) The log of the elevation difference between the elevation at the ground measurement location and the mean elevation within the GEOS Chem simulation grid cell multiplied by the inverse distance to the nearest urban land surface.

Discrete explanatory variables:

- (LOC) Binary variable indicating whether exact location of ground measurement is known.
- (TYPE) Binary variable indicating whether exact type of ground monitor is known.
- (CONV) Binary variable indicating whether ground measurement is $PM_{2.5}$ or converted from PM_{10}.

Interactions*:*

- Interactions between the binary variables and the effects of SAT.

Random effects:

- Regional temporal (random walk) hierarchical random-effects on the intercept
- Regional hierarchical random-effects for the coefficient associated with SAT
- Regional hierarchical random-effects for the coefficient associated with POP
- Smoothed, spatially varying random-effects for the intercept

- Smoothed, spatially varying random-effects for the coefficient associated with SAT

Inference and prediction

Due to both the complexity of the models and the size of the data, notably the number of spatial predictions that are required, recently developed techniques that perform "approximate" Bayesian inference based on integrated nested Laplace approximations (INLA) were used.[7] Computation was performed using the R interface to the INLA computational engine (R-INLA). GBD 2019 also makes use of an innovation in the way that samples from the (Bayesian) model are used to represent distributions of estimated concentrations in each grid-cell. Here estimates, and distributions representing uncertainty, of concentrations for each grid are obtained by taking repeated (joint) samples from the posterior distributions of the parameters and calculating estimates based on a linear combination of those samples and the input variables.[8]

DIMAQ2 was used to produce estimates of ambient $PM_{2.5}$ for 1990, 1995, and 2010–2019 by matching the gridded estimates with the corresponding coefficients from the calibration. As there is a lag in reporting ambient air pollution based quantities, the input variables were extrapolated (as in GBD 2017), allowing estimates for 2018 and 2019 to be produced in the same way as other years and, crucially, allowing measures of uncertainty to be produced within the BHM framework rather than by using post-hoc approximations.

Estimates from the satellites and the GEOS-Chem chemical transport model in 2018 and 2019 were produced by extrapolating estimates from 2000–2017 using generalised additive models,[9] on a cell-by-cell basis, except in those grid cells that saw a >100% increase between 2016 and 2017, in which case only the 2000–2016 estimates were used for extrapolating, in order to avoid unrealistic and/or unjustified extrapolation of trends. Population estimates for 2018 and 2019 were obtained by interpolation as described above.

Theoretical minimum-risk exposure level

The TMREL was assigned a uniform distribution with lower/upper bounds given by the average of the minimum and fifth percentiles of outdoor air pollution cohort studies exposure distributions conducted in North America, with the assumption that current evidence was insufficient to precisely characterise the shape of the concentration-response function below the fifth percentile of the exposure distributions. The TMREL was defined as a uniform distribution rather than a fixed value in order to represent the uncertainty regarding the level at which the scientific evidence was consistent with adverse effects of exposure. The specific outdoor air pollution cohort studies selected for this averaging were based on the criteria that their fifth percentiles were less than that of the American Cancer Society Cancer Prevention II (CPSII) cohort's fifth percentile of 8.2 based on Turner and colleagues (2016).[10] This criterion was selected since GBD 2010 used the minimum, 5.8, and fifth percentile solely from the CPS II cohort. The resulting lower/upper bounds of the distribution for GBD 2019 were 2.4 and 5.9. This has not changed since GBD 2015.

Relative risks and population attributable fractions

We create one set of cause-specific risk curves for both household air pollution and ambient air pollution as two different sources of $PM_{2.5}$. In GBD 2017, we estimated the particulate matter-attributable burden of disease based on the relation of long-term exposure to PM2.5 with Ischemic Heart Disease, stroke (ischemic and hemorrhagic), COPD, lung cancer, acute lower respiratory infection, and Type II Diabetes. In GBD 2019, we added adverse birth outcomes including low birthweight and short gestation. Because these are already risk factors (and not outcomes) in the

GBD, we performed a mediation analysis, in which a proportion of the burden attributable to low birthweight and short gestation was attributed to $PM_{2.5}$ pollution.

For the six non-mediated outcomes, we used results from cohort and case-control studies of ambient $PM_{2.5}$ pollution, cohort studies, case-control studies, and randomised-controlled trials of household use of solid fuel for cooking, and cohort and case-control studies of secondhand smoke. For the first time in GBD 2019, we no longer use active smoking data in the risk curves

For GBD 2019, we made several important changes to the risk functions. Previously, we have used relative risk estimates for active smoking, converting cigarettes-per-day to $PM_{2.5}$ exposure in order to estimate the $PM_{2.5}$ relative risk at the highest end of the $PM_{2.5}$ exposure-response curve. We took this approach because the vast majority of the air pollution epidemiological studies have been performed in low-pollution settings in high-income countries, preventing us from extrapolating the steep relationship at the beginning of the exposure range to locations with high exposure but no relative risk estimates, such as India and China. However, with the recent publication of studies in China and other higher-exposure settings and additional studies of HAP, we have been able to include more estimates at high $PM_{2.5}$ levels in the model.[11,12,13,14,15] Furthermore, in contrast to previous cycles of the GBD where the power function used to develop the IER required the inclusion of active smoking data to anchor the risk function, with the current use of splines and their flexibility, it is easier to fit functions to the (ambient, household, and SHS) data without active smoking data. Beginning in GBD 2019, we excluded active smoking studies from the risk curves. Removal of active smoking information removes an important source of uncertainty in our earlier estimates related to differences in dose rates and other aspects of exposure between active smoking and the other $PM_{2.5}$ sources, including differences in voluntary (active smoking) and involuntary (ambient and household $PM_{2.5}$, secondhand smoke) exposure.[16,17]

Additionally, in the past, we have built the curves for ischaemic heart disease and stroke based on studies of mortality and used evidence from three studies of both mortality and incidence to scale down the mortality curves to generate estimates of incidence risk. This year we extracted incidence and mortality from all available studies and included this as a covariate in the model. There was no significant difference between estimates of incidence risk and mortality risk, so we included both types of risk estimates in the curve fitting and used the same curve for both incidence and mortality. This is what was done for all other outcomes in the past and in GBD 2019.

For cardiovascular diseases, evidence suggests that the relative risk decreases with age.[18] To account for this in our model, we generate unique risk curves for every five-year age group from 25–29 to 95 and older for both ischaemic heart disease and stroke. Because we do not have risk data for every unique age group, we adjust each study based on the median age during follow-up to generate a full adjusted dataset for every curve. We calculate the median age of follow-up by taking the median (or mean) age at enrollment and adding one-half of median or mean follow-up time. If follow-up time is not available, we take 70% of total study period based on the observed ratio of follow-up time to total study period for other studies.

Once we have a median age during follow-up (a), we extrapolate each study to the full set of ages where the estimated datapoint for age, a_j, is calculated with the following equation and accompanying explanatory figure:

$$\log(RR)_{a_j} = \frac{\log(RR)_a - 0}{a - 110} * (a_j - 110)$$

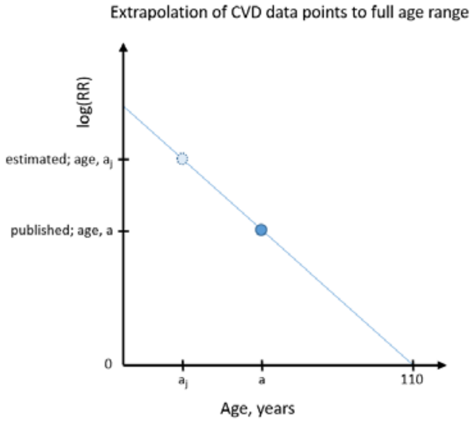

Previously we have used a fixed functional form to fit the risk curves.[16] In GBD 2019, we used MR-BRT (described in detail elsewhere) splines to fit the risk data with a more flexible shape. While previously we built in the TMREL estimates into the model fitting, this year we have fit the curve beginning at zero exposure and incorporate the TMREL into the relative risk calculation process. This allows others to use our risk curves with whatever counterfactual level is of interest to them. Relative risk curves are available upon request.

When fitting the risk curves, we consider the published relative risk over a range of exposure data. For OAP studies, the relative risk informs the curve from the fifth to the 95th percentile of observed exposure. When this is not available in the published study, we estimate the distribution from the provided information (mean and standard deviation, mean and IQR, etc.). We scale the RR to this range.

For HAP studies, we allow each study to inform the curve from the Exp_{OAP} to Exp_{OAP}+Exp_{HAP}, where Exp_{OAP} is the GBD 2017 estimate of the ambient exposure level in the study location and year, and Exp_{HAP} is the GBD 2017 estimate of the excess exposure for those who use solid fuel for cooking in the study location and year.

For SHS studies, we updated our strategy of exposure estimation in GBD 2019. For the first time, we are also accounting for outdoor exposure. Similar to the approach used for HAP, we allow each study to inform the curve from the Exp_{OAP} to Exp_{OAP}+Exp_{SHS}, where Exp_{OAP} is the GBD 2017 estimate of the ambient exposure level in the study location and year, and Exp_{SHS} is an estimate of the excess exposure for those who experience secondhand smoke. This is estimated from the number of cigarettes smoked per smoker per day in a given location and year, estimated by the smoking team of GBD, and from a study in Sweden, which measured the $PM_{2.5}$ exposure in homes of smokers.[19] We divided the household $PM_{2.5}$ exposure level by the average number of cigarettes smoked per smoker per day in Sweden over the study duration to estimate the SHS $PM_{2.5}$ exposure per cigarette (2.31 µg/m^3 [95% UI 1.53–3.39]). To calculate Exp_{SHS} we multiplied the estimated number of cigarettes per smoker per day by the average $PM_{2.5}$ exposures per cigarette to generate a predicted $PM_{2.5}$ exposure level.

MR-BRT risk splines

We fit splines on the datasets including studies of OAP, HAP, and SHS using the following functional form, where X and X_{CF} represent the range of exposure characterised by the effect size:

$$log\left(\frac{MRBRT(X)}{MRBRT(X_{CF})}\right) \sim \log(Published\ Effect\ Size)$$

For each of the risk-outcome pairs, we tested various model settings and priors in fitting the MR-BRT splines. The final models used third-order splines with two interior knots and a constraint on the right-most segment, forcing the fit to be linear rather than cubic. We used an ensemble approach to knot placement, wherein 100 different models were run with randomly placed knots and then combined by weighting based on a measure of fit that penalises excessive changes in the third derivative of the curve. Knots were free to be placed anywhere within the fifth and 95th percentile of the data, as long as a minimum width of 10% of that domain exists between them. We included shape constraints so that the risk curves were concave down and monotonically increasing, the most biologically plausible shape for the $PM_{2.5}$ risk curve. On the non-linear segments, we included a Gaussian prior on the third derivative of mean 0 and variance 0.01 to prevent over-fitting; on the linear segment, a stronger prior of mean 0 and variance 1e-6 was used to ensure that the risk curves do not continue to increase beyond the range of the data.

For chronic obstructive pulmonary disease, we used a looser Gaussian prior of mean 0 and variance 1e-4 on the linear segment of the risk function. For this outcome, we have epidemiological evidence from household air pollution that the risk continues to increase at higher levels of $PM_{2.5}$.

Table 2 summarizes relative risk input data for ambient particulate matter pollution and household air pollution.

Table 2: Relative Risk Input Data

Input data	Relative risk
Source count (total)	200
Number of countries with data	40

The following table includes all ambient and household sources used in generating risk curves.

Source	Citation
1	Abusalah A, Gavana M, Haidich AB, Smyrnakis E, Papadakis N, Papanikolaou A, Benos A. Low birth weight and prenatal exposure to indoor pollution from tobacco smoke and wood fuel smoke: a matched case-control study in Gaza Strip. Matern Child Health J. 2012; 16(8): 1718-27.
2	Akhtar T, Ullah Z, Khan MH, Nazli R. Chronic bronchitis in women using solid biomass fuel in rural Peshawar, Pakistan. Chest. 2007; 132(5): 1472–5.
3	Alam DS, Chowdhury MAH, Siddiquee AT, Ahmed S, Hossain MD, Pervin S, Streatfield K, Cravioto A, Niessen LW. Adult Cardiopulmonary Mortality and Indoor Air Pollution: A 10-Year Retrospective Cohort Study in a Low-Income Rural Setting. Glob Heart. 2012; 7(3): 215–21.
4	Alexander DA, Northcross A, Karrison T, Morhasson-Bello O, Wilson N, Atalabi OM, Dutta A, Adu D, Ibigbami T, Olamijulo J, Adepoju D, Ojengbede O, Olopade CO. Pregnancy outcomes and ethanol cook stove intervention: A randomized-controlled trial in Ibadan, Nigeria. Environ Int. 2018; 111: 152-163.
5	Al-Sonboli N, Hart CA, Al-Aghbari N, Al-Ansi A, Ashoor O, Cuevas LE. Human metapneumovirus and respiratory syncytial virus disease in children, Yemen. Emerg Infect Dis. 2006; 12(9): 1437–9.
6	Atkinson RW, Carey IM, Kent AJ, van Staa TP, Anderson HR, Cook DG. Long-term exposure to outdoor air pollution and the incidence of chronic obstructive pulmonary disease in a national English cohort. Occup Environ Med. 2015; 72(1): 42–8.
7	Azizi BH, Zulkifli HI, Kasim MS. Protective and risk factors for acute respiratory infections in hospitalized urban Malaysian children: a case control study. Southeast Asian J Trop Med Public Health. 1995; 26(2): 280–5.
8	Balakrishnan K, Ghosh S, Thangavel G, Sambandam S, Mukhopadhyay K, Puttaswamy N, Sadasivam A, Ramaswamy P, Johnson P, Kuppuswamy R, Natesan D, Maheshwari U, Natarajan A, Rajendran G, Ramasami R, Madhav S, Manivannan S, Nargunanadan S, Natarajan S, Saidam S, Chakraborty M, Balakrishnan L,

Source	Citation
	Thanasekaraan V. Exposures to fine particulate matter (PM2.5) and birthweight in a rural-urban, mother-child cohort in Tamil Nadu, India. Environ Res. 2018; 161: 524–31.
9	Basu R, Harris M, Sie L, Malig B, Broadwin R, Green R. Effects of fine particulate matter and its constituents on low birth weight among full-term infants in California. Environ Res. 2014; 128: 42–51.
10	Beelen R, Hoek G, van den Brandt PA, Goldbohm RA, Fischer P, Schouten LJ, Jerrett M, Hughes E, Armstrong B, Brunekreef B. Long-Term Effects of Traffic-Related Air Pollution on Mortality in a Dutch Cohort (NLCS-AIR Study) [Unpublished data]. Environ Health Perspect. 2008; 116(2): 196–202.
11	Beelen R, Hoek G, van den Brandt PA, Goldbohm RA, Fischer P, Schouten LJ, Jerrett M, Hughes E, Armstrong B, Brunekreef B. Long-Term Effects of Traffic-Related Air Pollution on Mortality in a Dutch Cohort (NLCS-AIR Study). Environ Health Perspect. 2008; 116(2): 196–202.
12	Beelen R, Stafoggia M, Raaschou-Nielsen O, Andersen ZJ, Xun WW, Katsouyanni K, Dimakopoulou K, Brunekreef B, Weinmayr G, Hoffmann B, Wolf K, Samoli E, Houthuijs D, Nieuwenhuijsen M, Oudin A, Forsberg B, Olsson D, Salomaa V, Lanki T, Yli-Tuomi T, Oftedal B, Aamodt G, Nafstad P, De Faire U, Pedersen NL, Östenson CG, Fratiglioni L, Penell J, Korek M, Pyko A, Eriksen KT, Tjønneland A, Becker T, Eeftens M, Bots M, Meliefste K, Wang M, Bueno-de-Mesquita B, Sugiri D, Krämer U, Heinrich J, de Hoogh K, Key T, Peters A, Cyrys J, Concin H, Nagel G, Ineichen A, Schaffner E, Probst-Hensch N, Dratva J, Ducret-Stich R, Vilier A, Clavel-Chapelon F, Stempfelet M, Grioni S, Krogh V, Tsai MY, Marcon A, Ricceri F, Sacerdote C, Galassi C, Migliore E, Ranzi A, Cesaroni G, Badaloni C, Forastiere F, Tamayo I, Amiano P, Dorronsoro M, Katsoulis M, Trichopoulou A, Vineis P, Hoek G. Long-term exposure to air pollution and cardiovascular mortality: an analysis of 22 European cohorts. Epidemiology. 2014; 25(3): 368–378.
13	Bell ML, Belanger K, Ebisu K, Gent JF, Lee HJ, Koutrakis P, Leaderer BP. Prenatal Exposure to Fine Particulate Matter and Birth Weight: Variations by Particulate Constituents and Sources. Epidemiology. 2010; 21(6): 884–91.
14	Bell ML, Ebisu K, Belanger K. Ambient Air Pollution and Low Birth Weight in Connecticut and Massachusetts. Environ Health Perspect. 2007; 115(7): 1118–24.
15	Benmarhnia T, Huang J, Basu R, Wu J, Bruckner TA. Decomposition Analysis of Black-White Disparities in Birth Outcomes: The Relative Contribution of Air Pollution and Social Factors in California. Environ Health Perspect. 2017; 125(10): 107003.
16	Bowe B, Xie Y, Li T, Yan Y, Xian H, Al-Aly Z. The 2016 global and national burden of diabetes mellitus attributable to PM2·5 air pollution. Lancet Planet Health. 2018; 2(7): e301–12.
17	Boy E, Bruce N, Delgado H. Birth weight and exposure to kitchen wood smoke during pregnancy in rural Guatemala. Environ Health Perspect. 2002; 110(1): 109-14.
18	Brauer M, Lencar C, Tamburic L, Koehoorn M, Demers P, Karr C. A cohort study of traffic-related air pollution impacts on birth outcomes. Environ Health Perspect. 2008; 116(5): 680-6.
19	Broor S, Pandey RM, Ghosh M, Maitreyi RS, Lodha R, Singhal T, Kabra SK. Risk factors for severe acute lower respiratory tract infection in under-five children. Indian Pediatr. 2001; 1361-9.
20	Burnett RT. Cox Proportional Survival Model Hazard Ratios from Census Year to 2011 for Adults Aged 25 to 89 in CanCHEC Cohort.
21	Carey IM, Atkinson RW, Kent AJ, van Staa T, Cook DG, Anderson HR. Mortality associations with long-term exposure to outdoor air pollution in a national English cohort. Am J Respir Crit Care Med. 2013; 187(11): 1226-33.
22	Cesaroni G, Badaloni C, Gariazzo C, Stafoggia M, Sozzi R, Davoli M, Forastiere F. Long-term exposure to urban air pollution and mortality in a cohort of more than a million adults in Rome. Environ Health Perspect. 2013; 121(3): 324–31.
23	Chang HH, Reich BJ, Miranda ML. A spatial time-to-event approach for estimating associations between air pollution and preterm birth. J R Stat Soc Ser C Appl Stat. 2013; 62(2).
24	Chen H, Burnett RT, Kwong JC, Villeneuve PJ, Goldberg MS, Brook RD, van Donkelaar A, Jerrett M, Martin RV, Brook JR, Copes R. Risk of incident diabetes in relation to long-term exposure to fine particulate matter in Ontario, Canada. Environ Health Perspect. 2013; 121(7): 804–10.
25	Chen LH, Knutsen SF, Shavlik D, Beeson WL, Petersen F, Ghamsary M, Abbey D. The association between fatal coronary heart disease and ambient particulate air pollution: Are females at greater risk?. Environ Health Perspect. 2005; 113(12): 1723-9.
26	Clark C, Sbihi H, Tamburic L, Brauer M, Frank LD, Davies HW. Association of Long-Term Exposure to Transportation Noise and Traffic-Related Air Pollution with the Incidence of Diabetes: A Prospective Cohort Study. Environ Health Perspect. 2017; 125(8): 087025.
27	Clemens T, Turner S, Dibben C. Maternal exposure to ambient air pollution and fetal growth in North-East Scotland: A population-based study using routine ultrasound scans. Environ Int. 2017; 107: 216–26.
28	Coker E, Ghosh J, Jerrett M, Gomez-Rubio V, Beckerman B, Cockburn M, Liverani S, Su J, Li A, Kile ML, Ritz B, Molitor J. Modeling spatial effects of PM(2.5) on term low birth weight in Los Angeles County. Environ Res. 2015; 142: 354-64.

Source	Citation
29	Collings DA, Sithole SD, Martin KS. Indoor woodsmoke pollution causing lower respiratory disease in children. Trop Doct. 1990; 20(4): 151–5.
30	Coogan PF, White LF, Yu J, Burnett RT, Seto E, Brook RD, Palmer JR, Rosenberg L, Jerrett M. PM2.5 and Diabetes and Hypertension Incidence in the Black Women's Health Study. Epidemiology. 2016; 27(2): 202–10.
31	Dadvand P, Ostro B, Figueras F, Foraster M, Basagaña X, Valentín A, Martinez D, Beelen R, Cirach M, Hoek G, Jerrett M, Brunekreef B, Nieuwenhuijsen MJ. Residential proximity to major roads and term low birth weight: the roles of air pollution, heat, noise, and road-adjacent trees. Epidemiology. 2014; 25(4): 518-25.
32	Darrow LA, Klein M, Strickland MJ, Mulholland JA, Tolbert PE. Ambient Air Pollution and Birth Weight in Full-Term Infants in Atlanta, 1994–2004. Environ Health Perspect. 2011; 119(5): 731–7.
33	Dennis RJ, Maldonado D, Norman S, Baena E, Martinez G. Woodsmoke exposure and risk for obstructive airways disease among women. Chest. 1996; 109(1): 115–9.
34	Dherani M, Pope D, Mascarenhas M, Smith KR, Weber M, Bruce N. Indoor air pollution from unprocessed solid fuel use and pneumonia risk in children aged under five years: a systematic review and meta-analysis. Bull World Health Organ. 2008; 86(5): 390-398C and Kossove D. and Jeena PM, Ayannusi OE, Annamalai K, Naidoo P, Coovadia HM, Guldner P. Risk factors for admission and the role of respiratory syncytial virus-specific cytotoxic T-lymphocyte responses in children with acute bronchiolitis. S Afr Med J. 2003; 93(4): 291–4.
35	Ebisu K, Bell ML. Airborne PM2.5 chemical components and low birth weight in the northeastern and mid-Atlantic regions of the United States. Environ Health Perspect. 2012; 120(12): 1746-52.
36	Ebisu K, Berman JD, Bell ML. Exposure to coarse particulate matter during gestation and birth weight in the U.S. Environ Int. 2016; 94: 519–24.
37	Erickson AC, Ostry A, Chan LH, Arbour L. The reduction of birth weight by fine particulate matter and its modification by maternal and neighbourhood-level factors: a multilevel analysis in British Columbia, Canada. Environ Health. 2016; 15: 51.
38	Ezzati M, Kammen DM. Indoor air pollution from biomass combustion and acute respiratory infections in Kenya: an exposure-response study. Lancet. 2001; 358(9282): 619–24.
39	Fleischer NL, Merialdi M, van Donkelaar A, Vadillo-Ortega F, Martin RV, Betran AP, Souza JP. Outdoor air pollution, preterm birth, and low birth weight: analysis of the world health organization global survey on maternal and perinatal health. Environ Health Perspect. 2014; 122(4): 425-30.
40	Fonseca W, Kirkwood BR, Victora CG, Fuchs SR, Flores JA, Misago C. Risk factors for childhood pneumonia among the urban poor in Fortaleza, Brazil: a case--control study. Bull World Health Organ. 1996; 74(2): 199–208.
41	Galeone C, Pelucchi C, La Vecchia C, Negri E, Bosetti C, Hu J. Indoor air pollution from solid fuel use, chronic lung diseases and lung cancer in Harbin, Northeast China. Eur J Cancer Prev. 2008; 17(5): 473–8.
42	Gan WQ, FitzGerald JM, Carlsten C, Sadatsafavi M, Brauer M. Associations of ambient air pollution with chronic obstructive pulmonary disease hospitalization and mortality. Am J Respir Crit Care Med. 2013; 187(7): 721–7.
43	Gan WQ, Koehoorn M, Davies HW, Demers PA, Tamburic L, Brauer M. Long-Term Exposure to Traffic-Related Air Pollution and the Risk of Coronary Heart Disease Hospitalization and Mortality. Environ Health Perspect. 2011; 119(4): 501–7.
44	Geer LA, Weedon J, Bell ML. Ambient air pollution and term birth weight in Texas from 1998 to 2004. J Air Waste Manag Assoc. 2012; 62(11): 1285–95.
45	Gehring U, Tamburic L, Sbihi H, Davies HW, Brauer M. Impact of Noise and Air Pollution on Pregnancy Outcomes. Epidemiology. 2014; 25(3): 351–8.
46	Gehring U, Wijga AH, Fischer P, de Jongste JC, Kerkhof M, Koppelman GH, Smit HA, Brunekreef B. Traffic-related air pollution, preterm birth and term birth weight in the PIAMA birth cohort study. Environ Res. 2011; 111(1): 125–35.
47	Ger LP, Hsu WL, Chen KT, Chen CJ. Risk Factors of Lung Cancer by Histological Category in Taiwan. Anticancer Res. 1993; 13(5A): 1491–500.
48	Gray SC, Edwards SE, Schultz BD, Miranda ML. Assessing the impact of race, social factors and air pollution on birth outcomes: a population-based study. Environ Health. 2014; 13(1): 4.
49	Gray SC, Gelfand AE, Miranda ML. Hierarchical spatial modeling of uncertainty in air pollution and birth weight study. Stat Med. 2011; 30(17): 2187-98.
50	Gupta D, Boffetta P, Gaborieau V, Jindal SK. Risk factors of lung cancer in Chandigarh, India. Indian J Med Res. 2001; 113: 142–50.
51	Ha S, Hu H, Roussos-Ross D, Haidong K, Roth J, Xu X. The effects of air pollution on adverse birth outcomes. Environ Res. 2014; 134: 198-204.
52	Hansen AB, Ravnskjær L, Loft S, Andersen KK, Bräuner EV, Baastrup R, Yao C, Ketzel M, Becker T, Brandt J, Hertel O, Andersen ZJ. Long-term exposure to fine particulate matter and incidence of diabetes in the Danish Nurse Cohort. Environ Int. 2016; 91: 243–50.

Source	Citation
53	Hao H, Chang HH, Holmes HA, Mulholland JA, Klein M, Darrow LA, Strickland MJ. Air Pollution and Preterm Birth in the U.S. State of Georgia (2002-2006): Associations with Concentrations of 11 Ambient Air Pollutants Estimated by Combining Community Multiscale Air Quality Model (CMAQ) Simulations with Stationary Monitor Measurements. Environ Health Perspect. 2016; 124(6): 875-80.
54	Hao Y, Strosnider H, Balluz L, Qualters JR. Geographic Variation in the Association between Ambient Fine Particulate Matter (PM2.5) and Term Low Birth Weight in the United States. Environ Health Perspect. 2016; 124(2): 250-5.
55	Harris G, Thompson WD, Fitzgerald E, Wartenberg D. The association of PM(2.5) with full term low birth weight at different spatial scales. Environ Res. 2014; 134: 427-34.
56	Hart J, Garshick E, Dockery D, Smith T, Ryan L, Laden F. Long-Term Ambient Multipollutant Exposures and Mortality. Am J Respir Crit Care Med. 2011; 183: 75–8.
57	Hart JE, Puett RC, Rexrode KM, Albert CM, Laden F. Effect Modification of Long-Term Air Pollution Exposures and the Risk of Incident Cardiovascular Disease in US Women. J Am Heart Assoc. 2015; 4(12).
58	Heft-Neal S, Burney J, Bendavid E, Burke M. Robust relationship between air quality and infant mortality in Africa. Nature. 2018; 559(7713): 2548.
59	Hertz-Picciotto I, Baker RJ, Yap P-S, Dostál M, Joad JP, Lipsett M, Greenfield T, Herr CEW, Benes I, Shumway RH, Pinkerton KE, Srám R. Early childhood lower respiratory illness and air pollution. Environ Health Perspect. 2007; 115(10): 1510-8.
60	Huang C, Zhang X, Qiao Z, Guan L, Peng S, Liu J, Xie R, Zheng L. A case-control study of dietary factors in patients with lung cancer. Biomed Environ Sci. 1992; 5(3): 257–65.
61	Huynh M, Woodruff TJ, Parker JD, Schoendorf KC. Relationships between air pollution and preterm birth in California. Paediatr Perinat Epidemiol. 2006; 20(6): 454-61.
62	Hyder A, Lee HJ, Ebisu K, Koutrakis P, Belanger K, Bell ML. PM2.5 Exposure and Birth Outcomes: Use of Satellite- and Monitor-Based Data. Epidemiology. 2014; 25(1): 58–67.
63	Hystad P, Demers PA, Johnson KC, Carpiano RM, Brauer M. Long-term residential exposure to air pollution and lung cancer risk. Epidemiology. 2013; 24(5): 762-72.
64	Hystad P, Duong M, Brauer M, Larkin A, Arku R, Kurmi OP, Fan WQ, Avezum A, Azam I, Chifamba J, Dans A, du Plessis JL, Gupta R, Kumar R, Lanas F, Liu Z, Lu Y, Lopez-Jaramillo P, Mony P, Mohan V, Mohan D, Nair S, Puoane T, Rahman O, Lap AT, Wang Y, Wei L, Yeates K, Rangarajan S, Teo K, Yusuf S, on behalf of Prospective Urban and Rural Epidemiological (PURE) Study investigators. Health Effects of Household Solid Fuel Use: Findings from 11 Countries within the Prospective Urban and Rural Epidemiology Study. Environ Health Perspect. 2019; 127(5): 57003.
65	Hystad P, Duong M, Brauer M, Larkin A, Arku R, Kurmi OP, Fan WQ, Avezum A, Azam I, Chifamba J, Dans A, du Plessis JL, Gupta R, Kumar R, Lanas F, Liu Z, Lu Y, Lopez-Jaramillo P, Mony P, Mohan V, Mohan D, Nair S, Puoane T, Rahman O, Lap AT, Wang Y, Wei L, Yeates K, Rangarajan S, Teo K, Yusuf S, on behalf of Prospective Urban and Rural Epidemiological (PURE) Study investigators. Health Effects of Household Solid Fuel Use: Findings from 11 Countries within the Prospective Urban and Rural Epidemiology Study [Unpublished]. Environ Health Perspect. 2019; 127(5): 57003.
66	Hystad P, Larkin A, Rangarajan S, PURE country investigators, Yusuf S, Brauer M. Outdoor fine particulate matter air pollution and cardiovascular disease: Results from 747 communities across 21 countries in the PURE Study [Unpublished].
67	Jedrychowski W, Perera F, Mrozek-Budzyn D, Mroz E, Flak E, Spengler JD, Edwards S, Jacek R, Kaim I, Skolicki Z. Gender differences in fetal growth of newborns exposed prenatally to airborne fine particulate matter. Environ Res. 2009; 109(4): 447-56.
68	Jin C, Rossignol AM. Effects of passive smoking on respiratory illness from birth to age eighteen months, in Shanghai, People's Republic of China. J Pediatr. 1993; 123(4): 553–8.
69	Johnson AW, Aderele WI. The association of household pollutants and socio-economic risk factors with the short-term outcome of acute lower respiratory infections in hospitalized pre-school Nigerian children. Ann Trop Paediatr. 1992; 12(4): 421–32.
70	Karr C, Lumley T, Schreuder A, Davis R, Larson T, Ritz B, Kaufman J. Effects of subchronic and chronic exposure to ambient air pollutants on infant bronchiolitis. Am J Epidemiol. 2007; 165(5): 553-60.
71	Karr CJ, Rudra CB, Miller KA, Gould TR, Larson T, Sathyanarayana S, Koenig JQ. Infant exposure to fine particulate matter and traffic and risk of hospitalization for RSV bronchiolitis in a region with lower ambient air pollution. Environ Res. 2009; 109(3): 321-7.
72	Katanoda K, Sobue T, Satoh H, Tajima K, Suzuki T, Nakatsuka H, Takezaki T, Nakayama T, Nitta H, Tanabe K, Tominaga S. An association between long-term exposure to ambient air pollution and mortality from lung cancer and respiratory diseases in Japan. J Epidemiol. 2011; 21(2): 132-43.
73	Kim C, Seow WJ, Shu X-O, Bassig BA, Rothman N, Chen BE, Xiang Y-B, Hosgood HD, Ji B-T, Hu W, Wen C, Chow W-H, Cai Q, Yang G, Gao Y-T, Zheng W, Lan Q. Cooking Coal Use and All-Cause and Cause-Specific Mortality in a Prospective Cohort Study of Women in Shanghai, China. Environ Health Perspect. 2016; 124(9): 1384–9.

Source	Citation
74	Kleinerman RA, Wang Z, Wang L, Metayer C, Zhang S, Brenner AV, Zhang S, Xia Y, Shang B, Lubin JH. Lung cancer and indoor exposure to coal and biomass in rural China. J Occup Environ Med. 2002; 44(4): 338–44.
75	Kloog I, Melly SJ, Ridgway WL, Coull BA, Schwartz J. Using new satellite based exposure methods to study the association between pregnancy pm2.5 exposure, premature birth and birth weight in Massachusetts. Environ Health. 2012; 11(1).
76	Ko YC, Lee CH, Chen MJ, Huang CC, Chang WY, Lin HJ, Wang HZ, Chang PY. Risk factors for primary lung cancer among non-smoking women in Taiwan. Int J Epidemiol. 1997; 26(1): 24-31.
77	Kumar N. Uncertainty in the relationship between criteria pollutants and low birth weight in Chicago. Atmos Environ. 2012; 49: 171–9.
78	Kumar S, Awasthi S, Jain A, Srivastava RC. Blood zinc levels in children hospitalized with severe pneumonia: a case control study. Indian Pediatr. 2004; 41(5): 486–91.
79	Lan Q, He X, Shen M, Tian L, Liu LZ, Lai H, Chen W, Berndt SI, Hosgood HD, Lee K-M, Zheng T, Blair A, Chapman RS. Variation in lung cancer risk by smoky coal subtype in Xuanwei, China. Int J Cancer. 2008; 123(9): 2164–9.
80	Laurent O, Hu J, Li L, Cockburn M, Escobedo L, Kleeman MJ, Wu J. Sources and contents of air pollution affecting term low birth weight in Los Angeles County, California, 2001-2008. Environ Res. 2014; 134: 488-95.
81	Laurent O, Hu J, Li L, Kleeman MJ, Bartell SM, Cockburn M, Escobedo L, Wu J. A Statewide Nested Case-Control Study of Preterm Birth and Air Pollution by Source and Composition: California, 2001-2008. Environ Health Perspect. 2016; 124(9): 1479-86.
82	Laurent O, Hu J, Li L, Kleeman MJ, Bartell SM, Cockburn M, Escobedo L, Wu J. Low birth weight and air pollution in California: Which sources and components drive the risk?. Environ Int. 2016; 92-93: 471-7.
83	Laurent O, Wu J, Li L, Chung J, Bartell S. Investigating the association between birth weight and complementary air pollution metrics: a cohort study. Environ Health. 2013; 12(1).
84	Lavigne E, Yasseen AS 3rd, Stieb DM, Hystad P, van Donkelaar A, Martin RV, Brook JR, Crouse DL, Burnett RT, Chen H, Weichenthal S, Johnson M, Villeneuve PJ, Walker M. Ambient air pollution and adverse birth outcomes: Differences by maternal comorbidities. Environ Res. 2016; 148: 457-466.
85	Le CH, Ko YC, Cheng LS, Lin YC, Lin HJ, Huang MS, Huang JJ, Kao EL, Wang HZ. The heterogeneity in risk factors of lung cancer and the difference of histologic distribution between genders in Taiwan. Cancer Causes Control. 2001; 12(4): 289–300.
86	Lepeule J, Laden F, Dockery D, Schwartz J. Chronic exposure to fine particles and mortality: an extended follow-up of the Harvard Six Cities study from 1974 to 2009 - Unpublished data. Environ Health Perspect. 2012; 120(7): 965-70.
87	Lepeule J, Laden F, Dockery D, Schwartz J. Chronic exposure to fine particles and mortality: an extended follow-up of the Harvard Six Cities study from 1974 to 2009. Environ Health Perspect. 2012; 120(7): 965-70.
88	Lim CC, Hayes RB, Ahn J, Shao Y, Silverman DT, Jones RR, Garcia C, Thurston GD. Association between long-term exposure to ambient air pollution and diabetes mortality in the US. Environ Res. 2018; 165: 330-36
89	Lipsett MJ, Ostro BD, Reynolds P, Goldberg D, Hertz A, Jerrett M, Smith DF, Garcia C, Chang ET, Bernstein L. Long-term exposure to air pollution and cardiorespiratory disease in the California teachers study cohort [Unpublished data]. Am J Respir Crit Care Med. 2011; 184(7): 828-35.
90	Lipsett MJ, Ostro BD, Reynolds P, Goldberg D, Hertz A, Jerrett M, Smith DF, Garcia C, Chang ET, Bernstein L. Long-term exposure to air pollution and cardiorespiratory disease in the California teachers study cohort. Am J Respir Crit Care Med. 2011; 184(7): 828-35.
91	Lissowska J, Bardin-Mikolajczak A, Fletcher T, Zaridze D, Szeszenia-Dabrowska N, Rudnai P, Fabianova E, Cassidy A, Mates D, Holcatova I, Vitova V, Janout V, Mannetje A, Brennan P, Boffetta P. Lung cancer and indoor pollution from heating and cooking with solid fuels: the IARC international multicentre case-control study in Eastern/Central Europe and the United Kingdom. Am J Epidemiol. 2005; 162(4): 326–33.
92	Luo RX, Wu B, Yi YN, Huang ZW, Lin RT. Indoor burning coal air pollution and lung cancer--a case-control study in Fuzhou, China. Lung Cancer. 1996; 14 Suppl 1: S113-119.
93	MacIntyre EA, Gehring U, Mölter A, Fuertes E, Klümper C, Krämer U, Quass U, Hoffmann B, Gascon M, Brunekreef B, Koppelman GH, Beelen R, Hoek G, Birk M, de Jongste JC, Smit HA, Cyrys J, Gruzieva O, Korek M, Bergström A, Agius RM, de Vocht F, Simpson A, Porta D, Forastiere F, Badaloni C, Cesaroni G, Esplugues A, Fernández-Somoano A, Lerxundi A, Sunyer J, Cirach M, Nieuwenhuijsen MJ, Pershagen G, Heinrich J. Air Pollution and Respiratory Infections during Early Childhood: An Analysis of 10 European Birth Cohorts within the ESCAPE Project. Environ Health Perspect. 2014; 122(1): 107–13.
94	Mahalanabis D, Gupta S, Paul D, Gupta A, Lahiri M, Khaled MA. Risk factors for pneumonia in infants and young children and the role of solid fuel for cooking: a case-control study. Epidemiol Infect. 2002; 129(1): 65–71.
95	Miller KA, Siscovick DS, Sheppard L, Shepherd K, Sullivan JH, Anderson GL, Kaufman JD. Long-term exposure to air pollution and incidence of cardiovascular events in women. N Engl J Med. 2007; 356(5): 447-58.
96	Morello-Frosch R, Jesdale BM, Sadd JL, Pastor M. Ambient air pollution exposure and full-term birth weight in California. Environ Health. 2010; 9(1).

Source	Citation
97	Naess Ø, Nafstad P, Aamodt G, Claussen B, Rosland P. Relation between concentration of air pollution and cause-specific mortality: four-year exposures to nitrogen dioxide and particulate matter pollutants in 470 neighborhoods in Oslo, Norway. Am J Epidemiol. 2007; 165(4): 435-43.
98	Park SK, Adar SD, O'Neill MS, Auchincloss AH, Szpiro A, Bertoni AG, Navas-Acien A, Kaufman JD, Diez-Roux AV. Long-term exposure to air pollution and type 2 diabetes mellitus in a multiethnic cohort. Am J Epidemiol. 2015; 181(5): 327–36.
99	Parker JD, Kravets N, Vaidyanathan A. Particulate Matter Air Pollution Exposure and Heart Disease Mortality Risks by Race and Ethnicity in the United States. Circulation. 2018; 137(16): 1688–97.
100	Parker JD, Woodruff TJ, Basu R, Schoendorf KC. Air Pollution and Birth Weight Among Term Infants in California. Pediatrics. 2005; 115(1): 121–8.
101	Parker JD, Woodruff TJ. Influences of study design and location on the relationship between particulate matter air pollution and birthweight. Paediatr Perinat Epidemiol. 2008; 22(3): 214–27.
102	Pereira G, Belanger K, Ebisu K, Bell ML. Fine particulate matter and risk of preterm birth in Connecticut in 2000-2006: a longitudinal study. Am J Epidemiol. 2014; 179(1): 67-74.
103	Pereira G, Bell ML, Belanger K, de Klerk N. Fine particulate matter and risk of preterm birth and pre-labor rupture of membranes in Perth, Western Australia 1997-2007: a longitudinal study. Environ Int. 2014; 73: 143-9.
104	Pinault L, Tjepkema M, Crouse DL, Weichenthal S, van Donkelaar A, Martin RV, Brauer M, Chen H, Burnett RT. Risk estimates of mortality attributed to low concentrations of ambient fine particulate matter in the Canadian community health survey cohort [Unpublished]. Environ Health. 2016; 15: 18.
105	Pinault L, Tjepkema M, Crouse DL, Weichenthal S, van Donkelaar A, Martin RV, Brauer M, Chen H, Burnett RT. Risk estimates of mortality attributed to low concentrations of ambient fine particulate matter in the Canadian community health survey cohort. Environ Health. 2016; 15(1): 18.
106	Puett RC, Hart JE, Schwartz J, Hu FB, Liese AD, Laden F. Are particulate matter exposures associated with risk of type 2 diabetes? Environ Health Perspect. 2011; 119(3): 384–9.
107	Puett RC, Hart JE, Suh H, Mittleman M, Laden F. Particulate matter exposures, mortality, and cardiovascular disease in the health professionals follow-up study. Environ Health Perspect. 2011; 119(8): 1130-5.
108	Puett RC, Hart JE, Yanosky JD, Paciorek C, Schwartz J, Suh H, Speizer FE, Laden F. Chronic fine and coarse particulate exposure, mortality, and coronary heart disease in the Nurses' Health Study. Environ Health Perspect. 2009; 117(11): 1697-701.
109	Qian Z, Liang S, Yang S, Trevathan E, Huang Z, Yang R, Wang J, Hu K, Zhang Y, Vaughn M, Shen L, Liu W, Li P, Ward P, Yang L, Zhang W, Chen W, Dong G, Zheng T, Xu S, Zhang B. Ambient air pollution and preterm birth: A prospective birth cohort study in Wuhan, China. Int J Hyg Environ Health. 2016; 219(2): 195-203.
110	Qiu H, Schooling CM, Sun S, Tsang H, Yang Y, Lee RS, Wong CM, Tian L. Long-term exposure to fine particulate matter air pollution and type 2 diabetes mellitus in elderly: A cohort study in Hong Kong. Environ Int. 2018; 113: 350-56.
111	Raaschou-Nielsen O, Andersen ZJ, Beelen R, Samoli E, Stafoggia M, Weinmayr G, Hoffmann B, Fischer P, Nieuwenhuijsen MJ, Brunekreef B, Xun WW, Katsouyanni K, Dimakopoulou K, Sommar J, Forsberg B, Modig L, Oudin A, Oftedal B, Schwarze PE, Nafstad P, De Faire U, Pedersen NL, Ostenson C-G, Fratiglioni L, Penell J, Korek M, Pershagen G, Eriksen KT, Sørensen M, Tjønneland A, Ellermann T, Eeftens M, Peeters PH, Meliefste K, Wang M, Bueno-de-Mesquita B, Key TJ, de Hoogh K, Concin H, Nagel G, Vilier A, Grioni S, Krogh V, Tsai M-Y, Ricceri F, Sacerdote C, Galassi C, Migliore E, Ranzi A, Cesaroni G, Badaloni C, Forastiere F, Tamayo I, Amiano P, Dorronsoro M, Trichopoulou A, Bamia C, Vineis P, Hoek G. Air pollution and lung cancer incidence in 17 European cohorts: prospective analyses from the European Study of Cohorts for Air Pollution Effects (ESCAPE). Lancet Oncol. 2013; 14(9): 813–22.
112	Robin LF, Less PS, Winget M, Steinhoff M, Moulton LH, Santosham M, Correa A. Wood-burning stoves and lower respiratory illnesses in Navajo children. Pediatr Infect Dis J. 1996; 15(10): 859–65.
113	Sapkota A, Gajalakshmi V, Jetly DH, Roychowdhury S, Dikshit RP, Brennan P, Hashibe M, Boffetta P. Indoor air pollution from solid fuels and risk of hypopharyngeal/laryngeal and lung cancers: a multicentric case-control study from India. Int J Epidemiol. 2008; 37(2): 321–8.
114	Sasco AJ, Merrill RM, Dari I, Benhaïm-Luzon V, Carriot F, Cann CI, Bartal M. A case-control study of lung cancer in Casablanca, Morocco. Cancer Causes Control. 2002; 13(7): 609–16.
115	Savitha MR, Nandeeshwara SB, Pradeep Kumar MJ, ul-Haque F, Raju CK. Modifiable risk factors for acute lower respiratory tract infections. Indian J Pediatr. 2007; 74(5): 477–82.
116	Savitz DA, Bobb JF, Carr JL, Clougherty JE, Dominici F, Elston B, Ito K, Ross Z, Yee M, Matte TD. Ambient Fine Particulate Matter, Nitrogen Dioxide, and Term Birth Weight in New York, New York. Am J Epidemiol. 2014; 179(4): 457–66.
117	Sezer H, Akkurt I, Guler N, Marako?lu K, Berk S. A case-control study on the effect of exposure to different substances on the development of COPD. Ann Epidemiol. 2006; 16(1): 59–62.
118	Shah N, Ramankutty V, Premila PG, Sathy N. Risk factors for severe pneumonia in children in south Kerala: a hospital-based case-control study. J Trop Pediatr. 1994; 40(4): 201–6.

Source	Citation
119	Shen M, Chapman RS, Vermeulen R, Tian L, Zheng T, Chen BE, Engels EA, He X, Blair A, Lan Q. Coal use, stove improvement, and adult pneumonia mortality in Xuanwei, China: a retrospective cohort study. Environ Health Perspect. 2009; 117(2): 261–6.
120	Siddiqui AR, Gold EB, Yang X, Lee K, Brown KH, Bhutta ZA. Prenatal exposure to wood fuel smoke and low birth weight. Environ Health Perspect. 2008; 116(4): 543-9.
121	Smith KR, McCracken JP, Weber MW, Hubbard A, Jenny A, Thompson LM, Balmes J, Diaz A, Arana B, Bruce N. Effect of reduction in household air pollution on childhood pneumonia in Guatemala (RESPIRE): a randomised controlled trial. Lancet. 2011; 378(9804): 1717-26.
122	Smith RB, Fecht D, Gulliver J, Beevers SD, Dajnak D, Blangiardo M, Ghosh RE, Hansell AL, Kelly FJ, Anderson HR, Toledano MB. Impact of London's road traffic air and noise pollution on birth weight: retrospective population based cohort study. BMJ. 2017; 359: j5299.
123	Stafoggia M, Cesaroni G, Peters A, Andersen ZJ, Badaloni C, Beelen R, Caracciolo B, Cyrys J, de Faire U, de Hoogh K, Eriksen KT, Fratiglioni L, Galassi C, Gigante B, Havulinna AS, Hennig F, Hilding A, Hoek G, Hoffmann B, Houthuijs D, Korek M, Lanki T, Leander K, Magnusson PK, Meisinger C, Migliore E, Overvad K, Ostenson C-G, Pedersen NL, Pekkanen J, Penell J, Pershagen G, Pundt N, Pyko A, Raaschou-Nielsen O, Ranzi A, Ricceri F, Sacerdote C, Swart WJR, Turunen AW, Vineis P, Weimar C, Weinmayr G, Wolf K, Brunekreef B, Forastiere F. Long-term exposure to ambient air pollution and incidence of cerebrovascular events: results from 11 European cohorts within the ESCAPE project. Environ Health Perspect. 2014; 122(9): 919–25.
124	Stieb DM, Chen L, Beckerman BS, Jerrett M, Crouse DL, Omariba DW, Peters PA, van Donkelaar A, Martin RV, Burnett RT, Gilbert NL, Tjepkema M, Liu S, Dugandzic RM. Associations of Pregnancy Outcomes and PM2.5 in a National Canadian Study. Environ Health Perspect. 2016; 124(2): 243-9.
125	Thompson LM, Bruce N, Eskenazi B, Diaz A, Pope D, Smith KR. Impact of reduced maternal exposures to wood smoke from an introduced chimney stove on newborn birth weight in rural Guatemala. Environ Health Perspect. 2011; 119(10): 1489-94.
126	Thurston GD, Ahn J, Cromar KR, Shao Y, Reynolds HR, Jerrett M, Lim CC, Shanley R, Park Y, Hayes RB. Ambient Particulate Matter Air Pollution Exposure and Mortality in the NIH-AARP Diet and Health Cohort [Unpublished]. Environ Health Perspect. 2016; 124(4): 484-90.
127	Tielsch JM, Katz J, Thulasiraj RD, Coles CL, Sheeladevi S, Yanik EL, Rahmathullah L. Exposure to indoor biomass fuel and tobacco smoke and risk of adverse reproductive outcomes, mortality, respiratory morbidity and growth among newborn infants in south India. Int J Epidemiol. 2009; 38(5): 1351-63.
128	To T, Zhu J, Villeneuve PJ, Simatovic J, Feldman L, Gao C, Williams D, Chen H, Weichenthal S, Wall C, Miller AB. Chronic disease prevalence in women and air pollution--A 30-year longitudinal cohort study. Environ Int. 2015; 80: 26–32.
129	Tseng E, Ho W-C, Lin M-H, Cheng T-J, Chen P-C, Lin H-H. Chronic exposure to particulate matter and risk of cardiovascular mortality: cohort study from Taiwan. BMC Public Health. 2015; 15: 936.
130	Turner MC,Jerrett M, Pope CA 3rd, Krewski D, Gapstur SM, Diver WR, Beckerman BS, Marshall JD, Su J, Crouse DL, Burnett RT. Long-term ozone exposure and mortality in a large prospective study . Am J Respir Crit Care Med. 2016; 193(10): 1134-42.
131	Victora CG, Fuchs SC, Flores JA, Fonseca W, Kirkwood B. Risk factors for pneumonia among children in a Brazilian metropolitan area. Pediatrics. 1994; 977-85.
132	Villeneuve PJ, Weichenthal SA, Crouse D, Miller AB, To T, Martin RV, van Donkelaar A, Wall C, Burnett RT. Long-term exposure to fine particulate matter air pollution and mortality among Canadian women. Epidemiology. 2015; 26(4): 536-45.
133	Wayse V, Yousafzai A, Mogale K, Filteau S. Association of subclinical vitamin D deficiency with severe acute lower respiratory infection in Indian children under 5 y. Eur J Clin Nutr. 2004; 58(4): 563–7.
134	Weichenthal S, Villeneuve PJ, Burnett RT, van Donkelaar A, Martin RV, Jones RR, DellaValle CT, Sandler DP, Ward MH, Hoppin JA. Long-term exposure to fine particulate matter: association with nonaccidental and cardiovascular mortality in the agricultural health study cohort. Environ Health Perspect. 2014; 122(6): 609-15.
135	Weinmayr G, Hennig F, Fuks K, Nonnemacher M, Jakobs H, Möhlenkamp S, Erbel R, Jöckel K-H, Hoffmann B, Moebus S, Heinz Nixdorf Recall Investigator Group. Long-term exposure to fine particulate matter and incidence of type 2 diabetes mellitus in a cohort study: effects of total and traffic-specific air pollution. Environ Health. 2015; 14: 53.
136	Wesley AG, Loening WE. Assessment and 2-year follow-up of some factors associated with severity of respiratory infections in early childhood. S Afr Med J. 1996; 86(4): 365–8.
137	Wilhelm M, Ghosh JK, Su J, Cockburn M, Jerrett M, Ritz B. Traffic-related air toxics and preterm birth: a population-based case-control study in Los Angeles County, California. Environ Health. 2011; 10: 89.
138	Wong CM, Lai HK, Tsang H, Thach TQ, Thomas GN, Lam KBH, Chan KP, Yang L, Lau AKH, Ayres JG, Lee SY, Man Chan W, Hedley AJ, Lam TH. Satellite-Based Estimates of Long-Term Exposure to Fine Particles and Association with Mortality in Elderly Hong Kong Residents. Environ Health Perspect. 2015; 123(11): 1167-72.

Source	Citation
139	Wu AH, Henderson BE, Pike MC, Yu MC. Smoking and other risk factors for lung cancer in women. J Natl Cancer Inst. 1985; 74(4): 747-51.
140	Wu J, Wilhelm M, Chung J, Ritz B. Comparing exposure assessment methods for traffic-related air pollution in an adverse pregnancy outcome study. Environ Res. 2011; 111(5): 685-92.
141	Wylie BJ, Coull BA, Hamer DH, Singh MP, Jack D, Yeboah-Antwi K, Sabin L, Singh N, MacLeod WB. Impact of biomass fuels on pregnancy outcomes in central East India. Environ Health. 2014; 13(1): 1.
142	Wylie BJ, Kishashu Y, Matechi E, Zhou Z, Coull B, Abioye AI, Dionisio KL, Mugusi F, Premji Z, Fawzi W, Hauser R, Ezzati M. Maternal exposure to carbon monoxide and fine particulate matter during pregnancy in an urban Tanzanian cohort. Indoor Air. 2017; 27(1): 136-146.
143	Yin P, Brauer M, Cohen A, Burnett RT, Liu J, Liu Y, Liang R, Wang W, Qi J, Wang L, Zhou M. Long-term Fine Particulate Matter Exposure and Nonaccidental and Cause-specific Mortality in a Large National Cohort of Chinese Men [Unpublished]. Environ Health Perspect. 2017; 125(11): 117002.
144	Yin P, Brauer M, Cohen A, Burnett RT, Liu J, Liu Y, Liang R, Wang W, Qi J, Wang L, Zhou M. Long-term Fine Particulate Matter Exposure and Nonaccidental and Cause-specific Mortality in a Large National Cohort of Chinese Men. Environ Health Perspect. 2017; 125(11): 117002.
145	Yu K, Qiu G, Chan K-H, Lam K-BH, Kurmi OP, Bennett DA, Yu C, Pan A, Lv J, Guo Y, Bian Z, Yang L, Chen Y, Hu FB, Chen Z, Li L, Wu T. Association of Solid Fuel Use With Risk of Cardiovascular and All-Cause Mortality in Rural China. JAMA. 2018; 319(13): 1351–61.
146	Yucra S, Tapia V, Steenland K, Naeher LP, Gonzales GF. Association between biofuel exposure and adverse birth outcomes at high altitudes in Peru: a matched case-control study. Int J Occup Environ Health. 2011; 17(4): 307-13.

The following figures display risk curves for each outcome. The dashed line depicts the GBD 2017 IER including active smoking data, the dotted line depicts the GBD 2019 IER including active smoking data and updates to the AS and SHS exposure incorporation, and the solid line depicts the GBD 2019 MR-BRT curve without the inclusion of active smoking data. The grey shaded areas represent the 95% CI. The red box represents the TMREL area of the curve. On each page, the first figure depicts the typical range of outdoor exposure, whereas the second plot includes higher levels typical of household air pollution exposure.

Each point or number represents one study effect size. Each is plotted at the 95th percentile of the exposure distribution (OAP), the expected level of exposure for individual using solid fuel (HAP), or the expected level of exposure for individuals experiencing SHS. The relative risk is plotted relative to the predicted relative risk at the fifth percentile of exposure distribution (OAP), the expected (ambient only) level of exposure for individuals not using solid fuel (HAP), or the expected (ambient only) level of exposure for individuals not exposed to SHS. For example, a study predicting a relative risk of 1.5 for an exposure range of 10 to 20 would be plotted at (20, MRBRT(10)*1.5). Arrows represent studies that would have been outside the range of the plot but have been moved to include on the figure.

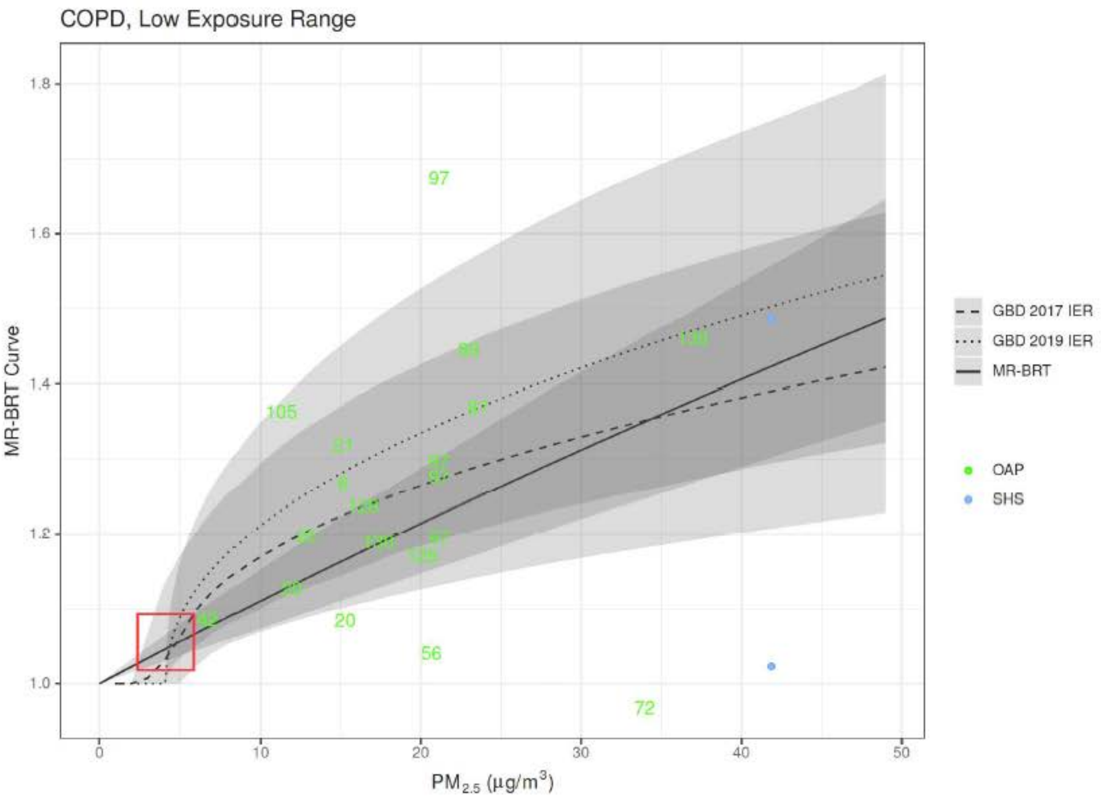
COPD, Low Exposure Range
MR-BRT Curve
PM2.5 (µg/m3)
GBD 2017 IER
GBD 2019 IER
MR-BRT
OAP
SHS
97
105
20
56
72
1.8
1.6
1.4
1.2
1.0
0
10
20
30
40
50

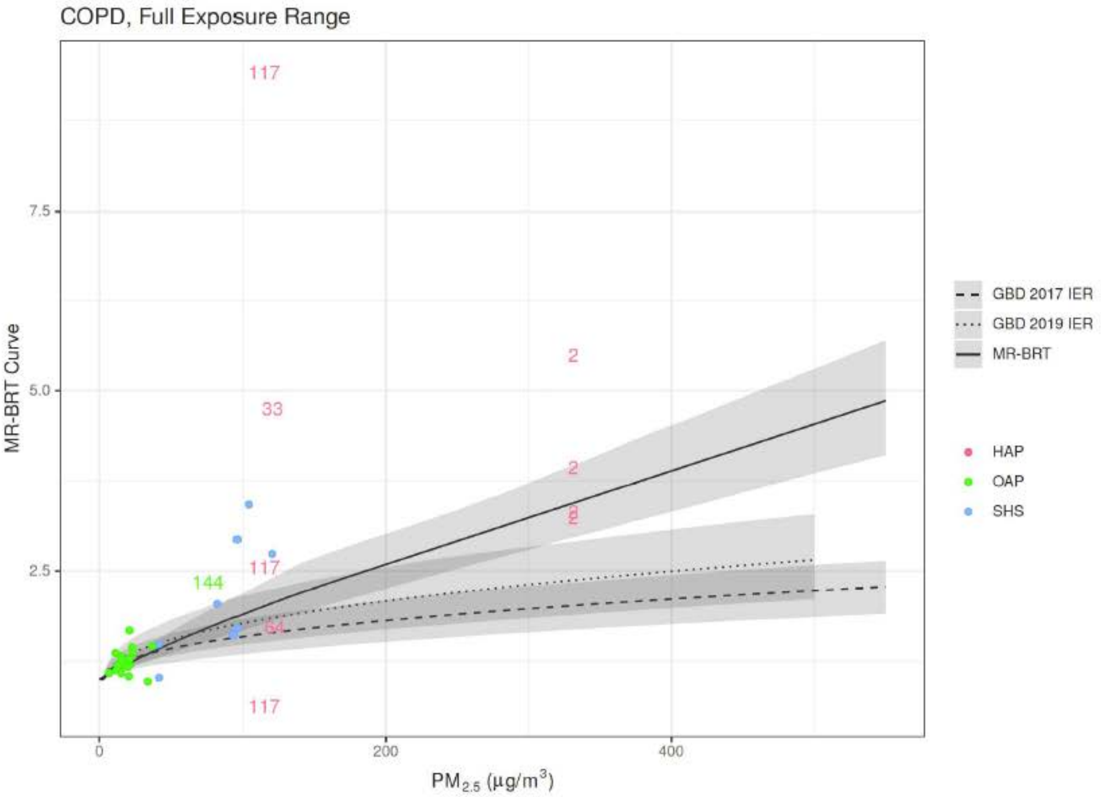
COPD, Full Exposure Range
MR-BRT Curve
PM2.5 (µg/m3)
GBD 2017 IER
GBD 2019 IER
MR-BRT
HAP
OAP
SHS
117
2
33
144
7.5
5.0
2.5
0
200
400

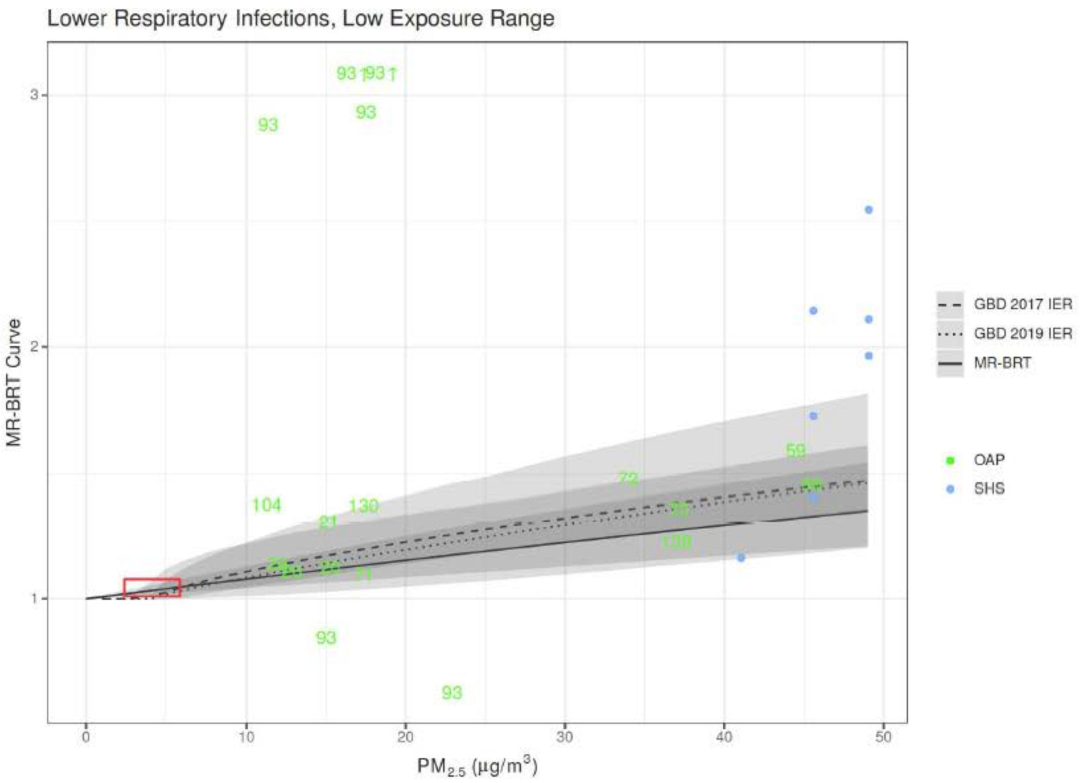
Lower Respiratory Infections, Low Exposure Range
MR-BRT Curve
PM$_{2.5}$ (µg/m^3)
GBD 2017 IER
GBD 2019 IER
MR-BRT
OAP
SHS

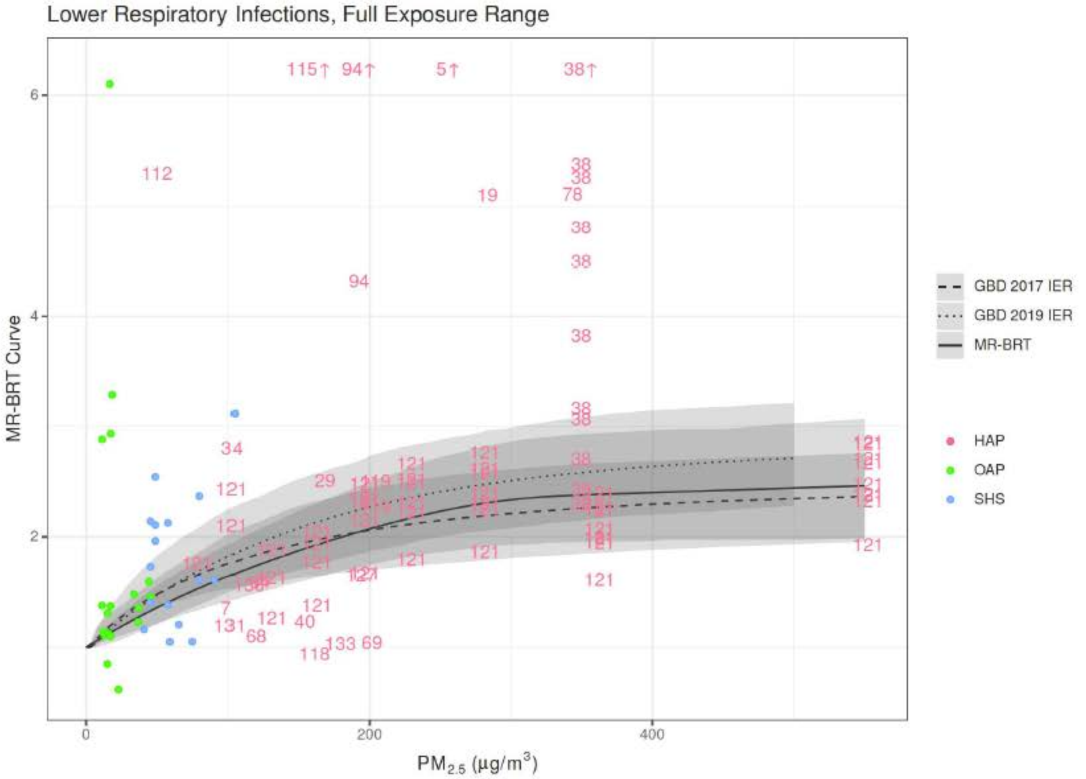
Lower Respiratory Infections, Full Exposure Range
MR-BRT Curve
PM$_{2.5}$ (µg/m^3)
GBD 2017 IER
GBD 2019 IER
MR-BRT
HAP
OAP
SHS

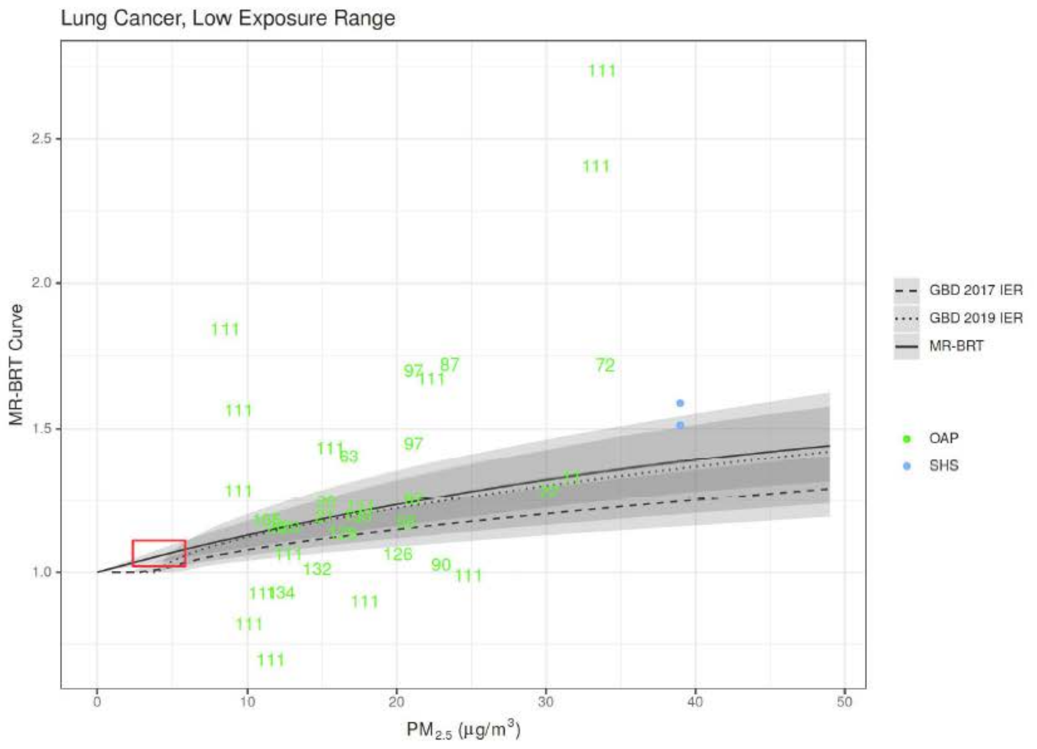
Lung Cancer, Low Exposure Range
MR-BRT Curve
PM2.5 (µg/m3)
GBD 2017 IER
GBD 2019 IER
MR-BRT
OAP
SHS

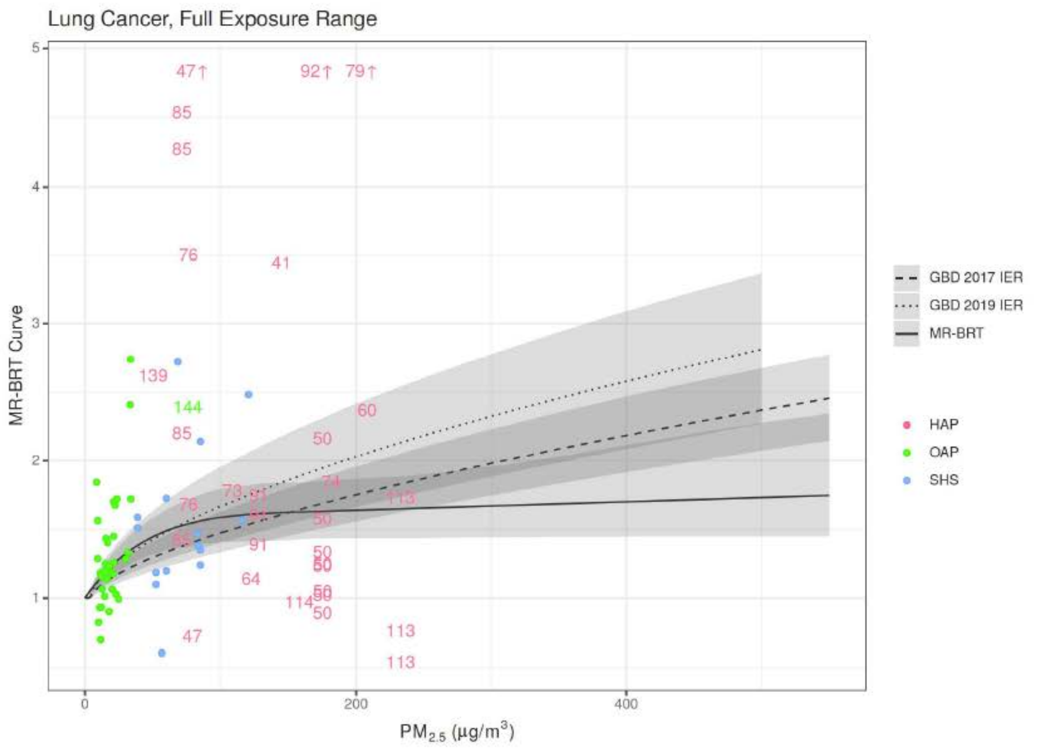
Lung Cancer, Full Exposure Range
MR-BRT Curve
PM2.5 (µg/m3)
GBD 2017 IER
GBD 2019 IER
MR-BRT
HAP
OAP
SHS

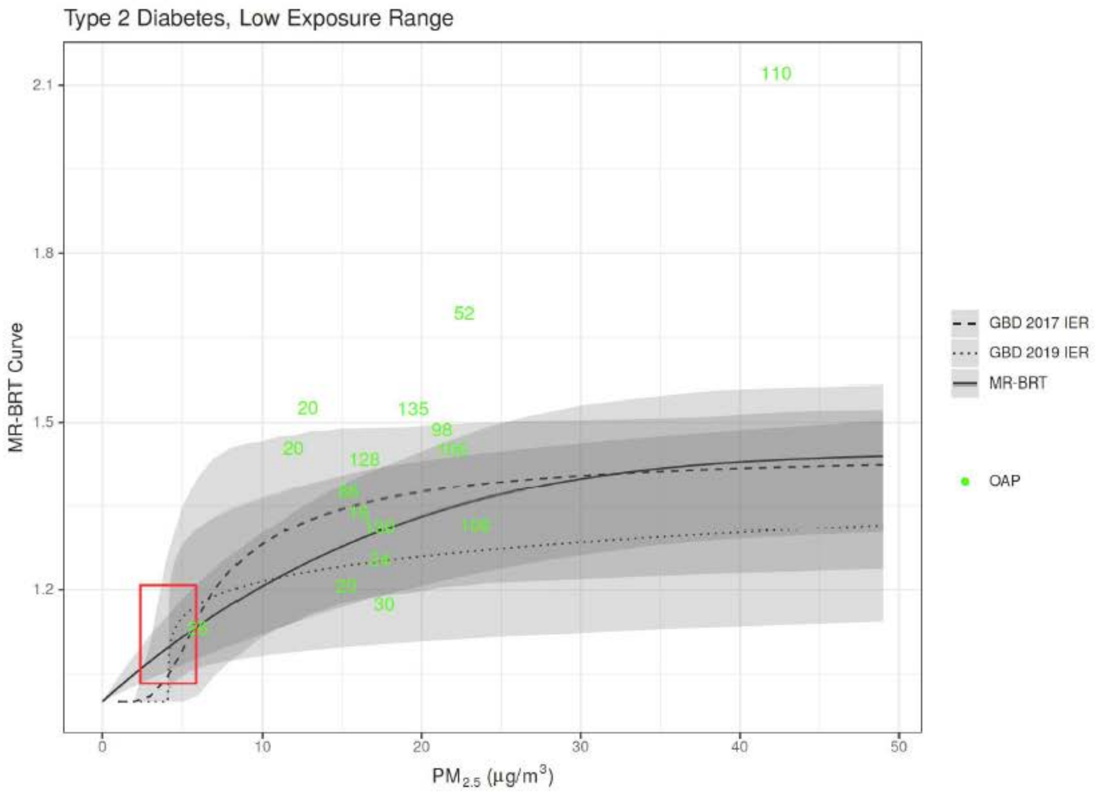
Type 2 Diabetes, Low Exposure Range
MR-BRT Curve
PM2.5 (μg/m³)
GBD 2017 IER
GBD 2019 IER
MR-BRT
OAP

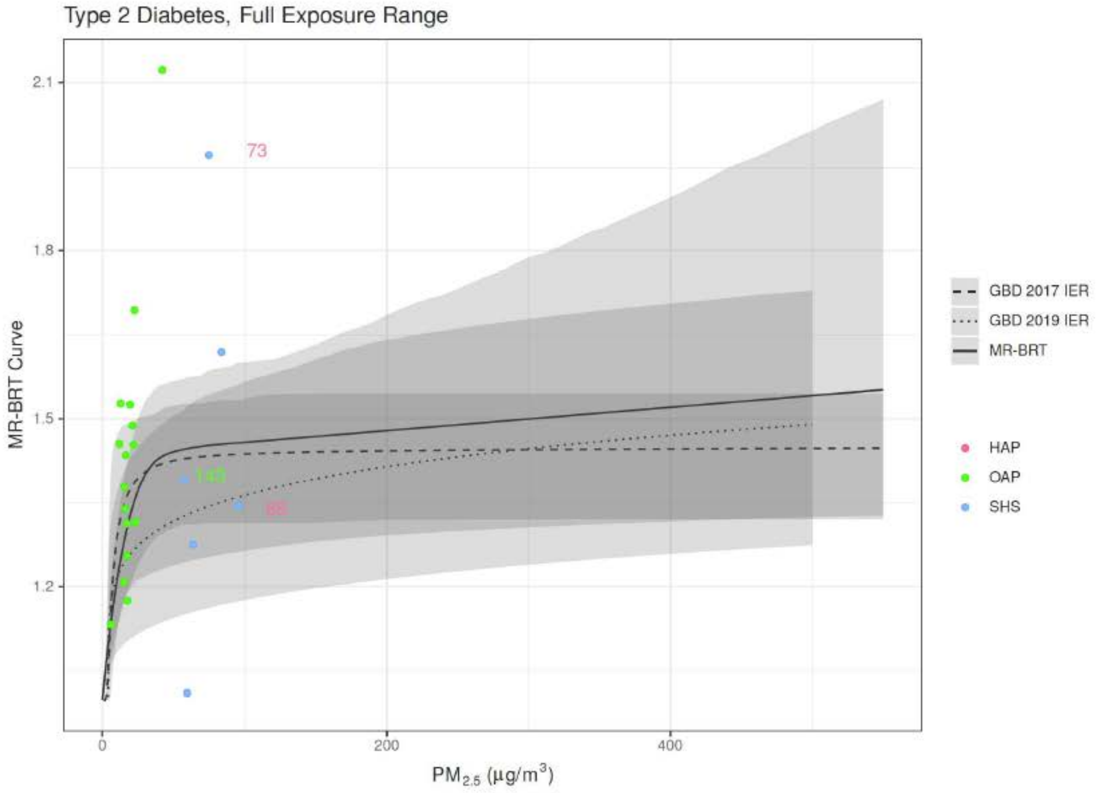
Type 2 Diabetes, Full Exposure Range
MR-BRT Curve
PM2.5 (μg/m³)
GBD 2017 IER
GBD 2019 IER
MR-BRT
HAP
OAP
SHS

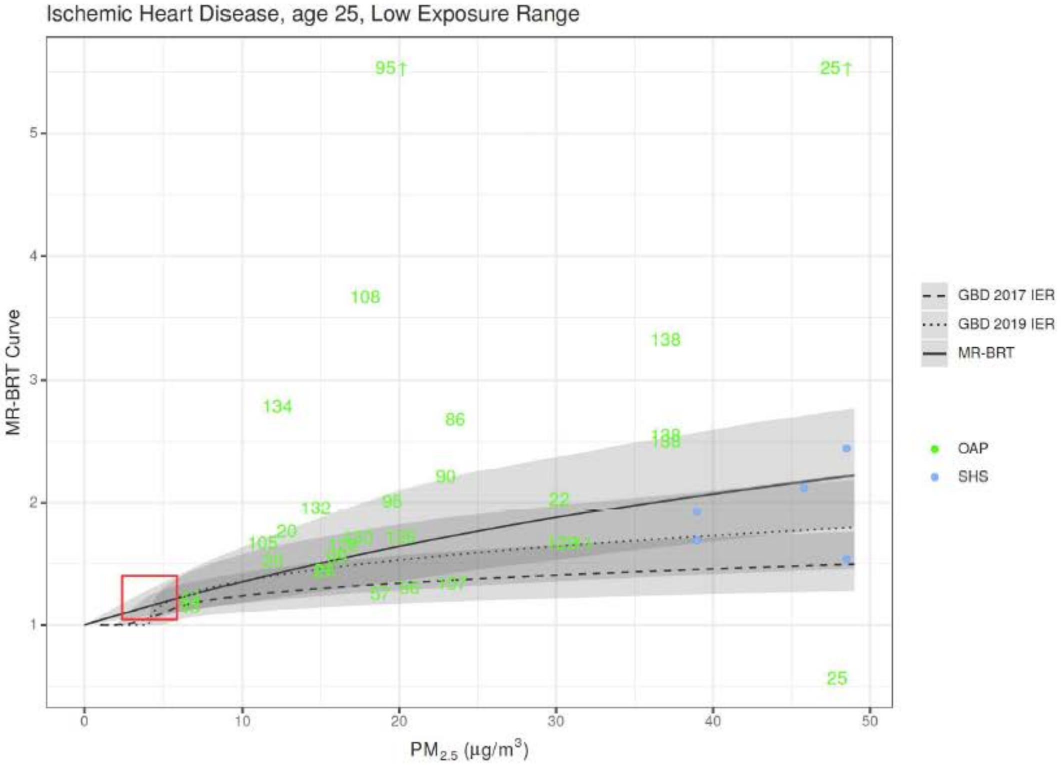
Ischemic Heart Disease, age 25, Low Exposure Range
MR-BRT Curve
PM2.5 (μg/m3)
GBD 2017 IER
GBD 2019 IER
MR-BRT
OAP
SHS

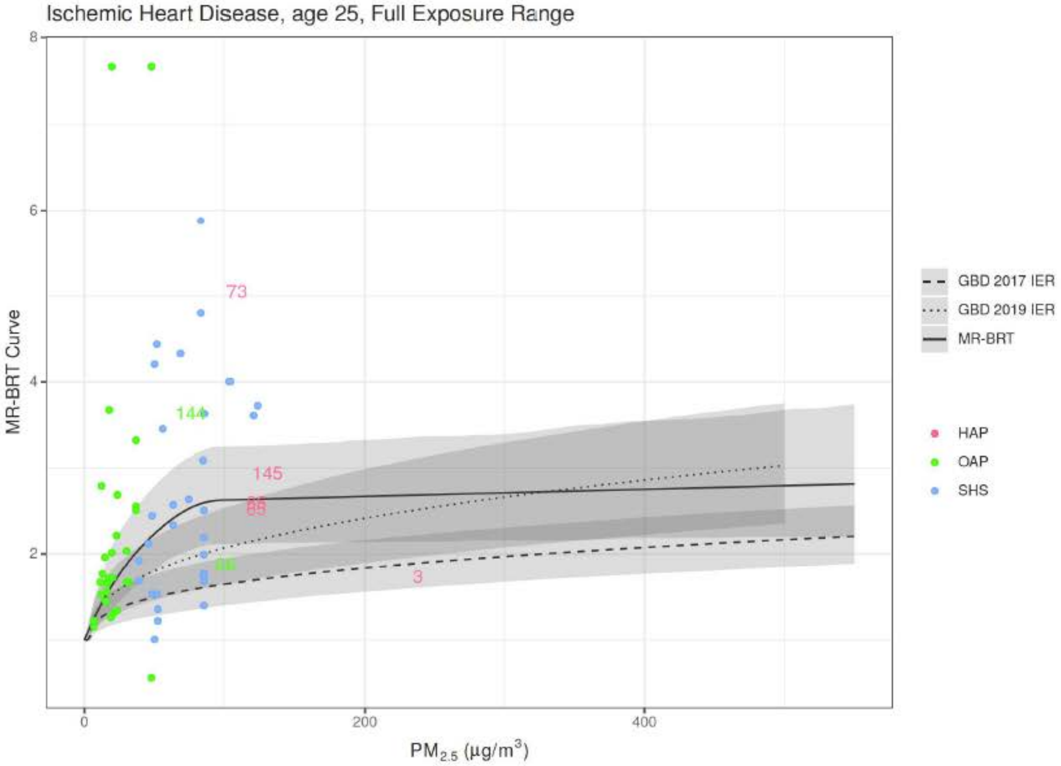
Ischemic Heart Disease, age 25, Full Exposure Range
MR-BRT Curve
PM2.5 (μg/m3)
GBD 2017 IER
GBD 2019 IER
MR-BRT
HAP
OAP
SHS

Ischemic Heart Disease, age 50, Low Exposure Range

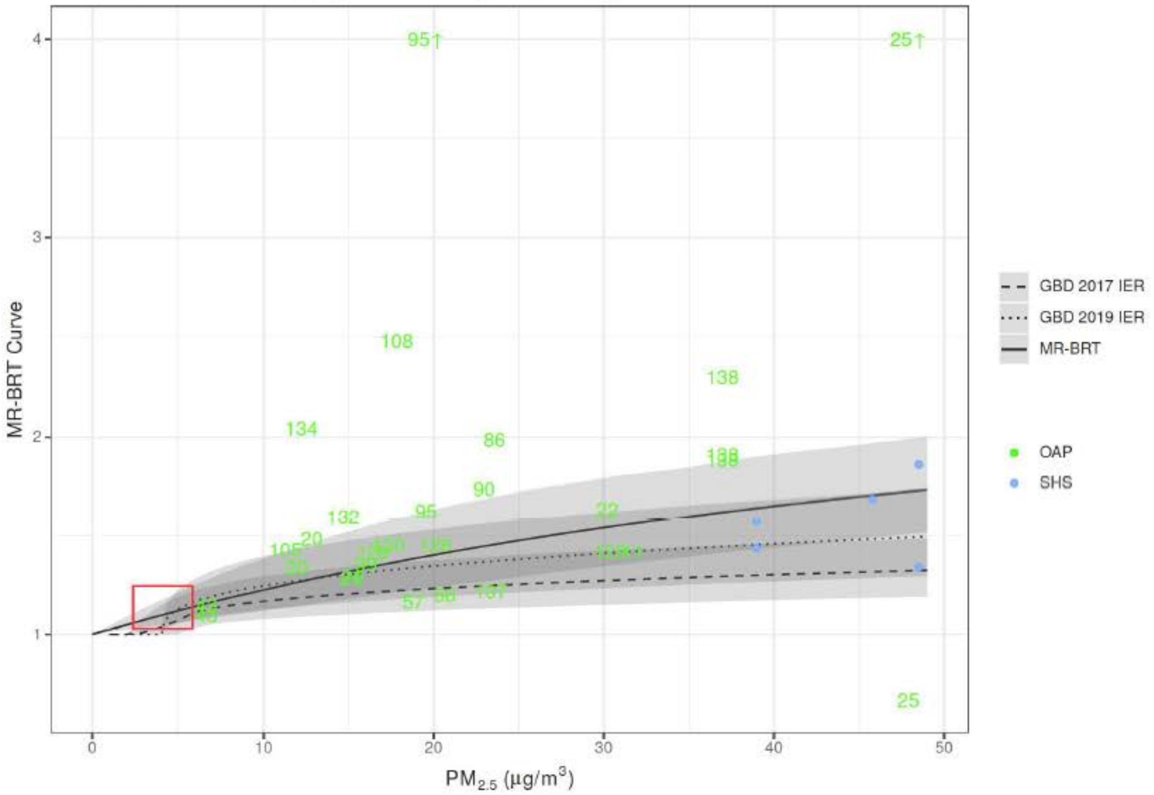

Ischemic Heart Disease, age 50, Full Exposure Range

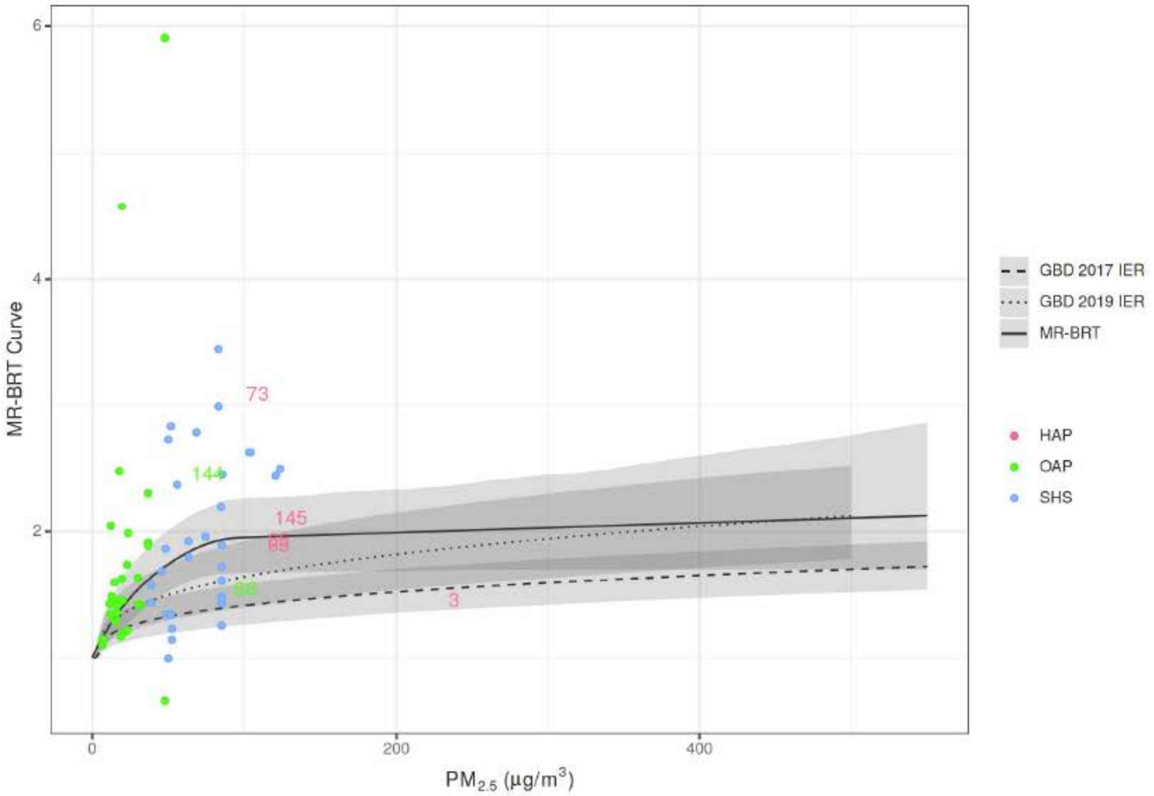

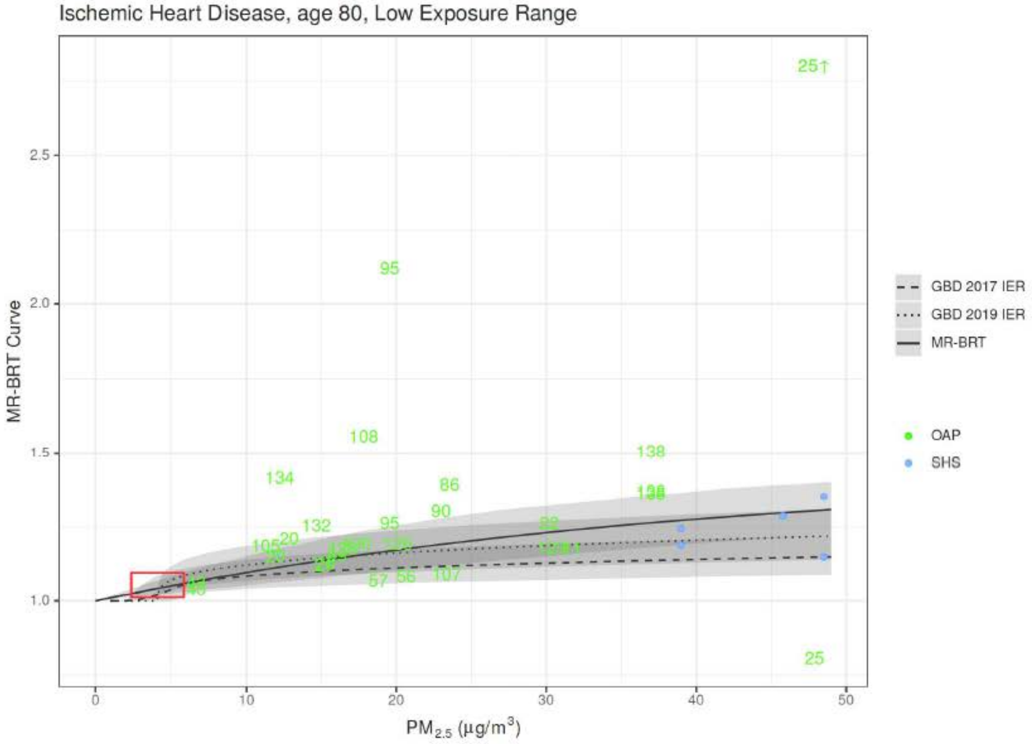
Ischemic Heart Disease, age 80, Low Exposure Range
MR-BRT Curve
PM2.5 (µg/m3)
GBD 2017 IER
GBD 2019 IER
MR-BRT
OAP
SHS

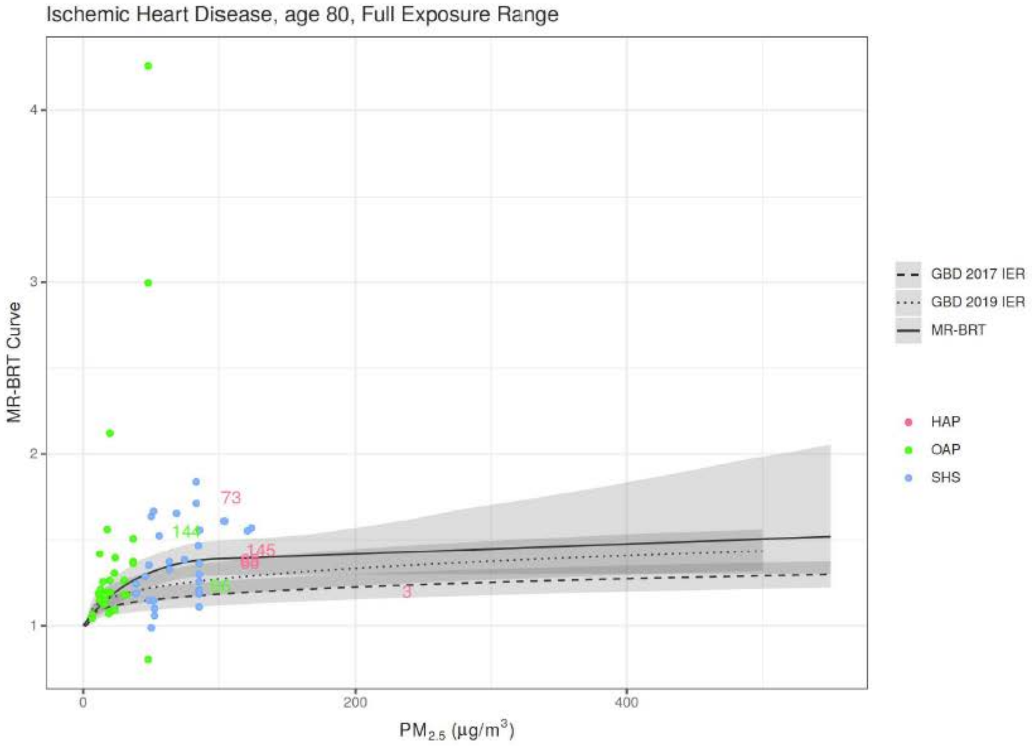
Ischemic Heart Disease, age 80, Full Exposure Range
MR-BRT Curve
PM2.5 (µg/m3)
GBD 2017 IER
GBD 2019 IER
MR-BRT
HAP
OAP
SHS

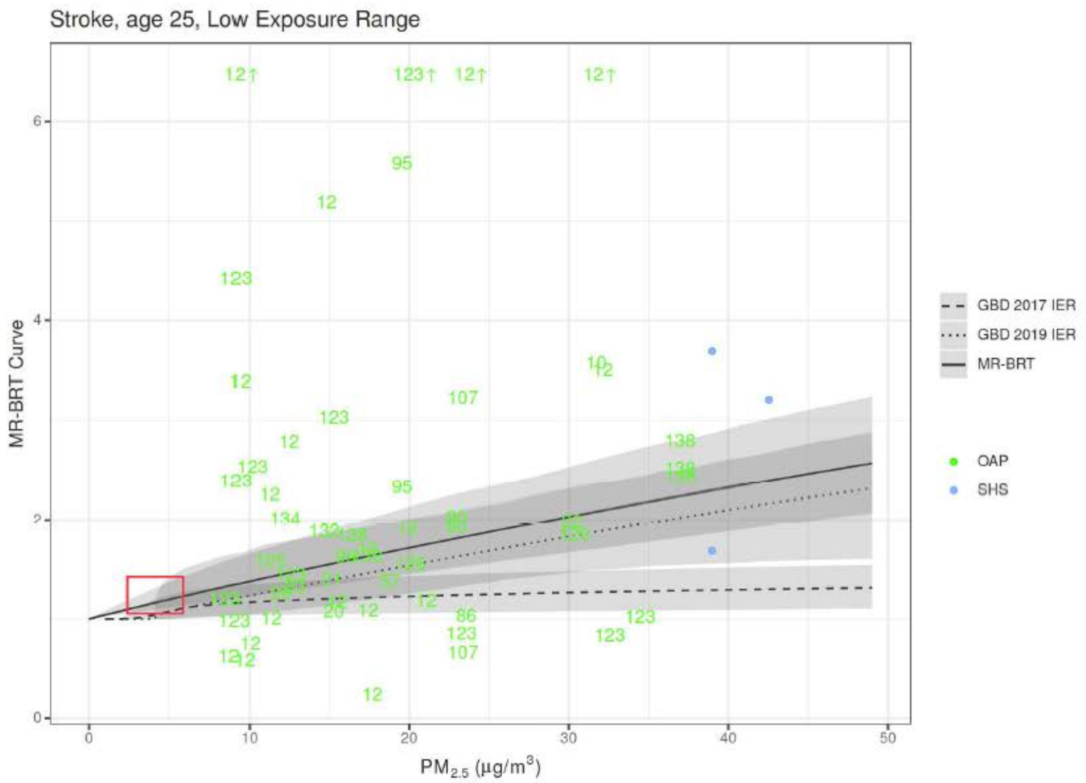
Stroke, age 25, Low Exposure Range
MR-BRT Curve
PM2.5 (µg/m3)
GBD 2017 IER
GBD 2019 IER
MR-BRT
OAP
SHS

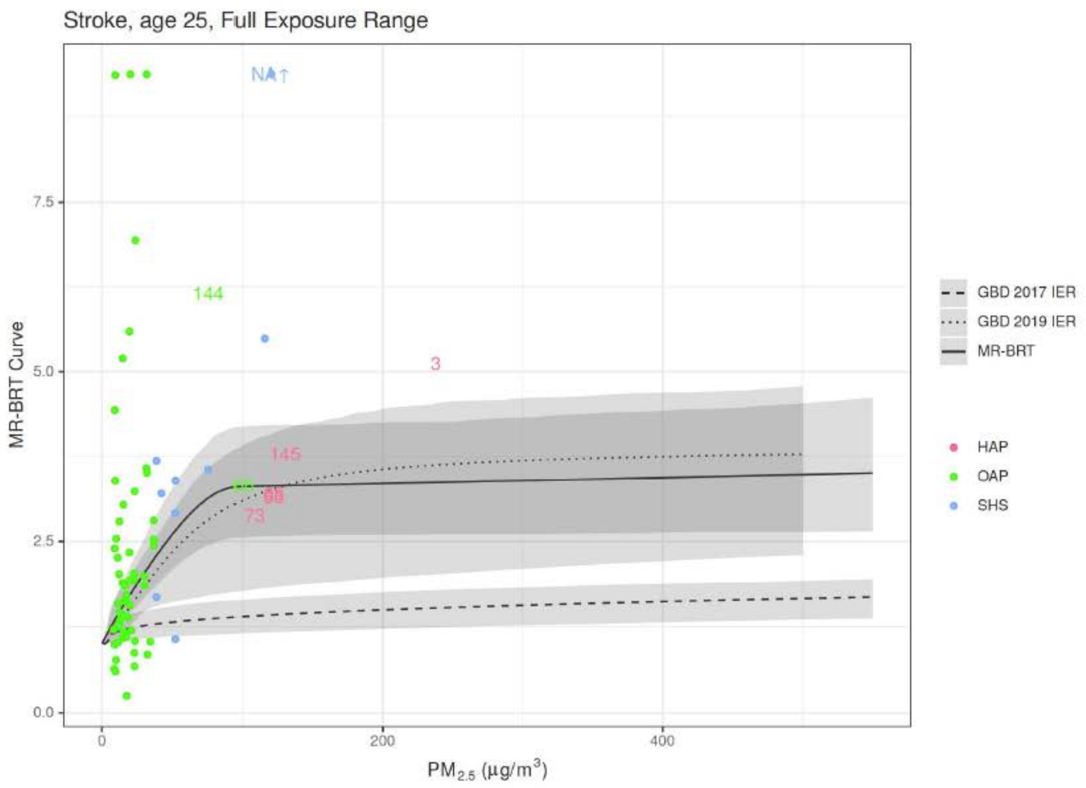
Stroke, age 25, Full Exposure Range
MR-BRT Curve
PM2.5 (µg/m3)
GBD 2017 IER
GBD 2019 IER
MR-BRT
HAP
OAP
SHS

Stroke, age 50, Low Exposure Range

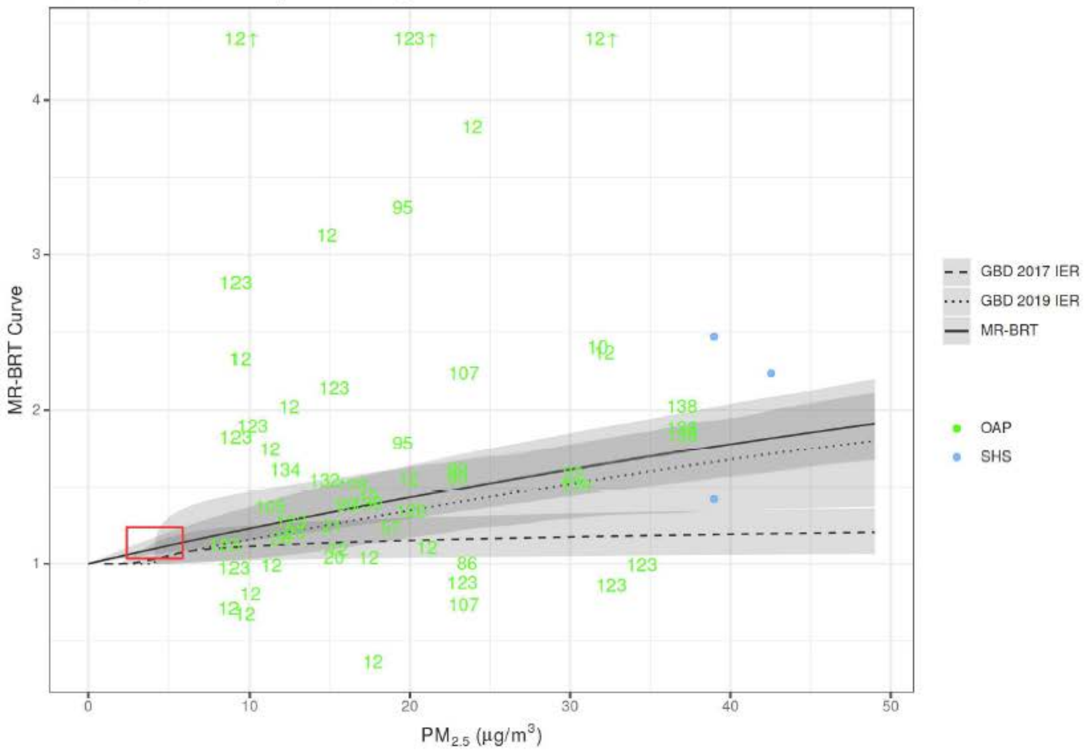

Stroke, age 50, Full Exposure Range

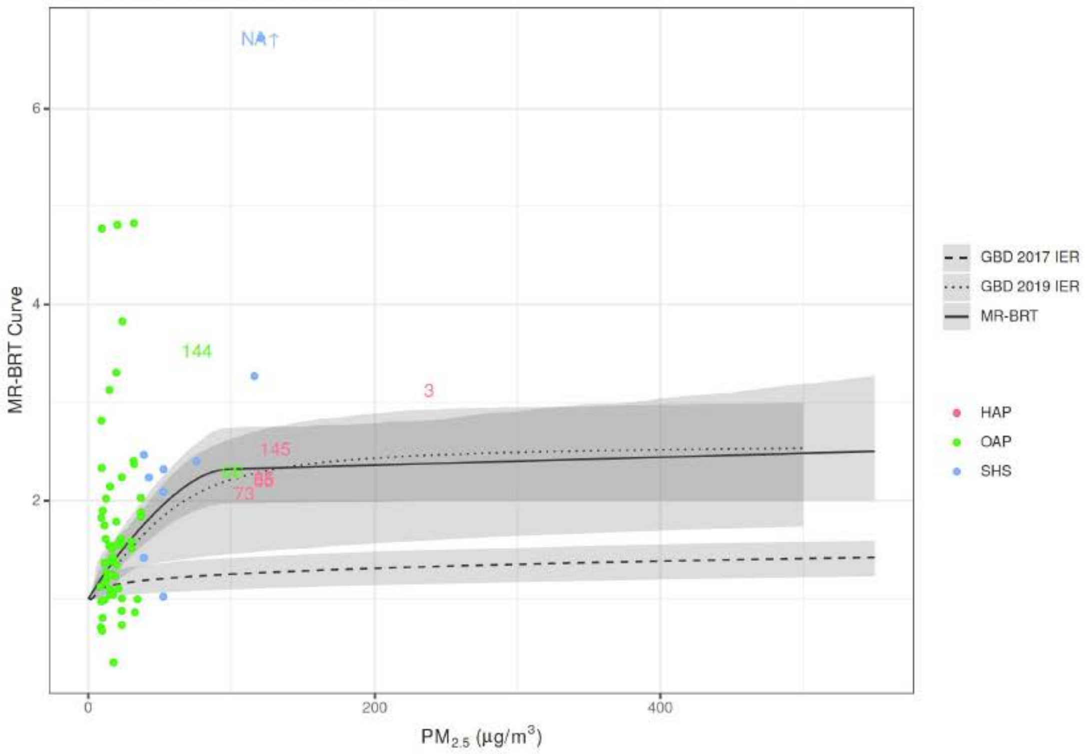

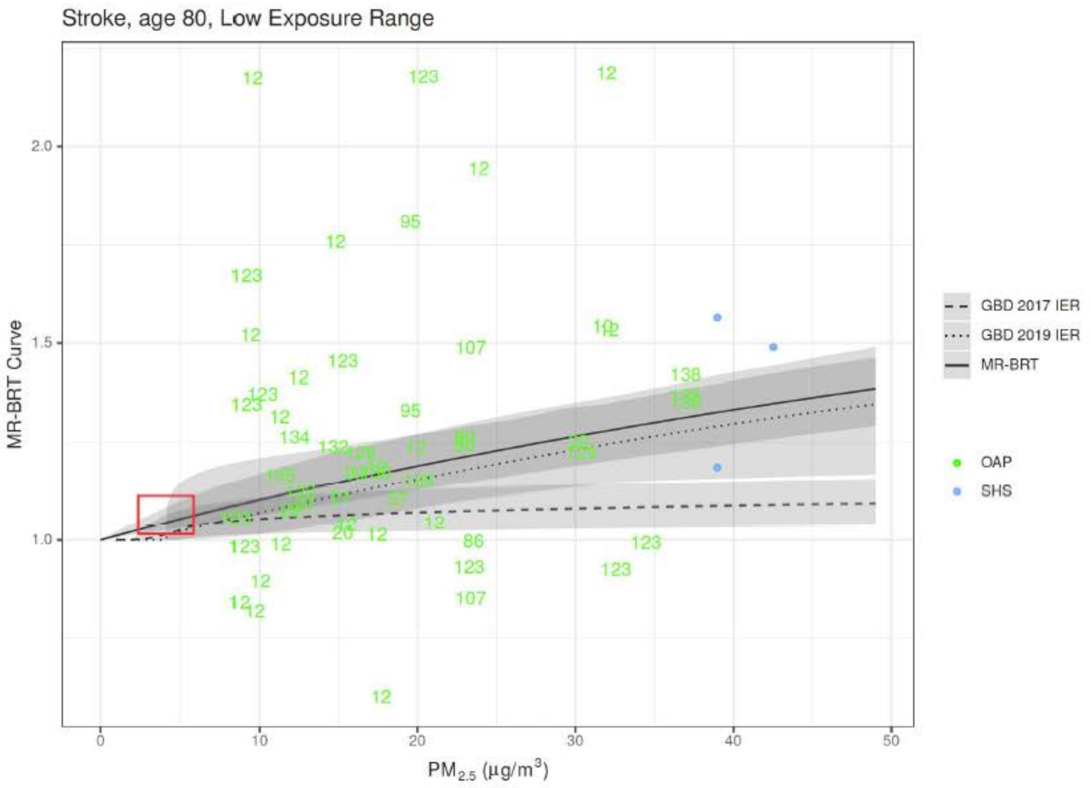

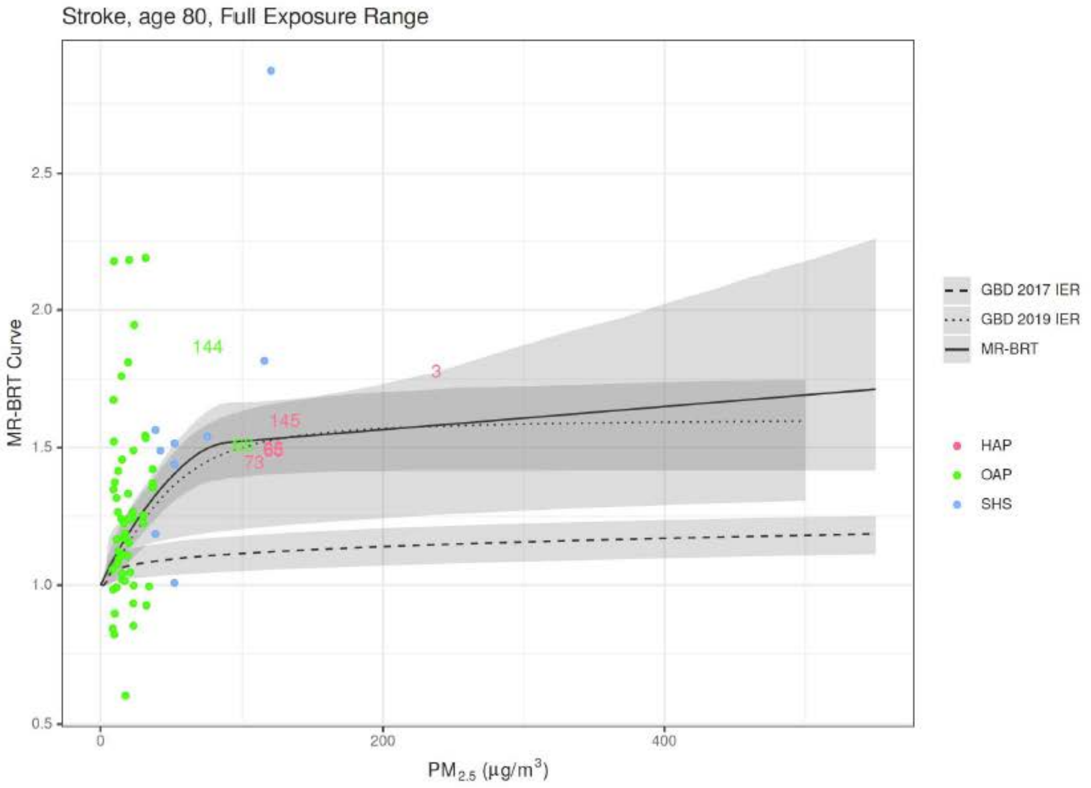

Low birthweight and short gestation mediation analysis

The outcomes of low birthweight and short gestation include mortality due to diarrhoeal diseases, lower respiratory infections, upper respiratory infections, otitis media, meningitis, encephalitis, neonatal preterm birth, neonatal encephalopathy due to birth asphyxia and trauma, neonatal sepsis

and other neonatal infections, haemolytic disease and other neonatal jaundice, and other neonatal disorders. We also calculate attributable YLDs for neonatal preterm birth. These are specific to ages 0-6 days and 7-27 days.

In partnership with Dr. Rakesh Ghosh at the University of California, San Francisco, we conducted a systematic review of all cohort, case-control, or randomised-controlled trial studies of ambient $PM_{2.5}$ pollution or household air pollution and birthweight or gestational age outcomes. Outcomes measured included continuous birthweight (bw), continuous gestational age (ga), low birthweight (LBW) (<2500 g), preterm birth (PTB) (<37 weeks), and very preterm birth (VPTB) (<32 weeks). We included any papers published until March 31, 2018. Systematic review PRISMA diagrams are below.

Ambient particulate matter pollution, low birth weight

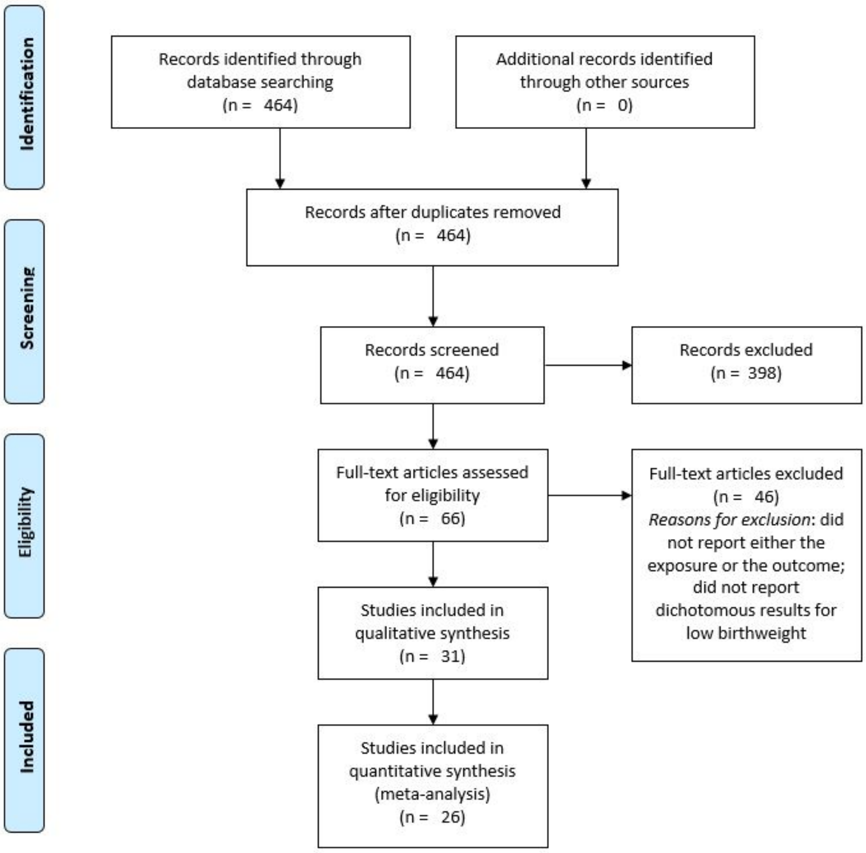

Ambient particulate matter pollution, preterm birth

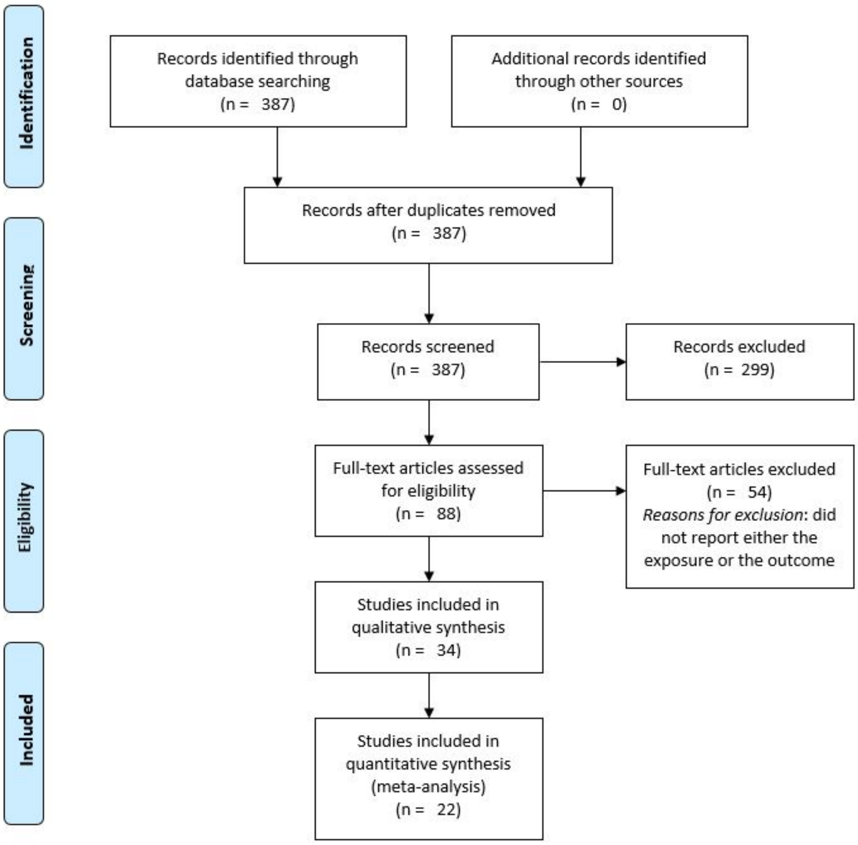

Household air pollution, all outcomes

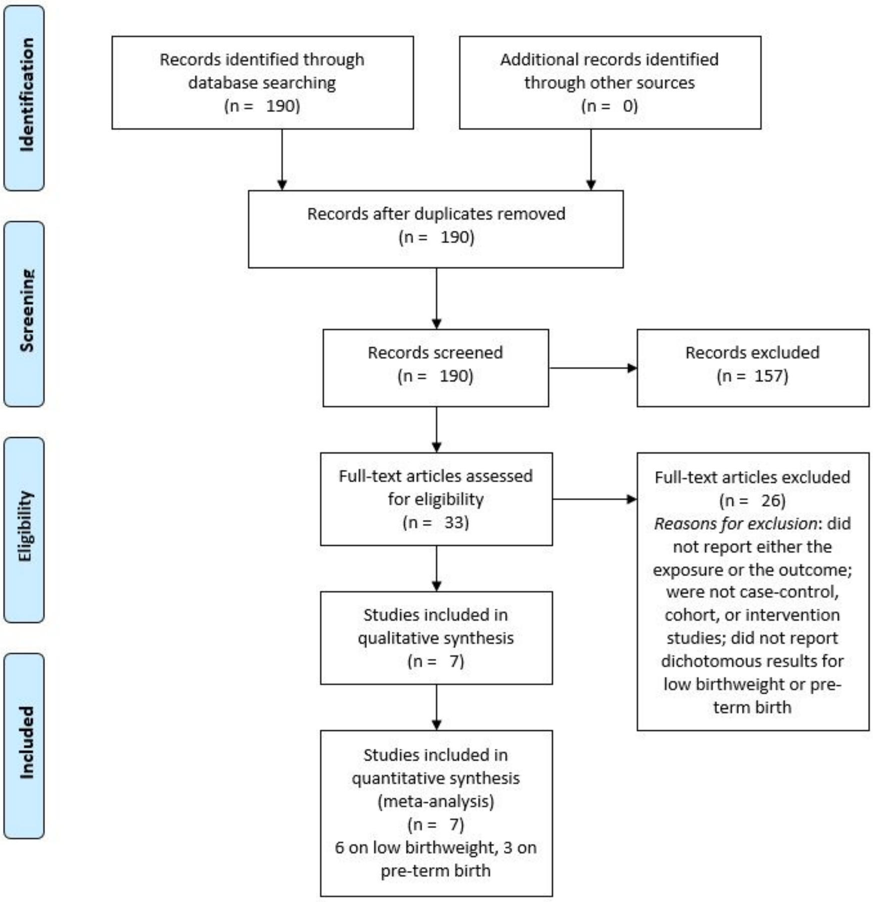

The following plots depict forest and funnel plots for studies of OAP and birthweight, low birthweight, and preterm birth. Note that these plots do not capture the exposure level of these studies but the linear risk or difference in birthweight per 10-unit increase in $PM_{2.5}$ exposure.

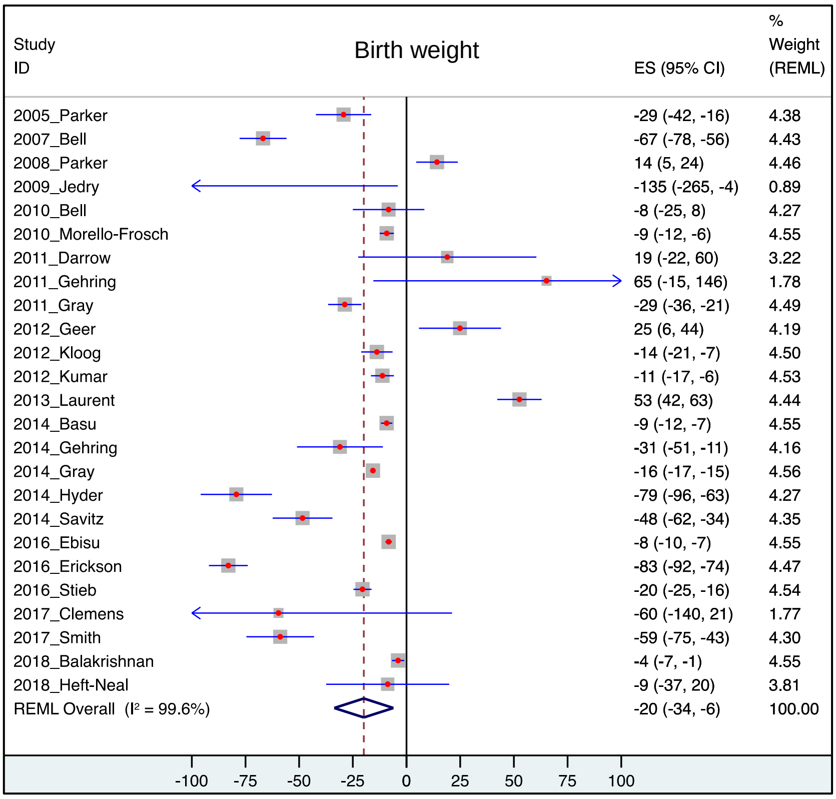
Study
ID
Birth weight
ES (95% CI)
%
Weight
(REML)
2005_Parker -29 (-42, -16) 4.38
2007_Bell -67 (-78, -56) 4.43
2008_Parker 14 (5, 24) 4.46
2009_Jedry -135 (-265, -4) 0.89
2010_Bell -8 (-25, 8) 4.27
2010_Morello-Frosch -9 (-12, -6) 4.55
2011_Darrow 19 (-22, 60) 3.22
2011_Gehring 65 (-15, 146) 1.78
2011_Gray -29 (-36, -21) 4.49
2012_Geer 25 (6, 44) 4.19
2012_Kloog -14 (-21, -7) 4.50
2012_Kumar -11 (-17, -6) 4.53
2013_Laurent 53 (42, 63) 4.44
2014_Basu -9 (-12, -7) 4.55
2014_Gehring -31 (-51, -11) 4.16
2014_Gray -16 (-17, -15) 4.56
2014_Hyder -79 (-96, -63) 4.27
2014_Savitz -48 (-62, -34) 4.35
2016_Ebisu -8 (-10, -7) 4.55
2016_Erickson -83 (-92, -74) 4.47
2016_Stieb -20 (-25, -16) 4.54
2017_Clemens -60 (-140, 21) 1.77
2017_Smith -59 (-75, -43) 4.30
2018_Balakrishnan -4 (-7, -1) 4.55
2018_Heft-Neal -9 (-37, 20) 3.81
REML Overall (I² = 99.6%) -20 (-34, -6) 100.00
-100 -75 -50 -25 0 25 50 75 100

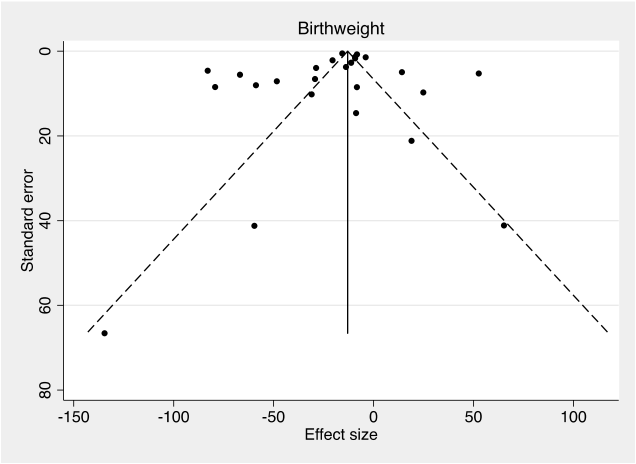
Birthweight
Standard error
0 20 40 60 80
-150 -100 -50 0 50 100
Effect size

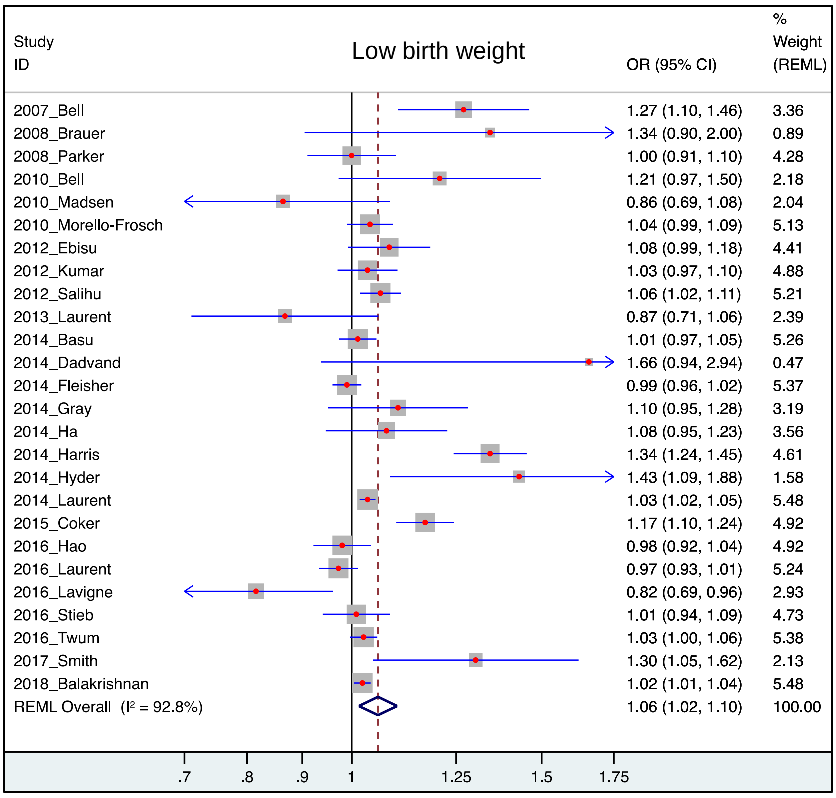
Low birth weight
Study ID
OR (95% CI)
% Weight (REML)
2007_Bell 1.27 (1.10, 1.46) 3.36
2008_Brauer 1.34 (0.90, 2.00) 0.89
2008_Parker 1.00 (0.91, 1.10) 4.28
2010_Bell 1.21 (0.97, 1.50) 2.18
2010_Madsen 0.86 (0.69, 1.08) 2.04
2010_Morello-Frosch 1.04 (0.99, 1.09) 5.13
2012_Ebisu 1.08 (0.99, 1.18) 4.41
2012_Kumar 1.03 (0.97, 1.10) 4.88
2012_Salihu 1.06 (1.02, 1.11) 5.21
2013_Laurent 0.87 (0.71, 1.06) 2.39
2014_Basu 1.01 (0.97, 1.05) 5.26
2014_Dadvand 1.66 (0.94, 2.94) 0.47
2014_Fleisher 0.99 (0.96, 1.02) 5.37
2014_Gray 1.10 (0.95, 1.28) 3.19
2014_Ha 1.08 (0.95, 1.23) 3.56
2014_Harris 1.34 (1.24, 1.45) 4.61
2014_Hyder 1.43 (1.09, 1.88) 1.58
2014_Laurent 1.03 (1.02, 1.05) 5.48
2015_Coker 1.17 (1.10, 1.24) 4.92
2016_Hao 0.98 (0.92, 1.04) 4.92
2016_Laurent 0.97 (0.93, 1.01) 5.24
2016_Lavigne 0.82 (0.69, 0.96) 2.93
2016_Stieb 1.01 (0.94, 1.09) 4.73
2016_Twum 1.03 (1.00, 1.06) 5.38
2017_Smith 1.30 (1.05, 1.62) 2.13
2018_Balakrishnan 1.02 (1.01, 1.04) 5.48
REML Overall (I² = 92.8%) 1.06 (1.02, 1.10) 100.00
.7 .8 .9 1 1.25 1.5 1.75

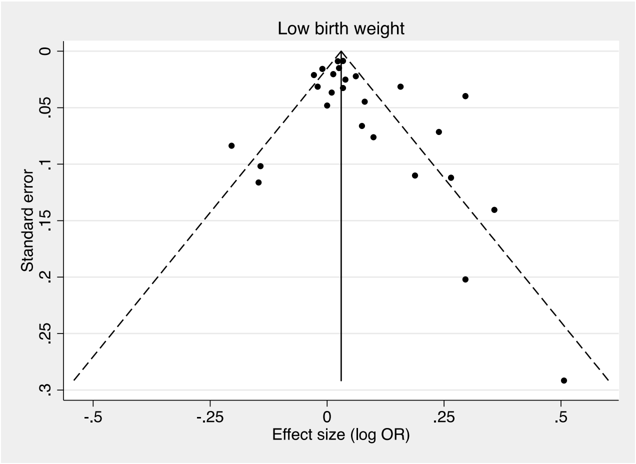
Low birth weight
Standard error
0 .05 .1 .15 .2 .25 .3
-.5 -.25 0 .25 .5
Effect size (log OR)

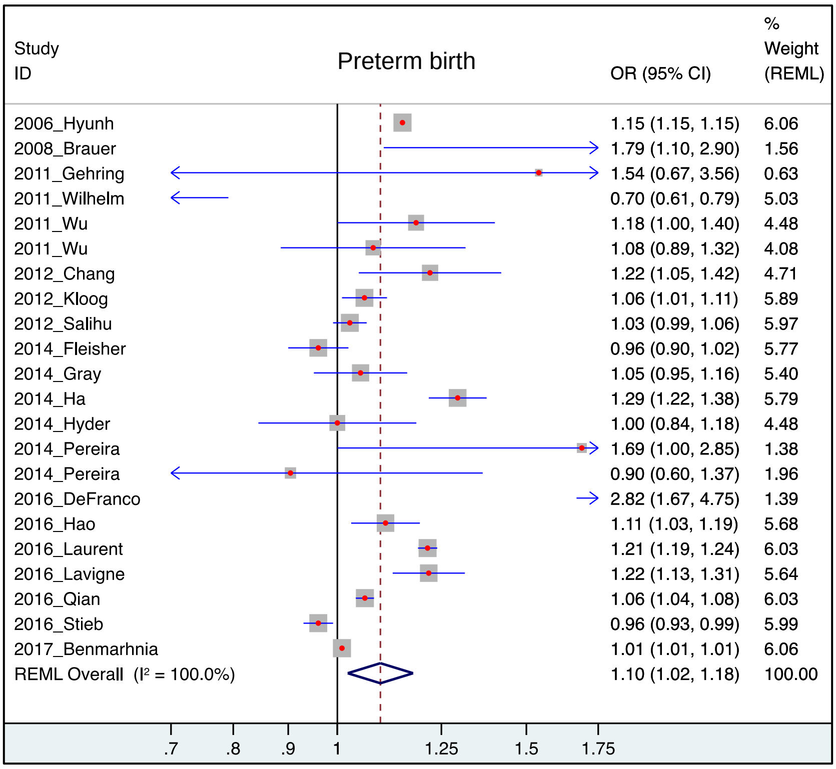

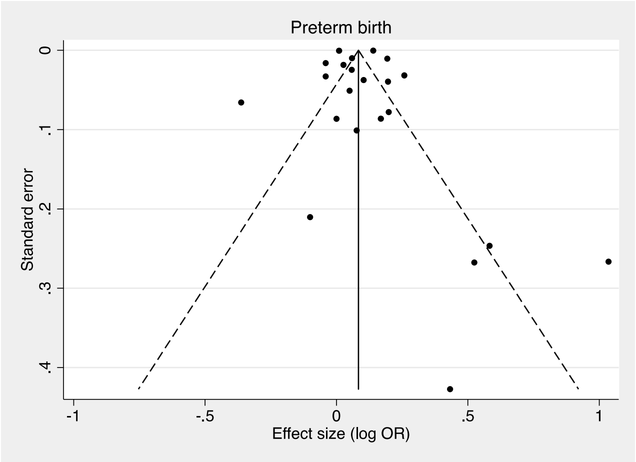

For studies of household air pollution, we used the same strategy described above to map them to $PM_{2.5}$ exposure values.

Because birthweight and gestational age are modelled using a continuous joint distribution for the GBD, we were interested in how those distributions changed under the influence of $PM_{2.5}$ pollution. We therefore estimated the continuous shift in birthweight (bw, in grams) and gestational age (ga, in weeks) at a given $PM_{2.5}$ exposure level.

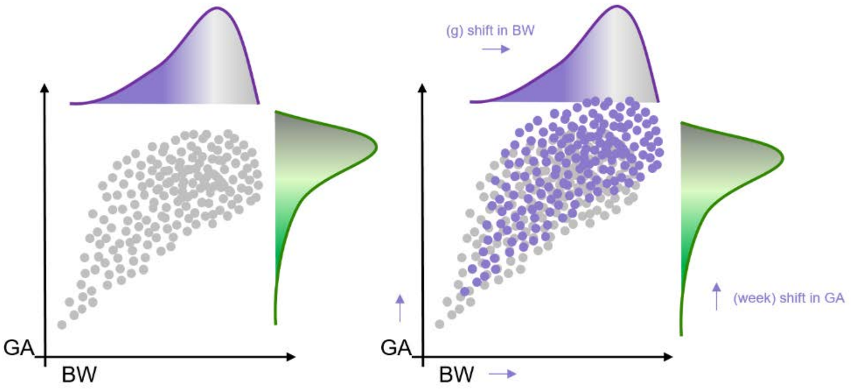

When available, we used estimates of continuous shift in bw or ga directly from each study. When that was not available, we used the published OR/RR/HR for LBW, PTB, or VPTB and the following strategy:

1. Extract the OR/RR/HR from the study.
2. Select the GBD 2017 estimated bw-ga joint distribution for the study location and year.
3. Calculate the number of grams or weeks required to shift the distribution such that the proportion of births under the specified threshold (P) is reduced by the study effect size to a counterfactual level (P_{cf}).
4. Save the resulting shift and 95% CI as the continuous effect.

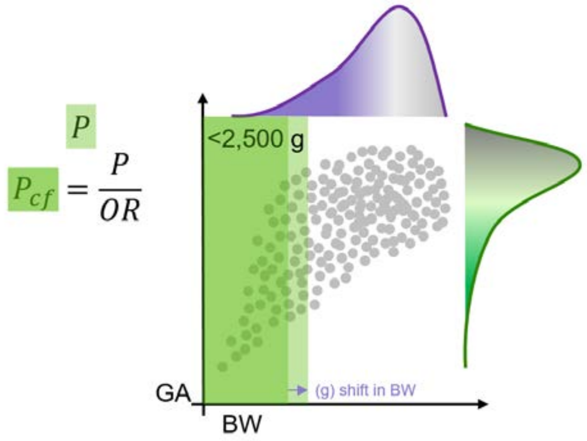

We then fit a MR-BRT spline to these studies, where the difference in the value of the model at the upper concentration (X) and the value of the model at the counterfactual concentration (X_{CF}) is equal to the published or calculated shift in bw or ga. We fit the same model and priors as the non-mediated outcomes (with the exception of COPD), except, because the change in birthweight and gestational age was expected to be negative, the shape constraints were monotonically decreasing and concave up.

$$MRBRT(X) - MRBRT(X_{CF}) \sim Shift$$

The following figures depict the MR-BRT curves for shift in grams (bw) and weeks (ga).

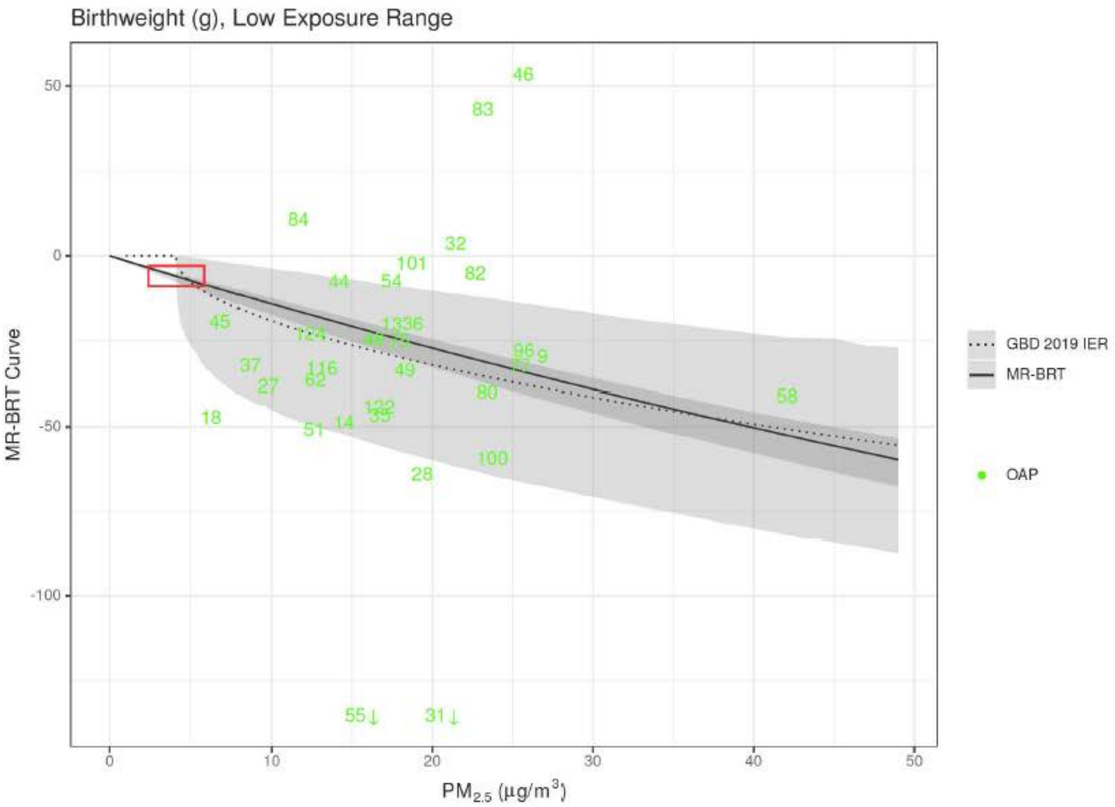
Birthweight (g), Low Exposure Range
MR-BRT Curve
PM2.5 (µg/m3)
GBD 2019 IER
MR-BRT
OAP

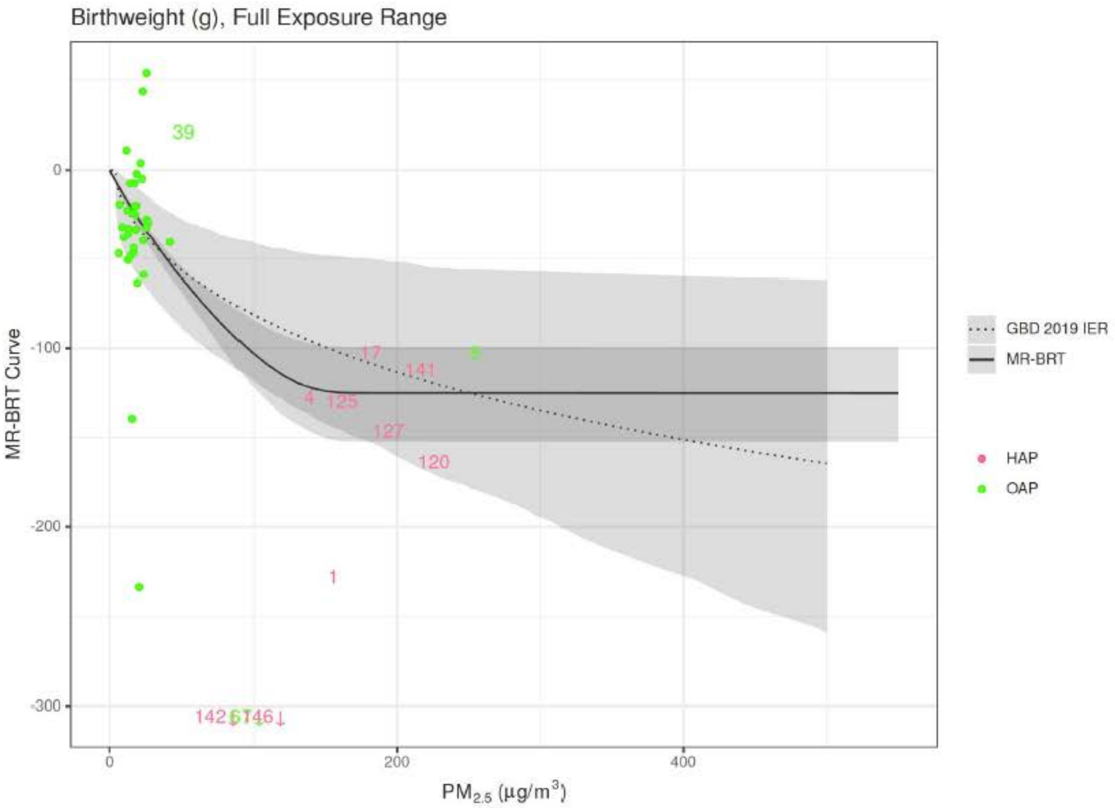
Birthweight (g), Full Exposure Range
MR-BRT Curve
PM2.5 (µg/m3)
GBD 2019 IER
MR-BRT
HAP
OAP

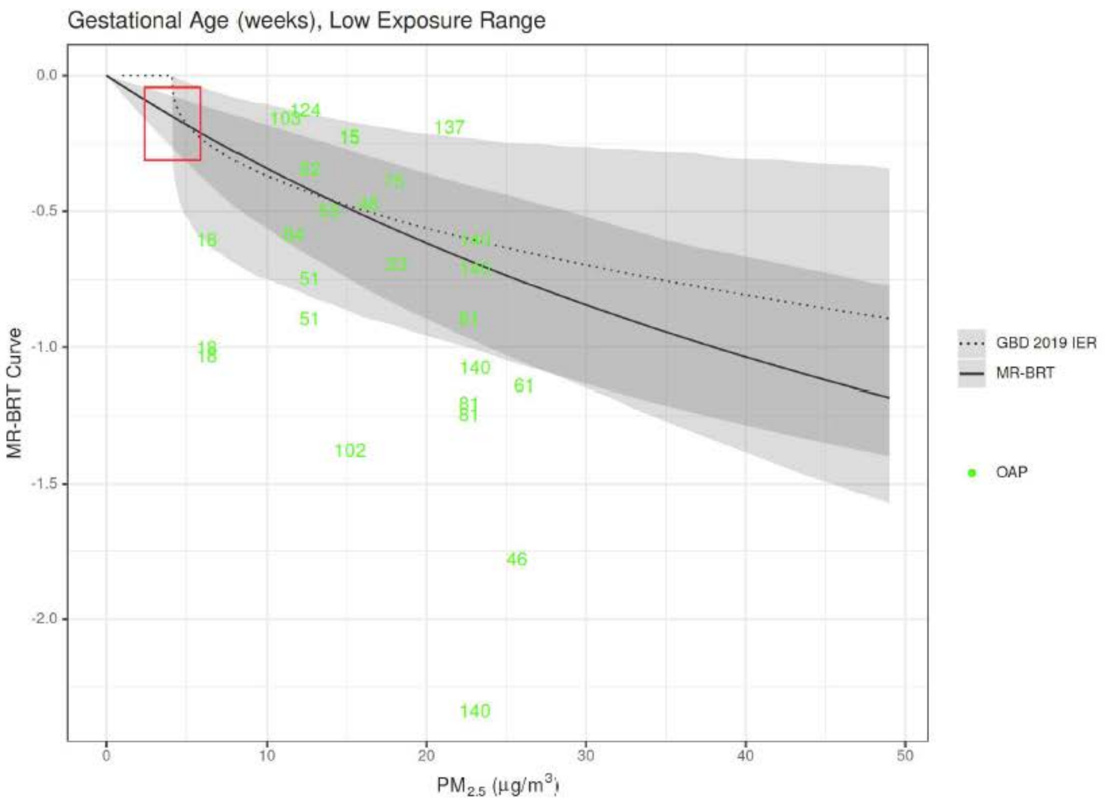

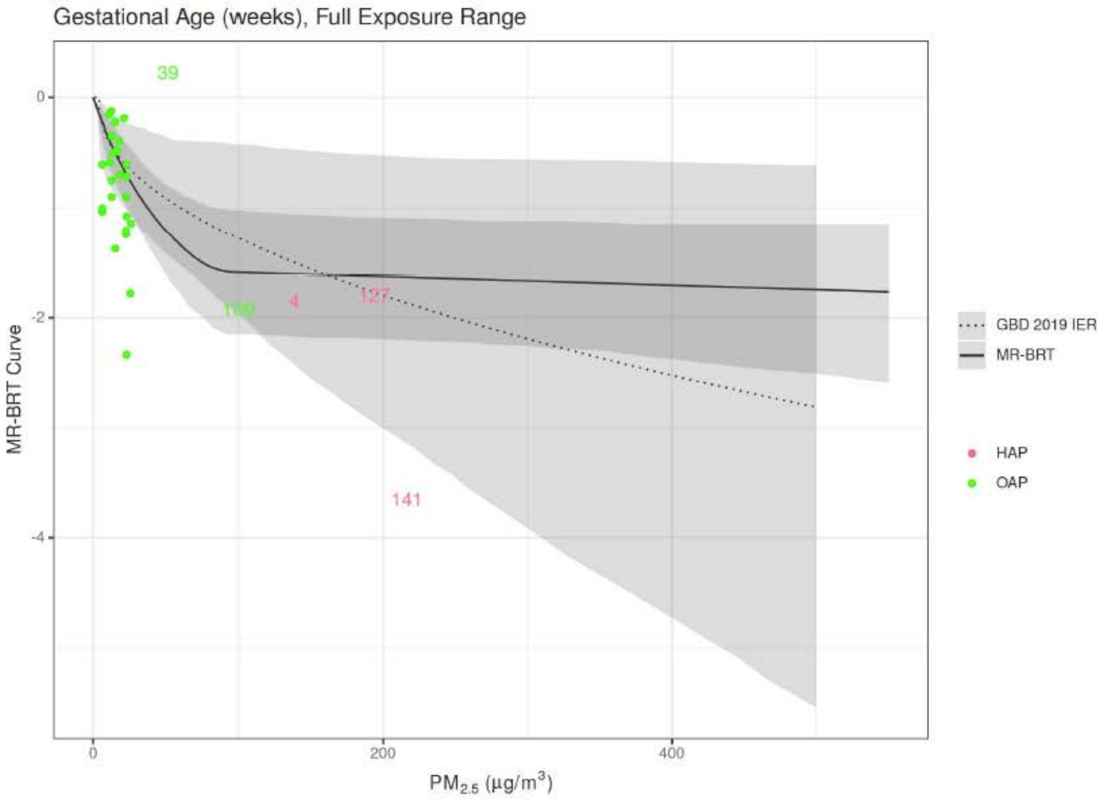

Once we had curves of estimated shifts across the exposure range, we predicted the shift in both birthweight and gestational age for total female particulate matter pollution exposure in each location and year. Because the epidemiological studies mutually controlled for birthweight and gestational age, we assumed these shifts are independent. We then shifted the observed

distributions to reflect the expected bwga distribution in the absence of particulate matter pollution. These shifted distributions were used as the counterfactual in the PAF calculation equation to calculate the burden attributable to $PM_{2.5}$ pollution.

To calculate PAFs, the distribution is divided into 56 bw-ga categories, each with a unique RR. Let p_i be the observed proportion of babies in category, *i* and p_i' be the counterfactual proportion of babies in category, *i* if there were no particulate matter pollution.

$$PAF_{PM} = \frac{\sum_{i \in bwga\ category} RR_i p_i - \sum_{i \in bwga\ category} RR_i p_i'}{\sum_{i \in bwga} RR_i p_i}$$

We proportionately split this PAF to ambient and HAP based on exposure as is described below. One important assumption to note is that we are assuming the shift in bw and ga is linear across the bwga distribution.

For lower respiratory infections, we have directly estimated PAFs attributable to $PM_{2.5}$ in addition to those mediated through birthweight and gestational age. We would expect that some of the directly estimated PAFs are mediated through bw and ga. Additionally, the directly estimated PAF is based on a summary of relative risks for all children under 5 years, so there is a chance that the mediated PAF, which is more finely resolved, could be greater. To avoid double-counting for these two age groups (0-6 days and 0-27 days), we take the max of the two PAF estimates. If the directly estimated PAF is greater than the bw-ga-mediated PAF, we take the direct estimate, and if the mediated PAF is greater, we take the mediated.

PTB incidence and mortality are both outcomes measured in the GBD. 100% of the burden for this cause is attributable to short gestation. To calculate the percentage attributable to particulate matter pollution, we estimated the percentage of babies born at less than 37 weeks (p_{ptb}) and the percentage of babies that would have been born at less than 37 weeks in the counterfactual scenario of no particulate matter pollution (p_{ptb}').

$$PAF_{ptb,pm} = 1 - \frac{p_{ptb}'}{p_{ptb}}$$

Limitations

Although in GBD 2019 we have not used active smoking data to estimate the risk curves, we are still using an integrated exposure response approach because we are integrating relative risk estimates across various exposure sources: ambient, SHS, and HAP. The use of various sources to construct a risk curve with $PM_{2.5}$ as the exposure indicator assumes equitoxicity of particles, despite some evidence suggesting differences in health impact by PM source, size, and chemical composition. However, in the absence of consistent and robust evidence of differential toxicity by source and sufficient estimates of source or composition-specific exposure-response relationships, integrating across OAP, SHS, and HAP studies is the approach most consistent with the current evidence, as reviewed by US EPA and WHO.[20,21] Use of a common risk function may affect the magnitude of risk estimates for HAP and OAP compared to separate risk functions. As more data from higher OAP concentration locations and from HAP studies for non-respiratory outcomes becomes available it may be possible to evaluate the strength of evidence for each and to develop separate risk functions.

Proportional PAF approach

Prior to GBD 2017, relative risks for both exposures were obtained from the IER as a function of exposure and relative to the same TMREL. In reality, were a country to reduce only one of these risk

factors, the other would remain. We did not consider the joint effects of particulate matter from outdoor exposure and burning solid fuels for cooking. For GBD 2017 we developed a new approach to use the IER for obtaining PAFs for both OAP and HAP:

Let Exp_{OAP} be the ambient $PM_{2.5}$ exposure level and Exp_{HAP} be the excess exposure for those who use solid fuel for cooking. Let P_{HAP} be the proportion of the population using solid fuel for cooking. We calculated PAFs at each 0.1°×0.1° grid cell. We assumed that the distribution of those using solid fuel for cooking (HAP) was equivalent across all grid cells of the GBD location.

For the proportion of the population not exposed to HAP the relative risk was:

$$RR_{OAP} = MRBRT(z = Exp_{OAP})/MRBRT(z = TMREL),$$

And for those exposed to HAP, the relative risk was

$$RR_{HAP} = MRBRT(z = Exp_{OAP} + Exp_{HAP})/MRBRT(z = TMREL).$$

We then calculate a population level RR and PAF for all particulate matter exposure.

$$RR_{PM} = RR_{OAP}(1 - P_{HAP}) + RR_{HAP}P_{HAP}$$

$$PAF_{PM} = \frac{RR_{PM} - 1}{RR_{PM}}$$

We population weight the grid-cell level particulate matter PAFs to get a country level PAF, and finally, we split this PAF based on the average exposure to each OAP and HAP.

$PAF_{OAP} = \frac{Exp_{OAP}}{Exp_{OAP}+P_{HAP}*Exp_{HAP}} PAF_{PM}$, and $PAF_{HAP} = \frac{P_{HAP}*Exp_{HAP}}{Exp_{OAP}+P_{HAP}*Exp_{HAP}} PAF_{PM}$.

With this strategy, $PAF_{PM} = PAF_{HAP} + PAF_{OAP}$, and no burden is counted twice.

References

1. Hammer, M. S., A. van Donkelaar, R. V. Martin, C. Li, A. Lyapustin, A. M. Sayer, C. N. Hsu, R. C. Levy, M. J. Garay, O. V. Kalashnikova, R. A. Kahn, M. Brauer, J. S. Apte, D. K. Henze, L. Zhang, and Q. Zhang (submitted), Improved Global Estimates of Fine Particulate Matter Concentrations and Trends Derived from Updated Satellite Retrievals, Modeling Advances, and Additional Ground-Based Monitors, *Environ. Sci. Technol*.
2. van Donkelaar, A.; Martin, R. V; Brauer, M.; Hsu, N. C.; Kahn, R. A.; Levy, R. C.; Lyapustin, A.; Sayer, A. M.; Winker, D. M. Global Estimates of Fine Particulate Matter using a Combined Geophysical-Statistical Method with Information from Satellites, Models, and Monitors. Environ. Sci. Technol. 2016, 50 (7), 3762–3772
3. Shaddick, G., Thomas, M.L., Jobling, A., Brauer, M., van Donkelaar, A., Burnett, R., Chang, H., Cohen, A., Van Dingenen, R., Dora, C. and Gumy, S., 2016. Data Integration Model for Air Quality: A Hierarchical Approach to the Global Estimation of Exposures to Ambient Air Pollution. Journal of Royal Statistical Society Series C (Applied Statistics). 2017. DOI: 10.1111/rssc.12227
4. Shaddick, G., Thomas, M. L., Mudu, P., Ruggeri, G. and Gumy, S. Half the world's population are exposed to increasing air pollution. Accepted by Nature Climate and Atmospheric Science.
5. Brauer, M.; Freedman, G.; Frostad, J.; van Donkelaar, A.; Martin, R. V; Dentener, F.; Van Dingenen, R.; Estep, K.; Amini, H.; Apte, J. S.; et al. Ambient Air Pollution Exposure Estimation for the Global Burden of Disease 2013. Environ. Sci. Technol. 2015, 50 (1), 79–88.

6. Shaddick G, Thomas M, Amini H, Broday DM, Cohen A, Frostad J, Green A, Gumy S, Liu Y, Martin RV, Prüss-Üstün A, Simpson D, van Donkelaar A, Brauer M. Data integration for the assessment of population exposure to ambient air pollution for global burden of disease assessment. Environ Sci Technol. 2018 Jun 29. doi: 10.1021/acs.est.8b02864
7. Rue, H.; Martino, S.; Chopin, N.; Approximate Bayesian inference for latent Gaussian models by using integrated nested Laplace approximations. Journal of the royal statistical society: Series b (statistical methodology). 2009;71(2):319-92.
8. Thomas, M. L., Shaddick, G., Simpson, D., de Hoogh, K. and Zidek, J. V. Spatio-temporal downscaling for continental–scale estimation of air pollution concentrations. arXiv preprint arXiv:1907.00093 (also been Submitted to the Journal of the Royal Statistical Society: Series C (Applied Statistics)).
9. Wood, S. N. (2017). Generalized additive models: an introduction with R. Chapman and Hall/CRC.
10. Turner MC,Jerrett M, Pope CA 3rd, Krewski D, Gapstur SM, Diver WR, Beckerman BS, Marshall JD, Su J, Crouse DL, Burnett RT. Long-term ozone exposure and mortality in a large prospective study . Am J Respir Crit Care Med. 2016; 193(10): 1134-42.
11. Yin P, Brauer M, Cohen A, et al. Long-term Fine Particulate Matter Exposure and Nonaccidental and Cause-specific Mortality in a Large National Cohort of Chinese Men. Environ Health Perspect 2017; 125: 117002.
12. Li T, Zhang Y, Wang J, et al. All-cause mortality risk associated with long-term exposure to ambient PM2·5 in China: a cohort study. Lancet Public Health 2018; 3: e470–7.
13. Yang Y, Tang R, Qiu H, et al. Long term exposure to air pollution and mortality in an elderly cohort in Hong Kong. Environ Int 2018; 117: 99–106.
14. Hystad P, Larkin A, Rangarajan S, AlHabib KF, Avezum A, Tumerdem Calik KB; Chifamba J, Dans A, Diaz R, du Plessis JL, Gupta R, Iqbal R, Khatib R, Kelishadi R, Lanas F, Liu Z, Lopez-Jaramillo P, Nair S, Poirier P, Rahman O, Rosengren A, Swidan H, Tse L-A, Wei L, Wielgosz A, Yeates K, Yusoff K, Zatoński T, Yusuf S, Brauer M. Outdoor fine particulate matter air pollution and cardiovascular disease: Results from 747 communities across 21 countries in the PURE Study. (Submitted to Lancet Global Health)
15. Joseph P, Rangarajan S, Islam S, Mente A, Hystad P, Brauer M, Raman Kutty V, Gupta R, Wielgosz A, AlHabib KF, Dans A, Lopez-Jaramillo P, Avezum A, Lanas F, Oguz A, Kruger IM, Diaz R, Yusoff K, Mony P, Chifamba J, Yeates K, Kelishadi R, Yusufali A, Khatib R, Rahman O, Zatonska K, Iqbal R, Wei L, Bo H, Rosengren A, Kaur M, Mohan V, Lear SA, Teo KK, O'Donnell M, McKee M, Dagenais G, Yusuf S. Modifiable risk factors, cardiovascular disease and mortality in 155,722 individuals from 21 high-, middle-, and low-income countries (PURE): a prospective cohort study. The Lancet. 2019. doi:10.1016/S0140-6736(19)32008-2
16. Burnett RT, Pope CA 3rd, Ezzati M, Olives C, Lim SS, Mehta S, Shin HH, Singh G, Hubbell B, Brauer M, Anderson HR, Smith KR, Balmes JR, Bruce NG, Kan H, Laden F, Prüss-Ustün A, Turner MC, Gapstur SM, Diver WR, Cohen A. An integrated risk function for estimating the global burden of disease attributable to ambient fine particulate matter exposure. *Environ Health Perspect*. 2014; 122(4): 397-403.
17. Pope CA III, Cohen AJ, Burnett RT. Cardiovascular Disease and Fine Particulate Matter: Lessons and Limitations of an Integrated Exposure Response Approach. Circulation Research. 2018;122:1645-1647.
18. Lind L, Sundström J, Ärnlöv J, Lampa E. Impact of Aging on the Strength of Cardiovascular Risk Factors: A Longitudinal Study Over 40 Years. *J Am Heart Assoc.* 2018;7(1):e007061. Published 2018 Jan 6. doi:10.1161/JAHA.117.007061
19. Semple S, Apsley A, Ibrahim TA, Turner SW, Cherrie JW. Fine particulate matter concentrations in smoking households: just how much secondhand smoke do you breathe in if you live with a smoker who smokes indoors? *Tob Control* 2015; 24: e205–11.

20. US Environmental Protection Agency. Integrated science assessment (ISA) for particulate matter (Final Report, Dec 2009). EPA/600/R-08/139F, 2009. Washington, DC: US Environmental Protection Agency; 2009. Available at: http://cfpub.epa.gov/ncea/risk/recordisplay.cfm?deid=216546
21. World Health Organization. Review of evidence on health aspects of air pollution – REVIHAAP Project technical report. Copenhagen: WHO Regional Office for Europe; 2013. Available at: http://www.euro.who.int/__data/assets/pdf_file/0004/193108/REVIHAAP-Final-technical-report-final-version.pdf?ua=1

Ambient ozone pollution

Flowchart

Ambient Ozone

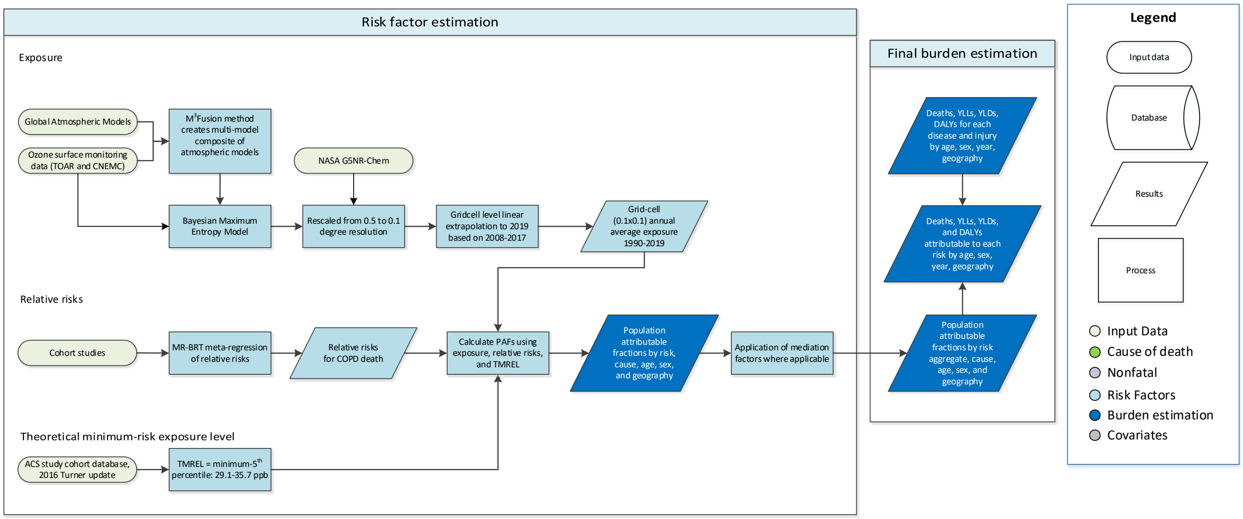

Input data and methodological summary

Exposure

To estimate the global distribution of exposure to ozone in ambient air (the highest seasonal [six-month] average of eight-hour daily maximum ozone concentrations, in parts per billion [ppb]) for the years 1990 to 2017, ozone ground measurement data were combined with chemical transport model estimates using Bayesian maximum entropy. Table 1 summarizes exposure input data.

Table 1: Exposure Input Data

Input data	Exposure
Source count (total)	14
Number of countries with data	1

Measurements data

Ozone monitoring data were obtained from the Tropospheric Ozone Assessment Report (TOAR), which contains the world's largest collection of surface ozone metrics (Schultz and colleagues, 2017). Since TOAR has released data until 2015 to the public, an update was made to include readily available datasets until 2017. In addition to TOAR, our analysis included ozone data from the China National Environmental Monitoring Center (CNEMC) Network, which contains surface ozone measurements for 2013–2017 in China (Lu and colleagues, 2018). All observations were processed to provide the six-month ozone season average of eight-hour daily maximum ozone concentrations.

Model combination

We used a combination of global atmospheric chemical transport models in our analysis, many of which simulated specified dynamics for the Chemistry-Climate Model Initiative (CCMI) (Morgenstern et al., 2017). Note that some of these modelling teams completed extra years of simulations beyond 2010 specifically for this project. The eight models and years available include the following: CHASER (1990–2010), MOCAGE (1988–2016), MRI-ESM (1988–2017), NASA MERRA2-GMI (1988–2017), NCAR CESM-Chem (1988–2010), NCAR WACCM (1988–2010), GFDL AM3 (1988–2014), and GFDL AM4 (2010–2016).

We obtained hourly ozone data for each of these models and then calculated the six-month maximum daily eight-hour maximum ozone mixing ratio (ppb). The M^3Fusion method (Chang and colleagues, 2019) was used to create a multi-model composite of the specified-dynamics models in each year from 1990 to 2017. This multi-model composite finds the linear combination of models available for each year that minimises the mean square error as compared to the observations in each world region, and in the process it corrects to minimise the mean model bias in each region. The world was divided geographically into eight regions: North America, South America, Europe, Africa, south central Asia, east Asia, Russia, and Oceania. In every region, each model was weighted to minimise the difference between the multi-model average and observations as described by the following:

Let s_g be the grid cell at resolution 0.5° × 0.5°, $\hat{y}(sg)$ be the interpolated observations, $\{\eta_k(sg); k = 1, \ldots, n\}$ be the model output registered onto the same grid from the n models available in a given year. α_r is a constant that allows adjustment to the overall (regional) underestimation or overestimation and β_{rk} is an optimal weight for the k-th model in region r.

$$\underset{\{\alpha_r, \beta_{rk}; k=1,..,n\}}{minimize} \sum_{s_g \in Region\ r} \left(\hat{y}(s_g) - \alpha_r - \sum_{k=1}^{n} \beta_{rk} \eta_k(s_g) \right)^2,$$

$$subject\ to \sum_{k=1}^{n} \beta_{rk} = 1\ and\ \beta_{rk} \geq 0$$

In the M^3Fusion method, weights are constrained to be positive and sum to 1. A constant offset,α_r, was included to guarantee that the residuals from this optimisation have a zero mean, through which the mean model bias is corrected (Chang and colleagues, 2019). In most regions and years, the multi-model mean ozone was biased high, so this method tends to decrease the average ozone.

Since the M^3Fusion method relies on surface measurements to change the weights, regions with sparse data had to be taken into account. North America and Europe use weights-based model and observation values for each individual year. The rest of the world regions (South America, Africa, south central Asia, east Asia, Russia, and Oceania) use individual year weights for 2000–2010, and apply weights calculated from the aggregated 2000 to 2010 period for 1990–1999. For 2011–2017, east Asia uses individual year weights, while South America, Africa, south central Asia, Russia, Oceania, and Antarctica use weights from the aggregated 2011–2014 period.

An example of the weighted values used to create the M^3Fusion model in North America and Europe are shown below, accompanied by a map of the M^3Fusion model in 2005.

	North America		Europe	
Year	*1995*	*2005*	*1995*	*2005*
CESM-Chem	0.38	0.29		
CHASER	0.04			
GFDL-AM3			0.18	0.62
MERRA		0.32	0.82	0.38
MOCAGE	0.36	0.01		
MRI-ESM	0.22	0.27		
WACCM		0.11		

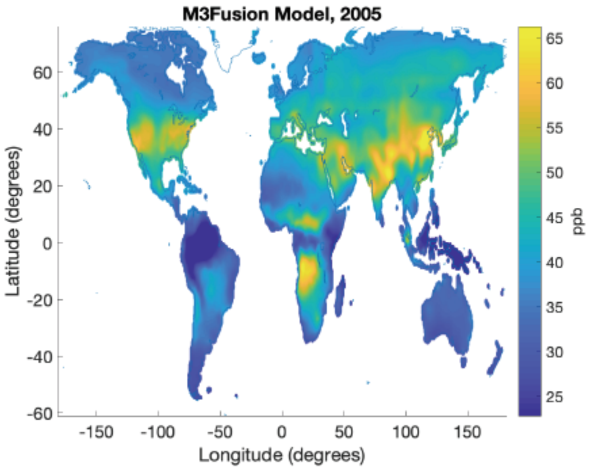

Bayesian maximum Entropy (BME)

BME is a geostatistical modelling tool that can be used to combine various knowledge bases for an air pollutant and combine them to create a single product. In this case, we use BME to combine site-specific measurements and modelled concentrations, making use of the correlations between measurement locations. BME uses the measurement values to correct the M^{3}Fusion Model locally around each station spatially and temporally, allowing future and past observations to provide input. Since more measurement locations became available through time, this method allows later measurements to influence ozone surfaces earlier in the period, which is particularly important in China and data-sparse regions. The range over which each measurement can correct the M^{3}Fusion Model and how each measurement's impact decreases over distance in time and space are calculated as part of BME.

Beyond combining these knowledge bases to provide an estimate of ozone pollution, BME also estimates a variance, which can be used to assess estimation confidence at different locations.

In short, the steps are:

1. Let **Z(p)** be a field of ozone concentration estimations in space and time and let **mo(p)** be the M^{3}Fusion model output values in space and time.
2. Subtract the M^{3}Fusion model output values at each measure point from the observed values, **z(p)**, to obtain residuals **x(p)**=**z(p)**-**mo(p)**. Examples for 2005 of **z** and **x** are shown below on the left and right, respectively:

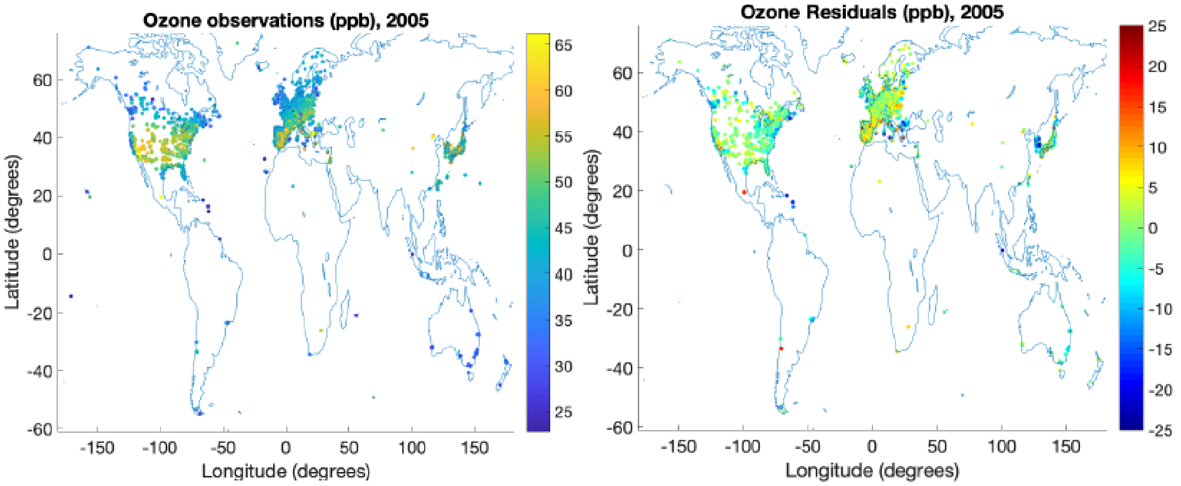

3. Model the covariance (the correlation between locations in space and time) $\mathbf{c_x}$ based on the residuals $\mathbf{x}$. The covariance of the residuals is the range of influence of a measurement to predict other concentrations in space and time. A shallower curve indicates that ozone values are correlated over a greater distance, while a steep dropoff indicates the reverse. The modelled spatial and temporal covariance are displayed below with the corresponding equation:

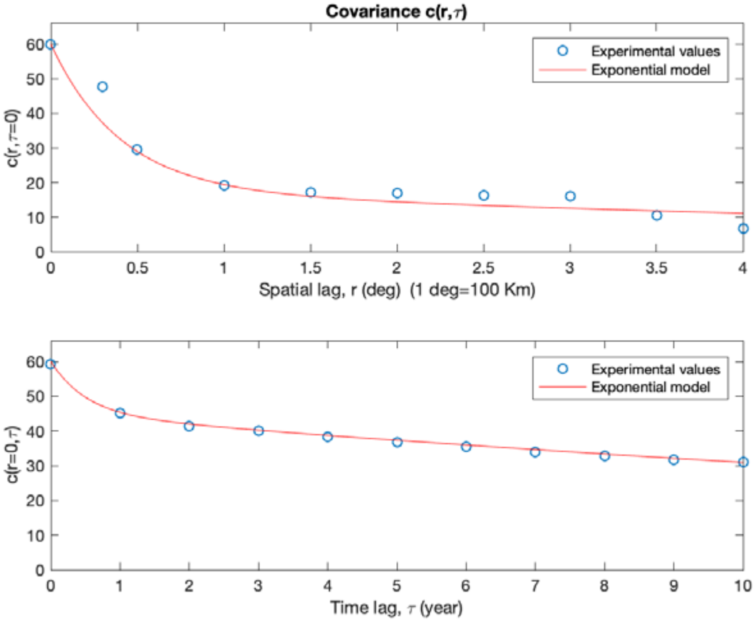

$$C_x(r,\tau) = 59.9938\ ppb^2(0.7\exp\left(-\frac{3r}{1.2\ degrees}\right)\exp\left(-\frac{3\tau}{80\ years}\right) + 0.05\exp\left(-\frac{3r}{25\ degrees}\right)\exp\left(-\frac{3\tau}{80\ years}\right) + 0.25\exp\left(-\frac{3r}{25\ degrees}\right)\exp\left(-\frac{3\tau}{1.5\ years}\right)$$

Where τ is temporal distance and r is spatial distance

Note that the spatial covariance drops off steeply spatially such that the influence of a measurement location becomes very small beyond 1 degree of distance. However, the temporal covariance remains high, meaning that observations can influence ozone estimates through time over several years.

4. Combine the observation data residuals **(x)**, covariance **(cx),** and estimation parameters to get the BME estimation **(xk)** and variance **(vk)** on a 0.5º by 0.5º grid, shown for 2005.

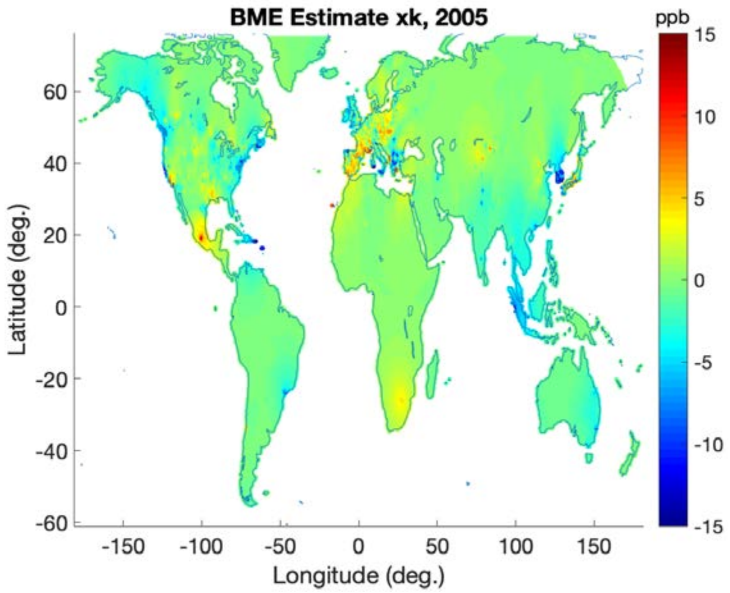

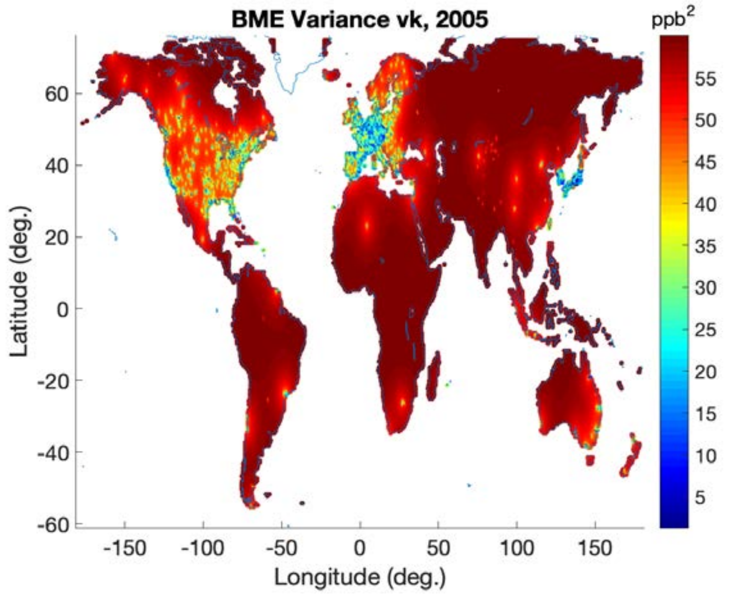

The variance is zero where the location of the estimation point matches an observation point in that year. The variance increases as the space-time distance from an observation increases, until the variance reaches a maximum value equal to the sill of the covariance equation (59.9938 ppb^2).

5. Obtain final BME estimation values **(z_k)**, shown for 2005 in the figure below, by adding back the previously subtracted model values **mo(p_k)** to the BME estimation **(x_k)**.

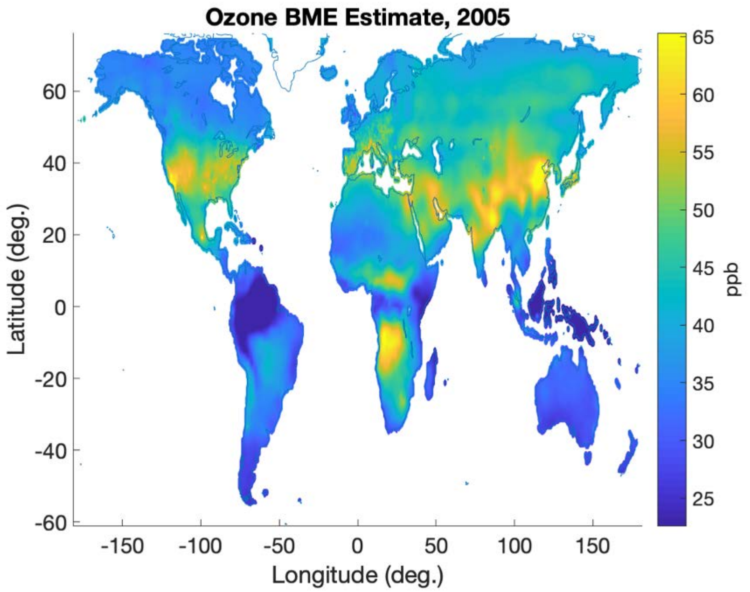

Adding fine resolution

Our results were calculated at 0.5° resolution, so to downscale estimates at finer resolution, we used the NASA G5NR-Chem model. The NASA G5NR-Chem model simulates surface ozone concentrations at 0.125° by 0.125° resolution for July 2013 to June 2014 (Hu and colleagues, 2018). We regridded the G5NR-Chem model from 0.125° resolution to 0.1° resolution. While we do not expect that the raw values for 2013–2014 hold true for every year, we believe that the spatial distribution of this model can be used to inform the fine-scale spatial pattern for each year. To add fine resolution, we performed the following steps:

1. Regrid NASA G5NR-Chem from 0.125° resolution to 0.1° resolution
2. Average each 0.5° NASA G5NR-Chem grid cell
3. Calculate the difference between our BME estimation results at 0.5° and the average NASA G5NR-Chem at 0.5°
4. Add the calculated difference to NASA G5NR-Chem at 0.1° to obtain our BME estimation at 0.1°

Adding fine resolution to our results keeps the average of each 0.5° grid cell the same as the original estimation at 0.5°, as well as the global average.

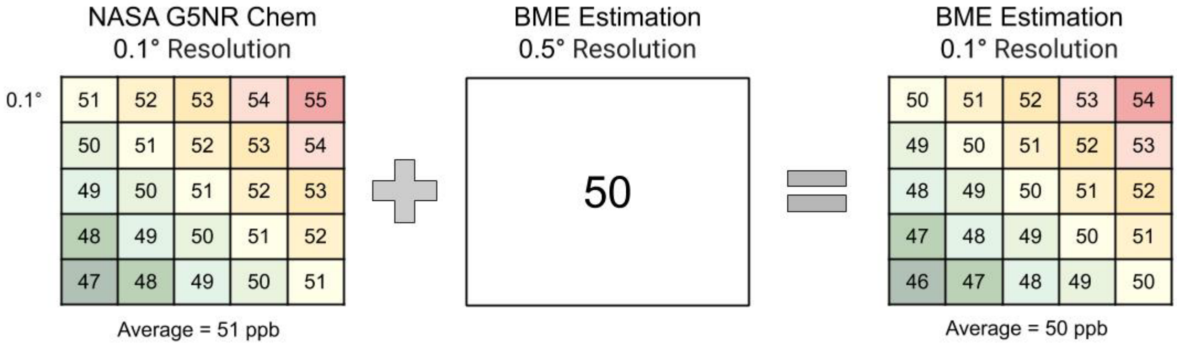

Final output

Three years are shown as an example below: 1995, 2005, and 2015. For each year, there are five maps displayed: the observations, M^3Fusion Model, BME Estimate, the difference between the BME Estimate and the M^3Fusion Model, and the variance. The difference map shows that the BME method corrects the M^3Fusion Model near monitoring stations, including stations in other years.

1995

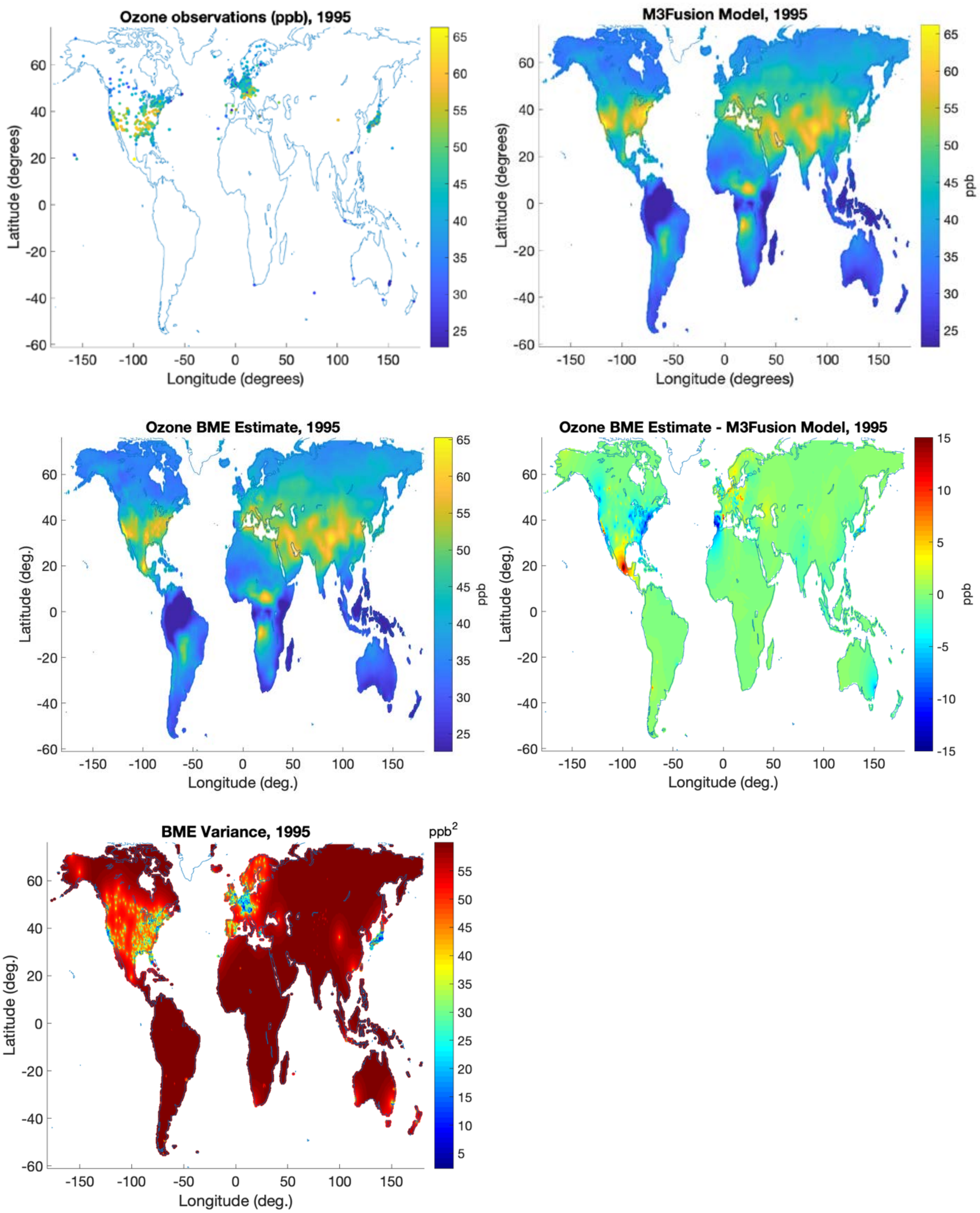
Ozone observations (ppb), 1995
Latitude (degrees)
Longitude (degrees)
M3Fusion Model, 1995
Latitude (degrees)
Longitude (degrees)
ppb
Ozone BME Estimate, 1995
Latitude (deg.)
Longitude (deg.)
ppb
Ozone BME Estimate - M3Fusion Model, 1995
Latitude (deg.)
Longitude (deg.)
ppb
BME Variance, 1995
Latitude (deg.)
Longitude (deg.)
ppb2

2005

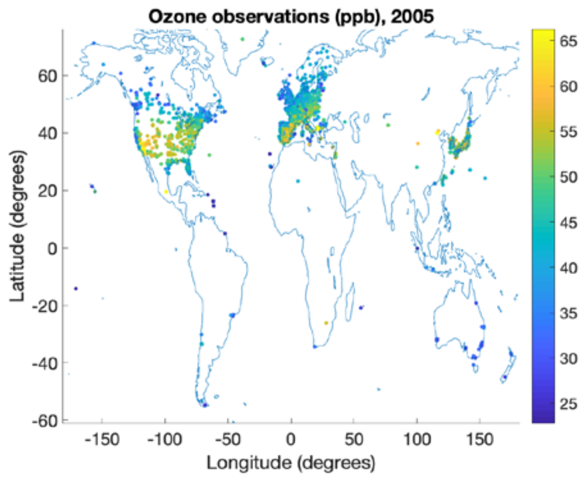
Ozone observations (ppb), 2005
Latitude (degrees)
Longitude (degrees)
60
40
20
0
-20
-40
-60
-150
-100
-50
0
50
100
150
65
60
55
50
45
40
35
30
25

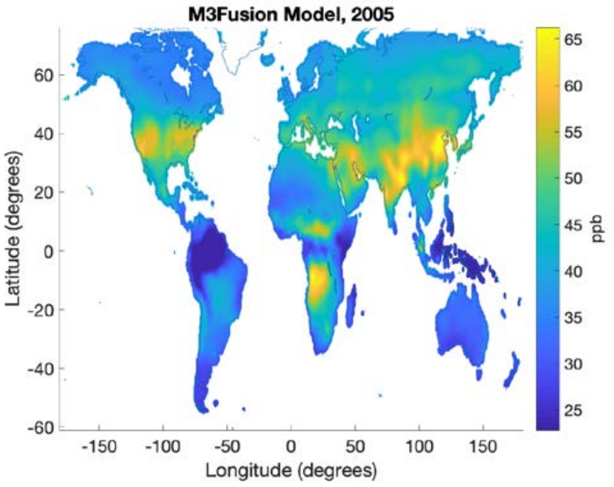
M3Fusion Model, 2005
Latitude (degrees)
Longitude (degrees)
ppb
60
40
20
0
-20
-40
-60
-150
-100
-50
0
50
100
150
65
60
55
50
45
40
35
30
25

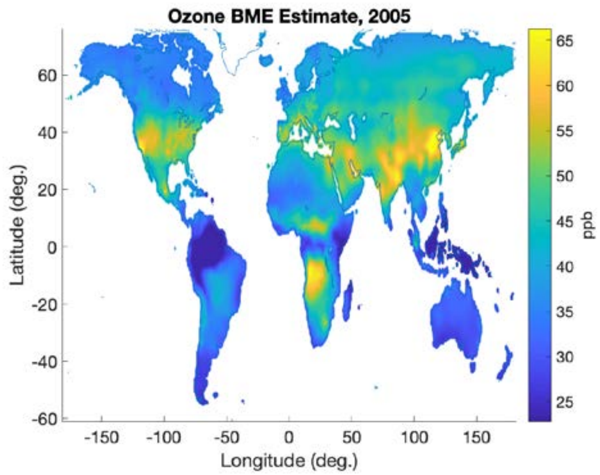
Ozone BME Estimate, 2005
Latitude (deg.)
Longitude (deg.)
ppb
60
40
20
0
-20
-40
-60
-150
-100
-50
0
50
100
150
65
60
55
50
45
40
35
30
25

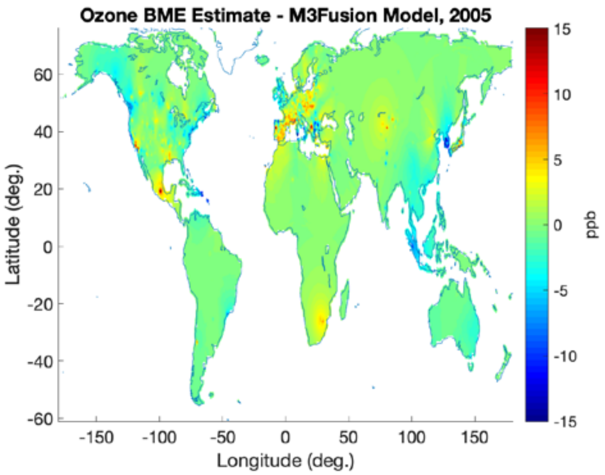
Ozone BME Estimate - M3Fusion Model, 2005
Latitude (deg.)
Longitude (deg.)
ppb
60
40
20
0
-20
-40
-60
-150
-100
-50
0
50
100
150
15
10
5
0
-5
-10
-15

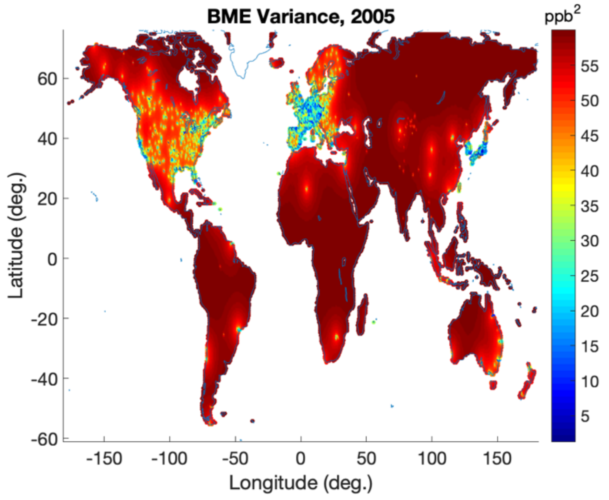
BME Variance, 2005
ppb^2
Latitude (deg.)
Longitude (deg.)
60
40
20
0
-20
-40
-60
-150
-100
-50
0
50
100
150
55
50
45
40
35
30
25
20
15
10
5

2015

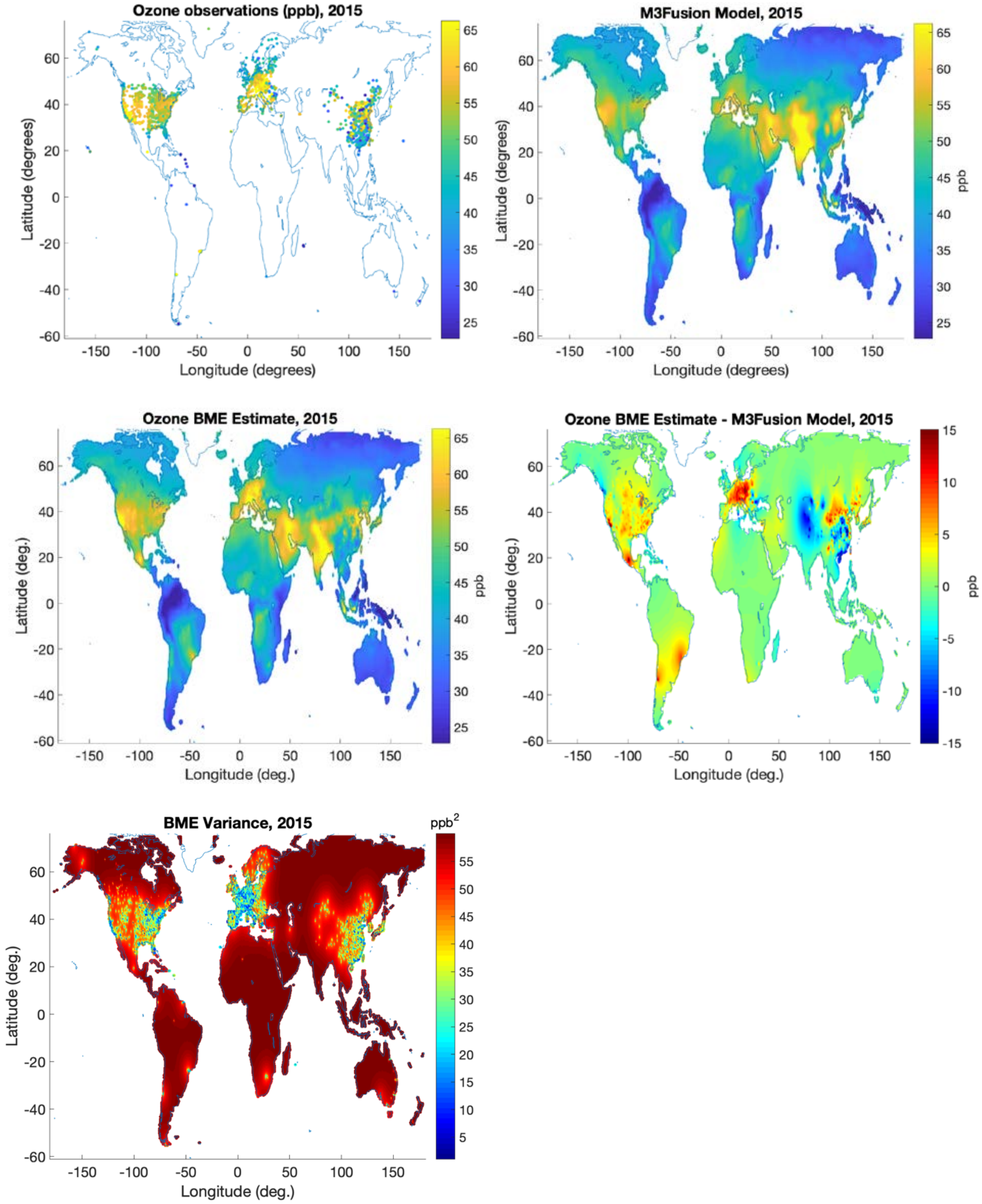

Ozone observations (ppb), 2015
Latitude (degrees)
Longitude (degrees)
M3Fusion Model, 2015
Latitude (degrees)
Longitude (degrees)
ppb
Ozone BME Estimate, 2015
Latitude (deg.)
Longitude (deg.)
ppb
Ozone BME Estimate - M3Fusion Model, 2015
Latitude (deg.)
Longitude (deg.)
ppb
BME Variance, 2015
Latitude (deg.)
Longitude (deg.)
ppb2

Extrapolation and annual means

To estimate global ozone in 2018 and 2019, for each 0.1° grid cell, we ran a log-linear model of the ozone estimates on year for the most recent ten years (2008–2017) of the following form:

$$\log(\text{ozone}) \sim \text{year}+1.$$

We considered using splines, but due to annual variation of ozone, we found a log-linear trend to provide the most reasonable prediction.

For burden estimation we are more interested in long-term trends and effects than annual variation; therefore, for the years 1991–2016, we used a three-year mean of exposure centered on the year of interest. This strategy aligns with the methodology used for ambient particulate matter air pollution. For 1990 and 2017, we used two-year means (1990/1991 and 2016/2017, respectively) because 1989 and 2018 were not available in the estimates.

To estimate the variance for the three-year mean to generate confidence intervals, we did not have information on the covariance between years, so a conservative estimate of the variance was made:

Let *X*, *Y*, and *Z* be random variables describing ozone exposure in a given 0.1 degree grid cell for years *i-1*, *i,* and *i+1*, respectively. By the laws of variance,

$$Var\left(\frac{1}{3}(X+Y+Z)\right)=\frac{1}{9}\Big(Var(X)+Var(Y)+Var(Z)+2Cov(X,Y)+2Cov(X,Z)+2Cov(Y,Z)\Big).$$

We do not know the covariance, but by the Cauchy-Schwartz inequality,

$$Cov(A,B)\leq\sqrt{Var(A)*Var(B)}.$$

Therefore,

$$Var\left(\frac{1}{3}(X+Y+Z)\right)\leq\frac{1}{9}\Big(Var(X)+Var(Y)+Var(Z)+2\sqrt{Var(X)*Var(Y)}+2\sqrt{Var(X)*Var(Z)}+2\sqrt{Var(Y)*Var(Z)}\Big).$$

This is a conservative estimate of the variance used when taking a three-year mean.

Difference from previous estimations

This method improves upon the GBD 2017 ozone exposure estimates (Chang and colleagues, 2019) in the following ways:

1. The previous estimates used observations in a specific year to correct the model within 2° of a monitoring station. In the current method, the radius of influence of each observation is defined by the spatial covariance. The spatial covariance shows that much of the influence of an observation is lost after 1°.
2. Measurements not only bias-correct the model in the year in which they were observed, but also influence other years according to the temporal covariance. This is important for regions that were not monitored over the entire 1990–2017 time period.
3. The fine spatial structure of the final product represents the spatial distribution of the 0.125° NASA G5NR-Chem model.

Theoretical minimum-risk exposure level

As in GBD 2017, the TMREL is based on the exposure distribution from the ACS CPS-II study (Turner and colleagues, 2016). It is a uniform distribution around the minimum and fifth percentile values observed in the cohort, ~U(29.1, 35.7), in ppb.

Relative risks

COPD mortality is the only included outcome for ambient ozone pollution.

In GBD 2017, we performed a literature review of studies examining long-term ozone exposure and COPD. We included five cohorts from Canada, the UK, and the USA, all of which reported ozone effects on COPD mortality (Turner and colleagues, 2016; Carey and colleagues, 2013, and Burnett RT. “Cox…”). For this reason, we only include mortality and not incidence as an outcome of ozone exposure. Table 2 summarizes relative risk input data.

Table 2: Relative Risk Input Data

Input data	Relative risk
Source count (total)	5
Number of countries with data	3

In GBD 2019, we updated our methodology to use MR-BRT for the meta-analysis of relative risks. Because we had only five datapoints, we included no study-level covariates. We also included no priors. The inverse-variance weighted meta-analysis of the five cohorts provided an estimated relative risk of 1.06 (95% CI 1.03, 1.10) with an estimated gamma (including between study heterogeneity) of 0.004.

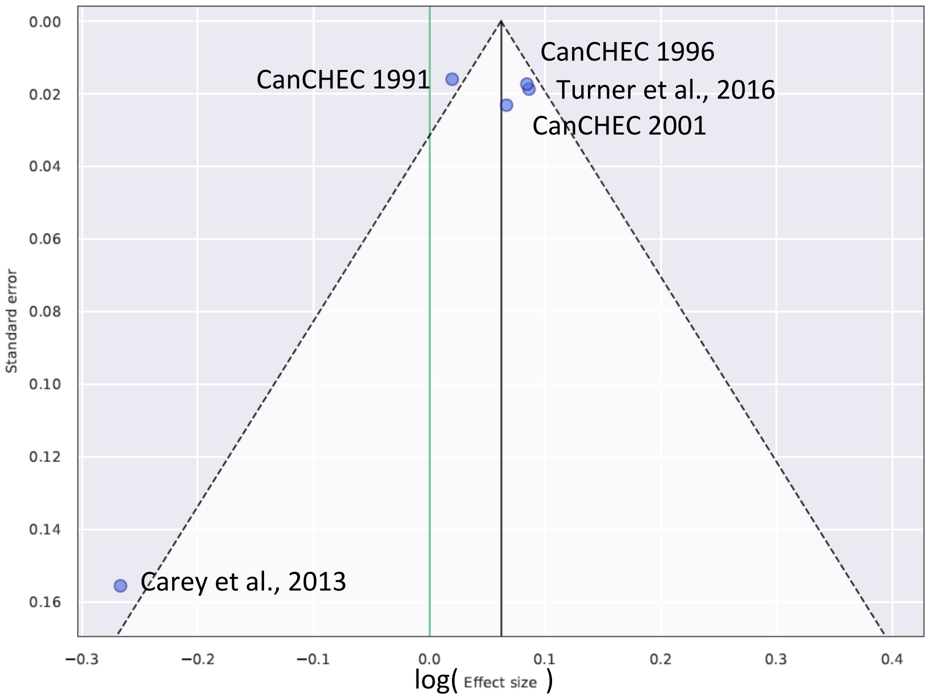

We calculated PAFs at the grid-cell level and aggregated up to GBD locations using population data from the Gridded Population of the World database. Estimates came from version 4 except for estimates for 1990 and 1995 from version 3. More details on these estimates are available in the Ambient particulate matter pollution methods appendix.

References

1. Schultz MG, Schröder S, Lyapina O, Cooper O, Galbally I, Petropavlovskikh I, et al. Tropospheric Ozone Assessment Report: Database and Metrics Data of Global Surface Ozone Observations. Elem Sci Anth. 2017;5:58. DOI: http://doi.org/10.1525/elementa.244
2. Lu, Xiao, Jiayun Hong, Lin Zhang, Owen R. Cooper, Martin G. Schultz, Xiaobin Xu, Tao Wang, Meng Gao, Yuanhong Zhao, and Yuanhang Zhang. Severe surface ozone pollution in China: a global perspective. Environmental Science & Technology Letters 2018 *5* (8), 487-49 DOI: 10.1021/acs.estlett.8b00366
3. Chang, Kai-Lan, R. Cooper, Owen, West, Jason, L. Serre, Marc, G. Schultz, Martin, Lin, Meiyun, Marecal, Virginie, Josse, B, Deushi, Makoto, Sudo, Kengo, Liu, Junhua & A. Keller, Christoph. (2019). A new method (M^3Fusion v1) for combining observations and multiple model output for an improved estimate of the global surface ozone distribution. Geoscientific Model Development. 12. 955-978. 10.5194/gmd-12-955-2019.
4. Hu, L., C. A. Keller, M. S. Long, T. Sherwen, B. Auer, A. Da Silva, J. E. Nielsen, S. Pawson, M. A. Thompson, A. L. Trayanov, K. R. Travis, S. K. Grange, M. J. Evans, D. J. Jacob (2018) Global simulation of tropospheric chemistry at 12.5 km resolution: performance and evaluation of the GEOS-Chem chemical module (v10-1) within the NASA GEOS Earth system model (GEOS-5 ESM), Geoscientific Model Development, 11, 4603-4620. 10.5194/gmd-11-4603-2018.
5. Morgenstern O, Hegglin MI, Rozanov E, O'Connor FM, Abraham NL, Akiyoshi H, Archibald AT, Bekki S, Butchard N, Chipperfield MP, Deushi M, Dhomse SS, Garcia RR, Hardiman SC, Horowitz LW, Jockel P, Josse B, Kinnison D, Lin M, Mancini E, Manyin ME, Marchand M, Marecal V, Michou M, Oman LD, Pitari G, Plummer DA, Revell LE, Saint-Martin D, Schofield R, Stenke A, Stone K, Sudo K, Tanaka TY, Tilmes S, Yamashita Y, Yoshida K, Zeng G. Review of the global models used within phase 1 of the Chemistry-Climate Model Initiative (CCMI). *Geosci. Model Dev.* 2017; 10: 639-671.
6. Turner MC, Jerrett M, Pope CA 3rd, Krewski D, Gapstur SM, Diver WR, Beckerman BS, Marshall JD, Su J, Crouse DL, Burnett RT. Long-term ozone exposure and mortality in a large prospective study. *Am J Respir Crit Care Med*. 2016; 193(10): 1134-42.
7. Carey IM, Atkinson RW, Kent AJ, van Staa T, Cook DG, Anderson HR. Mortality associations with long-term exposure to outdoor air pollution in a national English cohort. *Am J Respir Crit Care Med.* 2013; 187(11): 1226-33.
8. Burnett RT. Cox Proportional Survival Model Hazard Ratios from Census Years (1991,1996,2001) to 2011 for Adults Aged 25 to 89 in CanCHEC Cohort. Custom Anaylsis for GBD2017.

References for Atmospheric Chemical Transport Models

- CESM:

- Tilmes, S., Lamarque, J.-F., Emmons, L. K., Kinnison, D. E., Ma, P.-L., Liu, X., Ghan, S., Bardeen, C., Arnold, S., Deeter, M., Vitt, F., Ryerson, T., Elkins, J. W., Moore, F., Spackman, J. R., and Val Martin, M.: Description and evaluation of tropospheric chemistry and aerosols in the Community Earth System Model (CESM1.2), Geosci. Model Dev., 8, 1395–1426, doi:10.5194/gmd-8-1395-2015, 2015

- CESM WACCM:
  - Garcia, R. R., Smith, A. K., Kinnison, D. E., de la Cámara, Á., and Murphy, D.: Modifications of the gravity wave parameterization in the Whole Atmosphere Community Climate Model: Motivation and results, J. Geophys. Res.-Atmos., doi:10.1175/JAS-D16-0104.1, 2016.
  - Marsh, D., Mills, M. J., Kinnison, D. E., Garcia, R. R., Lamarque, J.-F., and Calvo, N.: Climate change from 1850–2005 simulated in CESM1 (WACCM), J. Climate, 26, 7372–7391, doi:10.1175/JCLI-D-12-00558.1, 2013
- CHASER:
  - Sudo, K., Takahashi, M., and Akimoto, H.: CHASER: A global chemical model of the troposphere, 2. Model results and evaluation, J. Geophys. Res., 107, 4586, https://doi.org/10.1029/2001JD001114, 2002a.
  - Sudo, K., Takahashi, M., Kurokawa, J., and Akimoto, H.: CHASER: A global chemical model of the troposphere, 1. Model description, J. Geophys. Res., 107, 4339, https://doi.org/10.1029/2001JD001113, 2002b.

  - Watanabe, S., Hajima, T., Sudo, K., Nagashima, T., Takemura, T., Okajima, H., Nozawa, T., Kawase, H., Abe, M., Yokohata, T., Ise, T., Sato, H., Kato, E., Takata, K., Emori, S., and Kawamiya, M.: MIROC-ESM 2010: model description and basic results of CMIP5-20c3m experiments, Geosci. Model Dev., 4, 845–872, https://doi.org/10.5194/gmd-4-845-2011, 2011.
- GEOSCCM:
  - Oman, L. D., Ziemke, J. R., Douglass, A. R., Waugh, D. W., Lang, C., Rodriguez, J. M., and Nielsen, J. E.: The response of tropical tropospheric ozone to ENSO, Geophys. Res. Lett., 38, L13706, https://doi.org/10.1029/2011GL047865, 2011.
- GFDL AM3 & AM4:
  - Lin, M., Fiore, A. M., Horowitz, L. W., Cooper, O. R., Naik, V., Holloway, J., Johnson, B. J., Middlebrook, A. M., Oltmans, S. J., Pollack, I. B., Ryerson, T. B., Warner, J. X., Wiedinmyer, C., Wilson, J., and Wyman, B.: Transport of Asian ozone pollution into surface air over the western United States in spring, J. Geophys. Res., 117, D00V07, https://doi.org/10.1029/2011JD016961, 2012.
  - Lin, M., Horowitz, L. W., Oltmans, S. J., Fiore, A. M., and Fan, S.: Tropospheric ozone trends at Mauna Loa Observatory tied to decadal climate variability, Nat. Geosci., 7, 136–143, https://doi.org/10.1038/NGEO2066, 2014.
  - Lin, M., Horowitz, L. W., Payton, R., Fiore, A. M., and Tonnesen, G.: US surface ozone trends and extremes from 1980 to 2014: quantifying the roles of rising Asian emissions, domestic controls, wildfires, and climate, Atmos. Chem. Phys., 17, 2943–2970, https://doi.org/10.5194/acp-17-2943-2017, 2017.
- MERRA GMI:

- Ziemke, J. R., Oman, L. D., Strode, S. A., Douglass, A. R., Olsen, M. A., McPeters, R. D., Bhartia, P. K., Froidevaux, L., Labow, G. J., Witte, J. C., Thompson, A. M., Haffner, D. P., Kramarova, N. A., Frith, S. M., Huang, L.-K., Jaross, G. R., Seftor, C. J., Deland, M. T., and Taylor, S. L.: Trends in global tropospheric ozone inferred from a composite record of TOMS/OMI/MLS/OMPS satellite measurements and the MERRA-2 GMI simulation , Atmos. Chem. Phys., 19, 3257–3269, https://doi.org/10.5194/acp-19-3257-2019, 2019.

- MOCAGE:
  - Josse, B., Simon, P., and Peuch, V.-H.: Radon global simulations with the multiscale chemistry and transport model MOCAGE, Tellus B, 56, 339–356, https://doi.org/10.1111/j.1600-0889.2004.00112.x, 2004.
  - Teyssèdre, H., Michou, M., Clark, H. L., Josse, B., Karcher, F., Olivié, D., Peuch, V.-H., Saint-Martin, D., Cariolle, D., Attié, J.-L., Nédélec, P., Ricaud, P., Thouret, V., van der A, R. J., Volz-Thomas, A., and Chéroux, F.: A new tropospheric and stratospheric Chemistry and Transport Model MOCAGE-Climat for multi-year studies: evaluation of the present-day climatology and sensitivity to surface processes, Atmos. Chem. Phys., 7, 5815–5860, https://doi.org/10.5194/acp-7-5815-2007, 2007.
- MRI-ESM1r1:
  - Adachi, Y., Yukimoto, S., Deushi, M., Obata, A., Taichu. Y. Tanaka, H. N., Hosaka, M., Sakami, T., Yoshimura, H., Hirabara, M., Shindo, E., Tsujino, H., Mizuta, R., Yabu, S., Koshiro, T., Ose, T., and Kitoh, A.: Basic performance of a new earth system model of the Meteorological Research Institute (MRI-ESM1), Pap. Meteorol. Geophys, 64, 1–18, https://doi.org/10.2467/mripapers.64.1, 2013.

Contributors:

- Marissa DeLang, Jacob S. Becker, Stephanie Cleland, Elyssa Collins, Marc L. Serre, J. Jason West, University of North Carolina at Chapel Hill
- Owen R. Cooper and Kai-Lan Chang, CIRES, University of Colorado, Boulder/NOAA Earth System Research Laboratory, Boulder, USA
- Martin G. Schultz and Sabine Schröder, Jülich Supercomputing Centre (JSC), Forschungszentrum Jülich, Jülich, DE
- Xiao Lu and Lin Zhang, Laboratory for Climate and Ocean-Atmosphere Studies, Dept. of Atmospheric and Oceanic Sciences, School of Physics, Peking University, Beijing, China
- CCMI and NASA modellers

Household air pollution

Flowchart

Household Air Pollution from Solid Fuels

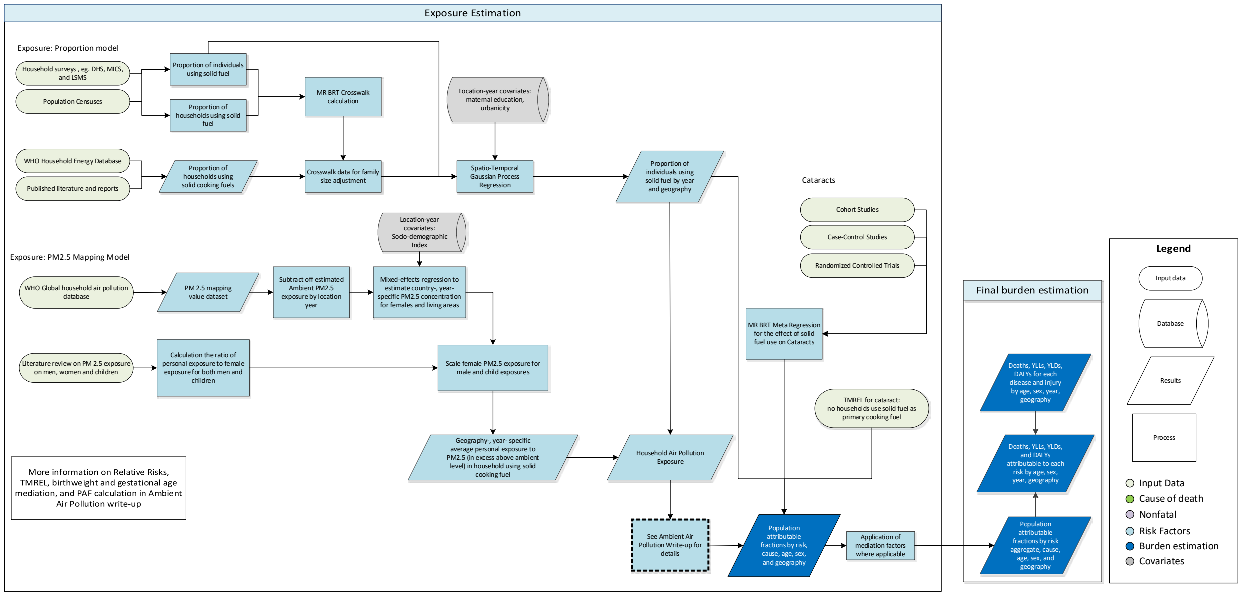

Input data and methodological summary

Exposure

Case definition

Exposure to household air pollution from solid fuels (HAP) is estimated from both the proportion of individuals using solid cooking fuels and the level of $PM_{2.5}$ air pollution exposure for these individuals. Solid fuels in our analysis include coal, wood, charcoal, dung, and agricultural residues.

Input data

We extracted information on use of solid fuels from the standard multi-country survey series such as Demographic and Health Surveys (DHS), Living Standards Measurement Surveys (LSMS), Multiple Indicator Cluster Surveys (MICS), and World Health Surveys (WHS), as well as censuses and country-specific survey series such as Kenya Welfare Monitoring Survey and South Africa General Household Survey. To fill the gaps of data in surveys and censuses, we also downloaded and updated estimates from WHO Energy Database and extracted from literature through systematic review. Each nationally or subnationally representative datapoint provided an estimate for the percentage of households using solid cooking fuels. We used studies from 1980 to 2019 to inform the time series.

We also excluded sources that did not distinguish specific primary fuel types, estimated fuel used for purposes other than cooking (eg, lighting or heating), failed to report standard error or sample size, had over 15% of households with missing responses, reported fuel use in physical units, or were secondary sources referencing primary analyses. Table 1 summarizes exposure input data.

Table 1: Exposure Input Data

Input data	Exposure
Source count (total)	1680
Number of countries with data	195

Family size crosswalk

Many estimates in the WHO Energy Database and other reports quantify the proportion of households using solid fuel for cooking; however, we are interested in the proportion of individuals using solid fuel for cooking. To crosswalk these estimates, whenever we had the available information, we extracted fuel use at both the individual and household levels. We included 3676 source-specific pairs in the MR-BRT crosswalk model.

MR-BRT crosswalk adjustment factors for household air pollution exposure

Data input	Reference or alternative case definition	Gamma	Beta coefficient, logit (95% CI)
Proportion of individuals	Ref	0.097	---
Proportion of Households	Alt		-0.095<br>(-0.100, -0.090)

We then apply this coefficient to household-only reports with the following formula:

$prop_{individ}$ = the proportion of individuals using solid fuel for cooking, and

$prop_{hh}$ = the proportion of households using solid fuel for cooking.

$$\log\left(\frac{prop_{individ}}{1 - prop_{individ}}\right) = \log\left(\frac{prop_{hh}}{1 - prop_{hh}}\right) - \beta$$

or

$$prop_{individ} = \frac{prop_{hh} * e^{-\beta}}{1 - prop_{hh} + prop_{hh} * e^{-\beta}}$$

The effect is that the household studies are inflated to account for bias. Larger households are more likely to use solid fuel for cooking. The following figure depicts the 3676 data points that informed the crosswalk model. There the red points indicate the 10% of studies that were trimmed as outliers.

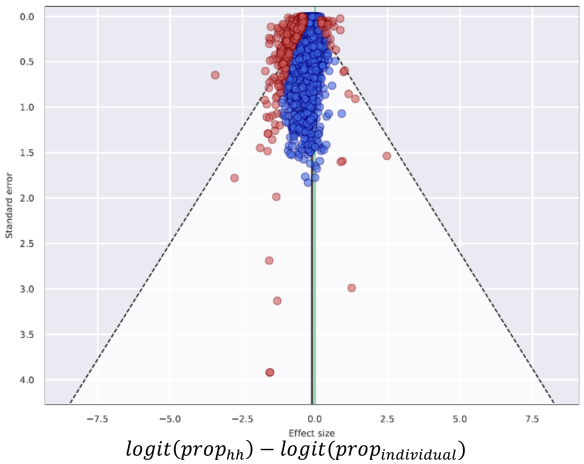

$$logit(prop_{hh}) - logit(prop_{individual})$$

Modelling strategy

Household air pollution was modelled at individual level using a three-step modelling strategy that uses linear regression, spatiotemporal regression, and Gaussian process regression (GPR). The first step is a mixed-effect linear regression of logit-transformed proportion of individuals using solid cooking fuels. The linear model contains maternal education and the proportion of population living in urban areas as covariates and has nested random effects by GBD region and GBD super-region. The full ST-GPR process is specified elsewhere this appendix. No substantial modelling changes were made in this round compared to GBD 2017.

First-stage linear model and coefficients

$$logit(proportion) \sim maternal\ education + urbanicity + (1|region) + (1|super - region)$$

**Variable**	**Beta (95% CI)**
Intercept	3.16 (1.59, 4.74)
Maternal education (years per capita)	-0.45 (-0.76, -0.15)
Urbanicity (proportion of population living in urban areas)	-1.42 (-2.67, -0.17)

Theoretical minimum-risk exposure level

For cataract, the TMREL is defined as no households using solid cooking fuel. For outcomes related to both ambient and household air pollution, the PAFs are estimated jointly and the TMREL is defined as uniform distribution between 2.4 and 5.9 ug/m^3 $PM_{2.5}$.

Relative risks

In addition to the previously included outcomes of lower respiratory infections (LRI), stroke, ischaemic heart disease (IHD), chronic obstructive pulmonary disease (COPD), lung cancer, type 2 diabetes, and cataract, in GBD 2019 we added low birthweight and short gestation as new outcomes of household air pollution through a mediation analyses. With the exception of cataract, all causes share risk curves and are jointly calculated with ambient $PM_{2.5}$ air pollution. Table 2 summarizes relative risk input data for ambient particulate matter pollution and household air pollution.

Table 2: Relative Risk Input Data

Input data	Relative risk
Source count (total)	200
Number of countries with data	40

Prior to GBD 2019, we utilised the results of an external meta-analysis with a summary relative of 2.47 with 95% CI (1.63, 3.73).[1] While this effect estimate was for both sexes, in the past we estimated burden for women only because women are known to have higher HAP exposure than men. In GBD 2019, we performed our own meta-regression analysis of household air pollution and cataracts. We extracted all of the components studies of the above meta-analysis paper but excluded one cross-sectional study. GBD risk factor analyses typically do not include cross-sectional analyses. In additional literature search, we found one additional paper describing different fuel types and cataracts.[4] We excluded this study because there was no comparison group without solid fuel use. Our resulting dataset contained eight estimates from six sources in India and Nepal.

On these eight estimates, we ran a MR BRT meta-regression to generate a summary effect size of 2.51 (1.58, 3.96). We included a study-level bias covariate of whether or not the study participants were blind to the exposure-outcome pair of interest. The prior on this covariate was a Gaussian distribution with mean 0 and variance 0.1. The prior on gamma was a Gaussian distribution with mean 0.04 and 0.1. The table and figure below provide the model coefficients and a visual representation.

MR-BRT relative risk meta-analysis for household air pollution and cataract

Covariate	Gamma	Beta coefficient, logit (95% CI)	Beta coefficient, adjusted (95% CI)
Intercept	0.40	0.918 (0.460, 1.377)	2.51 (1.58, 3.96)
Outcome unblinded		0.031 (-0.450, 0.512)	1.03 (0.64, 1.67)

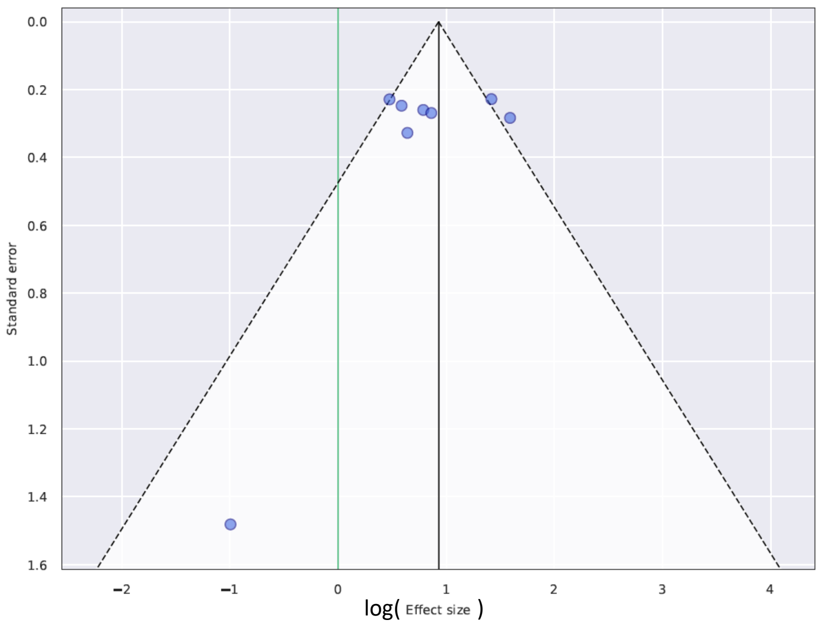

Studies reported effect sizes for males, females, and/or both sexes. In a sensitivity analysis we included a covariate for sex and found no significant difference in effect size by sex. Therefore, we now estimate cataract as an outcome of household air pollution in both males and females.

In GBD 2019, we also made substantial changes to our particulate matter risk curves. These risk curves, utilising splines in MR-BRT, the new mediation analysis with birthweight and gestational age, and the joint-estimation PAF approach are described in the ambient particulate matter appendix.

$PM_{2.5}$ mapping value

In order to use the particulate matter risk curves, we must estimate the level of exposure to particulate matter with diameter of less than 2.5 micrometers ($PM_{2.5}$) for individuals using solid fuels for cooking. The Global Household Air Pollution (HAP) Measurements database from WHO contains 196 studies with measurements from 43 countries of various pollution metrics in households using solid fuel for cooking.[2] From this database, we take all measurements of $PM_{2.5}$ using indoor or personal monitors. In addition to the WHO database, we included eight additional studies from a systematic review conducted in 2015 for GBD.

The final dataset included 336 estimates from 75 studies in 43 unique locations. We included 260, 64, nine, and three measurements indoors, on personal monitors for females, children (under 5), and males, respectively. 274 estimates were in households using solid fuels, 47 in households only using clean (gas or electricity) fuels, and 15 in households using a mixture of solid and clean fuels.

We use the following model:

$$\log(excess\ PM) \sim solid + measure\ group + 24\ hr\ measurement + SDI + (1|study)$$

Where,

- 24-hour measurement: binary variable equal to 1 if the measurement occurred over at least a 24-hour period and not only during mealtimes
- Measure group: categorical variable indicating indoor, female, male, or children

- Solid: indicator variable equal to 1 if the measurements were among households using solid fuel only, 0.5 if the measurements represented a mix of clean and solid fuels, and 0 if the households only used clean fuels.

We also included the Socio-demographic Index (SDI) as a variable to predict a unique value of HAP for each location and year based on development. We also included a random effect on study. We weighted each study by its sample size.

Before modelling, we calculated the excess particulate matter in households using solid fuel by subtracting off the predicted ambient $PM_{2.5}$ value in the study location and year based on the GBD 2017 $PM_{2.5}$ exposure model. The final model coefficients are included below:

HAP mapping model and coefficients

Variable	Beta, log (95% CI)	Beta, adjusted (95% CI)
Intercept	6.23 (4.58, 7.88)	506 (97, 2635)
Solid	2.60 (2.06, 3.13)	13.4 (7.8, 23.0)
Measure group<br>• Indoor (ref)<br>• Female<br>• Male<br>• Child	 <br>-0.56 (-1.15, 0.04)<br>-1.56 (-3.81, 0.70)<br>-1.13 (-2.06, -0.20)	 <br>0.57 (0.32, 1.04)<br>0.21 (0.02, 2.02)<br>0.32 (0.13, 0.82)
24-hour measurement	-0.29 (-1.04, 0.46)	0.75 (0.35, 1.59)
SDI	-6.42 (-9.30, -3.54)	1.6 e -3 (9.1 e -5, 2.9 e -2)

Therefore, for females in households using solid fuel, we would expect their long-term mean excess $PM_{2.5}$ exposure due to the use of solid fuels to be 1522, 117, and 9 μg/m^3 in SDI of 0.1, 0.5, and 0.9, respectively.

Because there are so few studies of personal monitoring in men and children, rather than directly using the results of the model, we generated ratios using studies that measured at least two of the population groups for any size particulate matter. For $PM_{2.5}$ we used the predicted ambient $PM_{2.5}$ value in the study location and year based on the GBD 2017 $PM_{2.5}$ exposure model as the "outdoor" measurement, and for PM_4 and PM_{10} we used published values in the studies themselves. We first subtracted off this outdoor value from each PM measurement, and then calculated the ratio of male to female and child to female exposure, weighted by sample size.

Study	Location	Year	Pollutant	Female N	Female PM	Group	N	PM	Outdoor
Balakrishnan et al., 2004	Andhra Pradesh, Rural	2004	PM_4	591	352	male	503	187	94
Gao X et al., 2009.	Tibet	2009	$PM_{2.5}$	52	127	male	85	111	27
Dasgupta et al., 2006	Bangladesh	2006	PM_{10}	944	209	male	944	166	50
Devkumar et al., 2014	Nepal	2014	$PM_{2.5}$	405	169	male	429	167	90
Balakrishnan et al., 2004	Andhra Pradesh, Rural	2004	PM_4	591	352	child	56	262	94

Dionisio et al., 2008.	The Gambia	2008	$PM_{2.5}$	13	275	child	13	219	31
Dasgupta et al., 2006	Bangladesh	2006	PM_{10}	944	209	child	944	199	50

The final ratios were 0.64 95% CI (0.45, 0.91) for males and 0.85 95% CI (0.56, 1.31) for children. We used these results to scale the $PM_{2.5}$ mapping model for these age and sex groups to input into the $PM_{2.5}$ risk curves.

References

1. Smith KR, Bruce N, Balakrishnan K, Adair-Rohani H, Balmes J, Chafe Z, *et al*. Millions Dead: How Do We Know and What Does It Mean? Methods Used in the Comparative Risk Assessment of Household Air Pollution. Annu Rev Public Health. 2014; **35**(1):185–206.
2. Shupler M, Balakrishnan K, Ghosh S, *et al*. Global household air pollution database: Kitchen concentrations and personal exposures of particulate matter and carbon monoxide. Data in Brief 2018; **21**: 1292–5.
3. Shupler M, Godwin W, Frostad J, Gustafson P, Arku RE, Brauer M. Global estimation of exposure to fine particulate matter (PM2.5) from household air pollution. Environment International 2018; **120**: 354–63.
4. Tanchangya J, Geater AF. Use of traditional cooking fuels and the risk of young adult cataract in rural Bangladesh: a hospital-based case-control study. *BMC Ophthalmology* 2011; **11**.

Occupational risk factors

Flowcharts

Occupational Risk Factors
(except asbestos and injuries)

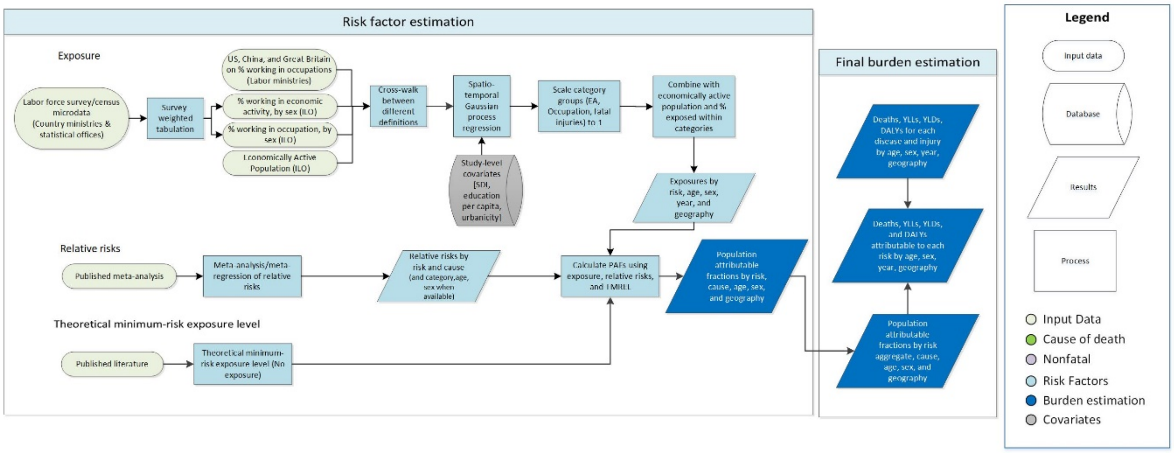

Occupational Risk Factors (Injuries)

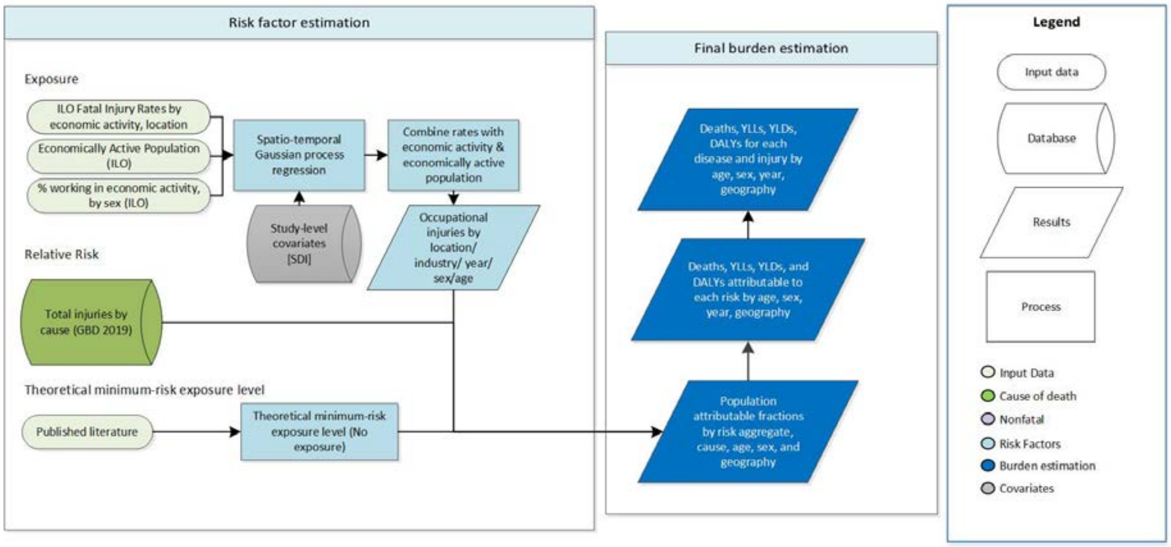

Occupational Risk Factors (asbestos)

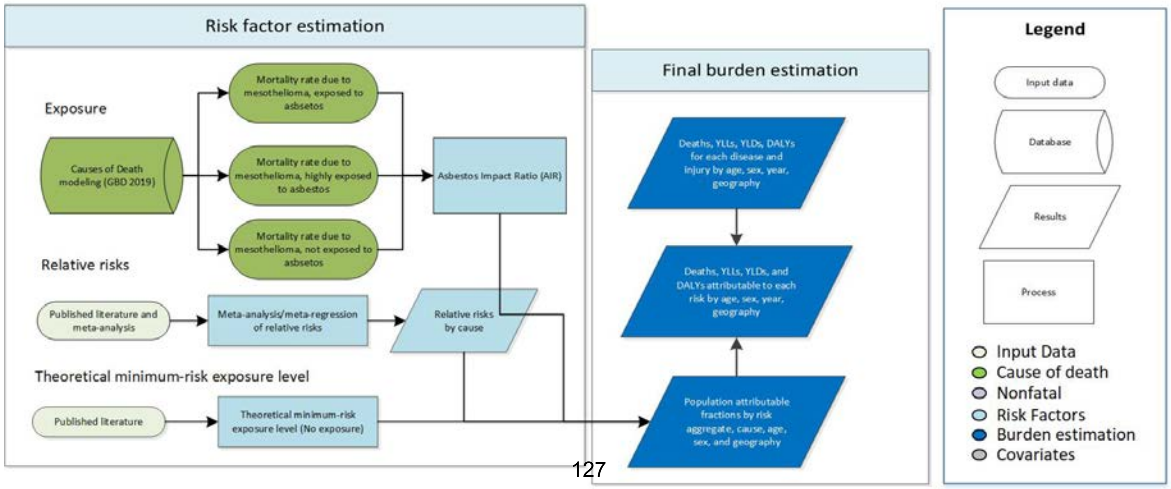

Exposure definitions

The following definitions were used for occupational risk factor exposures. All exposures were estimated for ages 15 and older.

Occupational asbestos	Proportion of the population occupationally exposed to asbestos, using mesothelioma death rate as an analogue
Occupational asthmagens	Proportion of the working population exposed to asthmagens, based on population distributions across nine occupational categories
Occupational carcinogens (arsenic, benzene, beryllium, cadmium, chromium, diesel engine exhaust, formaldehyde, nickel, polycyclic aromatic hydrocarbons, silica, sulfuric acid, and trichloroethylene)	Proportion of the population that was ever occupationally exposed to carcinogens at high or low exposure levels, based on population distributions across 17 economic activities
Occupational ergonomic factors	Proportion of the working population exposed to work that causes low back pain, based on population distributions across nine occupational categories
Occupational injuries	Proportion of injuries in the working-age population attributable to occupation, based on fatal injury rates in 17 economic activities
Occupational noise	Proportion of the population occupationally exposed to 85+ decibels of noise, based on population distributions across 17 economic activities
Occupational particulates	Proportion of the population occupationally exposed to particles, based on population distributions across 17 economic activities

Economic activities and occupations were coded according to the following categories:

**Economic activities**	**Occupations**
Agriculture, hunting, forestry	Legislators, senior officials, and managers
Fishing	Professionals
Mining and quarrying	Technicians and associate professionals
Manufacturing	Clerks
Electricity, gas, and water	Service workers and shop/market sales workers
Construction	Skilled agricultural and fishery workers
Wholesale and retail trade/repair	Plant and machine operators and assemblers
Hospitality	Craft and related workers
Transport, storage, and communication	Elementary occupations
Financial intermediation	
Real estate/renting	

Public administration/defense; compulsory social security
Education
Health and social work
Other community/social/personal service activities
Private households
Extra-territorial organisations/bodies

Input data

Primary inputs were obtained from the International Labour Organization (ILO).[1-4] These inputs included raw data on economic activity proportions, occupation proportions, fatal injury rates, and employment to population ratio estimates. No data on informal employment was included due to data sparseness. In 2017, a systematic review was conducted in order to collect the underlying microdata from the ILO's estimates to aid in re-extraction at greater levels of granularity. Where freely available, survey datasets were downloaded from the survey organisations in question. Other datasets were obtained through submission of requests to agencies and through the GBD collaborator network. Microdata were tabulated in order to create survey-weighted estimates of economic activities and occupations for the GBD geographies and years. Various classification systems were adjusted to match the ISIC Rev.3 classification (for economic activities) and ISCO 1988 classification (for occupations).

In 2019, a substantial amount of new ILO data were added. The new data comprise 1197 new unique location-years, including 174 unique locations and 13 unique years (2006–2018). Additionally, a number of old microdata were re-extracted.

For occupational asbestos, primary inputs were obtained through GBD 2019 cause of death estimates and published studies.[7,13,14]

Uncertainty for inputs where microdata were unavailable was generated by fitting a Loess curve to the data and determining the standard deviation of the data from the fitted curve.

Table 1 provides a summary of the exposure input data used.

Table 1: Data inputs for exposure

**Input data**	**Exposure**
Source count (total)	2485
Number of countries with data	199

Modelling strategies

A spatiotemporal Gaussian process regression (ST-GPR) was used to generate estimates for all years and locations for the primary inputs. Space-time parameters were chosen by maximising out-of-sample cross-validation and minimising RMSE. A number of different study-level covariates were used in the linear regression models. The linear models for each of the 46 different ST-GPR models used in occupational exposure estimation are listed below. Although there might appear to be duplicates, there is a distinction between occupation and economic activity (detailed in the footnotes). For example,

"skilled agriculture/fisheries" involves the proportion of the workforce doing agricultural and fishing work, while "agriculture, hunting, forestry" and "fishing" involve the proportion of the workforce employed in those respective industries (ie, one doesn't have to be actually doing agricultural or fishing work – someone who transports crops would count as being employed in this industry, but their occupation would fall under "plant and machine operators & assemblers"). Additionally, each model included random effects at the region and super-region levels. The covariates are explained in greater detail below.

ST-GPR model	Linear regression equation
Employment (% of population employed)	$logit(data) = gov\_exp + prop\_muslim + education$
Armed forces*	$logit(data) = sdi + education + urbanicity$
Management*	$logit(data) = sdi + education + urbanicity$
Professional occupations*	$logit(data) = sdi + education + urbanicity$
Scientific/technicians*	$logit(data) = sdi + education + urbanicity$
Clerical work*	$logit(data) = sdi + education + urbanicity$
Service & shop/market sales workers*	$logit(data) = sdi + education + urbanicity$
Skilled agriculture/fisheries*	$logit(data) = sdi + latitude + urbanicity$
Craft and related trades*	$logit(data) = sdi + education + urbanicity$
Plant and machine operators & assemblers*	$logit(data) = sdi + education + urbanicity$
Elementary occupations*	$logit(data) = sdi + education + urbanicity$
Agriculture, hunting, forestry†	$logit(data) = sdi + latitude + urbanicity$
Fishing†	$logit(data) = \log(coastal\_prop + 0.01)$
Mining/quarrying†	$logit(data) = sdi + \log(coastal\_prop + 0.01) + urbanicity + asbestos$
Manufacturing†	$logit(data) = sdi + education + urbanicity$
Electricity/gas/water supply†	$logit(data) = \log(sdi) + urbanicity + temperature$
Construction†	$logit(data) = sdi + urbanicity$
Wholesale and retail trade/repair†	$logit(data) = sdi + education + urbanicity$
Hospitality†	$logit(data) = sdi + urbanicity$
Transport/storage/communications†	$logit(data) = sdi + urbanicity + vehicles\_pc$
Financial intermediation†	$logit(data) = sdi + urbanicity$
Real estate/renting†	$logit(data) = sdi + urbanicity$
Public administration/defence†	$logit(data) = sdi + urbanicity$
Education†	$logit(data) = sdi + education + urbanicity$
Health and social work†	$logit(data) = \log(sdi) + \log(health\_exp)$
Other community/social/personal service activities†	$logit(data) = sdi + urbanicity$
Private households†	$logit(data) = sdi + urbanicity$
Extraterritorial organisations and bodies†	$logit(data) = sdi + urbanicity$
All occupational injuries models‡	$logit(data) = sdi$

**Proportion of workforce working this type of occupation*

†Proportion of workforce employed in this type of economic activity

‡There are 18 different models, corresponding to one for each type of economic activity and a "total" model

Covariate	Description
gov_exp	Total government expenditure
prop_muslim	Proportion of population that is Muslim
education	Age-standardised years of education per capita
sdi	Socio-demographic Index
urbanicity	Proportion of population living in urban areas
latitude	Absolute value of average latitude of country's center point
coastal_prop	Percentage of total country area within 10 km of a coastal zone
asbestos	Asbestos consumption (metric tons per year per capita)
temperature	Population-weighted mean temperature
vehicles_pc	Number of 2- and 4-wheeled vehicles per capita
health_exp	Total health expenditure per capita

For economic activity and occupation proportions, estimates from ST-GPR were then re-scaled to sum to 1 across categories by dividing each estimate by the sum of all the estimates.

The following sections describe the modelling approaches for each occupational risk's exposure prevalence. These approaches were developed for GBD 2016 and have not changed substantially since then. The GBD 2019 methods are largely the same as those from the previous cycle, with the exception of the data processing for occupational injuries, which is explained in greater detail in that section below.

Occupational carcinogens, occupational noise, and occupational particulates

Prevalence of exposure to these risks was determined using the following equation:

$$Prevalence\ of\ Exposure_{c,y,s,a,r,l} = \sum_{EA} Proportion_{EA,c,y} * EAP_{c,y,s,a} * Exposure\ rate_{EA,r,l,d}$$

where:

EAP = economically active population	c = country	r = risk
EA = economic activity	d = duration	s = sex
a = age	l = level of exposure	y = year

Exposure rate (proportion of population exposed) was provided by expert group recommendations and literature.[8-11] The CAREX (carcinogen exposure) database[7] was used in order to quantify the association between exposure by industry/carcinogen to SDI across all the countries in the database. This effect was used to predict exposure in countries that were not included in CAREX. Duration was considered for occupational carcinogens through application of occupational turnover factors[12] and for occupational noise and particulates by calculating cumulative exposure as the average exposure over the lifetime (the past 50 years) for each age/sex cohort.

Occupational ergonomic factors and occupational asthmagens

Prevalence of exposure to these risks was determined using the following equation:

$$Prevalence\ of\ Exposure_{c,y,s,a,r} = \sum_{EA} Proportion_{OCC,c,y} * EAP_{c,y,s,a}$$

where:

EAP = economically active population	c = country	r = risk
EA = economic activity	a = age	s = sex
OCC = occupation	y = year	

Occupational injuries

Occupational injury counts were estimated using the following equation:

$$Occupational\ fatal\ injuries_{c,y,a,s} = \sum_{EA} Injury\ rate_{EA,c,y,s} * Population_{c,y,a,s} * EAP_{c,y,s,a} * Proportion_{EA,c,y}$$

where:

EAP = economically active population	c = country	y = year
EA = economic activity	a = age	s = sex

Additionally, in GBD 2019, we updated our data processing to use MR-BRT to crosswalk our data. Occupational injuries exposure data come from a number of different sources: insurance records, labour inspectorate records, establishment surveys, establishment or business registers, labour force surveys, economic or establishment censuses, official estimates, records of employers' organizations, and other administrative records and related sources. We expect insurance records to be the gold-standard source, because people should have more incentive to report injuries when they stand to benefit from their insurance plans. As such, we wanted to correct the data reported from other sources for underreporting, and so we crosswalked all of the data with insurance records data as our reference. To do so, we ran a mixed-effects log-linear regression using MR-BRT, with fixed effects on type of data source and random effects on super-region and region. Table 2 shows the beta coefficients from the MR-BRT model, as well as the crosswalk adjustment factors.

Table 2: MR-BRT crosswalk adjustment factors for occupational injuries

Reference or alternative case definition	Gamma	Beta coefficient, log (95% CI)	Adjustment factor*
Reference (insurance records)	0.1	---	---
Alternative (labour inspectorate records)		0.09 (-0.10, 0.29)	0.91, (0.76, 1.13)
Alternative (establishment surveys)		-0.27 (-0.47, -0.08)	1.31 (1.10, 1.63)
Alternative (labour force surveys)		-0.85 (-1.05, -0.66)	2.34 (1.96, 2.91)
Alternative (economic or establishment censuses)		-0.07 (-0.26, 0.13)	1.07 (0.89, 1.33)
Alternative (official estimates)		-0.24 (-0.43, -0.04)	1.27 (1.06, 1.57)

Alternative (establishment or business registers)		-0.35 (-0.60, -0.09)	1.41 (1.13, 1.90)
Alternative (records of employers' organizations)		-0.99 (-1.19, -0.79)	2.69 (2.25, 3.35)
Alternative (other administrative records and related sources)		-0.45 (-0.64, -0.25)	1.56 (1.31, 1.94)

**Adjustment factor is the inverse transformed beta coefficient in normal space and can be interpreted as the factor by which the alternative case definition is adjusted to reflect what it would have been if measured as the reference.*

Occupational asbestos

Prevalence of exposure to asbestos was estimated using the asbestos impact ratio (AIR), which is equivalent to the excess deaths due to mesothelioma observed in a population divided by excess deaths due to mesothelioma in a population heavily exposed to asbestos. Formally, this is defined using the following equation:

$$AIR = \frac{Mort_{c,y,s} - N_{c,y,s}}{Mort^{*}_{c,ys,} - N_{c,y,s}}$$

where:

Mort = Mortality rate due to mesothelioma	c = country
Mort* = Mortality rate due to mesothelioma in population highly exposed to asbestos	y = year
N = Mortality rate due to mesothelioma in population not exposed to asbestos	s = sex

Mortality rate due to mesothelioma was estimated using GBD 2019 causes of death results. Mortality rate due to mesothelioma in populations not exposed to asbestos was calculated using the model in Lin and colleagues,[13] while the mortality rate due to high exposure to asbestos was estimated using Goodman and colleagues' model.[14] Asbestos exposure prevalence created using the AIR was used to estimate population attributable fractions (PAFs) for all asbestos-associated causes except for mesothelioma. Custom PAFs were calculated for mesothelioma by using the ratio of the excess mortality with respect to an unexposed population (Mort – N) divided by the mortality rate in the population in question (Mort). This calculation assumes that all mesothelioma is a product of occupational asbestos exposure and could potentially overestimate the burden due to occupational asbestos exposure in populations with high non-occupational asbestos exposure.

Theoretical minimum-risk exposure level

For all occupational risks, the theoretical minimum-risk exposure level was assumed to be no exposure to that risk.

Relative risks

Relative risks were obtained for all occupational risks by conducting a systematic review of published meta-analyses. This review was last updated for GBD 2016. Table 3 provides a summary of the relative risk input data used.

Table 3: Data inputs for relative risks

Input data	Relative risk
Source count (total)	21

Population attributable fractions (PAFs)

For all occupational risks, with the exception of injuries (outlined below) and asbestos (outlined above), PAFs were calculated using the exposure prevalences estimated above, using the PAF formula in outlined in the GBD 2019 methods appendix.

Occupational injuries PAF

The PAFs for occupational injuries were calculated using the following formula:

$$PAF_{c,y,a,s} = \frac{Occupational\ fatal\ injuries_{c,y,a,s} - TMREL}{Fatal\ injuries_{c,y,a,s}}$$

where:

c = country
y = year
a = age
s = sex

Fatal injury totals were obtained from GBD 2019 causes of death.

References

1. International Labour Organization (ILO). International Labour Organization Database (ILOSTAT) - Employment by Sex and Economic Activity. International Labour Organization (ILO).

2. International Labour Organization (ILO). International Labour Organization Database (ILOSTAT) - Employment by Sex and Occupation. International Labour Organization (ILO).

3. International Labour Organization (ILO). International Labour Organization Database (ILOSTAT) - Fatal Injuries by Sex and Economic Activity. International Labour Organization (ILO).

4. International Labour Organization (ILO). International Labour Organization LABORSTA Economically Active Population, Estimates and Projections, October 2011. International Labour Organization (ILO), 2011.

5. Office for National Statistics (United Kingdom). Nomis Official Labor Market Statistics - Annual Population Survey. Newport, United Kingdom: Office for National Statistics (United Kingdom).

6. National Bureau of Statistics of China. China 1% National Population Sample Survey 1995. Ann Arbor, United States: China Data Center, University of Michigan.

7. Kauppinen T, Toikkanen J, Pedersen D, *et al.* Occupational exposure to carcinogens in the European Union. *Occupational and Environmental Medicine* 2000; **57**: 10–8.

8. Wilson DH, Walsh PG, Sanchez L, *et al.* The epidemiology of hearing impairment in an Australian adult population. *Int J Epidemiol* 1999; 28: 247–52

9. Kauppinen T, Toikkanen J, Pederson D, Young R, Kogevinas M, Ahrens W, et al. Occupational Exposure to Carcinogens in the European Union in 1990-93. Helsinki, Finland: Finnish Institute of Occupational Health; 1998.

10. Kauppinen T, Toikkanen J, Pedersen D, Young R, Ahrens W, Boffetta P, et al. Occupational exposure to carcinogens in the European Union. *Occup Environ Med* 2000; 57(1): 10–18.

11. Driscoll T, et al. The global burden of non-malignant respiratory disease due to occupational airborne exposures. *American Journal of Industrial Medicine 2005; 48*(6): 432-445.

12. Nelson, D. I., Concha-Barrientos, M., Driscoll, T., Steenland, K., Fingerhut, M., Punnett, L. & Corvalan, C. (2005). The global burden of selected occupational diseases and injury risks: Methodology and summary. *American journal of industrial medicine*, *48*(6), 400-418

13. Lin R-T, Takahashi K, Karjalainen A, *et al.* Ecological association between asbestos-related diseases and historical asbestos consumption: an international analysis. *Lancet* 2007; **369**: 844–9.

14. Goodman M, Morgan RW, Ray R, Malloy CD, Zhao K. Cancer in asbestos-exposed occupational cohorts: a meta-analysis. *Cancer Causes Control* 1999; **10**: 453–65.

Calculating the burden of multiple risk factors

Validation studies have reported congruency between the true risk associated with multiple risk factors affecting the same outcome and a multiplicative aggregation of the PAFs of the individual risk factors (formula below).[1]

$$PAF_{1..i} = 1 - \prod_{i=1}^{n}(1 - PAF_i)$$

where *PAF* is the population attributable fraction and *i* is each individual risk factor.

The same validation studies also found that the overestimation from ignoring the covariance between risk factors is small. This small overestimation was important to note because few data sources exist from which we can draw information on covariance.

Reference

Lim, S.S., Carnahan, E., Nelson, E.C. et al. Validation of a new predictive risk model: measuring the impact of the major modifiable risks of death for patients and populations. Population Health Metrics 13, 27 (2015). https://doi.org/10.1186/s12963-015-0059-8

Inclusion of a risk–outcome pair in the GBD

Inclusion of a risk–outcome pair in the GBD was determined based on the World Cancer Research Fund criteria for convincing or probable evidence.[1,2] Convincing evidence requires the following: evidence from more than one study type; evidence from at least two independent cohort studies; no substantial unexplained heterogeneity within or between study types; good-quality studies to exclude the possibility of confounding, measurement error, and selection bias; and a biologically plausible dose–response relationship. Probable evidence requires: evidence from at least two independent cohort studies or five or more case-control studies; no substantial unexplained heterogeneity within or between study types; good-quality studies to rule out the possibility of confounding, measurement error, and selection bias; and biological plausibility.

The GBD also needed evidence based on past research on the likelihood of importance of a risk factor to disease burden or policy; the availability of adequate data and methodologies for estimating exposure distributions by country; adequate data to estimate outcome-specific effect sizes per unit of exposure; and evidence that effect sizes may be extrapolated to populations other than those studied in epidemiological studies or methods for doing so.[3] Lastly, for a newly evaluated risk–outcome pair, the GBD needed a statistically significant association ($p<0{\cdot}05$) after accounting for sources of potential bias.[4]

Reference

1. World Cancer Research Fund / American Institute for Cancer Research. Judging the evidence. https://www.wcrf.org/wp-content/uploads/2021/02/judging-the-evidence.pdf (accessed May 12, 2022).

2. World Cancer Research Fund/ American Institute for Cancer Research. Food, Nutrition, and Physical Activity, and the Prevention of Cancer. A Global Perspective. Washington D.C, 2007.

3. Lim SS, Vos T, Flaxman AD, et al. A comparative risk assessment of burden of disease and injury attributable to 67 risk factors and risk factor clusters in 21 regions, 1990–2010: a systematic analysis for the Global Burden of Disease Study 2010. The lancet 2013; 380(9859): 2224-60.

4. GBD 2019 Risk Factors Collaborators. Global burden of 87 risk factors in 204 countries and territories, 1990-2019: a systematic analysis for the Global Burden of Disease Study 2019. Lancet 2020; 396(10258): 1223-49.

Supplementary Tables and Figures

Table S1. GATHER checklist

#	GATHER checklist item	Description of Compliance	Reference
**Objectives and funding**			
1	Define the indicators, populations, and time periods for which estimates were made.	Narrative provided in paper and methods appendix describing indicators, definitions, and populations	Main text (Methods) and methodsappendix
2	List the funding sources for the work.	Funding sources listed in paper	Summary (Funding)
**Data Inputs**			
For all data inputs from multiple sources that are synthesized as part of the study:			
3	Describe how the data were identified and how the data were accessed.	Narrative description of data seeking methods provided	Methods appendix
4	Specify the inclusion and exclusion criteria. Identify all ad-hoc exclusions.	Narrative about inclusion and exclusion criteria by data type provided	Methods appendix
5	Provide information on all included data sources and their main characteristics. For each data source used, report reference information or contact name/institution, population represented, data collection method, year(s) of data collection, sex and age range, diagnostic criteria or measurement method, and sample size, as relevant.	An interactive, online data source tool that provides metadata for datasources by component, geography, cause, risk, or impairment has beendeveloped	Online data citation tools http://ghdx.healthdata.org/gbd-2019
6	Identify and describe any categories of input data that have potentially important biases (e.g., based on characteristics listed in item 5).	Summary of known biases bycause included in methods appendix	Methods appendix
For data inputs that contribute to the analysis but were not synthesized as part of the study:			
7	Describe and give sources for any other data inputs.	Included in online data source tool, http://ghdx.healthdata. org/gbd- 2019	Online data citation tools
**For all data inputs:**			
8	Provide all data inputs in a file format from which data can be efficiently extracted (e.g.,a spreadsheet as opposed to a PDF), including all relevant meta-data listed in item 5. For any data inputs that cannot be shared due to ethical or legal reasons, such as third-party ownership, provide a contact name or the name of the institution that retains the right to the data.	Downloads of input data available through online tools, including data visualization tools and data query tools, http://ghdx.healthdata.org/gbd-2019; input data not available in tools will be made available upon request	Online data visualization tools, dataquery tools, and the Global Health Data Exchange: http://ghdx.healthdata.org
**Data analysis**			
9	Provide a conceptual overview of the data analysis method. A diagram may be helpful.	Flow diagrams of the overall methodological processes, as wellas cause-specific modelling processes, have been provided	Methods appendix

**10**	Provide a detailed description of all steps of the analysis, including mathematical formulae. This description should cover, as relevant, data cleaning, data pre-processing, data adjustments and weighting of data sources, and mathematical or statistical model(s).	Flow diagrams and corresponding methodological writeups for each cause, as well as the demographicsand causes of death databases and modelling processes, have been provided	Main text (Methods) and methodsappendix
**11**	Describe how candidate models were evaluated and how the final model(s) were selected.	Provided in the methodological writeups	Methods appendix
**12**	Provide the results of an evaluation of modelperformance, if done, as well as the results of any relevant sensitivity analysis.	Provided in the methodological writeups	Methods appendix
**13**	Describe methods for calculating uncertaintyof the estimates. State which sources of uncertainty were, and were not, accounted for in the uncertainty analysis.	Provided in the methodological writeups	Methods appendix
**14**	State how analytic or statistical source code used to generate estimates can be accessed.	Access statement provided	Code is provided in an online repository: http://ghdx.healthdata.org/gbd-2019/code
**Results and Discussion**			
**15**	Provide published estimates in a file format from which data can be efficiently extracted.	GBD 2019 results are available through online data visualization tools, the Global Health Data Exchange, and the online dataquery tool	Main text, methods appendix,aapendices 2-4, and online data tools http://ghdx.healthdata.org/gbd-2019
**16**	Report a quantitative measure of the uncertainty of the estimates (e.g. uncertaintyintervals).	Uncertainty intervals are provided with all results	Main text, methods appendix, aapendices 2-4, and online data tools http://ghdx.healthdata.org/gbd-2019
**17**	Interpret results in light of existing evidence. If updating a previous set of estimates, describe the reasons for changes in estimates.	Discussion of methodological changes between GBD rounds provided in the narrative of theArticle and methods appendix	Main text (Methods and Discussion) and methods appendix
**18**	Discuss limitations of the estimates. Include a discussion of any modelling assumptions or data limitations that affect interpretation of the estimates.	Discussion of limitations providedin the narrative of the main paper, as well as in the methodological write-ups in the methods appendix	Main text (Discussion) and methodsappendix

Table S2. List of International Classification of Diseases (ICD) codes mapped to the Global Burden of Disease cause list for causes of death

Cause	ICD10	ICD9
**Chronic respiratory diseases**	D86-D86.2, D86.9, G47.3, J30-J35.9, J37-J39.9, J41-J46.9, J60-J63.8, J65-J68.9, J70, J70.8-J70.9, J82, J84-J84.9, J91, J91.8-J92.9	135-135.9, 327.2-327.8, 470, 470.9-474.9, 476-476.1, 477-479, 491-493.9, 495-504.9, 506-506.9, 508-509, 515, 516-517.8, 518.6, 518.9, 519.1-519.4
**Chronic obstructive pulmonary disease**	J41-J44.9	491-492.9, 496-499
**Pneumoconiosis**	J60-J63.8, J65-J65.0, J92.0	500-504.9
**Silicosis**	J62-J62.9	502-502.9, 503.0, 503.9
**Asbestosis**	J61-J61.0, J92.0	501
**Coal workers pneumoconiosis**	J60-J60.0	500-500.9, 501.0-501.9
**Other pneumoconiosis**	J63-J63.8, J65-J65.0	503, 503.1, 504-504.9
**Asthma**	J45-J46.9	493-493.9
**Interstitial lung disease and pulmonary sarcoidosis**	D86-D86.2, D86.9, J84-J84.9	135-135.9, 515, 516-516.9
**Other chronic respiratory diseases**	G47.3, J30-J35.9, J37-J39.9, J66-J68.9, J70, J70.8-J70.9, J82, J91, J91.8-J92, J92.9	327.2-327.8, 470, 470.9-474.9, 476-476.1, 477-479, 495-495.9, 506-506.9, 508-509, 517-517.8, 518.6, 518.9, 519.1-519.4

Table S3. Restrictions on age and sex by cause for GBD 2019

Cause	Minimum age	Maximum age	Sex Restrictions
**Chronic respiratory diseases**	28 days	Not applied	Not applied
**Chronic obstructive pulmonary disease**	28 days	Not applied	Not applied
**Pneumoconiosis**	15 years	Not applied	Not applied
**Silicosis**	15 years	Not applied	Not applied
**Asbestosis**	15 years	Not applied	Not applied
**Coal workers pneumoconiosis**	15 years	Not applied	Not applied
**Other pneumoconiosis**	15 years	Not applied	Not applied
**Asthma**	1 year	Not applied	Not applied
**Interstitial lung disease and pulmonary sarcoidosis**	1 year	Not applied	Not applied
**Other chronic respiratory diseases**	Not applied	Not applied	Not applied

Table S4. Total number of site years by cause and source type for 2019

Cause	Level	Vital Registration	Vital Registration - Sample	Verbal Autopsy	Surveillance
**Chronic respiratory diseases**	2	22568	825	1515	549
**Chronic obstructive pulmonary disease**	3	20371	825		
**Pneumoconiosis**	3	20365	448		
**Silicosis**	4	20216	448		
**Asbestosis**	4	20216	448		
**Coal workers pneumoconiosis**	4	20216	448		
**Other pneumoconiosis**	4	20216	448		
**Asthma**	3	20364	448		
**Interstitial lung disease and pulmonary sarcoidosis**	3	20361	448		
**Other chronic respiratory diseases**	3	20371	825		

Table S5. CODEm covariates used, level of covariate, and expected direction of covariate by cause, sex, and age (*Covariates that CODEm did not select during the covariate selection process have no draw counts listed.)*

Cause	Sex	Age Start	Age End	Model Type	Direction	Level	Covariate Name	Number of Draws
Chronic respiratory diseases	Female	1-4 years	95+ years	Data Rich	1	1	Cumulative Cigarettes (10 Years)	26
Chronic respiratory diseases	Female	1-4 years	95+ years	Data Rich	1	1	Cumulative Cigarettes (5 Years)	48
Chronic respiratory diseases	Female	1-4 years	95+ years	Data Rich	1	1	Healthcare access and quality index	--
Chronic respiratory diseases	Female	1-4 years	95+ years	Data Rich	1	1	Log-transformed SEV scalar: Chr Resp	--
Chronic respiratory diseases	Female	1-4 years	95+ years	Data Rich	1	2	Smoking Prevalence	3
Chronic respiratory diseases	Female	1-4 years	95+ years	Data Rich	1	2	Indoor Air Pollution (All Cooking Fuels)	120
Chronic respiratory diseases	Female	1-4 years	95+ years	Data Rich	1	2	Outdoor Air Pollution (PM2.5)	242
Chronic respiratory diseases	Female	1-4 years	95+ years	Data Rich	1	2	Elevation Over 1500m (proportion)	365
Chronic respiratory diseases	Female	1-4 years	95+ years	Data Rich	-1	3	Education (years per capita)	142
Chronic respiratory diseases	Female	1-4 years	95+ years	Data Rich	-1	3	LDI (I$ per capita)	--
Chronic respiratory diseases	Female	1-4 years	95+ years	Data Rich	0	3	Socio-demographic Index	246
Chronic respiratory diseases	Female	1-4 years	95+ years	Data Rich	1	3	Population Density (over 1000 ppl/sqkm, proportion)	0
Chronic respiratory diseases	Female	1-4 years	95+ years	Data Rich	1	3	Elevation 500 to 1500m (proportion)	434
Chronic respiratory diseases	Female	1-4 years	95+ years	Global	1	1	Cumulative Cigarettes (10 Years)	22
Chronic respiratory diseases	Female	1-4 years	95+ years	Global	1	1	Cumulative Cigarettes (5 Years)	23
Chronic respiratory diseases	Female	1-4 years	95+ years	Global	1	1	Healthcare access and quality index	--
Chronic respiratory diseases	Female	1-4 years	95+ years	Global	1	1	Log-transformed SEV scalar: Chr Resp	--
Chronic respiratory diseases	Female	1-4 years	95+ years	Global	1	2	Smoking Prevalence	11
Chronic respiratory diseases	Female	1-4 years	95+ years	Global	1	2	Elevation Over 1500m (proportion)	622
Chronic respiratory diseases	Female	1-4 years	95+ years	Global	1	2	Indoor Air Pollution (All Cooking Fuels)	622
Chronic respiratory diseases	Female	1-4 years	95+ years	Global	1	2	Outdoor Air Pollution (PM2.5)	--
Chronic respiratory diseases	Female	1-4 years	95+ years	Global	-1	3	Education (years per capita)	400
Chronic respiratory diseases	Female	1-4 years	95+ years	Global	-1	3	LDI (I$ per capita)	--
Chronic respiratory diseases	Female	1-4 years	95+ years	Global	0	3	Socio-demographic Index	153
Chronic respiratory diseases	Female	1-4 years	95+ years	Global	1	3	Elevation 500 to 1500m (proportion)	684
Chronic respiratory diseases	Female	1-4 years	95+ years	Global	1	3	Population Density (over 1000 ppl/sqkm, proportion)	--

Cause	Sex	Age Start	Age End	Model Type	Direction	Level	Covariate Name	Number of Draws
Chronic respiratory diseases	Male	1-4 years	95+ years	Data Rich	1	1	Cumulative Cigarettes (10 Years)	337
Chronic respiratory diseases	Male	1-4 years	95+ years	Data Rich	1	1	Cumulative Cigarettes (5 Years)	526
Chronic respiratory diseases	Male	1-4 years	95+ years	Data Rich	1	1	Log-transformed SEV scalar: Chr Resp	--
Chronic respiratory diseases	Male	1-4 years	95+ years	Data Rich	-1	2	Healthcare access and quality index	2
Chronic respiratory diseases	Male	1-4 years	95+ years	Data Rich	1	2	Indoor Air Pollution (All Cooking Fuels)	0
Chronic respiratory diseases	Male	1-4 years	95+ years	Data Rich	1	2	Smoking Prevalence	775
Chronic respiratory diseases	Male	1-4 years	95+ years	Data Rich	1	2	Elevation Over 1500m (proportion)	777
Chronic respiratory diseases	Male	1-4 years	95+ years	Data Rich	1	2	Outdoor Air Pollution (PM2.5)	777
Chronic respiratory diseases	Male	1-4 years	95+ years	Data Rich	-1	3	Education (years per capita)	140
Chronic respiratory diseases	Male	1-4 years	95+ years	Data Rich	-1	3	LDI (I$ per capita)	--
Chronic respiratory diseases	Male	1-4 years	95+ years	Data Rich	0	3	Socio-demographic Index	116
Chronic respiratory diseases	Male	1-4 years	95+ years	Data Rich	1	3	Population Density (over 1000 ppl/sqkm, proportion)	158
Chronic respiratory diseases	Male	1-4 years	95+ years	Data Rich	1	3	Elevation 500 to 1500m (proportion)	484
Chronic respiratory diseases	Male	1-4 years	95+ years	Global	1	1	Cumulative Cigarettes (10 Years)	75
Chronic respiratory diseases	Male	1-4 years	95+ years	Global	1	1	Indoor Air Pollution (All Cooking Fuels)	165
Chronic respiratory diseases	Male	1-4 years	95+ years	Global	1	1	Cumulative Cigarettes (5 Years)	234
Chronic respiratory diseases	Male	1-4 years	95+ years	Global	1	1	Smoking Prevalence	296
Chronic respiratory diseases	Male	1-4 years	95+ years	Global	1	1	Log-transformed SEV scalar: Chr Resp	--
Chronic respiratory diseases	Male	1-4 years	95+ years	Global	-1	2	Healthcare access and quality index	253
Chronic respiratory diseases	Male	1-4 years	95+ years	Global	1	2	Outdoor Air Pollution (PM2.5)	0
Chronic respiratory diseases	Male	1-4 years	95+ years	Global	1	2	Elevation Over 1500m (proportion)	548
Chronic respiratory diseases	Male	1-4 years	95+ years	Global	-1	3	Education (years per capita)	467
Chronic respiratory diseases	Male	1-4 years	95+ years	Global	-1	3	LDI (I$ per capita)	--
Chronic respiratory diseases	Male	1-4 years	95+ years	Global	0	3	Socio-demographic Index	262
Chronic respiratory diseases	Male	1-4 years	95+ years	Global	1	3	Population Density (over 1000 ppl/sqkm, proportion)	202
Chronic respiratory diseases	Male	1-4 years	95+ years	Global	1	3	Elevation 500 to 1500m (proportion)	846
Chronic obstructive pulmonary disease	Female	1-4 years	95+ years	Data Rich	1	1	Outdoor Air Pollution (PM2.5)	446
Chronic obstructive pulmonary disease	Female	1-4 years	95+ years	Data Rich	1	1	Elevation Over 1500m (proportion)	678
Chronic obstructive	Female	1-4 years	95+ years	Data Rich	1	1	Healthcare access and	706

Cause	Sex	Age Start	Age End	Model Type	Direct ion	Level	Covariate Name	Number of Draws
pulmonary disease							quality index	
Chronic obstructive pulmonary disease	Female	1-4 years	95+ years	Data Rich	1	1	Cumulative Cigarettes (10 Years)	--
Chronic obstructive pulmonary disease	Female	1-4 years	95+ years	Data Rich	1	1	Cumulative Cigarettes (5 Years)	--
Chronic obstructive pulmonary disease	Female	1-4 years	95+ years	Data Rich	1	1	Log-transformed SEV scalar: COPD	--
Chronic obstructive pulmonary disease	Female	1-4 years	95+ years	Data Rich	1	2	Indoor Air Pollution (All Cooking Fuels)	1
Chronic obstructive pulmonary disease	Female	1-4 years	95+ years	Data Rich	1	2	Smoking Prevalence	3
Chronic obstructive pulmonary disease	Female	1-4 years	95+ years	Data Rich	-1	3	Education (years per capita)	2
Chronic obstructive pulmonary disease	Female	1-4 years	95+ years	Data Rich	-1	3	LDI (I$ per capita)	--
Chronic obstructive pulmonary disease	Female	1-4 years	95+ years	Data Rich	0	3	Socio-demographic Index	782
Chronic obstructive pulmonary disease	Female	1-4 years	95+ years	Global	1	1	Healthcare access and quality index	588
Chronic obstructive pulmonary disease	Female	1-4 years	95+ years	Global	1	1	Elevation Over 1500m (proportion)	803
Chronic obstructive pulmonary disease	Female	1-4 years	95+ years	Global	1	1	Cumulative Cigarettes (10 Years)	--
Chronic obstructive pulmonary disease	Female	1-4 years	95+ years	Global	1	1	Cumulative Cigarettes (5 Years)	--
Chronic obstructive pulmonary disease	Female	1-4 years	95+ years	Global	1	1	Log-transformed SEV scalar: COPD	--
Chronic obstructive pulmonary disease	Female	1-4 years	95+ years	Global	1	1	Outdoor Air Pollution (PM2.5)	--
Chronic obstructive pulmonary disease	Female	1-4 years	95+ years	Global	1	2	Smoking Prevalence	3
Chronic obstructive pulmonary disease	Female	1-4 years	95+ years	Global	1	2	Indoor Air Pollution (All Cooking Fuels)	206
Chronic obstructive pulmonary disease	Female	1-4 years	95+ years	Global	-1	3	Education (years per capita)	35
Chronic obstructive pulmonary disease	Female	1-4 years	95+ years	Global	-1	3	LDI (I$ per capita)	--
Chronic obstructive pulmonary disease	Female	1-4 years	95+ years	Global	0	3	Socio-demographic Index	140
Chronic obstructive pulmonary disease	Male	1-4 years	95+ years	Data Rich	1	1	Cumulative Cigarettes (10 Years)	0
Chronic obstructive pulmonary disease	Male	1-4 years	95+ years	Data Rich	1	1	Cumulative Cigarettes (20 Years)	0
Chronic obstructive pulmonary disease	Male	1-4 years	95+ years	Data Rich	1	1	Cumulative Cigarettes (5 Years)	0
Chronic obstructive pulmonary disease	Male	1-4 years	95+ years	Data Rich	1	1	Elevation Over 1500m (proportion)	485
Chronic obstructive pulmonary disease	Male	1-4 years	95+ years	Data Rich	1	1	Outdoor Air Pollution (PM2.5)	551
Chronic obstructive pulmonary disease	Male	1-4 years	95+ years	Data Rich	1	1	Log-transformed SEV scalar: COPD	--
Chronic obstructive pulmonary disease	Male	1-4 years	95+ years	Data Rich	-1	2	Healthcare access and quality index	0
Chronic obstructive pulmonary disease	Male	1-4 years	95+ years	Data Rich	1	2	Indoor Air Pollution (All Cooking Fuels)	0

Cause	Sex	Age Start	Age End	Model Type	Direct ion	Level	Covariate Name	Number of Draws
Chronic obstructive pulmonary disease	Male	1-4 years	95+ years	Data Rich	1	2	Smoking Prevalence	488
Chronic obstructive pulmonary disease	Male	1-4 years	95+ years	Data Rich	-1	3	Education (years per capita)	813
Chronic obstructive pulmonary disease	Male	1-4 years	95+ years	Data Rich	-1	3	LDI (I$ per capita)	--
Chronic obstructive pulmonary disease	Male	1-4 years	95+ years	Data Rich	0	3	Socio-demographic Index	940
Chronic obstructive pulmonary disease	Male	1-4 years	95+ years	Global	1	1	Cumulative Cigarettes (20 Years)	0
Chronic obstructive pulmonary disease	Male	1-4 years	95+ years	Global	1	1	Cumulative Cigarettes (10 Years)	4
Chronic obstructive pulmonary disease	Male	1-4 years	95+ years	Global	1	1	Cumulative Cigarettes (5 Years)	7
Chronic obstructive pulmonary disease	Male	1-4 years	95+ years	Global	1	1	Outdoor Air Pollution (PM2.5)	124
Chronic obstructive pulmonary disease	Male	1-4 years	95+ years	Global	1	1	Elevation Over 1500m (proportion)	939
Chronic obstructive pulmonary disease	Male	1-4 years	95+ years	Global	1	1	Log-transformed SEV scalar: COPD	--
Chronic obstructive pulmonary disease	Male	1-4 years	95+ years	Global	-1	2	Healthcare access and quality index	477
Chronic obstructive pulmonary disease	Male	1-4 years	95+ years	Global	1	2	Indoor Air Pollution (All Cooking Fuels)	71
Chronic obstructive pulmonary disease	Male	1-4 years	95+ years	Global	1	2	Smoking Prevalence	380
Chronic obstructive pulmonary disease	Male	1-4 years	95+ years	Global	-1	3	Education (years per capita)	437
Chronic obstructive pulmonary disease	Male	1-4 years	95+ years	Global	-1	3	LDI (I$ per capita)	--
Chronic obstructive pulmonary disease	Male	1-4 years	95+ years	Global	0	3	Socio-demographic Index	664
Pneumoconiosis	Female	15-19 years	95+ years	Data Rich	1	1	Asbestos consumption (metric tons per year per capita)	260
Pneumoconiosis	Female	15-19 years	95+ years	Data Rich	1	1	Coal Production (per capita)	--
Pneumoconiosis	Female	15-19 years	95+ years	Data Rich	1	1	Gold production (kg) per capita	--
Pneumoconiosis	Female	15-19 years	95+ years	Data Rich	-1	2	Healthcare access and quality index	43
Pneumoconiosis	Female	15-19 years	95+ years	Data Rich	1	2	Cumulative Cigarettes (5 Years)	38
Pneumoconiosis	Female	15-19 years	95+ years	Data Rich	1	2	Indoor Air Pollution (All Cooking Fuels)	38
Pneumoconiosis	Female	15-19 years	95+ years	Data Rich	1	2	Smoking Prevalence	404
Pneumoconiosis	Female	15-19 years	95+ years	Data Rich	-1	3	Education (years per capita)	106
Pneumoconiosis	Female	15-19 years	95+ years	Data Rich	-1	3	Socio-demographic Index	430
Pneumoconiosis	Female	15-19 years	95+ years	Data Rich	-1	3	LDI (I$ per capita)	--
Pneumoconiosis	Female	15-19 years	95+ years	Global	1	1	Asbestos consumption (metric tons per	1000

Cause	Sex	Age Start	Age End	Model Type	Direction	Level	Covariate Name	Number of Draws
							year per capita)	
Pneumoconiosis	Female	15-19 years	95+ years	Global	1	1	Coal Production (per capita)	--
Pneumoconiosis	Female	15-19 years	95+ years	Global	1	1	Gold production (kg) per capita	--
Pneumoconiosis	Female	15-19 years	95+ years	Global	-1	2	Healthcare access and quality index	190
Pneumoconiosis	Female	15-19 years	95+ years	Global	1	2	Cumulative Cigarettes (5 Years)	24
Pneumoconiosis	Female	15-19 years	95+ years	Global	1	2	Indoor Air Pollution (All Cooking Fuels)	24
Pneumoconiosis	Female	15-19 years	95+ years	Global	1	2	Smoking Prevalence	193
Pneumoconiosis	Female	15-19 years	95+ years	Global	-1	3	Socio-demographic Index	144
Pneumoconiosis	Female	15-19 years	95+ years	Global	-1	3	Education (years per capita)	266
Pneumoconiosis	Female	15-19 years	95+ years	Global	-1	3	LDI (I$ per capita)	--
Pneumoconiosis	Male	15-19 years	95+ years	Data Rich	1	1	Asbestos consumption (metric tons per year per capita)	365
Pneumoconiosis	Male	15-19 years	95+ years	Data Rich	1	1	Coal Production (per capita)	805
Pneumoconiosis	Male	15-19 years	95+ years	Data Rich	1	1	Gold production (kg) per capita	--
Pneumoconiosis	Male	15-19 years	95+ years	Data Rich	-1	2	Healthcare access and quality index	34
Pneumoconiosis	Male	15-19 years	95+ years	Data Rich	1	2	Indoor Air Pollution (All Cooking Fuels)	0
Pneumoconiosis	Male	15-19 years	95+ years	Data Rich	1	2	Smoking Prevalence	154
Pneumoconiosis	Male	15-19 years	95+ years	Data Rich	1	2	Cumulative Cigarettes (5 Years)	167
Pneumoconiosis	Male	15-19 years	95+ years	Data Rich	-1	3	Socio-demographic Index	29
Pneumoconiosis	Male	15-19 years	95+ years	Data Rich	-1	3	Education (years per capita)	323
Pneumoconiosis	Male	15-19 years	95+ years	Data Rich	-1	3	LDI (I$ per capita)	--
Pneumoconiosis	Male	15-19 years	95+ years	Global	1	1	Coal Production (per capita)	567
Pneumoconiosis	Male	15-19 years	95+ years	Global	1	1	Asbestos consumption (metric tons per year per capita)	750
Pneumoconiosis	Male	15-19 years	95+ years	Global	1	1	Gold production (kg) per capita	--
Pneumoconiosis	Male	15-19 years	95+ years	Global	-1	2	Healthcare access and quality index	160
Pneumoconiosis	Male	15-19 years	95+ years	Global	1	2	Indoor Air Pollution (All Cooking Fuels)	2
Pneumoconiosis	Male	15-19 years	95+ years	Global	1	2	Smoking Prevalence	126
Pneumoconiosis	Male	15-19 years	95+ years	Global	1	2	Cumulative Cigarettes (5 Years)	154

Cause	Sex	Age Start	Age End	Model Type	Direction	Level	Covariate Name	Number of Draws
Pneumoconiosis	Male	15-19 years	95+ years	Global	-1	3	Education (years per capita)	299
Pneumoconiosis	Male	15-19 years	95+ years	Global	-1	3	Socio-demographic Index	413
Pneumoconiosis	Male	15-19 years	95+ years	Global	-1	3	LDI (I$ per capita)	--
Silicosis	Female	15-19 years	95+ years	Data Rich	1	1	Gold production (kg) per capita	--
Silicosis	Female	15-19 years	95+ years	Data Rich	-1	2	Healthcare access and quality index	568
Silicosis	Female	15-19 years	95+ years	Data Rich	1	2	Cumulative Cigarettes (5 Years)	--
Silicosis	Female	15-19 years	95+ years	Data Rich	1	2	Indoor Air Pollution (All Cooking Fuels)	--
Silicosis	Female	15-19 years	95+ years	Data Rich	1	2	Smoking Prevalence	--
Silicosis	Female	15-19 years	95+ years	Data Rich	-1	3	Socio-demographic Index	568
Silicosis	Female	15-19 years	95+ years	Data Rich	-1	3	Education (years per capita)	--
Silicosis	Female	15-19 years	95+ years	Data Rich	-1	3	LDI (I$ per capita)	--
Silicosis	Female	15-19 years	95+ years	Global	1	1	Gold production (kg) per capita	--
Silicosis	Female	15-19 years	95+ years	Global	-1	2	Healthcare access and quality index	546
Silicosis	Female	15-19 years	95+ years	Global	1	2	Cumulative Cigarettes (5 Years)	--
Silicosis	Female	15-19 years	95+ years	Global	1	2	Indoor Air Pollution (All Cooking Fuels)	--
Silicosis	Female	15-19 years	95+ years	Global	1	2	Smoking Prevalence	--
Silicosis	Female	15-19 years	95+ years	Global	-1	3	Socio-demographic Index	546
Silicosis	Female	15-19 years	95+ years	Global	-1	3	Education (years per capita)	--
Silicosis	Female	15-19 years	95+ years	Global	-1	3	LDI (I$ per capita)	--
Silicosis	Male	15-19 years	95+ years	Data Rich	1	1	Gold production (kg) per capita	823
Silicosis	Male	15-19 years	95+ years	Data Rich	-1	2	Healthcare access and quality index	110
Silicosis	Male	15-19 years	95+ years	Data Rich	1	2	Indoor Air Pollution (All Cooking Fuels)	10
Silicosis	Male	15-19 years	95+ years	Data Rich	1	2	Cumulative Cigarettes (5 Years)	70
Silicosis	Male	15-19 years	95+ years	Data Rich	1	2	Smoking Prevalence	504
Silicosis	Male	15-19 years	95+ years	Data Rich	-1	3	Education (years per capita)	277
Silicosis	Male	15-19 years	95+ years	Data Rich	-1	3	Socio-demographic Index	384
Silicosis	Male	15-19 years	95+ years	Data Rich	-1	3	LDI (I$ per capita)	--
Silicosis	Male	15-19	95+ years	Global	1	1	Gold production (kg)	1000

Cause	Sex	Age Start	Age End	Model Type	Direction	Level	Covariate Name	Number of Draws
		years					per capita	
Silicosis	Male	15-19 years	95+ years	Global	-1	2	Healthcare access and quality index	321
Silicosis	Male	15-19 years	95+ years	Global	1	2	Indoor Air Pollution (All Cooking Fuels)	121
Silicosis	Male	15-19 years	95+ years	Global	1	2	Cumulative Cigarettes (5 Years)	215
Silicosis	Male	15-19 years	95+ years	Global	1	2	Smoking Prevalence	--
Silicosis	Male	15-19 years	95+ years	Global	-1	3	Socio-demographic Index	192
Silicosis	Male	15-19 years	95+ years	Global	-1	3	Education (years per capita)	225
Silicosis	Male	15-19 years	95+ years	Global	-1	3	LDI (I$ per capita)	--
Asbestosis	Female	15-19 years	95+ years	Data Rich	1	1	Asbestos consumption (metric tons per year per capita)	--
Asbestosis	Female	15-19 years	95+ years	Data Rich	-1	2	Healthcare access and quality index	--
Asbestosis	Female	15-19 years	95+ years	Data Rich	1	2	Elevation Over 1500m (proportion)	744
Asbestosis	Female	15-19 years	95+ years	Data Rich	1	2	Cumulative Cigarettes (10 Years)	--
Asbestosis	Female	15-19 years	95+ years	Data Rich	1	2	Cumulative Cigarettes (5 Years)	--
Asbestosis	Female	15-19 years	95+ years	Data Rich	1	2	Elevation 500 to 1500m (proportion)	--
Asbestosis	Female	15-19 years	95+ years	Data Rich	1	2	Indoor Air Pollution (All Cooking Fuels)	--
Asbestosis	Female	15-19 years	95+ years	Data Rich	1	2	Smoking Prevalence	--
Asbestosis	Female	15-19 years	95+ years	Data Rich	-1	3	Education (years per capita)	--
Asbestosis	Female	15-19 years	95+ years	Data Rich	-1	3	LDI (I$ per capita)	--
Asbestosis	Female	15-19 years	95+ years	Data Rich	-1	3	Socio-demographic Index	--
Asbestosis	Female	15-19 years	95+ years	Global	1	1	Asbestos consumption (metric tons per year per capita)	--
Asbestosis	Female	15-19 years	95+ years	Global	-1	2	Healthcare access and quality index	546
Asbestosis	Female	15-19 years	95+ years	Global	1	2	Elevation Over 1500m (proportion)	546
Asbestosis	Female	15-19 years	95+ years	Global	1	2	Cumulative Cigarettes (10 Years)	--
Asbestosis	Female	15-19 years	95+ years	Global	1	2	Cumulative Cigarettes (5 Years)	--
Asbestosis	Female	15-19 years	95+ years	Global	1	2	Elevation 500 to 1500m (proportion)	--
Asbestosis	Female	15-19 years	95+ years	Global	1	2	Indoor Air Pollution (All Cooking Fuels)	--
Asbestosis	Female	15-19 years	95+ years	Global	1	2	Smoking Prevalence	--

Cause	Sex	Age Start	Age End	Model Type	Direct ion	Level	Covariate Name	Number of Draws
Asbestosis	Female	15-19 years	95+ years	Global	-1	3	Education (years per capita)	--
Asbestosis	Female	15-19 years	95+ years	Global	-1	3	LDI (I$ per capita)	--
Asbestosis	Female	15-19 years	95+ years	Global	-1	3	Socio-demographic Index	--
Asbestosis	Male	15-19 years	95+ years	Data Rich	1	1	Asbestos consumption (metric tons per year per capita)	--
Asbestosis	Male	15-19 years	95+ years	Data Rich	1	1	Smoking Prevalence	--
Asbestosis	Male	15-19 years	95+ years	Data Rich	-1	2	Healthcare access and quality index	--
Asbestosis	Male	15-19 years	95+ years	Data Rich	1	2	Elevation 500 to 1500m (proportion)	516
Asbestosis	Male	15-19 years	95+ years	Data Rich	1	2	Elevation Over 1500m (proportion)	516
Asbestosis	Male	15-19 years	95+ years	Data Rich	1	2	Cumulative Cigarettes (5 Years)	--
Asbestosis	Male	15-19 years	95+ years	Data Rich	1	2	Indoor Air Pollution (All Cooking Fuels)	--
Asbestosis	Male	15-19 years	95+ years	Data Rich	-1	3	Education (years per capita)	--
Asbestosis	Male	15-19 years	95+ years	Data Rich	-1	3	LDI (I$ per capita)	--
Asbestosis	Male	15-19 years	95+ years	Data Rich	0	3	Socio-demographic Index	742
Asbestosis	Male	15-19 years	95+ years	Global	1	1	Asbestos consumption (metric tons per year per capita)	--
Asbestosis	Male	15-19 years	95+ years	Global	-1	2	Healthcare access and quality index	--
Asbestosis	Male	15-19 years	95+ years	Global	1	2	Elevation 500 to 1500m (proportion)	1000
Asbestosis	Male	15-19 years	95+ years	Global	1	2	Elevation Over 1500m (proportion)	1000
Asbestosis	Male	15-19 years	95+ years	Global	1	2	Cumulative Cigarettes (5 Years)	--
Asbestosis	Male	15-19 years	95+ years	Global	1	2	Indoor Air Pollution (All Cooking Fuels)	--
Asbestosis	Male	15-19 years	95+ years	Global	1	2	Smoking Prevalence	--
Asbestosis	Male	15-19 years	95+ years	Global	-1	3	Education (years per capita)	--
Asbestosis	Male	15-19 years	95+ years	Global	-1	3	LDI (I$ per capita)	--
Asbestosis	Male	15-19 years	95+ years	Global	-1	3	Socio-demographic Index	--
Coal workers pneumoconiosis	Female	15-19 years	95+ years	Data Rich	1	1	Coal Production (per capita)	--
Coal workers pneumoconiosis	Female	15-19 years	95+ years	Data Rich	-1	2	Healthcare access and quality index	325
Coal workers pneumoconiosis	Female	15-19 years	95+ years	Data Rich	1	2	Smoking Prevalence	325
Coal workers	Female	15-19	95+ years	Data Rich	1	2	Cumulative Cigarettes	--

Cause	Sex	Age Start	Age End	Model Type	Direction	Level	Covariate Name	Number of Draws
pneumoconiosis		years					(5 Years)	
Coal workers pneumoconiosis	Female	15-19 years	95+ years	Data Rich	1	2	Indoor Air Pollution (All Cooking Fuels)	--
Coal workers pneumoconiosis	Female	15-19 years	95+ years	Data Rich	-1	3	Education (years per capita)	--
Coal workers pneumoconiosis	Female	15-19 years	95+ years	Data Rich	-1	3	LDI (I$ per capita)	--
Coal workers pneumoconiosis	Female	15-19 years	95+ years	Data Rich	-1	3	Socio-demographic Index	--
Coal workers pneumoconiosis	Female	15-19 years	95+ years	Global	1	1	Coal Production (per capita)	--
Coal workers pneumoconiosis	Female	15-19 years	95+ years	Global	-1	2	Healthcare access and quality index	105
Coal workers pneumoconiosis	Female	15-19 years	95+ years	Global	1	2	Smoking Prevalence	354
Coal workers pneumoconiosis	Female	15-19 years	95+ years	Global	1	2	Cumulative Cigarettes (5 Years)	--
Coal workers pneumoconiosis	Female	15-19 years	95+ years	Global	1	2	Indoor Air Pollution (All Cooking Fuels)	--
Coal workers pneumoconiosis	Female	15-19 years	95+ years	Global	-1	3	Socio-demographic Index	281
Coal workers pneumoconiosis	Female	15-19 years	95+ years	Global	-1	3	Education (years per capita)	--
Coal workers pneumoconiosis	Female	15-19 years	95+ years	Global	-1	3	LDI (I$ per capita)	--
Coal workers pneumoconiosis	Male	15-19 years	95+ years	Data Rich	1	1	Coal Production (per capita)	1000
Coal workers pneumoconiosis	Male	15-19 years	95+ years	Data Rich	-1	2	Healthcare access and quality index	327
Coal workers pneumoconiosis	Male	15-19 years	95+ years	Data Rich	1	2	Indoor Air Pollution (All Cooking Fuels)	68
Coal workers pneumoconiosis	Male	15-19 years	95+ years	Data Rich	1	2	Cumulative Cigarettes (5 Years)	250
Coal workers pneumoconiosis	Male	15-19 years	95+ years	Data Rich	1	2	Smoking Prevalence	274
Coal workers pneumoconiosis	Male	15-19 years	95+ years	Data Rich	-1	3	Socio-demographic Index	195
Coal workers pneumoconiosis	Male	15-19 years	95+ years	Data Rich	-1	3	Education (years per capita)	464
Coal workers pneumoconiosis	Male	15-19 years	95+ years	Data Rich	-1	3	LDI (I$ per capita)	--
Coal workers pneumoconiosis	Male	15-19 years	95+ years	Global	1	1	Coal Production (per capita)	1000
Coal workers pneumoconiosis	Male	15-19 years	95+ years	Global	-1	2	Healthcare access and quality index	461
Coal workers pneumoconiosis	Male	15-19 years	95+ years	Global	1	2	Indoor Air Pollution (All Cooking Fuels)	64
Coal workers pneumoconiosis	Male	15-19 years	95+ years	Global	1	2	Cumulative Cigarettes (5 Years)	334
Coal workers pneumoconiosis	Male	15-19 years	95+ years	Global	1	2	Smoking Prevalence	339
Coal workers pneumoconiosis	Male	15-19 years	95+ years	Global	-1	3	Socio-demographic Index	191
Coal workers pneumoconiosis	Male	15-19 years	95+ years	Global	-1	3	Education (years per capita)	232

Cause	Sex	Age Start	Age End	Model Type	Direction	Level	Covariate Name	Number of Draws
Coal workers pneumoconiosis	Male	15-19 years	95+ years	Global	-1	3	LDI (I$ per capita)	--
Other pneumoconiosis	Female	15-19 years	95+ years	Data Rich	-1	2	Healthcare access and quality index	89
Other pneumoconiosis	Female	15-19 years	95+ years	Data Rich	1	2	Indoor Air Pollution (All Cooking Fuels)	38
Other pneumoconiosis	Female	15-19 years	95+ years	Data Rich	1	2	Smoking Prevalence	408
Other pneumoconiosis	Female	15-19 years	95+ years	Data Rich	1	2	Cumulative Cigarettes (5 Years)	575
Other pneumoconiosis	Female	15-19 years	95+ years	Data Rich	-1	3	Socio-demographic Index	137
Other pneumoconiosis	Female	15-19 years	95+ years	Data Rich	-1	3	Education (years per capita)	--
Other pneumoconiosis	Female	15-19 years	95+ years	Data Rich	-1	3	LDI (I$ per capita)	--
Other pneumoconiosis	Female	15-19 years	95+ years	Global	-1	2	Healthcare access and quality index	38
Other pneumoconiosis	Female	15-19 years	95+ years	Global	1	2	Indoor Air Pollution (All Cooking Fuels)	63
Other pneumoconiosis	Female	15-19 years	95+ years	Global	1	2	Cumulative Cigarettes (5 Years)	431
Other pneumoconiosis	Female	15-19 years	95+ years	Global	1	2	Smoking Prevalence	625
Other pneumoconiosis	Female	15-19 years	95+ years	Global	-1	3	Education (years per capita)	22
Other pneumoconiosis	Female	15-19 years	95+ years	Global	-1	3	Socio-demographic Index	236
Other pneumoconiosis	Female	15-19 years	95+ years	Global	-1	3	LDI (I$ per capita)	--
Other pneumoconiosis	Male	15-19 years	95+ years	Data Rich	-1	2	Healthcare access and quality index	508
Other pneumoconiosis	Male	15-19 years	95+ years	Data Rich	1	2	Indoor Air Pollution (All Cooking Fuels)	169
Other pneumoconiosis	Male	15-19 years	95+ years	Data Rich	1	2	Cumulative Cigarettes (5 Years)	323
Other pneumoconiosis	Male	15-19 years	95+ years	Data Rich	1	2	Smoking Prevalence	323
Other pneumoconiosis	Male	15-19 years	95+ years	Data Rich	-1	3	Education (years per capita)	141
Other pneumoconiosis	Male	15-19 years	95+ years	Data Rich	-1	3	LDI (I$ per capita)	--
Other pneumoconiosis	Male	15-19 years	95+ years	Data Rich	-1	3	Socio-demographic Index	--
Other pneumoconiosis	Male	15-19 years	95+ years	Global	-1	2	Healthcare access and quality index	265
Other pneumoconiosis	Male	15-19 years	95+ years	Global	1	2	Indoor Air Pollution (All Cooking Fuels)	216
Other pneumoconiosis	Male	15-19 years	95+ years	Global	1	2	Cumulative Cigarettes (5 Years)	435
Other pneumoconiosis	Male	15-19 years	95+ years	Global	1	2	Smoking Prevalence	719
Other pneumoconiosis	Male	15-19 years	95+ years	Global	-1	3	Socio-demographic Index	52
Other pneumoconiosis	Male	15-19	95+ years	Global	-1	3	Education (years per	201

Cause	Sex	Age Start	Age End	Model Type	Direct ion	Level	Covariate Name	Number of Draws
		years					capita)	
Other pneumoconiosis	Male	15-19 years	95+ years	Global	-1	3	LDI (I$ per capita)	--
Asthma	Female	1-4 years	95+ years	Data Rich	-1	1	Healthcare access and quality index	566
Asthma	Female	1-4 years	95+ years	Data Rich	1	1	Cumulative Cigarettes (10 Years)	108
Asthma	Female	1-4 years	95+ years	Data Rich	1	1	Cumulative Cigarettes (5 Years)	199
Asthma	Female	1-4 years	95+ years	Data Rich	1	1	Log-transformed SEV scalar: Asthma	--
Asthma	Female	1-4 years	95+ years	Data Rich	1	2	Smoking Prevalence	5
Asthma	Female	1-4 years	95+ years	Data Rich	1	2	Indoor Air Pollution (All Cooking Fuels)	29
Asthma	Female	1-4 years	95+ years	Data Rich	1	2	Outdoor Air Pollution (PM2.5)	537
Asthma	Female	1-4 years	95+ years	Data Rich	-1	3	Socio-demographic Index	2
Asthma	Female	1-4 years	95+ years	Data Rich	-1	3	Education (years per capita)	180
Asthma	Female	1-4 years	95+ years	Data Rich	-1	3	LDI (I$ per capita)	--
Asthma	Female	1-4 years	95+ years	Global	-1	1	Healthcare access and quality index	593
Asthma	Female	1-4 years	95+ years	Global	1	1	Cumulative Cigarettes (5 Years)	145
Asthma	Female	1-4 years	95+ years	Global	1	1	Cumulative Cigarettes (10 Years)	160
Asthma	Female	1-4 years	95+ years	Global	1	1	Log-transformed SEV scalar: Asthma	--
Asthma	Female	1-4 years	95+ years	Global	1	2	Smoking Prevalence	2
Asthma	Female	1-4 years	95+ years	Global	1	2	Indoor Air Pollution (All Cooking Fuels)	91
Asthma	Female	1-4 years	95+ years	Global	1	2	Outdoor Air Pollution (PM2.5)	300
Asthma	Female	1-4 years	95+ years	Global	-1	3	Socio-demographic Index	12
Asthma	Female	1-4 years	95+ years	Global	-1	3	Education (years per capita)	273
Asthma	Female	1-4 years	95+ years	Global	-1	3	LDI (I$ per capita)	--
Asthma	Male	1-4 years	95+ years	Data Rich	-1	1	Healthcare access and quality index	159
Asthma	Male	1-4 years	95+ years	Data Rich	1	1	Cumulative Cigarettes (5 Years)	143
Asthma	Male	1-4 years	95+ years	Data Rich	1	1	Cumulative Cigarettes (10 Years)	283
Asthma	Male	1-4 years	95+ years	Data Rich	1	1	Log-transformed SEV scalar: Asthma	--
Asthma	Male	1-4 years	95+ years	Data Rich	1	2	Outdoor Air Pollution (PM2.5)	9
Asthma	Male	1-4 years	95+ years	Data Rich	1	2	Smoking Prevalence	27
Asthma	Male	1-4 years	95+ years	Data Rich	1	2	Indoor Air Pollution (All Cooking Fuels)	110
Asthma	Male	1-4 years	95+ years	Data Rich	-1	3	Socio-demographic Index	432
Asthma	Male	1-4 years	95+ years	Data Rich	-1	3	Education (years per	571

Cause	Sex	Age Start	Age End	Model Type	Direction	Level	Covariate Name	Number of Draws
							capita)	
Asthma	Male	1-4 years	95+ years	Data Rich	-1	3	LDI (I$ per capita)	--
Asthma	Male	1-4 years	95+ years	Global	-1	1	Healthcare access and quality index	788
Asthma	Male	1-4 years	95+ years	Global	1	1	Cumulative Cigarettes (5 Years)	268
Asthma	Male	1-4 years	95+ years	Global	1	1	Cumulative Cigarettes (10 Years)	427
Asthma	Male	1-4 years	95+ years	Global	1	1	Log-transformed SEV scalar: Asthma	--
Asthma	Male	1-4 years	95+ years	Global	1	2	Smoking Prevalence	63
Asthma	Male	1-4 years	95+ years	Global	1	2	Indoor Air Pollution (All Cooking Fuels)	70
Asthma	Male	1-4 years	95+ years	Global	1	2	Outdoor Air Pollution (PM2.5)	198
Asthma	Male	1-4 years	95+ years	Global	-1	3	Socio-demographic Index	76
Asthma	Male	1-4 years	95+ years	Global	-1	3	Education (years per capita)	549
Asthma	Male	1-4 years	95+ years	Global	-1	3	LDI (I$ per capita)	--
Interstitial lung disease and pulmonary sarcoidosis	Female	1-4 years	95+ years	Data Rich	1	1	Log-transformed SEV scalar: ILD	414
Interstitial lung disease and pulmonary sarcoidosis	Female	1-4 years	95+ years	Data Rich	1	1	Cumulative Cigarettes (10 Years)	--
Interstitial lung disease and pulmonary sarcoidosis	Female	1-4 years	95+ years	Data Rich	1	1	Cumulative Cigarettes (5 Years)	--
Interstitial lung disease and pulmonary sarcoidosis	Female	1-4 years	95+ years	Data Rich	1	1	Smoking Prevalence	--
Interstitial lung disease and pulmonary sarcoidosis	Female	1-4 years	95+ years	Data Rich	-1	2	Healthcare access and quality index	--
Interstitial lung disease and pulmonary sarcoidosis	Female	1-4 years	95+ years	Data Rich	1	2	Outdoor Air Pollution (PM2.5)	586
Interstitial lung disease and pulmonary sarcoidosis	Female	1-4 years	95+ years	Data Rich	1	2	Indoor Air Pollution (All Cooking Fuels)	--
Interstitial lung disease and pulmonary sarcoidosis	Female	1-4 years	95+ years	Data Rich	-1	3	Education (years per capita)	--
Interstitial lung disease and pulmonary sarcoidosis	Female	1-4 years	95+ years	Data Rich	-1	3	LDI (I$ per capita)	--
Interstitial lung disease and pulmonary sarcoidosis	Female	1-4 years	95+ years	Data Rich	0	3	Socio-demographic Index	618
Interstitial lung disease and pulmonary sarcoidosis	Female	1-4 years	95+ years	Global	1	1	Outdoor Air Pollution (PM2.5)	681
Interstitial lung disease and pulmonary sarcoidosis	Female	1-4 years	95+ years	Global	1	1	Log-transformed SEV scalar: ILD	739
Interstitial lung disease and pulmonary sarcoidosis	Female	1-4 years	95+ years	Global	1	1	Cumulative Cigarettes (10 Years)	--
Interstitial lung disease and pulmonary sarcoidosis	Female	1-4 years	95+ years	Global	1	1	Cumulative Cigarettes (5 Years)	--
Interstitial lung disease and pulmonary sarcoidosis	Female	1-4 years	95+ years	Global	1	1	Indoor Air Pollution (All Cooking Fuels)	--
Interstitial lung disease and pulmonary sarcoidosis	Female	1-4 years	95+ years	Global	1	1	Smoking Prevalence	--
Interstitial lung disease and pulmonary sarcoidosis	Female	1-4 years	95+ years	Global	-1	2	Healthcare access and quality index	--
Interstitial lung disease and	Female	1-4 years	95+ years	Global	-1	3	Education (years per	--

Cause	Sex	Age Start	Age End	Model Type	Direction	Level	Covariate Name	Number of Draws
pulmonary sarcoidosis							capita)	
Interstitial lung disease and pulmonary sarcoidosis	Female	1-4 years	95+ years	Global	-1	3	LDI (I$ per capita)	--
Interstitial lung disease and pulmonary sarcoidosis	Female	1-4 years	95+ years	Global	0	3	Socio-demographic Index	693
Interstitial lung disease and pulmonary sarcoidosis	Male	1-4 years	95+ years	Data Rich	1	1	Cumulative Cigarettes (10 Years)	--
Interstitial lung disease and pulmonary sarcoidosis	Male	1-4 years	95+ years	Data Rich	1	1	Cumulative Cigarettes (5 Years)	--
Interstitial lung disease and pulmonary sarcoidosis	Male	1-4 years	95+ years	Data Rich	1	1	Log-transformed SEV scalar: ILD	--
Interstitial lung disease and pulmonary sarcoidosis	Male	1-4 years	95+ years	Data Rich	1	1	Smoking Prevalence	--
Interstitial lung disease and pulmonary sarcoidosis	Male	1-4 years	95+ years	Data Rich	-1	2	Healthcare access and quality index	--
Interstitial lung disease and pulmonary sarcoidosis	Male	1-4 years	95+ years	Data Rich	1	2	Outdoor Air Pollution (PM2.5)	1000
Interstitial lung disease and pulmonary sarcoidosis	Male	1-4 years	95+ years	Data Rich	1	2	Indoor Air Pollution (All Cooking Fuels)	--
Interstitial lung disease and pulmonary sarcoidosis	Male	1-4 years	95+ years	Data Rich	-1	3	Education (years per capita)	--
Interstitial lung disease and pulmonary sarcoidosis	Male	1-4 years	95+ years	Data Rich	-1	3	LDI (I$ per capita)	--
Interstitial lung disease and pulmonary sarcoidosis	Male	1-4 years	95+ years	Data Rich	0	3	Socio-demographic Index	1000
Interstitial lung disease and pulmonary sarcoidosis	Male	1-4 years	95+ years	Global	1	1	Cumulative Cigarettes (10 Years)	--
Interstitial lung disease and pulmonary sarcoidosis	Male	1-4 years	95+ years	Global	1	1	Cumulative Cigarettes (5 Years)	--
Interstitial lung disease and pulmonary sarcoidosis	Male	1-4 years	95+ years	Global	1	1	Log-transformed SEV scalar: ILD	--
Interstitial lung disease and pulmonary sarcoidosis	Male	1-4 years	95+ years	Global	1	1	Smoking Prevalence	--
Interstitial lung disease and pulmonary sarcoidosis	Male	1-4 years	95+ years	Global	-1	2	Healthcare access and quality index	--
Interstitial lung disease and pulmonary sarcoidosis	Male	1-4 years	95+ years	Global	1	2	Outdoor Air Pollution (PM2.5)	422
Interstitial lung disease and pulmonary sarcoidosis	Male	1-4 years	95+ years	Global	1	2	Indoor Air Pollution (All Cooking Fuels)	--
Interstitial lung disease and pulmonary sarcoidosis	Male	1-4 years	95+ years	Global	-1	3	Education (years per capita)	--
Interstitial lung disease and pulmonary sarcoidosis	Male	1-4 years	95+ years	Global	-1	3	LDI (I$ per capita)	--
Interstitial lung disease and pulmonary sarcoidosis	Male	1-4 years	95+ years	Global	0	3	Socio-demographic Index	815
Other chronic respiratory diseases	Female	1-4 years	95+ years	Data Rich	1	1	Indoor Air Pollution (All Cooking Fuels)	242
Other chronic respiratory diseases	Female	1-4 years	95+ years	Data Rich	1	1	Outdoor Air Pollution (PM2.5)	536
Other chronic respiratory diseases	Female	1-4 years	95+ years	Data Rich	1	1	Cumulative Cigarettes (5 Years)	--
Other chronic respiratory diseases	Female	1-4 years	95+ years	Data Rich	1	1	Log-transformed SEV scalar: Oth Resp	--
Other chronic respiratory diseases	Female	1-4 years	95+ years	Data Rich	1	1	Smoking Prevalence	--

Cause	Sex	Age Start	Age End	Model Type	Direct ion	Level	Covariate Name	Number of Draws
Other chronic respiratory diseases	Female	1-4 years	95+ years	Data Rich	-1	2	Healthcare access and quality index	58
Other chronic respiratory diseases	Female	1-4 years	95+ years	Data Rich	1	2	Elevation Over 1500m (proportion)	12
Other chronic respiratory diseases	Female	1-4 years	95+ years	Data Rich	1	2	Elevation 500 to 1500m (proportion)	306
Other chronic respiratory diseases	Female	1-4 years	95+ years	Data Rich	1	2	Population Density (over 1000 ppl/sqkm, proportion)	--
Other chronic respiratory diseases	Female	1-4 years	95+ years	Data Rich	-1	3	Education (years per capita)	17
Other chronic respiratory diseases	Female	1-4 years	95+ years	Data Rich	-1	3	Socio-demographic Index	44
Other chronic respiratory diseases	Female	1-4 years	95+ years	Data Rich	-1	3	LDI (I$ per capita)	--
Other chronic respiratory diseases	Female	1-4 years	95+ years	Global	1	1	Indoor Air Pollution (All Cooking Fuels)	134
Other chronic respiratory diseases	Female	1-4 years	95+ years	Global	1	1	Cumulative Cigarettes (5 Years)	--
Other chronic respiratory diseases	Female	1-4 years	95+ years	Global	1	1	Log-transformed SEV scalar: Oth Resp	--
Other chronic respiratory diseases	Female	1-4 years	95+ years	Global	1	1	Outdoor Air Pollution (PM2.5)	--
Other chronic respiratory diseases	Female	1-4 years	95+ years	Global	1	1	Smoking Prevalence	--
Other chronic respiratory diseases	Female	1-4 years	95+ years	Global	-1	2	Healthcare access and quality index	215
Other chronic respiratory diseases	Female	1-4 years	95+ years	Global	1	2	Elevation 500 to 1500m (proportion)	337
Other chronic respiratory diseases	Female	1-4 years	95+ years	Global	1	2	Elevation Over 1500m (proportion)	405
Other chronic respiratory diseases	Female	1-4 years	95+ years	Global	1	2	Population Density (over 1000 ppl/sqkm, proportion)	--
Other chronic respiratory diseases	Female	1-4 years	95+ years	Global	-1	3	Socio-demographic Index	70
Other chronic respiratory diseases	Female	1-4 years	95+ years	Global	-1	3	Education (years per capita)	300
Other chronic respiratory diseases	Female	1-4 years	95+ years	Global	-1	3	LDI (I$ per capita)	--
Other chronic respiratory diseases	Male	1-4 years	95+ years	Data Rich	1	1	Smoking Prevalence	19
Other chronic respiratory diseases	Male	1-4 years	95+ years	Data Rich	1	1	Indoor Air Pollution (All Cooking Fuels)	230
Other chronic respiratory diseases	Male	1-4 years	95+ years	Data Rich	1	1	Outdoor Air Pollution (PM2.5)	253
Other chronic respiratory diseases	Male	1-4 years	95+ years	Data Rich	1	1	Cumulative Cigarettes (5 Years)	401
Other chronic respiratory diseases	Male	1-4 years	95+ years	Data Rich	1	1	Log-transformed SEV scalar: Oth Resp	--
Other chronic respiratory diseases	Male	1-4 years	95+ years	Data Rich	-1	2	Healthcare access and quality index	0
Other chronic respiratory diseases	Male	1-4 years	95+ years	Data Rich	1	2	Elevation Over 1500m (proportion)	60
Other chronic respiratory	Male	1-4 years	95+ years	Data Rich	1	2	Elevation 500 to	--

Cause	Sex	Age Start	Age End	Model Type	Direct ion	Level	Covariate Name	Number of Draws
diseases							1500m (proportion)	
Other chronic respiratory diseases	Male	1-4 years	95+ years	Data Rich	1	2	Population Density (over 1000 ppl/sqkm, proportion)	--
Other chronic respiratory diseases	Male	1-4 years	95+ years	Data Rich	-1	3	Education (years per capita)	0
Other chronic respiratory diseases	Male	1-4 years	95+ years	Data Rich	-1	3	Socio-demographic Index	0
Other chronic respiratory diseases	Male	1-4 years	95+ years	Data Rich	-1	3	LDI (I$ per capita)	--
Other chronic respiratory diseases	Male	1-4 years	95+ years	Global	1	1	Cumulative Cigarettes (5 Years)	132
Other chronic respiratory diseases	Male	1-4 years	95+ years	Global	1	1	Smoking Prevalence	139
Other chronic respiratory diseases	Male	1-4 years	95+ years	Global	1	1	Indoor Air Pollution (All Cooking Fuels)	256
Other chronic respiratory diseases	Male	1-4 years	95+ years	Global	1	1	Log-transformed SEV scalar: Oth Resp	--
Other chronic respiratory diseases	Male	1-4 years	95+ years	Global	1	1	Outdoor Air Pollution (PM2.5)	--
Other chronic respiratory diseases	Male	1-4 years	95+ years	Global	-1	2	Healthcare access and quality index	182
Other chronic respiratory diseases	Male	1-4 years	95+ years	Global	1	2	Elevation Over 1500m (proportion)	390
Other chronic respiratory diseases	Male	1-4 years	95+ years	Global	1	2	Elevation 500 to 1500m (proportion)	410
Other chronic respiratory diseases	Male	1-4 years	95+ years	Global	1	2	Population Density (over 1000 ppl/sqkm, proportion)	--
Other chronic respiratory diseases	Male	1-4 years	95+ years	Global	-1	3	Socio-demographic Index	69
Other chronic respiratory diseases	Male	1-4 years	95+ years	Global	-1	3	Education (years per capita)	168
Other chronic respiratory diseases	Male	1-4 years	95+ years	Global	-1	3	LDI (I$ per capita)	--

Table S6. Comparison of GBD 2017 and GBD 2019 covariates and level of covariates used in cause of death modeling

Cause	Sex	Covariate	Level 1: GBD 2019	Level 2: GBD 2019	Level 3: GBD 2019	Level 1: GBD 2017	Level 2: GBD 2017	Level 3: GBD 2017
Chronic respiratory diseases	Male	Outdoor Air Pollution (PM2.5)		X			X	
Chronic respiratory diseases	Male	Smoking Prevalence		X			X	
Chronic respiratory diseases	Female	Population Density (over 1000 ppl/sqkm, proportion)			X			X
Chronic respiratory diseases	Male	Population Density (over 1000 ppl/sqkm, proportion)			X			X
Chronic respiratory diseases	Female	Cumulative Cigarettes (5 Years)	X			X		
Chronic respiratory diseases	Male	Cumulative Cigarettes (5 Years)	X			X		
Chronic respiratory diseases	Female	Elevation 500 to 1500m (proportion)			X			X
Chronic respiratory diseases	Male	Elevation 500 to 1500m (proportion)			X			X
Chronic respiratory diseases	Male	Smoking Prevalence	X			X		
Chronic respiratory diseases	Male	Smoking Prevalence	X				X	
Chronic respiratory diseases	Male	Smoking Prevalence		X		X		
Chronic respiratory diseases	Female	Smoking Prevalence		X			X	
Chronic respiratory diseases	Male	Elevation Over 1500m (proportion)		X			X	
Chronic respiratory diseases	Female	Elevation Over 1500m (proportion)		X			X	
Chronic respiratory diseases	Female	Outdoor Air Pollution (PM2.5)		X			X	
Chronic respiratory diseases	Male	Indoor Air Pollution (All Cooking Fuels)	X			X		
Chronic respiratory diseases	Male	Indoor Air Pollution (All Cooking Fuels)	X				X	
Chronic respiratory diseases	Male	Indoor Air Pollution (All Cooking Fuels)		X		X		
Chronic respiratory diseases	Male	Indoor Air Pollution (All Cooking Fuels)		X			X	
Chronic respiratory diseases	Female	Indoor Air Pollution (All Cooking Fuels)		X			X	
Chronic respiratory diseases	Male	Healthcare access and quality index		X			X	
Chronic respiratory	Female	Healthcare access and	X			X		

Cause	Sex	Covariate	Level 1: GBD 2019	Level 2: GBD 2019	Level 3: GBD 2019	Level 1: GBD 2017	Level 2: GBD 2017	Level 3: GBD 2017
diseases		quality index						
Chronic respiratory diseases	Male	Socio-demographic Index			X			X
Chronic respiratory diseases	Female	Socio-demographic Index			X			X
Chronic respiratory diseases	Male	LDI (I$ per capita)			X			X
Chronic respiratory diseases	Female	LDI (I$ per capita)			X			X
Chronic respiratory diseases	Male	Education (years per capita)			X			X
Chronic respiratory diseases	Female	Education (years per capita)			X			X
Chronic respiratory diseases	Male	Cumulative Cigarettes (10 Years)	X			X		
Chronic respiratory diseases	Female	Cumulative Cigarettes (10 Years)	X			X		
Chronic obstructive pulmonary disease	Female	Cumulative Cigarettes (5 Years)	X			X		
Chronic obstructive pulmonary disease	Male	Cumulative Cigarettes (20 Years)	X			X		
Chronic obstructive pulmonary disease	Female	Education (years per capita)			X			X
Chronic obstructive pulmonary disease	Male	Cumulative Cigarettes (5 Years)	X			X		
Chronic obstructive pulmonary disease	Female	Cumulative Cigarettes (10 Years)	X			X		
Chronic obstructive pulmonary disease	Male	Education (years per capita)			X			X
Chronic obstructive pulmonary disease	Female	Smoking Prevalence		X			X	
Chronic obstructive pulmonary disease	Male	Smoking Prevalence		X			X	
Chronic obstructive pulmonary disease	Female	Elevation Over 1500m (proportion)	X			X		
Chronic obstructive pulmonary disease	Male	Elevation Over 1500m (proportion)	X			X		
Chronic obstructive pulmonary disease	Female	Outdoor Air Pollution (PM2.5)	X			X		
Chronic obstructive pulmonary disease	Male	Outdoor Air Pollution (PM2.5)	X			X		
Chronic obstructive pulmonary disease	Female	Indoor Air Pollution (All Cooking Fuels)		X			X	
Chronic obstructive pulmonary disease	Male	Cumulative Cigarettes (10 Years)	X			X		
Chronic obstructive pulmonary disease	Male	Log-transformed SEV scalar: COPD	X			X		
Chronic obstructive	Male	Indoor Air Pollution		X			X	

Cause	Sex	Covariate	Level 1: GBD 2019	Level 2: GBD 2019	Level 3: GBD 2019	Level 1: GBD 2017	Level 2: GBD 2017	Level 3: GBD 2017
pulmonary disease		(All Cooking Fuels)						
Chronic obstructive pulmonary disease	Female	Healthcare access and quality index	X			X		
Chronic obstructive pulmonary disease	Male	Healthcare access and quality index		X			X	
Chronic obstructive pulmonary disease	Female	Socio-demographic Index			X			X
Chronic obstructive pulmonary disease	Male	Socio-demographic Index			X			X
Chronic obstructive pulmonary disease	Female	LDI (I$ per capita)			X			X
Chronic obstructive pulmonary disease	Male	LDI (I$ per capita)			X			X
Chronic obstructive pulmonary disease	Female	Log-transformed SEV scalar: COPD	X			X		
Pneumoconiosis	Female	Cumulative Cigarettes (5 Years)		X			X	
Pneumoconiosis	Male	Cumulative Cigarettes (5 Years)		X			X	
Pneumoconiosis	Female	Smoking Prevalence		X			X	
Pneumoconiosis	Female	Indoor Air Pollution (All Cooking Fuels)		X			X	
Pneumoconiosis	Male	Indoor Air Pollution (All Cooking Fuels)		X			X	
Pneumoconiosis	Female	Healthcare access and quality index		X			X	
Pneumoconiosis	Female	LDI (I$ per capita)			X			X
Pneumoconiosis	Female	Socio-demographic Index			X			X
Pneumoconiosis	Male	Socio-demographic Index			X			X
Pneumoconiosis	Male	LDI (I$ per capita)			X			X
Pneumoconiosis	Female	Education (years per capita)			X			X
Pneumoconiosis	Male	Education (years per capita)			X			X
Pneumoconiosis	Male	Education (years per capita)			X			X
Pneumoconiosis	Male	Asbestos consumption (metric tons per year per capita)	X			X		
Pneumoconiosis	Male	Healthcare access and quality index		X			X	
Pneumoconiosis	Female	Asbestos consumption (metric tons per year per capita)	X			X		

Cause	Sex	Covariate	Level 1: GBD 2019	Level 2: GBD 2019	Level 3: GBD 2019	Level 1: GBD 2017	Level 2: GBD 2017	Level 3: GBD 2017
Pneumoconiosis	Male	Smoking Prevalence		X			X	
Silicosis	Female	Smoking Prevalence		X			X	
Silicosis	Female	Education (years per capita)			X			X
Silicosis	Male	LDI (I$ per capita)			X			X
Silicosis	Female	LDI (I$ per capita)			X			X
Silicosis	Female	Socio-demographic Index			X			X
Silicosis	Male	Healthcare access and quality index		X			X	
Silicosis	Male	Socio-demographic Index			X			X
Silicosis	Male	Indoor Air Pollution (All Cooking Fuels)		X			X	
Silicosis	Female	Indoor Air Pollution (All Cooking Fuels)		X			X	
Silicosis	Male	Smoking Prevalence		X			X	
Silicosis	Male	Education (years per capita)			X			X
Silicosis	Female	Cumulative Cigarettes (5 Years)		X			X	
Silicosis	Female	Healthcare access and quality index		X			X	
Silicosis	Male	Cumulative Cigarettes (5 Years)		X			X	
Asbestosis	Female	Socio-demographic Index			X			X
Asbestosis	Male	Education (years per capita)			X			X
Asbestosis	Female	Education (years per capita)			X			X
Asbestosis	Male	LDI (I$ per capita)			X			X
Asbestosis	Female	LDI (I$ per capita)			X			X
Asbestosis	Male	Socio-demographic Index			X			X
Asbestosis	Female	Healthcare access and quality index		X			X	
Asbestosis	Male	Indoor Air Pollution (All Cooking Fuels)		X			X	
Asbestosis	Female	Asbestos consumption (metric tons per year per capita)	X			X		
Asbestosis	Male	Asbestos consumption (metric tons per year per capita)	X			X		
Asbestosis	Female	Elevation 500 to		X			X	

Cause	Sex	Covariate	Level 1: GBD 2019	Level 2: GBD 2019	Level 3: GBD 2019	Level 1: GBD 2017	Level 2: GBD 2017	Level 3: GBD 2017
		1500m (proportion)						
Asbestosis	Male	Elevation 500 to 1500m (proportion)		X			X	
Asbestosis	Female	Cumulative Cigarettes (5 Years)		X			X	
Asbestosis	Male	Cumulative Cigarettes (5 Years)		X			X	
Asbestosis	Male	Healthcare access and quality index		X			X	
Asbestosis	Female	Cumulative Cigarettes (10 Years)		X			X	
Asbestosis	Male	Smoking Prevalence	X				X	
Asbestosis	Male	Smoking Prevalence	X			X		
Asbestosis	Male	Smoking Prevalence		X			X	
Asbestosis	Male	Smoking Prevalence		X		X		
Asbestosis	Female	Elevation Over 1500m (proportion)		X			X	
Asbestosis	Male	Elevation Over 1500m (proportion)		X			X	
Asbestosis	Female	Indoor Air Pollution (All Cooking Fuels)		X			X	
Asbestosis	Female	Smoking Prevalence		X			X	
Coal workers pneumoconiosis	Female	LDI (I$ per capita)			X			X
Coal workers pneumoconiosis	Male	LDI (I$ per capita)			X			X
Coal workers pneumoconiosis	Female	Socio-demographic Index			X			X
Coal workers pneumoconiosis	Male	Cumulative Cigarettes (5 Years)		X			X	
Coal workers pneumoconiosis	Female	Education (years per capita)			X			X
Coal workers pneumoconiosis	Male	Education (years per capita)			X			X
Coal workers pneumoconiosis	Female	Cumulative Cigarettes (5 Years)		X			X	
Coal workers pneumoconiosis	Male	Healthcare access and quality index		X			X	
Coal workers pneumoconiosis	Male	Socio-demographic Index			X			X
Coal workers pneumoconiosis	Male	Indoor Air Pollution (All Cooking Fuels)		X			X	
Coal workers pneumoconiosis	Female	Indoor Air Pollution (All Cooking Fuels)		X			X	
Coal workers pneumoconiosis	Male	Smoking Prevalence		X			X	
Coal workers pneumoconiosis	Female	Smoking Prevalence		X			X	

Cause	Sex	Covariate	Level 1: GBD 2019	Level 2: GBD 2019	Level 3: GBD 2019	Level 1: GBD 2017	Level 2: GBD 2017	Level 3: GBD 2017
Coal workers pneumoconiosis	Female	Healthcare access and quality index		X			X	
Other pneumoconiosis	Male	Indoor Air Pollution (All Cooking Fuels)		X			X	
Other pneumoconiosis	Female	Cumulative Cigarettes (5 Years)		X			X	
Other pneumoconiosis	Male	Cumulative Cigarettes (5 Years)		X			X	
Other pneumoconiosis	Male	Smoking Prevalence		X			X	
Other pneumoconiosis	Female	Indoor Air Pollution (All Cooking Fuels)		X			X	
Other pneumoconiosis	Female	Healthcare access and quality index		X			X	
Other pneumoconiosis	Female	Smoking Prevalence		X			X	
Other pneumoconiosis	Male	Education (years per capita)			X			X
Other pneumoconiosis	Female	Education (years per capita)			X			X
Other pneumoconiosis	Male	LDI (I$ per capita)			X			X
Other pneumoconiosis	Female	LDI (I$ per capita)			X			X
Other pneumoconiosis	Male	Socio-demographic Index			X			X
Other pneumoconiosis	Female	Socio-demographic Index			X			X
Other pneumoconiosis	Male	Healthcare access and quality index		X			X	
Asthma	Female	Cumulative Cigarettes (5 Years)	X			X		
Asthma	Male	Education (years per capita)			X			X
Asthma	Male	Cumulative Cigarettes (5 Years)	X			X		
Asthma	Female	Education (years per capita)			X			X
Asthma	Male	LDI (I$ per capita)			X			X
Asthma	Female	LDI (I$ per capita)			X			X
Asthma	Male	Socio-demographic Index			X			X
Asthma	Male	Log-transformed SEV scalar: Asthma	X			X		
Asthma	Female	Socio-demographic Index			X			X
Asthma	Male	Healthcare access and quality index	X			X		
Asthma	Female	Healthcare access and quality index	X			X		
Asthma	Male	Indoor Air Pollution (All Cooking Fuels)		X			X	

Cause	Sex	Covariate	Level 1: GBD 2019	Level 2: GBD 2019	Level 3: GBD 2019	Level 1: GBD 2017	Level 2: GBD 2017	Level 3: GBD 2017
Asthma	Female	Indoor Air Pollution (All Cooking Fuels)		X			X	
Asthma	Male	Outdoor Air Pollution (PM2.5)		X			X	
Asthma	Female	Outdoor Air Pollution (PM2.5)		X			X	
Asthma	Male	Smoking Prevalence		X			X	
Asthma	Female	Smoking Prevalence		X			X	
Asthma	Male	Cumulative Cigarettes (10 Years)	X			X		
Asthma	Female	Cumulative Cigarettes (10 Years)	X			X		
Asthma	Female	Log-transformed SEV scalar: Asthma	X			X		
Interstitial lung disease and pulmonary sarcoidosis	Male	Cumulative Cigarettes (10 Years)	X			X		
Interstitial lung disease and pulmonary sarcoidosis	Female	Cumulative Cigarettes (10 Years)	X			X		
Interstitial lung disease and pulmonary sarcoidosis	Female	Indoor Air Pollution (All Cooking Fuels)		X			X	
Interstitial lung disease and pulmonary sarcoidosis	Female	Cumulative Cigarettes (5 Years)	X			X		
Interstitial lung disease and pulmonary sarcoidosis	Male	Indoor Air Pollution (All Cooking Fuels)		X			X	
Interstitial lung disease and pulmonary sarcoidosis	Female	Healthcare access and quality index		X			X	
Interstitial lung disease and pulmonary sarcoidosis	Male	Healthcare access and quality index		X			X	
Interstitial lung disease and pulmonary sarcoidosis	Female	Socio-demographic Index			X			X
Interstitial lung disease and pulmonary sarcoidosis	Male	Socio-demographic Index			X			X
Interstitial lung disease and pulmonary sarcoidosis	Female	LDI (I$ per capita)			X			X
Interstitial lung disease and pulmonary sarcoidosis	Female	Indoor Air Pollution (All Cooking Fuels)		X		X		
Interstitial lung disease and pulmonary	Male	LDI (I$ per capita)			X			X

Cause	Sex	Covariate	Level 1: GBD 2019	Level 2: GBD 2019	Level 3: GBD 2019	Level 1: GBD 2017	Level 2: GBD 2017	Level 3: GBD 2017
sarcoidosis								
Interstitial lung disease and pulmonary sarcoidosis	Female	Education (years per capita)			X			X
Interstitial lung disease and pulmonary sarcoidosis	Female	Education (years per capita)			X			X
Interstitial lung disease and pulmonary sarcoidosis	Male	Education (years per capita)			X			X
Interstitial lung disease and pulmonary sarcoidosis	Female	Indoor Air Pollution (All Cooking Fuels)	X			X		
Interstitial lung disease and pulmonary sarcoidosis	Female	Indoor Air Pollution (All Cooking Fuels)	X				X	
Interstitial lung disease and pulmonary sarcoidosis	Male	Outdoor Air Pollution (PM2.5)		X			X	
Interstitial lung disease and pulmonary sarcoidosis	Female	Outdoor Air Pollution (PM2.5)		X		X		
Interstitial lung disease and pulmonary sarcoidosis	Female	Outdoor Air Pollution (PM2.5)		X			X	
Interstitial lung disease and pulmonary sarcoidosis	Female	Outdoor Air Pollution (PM2.5)	X			X		
Interstitial lung disease and pulmonary sarcoidosis	Male	Smoking Prevalence	X			X		
Interstitial lung disease and pulmonary sarcoidosis	Female	Smoking Prevalence	X			X		
Interstitial lung disease and pulmonary sarcoidosis	Male	Cumulative Cigarettes (5 Years)	X			X		
Interstitial lung disease and pulmonary sarcoidosis	Female	Outdoor Air Pollution (PM2.5)	X				X	
Other chronic respiratory diseases	Male	Outdoor Air Pollution (PM2.5)	X			X		
Other chronic respiratory diseases	Female	Outdoor Air Pollution (PM2.5)	X			X		
Other chronic respiratory diseases	Female	Elevation Over 1500m (proportion)		X			X	
Other chronic respiratory diseases	Male	Elevation Over 1500m (proportion)		X			X	
Other chronic	Male	Indoor Air Pollution	X			X		

Cause	Sex	Covariate	Level 1: GBD 2019	Level 2: GBD 2019	Level 3: GBD 2019	Level 1: GBD 2017	Level 2: GBD 2017	Level 3: GBD 2017
respiratory diseases		(All Cooking Fuels)						
Other chronic respiratory diseases	Female	Indoor Air Pollution (All Cooking Fuels)	X			X		
Other chronic respiratory diseases	Male	Healthcare access and quality index		X			X	
Other chronic respiratory diseases	Female	Healthcare access and quality index		X			X	
Other chronic respiratory diseases	Male	Socio-demographic Index			X			X
Other chronic respiratory diseases	Female	Socio-demographic Index			X			X
Other chronic respiratory diseases	Male	LDI (I$ per capita)			X			X
Other chronic respiratory diseases	Female	Population Density (over 1000 ppl/sqkm, proportion)		X			X	
Other chronic respiratory diseases	Male	Population Density (over 1000 ppl/sqkm, proportion)		X			X	
Other chronic respiratory diseases	Female	Cumulative Cigarettes (5 Years)	X			X		
Other chronic respiratory diseases	Male	Cumulative Cigarettes (5 Years)	X			X		
Other chronic respiratory diseases	Female	Elevation 500 to 1500m (proportion)		X			X	
Other chronic respiratory diseases	Male	Elevation 500 to 1500m (proportion)		X			X	
Other chronic respiratory diseases	Female	LDI (I$ per capita)			X			X
Other chronic respiratory diseases	Male	Education (years per capita)			X			X
Other chronic respiratory diseases	Female	Education (years per capita)			X			X
Other chronic respiratory diseases	Female	Education (years per capita)			X			X
Other chronic respiratory diseases	Female	Smoking Prevalence	X			X		
Other chronic respiratory diseases	Male	Smoking Prevalence	X			X		

Table S7. SDI quintiles for countries estimated in GBD 2019

SDI Quintile	Locations included based on SDI values in 2019 from GBD 2019 results
High SDI	Andorra, Australia, Austria, Belgium, Bermuda, Brunei, Canada, Cyprus, Czechia, Denmark, Estonia, Finland, France, Germany, Guam, Iceland, Ireland,Japan, Kuwait, Latvia, Lithuania, Luxembourg, Monaco, Netherlands, New Zealand, Norway, Puerto Rico, Qatar, San Marino, Saudi Arabia, Singapore, Slovakia, Slovenia, South Korea, Sweden, Switzerland, Taiwan (Province of China), United Arab Emirates, United Kingdom, United States of America
High-middle SDI	American Samoa, Antigua and Barbuda, Argentina, The Bahamas, Bahrain, Barbados, Belarus, Bosnia and Herzegovina, Bulgaria, Chile, Cook Islands, Croatia, Dominica, Georgia, Greece, Greenland, Hungary, Israel, Italy, Jordan, Kazakhstan, Lebanon, Libya, Malaysia, Malta, Mauritius, Moldova, Montenegro,Niue, North Macedonia, Northern Mariana Islands, Oman, Palau, Poland, Portugal, Romania, Russia, Saint Kitts and Nevis, Serbia, Seychelles, Spain, Sri Lanka, Trinidad and Tobago, Turkey, Ukraine, Virgin Islands, Uruguay
Middle SDI	Albania, Algeria, Armenia, Azerbaijan, Botswana, Brazil, China, Colombia,Costa Rica, Cuba, Ecuador, Egypt, Equatorial Guinea, Fiji, Gabon, Grenada,Guyana, Indonesia, Iran, Iraq, Jamaica, Mexico, Namibia, Nauru, Panama, Paraguay, Peru, Philippines, Saint Lucia, Saint Vincent and the Grenadines, Samoa, South Africa, Suriname, Syria, Thailand, Tokelau, Tonga, Tunisia, Turkmenistan, Uzbekistan, Vietnam
Low-middle SDI	Angola, Bangladesh, Belize, Bhutan, Bolivia, Cambodia, Cameroon, Cape Verde,Congo (Brazzaville), Djibouti, Dominican Republic, El Salvador, Eswatini, Federated States of Micronesia, Ghana, Guatemala, Honduras, India, Kenya, Kiribati, Kyrgyzstan, Laos, Lesotho, Maldives, Marshall Islands, Mauritania, Mongolia, Morocco, Myanmar, Nicaragua, Nigeria, North Korea, Palestine, São Tomé and Príncipe, Sudan, Tajikistan, Timor-Leste, Tuvalu, Vanuatu, Venezuela,Zambia, Zimbabwe
Low SDI	Afghanistan, Benin, Burkina Faso, Burundi, Central African Republic, Chad, Comoros, Côte d'Ivoire, DR Congo, Eritrea, Ethiopia, The Gambia, Guinea, Guinea-Bissau, Haiti, Liberia, Madagascar, Malawi, Mali, Mozambique, Nepal,Niger, Pakistan, Papua New Guinea, Rwanda, Senegal, Sierra Leone, Solomon Islands, Somalia, South Sudan, Tanzania, Togo, Uganda, Yemen

SDI = Socio-demographic Index; GBD = Global Burden of Disease study.

Figure S1. Classification of GBD regions and super regions

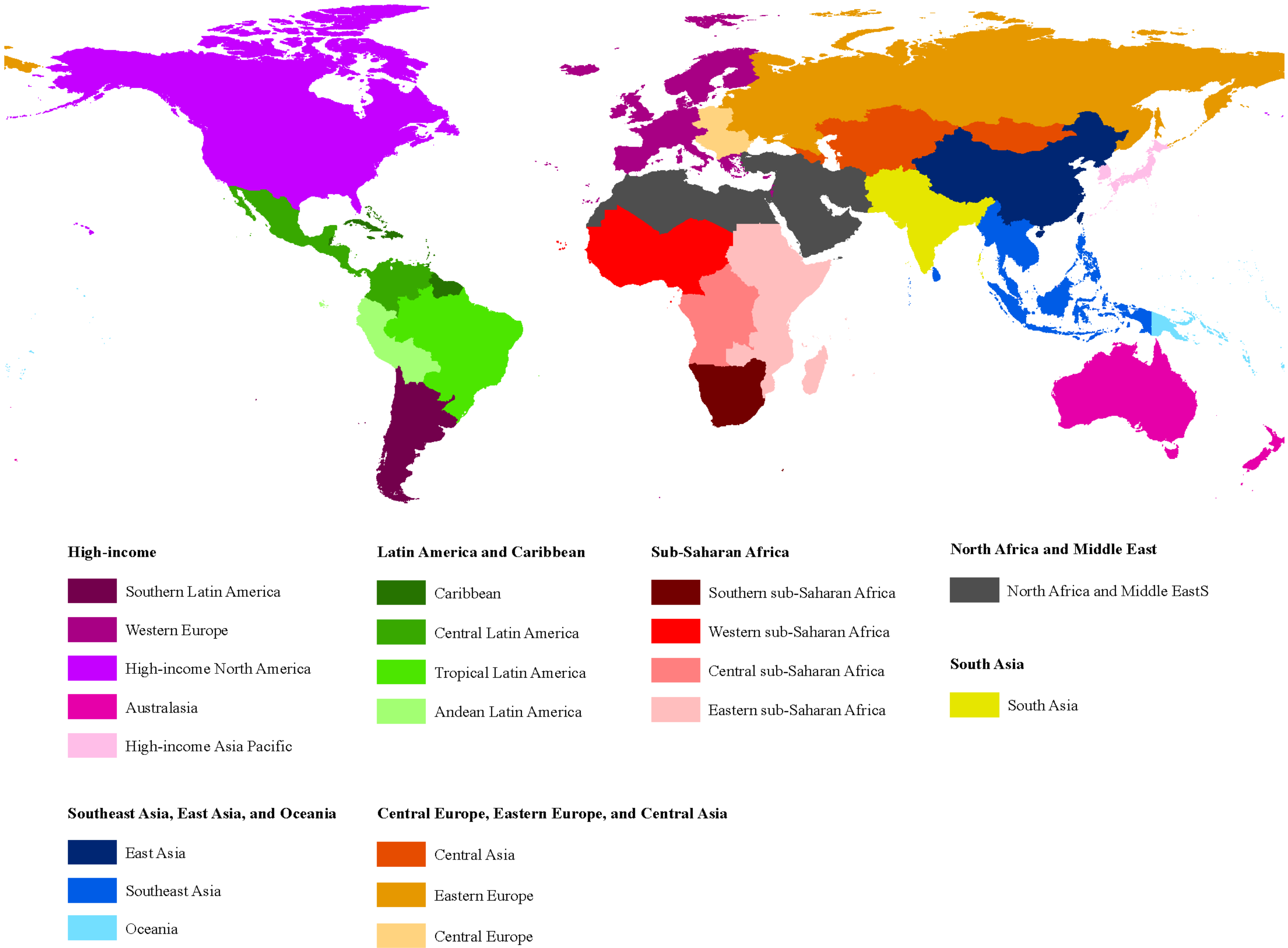

Appendix 2: Online Figures and Tables

Results appendix to "Global Burden of Chronic Respiratory Diseases and Risk Factors, 1990–2019: Update From the GBD 2019 Study"

This appendix provides more detailed results for "Global Burden of Chronic Respiratory Diseases and Risk Factors, 1990–2019: Update From the GBD 2019 Study".

B.3 Chronic respiratory diseases

Figure S1. Age-standardised rate of deaths and DALYs from chronic respiratory diseases in 2019 and their percent change from 1990 in men

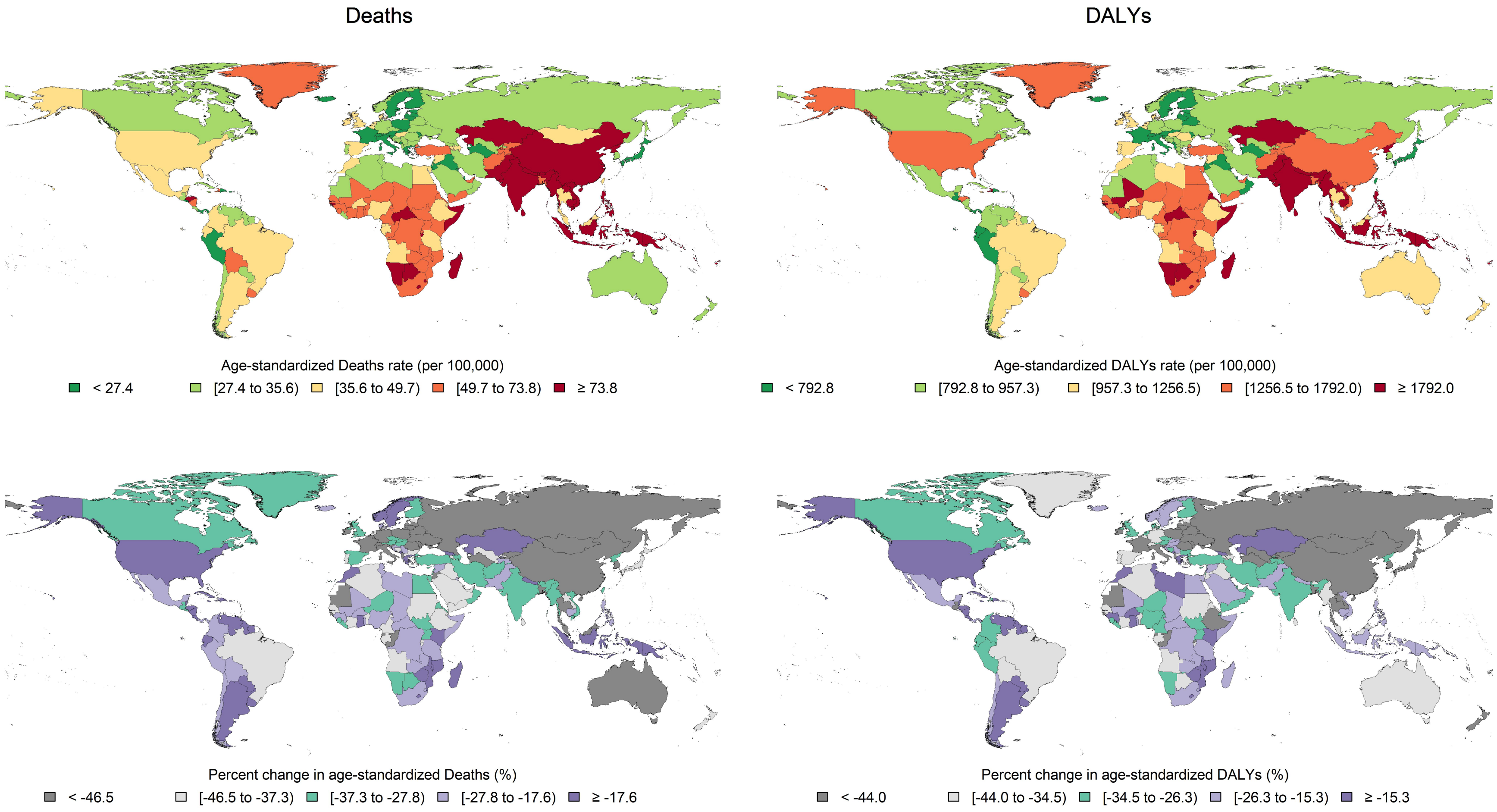

Figure S2. Age-standardised rate of deaths and DALYs from chronic respiratory diseases in 2019 and their percent change from 1990 to 2019 in women

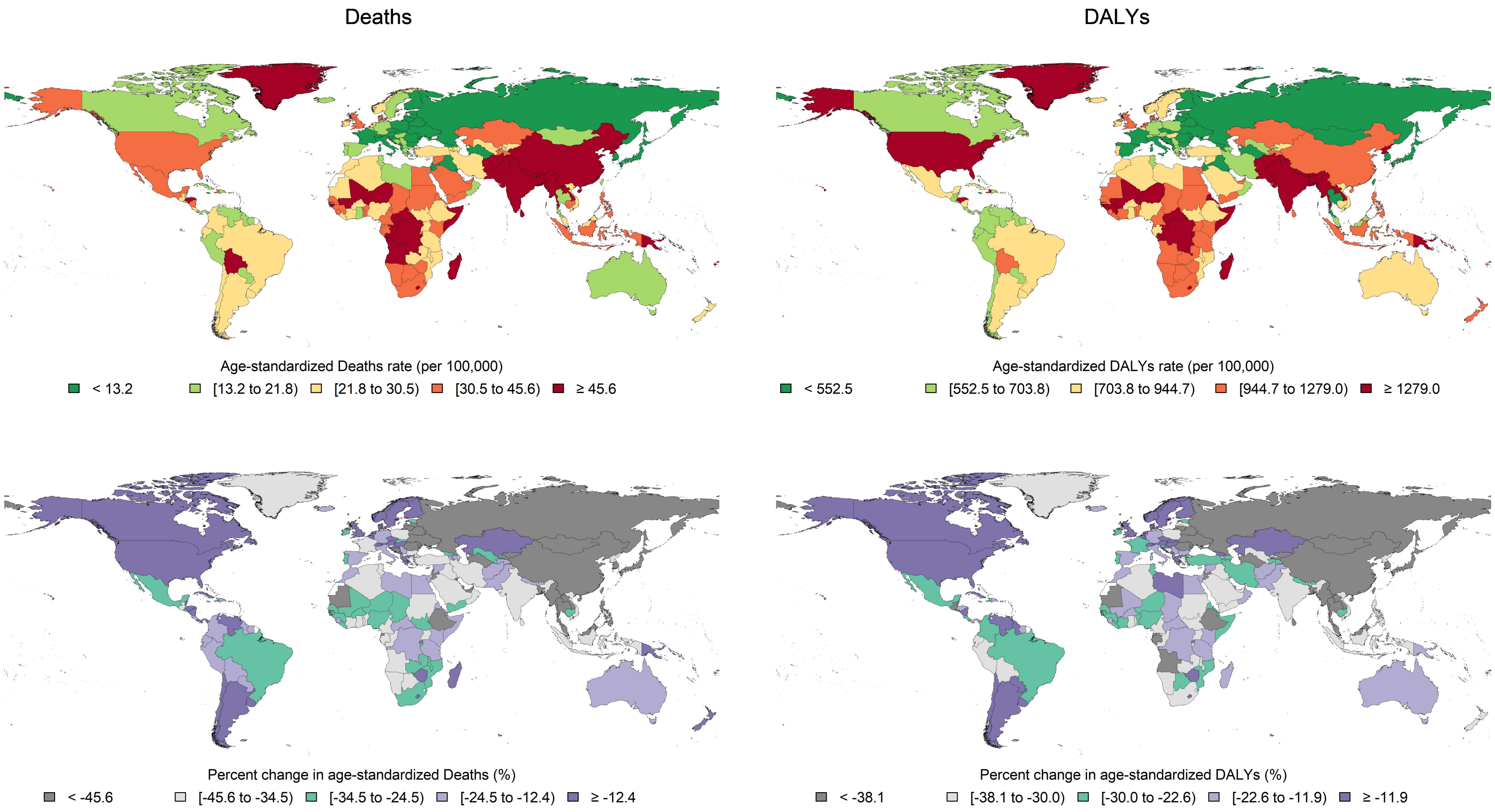

Figure S3. Age-standardised rate of prevalence and incidence of chronic respiratory diseases in 2019 and their percent change from 1990 to 2019 in both sexes

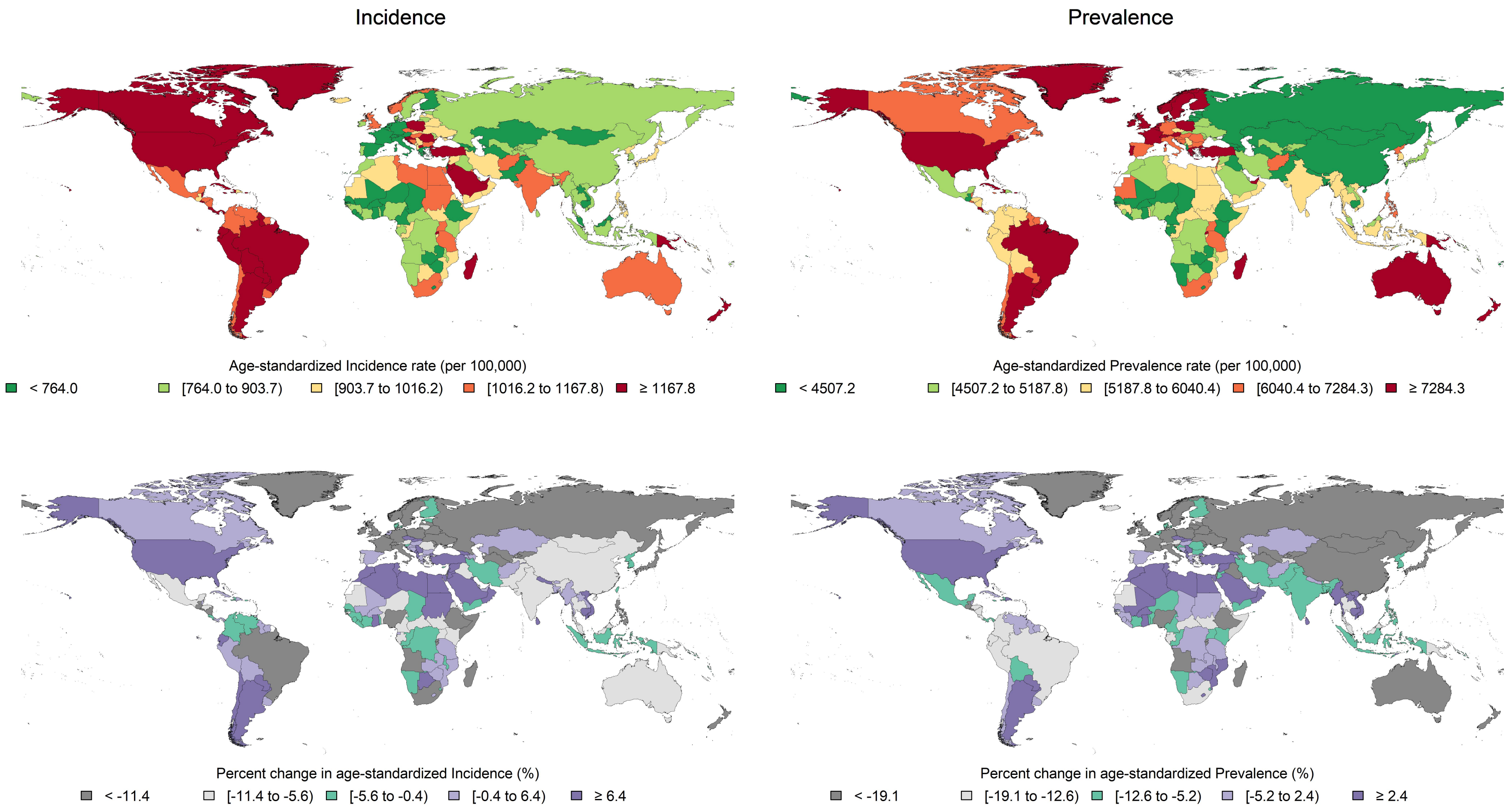

Figure S4. Age-standardised rate of prevalence and incidence of chronic respiratory diseases in 2019 and their percent change from 1990 to 2019 in men

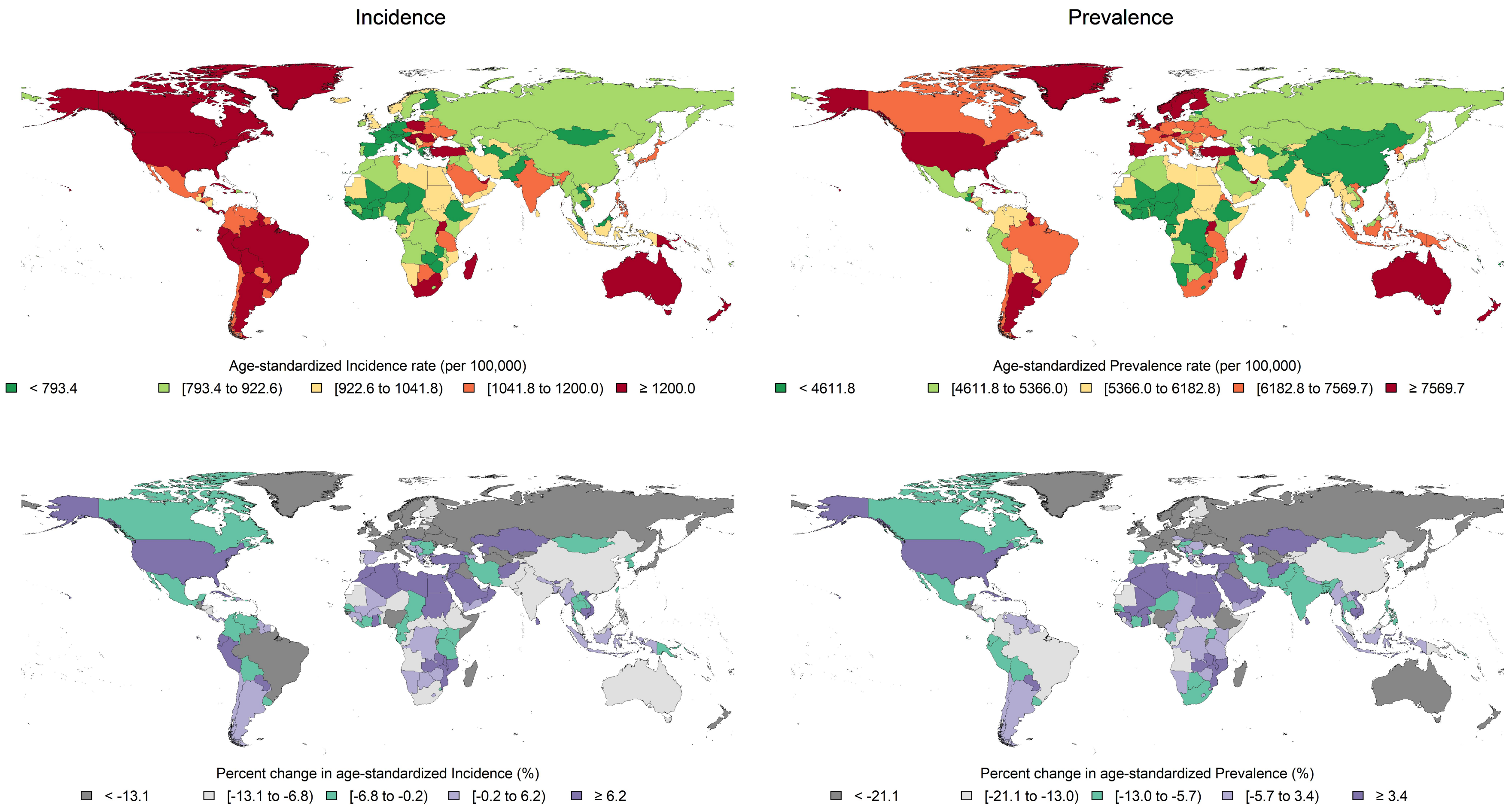

Figure S5. Age-standardised rate of prevalence and incidence of chronic respiratory diseases in 2019 and their percent change from 1990 to 2019 in women

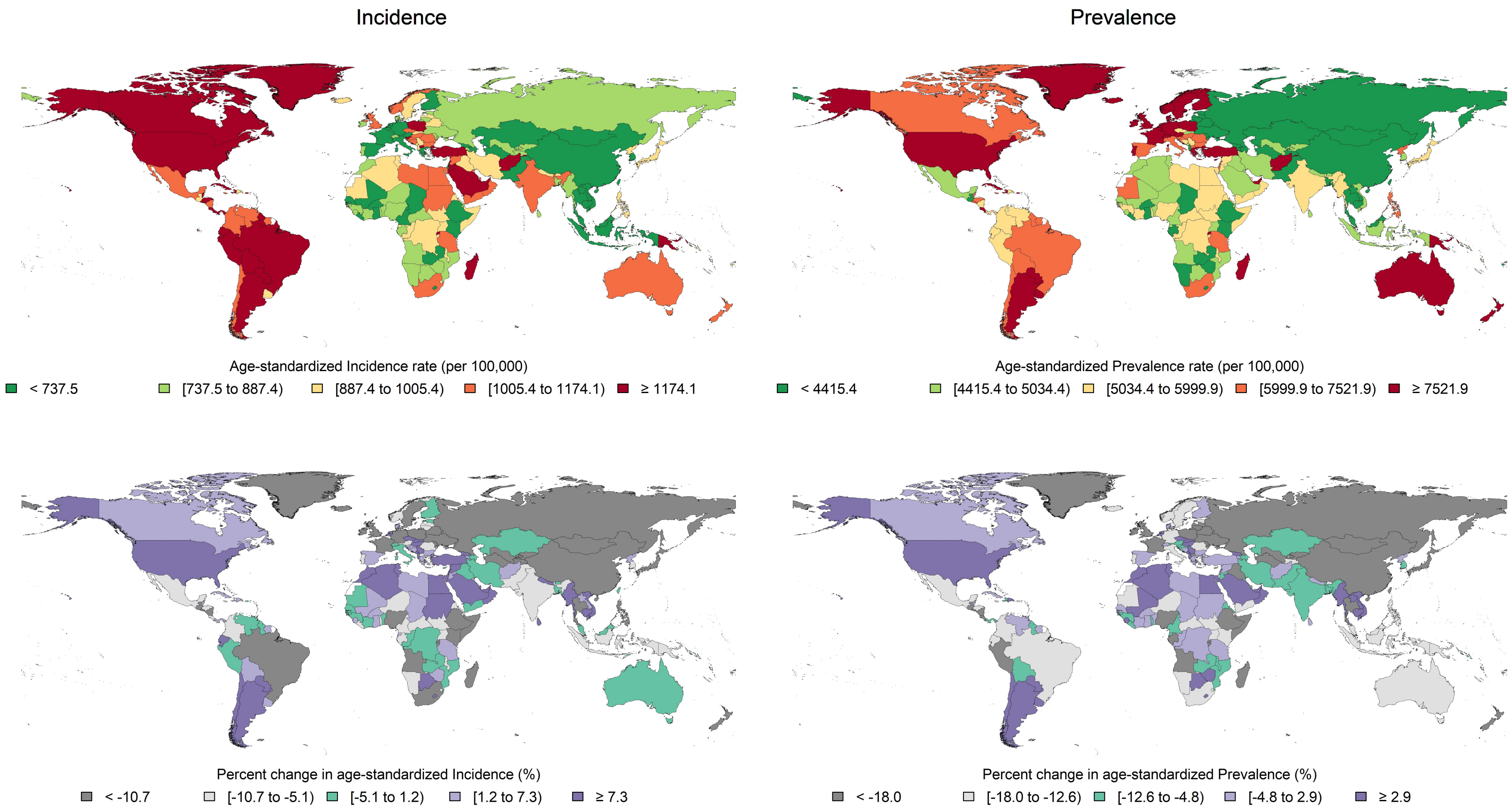

Figure S6. Absolute death rate due to chronic respiratory diseases by age and sex, 1990 and 2019

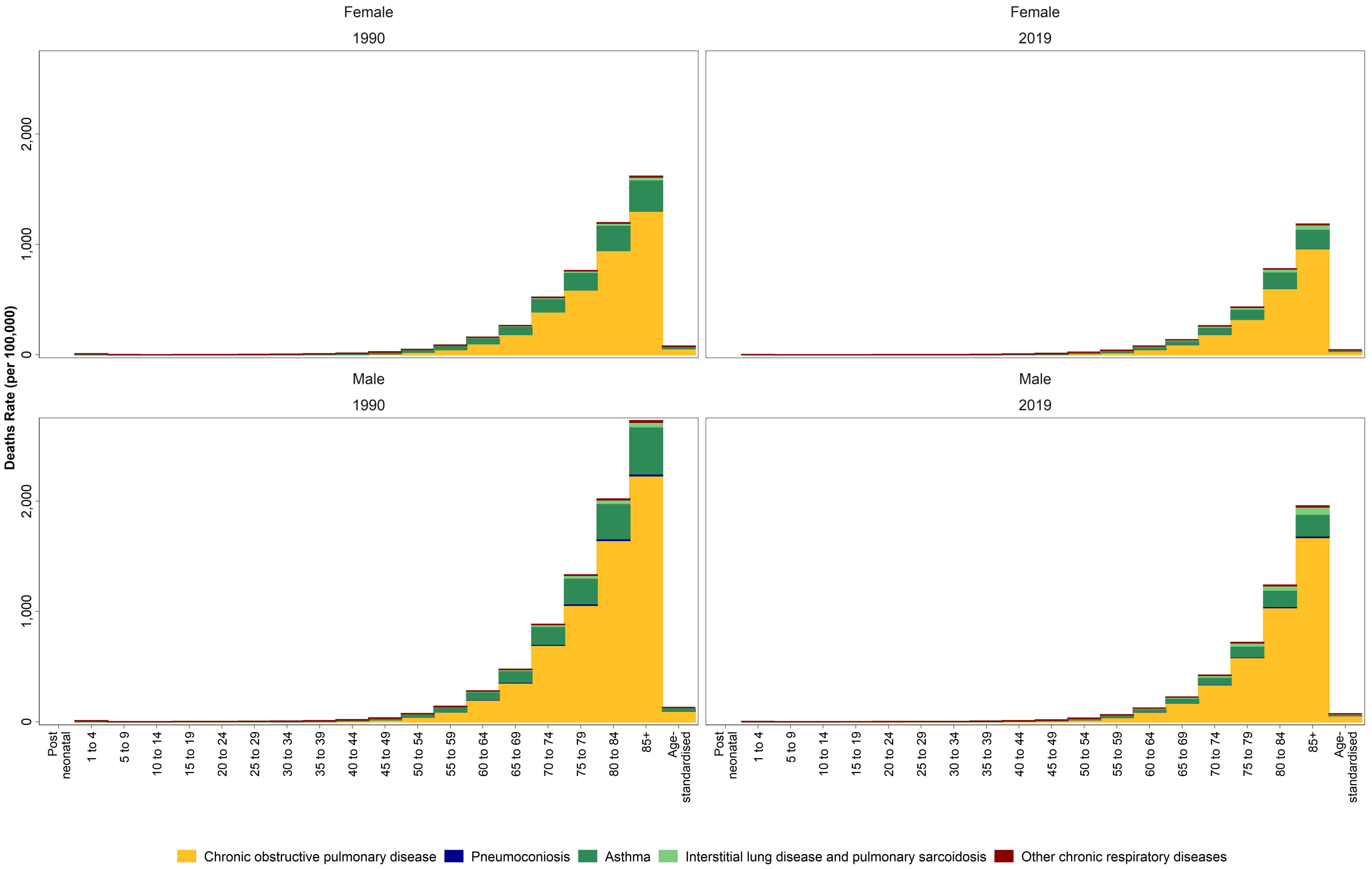

Figure S7. Absolute prevalence rate due to chronic respiratory diseases by age and sex, 1990 and 2019

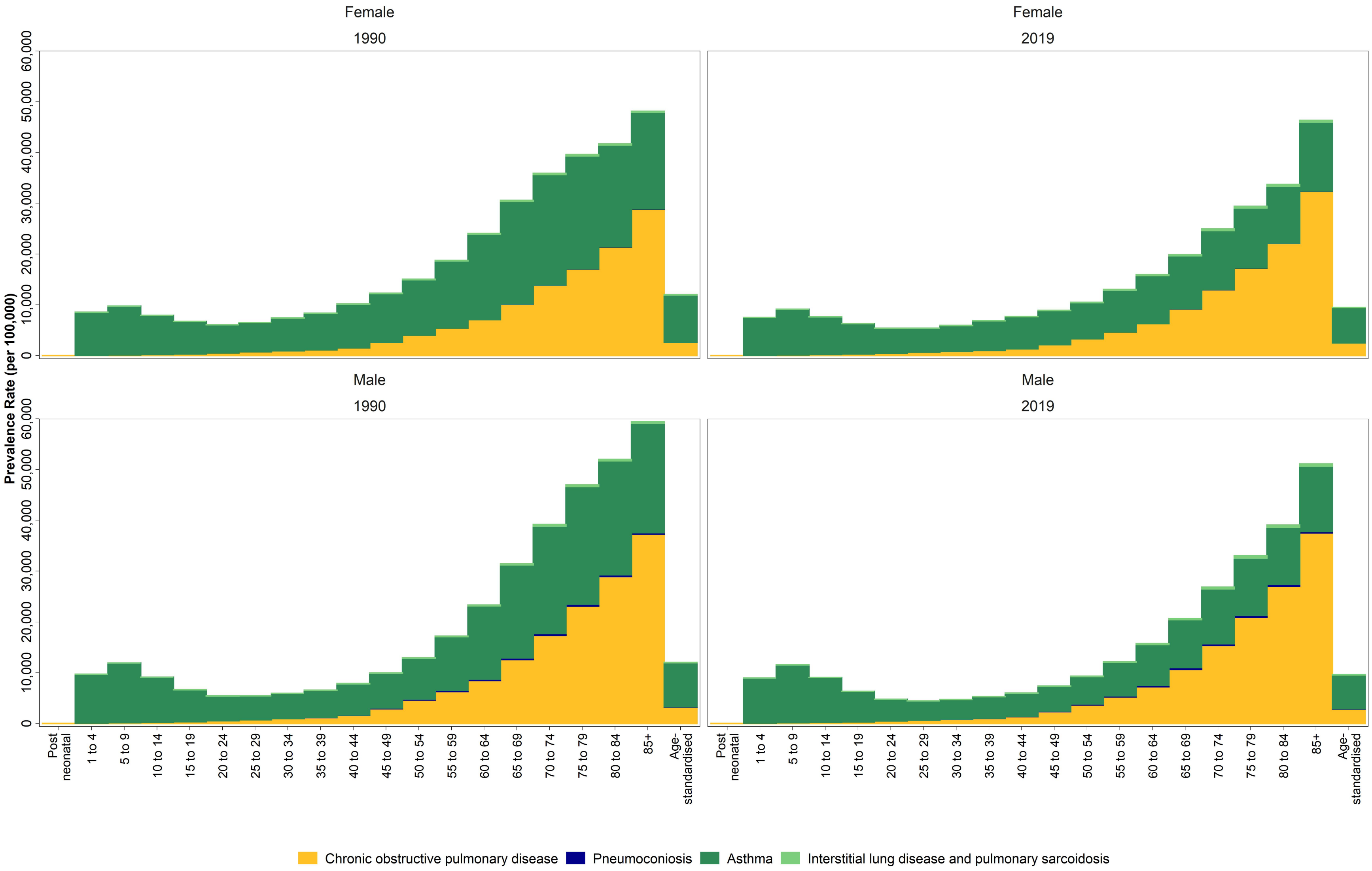

Figure S8. Absolute incidence rate due to chronic respiratory diseases by age, 2019 and sex, 1990 and 2019

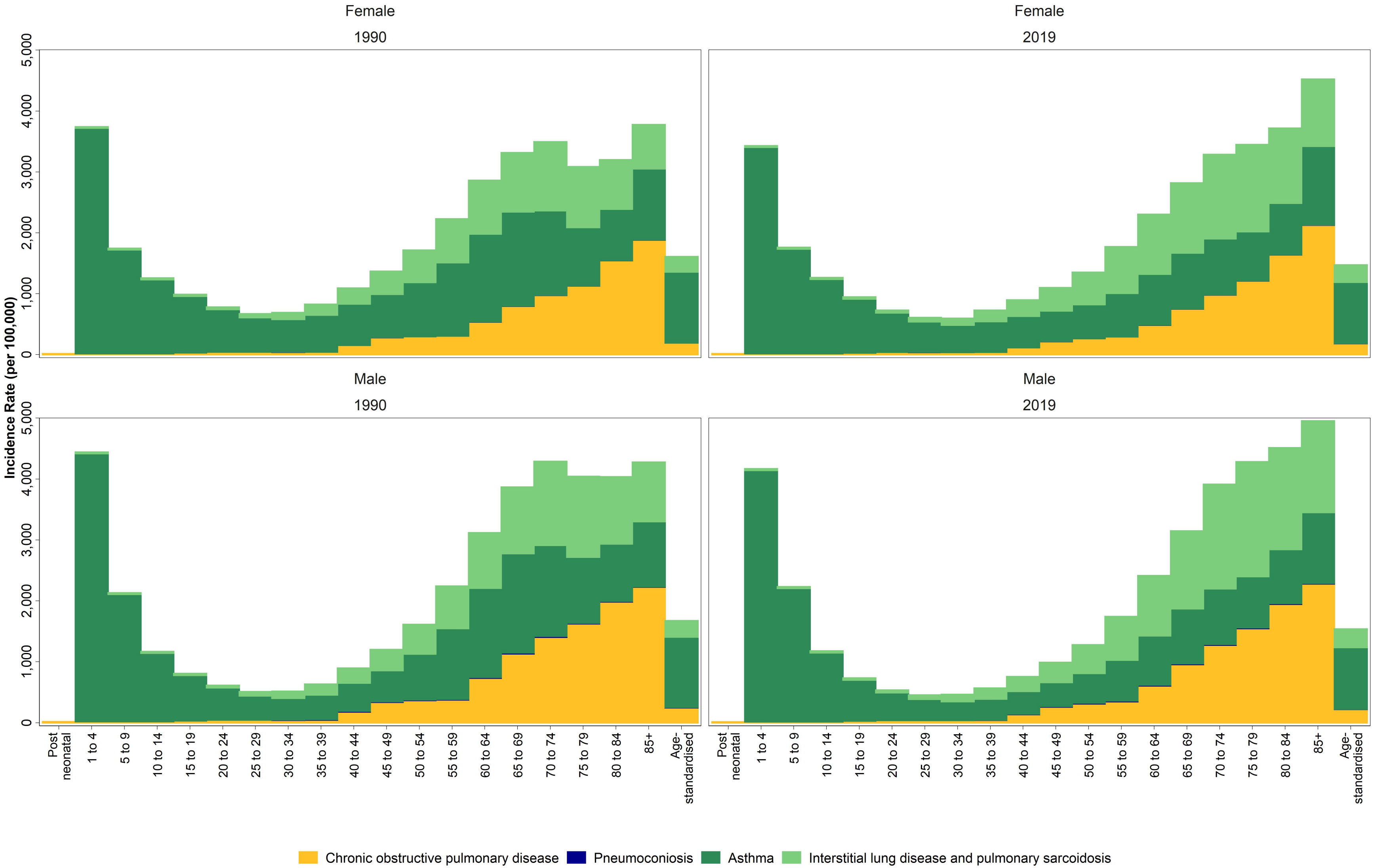

Figure S9. Contribution of YLLs and YLDs to age-standardised and age-specific DALYs from chronic respiratory diseases, 2019

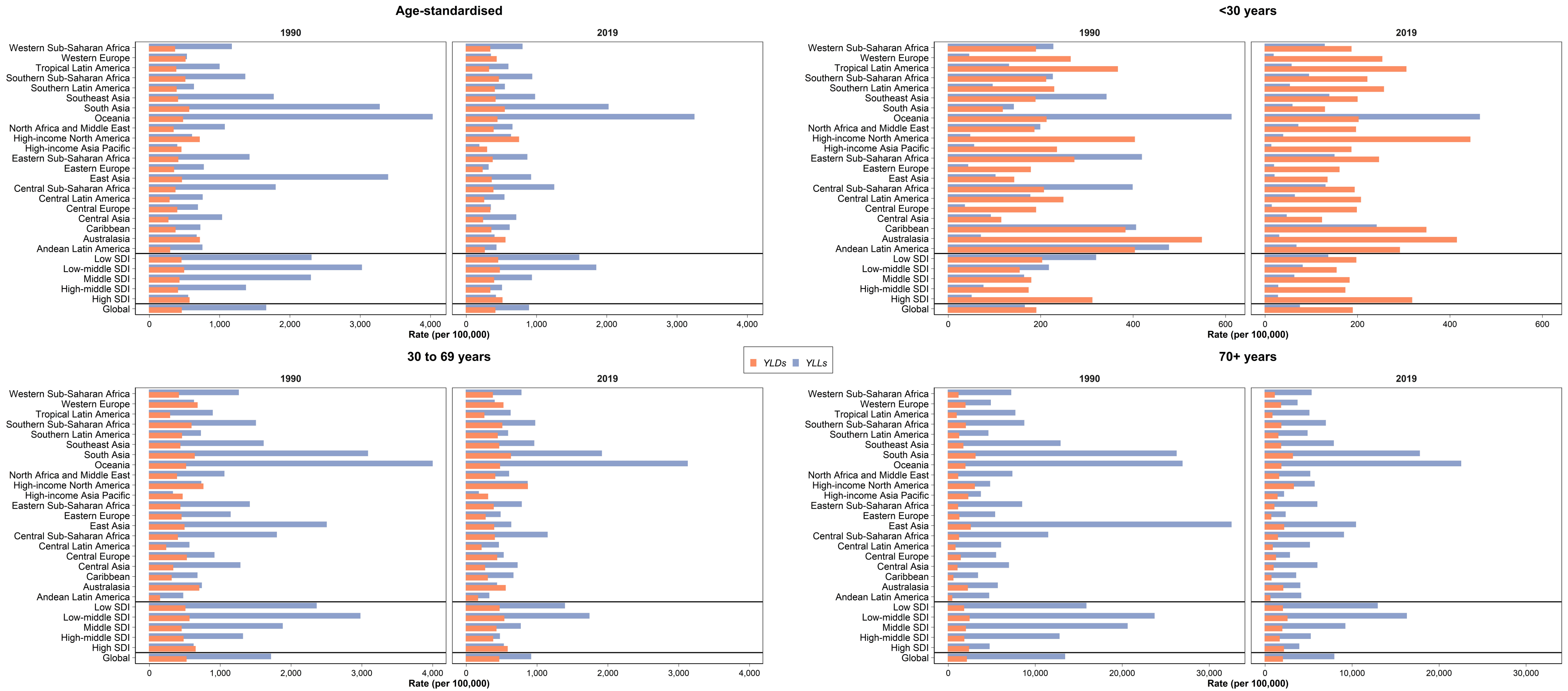

Figure S10. Correlation between the SDI and age-standardised DALYs rates due to chronic respiratory diseases in 1990, 2000, 2010, and 2019 globally

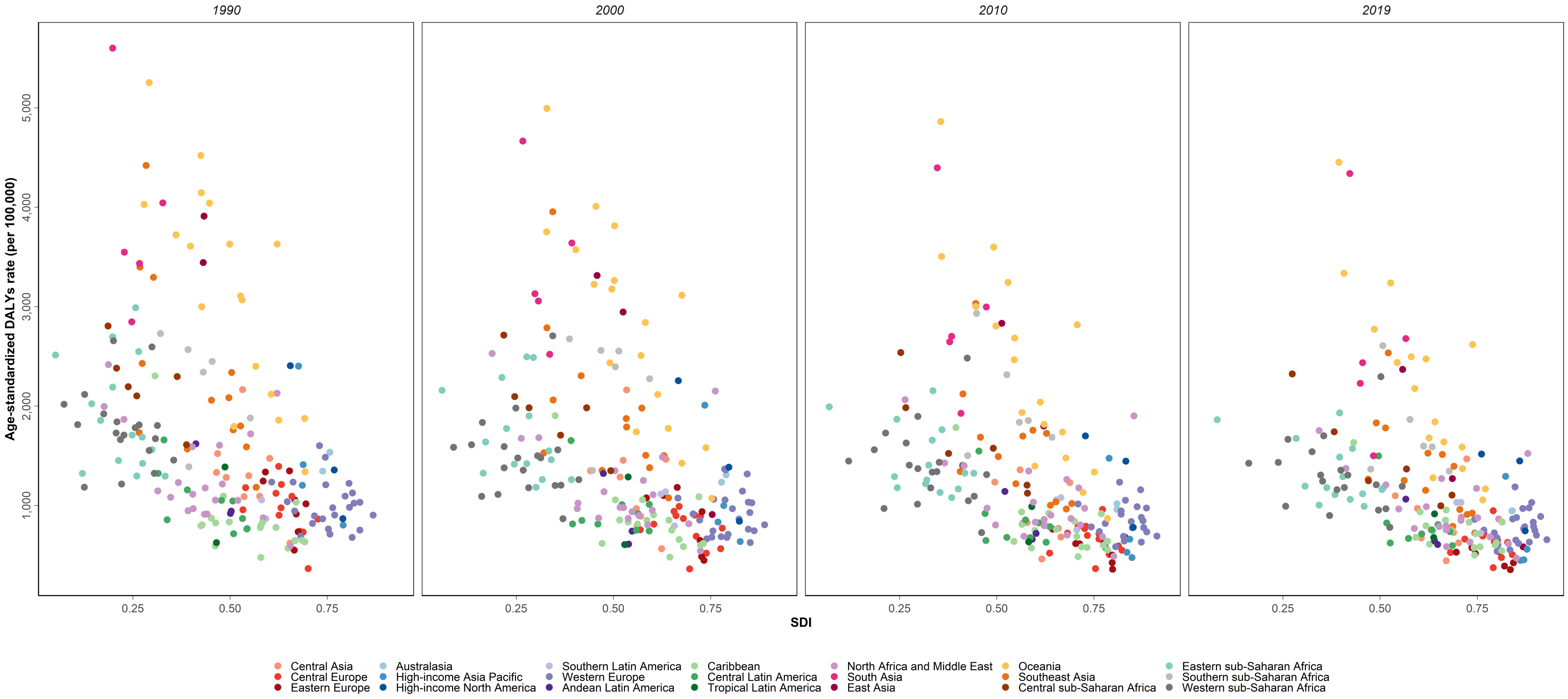

* Each country is represented by a point color coded according to the 21 GBD regions.

Figure S11. Correlation between the SDI and age-standardised death rates due to chronic respiratory diseases in 1990, 2000, 2010, and 2019 globally

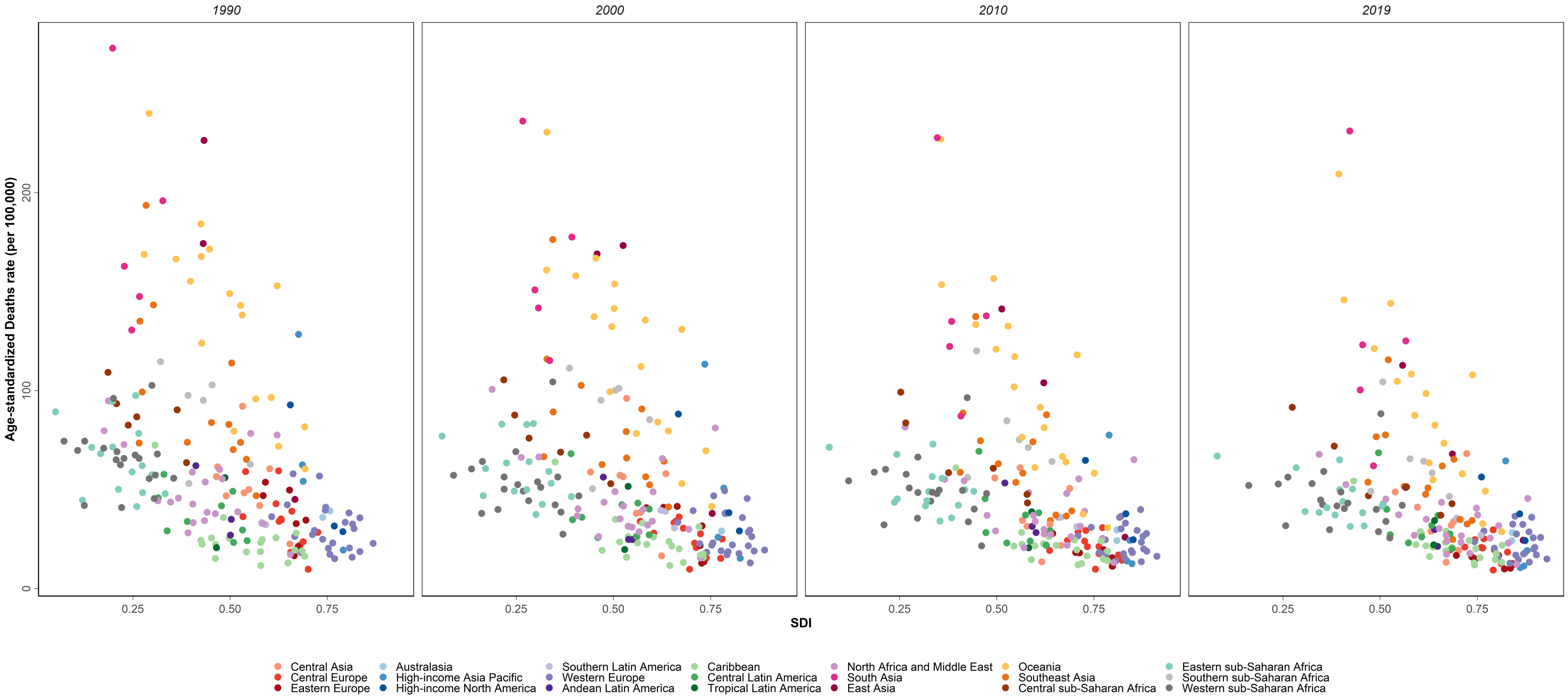

* Each country is represented by a point color coded according to the 21 GBD regions.

Figure S12. Correlation between the SDI and age-standardised prevalence rates due to chronic respiratory diseases in 1990, 2000, 2010, and 2019 globally

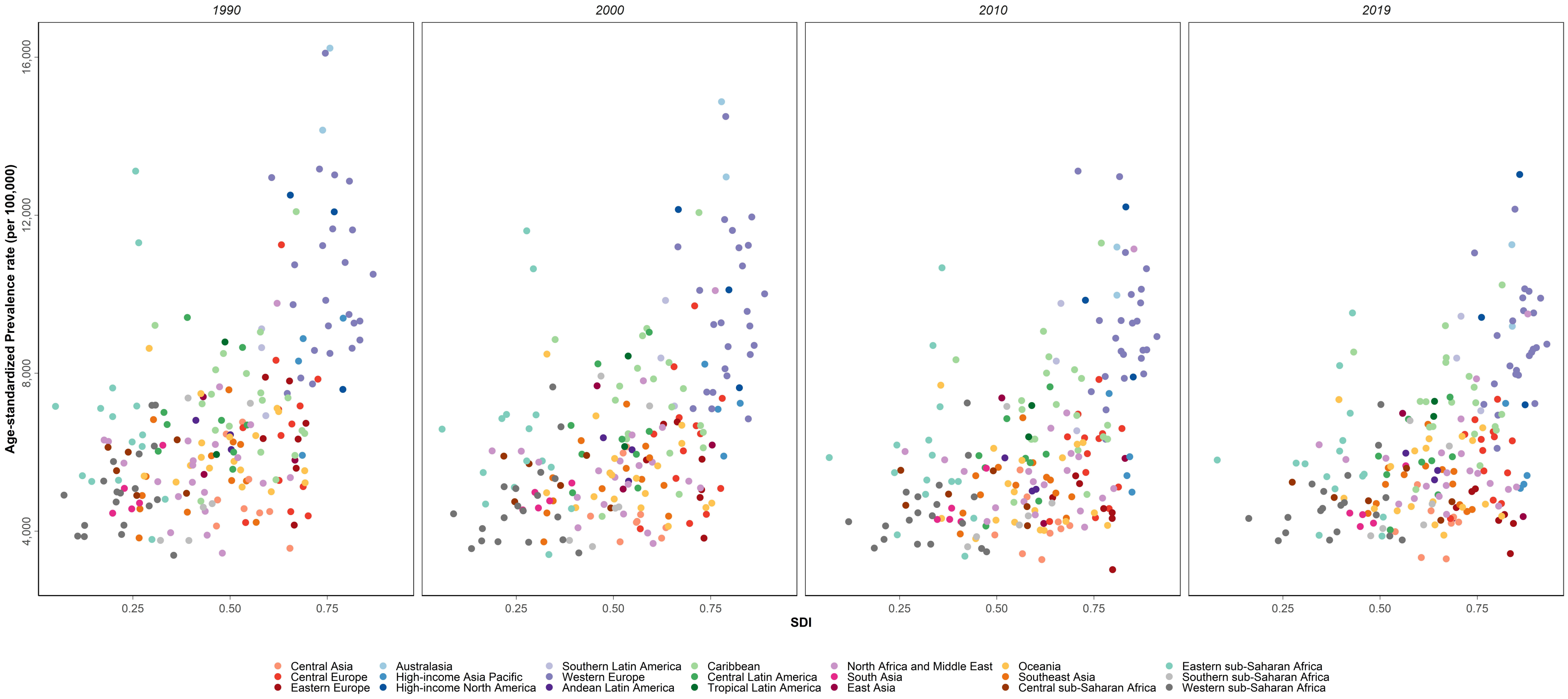

* Each country is represented by a point color coded according to the 21 GBD regions.

Figure S13. Correlation between the SDI and age-standardised incidence rates due to chronic respiratory diseases in 1990, 2000, 2010, and 2019 globally

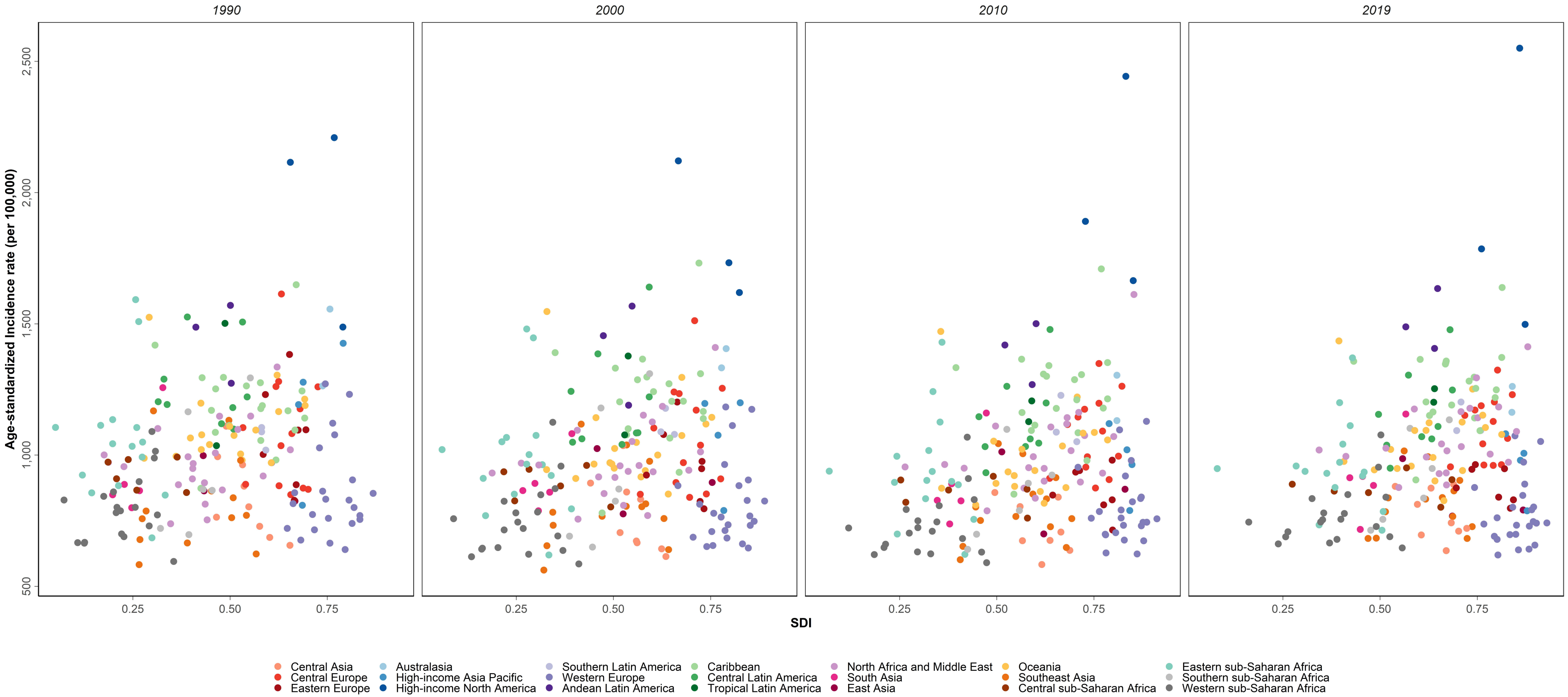

* Each country is represented by a point color coded according to the 21 GBD regions.

Figure S14. Ranked contribution of the specific sub-causes to the age-standardised DALYs from chronic respiratory diseases by region, 2019, for both sexes, females, and males

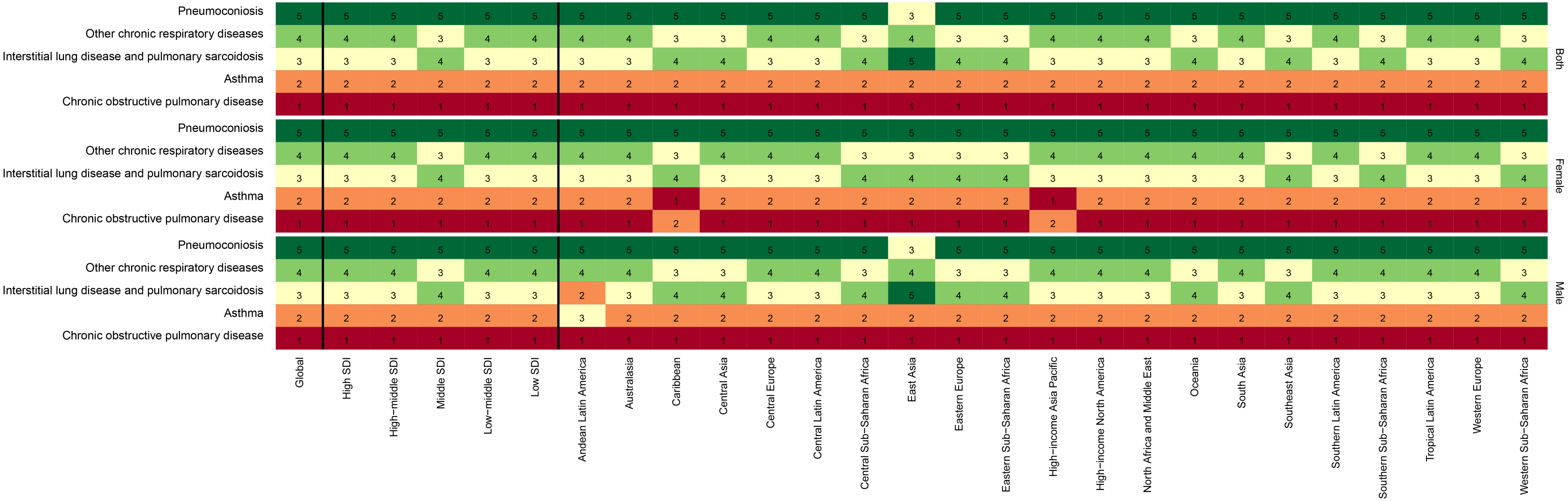

Figure S15. Ranked contribution of risk factors to the age-standardised death from chronic respiratory diseases by region, 2019, for both sexes, females, and males

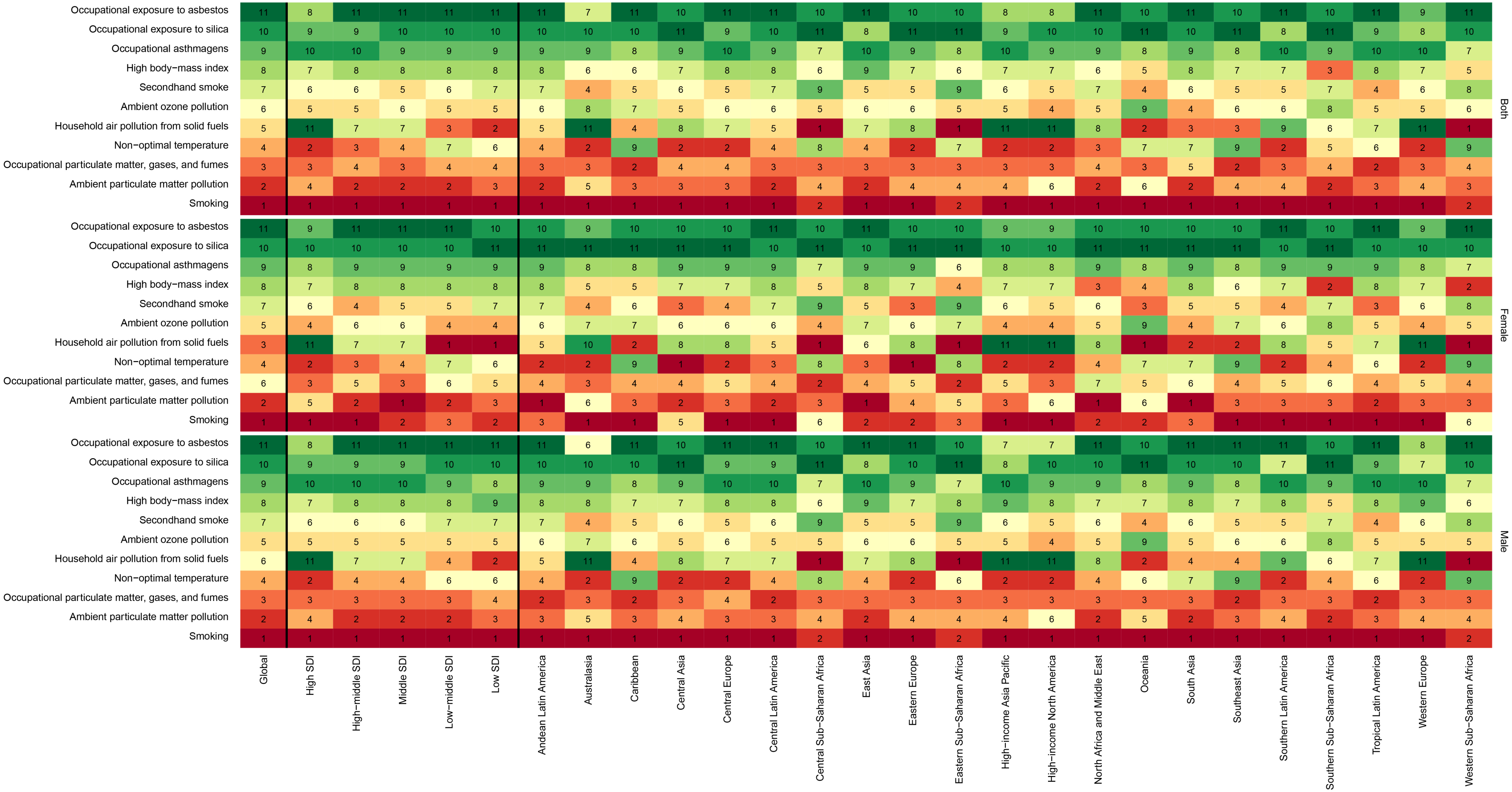

Figure S16. Age-standardised DALYs from chronic respiratory disease attributed to risk factors by sex and region in 1990 and 2019

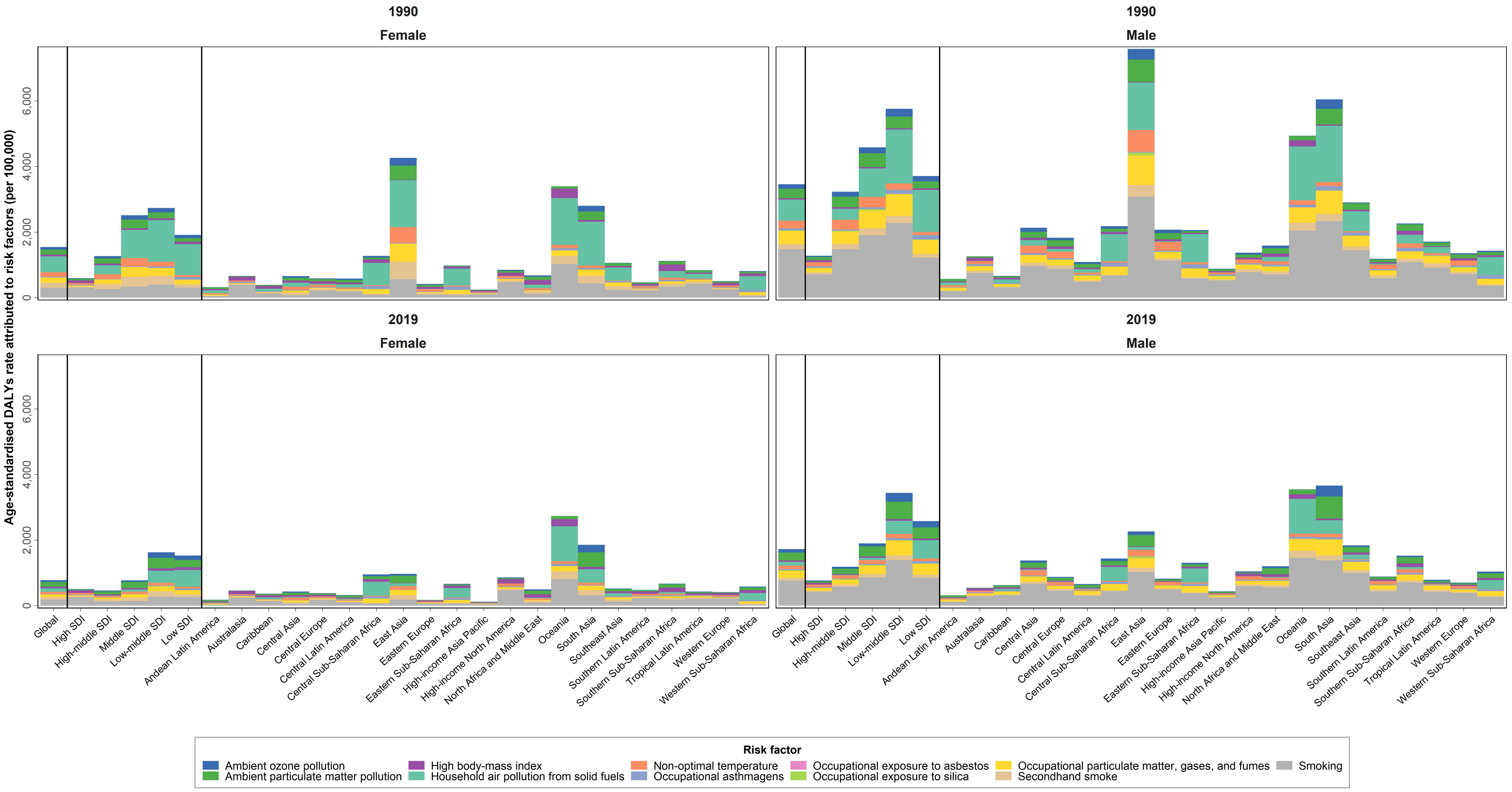

B.3.1 Chronic obstructive pulmonary diseases (COPD)

Figure S17. Global age-standardised rates of incidence, prevalence, deaths, and DALYs of COPD by sex, 1990-2019

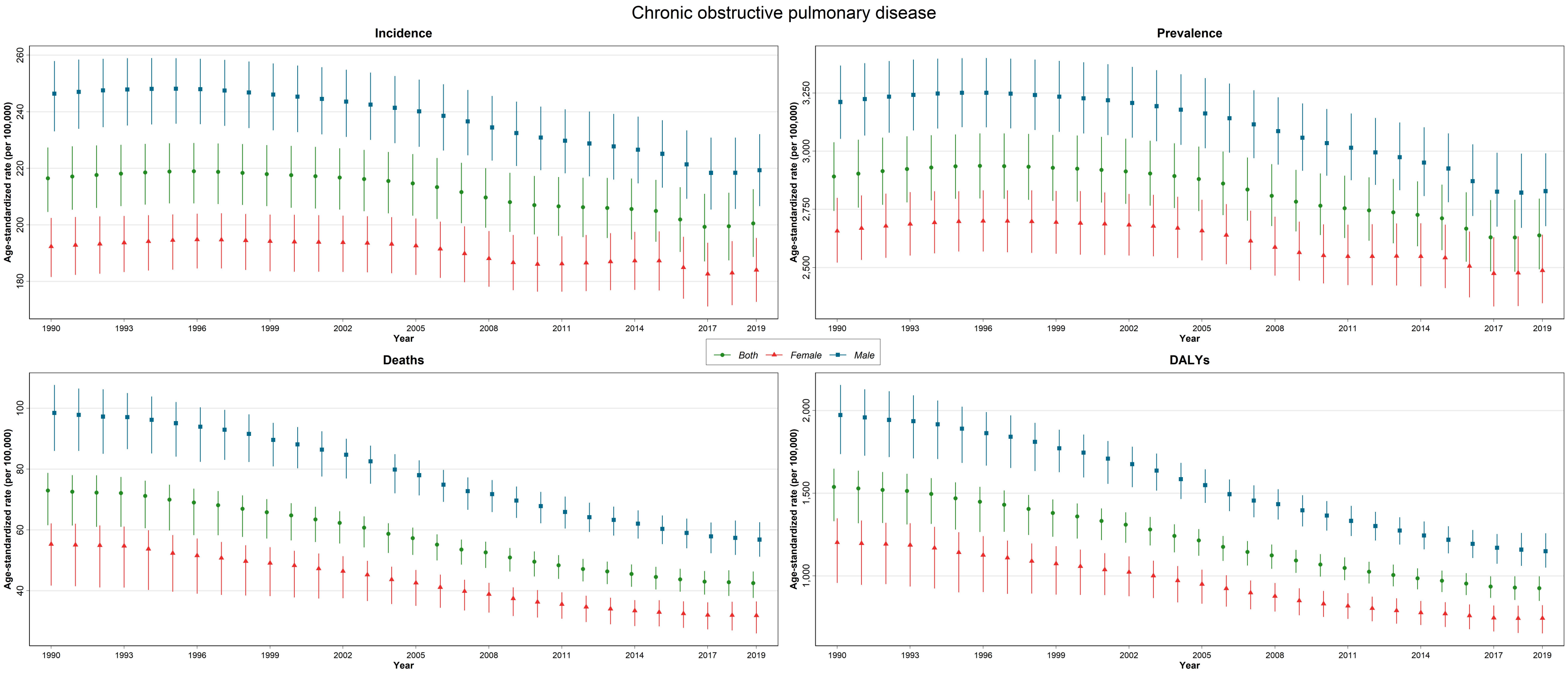

Figure S18. Global age-standardised rates of incidence, prevalence, deaths, and DALYs of COPD by SDI, both sexes, 1990-2019

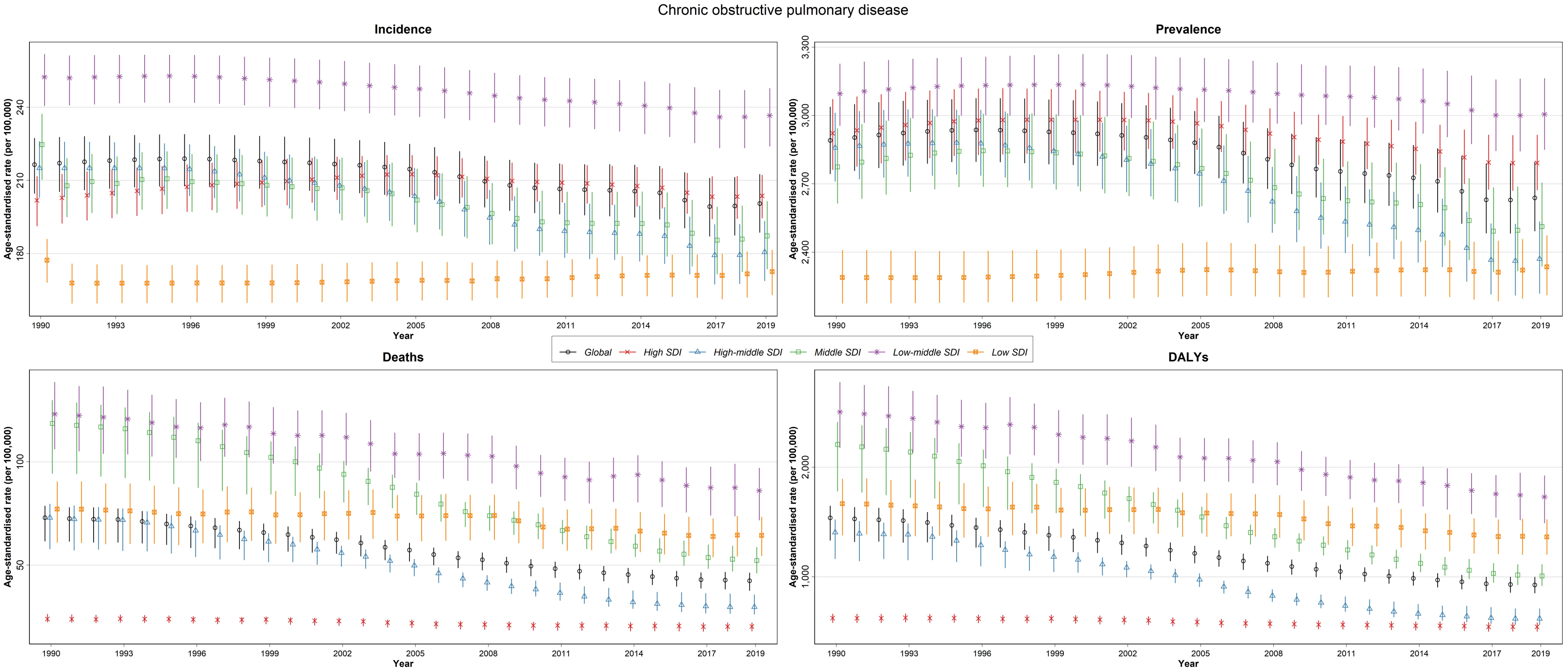

Figure S19. Ranked contribution of risk factors to the age-standardised DALYs from COPD by region, 2019, for both sexes, females, and males

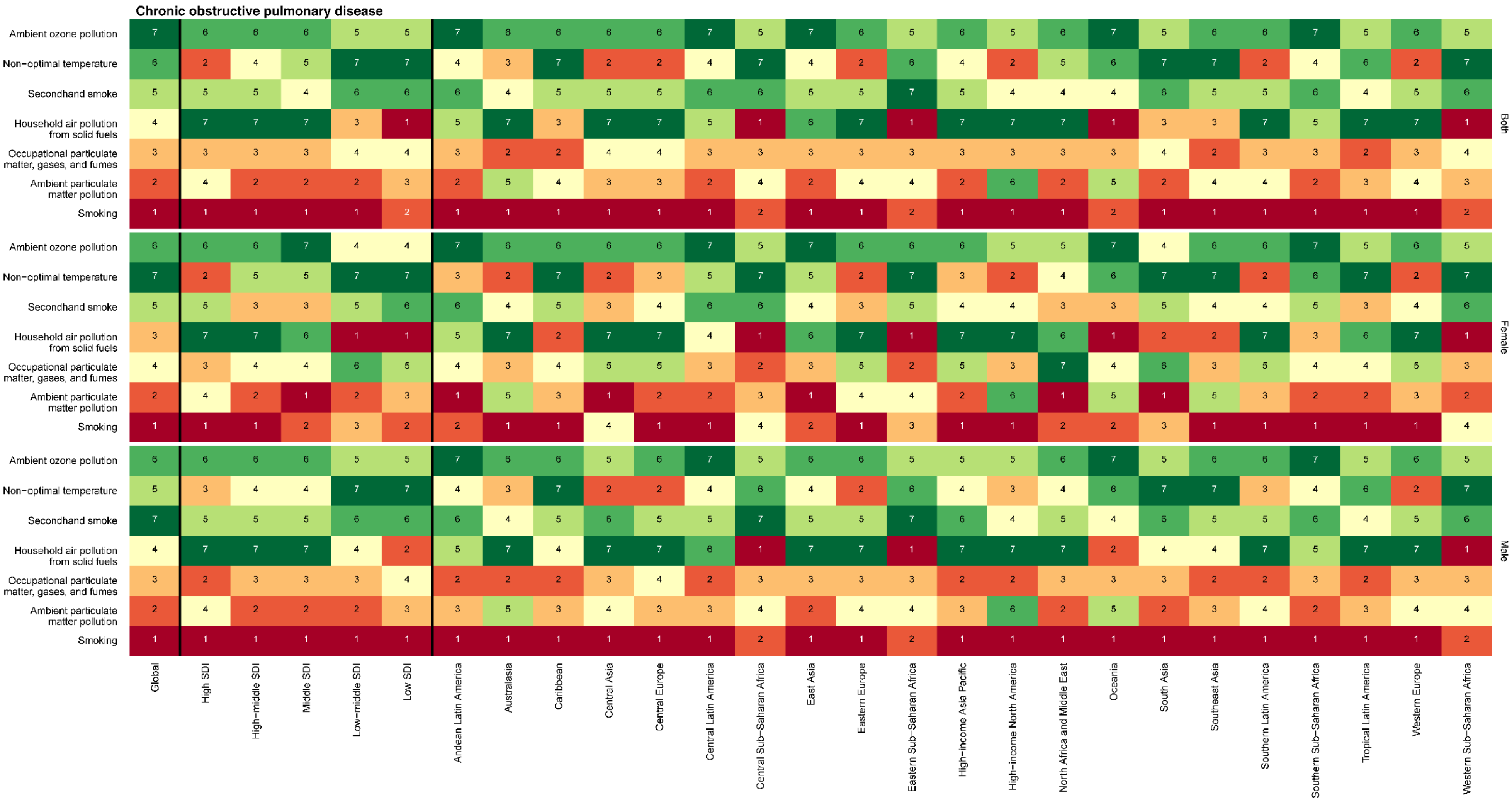

Figure S20. Age-standardised DALYs from COPD attributed to risk factors by sex and region in 1990 and 2019

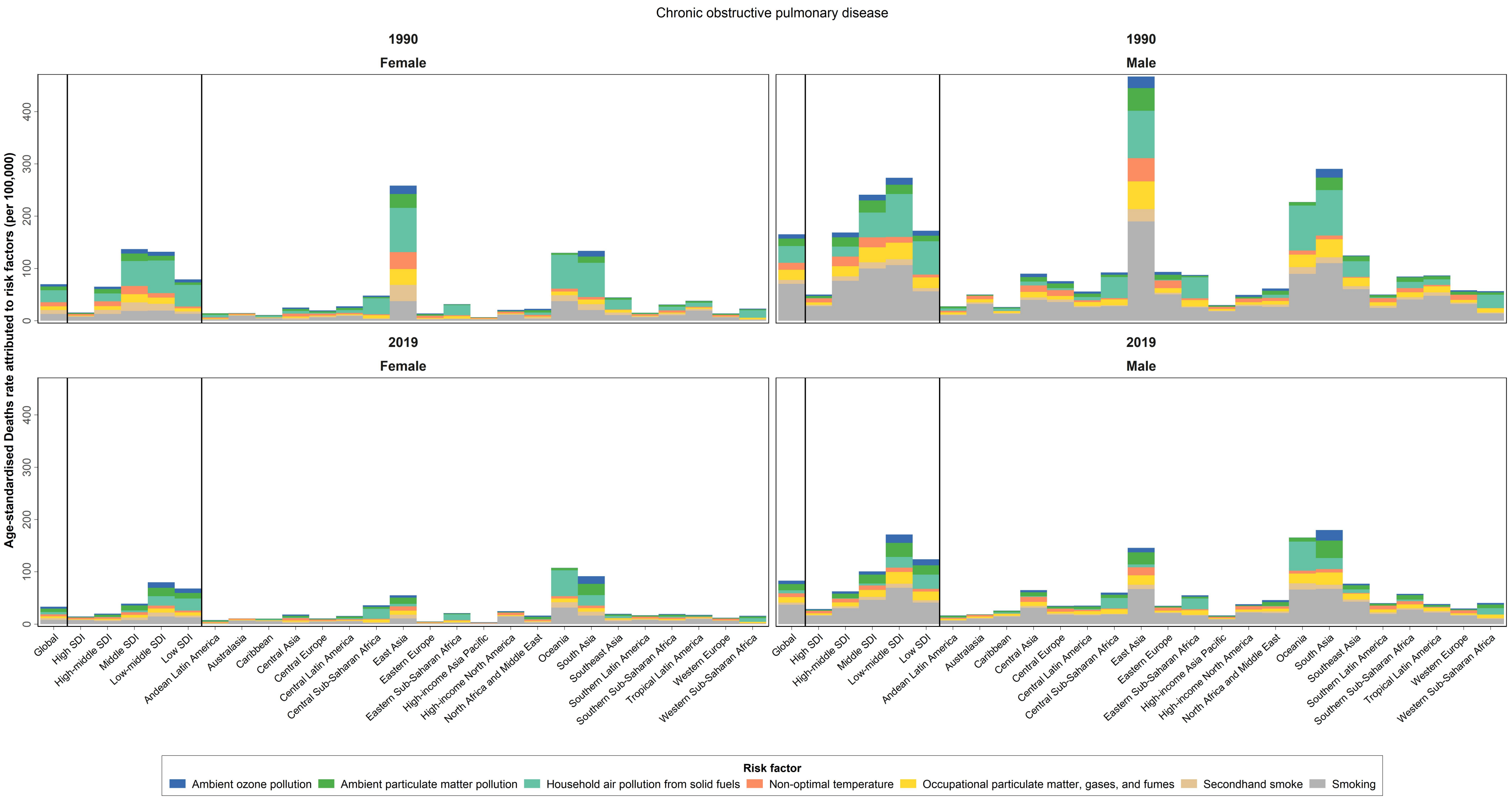

B.3.3 Asthma

Figure S21. Global age-standardised rates of incidence, prevalence, deaths, and DALYs of asthma by sex, 1990-2019

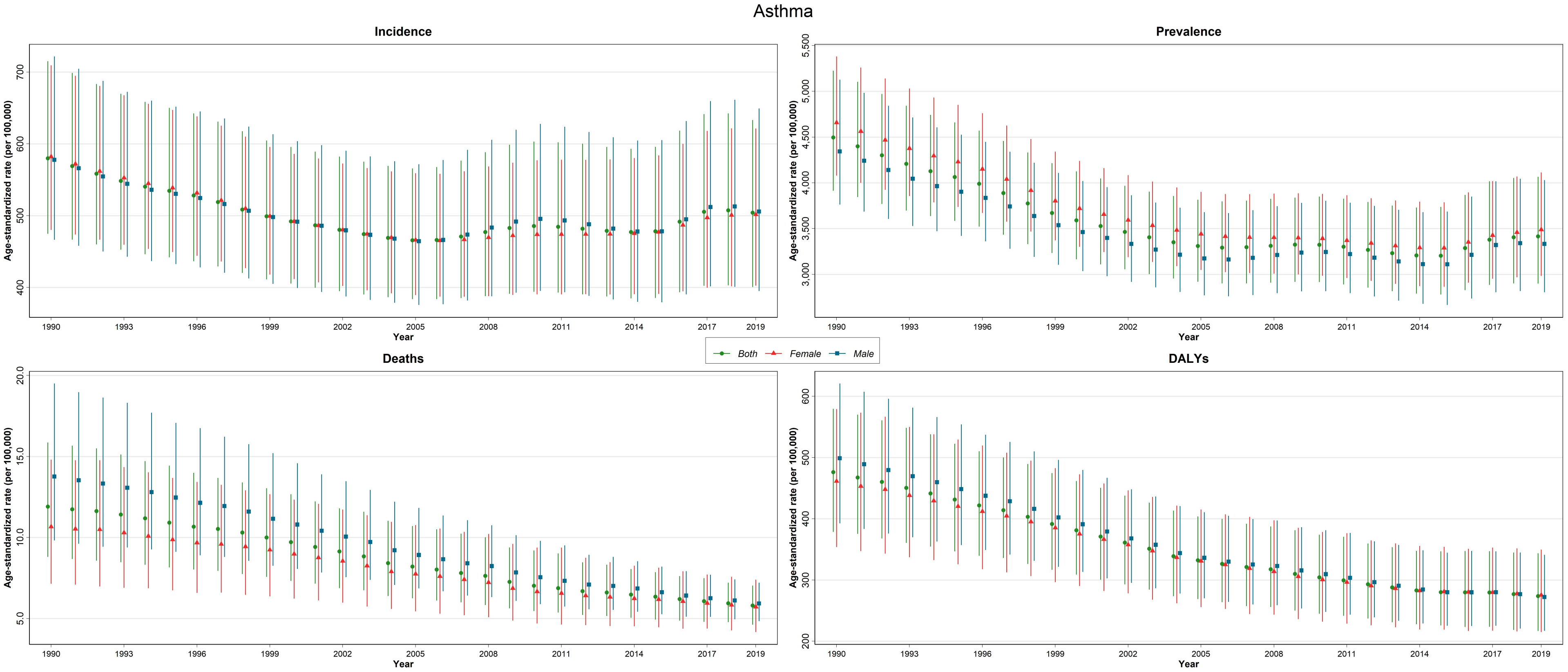

Figure S22. Global age-standardised rates of incidence, prevalence, deaths, and DALYs of asthma by SDI, both sexes, 1990-2019

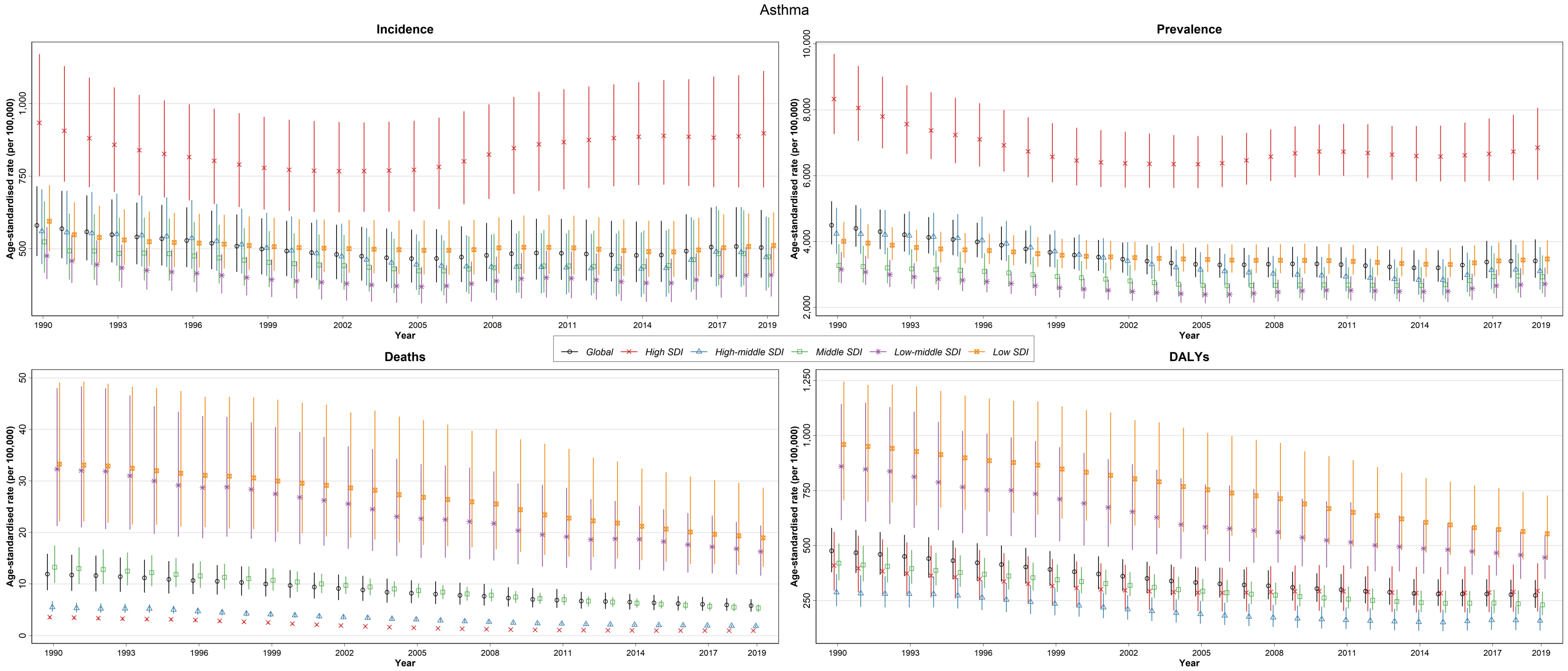

Figure S23. Ranked contribution of risk factors to the age-standardised DALYs from asthma by region, 2019, for both sexes, females, and males

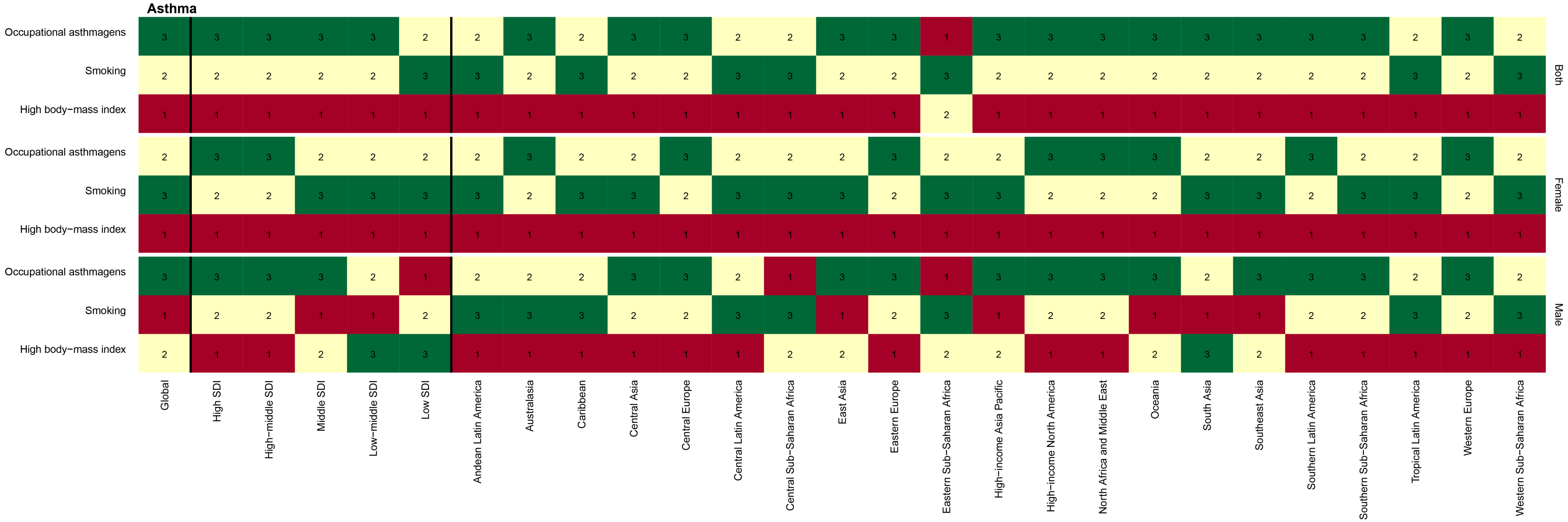

Figure S24. Age-standardised DALYs from asthma attributed to risk factors by sex and region in 1990 and 2019

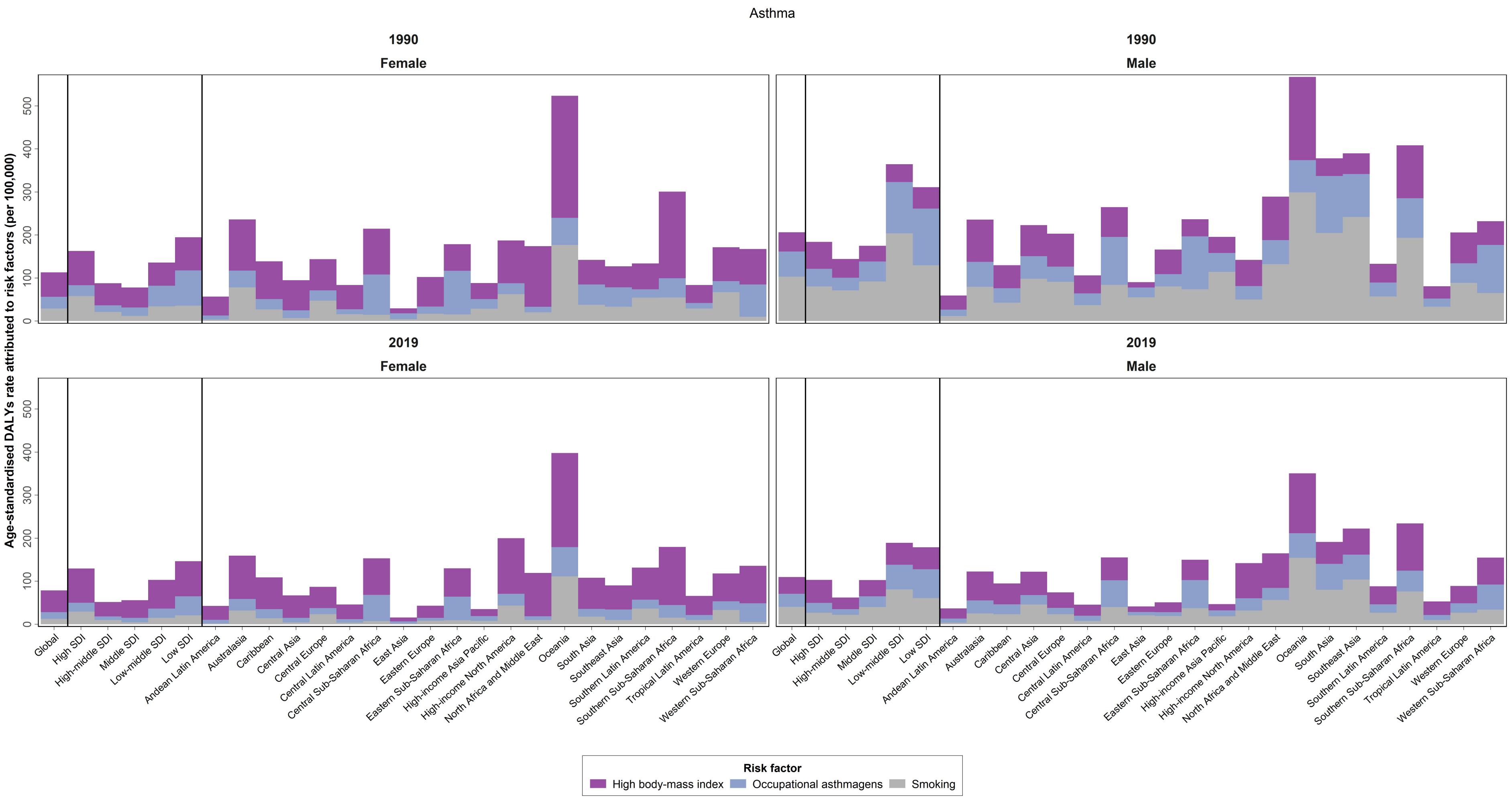

B.3.4 interstitial lung disease (ILD) and pulmonary sarcoidosis

Figure S25. Global age-standardised rates of incidence, prevalence, deaths, and DALYs of ILD and pulmonary sarcoidosis by sex, 1990-2019

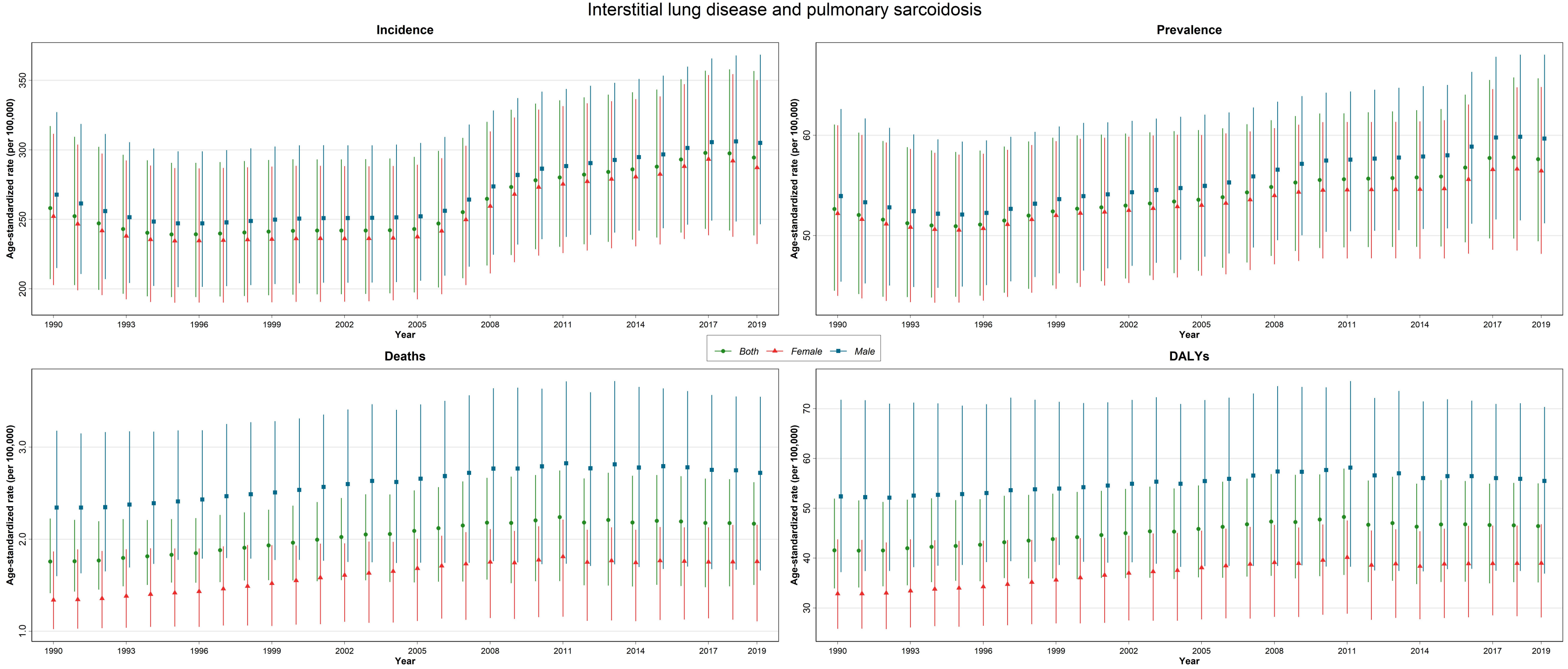

Figure S26. Global age-standardised rates of incidence, prevalence, deaths, and DALYs of ILD and pulmonary sarcoidosis by SDI, both sexes, 1990-2019

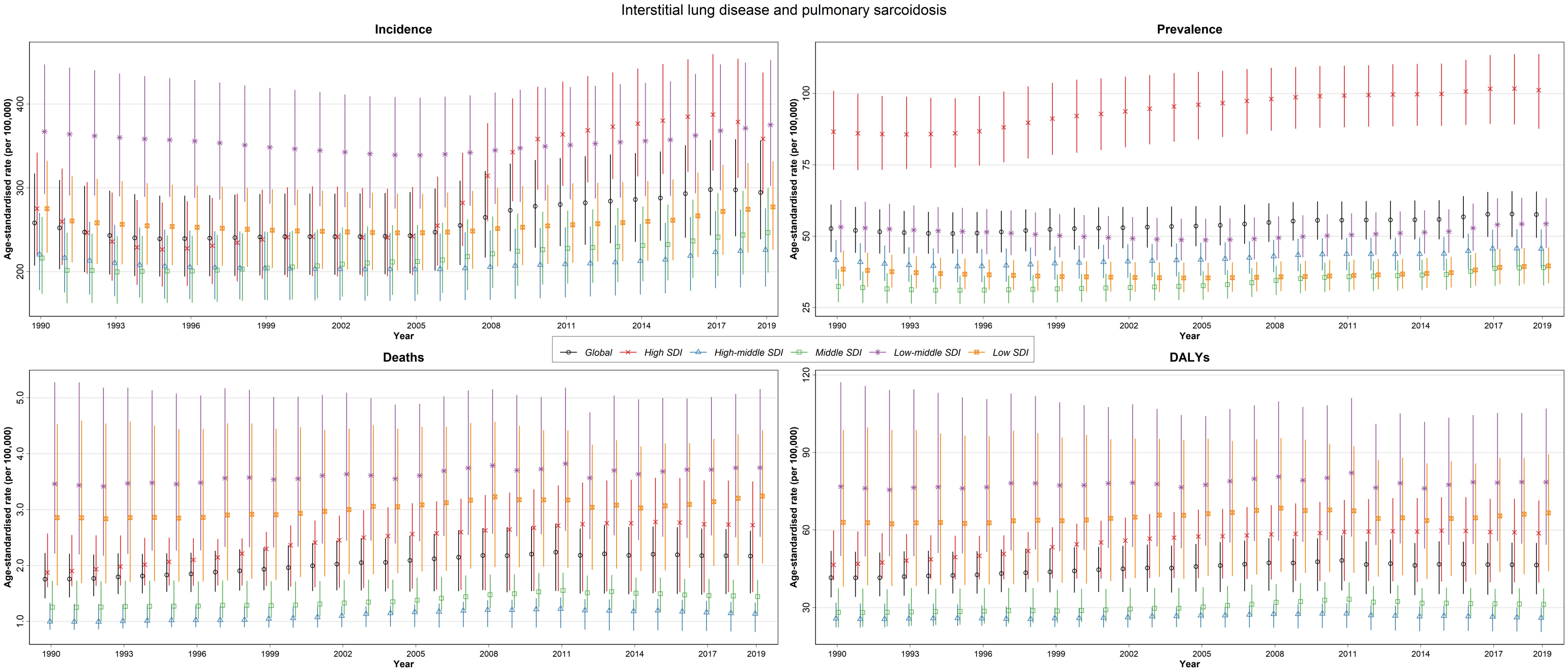

B.3.2 Pneumoconiosis (Coal Worker's Pneumoconiosis, Asbestosis, Silicosis, and Other Pneumoconiosis)

Figure S27. Global age-standardised rates of incidence, prevalence, deaths, and DALYs of pneumoconiosis by sex, 1990-2019

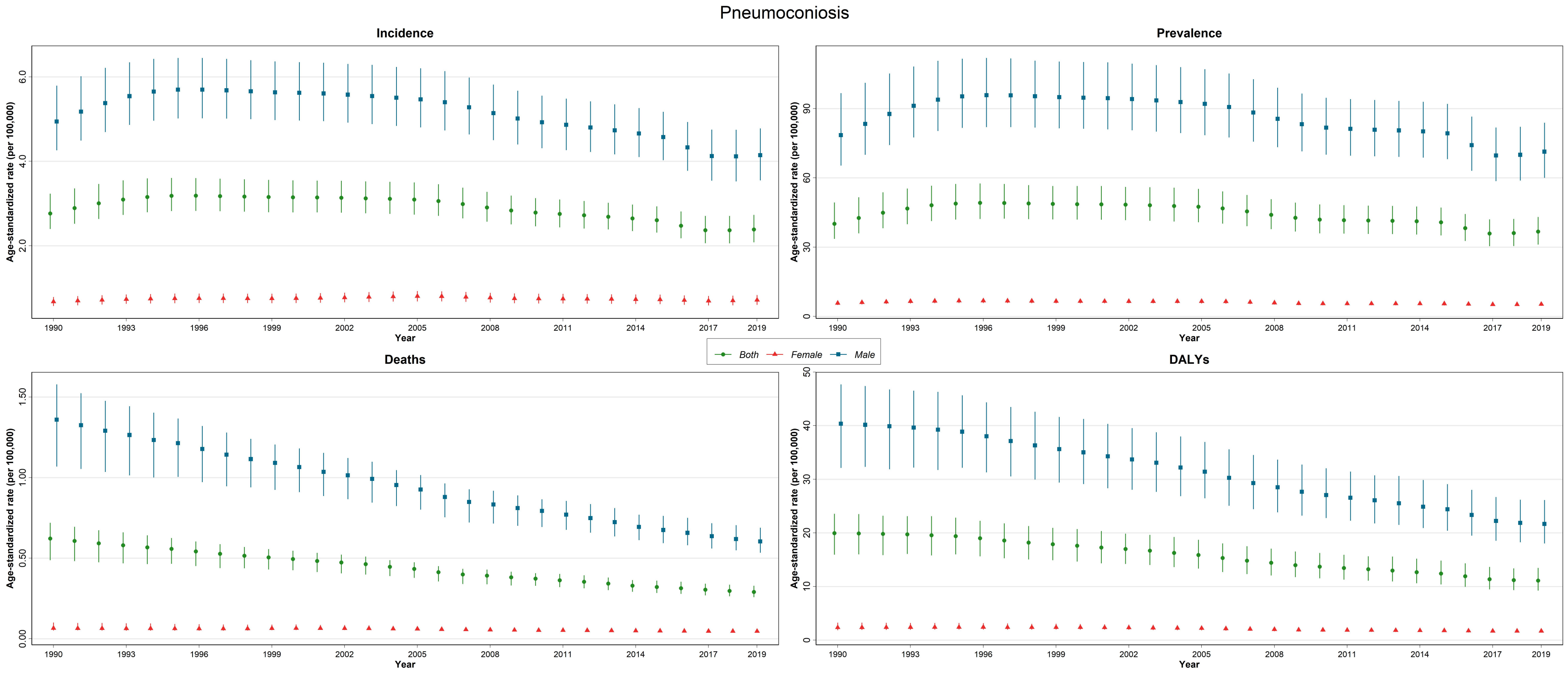

Figure S28. Global age-standardised rates of incidence, prevalence, deaths, and DALYs of pneumoconiosis by SDI, both sexes, 1990-2019

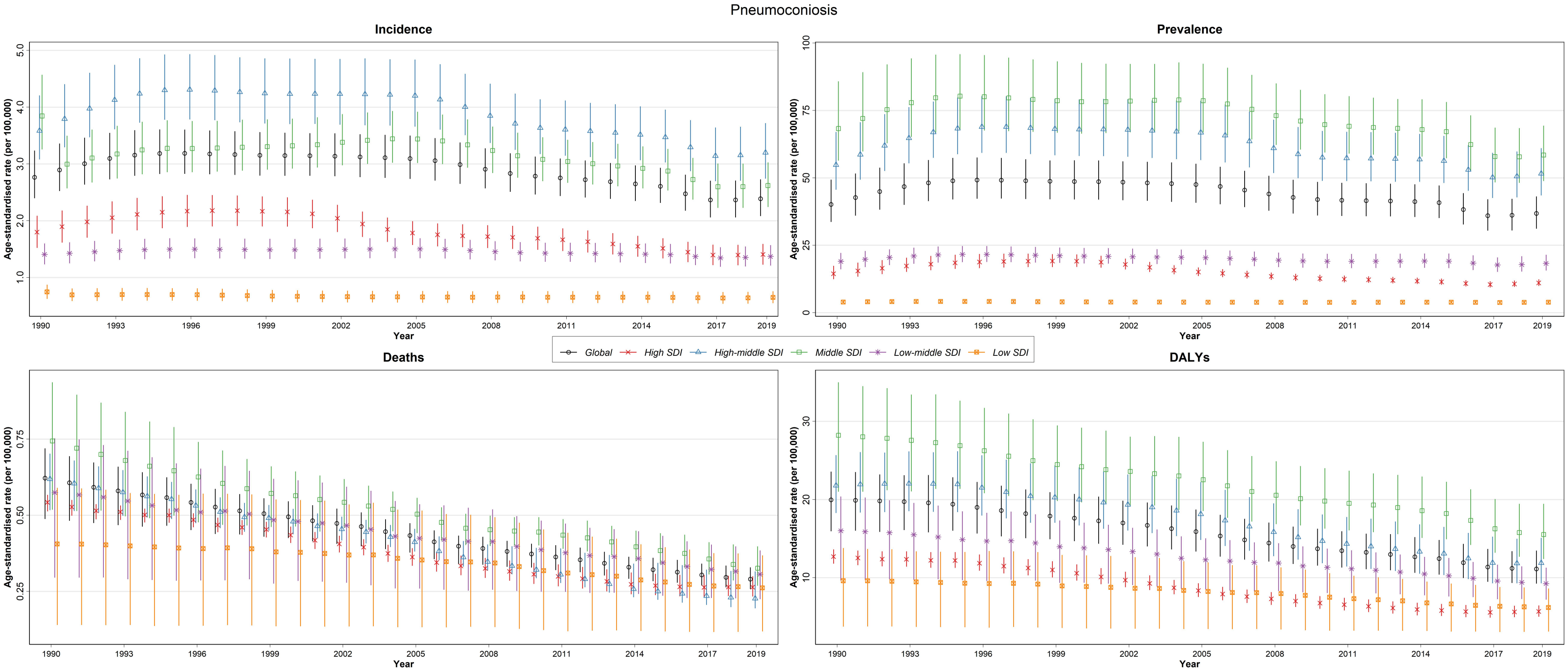

Figure S29. Ranked contribution of risk factors to the age-standardised DALYs from pneumoconiosis by region, 2019, for both sexes, females, and males

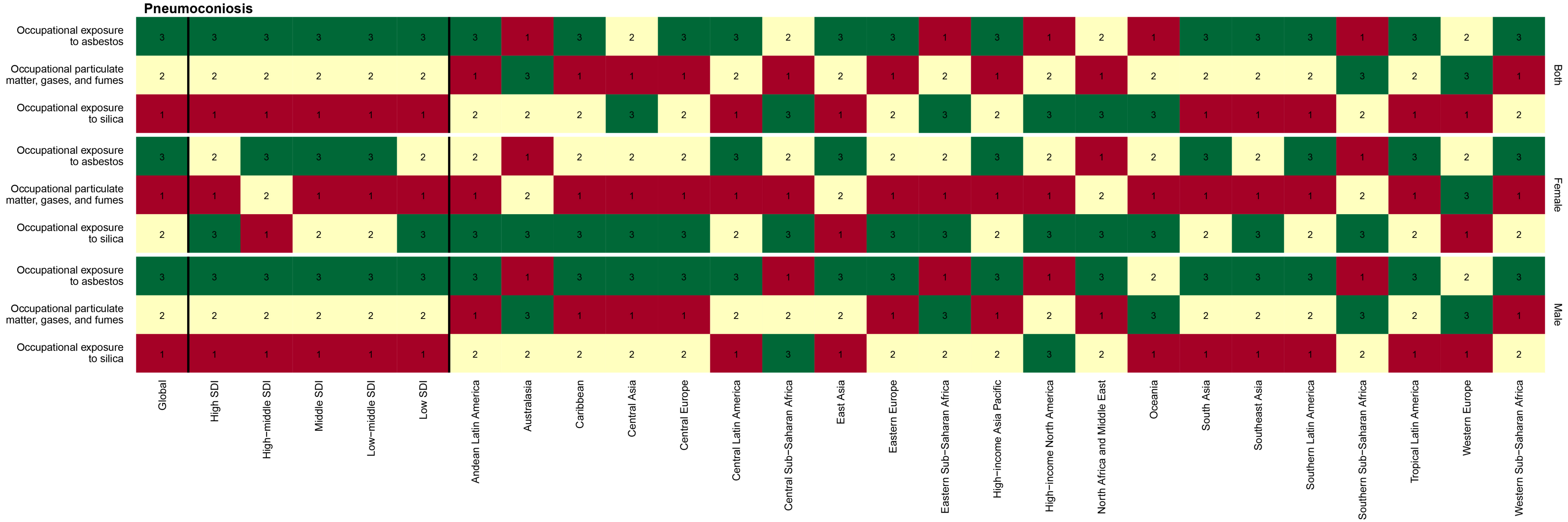

Figure S30. Age-standardised DALYs from pneumoconiosis attributed to risk factors by sex and region in 1990 and 2019

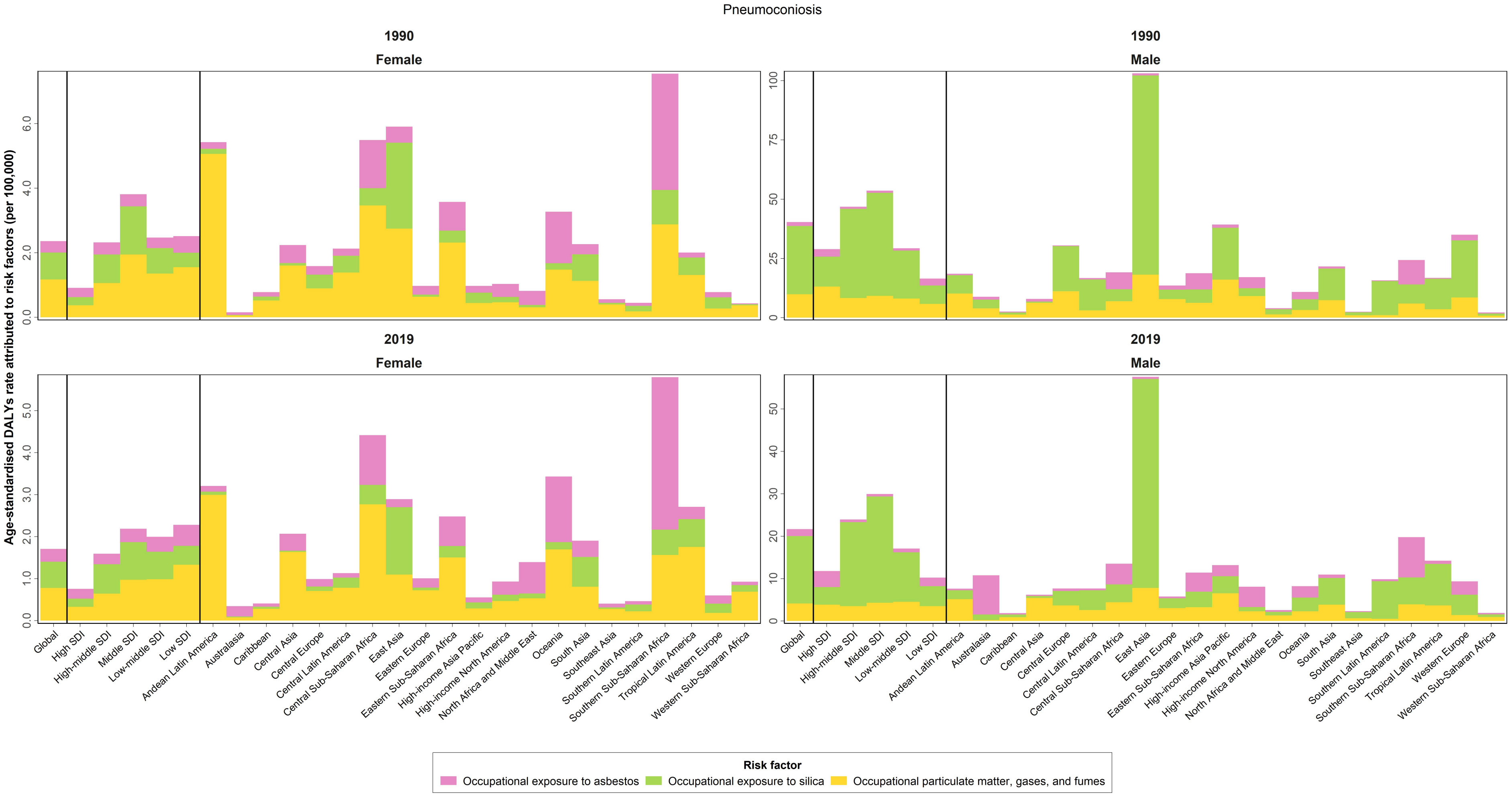

Figure S31. Ranked contribution of the specific sub-causes to the age-standardised DALYs from pneumoconiosis by region, 2019, for both sexes, females, and males

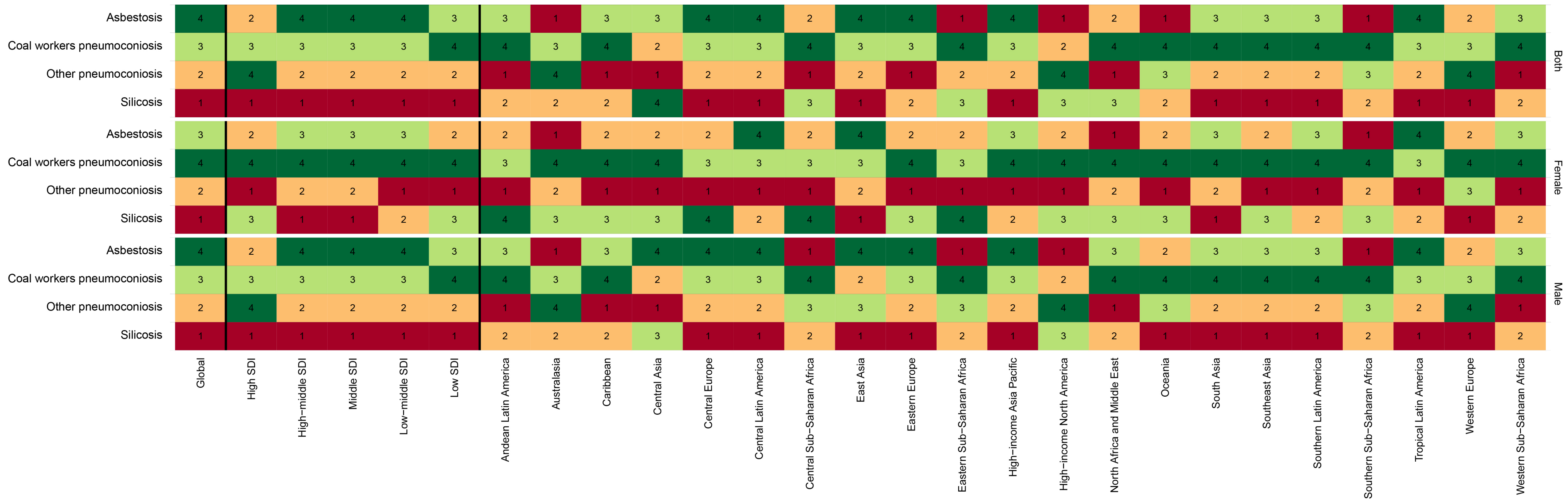

Tables

Table S1. Global incidence, prevalence, deaths, DALYs, YLLs, and YLDs from chronic respiratory diseases by sex

Measure	Age (metric)	Year	Sex	CRD	Cause specific				
					COPD	Pneumoconiosis	Asthma	ILD & pulmonary sarcoidosis	Other
Incidence	All ages (number)	% Change*	Female	50.4 (43.6 to 57)	91 (87.7 to 94)	97.1 (89.3 to 106.3)	15.5 (12.2 to 18.7)	118.6 (111.2 to 125.7)	
			Male	47.7 (40.7 to 54.5)	81.3 (77.3 to 85.3)	56.5 (37.7 to 74.3)	14.4 (11.3 to 17.7)	118.6 (108.9 to 128.2)	
		2019	Female	38,614,376 (34,362,796 to 43,392,020)	7,932,302 (7,436,213 to 8,430,721)	30,274 (25,464 to 35,152)	18,352,088 (14,956,288 to 22,378,285)	12,299,713 (9,911,071 to 15,038,089)	
			Male	39,010,924 (34,421,975 to 44,606,496)	8,282,527 (7,778,147 to 8,804,794)	168,851 (143,671 to 196,290)	18,627,180 (14,694,772 to 23,649,765)	11,932,367 (9,628,216 to 14,464,319)	
	Age-standardized (rate per 100,000)	% Change	Female	-5.2 (-7 to -3.6)	-4.3 (-5.7 to -2.9)	5.4 (1.1 to 10.2)	-13.9 (-16.9 to -11.3)	13.9 (11 to 17)	
			Male	-5.7 (-7.7 to -3.9)	-11 (-12.6 to -9.4)	-16.1 (-24.4 to -8.2)	-12.5 (-16.3 to -9.3)	13.9 (10.5 to 17.3)	
		2019	Female	973.4 (860.6 to 1108.6)	184 (172.7 to 195.3)	0.7 (0.6 to 0.8)	501.6 (402.4 to 621.4)	287.2 (232.3 to 350.1)	
			Male	1034.1 (910 to 1187.4)	219.3 (206.6 to 232)	4.1 (3.5 to 4.8)	505.8 (394.9 to 649.4)	304.9 (246.5 to 368.4)	
Prevalence	All ages (number)	% Change	Female	39.6 (35.9 to 42.9)	88.1 (85.2 to 91.2)	83.4 (74 to 93)	15.4 (12.4 to 18.7)	111.8 (104.6 to 119.3)	
			Male	39.9 (36.6 to 43.5)	81.4 (78 to 85)	83.9 (60.4 to 104.6)	15.9 (12.7 to 19.6)	116.8 (107.9 to 125.9)	
		2019	Female	231,152,957 (212,559,495 to 252,660,333)	107,368,252 (101,311,402 to 114,029,088)	228,490 (197,181 to 262,387)	135,641,710 (116,701,201 to 157,898,921)	2,432,444 (2,074,827 to 2,797,609)	
			Male	223,404,433 (203,679,747 to 248,407,883)	104,967,700 (99,208,838 to 111,045,776)	2,844,060 (2,389,878 to 3,356,589)	126,763,472 (106,760,386 to 152,696,253)	2,277,737 (1,946,302 to 2,613,887)	
	Age-standardized (rate per 100,000)	% Change	Female	-17.4 (-19.1 to -15.8)	-6.4 (-7.9 to -4.9)	-8.4 (-13 to -3.8)	-25.1 (-27.8 to -22.4)	8.1 (4.8 to 11.6)	
			Male	-16.8 (-18.6 to -14.8)	-11.9 (-13.4 to -10.3)	-9.1 (-20.2 to 0.1)	-23.2 (-27 to -19.4)	10.6 (7 to 14.2)	
		2019	Female	5695.9 (5209.3 to 6282.1)	2487.1 (2346.5 to 2640.6)	5.3 (4.6 to 6.1)	3488.5 (2982.6 to 4112.6)	56.5 (48.2 to 64.8)	
			Male	5907.7 (5382 to 6581.2)	2828.1 (2677.6 to 2990.5)	71.4 (60.1 to 83.9)	3332.8 (2804.3 to 4028.8)	59.7 (51.2 to 68)	
Deaths	All ages (number)	% Change	Female	27.9 (9 to 62.1)	27.8 (7.6 to 67.1)	48.5 (1.6 to 84.7)	8.9 (-12.4 to 32.9)	182.5 (96.3 to 266.2)	51.4 (11.2 to 93)
			Male	28.9 (13.1 to 49.9)	32 (13.8 to 54.4)	-6.1 (-22.8 to 27.4)	-8.3 (-23.6 to 13.2)	154.8 (79.2 to 274.5)	52.8 (27 to 89.8)
		2019	Female	1,740,667 (1,456,918 to 1,961,313)	1,399,301 (1,140,438 to 1,602,086)	2,043 (1,699 to 2,388)	247,395 (179,849 to 320,088)	76,869 (48,378 to 94,366)	15,059 (10,342 to 18,924)
			Male	2,233,647 (2,029,782 to 2,452,532)	1,881,335 (1,695,563 to 2,071,025)	20,972 (18,482 to 24,033)	213,674 (174,060 to 260,238)	92,964 (57,581 to 120,738)	24,702 (18,384 to 30,308)
	Age-standardized (rate per 100,000)	% Change	Female	-41.4 (-50.2 to -26.2)	-42.4 (-51.4 to -25.4)	-29.2 (-50.9 to -12.7)	-46.4 (-57.2 to -34.2)	31.2 (-10.6 to 70.9)	-18.3 (-36.5 to 0.8)
			Male	-42.9 (-49.6 to -34.1)	-42.3 (-49.8 to -33.4)	-55.6 (-63.1 to -40.7)	-56.9 (-64.9 to -46.5)	16.1 (-20.8 to 73.3)	-22.1 (-33.9 to -5.4)
		2019	Female	39.7 (33.2 to 44.7)	31.8 (26 to 36.5)	0 (0 to 0.1)	5.7 (4.2 to 7.4)	1.8 (1.1 to 2.2)	0.4 (0.2 to 0.5)
			Male	66.7 (60.5 to 73.1)	56.8 (51.2 to 62.5)	0.6 (0.5 to 0.7)	5.9 (4.8 to 7.2)	2.7 (1.7 to 3.5)	0.7 (0.5 to 0.8)

Measure	Age (metric)	Year	Sex	CRD	Cause specific				
					COPD	Pneumoconiosis	Asthma	ILD & pulmonary sarcoidosis	Other
DALYs	All ages (number)	% Change	Female	22.3 (10.3 to 43.8)	27.2 (12.2 to 57.8)	41.3 (5.2 to 65.3)	-0.1 (-10 to 11.1)	135.8 (88 to 183)	81.5 (39.9 to 115.7)
			Male	19.6 (8.1 to 35)	24.5 (9.8 to 43.1)	9.2 (-9.1 to 37.5)	-6.7 (-16.7 to 4.8)	113.4 (61.7 to 188.1)	72.7 (45.8 to 103.4)
		2019	Female	46,526,316 (41,119,608 to 51,370,913)	32,376,326 (28,369,785 to 35,792,806)	73,727 (61,638 to 90,232)	11,116,325 (8,674,558 to 14,075,192)	1,685,476 (1,210,211 to 2,023,720)	1,274,462 (1,074,509 to 1,475,285)
			Male	57,006,791 (51,895,441 to 62,641,862)	42,056,041 (38,371,483 to 46,150,422)	845,350 (699,045 to 1,027,815)	10,434,651 (8,318,876 to 13,050,199)	2,085,418 (1,399,512 to 2,652,671)	1,585,331 (1,316,177 to 1,838,523)
	Age-standardized (rate per 100,000)	% Change	Female	-36.7 (-42.7 to -25.2)	-38.1 (-45.4 to -23.4)	-27.5 (-45.8 to -15.8)	-40.3 (-47.6 to -32.5)	18.7 (-6.2 to 42.9)	21.3 (-3.9 to 43.7)
			Male	-40.9 (-46.5 to -33.1)	-41.7 (-48.4 to -33.5)	-46.3 (-55 to -32.7)	-45.4 (-53 to -37.3)	5.9 (-20.2 to 44.7)	7.6 (-7.8 to 26.1)
		2019	Female	1093 (965.7 to 1209)	744.1 (652.2 to 822.2)	1.7 (1.4 to 2.1)	275.2 (214.9 to 349.6)	39 (28.1 to 46.8)	33 (27.7 to 38.3)
			Male	1538.7 (1399.5 to 1690.3)	1149.1 (1050.1 to 1257.3)	21.7 (18 to 26.1)	272.2 (216.9 to 340.9)	55.5 (36.9 to 70.3)	40.2 (33.5 to 46.7)
YLLs	All ages (number)	% Change	Female	7.8 (-7 to 35.9)	9.5 (-7.9 to 45.6)	17.3 (-23.4 to 50.3)	-10.7 (-24.4 to 7.7)	141.3 (84.6 to 199.8)	25 (-15.4 to 67.6)
			Male	9.5 (-4 to 28.3)	13.6 (-2.6 to 34.6)	-20.4 (-36.4 to 11.6)	-20.7 (-31.7 to -4.2)	113.5 (55.4 to 202.2)	36.4 (9.3 to 72.5)
		2019	Female	29,719,823 (25,218,341 to 33,436,073)	21,829,500 (17,883,748 to 25,012,243)	39,419 (33,641 to 46,551)	5,884,271 (4,436,722 to 7,428,704)	1,439,099 (975,532 to 1,768,150)	527,534 (358,505 to 682,703)
			Male	41,425,922 (37,667,187 to 45,724,348)	32,765,398 (29,363,526 to 36,286,001)	439,920 (380,203 to 510,167)	5,470,441 (4,542,805 to 6,512,589)	1,851,957 (1,196,509 to 2,398,341)	898,205 (667,119 to 1,118,633)
	Age-standardized (rate per 100,000)	% Change	Female	-46.2 (-53.7 to -32.2)	-47.4 (-55.7 to -30.3)	-38.9 (-59.9 to -22.4)	-50.3 (-58.4 to -40)	21 (-8.2 to 50.7)	-18.8 (-41.7 to 7.2)
			Male	-47.3 (-53.8 to -38.6)	-47.1 (-54.5 to -37.8)	-60.4 (-67.9 to -45.3)	-57.1 (-63.7 to -47.3)	5.5 (-23.3 to 50.8)	-17.7 (-32.1 to 2.4)
		2019	Female	686.7 (582.2 to 772.6)	499 (409.1 to 571.9)	0.9 (0.8 to 1.1)	140.1 (106 to 176.7)	33.3 (22.5 to 40.8)	13.5 (9.1 to 17.7)
			Male	1127.5 (1027.5 to 1242.9)	902.2 (808.8 to 995.9)	11.5 (10 to 13.4)	141.7 (118.2 to 168.4)	49.4 (31.5 to 63.9)	22.7 (17 to 28.2)
YLDs	All ages (number)	% Change	Female	60.3 (52.2 to 68.8)	91 (86.7 to 95.3)	84.5 (73.8 to 95.6)	15.1 (12.2 to 18.3)	108 (100.8 to 116.3)	166.7 (157.5 to 174.9)
			Male	58.5 (51.1 to 66.3)	87.6 (83.4 to 91.9)	82.8 (59.7 to 103.5)	15.8 (12.7 to 19.4)	113 (104.2 to 122.9)	164.8 (156.1 to 172.7)
		2019	Female	16,806,492 (13,564,765 to 19,824,392)	10,546,826 (8,827,741 to 12,059,136)	34,308 (22,825 to 48,815)	5,232,055 (3,425,942 to 7,720,695)	246,376 (165,827 to 353,999)	746,927 (607,798 to 866,677)
			Male	15,580,870 (12,436,298 to 18,947,340)	9,290,643 (7,672,101 to 10,654,092)	405,429 (267,079 to 578,276)	4,964,210 (3,218,615 to 7,311,370)	233,462 (155,401 to 338,218)	687,126 (561,398 to 799,143)
	Age-standardized (rate per 100,000)	% Change	Female	-9.5 (-12.1 to -6.8)	-3.2 (-5.2 to -1.1)	-7.5 (-12.9 to -2.1)	-24.6 (-27.4 to -21.8)	6.8 (3.4 to 10.6)	83.7 (76.4 to 89.7)
			Male	-11 (-13.1 to -8.9)	-7.5 (-9.4 to -5.7)	-9.3 (-20 to 0.5)	-22.5 (-26.3 to -18.7)	9.3 (5.7 to 13.1)	78.6 (71.8 to 84.8)
		2019	Female	406.3 (326.2 to 484.4)	245.2 (205.4 to 280.3)	0.8 (0.5 to 1.1)	135.1 (88.4 to 199.6)	5.7 (3.9 to 8.2)	19.5 (15.8 to 22.7)
			Male	411.2 (328.6 to 499.3)	246.9 (204 to 282)	10.1 (6.7 to 14.5)	130.5 (84.6 to 192.8)	6.1 (4.1 to 8.8)	17.5 (14.3 to 20.4)

* % Change (1990 to 2019). Data in parentheses are 95% Uncertainty Intervals (95% UIs)
CRD= Chronic Respiratory Disease, COPD= Chronic Obstructive Pulmonary Disease; ILD= Interstitial Lung Disease; DALYs= Disability-Adjusted Life Years; YLLs= Years of Life Lost; YLDs= Years Lived with Disability

Table S2. Rate of incidence, prevalence, deaths, DALYs, YLLs, and YLDs from chronic respiratory diseases by SDI, by sex and for both sexes combined

SDI	Measure	Year	Sex	CRD	Cause specific				
					COPD	Pneumoconiosis	Asthma	ILD & pulmonary sarcoidosis	Other
High SDI	Incidence	% Change*	Both	3.4 (0.2 to 6.9)	0.9 (-0.6 to 2.7)	-21.8 (-28.3 to -15.4)	-3.9 (-8 to 0.6)	30.3 (23.5 to 36.9)	
			Female	5.9 (2.4 to 9.9)	11.9 (9 to 15.3)	-34 (-43.6 to -22.5)	-1.7 (-6 to 3.2)	30 (22.7 to 36.6)	
			Male	0.4 (-3.4 to 4.5)	-10.2 (-11.5 to -8.8)	-23.4 (-30.1 to -17.1)	-6 (-11.8 to 0.1)	29.4 (23.1 to 36.4)	
		2019	Both	1460.9 (1263.9 to 1686.9)	203.7 (194.5 to 212.2)	1.4 (1.2 to 1.6)	897.4 (711.3 to 1111.7)	358.5 (293.1 to 437.6)	
			Female	1474.6 (1288.7 to 1693.5)	184.7 (176.4 to 192.7)	0.4 (0.3 to 0.4)	945.5 (760.8 to 1157.5)	344.1 (280.7 to 418.2)	
			Male	1457.2 (1254.8 to 1692.8)	226 (215.8 to 235.3)	2.6 (2.3 to 2.9)	849.8 (660 to 1079.9)	378.7 (306.2 to 460.1)	
	Prevalence	% Change	Both	-12.8 (-15 to -10.4)	-4.4 (-5.8 to -2.8)	-23.6 (-34.2 to -15.7)	-17.7 (-20.9 to -14.2)	16.8 (11.1 to 22.3)	
			Female	-9.3 (-11.4 to -7)	3 (0.7 to 5.7)	-20.8 (-28.3 to -13)	-14.1 (-17.1 to -11)	15 (9.6 to 21)	
			Male	-17 (-19.7 to -14.1)	-13.7 (-15 to -12.4)	-30 (-39.8 to -22.2)	-21.7 (-26 to -17.2)	17.3 (11.5 to 22.7)	
		2019	Both	9110.3 (8211.2 to 10240.4)	2792 (2672.1 to 2914.8)	11 (9.9 to 12.3)	6855.1 (5876.7 to 8058.5)	101.2 (87.7 to 113.8)	
			Female	9591.8 (8617.9 to 10704.2)	2612.4 (2499 to 2730.3)	1.8 (1.5 to 2)	7566.6 (6559 to 8769.9)	96.9 (83.9 to 109.5)	
			Male	8683.7 (7822.2 to 9818.8)	3031.5 (2900.7 to 3160.1)	21.9 (19.6 to 24.6)	6143.6 (5194.8 to 7371)	107.9 (93 to 121.9)	
	Deaths	% Change	Both	-18.7 (-28 to -14.8)	-15.1 (-22.2 to -8.1)	-51.3 (-56.3 to -44.8)	-73 (-75.4 to -69.9)	45.1 (-26.5 to 99.2)	1.5 (-20.8 to 10.7)
			Female	-3.8 (-26.7 to 3.9)	5.5 (-21 to 16.8)	-5.2 (-19.4 to 10.6)	-68.2 (-71.5 to -64.2)	36.7 (-26.4 to 85.4)	-7 (-38.4 to 6.4)
			Male	-32.6 (-35.5 to -30.1)	-32.2 (-36.8 to -26.2)	-55.6 (-60.1 to -49.3)	-78.7 (-80.8 to -75.9)	44.8 (-29.3 to 104.1)	6.9 (-7.6 to 17.6)
		2019	Both	24.6 (21.5 to 26.1)	20.4 (18 to 22.2)	0.3 (0.2 to 0.3)	1 (0.9 to 1.1)	2.7 (1.5 to 3.5)	0.3 (0.2 to 0.4)
			Female	19.3 (14.5 to 20.9)	16.1 (12.2 to 17.8)	0 (0 to 0)	1 (0.8 to 1.1)	2 (1 to 2.5)	0.2 (0.1 to 0.3)
			Male	32.3 (29.7 to 34)	26.7 (23.9 to 29.3)	0.6 (0.5 to 0.7)	0.9 (0.8 to 1)	3.8 (1.8 to 5)	0.4 (0.3 to 0.5)
	DALYs	% Change	Both	-16.7 (-20.7 to -14.6)	-12.7 (-16.6 to -8.9)	-55.3 (-59.4 to -50)	-28.2 (-33.6 to -24.1)	26.3 (-18.1 to 57.1)	25.9 (14.6 to 32.7)
			Female	-7.4 (-15.5 to -4.2)	3.4 (-10.9 to 9.1)	-17.3 (-25 to -7.4)	-23.2 (-27.9 to -19.4)	21.2 (-12.7 to 46.3)	28 (7.2 to 38)
			Male	-27 (-28.9 to -25.1)	-27.4 (-30.6 to -23.4)	-59.3 (-63.1 to -54.3)	-34.1 (-40.1 to -29)	26.7 (-25.7 to 63.3)	23.2 (15.9 to 30.4)
		2019	Both	924.1 (797.2 to 1067)	543.5 (499.2 to 582.9)	5.7 (5 to 6.4)	293.7 (200.7 to 419.4)	58.8 (39.6 to 71.5)	22.4 (19.4 to 25)
			Female	854.2 (713.5 to 1009.5)	467.9 (396.9 to 507)	0.8 (0.6 to 0.9)	318.8 (216.9 to 457.6)	46.3 (31.1 to 55.3)	20.4 (16.9 to 23.5)
			Male	1021.8 (901.4 to 1163.9)	642.8 (591.9 to 696.6)	11.8 (10.4 to 13.3)	268.4 (183.1 to 384.5)	74.4 (43.9 to 92.1)	24.5 (21.4 to 27.4)
	YLLs	% Change	Both	-23.8 (-30.7 to -20.6)	-18.3 (-23.8 to -12.6)	-61.6 (-65.5 to -55.6)	-70.1 (-72.5 to -66.8)	29.1 (-24.5 to 67.1)	-4.4 (-18.1 to 3.1)
			Female	-9.6 (-27.8 to -3.4)	3.2 (-20.5 to 12.7)	-15.2 (-26.5 to -0.3)	-66.3 (-69.7 to -61.7)	23.7 (-18.8 to 55.3)	-11 (-34.7 to 0.4)
			Male	-35.8 (-38 to -33.5)	-34.2 (-38.4 to -28.8)	-64.8 (-68.3 to -59)	-74.2 (-76.6 to -70.9)	28.9 (-31.1 to 72.7)	-0.4 (-8.2 to 8.6)
		2019	Both	415.1 (371.8 to 433.9)	326.8 (295.3 to 352.1)	4.1 (3.7 to 4.6)	25.8 (23.8 to 28.3)	48.6 (29.9 to 59.1)	9.9 (8 to 11.4)
			Female	333.5 (254.2 to 355.6)	263.5 (200.2 to 286.9)	0.5 (0.4 to 0.6)	25.3 (22.5 to 28.5)	36.6 (21.8 to 43.5)	7.6 (4.7 to 9.9)
			Male	519.3 (489.5 to 540.7)	408.8 (378.2 to 446)	8.6 (7.7 to 9.9)	26 (23.7 to 29.1)	63.5 (33.6 to 79.6)	12.3 (10.3 to 13.8)
	YLDs	% Change	Both	-9.8 (-12.1 to -7)	-2.8 (-5 to -0.4)	-23.1 (-33.9 to -14.7)	-17.1 (-20.3 to -13.6)	14.5 (9.2 to 20.2)	68.2 (62.7 to 74.3)
			Female	-5.9 (-8.3 to -3)	3.8 (0.8 to 7.3)	-20.7 (-28.5 to -12.7)	-13.6 (-16.6 to -10.5)	12.7 (7 to 18.9)	73.5 (66.5 to 81.6)
			Male	-15.1 (-17.6 to -12.3)	-11.5 (-13.4 to -9.2)	-29.2 (-39.5 to -21.3)	-20.8 (-25.3 to -16.3)	15.2 (9.1 to 20.8)	61.9 (55.6 to 68.8)
		2019	Both	509 (384.2 to 647.9)	216.7 (179.1 to 245.1)	1.6 (1.1 to 2.2)	267.9 (175.4 to 393.6)	10.2 (6.8 to 14.6)	12.5 (10 to 14.7)

SDI	Measure	Year	Sex	CRD	Cause specific				
					COPD	Pneumoconiosis	Asthma	ILD & pulmonary sarcoidosis	Other
			Female	520.7 (391.8 to 671)	204.4 (168.4 to 234.6)	0.3 (0.2 to 0.4)	293.5 (190.2 to 431.6)	9.8 (6.6 to 13.8)	12.8 (10.2 to 15.2)
			Male	502.6 (383.5 to 634.6)	234 (193 to 269.2)	3.1 (2.2 to 4.4)	242.4 (157.9 to 357.8)	10.9 (7.3 to 15.5)	12.2 (9.7 to 14.6)
High-middle SDI	Incidence	% Change	Both	-11.8 (-14.8 to -9.1)	-16 (-18.3 to -13.6)	-10.7 (-20.7 to -2.3)	-15.9 (-21.5 to -11.3)	2.5 (-0.4 to 5.8)	
			Female	-11.3 (-14.4 to -8.6)	-14.1 (-16.6 to -11.5)	-2.4 (-6.7 to 2.2)	-15.4 (-21 to -10.7)	1.9 (-0.8 to 4.8)	
			Male	-12.7 (-15.5 to -9.8)	-18.8 (-21.4 to -16.3)	-12.6 (-23 to -3.6)	-16.5 (-22.1 to -11.6)	3.2 (-0.7 to 7.4)	
		2019	Both	880.4 (759.8 to 1029)	180.6 (168.8 to 193.3)	3.2 (2.7 to 3.7)	470.7 (356.3 to 610)	225.9 (182.2 to 275.5)	
			Female	849.2 (733.1 to 991)	157 (145.9 to 168.8)	0.7 (0.6 to 0.8)	475.1 (364.6 to 607.9)	216.4 (173.9 to 267)	
			Male	919.7 (792.3 to 1077.4)	208 (195.1 to 222.4)	5.8 (5 to 6.8)	465.6 (348.6 to 616.3)	240.3 (194.9 to 291)	
	Prevalence	% Change	Both	-21.5 (-24.3 to -18.7)	-17.1 (-19.5 to -14.6)	-5.9 (-17.8 to 4.7)	-26.8 (-32.1 to -22.2)	9.5 (5.5 to 13.7)	
			Female	-21.3 (-24.3 to -18.6)	-15.6 (-18.2 to -13)	-1.4 (-7.3 to 4.9)	-26.2 (-31.2 to -21.9)	10 (5.8 to 14.4)	
			Male	-22.2 (-24.9 to -19.4)	-19.7 (-22.1 to -17.2)	-9.5 (-21.3 to 1.2)	-27.9 (-33.7 to -22.6)	8.7 (4.4 to 13)	
		2019	Both	5296.9 (4736.2 to 6016.1)	2370.4 (2218 to 2534.4)	51.6 (43.5 to 61)	3103 (2541.3 to 3833.3)	45.6 (38.9 to 52.4)	
			Female	5141.3 (4587 to 5840)	2105.1 (1960.7 to 2258)	5.8 (4.9 to 6.8)	3230.1 (2670.7 to 3923.3)	44.9 (38 to 52)	
			Male	5520 (4940.7 to 6240.6)	2722 (2561.4 to 2909)	103.8 (87.4 to 123.2)	2965.3 (2403.3 to 3714.1)	47.1 (40.3 to 54)	
	Deaths	% Change	Both	-58.9 (-64.1 to -41.5)	-59.2 (-64.7 to -40.3)	-63.3 (-70.4 to -45.8)	-66.8 (-73 to -59.8)	13.9 (-20.8 to 49.3)	-61.1 (-67.1 to -28.1)
			Female	-61.6 (-69 to -34.8)	-62.8 (-70.3 to -33.4)	-41.2 (-63.7 to -22.1)	-63.3 (-72.1 to -53.3)	19.9 (-21 to 57.4)	-57 (-65.7 to -20.4)
			Male	-57.4 (-62.7 to -45.8)	-57.1 (-62.5 to -44.6)	-65.7 (-72.7 to -48.4)	-71 (-76.4 to -65)	6.6 (-32.2 to 49.4)	-66.3 (-72.5 to -20.5)
		2019	Both	33.2 (29.5 to 39.3)	29.8 (26.2 to 35.8)	0.2 (0.2 to 0.3)	1.8 (1.6 to 2.1)	1.1 (0.8 to 1.3)	0.3 (0.2 to 0.3)
			Female	23 (19.2 to 30.1)	20.1 (16.7 to 27.3)	0 (0 to 0)	1.7 (1.3 to 2)	1 (0.6 to 1.1)	0.2 (0.1 to 0.3)
			Male	49.2 (43.7 to 55.6)	44.9 (39.6 to 51.2)	0.5 (0.4 to 0.7)	2 (1.8 to 2.4)	1.4 (0.9 to 1.7)	0.4 (0.3 to 0.5)
	DALYs	% Change	Both	-52.6 (-57.3 to -39.9)	-56 (-60.8 to -40.7)	-45.5 (-55 to -32.6)	-44.8 (-51.8 to -38.4)	1.1 (-19.7 to 20.3)	-16.3 (-26.1 to 0.2)
			Female	-50.8 (-57.2 to -31.2)	-55.5 (-62.4 to -31.2)	-31.3 (-50.1 to -18.2)	-41.6 (-49.6 to -34.4)	6 (-20.3 to 25.2)	-2.2 (-15.3 to 21.2)
			Male	-54.8 (-59.5 to -44.9)	-57.1 (-62.1 to -46.3)	-48.8 (-57.9 to -35.9)	-49 (-56.2 to -41.6)	-4.1 (-31.8 to 21.9)	-29.1 (-39 to -2.1)
		2019	Both	837 (750.9 to 948.8)	617.7 (562.8 to 708.9)	11.9 (9.3 to 15.1)	158.3 (114 to 220.2)	26 (20.5 to 30)	23.1 (20 to 26.2)
			Female	670.8 (584 to 792)	464.6 (408.4 to 571.8)	1.6 (1.3 to 2)	160.1 (113.8 to 224.2)	22.5 (17.2 to 26.4)	21.9 (18.7 to 25.3)
			Male	1066.8 (964.9 to 1195.8)	830.7 (753.2 to 939.3)	23.9 (18.6 to 30.5)	157 (113.6 to 217.8)	30.5 (22.4 to 36.1)	24.6 (21.2 to 28.7)
	YLLs	% Change	Both	-63.3 (-67.9 to -48)	-63.9 (-68.7 to -46.8)	-67.7 (-74.8 to -50.6)	-70.2 (-75.1 to -63.3)	-0.4 (-24.8 to 22.7)	-52.2 (-59.2 to -36.2)
			Female	-65.3 (-71.9 to -40.4)	-66.7 (-73.6 to -39.1)	-49.7 (-70.4 to -31.1)	-67.8 (-74.4 to -57.1)	5.2 (-25.8 to 29.5)	-50.2 (-60.4 to -25.9)
			Male	-62.7 (-67.6 to -51.2)	-62.7 (-67.8 to -50.5)	-69.8 (-76.9 to -53)	-73 (-77.5 to -67.5)	-6 (-37.1 to 24.7)	-55.7 (-63.4 to -29.7)
		2019	Both	501.1 (452.6 to 598.9)	429.7 (383.9 to 523.6)	4.5 (3.8 to 6.5)	36.5 (32.3 to 41.3)	21.3 (16.3 to 24.1)	9.1 (8.1 to 11.5)
			Female	336.1 (287.9 to 450.6)	276.9 (233.6 to 380.9)	0.7 (0.5 to 0.9)	33.9 (28.4 to 40.2)	17.9 (12.7 to 21.1)	6.6 (5.3 to 9.2)
			Male	727.2 (653.7 to 842.9)	640.7 (573.5 to 746.6)	9.2 (7.6 to 13.6)	39.9 (34.6 to 47.8)	25.6 (17.9 to 30.5)	11.8 (10.2 to 15.2)
	YLDs	% Change	Both	-16.1 (-18.8 to -13.2)	-12.7 (-15.9 to -9.3)	-5.9 (-18.1 to 5.5)	-25.8 (-31.1 to -21.3)	8.4 (3.6 to 13.4)	63.5 (54.5 to 71.7)

SDI	Measure	Year	Sex	CRD	Cause specific				
					COPD	Pneumoconiosis	Asthma	ILD & pulmonary sarcoidosis	Other
			Female	-15.5 (-18.6 to -12.2)	-11.9 (-15.6 to -7.9)	-0.4 (-8 to 7.6)	-25.3 (-30.5 to -21)	8.9 (3.2 to 15)	67.5 (56.8 to 77.4)
			Male	-17.2 (-20 to -14.4)	-14.3 (-17.4 to -10.8)	-9.4 (-21.6 to 2.1)	-26.7 (-32.4 to -21.6)	7.5 (2.1 to 13.4)	59.3 (50.3 to 68.3)
		2019	Both	335.9 (265.7 to 410.6)	188 (154.3 to 215.5)	7.4 (4.9 to 10.5)	121.8 (77.6 to 185.5)	4.7 (3.1 to 6.8)	14 (11.2 to 16.6)
			Female	334.7 (264.7 to 409)	187.8 (152.6 to 219.1)	0.9 (0.6 to 1.2)	126.1 (80.7 to 190.4)	4.6 (3.1 to 6.7)	15.3 (12.3 to 18.5)
			Male	339.5 (266.4 to 420.3)	190 (153.2 to 221.6)	14.7 (9.8 to 21.1)	117.1 (74 to 177.4)	4.9 (3.3 to 7.1)	12.8 (10.1 to 15.4)
Middle SDI	Incidence	% Change	Both	-6.2 (-8 to -4.4)	-16.7 (-19 to -14.2)	-31.9 (-39.1 to -25.4)	-9.9 (-12.9 to -7)	14 (11.4 to 16.8)	
			Female	-4.4 (-6.4 to -2.3)	-14.1 (-16.8 to -11.2)	2.1 (-5.3 to 10.3)	-9.7 (-13.2 to -6.4)	17.9 (15.2 to 21.2)	
			Male	-8.1 (-9.7 to -6.4)	-19.4 (-21.5 to -17.2)	-35 (-42.9 to -27.8)	-10.1 (-13 to -7.3)	9.8 (6.9 to 12.8)	
		2019	Both	908.2 (789.5 to 1050.9)	187.3 (173.6 to 201.4)	2.6 (2.2 to 3)	471.8 (366.2 to 607.7)	246.6 (198.9 to 299.6)	
			Female	891.3 (777.3 to 1027.7)	183.5 (169.6 to 198.1)	0.9 (0.8 to 1.1)	454.8 (353.8 to 580.7)	252.1 (202.2 to 308.3)	
			Male	924.9 (802.9 to 1078.4)	191 (177.6 to 204.6)	4.3 (3.6 to 5.1)	487.7 (376.5 to 636.2)	241.8 (195.3 to 290.9)	
	Prevalence	% Change	Both	-9.6 (-11.4 to -7.6)	-9.4 (-12 to -6.8)	-14.4 (-24.7 to -5.1)	-10.5 (-13.5 to -7.3)	20.5 (16.6 to 24.5)	
			Female	-12.6 (-14.7 to -10.5)	-12 (-15 to -8.9)	-24.6 (-29.5 to -19.8)	-14.3 (-17.5 to -11.1)	19.5 (15.4 to 23.7)	
			Male	-6.6 (-8.4 to -4.6)	-6.5 (-8.9 to -4.1)	-11.2 (-22.7 to -0.8)	-7 (-10.2 to -3.7)	21.5 (17.4 to 25.5)	
		2019	Both	5264.2 (4747.4 to 5910.8)	2512.8 (2336.8 to 2705.6)	58.5 (48.8 to 69.3)	2927.1 (2429.3 to 3578.8)	39 (32.8 to 45.4)	
			Female	4964.6 (4486 to 5564.7)	2397.1 (2218.7 to 2587.9)	8.3 (7.2 to 9.6)	2757.7 (2277.9 to 3358.8)	38.9 (32.6 to 45.5)	
			Male	5574 (5029.6 to 6275.9)	2642.4 (2465.6 to 2840.7)	112.4 (93.1 to 134.2)	3093.8 (2570.3 to 3815.4)	39.4 (33.2 to 45.9)	
	Deaths	% Change	Both	-55.4 (-61.5 to -42.4)	-55.9 (-61.9 to -42.3)	-56.2 (-68.9 to -30.6)	-59.8 (-69.3 to -49)	15.1 (-11 to 48.1)	-3.5 (-32.9 to 29.5)
			Female	-60.9 (-69.2 to -40.7)	-62 (-70 to -41.1)	-48.4 (-70.3 to -27.7)	-60.5 (-72.7 to -45.2)	20.7 (-21.1 to 62.7)	-8.6 (-45.6 to 31.9)
			Male	-50 (-57.3 to -39.3)	-49.8 (-57.3 to -38.3)	-56.2 (-69.2 to -27.9)	-59.2 (-69.1 to -47.9)	10 (-16.9 to 72.8)	0.8 (-25.7 to 46)
		2019	Both	59.8 (52.3 to 66.6)	52.3 (46.1 to 58.7)	0.3 (0.3 to 0.4)	5.3 (4.6 to 6)	1.4 (1.1 to 1.7)	0.4 (0.3 to 0.5)
			Female	45.9 (37 to 55.3)	39.5 (32 to 48.2)	0.1 (0 to 0.1)	4.8 (3.6 to 5.6)	1.3 (0.9 to 1.7)	0.3 (0.2 to 0.4)
			Male	77.2 (68.6 to 87)	68.3 (60.2 to 77.1)	0.6 (0.5 to 0.8)	6 (5.2 to 7.2)	1.7 (1.3 to 2.1)	0.6 (0.4 to 0.7)
	DALYs	% Change	Both	-51.4 (-56.7 to -40.2)	-54.4 (-59.7 to -41.7)	-45 (-57.1 to -26.2)	-45.1 (-54 to -36.8)	10.8 (-8.2 to 36.7)	14.5 (-8.3 to 35.2)
			Female	-55.1 (-61.8 to -38.2)	-58.6 (-65.5 to -39.9)	-42.6 (-62.5 to -29.3)	-46.8 (-57.1 to -36.3)	15.5 (-16.2 to 46.7)	15.7 (-13.7 to 38.2)
			Male	-48 (-54.7 to -38.1)	-50.3 (-57.4 to -39.4)	-44.1 (-56.9 to -23.1)	-43.8 (-52.9 to -34)	7 (-17 to 54.8)	14.2 (-6.9 to 40.2)
		2019	Both	1316.9 (1200 to 1449.4)	1007.2 (915.7 to 1112.4)	15.5 (12.4 to 19.4)	230.6 (183.8 to 290.3)	31.2 (26.1 to 37.3)	32.4 (28.4 to 36.4)
			Female	1070.5 (937.1 to 1210.9)	798.6 (691.5 to 925.4)	2.2 (1.7 to 2.7)	212.6 (169.3 to 269.4)	27.8 (21.8 to 36.3)	29.4 (25.3 to 33.5)
			Male	1602.9 (1436.4 to 1781.8)	1252.3 (1119.3 to 1400.1)	30 (23.9 to 37.7)	250 (200.2 to 315.9)	35.2 (28.1 to 44.2)	35.5 (30.2 to 41.2)
	YLLs	% Change	Both	-59.5 (-65 to -47.7)	-60.6 (-66.1 to -47.5)	-60.9 (-72.7 to -35.4)	-60.4 (-67.8 to -52)	9.7 (-11.6 to 39.6)	-17.9 (-40.8 to 8.6)
			Female	-64.7 (-71.8 to -46)	-66.6 (-73.7 to -46.5)	-56.5 (-76.7 to -37.2)	-61.8 (-71 to -49.7)	15.2 (-19.5 to 52.3)	-24.9 (-53.2 to 5.1)
			Male	-54.8 (-61.7 to -43.9)	-55.3 (-62.6 to -43.7)	-60.5 (-73 to -32.4)	-59.3 (-67.5 to -48.6)	5.5 (-20.3 to 60.9)	-12.5 (-35.2 to 21.5)
		2019	Both	927.5 (823.1 to 1027)	762.8 (672.6 to 853.8)	7.2 (5.8 to 8.9)	115.7 (100.3 to 130.3)	27.2 (22.4 to 33.4)	14.6 (12.2 to 17.7)

SDI	Measure	Year	Sex	CRD	Cause specific				
					COPD	Pneumoconiosis	Asthma	ILD & pulmonary sarcoidosis	Other
			Female	688.5 (568.5 to 820.5)	548.3 (447.5 to 672.2)	1 (0.8 to 1.2)	104.6 (81.9 to 122.2)	23.8 (17.7 to 32.1)	10.8 (8.2 to 14.3)
			Male	1206.9 (1070.8 to 1361.6)	1015 (888.2 to 1150.4)	14.1 (11.2 to 17.7)	128.1 (111.7 to 153.6)	31.1 (23.8 to 40.3)	18.5 (14.2 to 23.5)
	YLDs	% Change	Both	-7.5 (-9.6 to -5.5)	-9.5 (-12.2 to -6.6)	-14.9 (-25.2 to -5.1)	-9.9 (-13 to -6.7)	18.6 (14.4 to 23.5)	69.8 (60.6 to 77.2)
			Female	-11.1 (-13.6 to -8.5)	-13.2 (-16.4 to -9.8)	-23.4 (-30 to -16.1)	-13.7 (-17.2 to -10.4)	17.6 (12.2 to 23.5)	68.6 (56.9 to 77.5)
			Male	-3.5 (-5.5 to -1.6)	-4.7 (-7.2 to -2.1)	-11.8 (-23.3 to -0.9)	-6.5 (-9.8 to -3.2)	19.7 (14.3 to 25.9)	71.6 (63.1 to 79.9)
		2019	Both	389.5 (313.1 to 462.8)	244.4 (203.2 to 278.2)	8.3 (5.5 to 12)	115 (74.2 to 172.5)	4 (2.7 to 5.9)	17.8 (14.4 to 20.7)
			Female	382 (307.8 to 451.2)	250.2 (207.3 to 288.6)	1.2 (0.8 to 1.7)	107.9 (69.6 to 161.4)	4 (2.7 to 5.8)	18.6 (15.1 to 21.8)
			Male	396 (314.3 to 482.7)	237.3 (195 to 273.6)	15.9 (10.4 to 22.9)	121.9 (78.5 to 182.5)	4.1 (2.7 to 6)	16.9 (13.7 to 20)
Low-middle SDI	Incidence	% Change	Both	-6.7 (-8.4 to -5.1)	-6.3 (-7.6 to -4.9)	-2.5 (-7.3 to 3)	-13.9 (-17.1 to -10.8)	2.2 (-0.6 to 4.9)	
			Female	-6 (-7.9 to -4.3)	-5.2 (-6.6 to -3.8)	40.2 (28 to 54.5)	-13.3 (-16.6 to -9.9)	2.4 (-1.2 to 5.7)	
			Male	-7.5 (-9.1 to -5.9)	-7 (-8.4 to -5.7)	-10.7 (-17 to -4.4)	-14.4 (-17.8 to -11.3)	1.6 (-1.2 to 4.6)	
		2019	Both	1022.4 (907.9 to 1142)	236.6 (223.9 to 247.7)	1.4 (1.2 to 1.6)	409.3 (335.6 to 505.7)	375.1 (302.4 to 452.6)	
			Female	1017.1 (906.9 to 1135.8)	230.8 (218.2 to 241.5)	0.8 (0.6 to 0.9)	402.4 (332.3 to 485.3)	383.2 (306.9 to 465.3)	
			Male	1027.4 (911.3 to 1149.8)	243.3 (230.6 to 254.5)	2 (1.7 to 2.3)	415.7 (336.8 to 519.6)	366.4 (295.9 to 441.6)	
	Prevalence	% Change	Both	-8.4 (-10.2 to -6.8)	-2.9 (-4.5 to -1.3)	-3.8 (-11.5 to 2.7)	-14 (-17.7 to -11)	2.1 (-0.6 to 4.9)	
			Female	-9.1 (-11.2 to -7.3)	-2.5 (-4.1 to -0.8)	0.9 (-4.8 to 7.1)	-15.8 (-19.7 to -12.7)	1.7 (-1.4 to 4.6)	
			Male	-7.8 (-9.6 to -6)	-3.5 (-5.2 to -1.8)	-0.2 (-9.2 to 7.5)	-12.1 (-16.1 to -9)	2 (-0.8 to 5.3)	
		2019	Both	5417.1 (5005.5 to 5903.1)	3005.2 (2849.4 to 3163.1)	18.3 (15.6 to 21.4)	2713.2 (2317.3 to 3209.7)	54.3 (45.8 to 63.3)	
			Female	5302.9 (4909.9 to 5726.8)	3014.1 (2857.4 to 3170.3)	4.6 (4 to 5.5)	2579.5 (2204.5 to 2998.1)	56.6 (47.7 to 66)	
			Male	5532.2 (5107.5 to 6060.7)	2995 (2841.5 to 3156.7)	32.9 (27.7 to 38.4)	2850.4 (2431.2 to 3416.3)	51.9 (43.7 to 60.4)	
	Deaths	% Change	Both	-33.1 (-44.2 to -23.8)	-30.1 (-41 to -20.1)	-46.7 (-58 to -16.5)	-49.6 (-60.7 to -39.2)	8.4 (-13.9 to 35.5)	-12.1 (-30.3 to 15.6)
			Female	-31.8 (-46.6 to -14.4)	-28.9 (-42.3 to -12.4)	-19.4 (-41.2 to 6.7)	-46.2 (-61.6 to -26)	24.9 (-7.2 to 70.1)	-11.7 (-36.2 to 20.2)
			Male	-33 (-45.4 to -20.6)	-29.5 (-43.8 to -16.3)	-46.2 (-58.7 to -9.7)	-53.2 (-65.5 to -34.4)	-1.2 (-25.8 to 64.6)	-10.9 (-31.4 to 35.9)
		2019	Both	107.3 (90.1 to 120.8)	86.1 (71.7 to 97)	0.3 (0.2 to 0.4)	16.3 (11.6 to 21.3)	3.7 (2.5 to 5.2)	0.9 (0.6 to 1.1)
			Female	91 (68.7 to 108.4)	70.1 (52.6 to 84.8)	0.1 (0.1 to 0.1)	16.8 (10.5 to 23.5)	3.3 (1.9 to 4.7)	0.6 (0.4 to 0.9)
			Male	126.3 (105.8 to 144.6)	104.7 (88 to 119.8)	0.6 (0.4 to 0.7)	15.6 (10.8 to 20.7)	4.2 (2.3 to 6.7)	1.1 (0.7 to 1.5)
	DALYs	% Change	Both	-34 (-42.5 to -26.5)	-31 (-40 to -22.7)	-42.3 (-53.6 to -18.2)	-48.1 (-57.1 to -39.5)	2.3 (-18.7 to 26.5)	9.2 (-12 to 34.8)
			Female	-32.4 (-43.4 to -18.9)	-29 (-39 to -15.7)	-19.2 (-36 to -1.9)	-46.2 (-58.3 to -31.5)	16.2 (-9.7 to 50.7)	15.6 (-13.4 to 44.5)
			Male	-34.3 (-45 to -23.5)	-31.2 (-44.2 to -19.3)	-41.7 (-54 to -13.9)	-50.1 (-60.4 to -35.4)	-5.2 (-29.7 to 51.3)	6.1 (-16.5 to 43.2)
		2019	Both	2314.2 (2029.7 to 2562.1)	1728.6 (1488.8 to 1923.7)	9.2 (7.2 to 11.3)	445.9 (348.5 to 546.3)	78.6 (54.3 to 107)	51.9 (43.2 to 59.9)
			Female	2000 (1615.7 to 2309.1)	1437.6 (1160.2 to 1690.3)	2 (1.6 to 2.5)	448.9 (313.8 to 577.4)	67.8 (41.1 to 96.4)	43.7 (36 to 52.7)
			Male	2664 (2301.4 to 3019)	2054.8 (1767.3 to 2331.9)	17.1 (13 to 21.1)	441.7 (339.1 to 568.8)	90.1 (51 to 140.5)	60.3 (46.4 to 73.6)
	YLLs	% Change	Both	-38.9 (-48.4 to -30.1)	-35.5 (-45.5 to -25.8)	-50.2 (-61.3 to -24.6)	-53.8 (-62.2 to -44.8)	2.2 (-20 to 29.5)	-17.1 (-36.2 to 11.7)

SDI	Measure	Year	Sex	CRD	Cause specific				
					COPD	Pneumoconiosis	Asthma	ILD & pulmonary sarcoidosis	Other
			Female	-38.2 (-50.9 to -22.3)	-34.6 (-47 to -18.5)	-27.2 (-47.2 to -2.8)	-51.2 (-63.4 to -35.2)	17.7 (-10.7 to 58.4)	-20.1 (-44.4 to 12.5)
			Male	-38.4 (-49.8 to -26.5)	-34.7 (-48.6 to -21.6)	-49.7 (-61.4 to -18.8)	-56.3 (-66.4 to -40.2)	-5.7 (-30.8 to 59.5)	-14.1 (-34.9 to 26)
		2019	Both	1843 (1554 to 2078.8)	1393 (1145.1 to 1575.9)	6.6 (4.8 to 8.4)	341.5 (250.4 to 429.7)	73 (50.1 to 101.3)	28.8 (21.2 to 36.4)
			Female	1525.3 (1153 to 1811.4)	1092.3 (808.2 to 1332.2)	1.3 (1 to 1.7)	350.1 (228 to 471.8)	62.1 (35.3 to 90.2)	19.7 (12.7 to 27.5)
			Male	2196.8 (1851.5 to 2539.1)	1729.9 (1450.5 to 2001.4)	12.4 (8.7 to 16)	331.6 (237.5 to 431.8)	84.8 (45.8 to 135.5)	38.2 (24.8 to 50.8)
	YLDs	% Change	Both	-3.4 (-5.3 to -1.6)	-3 (-4.9 to -1.2)	-3.8 (-12.6 to 4.4)	-13.7 (-17.4 to -10.5)	2.3 (-1.9 to 7.1)	80.6 (72.2 to 87.2)
			Female	-3.1 (-5.3 to -1)	-2.2 (-4.2 to -0.3)	1.1 (-5 to 8.7)	-15.5 (-19.5 to -12.3)	2.1 (-3.2 to 8)	82.1 (72.9 to 89.5)
			Male	-3.9 (-5.8 to -1.9)	-4.2 (-6.2 to -2.2)	-0.5 (-10.9 to 9.3)	-11.8 (-15.8 to -8.3)	2.2 (-3.2 to 8.4)	79 (69.7 to 87.4)
		2019	Both	471.3 (387.6 to 541.2)	335.6 (283.2 to 374.4)	2.6 (1.8 to 3.7)	104.4 (68.4 to 154.2)	5.5 (3.7 to 8.1)	23.1 (19 to 26.4)
			Female	474.6 (391 to 542)	345.3 (292.5 to 392.6)	0.7 (0.5 to 1)	98.9 (64.8 to 146.3)	5.7 (3.8 to 8.3)	24 (19.7 to 27.8)
			Male	467.1 (379.3 to 544.1)	324.9 (270.3 to 367.7)	4.7 (3.1 to 6.6)	110.1 (71.6 to 163)	5.3 (3.5 to 7.8)	22.1 (18.1 to 25.6)
Low SDI	Incidence	% Change	Both	-8.2 (-9.8 to -6.6)	-2.7 (-3.9 to -1.5)	-13.1 (-16.2 to -10)	-14 (-17 to -11)	0.8 (-1.8 to 3.4)	
			Female	-3.3 (-5.1 to -1.4)	4.4 (2.8 to 6)	46.6 (37.9 to 56.1)	-10.5 (-13.3 to -7.4)	7.5 (4 to 11.6)	
			Male	-13.3 (-15.4 to -11.4)	-9.2 (-10.8 to -7.7)	-32.9 (-36.2 to -29.4)	-17.6 (-21.2 to -14)	-6.7 (-9.3 to -4.1)	
		2019	Both	961.7 (852.9 to 1095)	172.6 (163 to 181.5)	0.6 (0.5 to 0.8)	511.2 (415.1 to 624.6)	277.2 (225.9 to 331.6)	
			Female	1020.3 (909.7 to 1150)	174.3 (164.8 to 183.2)	0.6 (0.5 to 0.7)	544.2 (448.7 to 657.6)	301.3 (245.3 to 359.7)	
			Male	902 (796.8 to 1034.4)	171.5 (161.4 to 180.9)	0.7 (0.6 to 0.8)	477.6 (381 to 602.2)	252.2 (206.2 to 301.7)	
	Prevalence	% Change	Both	-7.8 (-9.9 to -5.8)	2 (0.6 to 3.4)	-1.8 (-6 to 2.6)	-13.4 (-16.6 to -10.2)	2.9 (0.5 to 5.4)	
			Female	-9.2 (-10.9 to -7.2)	4.6 (2.6 to 6.3)	18.7 (8.5 to 30.1)	-16.6 (-19.1 to -13.8)	6.8 (3.3 to 10.4)	
			Male	-6.4 (-9.3 to -4)	-0.7 (-2.6 to 1.1)	-8 (-12.5 to -3.6)	-9.9 (-14.7 to -5.9)	-1.7 (-4.6 to 1.2)	
		2019	Both	5549.6 (5101.6 to 6120.7)	2335.7 (2211.1 to 2474.4)	3.9 (3.3 to 4.5)	3468.6 (3015.3 to 4035.8)	39.6 (33.5 to 46.1)	
			Female	5609.3 (5176.7 to 6130.7)	2366.1 (2242.5 to 2500.2)	2.6 (2.3 to 3.1)	3489.5 (3047 to 4005.6)	42.5 (36 to 49.6)	
			Male	5491 (5004.1 to 6116.1)	2307.5 (2179.4 to 2453.2)	5.1 (4.3 to 6.1)	3448 (2964.7 to 4110)	36.5 (30.9 to 42.6)	
	Deaths	% Change	Both	-23.4 (-33.7 to -13.8)	-16.5 (-27.3 to -4.4)	-35.5 (-50.3 to -8.3)	-43.1 (-51.1 to -33.4)	13.5 (-14.4 to 48)	-7.4 (-29.6 to 21.9)
			Female	-18 (-33.6 to 1.4)	-7.6 (-26.2 to 18.8)	-9.4 (-30.2 to 18.6)	-39 (-51.6 to -21.2)	37.3 (0.5 to 80)	-3.9 (-37.4 to 46.1)
			Male	-27.1 (-40.4 to -12.8)	-21.3 (-37.3 to -5.9)	-37.4 (-53.4 to -8.9)	-48.2 (-57.1 to -32.8)	-1.4 (-28.9 to 46.6)	-8.3 (-30.3 to 23.3)
		2019	Both	87.8 (74.2 to 97.7)	64.4 (54.4 to 73.3)	0.3 (0.1 to 0.4)	18.9 (13.4 to 28.7)	3.2 (2 to 4.4)	0.9 (0.7 to 1.2)
			Female	77.9 (58.1 to 93)	53 (38.5 to 66)	0.1 (0.1 to 0.1)	21.1 (12 to 33.7)	3 (1.6 to 4.4)	0.7 (0.4 to 0.9)
			Male	99.2 (84 to 112.8)	77.5 (63.3 to 88.9)	0.4 (0.1 to 0.7)	16.5 (11.2 to 25)	3.5 (1.6 to 5.5)	1.2 (0.7 to 1.7)
	DALYs	% Change	Both	-25.5 (-32.8 to -18.2)	-18.2 (-26.9 to -8.9)	-35.7 (-49.9 to -11.8)	-42.2 (-48.9 to -33)	5.8 (-19.5 to 35.5)	11.5 (-14.6 to 42.8)
			Female	-22.2 (-32.8 to -7.2)	-11.5 (-25.2 to 8.6)	-9.3 (-26.9 to 15.7)	-40.5 (-49.7 to -24.2)	26 (-4.1 to 62.1)	19 (-19.7 to 67.4)
			Male	-27.7 (-37.9 to -16.7)	-22 (-35.8 to -8.8)	-37.9 (-53.6 to -13.4)	-44.4 (-52.2 to -32.4)	-6.5 (-32.1 to 31.5)	6.9 (-17 to 39)
		2019	Both	2048.4 (1802 to 2247.1)	1364.1 (1201.6 to 1522.6)	6.2 (3.1 to 8.6)	553.9 (434.7 to 726.8)	66.6 (44.1 to 89.2)	57.6 (45.8 to 69.1)
			Female	1889.4 (1559.1 to 2169.1)	1176 (940.8 to 1401.5)	2.3 (1.6 to 3.4)	599.2 (405.6 to 848.5)	61.8 (36.3 to 87.2)	50.1 (37.5 to 63.8)
			Male	2221.4 (1970.7 to 2484.9)	1568.3 (1344.6 to 1785.3)	10.2 (3.8 to 15.6)	506.2 (387.4 to 668.2)	71.5 (37 to 110.6)	65.2 (46.7 to 81.6)
	YLLs	% Change	Both	-30.4 (-38.6 to -21.9)	-22.3 (-32.1 to -11.8)	-38 (-52.4 to -13.6)	-47.8 (-54.7 to -38.2)	6 (-20.7 to 38.3)	-11.6 (-36.8 to 23.2)
			Female	-27.4 (-39.7 to -9)	-15.9 (-32.1 to 9.8)	-13.8 (-32.7 to 14.2)	-45.1 (-55.2 to -26.4)	27.7 (-4.6 to 67.2)	-11.9 (-45.7 to 53.8)
			Male	-32.1 (-43.1 to -19.3)	-25.3 (-40.4 to -10)	-39.6 (-55.8 to -14.4)	-51.2 (-58.5 to -39)	-6.8 (-33.3 to 35.8)	-10.8 (-33.3 to 22.9)

SDI	Measure	Year	Sex	CRD	Cause specific				
					COPD	Pneumoconiosis	Asthma	ILD & pulmonary sarcoidosis	Other
		2019	Both	1601.3 (1372.3 to 1772.7)	1078.8 (919.7 to 1231.7)	5.6 (2.5 to 8)	419.8 (309.7 to 584.7)	62.5 (40 to 85.2)	34.7 (23.9 to 45.2)
			Female	1427.7 (1096.6 to 1696.9)	878.1 (646.6 to 1101.1)	1.9 (1.2 to 3)	464.7 (286.1 to 699.5)	57.4 (31.5 to 83)	25.6 (13.2 to 38.1)
			Male	1789.1 (1544.3 to 2038.1)	1295.6 (1067.7 to 1507.7)	9.4 (3 to 14.7)	372.4 (269.7 to 529)	67.7 (32.7 to 107.1)	44 (25.9 to 59.2)
	YLDs	% Change	Both	-0.7 (-2.8 to 1.6)	2.4 (0.4 to 4.2)	-1.3 (-7.1 to 4.5)	-13.1 (-16.4 to -9.7)	3.3 (-1.6 to 8.4)	84.8 (78 to 90.9)
			Female	-0.1 (-2.6 to 2.7)	4.9 (2.6 to 7.1)	18.6 (7.9 to 30.3)	-16.3 (-18.9 to -13.2)	6.8 (-0.1 to 14.2)	87.8 (79.7 to 95.3)
			Male	-1.5 (-3.8 to 0.7)	-0.7 (-2.9 to 1.6)	-7.5 (-14.4 to -0.1)	-9.5 (-14.3 to -5.4)	-0.9 (-7.3 to 5.2)	81.4 (73.7 to 89)
		2019	Both	447.1 (362.8 to 522.3)	285.3 (240.4 to 319.7)	0.6 (0.4 to 0.8)	134.1 (87.3 to 196.7)	4.1 (2.7 to 6.1)	22.9 (18.9 to 26.3)
			Female	461.6 (374.2 to 540.8)	297.9 (251.6 to 337.7)	0.4 (0.3 to 0.6)	134.5 (88.2 to 196.2)	4.4 (2.9 to 6.4)	24.5 (20.2 to 28.4)
			Male	432.3 (349.6 to 513.6)	272.7 (227.7 to 310)	0.8 (0.5 to 1.1)	133.7 (87.1 to 196.8)	3.9 (2.6 to 5.7)	21.2 (17.4 to 24.6)

* % Change (1990 to 2019). Data in parentheses are 95% Uncertainty Intervals (95% UIs)
CRD= Chronic Respiratory Disease, COPD= Chronic Obstructive Pulmonary Disease; ILD= Interstitial Lung Disease; DALYs= Disability-Adjusted Life Years; YLLs= Years of Life Lost; YLDs= Years Lived with Disability

Table S3. Results from decomposition analysis of the total incidence number in both sexes combined, by region and SDI

Location		New cases, No.		Expected new cases in 2019, No.		new cases change cause, 1990 to 2019, %			% 1990 - 2019 new cases overall change
		1990	2019	Given population growth alone	Given population growth and Aging	Due to Population Growth [a]	Due to Age Structure Change [b]	Due to Incidence Rates Change [c]	
**Global**		52,094,520	77,625,300	75,344,112	82,335,418	44.6%	13.4%	-9%	49%
**Socio-demographic index (SDI)**	High SDI	11,692,808	16,628,212	14,415,004	16,061,559	23.3%	14.1%	4.8%	42.2%
	High-middle SDI	11,008,278	13,350,581	13,687,284	15,616,398	24.3%	17.5%	-20.6%	21.3%
	Middle SDI	14,510,341	20,945,765	20,256,220	22,515,434	39.6%	15.6%	-10.8%	44.4%
	Low-middle SDI	9,797,254	15,677,195	15,298,734	16,968,784	56.2%	17%	-13.2%	60%
	Low SDI	5,054,489	9,354,697	10,801,832	10,442,090	113.7%	-7.1%	-21.5%	85.1%
**Global burden of Disease (GBD) Region**	Andean Latin America	593,228	925,985	988,200	901,389	66.6%	-14.6%	4.1%	56.1%
	Australasia	259,283	400,028	371,630	415,817	43.3%	17%	-6.1%	54.3%
	Caribbean	463,274	556,221	619,475	555,742	33.7%	-13.8%	0.1%	20.1%
	Central Asia	527,735	689,353	712,605	745,505	35%	6.2%	-10.6%	30.6%
	Central Europe	1,595,826	1,571,474	1,482,360	1,700,517	-7.1%	13.7%	-8.1%	-1.5%
	Central Latin America	1,874,163	2,588,455	2,855,044	2,800,534	52.3%	-2.9%	-11.3%	38.1%
	Central Sub-Saharan Africa	510,186	1,070,393	1,208,797	1,156,052	136.9%	-10.3%	-16.8%	109.8%
	East Asia	8,921,287	12,361,464	10,720,402	14,240,116	20.2%	39.5%	-21.1%	38.6%
	Eastern Europe	2,777,262	1,908,498	2,574,534	2,799,662	-7.3%	8.1%	-32.1%	-31.3%
	Eastern Sub-Saharan Africa	2,147,962	3,969,476	4,651,096	4,395,202	116.5%	-11.9%	-19.8%	84.8%
	High-income Asia Pacific	2,200,219	2,364,621	2,374,972	3,299,972	7.9%	42%	-42.5%	7.5%
	High-income North America	5,976,779	9,936,830	7,756,170	8,499,908	29.8%	12.4%	24%	66.3%
	North Africa and Middle East	3,154,020	5,803,364	5,564,468	5,389,298	76.4%	-5.6%	13.1%	84%
	Oceania	74,682	145,544	153,252	155,287	105.2%	2.7%	-13%	94.9%
	South Asia	8,725,665	16,529,328	14,350,591	17,770,461	64.5%	39.2%	-14.2%	89.4%
	Southeast Asia	3,666,592	5,302,086	5,292,494	5,302,295	44.3%	0.3%	0%	44.6%

Location	New cases, No.		Expected new cases in 2019, No.		new cases change cause, 1990 to 2019, %			% 1990 - 2019 new cases overall change
	1990	2019	Given population growth alone	Given population growth and Aging	Due to Population Growth [a]	Due to Age Structure Change [b]	Due to Incidence Rates Change [c]	
Southern Latin America	529,063	797,778	712,857	728,750	34.7%	3%	13%	50.8%
Southern Sub-Saharan Africa	511,314	718,466	765,368	799,550	49.7%	6.7%	-15.9%	40.5%
Tropical Latin America	2,291,772	2,490,194	3,351,819	3,002,106	46.3%	-15.3%	-22.3%	8.7%
Western Europe	3,611,012	3,912,148	4,096,576	4,590,023	13.4%	13.7%	-18.8%	8.3%
Western Sub-Saharan Africa	1,683,196	3,583,594	3,988,275	3,885,285	136.9%	-6.1%	-17.9%	112.9%

[a] **We** estimated the effect of population growth by applying the population size of 2019 onto the rate, sex, and age structure of 1990. In this hypothetical scenario, incidence of chronic respiratory diseases rose by 44.6% as the global population grew by 44.6% between 1990 and 2019, and incidence rates and age structure remained the same as in 1990.

[b] **We estimated the effect of aging by applying the age structure of 2019 onto the rate, sex distribution, and population size of 1990. Globally,** 13.4% of the change in incident cases between 1990 and 2019 can be attributed to the changing age structure of the population.

c We estimated the effect of changing incidence rate by applying the incidence rate for 1990 onto the population size and age structure of 2019. G**lobally, the change in incidence cases reported herein represents the proportion of the change in incident cases between 1990 and 2019 that is due to a change in incidence rates**.

Table S4. Age-standardised rate of incidence, prevalence, deaths, DALYs, YLLs, and YLDs from sub-causes of pneumoconiosis by SDI, by sex and for both sexes combined

Location	Measure	Year	Sex	Pneumoconiosis			
				Silicosis	Asbestosis	Coal workers pneumoconiosis	Other pneumoconiosis
Global	Incidence	% Change*	Both	-11.5 (-22.6 to -1)	-7.9 (-13.7 to -0.8)	-48.4 (-52.6 to -44.5)	-18.1 (-24 to -12.7)
			Female	-9.1 (-15.6 to -3.1)	27.2 (19.3 to 37.3)	-35.1 (-42.1 to -28.6)	-19.4 (-25.5 to -13.4)
			Male	-11.2 (-22.7 to -0.3)	-25.9 (-33.4 to -17)	-49.7 (-53.8 to -45.7)	-17.7 (-23.8 to -11.5)
		2019	Both	1.7 (1.4 to 2)	0.4 (0.3 to 0.5)	0.1 (0.1 to 0.1)	0.2 (0.2 to 0.3)
			Female	0.2 (0.1 to 0.2)	0.4 (0.3 to 0.5)	0 (0 to 0)	0.1 (0.1 to 0.1)
			Male	3.2 (2.6 to 3.8)	0.5 (0.4 to 0.6)	0.2 (0.1 to 0.2)	0.3 (0.2 to 0.4)
	Prevalence	% Change	Both	-4.6 (-17.8 to 6.3)	5.9 (-2.8 to 15.2)	-49 (-52.9 to -44.9)	-17.5 (-22.9 to -12.9)
			Female	-4.9 (-12 to 1.9)	36.2 (23.8 to 49)	-29.5 (-36.4 to -22.1)	-20.1 (-26 to -14.3)
			Male	-5.4 (-18.7 to 5.8)	-6.9 (-16.4 to 4)	-51.8 (-55.5 to -47.6)	-18.2 (-23.8 to -12.8)
		2019	Both	31.6 (26.1 to 37.9)	1 (0.8 to 1.2)	1.3 (1 to 1.6)	2.9 (2.4 to 3.6)
			Female	2.6 (2 to 3.3)	0.8 (0.6 to 1)	0.4 (0.3 to 0.5)	1.5 (1.2 to 1.9)
			Male	63.2 (52.1 to 76)	1.3 (1 to 1.5)	2.3 (1.8 to 2.8)	4.6 (3.7 to 5.7)
	Deaths	% Change	Both	-58.9 (-67.9 to -39.4)	15.6 (-8.5 to 33.6)	-68.8 (-75.1 to -54)	-34.3 (-48.1 to -9.6)
			Female	-36.2 (-63.2 to -14.3)	-17.4 (-35.2 to 6.8)	-46.8 (-62.2 to -20.8)	-22.4 (-46.9 to -3.4)
			Male	-60.3 (-69.1 to -41.1)	22.7 (-7.6 to 44.4)	-70.9 (-76.9 to -56.8)	-37.6 (-51.8 to -7.3)
		2019	Both	0.2 (0.1 to 0.2)	0 (0 to 0.1)	0 (0 to 0)	0 (0 to 0.1)
			Female	0 (0 to 0)	0 (0 to 0)	0 (0 to 0)	0 (0 to 0)
			Male	0.3 (0.3 to 0.4)	0.1 (0.1 to 0.1)	0.1 (0.1 to 0.1)	0.1 (0.1 to 0.1)
	DALYs	% Change	Both	-43.3 (-54.2 to -27.1)	-6.1 (-18.3 to 5.9)	-68.3 (-75.1 to -54.3)	-36.2 (-48 to -17.5)
			Female	-24.8 (-45.9 to -11.2)	-12.9 (-29.2 to 11.8)	-48.7 (-63 to -29.6)	-28.3 (-47.5 to -15.2)
			Male	-44.8 (-55.7 to -28.8)	-3.5 (-18.6 to 11.8)	-70.3 (-76.9 to -56.7)	-38.5 (-51.3 to -15.2)
		2019	Both	7.9 (6.2 to 10)	0.9 (0.7 to 1)	0.9 (0.7 to 1.2)	1.4 (1.1 to 1.8)
			Female	0.6 (0.5 to 0.8)	0.3 (0.2 to 0.4)	0.2 (0.1 to 0.2)	0.6 (0.5 to 0.8)
			Male	15.9 (12.6 to 20.1)	1.6 (1.2 to 1.9)	1.8 (1.3 to 2.3)	2.3 (1.8 to 3.1)
	YLLs	% Change	Both	-63 (-72 to -43.9)	-7.7 (-21 to 6.3)	-71.5 (-78.5 to -55)	-42.1 (-54.8 to -18.2)
			Female	-44.5 (-69 to -21.6)	-28.6 (-44.8 to -1.6)	-56.3 (-69.4 to -30.9)	-32.6 (-56.4 to -13.6)
			Male	-63.9 (-72.8 to -44.6)	-2.5 (-18.7 to 15.5)	-73 (-79.6 to -57.4)	-44.2 (-57.4 to -14.6)
		2019	Both	3.4 (2.8 to 4.2)	0.7 (0.5 to 0.9)	0.7 (0.5 to 1)	1 (0.8 to 1.3)
			Female	0.2 (0.2 to 0.3)	0.2 (0.1 to 0.2)	0.1 (0.1 to 0.2)	0.4 (0.3 to 0.5)
			Male	7 (5.7 to 8.7)	1.5 (1 to 1.7)	1.4 (1 to 2)	1.7 (1.2 to 2.4)
	YLDs	% Change	Both	-4.9 (-17.9 to 6.7)	3.1 (-5 to 12.8)	-47.2 (-51.8 to -42.2)	-16.8 (-22.4 to -11.8)
			Female	-3.2 (-11.8 to 5.1)	34.6 (22.1 to 47.8)	-29.2 (-39.1 to -16.9)	-19.6 (-26.4 to -12.3)
			Male	-5.6 (-18.6 to 6.3)	-10.8 (-20.2 to 0.1)	-49.9 (-54.7 to -44.7)	-17.3 (-23.3 to -11.5)
		2019	Both	4.5 (3 to 6.4)	0.1 (0.1 to 0.2)	0.2 (0.1 to 0.3)	0.4 (0.3 to 0.6)

Location	Measure	Year	Sex	Pneumoconiosis			
				Silicosis	Asbestosis	Coal workers pneumoconiosis	Other pneumoconiosis
			Female	0.4 (0.2 to 0.6)	0.1 (0.1 to 0.2)	0.1 (0 to 0.1)	0.2 (0.1 to 0.3)
			Male	8.9 (5.9 to 12.8)	0.2 (0.1 to 0.3)	0.4 (0.2 to 0.5)	0.7 (0.4 to 1)
High SDI	Incidence	% Change	Both	-31.8 (-43.8 to -19.2)	-6.5 (-16.3 to 4.7)	-40.1 (-43.5 to -36.2)	-32.8 (-41 to -24)
			Female	-8.4 (-23.3 to 6)	-39.7 (-49.8 to -26.4)	-17.6 (-27.7 to -5.3)	-7.7 (-16.5 to 0.7)
			Male	-36.5 (-47.2 to -24.6)	2.2 (-7.3 to 12.7)	-45.2 (-48.5 to -41.5)	-37.5 (-45.3 to -28.7)
		2019	Both	0.4 (0.4 to 0.5)	0.7 (0.6 to 0.9)	0.1 (0.1 to 0.1)	0.2 (0.1 to 0.2)
			Female	0 (0 to 0)	0.3 (0.2 to 0.3)	0 (0 to 0)	0 (0 to 0.1)
			Male	0.8 (0.7 to 1)	1.3 (1 to 1.6)	0.2 (0.1 to 0.2)	0.3 (0.3 to 0.4)
	Prevalence	% Change	Both	-24.9 (-41.7 to -11)	14.3 (1 to 29.6)	-42 (-46.5 to -37.5)	-30.7 (-39.3 to -22.7)
			Female	-7.8 (-25.4 to 8.2)	-42 (-52.1 to -28.3)	-17 (-25.8 to -5.6)	-10.3 (-19.7 to -1.3)
			Male	-32.5 (-47.1 to -19.5)	23.4 (10.3 to 37.9)	-48.4 (-52.5 to -44.1)	-37.8 (-45.3 to -30.1)
		2019	Both	5.8 (4.9 to 6.9)	1.9 (1.5 to 2.3)	0.9 (0.7 to 1)	2.4 (2 to 3.1)
			Female	0.4 (0.3 to 0.5)	0.4 (0.3 to 0.6)	0.2 (0.1 to 0.2)	0.8 (0.6 to 1)
			Male	12 (10.2 to 14.2)	3.7 (3 to 4.6)	1.7 (1.4 to 2)	4.6 (3.6 to 5.8)
	Deaths	% Change	Both	-66.2 (-72.1 to -50.1)	55.4 (-1.3 to 75.8)	-71 (-74.7 to -66.2)	-45.2 (-57.5 to 21.9)
			Female	-29.4 (-48.8 to 24.8)	16.7 (-35.5 to 53.7)	0 (-23.5 to 27)	-1.8 (-18.9 to 22.1)
			Male	-68.9 (-74.3 to -53.9)	48 (-8.5 to 68.8)	-74.2 (-77.5 to -70)	-48.7 (-60.6 to 20.7)
		2019	Both	0.1 (0.1 to 0.1)	0.1 (0.1 to 0.1)	0.1 (0 to 0.1)	0 (0 to 0)
			Female	0 (0 to 0)	0 (0 to 0)	0 (0 to 0)	0 (0 to 0)
			Male	0.2 (0.1 to 0.3)	0.2 (0.2 to 0.3)	0.1 (0.1 to 0.2)	0.1 (0 to 0.1)
	DALYs	% Change	Both	-63.8 (-69.3 to -54.6)	20.3 (-8.1 to 33.3)	-73.4 (-76.9 to -68.9)	-50 (-58.8 to -13.4)
			Female	-24.7 (-38.6 to 10.5)	-18.1 (-40.5 to 0.3)	-15.7 (-34.5 to 0.7)	-10 (-19.8 to 0)
			Male	-67.1 (-72.1 to -58.2)	18.1 (-12.6 to 31.6)	-76.1 (-79.2 to -72.1)	-54.8 (-63 to -18.2)
		2019	Both	2 (1.6 to 2.8)	1.8 (1.3 to 2)	1.1 (0.8 to 1.2)	0.8 (0.7 to 1.1)
			Female	0.2 (0.1 to 0.3)	0.2 (0.1 to 0.3)	0.1 (0.1 to 0.1)	0.2 (0.2 to 0.3)
			Male	4.2 (3.4 to 6)	3.8 (2.7 to 4.2)	2.3 (1.8 to 2.6)	1.5 (1.2 to 2.1)
	YLLs	% Change	Both	-73.8 (-78.2 to -61.6)	22.4 (-13.3 to 37.7)	-75.4 (-78.9 to -70.7)	-59 (-68.8 to -4.7)
			Female	-30.5 (-46.8 to 18.8)	-2.3 (-38.7 to 23.8)	-15.1 (-42.7 to 11.3)	-10.7 (-27.5 to 6.8)
			Male	-75.9 (-79.9 to -64.3)	18.1 (-17.6 to 33.5)	-77.7 (-80.8 to -73.5)	-62.7 (-72.1 to -5.9)
		2019	Both	1.1 (0.9 to 2)	1.5 (1 to 1.7)	1 (0.7 to 1.1)	0.5 (0.4 to 0.7)
			Female	0.1 (0.1 to 0.2)	0.2 (0.1 to 0.2)	0.1 (0 to 0.1)	0.1 (0.1 to 0.2)
			Male	2.5 (2 to 4.3)	3.2 (2.2 to 3.6)	2.1 (1.5 to 2.4)	0.9 (0.7 to 1.5)
	YLDs	% Change	Both	-23.8 (-41.8 to -8)	9.4 (-3.6 to 24.5)	-40.2 (-45.6 to -34.1)	-29.5 (-38 to -21.5)
			Female	-8.6 (-27.1 to 8.9)	-41.8 (-51.9 to -28.1)	-16.9 (-25.7 to -5.5)	-9.4 (-19 to 0.1)
			Male	-31.2 (-46.8 to -16.7)	18.5 (4.9 to 33.5)	-46.7 (-51.7 to -40.8)	-36.8 (-44.7 to -28.6)
		2019	Both	0.8 (0.6 to 1.2)	0.3 (0.2 to 0.4)	0.1 (0.1 to 0.2)	0.4 (0.2 to 0.5)
			Female	0.1 (0 to 0.1)	0.1 (0 to 0.1)	0 (0 to 0)	0.1 (0.1 to 0.2)

Location	Measure	Year	Sex	Pneumoconiosis			
				Silicosis	Asbestosis	Coal workers pneumoconiosis	Other pneumoconiosis
			Male	1.7 (1.2 to 2.4)	0.5 (0.4 to 0.8)	0.3 (0.2 to 0.4)	0.7 (0.4 to 1)
High-middle SDI	Incidence	% Change	Both	-6.1 (-19.4 to 5.6)	-23.4 (-31.8 to -14.4)	-44.1 (-49.6 to -37.9)	-10.6 (-18.3 to -5.2)
			Female	4.5 (-3.4 to 12.7)	1.7 (-6.3 to 9.5)	-22.9 (-34.5 to -11.1)	-17.8 (-25 to -11.3)
			Male	-7.6 (-20.5 to 4.2)	-38.8 (-47.8 to -28.6)	-45.7 (-51 to -39.9)	-8.8 (-18.8 to -2.4)
		2019	Both	2.5 (2.1 to 3)	0.3 (0.2 to 0.4)	0.1 (0.1 to 0.1)	0.2 (0.2 to 0.3)
			Female	0.2 (0.2 to 0.3)	0.3 (0.2 to 0.4)	0 (0 to 0)	0.1 (0.1 to 0.2)
			Male	4.9 (4 to 5.9)	0.3 (0.3 to 0.4)	0.2 (0.2 to 0.3)	0.4 (0.3 to 0.4)
	Prevalence	% Change	Both	-2.5 (-16.8 to 9.9)	-17.6 (-26.2 to -8.2)	-45 (-50.9 to -39.4)	-8.8 (-16.1 to -3.1)
			Female	9.2 (-0.5 to 20.3)	5.9 (-3.5 to 15)	-18.9 (-32 to -5.3)	-15.8 (-23.4 to -8.2)
			Male	-6.6 (-20.3 to 5.3)	-33.6 (-43.8 to -22.2)	-48 (-53.8 to -42.6)	-9.6 (-19.5 to -2.8)
		2019	Both	45.8 (37.8 to 55.3)	0.6 (0.4 to 0.7)	2 (1.6 to 2.5)	3.2 (2.6 to 4.1)
			Female	3.2 (2.5 to 4.2)	0.6 (0.5 to 0.8)	0.4 (0.3 to 0.5)	1.6 (1.2 to 2)
			Male	94.2 (77.8 to 113.6)	0.6 (0.4 to 0.7)	3.8 (3 to 4.7)	5.2 (4.1 to 6.6)
	Deaths	% Change	Both	-66.6 (-74 to -49.3)	-16 (-31.2 to -0.1)	-70.1 (-79.3 to -46)	-42 (-60.7 to -18.3)
			Female	-42.9 (-72.6 to -17.9)	-36.9 (-52.6 to 2.5)	-55.7 (-75.7 to -21.8)	-36.2 (-63.7 to -17.2)
			Male	-67.9 (-75 to -51.5)	-1.4 (-22.8 to 24.2)	-72.6 (-81.7 to -48.6)	-46.5 (-63.2 to -19.2)
		2019	Both	0.2 (0.1 to 0.2)	0 (0 to 0)	0 (0 to 0)	0 (0 to 0)
			Female	0 (0 to 0)	0 (0 to 0)	0 (0 to 0)	0 (0 to 0)
			Male	0.4 (0.3 to 0.5)	0 (0 to 0)	0.1 (0 to 0.1)	0 (0 to 0.1)
	DALYs	% Change	Both	-44 (-54.5 to -31)	-30.9 (-42.5 to -13.3)	-66 (-75.2 to -47.5)	-35.3 (-50.9 to -16.9)
			Female	-20.7 (-44.6 to -3.7)	-32.9 (-48.5 to 0.8)	-52 (-70.7 to -26.2)	-35 (-55.3 to -22.6)
			Male	-47.4 (-57.5 to -34.9)	-27.8 (-41.1 to -10.5)	-68.5 (-77.3 to -50.5)	-37.4 (-53.8 to -15.3)
		2019	Both	9.5 (7.1 to 12.4)	0.4 (0.4 to 0.5)	0.9 (0.6 to 1.1)	1.1 (0.9 to 1.4)
			Female	0.7 (0.5 to 1)	0.3 (0.2 to 0.3)	0.1 (0.1 to 0.2)	0.5 (0.4 to 0.6)
			Male	19.8 (15 to 25.7)	0.7 (0.5 to 0.8)	1.7 (1.3 to 2.3)	1.8 (1.5 to 2.4)
	YLLs	% Change	Both	-70.8 (-78.2 to -53.8)	-33.8 (-47 to -12.2)	-72.2 (-81.7 to -49.3)	-47.4 (-65.2 to -22)
			Female	-50.1 (-76.8 to -24.8)	-44.5 (-61.1 to -1.2)	-63.6 (-82 to -31.3)	-46.4 (-71.6 to -28.4)
			Male	-72.3 (-79.3 to -55.9)	-26.8 (-42 to -6.3)	-74 (-83.3 to -52.4)	-49.3 (-66.2 to -19.6)
		2019	Both	3 (2.4 to 4.7)	0.3 (0.3 to 0.4)	0.6 (0.4 to 0.8)	0.6 (0.5 to 0.9)
			Female	0.2 (0.1 to 0.3)	0.2 (0.1 to 0.2)	0.1 (0 to 0.1)	0.3 (0.2 to 0.3)
			Male	6.5 (5.3 to 10.1)	0.6 (0.5 to 0.7)	1.1 (0.7 to 1.6)	1 (0.8 to 1.5)
	YLDs	% Change	Both	-2.5 (-17.2 to 11.3)	-17.9 (-26.6 to -8.4)	-43.3 (-50.8 to -35.4)	-8.3 (-15.7 to -1.6)
			Female	12.3 (-2.4 to 28.5)	5.1 (-4.4 to 14.6)	-18 (-31.5 to -4)	-16 (-23.9 to -8.3)
			Male	-6.4 (-20.6 to 7)	-33.6 (-43.9 to -22.1)	-46.3 (-53.8 to -38.7)	-8.4 (-17.8 to 0.4)
		2019	Both	6.5 (4.3 to 9.3)	0.1 (0.1 to 0.1)	0.3 (0.2 to 0.4)	0.5 (0.3 to 0.7)
			Female	0.5 (0.3 to 0.7)	0.1 (0.1 to 0.1)	0.1 (0 to 0.1)	0.2 (0.2 to 0.4)

Location	Measure	Year	Sex	Pneumoconiosis			
				Silicosis	Asbestosis	Coal workers pneumoconiosis	Other pneumoconiosis
			Male	13.3 (8.8 to 19.1)	0.1 (0.1 to 0.1)	0.6 (0.4 to 0.9)	0.8 (0.5 to 1.1)
Middle SDI	Incidence	% Change	Both	-32.3 (-41.4 to -24.2)	-9.5 (-18.2 to 1.2)	-62.7 (-66.9 to -58.8)	-31 (-35.6 to -25.8)
			Female	-25.7 (-32.5 to -19.7)	75.2 (61 to 91.1)	-46.7 (-53.9 to -39.3)	-34.8 (-40.6 to -28.8)
			Male	-30.9 (-40.8 to -21.9)	-64.9 (-71.2 to -56.6)	-64.9 (-69.2 to -60.7)	-27.9 (-33.2 to -22.2)
		2019	Both	2 (1.6 to 2.4)	0.3 (0.3 to 0.4)	0.1 (0.1 to 0.1)	0.2 (0.2 to 0.3)
			Female	0.3 (0.2 to 0.3)	0.5 (0.4 to 0.6)	0 (0 to 0.1)	0.1 (0.1 to 0.2)
			Male	3.8 (3.1 to 4.5)	0.2 (0.1 to 0.2)	0.1 (0.1 to 0.2)	0.3 (0.2 to 0.4)
	Prevalence	% Change	Both	-11.3 (-23.5 to 0.2)	15 (3.1 to 29.4)	-55.6 (-60.5 to -51.1)	-21.7 (-26.5 to -16.5)
			Female	-24.8 (-31.4 to -18.2)	101.4 (85.6 to 119.2)	-41.6 (-49.7 to -33.5)	-35.9 (-41.8 to -29.8)
			Male	-8.3 (-21.2 to 4.1)	-57.8 (-65.8 to -47.8)	-57.8 (-62.9 to -52.6)	-11.5 (-16.9 to -5)
		2019	Both	52.1 (42.6 to 63.1)	0.7 (0.5 to 0.9)	1.8 (1.4 to 2.2)	3.9 (3.1 to 4.9)
			Female	4 (3.1 to 5.1)	1.1 (0.8 to 1.4)	0.8 (0.6 to 1)	2.4 (1.9 to 3)
			Male	103.9 (84.9 to 126.1)	0.3 (0.2 to 0.4)	2.7 (2.2 to 3.4)	5.5 (4.3 to 6.9)
	Deaths	% Change	Both	-58.2 (-71.8 to -24.7)	-29 (-46.1 to -7.1)	-65.2 (-79.3 to -21.3)	-38.7 (-57.9 to -8)
			Female	-47.6 (-77.9 to -16.4)	-43.5 (-59.9 to -7.7)	-69.3 (-81.9 to -42.6)	-41.7 (-66.7 to -17.2)
			Male	-58 (-72.3 to -22.1)	-22.3 (-43.9 to 15.1)	-64.4 (-79.6 to -14.2)	-37 (-58.3 to 8.8)
		2019	Both	0.2 (0.2 to 0.3)	0 (0 to 0)	0 (0 to 0.1)	0 (0 to 0.1)
			Female	0 (0 to 0)	0 (0 to 0)	0 (0 to 0)	0 (0 to 0)
			Male	0.5 (0.4 to 0.6)	0 (0 to 0)	0.1 (0 to 0.1)	0.1 (0.1 to 0.1)
	DALYs	% Change	Both	-43.5 (-56.8 to -22.7)	-25.7 (-43.9 to -4.4)	-65.8 (-78 to -37.1)	-40.2 (-57.6 to -18)
			Female	-39.9 (-61.5 to -24.1)	-15.9 (-40 to 29.5)	-62.1 (-77.8 to -43.6)	-44.9 (-64.2 to -31)
			Male	-42.5 (-56.5 to -19.7)	-29.2 (-49.7 to 3)	-65.7 (-79 to -32.2)	-37.3 (-57.6 to -2.9)
		2019	Both	12.5 (9.7 to 16.1)	0.5 (0.4 to 0.6)	1 (0.7 to 1.5)	1.6 (1.3 to 2.2)
			Female	0.9 (0.7 to 1.2)	0.3 (0.2 to 0.4)	0.2 (0.2 to 0.3)	0.8 (0.6 to 1)
			Male	25 (19.5 to 32.2)	0.6 (0.5 to 0.9)	1.8 (1.2 to 2.9)	2.5 (2 to 3.6)
	YLLs	% Change	Both	-62.7 (-75.7 to -33.6)	-32.7 (-50.8 to -8.6)	-69 (-82.1 to -27.3)	-47.1 (-65.8 to -15.7)
			Female	-56.7 (-81.7 to -28)	-48.3 (-65.9 to -10.2)	-74.2 (-85.6 to -49.5)	-51.5 (-74.1 to -28.8)
			Male	-62.1 (-75.7 to -31.3)	-25.4 (-48.7 to 13.9)	-68 (-82.3 to -20.4)	-44.8 (-65.4 to 0.8)
		2019	Both	5.1 (4 to 6.7)	0.4 (0.3 to 0.5)	0.7 (0.4 to 1.2)	1 (0.8 to 1.6)
			Female	0.3 (0.2 to 0.4)	0.2 (0.1 to 0.2)	0.1 (0.1 to 0.1)	0.4 (0.3 to 0.5)
			Male	10.4 (7.9 to 13.7)	0.6 (0.4 to 0.8)	1.3 (0.8 to 2.5)	1.7 (1.3 to 2.8)
	YLDs	% Change	Both	-11.8 (-24.2 to 0)	13.5 (1.2 to 28.5)	-53.8 (-59.9 to -47.5)	-21.7 (-27.8 to -14.8)
			Female	-23.1 (-32.8 to -12)	101.6 (84.4 to 122.7)	-41.5 (-54.3 to -24.9)	-35.2 (-43.5 to -25)
			Male	-8.9 (-21.8 to 3.8)	-57.5 (-65.5 to -47.6)	-55.6 (-62.1 to -48.5)	-11.8 (-19.9 to -2.8)
		2019	Both	7.4 (4.8 to 10.7)	0.1 (0.1 to 0.2)	0.3 (0.2 to 0.4)	0.6 (0.4 to 0.8)
			Female	0.6 (0.4 to 0.9)	0.2 (0.1 to 0.2)	0.1 (0.1 to 0.2)	0.4 (0.2 to 0.5)

Location	Measure	Year	Sex	Pneumoconiosis			
				Silicosis	Asbestosis	Coal workers pneumoconiosis	Other pneumoconiosis
			Male	14.6 (9.6 to 21.2)	0 (0 to 0.1)	0.4 (0.3 to 0.6)	0.8 (0.5 to 1.2)
Low-middle SDI	Incidence	% Change	Both	-8.4 (-15.6 to -0.9)	23.4 (15.6 to 31.9)	-37.3 (-43.1 to -32.2)	2.9 (-1.2 to 7)
			Female	-12 (-18.7 to -5.4)	81.8 (59.8 to 109.4)	-31.7 (-42.7 to -16.2)	12.8 (4.3 to 20.5)
			Male	-5 (-13.1 to 3.6)	-44.8 (-51.7 to -37)	-35.2 (-41.9 to -29.6)	1.7 (-2.5 to 6.2)
		2019	Both	0.8 (0.7 to 1)	0.3 (0.3 to 0.4)	0 (0 to 0.1)	0.2 (0.1 to 0.2)
			Female	0.1 (0.1 to 0.2)	0.5 (0.4 to 0.7)	0 (0 to 0)	0.1 (0.1 to 0.1)
			Male	1.5 (1.3 to 1.9)	0.1 (0.1 to 0.2)	0.1 (0.1 to 0.1)	0.2 (0.2 to 0.3)
	Prevalence	% Change	Both	-3.5 (-12.5 to 4.3)	32.2 (22.3 to 42.5)	-42.8 (-49.7 to -36.1)	4.5 (0 to 9.5)
			Female	-12.2 (-18.8 to -4.7)	97.9 (75.9 to 125.7)	-32.6 (-44.6 to -17.7)	9 (0.9 to 16.8)
			Male	1.9 (-8.5 to 10.9)	-43.3 (-50.7 to -35.3)	-42.3 (-50.1 to -34.3)	5.7 (0.5 to 11.4)
		2019	Both	15.5 (12.9 to 18.4)	0.5 (0.4 to 0.6)	0.5 (0.4 to 0.6)	1.8 (1.5 to 2.2)
			Female	2.6 (2.1 to 3.4)	0.8 (0.6 to 1)	0.2 (0.1 to 0.2)	1.1 (0.9 to 1.4)
			Male	29.2 (24.3 to 34.9)	0.2 (0.2 to 0.3)	0.8 (0.7 to 1)	2.6 (2.1 to 3.2)
	Deaths	% Change	Both	-51.9 (-63.5 to -13)	-7 (-32.1 to 43.9)	-58.8 (-71.4 to -26.5)	-32.1 (-51.6 to 15.2)
			Female	-17.2 (-49 to 24.4)	-2.4 (-31.3 to 51.9)	-41.6 (-60.5 to -7.3)	-17.3 (-40.1 to 13.6)
			Male	-50 (-62.5 to -6.7)	-5.1 (-34.9 to 85.5)	-58.7 (-72.7 to -20.3)	-32.6 (-53.5 to 40.6)
		2019	Both	0.2 (0.1 to 0.2)	0 (0 to 0)	0 (0 to 0)	0.1 (0.1 to 0.1)
			Female	0 (0 to 0)	0 (0 to 0)	0 (0 to 0)	0 (0 to 0)
			Male	0.3 (0.2 to 0.5)	0 (0 to 0.1)	0.1 (0 to 0.1)	0.1 (0.1 to 0.2)
	DALYs	% Change	Both	-43.8 (-56.5 to -15.2)	-8.2 (-31.8 to 34.1)	-60.6 (-72.4 to -33.9)	-33 (-50.8 to 6.8)
			Female	-17.5 (-37 to -2.4)	9.3 (-20.2 to 60.6)	-47.8 (-63.9 to -19.2)	-19.6 (-39.1 to 4.2)
			Male	-42.2 (-55.5 to -10.3)	-11.4 (-37.6 to 56.4)	-60.3 (-72.9 to -27.9)	-33.3 (-53.1 to 24.3)
		2019	Both	6 (4.5 to 7.5)	0.6 (0.4 to 0.8)	0.7 (0.4 to 1)	2 (1.3 to 2.9)
			Female	0.7 (0.5 to 0.9)	0.4 (0.2 to 0.5)	0.2 (0.1 to 0.3)	0.8 (0.6 to 1.1)
			Male	11.7 (8.7 to 14.7)	0.9 (0.5 to 1.3)	1.3 (0.7 to 1.9)	3.2 (1.9 to 5)
	YLLs	% Change	Both	-54.9 (-65.8 to -20.8)	-12.2 (-35.9 to 34.6)	-62.1 (-74.1 to -32.9)	-36.7 (-53.8 to 6.8)
			Female	-25.1 (-54.9 to 13.4)	-11.1 (-37.1 to 41.8)	-49.5 (-65.3 to -16.8)	-24.8 (-45.7 to 3.2)
			Male	-53.3 (-64.9 to -15.9)	-9.5 (-37.6 to 68.6)	-61.7 (-74.4 to -26.7)	-36.6 (-55.6 to 28.1)
		2019	Both	3.7 (2.5 to 4.9)	0.5 (0.3 to 0.7)	0.6 (0.3 to 0.9)	1.7 (1 to 2.6)
			Female	0.3 (0.2 to 0.4)	0.2 (0.1 to 0.4)	0.2 (0.1 to 0.3)	0.6 (0.4 to 0.9)
			Male	7.5 (4.9 to 10)	0.8 (0.4 to 1.3)	1.2 (0.6 to 1.7)	2.8 (1.5 to 4.6)
	YLDs	% Change	Both	-3.6 (-13.8 to 6.6)	31.9 (22.2 to 41.9)	-41.2 (-49.4 to -31.8)	4 (-2 to 10.1)
			Female	-11.8 (-19.7 to -2.8)	97.5 (75.3 to 125.6)	-32.3 (-44.2 to -17.6)	8 (-0.1 to 15.6)
			Male	1.7 (-10.2 to 13.5)	-42.8 (-50.1 to -34.8)	-40.4 (-50.1 to -29.4)	5.2 (-2.5 to 12.8)
		2019	Both	2.2 (1.5 to 3.1)	0.1 (0.1 to 0.1)	0.1 (0 to 0.1)	0.3 (0.2 to 0.4)
			Female	0.4 (0.3 to 0.6)	0.1 (0.1 to 0.2)	0 (0 to 0)	0.2 (0.1 to 0.2)
			Male	4.1 (2.8 to 5.9)	0 (0 to 0)	0.1 (0.1 to 0.2)	0.4 (0.3 to 0.6)

Location	Measure	Year	Sex	Pneumoconiosis			
				Silicosis	Asbestosis	Coal workers pneumoconiosis	Other pneumoconiosis
Low SDI	Incidence	% Change	Both	-18 (-23 to -13.7)	-14.5 (-19.1 to -9.9)	1.4 (-6.9 to 11)	3.3 (-1.8 to 8.4)
			Female	-4.1 (-16.7 to 10.4)	67.5 (53.8 to 82.3)	29.1 (15.3 to 46)	16.7 (8.4 to 26)
			Male	-18.4 (-23.4 to -13.8)	-46.5 (-50.1 to -43)	-3.4 (-13 to 7.6)	-3 (-9.6 to 3.9)
		2019	Both	0.2 (0.1 to 0.2)	0.4 (0.3 to 0.5)	0 (0 to 0)	0.1 (0.1 to 0.1)
			Female	0.1 (0 to 0.1)	0.4 (0.3 to 0.5)	0 (0 to 0)	0.1 (0.1 to 0.1)
			Male	0.3 (0.2 to 0.3)	0.3 (0.3 to 0.4)	0 (0 to 0)	0.1 (0.1 to 0.1)
	Prevalence	% Change	Both	-5.8 (-11.8 to -0.1)	-10.2 (-14.8 to -5.3)	16.2 (4.1 to 29.2)	19.6 (13.6 to 25.3)
			Female	0.2 (-13.3 to 18)	84.8 (71.4 to 101.2)	24.6 (14.1 to 36.6)	21.7 (13.2 to 30.5)
			Male	-5.3 (-10.9 to 0.4)	-44.4 (-48.1 to -40.5)	11.5 (-4.7 to 31.7)	19.3 (11.5 to 27.1)
		2019	Both	2.4 (1.9 to 3)	0.6 (0.5 to 0.7)	0.1 (0 to 0.1)	0.8 (0.6 to 1)
			Female	1.3 (1 to 1.8)	0.6 (0.5 to 0.8)	0 (0 to 0.1)	0.7 (0.5 to 0.8)
			Male	3.5 (2.8 to 4.4)	0.5 (0.4 to 0.7)	0.1 (0.1 to 0.1)	1 (0.8 to 1.3)
	Deaths	% Change	Both	-39.5 (-55.9 to 7.8)	-27 (-43.8 to 7.3)	-49.4 (-67.2 to -15.4)	-29.8 (-48.5 to 6.8)
			Female	2.2 (-26.1 to 48.1)	-6.8 (-33.2 to 57.7)	-17.9 (-44.3 to 28.5)	-10.9 (-32.5 to 20.2)
			Male	-38.8 (-56.2 to 12.8)	-28.6 (-49.8 to 13.2)	-54.8 (-70.3 to -19.6)	-34.1 (-53 to 12.2)
		2019	Both	0.1 (0 to 0.2)	0.1 (0 to 0.1)	0 (0 to 0)	0.1 (0 to 0.1)
			Female	0 (0 to 0)	0 (0 to 0)	0 (0 to 0)	0 (0 to 0.1)
			Male	0.2 (0 to 0.3)	0.1 (0 to 0.2)	0 (0 to 0.1)	0.1 (0 to 0.2)
	DALYs	% Change	Both	-38.9 (-55.4 to -0.1)	-28.5 (-45.2 to 1.9)	-52 (-68.5 to -20.8)	-29.9 (-48.2 to 5.1)
			Female	0.6 (-16.5 to 23.3)	-2.7 (-31.3 to 64.5)	-23.9 (-46.6 to 23.2)	-12.1 (-32.8 to 18.3)
			Male	-39 (-55.9 to 3.7)	-31.1 (-51.2 to 1.5)	-56.3 (-70.7 to -24.1)	-33.8 (-52.4 to 9)
		2019	Both	2.5 (1 to 4)	1.2 (0.5 to 2.2)	0.5 (0.2 to 0.7)	1.9 (1.1 to 2.9)
			Female	0.5 (0.3 to 0.7)	0.5 (0.2 to 1)	0.2 (0.1 to 0.8)	1.1 (0.7 to 1.9)
			Male	4.7 (1.5 to 7.7)	2 (0.5 to 4.1)	0.7 (0.2 to 1.2)	2.8 (1 to 4.7)
	YLLs	% Change	Both	-42.3 (-58.5 to 2.2)	-29.7 (-46.6 to 4.3)	-52.6 (-68.9 to -21.6)	-31.9 (-49.9 to 3.5)
			Female	1.2 (-26.6 to 47.2)	-12.9 (-39.1 to 50.7)	-24.8 (-47.4 to 23.3)	-14.6 (-35.2 to 17.2)
			Male	-41.7 (-58.5 to 5.5)	-30.4 (-51.6 to 9.8)	-56.7 (-71 to -24.6)	-35.5 (-54.3 to 7.3)
		2019	Both	2.2 (0.6 to 3.6)	1.1 (0.4 to 2.1)	0.4 (0.2 to 0.7)	1.8 (1 to 2.8)
			Female	0.2 (0.1 to 0.5)	0.4 (0.1 to 0.8)	0.2 (0.1 to 0.8)	1 (0.6 to 1.8)
			Male	4.2 (0.9 to 7.1)	1.9 (0.4 to 4.1)	0.7 (0.2 to 1.2)	2.6 (0.9 to 4.6)
	YLDs	% Change	Both	-5.1 (-13.7 to 3.5)	-10.3 (-14.8 to -5.6)	16 (4.2 to 29)	18.9 (12.9 to 25.1)
			Female	-0.1 (-13.8 to 19.7)	84.3 (70.9 to 100.1)	24.4 (14 to 36)	21.6 (12.3 to 31.4)
			Male	-4.3 (-14 to 6.2)	-43.6 (-47.3 to -39.7)	11.4 (-4.6 to 31.4)	18.4 (10.6 to 26.5)
		2019	Both	0.4 (0.2 to 0.5)	0.1 (0.1 to 0.1)	0 (0 to 0)	0.1 (0.1 to 0.2)
			Female	0.2 (0.1 to 0.3)	0.1 (0.1 to 0.1)	0 (0 to 0)	0.1 (0.1 to 0.2)
			Male	0.5 (0.3 to 0.8)	0.1 (0.1 to 0.1)	0 (0 to 0)	0.2 (0.1 to 0.2)

* % Change (1990 to 2019). Data in parentheses are 95% Uncertainty Intervals (95% UIs)
DALYs= Disability-Adjusted Life Years; YLLs= Years of Life Lost; YLDs= Years Lived with Disability

Table S5. Age-standardised rates and the total number of incidence, prevalence, deaths, DALYs, YLLs, and YLDs in CRDs and its sub causes (i.e., COPD, pneumoconiosis, asthma, ILD and pulmonary sarcoidosis, and other CRDs) in males, females, and both sexes combined in 7 GBD super regions, 21 GBD regions, and 204 countries and territories in 1990 and 2019 with percent change (1990 to 2019)

Chronic respiratory diseases

7 GBD super region	21 GBD region	Location	Measure	Age	1990			2019			Percent change (1990 to 2019)		
					Both	Female	Male	Both	Female	Male	Both	Female	Male
		Southeast Asia, East Asia, and Oceania	Incidence	All Ages	12,662,561 (10,906,361 to 15,113,976	5,834,925 (5,071,932 to 6,833,132	6,827,636 (5,785,353 to 8,192,721)	17,809,094 (15,848,820 to 20,002,722	8,123,040 (7,248,065 to 9,094,594)	9,686,054 (8,567,752 to 10,974,200)	40.6 (30.2 to 51.1)	39.2 (28.9 to 49.6)	41.9 (31.3 to 53)
		Southeast Asia, East Asia, and Oceania	Incidence	Age-standardized	872 (764.8 to 1002.2)	809.2 (713.9 to 923.4)	937.7 (820.5 to 1084.6)	816 (709.5 to 951.5)	730.8 (635.9 to 844.2)	904.6 (782.3 to 1058.7)	-6.4 (-8.7 to -4.2)	-9.7 (-12 to -7.5)	-3.5 (-6 to -1.2)
		Southeast Asia, East Asia, and Oceania	Prevalence	All Ages	77,661,671 (69,768,631 to 88,284,740	37,121,990 (33,527,034 to 41,690,360	40,539,681 (36,123,982 to 46,570,763	109,229,558 (100,009,247 to 119,727,457	52,516,768 (48,253,699 to 57,148,478	56,712,789 (51,902,073 to 62,517,425	40.6 (33.8 to 47.5)	41.5 (34.1 to 48.6)	39.9 (33.4 to 46.7)
		Southeast Asia, East Asia, and Oceania	Prevalence	Age-standardized	5619.1 (5145.4 to 6200)	5387 (4945.7 to 5926.3)	5834.8 (5323.5 to 6456.1)	4793.1 (4318.6 to 5388.8)	4466.8 (4040.5 to 4991.4)	5109.6 (4584 to 5790.2)	-14.7 (-17 to -12.2)	-17.1 (-19.5 to -14.7)	-12.4 (-14.7 to -10)
		Southeast Asia, East Asia, and Oceania	Deaths	All Ages	1,521,289 (1,138,010 to 1,697,250)	729,616 (482,578 to 841,148)	791,673 (604,513 to 906,326)	1,403,733 (1,227,839 to 1,639,633)	580,457 (469,378 to 751,371)	823,276 (705,468 to 960,962)	-7.7 (-22.5 to 35.8)	-20.4 (-39 to 37.6)	4 (-15.3 to 40.5)
		Southeast Asia, East Asia, and Oceania	Deaths	Age-standardized	187.3 (141.9 to 207.6)	162.8 (109 to 187.7)	221.5 (171.2 to 250.4)	65 (56.8 to 75.7)	47.5 (38.2 to 61.5)	90.5 (78.4 to 104.2)	-65.3 (-70.7 to -49.6)	-70.8 (-77.6 to -50.3)	-59.1 (-65.9 to -46.4)
		Southeast Asia, East Asia, and Oceania	DALYs	All Ages	36,055,742 (28,311,635 to 39,766,154	16,623,522 (11,745,412 to 18,890,219	19,432,220 (15,443,720 to 22,036,500	31,902,404 (28,759,956 to 35,875,171	13,445,450 (11,642,237 to 15,870,809	18,456,953 (16,268,810 to 20,883,059	-11.5 (-22.7 to 18.8)	-19.1 (-32.9 to 20.8)	-5 (-20 to 22.6)
		Southeast Asia, East Asia, and Oceania	DALYs	Age-standardized	3456.4 (2698.4 to 3803.6)	3019.3 (2130.5 to 3420.9)	3989 (3179 to 4511.4)	1329.4 (1196 to 1492.6)	1053.8 (912.3 to 1237.9)	1677.9 (1480.4 to 1895)	-61.5 (-66.4 to -47.9)	-65.1 (-71.1 to -47.3)	-57.9 (-64.3 to -46.3)
		Southeast Asia, East Asia, and Oceania	YLLs	All Ages	30,319,619 (22,545,114 to 33,885,015	13,625,741 (8,781,488 to 15,662,013	16,693,878 (12,691,749 to 19,184,590	22,908,326 (20,155,977 to 26,604,008	8,731,409 (7,133,473 to 11,115,628)	14,176,917 (12,135,640 to 16,515,915	-24.4 (-36.6 to 10)	-35.9 (-50.2 to 10)	-15.1 (-31.4 to 14.9)
		Southeast Asia, East Asia, and Oceania	YLLs	Age-standardized	3010.7 (2253.3 to 3359.8)	2556.4 (1685.8 to 2943.7)	3567 (2729.4 to 4062.3)	953.3 (842 to 1111.2)	675.7 (552 to 858.6)	1307.7 (1124.6 to 1519.2)	-68.3 (-73.3 to -54)	-73.6 (-79.4 to -55)	-63.3 (-70 to -51.1)
		Southeast Asia, East Asia, and Oceania	YLDs	All Ages	5,736,123 (4,581,005 to 6,901,990)	2,997,780 (2,414,680 to 3,535,633	2,738,342 (2,125,910 to 3,402,914)	8,994,078 (7,334,516 to 10,546,072	4,714,041 (3,826,067 to 5,503,241)	4,280,037 (3,390,643 to 5,227,294)	56.8 (48.9 to 65.8)	57.3 (48.8 to 66.8)	56.3 (48.4 to 65.2)
		Southeast Asia, East Asia, and Oceania	YLDs	Age-standardized	445.7 (361.2 to 525.4)	462.8 (377 to 540.2)	422 (332.8 to 513.8)	376.1 (303.8 to 445.5)	378.1 (306.6 to 444.9)	370.2 (292.2 to 453.4)	-15.6 (-18.5 to -12.8)	-18.3 (-21.5 to -15)	-12.3 (-15.1 to -9.6)
		East Asia	Incidence	All Ages	8,921,287 (7,711,141 to 10,521,559)	4,117,498 (3,600,885 to 4,827,057	4,803,788 (4,099,969 to 5,746,726)	12,361,464 (10,913,170 to 13,946,113	5,691,746 (5,070,292 to 6,398,399)	6,669,718 (5,855,290 to 7,583,223)	38.6 (26.9 to 50.8)	38.2 (26.6 to 49.8)	38.8 (27.1 to 52)
		East Asia	Incidence	Age-standardized	864.9 (757.7 to 998.6)	805.7 (711.4 to 923.7)	929 (811.3 to 1078.3)	771 (661.2 to 910.7)	694.2 (601 to 809.6)	851.5 (726 to 1008)	-10.9 (-13.7 to -8.3)	-13.8 (-16.6 to -11.2)	-8.3 (-11.5 to -5.5)
		East Asia	Prevalence	All Ages	54,752,610 (49,024,630 to 61,938,128	26,522,471 (23,992,058 to 29,838,540	28,230,139 (25,084,858 to 32,352,874	72,603,642 (66,122,037 to 79,486,904	36,334,420 (33,179,998 to 39,520,821	36,269,222 (32,954,823 to 40,111,474	32.6 (24.8 to 40.7)	37 (28.7 to 45.5)	28.5 (21.3 to 36.5)
		East Asia	Prevalence	Age-standardized	5478.2 (4977.4 to 6076.1)	5337.9 (4879.8 to 5884.1)	5567.2 (5032.1 to 6203)	4352.3 (3867.7 to 4978.7)	4176.3 (3726.1 to 4710.4)	4489.1 (3969.2 to 5168.8)	-20.6 (-23.5 to -17.5)	-21.8 (-24.8 to -18.7)	-19.4 (-22.3 to -16.2)
		East Asia	Deaths	All Ages	1,325,881 (975,800 to 1,485,484)	649,339 (427,790 to 751,864)	676,542 (510,754 to 783,993)	1,126,946 (969,591 to 1,362,127)	477,254 (375,996 to 639,963)	649,692 (539,161 to 782,846)	-15 (-30.6 to 31.9)	-26.5 (-45.1 to 34.8)	-4 (-24.8 to 37.3)
		East Asia	Deaths	Age-standardized	221.5 (166.3 to 246.5)	194 (129.7 to 223.6)	265.7 (206.4 to 302.4)	67.5 (57.8 to 82.3)	50 (39.2 to 66.9)	95.2 (80.6 to 112.5)	-69.5 (-74.8 to -53.5)	-74.2 (-80.6 to -54.2)	-64.2 (-71 to -51.4)
		East Asia	DALYs	All Ages	29,644,067 (22,556,379 to 33,046,668	13,892,011 (9,622,943 to 15,977,981	15,752,055 (11,961,458 to 18,262,462	23,441,600 (20,675,834 to 27,335,346	10,206,719 (8,656,087 to 12,581,281)	13,234,881 (11,273,205 to 15,510,182	-20.9 (-33 to 14.3)	-26.5 (-41.1 to 18.2)	-16 (-32.6 to 17.4)
		East Asia	DALYs	Age-standardized	3845.4 (2938.8 to 4279.4)	3398.9 (2360.8 to 3883.8)	4435.2 (3469.2 to 5082)	1270.9 (1120.5 to 1470.6)	1033.9 (874 to 1259.3)	1589.2 (1365.2 to 1845)	-67 (-71.9 to -52.6)	-69.6 (-75.5 to -51.5)	-64.2 (-70.7 to -51.3)
		East Asia	YLLs	All Ages	25,330,776 (18,393,528 to 28,691,296	11,550,821 (7,314,187 to 13,536,712	13,779,955 (10,174,652 to 16,142,515	17,074,996 (14,594,106 to 20,798,751	6,636,483 (5,231,729 to 8,923,804)	10,438,513 (8,539,120 to 12,690,152	-32.6 (-45.8 to 7.8)	-42.5 (-57.5 to 9.1)	-24.2 (-42 to 12.6)
		East Asia	YLLs	Age-standardized	3389.5 (2490.4 to 3804.6)	2906.7 (1893.8 to 3382.3)	4027.8 (3037.9 to 4630.9)	915.7 (786.7 to 1109.5)	654.3 (515.5 to 878.6)	1266.3 (1052 to 1525.1)	-73 (-78 to -57.8)	-77.5 (-83.3 to -58.4)	-68.6 (-75.3 to -55.2)
		East Asia	YLDs	All Ages	4,313,291 (3,471,397 to 5,115,075)	2,341,191 (1,895,914 to 2,763,387	1,972,100 (1,532,793 to 2,453,562)	6,366,604 (5,186,298 to 7,463,656	3,570,236 (2,907,434 to 4,200,794)	2,796,368 (2,197,926 to 3,446,290)	47.6 (38.7 to 57.6)	52.5 (42.4 to 63.1)	41.8 (33.2 to 51.1)
		East Asia	YLDs	Age-standardized	455.9 (373 to 537.2)	492.3 (401.2 to 576.2)	407.3 (319.6 to 499.6)	355.2 (287.6 to 422.4)	379.6 (308.1 to 448.7)	323 (252.1 to 403)	-22.1 (-25.5 to -18.6)	-22.9 (-26.5 to -19)	-20.7 (-24.3 to -17.1)
Southeast Asia, east Asia, and Oceania	East Asia	China	Incidence	All Ages	8,580,787 (7,416,100 to 10,131,160)	3,953,365 (3,454,757 to 4,636,335	4,627,422 (3,951,673 to 5,539,173)	11,907,502 (10,497,328 to 13,430,464	5,458,018 (4,847,601 to 6,145,232)	6,449,484 (5,660,642 to 7,347,522)	38.8 (26.9 to 51)	38.1 (26.3 to 49.7)	39.4 (27.4 to 52.9)
Southeast Asia, east Asia, and Oceania	East Asia	China	Incidence	Age-standardized	863.4 (756.5 to 998.1)	804 (709.2 to 921.7)	928 (809.1 to 1077.8)	767.5 (657.1 to 906.9)	689.4 (596.2 to 804)	849.3 (723.8 to 1004.8)	-11.1 (-13.9 to -8.5)	-14.2 (-17 to -11.6)	-8.5 (-11.6 to -5.6)
Southeast Asia, east Asia, and Oceania	East Asia	China	Prevalence	All Ages	52,390,890 (46,868,679 to 59,414,716	25,329,646 (22,901,392 to 28,529,080	27,061,244 (24,005,455 to 31,042,530	69,467,916 (63,164,092 to 76,091,527	34,583,660 (31,550,967 to 37,678,248	34,884,256 (31,635,188 to 38,608,279	32.6 (24.8 to 41)	36.5 (28.3 to 45.2)	28.9 (21.5 to 37.2)
Southeast Asia, east Asia, and Oceania	East Asia	China	Prevalence	Age-standardized	5436.5 (4929.7 to 6037.7)	5295.4 (4831.9 to 5836.5)	5524 (4987.2 to 6182.2)	4314.6 (3833.2 to 4932.8)	4127.4 (3676.4 to 4660.5)	4464.8 (3946.2 to 5139.6)	-20.6 (-23.6 to -17.5)	-22.1 (-25.2 to -18.9)	-19.2 (-22.2 to -15.9)
Southeast Asia, east Asia, and Oceania	East Asia	China	Deaths	All Ages	1,301,224 (955,216 to 1,458,961)	636,168 (417,647 to 736,503)	665,056 (500,797 to 771,944)	1,085,273 (929,797 to 1,320,157)	456,958 (357,118 to 619,556)	628,315 (518,961 to 759,702)	-16.6 (-32.3 to 30.3)	-28.2 (-46.8 to 32.8)	-5.5 (-26.5 to 35.9)
Southeast Asia, east Asia, and Oceania	East Asia	China	Deaths	Age-standardized	226.4 (170.2 to 251.4)	198.2 (132.7 to 228.2)	272.7 (212.1 to 310.4)	68 (57.8 to 83.4)	50.1 (39 to 67.9)	97.8 (82.2 to 115.8)	-70 (-75.2 to -53.9)	-74.7 (-81.1 to -54.6)	-64.1 (-71.2 to -51)
Southeast Asia, east Asia, and Oceania	East Asia	China	DALYs	All Ages	28,992,086 (22,016,545 to 32,387,823	13,563,126 (9,354,265 to 15,597,900	15,428,960 (11,690,964 to 17,938,478	22,520,549 (19,719,779 to 26,416,880	9,762,872 (8,202,723 to 12,068,535)	12,757,677 (10,792,960 to 15,031,868	-22.3 (-34.6 to 12.9)	-28 (-42.5 to 16.4)	-17.3 (-34 to 16.1)
Southeast Asia, east Asia, and Oceania	East Asia	China	DALYs	Age-standardized	3910.1 (2991.4 to 4343.3)	3453.8 (2395.2 to 3955.6)	4518.3 (3522.7 to 5188.2)	1270 (1114 to 1473.9)	1030.3 (866.8 to 1262.3)	1601.4 (1366.7 to 1870.7)	-67.5 (-72.6 to -53.1)	-70.2 (-76.2 to -52.3)	-64.6 (-71.2 to -51.8)
Southeast Asia, east Asia, and Oceania	East Asia	China	YLLs	All Ages	24,837,840 (18,033,553 to 28,115,667	11,311,547 (7,130,159 to 13,259,393	13,526,293 (9,969,899 to 15,899,913	16,390,267 (13,891,398 to 20,112,867	6,335,951 (4,961,240 to 8,611,122)	10,054,317 (8,162,621 to 12,340,456	-34 (-47.2 to 5.7)	-44 (-59.1 to 7.2)	-25.7 (-43.6 to 11.4)
Southeast Asia, east Asia, and Oceania	East Asia	China	YLLs	Age-standardized	3454.7 (2532 to 3878)	2961.9 (1926.5 to 3445.1)	4111.7 (3093.2 to 4737)	915.5 (781.7 to 1117.4)	651.9 (511.6 to 885)	1278.6 (1056.6 to 1545.6)	-73.5 (-78.6 to -58.4)	-78 (-83.7 to -58.8)	-68.9 (-75.7 to -55.6)
Southeast Asia, east Asia, and Oceania	East Asia	China	YLDs	All Ages	4,154,246 (3,340,119 to 4,921,403)	2,251,579 (1,821,318 to 2,656,691	1,902,667 (1,482,021 to 2,366,610)	6,130,282 (5,000,099 to 7,194,912	3,426,922 (2,782,704 to 4,038,126)	2,703,360 (2,123,785 to 3,332,636)	47.6 (38.5 to 57.8)	52.2 (41.8 to 62.9)	42.1 (33.4 to 51.7)
Southeast Asia, east Asia, and Oceania	East Asia	China	YLDs	Age-standardized	455.4 (372.6 to 537.6)	491.9 (401 to 577)	406.7 (319.2 to 499.3)	354.4 (287.1 to 421.8)	378.3 (306.5 to 448)	322.8 (252.1 to 402.8)	-22.2 (-25.8 to -18.6)	-23.1 (-26.9 to -19.1)	-20.6 (-24.4 to -17)
Southeast Asia, east Asia, and Oceania	East Asia	Democratic People's Republic of Korea	Incidence	All Ages	192,096 (164,563 to 227,951)	99,792 (86,196 to 117,861)	92,304 (77,937 to 112,634)	266,642 (243,942 to 292,020)	147,553 (135,977 to 160,367)	119,089 (107,146 to 133,301)	38.8 (26.5 to 51.9)	47.9 (30.4 to 63.1)	29 (17.2 to 40.6)
Southeast Asia, east Asia, and Oceania	East Asia	Democratic People's Republic of Korea	Incidence	Age-standardized	997.8 (881.2 to 1145.1)	945.3 (829.5 to 1097)	1068.3 (941.5 to 1235)	986.3 (875.6 to 1124)	978.3 (877.6 to 1097.2)	1004.2 (882.4 to 1156.3)	-1.1 (-7.6 to 2.7)	3.5 (-8.2 to 10.3)	-6 (-9.8 to -2)
Southeast Asia, east Asia, and Oceania	East Asia	Democratic People's Republic of Korea	Prevalence	All Ages	1,317,956 (1,190,579 to 1,501,193)	724,256 (647,921 to 868,367)	593,701 (536,095 to 669,594)	2,029,244 (1,912,115 to 2,164,843	1,207,696 (1,140,653 to 1,282,182)	821,548 (762,577 to 894,455)	54 (39.3 to 64.2)	66.7 (41 to 82)	38.4 (29.7 to 46.6)
Southeast Asia, east Asia, and Oceania	East Asia	Democratic People's Republic of Korea	Prevalence	Age-standardized	7401.6 (6787.5 to 8331.4)	7204.3 (6493.5 to 8552.8)	7570.8 (7026.6 to 8312.1)	6982 (6486.5 to 7650.3)	7310.6 (6807.2 to 7929.8)	6488.9 (5940.5 to 7230.2)	-5.7 (-14.3 to -1.3)	1.5 (-13.1 to 9.2)	-14.3 (-17.9 to -10.6)
Southeast Asia, east Asia, and Oceania	East Asia	Democratic People's Republic of Korea	Deaths	All Ages	19,507 (14,226 to 25,359)	11,389 (7,207 to 16,207)	8,118 (5,862 to 10,412)	31,704 (25,210 to 37,877)	17,118 (13,137 to 22,390)	14,586 (10,866 to 17,255)	62.5 (23.3 to 115.4)	50.3 (8.3 to 118.7)	79.7 (34.1 to 131.3)
Southeast Asia, east Asia, and Oceania	East Asia	Democratic People's Republic of Korea	Deaths	Age-standardized	174.3 (126.7 to 231.3)	153 (98 to 219.8)	223.6 (170.5 to 276.7)	112.8 (90.3 to 136.5)	90.3 (69.6 to 118.3)	159.9 (121.1 to 188.8)	-35.3 (-50.8 to -14.7)	-40.9 (-57 to -16.5)	-28.5 (-44.4 to -11.6)
Southeast Asia, east Asia, and Oceania	East Asia	Democratic People's Republic of Korea	DALYs	All Ages	492,235 (373,486 to 607,475)	267,533 (188,839 to 355,025)	224,703 (165,669 to 286,702)	720,735 (597,373 to 834,077)	369,229 (302,689 to 444,365)	351,506 (269,097 to 416,374)	46.4 (16.9 to 84.8)	38 (6.3 to 83.5)	56.4 (21.8 to 97.7)
Southeast Asia, east Asia, and Oceania	East Asia	Democratic People's Republic of Korea	DALYs	Age-standardized	3444.2 (2634.2 to 4285.7)	3028.7 (2145 to 4034.3)	4227.1 (3240.5 to 5258.5)	2369.3 (1964 to 2733.8)	1980 (1625.5 to 2359.6)	3038.4 (2349.5 to 3533.1)	-31.2 (-45.3 to -13.3)	-34.6 (-49.7 to -13.4)	-28.1 (-43.8 to -11.5)
Southeast Asia, east Asia, and Oceania	East Asia	Democratic People's Republic of Korea	YLLs	All Ages	394,666 (279,223 to 508,915)	207,327 (129,728 to 295,162)	187,339 (128,930 to 248,838)	555,222 (434,945 to 666,392)	262,286 (199,471 to 338,403)	292,937 (212,502 to 355,812)	40.7 (6.4 to 90.7)	26.5 (-9 to 85)	56.4 (14.9 to 108.7)
Southeast Asia, east Asia, and Oceania	East Asia	Democratic People's Republic of Korea	YLLs	Age-standardized	2859.8 (2074.7 to 3708.2)	2410.8 (1523.7 to 3413)	3711.2 (2718.2 to 4704.4)	1816.8 (1425.4 to 2176.4)	1364.6 (1038.8 to 1746.7)	2582.2 (1921.9 to 3067.1)	-36.5 (-51.7 to -14.7)	-43.4 (-59.3 to -17.8)	-30.4 (-47.7 to -11.4)
Southeast Asia, east Asia, and Oceania	East Asia	Democratic People's Republic of Korea	YLDs	All Ages	97,570 (78,115 to 115,845)	60,206 (48,915 to 70,823)	37,364 (28,698 to 46,884)	165,513 (135,096 to 190,409)	106,944 (88,472 to 123,086)	58,570 (45,228 to 71,851)	69.6 (57.7 to 79.5)	77.6 (59.9 to 89.1)	56.8 (44.9 to 69.1)
Southeast Asia, east Asia, and Oceania	East Asia	Democratic People's Republic of Korea	YLDs	Age-standardized	584.4 (478.3 to 678)	617.9 (506.9 to 720.1)	515.9 (406.9 to 628.9)	552.5 (448.4 to 642.2)	615.4 (505.4 to 713.2)	456.2 (353.6 to 559.5)	-5.5 (-10.9 to -1.6)	-0.4 (-8.8 to 5.1)	-11.6 (-16.2 to -7.1)
Southeast Asia, east Asia, and Oceania	East Asia	Taiwan (Province of China)	Incidence	All Ages	148,404 (127,403 to 177,895)	64,341 (55,125 to 76,418)	84,063 (71,966 to 101,545)	187,319 (165,950 to 210,505)	86,175 (75,608 to 97,672)	101,144 (89,016 to 114,922)	26.2 (12.3 to 39.8)	33.9 (19.8 to 50.1)	20.3 (4.8 to 35.5)
Southeast Asia, east Asia, and Oceania	East Asia	Taiwan (Province of China)	Incidence	Age-standardized	825 (712.3 to 979.1)	743.9 (642.2 to 876)	897.1 (769.5 to 1075.7)	790.9 (657.6 to 975.2)	707.7 (590.3 to 856.6)	878.3 (721.8 to 1089.6)	-4.1 (-13.1 to 7.4)	-4.9 (-13.7 to 6.2)	-2.1 (-13.1 to 11.3)
Southeast Asia, east Asia, and Oceania	East Asia	Taiwan (Province of China)	Prevalence	All Ages	1,043,764 (922,241 to 1,221,808)	468,570 (413,060 to 547,669)	575,194 (504,660 to 678,565)	1,106,482 (992,378 to 1,242,213)	543,064 (483,307 to 615,869)	563,417 (504,839 to 633,667)	6 (-3.8 to 16.1)	15.9 (5.7 to 26.9)	-2 (-13.3 to 9.4)
Southeast Asia, east Asia, and Oceania	East Asia	Taiwan (Province of China)	Prevalence	Age-standardized	5797.8 (5166.7 to 6705.2)	5413.3 (4824.9 to 6196.5)	6143.8 (5449.7 to 7170.4)	4368.2 (3727.1 to 5248.2)	4104.5 (3476.4 to 4932.6)	4638.1 (3964.4 to 5611.6)	-24.7 (-32.6 to -13.9)	-24.2 (-32.1 to -14.8)	-24.5 (-33.3 to -12.2)
Southeast Asia, east Asia, and Oceania	East Asia	Taiwan (Province of China)	Deaths	All Ages	5,150 (4,562 to 5,518)	1,782 (1,293 to 2,010)	3,368 (3,100 to 3,619)	9,969 (7,858 to 12,656)	3,178 (2,263 to 4,872)	6,790 (5,392 to 8,460)	93.6 (51.7 to 157.3)	78.4 (25.2 to 212.7)	101.6 (60.7 to 151)
Southeast Asia, east Asia, and Oceania	East Asia	Taiwan (Province of China)	Deaths	Age-standardized	45 (39.1 to 48.8)	32.3 (23.9 to 36.8)	58.8 (53.9 to 63.5)	24.4 (19.2 to 31.1)	13.9 (9.9 to 21.5)	37.5 (29.7 to 46.8)	-45.8 (-57.5 to -27.5)	-56.8 (-69.5 to -25.1)	-36.2 (-49.2 to -20)
Southeast Asia, east Asia, and Oceania	East Asia	Taiwan (Province of China)	DALYs	All Ages	159,745 (141,383 to 178,089)	61,352 (51,417 to 69,544)	98,393 (88,589 to 109,508)	200,315 (167,988 to 242,314)	74,618 (60,872 to 99,084)	125,698 (104,526 to 151,928)	25.4 (6.5 to 52.2)	21.6 (2.3 to 70.3)	27.8 (7 to 53.2)
Southeast Asia, east Asia, and Oceania	East Asia	Taiwan (Province of China)	DALYs	Age-standardized	1054.8 (941.4 to 1157.3)	832.5 (695.4 to 932.2)	1268.3 (1147.4 to 1388)	584.1 (491.3 to 710.3)	429.6 (347.1 to 556.3)	770.9 (642.1 to 929.7)	-44.6 (-52.4 to -33.5)	-48.4 (-56.5 to -29.7)	-39.2 (-48.5 to -27.7)
Southeast Asia, east Asia, and Oceania	East Asia	Taiwan (Province of China)	YLLs	All Ages	98,271 (87,705 to 104,549)	31,947 (22,693 to 35,578)	66,324 (61,417 to 71,113)	129,507 (100,598 to 168,901)	38,247 (27,422 to 60,368)	91,260 (71,429 to 115,460)	31.8 (1.6 to 76.9)	19.7 (-15.7 to 122.3)	37.6 (7.1 to 73.3)
Southeast Asia, east Asia, and Oceania	East Asia	Taiwan (Province of China)	YLLs	Age-standardized	700.1 (620.9 to 748.7)	479.8 (345.8 to 537.1)	914 (841.6 to 976.8)	329.8 (255.8 to 430.4)	178.9 (128.8 to 282.6)	512.2 (400.1 to 645.8)	-52.9 (-63.6 to -36.5)	-62.7 (-73.6 to -31.8)	-44 (-56.4 to -29.2)
Southeast Asia, east Asia, and Oceania	East Asia	Taiwan (Province of China)	YLDs	All Ages	61,474 (46,320 to 78,947)	29,405 (22,822 to 36,554)	32,069 (23,549 to 42,579)	70,809 (55,400 to 86,672)	36,371 (28,654 to 44,288)	34,438 (26,299 to 43,043)	15.2 (5.7 to 24.9)	23.7 (13.8 to 34.3)	7.4 (-3.6 to 18.7)
Southeast Asia, east Asia, and Oceania	East Asia	Taiwan (Province of China)	YLDs	Age-standardized	354.7 (272.7 to 446.7)	352.7 (276.9 to 431)	354.3 (264.4 to 459.4)	254.2 (190.2 to 328.2)	250.7 (189.3 to 320.5)	258.7 (188.8 to 340.5)	-28.3 (-34.7 to -20.6)	-28.9 (-35.6 to -20.8)	-27 (-34.3 to -17.3)
		Southeast Asia	Incidence	All Ages	3,666,592 (3,087,937 to 4,451,223)	1,681,972 (1,428,443 to 2,033,624	1,984,620 (1,659,793 to 2,430,307)	5,302,086 (4,662,608 to 6,156,062	2,362,545 (2,071,628 to 2,724,971)	2,939,541 (2,579,417 to 3,419,388)	44.6 (36.6 to 53.4)	40.5 (32.3 to 49.2)	48.1 (39.9 to 57)
		Southeast Asia	Incidence	Age-standardized	851.6 (749.2 to 986)	775 (681.6 to 898.9)	932.5 (819.4 to 1075.2)	853.5 (745.9 to 996.5)	748.5 (652 to 869.6)	965.7 (845.6 to 1124.6)	0.2 (-2 to 2.6)	-3.4 (-5.9 to -1.1)	3.6 (1.2 to 6.1)
		Southeast Asia	Prevalence	All Ages	22,527,769 (20,043,832 to 25,889,916	10,412,608 (9,282,804 to 11,951,662	12,115,160 (10,738,811 to 13,975,980	35,926,312 (32,695,055 to 39,797,804	15,832,199 (14,385,995 to 17,517,827	20,094,112 (18,337,730 to 22,310,626	59.5 (52.8 to 66.1)	52 (45.7 to 58.6)	65.9 (58.6 to 73)
		Southeast Asia	Prevalence	Age-standardized	5847.8 (5367.7 to 6464.5)	5270.4 (4825.7 to 5836.9)	6493.5 (5957.7 to 7169.9)	5651.1 (5157.9 to 6262.3)	4834 (4389.9 to 5386.5)	6583.2 (6037 to 7266)	-3.4 (-4.9 to -1.5)	-8.3 (-10.1 to -6.4)	1.4 (-0.4 to 3.5)
		Southeast Asia	Deaths	All Ages	190,752 (151,873 to 213,268)	78,179 (51,838 to 93,254)	112,573 (94,232 to 126,513)	267,847 (233,250 to 296,152)	98,997 (74,663 to 114,437)	168,850 (147,952 to 188,926)	40.4 (20.3 to 66)	26.6 (-1.5 to 68.7)	50 (27.2 to 88.4)
		Southeast Asia	Deaths	Age-standardized	90.4 (70.8 to 102.5)	67.3 (43.1 to 82.6)	120.7 (100.3 to 135.7)	53.7 (46.5 to 59.5)	35.5 (27 to 41)	78.8 (69 to 88.3)	-40.6 (-49.5 to -29)	-47.3 (-60.4 to -27.6)	-34.7 (-44.6 to -18.5)
		Southeast Asia	DALYs	All Ages	6,257,860 (5,435,239 to 6,867,517)	2,659,028 (2,073,764 to 3,002,730	3,598,832 (3,127,177 to 3,998,885)	8,177,396 (7,253,985 to 8,958,447	3,100,992 (2,575,809 to 3,491,005)	5,076,404 (4,472,840 to 5,626,315)	30.7 (18.2 to 46)	16.6 (3.1 to 35.8)	41.1 (24.3 to 67.5)
		Southeast Asia	DALYs	Age-standardized	2163.5 (1822.6 to 2374)	1690.5 (1255.2 to 1933.1)	2734.5 (2358.6 to 3025.6)	1383.1 (1235.3 to 1511.7)	980 (814.2 to 1101.1)	1886.8 (1667.4 to 2085.8)	-36.1 (-43 to -27.6)	-42 (-50.5 to -30)	-31 (-39.5 to -17.5)
		Southeast Asia	YLLs	All Ages	4,856,282 (4,080,208 to 5,322,727)	2,013,191 (1,415,551 to 2,286,094	2,843,091 (2,403,103 to 3,161,791)	5,593,106 (4,847,617 to 6,230,999	1,979,475 (1,468,997 to 2,308,478)	3,613,632 (3,129,920 to 4,086,107)	15.2 (0.6 to 34.3)	-1.7 (-17.5 to 22)	27.1 (8 to 59.4)
		Southeast Asia	YLLs	Age-standardized	1762.2 (1417.7 to 1954.3)	1336.4 (915.7 to 1551.4)	2279 (1907 to 2545.8)	972.8 (849.4 to 1081.8)	638.4 (471.6 to 738.7)	1394.8 (1221.6 to 1560.2)	-44.8 (-52.5 to -35)	-52.2 (-61.3 to -38.3)	-38.8 (-48 to -23.3)
		Southeast Asia	YLDs	All Ages	1,401,577 (1,071,445 to 1,779,598)	645,837 (491,969 to 824,242)	755,740 (576,413 to 961,633)	2,584,290 (2,034,667 to 3,143,726	1,121,518 (887,278 to 1,379,405)	1,462,772 (1,147,917 to 1,783,048)	84.4 (74 to 95.1)	73.7 (64.4 to 83.9)	93.6 (81.6 to 105.5)
		Southeast Asia	YLDs	Age-standardized	401.3 (314.8 to 490.6)	354.2 (278.6 to 434.1)	455.6 (357.8 to 557.3)	410.3 (324.7 to 495.6)	341.7 (271 to 418.3)	491.9 (387.9 to 594.3)	2.3 (0.5 to 4.2)	-3.5 (-5.5 to -1.5)	8 (5.6 to 10.4)
Southeast Asia, east Asia, and Oceania	Southeast Asia	Cambodia	Incidence	All Ages	59,353 (47,743 to 73,695)	28,254 (22,824 to 35,195)	31,099 (24,874 to 38,929)	102,125 (87,364 to 122,299)	47,499 (40,616 to 56,384)	54,626 (46,434 to 66,111)	72.1 (58.1 to 87.7)	68.1 (53.8 to 85.4)	75.7 (60.6 to 93.1)
Southeast Asia, east Asia, and Oceania	Southeast Asia	Cambodia	Incidence	Age-standardized	582.6 (511.6 to 670)	526.6 (454.1 to 610)	652.1 (573.2 to 748.5)	682.2 (598.7 to 797.1)	602.4 (522.6 to 707.5)	784.9 (693.1 to 909.8)	17.1 (11.8 to 23.5)	14.4 (8.4 to 21.5)	20.4 (13.9 to 28)
Southeast Asia, east Asia, and Oceania	Southeast Asia	Cambodia	Prevalence	All Ages	315,476 (273,303 to 372,245)	154,771 (134,872 to 181,360)	160,705 (137,762 to 191,978)	639,096 (574,223 to 725,459)	297,709 (267,054 to 336,886)	341,387 (306,447 to 390,076)	102.6 (88.8 to 117.1)	92.4 (79 to 108.5)	112.4 (95.3 to 131.3)
Southeast Asia, east Asia, and Oceania	Southeast Asia	Cambodia	Prevalence	Age-standardized	3828 (3523.3 to 4188.5)	3454.8 (3175.7 to 3809.8)	4312.2 (3969.8 to 4724.8)	4449.5 (4074.8 to 4933.3)	3846.7 (3487 to 4296.4)	5288.8 (4858.4 to 5830.8)	16.2 (11.5 to 21.4)	11.3 (5.5 to 17.7)	22.6 (16.5 to 29.2)
Southeast Asia, east Asia, and Oceania	Southeast Asia	Cambodia	Deaths	All Ages	2,738 (2,257 to 3,164)	1,073 (754 to 1,337)	1,664 (1,360 to 1,973)	4,959 (3,936 to 5,791)	1,986 (1,411 to 2,443)	2,973 (2,351 to 3,509)	81.2 (43.8 to 128.6)	85.1 (41.1 to 160.7)	78.6 (34.3 to 138.1)
Southeast Asia, east Asia, and Oceania	Southeast Asia	Cambodia	Deaths	Age-standardized	73.5 (59.9 to 85.8)	49.7 (34.4 to 61.5)	108.1 (87 to 132.1)	53.8 (42.7 to 62.1)	36.1 (25.3 to 44.4)	82.9 (66.4 to 97.3)	-26.9 (-41.3 to -8.9)	-27.3 (-43.4 to 2.5)	-23.3 (-43.3 to 1.8)
Southeast Asia, east Asia, and Oceania	Southeast Asia	Cambodia	DALYs	All Ages	96,700 (81,427 to 111,358)	40,674 (29,188 to 50,446)	56,026 (47,010 to 65,660)	150,283 (125,987 to 172,225)	61,228 (50,845 to 71,903)	89,055 (73,385 to 103,736)	55.4 (28 to 90.3)	50.5 (18.2 to 110.8)	59 (26 to 99.8)
Southeast Asia, east Asia, and Oceania	Southeast Asia	Cambodia	DALYs	Age-standardized	1732.9 (1475.9 to 1972.2)	1247.5 (932.1 to 1496.1)	2391.6 (2012 to 2801.3)	1264.6 (1062.2 to 1439.3)	896.3 (731.8 to 1045.8)	1821.2 (1512.6 to 2102.5)	-27 (-39.2 to -11.5)	-28.1 (-41.5 to -4.7)	-23.9 (-40 to -3.4)
Southeast Asia, east Asia, and Oceania	Southeast Asia	Cambodia	YLLs	All Ages	76,790 (61,903 to 91,025)	30,938 (19,555 to 40,440)	45,852 (37,557 to 55,403)	103,455 (81,615 to 123,103)	39,626 (29,252 to 48,995)	63,829 (49,689 to 76,311)	34.7 (2.7 to 77)	28.1 (-9.6 to 105.5)	39.2 (1.8 to 86.3)
Southeast Asia, east Asia, and Oceania	Southeast Asia	Cambodia	YLLs	Age-standardized	1456.4 (1201.1 to 1691.7)	1005.5 (694.8 to 1250)	2068.8 (1687 to 2467.6)	924.1 (734.6 to 1083.4)	610.5 (447.5 to 746.1)	1401.5 (1104.3 to 1653.8)	-36.6 (-49.8 to -19.3)	-39.3 (-53.8 to -12.8)	-32.3 (-49.1 to -9.5)
Southeast Asia, east Asia, and Oceania	Southeast Asia	Cambodia	YLDs	All Ages	19,910 (15,081 to 25,471)	9,736 (7,427 to 12,392)	10,174 (7,639 to 13,056)	46,828 (36,819 to 56,850)	21,602 (16,902 to 26,772)	25,226 (19,620 to 30,838)	135.2 (117.6 to 154.1)	121.9 (102.9 to 142.2)	147.9 (126 to 171.9)
Southeast Asia, east Asia, and Oceania	Southeast Asia	Cambodia	YLDs	Age-standardized	276.5 (218.4 to 333.5)	242 (191.3 to 294.3)	322.7 (253.3 to 391.2)	340.5 (269.8 to 406.7)	285.8 (225.3 to 350)	419.7 (332.7 to 499.5)	23.1 (17.5 to 29.2)	18.1 (11.4 to 25.6)	30 (22.3 to 37.8)
Southeast Asia, east Asia, and Oceania	Southeast Asia	Indonesia	Incidence	All Ages	1,449,028 (1,223,191 to 1,769,661)	648,515 (550,828 to 781,106)	800,513 (669,970 to 987,711)	1,963,624 (1,707,921 to 2,282,282	820,903 (720,419 to 948,646)	1,142,721 (989,694 to 1,337,129)	35.5 (27.3 to 44)	26.6 (18.7 to 34.7)	42.7 (34 to 52.1)
Southeast Asia, east Asia, and Oceania	Southeast Asia	Indonesia	Incidence	Age-standardized	863.1 (757.8 to 1001.5)	778.3 (683.3 to 901.2)	949.4 (829 to 1102.5)	838.1 (730.3 to 981.2)	699 (610.2 to 812.5)	982.1 (850.1 to 1157.3)	-2.9 (-5.8 to 0.3)	-10.2 (-13.2 to -6.9)	3.4 (0.1 to 7.1)
Southeast Asia, east Asia, and Oceania	Southeast Asia	Indonesia	Prevalence	All Ages	8,938,238 (7,876,436 to 10,342,889)	4,032,081 (3,578,634 to 4,628,073	4,906,158 (4,312,992 to 5,721,607)	13,362,704 (12,086,913 to 14,894,510	5,547,451 (5,013,336 to 6,173,541)	7,815,253 (7,045,833 to 8,784,186)	49.5 (42.3 to 56.6)	37.6 (31 to 44.4)	59.3 (51.1 to 67.6)
Southeast Asia, east Asia, and Oceania	Southeast Asia	Indonesia	Prevalence	Age-standardized	5907.2 (5357.8 to 6577.2)	5299.9 (4805.4 to 5896)	6551.2 (5948.2 to 7285.6)	5558.4 (5043.4 to 6182.6)	4549.6 (4114.9 to 5058.3)	6646.9 (6024.8 to 7405.1)	-5.9 (-8.1 to -3.2)	-14.2 (-16.5 to -11.6)	1.5 (-1.2 to 4.5)
Southeast Asia, east Asia, and Oceania	Southeast Asia	Indonesia	Deaths	All Ages	65,596 (56,281 to 74,089)	27,054 (20,944 to 34,069)	38,542 (31,498 to 44,541)	100,930 (85,012 to 113,831)	35,907 (28,046 to 44,018)	65,023 (52,995 to 77,307)	53.9 (23 to 89.9)	32.7 (-5 to 77.6)	68.7 (29.1 to 126.4)
Southeast Asia, east Asia, and Oceania	Southeast Asia	Indonesia	Deaths	Age-standardized	83.9 (71.3 to 96.8)	63.3 (48 to 83.2)	108.9 (88.9 to 126.5)	61.9 (52.5 to 70.2)	40.5 (31.7 to 49.7)	90.1 (74.4 to 105.2)	-26.1 (-40.7 to -9.4)	-36 (-55.7 to -11.5)	-17.3 (-35.7 to 9.3)
Southeast Asia, east Asia, and Oceania	Southeast Asia	Indonesia	DALYs	All Ages	2,335,848 (2,062,938 to 2,573,683)	1,033,819 (867,666 to 1,182,546)	1,302,029 (1,107,514 to 1,473,672)	3,181,366 (2,768,328 to 3,576,092	1,187,689 (990,804 to 1,393,998)	1,993,677 (1,658,437 to 2,342,775)	36.2 (17.6 to 56.2)	14.9 (-3.3 to 36.7)	53.1 (24.9 to 93.4)
Southeast Asia, east Asia, and Oceania	Southeast Asia	Indonesia	DALYs	Age-standardized	2058.9 (1812 to 2280.7)	1683.6 (1390.5 to 1995.8)	2491.7 (2103.6 to 2831)	1512.6 (1319.4 to 1687.8)	1069.3 (895.4 to 1248.6)	2041.2 (1720.9 to 2368.2)	-26.5 (-37.5 to -14.3)	-36.5 (-49.2 to -22)	-18.1 (-33.3 to 3.1)
Southeast Asia, east Asia, and Oceania	Southeast Asia	Indonesia	YLLs	All Ages	1,784,571 (1,551,833 to 1,980,738)	780,295 (617,391 to 907,481)	1,004,276 (820,268 to 1,166,589)	2,241,005 (1,871,784 to 2,573,321	792,200 (599,352 to 990,145)	1,448,804 (1,167,550 to 1,769,661)	25.6 (2.1 to 52.6)	1.5 (-21.6 to 30.1)	44.3 (9.1 to 97.2)
Southeast Asia, east Asia, and Oceania	Southeast Asia	Indonesia	YLLs	Age-standardized	1655.8 (1429.6 to 1859)	1320.1 (1042.5 to 1620.1)	2045.6 (1681.7 to 2358.9)	1113 (936.3 to 1258.1)	742.2 (572.8 to 911.4)	1559.7 (1272.9 to 1857.4)	-32.8 (-45.6 to -18.3)	-43.8 (-58.8 to -26.3)	-23.8 (-41.3 to 2)
Southeast Asia, east Asia, and Oceania	Southeast Asia	Indonesia	YLDs	All Ages	551,277 (416,569 to 705,503)	253,524 (193,903 to 319,686)	297,753 (224,254 to 386,939)	940,362 (733,759 to 1,159,385)	395,489 (310,639 to 487,425)	544,872 (423,262 to 678,855)	70.6 (61.2 to 80.5)	56 (47.8 to 64.9)	83 (71.8 to 94.6)
Southeast Asia, east Asia, and Oceania	Southeast Asia	Indonesia	YLDs	Age-standardized	403.1 (314.5 to 495.6)	363.5 (286.3 to 439.3)	446.1 (346.8 to 551.2)	399.7 (313.6 to 486.9)	327.2 (258.1 to 400)	481.5 (378.9 to 591.7)	-0.8 (-3 to 1.5)	-10 (-12.4 to -7.4)	7.9 (5 to 11.1)
Southeast Asia, east Asia, and Oceania	Southeast Asia	Lao People's Democratic Republic	Incidence	All Ages	26,996 (22,539 to 32,452)	11,710 (9,916 to 13,876)	15,286 (12,525 to 18,690)	41,702 (36,567 to 48,145)	18,847 (16,662 to 21,444)	22,855 (19,658 to 27,023)	54.5 (41.9 to 67.3)	60.9 (49.2 to 74.6)	49.5 (34 to 65.8)
Southeast Asia, east Asia, and Oceania	Southeast Asia	Lao People's Democratic Republic	Incidence	Age-standardized	677.9 (600.3 to 770.6)	602.4 (540.9 to 676)	754.4 (658.5 to 866.1)	683.4 (609.1 to 769.1)	618.7 (554.5 to 691.4)	749.3 (664.5 to 857.7)	0.8 (-4 to 5.5)	2.7 (-2.1 to 7.9)	-0.7 (-7.1 to 5.7)
Southeast Asia, east Asia, and Oceania	Southeast Asia	Lao People's Democratic Republic	Prevalence	All Ages	153,339 (136,738 to 175,615)	69,199 (61,860 to 78,245)	84,140 (73,921 to 97,823)	269,773 (246,910 to 297,242)	124,612 (113,953 to 135,661)	145,161 (132,081 to 163,226)	75.9 (64.9 to 87.7)	80.1 (68.3 to 93.3)	72.5 (59.2 to 87.1)
Southeast Asia, east Asia, and Oceania	Southeast Asia	Lao People's Democratic Republic	Prevalence	Age-standardized	4561.9 (4219.7 to 4964.1)	4154.2 (3827 to 4501.5)	4994.5 (4593.6 to 5456)	4698.3 (4377.1 to 5068)	4305.4 (3997.2 to 4613.8)	5118.6 (4755 to 5585.3)	3 (-0.7 to 6.8)	3.6 (-1 to 8.7)	2.5 (-2.8 to 7.8)
Southeast Asia, east Asia, and Oceania	Southeast Asia	Lao People's Democratic Republic	Deaths	All Ages	2,563 (1,993 to 3,123)	1,026 (714 to 1,352)	1,537 (1,128 to 1,921)	2,663 (2,092 to 3,360)	970 (702 to 1,422)	1,693 (1,265 to 2,170)	3.9 (-20.7 to 36.6)	-5.5 (-37.6 to 55.4)	10.2 (-17.6 to 45.2)
Southeast Asia, east Asia, and Oceania	Southeast Asia	Lao People's Democratic Republic	Deaths	Age-standardized	135.1 (106.1 to 165.8)	98.9 (69.3 to 134.4)	183.3 (136.5 to 228.1)	76.7 (61.3 to 96.1)	51.5 (37.4 to 76.8)	107.7 (81.7 to 135)	-43.2 (-55.3 to -27.4)	-47.9 (-64.3 to -22)	-41.2 (-56 to -22.5)
Southeast Asia, east Asia, and Oceania	Southeast Asia	Lao People's Democratic Republic	DALYs	All Ages	91,298 (71,047 to 112,879)	39,532 (25,672 to 55,373)	51,765 (38,839 to 64,572)	84,579 (69,439 to 102,747)	34,043 (26,466 to 45,994)	50,536 (39,343 to 62,904)	-7.4 (-29.5 to 23.4)	-13.9 (-43.6 to 51.9)	-2.4 (-23.4 to 24)
Southeast Asia, east Asia, and Oceania	Southeast Asia	Lao People's Democratic Republic	DALYs	Age-standardized	3398.2 (2733.3 to 4076.6)	2691.1 (1950.3 to 3470)	4237.5 (3224.1 to 5182.9)	1830.9 (1507.1 to 2206.3)	1368.2 (1063.6 to 1851.3)	2360.2 (1851.3 to 2905.1)	-46.1 (-57.2 to -31.3)	-49.2 (-64 to -21.9)	-44.3 (-56.5 to -28.5)
Southeast Asia, east Asia, and Oceania	Southeast Asia	Lao People's Democratic Republic	YLLs	All Ages	81,152 (60,781 to 102,741)	34,757 (20,977 to 50,655)	46,395 (33,574 to 59,239)	63,412 (48,645 to 80,528)	23,962 (16,617 to 35,687)	39,450 (28,836 to 51,802)	-21.9 (-43.9 to 10.3)	-31.1 (-58.2 to 40.8)	-15 (-36.7 to 13.2)
Southeast Asia, east Asia, and Oceania	Southeast Asia	Lao People's Democratic Republic	YLLs	Age-standardized	3053.7 (2381.6 to 3733.9)	2371 (1621 to 3147.9)	3866.1 (2844.4 to 4804.1)	1440.6 (1124.8 to 1819.4)	1004.7 (718 to 1480.8)	1940 (1451.4 to 2485.6)	-52.8 (-64.4 to -37.4)	-57.6 (-72.5 to -27.8)	-49.8 (-62.5 to -33.8)
Southeast Asia, east Asia, and Oceania	Southeast Asia	Lao People's Democratic Republic	YLDs	All Ages	10,146 (7,846 to 12,757)	4,776 (3,710 to 5,961)	5,370 (4,100 to 6,809)	21,167 (16,742 to 25,630)	10,081 (7,969 to 12,224)	11,086 (8,663 to 13,544)	108.6 (94.3 to 124.1)	111.1 (96.8 to 128.2)	106.4 (90.5 to 124.5)
Southeast Asia, east Asia, and Oceania	Southeast Asia	Lao People's Democratic Republic	YLDs	Age-standardized	344.5 (273.2 to 414.7)	320 (253.4 to 387.2)	371.4 (292.9 to 449.6)	390.2 (313.1 to 464)	363.5 (287.8 to 433.1)	420.2 (333.6 to 500.2)	13.3 (8.6 to 18.7)	13.6 (7.6 to 20.4)	13.1 (7.2 to 19.5)
Southeast Asia, east Asia, and Oceania	Southeast Asia	Malaysia	Incidence	All Ages	117,793 (101,333 to 138,192)	56,157 (47,981 to 66,076)	61,637 (52,626 to 73,413)	205,263 (178,495 to 240,937)	99,579 (86,053 to 116,155)	105,685 (91,891 to 124,785)	74.3 (61.4 to 90.9)	77.3 (62.2 to 97.7)	71.5 (58.1 to 89.9)
Southeast Asia, east Asia, and Oceania	Southeast Asia	Malaysia	Incidence	Age-standardized	770.5 (694.5 to 866.9)	722.7 (643.4 to 821.1)	821 (736.2 to 925.3)	727.4 (628.9 to 859.7)	720.8 (620.4 to 846.3)	734.5 (635.2 to 867.8)	-5.6 (-13.1 to 3.4)	-0.3 (-9.3 to 10.9)	-10.5 (-17.8 to -0.3)
Southeast Asia, east Asia, and Oceania	Southeast Asia	Malaysia	Prevalence	All Ages	715,448 (645,834 to 808,815)	348,138 (312,114 to 396,652)	367,310 (331,864 to 417,350)	1,308,482 (1,172,107 to 1,490,477	614,648 (544,199 to 699,779)	693,834 (624,111 to 793,178)	82.9 (72.2 to 95)	76.6 (62.9 to 91.4)	88.9 (76.5 to 103)
Southeast Asia, east Asia, and Oceania	Southeast Asia	Malaysia	Prevalence	Age-standardized	5270.4 (4897.1 to 5721)	4976.7 (4565.4 to 5472.4)	5595.4 (5207.4 to 6060)	4547.9 (4071.5 to 5193.2)	4338.1 (3836.1 to 4951.8)	4769.5 (4298.2 to 5444.2)	-13.7 (-19.1 to -7.1)	-12.8 (-19.8 to -4.6)	-14.8 (-20.1 to -7.9)
Southeast Asia, east Asia, and Oceania	Southeast Asia	Malaysia	Deaths	All Ages	5,101 (4,196 to 6,029)	1,850 (1,341 to 2,221)	3,251 (2,633 to 3,996)	7,473 (5,862 to 10,018)	2,585 (1,922 to 3,952)	4,888 (3,778 to 6,422)	46.5 (4.9 to 121.3)	39.7 (-3.4 to 132.6)	50.4 (1.2 to 135.6)
Southeast Asia, east Asia, and Oceania	Southeast Asia	Malaysia	Deaths	Age-standardized	65.3 (53.9 to 78.2)	45.2 (33.1 to 55.1)	87.1 (68.1 to 107.6)	34.4 (27 to 46.2)	23.8 (17.6 to 37.3)	45.1 (35 to 59.3)	-47.4 (-62.8 to -19.9)	-47.2 (-63.6 to -11.4)	-48.2 (-65.4 to -18.6)
Southeast Asia, east Asia, and Oceania	Southeast Asia	Malaysia	DALYs	All Ages	159,246 (136,871 to 179,176)	63,420 (49,635 to 73,359)	95,826 (82,159 to 110,432)	243,721 (204,326 to 295,877)	92,208 (75,584 to 115,083)	151,513 (126,530 to 181,194)	53 (24.3 to 98.8)	45.4 (16.8 to 96.7)	58.1 (22.6 to 113.4)
Southeast Asia, east Asia, and Oceania	Southeast Asia	Malaysia	DALYs	Age-standardized	1589.8 (1359.5 to 1807.2)	1180.8 (931 to 1365.9)	2031.1 (1698.2 to 2386.3)	923.1 (779.3 to 1122.2)	699.1 (576.6 to 891.1)	1149.9 (956.7 to 1390.9)	-41.9 (-53.8 to -23.3)	-40.8 (-52.9 to -18)	-43.4 (-57.1 to -21.4)
Southeast Asia, east Asia, and Oceania	Southeast Asia	Malaysia	YLLs	All Ages	115,287 (94,339 to 132,576)	43,382 (31,049 to 51,437)	71,905 (59,076 to 86,164)	149,601 (116,332 to 200,342)	51,311 (37,960 to 74,828)	98,290 (75,311 to 127,644)	29.8 (-5 to 91.1)	18.3 (-16.8 to 91.9)	36.7 (-5.6 to 109)
Southeast Asia, east Asia, and Oceania	Southeast Asia	Malaysia	YLLs	Age-standardized	1228.8 (1010.9 to 1432.2)	871.5 (621.7 to 1045.4)	1614.3 (1318.1 to 1983.6)	589.5 (462.4 to 792)	405.8 (299.7 to 604.5)	775.6 (598.8 to 1011.5)	-52 (-65.5 to -28.8)	-53.4 (-67.7 to -24.3)	-52 (-67.2 to -25.7)
Southeast Asia, east Asia, and Oceania	Southeast Asia	Malaysia	YLDs	All Ages	43,959 (33,739 to 55,965)	20,038 (14,955 to 26,174)	23,921 (18,634 to 29,907)	94,121 (74,340 to 115,057)	40,897 (31,774 to 50,591)	53,223 (41,767 to 64,679)	114.1 (99.1 to 129.9)	104.1 (87.1 to 123.8)	122.5 (105.6 to 141.1)
Southeast Asia, east Asia, and Oceania	Southeast Asia	Malaysia	YLDs	Age-standardized	361 (285.9 to 439.5)	309.3 (237.2 to 388.6)	416.7 (332.9 to 500.8)	333.6 (263.7 to 404.8)	293.3 (228.8 to 362.2)	374.3 (295 to 450.9)	-7.6 (-12.4 to -2.3)	-5.2 (-11.9 to 2.7)	-10.2 (-15.5 to -4.5)
Southeast Asia, east Asia, and Oceania	Southeast Asia	Maldives	Incidence	All Ages	2,382 (1,960 to 2,933)	1,220 (989 to 1,514)	1,162 (957 to 1,430)	3,757 (3,249 to 4,364)	1,704 (1,479 to 1,966)	2,053 (1,747 to 2,404)	57.7 (37.3 to 79.3)	39.7 (20.3 to 62.7)	76.6 (51.8 to 104.9)
Southeast Asia, east Asia, and Oceania	Southeast Asia	Maldives	Incidence	Age-standardized	1168.6 (1031.8 to 1319.5)	1272.9 (1127.1 to 1438.2)	1084.9 (955.2 to 1239.1)	1015.9 (884.3 to 1167.5)	1046.4 (912.7 to 1188)	997.7 (844.9 to 1161)	-13.1 (-19.8 to -6.5)	-17.8 (-24.5 to -10.6)	-8 (-16.3 to 0.8)
Southeast Asia, east Asia, and Oceania	Southeast Asia	Maldives	Prevalence	All Ages	11,988 (10,622 to 13,721)	6,278 (5,509 to 7,170)	5,710 (5,004 to 6,668)	20,938 (18,752 to 23,797)	9,516 (8,695 to 10,685)	11,422 (9,866 to 13,533)	74.7 (55.8 to 95.9)	51.6 (35.7 to 67.3)	100 (72.7 to 130.6)
Southeast Asia, east Asia, and Oceania	Southeast Asia	Maldives	Prevalence	Age-standardized	6825.5 (6380.3 to 7348.6)	7820.8 (7340.4 to 8362.8)	6072.4 (5638.1 to 6631.7)	5428.1 (4905.9 to 6056.4)	5876 (5434.1 to 6441.7)	5070.1 (4448.6 to 5849.7)	-20.5 (-26.1 to -14.1)	-24.9 (-29.7 to -19.5)	-16.5 (-24.5 to -7.3)
Southeast Asia, east Asia, and Oceania	Southeast Asia	Maldives	Deaths	All Ages	96 (73 to 113)	48 (30 to 60)	48 (35 to 58)	123 (102 to 147)	60 (47 to 74)	63 (51 to 75)	27.8 (1 to 72.2)	25.6 (-8.2 to 94.7)	30 (-2.1 to 92.7)
Southeast Asia, east Asia, and Oceania	Southeast Asia	Maldives	Deaths	Age-standardized	143.3 (113.8 to 164.2)	157.2 (104.7 to 191.5)	131.4 (96.2 to 157.6)	51.6 (42.4 to 61.4)	53.7 (42 to 66.3)	49.7 (40.5 to 59.5)	-64 (-71.2 to -53)	-65.8 (-74.4 to -51.5)	-62.2 (-71 to -43.3)
Southeast Asia, east Asia, and Oceania	Southeast Asia	Maldives	DALYs	All Ages	3,766 (2,882 to 4,515)	2,071 (1,340 to 2,639)	1,695 (1,274 to 2,079)	3,932 (3,422 to 4,499)	1,948 (1,677 to 2,242)	1,984 (1,706 to 2,286)	4.4 (-13.7 to 37.5)	-5.9 (-28.8 to 44.9)	17 (-8.2 to 60)
Southeast Asia, east Asia, and Oceania	Southeast Asia	Maldives	DALYs	Age-standardized	3295.1 (2643.6 to 3753.1)	3896.7 (2695.1 to 4670.4)	2813.3 (2177.5 to 3321.5)	1251.6 (1093.2 to 1428.3)	1392.6 (1190.8 to 1603.2)	1137 (978.9 to 1312.7)	-62 (-68.2 to -51.8)	-64.3 (-71.6 to -49.9)	-59.6 (-67.5 to -45.1)
Southeast Asia, east Asia, and Oceania	Southeast Asia	Maldives	YLLs	All Ages	3,021 (2,140 to 3,715)	1,693 (991 to 2,275)	1,328 (919 to 1,705)	2,260 (1,891 to 2,679)	1,137 (911 to 1,382)	1,123 (923 to 1,344)	-25.2 (-43.1 to 6.1)	-32.8 (-53 to 15.6)	-15.4 (-39.4 to 30.8)
Southeast Asia, east Asia, and Oceania	Southeast Asia	Maldives	YLLs	Age-standardized	2766.9 (2130 to 3217.2)	3292.8 (2094.4 to 4061.4)	2336.8 (1680.5 to 2817.6)	793.8 (662.1 to 944)	867.4 (686.8 to 1058.1)	733.4 (597.7 to 879)	-71.3 (-77.2 to -61.5)	-73.7 (-80.5 to -60.3)	-68.6 (-76.4 to -53)
Southeast Asia, east Asia, and Oceania	Southeast Asia	Maldives	YLDs	All Ages	746 (567 to 952)	378 (286 to 493)	367 (278 to 471)	1,672 (1,325 to 2,006)	811 (648 to 971)	861 (669 to 1,068)	124.3 (100.2 to 149.5)	114.5 (87.4 to 144.5)	134.4 (108 to 163.3)
Southeast Asia, east Asia, and Oceania	Southeast Asia	Maldives	YLDs	Age-standardized	528.2 (423 to 630)	604 (484.8 to 721)	476.6 (376 to 571.8)	457.8 (365.3 to 542.4)	525.2 (420.4 to 621.1)	403.6 (319.5 to 489.1)	-13.3 (-18 to -8.8)	-13 (-18.4 to -7.5)	-15.3 (-21.6 to -8.8)
Southeast Asia, east Asia, and Oceania	Southeast Asia	Mauritius	Incidence	All Ages	9,185 (7,971 to 10,643)	4,415 (3,829 to 5,133)	4,770 (4,126 to 5,544)	12,231 (10,650 to 13,893)	6,038 (5,188 to 7,013)	6,193 (5,411 to 7,055)	33.2 (19.3 to 49)	36.8 (20.9 to 55.3)	29.8 (16.3 to 46.7)
Southeast Asia, east Asia, and Oceania	Southeast Asia	Mauritius	Incidence	Age-standardized	979.8 (861.5 to 1116.6)	906.8 (792.1 to 1040.7)	1066.2 (944.4 to 1206.4)	903.3 (782.3 to 1050.1)	853.6 (727.4 to 995)	960.3 (830.1 to 1111.2)	-7.8 (-13.8 to -1.4)	-5.9 (-13.2 to 2.3)	-9.9 (-16 to -3.1)
Southeast Asia, east Asia, and Oceania	Southeast Asia	Mauritius	Prevalence	All Ages	57,811 (52,399 to 64,574)	27,767 (24,945 to 31,287)	30,044 (27,073 to 33,600)	64,907 (60,489 to 70,625)	29,504 (27,237 to 32,326)	35,403 (32,931 to 38,484)	12.3 (5.7 to 19.2)	6.3 (-1.3 to 14.5)	17.8 (10.3 to 25.6)
Southeast Asia, east Asia, and Oceania	Southeast Asia	Mauritius	Prevalence	Age-standardized	6194.4 (5712.7 to 6808.5)	5649.2 (5152.7 to 6260.9)	6926.1 (6394.5 to 7546)	4669.3 (4232.4 to 5270.2)	4179.7 (3744.3 to 4768.3)	5290.8 (4823.9 to 5899.1)	-24.6 (-28.8 to -20.3)	-26 (-31.5 to -21)	-23.6 (-28 to -18.7)
Southeast Asia, east Asia, and Oceania	Southeast Asia	Mauritius	Deaths	All Ages	450 (398 to 482)	173 (126 to 192)	277 (257 to 298)	537 (429 to 678)	232 (177 to 336)	305 (245 to 375)	19.4 (-4.6 to 55.6)	34.5 (0.5 to 117.9)	10 (-12.6 to 36.7)
Southeast Asia, east Asia, and Oceania	Southeast Asia	Mauritius	Deaths	Age-standardized	73.8 (64.5 to 79.5)	50.2 (36.5 to 56.3)	108.6 (99.2 to 117)	34.9 (28.1 to 44.2)	25.8 (19.6 to 37.5)	48 (39 to 58.6)	-52.7 (-62.1 to -38.2)	-48.6 (-61.6 to -16.5)	-55.8 (-64.7 to -45.4)
Southeast Asia, east Asia, and Oceania	Southeast Asia	Mauritius	DALYs	All Ages	14,010 (12,688 to 15,205)	5,458 (4,445 to 6,088)	8,552 (7,893 to 9,235)	14,833 (12,562 to 17,740)	6,035 (4,961 to 7,891)	8,799 (7,414 to 10,419)	5.9 (-9.8 to 28.1)	10.6 (-9.7 to 54.3)	2.9 (-13.6 to 22.3)
Southeast Asia, east Asia, and Oceania	Southeast Asia	Mauritius	DALYs	Age-standardized	1800 (1627.8 to 1940.3)	1270.8 (1026.2 to 1408.4)	2498.5 (2322.7 to 2683.5)	959.5 (811.3 to 1145.4)	740.1 (610.7 to 965.3)	1241.3 (1057.8 to 1467.3)	-46.7 (-54.5 to -35.7)	-41.8 (-52.2 to -18.5)	-50.3 (-58.1 to -41.5)
Southeast Asia, east Asia, and Oceania	Southeast Asia	Mauritius	YLLs	All Ages	10,371 (9,284 to 11,074)	3,801 (2,819 to 4,220)	6,570 (6,109 to 7,063)	9,838 (7,807 to 12,547)	3,942 (3,001 to 5,836)	5,896 (4,621 to 7,365)	-5.1 (-25.6 to 26.3)	3.7 (-23.5 to 68.6)	-10.3 (-30.4 to 14.7)
Southeast Asia, east Asia, and Oceania	Southeast Asia	Mauritius	YLLs	Age-standardized	1390.5 (1234.7 to 1486.5)	922.9 (681.1 to 1021.6)	2007.3 (1865.6 to 2150.7)	621.1 (496.5 to 786.4)	461.1 (352.5 to 679.2)	828.9 (658.5 to 1022.5)	-55.3 (-64.9 to -41)	-50 (-63.1 to -18)	-58.7 (-67.5 to -48)
Southeast Asia, east Asia, and Oceania	Southeast Asia	Mauritius	YLDs	All Ages	3,639 (2,804 to 4,568)	1,657 (1,263 to 2,112)	1,982 (1,539 to 2,462)	4,995 (4,008 to 5,977)	2,093 (1,655 to 2,544)	2,903 (2,318 to 3,438)	37.3 (26.3 to 49.8)	26.3 (15.4 to 39)	46.4 (33.5 to 60.9)
Southeast Asia, east Asia, and Oceania	Southeast Asia	Mauritius	YLDs	Age-standardized	409.5 (320.4 to 501.3)	347.9 (268.4 to 438.6)	491.3 (388.4 to 597.3)	338.4 (267.6 to 407.9)	279 (217.4 to 344.8)	412.4 (328.3 to 493.6)	-17.4 (-21.4 to -13.1)	-19.8 (-25.2 to -14.1)	-16.1 (-21 to -10.9)
Southeast Asia, east Asia, and Oceania	Southeast Asia	Myanmar	Incidence	All Ages	260,467 (229,703 to 297,564)	117,855 (104,751 to 133,696)	142,612 (124,153 to 164,865)	411,484 (374,414 to 453,399)	205,722 (186,341 to 225,014)	205,762 (186,073 to 230,223)	58 (48.1 to 68.2)	74.6 (63.3 to 86.4)	44.3 (34.3 to 56)
Southeast Asia, east Asia, and Oceania	Southeast Asia	Myanmar	Incidence	Age-standardized	787 (720 to 861.8)	715.8 (659 to 779.5)	861 (782.4 to 945.3)	836.8 (762.8 to 919)	776.2 (704.4 to 850)	898.9 (822.2 to 994.5)	6.3 (2.4 to 10.4)	8.4 (3.4 to 13.5)	4.4 (-0.6 to 9.7)
Southeast Asia, east Asia, and Oceania	Southeast Asia	Myanmar	Prevalence	All Ages	1,605,918 (1,484,324 to 1,756,972)	745,299 (690,194 to 805,073)	860,620 (785,382 to 956,610)	2,708,393 (2,569,011 to 2,862,584	1,379,358 (1,302,124 to 1,459,322)	1,329,035 (1,254,805 to 1,422,944)	68.7 (61.2 to 75.6)	85.1 (75.3 to 94)	54.4 (45.8 to 62.7)
Southeast Asia, east Asia, and Oceania	Southeast Asia	Myanmar	Prevalence	Age-standardized	5384.5 (5075.5 to 5701.6)	4942.9 (4638.8 to 5229.9)	5857.4 (5513.8 to 6255.6)	5601.8 (5329.8 to 5892.3)	5231 (4939.2 to 5541.7)	6001.8 (5716.2 to 6364.7)	4 (1.1 to 6.9)	5.8 (1.6 to 10.1)	2.5 (-1.5 to 6.7)
Southeast Asia, east Asia, and Oceania	Southeast Asia	Myanmar	Deaths	All Ages	37,654 (27,630 to 46,073)	15,871 (8,254 to 21,075)	21,783 (17,101 to 27,178)	43,284 (33,261 to 50,185)	17,734 (9,791 to 21,291)	25,551 (21,866 to 29,575)	15 (-8.2 to 44.6)	11.7 (-16.1 to 56)	17.3 (-9.3 to 52.6)
Southeast Asia, east Asia, and Oceania	Southeast Asia	Myanmar	Deaths	Age-standardized	193.6 (139.3 to 233.1)	149.3 (75.6 to 194.7)	252.4 (201.7 to 316.7)	115.6 (88.1 to 133.3)	80.8 (44.6 to 96.6)	168.7 (144.1 to 192.2)	-40.3 (-51.3 to -26.3)	-45.9 (-58.3 to -25.5)	-33.2 (-47.3 to -16.4)
Southeast Asia, east Asia, and Oceania	Southeast Asia	Myanmar	DALYs	All Ages	1,126,818 (893,021 to 1,359,951)	486,026 (281,126 to 642,596)	640,792 (520,507 to 784,728)	1,120,191 (921,733 to 1,292,963)	474,040 (323,433 to 562,596)	646,151 (554,614 to 750,636)	-0.6 (-21.5 to 25.4)	-2.5 (-27.6 to 39.2)	0.8 (-21.1 to 29.6)
Southeast Asia, east Asia, and Oceania	Southeast Asia	Myanmar	DALYs	Age-standardized	4420.3 (3432.3 to 5345.8)	3536.5 (2061.8 to 4590.3)	5494.9 (4468.3 to 6729.2)	2535.2 (2080.6 to 2902.6)	1896.7 (1282.6 to 2224.7)	3421.5 (2959.5 to 3900.9)	-42.6 (-53.6 to -28.8)	-46.4 (-58.7 to -27)	-37.7 (-50.9 to -21.4)
Southeast Asia, east Asia, and Oceania	Southeast Asia	Myanmar	YLLs	All Ages	1,002,639 (768,365 to 1,234,274)	425,835 (220,206 to 585,047)	576,804 (453,460 to 722,313)	879,333 (684,487 to 1,048,079)	346,713 (199,021 to 432,920)	532,620 (448,099 to 633,883)	-12.3 (-33.2 to 15.3)	-18.6 (-44.1 to 22.3)	-7.7 (-30.4 to 23.5)
Southeast Asia, east Asia, and Oceania	Southeast Asia	Myanmar	YLLs	Age-standardized	3962.2 (2945.3 to 4880.4)	3106.3 (1635.7 to 4143.5)	5005.7 (3940.2 to 6214.1)	2026.4 (1582.7 to 2381.9)	1409.4 (795.9 to 1734.8)	2888.5 (2469.3 to 3367.2)	-48.9 (-59.9 to -35)	-54.6 (-66.8 to -34.8)	-42.3 (-55.6 to -24.8)
Southeast Asia, east Asia, and Oceania	Southeast Asia	Myanmar	YLDs	All Ages	124,179 (99,179 to 147,761)	60,191 (48,229 to 71,588)	63,988 (50,831 to 77,274)	240,859 (195,381 to 281,944)	127,328 (102,991 to 148,826)	113,531 (91,292 to 133,950)	94 (84.6 to 104.2)	111.5 (98.2 to 125.6)	77.4 (66.4 to 89.2)
Southeast Asia, east Asia, and Oceania	Southeast Asia	Myanmar	YLDs	Age-standardized	458.1 (370.5 to 531.2)	430.2 (346.2 to 505.5)	489.2 (394.4 to 575)	508.8 (414.9 to 588.2)	487.3 (392.6 to 568.1)	533.1 (431.3 to 623.2)	11.1 (7.4 to 15.2)	13.3 (7.8 to 19.4)	9 (4.2 to 14.4)
Southeast Asia, east Asia, and Oceania	Southeast Asia	Philippines	Incidence	All Ages	780,984 (619,621 to 1,010,027)	371,114 (293,317 to 482,226)	409,870 (324,968 to 530,334)	1,093,088 (907,810 to 1,356,001)	502,713 (416,891 to 623,045)	590,376 (487,192 to 733,105)	40 (32.6 to 48.7)	35.5 (27.4 to 44.8)	44 (36.8 to 53)
Southeast Asia, east Asia, and Oceania	Southeast Asia	Philippines	Incidence	Age-standardized	1132.4 (948.6 to 1385.5)	1088.3 (906 to 1339.6)	1176.5 (980.3 to 1434.8)	994 (842.3 to 1200.1)	912.6 (771.8 to 1109.9)	1084.1 (923.6 to 1300.6)	-12.2 (-14.5 to -9.5)	-16.1 (-18.8 to -13.4)	-7.9 (-10.4 to -4.5)
Southeast Asia, east Asia, and Oceania	Southeast Asia	Philippines	Prevalence	All Ages	4,481,879 (3,807,242 to 5,433,310)	2,140,840 (1,811,856 to 2,599,967	2,341,039 (1,997,118 to 2,827,511)	6,895,160 (6,080,864 to 7,970,472	3,195,430 (2,805,453 to 3,710,307)	3,699,730 (3,264,314 to 4,271,344)	53.8 (46.4 to 62.1)	49.3 (41.5 to 57.7)	58 (50.5 to 66.5)
Southeast Asia, east Asia, and Oceania	Southeast Asia	Philippines	Prevalence	Age-standardized	7578 (6744.8 to 8734.5)	7138.4 (6295.8 to 8308.1)	8049.7 (7202.5 to 9190.6)	6704.6 (6024.8 to 7613.3)	6081.3 (5391.5 to 6944.3)	7484 (6748 to 8382.9)	-11.5 (-13.5 to -9.4)	-14.8 (-17 to -12.8)	-7 (-9.7 to -4.1)
Southeast Asia, east Asia, and Oceania	Southeast Asia	Philippines	Deaths	All Ages	20,035 (17,366 to 22,256)	7,753 (6,262 to 8,986)	12,282 (10,574 to 14,009)	33,229 (27,252 to 41,083)	11,129 (8,579 to 16,232)	22,100 (17,234 to 28,263)	65.9 (31 to 113.1)	43.6 (5.8 to 115.9)	79.9 (34.2 to 137.8)
Southeast Asia, east Asia, and Oceania	Southeast Asia	Philippines	Deaths	Age-standardized	83 (72.7 to 93.8)	62.9 (52 to 75.2)	105.2 (91.2 to 120.7)	50.8 (41.7 to 64.7)	31.7 (24.4 to 48.9)	77 (60.3 to 97.7)	-38.7 (-51.4 to -20.4)	-49.6 (-63.3 to -24.1)	-26.8 (-45 to -2.4)
Southeast Asia, east Asia, and Oceania	Southeast Asia	Philippines	DALYs	All Ages	822,278 (722,434 to 929,023)	341,421 (282,991 to 398,188)	480,857 (422,930 to 541,148)	1,304,851 (1,126,317 to 1,507,925	470,380 (394,235 to 567,142)	834,472 (692,363 to 1,006,377)	58.7 (39.2 to 81.6)	37.8 (16.5 to 71.6)	73.5 (46.1 to 107.7)
Southeast Asia, east Asia, and Oceania	Southeast Asia	Philippines	DALYs	Age-standardized	2083.8 (1858.2 to 2306.8)	1616.3 (1359.1 to 1842.2)	2594.2 (2295.9 to 2892.7)	1526.1 (1326.5 to 1767.3)	1020.2 (856.2 to 1258.7)	2159.6 (1793.8 to 2583.4)	-26.8 (-36.9 to -12.9)	-36.9 (-47.2 to -20.4)	-16.8 (-32.2 to 1.9)
Southeast Asia, east Asia, and Oceania	Southeast Asia	Philippines	YLLs	All Ages	568,315 (498,163 to 623,434)	227,920 (180,298 to 260,608)	340,395 (295,918 to 381,663)	822,654 (680,506 to 982,930)	274,431 (214,170 to 355,699)	548,223 (428,702 to 691,330)	44.8 (17.3 to 77.8)	20.4 (-8.7 to 69)	61.1 (23 to 108.5)
Southeast Asia, east Asia, and Oceania	Southeast Asia	Philippines	YLLs	Age-standardized	1587.9 (1387.7 to 1755.4)	1194.4 (960.5 to 1367.8)	2019.2 (1740.9 to 2301)	1021.2 (840.6 to 1231.8)	631.1 (491.9 to 859.1)	1514.1 (1185.1 to 1914.7)	-35.7 (-48.9 to -17.9)	-47.2 (-60.1 to -25.8)	-25 (-44.1 to -0.9)
Southeast Asia, east Asia, and Oceania	Southeast Asia	Philippines	YLDs	All Ages	253,963 (184,901 to 340,847)	113,501 (81,239 to 154,663)	140,462 (104,431 to 184,807)	482,197 (373,132 to 600,679)	195,949 (147,463 to 251,501)	286,249 (222,910 to 351,254)	89.9 (75.1 to 108.2)	72.6 (60.4 to 89.2)	103.8 (86.3 to 124.6)
Southeast Asia, east Asia, and Oceania	Southeast Asia	Philippines	YLDs	Age-standardized	495.9 (382.3 to 622.8)	421.9 (318 to 541.7)	575.1 (448.9 to 712.3)	504.9 (398.6 to 609.5)	389.1 (295.1 to 489.3)	645.5 (514 to 773.9)	1.8 (-2.7 to 7.1)	-7.8 (-11.5 to -2.8)	12.3 (5.7 to 19.6)
Southeast Asia, east Asia, and Oceania	Southeast Asia	Seychelles	Incidence	All Ages	439 (374 to 524)	198 (167 to 237)	242 (204 to 290)	658 (578 to 759)	278 (242 to 325)	380 (335 to 439)	49.7 (40.7 to 59.9)	40.7 (31.7 to 50.6)	57.1 (44.3 to 69.4)
Southeast Asia, east Asia, and Oceania	Southeast Asia	Seychelles	Incidence	Age-standardized	623.5 (543.2 to 727.3)	537 (463.9 to 633.3)	720.3 (632.8 to 837.9)	682.5 (587.1 to 808.4)	589.9 (497.8 to 709.8)	777.8 (673.7 to 917.5)	9.5 (5.4 to 13.7)	9.9 (4.6 to 16.4)	8 (2.9 to 13.2)
Southeast Asia, east Asia, and Oceania	Southeast Asia	Seychelles	Prevalence	All Ages	2,814 (2,516 to 3,211)	1,221 (1,088 to 1,395)	1,593 (1,420 to 1,810)	4,626 (4,257 to 5,106)	1,840 (1,669 to 2,047)	2,786 (2,557 to 3,060)	64.4 (56.1 to 72.9)	50.7 (42.1 to 60.3)	74.9 (64.2 to 85.7)
Southeast Asia, east Asia, and Oceania	Southeast Asia	Seychelles	Prevalence	Age-standardized	4224 (3844.3 to 4705)	3474.4 (3142.6 to 3912.2)	5140.5 (4705.8 to 5685.1)	4499.3 (4070.7 to 5065.5)	3672.2 (3285.1 to 4195.2)	5408.3 (4921 to 6068.6)	6.5 (2.7 to 10.3)	5.7 (0.5 to 11.3)	5.2 (0.2 to 10.1)
Southeast Asia, east Asia, and Oceania	Southeast Asia	Seychelles	Deaths	All Ages	26 (23 to 29)	10 (8 to 11)	17 (14 to 19)	31 (26 to 35)	12 (9 to 15)	18 (16 to 22)	16.1 (0.9 to 34.5)	23.3 (-1.7 to 50.6)	11.8 (-5.7 to 34.5)
Southeast Asia, east Asia, and Oceania	Southeast Asia	Seychelles	Deaths	Age-standardized	46.9 (40.4 to 51.4)	28.3 (22.9 to 33)	77 (64.3 to 85.9)	32.7 (28 to 37.2)	22.3 (16.9 to 27)	47.5 (39.9 to 55.6)	-30.3 (-39.2 to -19.3)	-21.1 (-36.8 to -3.6)	-38.3 (-47.8 to -26.2)
Southeast Asia, east Asia, and Oceania	Southeast Asia	Seychelles	DALYs	All Ages	702 (621 to 771)	256 (220 to 289)	447 (387 to 498)	950 (841 to 1,061)	339 (286 to 387)	611 (533 to 694)	35.3 (22.7 to 50)	32.5 (16.4 to 51.5)	36.9 (20.6 to 56.8)
Southeast Asia, east Asia, and Oceania	Southeast Asia	Seychelles	DALYs	Age-standardized	1182.9 (1041.8 to 1290.3)	760.5 (654.6 to 855.4)	1761.5 (1525 to 1948.3)	909.8 (805 to 1017.7)	632.5 (532.2 to 728)	1237.3 (1083.7 to 1403.6)	-23.1 (-30.4 to -14.5)	-16.8 (-26.9 to -5.3)	-29.8 (-38.2 to -19.4)
Southeast Asia, east Asia, and Oceania	Southeast Asia	Seychelles	YLLs	All Ages	509 (440 to 555)	173 (144 to 199)	335 (281 to 376)	589 (510 to 673)	201 (156 to 245)	387 (326 to 456)	15.8 (0.1 to 34.4)	16.1 (-7.3 to 43)	15.6 (-4.4 to 40.3)
Southeast Asia, east Asia, and Oceania	Southeast Asia	Seychelles	YLLs	Age-standardized	879.8 (759 to 961.1)	521.1 (430.2 to 594.8)	1378.5 (1150 to 1545.8)	569 (494.5 to 650.9)	367.6 (286.4 to 445.1)	811.6 (687.8 to 955.7)	-35.3 (-44 to -25.1)	-29.5 (-43.7 to -13.2)	-41.1 (-50.6 to -29.3)
Southeast Asia, east Asia, and Oceania	Southeast Asia	Seychelles	YLDs	All Ages	194 (152 to 239)	82 (64 to 103)	112 (87 to 137)	361 (287 to 432)	137 (109 to 167)	224 (177 to 269)	86.5 (75.3 to 97.5)	67.2 (56.2 to 79.2)	100.8 (86.6 to 115.7)
Southeast Asia, east Asia, and Oceania	Southeast Asia	Seychelles	YLDs	Age-standardized	303.2 (240.3 to 369.1)	239.5 (188.2 to 297)	383 (304.3 to 461.5)	340.8 (270.7 to 410.8)	264.9 (209.5 to 326.8)	425.7 (336.9 to 508.6)	12.4 (8 to 17.1)	10.6 (4.8 to 17.2)	11.2 (5.6 to 17.5)
Southeast Asia, east Asia, and Oceania	Southeast Asia	Sri Lanka	Incidence	All Ages	113,383 (98,446 to 131,604)	52,825 (45,616 to 61,707)	60,558 (52,616 to 70,184)	193,526 (174,046 to 214,902)	91,161 (81,237 to 102,132)	102,365 (92,170 to 114,564)	70.7 (58.5 to 83.6)	72.6 (59.3 to 87.1)	69 (55.9 to 83.4)
Southeast Asia, east Asia, and Oceania	Southeast Asia	Sri Lanka	Incidence	Age-standardized	761.3 (677.4 to 859.7)	707.8 (626.5 to 802.5)	814.2 (723.4 to 924.7)	863.6 (767.9 to 984.6)	772.8 (680.3 to 892.6)	972.6 (869.7 to 1103.4)	13.4 (8.5 to 19.1)	9.2 (3.1 to 16.8)	19.5 (12.6 to 26.2)
Southeast Asia, east Asia, and Oceania	Southeast Asia	Sri Lanka	Prevalence	All Ages	741,571 (676,070 to 823,963)	344,006 (311,266 to 382,711)	397,566 (360,340 to 439,439)	1,281,126 (1,186,836 to 1,392,201	598,630 (549,302 to 651,791)	682,496 (632,270 to 740,989)	72.8 (64.7 to 80.9)	74 (64.1 to 84.7)	71.7 (62.3 to 81.9)
Southeast Asia, east Asia, and Oceania	Southeast Asia	Sri Lanka	Prevalence	Age-standardized	5281.2 (4879.6 to 5726.3)	4835.8 (4452.1 to 5271.7)	5721.9 (5285.7 to 6180.5)	5513.1 (5056.6 to 6072.3)	4846 (4392.3 to 5395.4)	6352.6 (5863.6 to 6939.2)	4.4 (-0.1 to 9.4)	0.2 (-5 to 6.2)	11 (5.4 to 17.2)
Southeast Asia, east Asia, and Oceania	Southeast Asia	Sri Lanka	Deaths	All Ages	9,542 (8,298 to 10,391)	3,800 (2,950 to 4,371)	5,741 (5,062 to 6,342)	13,597 (9,536 to 17,634)	6,231 (3,882 to 8,374)	7,366 (5,154 to 9,697)	42.5 (3.6 to 88.7)	64 (3.3 to 125.3)	28.3 (-10.1 to 72.9)
Southeast Asia, east Asia, and Oceania	Southeast Asia	Sri Lanka	Deaths	Age-standardized	114 (99.1 to 124.2)	93.2 (71.6 to 107)	134.8 (119 to 149.1)	65.3 (46.3 to 83.6)	52 (31.9 to 69.8)	84.5 (59.8 to 109.4)	-42.7 (-58.1 to -25)	-44.2 (-65 to -23.7)	-37.3 (-56.1 to -16.6)
Southeast Asia, east Asia, and Oceania	Southeast Asia	Sri Lanka	DALYs	All Ages	257,721 (227,928 to 279,938)	103,903 (86,079 to 118,158)	153,818 (136,037 to 168,749)	327,289 (255,635 to 405,215)	139,357 (103,178 to 174,197)	187,932 (146,000 to 237,752)	27 (0.7 to 57.3)	34.1 (-1.1 to 71.2)	22.2 (-5.3 to 56.5)
Southeast Asia, east Asia, and Oceania	Southeast Asia	Sri Lanka	DALYs	Age-standardized	2338.1 (2057.3 to 2527.8)	1875.9 (1513.6 to 2131.8)	2797.3 (2491.7 to 3055.5)	1394 (1090.8 to 1714.2)	1081.3 (800.3 to 1339.1)	1803.3 (1411.5 to 2256.8)	-40.4 (-52.6 to -26.3)	-42.4 (-57.6 to -26.2)	-35.5 (-49.7 to -18.3)
Southeast Asia, east Asia, and Oceania	Southeast Asia	Sri Lanka	YLLs	All Ages	210,620 (182,036 to 230,167)	83,242 (66,775 to 95,959)	127,378 (112,286 to 141,002)	235,148 (165,912 to 310,612)	98,902 (64,257 to 134,256)	136,246 (94,647 to 183,057)	11.6 (-19.9 to 49.5)	18.8 (-23.4 to 64.6)	7 (-25.7 to 47.5)
Southeast Asia, east Asia, and Oceania	Southeast Asia	Sri Lanka	YLLs	Age-standardized	1989.7 (1721.8 to 2162.9)	1577 (1239 to 1811.6)	2399.7 (2109.9 to 2650.8)	1002.3 (714.5 to 1319)	758 (495.3 to 1019.1)	1324 (936.8 to 1746.5)	-49.6 (-63.6 to -33.3)	-51.9 (-68.7 to -33.8)	-44.8 (-61.2 to -25.4)
Southeast Asia, east Asia, and Oceania	Southeast Asia	Sri Lanka	YLDs	All Ages	47,101 (36,538 to 58,761)	20,661 (15,647 to 26,442)	26,440 (20,505 to 32,685)	92,141 (73,234 to 110,539)	40,455 (31,646 to 49,986)	51,686 (40,985 to 61,728)	95.6 (83.1 to 108.7)	95.8 (81.8 to 111.5)	95.5 (81.2 to 110.9)
Southeast Asia, east Asia, and Oceania	Southeast Asia	Sri Lanka	YLDs	Age-standardized	348.5 (272.9 to 427.3)	298.9 (230 to 377.9)	397.6 (310.7 to 486.9)	391.7 (311 to 472.9)	323.3 (252.3 to 399.5)	479.3 (381.5 to 576.5)	12.4 (7.1 to 17.6)	8.1 (2 to 14.5)	20.5 (13.3 to 27.8)
Southeast Asia, east Asia, and Oceania	Southeast Asia	Thailand	Incidence	All Ages	413,925 (361,118 to 480,925)	193,330 (167,371 to 225,232)	220,595 (190,692 to 256,933)	517,887 (462,552 to 582,026)	235,397 (209,289 to 264,550)	282,490 (252,119 to 319,283)	25.1 (15.3 to 35.3)	21.8 (11.1 to 33.2)	28.1 (16.7 to 39.5)
Southeast Asia, east Asia, and Oceania	Southeast Asia	Thailand	Incidence	Age-standardized	837.3 (747.5 to 954)	768.6 (679.1 to 884.1)	911 (812.5 to 1038.2)	766.1 (646.7 to 918.5)	678.2 (572.5 to 817.9)	861.7 (726.2 to 1029.5)	-8.5 (-15.6 to -0.5)	-11.8 (-19.7 to -3.1)	-5.4 (-13 to 3.2)
Southeast Asia, east Asia, and Oceania	Southeast Asia	Thailand	Prevalence	All Ages	2,920,668 (2,651,739 to 3,247,712)	1,364,306 (1,232,320 to 1,537,866	1,556,362 (1,410,047 to 1,733,935)	4,051,736 (3,731,730 to 4,426,815)	1,756,596 (1,596,582 to 1,944,602)	2,295,140 (2,112,920 to 2,499,728)	38.7 (31.2 to 46.4)	28.8 (19.5 to 37.8)	47.5 (38.8 to 57.1)
Southeast Asia, east Asia, and Oceania	Southeast Asia	Thailand	Prevalence	Age-standardized	6259 (5785.4 to 6800)	5665.1 (5193 to 6242.6)	6931.4 (6447.8 to 7538.9)	5192.6 (4631 to 5915.2)	4388 (3873 to 5093.7)	6109.2 (5477.6 to 6888.1)	-17 (-22 to -11.4)	-22.5 (-28.7 to -16.3)	-11.9 (-17.8 to -5.5)
Southeast Asia, east Asia, and Oceania	Southeast Asia	Thailand	Deaths	All Ages	20,374 (16,503 to 23,501)	8,099 (4,799 to 10,296)	12,276 (10,038 to 14,347)	23,925 (17,913 to 31,300)	8,037 (5,687 to 11,416)	15,888 (11,812 to 20,416)	17.4 (-16.4 to 64.1)	-0.8 (-37.6 to 89)	29.4 (-7.5 to 78.4)
Southeast Asia, east Asia, and Oceania	Southeast Asia	Thailand	Deaths	Age-standardized	70.2 (56.5 to 81.2)	51 (30.1 to 64.9)	96.5 (80.1 to 112.8)	24.8 (18.6 to 32.4)	14.6 (10.3 to 20.7)	38.5 (28.8 to 49.3)	-64.6 (-74.8 to -50.5)	-71.4 (-81.6 to -45.1)	-60 (-71.2 to -45.6)
Southeast Asia, east Asia, and Oceania	Southeast Asia	Thailand	DALYs	All Ages	663,556 (575,080 to 746,389)	261,824 (200,153 to 311,857)	401,732 (341,095 to 456,794)	712,558 (588,733 to 856,110)	250,752 (205,847 to 314,990)	461,805 (379,182 to 554,172)	7.4 (-13.4 to 32.6)	-4.2 (-27 to 36.6)	15 (-7.9 to 43.4)
Southeast Asia, east Asia, and Oceania	Southeast Asia	Thailand	DALYs	Age-standardized	1760.4 (1521.2 to 1980.3)	1304.7 (939.1 to 1561.9)	2310.7 (1957.6 to 2629.4)	790.1 (651.7 to 944.7)	531.4 (432.6 to 657.5)	1104 (908.4 to 1321.3)	-55.1 (-63.7 to -44.1)	-59.3 (-69 to -39.9)	-52.2 (-61.6 to -40.3)
Southeast Asia, east Asia, and Oceania	Southeast Asia	Thailand	YLLs	All Ages	469,193 (396,515 to 537,559)	174,705 (113,001 to 220,688)	294,488 (240,601 to 345,117)	409,227 (300,103 to 548,198)	129,361 (93,019 to 187,329)	279,866 (206,366 to 368,898)	-12.8 (-38.9 to 22.3)	-26 (-53.2 to 34.6)	-5 (-33.4 to 32.7)
Southeast Asia, east Asia, and Oceania	Southeast Asia	Thailand	YLLs	Age-standardized	1317.7 (1083.5 to 1515.2)	928.5 (568 to 1177.1)	1789.5 (1467.4 to 2090.1)	430 (318.2 to 572.3)	249.7 (180.8 to 361.2)	651.9 (489.4 to 848.5)	-67.4 (-77 to -53.3)	-73.1 (-82.8 to -48.7)	-63.6 (-74.3 to -49.3)
Southeast Asia, east Asia, and Oceania	Southeast Asia	Thailand	YLDs	All Ages	194,362 (151,854 to 243,488)	87,119 (67,169 to 111,141)	107,244 (83,620 to 132,711)	303,331 (241,008 to 364,615)	121,991 (95,348 to 149,836)	181,940 (143,370 to 216,635)	56.1 (46.6 to 66.1)	39.3 (29 to 50.4)	69.7 (56.7 to 82.6)
Southeast Asia, east Asia, and Oceania	Southeast Asia	Thailand	YLDs	Age-standardized	442.7 (348.2 to 541.2)	376.2 (292.7 to 464.8)	521.1 (412.9 to 630)	360.1 (280.3 to 445.9)	281.8 (216.2 to 359)	452 (354.6 to 547.5)	-18.7 (-23 to -14.3)	-25.1 (-31.1 to -19.5)	-13.3 (-18.4 to -8.2)
Southeast Asia, east Asia, and Oceania	Southeast Asia	Timor-Leste	Incidence	All Ages	6,090 (4,931 to 7,541)	3,017 (2,444 to 3,735)	3,073 (2,467 to 3,885)	9,891 (8,404 to 11,842)	4,658 (3,959 to 5,563)	5,233 (4,355 to 6,292)	62.4 (47.9 to 77.5)	54.4 (38.4 to 72.4)	70.3 (53 to 86.9)
Southeast Asia, east Asia, and Oceania	Southeast Asia	Timor-Leste	Incidence	Age-standardized	757.7 (659.7 to 874.4)	763.5 (661.5 to 880.2)	751.7 (650.7 to 878)	784.2 (694.1 to 896.5)	747.1 (659.9 to 858.3)	821.4 (722.3 to 941.8)	3.5 (-1.7 to 8.9)	-2.1 (-8.8 to 4.9)	9.3 (2.5 to 15.8)
Southeast Asia, east Asia, and Oceania	Southeast Asia	Timor-Leste	Prevalence	All Ages	32,652 (28,330 to 37,737)	16,506 (14,334 to 19,040)	16,145 (13,880 to 18,994)	58,720 (52,645 to 66,054)	27,948 (25,186 to 31,216)	30,772 (27,236 to 34,942)	79.8 (65.5 to 94.6)	69.3 (53.3 to 85.9)	90.6 (73.7 to 107.3)
Southeast Asia, east Asia, and Oceania	Southeast Asia	Timor-Leste	Prevalence	Age-standardized	4900 (4486.9 to 5367)	5049.4 (4630.3 to 5538.2)	4759.1 (4327.2 to 5256.2)	5185.2 (4803.8 to 5621.5)	4956.4 (4592.1 to 5361.7)	5422.1 (4983.3 to 5916.8)	5.8 (1 to 10.3)	-1.8 (-7.2 to 3.6)	13.9 (7.5 to 19.9)
Southeast Asia, east Asia, and Oceania	Southeast Asia	Timor-Leste	Deaths	All Ages	244 (184 to 311)	118 (78 to 157)	126 (84 to 169)	516 (391 to 651)	188 (129 to 285)	327 (231 to 439)	111.1 (53.9 to 184)	59.9 (7 to 149.1)	158.8 (79.4 to 251.7)
Southeast Asia, east Asia, and Oceania	Southeast Asia	Timor-Leste	Deaths	Age-standardized	99.3 (74.6 to 127.5)	87.6 (59.5 to 127.9)	112.7 (76.6 to 157.7)	77.7 (59.7 to 98)	55.4 (39.4 to 86.2)	100.5 (72.6 to 132.6)	-21.8 (-40.3 to 0.8)	-36.8 (-53.6 to -11.7)	-10.8 (-38 to 19.2)
Southeast Asia, east Asia, and Oceania	Southeast Asia	Timor-Leste	DALYs	All Ages	10,784 (7,961 to 14,335)	5,570 (3,604 to 8,278)	5,214 (3,722 to 6,845)	15,501 (12,040 to 18,954)	6,322 (4,540 to 8,557)	9,179 (6,810 to 11,755)	43.7 (1 to 101.8)	13.5 (-29.8 to 98.9)	76 (30.5 to 134.6)
Southeast Asia, east Asia, and Oceania	Southeast Asia	Timor-Leste	DALYs	Age-standardized	2429.2 (1905.8 to 2982.1)	2325.3 (1652.2 to 2983.3)	2544.6 (1793 to 3314.9)	1780.7 (1406.1 to 2175.6)	1399.2 (1025.9 to 1925.5)	2174.5 (1633.8 to 2779.9)	-26.7 (-43.2 to -7.3)	-39.8 (-56.1 to -17.1)	-14.5 (-37.6 to 10.6)
Southeast Asia, east Asia, and Oceania	Southeast Asia	Timor-Leste	YLLs	All Ages	8,908 (5,987 to 12,378)	4,634 (2,702 to 7,342)	4,275 (2,762 to 5,934)	11,501 (8,162 to 14,559)	4,450 (2,764 to 6,673)	7,051 (4,711 to 9,583)	29.1 (-17.6 to 97.2)	-4 (-48.2 to 96.5)	65 (11.6 to 139.2)
Southeast Asia, east Asia, and Oceania	Southeast Asia	Timor-Leste	YLLs	Age-standardized	2099.4 (1591.5 to 2648.9)	1993.6 (1316.9 to 2676.3)	2216.3 (1468 to 3000.7)	1391.5 (1026.8 to 1764.8)	1039.2 (688.1 to 1564.9)	1755.5 (1233 to 2349.9)	-33.7 (-51.8 to -11.5)	-47.9 (-65.5 to -22)	-20.8 (-46 to 6.8)
Southeast Asia, east Asia, and Oceania	Southeast Asia	Timor-Leste	YLDs	All Ages	1,876 (1,385 to 2,450)	936 (685 to 1,230)	939 (693 to 1,244)	4,000 (3,106 to 4,945)	1,873 (1,461 to 2,336)	2,127 (1,655 to 2,656)	113.2 (93.6 to 134.1)	100 (79.3 to 122.5)	126.5 (104.3 to 150)
Southeast Asia, east Asia, and Oceania	Southeast Asia	Timor-Leste	YLDs	Age-standardized	329.8 (256 to 405.5)	331.7 (255.8 to 409.5)	328.3 (254.8 to 406)	389.2 (308.4 to 464.3)	360 (285.1 to 435.7)	419 (332.4 to 505.5)	18 (11.6 to 24.3)	8.6 (1.9 to 15.4)	27.6 (19.7 to 35.8)
Southeast Asia, east Asia, and Oceania	Southeast Asia	Viet Nam	Incidence	All Ages	421,694 (347,870 to 516,011)	191,127 (159,031 to 232,727)	230,566 (187,850 to 284,772)	739,902 (658,481 to 842,860)	324,950 (287,792 to 367,955)	414,952 (369,449 to 475,268)	75.5 (59.7 to 94.8)	70 (54.1 to 87.8)	80 (62.4 to 101.4)
Southeast Asia, east Asia, and Oceania	Southeast Asia	Viet Nam	Incidence	Age-standardized	665.3 (583 to 777.2)	572.9 (497.1 to 668.4)	780.1 (684.7 to 897.4)	832.4 (737.2 to 962.1)	695.9 (606.9 to 808.2)	984.7 (874.2 to 1128.4)	25.1 (20.6 to 29.8)	21.5 (16.1 to 27.2)	26.2 (20.4 to 32.1)
Southeast Asia, east Asia, and Oceania	Southeast Asia	Viet Nam	Prevalence	All Ages	2,520,020 (2,210,994 to 2,939,130)	1,148,355 (1,005,772 to 1,341,024	1,371,665 (1,200,582 to 1,603,964)	5,213,582 (4,785,689 to 5,726,561	2,228,216 (2,040,774 to 2,463,502)	2,985,367 (2,748,818 to 3,280,822)	106.9 (92 to 122.3)	94 (78.7 to 110.1)	117.6 (99.5 to 136.4)
Southeast Asia, east Asia, and Oceania	Southeast Asia	Viet Nam	Prevalence	Age-standardized	4481.2 (4091.8 to 4962.6)	3775.7 (3418.2 to 4221.3)	5432.2 (5002.4 to 5947.3)	5648.2 (5165.8 to 6256)	4513 (4081.4 to 5071.4)	7088.9 (6517.9 to 7778.5)	26 (21.7 to 30.6)	19.5 (14.1 to 25.5)	30.5 (24.8 to 36.7)
Southeast Asia, east Asia, and Oceania	Southeast Asia	Viet Nam	Deaths	All Ages	26,078 (9,883 to 33,160)	11,200 (4,116 to 16,289)	14,878 (5,365 to 19,220)	36,229 (14,127 to 45,192)	13,795 (4,206 to 19,122)	22,434 (7,493 to 28,471)	38.9 (0.9 to 91.3)	23.2 (-35.1 to 89.9)	50.8 (11.7 to 135.2)
Southeast Asia, east Asia, and Oceania	Southeast Asia	Viet Nam	Deaths	Age-standardized	73.9 (27.6 to 93.7)	51.3 (18.6 to 75)	113.7 (40.8 to 146.6)	47.6 (18.7 to 59.3)	29.2 (8.9 to 40.4)	79.8 (26.6 to 100.6)	-35.6 (-53.6 to -12)	-43.1 (-70.1 to -12.1)	-29.8 (-47.9 to 8.2)
Southeast Asia, east Asia, and Oceania	Southeast Asia	Viet Nam	DALYs	All Ages	666,814 (354,562 to 817,379)	271,518 (149,777 to 353,598)	395,296 (195,939 to 490,462)	1,006,629 (595,014 to 1,204,734)	372,588 (226,106 to 468,529)	634,040 (332,198 to 780,023)	51 (20.1 to 89.4)	37.2 (-7.1 to 83.6)	60.4 (23.4 to 125.8)
Southeast Asia, east Asia, and Oceania	Southeast Asia	Viet Nam	DALYs	Age-standardized	1568.7 (775.9 to 1931.9)	1095.3 (566.3 to 1454.1)	2279.5 (1035.6 to 2834.6)	1153 (671.8 to 1371.1)	751.2 (453.4 to 944.7)	1743.4 (875 to 2118.7)	-26.5 (-42.6 to -5.1)	-31.4 (-54.7 to -5.9)	-23.5 (-41.1 to 10.1)
Southeast Asia, east Asia, and Oceania	Southeast Asia	Viet Nam	YLLs	All Ages	518,451 (209,003 to 653,061)	199,139 (79,364 to 277,450)	319,312 (119,176 to 413,116)	657,758 (255,828 to 830,537)	210,645 (71,188 to 297,596)	447,113 (149,849 to 584,518)	26.9 (-6.7 to 74.5)	5.8 (-41.9 to 64.9)	40 (1.7 to 122.5)
Southeast Asia, east Asia, and Oceania	Southeast Asia	Viet Nam	YLLs	Age-standardized	1286 (505.8 to 1635.3)	844.4 (322 to 1197.4)	1952.1 (706.1 to 2508.4)	775.2 (305.7 to 973.5)	428.9 (143.5 to 603.8)	1292.3 (437.6 to 1645)	-39.7 (-55.6 to -16.2)	-49.2 (-72.4 to -21.1)	-33.8 (-51.2 to 4.3)
Southeast Asia, east Asia, and Oceania	Southeast Asia	Viet Nam	YLDs	All Ages	148,363 (113,406 to 188,966)	72,379 (55,671 to 92,054)	75,984 (57,766 to 97,552)	348,871 (273,466 to 428,288)	161,944 (126,847 to 198,368)	186,927 (145,444 to 231,448)	135.1 (116.8 to 154.5)	123.7 (105 to 144.8)	146 (123.1 to 168)
Southeast Asia, east Asia, and Oceania	Southeast Asia	Viet Nam	YLDs	Age-standardized	282.7 (219.9 to 346.3)	250.8 (194.4 to 310.5)	327.4 (253.8 to 407.4)	377.8 (295.9 to 464.3)	322.3 (252.1 to 398.8)	451.1 (352.3 to 556.1)	33.7 (28.2 to 39.3)	28.5 (21.3 to 36)	37.8 (30.5 to 45.2)
		Oceania	Incidence	All Ages	74,682 (64,772 to 86,529)	35,455 (30,876 to 40,769)	39,227 (33,751 to 46,346)	145,544 (128,755 to 165,575)	68,748 (60,906 to 77,750)	76,796 (67,692 to 87,916)	94.9 (86.2 to 104.5)	93.9 (83.5 to 105.2)	95.8 (83.9 to 108)
		Oceania	Incidence	Age-standardized	1357.6 (1229.3 to 1499.3)	1328.4 (1194 to 1465.5)	1386.5 (1251 to 1548.5)	1291.2 (1172.3 to 1420.7)	1253.9 (1134.7 to 1380)	1326.2 (1197.7 to 1470.8)	-4.9 (-7.9 to -1.5)	-5.6 (-9.4 to -1.2)	-4.3 (-8.6 to 0)
		Oceania	Prevalence	All Ages	381,292 (347,259 to 424,961)	186,911 (170,405 to 206,830)	194,382 (174,984 to 220,103)	699,604 (640,666 to 766,013)	350,149 (319,260 to 381,501)	349,455 (316,898 to 389,675)	83.5 (77.4 to 89.5)	87.3 (78.1 to 96.4)	79.8 (72.5 to 87.2)
		Oceania	Prevalence	Age-standardized	7570.1 (7089.9 to 8107.3)	7816.3 (7282.4 to 8371.8)	7326 (6829.1 to 7883.9)	6504.6 (6095.2 to 6939)	6798 (6325 to 7263.4)	6200.7 (5780.3 to 6695.4)	-14.1 (-16.3 to -11.8)	-13 (-16.1 to -9.8)	-15.4 (-18 to -12.6)
		Oceania	Deaths	All Ages	4,656 (3,880 to 5,451)	2,098 (1,664 to 2,609)	2,558 (2,067 to 3,058)	8,940 (7,142 to 11,103)	4,206 (3,140 to 5,399)	4,734 (3,745 to 5,857)	92 (52.4 to 137.8)	100.5 (47 to 161.8)	85.1 (47 to 132.4)
		Oceania	Deaths	Age-standardized	201.8 (164.7 to 235.5)	178.4 (139.7 to 224.3)	228.3 (185.1 to 269.2)	166.3 (133.3 to 202.6)	154 (115.1 to 194.6)	179.6 (142.6 to 215.4)	-17.6 (-34.5 to 0.4)	-13.7 (-37.2 to 11.9)	-21.4 (-36.6 to -4.1)
		Oceania	DALYs	All Ages	153,816 (131,582 to 180,254)	72,482 (59,586 to 90,170)	81,334 (67,669 to 96,039)	283,407 (230,257 to 352,567)	137,739 (107,651 to 178,445)	145,669 (117,904 to 178,646)	84.3 (50.6 to 124.1)	90 (49.2 to 141.2)	79.1 (46.5 to 122)
		Oceania	DALYs	Age-standardized	4495.5 (3837.1 to 5182.7)	4183.3 (3414 to 5086.8)	4824 (3999.9 to 5689.5)	3677.6 (3020.9 to 4477.2)	3551.2 (2814.1 to 4445.2)	3805.9 (3080.1 to 4590)	-18.2 (-33.3 to -1)	-15.1 (-34.9 to 8.1)	-21.1 (-35.8 to -3.5)
		Oceania	YLLs	All Ages	132,561 (112,126 to 158,246)	61,730 (49,087 to 79,305)	70,831 (57,457 to 85,490)	240,223 (189,175 to 309,410)	115,451 (85,671 to 156,506)	124,772 (97,869 to 156,569)	81.2 (43.1 to 128.2)	87 (39.1 to 147.5)	76.2 (39 to 126)
		Oceania	YLLs	Age-standardized	4023.6 (3363.3 to 4699.9)	3677.9 (2935.6 to 4586)	4386.1 (3559.3 to 5193.1)	3240.2 (2588.4 to 4020)	3078.5 (2303.7 to 3966.6)	3404.4 (2715.4 to 4205.5)	-19.5 (-36.2 to -0.4)	-16.3 (-38.9 to 9.8)	-22.4 (-38.3 to -2.9)
		Oceania	YLDs	All Ages	21,254 (15,912 to 27,587)	10,752 (8,112 to 13,768)	10,502 (7,800 to 13,833)	43,184 (33,357 to 54,651)	22,287 (17,306 to 27,940)	20,897 (15,851 to 26,710)	103.2 (94.3 to 113.2)	107.3 (96.4 to 119.5)	99 (88.7 to 110)
		Oceania	YLDs	Age-standardized	471.9 (371.9 to 579)	505.5 (398.3 to 618.8)	437.9 (337.8 to 548.5)	437.5 (348.4 to 530.1)	472.7 (378.3 to 573)	401.5 (316.4 to 496.6)	-7.3 (-10.3 to -4.2)	-6.5 (-10 to -2.7)	-8.3 (-11.9 to -4.4)
Southeast Asia, east Asia, and Oceania	Oceania	American Samoa	Incidence	All Ages	432 (354 to 536)	210 (167 to 262)	222 (180 to 274)	497 (410 to 593)	240 (197 to 285)	256 (211 to 312)	14.9 (3.8 to 25)	14.4 (4.6 to 27.9)	15.3 (-2.3 to 27)
Southeast Asia, east Asia, and Oceania	Oceania	American Samoa	Incidence	Age-standardized	970.8 (837.1 to 1126.4)	978.6 (814.1 to 1147.7)	959.8 (826.8 to 1122.6)	941 (785.2 to 1111)	917.5 (755.3 to 1074.5)	959.6 (794.4 to 1146.2)	-3.1 (-10.8 to 3.8)	-6.2 (-11.5 to 4.3)	0 (-13.8 to 8.1)
Southeast Asia, east Asia, and Oceania	Oceania	American Samoa	Prevalence	All Ages	2,029 (1,702 to 2,472)	1,020 (788 to 1,272)	1,009 (849 to 1,246)	2,423 (1,877 to 2,918)	1,221 (932 to 1,440)	1,202 (941 to 1,477)	19.4 (3.7 to 32)	19.6 (9.3 to 39.2)	19.2 (-6.4 to 35.1)
Southeast Asia, east Asia, and Oceania	Oceania	American Samoa	Prevalence	Age-standardized	4996.2 (4325.9 to 5761.9)	5237 (4082.8 to 6224.8)	4753.2 (4227.2 to 5614.4)	4604.8 (3588.7 to 5445.7)	4665.2 (3577.2 to 5461.1)	4509.1 (3554.6 to 5419.4)	-7.8 (-19.3 to 2.6)	-10.9 (-17.2 to 5.1)	-5.1 (-24.1 to 6.4)
Southeast Asia, east Asia, and Oceania	Oceania	American Samoa	Deaths	All Ages	17 (15 to 19)	6 (5 to 8)	11 (9 to 12)	23 (19 to 28)	9 (7 to 12)	14 (12 to 16)	36.5 (9.4 to 72.1)	57.6 (4.8 to 126)	25 (3.2 to 52.1)
Southeast Asia, east Asia, and Oceania	Oceania	American Samoa	Deaths	Age-standardized	96.5 (82.7 to 109.3)	67.9 (52.5 to 86.2)	128.5 (111 to 145.6)	57.8 (48.7 to 69.9)	44.2 (33.2 to 58.8)	74 (64.1 to 87.6)	-40.1 (-51.9 to -24.7)	-34.9 (-56.2 to -7.7)	-42.4 (-52.3 to -29.8)
Southeast Asia, east Asia, and Oceania	Oceania	American Samoa	DALYs	All Ages	556 (491 to 628)	213 (175 to 262)	344 (298 to 389)	646 (552 to 771)	273 (222 to 342)	373 (320 to 440)	16.2 (-3.9 to 41.1)	28.4 (-5.7 to 67.8)	8.7 (-10.1 to 30.4)
Southeast Asia, east Asia, and Oceania	Oceania	American Samoa	DALYs	Age-standardized	2119.3 (1866.8 to 2360.4)	1577.7 (1283.5 to 1936.4)	2680.5 (2337.8 to 3005.9)	1370 (1177.3 to 1643.9)	1120 (914.1 to 1407.8)	1644.8 (1423.5 to 1918.5)	-35.4 (-46.8 to -21.3)	-29 (-48.1 to -6.3)	-38.6 (-48.2 to -26.6)
Southeast Asia, east Asia, and Oceania	Oceania	American Samoa	YLLs	All Ages	443 (382 to 504)	155 (121 to 202)	288 (246 to 329)	502 (414 to 623)	197 (148 to 262)	305 (254 to 367)	13.2 (-11.6 to 45)	27 (-17.4 to 84.4)	5.8 (-14.8 to 31.1)
Southeast Asia, east Asia, and Oceania	Oceania	American Samoa	YLLs	Age-standardized	1809.3 (1567.9 to 2044)	1251 (979.3 to 1597.8)	2388.7 (2069 to 2689.1)	1086 (905.3 to 1341.5)	823.4 (619.5 to 1093.2)	1376.8 (1161.2 to 1650.2)	-40 (-52.7 to -23.7)	-34.2 (-56.6 to -5.8)	-42.4 (-53 to -29.3)
Southeast Asia, east Asia, and Oceania	Oceania	American Samoa	YLDs	All Ages	113 (83 to 153)	57 (41 to 78)	56 (41 to 75)	145 (107 to 190)	76 (57 to 97)	69 (49 to 92)	28.1 (16.8 to 39.6)	32.4 (20.5 to 48.8)	23.7 (4.6 to 36.9)
Southeast Asia, east Asia, and Oceania	Oceania	American Samoa	YLDs	Age-standardized	310 (237.2 to 390.2)	326.6 (247.9 to 416.1)	291.8 (226.7 to 372)	284.1 (212.7 to 367)	296.6 (223.9 to 375.9)	268.1 (197 to 350.8)	-8.4 (-15.5 to -1.2)	-9.2 (-14.9 to 0.9)	-8.1 (-21 to 1.1)
Southeast Asia, east Asia, and Oceania	Oceania	Cook Islands	Incidence	All Ages	206 (174 to 248)	95 (79 to 114)	111 (94 to 135)	225 (194 to 255)	110 (95 to 124)	115 (100 to 131)	9.2 (-0.4 to 19.2)	16 (6 to 27.2)	3.4 (-6.1 to 14.3)
Southeast Asia, east Asia, and Oceania	Oceania	Cook Islands	Incidence	Age-standardized	1165.8 (1009.9 to 1357.9)	1129.4 (967.9 to 1310.1)	1199.5 (1042.2 to 1406.6)	1151.8 (987.6 to 1337.4)	1114.2 (954.6 to 1291)	1190.5 (1021.5 to 1394.5)	-1.2 (-4.8 to 3.9)	-1.3 (-5.2 to 3.4)	-0.8 (-6.2 to 7.9)
Southeast Asia, east Asia, and Oceania	Oceania	Cook Islands	Prevalence	All Ages	1,145 (968 to 1,349)	550 (459 to 642)	595 (499 to 712)	1,357 (1,155 to 1,502)	710 (600 to 791)	647 (552 to 725)	18.6 (9.7 to 29.8)	29.1 (19.2 to 40.1)	8.9 (-0.3 to 22.5)
Southeast Asia, east Asia, and Oceania	Oceania	Cook Islands	Prevalence	Age-standardized	7028.2 (6010 to 7983.3)	7143.1 (5990.3 to 8094.6)	6913.3 (5912.7 to 8035.1)	6617.4 (5620.6 to 7518.7)	6732.2 (5663.1 to 7641.5)	6479.4 (5517.7 to 7443.5)	-5.8 (-9.7 to 1.8)	-5.8 (-10 to 0)	-6.3 (-11.8 to 4.9)
Southeast Asia, east Asia, and Oceania	Oceania	Cook Islands	Deaths	All Ages	8 (6 to 9)	4 (3 to 4)	4 (3 to 5)	7 (6 to 9)	4 (3 to 5)	4 (3 to 4)	-1.5 (-20.1 to 21.2)	4.7 (-21.5 to 37.1)	-7.1 (-26.1 to 17.2)
Southeast Asia, east Asia, and Oceania	Oceania	Cook Islands	Deaths	Age-standardized	71.9 (60.1 to 82.6)	68.5 (51.9 to 83.6)	74.8 (62.9 to 86.2)	32.7 (27.1 to 40)	31.4 (24 to 40.2)	33.9 (28.5 to 40.3)	-54.4 (-62.8 to -44.1)	-54.1 (-65.6 to -39.8)	-54.7 (-63.8 to -42.8)
Southeast Asia, east Asia, and Oceania	Oceania	Cook Islands	DALYs	All Ages	251 (218 to 283)	122 (100 to 144)	129 (113 to 146)	237 (206 to 272)	121 (102 to 143)	116 (100 to 133)	-5.5 (-18.2 to 10.2)	-0.4 (-17.9 to 20.1)	-10.3 (-23.4 to 5.6)
Southeast Asia, east Asia, and Oceania	Oceania	Cook Islands	DALYs	Age-standardized	1858.2 (1627.8 to 2078.9)	1852.8 (1538.4 to 2192.1)	1860.1 (1619.3 to 2077.7)	1056.6 (910.2 to 1218.2)	1048.3 (881.9 to 1237)	1062.8 (915.2 to 1230.2)	-43.1 (-51.3 to -34)	-43.4 (-53.7 to -31.3)	-42.9 (-51.2 to -32.3)
Southeast Asia, east Asia, and Oceania	Oceania	Cook Islands	YLLs	All Ages	175 (148 to 204)	84 (65 to 104)	92 (78 to 105)	132 (107 to 163)	63 (48 to 82)	69 (57 to 83)	-24.9 (-40.7 to -4.4)	-24.8 (-45.4 to 1.5)	-24.9 (-41.1 to -4)
Southeast Asia, east Asia, and Oceania	Oceania	Cook Islands	YLLs	Age-standardized	1358.5 (1147.7 to 1566.3)	1318.9 (1030.9 to 1639.3)	1393.6 (1186.2 to 1596.5)	568.5 (458.2 to 709.1)	525.6 (394.4 to 688.1)	612.6 (503.6 to 739.8)	-58.2 (-66.9 to -46.9)	-60.1 (-71.5 to -46.3)	-56 (-65.6 to -44)
Southeast Asia, east Asia, and Oceania	Oceania	Cook Islands	YLDs	All Ages	75 (58 to 95)	38 (29 to 47)	37 (28 to 48)	105 (83 to 125)	58 (47 to 69)	47 (37 to 57)	39.6 (28.2 to 53)	53.3 (40.3 to 67.8)	25.8 (14 to 40.6)
Southeast Asia, east Asia, and Oceania	Oceania	Cook Islands	YLDs	Age-standardized	499.7 (393 to 603)	533.9 (418.4 to 642.7)	466.5 (364.1 to 577)	488.1 (381.3 to 589.3)	522.7 (416.5 to 626)	450.2 (350.1 to 553.3)	-2.3 (-6.1 to 2.7)	-2.1 (-6.6 to 2.9)	-3.5 (-8.7 to 4.8)
Southeast Asia, east Asia, and Oceania	Oceania	Fiji	Incidence	All Ages	6,674 (5,673 to 7,820)	3,074 (2,615 to 3,603)	3,600 (3,050 to 4,275)	7,142 (6,219 to 8,297)	3,283 (2,841 to 3,788)	3,859 (3,335 to 4,536)	7 (-0.3 to 15)	6.8 (-2.2 to 17.2)	7.2 (-3.1 to 17.4)
Southeast Asia, east Asia, and Oceania	Oceania	Fiji	Incidence	Age-standardized	1004 (890.4 to 1130.7)	918.7 (808.9 to 1040.4)	1095.1 (974.8 to 1246)	826.5 (725 to 949.5)	756.9 (657.2 to 868.6)	908.7 (799 to 1047.8)	-17.7 (-22 to -13)	-17.6 (-22.9 to -11.7)	-17 (-23.5 to -10.6)
Southeast Asia, east Asia, and Oceania	Oceania	Fiji	Prevalence	All Ages	34,516 (30,271 to 39,439)	16,079 (13,979 to 18,501)	18,437 (16,079 to 21,198)	33,500 (29,503 to 38,169)	15,714 (13,679 to 17,977)	17,786 (15,610 to 20,603)	-2.9 (-8.7 to 3.1)	-2.3 (-10.1 to 6.6)	-3.5 (-11.6 to 4.9)
Southeast Asia, east Asia, and Oceania	Oceania	Fiji	Prevalence	Age-standardized	5548.4 (4989 to 6176.7)	5153.1 (4627.2 to 5782.4)	5951.3 (5318.4 to 6679)	3897.6 (3462.6 to 4415)	3634.5 (3192.6 to 4133.7)	4202 (3716.1 to 4794.3)	-29.8 (-33.7 to -25.8)	-29.5 (-35 to -23.8)	-29.4 (-34.4 to -23.9)
Southeast Asia, east Asia, and Oceania	Oceania	Fiji	Deaths	All Ages	400 (320 to 495)	147 (107 to 209)	253 (201 to 307)	412 (321 to 534)	156 (112 to 211)	257 (201 to 325)	3.2 (-28.3 to 45.3)	6.2 (-40.8 to 67.1)	1.5 (-25.9 to 41.1)
Southeast Asia, east Asia, and Oceania	Oceania	Fiji	Deaths	Age-standardized	143 (115.8 to 177.7)	99.3 (72.2 to 141.7)	196.5 (158.3 to 236.1)	73.4 (58.7 to 92.7)	50.6 (37 to 67.9)	110.3 (88.9 to 133.8)	-48.7 (-64 to -29)	-49 (-71 to -21.4)	-43.9 (-58.3 to -24.8)
Southeast Asia, east Asia, and Oceania	Oceania	Fiji	DALYs	All Ages	12,981 (10,558 to 15,857)	5,145 (3,920 to 7,021)	7,836 (6,383 to 9,381)	12,115 (9,745 to 15,187)	4,735 (3,613 to 6,150)	7,380 (5,906 to 9,201)	-6.7 (-32.1 to 25.2)	-8 (-43.1 to 35.7)	-5.8 (-30.1 to 27.2)
Southeast Asia, east Asia, and Oceania	Oceania	Fiji	DALYs	Age-standardized	3107.2 (2537.1 to 3785.4)	2308.1 (1744.5 to 3178)	3981.9 (3261.5 to 4753)	1638.6 (1326.9 to 2043.9)	1214.1 (932.8 to 1570.1)	2201.1 (1791.1 to 2694.9)	-47.3 (-61.6 to -29.1)	-47.4 (-67.9 to -22.1)	-44.7 (-58.6 to -25.7)
Southeast Asia, east Asia, and Oceania	Oceania	Fiji	YLLs	All Ages	11,287 (8,973 to 14,077)	4,332 (3,145 to 6,218)	6,955 (5,531 to 8,544)	10,383 (8,015 to 13,548)	3,896 (2,804 to 5,317)	6,487 (5,036 to 8,334)	-8 (-36.5 to 29.2)	-10.1 (-48.8 to 42.5)	-6.7 (-33.3 to 30.6)
Southeast Asia, east Asia, and Oceania	Oceania	Fiji	YLLs	Age-standardized	2822 (2258.2 to 3506.5)	2034.2 (1479.3 to 2898.4)	3685.3 (2956.1 to 4445.6)	1432.3 (1119.6 to 1847.8)	1016.1 (735.6 to 1382.2)	1984.6 (1573 to 2482.4)	-49.2 (-64.5 to -29.1)	-50 (-71.8 to -21.4)	-46.1 (-60.7 to -25.7)
Southeast Asia, east Asia, and Oceania	Oceania	Fiji	YLDs	All Ages	1,694 (1,219 to 2,329)	813 (589 to 1,105)	880 (621 to 1,207)	1,733 (1,276 to 2,290)	840 (623 to 1,105)	893 (643 to 1,210)	2.3 (-4 to 9.1)	3.3 (-5.3 to 12.2)	1.4 (-6.7 to 10.9)

Chronic respiratory diseases

7 GBD super region	21 GBD region	Location	Measure	Age	1990 Both	1990 Female	1990 Male	2019 Both	2019 Female	2019 Male	Percent change (1990 to 2019) Both	Percent change (1990 to 2019) Female	Percent change (1990 to 2019) Male
[illegible]	[illegible]	[illegible]	[illegible]	[illegible]	[illegible]	[illegible]	[illegible]	[illegible]	[illegible]	[illegible]	[illegible]	[illegible]	[illegible]

Chronic respiratory diseases

7 GBD super region	21 GBD region	Location	Measure	Age	1990 Both	1990 Female	1990 Male	2019 Both	2019 Female	2019 Male	Percent change (1990 to 2019) Both	Percent change (1990 to 2019) Female	Percent change (1990 to 2019) Male
Central Europe, eastern Europe, and central Asia	Central Asia	Mongolia	YLDs	All Ages	3,276 (2,417 to 4,384)	1,744 (1,296 to 2,330)	1,532 (1,110 to 2,064)	5,041 (3,745 to 6,603)	2,638 (1,966 to 3,410)	2,403 (1,749 to 3,183)	53.9 (43.5 to 66)	51.3 (38.5 to 66.4)	56.8 (44 to 70.9)
Central Europe, eastern Europe, and central Asia	Central Asia	Mongolia	YLDs	Age-standardized	205.4 (155.2 to 262.5)	215.9 (162.7 to 275.2)	194.8 (144.7 to 250.2)	169.2 (126.9 to 217.3)	168.2 (126.5 to 215.7)	172.4 (129.2 to 223.1)	-17.6 (-22.6 to -12.6)	-22.1 (-28.4 to -15.7)	-11.5 (-17 to -5.7)
Central Europe, eastern Europe, and central Asia	Central Asia	Tajikistan	Incidence	All Ages	42,431 (35,887 to 50,482)	21,255 (17,887 to 25,154)	21,176 (17,899 to 25,607)	68,025 (57,310 to 80,653)	32,753 (27,599 to 38,857)	35,272 (29,411 to 42,306)	60.3 (52.8 to 68.1)	54.1 (44.9 to 63.9)	66.6 (56 to 76.5)
Central Europe, eastern Europe, and central Asia	Central Asia	Tajikistan	Incidence	Age-standardized	993.9 (871.6 to 1128)	968 (839.1 to 1113.3)	1032.5 (906.1 to 1171.6)	880.6 (768.8 to 1006.7)	843.2 (728.9 to 965.3)	922.4 (801.8 to 1060.3)	-11.4 (-15.8 to -7)	-12.9 (-18.8 to -6.7)	-10.7 (-16 to -5)
Central Europe, eastern Europe, and central Asia	Central Asia	Tajikistan	Prevalence	All Ages	192,262 (169,172 to 224,664)	99,769 (88,018 to 115,185)	92,493 (80,086 to 108,959)	304,980 (264,389 to 359,089)	151,986 (132,671 to 177,064)	152,994 (131,094 to 182,265)	58.6 (50 to 68.7)	52.3 (42.9 to 62.6)	65.4 (52.7 to 78.5)
Central Europe, eastern Europe, and central Asia	Central Asia	Tajikistan	Prevalence	Age-standardized	4789.2 (4404.2 to 5246.7)	4841.9 (4450.6 to 5307.6)	4770 (4360.7 to 5238.8)	3985.2 (3576.6 to 4494.7)	3972.7 (3571.9 to 4442.3)	4006.2 (3588.5 to 4537.9)	-16.8 (-20.9 to -12.1)	-18 (-22.4 to -13)	-16 (-21.5 to -10.1)
Central Europe, eastern Europe, and central Asia	Central Asia	Tajikistan	Deaths	All Ages	1,682 (1,436 to 1,852)	821 (618 to 946)	862 (774 to 949)	1,669 (1,337 to 2,395)	750 (558 to 1,196)	919 (738 to 1,252)	-0.8 (-23.1 to 57.2)	-8.6 (-38.1 to 74.7)	6.7 (-15.6 to 50.6)
Central Europe, eastern Europe, and central Asia	Central Asia	Tajikistan	Deaths	Age-standardized	61.5 (51.9 to 67.6)	51.4 (38.4 to 59.1)	76.8 (68.2 to 84.5)	48.8 (38.6 to 72.7)	41 (29.6 to 68.1)	59.6 (47.2 to 84.1)	-20.6 (-38.8 to 32.2)	-20.3 (-46.9 to 57.6)	-22.3 (-38.4 to 16.7)
Central Europe, eastern Europe, and central Asia	Central Asia	Tajikistan	DALYs	All Ages	47,585 (42,706 to 51,647)	23,632 (19,494 to 26,429)	23,954 (21,686 to 26,458)	58,894 (49,440 to 74,023)	27,352 (22,214 to 36,985)	31,542 (26,432 to 38,954)	23.8 (4.2 to 62.7)	15.7 (-7 to 74)	31.7 (9.8 to 62)
Central Europe, eastern Europe, and central Asia	Central Asia	Tajikistan	DALYs	Age-standardized	1522.2 (1349.9 to 1647.9)	1362.4 (1097.7 to 1519.2)	1762.8 (1601 to 1931.1)	1098.2 (914.2 to 1474)	965.1 (769.8 to 1401.4)	1259.5 (1050.8 to 1640.3)	-27.9 (-40.5 to 4.3)	-29.2 (-45.4 to 16.9)	-28.6 (-41.2 to -4.4)
Central Europe, eastern Europe, and central Asia	Central Asia	Tajikistan	YLLs	All Ages	37,789 (33,668 to 40,845)	18,274 (14,204 to 20,524)	19,514 (17,596 to 21,646)	42,773 (34,255 to 58,657)	18,847 (14,224 to 28,734)	23,926 (19,064 to 31,023)	13.2 (-10.9 to 61.9)	3.1 (-25.7 to 79.1)	22.6 (-3.5 to 59.4)
Central Europe, eastern Europe, and central Asia	Central Asia	Tajikistan	YLLs	Age-standardized	1271.2 (1106 to 1378.7)	1095.1 (823.6 to 1234.9)	1527.9 (1377.2 to 1682.5)	887.4 (710.5 to 1273.7)	743.8 (555 to 1184.7)	1059.2 (860.3 to 1444.4)	-30.2 (-45.5 to 8.6)	-32.1 (-51.7 to 28.2)	-30.7 (-44.8 to -3.3)
Central Europe, eastern Europe, and central Asia	Central Asia	Tajikistan	YLDs	All Ages	9,796 (7,359 to 12,811)	5,357 (4,032 to 6,886)	4,439 (3,226 to 5,898)	16,120 (12,027 to 20,947)	8,505 (6,417 to 10,856)	7,615 (5,527 to 10,063)	64.6 (55.3 to 74.8)	58.8 (48.2 to 70.3)	71.6 (57.1 to 86.9)
Central Europe, eastern Europe, and central Asia	Central Asia	Tajikistan	YLDs	Age-standardized	251.1 (191.7 to 314.9)	267.3 (204.5 to 335.2)	235 (178.2 to 298.5)	210.9 (161.2 to 267.8)	221.3 (168.7 to 276.9)	200.3 (151.6 to 256)	-16 (-20.5 to -11.6)	-17.2 (-22.2 to -12.1)	-14.7 (-21 to -8)
Central Europe, eastern Europe, and central Asia	Central Asia	Turkmenistan	Incidence	All Ages	26,623 (22,515 to 32,357)	13,355 (11,413 to 16,103)	13,268 (11,095 to 16,340)	31,107 (25,800 to 37,220)	15,307 (12,726 to 18,304)	15,800 (13,012 to 19,241)	16.8 (7.7 to 25.9)	14.6 (4.9 to 25.2)	19.1 (8.8 to 29)
Central Europe, eastern Europe, and central Asia	Central Asia	Turkmenistan	Incidence	Age-standardized	803 (715.8 to 918.5)	784.2 (695.7 to 892.5)	830.3 (737.6 to 953.8)	635.9 (535 to 754)	622.5 (520.8 to 740)	647.9 (544.4 to 771.8)	-20.8 (-27.7 to -15.3)	-20.6 (-27 to -14.4)	-22 (-29.1 to -16)
Central Europe, eastern Europe, and central Asia	Central Asia	Turkmenistan	Prevalence	All Ages	151,612 (134,854 to 173,522)	77,958 (69,792 to 88,226)	73,654 (64,596 to 84,879)	156,804 (133,970 to 184,852)	76,193 (65,114 to 88,939)	80,612 (68,413 to 96,217)	3.4 (-8 to 12.3)	-2.3 (-13.2 to 7.7)	9.4 (-3.1 to 20.9)
Central Europe, eastern Europe, and central Asia	Central Asia	Turkmenistan	Prevalence	Age-standardized	5311.4 (4920.5 to 5773.3)	5224 (4833.3 to 5705.7)	5488.5 (5083.4 to 5968.7)	3297.5 (2839.5 to 3828.6)	3160.5 (2709.2 to 3668.3)	3453.4 (2967.8 to 4024.4)	-37.9 (-45.2 to -32.4)	-39.5 (-46.9 to -33.4)	-37.1 (-44.5 to -30.8)
Central Europe, eastern Europe, and central Asia	Central Asia	Turkmenistan	Deaths	All Ages	843 (653 to 912)	412 (236 to 465)	431 (404 to 462)	442 (323 to 806)	202 (129 to 531)	240 (188 to 308)	-47.6 (-62.6 to 12.3)	-50.8 (-70.6 to 84.2)	-44.4 (-56.8 to -28.9)
Central Europe, eastern Europe, and central Asia	Central Asia	Turkmenistan	Deaths	Age-standardized	50 (38.3 to 54.4)	40.6 (23.5 to 45.7)	65.9 (60.8 to 71)	13.3 (9.6 to 25)	10.4 (6.6 to 28)	18.1 (14.5 to 22.7)	-73.5 (-81.1 to -41.9)	-74.3 (-84.7 to -3.4)	-72.6 (-78.3 to -65.5)
Central Europe, eastern Europe, and central Asia	Central Asia	Turkmenistan	DALYs	All Ages	29,320 (24,113 to 32,381)	14,008 (9,281 to 15,911)	15,311 (13,964 to 16,760)	19,112 (15,425 to 27,119)	8,809 (6,732 to 15,735)	10,303 (8,347 to 12,476)	-34.8 (-47 to 5.5)	-37.1 (-52.1 to 50.3)	-32.7 (-44.5 to -19)
Central Europe, eastern Europe, and central Asia	Central Asia	Turkmenistan	DALYs	Age-standardized	1298.8 (1064.1 to 1409.2)	1097.8 (732.9 to 1236.6)	1598.5 (1487.4 to 1712.3)	444.1 (356.4 to 662.4)	386.8 (293 to 720.3)	521.9 (430.4 to 627.6)	-65.8 (-72.6 to -42.3)	-64.8 (-73.5 to -14)	-67.3 (-73.2 to -60.8)
Central Europe, eastern Europe, and central Asia	Central Asia	Turkmenistan	YLLs	All Ages	21,801 (16,928 to 23,585)	9,975 (5,385 to 11,333)	11,826 (10,975 to 12,742)	11,030 (8,072 to 18,844)	4,681 (3,048 to 11,461)	6,348 (4,887 to 8,292)	-49.4 (-63.7 to 3.8)	-53.1 (-71.7 to 77.3)	-46.3 (-59.2 to -30)
Central Europe, eastern Europe, and central Asia	Central Asia	Turkmenistan	YLLs	Age-standardized	1027.4 (799.1 to 1107.9)	820.3 (461 to 926.2)	1331.2 (1244 to 1425.5)	273.9 (201.1 to 483.9)	215.4 (139.4 to 542.1)	352.4 (277.2 to 456.4)	-73.3 (-81.1 to -44.5)	-73.7 (-84.2 to -1.7)	-73.5 (-79.4 to -65.9)
Central Europe, eastern Europe, and central Asia	Central Asia	Turkmenistan	YLDs	All Ages	7,519 (5,590 to 9,894)	4,034 (3,019 to 5,213)	3,485 (2,538 to 4,613)	8,082 (6,054 to 10,599)	4,128 (3,086 to 5,363)	3,954 (2,876 to 5,266)	7.5 (-2.4 to 15.9)	2.3 (-6.7 to 12.1)	13.5 (1.6 to 24.5)
Central Europe, eastern Europe, and central Asia	Central Asia	Turkmenistan	YLDs	Age-standardized	271.4 (207.9 to 339.3)	277.5 (211.8 to 345.9)	267.3 (202.9 to 338.4)	170.2 (128.8 to 219.9)	171.3 (128.8 to 221.4)	169.5 (124.4 to 220.5)	-37.3 (-43.3 to -32)	-38.2 (-44.3 to -32.5)	-36.6 (-43.3 to -30)
Central Europe, eastern Europe, and central Asia	Central Asia	Uzbekistan	Incidence	All Ages	192,263 (163,761 to 229,109)	101,496 (86,035 to 120,755)	90,767 (77,009 to 108,115)	257,478 (219,593 to 303,123)	129,145 (110,105 to 152,168)	128,333 (109,170 to 152,372)	33.9 (26 to 41.9)	27.2 (17.6 to 36.4)	41.4 (32.6 to 51.1)
Central Europe, eastern Europe, and central Asia	Central Asia	Uzbekistan	Incidence	Age-standardized	1110 (974.5 to 1254)	1118.7 (975.4 to 1268.8)	1108.7 (976.2 to 1258.1)	874.3 (763.8 to 1008)	842.2 (727.5 to 975.3)	923.6 (805.9 to 1061.1)	-21.2 (-26.2 to -16.1)	-24.7 (-30.5 to -19)	-16.7 (-22.2 to -10.4)
Central Europe, eastern Europe, and central Asia	Central Asia	Uzbekistan	Prevalence	All Ages	1,016,336 (902,586 to 1,153,537)	546,988 (484,552 to 618,155)	469,348 (411,131 to 539,229)	1,289,560 (1,125,948 to 1,502,375	661,115 (578,163 to 763,629)	628,445 (544,656 to 742,764)	26.9 (18.3 to 35.5)	20.9 (11.5 to 30)	33.9 (23.7 to 45.1)
Central Europe, eastern Europe, and central Asia	Central Asia	Uzbekistan	Prevalence	Age-standardized	6457.2 (5892.5 to 7070.2)	6571.9 (5949.2 to 7242.5)	6352.6 (5760.4 to 6963.4)	4512.6 (4042.9 to 5101)	4423.3 (3937.1 to 4998.7)	4673.2 (4176.5 to 5324.1)	-30.1 (-34.6 to -25.4)	-32.7 (-38.1 to -27.5)	-26.4 (-31.7 to -20.9)
Central Europe, eastern Europe, and central Asia	Central Asia	Uzbekistan	Deaths	All Ages	4,929 (3,868 to 5,379)	2,333 (1,332 to 2,690)	2,597 (2,377 to 2,822)	3,837 (3,035 to 5,646)	1,783 (1,329 to 3,393)	2,053 (1,641 to 2,571)	-22.2 (-40 to 32.3)	-23.6 (-46.5 to 109.7)	-20.9 (-36.8 to 0.8)
Central Europe, eastern Europe, and central Asia	Central Asia	Uzbekistan	Deaths	Age-standardized	46.8 (36.6 to 51.3)	37 (21.2 to 42.8)	62.7 (56.8 to 67.9)	29 (23.2 to 47.5)	24.9 (18.3 to 53.6)	34.7 (28.7 to 41.5)	-38.1 (-51.9 to 16.7)	-32.6 (-54 to 107)	-44.6 (-54.6 to -32.4)
Central Europe, eastern Europe, and central Asia	Central Asia	Uzbekistan	DALYs	All Ages	163,058 (136,019 to 183,276)	78,130 (55,395 to 90,129)	84,928 (76,169 to 94,411)	167,293 (138,562 to 212,306)	79,897 (64,392 to 116,652)	87,396 (72,566 to 105,055)	2.6 (-13.1 to 41.9)	2.3 (-17.4 to 88.4)	2.9 (-12.5 to 22.7)
Central Europe, eastern Europe, and central Asia	Central Asia	Uzbekistan	DALYs	Age-standardized	1281.6 (1058.1 to 1421.4)	1080.5 (743.1 to 1235.8)	1579.9 (1439.9 to 1727.8)	754.6 (626.4 to 1032.2)	669.2 (538.1 to 1082.5)	871.1 (736.1 to 1024.2)	-41.1 (-50.7 to -10.4)	-38.1 (-51.2 to 31)	-44.9 (-53.2 to -34.6)
Central Europe, eastern Europe, and central Asia	Central Asia	Uzbekistan	YLLs	All Ages	113,559 (89,687 to 123,860)	50,664 (27,989 to 57,867)	62,895 (57,428 to 69,050)	103,448 (81,945 to 144,139)	45,958 (34,217 to 81,597)	57,490 (45,615 to 72,089)	-8.9 (-30 to 48.8)	-9.3 (-36 to 142.6)	-8.6 (-27.8 to 17.6)
Central Europe, eastern Europe, and central Asia	Central Asia	Uzbekistan	YLLs	Age-standardized	960.3 (751.6 to 1046.7)	744.2 (407.3 to 854.4)	1275.2 (1163.6 to 1392.8)	530.7 (424.2 to 801.8)	442.6 (331.3 to 865.8)	647 (528.3 to 791.3)	-44.7 (-57 to -2.5)	-40.5 (-58.3 to 75.6)	-49.3 (-59.1 to -36.4)
Central Europe, eastern Europe, and central Asia	Central Asia	Uzbekistan	YLDs	All Ages	49,499 (36,329 to 65,902)	27,465 (20,223 to 35,915)	22,033 (15,773 to 29,488)	63,845 (46,461 to 84,718)	33,939 (24,913 to 44,493)	29,906 (21,513 to 40,215)	29 (19.5 to 37.9)	23.6 (13.4 to 33.4)	35.7 (24.2 to 47.9)
Central Europe, eastern Europe, and central Asia	Central Asia	Uzbekistan	YLDs	Age-standardized	321.3 (238.9 to 413.5)	336.2 (249.5 to 433.1)	304.7 (226.1 to 390.4)	223.9 (165.3 to 290.3)	226.6 (169.6 to 291.9)	224 (164.3 to 292.5)	-30.3 (-35 to -25.5)	-32.6 (-38 to -27.4)	-26.5 (-32.3 to -20.4)
		Central Europe	Incidence	All Ages	1,595,826 (1,422,833 to 1,798,785)	795,430 (704,715 to 897,197)	800,396 (717,000 to 904,316)	1,571,474 (1,412,004 to 1,755,041	798,533 (714,166 to 891,356)	772,942 (693,091 to 859,342)	-1.5 (-5.8 to 2.6)	0.4 (-4.3 to 5)	-3.4 (-7.8 to 0.9)
		Central Europe	Incidence	Age-standardized	1235.6 (1098.4 to 1407.6)	1177.9 (1038.3 to 1352.4)	1315.4 (1173.8 to 1501.3)	1173.1 (1024.6 to 1357.6)	1139.1 (996.1 to 1321.1)	1223.4 (1064.4 to 1420)	-5.1 (-8.7 to -1.7)	-3.3 (-6.7 to 0)	-7 (-11 to -3.2)
		Central Europe	Prevalence	All Ages	10,365,365 (9,352,293 to 11,670,307)	5,364,607 (4,787,095 to 6,082,744	5,000,758 (4,536,647 to 5,589,200)	9,408,225 (8,600,853 to 10,356,267	4,976,979 (4,499,400 to 5,513,411)	4,431,246 (4,084,720 to 4,834,732)	-9.2 (-12.7 to -4.9)	-7.2 (-10.9 to -3)	-11.4 (-15.3 to -6.9)
		Central Europe	Prevalence	Age-standardized	7765.7 (6952.8 to 8821.5)	7566.5 (6686.9 to 8688.5)	8160.9 (7383.9 to 9179.6)	6405.2 (5664.3 to 7297.9)	6396.9 (5586.4 to 7409.8)	6554.6 (5852.5 to 7516.7)	-17.5 (-21.3 to -13.9)	-15.5 (-19.1 to -11.9)	-19.7 (-23.7 to -15.6)
		Central Europe	Deaths	All Ages	50,505 (46,626 to 52,058)	17,078 (13,600 to 18,111)	33,427 (31,953 to 34,426)	42,773 (37,191 to 49,286)	16,592 (13,937 to 21,425)	26,181 (22,788 to 29,733)	-15.3 (-25.9 to -2.3)	-2.8 (-17.4 to 28.4)	-21.7 (-31.3 to -11.6)
		Central Europe	Deaths	Age-standardized	37 (33.9 to 38.2)	21.2 (16.8 to 22.6)	61.5 (58.2 to 63.5)	19.1 (16.6 to 22)	12.1 (10.2 to 15.4)	30.1 (26.3 to 34.1)	-48.3 (-54.7 to -40.3)	-43.2 (-51.7 to -25.8)	-51.2 (-57 to -45.2)
		Central Europe	DALYs	All Ages	1,511,302 (1,366,472 to 1,674,472)	565,958 (481,188 to 655,194)	945,345 (875,482 to 1,019,100)	1,240,537 (1,091,708 to 1,395,421	521,341 (444,854 to 614,062)	719,196 (633,204 to 806,491)	-17.9 (-24.5 to -10.9)	-7.9 (-15.6 to 4.5)	-23.9 (-30.9 to -16.8)
		Central Europe	DALYs	Age-standardized	1072.4 (963.3 to 1197.1)	737.4 (620.8 to 868.6)	1545.9 (1430.5 to 1667.9)	678 (583.5 to 787.4)	537.4 (445.2 to 646.7)	876.3 (765.4 to 991.4)	-36.8 (-41.6 to -31.9)	-27.1 (-32.8 to -19.4)	-43.3 (-48.2 to -38.6)
		Central Europe	YLLs	All Ages	988,167 (922,873 to 1,013,765)	302,531 (241,730 to 318,775)	685,636 (662,935 to 705,262)	728,058 (628,465 to 832,955)	256,627 (218,318 to 319,947)	471,431 (405,410 to 539,918)	-26.3 (-36.1 to -16.2)	-15.2 (-27.7 to 6.7)	-31.2 (-40.5 to -21.8)
		Central Europe	YLLs	Age-standardized	683.5 (636 to 701.6)	369.1 (294.2 to 388.3)	1122.2 (1078.2 to 1154.6)	342.7 (295.2 to 392.1)	208.7 (179.5 to 258.2)	524.4 (450.5 to 599.1)	-49.9 (-56.6 to -42.9)	-43.4 (-51.8 to -29.6)	-53.3 (-59.4 to -47)
		Central Europe	YLDs	All Ages	523,135 (393,601 to 674,730)	263,426 (193,456 to 346,277)	259,709 (195,978 to 330,635)	512,480 (399,007 to 631,808)	264,714 (202,432 to 332,026)	247,766 (193,930 to 305,050)	-2 (-8.5 to 5.5)	0.5 (-6.9 to 9)	-4.6 (-11.7 to 3)
		Central Europe	YLDs	Age-standardized	388.8 (289.3 to 507.9)	368.3 (268.6 to 492)	423.6 (317.7 to 536.9)	335.3 (252.1 to 428.3)	328.6 (242.6 to 433.1)	351.9 (269.7 to 443.5)	-13.8 (-17.6 to -9.9)	-10.8 (-14.9 to -6.4)	-16.9 (-21 to -13)
Central Europe, eastern Europe, and central Asia	Central Europe	Albania	Incidence	All Ages	25,249 (22,107 to 29,360)	11,264 (9,800 to 13,125)	13,985 (12,210 to 16,454)	29,867 (26,351 to 33,622)	14,020 (12,063 to 16,066)	15,847 (14,251 to 17,676)	18.3 (7.3 to 29.1)	24.5 (12.4 to 36.5)	13.3 (2 to 25.1)
Central Europe, eastern Europe, and central Asia	Central Europe	Albania	Incidence	Age-standardized	888.6 (793 to 1005.2)	785.7 (689.4 to 895.6)	1011.3 (906 to 1141.1)	955.3 (831.1 to 1097.6)	892.1 (764.9 to 1037.6)	1026.2 (901 to 1169.2)	7.5 (1.7 to 14.6)	13.5 (6 to 22.1)	1.5 (-5.1 to 9.4)
Central Europe, eastern Europe, and central Asia	Central Europe	Albania	Prevalence	All Ages	114,846 (101,318 to 134,318)	50,280 (44,166 to 58,869)	64,566 (56,664 to 76,663)	138,322 (124,894 to 153,828)	63,125 (54,023 to 72,458)	75,196 (68,361 to 83,522)	20.4 (10.2 to 31.2)	25.5 (10.2 to 40.9)	16.5 (5.7 to 28.8)
Central Europe, eastern Europe, and central Asia	Central Europe	Albania	Prevalence	Age-standardized	4217.4 (3837 to 4739.9)	3532.4 (3167.5 to 4022.7)	5112.8 (4666.7 to 5733.6)	4318.6 (3803 to 4971)	3966.3 (3404.7 to 4681.3)	4760.8 (4235.4 to 5440.8)	2.4 (-4.9 to 10.6)	12.3 (1.1 to 23.6)	-6.9 (-13.6 to 1.9)
Central Europe, eastern Europe, and central Asia	Central Europe	Albania	Deaths	All Ages	1,006 (695 to 1,096)	360 (204 to 410)	646 (469 to 708)	815 (608 to 1,053)	279 (186 to 388)	536 (403 to 701)	-19 (-41 to 30.1)	-22.4 (-50.5 to 50.3)	-17.1 (-39.6 to 22.5)
Central Europe, eastern Europe, and central Asia	Central Europe	Albania	Deaths	Age-standardized	59.1 (39.6 to 64.7)	35.6 (19.9 to 40.8)	96.8 (67.3 to 106.5)	19.5 (14.6 to 25.2)	12.1 (8.1 to 16.7)	29.7 (22.4 to 38.6)	-66.9 (-75.9 to -46)	-66.1 (-78.1 to -33.6)	-69.4 (-77.5 to -53.8)
Central Europe, eastern Europe, and central Asia	Central Europe	Albania	DALYs	All Ages	24,582 (19,699 to 27,109)	8,706 (6,376 to 9,857)	15,876 (12,781 to 17,551)	19,951 (16,193 to 24,255)	7,411 (5,845 to 9,326)	12,540 (10,054 to 15,595)	-18.8 (-34.1 to 11.8)	-14.9 (-33.2 to 25.3)	-21 (-37.5 to 7.2)
Central Europe, eastern Europe, and central Asia	Central Europe	Albania	DALYs	Age-standardized	1179.9 (906.6 to 1288.3)	745.6 (528.6 to 841.5)	1765.3 (1349.4 to 1932.7)	527.3 (430.7 to 642.2)	395.4 (311.9 to 498.1)	689.3 (557.7 to 842.7)	-55.3 (-63.5 to -37.7)	-47 (-58.6 to -19.4)	-61 (-68.9 to -46)
Central Europe, eastern Europe, and central Asia	Central Europe	Albania	YLLs	All Ages	18,770 (14,017 to 20,247)	6,158 (3,938 to 6,916)	12,611 (9,622 to 13,766)	12,562 (9,292 to 16,694)	4,093 (2,715 to 5,866)	8,469 (6,315 to 11,440)	-33.1 (-52.2 to 5.3)	-33.5 (-57 to 22.5)	-32.8 (-51.8 to 0.6)
Central Europe, eastern Europe, and central Asia	Central Europe	Albania	YLLs	Age-standardized	960.5 (693.3 to 1042.1)	563.5 (347.4 to 634.4)	1495.1 (1087.3 to 1631.1)	302 (224.1 to 397.4)	190.6 (128.2 to 274.3)	437.1 (328.8 to 579.4)	-68.6 (-77.3 to -48.7)	-66.2 (-78.2 to -35.5)	-70.8 (-78.8 to -55.6)
Central Europe, eastern Europe, and central Asia	Central Europe	Albania	YLDs	All Ages	5,812 (4,354 to 7,634)	2,548 (1,888 to 3,345)	3,265 (2,424 to 4,320)	7,390 (5,586 to 9,304)	3,318 (2,460 to 4,269)	4,072 (3,086 to 5,090)	27.1 (15.6 to 38.2)	30.2 (16.7 to 45.3)	24.7 (11.6 to 39.4)
Central Europe, eastern Europe, and central Asia	Central Europe	Albania	YLDs	Age-standardized	219.4 (166.8 to 279.5)	182.1 (137.4 to 231.7)	270.1 (204.9 to 344.6)	225.3 (167.1 to 292.1)	204.7 (148.9 to 269.8)	252.3 (189.7 to 322.4)	2.7 (-3.9 to 10.4)	12.4 (2 to 23.6)	-6.6 (-13.4 to 1.2)
Central Europe, eastern Europe, and central Asia	Central Europe	Bosnia and Herzegovina	Incidence	All Ages	48,040 (42,317 to 55,504)	23,091 (20,204 to 26,462)	24,949 (21,920 to 29,300)	44,485 (39,980 to 49,792)	21,909 (19,391 to 24,788)	22,576 (20,298 to 25,273)	-7.4 (-14 to -0.9)	-5.1 (-12.5 to 1.8)	-9.5 (-16.5 to -2.6)
Central Europe, eastern Europe, and central Asia	Central Europe	Bosnia and Herzegovina	Incidence	Age-standardized	1104.4 (972.4 to 1274)	1013.8 (891 to 1165.4)	1202.7 (1056.3 to 1397.1)	1151.2 (1007.9 to 1339.9)	1061.1 (923.7 to 1223.8)	1250.5 (1085.2 to 1466.8)	4.2 (0.6 to 7.7)	4.2 (-0.8 to 9.2)	4 (-0.7 to 8.6)
Central Europe, eastern Europe, and central Asia	Central Europe	Bosnia and Herzegovina	Prevalence	All Ages	285,746 (252,266 to 326,881)	135,670 (119,713 to 155,317)	150,076 (131,828 to 173,980)	269,265 (243,210 to 300,772)	128,928 (114,808 to 144,255)	140,336 (126,828 to 157,633)	-5.8 (-11.5 to 0)	-5 (-11.6 to 2.1)	-6.5 (-12.9 to 0.1)
Central Europe, eastern Europe, and central Asia	Central Europe	Bosnia and Herzegovina	Prevalence	Age-standardized	6619.1 (5908.3 to 7522.3)	5953.8 (5275.3 to 6787.4)	7484.1 (6661.1 to 8521.5)	6449.2 (5670.7 to 7466.5)	5785.2 (5047.3 to 6692.5)	7248.3 (6382.9 to 8412.9)	-2.6 (-6.7 to 1.6)	-2.8 (-8.6 to 2.8)	-3.2 (-8.1 to 2.1)
Central Europe, eastern Europe, and central Asia	Central Europe	Bosnia and Herzegovina	Deaths	All Ages	1,162 (997 to 1,244)	493 (358 to 552)	669 (605 to 724)	1,204 (953 to 1,487)	457 (328 to 596)	747 (590 to 926)	3.6 (-17.5 to 29.1)	-7.2 (-30.6 to 30.2)	11.6 (-10.7 to 39.6)
Central Europe, eastern Europe, and central Asia	Central Europe	Bosnia and Herzegovina	Deaths	Age-standardized	36.3 (30.8 to 39.1)	26.9 (19.3 to 30.4)	50.4 (44.9 to 54.6)	21 (16.6 to 25.7)	13.7 (9.9 to 17.9)	31.5 (25.2 to 38.8)	-42.2 (-54 to -27.9)	-49.2 (-61.9 to -27)	-37.5 (-50.2 to -22.8)
Central Europe, eastern Europe, and central Asia	Central Europe	Bosnia and Herzegovina	DALYs	All Ages	37,307 (32,454 to 41,834)	15,957 (13,395 to 18,350)	21,350 (18,854 to 24,049)	33,919 (28,376 to 40,118)	13,640 (11,062 to 16,352)	20,279 (16,921 to 23,995)	-9.1 (-20.7 to 4.9)	-14.5 (-27.1 to 2.6)	-5 (-18.6 to 11.5)
Central Europe, eastern Europe, and central Asia	Central Europe	Bosnia and Herzegovina	DALYs	Age-standardized	956.1 (834.6 to 1062.3)	740.9 (612.2 to 847.8)	1259.1 (1123.6 to 1390.3)	662.3 (551.6 to 786.3)	500.9 (406 to 610.6)	874.6 (725.6 to 1037)	-30.7 (-39.1 to -20.9)	-32.4 (-41.9 to -18.4)	-30.5 (-39.9 to -20.1)
Central Europe, eastern Europe, and central Asia	Central Europe	Bosnia and Herzegovina	YLLs	All Ages	23,053 (20,247 to 24,499)	9,167 (7,058 to 10,139)	13,886 (12,779 to 15,013)	19,853 (15,721 to 24,856)	6,960 (5,082 to 9,217)	12,893 (10,036 to 16,201)	-13.9 (-32 to 8.4)	-24.1 (-43.5 to 4.7)	-7.1 (-27 to 18.4)
Central Europe, eastern Europe, and central Asia	Central Europe	Bosnia and Herzegovina	YLLs	Age-standardized	624.3 (542.7 to 666.4)	443 (333.2 to 491.9)	881.8 (803.3 to 953.3)	335.4 (266.4 to 418.7)	208 (152.9 to 278.8)	504.9 (396.8 to 632.1)	-46.3 (-57 to -32.7)	-53 (-64.8 to -32.6)	-42.7 (-54.5 to -28.2)
Central Europe, eastern Europe, and central Asia	Central Europe	Bosnia and Herzegovina	YLDs	All Ages	14,254 (10,546 to 18,594)	6,790 (5,034 to 8,855)	7,464 (5,454 to 9,770)	14,065 (10,812 to 17,653)	6,680 (5,036 to 8,462)	7,385 (5,578 to 9,305)	-1.3 (-8.3 to 6.5)	-1.6 (-9.3 to 7)	-1.1 (-9.8 to 8.9)
Central Europe, eastern Europe, and central Asia	Central Europe	Bosnia and Herzegovina	YLDs	Age-standardized	331.8 (247 to 427.6)	297.9 (221.4 to 387.3)	377.3 (279.7 to 489)	326.9 (246.1 to 424.8)	292.9 (216.3 to 382.9)	369.7 (273.5 to 478.9)	-1.5 (-5.5 to 2.7)	-1.7 (-7.5 to 4.2)	-2 (-7.5 to 3.5)
Central Europe, eastern Europe, and central Asia	Central Europe	Bulgaria	Incidence	All Ages	97,841 (86,732 to 110,088)	48,426 (42,058 to 55,263)	49,414 (44,078 to 55,840)	85,538 (76,213 to 95,941)	44,767 (39,340 to 50,889)	40,771 (36,485 to 45,311)	-12.6 (-17.3 to -7.3)	-7.6 (-13.7 to -1.1)	-17.5 (-22.8 to -11.6)
Central Europe, eastern Europe, and central Asia	Central Europe	Bulgaria	Incidence	Age-standardized	1035.8 (912 to 1187.9)	997.9 (867.3 to 1150.9)	1082.4 (951.9 to 1250.2)	1042.9 (906.8 to 1203.9)	1036.4 (897.7 to 1210.2)	1058.6 (917.2 to 1227)	0.7 (-3.9 to 5.1)	3.9 (-1.9 to 9.9)	-2.2 (-7.6 to 3.3)
Central Europe, eastern Europe, and central Asia	Central Europe	Bulgaria	Prevalence	All Ages	648,021 (588,008 to 721,506)	319,169 (281,944 to 364,437)	328,852 (300,843 to 361,703)	571,762 (524,260 to 622,523)	302,218 (275,505 to 331,261)	269,543 (248,525 to 293,373)	-11.8 (-17 to -5.3)	-5.3 (-12.5 to 3.3)	-18 (-22.8 to -11.8)
Central Europe, eastern Europe, and central Asia	Central Europe	Bulgaria	Prevalence	Age-standardized	6421.4 (5748.6 to 7224.6)	6149.3 (5363.9 to 7088.7)	6785.6 (6142.1 to 7637.3)	6042.6 (5362.4 to 6817.9)	6034.6 (5299 to 6916.4)	6174.3 (5502.4 to 6999.4)	-5.9 (-10.7 to -0.3)	-1.9 (-8.2 to 5.7)	-9 (-14 to -3.1)
Central Europe, eastern Europe, and central Asia	Central Europe	Bulgaria	Deaths	All Ages	3,786 (3,436 to 4,056)	1,266 (944 to 1,412)	2,520 (2,304 to 2,745)	3,036 (2,405 to 3,809)	1,183 (887 to 1,666)	1,853 (1,458 to 2,317)	-19.8 (-37.4 to 2.3)	-6.6 (-32.6 to 44.4)	-26.5 (-43.1 to -7.3)
Central Europe, eastern Europe, and central Asia	Central Europe	Bulgaria	Deaths	Age-standardized	35.2 (31.5 to 37.7)	22.3 (16.5 to 25)	51.5 (46.9 to 56.2)	20.5 (16.2 to 25.6)	13.1 (9.9 to 18.3)	31.4 (24.7 to 38.9)	-41.7 (-54.4 to -25.3)	-41.1 (-56.9 to -10.7)	-39 (-52.5 to -23.8)
Central Europe, eastern Europe, and central Asia	Central Europe	Bulgaria	DALYs	All Ages	111,059 (100,061 to 121,998)	39,579 (33,090 to 45,353)	71,480 (64,884 to 78,157)	83,661 (70,107 to 99,653)	34,399 (28,208 to 42,308)	49,263 (40,341 to 59,742)	-24.7 (-36.2 to -10.6)	-13.1 (-26.7 to 11.4)	-31.1 (-43.4 to -16.2)
Central Europe, eastern Europe, and central Asia	Central Europe	Bulgaria	DALYs	Age-standardized	975.7 (876.5 to 1082.4)	679.7 (570.7 to 791.2)	1328.3 (1205.5 to 1456.2)	692.7 (575.7 to 818.5)	537.6 (439 to 658.8)	902.9 (747.1 to 1087.2)	-29 (-38.9 to -17.8)	-20.9 (-31.7 to -2.7)	-32 (-43.3 to -19.1)
Central Europe, eastern Europe, and central Asia	Central Europe	Bulgaria	YLLs	All Ages	77,485 (70,237 to 83,545)	23,686 (17,742 to 26,263)	53,799 (48,709 to 58,848)	52,034 (40,601 to 66,305)	18,002 (13,517 to 25,581)	34,032 (26,115 to 43,634)	-32.8 (-48.7 to -13.5)	-24 (-45.5 to 17.7)	-36.7 (-52.3 to -17.9)
Central Europe, eastern Europe, and central Asia	Central Europe	Bulgaria	YLLs	Age-standardized	648.7 (587.5 to 697.4)	377.1 (280.9 to 417.7)	970.1 (886.7 to 1053.1)	371.6 (288.7 to 472.7)	221.3 (166.8 to 308.4)	568.6 (438.6 to 724.8)	-42.7 (-56.1 to -25.8)	-41.3 (-57.8 to -10.8)	-41.4 (-55.7 to -24.5)
[illegible]	[illegible]	[illegible]	[illegible]	[illegible]	[illegible]	[illegible]	[illegible]	[illegible]	[illegible]	[illegible]	[illegible]	[illegible]	[illegible]

Chronic respiratory diseases

7 GBD super region	21 GBD region	Location	Measure	Age	1990 Both	1990 Female	1990 Male	2019 Both	2019 Female	2019 Male	Percent change (1990 to 2019) Both	Percent change (1990 to 2019) Female	Percent change (1990 to 2019) Male
Central Europe, eastern Europe, and central Asia	Eastern Europe	Republic of Moldova	YLLs	Age-standardized	930.3 (775.9 to 994.4)	533.1 (300.4 to 600)	1508.8 (1416.9 to 1605.3)	302.9 (253.3 to 381.1)	162.4 (126.3 to 265.2)	506.6 (428.7 to 596.4)	-67.4 (-73.2 to -55.3)	-69.5 (-77.5 to -28.7)	-66.4 (-71.9 to -59.9)
Central Europe, eastern Europe, and central Asia	Eastern Europe	Republic of Moldova	YLDs	All Ages	13,899 (10,669 to 17,543)	7,711 (5,945 to 9,729)	6,188 (4,589 to 8,067)	9,895 (7,487 to 12,341)	5,149 (3,954 to 6,434)	4,745 (3,538 to 6,078)	-28.8 (-34.6 to -23.7)	-33.2 (-39 to -27.2)	-23.3 (-30.6 to -16.7)
Central Europe, eastern Europe, and central Asia	Eastern Europe	Republic of Moldova	YLDs	Age-standardized	315.2 (242.5 to 396.1)	313.5 (241.9 to 394.3)	317.6 (235.4 to 407.7)	226.6 (170 to 292.9)	216 (162.2 to 280)	242.7 (179.1 to 316.6)	-28.1 (-33.2 to -23.3)	-31.1 (-37.5 to -24.6)	-23.6 (-29.3 to -18.6)
Central Europe, eastern Europe, and central Asia	Eastern Europe	Russian Federation	Incidence	All Ages	1,726,366 (1,520,295 to 1,983,980)	926,405 (814,183 to 1,059,961)	799,961 (704,524 to 927,180)	1,303,724 (1,141,634 to 1,496,166)	694,118 (603,559 to 804,681)	609,606 (534,606 to 703,464)	-24.5 (-28.7 to -20.5)	-25.1 (-29.8 to -20.5)	-23.8 (-27.7 to -20.2)
Central Europe, eastern Europe, and central Asia	Eastern Europe	Russian Federation	Incidence	Age-standardized	1096.5 (955.5 to 1273.3)	1076.1 (927.9 to 1252.6)	1158.9 (1019.3 to 1336.5)	839.9 (712.1 to 1000.9)	826.5 (694.6 to 981.7)	865.4 (739.5 to 1027.4)	-23.4 (-27.4 to -19.5)	-23.2 (-27.4 to -19.4)	-25.3 (-29.1 to -21.5)
Central Europe, eastern Europe, and central Asia	Eastern Europe	Russian Federation	Prevalence	All Ages	11,030,338 (9,871,557 to 12,323,904)	6,164,111 (5,485,970 to 6,964,755	4,866,227 (4,367,887 to 5,426,182)	7,290,000 (6,611,072 to 8,062,459)	3,636,370 (3,267,658 to 4,022,916)	3,653,630 (3,329,583 to 4,042,674)	-33.9 (-36.7 to -30.9)	-41 (-43.7 to -38.3)	-24.9 (-28.3 to -21.1)
Central Europe, eastern Europe, and central Asia	Eastern Europe	Russian Federation	Prevalence	Age-standardized	6729.4 (5985.5 to 7567.2)	6536.7 (5757.4 to 7439.8)	7353.8 (6629.8 to 8141.2)	4271.8 (3765.8 to 4919.1)	3922 (3413.5 to 4571.8)	4906.2 (4388.2 to 5555.7)	-36.5 (-40 to -32.9)	-40 (-43.6 to -36.3)	-33.3 (-36.4 to -29.8)
Central Europe, eastern Europe, and central Asia	Eastern Europe	Russian Federation	Deaths	All Ages	57,982 (47,061 to 62,626)	22,780 (12,508 to 25,384)	35,202 (34,248 to 37,528)	39,390 (33,695 to 46,788)	14,638 (11,956 to 20,747)	24,753 (20,435 to 29,575)	-32.1 (-42.3 to -8.2)	-35.7 (-49.7 to 30.6)	-29.7 (-41.4 to -16.3)
Central Europe, eastern Europe, and central Asia	Eastern Europe	Russian Federation	Deaths	Age-standardized	34.6 (27.5 to 37.4)	20 (10.9 to 22.3)	70.6 (67.9 to 75.1)	16.7 (14.3 to 19.9)	9.1 (7.5 to 13.1)	31.7 (26.5 to 37.7)	-51.7 (-58.8 to -34)	-54.3 (-64.3 to -6.7)	-55.1 (-62.1 to -47.2)
Central Europe, eastern Europe, and central Asia	Eastern Europe	Russian Federation	DALYs	All Ages	1,769,632 (1,520,141 to 1,961,919)	722,157 (520,205 to 838,252)	1,047,475 (980,994 to 1,129,230)	1,110,124 (973,894 to 1,280,631)	435,708 (370,918 to 540,268)	674,416 (577,838 to 788,993)	-37.3 (-44 to -24)	-39.7 (-47.6 to -8.7)	-35.6 (-44.1 to -25.6)
Central Europe, eastern Europe, and central Asia	Eastern Europe	Russian Federation	DALYs	Age-standardized	1016.2 (866.3 to 1135.9)	686.6 (502.4 to 807.3)	1695.7 (1587.8 to 1820)	534.8 (465.4 to 621.5)	373.6 (312.7 to 463.2)	815.1 (697.6 to 948.9)	-47.4 (-52.9 to -36.8)	-45.6 (-52.1 to -22.1)	-51.9 (-57.9 to -45.1)
Central Europe, eastern Europe, and central Asia	Eastern Europe	Russian Federation	YLLs	All Ages	1,237,884 (1,038,579 to 1,341,707)	410,734 (219,054 to 460,825)	827,149 (803,911 to 886,937)	729,013 (617,921 to 865,355)	228,888 (184,997 to 332,531)	500,126 (410,962 to 607,388)	-41.1 (-50.6 to -21.9)	-44.3 (-57.2 to 18.6)	-39.5 (-50.1 to -27.4)
Central Europe, eastern Europe, and central Asia	Eastern Europe	Russian Federation	YLLs	Age-standardized	692.4 (575 to 750.8)	359.7 (191.1 to 405.3)	1362 (1324 to 1455.6)	315.4 (267.6 to 374.6)	157.7 (126.8 to 230.8)	583.2 (480.7 to 703.4)	-54.4 (-61.6 to -39.3)	-56.2 (-66.6 to -5.8)	-57.2 (-64.3 to -48.6)
Central Europe, eastern Europe, and central Asia	Eastern Europe	Russian Federation	YLDs	All Ages	531,748 (400,854 to 672,809)	311,423 (234,161 to 395,364)	220,325 (163,540 to 285,827)	381,111 (295,050 to 470,574)	206,821 (161,578 to 254,097)	174,290 (130,428 to 223,955)	-28.3 (-33.8 to -22)	-33.6 (-38.4 to -28)	-20.9 (-28.4 to -13)
Central Europe, eastern Europe, and central Asia	Eastern Europe	Russian Federation	YLDs	Age-standardized	323.8 (242.5 to 414.8)	326.9 (244.2 to 421.2)	333.8 (246.2 to 433.4)	219.4 (166.7 to 279.3)	215.9 (163.4 to 278)	231.9 (173.4 to 300.8)	-32.3 (-36.3 to -28)	-33.9 (-37.9 to -29.7)	-30.5 (-34.6 to -26.5)
Central Europe, eastern Europe, and central Asia	Eastern Europe	Ukraine	Incidence	All Ages	790,133 (697,475 to 903,193)	415,147 (364,053 to 468,395)	374,986 (328,567 to 434,168)	412,669 (358,811 to 474,955)	206,622 (178,090 to 238,939)	206,047 (179,388 to 238,360)	-47.8 (-50.3 to -45.1)	-50.2 (-53.3 to -46.9)	-45.1 (-48.3 to -41.4)
Central Europe, eastern Europe, and central Asia	Eastern Europe	Ukraine	Incidence	Age-standardized	1383.2 (1202.5 to 1608.8)	1272.4 (1110.1 to 1474.2)	1522.8 (1318.1 to 1779.9)	945.7 (781.3 to 1147.4)	854 (699.5 to 1032.7)	1052.1 (874.1 to 1287.4)	-31.6 (-36.5 to -26.1)	-32.9 (-38 to -28)	-30.9 (-36.4 to -24.5)
Central Europe, eastern Europe, and central Asia	Eastern Europe	Ukraine	Prevalence	All Ages	4,623,565 (4,195,012 to 5,108,837)	2,536,494 (2,320,661 to 2,786,326	2,087,071 (1,851,376 to 2,349,322)	2,498,061 (2,237,945 to 2,803,899)	1,129,918 (1,000,039 to 1,283,021)	1,368,143 (1,222,220 to 1,528,507)	-46 (-48.7 to -43)	-55.5 (-58.4 to -51.7)	-34.4 (-38.7 to -29.6)
Central Europe, eastern Europe, and central Asia	Eastern Europe	Ukraine	Prevalence	Age-standardized	7802.8 (6964 to 8795.7)	7192.7 (6439.4 to 8091.1)	8601.2 (7603 to 9724.9)	5003 (4305.2 to 5828.7)	4137.6 (3507.6 to 4847.8)	6201.4 (5411.5 to 7165.8)	-35.9 (-40.2 to -31.4)	-42.5 (-46.9 to -37.4)	-27.9 (-33.1 to -23.1)
Central Europe, eastern Europe, and central Asia	Eastern Europe	Ukraine	Deaths	All Ages	33,452 (26,048 to 36,237)	13,518 (6,683 to 15,618)	19,934 (18,690 to 21,169)	12,181 (9,742 to 18,471)	4,393 (3,005 to 10,285)	7,788 (6,178 to 9,601)	-63.6 (-71.8 to -35.1)	-67.5 (-79 to 23.5)	-60.9 (-69.7 to -51.3)
Central Europe, eastern Europe, and central Asia	Eastern Europe	Ukraine	Deaths	Age-standardized	49.8 (38.1 to 54.1)	29.8 (14.8 to 34.6)	92.9 (86.7 to 98.8)	16 (12.8 to 24.2)	8.6 (5.8 to 20)	29.4 (23.5 to 35.9)	-67.9 (-75.2 to -42.8)	-71.3 (-81.3 to 9.3)	-68.3 (-75.1 to -61.2)
Central Europe, eastern Europe, and central Asia	Eastern Europe	Ukraine	DALYs	All Ages	908,328 (765,428 to 990,023)	376,368 (258,161 to 427,850)	531,960 (494,601 to 573,157)	364,900 (305,132 to 474,322)	134,272 (104,682 to 230,009)	230,628 (189,856 to 277,698)	-59.8 (-66.4 to -43.1)	-64.3 (-72.2 to -21.5)	-56.6 (-64.6 to -48)
Central Europe, eastern Europe, and central Asia	Eastern Europe	Ukraine	DALYs	Age-standardized	1347.4 (1127.9 to 1486.1)	907.2 (640.1 to 1043)	2144.4 (1992.3 to 2305.3)	575.4 (476.2 to 733.5)	376.9 (291.1 to 582.5)	883 (729.6 to 1062.3)	-57.3 (-64 to -41.3)	-58.4 (-66.8 to -18.9)	-58.8 (-65.9 to -51.4)
Central Europe, eastern Europe, and central Asia	Eastern Europe	Ukraine	YLLs	All Ages	662,399 (538,803 to 711,275)	226,789 (113,144 to 258,004)	435,609 (408,692 to 465,530)	235,863 (186,383 to 342,923)	69,541 (47,374 to 165,337)	166,322 (130,372 to 208,990)	-64.4 (-72.8 to -40.8)	-69.3 (-80.3 to 17.6)	-61.8 (-71 to -51.7)
Central Europe, eastern Europe, and central Asia	Eastern Europe	Ukraine	YLLs	Age-standardized	941.4 (762.9 to 1010.1)	499.9 (251.4 to 568)	1748.1 (1644 to 1855.5)	324.3 (257 to 469.3)	151.4 (103.2 to 355.7)	595.1 (471.8 to 740.9)	-65.6 (-73.6 to -43.1)	-69.7 (-80.6 to 15.7)	-66 (-73.7 to -57.3)
Central Europe, eastern Europe, and central Asia	Eastern Europe	Ukraine	YLDs	All Ages	245,929 (190,387 to 303,344)	149,579 (116,391 to 183,027)	96,350 (71,129 to 125,707)	129,037 (99,139 to 161,473)	64,731 (49,620 to 79,963)	64,306 (48,073 to 81,990)	-47.5 (-50.6 to -44.2)	-56.7 (-60.3 to -52.6)	-33.3 (-38.9 to -27.5)
[illegible]	[illegible]	[illegible]	[illegible]	[illegible]	[illegible]	[illegible]	[illegible]	[illegible]	[illegible]	[illegible]	[illegible]	[illegible]	[illegible]

Chronic respiratory diseases

7 GBD super region	21 GBD region	Location	Measure	Age	1990			2019			Percent change (1990 to 2019)		
					Both	Female	Male	Both	Female	Male	Both	Female	Male
High income	Western Europe	Ireland	YLLs	All Ages	39,196 (34,928 to 41,446)	15,001 (10,983 to 16,409)	24,195 (22,689 to 25,729)	35,307 (30,438 to 39,322)	16,146 (12,093 to 18,920)	19,161 (16,688 to 21,732)	-9.9 (-19.9 to 1.3)	7.6 (-8 to 29.6)	-20.8 (-32 to -7.7)
High income	Western Europe	Ireland	YLLs	Age-standardized	941.6 (839.8 to 994.4)	653.6 (481.8 to 713.6)	1340.4 (1260.2 to 1421)	463.2 (400.4 to 514.4)	391.5 (298.5 to 455.5)	555.1 (483.6 to 630.8)	-50.8 (-56.1 to -44.9)	-40.1 (-48.3 to -27.6)	-58.6 (-64.2 to -51.7)
High income	Western Europe	Ireland	YLDs	All Ages	25,129 (18,586 to 32,492)	13,069 (9,747 to 16,833)	12,060 (8,799 to 15,951)	30,438 (23,320 to 38,448)	16,522 (12,574 to 20,843)	13,916 (10,514 to 17,629)	21.1 (13.1 to 29.7)	26.4 (17.4 to 35.4)	15.4 (5.5 to 26.4)
High income	Western Europe	Ireland	YLDs	Age-standardized	660.1 (486 to 861.4)	653.9 (483.4 to 853.1)	665.9 (488.1 to 878.2)	506.2 (378.7 to 656.3)	523.5 (388.9 to 682.3)	487.3 (358.7 to 642.5)	-23.3 (-27.6 to -18.5)	-19.9 (-25.2 to -14.2)	-26.8 (-32.4 to -21)
High income	Western Europe	Israel	Incidence	All Ages	35,599 (30,293 to 41,758)	17,697 (15,086 to 20,860)	17,902 (14,883 to 21,447)	60,919 (51,666 to 70,762)	31,082 (26,704 to 35,703)	29,837 (24,844 to 35,328)	71.1 (61.5 to 80.6)	75.6 (62.8 to 88.1)	66.7 (54.5 to 78.2)
High income	Western Europe	Israel	Incidence	Age-standardized	715.3 (617.1 to 833.6)	698.7 (599.4 to 816.4)	730 (618 to 858.8)	619.5 (518.5 to 730.6)	613.7 (516.3 to 719.2)	621.1 (513.7 to 740.3)	-13.4 (-18.6 to -8.5)	-12.2 (-18.6 to -6.1)	-14.9 (-21.3 to -8.6)
High income	Western Europe	Israel	Prevalence	All Ages	415,288 (367,668 to 470,621)	205,703 (182,009 to 233,877)	209,585 (184,066 to 240,022)	687,888 (613,391 to 778,410)	350,585 (312,000 to 395,770)	337,304 (298,347 to 384,087)	65.6 (56.3 to 74.8)	70.4 (59.3 to 82.4)	60.9 (50.4 to 71.7)
High income	Western Europe	Israel	Prevalence	Age-standardized	8575.4 (7594.6 to 9653.6)	8225.5 (7266.7 to 9317.4)	8939.6 (7898.5 to 10139.7)	6935.5 (6117.9 to 7941.8)	6843.3 (6000.6 to 7867.9)	7025.8 (6177.8 to 8057.4)	-19.1 (-23.9 to -14.4)	-16.8 (-22.5 to -10.3)	-21.4 (-26.6 to -16)
High income	Western Europe	Israel	Deaths	All Ages	1,328 (1,222 to 1,429)	578 (504 to 651)	749 (698 to 807)	2,027 (1,763 to 2,482)	967 (781 to 1,364)	1,060 (921 to 1,217)	52.7 (35.4 to 79)	67.1 (38.5 to 116.3)	41.5 (23.7 to 65)
High income	Western Europe	Israel	Deaths	Age-standardized	29.4 (26.8 to 31.8)	23.7 (20.3 to 26.6)	36.1 (33.4 to 38.8)	16.2 (14.2 to 19.8)	13.2 (10.7 to 18.6)	19.8 (17.3 to 22.7)	-44.9 (-51 to -34.7)	-44.4 (-53.7 to -25.9)	-45 (-52 to -36.3)
High income	Western Europe	Israel	DALYs	All Ages	43,068 (37,291 to 49,997)	19,654 (16,540 to 23,096)	23,415 (20,531 to 27,176)	64,747 (55,570 to 76,231)	30,611 (25,264 to 37,271)	34,136 (29,191 to 40,003)	50.3 (41.1 to 64.1)	55.8 (41.8 to 81.4)	45.8 (35 to 57.9)
High income	Western Europe	Israel	DALYs	Age-standardized	895.8 (777.5 to 1037.6)	773.9 (650.1 to 909.2)	1038.1 (916.6 to 1199.6)	601.4 (510.6 to 717.3)	533.7 (434.1 to 661.9)	678.7 (579.9 to 800.7)	-32.9 (-37.4 to -27.2)	-31 (-37.5 to -20.3)	-34.6 (-39.5 to -29.3)
High income	Western Europe	Israel	YLLs	All Ages	23,128 (21,571 to 24,782)	9,878 (8,667 to 11,158)	13,250 (12,393 to 14,254)	30,068 (26,710 to 36,431)	12,993 (10,780 to 18,693)	17,075 (14,983 to 19,490)	30 (16 to 53.8)	31.5 (9.9 to 83.9)	28.9 (12.5 to 48.6)
High income	Western Europe	Israel	YLLs	Age-standardized	481.8 (447 to 516.1)	382.4 (331.2 to 427.4)	599.4 (560.6 to 642.3)	258 (229.5 to 312.1)	198.8 (166.8 to 288.1)	326.6 (286.6 to 373.2)	-46.4 (-52.3 to -36.2)	-48 (-56.5 to -25.6)	-45.5 (-52.3 to -37.3)
High income	Western Europe	Israel	YLDs	All Ages	19,940 (14,552 to 26,376)	9,776 (7,095 to 12,986)	10,164 (7,344 to 13,585)	34,678 (25,786 to 44,671)	17,618 (13,137 to 22,577)	17,060 (12,702 to 22,277)	73.9 (63 to 85.2)	80.2 (66.4 to 95.3)	67.8 (55.9 to 80)
High income	Western Europe	Israel	YLDs	Age-standardized	414.1 (302.4 to 547.1)	391.5 (283.5 to 517.8)	438.7 (319.2 to 579.4)	343.4 (252.4 to 450)	334.9 (243.8 to 437.7)	352.1 (261.1 to 464.2)	-17.1 (-21.8 to -12.2)	-14.5 (-20.4 to -7.7)	-19.7 (-25.2 to -14.1)
High income	Western Europe	Italy	Incidence	All Ages	513,272 (457,200 to 571,744)	250,514 (221,205 to 282,009)	262,758 (235,326 to 292,277)	515,349 (468,849 to 562,705)	271,221 (243,754 to 297,855)	244,128 (221,581 to 266,850)	0.4 (-6.2 to 7.5)	8.3 (1 to 15.8)	-7.1 (-14.2 to 0.9)
High income	Western Europe	Italy	Incidence	Age-standardized	773.5 (677.4 to 890.5)	718.1 (621 to 834)	841 (744.1 to 961.3)	678.9 (561.9 to 807)	689.7 (562.9 to 829.7)	672.4 (564.7 to 793.9)	-12.2 (-20.1 to -3.2)	-4 (-12.3 to 6)	-20 (-28.1 to -11.6)
High income	Western Europe	Italy	Prevalence	All Ages	5,703,200 (5,146,015 to 6,322,793)	2,782,822 (2,481,486 to 3,125,441	2,920,378 (2,628,359 to 3,236,501)	5,497,358 (4,973,307 to 6,064,704)	2,832,311 (2,536,317 to 3,154,353)	2,665,047 (2,418,258 to 2,938,778)	-3.6 (-8.6 to 1.3)	1.8 (-3.3 to 7.2)	-8.7 (-14.4 to -2.2)
High income	Western Europe	Italy	Prevalence	Age-standardized	7725.1 (6898 to 8723.7)	7064.2 (6222.2 to 8065)	8681.4 (7812 to 9700.8)	6096.6 (5266.6 to 7094.6)	6073.4 (5150.1 to 7168.8)	6206.3 (5406.3 to 7206.9)	-21.1 (-26.5 to -15)	-14 (-20.4 to -7.1)	-28.5 (-33.9 to -22.4)
High income	Western Europe	Italy	Deaths	All Ages	24,263 (22,611 to 25,175)	7,705 (6,662 to 8,596)	16,557 (15,818 to 17,037)	31,345 (26,440 to 34,246)	12,898 (9,413 to 14,649)	18,447 (16,425 to 19,825)	29.2 (16.2 to 38.3)	67.4 (38.8 to 84.3)	11.4 (2.2 to 19)
High income	Western Europe	Italy	Deaths	Age-standardized	27.2 (25.1 to 28.3)	14.2 (12.2 to 15.9)	49.5 (46.4 to 51.2)	16.8 (14.5 to 18.2)	10.9 (8.4 to 12.3)	26.3 (23.5 to 28.3)	-38.5 (-43.1 to -34.6)	-23.5 (-31.8 to -16.8)	-46.8 (-50.5 to -43.4)
High income	Western Europe	Italy	DALYs	All Ages	676,664 (602,868 to 761,843)	247,142 (210,870 to 290,966)	429,521 (387,689 to 473,851)	668,768 (592,133 to 744,430)	291,053 (245,337 to 334,221)	377,716 (337,701 to 416,726)	-1.2 (-6.6 to 3.9)	17.8 (7.9 to 26.7)	-12.1 (-16.7 to -7.4)
High income	Western Europe	Italy	DALYs	Age-standardized	818.9 (717.7 to 936.7)	555.4 (463.8 to 667.2)	1211.1 (1087.7 to 1344.2)	538.6 (454.1 to 635.2)	448.3 (363.3 to 557.1)	668 (581.2 to 758.6)	-34.2 (-37.7 to -30.7)	-19.3 (-23.6 to -14.1)	-44.8 (-47.7 to -41.9)
High income	Western Europe	Italy	YLLs	All Ages	382,067 (364,996 to 401,870)	111,363 (100,797 to 131,384)	270,703 (262,606 to 277,193)	368,642 (323,175 to 398,328)	140,706 (110,308 to 159,883)	227,936 (207,423 to 243,358)	-3.5 (-11.7 to 2.9)	26.3 (8 to 38.2)	-15.8 (-22 to -10.2)
High income	Western Europe	Italy	YLLs	Age-standardized	429.5 (408.3 to 449.5)	218.1 (197.9 to 254.8)	746.8 (717.8 to 766.7)	229.5 (205.5 to 249.8)	149.9 (125.3 to 182.6)	342 (312.9 to 364.7)	-46.6 (-50 to -42.7)	-31.3 (-36.8 to -23.1)	-54.2 (-57.1 to -51.4)
High income	Western Europe	Italy	YLDs	All Ages	294,597 (222,587 to 376,917)	135,779 (100,926 to 177,196)	158,818 (118,666 to 201,146)	300,126 (233,607 to 366,377)	150,347 (115,343 to 187,066)	149,779 (113,931 to 184,269)	1.9 (-6 to 10.7)	10.7 (1.8 to 21.3)	-5.7 (-14.2 to 3.6)
High income	Western Europe	Italy	YLDs	Age-standardized	389.4 (291.4 to 503)	337.4 (246.6 to 445.3)	464.3 (346.2 to 590)	309.1 (231.7 to 400.7)	298.5 (218.2 to 403.7)	326 (243.2 to 412.3)	-20.6 (-25.6 to -15.2)	-11.5 (-17.2 to -5.2)	-29.8 (-34.8 to -24.5)
High income	Western Europe	Luxembourg	Incidence	All Ages	3,509 (3,133 to 4,174)	1,647 (1,479 to 1,825)	1,862 (1,621 to 2,423)	5,248 (4,750 to 5,806)	2,682 (2,429 to 2,951)	2,566 (2,303 to 2,879)	49.6 (30.7 to 58)	62.9 (53 to 73.4)	37.8 (8.8 to 49.1)
High income	Western Europe	Luxembourg	Incidence	Age-standardized	905.6 (775.1 to 1086.9)	780 (671.8 to 900.7)	1037.5 (879.6 to 1318.6)	802.2 (685 to 934.4)	771.4 (661.4 to 893.9)	830.5 (704 to 977.1)	-11.4 (-21.6 to -6.6)	-1.1 (-7.1 to 5.6)	-20 (-34.7 to -13.5)
High income	Western Europe	Luxembourg	Prevalence	All Ages	51,154 (45,723 to 60,039)	23,070 (20,851 to 25,445)	28,084 (24,113 to 35,557)	71,771 (64,769 to 79,269)	35,862 (32,490 to 39,722)	35,909 (31,816 to 40,332)	40.3 (24.2 to 48.9)	55.4 (46.5 to 65.9)	27.9 (3.9 to 41.4)
High income	Western Europe	Luxembourg	Prevalence	Age-standardized	11628.1 (10215.4 to 13555.7)	9626.1 (8536.5 to 10841.9)	13920.3 (11893.6 to 17344.1)	9524 (8374.4 to 10840.7)	8976.1 (7937.2 to 10169.5)	10065.3 (8718.9 to 11678.7)	-18.1 (-26.5 to -13.1)	-6.8 (-12.7 to -0.2)	-27.7 (-40.1 to -20.1)
High income	Western Europe	Luxembourg	Deaths	All Ages	176 (163 to 195)	63 (54 to 81)	114 (104 to 124)	221 (183 to 260)	104 (77 to 129)	118 (97 to 143)	25.5 (4.4 to 48.5)	65.6 (22.8 to 106.8)	3.4 (-16 to 27.2)
High income	Western Europe	Luxembourg	Deaths	Age-standardized	32.6 (30 to 36)	18.6 (16.3 to 23.5)	58.7 (53.7 to 63.8)	19.9 (16.5 to 23.3)	15.4 (11.5 to 19)	26.7 (22 to 32.2)	-38.9 (-49 to -27.9)	-17.5 (-37.3 to 2.3)	-54.6 (-62.9 to -44.7)
High income	Western Europe	Luxembourg	DALYs	All Ages	5,580 (4,859 to 6,451)	2,151 (1,820 to 2,563)	3,429 (2,983 to 4,003)	6,910 (5,878 to 8,100)	3,285 (2,724 to 3,891)	3,625 (3,046 to 4,288)	23.8 (12.3 to 36)	52.7 (34.3 to 69.9)	5.7 (-6.6 to 18.3)
High income	Western Europe	Luxembourg	DALYs	Age-standardized	1126.2 (963.3 to 1321.3)	780.2 (642.7 to 940.5)	1641 (1420.9 to 1922.5)	787.3 (652.6 to 952.6)	696.4 (561.8 to 847.1)	897.2 (734.6 to 1079.3)	-30.1 (-36.1 to -23.8)	-10.7 (-19.5 to -1.2)	-45.3 (-51.3 to -39)
High income	Western Europe	Luxembourg	YLLs	All Ages	3,011 (2,783 to 3,280)	996 (879 to 1,265)	2,015 (1,839 to 2,214)	3,111 (2,624 to 3,665)	1,349 (1,020 to 1,675)	1,762 (1,469 to 2,132)	3.3 (-13.9 to 22.7)	35.5 (2.8 to 67.1)	-12.6 (-29.3 to 6.9)
High income	Western Europe	Luxembourg	YLLs	Age-standardized	557.7 (515.9 to 606.8)	315.3 (279.3 to 390)	947 (866.4 to 1036)	306.5 (257.6 to 361.3)	239.3 (182.4 to 297.7)	392.9 (328.8 to 474.8)	-45 (-54.4 to -35)	-24.1 (-41.2 to -5.1)	-58.5 (-66.1 to -49.2)
High income	Western Europe	Luxembourg	YLDs	All Ages	2,569 (1,899 to 3,385)	1,156 (856 to 1,490)	1,414 (1,021 to 1,975)	3,798 (2,907 to 4,791)	1,936 (1,489 to 2,418)	1,862 (1,399 to 2,383)	47.8 (29.4 to 60.1)	67.6 (55.4 to 81.9)	31.7 (7.1 to 45.9)
High income	Western Europe	Luxembourg	YLDs	Age-standardized	568.5 (414.1 to 764.3)	464.9 (334.8 to 613.4)	694 (498.2 to 966.6)	480.8 (359.3 to 623.6)	457.1 (340.4 to 594)	504.2 (370.8 to 665)	-15.4 (-24.5 to -9.5)	-1.7 (-8.4 to 6.4)	-27.3 (-40.2 to -20.3)
High income	Western Europe	Malta	Incidence	All Ages	3,205 (2,842 to 3,599)	1,619 (1,410 to 1,846)	1,585 (1,394 to 1,791)	3,829 (3,472 to 4,232)	1,923 (1,708 to 2,177)	1,906 (1,737 to 2,100)	19.5 (11.6 to 27.9)	18.8 (9.7 to 28.9)	20.2 (10.5 to 31.4)
High income	Western Europe	Malta	Incidence	Age-standardized	856.5 (752.8 to 980.4)	843.4 (731.1 to 977.5)	882.7 (770.4 to 1008.4)	760.9 (645.1 to 895.7)	774.9 (651.9 to 927.4)	752.4 (635.4 to 883.5)	-11.2 (-18.3 to -2.7)	-8.1 (-16.5 to 1.4)	-14.8 (-24.1 to -5.9)
High income	Western Europe	Malta	Prevalence	All Ages	42,693 (38,191 to 48,120)	23,074 (20,335 to 26,497)	19,619 (17,331 to 22,288)	51,640 (46,484 to 57,969)	28,076 (24,837 to 32,053)	23,564 (21,404 to 26,409)	21 (13.3 to 28.8)	21.7 (12.6 to 31)	20.1 (10.4 to 32.4)
High income	Western Europe	Malta	Prevalence	Age-standardized	10742.2 (9593 to 12150.5)	11088.3 (9727.4 to 12803.5)	10547.5 (9366.5 to 11970.2)	8951.8 (7779.5 to 10400.4)	9738.7 (8331.6 to 11479)	8288.7 (7289 to 9666.3)	-16.7 (-23.2 to -9.8)	-12.2 (-19.4 to -3.6)	-21.4 (-28.7 to -12.8)
High income	Western Europe	Malta	Deaths	All Ages	106 (96 to 117)	25 (21 to 30)	81 (73 to 90)	157 (132 to 181)	44 (33 to 55)	113 (95 to 132)	48.1 (24.3 to 71.3)	77.4 (40.3 to 123.5)	39.2 (14.6 to 66.2)
High income	Western Europe	Malta	Deaths	Age-standardized	26.3 (23.7 to 28.9)	10.6 (9 to 12.9)	50.6 (45.2 to 56.4)	15.4 (13 to 17.7)	7.3 (5.6 to 9.1)	27.2 (22.7 to 31.6)	-41.4 (-50.6 to -32.4)	-30.8 (-44.3 to -13.2)	-46.3 (-55.7 to -35.4)
High income	Western Europe	Malta	DALYs	All Ages	3,866 (3,278 to 4,567)	1,481 (1,163 to 1,870)	2,385 (2,092 to 2,716)	4,923 (4,167 to 5,696)	1,964 (1,564 to 2,434)	2,959 (2,559 to 3,371)	27.3 (17 to 38.2)	32.6 (21.2 to 47.8)	24 (10.8 to 38.8)
High income	Western Europe	Malta	DALYs	Age-standardized	944.2 (799.3 to 1120.6)	684.8 (531.5 to 882)	1315.9 (1158.9 to 1488.3)	674.1 (547.5 to 827.5)	570.1 (430.9 to 746.2)	820.6 (692 to 965.8)	-28.6 (-34.6 to -22.5)	-16.8 (-23.5 to -9.1)	-37.6 (-44.3 to -30.4)
High income	Western Europe	Malta	YLLs	All Ages	1,815 (1,661 to 1,983)	421 (360 to 515)	1,395 (1,262 to 1,545)	2,282 (1,940 to 2,627)	601 (472 to 745)	1,682 (1,404 to 1,957)	25.7 (6.6 to 47.2)	42.9 (15.4 to 79.8)	20.6 (-1.3 to 43.9)
High income	Western Europe	Malta	YLLs	Age-standardized	430.6 (394 to 469.7)	178.8 (153.2 to 217.1)	780.3 (706.8 to 861.6)	242 (206.7 to 278.7)	119.4 (94.7 to 145.4)	398.5 (333.4 to 462.1)	-43.8 (-52 to -34.4)	-33.2 (-45.3 to -16.6)	-48.9 (-57.9 to -39)
High income	Western Europe	Malta	YLDs	All Ages	2,051 (1,480 to 2,759)	1,060 (747 to 1,456)	991 (720 to 1,305)	2,640 (1,982 to 3,349)	1,363 (1,003 to 1,775)	1,277 (966 to 1,614)	28.7 (18.6 to 39.9)	28.5 (17.8 to 40.7)	29 (16.3 to 43.2)
High income	Western Europe	Malta	YLDs	Age-standardized	513.6 (370.4 to 689.7)	506 (354.8 to 699.7)	535.6 (390.8 to 702.4)	432.1 (312.1 to 584)	450.7 (314.5 to 623.2)	422.1 (311.3 to 556.5)	-15.9 (-22 to -9.2)	-10.9 (-18.2 to -2.5)	-21.2 (-28 to -13.4)
High income	Western Europe	Monaco	Incidence	All Ages	274 (250 to 297)	135 (123 to 148)	139 (123 to 153)	353 (324 to 383)	181 (164 to 199)	172 (158 to 187)	28.9 (23.4 to 39.9)	34.4 (27.4 to 41)	23.5 (16.4 to 41.3)
High income	Western Europe	Monaco	Incidence	Age-standardized	754.6 (646.4 to 890.7)	696.4 (582.9 to 839.6)	818.3 (699.5 to 954.4)	740.6 (628.1 to 869.8)	730.8 (612.6 to 865)	752.1 (638 to 882.9)	-1.9 (-5.9 to 3.1)	4.9 (-1.5 to 10.6)	-8.1 (-13.7 to -0.7)
High income	Western Europe	Monaco	Prevalence	All Ages	3,813 (3,478 to 4,190)	1,859 (1,684 to 2,080)	1,954 (1,764 to 2,139)	4,806 (4,427 to 5,246)	2,454 (2,229 to 2,730)	2,353 (2,174 to 2,549)	26.1 (21 to 33.2)	32 (24.1 to 39.7)	20.4 (14.3 to 32.5)
High income	Western Europe	Monaco	Prevalence	Age-standardized	8839.6 (7799.6 to 10098.7)	8203.8 (7085 to 9636.7)	9674.6 (8580.1 to 11029.2)	8646.1 (7618.9 to 9826.3)	8588.6 (7412.7 to 10021.6)	8742.6 (7712 to 10032)	-2.2 (-6.2 to 2.4)	4.7 (-2.3 to 11.3)	-9.6 (-14.8 to -3.3)
High income	Western Europe	Monaco	Deaths	All Ages	15 (12 to 19)	6 (4 to 9)	9 (8 to 11)	20 (16 to 23)	9 (6 to 11)	11 (9 to 13)	29.3 (-2.3 to 65.7)	48.1 (-8.8 to 109.8)	17.5 (-5.7 to 49.3)
High income	Western Europe	Monaco	Deaths	Age-standardized	18.7 (14.8 to 23.3)	11.5 (8.4 to 17.5)	30.6 (24.8 to 36.7)	17 (13.7 to 20.1)	12.9 (9.4 to 16.3)	22.6 (18.6 to 26.9)	-8.8 (-31.2 to 17.6)	11.4 (-31.2 to 58.3)	-26 (-40.3 to -6.3)
High income	Western Europe	Monaco	DALYs	All Ages	429 (364 to 496)	179 (147 to 223)	249 (210 to 290)	525 (450 to 602)	244 (203 to 286)	281 (240 to 325)	22.6 (5.4 to 39.9)	36.2 (8.4 to 60.6)	12.8 (-1.9 to 30.7)
High income	Western Europe	Monaco	DALYs	Age-standardized	751.5 (618.1 to 907.3)	595.6 (470.6 to 748.2)	973.1 (816 to 1151)	701.1 (575.1 to 845.5)	632.9 (504.4 to 780.6)	785.3 (652.6 to 936.8)	-6.7 (-17.2 to 3.6)	6.3 (-10.3 to 20.4)	-19.3 (-28.5 to -9.1)
High income	Western Europe	Monaco	YLLs	All Ages	220 (173 to 275)	79 (57 to 122)	141 (112 to 172)	259 (207 to 307)	108 (79 to 136)	151 (124 to 180)	17.8 (-11.6 to 55.5)	36.9 (-16.2 to 97.1)	7 (-15.5 to 38.3)
High income	Western Europe	Monaco	YLLs	Age-standardized	308.4 (242.2 to 385.1)	194.3 (143.7 to 289)	474.4 (381.5 to 571.6)	265 (209.9 to 315.9)	203.1 (150 to 256.6)	340.3 (276.7 to 408.4)	-14.1 (-36.4 to 13.1)	4.5 (-34.9 to 49.3)	-28.3 (-43.7 to -7.8)
High income	Western Europe	Monaco	YLDs	All Ages	208 (160 to 259)	100 (77 to 125)	108 (82 to 136)	266 (206 to 328)	136 (105 to 168)	130 (100 to 161)	27.7 (21.9 to 37.2)	35.7 (27.1 to 44.5)	20.2 (13.2 to 36.1)
High income	Western Europe	Monaco	YLDs	Age-standardized	443.1 (331.6 to 585.6)	401.3 (294.9 to 537.5)	498.6 (374.3 to 653.1)	436.2 (327.2 to 570.8)	429.8 (318.5 to 567.8)	445 (328.3 to 581.6)	-1.6 (-5.7 to 3.6)	7.1 (0.3 to 14.2)	-10.8 (-15.9 to -2.7)
High income	Western Europe	Netherlands	Incidence	All Ages	101,780 (94,138 to 110,255)	52,513 (47,885 to 57,795)	49,267 (45,666 to 53,008)	145,207 (135,511 to 154,112)	80,497 (74,822 to 86,370)	64,710 (60,534 to 68,903)	42.7 (36.3 to 50.8)	53.3 (43.5 to 64.6)	31.3 (25 to 38.8)
High income	Western Europe	Netherlands	Incidence	Age-standardized	640 (578.8 to 714.5)	628.4 (554.6 to 712.9)	661.9 (601.3 to 730.4)	641.6 (576.4 to 712.3)	702.8 (625.5 to 789.8)	580.7 (521.7 to 647.2)	0.2 (-4.6 to 8.3)	11.8 (4.8 to 23.4)	-12.3 (-17.6 to -5.6)
High income	Western Europe	Netherlands	Prevalence	All Ages	1,837,423 (1,599,174 to 2,098,844)	973,289 (850,210 to 1,118,444)	864,134 (743,206 to 990,686)	2,372,614 (2,171,038 to 2,610,071)	1,359,029 (1,237,541 to 1,509,468)	1,013,585 (925,824 to 1,111,741)	29.1 (19.3 to 44.7)	39.6 (27.7 to 57.7)	17.3 (6.7 to 31.7)
High income	Western Europe	Netherlands	Prevalence	Age-standardized	10804 (9244.5 to 12546.6)	10880.1 (9290.7 to 12796.8)	10924.2 (9376.3 to 12662.2)	10073.9 (8888 to 11406.3)	11228.6 (9906.2 to 12994.4)	8924.9 (7878.5 to 10156)	-6.8 (-13.4 to 5.9)	3.2 (-5.9 to 19.2)	-18.3 (-25.4 to -7.7)
High income	Western Europe	Netherlands	Deaths	All Ages	6,934 (6,378 to 7,449)	2,152 (1,847 to 2,584)	4,782 (4,420 to 5,097)	10,766 (8,586 to 12,240)	4,876 (2,862 to 6,016)	5,890 (5,114 to 6,717)	55.3 (25.7 to 75.7)	126.6 (38.5 to 182.6)	23.2 (7.4 to 39.9)
High income	Western Europe	Netherlands	Deaths	Age-standardized	33.5 (30.8 to 36)	16.8 (14.4 to 20.1)	63.8 (58.3 to 68.2)	28.6 (22.8 to 32.4)	22.5 (13.1 to 27.5)	38.3 (33.4 to 43.7)	-14.5 (-30.9 to -3.3)	34.4 (-18 to 65.3)	-40 (-47.6 to -31.5)
High income	Western Europe	Netherlands	DALYs	All Ages	206,630 (181,190 to 237,410)	81,783 (68,302 to 97,650)	124,847 (111,769 to 140,685)	291,490 (249,108 to 329,972)	145,414 (110,703 to 169,233)	146,077 (127,382 to 164,424)	41.1 (24.9 to 55.3)	77.8 (39.7 to 105.2)	17 (6.3 to 29.5)
High income	Western Europe	Netherlands	DALYs	Age-standardized	1093.9 (946.8 to 1285.2)	810.4 (654.7 to 998.9)	1554.2 (1385.7 to 1753.9)	976.5 (816 to 1135.6)	949 (725.3 to 1135.5)	1037.8 (893.2 to 1198.8)	-10.7 (-19.7 to -2.7)	17.1 (-4.1 to 33.1)	-33.2 (-38.9 to -26.9)
High income	Western Europe	Netherlands	YLLs	All Ages	109,051 (102,205 to 116,316)	32,533 (28,873 to 39,098)	76,518 (71,785 to 81,220)	150,956 (121,509 to 169,064)	67,967 (38,971 to 81,215)	82,989 (72,952 to 93,640)	38.4 (10.8 to 55.6)	108.9 (25.4 to 154.1)	8.5 (-5.2 to 23.6)
High income	Western Europe	Netherlands	YLLs	Age-standardized	534.9 (503.1 to 571.1)	277.5 (248.7 to 333.6)	947.7 (884.6 to 1006.8)	428.1 (347.5 to 477.5)	361 (209.5 to 426.8)	526.1 (464.3 to 592.3)	-20 (-35.8 to -10.1)	30.1 (-21.1 to 56.3)	-44.5 (-51.3 to -36.9)
High income	Western Europe	Netherlands	YLDs	All Ages	97,579 (73,062 to 126,754)	49,250 (36,220 to 64,417)	48,330 (36,825 to 62,847)	140,534 (111,141 to 171,637)	77,447 (60,481 to 95,692)	63,087 (49,584 to 76,535)	44 (27.9 to 67.6)	57.3 (37.4 to 85)	30.5 (13.8 to 52.4)
High income	Western Europe	Netherlands	YLDs	Age-standardized	559 (416.8 to 738.5)	532.9 (383.9 to 718.9)	606.5 (460.7 to 790.8)	548.4 (418.6 to 695)	588 (440 to 758.3)	511.7 (398.2 to 644.6)	-1.9 (-10.5 to 11.6)	10.3 (-1.2 to 28.5)	-15.6 (-24.2 to -4.6)
High income	Western Europe	Norway	Incidence	All Ages	54,806 (48,358 to 61,690)	26,709 (23,417 to 30,083)	28,097 (24,926 to 31,828)	61,768 (55,487 to 68,953)	32,152 (28,918 to 35,661)	29,615 (26,456 to 33,420)	12.7 (6.8 to 19)	20.4 (14.2 to 27.2)	5.4 (-1.1 to 11.9)
High income	Western Europe	Norway	Incidence	Age-standardized	1231.6 (1062.7 to 1436.5)	1169.6 (1002.5 to 1364.8)	1303.2 (1132.9 to 1524.5)	1051.7 (897.5 to 1235)	1071.2 (915 to 1260.6)	1034.2 (877.2 to 1224.8)	-14.6 (-20.4 to -9.2)	-8.4 (-14 to -2.7)	-20.6 (-27 to -14.8)
High income	Western Europe	Norway	Prevalence	All Ages	669,791 (602,916 to 745,952)	330,542 (297,552 to 369,837)	339,249 (304,635 to 380,223)	666,686 (604,066 to 740,039)	355,700 (323,443 to 395,657)	310,986 (279,723 to 347,419)	-0.5 (-5.5 to 4.3)	7.6 (2 to 13.4)	-8.3 (-13.5 to -2.9)
High income	Western Europe	Norway	Prevalence	Age-standardized	12861.1 (11400.8 to 14541.3)	12041.2 (10640.3 to 13698.9)	13873.5 (12291.5 to 15819.4)	9898.3 (8700.7 to 11300.4)	10139.7 (8880.8 to 11619.1)	9660.2 (8441.8 to 11111.2)	-23 (-26.8 to -19.3)	-15.8 (-20.2 to -11.4)	-30.4 (-34.5 to -26.1)
High income	Western Europe	Norway	Deaths	All Ages	1,523 (1,414 to 1,785)	593 (523 to 837)	930 (884 to 968)	2,771 (2,117 to 3,026)	1,334 (723 to 1,523)	1,437 (1,300 to 1,539)	81.9 (31.8 to 100.6)	124.9 (7.1 to 162.8)	54.5 (44.4 to 66.6)
High income	Western Europe	Norway	Deaths	Age-standardized	20.5 (19.1 to 24)	13.6 (12.1 to 18.3)	31.5 (29.8 to 32.8)	25.9 (19.7 to 28.2)	21.9 (11.1 to 25)	31.6 (28.6 to 33.8)	26 (-9.1 to 39.1)	61.5 (-23.7 to 87.8)	0.1 (-6.3 to 7.9)
High income	Western Europe	Norway	DALYs	All Ages	58,177 (48,808 to 68,757)	25,291 (20,696 to 30,639)	32,886 (27,998 to 38,634)	73,971 (60,676 to 83,738)	36,326 (25,706 to 42,189)	37,645 (32,990 to 42,338)	27.1 (7.2 to 39.6)	43.6 (3.2 to 63.8)	14.5 (6.2 to 23.3)
High income	Western Europe	Norway	DALYs	Age-standardized	981.7 (805.8 to 1202.8)	810.9 (645.4 to 1004.3)	1215 (1019.1 to 1454.1)	889.2 (718.5 to 1049)	838.4 (608 to 1008.6)	957.1 (819.3 to 1121.1)	-9.4 (-21.1 to -2)	3.4 (-20 to 15.6)	-21.2 (-25.8 to -15.9)
High income	Western Europe	Norway	YLLs	All Ages	25,532 (24,208 to 28,553)	9,666 (8,807 to 12,240)	15,866 (15,208 to 16,459)	39,911 (30,153 to 43,405)	18,656 (9,282 to 21,202)	21,255 (19,732 to 22,766)	56.3 (16.7 to 72.4)	93 (-5.7 to 122.3)	34 (25.1 to 45.5)
High income	Western Europe	Norway	YLLs	Age-standardized	371.3 (352.9 to 413.7)	253.9 (233.3 to 309.4)	535.1 (512.4 to 554.9)	404.2 (306.1 to 438.9)	353 (170.8 to 399.8)	470.8 (436.8 to 504.6)	8.9 (-18.4 to 19.9)	39 (-30.6 to 58.7)	-12 (-17.8 to -4.4)
High income	Western Europe	Norway	YLDs	All Ages	32,645 (23,589 to 43,012)	15,625 (11,341 to 20,491)	17,020 (12,239 to 22,699)	34,060 (26,018 to 42,555)	17,670 (13,411 to 22,123)	16,390 (12,518 to 20,759)	4.3 (-5.4 to 15.3)	13.1 (0.6 to 26)	-3.7 (-12.4 to 6.6)
High income	Western Europe	Norway	YLDs	Age-standardized	610.4 (437.4 to 828.2)	557 (398.1 to 747.7)	679.9 (487.2 to 917.4)	484.9 (359.6 to 634.4)	485.5 (357.2 to 637.2)	486.3 (361.5 to 643)	-20.6 (-26.3 to -14.1)	-12.8 (-19.6 to -5.1)	-28.5 (-33.9 to -22.5)
High income	Western Europe	Portugal	Incidence	All Ages	102,836 (90,896 to 118,327)	53,425 (46,165 to 61,736)	49,410 (43,664 to 58,121)	109,547 (100,527 to 119,142)	59,970 (54,600 to 65,641)	49,577 (45,280 to 54,295)	6.5 (-2.8 to 14.5)	12.2 (1.1 to 23.3)	0.3 (-9.5 to 9.1)
High income	Western Europe	Portugal	Incidence	Age-standardized	970.7 (850.8 to 1142.1)	954.9 (819.8 to 1123.7)	989.3 (863 to 1180.1)	876.5 (743.9 to 1023.9)	880.3 (739.7 to 1036.2)	872 (739.4 to 1022.5)	-9.7 (-17.3 to -1.4)	-7.8 (-16.3 to 1.6)	-11.9 (-20.1 to -1.8)
High income	Western Europe	Portugal	Prevalence	All Ages	1,494,331 (1,315,900 to 1,711,181)	799,963 (690,698 to 929,981)	694,368 (610,534 to 797,330)	1,579,044 (1,416,698 to 1,761,149)	860,431 (766,241 to 967,196)	718,613 (645,020 to 801,760)	5.7 (-3 to 15.3)	7.6 (-3.3 to 19)	3.5 (-5 to 14.4)
High income	Western Europe	Portugal	Prevalence	Age-standardized	12953.4 (11245 to 15071.1)	13044.7 (11159 to 15463.4)	12965.6 (11347.7 to 15124.9)	11045.5 (9576.7 to 12854.9)	11237.9 (9561.4 to 13191.2)	10885.2 (9375.5 to 12671.6)	-14.7 (-22.1 to -5)	-13.9 (-23.2 to -2.1)	-16 (-24.4 to -4.2)
High income	Western Europe	Portugal	Deaths	All Ages	4,624 (4,279 to 5,029)	1,754 (1,523 to 2,067)	2,871 (2,676 to 3,082)	7,052 (5,903 to 8,062)	3,160 (2,270 to 3,853)	3,892 (3,337 to 4,497)	52.5 (31.6 to 75.5)	80.2 (39.2 to 123.8)	35.6 (15.9 to 58.2)
High income	Western Europe	Portugal	Deaths	Age-standardized	36.3 (33.3 to 39.3)	23 (19.8 to 26.6)	58.4 (54.1 to 63)	23.7 (20.2 to 26.9)	16.4 (12.6 to 20)	34.9 (30.1 to 40)	-34.8 (-42.8 to -26.1)	-28.7 (-42.4 to -12.7)	-40.3 (-48.5 to -30.8)
High income	Western Europe	Portugal	DALYs	All Ages	154,174 (132,584 to 180,964)	65,921 (53,570 to 80,875)	88,254 (78,007 to 100,199)	167,668 (145,033 to 194,773)	78,997 (64,712 to 94,787)	88,671 (77,225 to 101,647)	8.8 (0.5 to 18.3)	19.8 (7.1 to 34.4)	0.5 (-8.7 to 11.2)
High income	Western Europe	Portugal	DALYs	Age-standardized	1234.7 (1047.7 to 1471.1)	977.8 (777.1 to 1231.9)	1604 (1410.3 to 1835.8)	860.7 (706.2 to 1063.8)	747.7 (580.7 to 963.2)	1017.4 (856.7 to 1212.6)	-30.3 (-35.6 to -24.1)	-23.5 (-30.6 to -15.2)	-36.6 (-42.2 to -30.3)
High income	Western Europe	Portugal	YLLs	All Ages	79,804 (74,289 to 87,362)	26,965 (24,066 to 33,373)	52,839 (49,295 to 56,413)	86,002 (74,936 to 97,075)	34,460 (27,288 to 42,116)	51,542 (44,876 to 58,704)	7.8 (-4.6 to 22.2)	27.8 (4 to 55.8)	-2.5 (-15.9 to 13.4)
High income	Western Europe	Portugal	YLLs	Age-standardized	601.6 (560.4 to 649.3)	356.9 (320.6 to 428.6)	948.8 (884.5 to 1013.1)	330.8 (292.2 to 375.3)	215.6 (174.9 to 285.8)	487.9 (427.4 to 554)	-45 (-50.9 to -38.1)	-39.6 (-49.6 to -26.1)	-48.6 (-55.2 to -40.9)
High income	Western Europe	Portugal	YLDs	All Ages	74,370 (53,661 to 102,286)	38,956 (27,769 to 53,785)	35,414 (25,653 to 47,371)	81,666 (61,344 to 105,266)	44,536 (33,163 to 57,611)	37,130 (27,520 to 48,211)	9.8 (-0.5 to 21.1)	14.3 (0.9 to 29.2)	4.8 (-5.4 to 17.6)
High income	Western Europe	Portugal	YLDs	Age-standardized	633.1 (453.9 to 875.7)	620.9 (436.5 to 870.9)	655.2 (472.5 to 883)	529.8 (378.8 to 723.7)	532.1 (375 to 727.3)	529.5 (379.4 to 721.2)	-16.3 (-23.6 to -6.5)	-14.3 (-23.4 to -2.9)	-19.2 (-27.3 to -7.9)
High income	Western Europe	San Marino	Incidence	All Ages	178 (159 to 203)	88 (77 to 101)	91 (81 to 103)	274 (249 to 303)	145 (130 to 161)	129 (116 to 143)	53.5 (46.9 to 61)	65.4 (55.8 to 76.2)	42 (35.7 to 52.9)
High income	Western Europe	San Marino	Incidence	Age-standardized	739.4 (636.2 to 867.9)	705.5 (595.1 to 843.7)	775.4 (665.9 to 908.1)	729.2 (616.7 to 856.7)	729.7 (614.1 to 861.4)	722.9 (609.9 to 861.1)	-1.4 (-5.3 to 3)	3.4 (-3.3 to 9.9)	-6.8 (-12 to -1)
High income	Western Europe	San Marino	Prevalence	All Ages	2,316 (2,079 to 2,575)	1,164 (1,027 to 1,316)	1,151 (1,037 to 1,278)	3,648 (3,313 to 4,037)	1,976 (1,772 to 2,203)	1,672 (1,522 to 1,860)	57.5 (50.3 to 65.7)	69.7 (59.3 to 81.1)	45.2 (37.1 to 56.1)
High income	Western Europe	San Marino	Prevalence	Age-standardized	8630.9 (7667.1 to 9822)	8484.4 (7332.7 to 9784.1)	8914.1 (7940.9 to 10037.5)	8443.3 (7456.7 to 9734.8)	8716.8 (7560.9 to 10096.7)	8123 (7137.2 to 9397.9)	-2.2 (-6.4 to 2.7)	2.7 (-3.7 to 10.3)	-8.9 (-14.2 to -2.4)
High income	Western Europe	San Marino	Deaths	All Ages	5 (4 to 6)	2 (2 to 3)	3 (2 to 3)	11 (7 to 14)	5 (4 to 8)	5 (4 to 7)	103.9 (42.7 to 186.1)	124.5 (51.3 to 230.3)	86.2 (25.3 to 165.8)
High income	Western Europe	San Marino	Deaths	Age-standardized	15.8 (13.2 to 19.1)	12.5 (9.9 to 16.1)	20.8 (17.2 to 24.9)	13.4 (9.3 to 18.4)	11.5 (7.5 to 16.6)	15.8 (10.9 to 21.4)	-15.4 (-41.9 to 20.8)	-8.1 (-38.8 to 41.3)	-24.1 (-49.1 to 7.4)
High income	Western Europe	San Marino	DALYs	All Ages	200 (167 to 237)	93 (76 to 113)	107 (90 to 126)	334 (271 to 407)	170 (135 to 210)	164 (131 to 199)	66.7 (42.5 to 99.3)	82.4 (55.1 to 120.2)	53 (26.8 to 85.5)
High income	Western Europe	San Marino	DALYs	Age-standardized	678.2 (559.3 to 815.5)	602.8 (483.6 to 748.9)	780.2 (647.7 to 925.3)	631.3 (502.1 to 782.2)	608.7 (476.5 to 774)	656.6 (523.5 to 806)	-6.9 (-18.7 to 8.6)	1 (-11.5 to 18.3)	-15.8 (-28.2 to -0.7)
High income	Western Europe	San Marino	YLLs	All Ages	81 (67 to 97)	35 (28 to 46)	46 (37 to 55)	137 (93 to 191)	65 (42 to 94)	72 (49 to 101)	69.5 (12.4 to 147.9)	84 (21.5 to 185.6)	58.4 (2 to 132.2)
High income	Western Europe	San Marino	YLLs	Age-standardized	245.6 (205.6 to 292.7)	192.6 (155.1 to 245.3)	315.8 (261.9 to 377.4)	204.9 (137.5 to 294)	175.1 (113 to 265.7)	239.1 (158.9 to 337.7)	-16.6 (-45.6 to 25.1)	-9.1 (-41.2 to 44.7)	-24.3 (-51.4 to 11.4)
High income	Western Europe	San Marino	YLDs	All Ages	119 (91 to 151)	58 (44 to 75)	61 (46 to 77)	197 (153 to 244)	106 (81 to 133)	91 (70 to 114)	64.9 (55.3 to 75.1)	81.5 (69 to 95.3)	49.1 (39.2 to 62.6)
High income	Western Europe	San Marino	YLDs	Age-standardized	432.6 (322.3 to 564.4)	410.2 (303 to 546.1)	464.4 (347.1 to 604.2)	426.3 (322.4 to 558.6)	433.6 (320.9 to 577.5)	417.6 (311.2 to 546.8)	-1.4 (-6 to 3.7)	5.7 (-0.9 to 12.8)	-10.1 (-15.7 to -2.8)
High income	Western Europe	Spain	Incidence	All Ages	306,785 (277,170 to 342,762)	149,310 (133,423 to 166,889)	157,474 (141,958 to 176,583)	419,333 (385,996 to 454,765)	212,161 (194,139 to 231,345)	207,171 (191,444 to 225,777)	36.7 (29.5 to 44.2)	42.1 (32.8 to 52.3)	31.6 (23.9 to 39.6)
High income	Western Europe	Spain	Incidence	Age-standardized	720.9 (635.7 to 828.3)	656 (567.6 to 758.8)	790.4 (702.6 to 913.4)	737.3 (635.2 to 859.2)	679.8 (583.6 to 797.7)	792.7 (684.3 to 923.3)	2.3 (-4.1 to 11.1)	3.6 (-4.9 to 14.1)	0.3 (-7.1 to 9.8)
High income	Western Europe	Spain	Prevalence	All Ages	3,445,965 (3,163,330 to 3,820,702)	1,654,188 (1,487,645 to 1,846,498	1,791,776 (1,639,107 to 1,986,814)	4,791,673 (4,446,201 to 5,219,359)	2,394,879 (2,195,052 to 2,640,554)	2,396,794 (2,220,288 to 2,600,164)	39.1 (31.1 to 49.7)	44.8 (34.1 to 59.7)	33.8 (25.6 to 43.3)
High income	Western Europe	Spain	Prevalence	Age-standardized	7487.6 (6771.2 to 8507.9)	6757.7 (5941.7 to 7775.2)	8430.5 (7691.1 to 9502.4)	7208.9 (6417.5 to 8184.1)	6801 (5962.9 to 7795.2)	7707.7 (6909.2 to 8810.8)	-3.7 (-10.7 to 6.6)	0.6 (-8.5 to 14.7)	-8.6 (-15.1 to -0.2)
High income	Western Europe	Spain	Deaths	All Ages	22,694 (20,391 to 24,152)	7,555 (5,813 to 8,500)	15,139 (14,104 to 15,955)	36,560 (29,180 to 42,491)	14,736 (9,076 to 18,872)	21,824 (18,455 to 24,963)	61.1 (39.5 to 84.6)	95 (51.6 to 146.1)	44.2 (24.6 to 66.3)
High income	Western Europe	Spain	Deaths	Age-standardized	42.3 (37.6 to 45.2)	23.1 (17.6 to 26.1)	73.3 (67.5 to 77.6)	29 (24.1 to 33.2)	17.7 (11.9 to 22.1)	46.6 (39.7 to 52.9)	-31.3 (-39.3 to -22.1)	-23.3 (-36.4 to -5.8)	-36.5 (-44.9 to -27.1)
High income	Western Europe	Spain	DALYs	All Ages	536,664 (490,051 to 587,746)	186,553 (160,480 to 212,468)	350,111 (323,183 to 379,073)	677,881 (594,319 to 757,799)	272,001 (220,133 to 316,780)	405,880 (359,020 to 454,541)	26.3 (16.3 to 36.3)	45.8 (30.4 to 64.4)	15.9 (5.9 to 26.8)
High income	Western Europe	Spain	DALYs	Age-standardized	1037.8 (939 to 1153.5)	656.9 (558.9 to 766.5)	1575.7 (1452.4 to 1716.3)	752.3 (648.9 to 863.1)	550.9 (455.7 to 665.8)	1020.6 (894.7 to 1159.3)	-27.5 (-32.7 to -22.3)	-16.1 (-23.2 to -7.3)	-35.2 (-40.6 to -29.8)
High income	Western Europe	Spain	YLLs	All Ages	361,307 (335,801 to 379,643)	106,556 (87,398 to 118,102)	254,751 (239,633 to 267,954)	421,093 (360,359 to 475,441)	150,158 (104,121 to 185,037)	270,935 (235,492 to 305,425)	16.5 (2.5 to 30.9)	40.9 (15.6 to 71.2)	6.4 (-7.1 to 20.8)
High income	Western Europe	Spain	YLLs	Age-standardized	664.7 (617 to 699.3)	337.7 (280.2 to 374.1)	1132.1 (1063.3 to 1190)	388.9 (342.4 to 431.4)	225.6 (174 to 268.1)	611.2 (534.9 to 686.2)	-41.5 (-47.5 to -35.1)	-33.2 (-42.9 to -20.6)	-46 (-52.7 to -38.9)
High income	Western Europe	Spain	YLDs	All Ages	175,357 (133,271 to 220,940)	79,997 (59,239 to 102,782)	95,360 (73,831 to 120,723)	256,788 (200,607 to 314,580)	121,843 (93,519 to 151,391)	134,945 (103,938 to 166,840)	46.4 (36.2 to 57.2)	52.3 (40.1 to 67.8)	41.5 (30.6 to 52.5)
High income	Western Europe	Spain	YLDs	Age-standardized	373.1 (280.4 to 480.9)	319.2 (231.6 to 421.4)	443.6 (341.3 to 568.3)	363.4 (273.9 to 461.6)	325.4 (240 to 426.7)	409.4 (313 to 520)	-2.6 (-8.9 to 5.8)	1.9 (-6.8 to 15)	-7.7 (-14.2 to -0.2)
High income	Western Europe	Sweden	Incidence	All Ages	100,864 (88,797 to 113,244)	53,864 (46,547 to 61,532)	47,000 (41,492 to 52,951)	105,254 (94,238 to 118,312)	57,479 (51,402 to 64,413)	47,774 (41,934 to 54,572)	4.4 (-1.5 to 11.1)	6.7 (-0.4 to 15.1)	1.6 (-5 to 9.2)
High income	Western Europe	Sweden	Incidence	Age-standardized	1077.1 (924.2 to 1267.5)	1129.3 (959.6 to 1341.4)	1033.5 (888 to 1218.2)	889.5 (746 to 1065.6)	950.1 (806.2 to 1139.3)	830.8 (679.2 to 1014.7)	-17.4 (-23.2 to -11.5)	-15.9 (-22.4 to -9)	-19.6 (-26.7 to -12.2)
High income	Western Europe	Sweden	Prevalence	All Ages	1,417,462 (1,257,254 to 1,600,771)	763,386 (676,088 to 861,935)	654,077 (578,129 to 744,684)	1,356,262 (1,211,951 to 1,527,875)	763,792 (684,349 to 862,581)	592,470 (522,352 to 676,496)	-4.3 (-9.5 to 2)	0.1 (-6.4 to 7.1)	-9.4 (-15.6 to -2.4)
High income	Western Europe	Sweden	Prevalence	Age-standardized	13019.9 (11395.8 to 14976.9)	13420.2 (11646.7 to 15525.2)	12734.1 (11084.4 to 14768)	10136.2 (8677.2 to 11885.8)	11154.7 (9549.5 to 13142)	9144.9 (7758.2 to 10910.6)	-22.1 (-26.8 to -16.5)	-16.9 (-22.7 to -10.1)	-28.2 (-33.6 to -21.8)
High income	Western Europe	Sweden	Deaths	All Ages	2,481 (2,271 to 2,978)	1,017 (881 to 1,453)	1,464 (1,368 to 1,553)	4,478 (3,398 to 5,046)	2,318 (1,333 to 2,789)	2,160 (1,916 to 2,387)	80.5 (28.3 to 105.9)	127.9 (13.7 to 185)	47.5 (31.2 to 64)
High income	Western Europe	Sweden	Deaths	Age-standardized	15.1 (13.9 to 18)	10.6 (9.4 to 14.5)	22.4 (20.9 to 23.8)	17.8 (13.6 to 20)	16.4 (9.2 to 19.4)	20 (17.8 to 22.1)	18.1 (-15.6 to 33.8)	53.8 (-21.2 to 89.7)	-10.8 (-20.2 to -1.3)
High income	Western Europe	Sweden	DALYs	All Ages	112,148 (92,088 to 136,021)	56,229 (44,981 to 69,059)	55,918 (46,866 to 67,604)	134,877 (112,002 to 156,084)	75,399 (57,985 to 88,988)	59,478 (51,480 to 69,113)	20.3 (3.8 to 32.8)	34.1 (5.2 to 53.2)	6.4 (-2.5 to 15.9)
High income	Western Europe	Sweden	DALYs	Age-standardized	903.4 (718.7 to 1137.7)	867.3 (673.2 to 1115.1)	972.9 (796.3 to 1205.1)	785.8 (633.9 to 962.6)	846.6 (652.1 to 1053.5)	728.8 (603 to 889.2)	-13 (-22.1 to -5.8)	-2.4 (-17.2 to 8.3)	-25.1 (-30.3 to -19.6)
High income	Western Europe	Sweden	YLLs	All Ages	40,022 (37,443 to 45,687)	16,139 (14,426 to 20,904)	23,883 (22,474 to 25,254)	59,960 (45,333 to 67,005)	30,282 (16,582 to 35,788)	29,678 (26,675 to 32,935)	49.8 (9.6 to 69)	87.6 (-1.5 to 128.7)	24.3 (11.2 to 37.7)
High income	Western Europe	Sweden	YLLs	Age-standardized	266.1 (249.9 to 298.5)	196.2 (177.6 to 240.2)	365.3 (344.2 to 385.4)	268.7 (205.2 to 299.6)	253.6 (140.8 to 297.7)	289.7 (261.8 to 320.1)	1 (-24.2 to 13.1)	29.3 (-28 to 55.1)	-20.7 (-28.9 to -12.1)
High income	Western Europe	Sweden	YLDs	All Ages	72,125 (52,168 to 94,339)	40,091 (29,347 to 52,154)	32,035 (22,916 to 43,419)	74,917 (57,062 to 94,223)	45,117 (34,992 to 56,571)	29,800 (22,082 to 39,150)	3.9 (-5.1 to 14)	12.5 (1.5 to 25.4)	-7 (-15.1 to 2.5)
High income	Western Europe	Sweden	YLDs	Age-standardized	637.3 (452.1 to 863.5)	671.1 (484.2 to 909.5)	607.7 (428.6 to 835.3)	517.1 (377.6 to 683.8)	593 (436.8 to 791.4)	439.1 (315.8 to 595.5)	-18.9 (-24.4 to -12.1)	-11.6 (-18.5 to -3.4)	-27.7 (-33.6 to -21.2)
High income	Western Europe	Switzerland	Incidence	All Ages	60,434 (54,263 to 67,180)	28,150 (25,030 to 31,582)	32,283 (28,778 to 36,196)	71,197 (64,153 to 78,389)	38,038 (34,246 to 42,236)	33,159 (29,603 to 37,117)	17.8 (11.2 to 25.4)	35.1 (26.6 to 44.9)	2.7 (-4.7 to 10.5)
High income	Western Europe	Switzerland	Incidence	Age-standardized	854.3 (738.6 to 995.7)	762.7 (650.1 to 891.5)	959 (829.3 to 1114.8)	741.5 (628 to 884.7)	753.1 (637.2 to 901.8)	731 (606.6 to 885.7)	-13.2 (-19.4 to -6.8)	-1.3 (-8.5 to 5.9)	-23.8 (-31 to -16.2)
High income	Western Europe	Switzerland	Prevalence	All Ages	851,583 (769,106 to 952,412)	407,284 (362,227 to 460,010)	444,299 (401,310 to 500,164)	970,855 (876,306 to 1,086,663)	531,315 (474,769 to 600,559)	439,540 (392,375 to 495,491)	14 (5.9 to 22.6)	30.5 (20.5 to 41.3)	-1.1 (-9.3 to 8.1)
High income	Western Europe	Switzerland	Prevalence	Age-standardized	10504.2 (9289.8 to 11995.5)	9555 (8272.5 to 11086)	11773.9 (10506.3 to 13466.1)	8734.7 (7566.6 to 10075)	9157.2 (7938.7 to 10688.4)	8345.6 (7175.7 to 9795.8)	-16.8 (-23.6 to -8.9)	-4.2 (-12.2 to 5.9)	-29.1 (-36 to -20.9)
High income	Western Europe	Switzerland	Deaths	All Ages	2,537 (2,300 to 2,744)	844 (699 to 988)	1,693 (1,535 to 1,832)	3,074 (2,563 to 3,504)	1,348 (977 to 1,635)	1,727 (1,471 to 2,007)	21.2 (4.6 to 38.7)	59.7 (30.2 to 97)	2 (-13.1 to 20.1)
High income	Western Europe	Switzerland	Deaths	Age-standardized	22.8 (20.7 to 24.7)	12.1 (10.2 to 14)	40.9 (36.9 to 44.4)	14.9 (12.6 to 16.9)	11.1 (7.9 to 13.2)	20.5 (17.6 to 23.8)	-34.6 (-42.8 to -25.6)	-8.5 (-25.6 to 10.7)	-49.7 (-57 to -41.2)
High income	Western Europe	Switzerland	DALYs	All Ages	83,694 (72,360 to 96,772)	32,316 (26,648 to 38,581)	51,378 (44,836 to 58,500)	92,008 (79,093 to 106,710)	44,792 (37,092 to 53,204)	47,216 (40,656 to 54,379)	9.9 (2.5 to 17.9)	38.6 (25.7 to 51.4)	-8.1 (-15.3 to 0.5)
High income	Western Europe	Switzerland	DALYs	Age-standardized	902.8 (761.5 to 1080.1)	653.8 (516.6 to 815)	1260 (1086.1 to 1452.4)	656.4 (537.1 to 802)	616.9 (486.4 to 772.3)	713.8 (597 to 854.9)	-27.3 (-32.1 to -22.2)	-5.6 (-13.8 to 2.3)	-43.3 (-48.3 to -38.2)
High income	Western Europe	Switzerland	YLLs	All Ages	41,310 (38,064 to 44,171)	12,621 (10,706 to 14,418)	28,689 (26,477 to 30,816)	41,615 (35,463 to 46,720)	17,377 (12,292 to 20,629)	24,238 (20,963 to 27,624)	0.7 (-11.7 to 13.6)	37.7 (11.7 to 65.6)	-15.5 (-27 to -1.6)
High income	Western Europe	Switzerland	YLLs	Age-standardized	396 (365.1 to 423.2)	207.5 (174.9 to 234.4)	670.1 (618.6 to 719.6)	230.4 (197.9 to 256.5)	175.1 (121.6 to 206.2)	301.4 (261.8 to 343.5)	-41.8 (-48.6 to -34.6)	-15.6 (-31.4 to 0.7)	-55 (-60.9 to -47.5)
High income	Western Europe	Switzerland	YLDs	All Ages	42,384 (30,909 to 55,224)	19,695 (14,522 to 25,788)	22,688 (16,398 to 29,617)	50,393 (37,905 to 63,780)	27,415 (20,916 to 35,021)	22,978 (17,232 to 29,355)	18.9 (10.1 to 28.3)	39.2 (27.7 to 51.8)	1.3 (-7.6 to 10.6)
High income	Western Europe	Switzerland	YLDs	Age-standardized	506.8 (363.8 to 679.3)	446.3 (319.4 to 604.5)	589.9 (423.3 to 783.3)	426 (311.5 to 569.3)	441.8 (317.1 to 592.4)	412.4 (296.1 to 554.3)	-15.9 (-21.9 to -8.8)	-1 (-8.9 to 8.6)	-30.1 (-36.5 to -23)
High income	Western Europe	United Kingdom	Incidence	All Ages	723,168 (632,842 to 822,332)	400,748 (349,776 to 455,825)	322,420 (283,721 to 371,277)	773,975 (692,483 to 868,569)	434,752 (390,374 to 485,108)	339,223 (301,581 to 383,929)	7 (3.2 to 10.7)	8.5 (3.6 to 13.2)	5.2 (1.2 to 9.3)
High income	Western Europe	United Kingdom	Incidence	Age-standardized	1271.2 (1073.9 to 1497.6)	1349 (1134.1 to 1599.4)	1197.7 (1008 to 1428.6)	1073.1 (903.3 to 1275.2)	1171.3 (990.6 to 1392)	974.5 (813.2 to 1175.4)	-15.6 (-18.9 to -12.4)	-13.2 (-17.5 to -9.3)	-18.6 (-22.2 to -15.3)
High income	Western Europe	United Kingdom	Prevalence	All Ages	10,647,235 (9,611,573 to 11,795,879)	5,964,837 (5,362,773 to 6,630,353	4,682,399 (4,195,639 to 5,212,519)	10,188,015 (9,254,912 to 11,185,536	5,953,771 (5,383,589 to 6,578,326)	4,234,244 (3,843,922 to 4,691,605)	-4.3 (-7.1 to -1.3)	-0.2 (-3.4 to 3.4)	-9.6 (-12.8 to -5.9)
High income	Western Europe	United Kingdom	Prevalence	Age-standardized	16103.3 (14316.5 to 18190.1)	16836.4 (14830.3 to 19151.2)	15382.9 (13616.4 to 17386.5)	12152 (10750.5 to 13846.1)	13758.9 (12118.8 to 15676.8)	10522.6 (9276.8 to 12039.1)	-24.5 (-26.8 to -22.1)	-18.3 (-21.2 to -15.3)	-31.6 (-34.1 to -28.9)
High income	Western Europe	United Kingdom	Deaths	All Ages	37,657 (35,478 to 43,241)	14,901 (13,458 to 20,138)	22,756 (21,994 to 23,247)	51,879 (43,733 to 55,229)	25,012 (17,192 to 27,542)	26,867 (24,833 to 28,198)	37.8 (12.4 to 46.3)	67.9 (5.7 to 86.5)	18.1 (11.7 to 22.6)
High income	Western Europe	United Kingdom	Deaths	Age-standardized	39.6 (37.3 to 45.6)	25.9 (23.5 to 33.9)	65.6 (62.7 to 67.2)	36.5 (30.8 to 38.8)	30.8 (21 to 33.6)	44.5 (41.2 to 46.7)	-7.7 (-24.8 to -2.3)	18.9 (-23.3 to 30.6)	-32.1 (-35 to -29.7)
High income	Western Europe	United Kingdom	DALYs	All Ages	1,175,413 (1,031,508 to 1,346,135)	540,000 (453,382 to 648,361)	635,413 (568,571 to 713,793)	1,285,743 (1,113,883 to 1,444,486)	649,390 (521,446 to 749,525)	636,352 (575,617 to 701,355)	9.4 (-1.6 to 14.9)	20.3 (-1.6 to 30)	0.1 (-3.9 to 3.9)
High income	Western Europe	United Kingdom	DALYs	Age-standardized	1484.8 (1260.2 to 1763.3)	1277.3 (1032.9 to 1567.5)	1833.5 (1614.3 to 2095.7)	1186.9 (1004 to 1391.9)	1158.7 (924.6 to 1395.3)	1236.5 (1094.2 to 1406.3)	-20.1 (-26.4 to -16.9)	-9.3 (-21.5 to -3.6)	-32.6 (-34.5 to -30.5)
High income	Western Europe	United Kingdom	YLLs	All Ages	639,660 (613,755 to 708,709)	246,527 (229,062 to 307,865)	393,133 (383,510 to 400,517)	739,342 (625,840 to 778,552)	343,310 (230,115 to 372,276)	396,032 (373,251 to 413,223)	15.6 (-2.5 to 21.4)	39.3 (-6.8 to 51.8)	0.7 (-3.8 to 4.4)
High income	Western Europe	United Kingdom	YLLs	Age-standardized	704.4 (676.5 to 777.6)	481 (447.1 to 578.3)	1059.9 (1029.6 to 1080.9)	575.5 (489.3 to 603.5)	492.3 (335.2 to 530.1)	678.9 (641.1 to 707.3)	-18.3 (-30.4 to -14.4)	2.4 (-28.1 to 10.3)	-35.9 (-38.5 to -33.7)
High income	Western Europe	United Kingdom	YLDs	All Ages	535,753 (396,758 to 706,248)	293,473 (213,596 to 386,690)	242,280 (180,699 to 318,263)	546,401 (421,841 to 686,759)	306,080 (229,191 to 393,429)	240,320 (187,151 to 299,244)	2 (-4.1 to 9.2)	4.3 (-1.3 to 10.9)	-0.8 (-8.3 to 8.3)
High income	Western Europe	United Kingdom	YLDs	Age-standardized	780.4 (561.7 to 1051.9)	796.3 (564.6 to 1080)	773.6 (564.6 to 1030)	611.4 (456.5 to 810.7)	666.4 (486.6 to 899.1)	557.6 (421.4 to 718.9)	-21.7 (-24.9 to -17.6)	-16.3 (-20 to -12.3)	-27.9 (-31.7 to -23.5)
		Southern Latin America	Incidence	All Ages	529,063 (463,814 to 615,152)	262,476 (228,238 to 306,900)	266,587 (231,092 to 310,419)	797,778 (702,254 to 920,107)	414,273 (363,919 to 472,464)	383,505 (335,689 to 453,441)	50.8 (42.3 to 59.7)	57.8 (48 to 68.9)	43.9 (35.2 to 53.8)
		Southern Latin America	Incidence	Age-standardized	1075.9 (949.4 to 1243)	1032.5 (900.2 to 1203.5)	1129.6 (991.8 to 1298.7)	1169 (1005.5 to 1388)	1150.5 (988 to 1352.7)	1191.7 (1026.4 to 1430.6)	8.7 (2.3 to 16.2)	11.4 (3.6 to 20.3)	5.5 (-1.4 to 13.5)
		Southern Latin America	Prevalence	All Ages	4,106,470 (3,681,444 to 4,634,942)	2,177,670 (1,926,996 to 2,460,930	1,928,800 (1,734,729 to 2,192,583)	6,289,607 (5,692,879 to 7,053,892)	3,542,519 (3,175,458 to 3,970,963)	2,747,088 (2,469,065 to 3,100,365)	53.2 (45.5 to 62.3)	62.7 (52.9 to 72.6)	42.4 (34 to 52.3)
		Southern Latin America	Prevalence	Age-standardized	8516.1 (7663.9 to 9556.9)	8615.7 (7605 to 9726)	8499 (7717.9 to 9521.5)	8716.1 (7771 to 10037.5)	9213.1 (8144.7 to 10549.2)	8234.3 (7314 to 9503.2)	2.3 (-3.9 to 9)	6.9 (-0.3 to 14.4)	-3.1 (-9.1 to 4.2)
		Southern Latin America	Deaths	All Ages	13,966 (13,014 to 15,616)	5,194 (4,634 to 6,702)	8,772 (8,263 to 9,302)	27,893 (24,150 to 30,844)	12,780 (9,602 to 14,632)	15,113 (13,456 to 16,732)	99.7 (70.9 to 122.4)	146.1 (80.3 to 190)	72.3 (52.9 to 92.7)
		Southern Latin America	Deaths	Age-standardized	32.7 (30.2 to 37.1)	21.3 (18.8 to 27.7)	48.9 (45.7 to 52.1)	32.6 (28.2 to 35.9)	24.6 (18.6 to 28)	44.2 (39.3 to 49)	-0.5 (-15.2 to 10.4)	15.3 (-14.4 to 35.4)	-9.6 (-19.9 to 1.1)
		Southern Latin America	DALYs	All Ages	470,413 (414,337 to 537,764)	198,748 (165,256 to 237,975)	271,665 (244,127 to 303,146)	739,935 (648,037 to 840,683)	353,528 (297,705 to 414,077)	386,408 (345,527 to 435,491)	57.3 (46.5 to 68.2)	77.9 (56.6 to 94.2)	42.2 (31.3 to 54.1)
		Southern Latin America	DALYs	Age-standardized	1006.4 (889.7 to 1149.6)	787 (653.3 to 943.1)	1295.7 (1174.2 to 1432.5)	942.1 (817.9 to 1086.8)	824 (682.8 to 981.7)	1106.6 (984.6 to 1254)	-6.4 (-13 to -0.8)	4.7 (-6.6 to 13.1)	-14.6 (-21 to -7.7)
		Southern Latin America	YLLs	All Ages	287,559 (272,720 to 312,781)	102,302 (93,473 to 123,397)	185,257 (175,970 to 195,294)	449,307 (401,622 to 490,946)	189,696 (148,536 to 212,741)	259,610 (233,780 to 285,836)	56.2 (39.6 to 71.6)	85.4 (49.9 to 112.6)	40.1 (25.3 to 55.9)
		Southern Latin America	YLLs	Age-standardized	626.4 (592.5 to 683.4)	405.3 (369.7 to 489.4)	912.6 (865.5 to 962.9)	542.2 (486.1 to 590.9)	401.8 (320 to 449.8)	728 (657.1 to 800.4)	-13.4 (-22.4 to -5.3)	-0.9 (-18.5 to 13)	-20.2 (-28.7 to -11.3)
		Southern Latin America	YLDs	All Ages	182,854 (130,351 to 246,840)	96,446 (67,245 to 131,746)	86,408 (61,624 to 116,356)	290,629 (213,032 to 382,842)	163,831 (118,593 to 219,179)	126,797 (92,487 to 166,229)	58.9 (49 to 70.3)	69.9 (57.8 to 83.2)	46.7 (36.8 to 58.8)
		Southern Latin America	YLDs	Age-standardized	380 (273.6 to 511.2)	381.7 (266.4 to 522.4)	383 (275.6 to 508.7)	399.9 (289.1 to 533.8)	422.2 (301.7 to 575.4)	378.6 (278.6 to 503.7)	5.2 (-1.3 to 11.9)	10.6 (2.7 to 18.8)	-1.1 (-7.8 to 6.2)
High income	Southern Latin America	Argentina	Incidence	All Ages	368,748 (323,736 to 430,252)	185,081 (160,884 to 217,833)	183,666 (158,783 to 214,500)	546,399 (477,296 to 636,960)	286,034 (248,265 to 330,157)	260,365 (226,249 to 310,317)	48.2 (38.7 to 58.4)	54.5 (42.5 to 67.7)	41.8 (31 to 52.8)
High income	Southern Latin America	Argentina	Incidence	Age-standardized	1104.9 (972 to 1280.9)	1076.3 (933.8 to 1265.6)	1143.7 (1000.6 to 1320.3)	1202.2 (1030.3 to 1436.7)	1196.3 (1023.8 to 1405.3)	1211.8 (1040.4 to 1466.7)	8.8 (1.1 to 18.2)	11.2 (1.3 to 21.6)	6 (-2.4 to 15.8)
High income	Southern Latin America	Argentina	Prevalence	All Ages	2,993,724 (2,667,601 to 3,377,182)	1,617,318 (1,421,188 to 1,837,087	1,376,406 (1,234,045 to 1,563,524)	4,519,829 (4,071,061 to 5,092,282)	2,572,627 (2,290,302 to 2,907,736)	1,947,202 (1,742,999 to 2,210,459)	51 (42.2 to 61.5)	59.1 (48.1 to 70.8)	41.5 (31.6 to 53.3)
High income	Southern Latin America	Argentina	Prevalence	Age-standardized	9122.8 (8138.4 to 10268)	9434.9 (8281.8 to 10704.7)	8882.6 (8035.7 to 9974.5)	9441.3 (8386.9 to 10805.9)	10102.8 (8876.5 to 11589.8)	8787.7 (7806.2 to 10148.3)	3.5 (-3.5 to 11.2)	7.1 (-1.5 to 16.1)	-1.1 (-8.2 to 7.7)
High income	Southern Latin America	Argentina	Deaths	All Ages	9,894 (9,154 to 11,385)	3,641 (3,178 to 5,024)	6,253 (5,806 to 6,754)	19,348 (16,775 to 21,811)	8,772 (6,938 to 10,306)	10,576 (9,132 to 12,056)	95.6 (66.8 to 123.5)	140.9 (79.1 to 197.3)	69.1 (44.7 to 95.7)
High income	Southern Latin America	Argentina	Deaths	Age-standardized	33 (30.2 to 38.6)	21.2 (18.4 to 29.4)	50.1 (46.1 to 54.3)	34.9 (30.3 to 39.3)	25.8 (20.6 to 30)	48.3 (41.4 to 55.3)	5.6 (-11.1 to 20.9)	21.6 (-8.1 to 49.2)	-3.6 (-18.3 to 11.9)
High income	Southern Latin America	Argentina	DALYs	All Ages	341,074 (298,946 to 392,042)	144,795 (119,826 to 176,770)	196,278 (176,314 to 219,242)	529,348 (461,244 to 602,800)	251,733 (210,308 to 296,395)	277,616 (244,765 to 315,911)	55.2 (43.4 to 68)	73.9 (53.9 to 93.1)	41.4 (28.1 to 56.6)
High income	Southern Latin America	Argentina	DALYs	Age-standardized	1057 (927.6 to 1211.3)	836.4 (690.5 to 1018)	1351.9 (1219.9 to 1497.1)	1031.3 (893 to 1194.1)	896.7 (743.7 to 1071.3)	1220.8 (1072.2 to 1391.7)	-2.4 (-9.9 to 5)	7.2 (-4.1 to 18.1)	-9.7 (-18.2 to -0.6)
High income	Southern Latin America	Argentina	YLLs	All Ages	207,957 (195,764 to 228,899)	73,293 (65,762 to 91,469)	134,663 (125,971 to 144,325)	321,485 (284,749 to 356,982)	133,325 (108,773 to 154,060)	188,160 (164,836 to 212,949)	54.6 (36.1 to 74.1)	81.9 (49.8 to 114.5)	39.7 (20.6 to 60.4)
High income	Southern Latin America	Argentina	YLLs	Age-standardized	650.8 (611.4 to 721.8)	419.4 (377.3 to 521.1)	952.3 (891 to 1020.3)	599.3 (532.5 to 665.5)	435.1 (362 to 501.6)	817.9 (718.1 to 924.1)	-7.9 (-18.9 to 3.7)	3.8 (-13.4 to 22)	-14.1 (-25.8 to -1.6)
High income	Southern Latin America	Argentina	YLDs	All Ages	133,117 (94,766 to 179,857)	71,502 (49,778 to 98,345)	61,615 (43,700 to 82,887)	207,863 (151,684 to 278,214)	118,407 (85,293 to 159,854)	89,456 (65,179 to 117,150)	56.2 (45.4 to 68.3)	65.6 (52.4 to 79.8)	45.2 (33.4 to 59.1)
High income	Southern Latin America	Argentina	YLDs	Age-standardized	406.2 (289.7 to 548.5)	417 (290.6 to 574.7)	399.5 (285.9 to 533)	431.9 (312.7 to 580.4)	461.6 (329 to 635.5)	402.9 (295.3 to 535.5)	6.3 (-1.1 to 14.2)	10.7 (1.4 to 20.2)	0.8 (-6.9 to 10.1)
High income	Southern Latin America	Chile	Incidence	All Ages	124,924 (107,311 to 147,972)	61,479 (52,606 to 72,528)	63,445 (54,492 to 74,975)	210,645 (185,584 to 238,892)	108,192 (95,028 to 122,283)	102,453 (89,029 to 119,519)	68.6 (55.1 to 82.9)	76 (57.7 to 93.7)	61.5 (47.5 to 77.4)
High income	Southern Latin America	Chile	Incidence	Age-standardized	1019.1 (891.8 to 1192.3)	967.5 (834.4 to 1129.6)	1078.8 (940.2 to 1243.6)	1093.6 (947.7 to 1283.9)	1062.2 (914.5 to 1244.4)	1127.9 (966.5 to 1349.4)	7.3 (1 to 14.5)	9.8 (2 to 18.3)	4.5 (-2.4 to 13.5)
High income	Southern Latin America	Chile	Prevalence	All Ages	819,004 (728,115 to 945,142)	427,455 (375,735 to 486,786)	391,549 (346,485 to 456,983)	1,424,931 (1,288,202 to 1,586,154)	793,881 (710,570 to 886,492)	631,050 (567,368 to 716,812)	74 (62.3 to 86)	85.7 (71.5 to 99.9)	61.2 (49.3 to 74.3)
High income	Southern Latin America	Chile	Prevalence	Age-standardized	6923.1 (6260.8 to 7839)	6875.6 (6138.8 to 7709.4)	7015.5 (6340.1 to 7942)	7048.6 (6233.2 to 8057.3)	7349.6 (6466.2 to 8329.7)	6745.9 (5976.1 to 7783.9)	1.8 (-4.3 to 8.4)	6.9 (-0.5 to 14.7)	-3.8 (-10.7 to 4.1)
High income	Southern Latin America	Chile	Deaths	All Ages	2,781 (2,444 to 2,956)	1,151 (839 to 1,268)	1,630 (1,509 to 1,749)	6,389 (4,943 to 7,227)	3,128 (1,785 to 3,770)	3,261 (2,812 to 3,696)	129.8 (95.4 to 159.1)	171.8 (105.7 to 222.4)	100.1 (74 to 128.8)
High income	Southern Latin America	Chile	Deaths	Age-standardized	32.3 (28.1 to 34.5)	24.4 (17.6 to 27.1)	42.6 (38.9 to 45.9)	26.9 (20.8 to 30.4)	22.5 (13 to 27)	32.9 (28.5 to 37.2)	-16.8 (-28.6 to -6.7)	-7.8 (-28.5 to 9)	-22.8 (-32.7 to -11.9)
High income	Southern Latin America	Chile	DALYs	All Ages	90,726 (78,355 to 104,594)	40,504 (33,930 to 47,879)	50,222 (44,497 to 57,533)	160,293 (136,186 to 184,274)	80,679 (62,424 to 95,688)	79,614 (69,800 to 90,713)	76.7 (62.9 to 91)	99.2 (75.9 to 120.4)	58.5 (45.1 to 73.6)
High income	Southern Latin America	Chile	DALYs	Age-standardized	867 (756.8 to 982.7)	711.4 (591.6 to 828.4)	1064.3 (959.6 to 1190.4)	721 (609.8 to 845.9)	666.5 (524.8 to 803.3)	796 (694.2 to 914.5)	-16.8 (-22.9 to -10.3)	-6.3 (-14.8 to 2.2)	-25.2 (-31.8 to -18.3)
High income	Southern Latin America	Chile	YLLs	All Ages	54,226 (49,323 to 57,559)	21,477 (16,586 to 23,464)	32,749 (30,594 to 34,969)	93,688 (76,681 to 104,574)	43,524 (27,954 to 51,460)	50,164 (43,970 to 56,815)	72.8 (51.2 to 93.9)	102.6 (61 to 137.6)	53.2 (33.4 to 75.1)
High income	Southern Latin America	Chile	YLLs	Age-standardized	556.4 (499 to 590.7)	404 (304.4 to 441.3)	747.8 (696.9 to 801)	394.6 (324.2 to 440.3)	326.2 (215 to 383.2)	483.4 (424 to 546.9)	-29.1 (-36.9 to -20.7)	-19.3 (-33 to -6.2)	-35.4 (-43.3 to -26.4)
High income	Southern Latin America	Chile	YLDs	All Ages	36,500 (26,130 to 49,787)	19,027 (13,335 to 26,330)	17,473 (12,338 to 24,054)	66,605 (48,734 to 86,584)	37,156 (27,043 to 48,611)	29,450 (21,633 to 38,739)	82.5 (68.1 to 97.9)	95.3 (77.7 to 114.1)	68.5 (53.7 to 85.8)
High income	Southern Latin America	Chile	YLDs	Age-standardized	310.7 (225.4 to 417.7)	307.4 (219.5 to 418.8)	316.5 (227.8 to 421.6)	326.4 (237.9 to 432.6)	340.3 (242.1 to 458.7)	312.6 (227.4 to 417.6)	5.1 (-1.4 to 11.8)	10.7 (2.3 to 18.6)	-1.2 (-8.4 to 6.8)
High income	Southern Latin America	Uruguay	Incidence	All Ages	35,369 (31,026 to 40,905)	15,905 (13,782 to 18,349)	19,464 (17,081 to 22,625)	40,694 (36,372 to 46,306)	20,026 (17,750 to 22,628)	20,668 (18,392 to 23,732)	15.1 (8.5 to 21.6)	25.9 (17 to 35.6)	6.2 (-0.9 to 13.5)
High income	Southern Latin America	Uruguay	Incidence	Age-standardized	1088.8 (945.4 to 1271.8)	950.8 (813.9 to 1110)	1246.2 (1087 to 1456.1)	1093.2 (949.3 to 1291.3)	997.2 (851.9 to 1178)	1199.3 (1036.3 to 1420.4)	0.4 (-5.6 to 6.5)	4.9 (-2.4 to 12.6)	-3.8 (-10.5 to 3.8)
High income	Southern Latin America	Uruguay	Prevalence	All Ages	293,576 (269,994 to 321,114)	132,809 (120,449 to 146,557)	160,767 (146,845 to 177,031)	344,532 (318,402 to 376,311)	175,833 (161,634 to 191,909)	168,698 (155,513 to 184,765)	17.4 (11.7 to 23)	32.4 (24.8 to 39.5)	4.9 (-1.7 to 11.9)
High income	Southern Latin America	Uruguay	Prevalence	Age-standardized	8647.2 (7886.2 to 9578.4)	7502.5 (6743.7 to 8402.8)	10076.4 (9160.7 to 11185.6)	8381.6 (7542.4 to 9439.3)	7903.4 (7038.5 to 9003.9)	9034.4 (8094.4 to 10224.8)	-3.1 (-8.7 to 2.5)	5.3 (-1.9 to 11.7)	-10.3 (-16.9 to -3.7)
High income	Southern Latin America	Uruguay	Deaths	All Ages	1,291 (1,174 to 1,538)	402 (338 to 622)	889 (811 to 973)	2,155 (1,888 to 2,389)	879 (698 to 1,046)	1,275 (1,120 to 1,426)	66.9 (40.4 to 89.5)	119 (44.4 to 178.9)	43.4 (24.2 to 63.2)
High income	Southern Latin America	Uruguay	Deaths	Age-standardized	33.4 (30.3 to 40.1)	17.9 (15.2 to 27.4)	56 (51.1 to 61.1)	35.6 (31.4 to 39.2)	23 (18 to 26.9)	55.4 (48.6 to 61.8)	6.7 (-11.1 to 21.3)	28.3 (-15.8 to 62)	-1.1 (-14.1 to 12.5)
High income	Southern Latin America	Uruguay	DALYs	All Ages	38,594 (34,176 to 43,782)	13,440 (11,427 to 16,963)	25,153 (22,471 to 27,979)	50,257 (44,652 to 55,705)	21,098 (17,627 to 24,174)	29,159 (25,900 to 32,674)	30.2 (16.3 to 43.2)	57 (26.4 to 80)	15.9 (3.5 to 29.2)
High income	Southern Latin America	Uruguay	DALYs	Age-standardized	1055.8 (931.5 to 1207.5)	698.7 (586.8 to 861.5)	1522.8 (1363.1 to 1699.8)	1031.2 (898.5 to 1168)	787.6 (653.3 to 918.4)	1373 (1213.5 to 1556.9)	-2.3 (-11.9 to 6.4)	12.7 (-7.5 to 26.9)	-9.8 (-19.1 to -0.2)
High income	Southern Latin America	Uruguay	YLLs	All Ages	25,364 (23,342 to 28,767)	7,527 (6,598 to 10,455)	17,837 (16,280 to 19,506)	34,111 (30,218 to 37,494)	12,838 (9,920 to 14,842)	21,273 (18,664 to 24,063)	34.5 (14.9 to 52.8)	70.6 (18.7 to 111.3)	19.3 (2.2 to 37.8)
High income	Southern Latin America	Uruguay	YLLs	Age-standardized	667.9 (617.5 to 753.1)	365.6 (323.2 to 489.9)	1065.2 (973.2 to 1163.3)	643.2 (569.5 to 710.7)	420.7 (316 to 483.5)	954.9 (838.2 to 1080.2)	-3.7 (-18 to 9.4)	15.1 (-19.5 to 41.5)	-10.4 (-22.9 to 3.3)
High income	Southern Latin America	Uruguay	YLDs	All Ages	13,230 (9,608 to 17,412)	5,913 (4,220 to 7,896)	7,316 (5,339 to 9,674)	16,146 (12,069 to 20,504)	8,260 (6,136 to 10,644)	7,885 (5,747 to 10,192)	22 (13.1 to 32.3)	39.7 (27.2 to 53.7)	7.8 (-1.9 to 17.7)
High income	Southern Latin America	Uruguay	YLDs	Age-standardized	387.9 (279.1 to 516.5)	333.1 (236.5 to 450.5)	457.6 (332.6 to 606.8)	387.9 (285.1 to 507.4)	367 (266 to 486.9)	418 (305.2 to 547.6)	0 (-6.6 to 6.8)	10.2 (1.5 to 19.2)	-8.7 (-16.4 to -1.1)
		High-income North America	Incidence	All Ages	5,976,779 (5,184,353 to 6,933,632)	3,168,066 (2,767,059 to 3,649,625	2,808,713 (2,407,176 to 3,316,870)	9,936,830 (8,762,136 to 11,103,848	5,314,340 (4,715,155 to 5,955,141)	4,622,490 (4,038,274 to 5,228,576)	66.3 (53.5 to 78.2)	67.7 (57.3 to 78.8)	64.6 (49.6 to 78.6)
		High-income North America	Incidence	Age-standardized	2140.1 (1827.9 to 2567.5)	2166.5 (1862.7 to 2567)	2129.7 (1796.7 to 2577.7)	2445.9 (2134.8 to 2810.4)	2505.3 (2189.2 to 2866.5)	2392.7 (2059.1 to 2784.5)	14.3 (6.9 to 21.4)	15.6 (8.2 to 23)	12.4 (3.8 to 20.6)
		High-income North America	Prevalence	All Ages	34,339,356 (30,978,426 to 38,645,830	19,055,084 (17,158,906 to 21,263,806	15,284,273 (13,660,408 to 17,447,915	52,638,477 (49,184,741 to 56,555,381	29,740,244 (27,770,856 to 31,871,059	22,898,233 (21,305,374 to 24,801,238	53.3 (44.8 to 61.6)	56.1 (47.5 to 64.3)	49.8 (40.5 to 59.3)
		High-income North America	Prevalence	Age-standardized	11647.4 (10322 to 13333.1)	12062.8 (10660.4 to 13745.4)	11279.6 (9911.7 to 13080)	12449.6 (11351.8 to 13803.5)	13208.5 (12090.5 to 14571.7)	11642.9 (10511.3 to 13005.5)	6.9 (1.1 to 13.3)	9.5 (3 to 16.7)	3.2 (-2.8 to 9.9)
		High-income North America	Deaths	All Ages	115,443 (107,761 to 133,643)	50,102 (44,853 to 66,716)	65,341 (62,590 to 67,186)	243,409 (196,086 to 260,316)	121,023 (76,342 to 134,340)	122,385 (113,326 to 128,065)	110.8 (64.3 to 125.2)	141.6 (42.9 to 169.9)	87.3 (80.3 to 94.2)
		High-income North America	Deaths	Age-standardized	31.3 (29.2 to 36.2)	22.9 (20.8 to 29.9)	45.3 (43.1 to 46.6)	36.2 (29.6 to 38.6)	31.6 (20.6 to 34.7)	42.7 (39.5 to 44.7)	15.8 (-9 to 23.4)	38 (-14.7 to 52.8)	-5.6 (-8.9 to -2.2)
		High-income North America	DALYs	All Ages	4,312,309 (3,807,518 to 4,869,085)	2,113,466 (1,818,306 to 2,458,862	2,198,844 (1,977,672 to 2,461,569)	7,451,116 (6,536,326 to 8,268,824)	3,862,531 (3,157,203 to 4,337,025)	3,588,585 (3,260,772 to 3,936,816)	72.8 (55.3 to 80.7)	82.8 (49.8 to 95.3)	63.2 (57.6 to 68.7)
		High-income North America	DALYs	Age-standardized	1309.3 (1138.9 to 1514.6)	1174.2 (993.1 to 1393.3)	1522.5 (1360.3 to 1723.4)	1374 (1181 to 1570.2)	1334.6 (1097.1 to 1549.5)	1431.2 (1264.8 to 1622.9)	4.9 (-4.6 to 8.9)	13.7 (-3.5 to 20)	-6 (-8.6 to -3)
		High-income North America	YLLs	All Ages	2,114,811 (2,015,719 to 2,394,482)	910,016 (842,748 to 1,157,670)	1,204,795 (1,170,138 to 1,232,219)	3,924,671 (3,294,638 to 4,128,741)	1,898,925 (1,274,586 to 2,054,296)	2,025,746 (1,924,326 to 2,102,629)	85.6 (52.3 to 95.6)	108.7 (37.3 to 128.1)	68.1 (62.6 to 73.8)
		High-income North America	YLLs	Age-standardized	599.6 (573.1 to 676.6)	459.4 (429.3 to 581.2)	806.9 (781.4 to 826)	629.6 (531.9 to 660)	560 (385.4 to 601.1)	718.1 (682.2 to 744.1)	5 (-12.8 to 10.5)	21.9 (-16.6 to 32.5)	-11 (-13.9 to -8.1)
		High-income North America	YLDs	All Ages	2,197,498 (1,713,767 to 2,751,407)	1,203,449 (926,206 to 1,500,131)	994,049 (769,276 to 1,249,805)	3,526,445 (2,793,830 to 4,267,071)	1,963,606 (1,557,412 to 2,373,425)	1,562,839 (1,242,500 to 1,895,026)	60.5 (52.3 to 68.1)	63.2 (55 to 71.1)	57.2 (49 to 65.4)
		High-income North America	YLDs	Age-standardized	709.7 (545 to 910.5)	714.8 (542.7 to 921.2)	715.6 (548.8 to 910.4)	744.4 (568.5 to 936.9)	774.6 (586.5 to 984.9)	713.1 (547 to 890)	4.9 (0.4 to 9.6)	8.4 (3.1 to 13.7)	-0.4 (-4.8 to 4.5)
High income	High-income North America	Canada	Incidence	All Ages	405,590 (359,689 to 462,220)	196,432 (172,394 to 225,873)	209,159 (184,119 to 239,341)	663,786 (580,997 to 760,807)	332,422 (290,929 to 377,937)	331,364 (286,420 to 383,049)	63.7 (50 to 77.2)	69.2 (52.6 to 84.1)	58.4 (44.7 to 74.8)
High income	High-income North America	Canada	Incidence	Age-standardized	1487.7 (1294.6 to 1732.2)	1377.4 (1192.5 to 1637.7)	1622.8 (1410.5 to 1891.2)	1497.9 (1301.8 to 1754.5)	1427.3 (1227.7 to 1654.6)	1571.3 (1348.6 to 1871.5)	0.7 (-7.7 to 6.6)	3.6 (-9 to 11.5)	-3.2 (-10 to 4.4)
High income	High-income North America	Canada	Prevalence	All Ages	2,148,690 (1,921,460 to 2,445,514)	1,089,957 (976,233 to 1,247,438)	1,058,732 (929,830 to 1,209,785)	3,310,838 (3,022,305 to 3,634,133)	1,794,309 (1,643,573 to 1,966,080)	1,516,529 (1,378,382 to 1,678,099)	54.1 (36.2 to 64.3)	64.6 (43.6 to 77.6)	43.2 (28 to 54.1)
High income	High-income North America	Canada	Prevalence	Age-standardized	7589.5 (6678.8 to 8735.5)	7239.3 (6353.3 to 8530.2)	8096.5 (7066.8 to 9324.5)	7199.4 (6329.2 to 8328.5)	7225.2 (6349.9 to 8315.4)	7155.9 (6178.5 to 8388.9)	-5.1 (-16.8 to 0.6)	-0.2 (-15.7 to 8.2)	-11.6 (-20.8 to -5.1)
High income	High-income North America	Canada	Deaths	All Ages	9,319 (8,590 to 10,076)	3,379 (2,942 to 3,936)	5,940 (5,545 to 6,279)	18,385 (14,506 to 20,999)	8,537 (5,204 to 10,424)	9,848 (8,405 to 11,214)	97.3 (60.4 to 123.5)	152.6 (64.6 to 208.3)	65.8 (43.6 to 89.3)
High income	High-income North America	Canada	Deaths	Age-standardized	28.8 (26.4 to 31)	17.6 (15.3 to 20.6)	47.3 (43.7 to 50.2)	24.2 (19.5 to 27.6)	19.6 (12.6 to 23.6)	30.8 (26.2 to 35)	-15.8 (-30.3 to -5.4)	11.5 (-23.6 to 34)	-35.1 (-43.8 to -25.8)
High income	High-income North America	Canada	DALYs	All Ages	269,100 (239,674 to 302,712)	113,229 (97,707 to 131,480)	155,871 (139,634 to 174,154)	446,332 (388,801 to 499,640)	218,676 (179,733 to 248,165)	227,656 (199,895 to 256,899)	65.9 (49.3 to 79.2)	93.1 (59.5 to 117)	46.1 (33.8 to 60)
High income	High-income North America	Canada	DALYs	Age-standardized	868 (763 to 993.3)	675 (571 to 794.9)	1152.8 (1030 to 1293.8)	744.4 (640.9 to 860.9)	685.5 (569.9 to 797.4)	820.1 (709.7 to 952.4)	-14.2 (-21.8 to -8.5)	1.6 (-13 to 11.2)	-28.9 (-34.8 to -22.8)
High income	High-income North America	Canada	YLLs	All Ages	158,255 (148,921 to 171,827)	57,477 (51,818 to 68,508)	100,778 (94,603 to 106,474)	263,607 (221,258 to 293,688)	119,162 (82,201 to 139,707)	144,445 (126,153 to 163,353)	66.6 (40.9 to 86.6)	107.3 (48.5 to 144.5)	43.3 (24.4 to 63.7)
High income	High-income North America	Canada	YLLs	Age-standardized	485.1 (456.6 to 527.3)	315 (286.4 to 377.8)	735.2 (689.4 to 775.7)	375.1 (320.8 to 416.9)	313.4 (227.1 to 361.9)	454 (397.1 to 511)	-22.7 (-33.4 to -13.6)	-0.5 (-25.5 to 16.4)	-38.3 (-46.2 to -29.9)
High income	High-income North America	Canada	YLDs	All Ages	110,845 (83,561 to 141,892)	55,752 (42,373 to 70,791)	55,093 (41,031 to 72,841)	182,725 (142,221 to 222,475)	99,514 (78,242 to 120,975)	83,211 (63,009 to 104,596)	64.8 (48.2 to 79.6)	78.5 (56.3 to 98.8)	51 (37.4 to 64.1)
High income	High-income North America	Canada	YLDs	Age-standardized	382.9 (288 to 500.2)	360 (267.6 to 471.8)	417.6 (310.3 to 557.6)	369.3 (276.9 to 477.4)	372.1 (284 to 477.5)	366.2 (266.1 to 486.6)	-3.6 (-13.3 to 2.2)	3.4 (-10.1 to 12.4)	-12.3 (-19.6 to -6.4)
High income	High-income North America	Greenland	Incidence	All Ages	1,003 (851 to 1,183)	497 (415 to 589)	505 (419 to 610)	1,049 (925 to 1,196)	520 (459 to 592)	529 (461 to 610)	4.6 (-4.7 to 16.4)	4.6 (-6.3 to 22.5)	4.7 (-7.2 to 18.4)
High income	High-income North America	Greenland	Incidence	Age-standardized	2115.5 (1808.1 to 2460.6)	2195.3 (1857.1 to 2562.5)	2091.4 (1757 to 2480.9)	1785.5 (1552.6 to 2075.4)	1855.7 (1625.9 to 2138.6)	1725.3 (1486.7 to 2058)	-15.6 (-20.7 to -8.8)	-15.5 (-23 to -3.5)	-17.5 (-23.9 to -10.8)
High income	High-income North America	Greenland	Prevalence	All Ages	5,754 (4,857 to 6,597)	3,151 (2,512 to 3,633)	2,603 (2,229 to 3,002)	5,522 (5,091 to 6,053)	3,081 (2,848 to 3,342)	2,441 (2,203 to 2,729)	-4 (-11.2 to 8.7)	-2.2 (-11.8 to 18.6)	-6.2 (-13.3 to 2.6)
High income	High-income North America	Greenland	Prevalence	Age-standardized	12507.7 (10707 to 14166.1)	14373.5 (11525 to 16364.1)	11065.3 (9652.1 to 12609.7)	9412.8 (8507.9 to 10584)	10754.9 (9853 to 11881)	8245.3 (7240.1 to 9496.3)	-24.7 (-30.1 to -15.1)	-25.2 (-32.4 to -10)	-25.5 (-30.7 to -19.3)
High income	High-income North America	Greenland	Deaths	All Ages	24 (19 to 27)	15 (11 to 17)	9 (8 to 10)	32 (24 to 38)	17 (11 to 21)	15 (12 to 17)	34.6 (9.2 to 67.7)	18.1 (-10.6 to 59.6)	61.3 (27.4 to 99.7)
High income	High-income North America	Greenland	Deaths	Age-standardized	92.8 (73 to 105.6)	103.7 (74.6 to 121.8)	80.3 (65.5 to 90.9)	56.3 (40.9 to 66.5)	61.3 (39.3 to 75.6)	52.1 (40.8 to 60.9)	-39.3 (-49.9 to -25.2)	-40.9 (-54.6 to -21.1)	-35.1 (-47.6 to -21.2)
High income	High-income North America	Greenland	DALYs	All Ages	833 (712 to 949)	490 (406 to 567)	344 (291 to 401)	955 (794 to 1,102)	522 (420 to 613)	433 (358 to 503)	14.6 (-1.6 to 36.2)	6.7 (-10.9 to 33.3)	25.9 (6.4 to 49.7)
High income	High-income North America	Greenland	DALYs	Age-standardized	2406 (2043.3 to 2700.6)	2783.8 (2273.8 to 3190.7)	2039.4 (1763.4 to 2314.5)	1517.2 (1259.4 to 1749.9)	1733.1 (1377.7 to 2039.7)	1332.1 (1100.7 to 1535.8)	-36.9 (-45.1 to -26.1)	-37.7 (-47.4 to -23)	-34.7 (-44.7 to -24)
High income	High-income North America	Greenland	YLLs	All Ages	537 (446 to 609)	324 (254 to 381)	213 (179 to 247)	623 (476 to 745)	332 (238 to 414)	291 (226 to 351)	16.1 (-7.9 to 47.8)	2.5 (-23.6 to 42.8)	36.7 (6 to 73.6)
High income	High-income North America	Greenland	YLLs	Age-standardized	1715.4 (1395.7 to 1933.5)	1983.3 (1511.4 to 2337)	1440.3 (1201.3 to 1650.6)	967.6 (731.3 to 1150.2)	1085.3 (761.7 to 1343.9)	868.9 (682.4 to 1033.3)	-43.6 (-54.4 to -28.9)	-45.3 (-58.2 to -24.6)	-39.7 (-52.4 to -25)
High income	High-income North America	Greenland	YLDs	All Ages	296 (216 to 393)	166 (119 to 220)	131 (94 to 181)	331 (258 to 413)	190 (151 to 235)	141 (106 to 181)	11.9 (-0.4 to 28)	14.7 (0.4 to 38)	8.2 (-3.6 to 22.2)
High income	High-income North America	Greenland	YLDs	Age-standardized	690.6 (524.3 to 895.5)	800.5 (585.9 to 1041.9)	599.1 (444.8 to 795)	549.6 (422.6 to 698.2)	647.8 (508.6 to 810.5)	463.3 (345.4 to 598.7)	-20.4 (-26.3 to -11.8)	-19.1 (-27.1 to -5.2)	-22.7 (-28 to -16.8)
High income	High-income North America	United States of America	Incidence	All Ages	5,570,049 (4,827,880 to 6,493,878)	2,971,065 (2,585,466 to 3,424,508	2,598,984 (2,223,106 to 3,081,539)	9,271,837 (8,170,488 to 10,346,925	4,981,313 (4,409,512 to 5,579,735)	4,290,524 (3,739,989 to 4,851,531)	66.5 (53.1 to 78.8)	67.7 (57 to 79.3)	65.1 (49.3 to 80.1)
High income	High-income North America	United States of America	Incidence	Age-standardized	2209.4 (1876.3 to 2663)	2250 (1932.2 to 2675.1)	2183.5 (1831.2 to 2653.2)	2550 (2228.8 to 2931.8)	2622.9 (2292.2 to 2996.7)	2483.8 (2136.5 to 2894.9)	15.4 (7.4 to 23.1)	16.6 (8.4 to 24.3)	13.8 (4.5 to 22.8)
High income	High-income North America	United States of America	Prevalence	All Ages	32,184,128 (29,006,940 to 36,375,344	17,961,539 (16,146,139 to 20,016,500	14,222,588 (12,710,652 to 16,266,907	49,321,281 (46,068,479 to 52,947,606	27,942,382 (26,107,284 to 29,954,325	21,378,899 (19,902,793 to 23,116,445	53.2 (44.2 to 61.9)	55.6 (46.8 to 64.1)	50.3 (40.4 to 60.6)
High income	High-income North America	United States of America	Prevalence	Age-standardized	12084 (10706.7 to 13846.3)	12575.6 (11099.4 to 14348.4)	11626.2 (10204 to 13477.2)	13030.4 (11908.3 to 14412)	13871 (12712.2 to 15268.2)	12138.3 (10964.4 to 13514.4)	7.8 (1.6 to 14.5)	10.3 (3.1 to 17.8)	4.4 (-2.1 to 11.7)
High income	High-income North America	United States of America	Deaths	All Ages	106,098 (98,990 to 124,041)	46,707 (41,676 to 62,787)	59,391 (56,950 to 60,990)	224,988 (181,616 to 240,468)	112,467 (71,153 to 124,676)	112,521 (104,079 to 117,708)	112.1 (63.9 to 126.6)	140.8 (41.7 to 169.6)	89.5 (82.6 to 96.5)
High income	High-income North America	United States of America	Deaths	Age-standardized	31.6 (29.6 to 36.8)	23.4 (21.2 to 30.9)	45.2 (43.1 to 46.5)	37.7 (30.8 to 40.2)	33 (21.5 to 36.2)	44.2 (40.8 to 46.3)	19.3 (-6.7 to 27)	41 (-13.5 to 56.4)	-2.1 (-5.1 to 1.4)
High income	High-income North America	United States of America	DALYs	All Ages	4,042,277 (3,568,362 to 4,568,025)	1,999,699 (1,717,588 to 2,329,282	2,042,578 (1,834,622 to 2,291,394)	7,003,710 (6,141,549 to 7,768,072)	3,643,271 (2,978,719 to 4,098,273)	3,360,439 (3,048,783 to 3,690,174)	73.3 (55.2 to 81.3)	82.2 (49.1 to 94.8)	64.5 (58.7 to 70.2)
High income	High-income North America	United States of America	DALYs	Age-standardized	1356.6 (1179.7 to 1568.6)	1226.3 (1034.3 to 1456.9)	1563.8 (1392.5 to 1768.3)	1447.8 (1244.5 to 1654.9)	1409.8 (1157.7 to 1633.7)	1503.9 (1328.1 to 1704.7)	6.7 (-3.1 to 11.1)	15 (-2.5 to 21.5)	-3.8 (-6.7 to -0.8)
High income	High-income North America	United States of America	YLLs	All Ages	1,955,971 (1,864,654 to 2,226,085)	852,195 (788,010 to 1,089,815)	1,103,776 (1,070,570 to 1,127,862)	3,660,378 (3,055,696 to 3,848,418)	1,779,401 (1,192,730 to 1,922,311)	1,880,977 (1,789,907 to 1,953,184)	87.1 (52.8 to 97.5)	108.8 (36.5 to 128.7)	70.4 (64.8 to 76.1)
High income	High-income North America	United States of America	YLLs	Age-standardized	612.3 (585.6 to 693.4)	474.5 (444 to 601.5)	816.1 (791.3 to 834.4)	660.4 (554.6 to 692.4)	589.4 (402.4 to 632.2)	750.6 (713.8 to 778.8)	7.8 (-10.8 to 13.5)	24.2 (-15.6 to 35.3)	-8 (-10.9 to -5)
High income	High-income North America	United States of America	YLDs	All Ages	2,086,306 (1,629,377 to 2,605,207)	1,147,504 (885,116 to 1,430,326)	938,803 (727,917 to 1,179,631)	3,343,332 (2,644,497 to 4,044,454)	1,863,871 (1,472,651 to 2,255,661)	1,479,461 (1,176,559 to 1,794,687)	60.3 (52 to 67.9)	62.4 (54.2 to 70.3)	57.6 (49.3 to 66)
High income	High-income North America	United States of America	YLDs	Age-standardized	744.3 (572.8 to 954.5)	751.9 (571.4 to 969.4)	747.7 (572.2 to 949)	787.4 (601.9 to 989.4)	820.4 (618.3 to 1042.9)	753.3 (578.7 to 939.2)	5.8 (0.8 to 10.9)	9.1 (3.7 to 14.7)	0.8 (-4 to 5.9)
		Latin America and Caribbean	Incidence	All Ages	5,222,438 (4,181,982 to 6,503,236)	2,658,723 (2,159,517 to 3,278,888	2,563,714 (2,017,213 to 3,225,872)	6,560,855 (5,488,918 to 7,819,117)	3,428,670 (2,900,974 to 4,065,691)	3,132,185 (2,601,905 to 3,783,444)	25.6 (17.5 to 34.7)	29 (20.3 to 38.1)	22.2 (14.7 to 31.1)
		Latin America and Caribbean	Incidence	Age-standardized	1322.9 (1109.6 to 1583.4)	1342.9 (1129.2 to 1597)	1301.2 (1084.2 to 1567.5)	1197.5 (991.9 to 1442.5)	1213.1 (1005.1 to 1452.2)	1181.9 (976.6 to 1434.5)	-9.5 (-12.8 to -6.5)	-9.7 (-13.4 to -6.2)	-9.2 (-12.6 to -6.3)
		Latin America and Caribbean	Prevalence	All Ages	27,680,068 (23,167,670 to 33,609,803	14,263,121 (11,982,147 to 17,115,678	13,416,947 (11,062,330 to 16,479,853	35,567,363 (30,762,828 to 41,387,677	18,594,361 (16,198,087 to 21,445,410	16,973,003 (14,581,206 to 19,977,303	28.5 (21.3 to 36.7)	30.4 (22.5 to 38.7)	26.5 (19.7 to 34.5)
		Latin America and Caribbean	Prevalence	Age-standardized	7378.2 (6453.7 to 8592.2)	7525 (6579.7 to 8718.3)	7230 (6290.7 to 8463.9)	6287.2 (5387.1 to 7386.1)	6339.4 (5448.3 to 7405.9)	6246 (5353.9 to 7381.9)	-14.8 (-18.1 to -11.6)	-15.8 (-19.4 to -12.4)	-13.6 (-16.9 to -10.5)
		Latin America and Caribbean	Deaths	All Ages	85,247 (78,564 to 88,637)	36,444 (30,609 to 38,897)	48,803 (46,161 to 50,661)	180,256 (159,394 to 197,876)	84,302 (69,964 to 95,552)	95,954 (86,063 to 105,042)	111.5 (94.3 to 128.5)	131.3 (106.7 to 154)	96.6 (81.3 to 114)
		Latin America and Caribbean	Deaths	Age-standardized	44.4 (40.3 to 46.6)	35.6 (30.4 to 38.6)	54.6 (50.8 to 57)	32.5 (28.7 to 35.7)	27 (22.4 to 30.6)	39.6 (35.4 to 43.5)	-26.7 (-32.2 to -21)	-24.2 (-32 to -17)	-27.5 (-32.8 to -21.1)
		Latin America and Caribbean	DALYs	All Ages	3,283,904 (2,868,313 to 3,822,003)	1,533,950 (1,296,694 to 1,816,434	1,749,954 (1,540,065 to 2,021,111)	4,774,225 (4,255,489 to 5,374,264)	2,288,915 (2,000,597 to 2,626,334)	2,485,311 (2,228,236 to 2,782,350)	45.4 (35.4 to 56.6)	49.2 (37.9 to 63.7)	42 (32 to 53.3)
		Latin America and Caribbean	DALYs	Age-standardized	1163.4 (1055.5 to 1287.7)	1018.7 (885.8 to 1147.1)	1327.8 (1218.7 to 1453.4)	837.6 (744.7 to 946.1)	751.2 (654.4 to 865.9)	944 (846.5 to 1053.4)	-28 (-31.8 to -23.6)	-26.3 (-31.1 to -20.8)	-28.9 (-33.1 to -24.1)
		Latin America and Caribbean	YLLs	All Ages	2,070,089 (1,892,241 to 2,163,540)	900,618 (742,295 to 957,239)	1,169,471 (1,101,959 to 1,226,116)	3,166,352 (2,859,624 to 3,492,159)	1,429,058 (1,235,278 to 1,614,875)	1,737,294 (1,581,506 to 1,899,897)	53 (40.6 to 68)	58.7 (42.7 to 79.9)	48.6 (35.3 to 62.9)
		Latin America and Caribbean	YLLs	Age-standardized	838.4 (773.9 to 871.3)	681.7 (571.8 to 721.9)	1015.3 (963.8 to 1053.6)	554.4 (499.4 to 610.7)	459.5 (397.9 to 519.4)	669.7 (608.5 to 732.7)	-33.9 (-38.9 to -28)	-32.6 (-39.2 to -24.8)	-34 (-39.2 to -27.7)
		Latin America and Caribbean	YLDs	All Ages	1,213,815 (831,183 to 1,723,739)	633,331 (437,297 to 892,117)	580,483 (396,069 to 827,749)	1,607,873 (1,161,706 to 2,142,351)	859,857 (625,698 to 1,134,048)	748,016 (531,000 to 1,012,509)	32.5 (21.2 to 47.6)	35.8 (23.7 to 51)	28.9 (16.9 to 43.8)
		Latin America and Caribbean	YLDs	Age-standardized	325 (232.4 to 440.2)	337 (240 to 453.1)	312.5 (220.5 to 427.3)	283.2 (203.9 to 381.6)	291.7 (209.8 to 389.3)	274.3 (194.6 to 373.3)	-12.9 (-16.8 to -9.1)	-13.4 (-17.3 to -9.5)	-12.2 (-16.2 to -8.4)
		Caribbean	Incidence	All Ages	463,274 (381,499 to 561,831)	243,322 (203,114 to 290,423)	219,952 (176,658 to 272,040)	556,221 (470,071 to 659,079)	292,173 (249,771 to 341,653)	264,048 (220,689 to 318,711)	20.1 (14.8 to 26.4)	20.1 (13.5 to 26.8)	20 (14.1 to 27.4)
		Caribbean	Incidence	Age-standardized	1247.2 (1041.3 to 1494.4)	1301.9 (1104.1 to 1541.6)	1188.7 (973.4 to 1447.7)	1237.4 (1030 to 1487.3)	1275.7 (1077.5 to 1510.2)	1197.3 (991 to 1460.6)	-0.8 (-4.2 to 2.4)	-2 (-6.5 to 2.1)	0.7 (-3.2 to 4.7)
		Caribbean	Prevalence	All Ages	2,937,317 (2,546,756 to 3,416,900)	1,591,845 (1,390,996 to 1,827,061	1,345,472 (1,144,184 to 1,590,845)	3,643,997 (3,236,341 to 4,150,963)	1,959,803 (1,747,203 to 2,206,505)	1,684,194 (1,470,580 to 1,937,361)	24.1 (19.3 to 29.5)	23.1 (17.8 to 29.2)	25.2 (19.5 to 31.4)
		Caribbean	Prevalence	Age-standardized	8331.6 (7374.8 to 9528.2)	8943.9 (7919.4 to 10122)	7684.1 (6713.2 to 8887.2)	7804.4 (6858.7 to 8982.4)	8172.8 (7196.6 to 9299.7)	7424.2 (6436.7 to 8600.8)	-6.3 (-9.5 to -3.3)	-8.6 (-12.4 to -4.9)	-3.4 (-7.1 to 0.3)
		Caribbean	Deaths	All Ages	7,309 (6,432 to 8,172)	3,137 (2,610 to 3,747)	4,173 (3,505 to 4,672)	13,766 (11,412 to 16,209)	5,975 (4,313 to 7,269)	7,791 (6,504 to 9,062)	88.3 (61.3 to 116)	90.5 (49 to 125.6)	86.7 (59.1 to 119.2)
		Caribbean	Deaths	Age-standardized	28.3 (24.9 to 31.7)	23.2 (19.2 to 28.2)	34.1 (29.1 to 37.8)	26.9 (22.2 to 31.7)	21.4 (15.5 to 26)	33.5 (27.9 to 39.1)	-5 (-19.2 to 9.3)	-7.5 (-27.9 to 10.3)	-1.8 (-16 to 14.5)
		Caribbean	DALYs	All Ages	347,478 (286,973 to 414,111)	165,541 (136,299 to 199,410)	181,936 (142,938 to 219,158)	465,876 (389,501 to 539,116)	219,994 (176,535 to 258,175)	245,883 (201,106 to 287,682)	34.1 (18.6 to 51.7)	32.9 (15.1 to 51)	35.1 (16.2 to 58.5)

Chronic respiratory diseases

7 GBD super region	21 GBD region	Location	Measure	Age	1990			2019			Percent change (1990 to 2019)		
					Both	Female	Male	Both	Female	Male	Both	Female	Male
		Caribbean	DALYs	Age-standardized	1082.2 (917.7 to 1261.6)	1004.9 (836.9 to 1187.9)	1165.2 (946.4 to 1368.1)	960.2 (797.9 to 1118.5)	872.7 (705.7 to 1025.8)	1060.5 (860.1 to 1249.5)	-11.3 (-20.4 to -0.9)	-13.2 (-23.1 to -2.9)	-9 (-20.5 to 4.3)
		Caribbean	YLLs	All Ages	219,446 (178,252 to 257,287)	95,717 (77,009 to 115,265)	123,729 (91,933 to 152,599)	302,201 (246,234 to 361,709)	130,702 (97,856 to 159,546)	171,499 (135,095 to 207,971)	37.7 (13.9 to 65.9)	36.6 (8 to 66.6)	38.6 (11.2 to 71.7)
		Caribbean	YLLs	Age-standardized	717.7 (603.9 to 817.5)	610.6 (502.8 to 714.4)	832.2 (657.9 to 984.6)	610.7 (495.3 to 735.2)	502.2 (377.1 to 614.5)	733 (573.1 to 892.7)	-14.9 (-28 to 0.1)	-17.8 (-33 to -0.5)	-11.9 (-27.5 to 7.1)
		Caribbean	YLDs	All Ages	128,032 (87,359 to 178,378)	69,825 (47,639 to 96,624)	58,207 (39,318 to 82,907)	163,676 (116,628 to 217,698)	89,292 (64,209 to 117,857)	74,384 (51,969 to 100,991)	27.8 (19.5 to 38.9)	27.9 (19.2 to 39.3)	27.8 (18.3 to 39.3)
		Caribbean	YLDs	Age-standardized	364.5 (253.9 to 501)	394.3 (272.6 to 539.7)	333 (229.9 to 464.2)	349.5 (247.7 to 473.1)	370.5 (263.4 to 496.8)	327.5 (227.7 to 450.8)	-4.1 (-8.5 to 0.6)	-6 (-10.8 to -0.7)	-1.7 (-7.1 to 3.8)
Latin America and Caribbean	Caribbean	Antigua and Barbuda	Incidence	All Ages	663 (533 to 838)	340 (278 to 420)	324 (252 to 420)	836 (694 to 999)	453 (382 to 538)	383 (314 to 464)	26.1 (17 to 36)	33.5 (23.2 to 45.7)	18.3 (7.6 to 29.5)
Latin America and Caribbean	Caribbean	Antigua and Barbuda	Incidence	Age-standardized	1055.6 (856.1 to 1324.2)	1055 (866.2 to 1307.6)	1055.7 (835.2 to 1351.8)	1141.3 (917 to 1405.9)	1178.5 (947.3 to 1449.8)	1097.7 (866.2 to 1379.2)	8.1 (3.4 to 13.3)	11.7 (4.7 to 19.4)	4 (-2.9 to 10.5)
Latin America and Caribbean	Caribbean	Antigua and Barbuda	Prevalence	All Ages	3,721 (3,046 to 4,613)	1,906 (1,567 to 2,313)	1,815 (1,465 to 2,318)	5,189 (4,422 to 6,155)	2,776 (2,389 to 3,310)	2,412 (2,016 to 2,891)	39.4 (30 to 50.6)	45.7 (34.5 to 59.7)	32.9 (20.7 to 45.5)
Latin America and Caribbean	Caribbean	Antigua and Barbuda	Prevalence	Age-standardized	5997.4 (4963.7 to 7355.7)	5943.1 (4939.1 to 7181.2)	6082.7 (5034.8 to 7612.1)	6458.8 (5288.7 to 7846.1)	6583.3 (5443.2 to 7938.2)	6304 (5092.6 to 7713.4)	7.7 (1.9 to 13.6)	10.8 (3.1 to 19.7)	3.6 (-3.9 to 11.9)
Latin America and Caribbean	Caribbean	Antigua and Barbuda	Deaths	All Ages	7 (6 to 8)	3 (2 to 3)	4 (4 to 5)	11 (9 to 13)	5 (3 to 6)	6 (5 to 7)	57.7 (28.5 to 91.5)	80.9 (36.2 to 132.5)	43.7 (12 to 85.5)
Latin America and Caribbean	Caribbean	Antigua and Barbuda	Deaths	Age-standardized	11.6 (10.3 to 13.1)	7.1 (5.9 to 8.5)	19.8 (17.2 to 23)	11.9 (9.7 to 14.2)	9.3 (6.7 to 11.4)	15.6 (12.5 to 19.2)	2.8 (-16.3 to 23.7)	29.9 (-2.8 to 66.5)	-21.6 (-39.3 to 0.3)
Latin America and Caribbean	Caribbean	Antigua and Barbuda	DALYs	All Ages	282 (227 to 357)	128 (99 to 166)	154 (127 to 192)	435 (354 to 534)	213 (168 to 265)	222 (178 to 272)	54.2 (37.3 to 73.1)	66.1 (44.7 to 90.4)	44.2 (25.2 to 67.8)
Latin America and Caribbean	Caribbean	Antigua and Barbuda	DALYs	Age-standardized	477.7 (391.1 to 595.2)	403.2 (314.5 to 520.5)	591.4 (498.2 to 712.2)	501.5 (398.7 to 628.5)	467.5 (361.4 to 597.8)	542.9 (428.4 to 675)	5 (-5.2 to 15.9)	16 (2.5 to 31.2)	-8.2 (-19.8 to 5.6)
Latin America and Caribbean	Caribbean	Antigua and Barbuda	YLLs	All Ages	123 (110 to 136)	46 (39 to 54)	77 (67 to 88)	207 (168 to 251)	89 (70 to 112)	117 (93 to 147)	68.4 (35.5 to 107.1)	94 (45 to 150)	53 (18.6 to 97.6)
Latin America and Caribbean	Caribbean	Antigua and Barbuda	YLLs	Age-standardized	219.9 (196.8 to 245.7)	146.2 (124.1 to 173.7)	331.7 (286.6 to 379.8)	217.9 (178.4 to 263.9)	175.8 (130.9 to 221.3)	269.1 (216.4 to 332.5)	-0.9 (-20 to 20.9)	20.3 (-10.6 to 56.5)	-18.9 (-36.4 to 3.9)
Latin America and Caribbean	Caribbean	Antigua and Barbuda	YLDs	All Ages	160 (107 to 234)	82 (55 to 119)	77 (51 to 114)	229 (157 to 319)	124 (86 to 172)	105 (71 to 149)	43.2 (31.7 to 56.7)	50.4 (36.7 to 66.8)	35.5 (21.3 to 51.1)
Latin America and Caribbean	Caribbean	Antigua and Barbuda	YLDs	Age-standardized	257.9 (174.3 to 373.1)	257 (174 to 371.9)	259.8 (175.3 to 376.6)	283.6 (190.2 to 405.6)	291.7 (198.5 to 419.1)	273.8 (182.3 to 398.1)	10 (3.3 to 16.8)	13.5 (4.8 to 23.6)	5.4 (-3.2 to 14.6)
Latin America and Caribbean	Caribbean	Bahamas	Incidence	All Ages	2,873 (2,327 to 3,585)	1,474 (1,211 to 1,841)	1,399 (1,104 to 1,788)	3,917 (3,266 to 4,664)	2,123 (1,774 to 2,515)	1,793 (1,483 to 2,181)	36.3 (26.6 to 47.9)	44 (30.9 to 58.6)	28.2 (18.7 to 41.3)
Latin America and Caribbean	Caribbean	Bahamas	Incidence	Age-standardized	1141.1 (940.4 to 1401.6)	1147.1 (956.8 to 1402.9)	1130.3 (915.3 to 1411.6)	1218.8 (990.2 to 1504.1)	1245.6 (1013.5 to 1524.9)	1183.5 (948.4 to 1479.9)	6.8 (1.1 to 12.7)	8.6 (0.9 to 17.2)	4.7 (-2.5 to 12.4)
Latin America and Caribbean	Caribbean	Bahamas	Prevalence	All Ages	16,528 (13,741 to 19,840)	8,216 (6,796 to 9,886)	8,313 (6,813 to 10,131)	23,202 (19,866 to 27,404)	11,693 (10,001 to 13,926)	11,509 (9,773 to 13,783)	40.4 (31.8 to 49.8)	42.3 (30.3 to 56.5)	38.5 (28.1 to 49.8)
Latin America and Caribbean	Caribbean	Bahamas	Prevalence	Age-standardized	6473.9 (5510.2 to 7631.1)	6203 (5240.7 to 7350)	6810.9 (5797.5 to 8093)	6648.8 (5578.3 to 8028.2)	6411.8 (5335.3 to 7763.2)	6916.5 (5724.7 to 8440)	2.7 (-3.1 to 9.3)	3.4 (-4.8 to 12.9)	1.6 (-5.9 to 9.3)
Latin America and Caribbean	Caribbean	Bahamas	Deaths	All Ages	24 (21 to 27)	9 (8 to 11)	14 (12 to 17)	51 (41 to 63)	19 (15 to 25)	32 (25 to 40)	115.6 (69.7 to 170.5)	109.5 (57.5 to 176.8)	119.4 (67.8 to 188.4)
Latin America and Caribbean	Caribbean	Bahamas	Deaths	Age-standardized	16.4 (14.4 to 18.5)	10.8 (8.7 to 13.1)	25.9 (22.1 to 30.1)	14.6 (11.7 to 18.1)	9.7 (7.3 to 12.4)	21.5 (16.9 to 26.9)	-10.9 (-29.5 to 11.6)	-10.3 (-33.1 to 19)	-16.7 (-36 to 7.6)
Latin America and Caribbean	Caribbean	Bahamas	DALYs	All Ages	1,329 (1,092 to 1,658)	601 (480 to 770)	728 (597 to 887)	2,211 (1,822 to 2,689)	975 (778 to 1,203)	1,236 (1,017 to 1,506)	66.4 (44.8 to 93.8)	62.2 (40.2 to 89.3)	69.8 (43.8 to 105.3)
Latin America and Caribbean	Caribbean	Bahamas	DALYs	Age-standardized	634.8 (539.7 to 750)	519.4 (426 to 639.3)	796.5 (683.1 to 929.2)	602.2 (491 to 746.1)	500.8 (393.5 to 629.5)	729.1 (598.7 to 901.6)	-5.1 (-17.1 to 8.5)	-3.6 (-16.5 to 11.1)	-8.5 (-22.6 to 9.9)
Latin America and Caribbean	Caribbean	Bahamas	YLLs	All Ages	615 (545 to 697)	244 (203 to 290)	371 (320 to 433)	1,179 (936 to 1,469)	449 (335 to 575)	730 (565 to 930)	91.6 (49.4 to 146)	84 (36.8 to 142.9)	96.7 (48.7 to 163.4)
Latin America and Caribbean	Caribbean	Bahamas	YLLs	Age-standardized	354.4 (312.6 to 401.2)	248.8 (205.8 to 294.9)	504 (434.4 to 589.2)	307.9 (244.9 to 383.3)	214.2 (160.5 to 274.3)	426.1 (332.6 to 536.1)	-13.1 (-32.5 to 10.4)	-13.9 (-36 to 13.9)	-15.5 (-35.8 to 11.8)
Latin America and Caribbean	Caribbean	Bahamas	YLDs	All Ages	714 (480 to 1,025)	357 (240 to 515)	357 (237 to 519)	1,032 (718 to 1,439)	526 (365 to 725)	506 (348 to 710)	44.6 (33.8 to 56.9)	47.4 (34.2 to 64.3)	41.8 (29.4 to 55.8)
Latin America and Caribbean	Caribbean	Bahamas	YLDs	Age-standardized	280.4 (192.4 to 395.2)	270.6 (184.9 to 383.4)	292.4 (200.2 to 412.6)	294.3 (201.8 to 423.7)	286.5 (195.7 to 409)	303 (205.6 to 436.1)	5 (-1.4 to 11.7)	5.9 (-3.2 to 15.2)	3.6 (-4.5 to 12)
Latin America and Caribbean	Caribbean	Barbados	Incidence	All Ages	2,822 (2,386 to 3,400)	1,478 (1,254 to 1,746)	1,344 (1,121 to 1,644)	3,380 (2,913 to 3,939)	1,825 (1,569 to 2,105)	1,555 (1,326 to 1,835)	19.8 (10.4 to 30.3)	23.5 (13.3 to 35.4)	15.7 (4.4 to 28.2)
Latin America and Caribbean	Caribbean	Barbados	Incidence	Age-standardized	1168.6 (980.1 to 1419.4)	1165.1 (977.2 to 1402)	1174.6 (981 to 1441.8)	1296.8 (1056.8 to 1589.5)	1315.5 (1079.5 to 1604.2)	1273.9 (1017.4 to 1598.6)	11 (2.1 to 22.6)	12.9 (2.5 to 25.7)	8.5 (-2 to 20.8)
Latin America and Caribbean	Caribbean	Barbados	Prevalence	All Ages	18,690 (16,253 to 21,744)	9,375 (8,064 to 10,959)	9,316 (8,058 to 11,083)	21,636 (18,873 to 24,807)	11,272 (9,755 to 13,000)	10,364 (9,030 to 12,035)	15.8 (7.6 to 25)	20.2 (10.3 to 30.6)	11.3 (2.1 to 22)
Latin America and Caribbean	Caribbean	Barbados	Prevalence	Age-standardized	7375.4 (6376.8 to 8668.5)	7083.7 (6061.3 to 8311.2)	7754.7 (6715.7 to 9174.4)	7643.9 (6368.1 to 9144.7)	7620.6 (6327.5 to 9180.2)	7664.4 (6399.4 to 9287.8)	3.6 (-5.5 to 14.1)	7.6 (-2.6 to 19.2)	-1.2 (-11 to 9.9)
Latin America and Caribbean	Caribbean	Barbados	Deaths	All Ages	40 (35 to 45)	16 (13 to 20)	24 (20 to 27)	63 (50 to 76)	25 (19 to 32)	37 (30 to 46)	57.8 (27.2 to 91.7)	59.3 (19.4 to 108.5)	56.8 (19.6 to 98.4)
Latin America and Caribbean	Caribbean	Barbados	Deaths	Age-standardized	12.9 (11.4 to 14.6)	8.8 (7.3 to 10.7)	19.5 (16.7 to 22.4)	13 (10.4 to 15.6)	9.3 (7.1 to 11.7)	17.9 (14.2 to 21.8)	0.4 (-18.4 to 21.6)	5.6 (-20.5 to 36.1)	-8.5 (-30.1 to 14.6)
Latin America and Caribbean	Caribbean	Barbados	DALYs	All Ages	1,536 (1,262 to 1,850)	698 (554 to 863)	839 (695 to 1,016)	2,059 (1,703 to 2,482)	947 (756 to 1,162)	1,112 (915 to 1,325)	34 (18.5 to 52.7)	35.7 (18 to 57.6)	32.6 (13.4 to 57.3)
Latin America and Caribbean	Caribbean	Barbados	DALYs	Age-standardized	570.3 (461.1 to 699.1)	488.5 (382.7 to 613)	687.3 (569 to 838.3)	576.7 (457.1 to 731.6)	516.4 (394.2 to 668.3)	652 (523.5 to 811)	1.1 (-9.7 to 11.7)	5.7 (-5.8 to 18)	-5.1 (-17.5 to 8.9)
Latin America and Caribbean	Caribbean	Barbados	YLLs	All Ages	739 (662 to 827)	295 (247 to 354)	444 (387 to 507)	1,116 (896 to 1,348)	450 (341 to 563)	665 (524 to 822)	51 (21.6 to 85.9)	52.7 (14.3 to 96.3)	49.9 (15.4 to 92.1)
Latin America and Caribbean	Caribbean	Barbados	YLLs	Age-standardized	255.4 (228.6 to 286.9)	184.4 (155.6 to 220.4)	358.3 (311.4 to 410)	243.7 (195.8 to 294.4)	182.4 (138.7 to 227.8)	320.5 (253.6 to 393.9)	-4.6 (-22.9 to 17.3)	-1.1 (-25.1 to 27.6)	-10.5 (-31.1 to 13.7)
Latin America and Caribbean	Caribbean	Barbados	YLDs	All Ages	798 (540 to 1,125)	403 (272 to 567)	395 (267 to 559)	943 (657 to 1,297)	496 (346 to 678)	447 (310 to 612)	18.2 (9 to 29.1)	23.3 (11.9 to 35.5)	13.1 (1.9 to 25.8)
Latin America and Caribbean	Caribbean	Barbados	YLDs	Age-standardized	314.9 (213.6 to 448.8)	304.2 (205.7 to 433.5)	329.1 (221 to 468.4)	333 (222.8 to 476.3)	334 (224.6 to 474.2)	331.4 (224.2 to 473)	5.7 (-3.6 to 17.6)	9.8 (-0.3 to 22.3)	0.7 (-9.7 to 12.7)
Latin America and Caribbean	Caribbean	Belize	Incidence	All Ages	2,798 (2,218 to 3,492)	1,329 (1,054 to 1,658)	1,469 (1,154 to 1,853)	5,272 (4,357 to 6,331)	2,502 (2,047 to 3,031)	2,770 (2,276 to 3,349)	88.4 (72.1 to 107.9)	88.3 (69.5 to 107.4)	88.6 (69.1 to 109.6)
Latin America and Caribbean	Caribbean	Belize	Incidence	Age-standardized	1294.8 (1079.8 to 1557)	1236.2 (1030.9 to 1480.3)	1352.4 (1126.7 to 1629.5)	1364.5 (1142.2 to 1618.6)	1264.4 (1047.7 to 1514.4)	1458.8 (1210.7 to 1747)	5.4 (-1.4 to 12.7)	2.3 (-5.6 to 10.2)	7.9 (-1.1 to 17.2)
Latin America and Caribbean	Caribbean	Belize	Prevalence	All Ages	14,569 (12,097 to 17,932)	6,980 (5,736 to 8,539)	7,589 (6,267 to 9,349)	29,175 (24,539 to 34,675)	14,148 (11,814 to 16,822)	15,027 (12,567 to 18,254)	100.3 (84.7 to 117.6)	102.7 (85.6 to 122.3)	98 (80 to 118.9)
Latin America and Caribbean	Caribbean	Belize	Prevalence	Age-standardized	7224.4 (6287.5 to 8429.1)	7164.8 (6201.9 to 8317.2)	7305.8 (6361.4 to 8570.5)	7283.5 (6242.7 to 8610.3)	6934.9 (5894 to 8174.4)	7602.1 (6464.1 to 9059.4)	0.8 (-5.9 to 8.2)	-3.2 (-10.3 to 4.7)	4.1 (-4.3 to 13.4)
Latin America and Caribbean	Caribbean	Belize	Deaths	All Ages	22 (19 to 25)	8 (7 to 10)	14 (12 to 16)	74 (62 to 87)	21 (17 to 26)	52 (44 to 62)	234.2 (175.2 to 303.6)	156.2 (96.1 to 231.2)	281.2 (203.8 to 376)
Latin America and Caribbean	Caribbean	Belize	Deaths	Age-standardized	22.4 (19.5 to 25.4)	16.1 (13.2 to 20.8)	29.4 (25.3 to 34.3)	28.8 (24.1 to 34)	16.2 (12.8 to 19.8)	41.5 (34.3 to 49.2)	28.6 (5.5 to 56.9)	0.4 (-24.7 to 31.2)	40.9 (11.6 to 76.2)
Latin America and Caribbean	Caribbean	Belize	DALYs	All Ages	1,247 (1,022 to 1,530)	543 (433 to 680)	704 (575 to 865)	3,128 (2,620 to 3,733)	1,194 (958 to 1,478)	1,935 (1,629 to 2,297)	150.9 (122.9 to 187.4)	119.9 (94.4 to 153.2)	174.7 (136.1 to 222.2)
Latin America and Caribbean	Caribbean	Belize	DALYs	Age-standardized	804.3 (689.8 to 931.2)	684.2 (567.1 to 819.7)	929.8 (793.8 to 1080)	935.9 (793.6 to 1092.2)	674.1 (559.8 to 807.8)	1190.6 (1010.6 to 1394.2)	16.4 (2.7 to 32.7)	-1.5 (-14.8 to 13.9)	28 (9.2 to 50)
Latin America and Caribbean	Caribbean	Belize	YLLs	All Ages	613 (535 to 685)	238 (194 to 290)	375 (325 to 425)	1,813 (1,531 to 2,138)	551 (440 to 680)	1,263 (1,051 to 1,513)	195.8 (144 to 259.8)	131.5 (80 to 200.9)	236.5 (167.4 to 322.5)
Latin America and Caribbean	Caribbean	Belize	YLLs	Age-standardized	485.9 (430.6 to 540.8)	366.5 (305.1 to 449.7)	609.6 (527.3 to 697.3)	605.9 (508.8 to 715.9)	356.9 (285.3 to 437.8)	849 (705.9 to 1010.1)	24.7 (2.3 to 50.9)	-2.6 (-25.8 to 25.8)	39.3 (10.9 to 73.8)
Latin America and Caribbean	Caribbean	Belize	YLDs	All Ages	634 (425 to 911)	305 (202 to 435)	329 (216 to 477)	1,315 (902 to 1,837)	643 (443 to 899)	672 (461 to 944)	107.4 (89 to 129.1)	110.9 (90.7 to 135.4)	104.3 (82.7 to 129.9)
Latin America and Caribbean	Caribbean	Belize	YLDs	Age-standardized	318.4 (221.2 to 433.4)	317.8 (219.1 to 435.1)	320.2 (222.7 to 440.8)	330.1 (232.8 to 456.9)	317.2 (222.5 to 432.5)	341.6 (240.4 to 471)	3.6 (-3.9 to 11.5)	-0.2 (-7.7 to 8.7)	6.7 (-2.4 to 17.3)
Latin America and Caribbean	Caribbean	Bermuda	Incidence	All Ages	650 (550 to 770)	323 (274 to 384)	327 (273 to 390)	852 (728 to 984)	414 (353 to 474)	438 (375 to 509)	31.1 (18.7 to 44.2)	28 (15.6 to 41.3)	34.1 (20.1 to 50.1)
Latin America and Caribbean	Caribbean	Bermuda	Incidence	Age-standardized	1244.5 (1032.8 to 1510.5)	1186.8 (975.9 to 1452.6)	1316.1 (1073.9 to 1601.5)	1371.9 (1117.2 to 1664.3)	1308.6 (1068.6 to 1588.6)	1445.7 (1173.4 to 1753)	10.2 (5.2 to 15.8)	10.3 (4.3 to 17.8)	9.9 (3.4 to 16.8)
Latin America and Caribbean	Caribbean	Bermuda	Prevalence	All Ages	3,618 (3,101 to 4,243)	1,823 (1,557 to 2,151)	1,795 (1,526 to 2,121)	4,140 (3,662 to 4,716)	2,027 (1,756 to 2,353)	2,114 (1,883 to 2,404)	14.4 (7.1 to 23.1)	11.1 (2.1 to 22.3)	17.7 (8.9 to 27.5)
Latin America and Caribbean	Caribbean	Bermuda	Prevalence	Age-standardized	6545.4 (5477.3 to 7844.7)	6261 (5230.5 to 7581.7)	6932 (5775.9 to 8330.8)	6956.7 (5711.2 to 8430.7)	6781.5 (5559.2 to 8311.8)	7218.1 (5928.7 to 8915.3)	6.3 (0.2 to 12.6)	8.3 (-0.1 to 17.5)	4.1 (-3.4 to 12.2)
Latin America and Caribbean	Caribbean	Bermuda	Deaths	All Ages	11 (10 to 12)	4 (3 to 5)	7 (6 to 8)	18 (15 to 22)	6 (5 to 8)	12 (10 to 15)	65.7 (36.8 to 104.9)	53.1 (18.3 to 101.3)	73.1 (37.5 to 120.8)
Latin America and Caribbean	Caribbean	Bermuda	Deaths	Age-standardized	19 (16.9 to 21.5)	11.9 (9.7 to 14.5)	30.7 (26.8 to 34.8)	13.2 (10.9 to 16)	7.4 (5.5 to 9.5)	21.8 (17.5 to 26.6)	-30.6 (-43 to -14.5)	-37.9 (-51.5 to -18.1)	-28.9 (-43.2 to -9.9)
Latin America and Caribbean	Caribbean	Bermuda	DALYs	All Ages	381 (326 to 445)	159 (129 to 193)	222 (192 to 257)	473 (399 to 561)	180 (147 to 221)	293 (244 to 345)	24.2 (9.2 to 44.6)	13.2 (-1.2 to 31.8)	32.1 (11.5 to 59.6)
Latin America and Caribbean	Caribbean	Bermuda	DALYs	Age-standardized	650.5 (550.4 to 772.7)	505.9 (405.5 to 622.6)	854 (734.8 to 997.8)	544.7 (435.4 to 681.6)	434.7 (328.7 to 568.4)	688.6 (562.7 to 843.2)	-16.3 (-25.4 to -6.3)	-14.1 (-24.2 to -3.3)	-19.4 (-29.6 to -5.5)
Latin America and Caribbean	Caribbean	Bermuda	YLLs	All Ages	221 (200 to 248)	78 (66 to 94)	143 (127 to 163)	285 (234 to 348)	87 (66 to 113)	197 (159 to 246)	28.5 (4.5 to 61.4)	11.4 (-13.1 to 46.4)	37.8 (7.8 to 79.7)
Latin America and Caribbean	Caribbean	Bermuda	YLLs	Age-standardized	363.7 (328 to 407.4)	230.6 (194.6 to 275.8)	551.4 (486.9 to 622.2)	234.3 (192.4 to 287.6)	131.8 (102.2 to 168.1)	367 (296.9 to 452.5)	-35.6 (-47.6 to -19.9)	-42.9 (-55.2 to -25.7)	-33.4 (-47.8 to -13.7)
Latin America and Caribbean	Caribbean	Bermuda	YLDs	All Ages	159 (110 to 221)	81 (56 to 113)	79 (54 to 109)	188 (136 to 245)	93 (66 to 124)	96 (69 to 125)	18.4 (7.9 to 31.4)	15 (4 to 28.1)	21.8 (8.3 to 38)
Latin America and Caribbean	Caribbean	Bermuda	YLDs	Age-standardized	286.9 (196.2 to 406.2)	275.4 (187.6 to 390.9)	302.7 (206.8 to 427)	310.4 (211.7 to 439.7)	303 (201.8 to 429.6)	321.6 (220.4 to 457.2)	8.2 (1.7 to 15.2)	10 (1.2 to 19.3)	6.3 (-2 to 15.3)
Latin America and Caribbean	Caribbean	Cuba	Incidence	All Ages	129,478 (107,461 to 157,437)	68,577 (57,105 to 83,917)	60,901 (48,333 to 75,680)	132,095 (114,627 to 152,802)	68,280 (59,488 to 78,379)	63,815 (54,641 to 75,284)	2 (-5.9 to 10.5)	-0.4 (-10.5 to 10.2)	4.8 (-4.5 to 15.5)
Latin America and Caribbean	Caribbean	Cuba	Incidence	Age-standardized	1275.7 (1045.1 to 1570.4)	1327.9 (1113.2 to 1615.9)	1223.4 (959.3 to 1542.3)	1346.7 (1092.6 to 1653.6)	1364.5 (1117.9 to 1655)	1332 (1068.4 to 1665.5)	5.6 (-0.5 to 11.6)	2.8 (-6.1 to 12.3)	8.9 (1.8 to 15.9)
Latin America and Caribbean	Caribbean	Cuba	Prevalence	All Ages	959,573 (821,440 to 1,120,226)	532,040 (459,101 to 625,818)	427,532 (360,180 to 506,988)	1,081,427 (974,990 to 1,209,397)	584,497 (522,704 to 654,046)	496,929 (443,433 to 564,898)	12.7 (4.4 to 22.6)	9.9 (-0.3 to 21.4)	16.2 (6.5 to 28.3)
Latin America and Caribbean	Caribbean	Cuba	Prevalence	Age-standardized	9042 (7776.7 to 10676.4)	9873 (8526.7 to 11525.3)	8195.1 (6889.8 to 9800)	9203.6 (7831.7 to 10865.2)	9579.9 (8171.2 to 11262.3)	8839.2 (7421.6 to 10662.7)	1.8 (-4.5 to 8)	-3 (-10.9 to 5.3)	7.9 (0.3 to 15.8)
Latin America and Caribbean	Caribbean	Cuba	Deaths	All Ages	1,866 (1,712 to 1,989)	815 (688 to 907)	1,052 (977 to 1,124)	4,586 (3,549 to 5,603)	2,033 (1,211 to 2,588)	2,554 (2,031 to 3,138)	145.8 (93.9 to 200)	149.5 (64.7 to 222.9)	142.8 (91.4 to 199.2)
Latin America and Caribbean	Caribbean	Cuba	Deaths	Age-standardized	18.7 (17.1 to 20)	15.9 (13.6 to 17.8)	21.6 (20 to 23.2)	23.3 (17.9 to 28.4)	19.2 (11.4 to 24.5)	28.4 (22.6 to 34.8)	24.8 (-2.4 to 51.7)	20.7 (-22.7 to 56.6)	31.1 (3.8 to 61.7)
Latin America and Caribbean	Caribbean	Cuba	DALYs	All Ages	81,812 (67,823 to 98,774)	42,509 (34,641 to 51,624)	39,303 (33,319 to 47,299)	130,377 (105,524 to 154,175)	64,508 (45,977 to 77,949)	65,868 (54,896 to 79,757)	59.4 (33.6 to 89)	51.8 (19.9 to 84.8)	67.6 (40.2 to 100.8)
Latin America and Caribbean	Caribbean	Cuba	DALYs	Age-standardized	777.6 (645.2 to 940.9)	796 (649.1 to 966.2)	761 (644.5 to 919.7)	852.6 (683.8 to 1038.3)	823.7 (614.2 to 1018.1)	892.9 (726.4 to 1096.4)	9.6 (-3.3 to 24.8)	3.5 (-13.4 to 19.2)	17.3 (3 to 34.6)
Latin America and Caribbean	Caribbean	Cuba	YLLs	All Ages	39,766 (36,041 to 42,147)	18,963 (15,452 to 20,813)	20,803 (19,367 to 22,249)	80,852 (60,800 to 100,585)	37,011 (20,934 to 47,633)	43,841 (34,476 to 54,951)	103.3 (58.4 to 152.7)	95.2 (27.2 to 154.5)	110.7 (64.2 to 165.8)
Latin America and Caribbean	Caribbean	Cuba	YLLs	Age-standardized	381 (346.7 to 404.4)	358.5 (295.1 to 392.9)	406 (378 to 434.5)	437.4 (332 to 541.5)	383.5 (227.1 to 492.7)	502.6 (395.2 to 628.7)	14.8 (-10.3 to 42.4)	7 (-27 to 40.3)	23.8 (-4 to 55.3)
Latin America and Caribbean	Caribbean	Cuba	YLDs	All Ages	42,046 (29,012 to 58,110)	23,546 (16,249 to 32,393)	18,500 (12,530 to 26,242)	49,524 (36,761 to 63,520)	27,497 (20,248 to 35,523)	22,027 (15,769 to 28,877)	17.8 (4.7 to 34.9)	16.8 (1.4 to 35.8)	19.1 (4.4 to 37.7)
Latin America and Caribbean	Caribbean	Cuba	YLDs	Age-standardized	396.6 (274.3 to 555.2)	437.5 (302.4 to 601.6)	355 (242.4 to 509.9)	415.3 (292.4 to 572.3)	440.2 (309.9 to 597.2)	390.3 (268.3 to 547.8)	4.7 (-2.3 to 12.6)	0.6 (-8.4 to 10.6)	9.9 (1.6 to 19.1)
Latin America and Caribbean	Caribbean	Dominica	Incidence	All Ages	936 (762 to 1,158)	455 (374 to 552)	481 (385 to 611)	797 (677 to 935)	376 (318 to 441)	421 (353 to 500)	-14.9 (-21.3 to -8.4)	-17.5 (-24.3 to -10)	-12.4 (-21.4 to -3)
Latin America and Caribbean	Caribbean	Dominica	Incidence	Age-standardized	1176.8 (973.4 to 1438.9)	1142.9 (946.1 to 1376.4)	1222.9 (1007.6 to 1526.2)	1281.7 (1054.9 to 1548.4)	1210.6 (1000.5 to 1464)	1354.6 (1103.6 to 1655.2)	8.9 (2.7 to 14.6)	5.9 (-1.2 to 13.4)	10.8 (2.3 to 19.8)
Latin America and Caribbean	Caribbean	Dominica	Prevalence	All Ages	5,703 (4,879 to 6,746)	2,670 (2,265 to 3,181)	3,032 (2,568 to 3,651)	5,309 (4,751 to 6,001)	2,316 (2,040 to 2,664)	2,993 (2,679 to 3,394)	-6.9 (-12.7 to -0.6)	-13.3 (-20.1 to -6.5)	-1.3 (-9.1 to 7.3)
Latin America and Caribbean	Caribbean	Dominica	Prevalence	Age-standardized	7499.1 (6522.7 to 8745.8)	7036.2 (6046.1 to 8262.2)	8101.4 (7072.7 to 9457.1)	7917.7 (6922.9 to 9189)	7013.5 (6025.5 to 8203.7)	8820.1 (7697.3 to 10233.9)	5.6 (1 to 10.5)	-0.3 (-6.6 to 6.9)	8.9 (2.5 to 16.2)
Latin America and Caribbean	Caribbean	Dominica	Deaths	All Ages	17 (15 to 21)	6 (5 to 9)	11 (10 to 14)	22 (17 to 27)	7 (4 to 9)	15 (12 to 19)	26.3 (-11.9 to 59.6)	7.4 (-43.4 to 43.1)	37.3 (-6.7 to 78.3)
Latin America and Caribbean	Caribbean	Dominica	Deaths	Age-standardized	23.3 (20.7 to 27.8)	14.3 (12.1 to 19)	38 (33.3 to 47.8)	24.3 (19 to 29.7)	13.9 (8.7 to 17.6)	37.5 (29.5 to 46.4)	4.3 (-27.2 to 30.8)	-2.4 (-48.1 to 29.9)	-1.2 (-33.1 to 27.9)
Latin America and Caribbean	Caribbean	Dominica	DALYs	All Ages	589 (496 to 708)	240 (198 to 298)	349 (295 to 424)	646 (524 to 769)	227 (175 to 277)	419 (336 to 504)	9.7 (-11.8 to 29.7)	-5.4 (-30.7 to 12.9)	20 (-8.4 to 46.3)
Latin America and Caribbean	Caribbean	Dominica	DALYs	Age-standardized	797.1 (673.3 to 948.7)	610.4 (499.8 to 756.5)	1065.1 (915 to 1281.9)	831.4 (674.4 to 1007.7)	598.5 (466.3 to 743.5)	1083.6 (864.7 to 1313.3)	4.3 (-15.6 to 21.5)	-2 (-26 to 14.7)	1.7 (-22.7 to 21.6)
Latin America and Caribbean	Caribbean	Dominica	YLLs	All Ages	344 (304 to 409)	125 (105 to 164)	220 (190 to 273)	413 (321 to 512)	124 (78 to 159)	289 (223 to 363)	20 (-17.7 to 53.2)	-0.5 (-46 to 33.5)	31.6 (-11.8 to 72.7)
Latin America and Caribbean	Caribbean	Dominica	YLLs	Age-standardized	475.5 (418.7 to 567.5)	307 (260.5 to 403.8)	719.7 (624.2 to 897.3)	485.3 (372.9 to 604.5)	288.5 (179.7 to 371.1)	701.4 (540.6 to 879.4)	2.1 (-30.5 to 30.9)	-6 (-49.1 to 27.4)	-2.5 (-35.4 to 27.3)
Latin America and Caribbean	Caribbean	Dominica	YLDs	All Ages	244 (164 to 341)	115 (78 to 162)	129 (87 to 183)	232 (165 to 314)	103 (72 to 139)	130 (91 to 176)	-4.9 (-12.4 to 3.5)	-10.7 (-19.1 to -2.1)	0.3 (-9 to 11.3)
Latin America and Caribbean	Caribbean	Dominica	YLDs	Age-standardized	321.6 (218.3 to 447)	303.5 (207.2 to 427.5)	345.4 (234.8 to 483.8)	346.1 (239.5 to 481.6)	310 (212.6 to 433.2)	382.2 (261.7 to 530.5)	7.6 (2.1 to 13.3)	2.2 (-5.4 to 10.1)	10.7 (3.5 to 18.7)
Latin America and Caribbean	Caribbean	Dominican Republic	Incidence	All Ages	72,593 (57,842 to 89,199)	39,070 (31,247 to 47,931)	33,522 (26,239 to 41,646)	96,450 (79,380 to 118,642)	51,213 (42,740 to 61,668)	45,237 (36,285 to 57,284)	32.9 (21.5 to 45.5)	31.1 (18.5 to 45.8)	34.9 (19.7 to 50.7)
Latin America and Caribbean	Caribbean	Dominican Republic	Incidence	Age-standardized	873.3 (718.8 to 1042.6)	941.3 (775.7 to 1125.4)	800.5 (651.7 to 970.4)	905.2 (744.6 to 1113.2)	961.9 (800.4 to 1161.1)	848.7 (683.5 to 1069.7)	3.7 (-4.3 to 12.5)	2.2 (-7 to 11.7)	6 (-4.3 to 17.4)
Latin America and Caribbean	Caribbean	Dominican Republic	Prevalence	All Ages	364,518 (305,939 to 435,843)	201,599 (167,288 to 239,937)	162,918 (135,483 to 196,163)	536,784 (457,799 to 636,207)	290,706 (249,966 to 343,267)	246,079 (205,700 to 298,482)	47.3 (34.1 to 61.6)	44.2 (31.1 to 60.6)	51 (33.8 to 69.6)
Latin America and Caribbean	Caribbean	Dominican Republic	Prevalence	Age-standardized	4758 (4121.5 to 5507.8)	5249.8 (4535.1 to 6066.8)	4232.7 (3655.2 to 4929.9)	5054.1 (4334.6 to 5983.9)	5445.2 (4696.6 to 6415.7)	4674.1 (3945.8 to 5624.3)	6.2 (-2.2 to 15.5)	3.7 (-4.7 to 13.5)	10.4 (-0.9 to 22.9)
Latin America and Caribbean	Caribbean	Dominican Republic	Deaths	All Ages	915 (774 to 1,029)	420 (328 to 489)	495 (424 to 572)	1,937 (1,436 to 2,516)	853 (553 to 1,181)	1,083 (801 to 1,503)	111.6 (60.8 to 178.8)	103.1 (38.4 to 180.2)	118.9 (60.9 to 204.6)
Latin America and Caribbean	Caribbean	Dominican Republic	Deaths	Age-standardized	24.3 (20.6 to 27.4)	22.1 (17.1 to 25.6)	26.6 (23 to 30.7)	22.2 (16.5 to 28.7)	18.4 (11.9 to 25.2)	26.4 (19.8 to 36.1)	-8.8 (-30.6 to 20.2)	-16.5 (-44.1 to 13.4)	-0.7 (-26.2 to 36)
Latin America and Caribbean	Caribbean	Dominican Republic	DALYs	All Ages	49,587 (40,660 to 58,989)	24,369 (18,854 to 30,068)	25,217 (20,124 to 30,782)	67,306 (52,759 to 83,322)	31,613 (23,623 to 40,508)	35,693 (27,078 to 46,945)	35.7 (8.8 to 67.4)	29.7 (1.5 to 63.4)	41.5 (7.9 to 83.7)
Latin America and Caribbean	Caribbean	Dominican Republic	DALYs	Age-standardized	796 (681.2 to 912.4)	767 (636.5 to 902.8)	823.5 (703.4 to 960)	679.2 (532.6 to 834.9)	619.9 (463.9 to 794.7)	745.5 (570.3 to 981.4)	-14.7 (-30.6 to 4.1)	-19.2 (-36.9 to 1.1)	-9.5 (-29.6 to 17.2)
Latin America and Caribbean	Caribbean	Dominican Republic	YLLs	All Ages	33,661 (26,642 to 40,667)	15,468 (10,917 to 19,916)	18,193 (14,381 to 23,010)	43,061 (31,278 to 56,877)	18,288 (11,798 to 26,111)	24,773 (17,920 to 34,968)	27.9 (-8.1 to 74.5)	18.2 (-23.7 to 70.4)	36.2 (-6 to 94.7)
Latin America and Caribbean	Caribbean	Dominican Republic	YLLs	Age-standardized	586.5 (492.7 to 665.8)	532.9 (410.4 to 633.3)	640 (543.2 to 747.6)	451.1 (331.8 to 593.5)	370.4 (240.5 to 524.3)	538.4 (393.5 to 757.6)	-23.1 (-43.6 to 2.9)	-30.5 (-54.5 to -1)	-15.9 (-40.2 to 18.8)
Latin America and Caribbean	Caribbean	Dominican Republic	YLDs	All Ages	15,925 (10,701 to 22,765)	8,901 (6,076 to 12,770)	7,025 (4,630 to 10,089)	24,244 (17,189 to 33,079)	13,325 (9,459 to 18,117)	10,919 (7,505 to 15,414)	52.2 (37.3 to 70)	49.7 (33.8 to 69)	55.4 (35.6 to 78.3)
Latin America and Caribbean	Caribbean	Dominican Republic	YLDs	Age-standardized	209.6 (146.9 to 290.2)	234 (165.6 to 325.7)	183.5 (127.3 to 252.6)	228.1 (161.9 to 310)	249.6 (178 to 339.5)	207.1 (144 to 290.1)	8.8 (0.9 to 17.8)	6.6 (-2.7 to 17.4)	12.8 (0.9 to 26.8)
Latin America and Caribbean	Caribbean	Grenada	Incidence	All Ages	1,216 (960 to 1,528)	617 (492 to 762)	599 (466 to 779)	1,242 (1,045 to 1,493)	653 (555 to 775)	589 (479 to 727)	2.1 (-6 to 12.9)	5.8 (-3.2 to 20)	-1.7 (-10.7 to 9)
Latin America and Caribbean	Caribbean	Grenada	Incidence	Age-standardized	1252 (1017.4 to 1536.2)	1271.1 (1039.7 to 1543.9)	1236.8 (998.6 to 1546.6)	1352 (1110.7 to 1666.1)	1400.2 (1170.3 to 1705.4)	1307.2 (1059.1 to 1658.4)	8 (1.8 to 14)	10.2 (2.4 to 18.3)	5.7 (-2.8 to 13.7)
Latin America and Caribbean	Caribbean	Grenada	Prevalence	All Ages	7,250 (5,985 to 8,833)	3,735 (3,083 to 4,460)	3,514 (2,827 to 4,408)	8,142 (6,929 to 9,677)	4,288 (3,626 to 5,060)	3,855 (3,210 to 4,629)	12.3 (3.2 to 22.2)	14.8 (4.7 to 26.7)	9.7 (-1 to 21)
Latin America and Caribbean	Caribbean	Grenada	Prevalence	Age-standardized	8085.2 (6830.9 to 9596.7)	8378.4 (7046.2 to 9854.4)	7827.4 (6528.2 to 9456.5)	8272.7 (6945.4 to 10011)	8650.7 (7246.8 to 10305.3)	7925.5 (6534.7 to 9656)	2.3 (-3.7 to 8.4)	3.2 (-4.8 to 11.3)	1.3 (-6.8 to 10.3)
Latin America and Caribbean	Caribbean	Grenada	Deaths	All Ages	18 (16 to 20)	7 (6 to 8)	11 (9 to 13)	19 (16 to 22)	8 (6 to 10)	11 (9 to 13)	7.3 (-9.7 to 26.6)	17.1 (-14.2 to 50.4)	1.3 (-18.1 to 25.1)
Latin America and Caribbean	Caribbean	Grenada	Deaths	Age-standardized	22.3 (19.6 to 25.4)	14.3 (11.9 to 17.6)	35 (30 to 40.3)	19.1 (16.4 to 21.7)	14.1 (11.3 to 17.1)	27.4 (23.1 to 32.1)	-14.5 (-27.8 to 1)	-1.2 (-25.9 to 25)	-21.7 (-36.6 to -3.6)
Latin America and Caribbean	Caribbean	Grenada	DALYs	All Ages	671 (555 to 810)	301 (241 to 374)	369 (309 to 443)	782 (654 to 941)	358 (286 to 441)	424 (358 to 510)	16.6 (4.2 to 28.6)	18.8 (4.1 to 34.8)	14.9 (-0.8 to 32.2)
Latin America and Caribbean	Caribbean	Grenada	DALYs	Age-standardized	824.2 (693.2 to 972.6)	694.4 (560.6 to 858.2)	1015.2 (867.5 to 1186.7)	754.8 (622.7 to 917.9)	681.7 (540.5 to 852.1)	857.7 (723.1 to 1032.2)	-8.4 (-17.9 to 1.2)	-1.8 (-13.5 to 10.1)	-15.5 (-27 to -3.8)
Latin America and Caribbean	Caribbean	Grenada	YLLs	All Ages	357 (314 to 406)	139 (117 to 169)	218 (189 to 255)	422 (363 to 483)	166 (131 to 202)	255 (212 to 303)	17.9 (-1.6 to 41.4)	19.5 (-8.3 to 50.5)	17 (-7.1 to 44.7)
Latin America and Caribbean	Caribbean	Grenada	YLLs	Age-standardized	473.7 (416.1 to 538.8)	329.2 (274.5 to 399)	678.4 (583.4 to 795.6)	389.2 (335.5 to 443.6)	296.2 (234.9 to 357.1)	511.2 (432.1 to 596.7)	-17.8 (-31.6 to -1.8)	-10 (-30.8 to 13.1)	-24.6 (-39.4 to -7.3)
Latin America and Caribbean	Caribbean	Grenada	YLDs	All Ages	313 (208 to 449)	162 (108 to 231)	151 (99 to 222)	361 (250 to 504)	192 (131 to 267)	169 (115 to 239)	15.1 (5 to 27.5)	18.2 (6.7 to 31.8)	11.8 (-0.1 to 25.9)
Latin America and Caribbean	Caribbean	Grenada	YLDs	Age-standardized	350.5 (234.5 to 493.8)	365.3 (246.8 to 519.5)	336.8 (225 to 479.1)	365.5 (251.2 to 521.2)	385.5 (263.3 to 548.2)	346.5 (234 to 500.5)	4.3 (-2.1 to 11.4)	5.5 (-3.1 to 14.3)	2.9 (-5.6 to 13)
Latin America and Caribbean	Caribbean	Guyana	Incidence	All Ages	9,874 (7,896 to 12,219)	5,054 (4,077 to 6,168)	4,820 (3,790 to 6,254)	8,955 (7,362 to 10,876)	4,505 (3,743 to 5,472)	4,450 (3,593 to 5,479)	-9.3 (-15.5 to -2.7)	-10.9 (-18.5 to -1.3)	-7.7 (-15.9 to 1.7)
Latin America and Caribbean	Caribbean	Guyana	Incidence	Age-standardized	1170.2 (970 to 1407.7)	1181.5 (980.9 to 1408.2)	1157.7 (940.3 to 1449.1)	1202.4 (990.5 to 1460.6)	1192.3 (989 to 1445.1)	1210.7 (984.2 to 1485.4)	2.8 (-3.6 to 8.2)	0.9 (-7 to 9.5)	4.6 (-4 to 13.5)
Latin America and Caribbean	Caribbean	Guyana	Prevalence	All Ages	58,068 (49,067 to 69,462)	29,751 (25,409 to 35,192)	28,317 (23,545 to 34,515)	55,223 (47,231 to 64,772)	26,820 (23,122 to 31,427)	28,404 (24,080 to 33,471)	-4.9 (-10.2 to 0.9)	-9.9 (-16.2 to -2.4)	0.3 (-7 to 8.1)
Latin America and Caribbean	Caribbean	Guyana	Prevalence	Age-standardized	7466.5 (6569.1 to 8638.5)	7533.7 (6638.4 to 8652.3)	7409.7 (6465.6 to 8671.2)	7276.8 (6267.4 to 8488.7)	6932.5 (6021.8 to 8132.3)	7639.2 (6522.9 to 8962.3)	-2.5 (-7.7 to 2.8)	-8 (-14.3 to -1.5)	3.1 (-3.9 to 10.2)
Latin America and Caribbean	Caribbean	Guyana	Deaths	All Ages	90 (77 to 104)	31 (25 to 38)	59 (49 to 71)	119 (92 to 148)	42 (31 to 56)	76 (58 to 96)	31.4 (0.1 to 71.6)	35.2 (-1.4 to 85.9)	29.4 (-5.7 to 72.7)
Latin America and Caribbean	Caribbean	Guyana	Deaths	Age-standardized	25.6 (22 to 29.5)	16.2 (13.3 to 20.2)	36.6 (30.6 to 43.5)	21.6 (16.9 to 26.6)	14 (10.3 to 18.4)	31.2 (24 to 39)	-15.7 (-35.7 to 8.7)	-13.8 (-36.9 to 17.6)	-14.6 (-36.7 to 12.8)
Latin America and Caribbean	Caribbean	Guyana	DALYs	All Ages	5,008 (4,090 to 6,144)	2,207 (1,742 to 2,775)	2,801 (2,291 to 3,379)	5,538 (4,391 to 6,820)	2,318 (1,822 to 2,883)	3,220 (2,551 to 3,964)	10.6 (-6.4 to 32)	5 (-11.2 to 26.7)	15 (-5.6 to 40.4)
Latin America and Caribbean	Caribbean	Guyana	DALYs	Age-standardized	872.8 (739.9 to 1023.3)	697.1 (572.2 to 847.7)	1069.2 (899.4 to 1254.4)	795.9 (635.7 to 974.8)	631.5 (499.4 to 782.8)	987.5 (795.1 to 1197.3)	-8.8 (-23.7 to 8.8)	-9.4 (-24.1 to 8.3)	-7.6 (-25.6 to 14.1)
Latin America and Caribbean	Caribbean	Guyana	YLLs	All Ages	2,544 (2,146 to 2,980)	937 (756 to 1,144)	1,607 (1,325 to 1,925)	3,124 (2,390 to 3,972)	1,130 (823 to 1,526)	1,994 (1,492 to 2,546)	22.8 (-8.9 to 63)	20.6 (-15.8 to 64.7)	24.1 (-10.5 to 69.9)
Latin America and Caribbean	Caribbean	Guyana	YLLs	Age-standardized	556 (471.5 to 647.9)	375 (304.1 to 460.6)	757.5 (621.2 to 914.6)	477.8 (369.5 to 601.5)	324.2 (238.7 to 433.9)	658.2 (497.6 to 830)	-14.1 (-35.9 to 13.2)	-13.5 (-38.4 to 18.4)	-13.1 (-36.5 to 17.3)
Latin America and Caribbean	Caribbean	Guyana	YLDs	All Ages	2,464 (1,643 to 3,528)	1,270 (849 to 1,806)	1,194 (781 to 1,727)	2,415 (1,658 to 3,373)	1,189 (818 to 1,646)	1,226 (833 to 1,742)	-2 (-8.4 to 5.7)	-6.4 (-13.7 to 3.3)	2.7 (-6.2 to 12.4)
Latin America and Caribbean	Caribbean	Guyana	YLDs	Age-standardized	316.8 (215.9 to 442.1)	322.2 (220.8 to 447.1)	311.7 (211 to 434.2)	318.1 (219.9 to 443.9)	307.3 (213 to 426.3)	329.3 (227.6 to 463.1)	0.4 (-5.6 to 6.9)	-4.6 (-11.7 to 3.4)	5.6 (-1.9 to 13.7)
Latin America and Caribbean	Caribbean	Haiti	Incidence	All Ages	111,332 (88,291 to 136,344)	58,705 (47,216 to 70,984)	52,627 (40,653 to 65,430)	174,853 (143,434 to 212,386)	92,027 (75,747 to 110,734)	82,826 (66,782 to 101,725)	57.1 (46 to 68.7)	56.8 (42 to 71.3)	57.4 (43.5 to 72.6)
Latin America and Caribbean	Caribbean	Haiti	Incidence	Age-standardized	1418.8 (1177.5 to 1682)	1508.1 (1256.7 to 1784.3)	1318.6 (1071.7 to 1590.8)	1357.4 (1144 to 1605.7)	1419.9 (1198.6 to 1676.7)	1284.2 (1071.8 to 1529.4)	-4.3 (-10.1 to 1.5)	-5.8 (-13.3 to 1.9)	-2.6 (-9.8 to 4.9)
Latin America and Caribbean	Caribbean	Haiti	Prevalence	All Ages	637,290 (545,714 to 748,530)	355,229 (310,658 to 413,429)	282,061 (234,235 to 343,718)	1,044,343 (907,757 to 1,213,420)	579,987 (509,575 to 660,606)	464,356 (391,135 to 558,521)	63.9 (53.8 to 74.1)	63.3 (51.6 to 75.3)	64.6 (51.9 to 78.6)
Latin America and Caribbean	Caribbean	Haiti	Prevalence	Age-standardized	9209.4 (8198.5 to 10425.4)	10365.6 (9318 to 11619.3)	7929.4 (6894.2 to 9237.7)	8533.3 (7574.2 to 9668.5)	9301.2 (8355.5 to 10395)	7629.3 (6642.2 to 8895.3)	-7.3 (-11.5 to -3.1)	-10.3 (-15.5 to -5.4)	-3.8 (-9.8 to 2.4)
Latin America and Caribbean	Caribbean	Haiti	Deaths	All Ages	2,420 (1,642 to 3,185)	1,020 (645 to 1,586)	1,400 (788 to 1,857)	3,420 (2,266 to 4,792)	1,468 (871 to 2,564)	1,952 (1,093 to 2,741)	41.3 (8.8 to 85.7)	44 (2.8 to 100.1)	39.4 (4.6 to 88.6)
Latin America and Caribbean	Caribbean	Haiti	Deaths	Age-standardized	72.4 (44.9 to 105.2)	57.3 (32.5 to 107.6)	92 (51.9 to 119.8)	54.3 (35 to 77.5)	42.4 (24.4 to 79)	67.7 (37.9 to 95.1)	-25 (-43.4 to -1.7)	-26 (-49.7 to 4.2)	-26.4 (-43.3 to -3.1)
Latin America and Caribbean	Caribbean	Haiti	DALYs	All Ages	126,401 (86,503 to 162,759)	56,711 (40,135 to 75,708)	69,691 (38,714 to 97,089)	156,415 (113,032 to 200,312)	74,055 (53,231 to 99,618)	82,360 (52,731 to 109,891)	23.7 (-4.3 to 61.7)	30.6 (-3.3 to 78.1)	18.2 (-12.3 to 63)
Latin America and Caribbean	Caribbean	Haiti	DALYs	Age-standardized	2304.3 (1696.7 to 2922.2)	2015.4 (1422.5 to 2786.5)	2632.4 (1618.1 to 3415.7)	1633.9 (1183.4 to 2135.6)	1450.2 (1006.1 to 2147.5)	1829.4 (1159.6 to 2430.6)	-29.1 (-41.9 to -11.7)	-28 (-43.2 to -8.9)	-30.5 (-45.4 to -9.7)
Latin America and Caribbean	Caribbean	Haiti	YLLs	All Ages	98,770 (61,408 to 134,139)	41,317 (25,606 to 59,507)	57,453 (27,142 to 85,327)	110,550 (72,252 to 152,414)	48,505 (29,972 to 73,989)	62,045 (33,410 to 89,354)	11.9 (-20.7 to 61.4)	17.4 (-24.3 to 85.4)	8 (-26.8 to 62.8)
Latin America and Caribbean	Caribbean	Haiti	YLLs	Age-standardized	1904.3 (1296.1 to 2481.5)	1564.1 (1006.1 to 2313.6)	2289.2 (1291.8 to 3034.8)	1257.7 (823.9 to 1745.4)	1038.2 (628.8 to 1729.9)	1495.4 (834 to 2083.8)	-34 (-48.9 to -13.5)	-33.6 (-52.7 to -8.4)	-34.7 (-51.3 to -11)
Latin America and Caribbean	Caribbean	Haiti	YLDs	All Ages	27,631 (18,519 to 39,999)	15,393 (10,271 to 21,983)	12,238 (7,919 to 17,987)	45,865 (31,271 to 64,173)	25,549 (17,601 to 35,525)	20,316 (13,580 to 29,293)	66 (54.2 to 78.7)	66 (52 to 81)	66 (50.6 to 82.6)
Latin America and Caribbean	Caribbean	Haiti	YLDs	Age-standardized	400.1 (273.8 to 556.8)	451.3 (312.8 to 624.7)	343.2 (234.1 to 481.3)	376.1 (265.4 to 512.8)	412 (287.9 to 558.6)	334.1 (233.2 to 463.4)	-6 (-11.3 to -0.2)	-8.7 (-15.1 to -2.1)	-2.7 (-10.3 to 5.3)
Latin America and Caribbean	Caribbean	Jamaica	Incidence	All Ages	32,808 (26,216 to 41,084)	16,463 (13,118 to 20,306)	16,345 (12,856 to 20,902)	29,548 (24,760 to 35,480)	14,374 (11,958 to 17,419)	15,174 (12,657 to 18,441)	-9.9 (-17 to -1.9)	-12.7 (-20.8 to -3.1)	-7.2 (-16.1 to 3.7)
Latin America and Caribbean	Caribbean	Jamaica	Incidence	Age-standardized	1263.6 (1039 to 1551.2)	1252.1 (1018.7 to 1513.9)	1275 (1033.8 to 1591.7)	1190.6 (970.9 to 1462.1)	1133.4 (919.2 to 1410.5)	1246.7 (1012.8 to 1550.5)	-5.8 (-12.9 to 0.9)	-9.5 (-17.8 to -0.6)	-2.2 (-11.7 to 7.2)
Latin America and Caribbean	Caribbean	Jamaica	Prevalence	All Ages	196,449 (166,795 to 235,381)	96,995 (81,597 to 114,845)	99,454 (83,377 to 119,964)	195,364 (167,757 to 229,607)	88,856 (75,202 to 105,031)	106,508 (92,467 to 124,519)	-0.6 (-7.8 to 7.1)	-8.4 (-17.7 to 0.4)	7.1 (-1.4 to 17.9)
Latin America and Caribbean	Caribbean	Jamaica	Prevalence	Age-standardized	7992 (6975.6 to 9370.9)	7730.1 (6663 to 9001.2)	8300.2 (7194.5 to 9734.8)	7263.4 (6117.9 to 8628.5)	6477.8 (5388.3 to 7854.7)	8075.7 (6909.4 to 9520.4)	-9.1 (-15.9 to -2.5)	-16.2 (-24.8 to -7.2)	-2.7 (-10.2 to 5.1)
Latin America and Caribbean	Caribbean	Jamaica	Deaths	All Ages	345 (310 to 392)	124 (104 to 162)	221 (199 to 247)	642 (494 to 800)	171 (123 to 271)	471 (359 to 591)	85.9 (44.4 to 135.2)	37.9 (1.7 to 83.9)	112.8 (61.2 to 173.3)
Latin America and Caribbean	Caribbean	Jamaica	Deaths	Age-standardized	18.5 (16.6 to 21.1)	11.9 (10 to 15.9)	26.7 (24 to 29.8)	20.7 (15.9 to 26.1)	9.6 (6.9 to 15.6)	33.7 (25.8 to 42.2)	12.3 (-13.6 to 43.1)	-19.8 (-41.4 to 9)	26.5 (-4.4 to 62)
Latin America and Caribbean	Caribbean	Jamaica	DALYs	All Ages	16,691 (13,734 to 20,334)	7,462 (5,977 to 9,266)	9,229 (7,655 to 11,178)	21,039 (17,078 to 25,549)	7,189 (5,588 to 9,422)	13,850 (11,224 to 16,739)	26 (6.4 to 50.5)	-3.7 (-17.8 to 22.2)	50.1 (24.4 to 83.7)
Latin America and Caribbean	Caribbean	Jamaica	DALYs	Age-standardized	760.4 (640.5 to 901.6)	638.7 (518.9 to 780.9)	905.6 (777.7 to 1056)	741.3 (597.9 to 906.5)	495.3 (377 to 646.1)	1007.1 (814.6 to 1228.9)	-2.5 (-16.7 to 14)	-22.5 (-34.4 to -6.1)	11.2 (-6.4 to 34.2)
Latin America and Caribbean	Caribbean	Jamaica	YLLs	All Ages	8,126 (7,346 to 8,943)	3,216 (2,668 to 3,751)	4,910 (4,407 to 5,458)	12,295 (9,318 to 15,771)	3,197 (2,278 to 5,208)	9,098 (6,902 to 11,604)	51.3 (13.7 to 100.5)	-0.6 (-30.8 to 56.7)	85.3 (38.3 to 144)
Latin America and Caribbean	Caribbean	Jamaica	YLLs	Age-standardized	410.1 (372 to 450.4)	298.4 (253.7 to 350.5)	543.3 (489.2 to 605.8)	417.3 (316.3 to 534.9)	205.6 (146.5 to 336.7)	647.9 (493.4 to 825.9)	1.8 (-23.8 to 32.3)	-31.1 (-51.9 to 4.9)	19.3 (-10.7 to 56.1)
Latin America and Caribbean	Caribbean	Jamaica	YLDs	All Ages	8,565 (5,839 to 12,116)	4,246 (2,873 to 6,026)	4,319 (2,913 to 6,147)	8,744 (6,232 to 11,784)	3,992 (2,780 to 5,468)	4,752 (3,366 to 6,441)	2.1 (-7.6 to 12.7)	-6 (-15.8 to 4.5)	10 (-1.7 to 24.8)
Latin America and Caribbean	Caribbean	Jamaica	YLDs	Age-standardized	350.3 (241.6 to 480.3)	340.3 (234.1 to 475.3)	362.3 (251.7 to 500.7)	323.9 (227.2 to 449.6)	289.7 (197.4 to 409.6)	359.2 (252.4 to 493.6)	-7.5 (-14.9 to -0.2)	-14.9 (-23.6 to -5.8)	-0.9 (-9.7 to 8.7)
Latin America and Caribbean	Caribbean	Puerto Rico	Incidence	All Ages	58,126 (47,984 to 69,732)	30,644 (25,340 to 36,315)	27,481 (22,321 to 33,487)	54,200 (47,668 to 61,527)	30,624 (26,939 to 34,890)	23,576 (20,501 to 27,108)	-6.8 (-15.1 to 4.8)	-0.1 (-10.1 to 14.2)	-14.2 (-22.8 to -3.8)
Latin America and Caribbean	Caribbean	Puerto Rico	Incidence	Age-standardized	1648.9 (1360 to 1979.3)	1691.2 (1392.5 to 2009.4)	1598.3 (1302.5 to 1960.7)	1638.5 (1361.9 to 1989.1)	1690.4 (1422.8 to 2037)	1576.7 (1287 to 1934.3)	-0.6 (-6.7 to 5.6)	0 (-7.6 to 8.8)	-1.4 (-8.6 to 5.6)
Latin America and Caribbean	Caribbean	Puerto Rico	Prevalence	All Ages	434,476 (376,693 to 498,268)	231,243 (202,490 to 265,509)	203,233 (174,943 to 236,840)	367,886 (331,042 to 414,390)	203,446 (182,108 to 228,685)	164,440 (146,449 to 185,460)	-15.3 (-21.7 to -8.9)	-12 (-18.4 to -4.7)	-19.1 (-26.5 to -11.7)
Latin America and Caribbean	Caribbean	Puerto Rico	Prevalence	Age-standardized	12090.8 (10481.2 to 13894.4)	12408.4 (10826.4 to 14318)	11685.4 (10116.1 to 13541.2)	10234.1 (8572.5 to 12124.4)	10565.6 (8942.3 to 12577.5)	9848.5 (8156.3 to 11790.4)	-15.4 (-21.8 to -9.1)	-14.9 (-22 to -7.6)	-15.7 (-23.5 to -7.8)
Latin America and Caribbean	Caribbean	Puerto Rico	Deaths	All Ages	964 (863 to 1,053)	438 (347 to 502)	526 (477 to 579)	1,832 (1,391 to 2,281)	931 (610 to 1,202)	901 (694 to 1,143)	90.1 (49.1 to 139.1)	112.6 (59.5 to 172.4)	71.4 (32.2 to 121.3)
Latin America and Caribbean	Caribbean	Puerto Rico	Deaths	Age-standardized	28 (24.9 to 30.7)	23.3 (18.3 to 26.7)	33.9 (30.7 to 37.5)	21.9 (17 to 27.6)	18.7 (12.9 to 23.9)	26.3 (20.2 to 33.6)	-21.8 (-38.3 to -1.5)	-19.8 (-39.3 to 3.6)	-22.4 (-40.2 to 0.5)
Latin America and Caribbean	Caribbean	Puerto Rico	DALYs	All Ages	37,075 (30,798 to 44,826)	18,217 (14,775 to 22,387)	18,858 (15,950 to 22,751)	42,568 (35,116 to 51,183)	21,748 (17,422 to 25,980)	20,820 (17,092 to 25,538)	14.8 (-2.7 to 37)	19.4 (0.8 to 41.1)	10.4 (-8.2 to 36)
Latin America and Caribbean	Caribbean	Puerto Rico	DALYs	Age-standardized	1032.7 (856.9 to 1254)	963.7 (778.7 to 1186.8)	1113.3 (944.6 to 1334.7)	832.3 (658.7 to 1035.1)	795.2 (620.5 to 1009.2)	881.4 (700.1 to 1099.2)	-19.4 (-28.3 to -8.8)	-17.5 (-26.3 to -7)	-20.8 (-32.1 to -7.9)
Latin America and Caribbean	Caribbean	Puerto Rico	YLLs	All Ages	18,134 (16,718 to 19,719)	8,086 (7,082 to 9,174)	10,048 (9,213 to 10,934)	25,908 (20,147 to 32,745)	12,392 (8,954 to 15,857)	13,516 (10,332 to 17,462)	42.9 (10.6 to 81.4)	53.2 (16.4 to 97.7)	34.5 (1.8 to 78.3)
Latin America and Caribbean	Caribbean	Puerto Rico	YLLs	Age-standardized	505.2 (465.7 to 550.1)	420.2 (369.3 to 477.1)	606.2 (555.5 to 660.3)	375 (289.6 to 474.7)	320.2 (241.2 to 416.2)	445.3 (339.3 to 572.7)	-25.8 (-42.7 to -5.4)	-23.8 (-41.9 to -1)	-26.5 (-44.4 to -3.5)
Latin America and Caribbean	Caribbean	Puerto Rico	YLDs	All Ages	18,941 (12,969 to 26,528)	10,131 (6,957 to 14,085)	8,810 (5,994 to 12,519)	16,659 (12,213 to 21,542)	9,356 (6,867 to 12,194)	7,304 (5,261 to 9,538)	-12 (-21.9 to 0.2)	-7.6 (-17.5 to 4.9)	-17.1 (-28.7 to -4.3)
Latin America and Caribbean	Caribbean	Puerto Rico	YLDs	Age-standardized	527.5 (362.1 to 738.5)	543.5 (371.7 to 757.6)	507.1 (346.7 to 714.2)	457.2 (314.2 to 636.6)	475 (329 to 662.7)	436.1 (295.1 to 613.3)	-13.3 (-19.8 to -6.1)	-12.6 (-19.7 to -4.5)	-14 (-23.3 to -5.1)
Latin America and Caribbean	Caribbean	Saint Kitts and Nevis	Incidence	All Ages	523 (419 to 650)	273 (223 to 332)	250 (193 to 321)	638 (540 to 758)	339 (287 to 399)	300 (250 to 364)	22 (13.2 to 33.2)	23.9 (14.2 to 35.2)	20 (10 to 33)
Latin America and Caribbean	Caribbean	Saint Kitts and Nevis	Incidence	Age-standardized	1188.3 (968.9 to 1449.9)	1230.2 (1021 to 1474.1)	1140.4 (913.5 to 1435.2)	1254.2 (1027.8 to 1553.6)	1284.4 (1062.2 to 1569.5)	1220.1 (984.2 to 1527.3)	5.5 (0.9 to 11.9)	4.4 (-1.9 to 11.7)	7 (0.6 to 14.6)
Latin America and Caribbean	Caribbean	Saint Kitts and Nevis	Prevalence	All Ages	3,074 (2,565 to 3,711)	1,625 (1,385 to 1,933)	1,450 (1,175 to 1,813)	4,004 (3,459 to 4,653)	2,080 (1,797 to 2,419)	1,925 (1,650 to 2,283)	30.2 (21.2 to 40.7)	28 (18.1 to 38.9)	32.8 (20.8 to 46.7)
Latin America and Caribbean	Caribbean	Saint Kitts and Nevis	Prevalence	Age-standardized	7313.7 (6250.8 to 8639.6)	7674.1 (6668.5 to 8936.6)	6949.4 (5793.2 to 8407.9)	7251.7 (6075.2 to 8637.9)	7306.4 (6129.3 to 8715)	7221.4 (5949.3 to 8777.3)	-0.8 (-6.9 to 4.9)	-4.8 (-12.2 to 2.8)	3.9 (-3.3 to 11)
Latin America and Caribbean	Caribbean	Saint Kitts and Nevis	Deaths	All Ages	9 (8 to 10)	4 (4 to 5)	5 (4 to 5)	11 (9 to 13)	4 (3 to 6)	6 (5 to 8)	22.6 (0.2 to 51.3)	4.2 (-21 to 38.9)	39.8 (6.3 to 81.1)
Latin America and Caribbean	Caribbean	Saint Kitts and Nevis	Deaths	Age-standardized	24.8 (21.9 to 28.2)	20.9 (17.4 to 25.1)	31.3 (26.6 to 37)	20.2 (17.1 to 24)	15.2 (11.5 to 19.1)	27.1 (22.7 to 32.2)	-18.4 (-32.8 to -0.9)	-27.5 (-44.3 to -4.1)	-13.1 (-31.8 to 8.5)
Latin America and Caribbean	Caribbean	Saint Kitts and Nevis	DALYs	All Ages	325 (278 to 384)	167 (139 to 199)	158 (132 to 191)	424 (347 to 505)	190 (151 to 233)	234 (190 to 280)	30.7 (12.8 to 52.5)	13.9 (-4.3 to 35.8)	48.6 (22.1 to 80.1)
Latin America and Caribbean	Caribbean	Saint Kitts and Nevis	DALYs	Age-standardized	825.2 (715.4 to 954.6)	800.9 (670.2 to 952.3)	877.1 (746.2 to 1040.1)	715.5 (583.7 to 865)	626.5 (495.1 to 779.6)	821.7 (676.8 to 994.9)	-13.3 (-24.8 to -1)	-21.8 (-33.8 to -7.3)	-6.3 (-21.8 to 10.8)
Latin America and Caribbean	Caribbean	Saint Kitts and Nevis	YLLs	All Ages	191 (169 to 217)	96 (79 to 114)	95 (81 to 113)	243 (193 to 300)	94 (69 to 123)	149 (116 to 189)	27.1 (-2.1 to 63.7)	-2 (-28.7 to 36.1)	56.3 (13.9 to 108.1)
Latin America and Caribbean	Caribbean	Saint Kitts and Nevis	YLLs	Age-standardized	506.4 (449.1 to 577.3)	463 (383.9 to 555.6)	578.2 (492.9 to 681.7)	390.1 (315.9 to 474)	292.4 (215.9 to 380.1)	504.3 (404.7 to 616.5)	-23 (-39.4 to -2.3)	-36.8 (-53.2 to -12.8)	-12.8 (-33.7 to 12.8)
Latin America and Caribbean	Caribbean	Saint Kitts and Nevis	YLDs	All Ages	133 (90 to 188)	71 (49 to 99)	62 (41 to 90)	181 (129 to 243)	96 (70 to 128)	85 (60 to 117)	35.9 (23.4 to 49.7)	35.2 (22.2 to 50.5)	36.7 (21.5 to 54.6)
Latin America and Caribbean	Caribbean	Saint Kitts and Nevis	YLDs	Age-standardized	318.8 (220.7 to 442.3)	337.9 (234.1 to 465.8)	298.9 (204.8 to 423)	325.4 (227.6 to 453.1)	334.1 (235.8 to 456.3)	317.4 (218.5 to 452.5)	2.1 (-5 to 8.5)	-1.1 (-9.1 to 7.4)	6.2 (-2.6 to 14.7)
Latin America and Caribbean	Caribbean	Saint Lucia	Incidence	All Ages	1,961 (1,580 to 2,416)	926 (748 to 1,127)	1,035 (814 to 1,308)	2,068 (1,777 to 2,378)	993 (851 to 1,149)	1,074 (917 to 1,244)	5.4 (-5.2 to 17.3)	7.2 (-3 to 20.2)	3.8 (-8.9 to 18.1)
Latin America and Caribbean	Caribbean	Saint Lucia	Incidence	Age-standardized	1296.1 (1079.4 to 1549.8)	1208.9 (1006.5 to 1435.9)	1392.3 (1137.6 to 1693.4)	1358.3 (1123.3 to 1629.7)	1272.5 (1051 to 1526.7)	1445.9 (1192 to 1729)	4.8 (-1 to 10.3)	5.3 (-2.1 to 13.5)	3.8 (-3.8 to 12.5)
Latin America and Caribbean	Caribbean	Saint Lucia	Prevalence	All Ages	11,710 (9,781 to 14,101)	5,310 (4,506 to 6,345)	6,400 (5,204 to 7,782)	14,162 (12,696 to 15,868)	6,140 (5,432 to 7,001)	8,021 (7,173 to 8,994)	20.9 (10.2 to 33.3)	15.6 (4.9 to 27.1)	25.3 (11.5 to 42.7)
Latin America and Caribbean	Caribbean	Saint Lucia	Prevalence	Age-standardized	8497.9 (7372.9 to 9856.7)	7585 (6655.1 to 8775.6)	9551.3 (8067.8 to 11344.4)	8391.2 (7304 to 9732.6)	7199.6 (6170.7 to 8400.3)	9629.3 (8404.5 to 11189.7)	-1.3 (-7.3 to 4.2)	-5.1 (-12.2 to 2)	0.8 (-9.1 to 10.6)
Latin America and Caribbean	Caribbean	Saint Lucia	Deaths	All Ages	30 (27 to 33)	10 (9 to 12)	20 (18 to 22)	59 (48 to 70)	17 (13 to 22)	41 (33 to 50)	97.7 (60.6 to 140)	74.3 (36 to 123.4)	109.4 (66.6 to 163.4)
Latin America and Caribbean	Caribbean	Saint Lucia	Deaths	Age-standardized	35.9 (32.5 to 39.7)	20.6 (17.7 to 24.5)	61.7 (54.9 to 69.5)	28.9 (23.7 to 34.3)	15.3 (11.6 to 19.1)	47.6 (38.5 to 56.8)	-19.4 (-34.2 to -2.2)	-26 (-42.2 to -5.9)	-22.7 (-38 to -4.6)
Latin America and Caribbean	Caribbean	Saint Lucia	DALYs	All Ages	1,204 (1,017 to 1,427)	466 (385 to 567)	738 (628 to 872)	1,828 (1,536 to 2,126)	618 (501 to 734)	1,210 (1,012 to 1,426)	51.8 (30.9 to 76.4)	32.7 (14.8 to 55.5)	63.9 (36.5 to 96.7)
Latin America and Caribbean	Caribbean	Saint Lucia	DALYs	Age-standardized	1093.5 (951.6 to 1249.8)	771.4 (649.7 to 910.8)	1537 (1350.4 to 1756.7)	954.6 (793.4 to 1128.3)	641.8 (512.3 to 783.4)	1314.8 (1097.8 to 1555.7)	-12.7 (-23.9 to -0.5)	-16.8 (-27.9 to -3.7)	-14.5 (-28 to 0.8)
Latin America and Caribbean	Caribbean	Saint Lucia	YLLs	All Ages	698 (638 to 768)	235 (202 to 279)	462 (412 to 518)	1,195 (966 to 1,442)	339 (260 to 421)	856 (688 to 1,044)	71.2 (37.7 to 109.3)	44 (11.3 to 86.2)	85 (44.1 to 132.9)
Latin America and Caribbean	Caribbean	Saint Lucia	YLLs	Age-standardized	724.6 (660.5 to 799.3)	440.8 (379.4 to 524.1)	1123.7 (1001.2 to 1260.5)	581.6 (472.2 to 701.7)	317.7 (244.8 to 395.8)	891 (725.1 to 1080)	-19.7 (-35.5 to -2.2)	-27.9 (-44.4 to -6.7)	-20.7 (-37.8 to -1)
Latin America and Caribbean	Caribbean	Saint Lucia	YLDs	All Ages	506 (338 to 724)	230 (155 to 325)	276 (182 to 402)	633 (455 to 839)	279 (199 to 370)	354 (254 to 474)	25.1 (11 to 43.7)	21.2 (7.7 to 38.2)	28.4 (10.4 to 51.2)
Latin America and Caribbean	Caribbean	Saint Lucia	YLDs	Age-standardized	369 (256.4 to 515.5)	330.6 (228.2 to 457.1)	413.3 (284.2 to 583.6)	373 (259.8 to 514.9)	324.1 (224.1 to 448.4)	423.8 (296.1 to 585)	1.1 (-5.7 to 7.5)	-2 (-9.8 to 5.6)	2.5 (-7.9 to 12.6)
Latin America and Caribbean	Caribbean	Saint Vincent and the Grenadines	Incidence	All Ages	1,347 (1,070 to 1,720)	666 (531 to 828)	682 (529 to 885)	1,165 (968 to 1,402)	563 (467 to 679)	602 (502 to 728)	-13.6 (-19.7 to -5.9)	-15.5 (-21.8 to -7.6)	-11.7 (-20.4 to -1.4)
Latin America and Caribbean	Caribbean	Saint Vincent and the Grenadines	Incidence	Age-standardized	1086.4 (889.8 to 1346.1)	1070.9 (878.6 to 1306.1)	1108.2 (897 to 1399.3)	1150.6 (938.9 to 1415.2)	1125 (917.5 to 1381.2)	1176.5 (949.4 to 1464.6)	5.9 (0.5 to 11.2)	5.1 (-2.1 to 13.2)	6.2 (-1.2 to 13.8)
Latin America and Caribbean	Caribbean	Saint Vincent and the Grenadines	Prevalence	All Ages	7,603 (6,223 to 9,316)	3,702 (3,033 to 4,500)	3,901 (3,160 to 4,937)	7,154 (6,171 to 8,439)	3,303 (2,821 to 3,936)	3,851 (3,303 to 4,499)	-5.9 (-12.5 to 1.7)	-10.8 (-18 to -2.5)	-1.3 (-10.5 to 8.9)
Latin America and Caribbean	Caribbean	Saint Vincent and the Grenadines	Prevalence	Age-standardized	6552.9 (5574.7 to 7788.1)	6405.4 (5451.6 to 7556.3)	6771.9 (5739.1 to 8188.8)	6635.3 (5600.1 to 8021.5)	6258.6 (5268.6 to 7561.7)	7000.6 (5845.6 to 8434)	1.3 (-4 to 6.9)	-2.3 (-9.2 to 5.7)	3.4 (-4 to 10.6)
Latin America and Caribbean	Caribbean	Saint Vincent and the Grenadines	Deaths	All Ages	11 (9 to 12)	4 (3 to 5)	7 (6 to 8)	20 (17 to 23)	5 (4 to 7)	15 (12 to 17)	87.3 (49.2 to 123.3)	38.2 (-0.8 to 78.1)	115.6 (70.5 to 163.8)
Latin America and Caribbean	Caribbean	Saint Vincent and the Grenadines	Deaths	Age-standardized	15.2 (13.4 to 17.4)	9.7 (8 to 12.2)	24.4 (21 to 28.1)	16.1 (13.7 to 18.5)	8.6 (6.6 to 10.6)	24.5 (20.6 to 28)	6 (-15.5 to 26)	-11.1 (-36.8 to 14.3)	0.2 (-19.8 to 22.1)
Latin America and Caribbean	Caribbean	Saint Vincent and the Grenadines	DALYs	All Ages	575 (461 to 731)	247 (193 to 324)	328 (264 to 411)	727 (608 to 863)	258 (204 to 325)	469 (396 to 547)	26.3 (10 to 45.2)	4.5 (-9.2 to 20)	42.7 (21.1 to 68.7)
Latin America and Caribbean	Caribbean	Saint Vincent and the Grenadines	DALYs	Age-standardized	593.1 (492.7 to 714.7)	477.7 (382.1 to 608.3)	749.5 (633.9 to 888.3)	610.3 (502.5 to 731.1)	454.2 (351.8 to 580.1)	765.9 (640.4 to 905.3)	2.9 (-9.2 to 14.5)	-4.9 (-18.5 to 7.6)	2.2 (-11 to 17)
Latin America and Caribbean	Caribbean	Saint Vincent and the Grenadines	YLLs	All Ages	250 (223 to 285)	88 (74 to 111)	162 (140 to 187)	413 (343 to 484)	112 (86 to 140)	301 (248 to 353)	64.9 (30.9 to 99.8)	27 (-7.5 to 64.8)	85.6 (47.6 to 128.2)
Latin America and Caribbean	Caribbean	Saint Vincent and the Grenadines	YLLs	Age-standardized	312.6 (277.2 to 358.8)	202.6 (167.9 to 257.8)	460.6 (396.3 to 532.6)	319.6 (265.8 to 374)	178.5 (136.6 to 221.3)	460.7 (382.8 to 537.7)	2.3 (-18.7 to 24.3)	-11.9 (-35.5 to 13.5)	0 (-20.7 to 22.6)
Latin America and Caribbean	Caribbean	Saint Vincent and the Grenadines	YLDs	All Ages	325 (215 to 476)	159 (105 to 233)	166 (108 to 246)	314 (217 to 433)	146 (102 to 205)	168 (116 to 230)	-3.4 (-11.3 to 6.8)	-8 (-16.9 to 2.5)	1 (-11.5 to 15.8)
Latin America and Caribbean	Caribbean	Saint Vincent and the Grenadines	YLDs	Age-standardized	280.6 (189.2 to 398.9)	275.1 (186 to 394.6)	288.9 (192.8 to 414.4)	290.7 (197.4 to 409.1)	275.7 (186.8 to 394.6)	305.1 (205.7 to 429.9)	3.6 (-2.2 to 10.2)	0.2 (-7.4 to 9.2)	5.6 (-2.6 to 14.7)
Latin America and Caribbean	Caribbean	Suriname	Incidence	All Ages	4,543 (3,647 to 5,728)	2,295 (1,855 to 2,840)	2,248 (1,781 to 2,879)	6,301 (5,239 to 7,639)	3,160 (2,607 to 3,821)	3,141 (2,595 to 3,890)	38.7 (29.3 to 50)	37.7 (26.5 to 49.8)	39.7 (27.5 to 53.6)
Latin America and Caribbean	Caribbean	Suriname	Incidence	Age-standardized	1112.9 (911.9 to 1372.3)	1122.8 (924.4 to 1360.5)	1109.7 (902.9 to 1382)	1163.9 (956.4 to 1426)	1154.3 (938.8 to 1415)	1176.3 (962.7 to 1469)	4.6 (-1.1 to 10.9)	2.8 (-4.7 to 10.1)	6 (-1 to 14.2)
Latin America and Caribbean	Caribbean	Suriname	Prevalence	All Ages	26,142 (22,128 to 31,220)	13,318 (11,388 to 15,685)	12,824 (10,635 to 15,695)	37,298 (32,148 to 44,184)	18,933 (16,279 to 22,291)	18,365 (15,645 to 22,093)	42.7 (34.6 to 51.4)	42.2 (32.1 to 53.1)	43.2 (32.7 to 55.6)
Latin America and Caribbean	Caribbean	Suriname	Prevalence	Age-standardized	6658.5 (5788.4 to 7738.3)	6829.9 (5966.7 to 7898.8)	6519.4 (5582.8 to 7727.4)	6648.7 (5661.1 to 7921.8)	6638.8 (5623.4 to 7926.9)	6662.5 (5628.8 to 8107.4)	-0.1 (-6.1 to 5.6)	-2.8 (-9.7 to 4.8)	2.2 (-5.3 to 9.9)
Latin America and Caribbean	Caribbean	Suriname	Deaths	All Ages	63 (56 to 73)	28 (24 to 36)	35 (31 to 41)	117 (97 to 139)	49 (38 to 60)	68 (55 to 82)	84.6 (51.6 to 124.9)	76.2 (31.1 to 119.4)	91.2 (53.1 to 138.4)
Latin America and Caribbean	Caribbean	Suriname	Deaths	Age-standardized	25.4 (22.7 to 29.1)	20.3 (17.4 to 25.7)	32.3 (28.4 to 36.9)	21 (17.4 to 24.9)	15.7 (12 to 19.3)	28.1 (23 to 34.2)	-17.4 (-32.1 to -0.1)	-22.5 (-41.6 to -3.5)	-13 (-30.2 to 7.9)
Latin America and Caribbean	Caribbean	Suriname	DALYs	All Ages	2,689 (2,238 to 3,229)	1,283 (1,050 to 1,557)	1,406 (1,172 to 1,691)	4,273 (3,554 to 5,068)	1,963 (1,606 to 2,363)	2,310 (1,899 to 2,773)	58.9 (40 to 83.7)	53 (33.7 to 78.2)	64.3 (41.6 to 95.4)
Latin America and Caribbean	Caribbean	Suriname	DALYs	Age-standardized	834.5 (713 to 969.1)	764.3 (637.8 to 904)	923.1 (794.3 to 1079.6)	741.6 (614.3 to 882.1)	653.3 (531.9 to 789.9)	852.6 (701.6 to 1024.4)	-11.1 (-21.6 to 1.9)	-14.5 (-25.8 to -2)	-7.6 (-21.1 to 8.5)
Latin America and Caribbean	Caribbean	Suriname	YLLs	All Ages	1,555 (1,330 to 1,794)	698 (565 to 839)	857 (726 to 992)	2,602 (2,135 to 3,129)	1,098 (838 to 1,349)	1,504 (1,224 to 1,849)	67.4 (35 to 109.7)	57.4 (21.7 to 103.8)	75.5 (38.3 to 123.8)
Latin America and Caribbean	Caribbean	Suriname	YLLs	Age-standardized	544.1 (477.1 to 623)	462.2 (386.6 to 558.6)	643.2 (560.3 to 739.7)	444.7 (364.2 to 533.1)	351.8 (268.3 to 432.5)	560.7 (458.1 to 684.1)	-18.3 (-33.2 to 1)	-23.9 (-41.3 to -3.5)	-12.8 (-30.2 to 9.9)
Latin America and Caribbean	Caribbean	Suriname	YLDs	All Ages	1,134 (772 to 1,599)	585 (405 to 820)	549 (368 to 790)	1,671 (1,171 to 2,289)	865 (610 to 1,162)	806 (560 to 1,138)	47.3 (36.9 to 59.8)	47.8 (35 to 61.8)	46.8 (33.4 to 63.1)
Latin America and Caribbean	Caribbean	Suriname	YLDs	Age-standardized	290.4 (203.5 to 402.5)	302.1 (213.3 to 413.4)	279.8 (194.6 to 390.1)	296.9 (206 to 412.6)	301.6 (210.8 to 411.5)	292 (202.5 to 413.4)	2.2 (-4.2 to 8.7)	-0.2 (-7.3 to 7.5)	4.3 (-4.2 to 13.4)
Latin America and Caribbean	Caribbean	Trinidad and Tobago	Incidence	All Ages	12,154 (9,841 to 15,034)	5,936 (4,902 to 7,139)	6,218 (4,929 to 7,913)	13,509 (11,375 to 15,989)	7,186 (5,949 to 8,631)	6,323 (5,309 to 7,502)	11.1 (0.5 to 23)	21 (4.2 to 36.1)	1.7 (-8 to 13)
Latin America and Caribbean	Caribbean	Trinidad and Tobago	Incidence	Age-standardized	981.4 (814.8 to 1184.5)	968.2 (818.3 to 1146.3)	995.2 (816.2 to 1232.7)	1092.8 (888.2 to 1349.7)	1143.9 (915.9 to 1436)	1042.5 (839.6 to 1295.4)	11.4 (1.7 to 20.4)	18.1 (2.2 to 32.5)	4.8 (-3.7 to 13.7)
Latin America and Caribbean	Caribbean	Trinidad and Tobago	Prevalence	All Ages	64,314 (54,316 to 77,015)	30,064 (25,618 to 35,452)	34,249 (28,570 to 41,482)	73,349 (62,250 to 86,325)	36,734 (28,107 to 44,881)	36,615 (31,968 to 42,418)	14 (-0.3 to 26.6)	22.2 (-5.1 to 40.6)	6.9 (-1.7 to 17.8)
Latin America and Caribbean	Caribbean	Trinidad and Tobago	Prevalence	Age-standardized	5301.8 (4591.6 to 6205.2)	4933.6 (4283.3 to 5728)	5710.1 (4910.5 to 6727.9)	5657.9 (4656.3 to 6895.4)	5717.8 (4369.5 to 7228.2)	5633 (4753.9 to 6760.8)	6.7 (-5.8 to 17.8)	15.9 (-9.1 to 33.4)	-1.4 (-9.4 to 7.2)
Latin America and Caribbean	Caribbean	Trinidad and Tobago	Deaths	All Ages	201 (188 to 218)	79 (69 to 92)	122 (113 to 131)	271 (201 to 360)	96 (68 to 153)	175 (128 to 229)	35.1 (0.1 to 77.6)	22 (-13.9 to 82.5)	43.6 (4.6 to 90.4)
Latin America and Caribbean	Caribbean	Trinidad and Tobago	Deaths	Age-standardized	25.7 (23.8 to 28)	18.5 (16.2 to 21.6)	35.9 (33 to 38.7)	15.6 (11.6 to 20.6)	10.2 (7.3 to 16)	22.2 (16.5 to 28.8)	-39.3 (-54.2 to -21)	-44.6 (-60.6 to -18.9)	-38.2 (-54.2 to -19.2)
Latin America and Caribbean	Caribbean	Trinidad and Tobago	DALYs	All Ages	7,897 (6,962 to 9,093)	3,416 (2,884 to 4,008)	4,481 (3,939 to 5,142)	8,977 (7,099 to 11,326)	3,620 (2,770 to 4,904)	5,356 (4,202 to 6,752)	13.7 (-7 to 39.8)	6 (-15.4 to 41)	19.5 (-5.5 to 50.4)
Latin America and Caribbean	Caribbean	Trinidad and Tobago	DALYs	Age-standardized	779.4 (702.5 to 872.8)	642.4 (555.4 to 742.5)	947 (855.5 to 1052.7)	583.9 (457.9 to 732.3)	481.2 (363.4 to 633.2)	700.3 (552.9 to 876.1)	-25.1 (-37.8 to -9.9)	-25.1 (-40.2 to -5.3)	-26.1 (-40.4 to -10.3)
Latin America and Caribbean	Caribbean	Trinidad and Tobago	YLLs	All Ages	5,092 (4,689 to 5,499)	2,096 (1,730 to 2,366)	2,996 (2,762 to 3,246)	5,701 (4,133 to 7,773)	1,979 (1,382 to 3,119)	3,722 (2,696 to 4,963)	12 (-19.2 to 52.8)	-5.6 (-35.1 to 56.7)	24.2 (-11.6 to 68.5)
Latin America and Caribbean	Caribbean	Trinidad and Tobago	YLLs	Age-standardized	547.2 (507.7 to 591.6)	424.6 (363.6 to 482)	698.6 (648 to 753.6)	332.9 (243.5 to 447.5)	227.5 (160.7 to 345.3)	450.5 (330.7 to 596.1)	-39.2 (-55.7 to -18.4)	-46.4 (-62.5 to -17.3)	-35.5 (-53.2 to -14)
Latin America and Caribbean	Caribbean	Trinidad and Tobago	YLDs	All Ages	2,806 (1,917 to 3,912)	1,320 (913 to 1,856)	1,485 (1,004 to 2,099)	3,276 (2,293 to 4,495)	1,641 (1,068 to 2,338)	1,634 (1,176 to 2,175)	16.7 (2.8 to 30.3)	24.3 (-1.1 to 43.1)	10 (-1.7 to 25.8)
Latin America and Caribbean	Caribbean	Trinidad and Tobago	YLDs	Age-standardized	232.2 (162.6 to 319)	217.7 (153.1 to 299.3)	248.3 (170.4 to 347)	251 (171.5 to 357.8)	253.6 (161 to 373.3)	249.7 (176.3 to 346.9)	8.1 (-3.8 to 20.1)	16.5 (-7.7 to 36)	0.6 (-7.9 to 9.6)
Latin America and Caribbean	Caribbean	United States Virgin Islands	Incidence	All Ages	1,142 (927 to 1,419)	589 (486 to 714)	553 (440 to 700)	1,304 (1,124 to 1,514)	668 (577 to 775)	635 (535 to 754)	14.1 (3.1 to 27.8)	13.5 (1.8 to 28.2)	14.8 (2.5 to 29.9)
Latin America and Caribbean	Caribbean	United States Virgin Islands	Incidence	Age-standardized	1095.4 (898.7 to 1346.5)	1109.6 (921.4 to 1334.5)	1072.7 (869.5 to 1339.1)	1248.5 (1031.7 to 1516.3)	1205.5 (1000.5 to 1456.8)	1287.8 (1051.3 to 1584.1)	14 (7.9 to 19.4)	8.6 (1.9 to 15.5)	20 (11 to 29.7)
Latin America and Caribbean	Caribbean	United States Virgin Islands	Prevalence	All Ages	6,173 (5,174 to 7,430)	3,234 (2,736 to 3,847)	2,939 (2,401 to 3,638)	6,769 (6,011 to 7,727)	3,394 (3,017 to 3,885)	3,375 (2,965 to 3,912)	9.6 (2.4 to 17.7)	5 (-3.1 to 13.6)	14.8 (5.3 to 25.7)
Latin America and Caribbean	Caribbean	United States Virgin Islands	Prevalence	Age-standardized	5917.2 (5003.9 to 7066.5)	6016.3 (5121.4 to 7160.2)	5770.4 (4798.8 to 7031)	6553.7 (5532.6 to 7824.1)	6255.2 (5247.4 to 7505.8)	6849.8 (5730.3 to 8324.5)	10.8 (5.6 to 16.1)	4 (-2.8 to 11.7)	18.7 (11.4 to 27.3)
Latin America and Caribbean	Caribbean	United States Virgin Islands	Deaths	All Ages	14 (12 to 17)	7 (6 to 9)	7 (6 to 9)	31 (25 to 35)	12 (9 to 15)	19 (15 to 22)	117.6 (70.4 to 170.3)	69.3 (25.8 to 124.6)	165.4 (98.3 to 239.9)
Latin America and Caribbean	Caribbean	United States Virgin Islands	Deaths	Age-standardized	19.4 (16.4 to 23.3)	17.2 (13.7 to 21.7)	23.1 (19.3 to 27.7)	17.6 (14.5 to 20.3)	12.1 (9 to 15)	25.5 (20.4 to 29.5)	-9.4 (-28.4 to 11.1)	-29.8 (-47.3 to -7.8)	10.1 (-16.3 to 39.7)
Latin America and Caribbean	Caribbean	United States Virgin Islands	DALYs	All Ages	604 (501 to 730)	304 (248 to 379)	300 (248 to 365)	890 (751 to 1,029)	368 (300 to 441)	522 (427 to 606)	47.2 (24.6 to 74)	21.1 (1.4 to 45.3)	73.7 (40.9 to 108.5)
Latin America and Caribbean	Caribbean	United States Virgin Islands	DALYs	Age-standardized	646.7 (544.5 to 769)	612.2 (502.4 to 757.3)	692.9 (578.1 to 822.2)	626.3 (514 to 759)	505.6 (403.9 to 629.9)	777.6 (631.1 to 925.9)	-3.2 (-16.6 to 10.9)	-17.4 (-30.1 to -3.5)	12.2 (-6.5 to 32.3)
Latin America and Caribbean	Caribbean	United States Virgin Islands	YLLs	All Ages	336 (284 to 404)	162 (130 to 201)	174 (144 to 210)	586 (477 to 683)	213 (162 to 266)	373 (296 to 440)	74.3 (35.5 to 121.7)	31.3 (-3 to 75.1)	114.3 (58.4 to 176.4)
Latin America and Caribbean	Caribbean	United States Virgin Islands	YLLs	Age-standardized	389.6 (328.7 to 467.4)	348.6 (281.2 to 431.1)	444.7 (370 to 535.4)	334.9 (272.2 to 394.6)	224.4 (172 to 281.2)	476.9 (379.9 to 565.1)	-14 (-33.3 to 8.6)	-35.6 (-52.6 to -14)	7.2 (-20.3 to 38.6)
Latin America and Caribbean	Caribbean	United States Virgin Islands	YLDs	All Ages	268 (182 to 386)	142 (96 to 200)	126 (84 to 186)	304 (218 to 408)	155 (111 to 205)	149 (106 to 205)	13.3 (2.2 to 27)	9.4 (-1.4 to 22.9)	17.8 (4.1 to 35.5)
Latin America and Caribbean	Caribbean	United States Virgin Islands	YLDs	Age-standardized	257.1 (176.3 to 365.9)	263.5 (180.1 to 368.6)	248.2 (168.2 to 358)	291.3 (199.9 to 415.1)	281.2 (193.2 to 393.8)	300.7 (208.7 to 431.3)	13.3 (7.5 to 19.7)	6.7 (-0.7 to 14.9)	21.2 (12.7 to 31.3)
		Andean Latin America	Incidence	All Ages	593,228 (468,076 to 732,421)	304,708 (241,553 to 373,283)	288,520 (225,799 to 362,868)	925,985 (777,097 to 1,113,322)	473,337 (396,971 to 563,406)	452,648 (374,419 to 551,409)	56.1 (37.1 to 75.7)	55.3 (35.1 to 76.6)	56.9 (38 to 78.3)
		Andean Latin America	Incidence	Age-standardized	1478.3 (1225.4 to 1746.1)	1531.9 (1279.5 to 1801.4)	1423 (1165.2 to 1715.4)	1547.1 (1297.3 to 1850.7)	1566.6 (1307.5 to 1858.5)	1526.9 (1270.9 to 1838.8)	4.7 (-5.3 to 12.7)	2.3 (-8.1 to 11.3)	7.3 (-3.1 to 16.4)
		Andean Latin America	Prevalence	All Ages	2,794,666 (2,264,516 to 3,442,478)	1,462,813 (1,179,227 to 1,800,590)	1,331,853 (1,072,768 to 1,658,929)	3,434,185 (2,812,614 to 4,232,116)	1,746,574 (1,439,663 to 2,122,835)	1,687,611 (1,360,493 to 2,113,116)	22.9 (9.2 to 36.5)	19.4 (5.4 to 34)	26.7 (12.6 to 41.4)
		Andean Latin America	Prevalence	Age-standardized	6388.9 (5364.6 to 7647.3)	6750.5 (5636.5 to 8065.7)	6023.8 (5028.8 to 7238.6)	5519.3 (4546.8 to 6761.3)	5620.2 (4630.5 to 6826.2)	5415.9 (4423.1 to 6697)	-13.6 (-22.2 to -5)	-16.7 (-25.3 to -7.8)	-10.1 (-19.6 to -0.9)
		Andean Latin America	Deaths	All Ages	6,889 (6,131 to 7,876)	3,138 (2,731 to 3,710)	3,751 (3,274 to 4,353)	14,209 (11,012 to 17,013)	6,612 (4,698 to 8,094)	7,597 (5,997 to 9,172)	106.3 (60.9 to 150.2)	110.7 (58.7 to 163.3)	102.5 (51.9 to 152.6)
		Andean Latin America	Deaths	Age-standardized	33.7 (30.1 to 40.6)	29.7 (25.8 to 38.6)	38.2 (33.7 to 47.2)	26.8 (20.8 to 32.1)	23.3 (16.6 to 28.6)	30.8 (24.4 to 37.1)	-20.4 (-41 to -2.8)	-21.3 (-46.5 to -0.5)	-19.3 (-42.9 to -0.4)
		Andean Latin America	DALYs	All Ages	348,007 (286,044 to 410,810)	169,254 (132,621 to 206,659)	178,753 (149,497 to 212,476)	393,916 (323,481 to 472,210)	188,659 (153,066 to 228,002)	205,257 (166,371 to 247,637)	13.2 (-1.9 to 30.5)	11.5 (-4.7 to 30.5)	14.8 (-3.5 to 36.2)
		Andean Latin America	DALYs	Age-standardized	1035.1 (903.6 to 1176.3)	970.7 (814.2 to 1131.2)	1105.8 (959.2 to 1266.6)	680.1 (559.5 to 811.8)	631.9 (512.1 to 763.9)	733.9 (599.3 to 877.9)	-34.3 (-43.4 to -24.4)	-34.9 (-43.8 to -25.3)	-33.6 (-45 to -21.6)
		Andean Latin America	YLLs	All Ages	223,782 (186,691 to 255,729)	103,582 (75,552 to 125,607)	120,200 (100,718 to 139,657)	234,384 (184,249 to 285,071)	106,079 (79,630 to 131,254)	128,304 (102,602 to 156,173)	4.7 (-16.1 to 29.7)	2.4 (-20.6 to 31)	6.7 (-17.8 to 37.5)
		Andean Latin America	YLLs	Age-standardized	746.9 (659.1 to 837.8)	662.7 (546.8 to 775.9)	837.6 (728.7 to 957.5)	423 (332.5 to 513.3)	366 (274 to 452.6)	485.9 (388.8 to 590.3)	-43.4 (-54.6 to -30.7)	-44.8 (-55.7 to -31.4)	-42 (-55.9 to -26.5)
		Andean Latin America	YLDs	All Ages	124,225 (81,967 to 182,201)	65,672 (43,436 to 95,535)	58,553 (38,174 to 86,202)	159,532 (111,644 to 227,695)	82,580 (58,212 to 115,968)	76,952 (52,500 to 110,590)	28.4 (12.8 to 46.2)	25.7 (9.2 to 44.5)	31.4 (15 to 50.1)
		Andean Latin America	YLDs	Age-standardized	288.2 (199.7 to 404.8)	307.9 (212.1 to 430.4)	268.2 (182.1 to 375.5)	257.1 (181.8 to 364.3)	265.9 (187.5 to 371.7)	248 (171 to 353.5)	-10.8 (-20 to -1.2)	-13.7 (-23.2 to -4)	-7.5 (-17.7 to 3)
Latin America and Caribbean	Andean Latin America	Bolivia (Plurinational State of)	Incidence	All Ages	105,023 (82,874 to 129,695)	52,113 (42,173 to 63,917)	52,910 (40,658 to 65,994)	170,325 (140,359 to 208,257)	87,105 (72,235 to 104,378)	83,220 (67,759 to 104,520)	62.2 (44.5 to 79.3)	67.1 (48.4 to 86.9)	57.3 (38.9 to 77.2)
Latin America and Caribbean	Andean Latin America	Bolivia (Plurinational State of)	Incidence	Age-standardized	1487.2 (1260.5 to 1748.4)	1506.5 (1279.9 to 1759.8)	1460 (1208 to 1729.2)	1488.6 (1254.4 to 1759.6)	1524.9 (1290.9 to 1786.3)	1448.2 (1210.1 to 1750.7)	0.1 (-7.7 to 7.3)	1.2 (-7.8 to 10)	-0.8 (-10 to 7.5)
Latin America and Caribbean	Andean Latin America	Bolivia (Plurinational State of)	Prevalence	All Ages	498,145 (403,680 to 611,574)	246,887 (201,998 to 303,492)	251,258 (198,875 to 309,379)	708,048 (579,436 to 865,706)	357,904 (295,937 to 432,066)	350,144 (281,110 to 437,589)	42.1 (27.5 to 56.8)	45 (27.2 to 62.3)	39.4 (22.9 to 56.6)
Latin America and Caribbean	Andean Latin America	Bolivia (Plurinational State of)	Prevalence	Age-standardized	6805.4 (5787.7 to 8011.6)	6889.3 (5903.9 to 8114.4)	6691.8 (5565.8 to 7943.6)	5973.7 (5033.4 to 7169.2)	6054.8 (5133.1 to 7157.8)	5892.7 (4898.3 to 7193.5)	-12.2 (-19.4 to -5.3)	-12.1 (-20.9 to -3.1)	-11.9 (-20.5 to -3.2)
Latin America and Caribbean	Andean Latin America	Bolivia (Plurinational State of)	Deaths	All Ages	1,856 (1,475 to 2,221)	909 (649 to 1,153)	947 (718 to 1,172)	3,578 (2,812 to 4,445)	1,773 (1,277 to 2,259)	1,804 (1,338 to 2,309)	92.8 (50.4 to 144.4)	95.1 (50.2 to 159.1)	90.5 (40.9 to 160.4)
Latin America and Caribbean	Andean Latin America	Bolivia (Plurinational State of)	Deaths	Age-standardized	62.1 (51.3 to 73.1)	55 (42.7 to 68)	71.6 (55.1 to 88.1)	51.1 (40.1 to 63.1)	47.2 (33.6 to 59.9)	55.7 (41.2 to 71.6)	-17.7 (-35.6 to 3)	-14.3 (-33.8 to 12.1)	-22.2 (-42 to 3.6)
Latin America and Caribbean	Andean Latin America	Bolivia (Plurinational State of)	DALYs	All Ages	86,366 (63,562 to 108,920)	42,764 (27,813 to 60,115)	43,602 (31,823 to 56,865)	98,267 (79,671 to 118,546)	49,096 (38,461 to 59,677)	49,171 (39,113 to 60,599)	13.8 (-11.7 to 49.4)	14.8 (-17.8 to 58)	12.8 (-15.7 to 52.4)
Latin America and Caribbean	Andean Latin America	Bolivia (Plurinational State of)	DALYs	Age-standardized	1619.8 (1317.5 to 1905.4)	1524.8 (1133.2 to 1925.8)	1740.6 (1372.2 to 2109)	1063.5 (870.2 to 1274.7)	1018.1 (794.7 to 1241.6)	1117.9 (878.7 to 1368.8)	-34.3 (-46.2 to -20.6)	-33.2 (-46.7 to -16.5)	-35.8 (-49.7 to -17.7)
Latin America and Caribbean	Andean Latin America	Bolivia (Plurinational State of)	YLLs	All Ages	64,176 (42,945 to 85,592)	31,643 (17,560 to 49,642)	32,533 (22,108 to 44,752)	65,797 (51,744 to 81,673)	32,299 (23,223 to 41,694)	33,498 (25,178 to 43,017)	2.5 (-26.6 to 49.7)	2.1 (-33.4 to 62.2)	3 (-31.6 to 55.7)
Latin America and Caribbean	Andean Latin America	Bolivia (Plurinational State of)	YLLs	Age-standardized	1311.7 (1022.4 to 1569.6)	1207.8 (824 to 1590)	1443.3 (1094.6 to 1784.9)	786.6 (619.6 to 974.5)	730.6 (529.3 to 936.7)	852 (631.4 to 1090.7)	-40 (-53.3 to -23.7)	-39.5 (-55.1 to -19)	-41 (-56.8 to -19.2)
Latin America and Caribbean	Andean Latin America	Bolivia (Plurinational State of)	YLDs	All Ages	22,191 (14,653 to 32,505)	11,121 (7,456 to 16,054)	11,069 (7,106 to 16,481)	32,470 (22,635 to 45,688)	16,797 (11,910 to 23,188)	15,673 (10,597 to 22,604)	46.3 (30 to 64.4)	51 (32.2 to 72.7)	41.6 (23 to 62.2)
Latin America and Caribbean	Andean Latin America	Bolivia (Plurinational State of)	YLDs	Age-standardized	308.2 (216.3 to 425.2)	317.1 (225.4 to 435.9)	297.3 (205.5 to 414.6)	276.9 (196.6 to 380.7)	287.5 (206.6 to 387.3)	265.9 (184.8 to 371.4)	-10.2 (-17.9 to -2)	-9.3 (-18.3 to 0.1)	-10.6 (-20.5 to -0.7)
Latin America and Caribbean	Andean Latin America	Ecuador	Incidence	All Ages	138,760 (107,440 to 174,462)	70,302 (54,494 to 88,558)	68,458 (53,058 to 87,116)	229,589 (191,154 to 276,088)	115,745 (96,970 to 137,687)	113,844 (93,969 to 138,866)	65.5 (44.6 to 88.5)	64.6 (41.9 to 91.8)	66.3 (43.7 to 90.7)
Latin America and Caribbean	Andean Latin America	Ecuador	Incidence	Age-standardized	1273.7 (1038.9 to 1536.4)	1298.5 (1051.5 to 1574.6)	1248.9 (1015 to 1513.6)	1406.2 (1179 to 1679.7)	1397.7 (1172.8 to 1657.6)	1415.1 (1174.9 to 1702.2)	10.4 (-1.1 to 21.4)	7.6 (-4.6 to 20.3)	13.3 (-0.1 to 26.1)
Latin America and Caribbean	Andean Latin America	Ecuador	Prevalence	All Ages	687,727 (543,258 to 858,482)	353,651 (277,157 to 441,086)	334,076 (264,283 to 419,048)	901,984 (734,423 to 1,126,682)	453,342 (370,525 to 563,218)	448,643 (360,601 to 562,349)	31.2 (15.5 to 47.9)	28.2 (10.8 to 47.5)	34.3 (17.2 to 54.4)
Latin America and Caribbean	Andean Latin America	Ecuador	Prevalence	Age-standardized	6066.5 (5016 to 7333.1)	6264.1 (5126.3 to 7565.6)	5867.3 (4853 to 7116.7)	5298.4 (4352.9 to 6561)	5290.6 (4355.2 to 6551.8)	5314 (4339.7 to 6603.4)	-12.7 (-22 to -3)	-15.5 (-25.8 to -4.8)	-9.4 (-20 to 2.1)
Latin America and Caribbean	Andean Latin America	Ecuador	Deaths	All Ages	1,713 (1,424 to 1,860)	768 (564 to 861)	945 (819 to 1,030)	3,670 (2,838 to 4,523)	1,606 (1,114 to 1,964)	2,064 (1,555 to 2,609)	114.3 (76.1 to 164.2)	109.2 (65.3 to 158.8)	118.4 (74.9 to 172.5)
Latin America and Caribbean	Andean Latin America	Ecuador	Deaths	Age-standardized	35 (30.2 to 37.6)	31.7 (24.7 to 35.2)	38.7 (33.3 to 42)	29.8 (22.5 to 36.6)	24.2 (16.5 to 29.8)	36.5 (26.8 to 45.7)	-14.7 (-31.2 to 3.9)	-23.6 (-42.4 to -5.8)	-5.5 (-24.3 to 17.6)
Latin America and Caribbean	Andean Latin America	Ecuador	DALYs	All Ages	81,140 (63,436 to 98,798)	39,323 (27,494 to 48,806)	41,817 (33,731 to 50,157)	97,494 (80,361 to 118,125)	45,077 (36,859 to 55,508)	52,417 (41,947 to 64,535)	20.2 (3 to 47.1)	14.6 (-4 to 50.4)	25.3 (7.2 to 52.8)
Latin America and Caribbean	Andean Latin America	Ecuador	DALYs	Age-standardized	943.2 (794.2 to 1082.3)	888.4 (678 to 1042.3)	1003.6 (870 to 1137.3)	644 (535.2 to 774.7)	570.5 (466.8 to 697)	729.5 (593.7 to 889.2)	-31.7 (-40.3 to -20)	-35.8 (-44.7 to -22.3)	-27.3 (-37.7 to -13.8)
Latin America and Caribbean	Andean Latin America	Ecuador	YLLs	All Ages	50,586 (35,218 to 58,964)	23,438 (13,477 to 28,915)	27,148 (19,748 to 31,068)	55,921 (44,459 to 69,921)	23,828 (18,326 to 29,667)	32,093 (25,134 to 40,984)	10.5 (-12.8 to 52.7)	1.7 (-23.4 to 63.6)	18.2 (-7.1 to 56.2)
Latin America and Caribbean	Andean Latin America	Ecuador	YLLs	Age-standardized	670.5 (539 to 739.4)	603 (415 to 697.3)	743.7 (636.6 to 815.4)	399.1 (314.2 to 495.1)	322 (243.7 to 395.4)	488 (378.6 to 620.7)	-40.5 (-51.5 to -24.1)	-46.6 (-57.4 to -28.8)	-34.4 (-47.7 to -16.9)
Latin America and Caribbean	Andean Latin America	Ecuador	YLDs	All Ages	30,554 (19,678 to 44,949)	15,885 (10,340 to 23,385)	14,669 (9,552 to 21,869)	41,573 (28,779 to 59,229)	21,249 (14,779 to 29,849)	20,323 (13,751 to 29,517)	36.1 (17.9 to 58.3)	33.8 (14.4 to 57.8)	38.5 (18.7 to 62.8)
Latin America and Caribbean	Andean Latin America	Ecuador	YLDs	Age-standardized	272.7 (187.6 to 385.7)	285.4 (194.7 to 405.1)	259.9 (177.3 to 369.4)	245 (171.8 to 344.7)	248.5 (174.5 to 347.6)	241.5 (166.3 to 344.7)	-10.2 (-20.6 to 1.1)	-12.9 (-24.1 to -0.4)	-7.1 (-18.3 to 6.5)
Latin America and Caribbean	Andean Latin America	Peru	Incidence	All Ages	349,445 (275,578 to 433,351)	182,293 (144,723 to 224,258)	167,152 (129,865 to 212,455)	526,071 (435,977 to 631,662)	270,487 (224,145 to 324,844)	255,584 (213,014 to 309,632)	50.5 (29.4 to 73.4)	48.4 (23.1 to 74.3)	52.9 (32.9 to 78.1)
Latin America and Caribbean	Andean Latin America	Peru	Incidence	Age-standardized	1570.2 (1295.7 to 1854.9)	1648.6 (1364.5 to 1940.8)	1490.5 (1217.2 to 1810.7)	1634.5 (1352.7 to 1971.1)	1665.9 (1377.6 to 2010.6)	1602.9 (1330.8 to 1937.3)	4.1 (-7.5 to 14.4)	1.1 (-11.8 to 13.4)	7.5 (-3.8 to 19.4)
Latin America and Caribbean	Andean Latin America	Peru	Prevalence	All Ages	1,608,794 (1,296,288 to 1,983,266)	862,275 (694,779 to 1,063,187)	746,519 (597,019 to 925,956)	1,824,153 (1,485,934 to 2,262,887)	935,328 (765,772 to 1,153,613)	888,825 (715,616 to 1,113,474)	13.4 (-1.3 to 29)	8.5 (-6.6 to 25.7)	19.1 (2.8 to 36.7)
Latin America and Caribbean	Andean Latin America	Peru	Prevalence	Age-standardized	6440.3 (5377.5 to 7728.9)	6964.9 (5771.7 to 8374.3)	5914.8 (4902.7 to 7160.2)	5534.5 (4478.9 to 6891)	5700.8 (4620.7 to 7107.2)	5364.5 (4319 to 6722.9)	-14.1 (-24.4 to -3.5)	-18.1 (-28.7 to -6)	-9.3 (-21.1 to 3)
Latin America and Caribbean	Andean Latin America	Peru	Deaths	All Ages	3,320 (2,825 to 4,232)	1,461 (1,207 to 2,016)	1,859 (1,559 to 2,493)	6,961 (5,132 to 8,990)	3,233 (2,168 to 4,245)	3,728 (2,671 to 4,882)	109.7 (41.8 to 189.9)	121.2 (40.4 to 215.3)	100.6 (27.5 to 176.9)
Latin America and Caribbean	Andean Latin America	Peru	Deaths	Age-standardized	27 (22.7 to 36.9)	23 (18.8 to 34.5)	31.3 (26 to 45)	21.3 (15.6 to 27.6)	18.5 (12.3 to 24.3)	24.4 (17.5 to 32)	-21.1 (-49.2 to 8.9)	-19.7 (-54 to 16.8)	-21.9 (-53.7 to 8.6)

Chronic respiratory diseases

7 GBD super region	21 GBD region	Location	Measure	Age	1990 Both	1990 Female	1990 Male	2019 Both	2019 Female	2019 Male	Percent change (1990 to 2019) Both	Percent change (1990 to 2019) Female	Percent change (1990 to 2019) Male
Latin America and Caribbean	Andean Latin America	Peru	DALYs	All Ages	180,501 (148,122 to 219,116)	87,167 (68,368 to 111,156)	93,334 (75,784 to 114,101)	198,155 (156,432 to 246,443)	94,486 (73,777 to 117,441)	103,669 (80,725 to 129,639)	9.8 (-9.9 to 35.4)	8.4 (-12.8 to 33.2)	11.1 (-12.7 to 40.7)
Latin America and Caribbean	Andean Latin America	Peru	DALYs	Age-standardized	920.4 (783.4 to 1088.7)	855.5 (705.3 to 1046.4)	990.4 (829.8 to 1190.6)	607.6 (478.4 to 758.1)	566.9 (442.9 to 704.2)	652 (503.7 to 814.8)	-34 (-47.4 to -19.3)	-33.7 (-46.2 to -19)	-34.2 (-50.6 to -16.4)
Latin America and Caribbean	Andean Latin America	Peru	YLLs	All Ages	109,020 (91,640 to 129,863)	48,501 (36,717 to 63,565)	60,519 (48,717 to 75,493)	112,665 (82,680 to 145,428)	49,952 (33,833 to 65,859)	62,713 (44,304 to 83,797)	3.3 (-26.1 to 42.4)	3 (-27.6 to 40)	3.6 (-29.2 to 53.3)
Latin America and Caribbean	Andean Latin America	Peru	YLLs	Age-standardized	629.8 (540.4 to 754.8)	538.4 (439.8 to 674.4)	726.4 (601.4 to 895.1)	348.5 (255.1 to 458.9)	296.7 (202.2 to 391.7)	404.3 (284.3 to 541.4)	-44.7 (-61.5 to -23.8)	-44.9 (-61.7 to -23)	-44.3 (-63.3 to -22.1)
Latin America and Caribbean	Andean Latin America	Peru	YLDs	All Ages	71,481 (47,327 to 104,110)	38,666 (25,297 to 56,630)	32,815 (21,467 to 48,466)	85,490 (59,653 to 121,111)	44,534 (31,529 to 62,736)	40,956 (28,324 to 58,818)	19.6 (3 to 38.6)	15.2 (-2.5 to 37.2)	24.8 (6.8 to 44.8)
Latin America and Caribbean	Andean Latin America	Peru	YLDs	Age-standardized	290.6 (198.6 to 408.3)	317.1 (215.3 to 449.5)	264 (178 to 375)	259.1 (180 to 368.7)	270.2 (189.2 to 384.1)	247.6 (171.6 to 355.6)	-10.9 (-22 to 0.7)	-14.8 (-26.2 to -1.9)	-6.2 (-18.4 to 6.7)
[illegible]	[illegible]	[illegible]	[illegible]	[illegible]	[illegible]	[illegible]	[illegible]	[illegible]	[illegible]	[illegible]	[illegible]	[illegible]	[illegible]

Chronic respiratory diseases

7 GBD super region	21 GBD region	Location	Measure	Age	1990 Both	1990 Female	1990 Male	2019 Both	2019 Female	2019 Male	Percent change (1990 to 2019) Both	Percent change (1990 to 2019) Female	Percent change (1990 to 2019) Male
North Africa and Middle East	North Africa and Middle East	Kuwait	Deaths	Age-standardized	17.2 (14.4 to 19.6)	15.8 (12 to 19)	19.1 (15.9 to 22.1)	12.2 (9.7 to 14.9)	8.4 (6.2 to 11.3)	14.8 (11.2 to 18.7)	-29 (-43 to -11.9)	-47 (-61.3 to -23.3)	-22.7 (-40.2 to -0.4)
North Africa and Middle East	North Africa and Middle East	Kuwait	DALYs	All Ages	6,473 (5,196 to 8,224)	2,700 (2,178 to 3,393)	3,773 (2,988 to 4,796)	15,860 (12,443 to 19,931)	6,866 (5,300 to 8,850)	8,993 (7,085 to 11,301)	145 (121.9 to 174)	154.3 (120.3 to 196.6)	138.4 (112.2 to 170.6)
North Africa and Middle East	North Africa and Middle East	Kuwait	DALYs	Age-standardized	595.5 (501.7 to 697.7)	589.8 (487.5 to 697.5)	615.8 (515.5 to 731.4)	470.2 (383.1 to 575.6)	412.6 (328.8 to 515.2)	507.9 (407.8 to 622.2)	-21 (-29.9 to -10.9)	-30.1 (-40.6 to -15.8)	-17.5 (-28.5 to -5.2)
North Africa and Middle East	North Africa and Middle East	Kuwait	YLLs	All Ages	2,301 (2,028 to 2,565)	967 (759 to 1,121)	1,334 (1,147 to 1,541)	4,476 (3,644 to 5,544)	1,506 (1,161 to 2,152)	2,970 (2,283 to 3,733)	94.5 (55.3 to 148.4)	55.6 (15.4 to 138.9)	122.7 (69.9 to 195.2)
North Africa and Middle East	North Africa and Middle East	Kuwait	YLLs	Age-standardized	315.8 (271.3 to 356.9)	308.4 (236.4 to 365.6)	330.6 (278.8 to 381.8)	191.9 (154.5 to 233)	138.6 (106.2 to 191.3)	226.6 (174 to 288.9)	-39.2 (-51.3 to -23.7)	-55.1 (-67.2 to -33.8)	-31.5 (-47.7 to -11.1)
North Africa and Middle East	North Africa and Middle East	Kuwait	YLDs	All Ages	4,172 (2,898 to 5,818)	1,733 (1,228 to 2,411)	2,439 (1,686 to 3,449)	11,384 (8,166 to 15,348)	5,360 (3,860 to 7,278)	6,023 (4,240 to 8,130)	172.8 (146.5 to 207.3)	209.3 (172.2 to 253.3)	146.9 (121.6 to 179.4)
North Africa and Middle East	North Africa and Middle East	Kuwait	YLDs	Age-standardized	279.7 (202.5 to 375.9)	281.4 (206 to 374.9)	285.2 (203.5 to 385.5)	278.3 (201.4 to 376.1)	274 (198.1 to 369.8)	281.4 (201.3 to 379.3)	-0.5 (-8.7 to 7.9)	-2.6 (-12.4 to 8.6)	-1.4 (-10.2 to 8.5)
North Africa and Middle East	North Africa and Middle East	Lebanon	Incidence	All Ages	32,832 (27,629 to 39,786)	16,288 (13,826 to 19,704)	16,545 (13,563 to 20,271)	59,704 (51,948 to 69,189)	31,598 (27,763 to 36,037)	28,107 (24,160 to 33,080)	81.8 (68.2 to 96.4)	94 (77 to 111.7)	69.9 (56.5 to 85.7)
North Africa and Middle East	North Africa and Middle East	Lebanon	Incidence	Age-standardized	1008.1 (871.8 to 1178.4)	1023.8 (890.9 to 1196.4)	990.3 (846.7 to 1166.7)	1165.2 (1008.8 to 1352.9)	1190 (1038.5 to 1373.1)	1134.3 (975.1 to 1331.1)	15.6 (9.7 to 21.1)	16.2 (9.1 to 23.4)	14.5 (7.8 to 21.5)
North Africa and Middle East	North Africa and Middle East	Lebanon	Prevalence	All Ages	183,462 (159,818 to 212,853)	90,914 (79,911 to 105,479)	92,548 (79,528 to 108,826)	352,118 (313,588 to 399,702)	183,897 (163,411 to 208,628)	168,221 (148,615 to 191,842)	91.9 (79.3 to 106.2)	102.3 (87 to 119.5)	81.8 (69.5 to 97.7)
North Africa and Middle East	North Africa and Middle East	Lebanon	Prevalence	Age-standardized	6195.7 (5550.1 to 6968.9)	6199 (5509.8 to 6989.3)	6179.9 (5494.3 to 6992.3)	6840.3 (6054.3 to 7766.5)	6813.7 (6004.3 to 7780.3)	6859.9 (6063.6 to 7795.4)	10.4 (4.3 to 16.8)	9.9 (2.2 to 17.7)	11 (4.7 to 18.7)
North Africa and Middle East	North Africa and Middle East	Lebanon	Deaths	All Ages	695 (564 to 865)	302 (222 to 397)	393 (296 to 524)	1,185 (918 to 1,499)	474 (321 to 722)	711 (475 to 896)	70.6 (28.2 to 112.1)	56.9 (12 to 183.8)	81.1 (17.9 to 140.8)
North Africa and Middle East	North Africa and Middle East	Lebanon	Deaths	Age-standardized	37.8 (30.6 to 47.4)	31.6 (23.1 to 41.9)	44.9 (34 to 60.8)	23.3 (18.1 to 29.7)	16.9 (11.5 to 25.7)	31.5 (21 to 39.5)	-38.3 (-53.4 to -23.9)	-46.6 (-62 to -2.8)	-29.8 (-54.9 to -6.9)
North Africa and Middle East	North Africa and Middle East	Lebanon	DALYs	All Ages	25,423 (21,230 to 30,184)	11,656 (9,382 to 14,067)	13,767 (11,104 to 16,856)	44,044 (36,701 to 51,407)	20,342 (16,588 to 24,923)	23,702 (18,930 to 28,172)	73.2 (46.6 to 98)	74.5 (47.2 to 128.8)	72.2 (34.9 to 105.2)
North Africa and Middle East	North Africa and Middle East	Lebanon	DALYs	Age-standardized	1053 (886.8 to 1241.3)	942.3 (755.9 to 1135.9)	1169.8 (950.7 to 1447.9)	850.4 (708.1 to 992.9)	733 (596.6 to 902.5)	993.8 (797.1 to 1181.7)	-19.2 (-32.4 to -7.3)	-22.2 (-35.7 to 1.3)	-15 (-35.4 to 2.1)
North Africa and Middle East	North Africa and Middle East	Lebanon	YLLs	All Ages	15,002 (11,976 to 18,744)	6,493 (4,733 to 8,570)	8,509 (6,384 to 11,315)	20,753 (15,861 to 26,138)	8,144 (5,599 to 12,068)	12,609 (8,579 to 16,115)	38.3 (2.2 to 75.8)	25.4 (-10.1 to 118)	48.2 (-3 to 97.6)
North Africa and Middle East	North Africa and Middle East	Lebanon	YLLs	Age-standardized	681.7 (546.2 to 848.5)	575.8 (423.4 to 757)	793.3 (599.1 to 1066.3)	399.3 (305.8 to 502.6)	287 (197.6 to 425.4)	536.6 (365 to 684.4)	-41.4 (-56.8 to -26.3)	-50.2 (-64.5 to -14.9)	-32.4 (-55.9 to -9.6)
North Africa and Middle East	North Africa and Middle East	Lebanon	YLDs	All Ages	10,421 (7,700 to 13,655)	5,163 (3,825 to 6,768)	5,258 (3,859 to 6,963)	23,292 (17,942 to 28,838)	12,198 (9,370 to 15,166)	11,094 (8,472 to 13,887)	123.5 (104.3 to 145.7)	136.2 (114.5 to 164.3)	111 (91.3 to 134)
North Africa and Middle East	North Africa and Middle East	Lebanon	YLDs	Age-standardized	371.3 (280.3 to 473.5)	366.5 (275.6 to 467.2)	376.4 (282.7 to 481.7)	451 (347.4 to 559.6)	446 (341 to 555.3)	457.2 (349.6 to 569)	21.5 (13.6 to 30)	21.7 (11.8 to 33.4)	21.4 (12.5 to 31.7)
North Africa and Middle East	North Africa and Middle East	Libya	Incidence	All Ages	39,110 (31,554 to 48,049)	18,842 (15,274 to 23,184)	20,268 (16,368 to 25,202)	61,083 (53,615 to 69,479)	30,409 (26,879 to 34,795)	30,674 (26,739 to 34,972)	56.2 (40.5 to 75.4)	61.4 (43.4 to 82.8)	51.3 (35.6 to 70.2)
North Africa and Middle East	North Africa and Middle East	Libya	Incidence	Age-standardized	967.7 (845 to 1116.1)	981.1 (857.7 to 1127.5)	963.6 (834.4 to 1116.6)	1032.1 (904.7 to 1189.4)	1038.6 (909.5 to 1200.1)	1026.3 (886.8 to 1187)	6.6 (2.6 to 10.8)	5.9 (-0.1 to 11.5)	6.5 (1.4 to 12.5)
North Africa and Middle East	North Africa and Middle East	Libya	Prevalence	All Ages	202,103 (171,610 to 243,517)	94,307 (79,981 to 114,142)	107,796 (91,446 to 129,277)	364,029 (326,892 to 410,352)	174,090 (155,619 to 196,931)	189,938 (169,165 to 215,026)	80.1 (64.8 to 97)	84.6 (66.6 to 105.8)	76.2 (60.6 to 92.2)
North Africa and Middle East	North Africa and Middle East	Libya	Prevalence	Age-standardized	5780.9 (5226.5 to 6492.4)	5715.1 (5180.3 to 6394.1)	5886.9 (5302.9 to 6620.8)	5961.9 (5335.9 to 6756.3)	5744.4 (5154.5 to 6515.4)	6179.9 (5488.8 to 7024.9)	3.1 (-1.2 to 7.2)	0.5 (-4.5 to 6.3)	5 (-0.2 to 10.2)
North Africa and Middle East	North Africa and Middle East	Libya	Deaths	All Ages	559 (433 to 722)	221 (155 to 297)	338 (255 to 480)	1,173 (882 to 1,477)	455 (328 to 604)	718 (512 to 951)	109.9 (49.4 to 182.4)	106.1 (36.3 to 209.3)	112.3 (37 to 197)
North Africa and Middle East	North Africa and Middle East	Libya	Deaths	Age-standardized	33.3 (25.3 to 43.5)	26.7 (18.7 to 36.1)	39.8 (29.9 to 57.6)	26.6 (19.9 to 33.3)	20.6 (15.2 to 27.2)	32.6 (23.6 to 43)	-20.2 (-42.8 to 6.2)	-22.9 (-48.5 to 15)	-18.2 (-46.7 to 13.7)
North Africa and Middle East	North Africa and Middle East	Libya	DALYs	All Ages	23,961 (19,678 to 28,954)	10,426 (8,075 to 12,807)	13,534 (10,868 to 16,707)	48,710 (40,076 to 57,385)	21,086 (17,097 to 25,492)	27,624 (21,938 to 33,654)	103.3 (68.9 to 144.1)	102.2 (63 to 155)	104.1 (61.5 to 152.5)
North Africa and Middle East	North Africa and Middle East	Libya	DALYs	Age-standardized	967.4 (794.6 to 1158)	860 (669.6 to 1052)	1072.7 (864.1 to 1360.2)	882.1 (730.7 to 1036.1)	761.2 (622.3 to 917.3)	999.9 (795.2 to 1209.2)	-8.8 (-25.8 to 10.1)	-11.5 (-30.1 to 12.7)	-6.8 (-29.8 to 17.3)
North Africa and Middle East	North Africa and Middle East	Libya	YLLs	All Ages	13,177 (10,342 to 16,787)	5,390 (3,692 to 7,076)	7,787 (5,994 to 10,532)	24,941 (18,277 to 31,999)	9,778 (6,588 to 13,414)	15,163 (10,482 to 20,268)	89.3 (31.8 to 159.4)	81.4 (15.3 to 179)	94.7 (26.3 to 174.9)
North Africa and Middle East	North Africa and Middle East	Libya	YLLs	Age-standardized	631.2 (488.5 to 806)	529.9 (365.4 to 700.3)	728.5 (550.7 to 1003.2)	488.8 (359.2 to 619)	385.3 (267.3 to 520)	589.1 (417 to 785.9)	-22.6 (-45.3 to 5.7)	-27.3 (-53 to 11.2)	-19.1 (-48.9 to 15.5)
North Africa and Middle East	North Africa and Middle East	Libya	YLDs	All Ages	10,784 (7,788 to 14,599)	5,036 (3,668 to 6,829)	5,747 (4,143 to 7,783)	23,768 (18,222 to 29,592)	11,308 (8,669 to 14,141)	12,461 (9,490 to 15,684)	120.4 (97.5 to 147)	124.5 (99.9 to 154.8)	116.8 (92.8 to 144.8)
North Africa and Middle East	North Africa and Middle East	Libya	YLDs	Age-standardized	336.2 (253.4 to 428.4)	330.2 (249.8 to 421.8)	344.2 (258.9 to 440.5)	393.3 (302.6 to 488.8)	375.9 (289.8 to 466.5)	410.8 (316.4 to 513.9)	17 (10.6 to 24.4)	13.9 (6.9 to 22)	19.3 (11.3 to 28.2)
North Africa and Middle East	North Africa and Middle East	Morocco	Incidence	All Ages	177,896 (149,894 to 211,359)	90,451 (76,153 to 107,243)	87,445 (72,704 to 104,319)	296,906 (259,001 to 344,551)	149,546 (131,035 to 171,893)	147,360 (127,278 to 172,299)	66.9 (55 to 80.1)	65.3 (51.7 to 80)	68.5 (54.5 to 83)
North Africa and Middle East	North Africa and Middle East	Morocco	Incidence	Age-standardized	738.3 (644.7 to 842.5)	755.6 (656.1 to 863.2)	720.7 (626.3 to 824.8)	876.4 (764.2 to 1016.5)	877.2 (768.6 to 1009.5)	876.3 (759.4 to 1021.2)	18.7 (13.2 to 24.4)	16.1 (9.9 to 23)	21.6 (14.9 to 28.1)
North Africa and Middle East	North Africa and Middle East	Morocco	Prevalence	All Ages	874,867 (759,825 to 1,011,896)	447,103 (390,660 to 515,551)	427,764 (366,862 to 500,133)	1,595,027 (1,428,951 to 1,804,287)	785,871 (702,462 to 885,478)	809,156 (723,876 to 922,339)	82.3 (71 to 95)	75.8 (63.6 to 89.8)	89.2 (75.5 to 105.3)
[illegible]	[illegible]	[illegible]	[illegible]	[illegible]	[illegible]	[illegible]	[illegible]	[illegible]	[illegible]	[illegible]	[illegible]	[illegible]	[illegible]

Chronic respiratory diseases

7 GBD super region	21 GBD region	Location	Measure	Age	1990 Both	1990 Female	1990 Male	2019 Both	2019 Female	2019 Male	Percent change (1990 to 2019) Both	Percent change (1990 to 2019) Female	Percent change (1990 to 2019) Male
Sub-Saharan Africa	Central sub-Saharan Africa	Angola	Deaths	All Ages	2,794 (2,084 to 3,626)	1,447 (863 to 2,241)	1,347 (988 to 1,728)	[illegible]	[illegible]	[illegible]	[illegible]	[illegible]	[illegible]
Sub-Saharan Africa	Central sub-Saharan Africa	Angola	Deaths	Age-standardized	82.6 (60 to 118.9)	81.1 (47 to 148.1)	83.5 (61.9 to 104.2)	[illegible]	[illegible]	[illegible]	[illegible]	[illegible]	[illegible]
Sub-Saharan Africa	Central sub-Saharan Africa	Angola	DALYs	All Ages	128,386 (97,462 to 166,379)	70,554 (42,946 to 104,165)	57,832 (44,362 to 72,579)	[illegible]	[illegible]	[illegible]	[illegible]	[illegible]	[illegible]
Sub-Saharan Africa	Central sub-Saharan Africa	Angola	DALYs	Age-standardized	2194.1 (1705.3 to 2779.6)	2243.5 (1507.4 to 3340.7)	2141.4 (1668.5 to 2637.3)	[illegible]	[illegible]	[illegible]	[illegible]	[illegible]	[illegible]
Sub-Saharan Africa	Central sub-Saharan Africa	Angola	YLLs	All Ages	97,421 (69,682 to 135,475)	53,396 (27,068 to 87,162)	44,026 (31,191 to 57,993)	[illegible]	[illegible]	[illegible]	[illegible]	[illegible]	[illegible]
Sub-Saharan Africa	Central sub-Saharan Africa	Angola	YLLs	Age-standardized	1796.4 (1335.1 to 2374.7)	1800.7 (1055 to 2907.5)	1790.1 (1320.6 to 2286.6)	[illegible]	[illegible]	[illegible]	[illegible]	[illegible]	[illegible]
Sub-Saharan Africa	Central sub-Saharan Africa	Angola	YLDs	All Ages	30,965 (22,907 to 40,579)	17,158 (12,709 to 22,587)	13,807 (10,070 to 18,411)	[illegible]	[illegible]	[illegible]	[illegible]	[illegible]	[illegible]
Sub-Saharan Africa	Central sub-Saharan Africa	Angola	YLDs	Age-standardized	397.7 (308.6 to 488)	442.8 (345.7 to 543.7)	351.2 (270.9 to 439.4)	[illegible]	[illegible]	[illegible]	[illegible]	[illegible]	[illegible]
Sub-Saharan Africa	Central sub-Saharan Africa	Central African Republic	Incidence	All Ages	26,620 (22,053 to 32,285)	13,565 (11,146 to 16,292)	13,054 (10,535 to 16,225)	[illegible]	[illegible]	[illegible]	[illegible]	[illegible]	[illegible]
Sub-Saharan Africa	Central sub-Saharan Africa	Central African Republic	Incidence	Age-standardized	973 (856.1 to 1106.6)	974.2 (851.3 to 1105.6)	966.4 (839.1 to 1113.2)	[illegible]	[illegible]	[illegible]	[illegible]	[illegible]	[illegible]
Sub-Saharan Africa	Central sub-Saharan Africa	Central African Republic	Prevalence	All Ages	142,288 (124,764 to 163,675)	72,935 (63,382 to 84,344)	69,354 (60,027 to 80,828)	[illegible]	[illegible]	[illegible]	[illegible]	[illegible]	[illegible]
Sub-Saharan Africa	Central sub-Saharan Africa	Central African Republic	Prevalence	Age-standardized	6121.9 (5624.5 to 6687.5)	6011.1 (5488.2 to 6588.7)	6244.7 (5693.7 to 6872.4)	[illegible]	[illegible]	[illegible]	[illegible]	[illegible]	[illegible]
Sub-Saharan Africa	Central sub-Saharan Africa	Central African Republic	Deaths	All Ages	1,074 (766 to 1,444)	515 (293 to 823)	559 (393 to 721)	[illegible]	[illegible]	[illegible]	[illegible]	[illegible]	[illegible]
Sub-Saharan Africa	Central sub-Saharan Africa	Central African Republic	Deaths	Age-standardized	109.2 (73.9 to 161.9)	96.7 (51.5 to 174.7)	123.1 (86.4 to 162.9)	[illegible]	[illegible]	[illegible]	[illegible]	[illegible]	[illegible]
Sub-Saharan Africa	Central sub-Saharan Africa	Central African Republic	DALYs	All Ages	42,125 (32,682 to 52,085)	20,832 (13,669 to 27,704)	21,292 (16,027 to 26,738)	[illegible]	[illegible]	[illegible]	[illegible]	[illegible]	[illegible]
Sub-Saharan Africa	Central sub-Saharan Africa	Central African Republic	DALYs	Age-standardized	2805.5 (2101 to 3690.4)	2512 (1588.1 to 3817.7)	3143.5 (2336.3 to 3952.6)	[illegible]	[illegible]	[illegible]	[illegible]	[illegible]	[illegible]
Sub-Saharan Africa	Central sub-Saharan Africa	Central African Republic	YLLs	All Ages	33,771 (24,431 to 42,996)	16,389 (9,346 to 23,021)	17,382 (12,174 to 22,666)	[illegible]	[illegible]	[illegible]	[illegible]	[illegible]	[illegible]
Sub-Saharan Africa	Central sub-Saharan Africa	Central African Republic	YLLs	Age-standardized	2390.9 (1684.3 to 3251.9)	2087.6 (1181 to 3337.6)	2742.1 (1940.8 to 3526.7)	[illegible]	[illegible]	[illegible]	[illegible]	[illegible]	[illegible]
Sub-Saharan Africa	Central sub-Saharan Africa	Central African Republic	YLDs	All Ages	8,353 (6,296 to 10,724)	4,443 (3,382 to 5,726)	3,910 (2,901 to 5,057)	[illegible]	[illegible]	[illegible]	[illegible]	[illegible]	[illegible]
Sub-Saharan Africa	Central sub-Saharan Africa	Central African Republic	YLDs	Age-standardized	414.7 (324.9 to 501.4)	424.3 (334.2 to 515.7)	401.4 (312.8 to 493.7)	[illegible]	[illegible]	[illegible]	[illegible]	[illegible]	[illegible]
Sub-Saharan Africa	Central sub-Saharan Africa	Congo	Incidence	All Ages	24,032 (19,511 to 29,528)	11,194 (9,307 to 13,477)	12,838 (10,121 to 16,257)	[illegible]	[illegible]	[illegible]	[illegible]	[illegible]	[illegible]
Sub-Saharan Africa	Central sub-Saharan Africa	Congo	Incidence	Age-standardized	992.1 (864.9 to 1148.8)	936.2 (820.9 to 1068.6)	1045.7 (896.7 to 1231.1)	[illegible]	[illegible]	[illegible]	[illegible]	[illegible]	[illegible]
Sub-Saharan Africa	Central sub-Saharan Africa	Congo	Prevalence	All Ages	129,688 (112,531 to 152,112)	60,282 (52,905 to 69,566)	69,406 (58,637 to 83,098)	[illegible]	[illegible]	[illegible]	[illegible]	[illegible]	[illegible]
[illegible]	[illegible]	[illegible]	[illegible]	[illegible]	[illegible]	[illegible]	[illegible]	[illegible]	[illegible]	[illegible]	[illegible]	[illegible]	[illegible]

Chronic respiratory diseases

7 GBD super region	21 GBD region	Location	Measure	Age	1990 Both	1990 Female	1990 Male	2019 Both	2019 Female	2019 Male	Percent change (1990 to 2019) Both	Percent change (1990 to 2019) Female	Percent change (1990 to 2019) Male
Sub-Saharan Africa	Southern Sub-Saharan Africa	Botswana	Prevalence	Age-standardized	4603.5 (4278.0 to 4981.2)	3965.4 (3650.5 to 4317.4)	5484.1 (5112.5 to 5916.1)	4689 (4270.6 to 5173.5)	4659.2 (4241.6 to 5161.5)	4915.4 (4461.4 to 5444.1)	1.9 (-3.8 to 10.1)	17.5 (10.7 to 26.8)	-10.4 (-16.8 to -1.7)
[illegible]	[illegible]	[illegible]	[illegible]	[illegible]	[illegible]	[illegible]	[illegible]	[illegible]	[illegible]	[illegible]	[illegible]	[illegible]	[illegible]

Chronic respiratory diseases

7 GBD super region	21 GBD region	Location	Measure	Age	1990			2019			Percent change (1990 to 2019)		
					Both	Female	Male	Both	Female	Male	Both	Female	Male
Sub-Saharan Africa	Western sub-Saharan Africa	Senegal	Prevalence	All Ages	251,275 (218,490 to 297,236)	142,043 (123,902 to 167,738)	109,232 (93,734 to 129,778)	499,548 (431,723 to 588,052)	279,885 (242,945 to 330,965)	219,663 (185,333 to 263,161)	98.8 (86.8 to 109.6)	97 (83.3 to 110.5)	101.1 (85.5 to 117.4)
Sub-Saharan Africa	Western sub-Saharan Africa	Senegal	Prevalence	Age-standardized	4148.9 (3808.9 to 4558.4)	4556.6 (4159.6 to 5026.2)	3723.2 (3423.2 to 4133.1)	3981.5 (3617.6 to 4441.9)	4440.9 (4008 to 4963.4)	3504.5 (3161.6 to 3909.7)	-4 (-8.3 to -0.2)	-2.5 (-7.8 to 2.7)	-5.9 (-11 to -0.7)
Sub-Saharan Africa	Western sub-Saharan Africa	Senegal	Deaths	All Ages	1,950 (1,499 to 2,346)	741 (541 to 937)	1,209 (891 to 1,500)	2,763 (2,158 to 3,433)	1,181 (903 to 1,531)	1,582 (1,204 to 2,037)	41.7 (8.3 to 88.8)	59.3 (16.2 to 117.5)	30.9 (-1.8 to 85.2)
Sub-Saharan Africa	Western sub-Saharan Africa	Senegal	Deaths	Age-standardized	65.8 (50.9 to 79.1)	49.1 (36.4 to 61.7)	83.3 (61.8 to 103.9)	41.8 (33.5 to 50.9)	33.8 (26.1 to 43.5)	51 (39.6 to 64.2)	-36.5 (-49.9 to -16)	-31.2 (-47.9 to -8.1)	-38.7 (-52.8 to -14.8)
Sub-Saharan Africa	Western sub-Saharan Africa	Senegal	DALYs	All Ages	72,390 (58,186 to 84,503)	31,293 (24,488 to 38,298)	41,097 (31,578 to 49,829)	106,791 (87,089 to 128,948)	49,830 (39,970 to 61,651)	56,961 (45,619 to 70,278)	47.5 (16.6 to 86.1)	59.2 (24.5 to 104.1)	38.6 (6.6 to 83.3)
Sub-Saharan Africa	Western sub-Saharan Africa	Senegal	DALYs	Age-standardized	1708.2 (1377.9 to 2007.5)	1393.6 (1093.2 to 1682.7)	2023.9 (1553.3 to 2465)	1158.6 (957.3 to 1377.9)	1026.9 (838.1 to 1253.6)	1306 (1053 to 1613.7)	-32.2 (-45.4 to -14.6)	-26.3 (-42.5 to -6.2)	-35.5 (-49.7 to -14.6)
Sub-Saharan Africa	Western sub-Saharan Africa	Senegal	YLLs	All Ages	55,815 (42,101 to 67,461)	22,224 (15,726 to 28,529)	33,591 (24,281 to 42,089)	70,250 (53,126 to 90,051)	29,885 (21,783 to 40,179)	40,365 (29,508 to 52,848)	25.9 (-9.8 to 72.6)	34.5 (-7.2 to 97.3)	20.2 (-15.2 to 73)
Sub-Saharan Africa	Western sub-Saharan Africa	Senegal	YLLs	Age-standardized	1393.4 (1062.8 to 1674.8)	1063 (774.6 to 1346)	1725.3 (1262.3 to 2165.7)	837.1 (647.5 to 1050.7)	684 (517.8 to 890.9)	1006.3 (765.1 to 1302.7)	-39.9 (-54.7 to -19.4)	-35.7 (-54.2 to -10.3)	-41.7 (-57 to -18.1)
Sub-Saharan Africa	Western sub-Saharan Africa	Senegal	YLDs	All Ages	16,575 (12,778 to 20,906)	9,070 (6,937 to 11,548)	7,506 (5,854 to 9,443)	36,541 (28,630 to 45,415)	19,945 (15,449 to 24,675)	16,596 (13,025 to 20,691)	120.5 (108.1 to 133.3)	119.9 (104 to 136.6)	121.1 (105.5 to 137.9)
Sub-Saharan Africa	Western sub-Saharan Africa	Senegal	YLDs	Age-standardized	314.8 (252.9 to 375.9)	330.6 (259.4 to 398.5)	298.6 (240.2 to 358.4)	321.5 (258 to 382.4)	342.9 (272.8 to 413.6)	299.7 (241.4 to 357.5)	2.1 (-2.4 to 6.3)	3.7 (-1.7 to 9.5)	0.4 (-5.2 to 5.8)
Sub-Saharan Africa	Western sub-Saharan Africa	Sierra Leone	Incidence	All Ages	29,798 (24,250 to 37,311)	14,348 (11,686 to 17,514)	15,450 (12,448 to 19,651)	60,061 (49,191 to 74,347)	30,729 (25,498 to 37,439)	29,332 (23,257 to 37,115)	101.6 (87 to 116.6)	114.2 (96.9 to 132)	89.8 (71.1 to 109.5)
Sub-Saharan Africa	Western sub-Saharan Africa	Sierra Leone	Incidence	Age-standardized	780.7 (677.3 to 905.9)	755.4 (652.9 to 872.6)	802.3 (690.5 to 953.9)	745 (646.2 to 866.9)	776.3 (680.9 to 896)	714.4 (611.8 to 849.3)	-4.6 (-9.5 to 0.7)	2.8 (-3.5 to 9.1)	-11 (-17.2 to -4)
Sub-Saharan Africa	Western sub-Saharan Africa	Sierra Leone	Prevalence	All Ages	151,671 (133,222 to 176,130)	75,245 (66,831 to 86,576)	76,426 (65,983 to 89,634)	309,017 (268,123 to 364,004)	164,500 (145,113 to 189,790)	144,517 (122,259 to 174,411)	103.7 (91.7 to 117.1)	118.6 (104.2 to 134.1)	89.1 (73.2 to 106.4)
Sub-Saharan Africa	Western sub-Saharan Africa	Sierra Leone	Prevalence	Age-standardized	4737 (4358.2 to 5209.5)	4750.3 (4377.4 to 5212.3)	4723.1 (4297 to 5265.5)	4518.6 (4141.3 to 4998.1)	4975.8 (4581.2 to 5467.2)	4068.7 (3646.9 to 4605.4)	-4.6 (-8.5 to -0.4)	4.7 (-0.4 to 9.9)	-13.9 (-18.7 to -8.3)
Sub-Saharan Africa	Western sub-Saharan Africa	Sierra Leone	Deaths	All Ages	1,186 (911 to 1,461)	449 (324 to 591)	738 (545 to 906)	1,479 (1,108 to 1,908)	674 (491 to 921)	805 (557 to 1,093)	24.7 (-5 to 66.9)	50.2 (10.6 to 109.9)	9.1 (-20.5 to 56.7)
Sub-Saharan Africa	Western sub-Saharan Africa	Sierra Leone	Deaths	Age-standardized	65.1 (50.5 to 79.2)	49.9 (36.3 to 65.9)	79.9 (59.5 to 97.7)	44.7 (34.4 to 56.6)	39.5 (29.1 to 53.8)	50.7 (35.4 to 67.6)	-31.3 (-45.9 to -10.1)	-21 (-41 to 8.7)	-36.5 (-53.3 to -11.7)
Sub-Saharan Africa	Western sub-Saharan Africa	Sierra Leone	DALYs	All Ages	41,777 (33,168 to 50,833)	17,643 (13,391 to 22,072)	24,134 (18,739 to 29,854)	64,602 (51,089 to 79,811)	31,456 (24,637 to 39,373)	33,146 (24,833 to 42,855)	54.6 (21.6 to 98.9)	78.3 (37.9 to 131.8)	37.3 (3.7 to 83.5)
Sub-Saharan Africa	Western sub-Saharan Africa	Sierra Leone	DALYs	Age-standardized	1729.1 (1381.1 to 2077.6)	1427.1 (1096.5 to 1770.1)	2019.4 (1570.6 to 2439.3)	1305.5 (1052.3 to 1608)	1253.5 (992.8 to 1589.7)	1364.9 (1028.8 to 1752.6)	-24.5 (-38.9 to -4.1)	-12.2 (-30.5 to 13.6)	-32.4 (-47.5 to -9.7)
Sub-Saharan Africa	Western sub-Saharan Africa	Sierra Leone	YLLs	All Ages	32,213 (23,767 to 40,999)	12,773 (8,763 to 17,150)	19,440 (14,140 to 25,195)	43,096 (31,262 to 57,793)	19,767 (13,817 to 27,411)	23,329 (15,565 to 32,797)	33.8 (-5 to 89.4)	54.8 (6.2 to 127)	20 (-17.2 to 75.3)
Sub-Saharan Africa	Western sub-Saharan Africa	Sierra Leone	YLLs	Age-standardized	1393.5 (1054.8 to 1725.2)	1083.9 (769.8 to 1433.9)	1691 (1254.2 to 2104.6)	950.1 (702.5 to 1241.8)	857.6 (612.3 to 1178.6)	1049 (721.3 to 1424.8)	-31.8 (-48.7 to -6.6)	-20.9 (-42.8 to 12)	-38 (-54.9 to -11.2)
Sub-Saharan Africa	Western sub-Saharan Africa	Sierra Leone	YLDs	All Ages	9,564 (7,322 to 11,941)	4,870 (3,762 to 6,167)	4,694 (3,558 to 5,967)	21,507 (16,639 to 26,642)	11,690 (9,178 to 14,367)	9,817 (7,516 to 12,533)	124.9 (113.3 to 137.8)	140 (123.5 to 156.7)	109.2 (94.5 to 125.8)
Sub-Saharan Africa	Western sub-Saharan Africa	Sierra Leone	YLDs	Age-standardized	335.6 (265.3 to 403.1)	343.2 (270.8 to 412.8)	328.4 (257.5 to 402.2)	355.4 (285.8 to 422.5)	395.9 (314.1 to 467.1)	315.9 (253.2 to 382)	5.9 (1.3 to 10.9)	15.4 (8.6 to 22)	-3.8 (-9.3 to 2.4)
Sub-Saharan Africa	Western sub-Saharan Africa	Togo	Incidence	All Ages	33,093 (26,104 to 42,116)	16,834 (13,480 to 21,285)	16,259 (12,463 to 21,273)	65,741 (53,036 to 82,082)	33,388 (27,295 to 41,346)	32,353 (25,373 to 41,274)	98.7 (83 to 115.1)	98.3 (78.5 to 115.1)	99 (78.5 to 119.1)
Sub-Saharan Africa	Western sub-Saharan Africa	Togo	Incidence	Age-standardized	898.8 (777.4 to 1053.7)	902.7 (778.5 to 1044.5)	892.5 (768.7 to 1054.1)	849.3 (724.4 to 1010.3)	853.1 (730.3 to 1009.2)	847.6 (719.5 to 1016.2)	-5.5 (-10.8 to 0.2)	-5.5 (-12.5 to 1)	-5 (-11.8 to 2.2)
Sub-Saharan Africa	Western sub-Saharan Africa	Togo	Prevalence	All Ages	171,530 (144,504 to 206,015)	90,969 (76,927 to 108,065)	80,561 (66,554 to 98,311)	358,586 (307,990 to 426,197)	188,880 (163,700 to 221,405)	169,706 (143,373 to 205,668)	109.1 (93.8 to 124.6)	107.6 (90.5 to 128.7)	110.7 (90.7 to 130.3)
Sub-Saharan Africa	Western sub-Saharan Africa	Togo	Prevalence	Age-standardized	5954.5 (5363.7 to 6630.5)	6229.7 (5556.6 to 6968.9)	5632.2 (5057.2 to 6342.4)	5411.6 (4865.5 to 6079)	5530.3 (4932.9 to 6237.6)	5257.3 (4705.4 to 5930.4)	-9.1 (-13.6 to -4.1)	-11.2 (-17.1 to -4.6)	-6.7 (-13 to -0.1)
Sub-Saharan Africa	Western sub-Saharan Africa	Togo	Deaths	All Ages	741 (591 to 881)	330 (246 to 425)	412 (314 to 500)	1,332 (985 to 1,729)	609 (440 to 889)	722 (499 to 972)	79.6 (33.1 to 138.2)	84.8 (35.3 to 157.1)	75.5 (21.3 to 141.4)
Sub-Saharan Africa	Western sub-Saharan Africa	Togo	Deaths	Age-standardized	65.7 (52 to 78.4)	54.1 (40.1 to 70.1)	80 (60.9 to 96.6)	43.2 (33.1 to 55.4)	34.2 (25.1 to 49.2)	57.9 (41.6 to 75.5)	-34.3 (-49.9 to -14.4)	-36.8 (-52.5 to -13.2)	-27.6 (-48.6 to -1.8)
Sub-Saharan Africa	Western sub-Saharan Africa	Togo	DALYs	All Ages	32,841 (27,567 to 38,497)	15,726 (12,773 to 19,208)	17,115 (13,926 to 20,276)	62,476 (50,227 to 76,320)	29,357 (23,322 to 37,702)	33,119 (25,239 to 42,074)	90.2 (56 to 131.4)	86.7 (52.5 to 131.4)	93.5 (50.7 to 139.8)
Sub-Saharan Africa	Western sub-Saharan Africa	Togo	DALYs	Age-standardized	1810 (1508.9 to 2107.1)	1597.5 (1278.8 to 1950.8)	2055.5 (1658.2 to 2424.5)	1305.4 (1067.3 to 1593.2)	1109.8 (888.5 to 1435.5)	1582.9 (1214 to 2022.1)	-27.9 (-41.4 to -11.6)	-30.5 (-43.7 to -12.6)	-23 (-41.4 to -1.4)
Sub-Saharan Africa	Western sub-Saharan Africa	Togo	YLLs	All Ages	22,778 (18,182 to 27,516)	10,319 (7,645 to 13,631)	12,459 (9,567 to 15,228)	38,575 (27,938 to 51,017)	16,630 (11,582 to 24,586)	21,944 (14,809 to 30,074)	69.4 (22.8 to 128.4)	61.2 (14.4 to 129.4)	76.1 (22.5 to 139.8)
Sub-Saharan Africa	Western sub-Saharan Africa	Togo	YLLs	Age-standardized	1400.7 (1111.2 to 1672.2)	1170.6 (867.2 to 1512.2)	1665.7 (1269.5 to 2020.2)	904.9 (670.3 to 1176.4)	705.5 (502.9 to 1038.1)	1186 (828.5 to 1584.8)	-35.4 (-52.3 to -14.1)	-39.7 (-56.2 to -15.3)	-28.8 (-50.8 to -1.7)
Sub-Saharan Africa	Western sub-Saharan Africa	Togo	YLDs	All Ages	10,063 (7,553 to 13,164)	5,407 (4,063 to 7,023)	4,656 (3,427 to 6,154)	23,901 (18,364 to 30,475)	12,726 (9,837 to 16,016)	11,175 (8,543 to 14,437)	137.5 (120.8 to 155)	135.4 (115.7 to 155.8)	140 (118.6 to 161.4)
Sub-Saharan Africa	Western sub-Saharan Africa	Togo	YLDs	Age-standardized	409.3 (323.3 to 501.2)	426.9 (330.6 to 521.2)	389.9 (304.9 to 480.2)	400.6 (321.4 to 485.4)	404.3 (320.6 to 491.7)	396.9 (316.3 to 486.4)	-2.1 (-6.7 to 2.4)	-5.3 (-10.7 to 0.4)	1.8 (-4.5 to 8.2)

Chronic obstructive pulmonary disease

7 GBD super region	21 GBD region	Location	Measure	Age	1990 Both	1990 Female	1990 Male	2019 Both	2019 Female	2019 Male	Percent change (1990 to 2019) Both	Percent change (1990 to 2019) Female	Percent change (1990 to 2019) Male
		Southeast Asia, East Asia, and Oceania	Incidence	All Ages	2,956,635 (2,778,457 to 3,123,394)	1,494,412 (1,412,512 to 1,569,987)	1,462,223 (1,367,401 to 1,553,337)	5,212,829 (4,791,717 to 5,679,522)	2,619,088 (2,402,229 to 2,848,276)	2,593,741 (2,376,101 to 2,831,987)	76.3 (68.1 to 84.9)	75.3 (66.9 to 84.5)	77.4 (69 to 86.2)
		Southeast Asia, East Asia, and Oceania	Incidence	Age-standardized	261.8 (246.5 to 275)	255.1 (241.2 to 266.9)	270.2 (253.3 to 284.9)	202.7 (187.3 to 218.7)	193.3 (178.6 to 208.4)	213.4 (197.4 to 230.3)	-22.6 (-25.6 to -19.3)	-24.2 (-27.3 to -20.8)	-21 (-24 to -17.9)
[illegible]	[illegible]	[illegible]	[illegible]	[illegible]	[illegible]	[illegible]	[illegible]	[illegible]	[illegible]	[illegible]	[illegible]	[illegible]	[illegible]

Chronic obstructive pulmonary disease

7 GBD super region	21 GBD region	Location	Measure	Age	1990			2019			Percent change (1990 to 2019)		
					Both	Female	Male	Both	Female	Male	Both	Female	Male
Southeast Asia, east Asia, and Oceania	Oceania	Fiji	YLDs	Age-standardized	90 (73.9 to 102.9)	95.7 (78.4 to 112.5)	84 (68 to 98.1)	64.3 (52.7 to 74.2)	65.7 (53.4 to 77.2)	63.8 (51.6 to 74.4)	-28.5 (-33.2 to -23.6)	-31.3 (-37.3 to -24.8)	-24 (-30.6 to -17.2)
Southeast Asia, east Asia, and Oceania	Oceania	Guam	Incidence	All Ages	85 (80 to 90)	36 (33 to 38)	49 (46 to 52)	167 (158 to 177)	82 (76 to 88)	85 (81 to 90)	96.3 (87.7 to 104.9)	127.8 (113.9 to 143.6)	73.3 (64.7 to 82.2)
Southeast Asia, east Asia, and Oceania	Oceania	Guam	Incidence	Age-standardized	127.4 (120.3 to 134.7)	107 (100 to 113.9)	150.5 (141.9 to 159.4)	91.7 (86.6 to 97.4)	83.6 (77.7 to 90)	103.2 (97.9 to 109.1)	-28 (-30.7 to -25.5)	-21.9 (-26 to -17.7)	-31.4 (-34.6 to -28.1)
Southeast Asia, east Asia, and Oceania	Oceania	Guam	Prevalence	All Ages	985 (920 to 1,049)	444 (408 to 479)	542 (502 to 579)	1,828 (1,724 to 1,945)	952 (889 to 1,018)	876 (824 to 932)	85.6 (78 to 94.5)	114.7 (101.8 to 129.5)	61.7 (53.7 to 70.2)
Southeast Asia, east Asia, and Oceania	Oceania	Guam	Prevalence	Age-standardized	1311.4 (1234.9 to 1389.4)	1203.2 (1123.2 to 1281.5)	1441.1 (1350.9 to 1536.5)	1010 (951 to 1074)	985.3 (919.5 to 1053.6)	1034.4 (970.9 to 1101.3)	-23 (-25.6 to -20)	-18.1 (-22 to -13.9)	-28.2 (-31.7 to -24.6)
Southeast Asia, east Asia, and Oceania	Oceania	Guam	Deaths	All Ages	21 (19 to 24)	7 (5 to 8)	15 (12 to 17)	38 (30 to 47)	14 (11 to 19)	24 (19 to 29)	77 (41 to 118.2)	105.3 (59.9 to 165.4)	63.8 (25.6 to 108.8)
Southeast Asia, east Asia, and Oceania	Oceania	Guam	Deaths	Age-standardized	44.8 (38.7 to 50.8)	28.9 (21.9 to 36.5)	64.1 (54.3 to 73.6)	20.4 (16.5 to 25.1)	13.4 (10.4 to 18)	29.9 (23.9 to 37.1)	-54.6 (-63.6 to -44.4)	-53.6 (-63.6 to -39.2)	-53.3 (-63.9 to -41.2)
Southeast Asia, east Asia, and Oceania	Oceania	Guam	DALYs	All Ages	576 (513 to 644)	194 (161 to 228)	382 (332 to 438)	859 (726 to 1,029)	337 (279 to 408)	522 (428 to 631)	49.1 (24 to 78.7)	73.3 (43.9 to 109.5)	36.7 (7.8 to 69.1)
Southeast Asia, east Asia, and Oceania	Oceania	Guam	DALYs	Age-standardized	839.4 (745.4 to 936.4)	570 (461.2 to 667.3)	1143.3 (999 to 1299.6)	464.1 (392.4 to 554.9)	348.1 (288.5 to 421.2)	599.4 (493.4 to 718.4)	-44.7 (-54 to -34.3)	-38.9 (-48.8 to -25.4)	-47.6 (-58.2 to -35.8)
Southeast Asia, east Asia, and Oceania	Oceania	Guam	YLLs	All Ages	479 (419 to 543)	148 (117 to 179)	331 (281 to 386)	681 (554 to 851)	240 (189 to 315)	441 (352 to 548)	42.2 (11.6 to 77.1)	62.5 (27.3 to 108.7)	33.2 (0 to 70.8)
Southeast Asia, east Asia, and Oceania	Oceania	Guam	YLLs	Age-standardized	713.4 (627.1 to 804.5)	447.2 (348.7 to 546.5)	1012.6 (866.1 to 1167.4)	365.6 (298.4 to 456.9)	246.9 (192.6 to 325.1)	504.2 (405 to 622.4)	-48.8 (-59.5 to -36.5)	-44.8 (-56.2 to -28.1)	-50.2 (-62 to -36.8)
Southeast Asia, east Asia, and Oceania	Oceania	Guam	YLDs	All Ages	98 (80 to 113)	47 (38 to 55)	51 (41 to 60)	178 (146 to 204)	97 (78 to 113)	81 (66 to 95)	82.7 (69.6 to 95.9)	107.4 (87.2 to 130.1)	59.8 (44.9 to 75.8)
Southeast Asia, east Asia, and Oceania	Oceania	Guam	YLDs	Age-standardized	125.9 (104 to 143.8)	122.8 (100.8 to 143.2)	130.6 (105.3 to 152.4)	98.5 (81 to 112.5)	101.3 (81.1 to 118)	95.2 (77.5 to 111.7)	-21.8 (-26.3 to -17.7)	-17.5 (-23.6 to -10.7)	-27.1 (-32.6 to -21.6)
Southeast Asia, east Asia, and Oceania	Oceania	Kiribati	Incidence	All Ages	39 (37 to 41)	15 (14 to 16)	24 (23 to 25)	66 (63 to 71)	28 (26 to 31)	38 (36 to 40)	69.7 (64.7 to 75)	85.8 (78.3 to 94.1)	59.4 (53.2 to 65.7)
Southeast Asia, east Asia, and Oceania	Oceania	Kiribati	Incidence	Age-standardized	97.8 (92.5 to 103.5)	68.7 (63.6 to 73.7)	139.3 (132.2 to 147.3)	99.8 (94.3 to 106.1)	75.9 (70.4 to 81.9)	137.2 (130.3 to 145.3)	2.1 (-0.7 to 5.2)	10.3 (5.8 to 15.2)	-1.6 (-5.1 to 1.9)
Southeast Asia, east Asia, and Oceania	Oceania	Kiribati	Prevalence	All Ages	463 (437 to 488)	202 (187 to 215)	262 (247 to 276)	796 (750 to 843)	363 (336 to 390)	433 (408 to 458)	71.8 (66.3 to 77.5)	79.9 (72.1 to 88.6)	65.6 (58.8 to 72.1)
Southeast Asia, east Asia, and Oceania	Oceania	Kiribati	Prevalence	Age-standardized	1021.7 (963.6 to 1078.8)	831 (771.9 to 889.5)	1267.4 (1195.6 to 1345.6)	1019.4 (959.2 to 1084.8)	870.6 (807.1 to 937.1)	1213.2 (1145.9 to 1290.9)	-0.2 (-3.2 to 2.9)	4.8 (0.6 to 9.5)	-4.3 (-8.5 to -0.2)
Southeast Asia, east Asia, and Oceania	Oceania	Kiribati	Deaths	All Ages	21 (17 to 26)	6 (5 to 9)	15 (12 to 19)	28 (21 to 36)	10 (7 to 13)	18 (14 to 24)	31.8 (-4 to 73)	51.4 (0.2 to 117.2)	23.2 (-15 to 70.3)
Southeast Asia, east Asia, and Oceania	Oceania	Kiribati	Deaths	Age-standardized	70.5 (57.2 to 84.8)	37.2 (26.5 to 57.9)	120.4 (96.3 to 147.7)	56.6 (43.7 to 69.5)	33.8 (23 to 47.2)	95 (71.6 to 119.6)	-19.7 (-40.2 to 2.4)	-9.1 (-39.6 to 29.3)	-21.1 (-44.7 to 5.8)
Southeast Asia, east Asia, and Oceania	Oceania	Kiribati	DALYs	All Ages	628 (503 to 759)	197 (150 to 249)	431 (338 to 541)	817 (633 to 1,023)	285 (204 to 377)	532 (404 to 689)	30.1 (-1.6 to 69.4)	44.7 (0.6 to 97.5)	23.5 (-12.1 to 69.7)
Southeast Asia, east Asia, and Oceania	Oceania	Kiribati	DALYs	Age-standardized	1597.5 (1293.1 to 1898.5)	879.6 (663.4 to 1197.2)	2556.8 (2047.2 to 3166.4)	1198.7 (938.9 to 1474.3)	742.4 (533.4 to 982.4)	1871.1 (1427.3 to 2386.8)	-25 (-43.2 to -4.1)	-15.6 (-40.9 to 14.8)	-26.8 (-48 to -2.9)
Southeast Asia, east Asia, and Oceania	Oceania	Kiribati	YLLs	All Ages	582 (458 to 710)	175 (129 to 227)	407 (315 to 518)	738 (558 to 942)	246 (167 to 338)	492 (363 to 648)	26.8 (-6.4 to 69.3)	40.4 (-8.8 to 100.9)	20.9 (-16.2 to 70)
Southeast Asia, east Asia, and Oceania	Oceania	Kiribati	YLLs	Age-standardized	1498.2 (1194.1 to 1801.7)	793.3 (580.5 to 1104)	2440.5 (1929.9 to 3043.2)	1099.2 (840.9 to 1376.2)	652.3 (444 to 888.8)	1759.5 (1326 to 2267.3)	-26.6 (-46 to -4.6)	-17.8 (-45 to 16.6)	-27.9 (-49.7 to -2.9)
Southeast Asia, east Asia, and Oceania	Oceania	Kiribati	YLDs	All Ages	46 (37 to 53)	21 (17 to 25)	24 (20 to 29)	79 (65 to 92)	39 (31 to 46)	41 (33 to 48)	72.5 (59.6 to 85.9)	79.5 (59.2 to 99.5)	66.3 (49.5 to 83.5)
Southeast Asia, east Asia, and Oceania	Oceania	Kiribati	YLDs	Age-standardized	99.3 (81.1 to 113.9)	86.2 (69.9 to 100.6)	116.2 (94.5 to 135.3)	99.5 (81.8 to 114)	90.1 (73.4 to 106.5)	111.6 (90.6 to 129.8)	0.2 (-5.5 to 6.2)	4.5 (-4.3 to 13.6)	-4 (-11.3 to 3.4)
Southeast Asia, east Asia, and Oceania	Oceania	Marshall Islands	Incidence	All Ages	37 (35 to 39)	15 (14 to 16)	22 (21 to 23)	51 (48 to 54)	19 (18 to 21)	32 (30 to 33)	38.2 (33.8 to 43.4)	31.1 (24.6 to 39.1)	42.9 (37.8 to 48.4)
Southeast Asia, east Asia, and Oceania	Oceania	Marshall Islands	Incidence	Age-standardized	198.3 (186.9 to 210.4)	148.1 (136.7 to 160.3)	255.5 (242 to 269.5)	157.8 (149.5 to 166.7)	118.1 (109.7 to 127.2)	194.3 (184.7 to 204.5)	-20.4 (-22.8 to -17.8)	-20.2 (-24.1 to -16.2)	-23.9 (-26.6 to -21.1)
Southeast Asia, east Asia, and Oceania	Oceania	Marshall Islands	Prevalence	All Ages	391 (373 to 408)	175 (165 to 184)	216 (206 to 226)	591 (562 to 619)	264 (249 to 281)	327 (310 to 344)	51.3 (47 to 55.5)	51.3 (44.8 to 58.2)	51.3 (46.1 to 56.7)
Southeast Asia, east Asia, and Oceania	Oceania	Marshall Islands	Prevalence	Age-standardized	1951.2 (1837.5 to 2070)	1690.9 (1577.6 to 1809.5)	2240 (2101.6 to 2381.3)	1581.6 (1494.2 to 1673.7)	1425.3 (1334.4 to 1523.5)	1724.4 (1627.8 to 1826.6)	-18.9 (-21.5 to -16.4)	-15.7 (-19.7 to -11.5)	-23 (-26.1 to -19.7)
Southeast Asia, east Asia, and Oceania	Oceania	Marshall Islands	Deaths	All Ages	14 (11 to 19)	5 (2 to 7)	9 (7 to 14)	17 (12 to 23)	7 (3 to 10)	10 (7 to 14)	22.8 (-13.7 to 59.8)	34.2 (-6.9 to 88)	16.2 (-19.3 to 54.8)
Southeast Asia, east Asia, and Oceania	Oceania	Marshall Islands	Deaths	Age-standardized	101.8 (78.5 to 138.5)	70.2 (26.5 to 96.6)	144.9 (107.8 to 235.9)	71.9 (53.3 to 95.1)	56.1 (24.6 to 81.8)	86 (63.9 to 118.5)	-29.4 (-48.3 to -11)	-20.1 (-43 to 10.3)	-40.6 (-59.1 to -22.2)
Southeast Asia, east Asia, and Oceania	Oceania	Marshall Islands	DALYs	All Ages	399 (321 to 536)	152 (66 to 203)	248 (189 to 399)	519 (388 to 682)	221 (106 to 311)	299 (219 to 413)	30.1 (-5.5 to 67.4)	45.4 (6.2 to 95.1)	20.7 (-14.7 to 56.9)
Southeast Asia, east Asia, and Oceania	Oceania	Marshall Islands	DALYs	Age-standardized	2214.4 (1761.1 to 2985.7)	1604.5 (704.6 to 2155.2)	2934.5 (2236.9 to 4694.7)	1512.2 (1150.8 to 1968.4)	1261.9 (615.6 to 1783.1)	1739.5 (1305.7 to 2338.8)	-31.7 (-50.1 to -13.6)	-21.4 (-41.1 to 5.7)	-40.7 (-57.9 to -23.3)
Southeast Asia, east Asia, and Oceania	Oceania	Marshall Islands	YLLs	All Ages	361 (283 to 497)	133 (45 to 184)	228 (169 to 378)	461 (333 to 624)	193 (79 to 286)	269 (188 to 381)	27.9 (-10.8 to 69.2)	44.8 (0 to 104.3)	18 (-19.9 to 57.9)
Southeast Asia, east Asia, and Oceania	Oceania	Marshall Islands	YLLs	Age-standardized	2027.6 (1576.1 to 2787.1)	1431.3 (497.9 to 1973.3)	2732.7 (2027.9 to 4481.7)	1361.1 (998.5 to 1823.9)	1116.3 (465.5 to 1637.2)	1583.7 (1153.9 to 2171.7)	-32.9 (-52.4 to -13.1)	-22 (-44.4 to 8.5)	-42 (-59.3 to -23.2)
Southeast Asia, east Asia, and Oceania	Oceania	Marshall Islands	YLDs	All Ages	39 (32 to 44)	19 (15 to 22)	20 (16 to 24)	58 (48 to 67)	28 (23 to 33)	30 (24 to 36)	50.6 (41.9 to 60.6)	50.2 (36.3 to 64.7)	50.9 (39.6 to 64.1)
Southeast Asia, east Asia, and Oceania	Oceania	Marshall Islands	YLDs	Age-standardized	186.8 (153.5 to 213.7)	173.2 (141.6 to 201.6)	201.8 (164.1 to 236.4)	151 (124 to 172.5)	145.6 (119.1 to 168.7)	155.8 (125.3 to 182.6)	-19.2 (-23 to -15.3)	-15.9 (-21.8 to -9.6)	-22.8 (-27.3 to -17.6)
Southeast Asia, east Asia, and Oceania	Oceania	Micronesia (Federated States of)	Incidence	All Ages	92 (86 to 98)	43 (40 to 46)	49 (46 to 52)	94 (89 to 100)	44 (41 to 47)	50 (47 to 53)	2.4 (-1.7 to 6.6)	3.1 (-2.3 to 9)	1.7 (-3.6 to 6.9)
Southeast Asia, east Asia, and Oceania	Oceania	Micronesia (Federated States of)	Incidence	Age-standardized	189 (177.4 to 201.8)	171.6 (158.8 to 186)	209.5 (197.7 to 221.5)	152.1 (143.6 to 160.9)	135 (126.1 to 145.3)	173.9 (165.3 to 183.8)	-19.5 (-22.4 to -16.7)	-21.3 (-25.3 to -17.2)	-17 (-20.3 to -13.5)
Southeast Asia, east Asia, and Oceania	Oceania	Micronesia (Federated States of)	Prevalence	All Ages	979 (930 to 1,035)	493 (464 to 526)	486 (462 to 511)	1,092 (1,030 to 1,156)	552 (516 to 591)	541 (510 to 571)	11.5 (7.1 to 15.8)	11.9 (6 to 18.4)	11.2 (6.4 to 16.3)
Southeast Asia, east Asia, and Oceania	Oceania	Micronesia (Federated States of)	Prevalence	Age-standardized	1887.9 (1776.3 to 2023.5)	1909.6 (1779.7 to 2061.7)	1856.2 (1744.2 to 1978)	1575.6 (1485.8 to 1669.6)	1544.1 (1444.9 to 1654.6)	1610.6 (1520.8 to 1711.9)	-16.5 (-20.3 to -13.2)	-19.1 (-23.4 to -14.6)	-13.2 (-17.2 to -9.1)
Southeast Asia, east Asia, and Oceania	Oceania	Micronesia (Federated States of)	Deaths	All Ages	42 (33 to 53)	16 (11 to 22)	26 (19 to 35)	38 (28 to 50)	17 (10 to 23)	22 (15 to 28)	-9.8 (-37.3 to 24.8)	2.5 (-38.2 to 49.1)	-17.4 (-45.8 to 19.7)
Southeast Asia, east Asia, and Oceania	Oceania	Micronesia (Federated States of)	Deaths	Age-standardized	111 (86.9 to 139.4)	81.1 (55.3 to 115.4)	148.6 (112.4 to 204.3)	74.9 (58.3 to 94.9)	59.7 (34.9 to 81.7)	96.4 (74.2 to 122.5)	-32.5 (-50.5 to -10.8)	-26.4 (-54.8 to 4.9)	-35.1 (-54.1 to -9.9)
Southeast Asia, east Asia, and Oceania	Oceania	Micronesia (Federated States of)	DALYs	All Ages	1,222 (972 to 1,505)	475 (322 to 639)	748 (565 to 979)	1,087 (786 to 1,402)	464 (278 to 642)	623 (443 to 823)	-11.1 (-37.4 to 21.5)	-2.2 (-36.9 to 39.4)	-16.7 (-45.2 to 18.7)
Southeast Asia, east Asia, and Oceania	Oceania	Micronesia (Federated States of)	DALYs	Age-standardized	2443.1 (1947 to 2998.2)	1865.5 (1292.3 to 2495.9)	3084.5 (2349.1 to 4046.4)	1587 (1200.2 to 2006.8)	1298.2 (807.7 to 1755.1)	1948.1 (1437.7 to 2495.9)	-35 (-53.4 to -13.1)	-30.4 (-54.7 to -2.5)	-36.8 (-56.4 to -11.9)
Southeast Asia, east Asia, and Oceania	Oceania	Micronesia (Federated States of)	YLLs	All Ages	1,125 (875 to 1,411)	422 (273 to 586)	702 (521 to 935)	979 (680 to 1,284)	407 (221 to 584)	572 (390 to 781)	-13 (-41.5 to 22.5)	-3.8 (-42.1 to 43.2)	-18.5 (-48.3 to 19.1)
Southeast Asia, east Asia, and Oceania	Oceania	Micronesia (Federated States of)	YLLs	Age-standardized	2259.7 (1768.4 to 2816.1)	1669.7 (1100.4 to 2301.5)	2915.2 (2165.1 to 3871.6)	1434.5 (1045.7 to 1854.4)	1140.6 (647 to 1606.7)	1801.5 (1292.6 to 2354.8)	-36.5 (-56.1 to -12.8)	-31.7 (-58.3 to -0.2)	-38.2 (-58.2 to -11.7)
Southeast Asia, east Asia, and Oceania	Oceania	Micronesia (Federated States of)	YLDs	All Ages	97 (80 to 112)	52 (42 to 61)	45 (36 to 54)	108 (88 to 125)	58 (47 to 67)	50 (40 to 59)	10.8 (3.7 to 18)	10.8 (0.9 to 20.9)	10.8 (1.4 to 20.8)
Southeast Asia, east Asia, and Oceania	Oceania	Micronesia (Federated States of)	YLDs	Age-standardized	183.4 (150.3 to 210.4)	195.9 (160.3 to 228.3)	169.3 (136.7 to 198.5)	152.5 (124.7 to 175.2)	157.6 (128.6 to 183.5)	146.6 (118.9 to 171.7)	-16.8 (-21.2 to -12.5)	-19.5 (-25.7 to -13.5)	-13.4 (-19.1 to -7.2)
Southeast Asia, east Asia, and Oceania	Oceania	Nauru	Incidence	All Ages	12 (12 to 13)	6 (6 to 6)	7 (6 to 7)	13 (12 to 13)	7 (6 to 7)	6 (6 to 6)	2.8 (-0.8 to 6)	15.4 (10.2 to 21)	-8.3 (-12.3 to -4.3)
Southeast Asia, east Asia, and Oceania	Oceania	Nauru	Incidence	Age-standardized	284.1 (272 to 297.2)	271.9 (256.9 to 288.3)	295.7 (284 to 307.4)	272.4 (260.4 to 285.2)	262.9 (247.6 to 279)	283.2 (272.5 to 294)	-4.2 (-6.9 to -1.5)	-3.3 (-7.2 to 0.7)	-4.2 (-7.6 to -0.9)
Southeast Asia, east Asia, and Oceania	Oceania	Nauru	Prevalence	All Ages	134 (130 to 139)	66 (64 to 69)	68 (66 to 70)	142 (136 to 148)	78 (74 to 82)	64 (61 to 67)	5.8 (2 to 9.2)	17.3 (12 to 21.8)	-5.3 (-9.4 to -1.1)
Southeast Asia, east Asia, and Oceania	Oceania	Nauru	Prevalence	Age-standardized	3206.2 (3054.9 to 3357.1)	3384.6 (3201.1 to 3573.8)	3036.5 (2895.2 to 3182.1)	3076.7 (2925 to 3238)	3269.3 (3085.7 to 3467)	2866.1 (2720.9 to 3011.5)	-4 (-7.6 to -0.9)	-3.4 (-7.8 to 0.9)	-5.6 (-10 to -1.2)
Southeast Asia, east Asia, and Oceania	Oceania	Nauru	Deaths	All Ages	3 (2 to 4)	1 (1 to 1)	2 (1 to 3)	2 (1 to 3)	1 (0 to 1)	1 (1 to 2)	-34.7 (-50.3 to -13.5)	-22.7 (-53.5 to 15.5)	-40.8 (-57.6 to -15.5)
Southeast Asia, east Asia, and Oceania	Oceania	Nauru	Deaths	Age-standardized	105.1 (79.2 to 129.5)	71.3 (48.9 to 92.1)	136.4 (102.1 to 174.9)	71.7 (55.1 to 87.5)	50.9 (27.2 to 69.5)	92.4 (69.9 to 121.3)	-31.8 (-46.4 to -12.1)	-28.6 (-56.8 to 1.9)	-32.3 (-49.3 to -7.2)
Southeast Asia, east Asia, and Oceania	Oceania	Nauru	DALYs	All Ages	100 (73 to 127)	37 (25 to 48)	63 (45 to 86)	74 (56 to 92)	32 (20 to 44)	42 (31 to 57)	-26 (-41.1 to -6)	-13.1 (-37.5 to 20.8)	-33.5 (-51.2 to -7)
Southeast Asia, east Asia, and Oceania	Oceania	Nauru	DALYs	Age-standardized	2372.3 (1793.1 to 2925.8)	1771.1 (1285.7 to 2224.3)	2932.4 (2179.8 to 3776.6)	1645 (1301.6 to 1972.1)	1314.1 (840.8 to 1710.3)	2011.3 (1532.3 to 2590.5)	-30.7 (-44.2 to -12.3)	-25.8 (-47.6 to -0.3)	-31.4 (-47.9 to -7.1)
Southeast Asia, east Asia, and Oceania	Oceania	Nauru	YLLs	All Ages	87 (60 to 114)	30 (18 to 41)	57 (39 to 80)	60 (42 to 77)	24 (12 to 35)	36 (25 to 51)	-30.9 (-48 to -8)	-20.1 (-51.4 to 21.5)	-36.6 (-55.8 to -7.3)
Southeast Asia, east Asia, and Oceania	Oceania	Nauru	YLLs	Age-standardized	2063.6 (1478.4 to 2625.1)	1427.3 (944.7 to 1875.3)	2656.8 (1906.6 to 3511.1)	1349.8 (998.7 to 1672.4)	985.1 (507 to 1380.3)	1753.6 (1282.8 to 2346.3)	-34.6 (-49.5 to -14)	-31 (-58.6 to 1.6)	-34 (-52 to -7.1)
Southeast Asia, east Asia, and Oceania	Oceania	Nauru	YLDs	All Ages	13 (11 to 15)	7 (6 to 8)	6 (5 to 7)	14 (12 to 16)	8 (7 to 10)	6 (5 to 7)	6.2 (0.5 to 11.9)	16.7 (8.6 to 25.3)	-5.5 (-12.7 to 2.4)
Southeast Asia, east Asia, and Oceania	Oceania	Nauru	YLDs	Age-standardized	308.7 (256.1 to 354.1)	343.8 (281.8 to 401.2)	275.6 (221.8 to 322.5)	295.2 (245 to 337.5)	329 (270.4 to 381.9)	257.8 (210.9 to 299.8)	-4.4 (-8.4 to -0.2)	-4.3 (-9.5 to 1.1)	-6.5 (-11.7 to -0.7)
Southeast Asia, east Asia, and Oceania	Oceania	Niue	Incidence	All Ages	6 (5 to 6)	3 (3 to 3)	3 (3 to 3)	5 (5 to 6)	3 (2 to 3)	2 (2 to 3)	-4.2 (-7.1 to -1.3)	-6.3 (-10.4 to -2.3)	-1.5 (-5.6 to 2.7)
Southeast Asia, east Asia, and Oceania	Oceania	Niue	Incidence	Age-standardized	257.4 (244.7 to 269.4)	252.7 (238 to 268.2)	266.9 (255.2 to 277.2)	258.3 (247.1 to 271.3)	255.7 (240.4 to 272.4)	263.8 (253.7 to 276.1)	0.4 (-2.7 to 3.6)	1.2 (-2.7 to 5)	-1.2 (-5.1 to 3)
Southeast Asia, east Asia, and Oceania	Oceania	Niue	Prevalence	All Ages	70 (66 to 74)	42 (40 to 45)	28 (26 to 29)	67 (63 to 70)	39 (36 to 41)	28 (26 to 30)	-4.7 (-8.1 to -1.4)	-8.3 (-12.2 to -4.3)	0.8 (-4.1 to 5.6)
Southeast Asia, east Asia, and Oceania	Oceania	Niue	Prevalence	Age-standardized	3161 (3001.3 to 3330.6)	3304.2 (3125.9 to 3497.1)	2947.4 (2792.1 to 3094.2)	3191.1 (3032.2 to 3371.9)	3371.8 (3172.6 to 3588)	2953.4 (2800.4 to 3132.2)	1 (-2.5 to 4.3)	2 (-1.9 to 6.1)	0.2 (-4.4 to 5.1)
Southeast Asia, east Asia, and Oceania	Oceania	Niue	Deaths	All Ages	2 (1 to 2)	1 (0 to 1)	1 (1 to 1)	1 (1 to 1)	0 (0 to 1)	1 (0 to 1)	-42.8 (-55 to -28.9)	-48.2 (-65.3 to -29.9)	-37.9 (-52.5 to -21.5)
Southeast Asia, east Asia, and Oceania	Oceania	Niue	Deaths	Age-standardized	66.9 (53 to 82)	49.4 (30.9 to 66.2)	97.3 (76.7 to 125.4)	43 (33.3 to 53.3)	31.4 (18.1 to 44.6)	60.5 (49 to 77)	-35.7 (-49.3 to -19.6)	-36.5 (-57.1 to -13.1)	-37.8 (-52.5 to -21.5)
Southeast Asia, east Asia, and Oceania	Oceania	Niue	DALYs	All Ages	35 (29 to 42)	16 (12 to 20)	19 (15 to 23)	22 (18 to 27)	10 (8 to 13)	12 (10 to 15)	-35.4 (-47.2 to -22.6)	-35.9 (-49.9 to -18.8)	-35.1 (-49.4 to -20.6)
Southeast Asia, east Asia, and Oceania	Oceania	Niue	DALYs	Age-standardized	1573.1 (1295.8 to 1878.9)	1258.1 (915 to 1592.3)	2025.4 (1613.7 to 2544.6)	1068.4 (871.2 to 1272.9)	902 (652.7 to 1157.1)	1297.5 (1059.8 to 1619.1)	-32.1 (-44.6 to -18.4)	-28.3 (-43.9 to -8.2)	-35.9 (-50 to -22)
Southeast Asia, east Asia, and Oceania	Oceania	Niue	YLLs	All Ages	28 (22 to 35)	12 (8 to 16)	16 (12 to 21)	16 (12 to 20)	7 (4 to 9)	10 (7 to 12)	-42.5 (-56 to -27)	-45.2 (-63.2 to -23.6)	-40.5 (-55.8 to -24.4)
Southeast Asia, east Asia, and Oceania	Oceania	Niue	YLLs	Age-standardized	1266.7 (990.8 to 1583.2)	922.4 (580.2 to 1248)	1758.6 (1356.8 to 2289.8)	763.9 (578.7 to 965.5)	564.8 (322 to 811.6)	1033.7 (808.9 to 1343.3)	-39.7 (-54.1 to -22.8)	-38.8 (-59 to -12.7)	-41.2 (-56.3 to -25.7)
Southeast Asia, east Asia, and Oceania	Oceania	Niue	YLDs	All Ages	7 (6 to 8)	4 (3 to 5)	3 (2 to 3)	6 (5 to 7)	4 (3 to 4)	2 (2 to 3)	-6.3 (-10.5 to -2.1)	-9.6 (-14.1 to -4.5)	-0.7 (-7 to 5.8)
Southeast Asia, east Asia, and Oceania	Oceania	Niue	YLDs	Age-standardized	306.5 (250.7 to 350.8)	335.7 (274.6 to 390)	266.8 (214.4 to 311.3)	304.5 (251 to 346.9)	337.2 (275.7 to 389)	263.8 (213.8 to 307.6)	-0.6 (-4.8 to 3.6)	0.5 (-4.7 to 5.8)	-1.1 (-7.1 to 5)
Southeast Asia, east Asia, and Oceania	Oceania	Northern Mariana Islands	Incidence	All Ages	24 (23 to 26)	10 (10 to 11)	14 (13 to 15)	44 (41 to 47)	19 (18 to 21)	25 (24 to 27)	81 (70.9 to 91.5)	87.4 (74.8 to 101.3)	76.5 (65.6 to 88.1)
Southeast Asia, east Asia, and Oceania	Oceania	Northern Mariana Islands	Incidence	Age-standardized	153.2 (144.6 to 162)	145.8 (135.9 to 155.6)	156.6 (148 to 165.9)	114.4 (107.8 to 120.8)	101.3 (94.5 to 109)	127 (119.7 to 134)	-25.3 (-28 to -22.5)	-30.5 (-34.1 to -26.6)	-18.9 (-22.5 to -15.4)
Southeast Asia, east Asia, and Oceania	Oceania	Northern Mariana Islands	Prevalence	All Ages	333 (309 to 358)	141 (129 to 153)	193 (178 to 208)	470 (440 to 501)	210 (196 to 225)	259 (242 to 276)	40.8 (32.4 to 48.9)	49.3 (38.1 to 61)	34.7 (25.8 to 43.8)
Southeast Asia, east Asia, and Oceania	Oceania	Northern Mariana Islands	Prevalence	Age-standardized	1608.8 (1514.8 to 1709.4)	1579.8 (1476.9 to 1685.9)	1614.7 (1511.3 to 1725.1)	1167.6 (1096.8 to 1238.3)	1070.9 (997 to 1146)	1258.5 (1182.4 to 1337)	-27.4 (-30.1 to -24.7)	-32.2 (-35.9 to -28.6)	-22.1 (-25.9 to -18.1)
Southeast Asia, east Asia, and Oceania	Oceania	Northern Mariana Islands	Deaths	All Ages	7 (6 to 9)	2 (1 to 3)	5 (4 to 6)	13 (11 to 16)	4 (3 to 5)	9 (8 to 11)	83.7 (49 to 143)	95 (39.5 to 184.2)	79.4 (44.9 to 135.6)
Southeast Asia, east Asia, and Oceania	Oceania	Northern Mariana Islands	Deaths	Age-standardized	63 (53.3 to 72.8)	39.3 (29 to 49.5)	85.1 (71.9 to 99.2)	38.3 (32.9 to 45.9)	22.1 (17.2 to 29.5)	56.6 (48.7 to 67.8)	-39.3 (-49.1 to -21.7)	-43.9 (-59.5 to -19.3)	-33.5 (-46.1 to -16.5)
Southeast Asia, east Asia, and Oceania	Oceania	Northern Mariana Islands	DALYs	All Ages	243 (199 to 292)	73 (54 to 91)	170 (141 to 206)	338 (291 to 402)	107 (87 to 137)	231 (197 to 278)	39.2 (11.8 to 81.8)	46.6 (10.5 to 98.3)	36 (7.2 to 78.9)
Southeast Asia, east Asia, and Oceania	Oceania	Northern Mariana Islands	DALYs	Age-standardized	1282.3 (1099.4 to 1484)	874.6 (675.1 to 1074.3)	1619.3 (1405.8 to 1886.7)	756.1 (658.3 to 887.9)	482.4 (399.6 to 614.3)	1041.1 (912.4 to 1231.5)	-41 (-50.8 to -25.4)	-44.8 (-57.7 to -26.4)	-35.7 (-46.2 to -19.3)
Southeast Asia, east Asia, and Oceania	Oceania	Northern Mariana Islands	YLLs	All Ages	209 (167 to 258)	58 (40 to 76)	152 (122 to 187)	292 (244 to 359)	85 (66 to 116)	207 (173 to 253)	39.5 (9 to 90.4)	47.5 (2.5 to 118.1)	36.5 (5.4 to 83.7)
Southeast Asia, east Asia, and Oceania	Oceania	Northern Mariana Islands	YLLs	Age-standardized	1127 (952.4 to 1334.6)	713.8 (522.9 to 908)	1470.4 (1251.5 to 1736.6)	643.2 (548.5 to 786.1)	373.2 (292.6 to 506.5)	925.2 (789.2 to 1119.8)	-42.9 (-53.7 to -25.2)	-47.7 (-62.3 to -24.4)	-37.1 (-48.6 to -18.9)
Southeast Asia, east Asia, and Oceania	Oceania	Northern Mariana Islands	YLDs	All Ages	34 (27 to 39)	15 (12 to 18)	18 (15 to 22)	46 (38 to 53)	22 (18 to 25)	24 (20 to 28)	37.1 (25.3 to 50)	43.2 (25.3 to 62.1)	32 (17.1 to 49.5)
Southeast Asia, east Asia, and Oceania	Oceania	Northern Mariana Islands	YLDs	Age-standardized	155.3 (127.7 to 177.7)	160.8 (130.9 to 186.5)	148.9 (121.9 to 174.6)	112.9 (93.4 to 128.1)	109.2 (89.1 to 126.5)	115.9 (95.4 to 134.9)	-27.3 (-31.1 to -23.6)	-32.1 (-37.2 to -26.9)	-22.2 (-27.8 to -16.5)
Southeast Asia, east Asia, and Oceania	Oceania	Palau	Incidence	All Ages	29 (27 to 30)	14 (13 to 15)	15 (14 to 15)	54 (51 to 56)	26 (24 to 28)	27 (26 to 29)	87 (80.3 to 93.8)	85 (75.6 to 94.9)	88.9 (80.7 to 97.8)
Southeast Asia, east Asia, and Oceania	Oceania	Palau	Incidence	Age-standardized	273.4 (260.7 to 285)	267.6 (252.1 to 283)	280.9 (269.1 to 292.1)	260.4 (249.3 to 271.9)	256.9 (242 to 272.2)	265.5 (255.5 to 275.8)	-4.8 (-7.6 to -1.9)	-4 (-8.3 to 0.4)	-5.5 (-9.1 to -1.9)
Southeast Asia, east Asia, and Oceania	Oceania	Palau	Prevalence	All Ages	336 (321 to 349)	176 (167 to 185)	160 (153 to 166)	632 (601 to 664)	330 (311 to 349)	302 (286 to 319)	88.2 (80.1 to 95.3)	87.2 (78 to 96.1)	89.3 (79.4 to 99.5)
Southeast Asia, east Asia, and Oceania	Oceania	Palau	Prevalence	Age-standardized	3339 (3172.5 to 3489.5)	3504.3 (3317.4 to 3689)	3150 (2996.9 to 3297.2)	3186.1 (3040.7 to 3344)	3379.8 (3193.5 to 3577.5)	2971 (2821.3 to 3124.8)	-4.6 (-8.2 to -1.3)	-3.6 (-7.9 to 0.9)	-5.7 (-10 to -1.1)
Southeast Asia, east Asia, and Oceania	Oceania	Palau	Deaths	All Ages	7 (6 to 10)	4 (3 to 5)	4 (3 to 5)	10 (8 to 13)	5 (4 to 6)	5 (4 to 6)	37.2 (-2.8 to 81)	33.4 (-15.6 to 91.4)	41.2 (-1.1 to 90.9)
Southeast Asia, east Asia, and Oceania	Oceania	Palau	Deaths	Age-standardized	99.2 (78.7 to 127.8)	95 (68 to 136.7)	106.4 (86.5 to 138.2)	71 (57.1 to 85.6)	66.3 (49.7 to 82.7)	77.4 (62.9 to 94.7)	-28.4 (-48.7 to -6.5)	-30.3 (-55.5 to 0)	-27.3 (-48 to -4.2)
Southeast Asia, east Asia, and Oceania	Oceania	Palau	DALYs	All Ages	199 (162 to 248)	103 (78 to 140)	96 (78 to 122)	285 (236 to 344)	143 (113 to 176)	142 (116 to 175)	43.2 (7.6 to 82.4)	38.2 (-4.1 to 86)	48.6 (9 to 93.2)
Southeast Asia, east Asia, and Oceania	Oceania	Palau	DALYs	Age-standardized	2075.2 (1693.6 to 2580.4)	2090.5 (1580.3 to 2796)	2082.4 (1720.7 to 2621.9)	1500.4 (1252.8 to 1786.4)	1496.3 (1191.4 to 1813.2)	1521.5 (1267.7 to 1843.8)	-27.7 (-45.9 to -9.1)	-28.4 (-50.4 to -3.9)	-26.9 (-45.9 to -5.8)
Southeast Asia, east Asia, and Oceania	Oceania	Palau	YLLs	All Ages	166 (129 to 215)	85 (61 to 121)	81 (63 to 107)	224 (176 to 283)	109 (80 to 143)	115 (88 to 147)	34.9 (-5.8 to 82)	28.5 (-17.7 to 86.1)	41.6 (-1.6 to 94.3)
Southeast Asia, east Asia, and Oceania	Oceania	Palau	YLLs	Age-standardized	1751 (1373.2 to 2241.7)	1731.8 (1247.4 to 2448.3)	1795.9 (1425.4 to 2336.1)	1195.1 (949.9 to 1487.6)	1154.7 (852.8 to 1478.1)	1254.2 (998.3 to 1577.9)	-31.8 (-52.2 to -9.8)	-33.3 (-57.3 to -3.3)	-30.2 (-51.4 to -5.5)
Southeast Asia, east Asia, and Oceania	Oceania	Palau	YLDs	All Ages	33 (27 to 38)	18 (15 to 21)	15 (12 to 17)	61 (50 to 70)	34 (27 to 39)	28 (22 to 32)	85.1 (75.4 to 94.5)	83.6 (71.6 to 95.3)	87.1 (74.4 to 100.8)
Southeast Asia, east Asia, and Oceania	Oceania	Palau	YLDs	Age-standardized	324.1 (265.3 to 370.1)	358.7 (292.5 to 415.1)	286.5 (231.6 to 334.4)	305.3 (252.1 to 348.2)	341.6 (280.1 to 396.2)	267.3 (218 to 312.1)	-5.8 (-9.8 to -1.8)	-4.8 (-9.9 to 0.7)	-6.7 (-12 to -1.1)
Southeast Asia, east Asia, and Oceania	Oceania	Papua New Guinea	Incidence	All Ages	6,591 (6,471 to 6,685)	3,118 (3,034 to 3,182)	3,473 (3,413 to 3,514)	16,672 (16,277 to 17,002)	7,731 (7,479 to 7,942)	8,940 (8,739 to 9,096)	153 (148 to 157)	148 (141.3 to 153.6)	157.4 (151.7 to 161.7)
Southeast Asia, east Asia, and Oceania	Oceania	Papua New Guinea	Incidence	Age-standardized	343.5 (337.5 to 348.6)	331.5 (322.3 to 338.8)	357.2 (351.3 to 361.4)	341 (334.1 to 346.8)	327.2 (316.3 to 335.7)	355.6 (348.9 to 360.7)	-0.7 (-2.2 to 0.5)	-1.3 (-3.7 to 0.9)	-0.4 (-2 to 0.8)
Southeast Asia, east Asia, and Oceania	Oceania	Papua New Guinea	Prevalence	All Ages	60,711 (59,419 to 61,815)	31,295 (30,476 to 31,981)	29,416 (28,793 to 29,998)	156,629 (152,109 to 160,383)	79,735 (77,037 to 81,908)	76,893 (74,404 to 78,875)	158 (151.7 to 162.8)	154.8 (147.3 to 161)	161.4 (153.7 to 167.2)
Southeast Asia, east Asia, and Oceania	Oceania	Papua New Guinea	Prevalence	Age-standardized	3120 (3045.5 to 3191.2)	3403.6 (3302 to 3493)	2823.8 (2752.5 to 2891.2)	3110.8 (3012.9 to 3197.4)	3408 (3278.2 to 3518.1)	2817 (2723.1 to 2900)	-0.3 (-2.6 to 1.6)	0.1 (-3.1 to 2.8)	-0.2 (-3.1 to 2.1)
Southeast Asia, east Asia, and Oceania	Oceania	Papua New Guinea	Deaths	All Ages	2,127 (1,656 to 2,617)	929 (623 to 1,236)	1,198 (865 to 1,609)	4,739 (3,596 to 6,212)	2,141 (1,349 to 2,928)	2,598 (1,884 to 3,418)	122.8 (73.7 to 183.3)	130.5 (68.9 to 208.7)	116.8 (62.4 to 192.5)
Southeast Asia, east Asia, and Oceania	Oceania	Papua New Guinea	Deaths	Age-standardized	159.7 (125.1 to 194.9)	134.8 (87.5 to 179)	188.4 (139.9 to 240.5)	145 (107.9 to 184.2)	131.7 (80.6 to 180.4)	159.7 (117.1 to 201.1)	-9.2 (-28.2 to 12.3)	-2.3 (-28.3 to 27.9)	-15.2 (-34.4 to 8.9)
Southeast Asia, east Asia, and Oceania	Oceania	Papua New Guinea	DALYs	All Ages	59,782 (48,700 to 73,478)	27,885 (19,780 to 36,039)	31,897 (23,730 to 42,856)	130,156 (100,852 to 167,224)	61,923 (41,502 to 83,350)	68,233 (50,960 to 89,972)	117.7 (71.6 to 174)	122.1 (67.8 to 193.8)	113.9 (62.7 to 186.4)
Southeast Asia, east Asia, and Oceania	Oceania	Papua New Guinea	DALYs	Age-standardized	3272.5 (2624.2 to 3944.9)	2976.8 (2109.5 to 3877.5)	3587.1 (2693.3 to 4672.3)	2902.7 (2264.5 to 3716)	2787.5 (1885.2 to 3689.2)	3019.7 (2258 to 3862.6)	-11.3 (-29.1 to 9.6)	-6.4 (-28.5 to 21.8)	-15.8 (-35 to 10)
Southeast Asia, east Asia, and Oceania	Oceania	Papua New Guinea	YLLs	All Ages	53,815 (43,041 to 67,287)	24,630 (16,460 to 33,016)	29,185 (21,044 to 40,358)	114,800 (85,708 to 151,813)	53,644 (33,587 to 75,134)	61,156 (43,750 to 82,405)	113.3 (63.2 to 176.8)	117.8 (57.5 to 198.8)	109.5 (55.2 to 188.3)
Southeast Asia, east Asia, and Oceania	Oceania	Papua New Guinea	YLLs	Age-standardized	2972.4 (2332.1 to 3656.6)	2632.8 (1773.3 to 3515.5)	3331.4 (2433.3 to 4415.6)	2604.8 (1980.1 to 3404.7)	2444.6 (1534.4 to 3342.4)	2765.1 (2018.5 to 3616)	-12.4 (-31.6 to 10.7)	-7.1 (-32.3 to 24.7)	-17 (-37.2 to 10.7)
Southeast Asia, east Asia, and Oceania	Oceania	Papua New Guinea	YLDs	All Ages	5,967 (4,910 to 6,777)	3,254 (2,677 to 3,737)	2,712 (2,193 to 3,158)	15,355 (12,674 to 17,496)	8,279 (6,813 to 9,582)	7,076 (5,744 to 8,266)	157.4 (145.8 to 168.7)	154.4 (140.2 to 170)	160.9 (145.8 to 177)
Southeast Asia, east Asia, and Oceania	Oceania	Papua New Guinea	YLDs	Age-standardized	300.1 (248.9 to 338.9)	344 (285.7 to 394.8)	255.7 (208.6 to 295.7)	297.9 (246 to 337.4)	343 (285.1 to 397.6)	254.7 (207.7 to 296.3)	-0.7 (-4 to 2.6)	-0.3 (-4.8 to 4.2)	-0.4 (-5.2 to 4.4)
Southeast Asia, east Asia, and Oceania	Oceania	Samoa	Incidence	All Ages	146 (139 to 155)	68 (64 to 73)	78 (74 to 83)	178 (168 to 189)	90 (84 to 96)	89 (83 to 94)	21.9 (17.6 to 26.4)	31.8 (24.7 to 38.5)	13.2 (8.3 to 18.2)
Southeast Asia, east Asia, and Oceania	Oceania	Samoa	Incidence	Age-standardized	162.5 (153.2 to 172.2)	144.7 (133.7 to 157)	182.9 (172.6 to 193.8)	129.3 (121.1 to 137.4)	123.1 (114.2 to 132.3)	136.2 (128 to 144.5)	-20.4 (-23.1 to -17.7)	-14.9 (-19.6 to -10.3)	-25.6 (-28.5 to -22.3)
Southeast Asia, east Asia, and Oceania	Oceania	Samoa	Prevalence	All Ages	1,638 (1,558 to 1,719)	813 (766 to 865)	825 (783 to 870)	1,894 (1,774 to 2,013)	1,033 (964 to 1,102)	861 (804 to 921)	15.6 (10.5 to 20.9)	27 (20.2 to 34)	4.4 (-2.1 to 10.7)
Southeast Asia, east Asia, and Oceania	Oceania	Samoa	Prevalence	Age-standardized	1734.9 (1636.6 to 1842.9)	1683.7 (1575.5 to 1807.1)	1781.1 (1679.6 to 1889.8)	1287.6 (1203.2 to 1375.8)	1360.1 (1267.4 to 1452.8)	1212.6 (1131.3 to 1299.3)	-25.8 (-28.9 to -22.7)	-19.2 (-23.7 to -14.9)	-31.9 (-35.3 to -28.1)
Southeast Asia, east Asia, and Oceania	Oceania	Samoa	Deaths	All Ages	68 (55 to 91)	27 (18 to 41)	42 (33 to 55)	72 (58 to 92)	33 (24 to 44)	39 (32 to 54)	5.4 (-21.3 to 37.8)	22.3 (-27.7 to 79.6)	-5.5 (-26.8 to 24.2)
Southeast Asia, east Asia, and Oceania	Oceania	Samoa	Deaths	Age-standardized	92.9 (75 to 123.9)	66.6 (46.4 to 102.5)	129.3 (101.7 to 169.6)	58 (47 to 73.3)	48.6 (35.1 to 64.7)	69.9 (57.6 to 95)	-37.6 (-53.3 to -19.8)	-27.1 (-57.1 to 6)	-45.9 (-57.3 to -30.5)
Southeast Asia, east Asia, and Oceania	Oceania	Samoa	DALYs	All Ages	1,688 (1,346 to 2,216)	670 (479 to 1,006)	1,018 (798 to 1,338)	1,655 (1,322 to 2,073)	782 (577 to 1,026)	873 (707 to 1,174)	-2 (-26.6 to 28.6)	16.7 (-28.1 to 67.1)	-14.3 (-34.7 to 12.8)
Southeast Asia, east Asia, and Oceania	Oceania	Samoa	DALYs	Age-standardized	1907.5 (1538.5 to 2487.9)	1448.4 (1040.2 to 2161.4)	2456.9 (1952.3 to 3222.4)	1160.1 (942.9 to 1437)	1051.2 (779.2 to 1361.1)	1296.5 (1071.7 to 1735.9)	-39.2 (-54 to -20.8)	-27.4 (-55.3 to 3.1)	-47.2 (-58.6 to -31.1)
Southeast Asia, east Asia, and Oceania	Oceania	Samoa	YLLs	All Ages	1,526 (1,188 to 2,053)	584 (395 to 926)	941 (720 to 1,261)	1,468 (1,133 to 1,895)	675 (468 to 916)	793 (627 to 1,094)	-3.8 (-30.4 to 30.3)	15.5 (-32.8 to 74.6)	-15.8 (-37.6 to 13.6)
Southeast Asia, east Asia, and Oceania	Oceania	Samoa	YLLs	Age-standardized	1738.6 (1372.8 to 2303.8)	1274.8 (870.8 to 1993.5)	2294.2 (1788.7 to 3052.9)	1034.8 (816.5 to 1318.8)	912.1 (635.8 to 1229)	1185.7 (957.7 to 1623.7)	-40.5 (-56.3 to -20.2)	-28.5 (-58 to 6.9)	-48.3 (-60.4 to -31.3)
Southeast Asia, east Asia, and Oceania	Oceania	Samoa	YLDs	All Ages	163 (133 to 186)	86 (70 to 99)	77 (62 to 91)	187 (155 to 216)	107 (88 to 126)	80 (65 to 94)	15.1 (7.1 to 22.9)	25.3 (14.7 to 35.9)	3.8 (-6.8 to 14.5)
Southeast Asia, east Asia, and Oceania	Oceania	Samoa	YLDs	Age-standardized	168.9 (139.3 to 191.9)	173.6 (141.5 to 201.4)	162.7 (131.6 to 189.7)	125.3 (103.9 to 143.3)	139.2 (114 to 161.9)	110.8 (90.4 to 129.5)	-25.8 (-30.1 to -21.5)	-19.8 (-25.8 to -13.9)	-31.9 (-36.8 to -26.5)
Southeast Asia, east Asia, and Oceania	Oceania	Solomon Islands	Incidence	All Ages	235 (220 to 249)	102 (96 to 110)	132 (125 to 141)	438 (413 to 463)	213 (198 to 228)	225 (213 to 238)	86.5 (80.2 to 92.8)	107.6 (97.1 to 118.7)	70.2 (62.8 to 77.4)
Southeast Asia, east Asia, and Oceania	Oceania	Solomon Islands	Incidence	Age-standardized	165.6 (155.7 to 175.9)	156.7 (145 to 169.1)	171.5 (161.4 to 181.7)	150.4 (142.3 to 159.7)	146.6 (136 to 157.8)	154.3 (146.5 to 162.6)	-9.2 (-12.1 to -6.1)	-6.4 (-11.4 to -1.5)	-10 (-13.7 to -6.2)
Southeast Asia, east Asia, and Oceania	Oceania	Solomon Islands	Prevalence	All Ages	2,541 (2,391 to 2,690)	1,215 (1,136 to 1,298)	1,327 (1,248 to 1,409)	4,941 (4,617 to 5,265)	2,606 (2,421 to 2,807)	2,335 (2,174 to 2,486)	94.4 (86.4 to 102.4)	114.6 (104.1 to 127.4)	76 (66.5 to 85.8)
Southeast Asia, east Asia, and Oceania	Oceania	Solomon Islands	Prevalence	Age-standardized	1649.8 (1547 to 1757.1)	1754.9 (1629.9 to 1889.9)	1564.8 (1467.9 to 1670.6)	1478.5 (1386.4 to 1575.5)	1609.4 (1497.2 to 1734)	1358.9 (1274.3 to 1446.3)	-10.4 (-13.7 to -6.9)	-8.3 (-13.1 to -3.1)	-13.2 (-17.2 to -9)
Southeast Asia, east Asia, and Oceania	Oceania	Solomon Islands	Deaths	All Ages	120 (89 to 170)	36 (24 to 52)	84 (61 to 131)	232 (178 to 289)	81 (56 to 109)	151 (113 to 195)	93.1 (46 to 155.1)	123.1 (55.8 to 216.1)	80.1 (29.4 to 143)
Southeast Asia, east Asia, and Oceania	Oceania	Solomon Islands	Deaths	Age-standardized	113.9 (86.6 to 159.1)	72.6 (49 to 101)	145.2 (106.2 to 226.5)	103 (81.5 to 123.2)	69.8 (46.9 to 91.8)	133.9 (104.8 to 168.6)	-9.6 (-30.7 to 14)	-3.9 (-32 to 33.6)	-7.8 (-33.6 to 20)
Southeast Asia, east Asia, and Oceania	Oceania	Solomon Islands	DALYs	All Ages	3,656 (2,742 to 4,999)	1,255 (842 to 1,775)	2,401 (1,748 to 3,673)	6,805 (5,286 to 8,556)	2,592 (1,824 to 3,427)	4,213 (3,140 to 5,566)	86.2 (41.5 to 145.8)	106.6 (50.4 to 186.2)	75.5 (27.9 to 135.8)
Southeast Asia, east Asia, and Oceania	Oceania	Solomon Islands	DALYs	Age-standardized	2538.7 (1931.8 to 3526.6)	1798.2 (1239.8 to 2491.6)	3103 (2286.6 to 4802.7)	2178.6 (1719.5 to 2655.8)	1605.3 (1158.9 to 2089.4)	2724.7 (2087.8 to 3470.8)	-14.2 (-33.7 to 9.8)	-10.7 (-34.7 to 20.8)	-12.2 (-35.8 to 15.6)
Southeast Asia, east Asia, and Oceania	Oceania	Solomon Islands	YLLs	All Ages	3,401 (2,493 to 4,750)	1,125 (714 to 1,637)	2,276 (1,622 to 3,538)	6,311 (4,766 to 8,062)	2,316 (1,543 to 3,153)	3,995 (2,924 to 5,352)	85.6 (38.4 to 150.2)	106 (44.8 to 198.6)	75.5 (25.9 to 139.8)
Southeast Asia, east Asia, and Oceania	Oceania	Solomon Islands	YLLs	Age-standardized	2378.6 (1772.6 to 3350.8)	1618.4 (1064.5 to 2288.9)	2958.9 (2149.2 to 4645.1)	2035.5 (1568.7 to 2518.5)	1466.5 (1009.9 to 1922.1)	2600.6 (1960.1 to 3339)	-14.4 (-35 to 12)	-10.9 (-37.3 to 24.8)	-12.1 (-36.7 to 17.4)
Southeast Asia, east Asia, and Oceania	Oceania	Solomon Islands	YLDs	All Ages	255 (209 to 296)	130 (106 to 154)	125 (100 to 147)	494 (403 to 577)	276 (224 to 326)	218 (174 to 259)	94 (80.1 to 109.2)	112.1 (90.5 to 134.9)	75.2 (56.3 to 95.1)
Southeast Asia, east Asia, and Oceania	Oceania	Solomon Islands	YLDs	Age-standardized	160.2 (131.7 to 184.1)	179.8 (147 to 210.4)	144.1 (116 to 169.3)	143.1 (116.2 to 164.2)	163.5 (132.2 to 191.2)	124.1 (99.5 to 144.7)	-10.6 (-15.6 to -5.5)	-9.1 (-15.6 to -2.6)	-13.9 (-20.4 to -7.3)
Southeast Asia, east Asia, and Oceania	Oceania	Tokelau	Incidence	All Ages	4 (4 to 4)	2 (2 to 2)	2 (2 to 2)	4 (4 to 4)	2 (2 to 2)	2 (2 to 2)	-5.5 (-8.6 to -2.2)	-12 (-15.9 to -8)	1.4 (-2.7 to 5.8)
Southeast Asia, east Asia, and Oceania	Oceania	Tokelau	Incidence	Age-standardized	291.6 (278.7 to 303.9)	277.5 (261.5 to 292.2)	308.1 (295.4 to 319.4)	282.3 (270.2 to 294.4)	271.6 (256.6 to 287.8)	293.2 (282 to 304.4)	-3.2 (-5.8 to -0.4)	-2.1 (-5.8 to 1.6)	-4.8 (-8.1 to -1.2)
Southeast Asia, east Asia, and Oceania	Oceania	Tokelau	Prevalence	All Ages	42 (40 to 44)	24 (23 to 25)	18 (17 to 19)	42 (39 to 44)	22 (21 to 23)	20 (19 to 21)	-1.9 (-5.3 to 1.7)	-8.4 (-12.2 to -4.5)	6.6 (1.4 to 12)
Southeast Asia, east Asia, and Oceania	Oceania	Tokelau	Prevalence	Age-standardized	3162.2 (3013.2 to 3304.8)	3318 (3135.3 to 3499.7)	2973.9 (2839.3 to 3106.8)	3210.1 (3052.1 to 3364.8)	3395.7 (3204.2 to 3608.6)	3021.8 (2865.7 to 3171.3)	1.5 (-1.8 to 5.1)	2.3 (-1.7 to 6.4)	1.6 (-3 to 6.4)
Southeast Asia, east Asia, and Oceania	Oceania	Tokelau	Deaths	All Ages	1 (1 to 1)	1 (0 to 1)	1 (0 to 1)	1 (0 to 1)	0 (0 to 0)	0 (0 to 0)	-49.4 (-61.2 to -34.9)	-48.4 (-64.6 to -27.4)	-50.4 (-62.4 to -32.8)
Southeast Asia, east Asia, and Oceania	Oceania	Tokelau	Deaths	Age-standardized	83.4 (67.6 to 103.5)	75.8 (54.7 to 101.4)	92.1 (70.7 to 127)	45.4 (36.1 to 56.7)	44.3 (30.7 to 58.9)	46.8 (37.6 to 59.3)	-45.6 (-58.2 to -30.4)	-41.6 (-60 to -17.7)	-49.2 (-61.5 to -31.9)
Southeast Asia, east Asia, and Oceania	Oceania	Tokelau	DALYs	All Ages	26 (21 to 32)	14 (10 to 18)	12 (9 to 16)	14 (12 to 17)	8 (6 to 10)	7 (6 to 8)	-44.3 (-56 to -30.8)	-45 (-59.3 to -27)	-43.4 (-56 to -27.1)
Southeast Asia, east Asia, and Oceania	Oceania	Tokelau	DALYs	Age-standardized	1901.4 (1574.8 to 2354.4)	1905.4 (1425.1 to 2486.8)	1888.8 (1503.3 to 2502.6)	1111.8 (908.8 to 1335.4)	1180.5 (891.8 to 1492.9)	1047.2 (871.6 to 1267.9)	-41.5 (-53.6 to -27.6)	-38 (-54.2 to -17.7)	-44.6 (-56.7 to -28.9)
Southeast Asia, east Asia, and Oceania	Oceania	Tokelau	YLLs	All Ages	22 (17 to 28)	12 (8 to 16)	10 (8 to 14)	10 (8 to 13)	5 (4 to 7)	5 (4 to 6)	-52.2 (-64.7 to -36.4)	-52.6 (-68.4 to -31.2)	-51.7 (-64.1 to -34)
Southeast Asia, east Asia, and Oceania	Oceania	Tokelau	YLLs	Age-standardized	1594.1 (1272.1 to 2047.2)	1568.1 (1087.7 to 2157.3)	1617.2 (1229 to 2239.7)	804.3 (619.3 to 1028.2)	838.8 (559.9 to 1145.3)	773.8 (611.1 to 982.6)	-49.5 (-62.7 to -34.1)	-46.5 (-64.5 to -22.9)	-52.2 (-64.2 to -34.8)
Southeast Asia, east Asia, and Oceania	Oceania	Tokelau	YLDs	All Ages	4 (3 to 5)	2 (2 to 3)	2 (1 to 2)	4 (3 to 5)	2 (2 to 3)	2 (1 to 2)	-2.9 (-7.2 to 1.5)	-9.1 (-14.2 to -3.7)	6.1 (-0.2 to 12.6)
Southeast Asia, east Asia, and Oceania	Oceania	Tokelau	YLDs	Age-standardized	307.3 (253.6 to 350.4)	337.3 (274.4 to 391.4)	271.5 (220.9 to 317.7)	307.5 (253.3 to 349.5)	341.7 (278.4 to 395.1)	273.3 (220.5 to 321.3)	0.1 (-4 to 4.5)	1.3 (-4 to 7)	0.7 (-4.9 to 6.6)
Southeast Asia, east Asia, and Oceania	Oceania	Tonga	Incidence	All Ages	83 (79 to 88)	37 (34 to 39)	47 (44 to 49)	102 (97 to 107)	47 (44 to 50)	55 (52 to 58)	22.6 (18.4 to 27)	28.6 (22.1 to 35.4)	17.9 (12.4 to 23)
Southeast Asia, east Asia, and Oceania	Oceania	Tonga	Incidence	Age-standardized	158.6 (150.1 to 167.4)	133.8 (124.7 to 143.3)	184.4 (175.2 to 194.3)	128.7 (122 to 136.1)	104.3 (97 to 112)	155.9 (148.2 to 164.6)	-18.8 (-21.5 to -16.2)	-22 (-25.6 to -18.3)	-15.5 (-19.1 to -11.9)
Southeast Asia, east Asia, and Oceania	Oceania	Tonga	Prevalence	All Ages	817 (766 to 866)	398 (371 to 424)	419 (392 to 445)	978 (918 to 1,034)	464 (430 to 496)	514 (482 to 548)	19.7 (15.6 to 24.2)	16.7 (11 to 23)	22.6 (16.6 to 28.8)
Southeast Asia, east Asia, and Oceania	Oceania	Tonga	Prevalence	Age-standardized	1482.9 (1394.2 to 1575.7)	1372.4 (1280.1 to 1468.2)	1593.4 (1490 to 1693.9)	1200.7 (1126.4 to 1273.4)	1016.3 (940.9 to 1087.9)	1400.5 (1311.6 to 1494.1)	-19 (-21.8 to -16.1)	-25.9 (-29.3 to -22.3)	-12.1 (-16.6 to -7.8)
Southeast Asia, east Asia, and Oceania	Oceania	Tonga	Deaths	All Ages	25 (21 to 30)	7 (5 to 9)	18 (15 to 23)	30 (24 to 36)	9 (5 to 12)	21 (17 to 26)	19.3 (-8.7 to 58.6)	30.5 (-24.5 to 93.6)	15.3 (-12.5 to 58.2)
Southeast Asia, east Asia, and Oceania	Oceania	Tonga	Deaths	Age-standardized	56 (46 to 66.3)	26.8 (20.2 to 34.9)	93.5 (74.2 to 113.6)	39.5 (31.7 to 47.4)	19.5 (12.1 to 27.1)	68.7 (55.1 to 83.3)	-29.4 (-45.8 to -6.7)	-27.3 (-57.8 to 8.3)	-26.6 (-43.5 to 0.9)
Southeast Asia, east Asia, and Oceania	Oceania	Tonga	DALYs	All Ages	591 (496 to 695)	186 (149 to 231)	404 (329 to 494)	619 (507 to 746)	196 (141 to 257)	423 (340 to 515)	4.8 (-17.8 to 36.2)	5.1 (-29.6 to 45.1)	4.7 (-19.2 to 41.4)
Southeast Asia, east Asia, and Oceania	Oceania	Tonga	DALYs	Age-standardized	1112.8 (938.7 to 1302.2)	644.4 (515.3 to 798.2)	1650 (1347.6 to 2001.1)	787.2 (646 to 944.4)	447.1 (320.4 to 588)	1220.8 (990.9 to 1477.7)	-29.3 (-44.2 to -8.4)	-30.6 (-54 to -4.3)	-26 (-42.5 to -1.2)
Southeast Asia, east Asia, and Oceania	Oceania	Tonga	YLLs	All Ages	510 (418 to 609)	144 (107 to 187)	365 (291 to 453)	524 (414 to 644)	148 (93 to 210)	375 (294 to 468)	2.7 (-22.5 to 39.6)	2.6 (-40.3 to 56.1)	2.8 (-23.2 to 44.4)
Southeast Asia, east Asia, and Oceania	Oceania	Tonga	YLLs	Age-standardized	968.1 (797.2 to 1153.6)	502.6 (374.8 to 650.4)	1503.5 (1205.4 to 1847.8)	670.3 (533 to 821.7)	342.4 (215.9 to 485.3)	1091.5 (861.2 to 1353.3)	-30.8 (-47.5 to -6.6)	-31.9 (-60.2 to 3.6)	-27.4 (-44.9 to 0.3)
Southeast Asia, east Asia, and Oceania	Oceania	Tonga	YLDs	All Ages	81 (67 to 93)	42 (34 to 49)	39 (32 to 46)	96 (79 to 109)	48 (39 to 56)	48 (39 to 56)	17.9 (10.5 to 25.4)	14 (4.2 to 24.6)	22.1 (12.5 to 33.3)
Southeast Asia, east Asia, and Oceania	Oceania	Tonga	YLDs	Age-standardized	144.7 (119.6 to 164.9)	141.8 (114.7 to 164.7)	146.5 (119.3 to 171.4)	116.9 (96.9 to 133.4)	104.7 (85.4 to 121.9)	129.3 (106.1 to 151.2)	-19.2 (-23.3 to -15)	-26.1 (-31.3 to -20.3)	-11.8 (-18 to -5.2)
Southeast Asia, east Asia, and Oceania	Oceania	Tuvalu	Incidence	All Ages	20 (18 to 21)	11 (10 to 11)	9 (9 to 9)	27 (25 to 28)	13 (13 to 14)	13 (13 to 14)	35.9 (31.6 to 40.6)	27 (21.6 to 32.6)	46.5 (40.6 to 52.9)
Southeast Asia, east Asia, and Oceania	Oceania	Tuvalu	Incidence	Age-standardized	281.3 (268.2 to 294.6)	269.5 (254.3 to 285.9)	302.1 (290.1 to 314.1)	263.9 (251.8 to 276.7)	253.3 (238.2 to 269.2)	277.9 (267.3 to 289.6)	-6.2 (-8.8 to -3.2)	-6 (-9.5 to -2.1)	-8 (-11 to -4.9)
Southeast Asia, east Asia, and Oceania	Oceania	Tuvalu	Prevalence	All Ages	203 (192 to 213)	120 (113 to 127)	83 (78 to 87)	289 (274 to 306)	161 (150 to 172)	129 (122 to 136)	42.8 (37.7 to 48.3)	33.9 (27.9 to 40.1)	55.8 (48.6 to 63.6)
Southeast Asia, east Asia, and Oceania	Oceania	Tuvalu	Prevalence	Age-standardized	3025.2 (2862.8 to 3187.1)	3161.3 (2968.5 to 3352.1)	2813.4 (2671.2 to 2955.5)	2891.4 (2732.1 to 3064.5)	3070.7 (2872.6 to 3273.1)	2678.5 (2539.8 to 2832.4)	-4.4 (-7.8 to -0.7)	-2.9 (-7.1 to 1.5)	-4.8 (-9.1 to -0.1)
Southeast Asia, east Asia, and Oceania	Oceania	Tuvalu	Deaths	All Ages	6 (5 to 8)	3 (2 to 4)	3 (2 to 4)	5 (4 to 7)	2 (1 to 3)	3 (2 to 4)	-16.3 (-39.3 to 14.6)	-17.6 (-48 to 23)	-15.2 (-37.8 to 23.4)
Southeast Asia, east Asia, and Oceania	Oceania	Tuvalu	Deaths	Age-standardized	108.7 (85.9 to 139.1)	84.2 (58.6 to 120.5)	153.1 (113.7 to 202.9)	61.1 (47.8 to 78.6)	50 (30.2 to 67.2)	74.9 (57.8 to 98.4)	-43.8 (-59.5 to -24)	-40.6 (-63.4 to -13.2)	-51.1 (-63.4 to -29.9)
Southeast Asia, east Asia, and Oceania	Oceania	Tuvalu	DALYs	All Ages	177 (144 to 217)	85 (61 to 113)	91 (70 to 118)	140 (112 to 176)	66 (47 to 86)	73 (58 to 95)	-20.9 (-39.9 to 4.2)	-22.1 (-46.2 to 9.1)	-19.7 (-40.3 to 13.9)
Southeast Asia, east Asia, and Oceania	Oceania	Tuvalu	DALYs	Age-standardized	2549.7 (2084.5 to 3136.8)	2140.5 (1567.6 to 2830.9)	3201 (2449.8 to 4073.4)	1409.9 (1140.9 to 1772.1)	1260.8 (894.6 to 1618.4)	1589.3 (1270.2 to 2037.6)	-44.7 (-57.9 to -27.7)	-41.1 (-58.7 to -18.1)	-50.4 (-62.1 to -31)
Southeast Asia, east Asia, and Oceania	Oceania	Tuvalu	YLLs	All Ages	157 (123 to 197)	73 (49 to 100)	84 (62 to 110)	112 (85 to 149)	50 (30 to 70)	62 (46 to 83)	-28.7 (-48.9 to -1.1)	-31.3 (-56.9 to 4.1)	-26.5 (-47.6 to 9.5)
Southeast Asia, east Asia, and Oceania	Oceania	Tuvalu	YLLs	Age-standardized	2254 (1789.4 to 2844.8)	1818.3 (1235.2 to 2516.7)	2943.9 (2191.4 to 3827)	1131.5 (867.9 to 1482.7)	951 (581.1 to 1316.4)	1346.7 (1019.6 to 1792.1)	-49.8 (-63.7 to -30.8)	-47.7 (-67 to -21.6)	-54.3 (-66.3 to -34)
Southeast Asia, east Asia, and Oceania	Oceania	Tuvalu	YLDs	All Ages	20 (16 to 23)	12 (10 to 14)	8 (6 to 9)	28 (23 to 32)	16 (13 to 19)	12 (9 to 14)	40.3 (33.9 to 48)	31.9 (24.1 to 40.3)	53.9 (44.5 to 65.1)
Southeast Asia, east Asia, and Oceania	Oceania	Tuvalu	YLDs	Age-standardized	295.8 (242.4 to 338.6)	322.2 (261.1 to 375.5)	257.1 (207.1 to 300.6)	278.3 (227.2 to 319.2)	309.8 (253.7 to 359.9)	242.6 (196.1 to 282.6)	-5.9 (-10 to -1.1)	-3.8 (-9.1 to 1.9)	-5.7 (-11 to 0.2)
Southeast Asia, east Asia, and Oceania	Oceania	Vanuatu	Incidence	All Ages	140 (132 to 149)	62 (57 to 67)	78 (74 to 83)	283 (264 to 305)	133 (122 to 146)	150 (140 to 160)	102.6 (95.4 to 110)	116.8 (105 to 130.5)	91.4 (84.2 to 99.1)
Southeast Asia, east Asia, and Oceania	Oceania	Vanuatu	Incidence	Age-standardized	203 (190.4 to 217)	191 (175.1 to 208.2)	212.3 (200.7 to 225.1)	166.4 (155.4 to 178)	162 (148.7 to 177.5)	170.3 (160.3 to 180.6)	-18 (-20.9 to -15.2)	-15.2 (-19.8 to -9.9)	-19.8 (-22.9 to -16.6)
Southeast Asia, east Asia, and Oceania	Oceania	Vanuatu	Prevalence	All Ages	1,478 (1,400 to 1,560)	716 (672 to 765)	762 (724 to 802)	3,031 (2,844 to 3,231)	1,559 (1,438 to 1,685)	1,472 (1,385 to 1,563)	105.1 (97.1 to 112.4)	117.9 (105.2 to 130)	93.1 (85.4 to 100.6)
Southeast Asia, east Asia, and Oceania	Oceania	Vanuatu	Prevalence	Age-standardized	1980.4 (1858 to 2121)	2111.7 (1950.9 to 2304.5)	1874.9 (1764.8 to 1997.2)	1627.4 (1520.1 to 1741.7)	1769.4 (1624.4 to 1927.6)	1498.7 (1403.3 to 1599.5)	-17.8 (-21.2 to -14.8)	-16.2 (-21 to -11.1)	-20.1 (-23.5 to -16.7)
Southeast Asia, east Asia, and Oceania	Oceania	Vanuatu	Deaths	All Ages	58 (40 to 80)	17 (10 to 27)	41 (27 to 58)	114 (85 to 150)	38 (24 to 56)	76 (54 to 102)	97.7 (48.3 to 166)	121.6 (52 to 229.2)	87.7 (39.1 to 148.6)
Southeast Asia, east Asia, and Oceania	Oceania	Vanuatu	Deaths	Age-standardized	112.4 (78.9 to 154.3)	72.3 (44.8 to 118.7)	144.5 (98.2 to 204.6)	83.3 (63.2 to 107.9)	56.7 (36.9 to 83.3)	107.5 (77.5 to 142.1)	-25.9 (-43.3 to -3.4)	-21.5 (-44.6 to 13.2)	-25.6 (-43.2 to -3.7)
Southeast Asia, east Asia, and Oceania	Oceania	Vanuatu	DALYs	All Ages	1,603 (1,153 to 2,187)	531 (344 to 806)	1,072 (735 to 1,518)	3,033 (2,297 to 3,999)	1,106 (764 to 1,585)	1,927 (1,402 to 2,582)	89.2 (42 to 155)	108.2 (47.6 to 200.7)	79.8 (32.9 to 140.8)
Southeast Asia, east Asia, and Oceania	Oceania	Vanuatu	DALYs	Age-standardized	2338.8 (1689.7 to 3174.8)	1642.1 (1081.3 to 2528.8)	2902.7 (2000 to 4079.3)	1735.4 (1326.7 to 2253.6)	1287.9 (894.7 to 1823.3)	2144.9 (1570.6 to 2821.2)	-25.8 (-43.6 to -2)	-21.6 (-43.8 to 10.9)	-26.1 (-44.4 to -2.7)
Southeast Asia, east Asia, and Oceania	Oceania	Vanuatu	YLLs	All Ages	1,457 (1,005 to 2,047)	456 (266 to 732)	1,001 (663 to 1,449)	2,732 (2,003 to 3,705)	942 (598 to 1,415)	1,789 (1,266 to 2,451)	87.6 (36.7 to 160.6)	106.9 (39.1 to 222.6)	78.8 (29.2 to 144.5)
Southeast Asia, east Asia, and Oceania	Oceania	Vanuatu	YLLs	Age-standardized	2147.2 (1496.9 to 2997.2)	1426.2 (864.6 to 2307.5)	2731.3 (1831 to 3904.4)	1577 (1172.9 to 2098.9)	1107.5 (711.3 to 1639)	2006.8 (1437.4 to 2686.5)	-26.6 (-45.3 to -0.3)	-22.3 (-46.9 to 16.3)	-26.5 (-45.7 to -1.8)
Southeast Asia, east Asia, and Oceania	Oceania	Vanuatu	YLDs	All Ages	147 (120 to 169)	76 (62 to 88)	71 (57 to 83)	301 (247 to 345)	163 (133 to 192)	138 (112 to 161)	105.2 (93 to 119.1)	115.8 (98 to 136.2)	94 (78.8 to 110.8)
Southeast Asia, east Asia, and Oceania	Oceania	Vanuatu	YLDs	Age-standardized	191.6 (158 to 219.7)	215.9 (176.2 to 251.4)	171.4 (139.4 to 200.3)	158.4 (130.5 to 182)	180.4 (147.1 to 212.5)	138.1 (112.7 to 161.2)	-17.3 (-21.5 to -12.6)	-16.4 (-22 to -9.6)	-19.4 (-24.9 to -14.1)
		Central Europe, Eastern Europe, and Central Asia	Incidence	All Ages	780,155 (729,795 to 827,453)	351,357 (323,171 to 380,017)	428,798 (403,417 to 451,843)	789,947 (739,487 to 844,434)	322,439 (299,280 to 347,090)	467,507 (435,785 to 500,757)	1.3 (-0.7 to 3.6)	-8.2 (-11.6 to -4.1)	9 (6.1 to 12.1)
		Central Europe, Eastern Europe, and Central Asia	Incidence	Age-standardized	174.5 (164.2 to 184.6)	132.5 (123.5 to 141.8)	240 (226.9 to 252.5)	137.3 (129.8 to 145.5)	96.6 (90.8 to 103)	194.1 (182.7 to 206.5)	-21.3 (-22.5 to -19.9)	-27.1 (-29.1 to -24.9)	-19.1 (-20.8 to -17.4)
		Central Europe, Eastern Europe, and Central Asia	Prevalence	All Ages	11,536,376 (10,972,370 to 12,169,761)	5,619,563 (5,280,908 to 6,003,898)	5,916,813 (5,642,191 to 6,183,970)	12,158,928 (11,514,078 to 12,904,784)	5,344,734 (5,025,748 to 5,704,897)	6,814,194 (6,466,413 to 7,231,002)	5.4 (3.5 to 7.2)	-4.9 (-7.4 to -2.1)	15.2 (12.4 to 18)
		Central Europe, Eastern Europe, and Central Asia	Prevalence	Age-standardized	2542.3 (2414.9 to 2678.7)	2074.4 (1960.6 to 2203.6)	3394.4 (3225.9 to 3554.9)	2076.4 (1970.4 to 2196.9)	1593.9 (1498.2 to 1698.1)	2849.7 (2703.6 to 3016.1)	-18.3 (-19.9 to -16.8)	-23.2 (-25.2 to -21.1)	-16 (-17.9 to -14.2)
		Central Europe, Eastern Europe, and Central Asia	Deaths	All Ages	144,796 (122,474 to 151,835)	53,994 (33,297 to 58,865)	90,802 (87,942 to 93,580)	108,381 (97,258 to 130,087)	40,768 (35,061 to 59,338)	67,613 (60,957 to 74,345)	-25.1 (-33 to -2)	-24.5 (-36.6 to 42.7)	-25.5 (-32.6 to -19)
		Central Europe, Eastern Europe, and Central Asia	Deaths	Age-standardized	33.2 (27.7 to 34.9)	19.1 (11.7 to 20.9)	61.4 (58.6 to 63.5)	17.2 (15.4 to 20.6)	9.9 (8.6 to 14.5)	29.9 (26.9 to 32.8)	-48.3 (-53.7 to -32)	-48.1 (-56.3 to -1.7)	-51.3 (-55.7 to -47.2)
		Central Europe, Eastern Europe, and Central Asia	DALYs	All Ages	3,535,469 (3,129,856 to 3,729,216)	1,281,210 (916,911 to 1,403,934)	2,254,260 (2,159,343 to 2,351,729)	2,662,715 (2,426,824 to 3,010,379)	989,060 (875,096 to 1,262,268)	1,673,655 (1,523,188 to 1,836,983)	-24.7 (-30.8 to -9.6)	-22.8 (-31.2 to 19)	-25.8 (-32 to -19.7)
		Central Europe, Eastern Europe, and Central Asia	DALYs	Age-standardized	761.8 (670.9 to 804.1)	452.2 (326.4 to 495.7)	1298.8 (1243.4 to 1353.6)	435.7 (397 to 491.7)	274.6 (243.2 to 343)	685.6 (624 to 752)	-42.8 (-47.5 to -31.4)	-39.3 (-45.6 to -8.2)	-47.2 (-51.5 to -43.2)
		Central Europe, Eastern Europe, and Central Asia	YLLs	All Ages	2,856,870 (2,474,965 to 2,981,295)	904,653 (564,734 to 982,649)	1,952,217 (1,895,029 to 2,011,028)	1,958,209 (1,766,876 to 2,291,819)	636,363 (556,532 to 917,249)	1,321,845 (1,187,729 to 1,460,144)	-31.5 (-38.8 to -13.1)	-29.7 (-40.9 to 33.2)	-32.3 (-38.8 to -26)
		Central Europe, Eastern Europe, and Central Asia	YLLs	Age-standardized	612.2 (527.6 to 640)	312.1 (194.1 to 339.3)	1127.4 (1092.7 to 1161.7)	314.2 (283.8 to 367.4)	167 (146.5 to 239.5)	538.6 (485.4 to 593.9)	-48.7 (-54.1 to -34.7)	-46.5 (-54.9 to 1.5)	-52.2 (-56.7 to -47.9)
		Central Europe, Eastern Europe, and Central Asia	YLDs	All Ages	678,599 (531,756 to 808,373)	376,557 (296,406 to 447,806)	302,043 (232,306 to 374,140)	704,506 (554,543 to 835,866)	352,696 (280,408 to 419,610)	351,810 (269,020 to 433,406)	3.8 (1.2 to 6.6)	-6.3 (-9.9 to -2.6)	16.5 (12.8 to 20.1)
		Central Europe, Eastern Europe, and Central Asia	YLDs	Age-standardized	149.6 (116.9 to 177.3)	140.1 (111 to 166.3)	171.4 (129.9 to 212.7)	121.5 (95.6 to 144)	107.7 (85.3 to 127.6)	147 (112.2 to 180.6)	-18.8 (-20.8 to -16.4)	-23.2 (-26.1 to -20.2)	-14.3 (-16.5 to -12.1)
		Central Asia	Incidence	All Ages	84,458 (81,884 to 87,135)	40,318 (38,708 to 41,986)	44,140 (43,037 to 45,279)	111,587 (107,016 to 116,383)	49,966 (47,608 to 52,556)	61,621 (59,329 to 63,915)	32.1 (29.8 to 34.4)	23.9 (20.9 to 27)	39.6 (36.7 to 42.4)
		Central Asia	Incidence	Age-standardized	171.4 (165.9 to 177)	144.8 (139.2 to 150.5)	211.7 (205.5 to 217.9)	150.5 (144.4 to 156.7)	122.8 (117.2 to 128.7)	188 (180.6 to 195.2)	-12.2 (-13.6 to -10.8)	-15.2 (-17 to -13.4)	-11.2 (-12.9 to -9.4)
		Central Asia	Prevalence	All Ages	1,275,115 (1,240,112 to 1,313,192)	664,509 (641,774 to 690,983)	610,606 (596,998 to 624,029)	1,677,622 (1,613,858 to 1,745,238)	821,918 (783,220 to 861,703)	855,704 (826,056 to 885,689)	31.6 (28.8 to 34.1)	23.7 (20.2 to 27.1)	40.1 (37 to 43.2)
		Central Asia	Prevalence	Age-standardized	2579.6 (2503.8 to 2661.4)	2343.7 (2263.5 to 2437.3)	2986 (2907 to 3069.3)	2257.2 (2169.4 to 2349.1)	1970 (1881.3 to 2063.7)	2702.2 (2601.9 to 2798.2)	-12.5 (-14.2 to -10.9)	-15.9 (-18.2 to -13.8)	-9.5 (-11.4 to -7.7)
		Central Asia	Deaths	All Ages	15,802 (13,515 to 16,861)	6,857 (4,988 to 7,617)	8,945 (8,355 to 9,456)	17,223 (15,377 to 20,011)	7,222 (6,172 to 9,527)	10,001 (8,958 to 11,290)	9 (-4.5 to 39.9)	5.3 (-11.8 to 62.6)	11.8 (-2.2 to 29)
		Central Asia	Deaths	Age-standardized	37.6 (32.2 to 40.3)	26.2 (19 to 29.2)	57.9 (53.5 to 61.3)	31.5 (27.9 to 36.8)	22.5 (19.2 to 29.3)	46.3 (41.7 to 52.1)	-16.4 (-26.6 to 6.4)	-14.3 (-28.2 to 30.4)	-19.9 (-29.4 to -8.2)
		Central Asia	DALYs	All Ages	412,933 (362,876 to 439,217)	175,697 (134,406 to 192,232)	237,236 (222,843 to 251,589)	450,927 (408,701 to 509,389)	186,707 (165,106 to 231,760)	264,220 (237,639 to 295,171)	9.2 (-2.3 to 34.2)	6.3 (-6.4 to 52.4)	11.4 (-1 to 26)
		Central Asia	DALYs	Age-standardized	873.8 (765.3 to 929.7)	628.6 (481.2 to 687)	1264.6 (1185.6 to 1341)	662.8 (602.4 to 747.8)	484.7 (426.7 to 599.3)	925.1 (834.4 to 1029.7)	-24.1 (-32 to -6.9)	-22.9 (-32.1 to 10)	-26.8 (-34.9 to -17.4)
		Central Asia	YLLs	All Ages	339,015 (290,259 to 360,417)	133,348 (93,918 to 147,423)	205,668 (193,298 to 217,733)	354,338 (315,535 to 409,758)	134,220 (115,505 to 179,975)	220,117 (196,892 to 248,194)	4.5 (-9.2 to 34.3)	0.7 (-15 to 63.7)	7 (-6.8 to 23.8)
		Central Asia	YLLs	Age-standardized	726 (621.5 to 772.9)	480.4 (341.5 to 532)	1112.9 (1042.3 to 1176.4)	535.2 (478.2 to 621.3)	360.9 (309.7 to 474.7)	789 (707.5 to 888.6)	-26.3 (-35.4 to -5.5)	-24.9 (-36.6 to 19.8)	-29.1 (-38 to -18.5)
		Central Asia	YLDs	All Ages	73,918 (57,188 to 89,659)	42,350 (32,700 to 51,389)	31,568 (23,897 to 39,591)	96,589 (74,915 to 117,162)	52,486 (40,972 to 63,615)	44,103 (32,995 to 56,063)	30.7 (26.7 to 35.1)	23.9 (18.9 to 29)	39.7 (34.8 to 45.1)
		Central Asia	YLDs	Age-standardized	147.8 (113.7 to 179.4)	148.2 (113.4 to 180.2)	151.7 (113.3 to 191.6)	127.6 (98.9 to 153.9)	123.7 (95.6 to 150.1)	136.2 (100.5 to 172)	-13.7 (-16.2 to -11)	-16.5 (-19.7 to -13.3)	-10.2 (-13.2 to -7)
Central Europe, eastern Europe, and central Asia	Central Asia	Armenia	Incidence	All Ages	5,435 (5,208 to 5,652)	2,240 (2,120 to 2,367)	3,195 (3,046 to 3,334)	7,612 (7,326 to 7,925)	3,702 (3,542 to 3,868)	3,910 (3,720 to 4,113)	40 (35 to 45.4)	65.3 (57.1 to 74.5)	22.4 (16.3 to 28.6)
Central Europe, eastern Europe, and central Asia	Central Asia	Armenia	Incidence	Age-standardized	203.4 (195.2 to 211.5)	152.1 (144.7 to 159.9)	269.1 (257.8 to 280.6)	193.4 (186.3 to 201.8)	161 (154 to 168.5)	232.3 (221.4 to 243.6)	-4.9 (-8.1 to -1.7)	5.9 (1 to 11.2)	-13.7 (-17.5 to -9.8)
Central Europe, eastern Europe, and central Asia	Central Asia	Armenia	Prevalence	All Ages	74,339 (71,481 to 77,080)	31,894 (30,039 to 33,840)	42,445 (40,674 to 44,044)	93,518 (89,021 to 98,603)	43,738 (41,373 to 46,388)	49,781 (47,015 to 52,867)	25.8 (21.1 to 30.3)	37.1 (30.9 to 44.2)	17.3 (11.1 to 23.6)
Central Europe, eastern Europe, and central Asia	Central Asia	Armenia	Prevalence	Age-standardized	2753.8 (2642.4 to 2865.3)	2103.8 (1989.4 to 2221)	3694.3 (3523.5 to 3856.3)	2388 (2273.7 to 2515.4)	1958.3 (1846.1 to 2076.4)	2986.3 (2823.7 to 3172.5)	-13.3 (-16.5 to -9.9)	-6.9 (-11.2 to -2.5)	-19.2 (-23.3 to -14.7)
Central Europe, eastern Europe, and central Asia	Central Asia	Armenia	Deaths	All Ages	962 (799 to 1,036)	334 (190 to 384)	628 (584 to 672)	1,197 (932 to 1,435)	545 (288 to 703)	652 (533 to 784)	24.5 (2.4 to 50.7)	63.2 (26.8 to 107.1)	3.8 (-15.3 to 27.7)
Central Europe, eastern Europe, and central Asia	Central Asia	Armenia	Deaths	Age-standardized	43.6 (35.3 to 47.2)	26.1 (14.5 to 30.2)	71.3 (65.8 to 76.7)	30.6 (23.5 to 36.7)	22.9 (12.2 to 29.5)	41.9 (34.3 to 50.2)	-29.8 (-42.2 to -15.4)	-12.4 (-31.5 to 11.3)	-41.2 (-52 to -28.3)
Central Europe, eastern Europe, and central Asia	Central Asia	Armenia	DALYs	All Ages	22,950 (20,143 to 24,705)	7,657 (5,457 to 8,529)	15,293 (14,177 to 16,470)	23,032 (19,507 to 26,673)	9,647 (7,116 to 11,448)	13,385 (11,189 to 15,856)	0.4 (-13.4 to 19.5)	26 (5.9 to 55.1)	-12.5 (-26.6 to 5.8)
Central Europe, eastern Europe, and central Asia	Central Asia	Armenia	DALYs	Age-standardized	898.8 (774 to 965.5)	531.5 (368.6 to 594.5)	1440.6 (1341.9 to 1550.2)	572.3 (485.3 to 660.2)	409.9 (306.5 to 486.6)	798.7 (673.8 to 941.9)	-36.3 (-45 to -24.5)	-22.9 (-34.8 to -3.1)	-44.6 (-53.4 to -33.3)
Central Europe, eastern Europe, and central Asia	Central Asia	Armenia	YLLs	All Ages	18,761 (16,196 to 20,127)	5,634 (3,482 to 6,420)	13,128 (12,136 to 14,139)	17,859 (14,632 to 21,381)	6,981 (4,548 to 8,697)	10,878 (8,824 to 13,213)	-4.8 (-21.8 to 18.4)	23.9 (-2.9 to 63.3)	-17.1 (-33.4 to 4.4)
Central Europe, eastern Europe, and central Asia	Central Asia	Armenia	YLLs	Age-standardized	745.6 (629.5 to 801)	399.7 (240.1 to 456.5)	1255.3 (1172.4 to 1346.4)	439.6 (359.5 to 523.6)	288.9 (192.1 to 358.5)	648.8 (527.8 to 786.6)	-41 (-51.3 to -26.7)	-27.7 (-43.1 to -1.5)	-48.3 (-58.1 to -35.4)
Central Europe, eastern Europe, and central Asia	Central Asia	Armenia	YLDs	All Ages	4,189 (3,246 to 5,089)	2,023 (1,571 to 2,453)	2,166 (1,599 to 2,745)	5,173 (4,005 to 6,312)	2,666 (2,059 to 3,268)	2,506 (1,865 to 3,195)	23.5 (15.6 to 31.6)	31.8 (21 to 44)	15.7 (5.4 to 26.6)
Central Europe, eastern Europe, and central Asia	Central Asia	Armenia	YLDs	Age-standardized	153.2 (119.3 to 185.8)	131.8 (102.5 to 159.7)	185.3 (137.3 to 234.2)	132.8 (103.4 to 161)	121 (93.2 to 147.7)	149.8 (111.7 to 191.1)	-13.3 (-18 to -8.5)	-8.2 (-14.8 to -0.7)	-19.2 (-24.8 to -12.8)
Central Europe, eastern Europe, and central Asia	Central Asia	Azerbaijan	Incidence	All Ages	8,747 (8,348 to 9,119)	4,256 (4,043 to 4,493)	4,491 (4,284 to 4,698)	14,906 (13,892 to 15,947)	6,697 (6,164 to 7,310)	8,209 (7,659 to 8,745)	70.4 (62.6 to 78)	57.3 (48.4 to 67.6)	82.8 (73.8 to 92.2)
Central Europe, eastern Europe, and central Asia	Central Asia	Azerbaijan	Incidence	Age-standardized	163.5 (155.7 to 170.7)	139.4 (131.9 to 147)	196.8 (187.8 to 206)	167.1 (157.9 to 177.4)	137.4 (128 to 148.3)	208.2 (197.1 to 220)	2.2 (-1.5 to 6)	-1.4 (-6 to 4.4)	5.8 (0.8 to 10.6)
Central Europe, eastern Europe, and central Asia	Central Asia	Azerbaijan	Prevalence	All Ages	134,730 (129,181 to 139,871)	68,575 (65,198 to 72,162)	66,155 (63,248 to 68,850)	216,758 (203,899 to 230,539)	108,401 (100,998 to 116,217)	108,356 (102,003 to 115,017)	60.9 (53.9 to 67.9)	58.1 (49.7 to 67)	63.8 (55.3 to 73.1)
Central Europe, eastern Europe, and central Asia	Central Asia	Azerbaijan	Prevalence	Age-standardized	2477.8 (2367.1 to 2579.9)	2208.9 (2098.4 to 2329.1)	2895.7 (2758.2 to 3036.3)	2357.9 (2224 to 2504.5)	2119.9 (1986.1 to 2271.5)	2708.7 (2553.8 to 2866.4)	-4.8 (-8.5 to -1.1)	-4 (-8.4 to 1.5)	-6.5 (-11.3 to -1.7)
Central Europe, eastern Europe, and central Asia	Central Asia	Azerbaijan	Deaths	All Ages	1,356 (1,188 to 1,530)	591 (471 to 774)	765 (669 to 850)	1,512 (1,184 to 2,281)	627 (391 to 1,134)	885 (684 to 1,274)	11.5 (-15.1 to 66.9)	6 (-35.8 to 73.8)	15.8 (-14.3 to 70.3)
Central Europe, eastern Europe, and central Asia	Central Asia	Azerbaijan	Deaths	Age-standardized	29.7 (25.8 to 34.1)	20.6 (16.3 to 27.2)	45.9 (40.4 to 51.2)	24.9 (18.7 to 38.9)	19.2 (11.1 to 35.4)	33 (24.8 to 48.3)	-16.1 (-37.8 to 26.1)	-6.7 (-48.4 to 53.7)	-28.2 (-48 to 3.9)
Central Europe, eastern Europe, and central Asia	Central Asia	Azerbaijan	DALYs	All Ages	37,874 (34,203 to 41,195)	15,896 (13,702 to 18,721)	21,978 (19,401 to 24,338)	44,064 (36,883 to 57,745)	18,608 (14,486 to 27,468)	25,456 (20,387 to 33,610)	16.3 (-3.9 to 56.9)	17.1 (-9 to 64.7)	15.8 (-8.2 to 56.6)
Central Europe, eastern Europe, and central Asia	Central Asia	Azerbaijan	DALYs	Age-standardized	730.3 (658.8 to 799.4)	523.7 (449.4 to 624.6)	1047.4 (933.6 to 1154.3)	531.2 (437.9 to 722)	415.3 (313.1 to 631.1)	684.1 (550.1 to 910.7)	-27.3 (-40.1 to -0.6)	-20.7 (-41.4 to 14.3)	-34.7 (-48.4 to -11.5)
Central Europe, eastern Europe, and central Asia	Central Asia	Azerbaijan	YLLs	All Ages	30,032 (26,696 to 33,113)	11,495 (9,592 to 14,112)	18,537 (16,151 to 20,687)	31,539 (25,016 to 45,553)	11,672 (7,991 to 20,629)	19,867 (15,335 to 28,026)	5 (-18.9 to 55.5)	1.5 (-32.6 to 65.6)	7.2 (-20.6 to 56)
Central Europe, eastern Europe, and central Asia	Central Asia	Azerbaijan	YLLs	Age-standardized	587.8 (522.7 to 652.5)	383.2 (317.7 to 479.8)	899.3 (794.3 to 1001.3)	397.9 (312.4 to 592.3)	281.9 (182 to 501.9)	547.6 (423.7 to 781.9)	-32.3 (-47.9 to 0.6)	-26.4 (-53.3 to 19.7)	-39.1 (-54.8 to -11.7)
Central Europe, eastern Europe, and central Asia	Central Asia	Azerbaijan	YLDs	All Ages	7,842 (6,072 to 9,560)	4,400 (3,450 to 5,381)	3,441 (2,539 to 4,362)	12,525 (9,626 to 15,263)	6,936 (5,395 to 8,545)	5,590 (4,115 to 7,151)	59.7 (49.7 to 70.1)	57.6 (43.8 to 71.3)	62.4 (48 to 77.6)
Central Europe, eastern Europe, and central Asia	Central Asia	Azerbaijan	YLDs	Age-standardized	142.6 (110.1 to 173.6)	140.5 (110 to 171.8)	148 (108.2 to 187.9)	133.3 (102.9 to 162.3)	133.3 (103.5 to 163.6)	136.6 (99.6 to 173.3)	-6.5 (-11.7 to -0.7)	-5.1 (-12.6 to 2.8)	-7.7 (-14.6 to -1)
Central Europe, eastern Europe, and central Asia	Central Asia	Georgia	Incidence	All Ages	5,118 (4,841 to 5,413)	2,449 (2,295 to 2,602)	2,669 (2,523 to 2,840)	8,631 (8,147 to 9,119)	4,267 (3,999 to 4,540)	4,364 (4,098 to 4,634)	68.6 (61.4 to 76.1)	74.3 (63.9 to 84.8)	63.5 (54.1 to 72.6)
Central Europe, eastern Europe, and central Asia	Central Asia	Georgia	Incidence	Age-standardized	91 (86.9 to 95.7)	75 (71 to 79.1)	113.3 (108.1 to 119.1)	158 (150.2 to 166.1)	127.1 (120.6 to 134.2)	195.3 (185.2 to 206.3)	73.5 (67.7 to 79.6)	69.3 (61.7 to 77.5)	72.3 (64.6 to 80.2)
Central Europe, eastern Europe, and central Asia	Central Asia	Georgia	Prevalence	All Ages	90,103 (85,628 to 94,865)	44,184 (41,380 to 46,946)	45,919 (43,494 to 48,561)	132,801 (126,067 to 140,137)	64,970 (61,434 to 68,893)	67,831 (63,842 to 71,969)	47.4 (41.5 to 53.4)	47 (39.5 to 55.5)	47.7 (39.9 to 56)
Central Europe, eastern Europe, and central Asia	Central Asia	Georgia	Prevalence	Age-standardized	1531.7 (1459 to 1608.2)	1315 (1231 to 1395.5)	1868.1 (1777.2 to 1964.5)	2373.1 (2260.9 to 2495.2)	1949.8 (1851.1 to 2061.5)	2965.6 (2793.8 to 3135.3)	54.9 (49.4 to 61)	48.3 (41.4 to 56.4)	58.7 (51 to 66.7)
Central Europe, eastern Europe, and central Asia	Central Asia	Georgia	Deaths	All Ages	729 (627 to 1,045)	349 (278 to 650)	380 (331 to 439)	1,000 (793 to 1,392)	426 (300 to 775)	574 (458 to 709)	37.1 (9.1 to 68.6)	22.2 (-7.2 to 57.5)	50.8 (15.7 to 91.3)
Central Europe, eastern Europe, and central Asia	Central Asia	Georgia	Deaths	Age-standardized	14.3 (12.3 to 20.9)	10.7 (8.6 to 19.6)	22 (19.3 to 25)	15.6 (12.4 to 21.3)	9.9 (7.1 to 17.7)	25 (20 to 30.6)	9.2 (-11.8 to 33.8)	-7.7 (-29.3 to 17.6)	13.6 (-10.8 to 41.7)
Central Europe, eastern Europe, and central Asia	Central Asia	Georgia	DALYs	All Ages	19,395 (16,997 to 24,418)	8,841 (7,330 to 13,804)	10,553 (9,270 to 11,990)	24,689 (20,728 to 30,835)	10,173 (8,102 to 14,932)	14,516 (11,988 to 17,606)	27.3 (7.2 to 48.7)	15.1 (-2.8 to 35.9)	37.5 (10.5 to 68.6)
Central Europe, eastern Europe, and central Asia	Central Asia	Georgia	DALYs	Age-standardized	334 (293.9 to 424.4)	255.9 (214.2 to 390.9)	467.9 (415.3 to 525.3)	433.3 (365.4 to 533)	292.2 (235.3 to 413.6)	630.6 (525.3 to 762.2)	29.7 (9.7 to 51.1)	14.2 (-2.5 to 34.6)	34.8 (10.1 to 62.7)
Central Europe, eastern Europe, and central Asia	Central Asia	Georgia	YLLs	All Ages	14,183 (12,149 to 19,239)	6,007 (4,809 to 11,009)	8,176 (7,054 to 9,587)	17,342 (13,763 to 23,078)	6,222 (4,523 to 10,913)	11,120 (8,875 to 13,917)	22.3 (-3.2 to 51.5)	3.6 (-20.3 to 32.5)	36 (2.9 to 76.5)
Central Europe, eastern Europe, and central Asia	Central Asia	Georgia	YLLs	Age-standardized	245.3 (211.8 to 337.1)	170.9 (138.3 to 305.9)	372.1 (325 to 428.2)	300.1 (239.9 to 391.8)	169.7 (126 to 287.1)	481.7 (385.9 to 597.9)	22.3 (-2.2 to 50.8)	-0.7 (-22.3 to 26.7)	29.4 (0 to 64.2)
Central Europe, eastern Europe, and central Asia	Central Asia	Georgia	YLDs	All Ages	5,212 (3,984 to 6,319)	2,834 (2,173 to 3,494)	2,378 (1,791 to 3,013)	7,346 (5,707 to 8,983)	3,950 (3,073 to 4,845)	3,396 (2,478 to 4,334)	41 (30.5 to 51.1)	39.4 (25.7 to 53.1)	42.8 (29.6 to 57.8)
Central Europe, eastern Europe, and central Asia	Central Asia	Georgia	YLDs	Age-standardized	88.7 (68.3 to 107.6)	84.9 (65.7 to 104.4)	95.8 (72.1 to 120.5)	133.2 (102.8 to 161.8)	122.5 (95.2 to 149)	148.9 (110.3 to 189.3)	50.2 (40.5 to 60)	44.3 (31.4 to 58.1)	55.5 (43.5 to 68.3)
Central Europe, eastern Europe, and central Asia	Central Asia	Kazakhstan	Incidence	All Ages	27,007 (26,258 to 27,872)	12,259 (11,678 to 12,898)	14,748 (14,414 to 15,098)	35,947 (34,558 to 37,284)	15,363 (14,478 to 16,209)	20,584 (19,892 to 21,235)	33.1 (29.4 to 36.8)	25.3 (19.3 to 31.2)	39.6 (35.1 to 43.4)
Central Europe, eastern Europe, and central Asia	Central Asia	Kazakhstan	Incidence	Age-standardized	207.3 (200.9 to 214.4)	163.2 (155.8 to 171.1)	288.2 (279.1 to 298.2)	204.1 (196.2 to 212)	155.5 (147 to 163.6)	273.2 (262.6 to 284.6)	-1.5 (-4.4 to 1.3)	-4.8 (-9 to -0.5)	-5.2 (-9 to -2)
Central Europe, eastern Europe, and central Asia	Central Asia	Kazakhstan	Prevalence	All Ages	397,675 (387,352 to 409,104)	211,531 (203,106 to 221,696)	186,144 (182,731 to 189,422)	524,697 (508,006 to 542,490)	252,993 (239,723 to 267,192)	271,703 (264,026 to 278,522)	31.9 (28.1 to 35.5)	19.6 (13.8 to 25.6)	46 (42 to 49.4)
Central Europe, eastern Europe, and central Asia	Central Asia	Kazakhstan	Prevalence	Age-standardized	3057.1 (2968.1 to 3154.1)	2717.2 (2608.7 to 2847.5)	3840.2 (3735.7 to 3951.3)	3052.6 (2948.6 to 3158.8)	2534.7 (2407.5 to 2670.1)	3934.5 (3807 to 4059.4)	-0.1 (-3.1 to 2.8)	-6.7 (-11.3 to -2.1)	2.5 (-0.9 to 5.6)
Central Europe, eastern Europe, and central Asia	Central Asia	Kazakhstan	Deaths	All Ages	5,264 (4,683 to 5,767)	2,081 (1,519 to 2,414)	3,184 (2,892 to 3,465)	8,051 (6,730 to 9,545)	3,164 (2,181 to 3,853)	4,887 (4,043 to 5,921)	52.9 (26.7 to 83.2)	52.1 (9.4 to 85.1)	53.5 (26.5 to 91)
Central Europe, eastern Europe, and central Asia	Central Asia	Kazakhstan	Deaths	Age-standardized	46.5 (41.1 to 51)	28.4 (20.8 to 33)	84 (76.4 to 90.9)	56.7 (47.3 to 67)	36.1 (25 to 43.8)	95.7 (80.1 to 116.2)	22 (1 to 44.7)	27 (-9.6 to 54.5)	13.9 (-4.4 to 41.2)
Central Europe, eastern Europe, and central Asia	Central Asia	Kazakhstan	DALYs	All Ages	139,190 (125,012 to 150,335)	53,960 (41,334 to 60,915)	85,230 (77,824 to 92,639)	190,213 (162,352 to 222,406)	71,344 (54,883 to 83,914)	118,869 (99,444 to 142,827)	36.7 (16.6 to 61.1)	32.2 (8.7 to 53.6)	39.5 (16.3 to 71.7)
Central Europe, eastern Europe, and central Asia	Central Asia	Kazakhstan	DALYs	Age-standardized	1098.1 (986.1 to 1188.4)	698.1 (538.3 to 786.3)	1824.1 (1671.2 to 1974.7)	1163.5 (996.2 to 1355)	737.8 (568.5 to 865.1)	1865.7 (1572.6 to 2227.5)	6 (-9.2 to 23.9)	5.7 (-13.7 to 22.6)	2.3 (-13.8 to 24.2)
Central Europe, eastern Europe, and central Asia	Central Asia	Kazakhstan	YLLs	All Ages	116,303 (103,086 to 127,263)	40,612 (28,350 to 47,179)	75,691 (68,466 to 83,284)	160,535 (132,989 to 192,684)	55,514 (39,617 to 67,960)	105,022 (86,169 to 128,072)	38 (14.4 to 67.9)	36.7 (5.4 to 65.8)	38.8 (13 to 75.2)
Central Europe, eastern Europe, and central Asia	Central Asia	Kazakhstan	YLLs	Age-standardized	924 (818.8 to 1012.2)	527.3 (371.2 to 612.4)	1631.2 (1476.3 to 1776.3)	992.3 (827.7 to 1180.9)	579.8 (407.4 to 706.4)	1668.4 (1382.5 to 2029.7)	7.4 (-10.9 to 28.7)	10 (-15.7 to 33.2)	2.3 (-15.6 to 27)
Central Europe, eastern Europe, and central Asia	Central Asia	Kazakhstan	YLDs	All Ages	22,888 (17,709 to 27,805)	13,348 (10,288 to 16,287)	9,539 (7,178 to 12,020)	29,678 (23,029 to 36,000)	15,831 (12,299 to 19,274)	13,848 (10,260 to 17,634)	29.7 (23.1 to 36.4)	18.6 (10.4 to 27.6)	45.2 (36.3 to 54.4)
Central Europe, eastern Europe, and central Asia	Central Asia	Kazakhstan	YLDs	Age-standardized	174.1 (133.8 to 211.6)	170.8 (131.6 to 208.1)	192.9 (142.2 to 243.5)	171.2 (132.3 to 206.8)	158 (123.5 to 191.2)	197.2 (146.2 to 250.5)	-1.7 (-6.4 to 3.1)	-7.5 (-13.8 to -0.8)	2.2 (-2.8 to 7.6)
Central Europe, eastern Europe, and central Asia	Central Asia	Kyrgyzstan	Incidence	All Ages	8,990 (8,763 to 9,202)	4,940 (4,713 to 5,149)	4,050 (4,011 to 4,074)	10,067 (9,639 to 10,470)	4,319 (4,117 to 4,527)	5,748 (5,472 to 6,001)	12 (8 to 15.6)	-12.6 (-16.9 to -8.5)	41.9 (35.1 to 48.1)
Central Europe, eastern Europe, and central Asia	Central Asia	Kyrgyzstan	Incidence	Age-standardized	292.7 (285.1 to 299.9)	269.6 (258.2 to 281.1)	333.1 (329.5 to 335.4)	213.2 (204 to 221.8)	164.7 (156.4 to 172.6)	277.6 (265.2 to 289.4)	-27.2 (-29.6 to -24.7)	-38.9 (-41.9 to -36.1)	-16.7 (-20.4 to -13.3)
Central Europe, eastern Europe, and central Asia	Central Asia	Kyrgyzstan	Prevalence	All Ages	115,349 (112,489 to 118,304)	68,265 (65,510 to 71,097)	47,084 (46,645 to 47,426)	138,722 (133,002 to 144,148)	64,743 (61,547 to 68,084)	73,979 (70,531 to 77,232)	20.3 (15.3 to 24.6)	-5.2 (-10.4 to -0.1)	57.1 (50 to 64)
Central Europe, eastern Europe, and central Asia	Central Asia	Kyrgyzstan	Prevalence	Age-standardized	3725.9 (3620 to 3833)	3662 (3511.5 to 3816.5)	3876.5 (3832.3 to 3913)	2955.6 (2821.8 to 3080.1)	2426.3 (2303.5 to 2551)	3728.7 (3539.2 to 3912.3)	-20.7 (-23.7 to -17.8)	-33.7 (-37.3 to -30.3)	-3.8 (-8.6 to 0.6)
Central Europe, eastern Europe, and central Asia	Central Asia	Kyrgyzstan	Deaths	All Ages	2,471 (1,863 to 2,735)	1,137 (577 to 1,333)	1,334 (1,216 to 1,452)	1,434 (1,217 to 1,778)	620 (498 to 928)	814 (694 to 952)	-42 (-52.2 to -13.4)	-45.4 (-59.1 to 29.6)	-39 (-48.9 to -27.8)
Central Europe, eastern Europe, and central Asia	Central Asia	Kyrgyzstan	Deaths	Age-standardized	86 (64.2 to 95.2)	60.3 (30.7 to 70.3)	134 (121.8 to 145.5)	39.3 (33.4 to 49)	28.3 (22.7 to 42.1)	56.6 (48.5 to 65.9)	-54.3 (-62.3 to -31.6)	-53 (-64.7 to 9.8)	-57.8 (-64.4 to -50.2)
Central Europe, eastern Europe, and central Asia	Central Asia	Kyrgyzstan	DALYs	All Ages	56,771 (46,653 to 61,764)	24,366 (15,159 to 27,540)	32,406 (29,519 to 35,373)	35,383 (30,648 to 42,816)	14,674 (12,346 to 20,870)	20,708 (17,801 to 24,248)	-37.7 (-47.2 to -14.7)	-39.8 (-51.9 to 19.8)	-36.1 (-46.5 to -24)
Central Europe, eastern Europe, and central Asia	Central Asia	Kyrgyzstan	DALYs	Age-standardized	1861.8 (1518.1 to 2029.8)	1306.2 (804.8 to 1477)	2762.8 (2534.1 to 2999.3)	815 (711.2 to 981.9)	589.3 (495.5 to 830.2)	1137.3 (981.2 to 1327)	-56.2 (-62.8 to -39.8)	-54.9 (-64.1 to -11.4)	-58.8 (-65.1 to -51.7)
Central Europe, eastern Europe, and central Asia	Central Asia	Kyrgyzstan	YLLs	All Ages	50,098 (40,634 to 54,979)	20,115 (11,034 to 23,157)	29,983 (27,142 to 32,880)	27,393 (23,188 to 34,410)	10,522 (8,382 to 16,893)	16,871 (14,154 to 19,891)	-45.3 (-55.4 to -20.2)	-47.7 (-61 to 27.4)	-43.7 (-53.9 to -31.6)
Central Europe, eastern Europe, and central Asia	Central Asia	Kyrgyzstan	YLLs	Age-standardized	1647.8 (1320.4 to 1807.7)	1078.4 (588 to 1243)	2566.6 (2342.9 to 2804.7)	647.7 (551.7 to 807.1)	436 (349.8 to 683.2)	947.4 (804.1 to 1113.5)	-60.7 (-67.7 to -42.7)	-59.6 (-70 to -3.6)	-63.1 (-69.4 to -56)
Central Europe, eastern Europe, and central Asia	Central Asia	Kyrgyzstan	YLDs	All Ages	6,673 (5,124 to 8,028)	4,250 (3,254 to 5,145)	2,423 (1,808 to 3,032)	7,989 (6,154 to 9,746)	4,153 (3,233 to 5,085)	3,837 (2,845 to 4,879)	19.7 (12 to 27.5)	-2.3 (-9.7 to 6.2)	58.4 (46.7 to 70.4)
Central Europe, eastern Europe, and central Asia	Central Asia	Kyrgyzstan	YLDs	Age-standardized	214 (164.5 to 257.3)	227.8 (174.1 to 275.7)	196.2 (145.7 to 246.1)	167.4 (129 to 204.1)	153.3 (117.8 to 186.6)	189.9 (139.3 to 240.7)	-21.8 (-26.1 to -17.3)	-32.7 (-37.2 to -27.8)	-3.2 (-9.2 to 2.7)
Central Europe, eastern Europe, and central Asia	Central Asia	Mongolia	Incidence	All Ages	1,415 (1,344 to 1,492)	677 (642 to 716)	739 (697 to 782)	2,016 (1,889 to 2,154)	906 (839 to 983)	1,110 (1,037 to 1,182)	42.4 (36.4 to 48.6)	33.8 (26.1 to 41.2)	50.3 (42.6 to 58.5)
Central Europe, eastern Europe, and central Asia	Central Asia	Mongolia	Incidence	Age-standardized	112.4 (106.2 to 119)	98 (92.1 to 104.5)	131.2 (123.8 to 139.1)	84.3 (79.3 to 89.7)	69.6 (64.7 to 75)	107 (100.8 to 113.4)	-25 (-27.8 to -22.1)	-29 (-32.5 to -25)	-18.5 (-22.4 to -14.2)
Central Europe, eastern Europe, and central Asia	Central Asia	Mongolia	Prevalence	All Ages	20,577 (19,548 to 21,624)	10,392 (9,833 to 10,929)	10,185 (9,579 to 10,781)	33,038 (30,410 to 35,625)	16,433 (14,945 to 18,003)	16,604 (15,298 to 17,917)	60.6 (49.9 to 71.3)	58.1 (44.4 to 71.2)	63 (51.6 to 74.1)
Central Europe, eastern Europe, and central Asia	Central Asia	Mongolia	Prevalence	Age-standardized	1641.3 (1549.7 to 1737.3)	1546.6 (1455.9 to 1639.2)	1785.3 (1677.8 to 1896.9)	1266.2 (1179.5 to 1354.7)	1139.6 (1047.1 to 1238.2)	1469.4 (1371.7 to 1566.8)	-22.9 (-26.6 to -18.6)	-26.3 (-31.5 to -20.9)	-17.7 (-22.2 to -13.2)
Central Europe, eastern Europe, and central Asia	Central Asia	Mongolia	Deaths	All Ages	322 (259 to 383)	145 (105 to 184)	177 (138 to 222)	307 (243 to 402)	129 (98 to 179)	178 (139 to 240)	-4.7 (-28.8 to 37.4)	-11.1 (-37.1 to 37.9)	0.6 (-28.6 to 45.6)
Central Europe, eastern Europe, and central Asia	Central Asia	Mongolia	Deaths	Age-standardized	36.3 (28.6 to 43.1)	27.9 (20 to 34.9)	50.2 (39.7 to 61.4)	19.6 (16 to 24.7)	14.9 (11.6 to 19.5)	27.3 (22 to 34.5)	-46 (-58.3 to -26.6)	-46.5 (-61.1 to -20.5)	-45.6 (-60.1 to -24.3)
Central Europe, eastern Europe, and central Asia	Central Asia	Mongolia	DALYs	All Ages	8,736 (7,119 to 10,397)	4,156 (3,102 to 5,251)	4,580 (3,602 to 5,660)	9,153 (7,442 to 11,796)	3,824 (3,111 to 5,127)	5,329 (4,202 to 7,123)	4.8 (-20.2 to 45.5)	-8 (-33.2 to 40)	16.4 (-14.5 to 65.8)
Central Europe, eastern Europe, and central Asia	Central Asia	Mongolia	DALYs	Age-standardized	769.8 (635 to 902.7)	633.4 (493.8 to 770.2)	972.4 (778.2 to 1189.6)	411.3 (339.2 to 516.7)	313.5 (253.8 to 406.6)	551.5 (445.8 to 713.2)	-46.6 (-58.4 to -29.6)	-50.5 (-63.8 to -30.9)	-43.3 (-57.3 to -22.9)
Central Europe, eastern Europe, and central Asia	Central Asia	Mongolia	YLLs	All Ages	7,516 (6,028 to 9,185)	3,471 (2,422 to 4,556)	4,045 (3,119 to 5,146)	7,198 (5,518 to 9,871)	2,737 (2,032 to 4,028)	4,461 (3,379 to 6,236)	-4.2 (-31.4 to 43.4)	-21.1 (-47.5 to 35.7)	10.3 (-24.3 to 65.3)
Central Europe, eastern Europe, and central Asia	Central Asia	Mongolia	YLLs	Age-standardized	675 (548.6 to 803.7)	534.2 (399.8 to 675.9)	881.1 (690.8 to 1095.4)	338.2 (266.7 to 440.5)	240 (182.5 to 333.2)	476.5 (375 to 631.2)	-49.9 (-63.1 to -30.8)	-55.1 (-68.2 to -31.8)	-45.9 (-60.9 to -23.6)

Chronic obstructive pulmonary disease

7 GBD super region	21 GBD region	Location	Measure	Age	1990			2019			Percent change (1990 to 2019)		
					Both	Female	Male	Both	Female	Male	Both	Female	Male
Central Europe, eastern Europe, and central Asia	Central Asia	Mongolia	YLDs	All Ages	1,220 (950 to 1,487)	686 (541 to 833)	534 (398 to 682)	[illegible]	[illegible]	[illegible]	[illegible]	[illegible]	[illegible]
Central Europe, eastern Europe, and central Asia	Central Asia	Mongolia	YLDs	Age-standardized	94.8 (73.4 to 115.2)	99.3 (78.6 to 120.8)	91.3 (68.2 to 116.6)	[illegible]	[illegible]	[illegible]	[illegible]	[illegible]	[illegible]
Central Europe, eastern Europe, and central Asia	Central Asia	Tajikistan	Incidence	All Ages	4,562 (4,362 to 4,775)	2,176 (2,071 to 2,292)	2,387 (2,278 to 2,502)	[illegible]	[illegible]	[illegible]	[illegible]	[illegible]	[illegible]
Central Europe, eastern Europe, and central Asia	Central Asia	Tajikistan	Incidence	Age-standardized	135.9 (129.2 to 143)	123.5 (117 to 130.9)	156.5 (148.2 to 165.2)	[illegible]	[illegible]	[illegible]	[illegible]	[illegible]	[illegible]
Central Europe, eastern Europe, and central Asia	Central Asia	Tajikistan	Prevalence	All Ages	73,630 (70,600 to 76,891)	39,212 (37,426 to 41,023)	34,418 (32,803 to 36,227)	[illegible]	[illegible]	[illegible]	[illegible]	[illegible]	[illegible]
Central Europe, eastern Europe, and central Asia	Central Asia	Tajikistan	Prevalence	Age-standardized	2294.5 (2183 to 2411.5)	2281.7 (2168.2 to 2398.7)	2362.2 (2235.6 to 2504.6)	[illegible]	[illegible]	[illegible]	[illegible]	[illegible]	[illegible]
Central Europe, eastern Europe, and central Asia	Central Asia	Tajikistan	Deaths	All Ages	1,037 (860 to 1,218)	523 (396 to 692)	514 (423 to 625)	[illegible]	[illegible]	[illegible]	[illegible]	[illegible]	[illegible]
Central Europe, eastern Europe, and central Asia	Central Asia	Tajikistan	Deaths	Age-standardized	38.2 (31.5 to 45.4)	33.1 (25 to 43.6)	46.1 (37.8 to 56.7)	[illegible]	[illegible]	[illegible]	[illegible]	[illegible]	[illegible]
Central Europe, eastern Europe, and central Asia	Central Asia	Tajikistan	DALYs	All Ages	28,280 (23,882 to 31,431)	14,799 (11,981 to 17,014)	13,482 (11,512 to 16,026)	[illegible]	[illegible]	[illegible]	[illegible]	[illegible]	[illegible]
Central Europe, eastern Europe, and central Asia	Central Asia	Tajikistan	DALYs	Age-standardized	949.5 (806.6 to 1070)	886.7 (722.7 to 1022.9)	1057.7 (897 to 1267.2)	[illegible]	[illegible]	[illegible]	[illegible]	[illegible]	[illegible]
Central Europe, eastern Europe, and central Asia	Central Asia	Tajikistan	YLLs	All Ages	23,930 (19,840 to 27,055)	12,244 (9,426 to 14,345)	11,686 (9,733 to 14,202)	[illegible]	[illegible]	[illegible]	[illegible]	[illegible]	[illegible]
Central Europe, eastern Europe, and central Asia	Central Asia	Tajikistan	YLLs	Age-standardized	815.9 (689.3 to 926.4)	740 (577.7 to 874.8)	936.4 (779.9 to 1140.7)	[illegible]	[illegible]	[illegible]	[illegible]	[illegible]	[illegible]
Central Europe, eastern Europe, and central Asia	Central Asia	Tajikistan	YLDs	All Ages	4,351 (3,384 to 5,295)	2,555 (1,981 to 3,128)	1,796 (1,344 to 2,254)	[illegible]	[illegible]	[illegible]	[illegible]	[illegible]	[illegible]
Central Europe, eastern Europe, and central Asia	Central Asia	Tajikistan	YLDs	Age-standardized	133.7 (102.9 to 162.9)	146.7 (114 to 179.6)	121.3 (89.9 to 153)	[illegible]	[illegible]	[illegible]	[illegible]	[illegible]	[illegible]
Central Europe, eastern Europe, and central Asia	Central Asia	Turkmenistan	Incidence	All Ages	3,541 (3,406 to 3,674)	1,690 (1,614 to 1,768)	1,851 (1,781 to 1,925)	[illegible]	[illegible]	[illegible]	[illegible]	[illegible]	[illegible]
Central Europe, eastern Europe, and central Asia	Central Asia	Turkmenistan	Incidence	Age-standardized	164.5 (157.9 to 171)	142.8 (136.1 to 149.6)	193.1 (185.1 to 201.4)	[illegible]	[illegible]	[illegible]	[illegible]	[illegible]	[illegible]
Central Europe, eastern Europe, and central Asia	Central Asia	Turkmenistan	Prevalence	All Ages	54,792 (52,799 to 56,772)	27,874 (26,579 to 29,132)	26,919 (25,913 to 27,879)	[illegible]	[illegible]	[illegible]	[illegible]	[illegible]	[illegible]
Central Europe, eastern Europe, and central Asia	Central Asia	Turkmenistan	Prevalence	Age-standardized	2548.3 (2440.3 to 2649)	2320.1 (2202.4 to 2433.9)	2904.7 (2774.3 to 3035.1)	[illegible]	[illegible]	[illegible]	[illegible]	[illegible]	[illegible]
Central Europe, eastern Europe, and central Asia	Central Asia	Turkmenistan	Deaths	All Ages	596 (462 to 670)	274 (154 to 334)	322 (296 to 351)	[illegible]	[illegible]	[illegible]	[illegible]	[illegible]	[illegible]
Central Europe, eastern Europe, and central Asia	Central Asia	Turkmenistan	Deaths	Age-standardized	37.4 (28.5 to 42.1)	28.4 (16 to 34.4)	52.6 (48.1 to 57.2)	[illegible]	[illegible]	[illegible]	[illegible]	[illegible]	[illegible]
Central Europe, eastern Europe, and central Asia	Central Asia	Turkmenistan	DALYs	All Ages	16,783 (13,712 to 18,671)	7,499 (4,817 to 9,047)	9,285 (8,508 to 10,159)	[illegible]	[illegible]	[illegible]	[illegible]	[illegible]	[illegible]
Central Europe, eastern Europe, and central Asia	Central Asia	Turkmenistan	DALYs	Age-standardized	846.8 (688.2 to 939.2)	652.4 (423.6 to 780.4)	1136.8 (1050.4 to 1231.4)	[illegible]	[illegible]	[illegible]	[illegible]	[illegible]	[illegible]
Central Europe, eastern Europe, and central Asia	Central Asia	Turkmenistan	YLLs	All Ages	13,571 (10,574 to 15,290)	5,694 (3,044 to 7,134)	7,877 (7,149 to 8,627)	[illegible]	[illegible]	[illegible]	[illegible]	[illegible]	[illegible]
Central Europe, eastern Europe, and central Asia	Central Asia	Turkmenistan	YLLs	Age-standardized	700.4 (548.4 to 787.2)	505.2 (276.6 to 622.2)	988.2 (908.6 to 1075.3)	[illegible]	[illegible]	[illegible]	[illegible]	[illegible]	[illegible]
Central Europe, eastern Europe, and central Asia	Central Asia	Turkmenistan	YLDs	All Ages	3,212 (2,508 to 3,896)	1,805 (1,397 to 2,204)	1,407 (1,042 to 1,776)	[illegible]	[illegible]	[illegible]	[illegible]	[illegible]	[illegible]
Central Europe, eastern Europe, and central Asia	Central Asia	Turkmenistan	YLDs	Age-standardized	146.4 (113.9 to 178.2)	147.2 (112.5 to 181.9)	148.5 (110.8 to 187.6)	[illegible]	[illegible]	[illegible]	[illegible]	[illegible]	[illegible]
[illegible]	[illegible]	[illegible]	[illegible]	[illegible]	[illegible]	[illegible]	[illegible]	[illegible]	[illegible]	[illegible]	[illegible]	[illegible]	[illegible]

Chronic obstructive pulmonary disease

7 GBD super region	21 GBD region	Location	Measure	Age	1990			2019			Percent change (1990 to 2019)		
					Both	Female	Male	Both	Female	Male	Both	Female	Male
Central Europe, eastern Europe, and central Asia	Eastern Europe	Republic of Moldova	YLLs	Age-standardized	839.2 (700.1 to 898.2)	462.9 (258.1 to 524.9)	1389.8 (1301.9 to 1477.3)	280.9 (235.6 to 350.2)	146.4 (113.3 to 236.8)	476.9 (403.8 to 561.4)	-66.5 (-72.6 to -53)	-68.4 (-76.4 to -23.8)	-65.7 (-71.4 to -59)
Central Europe, eastern Europe, and central Asia	Eastern Europe	Republic of Moldova	YLDs	All Ages	7,052 (5,503 to 8,564)	4,313 (3,415 to 5,225)	2,739 (1,885 to 3,553)	5,434 (4,185 to 6,622)	2,833 (2,241 to 3,458)	2,601 (1,775 to 3,451)	-23 (-28 to -18.2)	-34.3 (-39.7 to -28.2)	-5 (-12.2 to 2.4)
Central Europe, eastern Europe, and central Asia	Eastern Europe	Republic of Moldova	YLDs	Age-standardized	162.7 (126.8 to 196.5)	172.2 (136 to 208.1)	153.6 (107.9 to 199.6)	102.2 (79 to 124.8)	95.3 (75.4 to 116.1)	115 (79.9 to 152.4)	-37.1 (-41.1 to -33.3)	-44.6 (-49.5 to -39.4)	-25.1 (-30.3 to -18.9)
Central Europe, eastern Europe, and central Asia	Eastern Europe	Russian Federation	Incidence	All Ages	259,145 (235,324 to 282,163)	113,617 (100,917 to 127,460)	145,528 (133,935 to 157,320)	248,756 (225,799 to 272,730)	98,737 (88,224 to 110,508)	150,020 (135,376 to 165,375)	-4 (-6.6 to -1.2)	-13.1 (-17.4 to -8)	3.1 (-0.4 to 7.3)
Central Europe, eastern Europe, and central Asia	Eastern Europe	Russian Federation	Incidence	Age-standardized	154.2 (141.5 to 167.4)	108.9 (98.9 to 119.7)	234.2 (214.3 to 252.6)	118.4 (109.1 to 128.4)	77.9 (71.1 to 85.7)	175.9 (160.5 to 191.9)	-23.2 (-24.8 to -21.6)	-28.5 (-30.9 to -25.8)	-24.9 (-27.2 to -22.4)
Central Europe, eastern Europe, and central Asia	Eastern Europe	Russian Federation	Prevalence	All Ages	3,819,371 (3,549,316 to 4,102,811)	1,818,821 (1,657,559 to 1,995,138	2,000,550 (1,870,091 to 2,130,636)	3,921,088 (3,641,585 to 4,227,149)	1,658,534 (1,520,030 to 1,817,996	2,262,554 (2,095,690 to 2,445,037)	2.7 (0.8 to 4.5)	-8.8 (-12 to -5.2)	13.1 (9.7 to 16.6)
Central Europe, eastern Europe, and central Asia	Eastern Europe	Russian Federation	Prevalence	Age-standardized	2229 (2074 to 2393.2)	1714.6 (1577.9 to 1865.6)	3352.4 (3114 to 3584.8)	1823.8 (1699.8 to 1960.3)	1323.5 (1219.2 to 1436.6)	2674.7 (2477.9 to 2886.1)	-18.2 (-19.6 to -16.7)	-22.8 (-25.3 to -20.3)	-20.2 (-22.5 to -17.7)
Central Europe, eastern Europe, and central Asia	Eastern Europe	Russian Federation	Deaths	All Ages	47,723 (39,012 to 51,242)	17,912 (9,618 to 19,979)	29,811 (28,986 to 31,472)	36,483 (31,203 to 43,147)	13,020 (10,605 to 18,557)	23,463 (19,417 to 28,102)	-23.6 (-34.9 to 3.2)	-27.3 (-43 to 57.4)	-21.3 (-34.3 to -6.2)
Central Europe, eastern Europe, and central Asia	Eastern Europe	Russian Federation	Deaths	Age-standardized	28.7 (23 to 30.9)	15.7 (8.4 to 17.5)	61.7 (59.3 to 65.2)	15.4 (13.2 to 18.3)	8 (6.5 to 11.4)	30.1 (25.2 to 35.8)	-46.3 (-54.3 to -26.6)	-49 (-60 to 9.6)	-51.2 (-58.8 to -42.5)
Central Europe, eastern Europe, and central Asia	Eastern Europe	Russian Federation	DALYs	All Ages	1,182,475 (1,023,412 to 1,268,712)	418,530 (270,791 to 465,566)	763,945 (731,532 to 807,777)	880,444 (765,845 to 1,008,291)	308,956 (263,875 to 387,403)	571,488 (484,370 to 673,245)	-25.5 (-34.8 to -8.3)	-26.2 (-37.7 to 26.3)	-25.2 (-36.1 to -12.6)
Central Europe, eastern Europe, and central Asia	Eastern Europe	Russian Federation	DALYs	Age-standardized	666.5 (573.1 to 715.1)	368.5 (241.5 to 411.5)	1289.4 (1233.6 to 1359.4)	386.6 (336.7 to 442.2)	223.4 (192.1 to 276.9)	667.9 (568.1 to 783)	-42 (-49.2 to -28.5)	-39.4 (-48.2 to 0.6)	-48.2 (-55.4 to -39.8)
Central Europe, eastern Europe, and central Asia	Eastern Europe	Russian Federation	YLLs	All Ages	964,988 (818,598 to 1,035,639)	292,948 (151,740 to 328,350)	672,040 (652,973 to 711,419)	661,553 (559,181 to 784,519)	194,203 (157,137 to 278,780)	467,350 (385,066 to 566,702)	-31.4 (-42.4 to -10.3)	-33.7 (-48.9 to 50.9)	-30.5 (-42.7 to -15.8)
Central Europe, eastern Europe, and central Asia	Eastern Europe	Russian Federation	YLLs	Age-standardized	539 (452.7 to 579)	248.6 (128.6 to 279.2)	1138.2 (1104.2 to 1203.1)	283.1 (239.4 to 335.8)	129.1 (104.4 to 185.5)	545.1 (450.7 to 657.3)	-47.5 (-55.9 to -31)	-48.1 (-60.2 to 18.1)	-52.1 (-60.3 to -42.5)
Central Europe, eastern Europe, and central Asia	Eastern Europe	Russian Federation	YLDs	All Ages	217,488 (167,459 to 263,421)	125,582 (96,949 to 151,417)	91,905 (66,870 to 117,158)	218,891 (167,577 to 265,629)	114,753 (89,296 to 138,079)	104,138 (74,887 to 134,485)	0.6 (-2.1 to 3.3)	-8.6 (-11.9 to -4.9)	13.3 (8.6 to 17.9)
Central Europe, eastern Europe, and central Asia	Eastern Europe	Russian Federation	YLDs	Age-standardized	127.5 (98.8 to 153.8)	119.9 (93.7 to 144)	151.2 (109.5 to 193.5)	103.5 (80 to 125.3)	94.3 (74.1 to 112.8)	122.8 (88.7 to 157.1)	-18.9 (-21 to -16.7)	-21.3 (-24.2 to -18.3)	-18.8 (-21.5 to -16)
Central Europe, eastern Europe, and central Asia	Eastern Europe	Ukraine	Incidence	All Ages	162,081 (147,298 to 175,575)	90,425 (79,462 to 100,706)	71,656 (66,020 to 76,057)	79,840 (71,493 to 89,095)	31,665 (27,638 to 36,573)	48,175 (43,122 to 53,923)	-50.7 (-53.8 to -46.8)	-65 (-68.7 to -59.8)	-32.8 (-38.8 to -26.4)
Central Europe, eastern Europe, and central Asia	Eastern Europe	Ukraine	Incidence	Age-standardized	241.9 (222.5 to 259.9)	216.8 (195.9 to 237.3)	289.7 (270.8 to 305.6)	120 (109.3 to 131.8)	77.2 (69.1 to 86.3)	182.4 (165 to 202.4)	-50.4 (-53.2 to -47.2)	-64.4 (-67.6 to -60.1)	-37.1 (-42.1 to -31.8)
Central Europe, eastern Europe, and central Asia	Eastern Europe	Ukraine	Prevalence	All Ages	2,326,649 (2,161,765 to 2,500,178)	1,398,772 (1,273,219 to 1,526,683	927,878 (864,838 to 981,974)	1,284,752 (1,180,459 to 1,402,555)	525,008 (475,027 to 580,102)	759,744 (690,568 to 837,178)	-44.8 (-48 to -41.1)	-62.5 (-65.9 to -57.9)	-18.1 (-24.2 to -11.5)
Central Europe, eastern Europe, and central Asia	Eastern Europe	Ukraine	Prevalence	Age-standardized	3451.3 (3219.3 to 3691.4)	3270.2 (3010 to 3546.4)	3898.1 (3631.8 to 4130.4)	1878.7 (1736.3 to 2032.2)	1317.6 (1196 to 1441.5)	2817.2 (2579.3 to 3086.4)	-45.6 (-48.8 to -42.2)	-59.7 (-63.3 to -55.3)	-27.7 (-33.1 to -22.2)
Central Europe, eastern Europe, and central Asia	Eastern Europe	Ukraine	Deaths	All Ages	30,550 (23,854 to 33,289)	11,905 (5,910 to 13,915)	18,645 (17,355 to 19,903)	11,360 (9,083 to 17,014)	3,989 (2,713 to 9,344)	7,371 (5,836 to 9,105)	-62.8 (-71.3 to -34.8)	-66.5 (-78.5 to 27.5)	-60.5 (-69.3 to -50.9)
Central Europe, eastern Europe, and central Asia	Eastern Europe	Ukraine	Deaths	Age-standardized	45.4 (35 to 49.6)	26.1 (13 to 30.6)	87.5 (81.4 to 93.7)	14.8 (11.9 to 22.1)	7.6 (5.2 to 17.8)	27.8 (22.2 to 34)	-67.4 (-74.9 to -42.9)	-70.7 (-81 to 11.2)	-68.2 (-75 to -61.1)
Central Europe, eastern Europe, and central Asia	Eastern Europe	Ukraine	DALYs	All Ages	730,890 (615,390 to 789,357)	287,560 (190,848 to 325,686)	443,331 (412,396 to 474,385)	286,978 (238,049 to 379,419)	97,203 (74,876 to 181,701)	189,775 (151,502 to 231,209)	-60.7 (-68.1 to -42.3)	-66.2 (-74.7 to -15.5)	-57.2 (-66 to -47.4)
Central Europe, eastern Europe, and central Asia	Eastern Europe	Ukraine	DALYs	Age-standardized	1042.2 (875.3 to 1126.8)	639.4 (431.2 to 723)	1792.6 (1676.4 to 1912.9)	397.7 (331.4 to 518.9)	221 (172.1 to 391.3)	681 (549.1 to 825.3)	-61.8 (-69 to -44.8)	-65.4 (-73.7 to -18.4)	-62 (-69.6 to -53.7)
Central Europe, eastern Europe, and central Asia	Eastern Europe	Ukraine	YLLs	All Ages	593,072 (483,427 to 641,630)	192,189 (96,608 to 221,969)	400,884 (372,435 to 430,556)	215,413 (169,990 to 309,179)	60,625 (41,474 to 146,196)	154,788 (120,982 to 194,594)	-63.7 (-72.3 to -40.4)	-68.5 (-79.8 to 21.1)	-61.4 (-70.6 to -50.9)
Central Europe, eastern Europe, and central Asia	Eastern Europe	Ukraine	YLLs	Age-standardized	837.2 (677.4 to 905.7)	413.5 (207.4 to 477.6)	1616.4 (1506.8 to 1724.3)	291.2 (229.9 to 414.4)	126.3 (86.3 to 301.4)	551.3 (436.7 to 688.5)	-65.2 (-73.4 to -43.6)	-69.5 (-80.6 to 15.9)	-65.9 (-73.7 to -57.2)
Central Europe, eastern Europe, and central Asia	Eastern Europe	Ukraine	YLDs	All Ages	137,818 (106,854 to 166,925)	95,371 (73,613 to 117,246)	42,447 (30,593 to 54,856)	71,565 (55,076 to 88,031)	36,578 (28,564 to 44,647)	34,987 (25,034 to 45,017)	-48.1 (-52.3 to -43.6)	-61.6 (-65.5 to -56.9)	-17.6 (-25 to -10.1)
Central Europe, eastern Europe, and central Asia	Eastern Europe	Ukraine	YLDs	Age-standardized	205 (159.5 to 247.7)	225.9 (176.6 to 275.2)	176.2 (127.7 to 225.7)	106.6 (82.1 to 131)	94.7 (74.5 to 114.5)	129.7 (93.2 to 166.4)	-48 (-52.3 to -43.1)	-58.1 (-62.5 to -52.9)	-26.4 (-32.7 to -19.6)
		High-income	Incidence	All Ages	2,373,738 (2,243,180 to 2,493,671)	1,084,995 (1,011,321 to 1,158,396	1,288,743 (1,227,861 to 1,341,704)	4,166,240 (3,967,603 to 4,344,215)	2,051,375 (1,951,767 to 2,148,953	2,114,865 (2,012,850 to 2,203,467)	75.5 (72.3 to 79.1)	89.1 (83.2 to 95.5)	64.1 (61.2 to 67.3)
		High-income	Incidence	Age-standardized	205.5 (195.3 to 215.4)	165.5 (155.5 to 175.2)	258.1 (246.9 to 268)	211 (201.7 to 219.8)	189.1 (180.6 to 197.2)	236.2 (225.9 to 245.5)	2.7 (1.4 to 4.2)	14.3 (11.5 to 17.4)	-8.5 (-9.7 to -7.3)
		High-income	Prevalence	All Ages	34,287,771 (32,422,304 to 36,062,566	16,705,363 (15,662,236 to 17,714,192	17,582,408 (16,758,325 to 18,343,526	58,645,242 (56,052,138 to 61,288,904	29,743,869 (28,447,218 to 31,102,992	28,901,373 (27,581,357 to 30,195,032	71 (68.4 to 74.1)	78 (73.8 to 83.2)	64.4 (61.8 to 67.4)
		High-income	Prevalence	Age-standardized	2940.6 (2786.7 to 3088.7)	2495 (2347.4 to 2643.7)	3603.2 (3441.5 to 3753.3)	2863.4 (2739.4 to 2989.1)	2630.2 (2517.1 to 2750)	3164.7 (3023.8 to 3302)	-2.6 (-3.8 to -1.2)	5.4 (3.3 to 8)	-12.2 (-13.3 to -10.9)
		High-income	Deaths	All Ages	292,549 (272,993 to 320,375)	111,401 (98,279 to 137,668)	181,148 (172,802 to 186,142)	507,281 (436,990 to 557,981)	232,570 (170,342 to 262,954)	274,711 (246,908 to 301,510)	73.4 (55.1 to 88.8)	108.8 (51.2 to 134.3)	51.7 (40.7 to 64.7)
		High-income	Deaths	Age-standardized	23.8 (22.2 to 26)	14.7 (13 to 18)	39.6 (37.4 to 40.9)	20.6 (18 to 22.5)	16 (11.8 to 17.8)	27.4 (24.7 to 30.1)	-13.4 (-21.9 to -6.4)	9.1 (-20 to 21)	-30.8 (-35.2 to -25.1)
		High-income	DALYs	All Ages	7,280,251 (6,769,658 to 7,763,691)	2,957,073 (2,683,517 to 3,323,572	4,323,178 (4,067,183 to 4,545,069)	11,435,787 (10,435,329 to 12,288,993	5,319,520 (4,405,601 to 5,799,746	6,116,267 (5,634,962 to 6,628,504)	57.1 (48 to 65)	79.9 (50.7 to 91.6)	41.5 (35.2 to 48.9)
		High-income	DALYs	Age-standardized	605.4 (562.8 to 646.4)	425.5 (385.8 to 476.1)	879.8 (827.5 to 925.7)	535.5 (491.1 to 574.8)	453.3 (381.4 to 492.6)	644.3 (593.9 to 697)	-11.5 (-16.1 to -7.6)	6.5 (-9 to 12.3)	-26.8 (-29.7 to -23.2)
		High-income	YLLs	All Ages	4,786,612 (4,544,678 to 5,172,982)	1,730,357 (1,572,678 to 2,081,816	3,056,255 (2,948,980 to 3,129,147)	7,163,076 (6,380,173 to 7,752,946)	3,158,327 (2,340,049 to 3,489,510	4,004,749 (3,694,944 to 4,359,465)	49.6 (36.9 to 61)	82.5 (35.8 to 101.5)	31 (22.9 to 41.5)
		High-income	YLLs	Age-standardized	390.9 (371.1 to 422.3)	240.4 (219.8 to 287.2)	620.6 (597.2 to 636.5)	324.3 (291.9 to 349.2)	257.7 (192.7 to 282.3)	412.3 (382 to 448.1)	-17 (-23.7 to -11.3)	7.2 (-18.8 to 16.9)	-33.6 (-37.3 to -28.5)
		High-income	YLDs	All Ages	2,493,639 (2,038,084 to 2,846,707)	1,226,716 (997,179 to 1,417,892)	1,266,923 (1,033,201 to 1,471,023)	4,272,712 (3,533,580 to 4,851,231)	2,161,193 (1,775,428 to 2,491,228	2,111,518 (1,724,365 to 2,443,436)	71.3 (67 to 76.4)	76.2 (70.2 to 83.2)	66.7 (62.1 to 71.5)
		High-income	YLDs	Age-standardized	214.5 (175.2 to 244.7)	185.1 (150.6 to 214.5)	259.1 (211.7 to 299.9)	211.2 (174.4 to 239.9)	195.6 (161.1 to 225.4)	232 (190.4 to 267.4)	-1.5 (-3.5 to 0.8)	5.7 (2.9 to 9.1)	-10.5 (-12.3 to -8.4)
		High-income Asia Pacific	Incidence	All Ages	279,248 (255,582 to 303,871)	109,200 (98,666 to 121,361)	170,048 (154,922 to 185,294)	572,483 (526,753 to 618,868)	221,753 (197,009 to 247,076)	350,730 (320,829 to 378,859)	105 (93.4 to 117.4)	103.1 (85.4 to 120.8)	106.3 (94.9 to 118.7)
		High-income Asia Pacific	Incidence	Age-standardized	146.7 (135 to 159.2)	103.2 (93.5 to 114.1)	205.1 (188.7 to 222)	124 (115.1 to 133.1)	79.1 (72.6 to 86.2)	176.4 (162.8 to 189.5)	-15.5 (-18 to -13.1)	-23.4 (-27.2 to -19.7)	-14 (-17 to -10.8)
		High-income Asia Pacific	Prevalence	All Ages	3,599,283 (3,292,235 to 3,929,485)	1,507,615 (1,365,313 to 1,659,284	2,091,667 (1,908,829 to 2,290,673)	6,581,036 (6,084,156 to 7,107,505)	2,565,849 (2,347,854 to 2,813,303	4,015,186 (3,671,987 to 4,377,051)	82.8 (74.6 to 93.1)	70.2 (59.5 to 82.9)	92 (81.6 to 103.7)
		High-income Asia Pacific	Prevalence	Age-standardized	1887.8 (1734.4 to 2059.4)	1417.8 (1288.5 to 1555.8)	2598.3 (2376.4 to 2832.5)	1500.7 (1384.2 to 1621.3)	1092.6 (991.2 to 1199)	2054.4 (1888.4 to 2222.5)	-20.5 (-23 to -17.5)	-22.9 (-26.7 to -18.7)	-20.9 (-24.2 to -17.3)
		High-income Asia Pacific	Deaths	All Ages	24,348 (22,148 to 26,103)	8,871 (6,931 to 9,943)	15,477 (14,404 to 16,467)	49,303 (37,721 to 63,059)	17,589 (12,411 to 24,753)	31,714 (25,091 to 38,983)	102.5 (64.5 to 158.4)	98.3 (50.3 to 192.7)	104.9 (70.9 to 143.1)
		High-income Asia Pacific	Deaths	Age-standardized	14 (12.5 to 15.1)	8.4 (6.5 to 9.4)	23.7 (21.7 to 25.3)	8.2 (6.4 to 10.4)	4.5 (3.3 to 6.2)	14 (11.1 to 17.2)	-41.8 (-51.4 to -25.6)	-46.9 (-57.8 to -20.8)	-40.9 (-49.8 to -30.2)
		High-income Asia Pacific	DALYs	All Ages	651,853 (594,512 to 705,280)	225,062 (193,461 to 250,889)	426,792 (390,434 to 463,985)	1,071,208 (912,742 to 1,249,683)	346,057 (281,018 to 421,937)	725,151 (618,496 to 838,245)	64.3 (48.7 to 88.9)	53.8 (34.6 to 93.5)	69.9 (54.4 to 89.7)
		High-income Asia Pacific	DALYs	Age-standardized	342.5 (312.8 to 370.1)	206.7 (176.9 to 230.6)	548.9 (503 to 595.5)	224.5 (193.6 to 258.4)	130.9 (109.4 to 156.7)	351.4 (301.7 to 404.5)	-34.4 (-39.5 to -25.6)	-36.7 (-42.2 to -22)	-36 (-41.2 to -29.4)
		High-income Asia Pacific	YLLs	All Ages	377,003 (348,002 to 401,461)	126,519 (100,865 to 141,159)	250,484 (236,687 to 266,900)	573,451 (454,576 to 731,562)	183,583 (136,464 to 256,177)	389,868 (316,551 to 481,940)	52.1 (26.6 to 93.6)	45.1 (13.6 to 113.6)	55.6 (32 to 86.8)
		High-income Asia Pacific	YLLs	Age-standardized	199.3 (183.6 to 212.8)	113.8 (90.7 to 126.8)	331.7 (309.5 to 353.2)	108.8 (88.6 to 138.7)	58.4 (45.3 to 82.6)	178.6 (146.3 to 220.7)	-45.4 (-53.4 to -30.5)	-48.6 (-57.5 to -21.8)	-46.1 (-53.7 to -35.4)
		High-income Asia Pacific	YLDs	All Ages	274,850 (223,167 to 322,737)	98,543 (77,435 to 119,144)	176,307 (141,403 to 208,802)	497,757 (405,346 to 579,260)	162,474 (126,869 to 196,514)	335,283 (266,800 to 399,081)	81.1 (71.5 to 91.3)	64.9 (51.7 to 79.4)	90.2 (78.1 to 102.7)
		High-income Asia Pacific	YLDs	Age-standardized	143.1 (116.2 to 167.7)	92.9 (73.4 to 112.6)	217.2 (174.2 to 256.6)	115.7 (94.5 to 135.8)	72.5 (57.1 to 87.8)	172.8 (138.8 to 205.1)	-19.2 (-22.1 to -15.8)	-22 (-26.3 to -17.2)	-20.4 (-24.1 to -16.3)
High income	High-income Asia Pacific	Brunei Darussalam	Incidence	All Ages	289 (276 to 303)	135 (126 to 143)	155 (148 to 162)	682 (644 to 723)	313 (293 to 335)	369 (347 to 393)	135.7 (126.4 to 146.5)	132.6 (120 to 146.3)	138.3 (126.5 to 154.1)
High income	High-income Asia Pacific	Brunei Darussalam	Incidence	Age-standardized	267.4 (255.5 to 278.2)	245.8 (228.6 to 262.7)	293 (284.5 to 300.6)	235.9 (225.8 to 246.1)	210.9 (198.8 to 223.5)	261.5 (251.1 to 273.4)	-11.8 (-14.5 to -8.7)	-14.2 (-18.6 to -9.6)	-10.8 (-14 to -7)
High income	High-income Asia Pacific	Brunei Darussalam	Prevalence	All Ages	3,531 (3,295 to 3,750)	1,768 (1,648 to 1,886)	1,763 (1,633 to 1,891)	8,122 (7,549 to 8,690)	3,905 (3,606 to 4,209)	4,217 (3,901 to 4,536)	130 (120.2 to 140.5)	120.8 (106.8 to 134.9)	139.2 (126.7 to 153.9)
High income	High-income Asia Pacific	Brunei Darussalam	Prevalence	Age-standardized	3467.8 (3286.4 to 3644)	3373.5 (3144.3 to 3593)	3666.7 (3499.8 to 3822.3)	2937.8 (2771 to 3106.4)	2693.6 (2521 to 2879.9)	3273.7 (3094.9 to 3466)	-15.3 (-18.4 to -11.8)	-20.2 (-24.8 to -15.6)	-10.7 (-14.6 to -6)
High income	High-income Asia Pacific	Brunei Darussalam	Deaths	All Ages	49 (34 to 56)	21 (11 to 26)	28 (20 to 33)	68 (58 to 78)	31 (25 to 36)	37 (30 to 44)	39.1 (14.6 to 108.3)	48.7 (14 to 148.2)	32 (2.4 to 97.3)
High income	High-income Asia Pacific	Brunei Darussalam	Deaths	Age-standardized	91.6 (59.1 to 106.5)	68.3 (34.9 to 84.6)	142.2 (97.8 to 166.6)	48.9 (40.9 to 56)	37.1 (28.9 to 43.8)	79.9 (66.1 to 93.4)	-46.6 (-55.4 to -22.3)	-45.6 (-57.8 to -11.4)	-43.8 (-54.6 to -20.8)
High income	High-income Asia Pacific	Brunei Darussalam	DALYs	All Ages	1,175 (916 to 1,327)	484 (331 to 573)	691 (541 to 798)	1,791 (1,583 to 2,043)	758 (655 to 893)	1,033 (890 to 1,193)	52.5 (30.7 to 107.8)	56.7 (27.4 to 130.9)	49.5 (23.6 to 103)
High income	High-income Asia Pacific	Brunei Darussalam	DALYs	Age-standardized	1547.7 (1135.6 to 1751.1)	1157.2 (733.7 to 1390.2)	2246.4 (1652.1 to 2582.3)	842.8 (746.3 to 941.8)	642.4 (551.7 to 737.5)	1242.9 (1070.5 to 1415)	-45.5 (-53.3 to -25.4)	-44.5 (-54.8 to -17.7)	-44.7 (-53.7 to -25)
High income	High-income Asia Pacific	Brunei Darussalam	YLLs	All Ages	910 (650 to 1,050)	368 (218 to 455)	543 (401 to 642)	1,180 (1,016 to 1,417)	504 (419 to 641)	676 (547 to 811)	29.6 (5.4 to 98.7)	37.1 (4.6 to 132.8)	24.5 (-4.6 to 90.4)
High income	High-income Asia Pacific	Brunei Darussalam	YLLs	Age-standardized	1298.1 (881.9 to 1500.9)	944.2 (527.8 to 1169.3)	1942.9 (1366.7 to 2282.8)	630.2 (538.4 to 722.8)	472.4 (387.9 to 559.2)	970.8 (804 to 1138.5)	-51.5 (-59.7 to -28.1)	-50 (-61.3 to -17.5)	-50 (-59.8 to -27.8)
High income	High-income Asia Pacific	Brunei Darussalam	YLDs	All Ages	265 (214 to 311)	116 (90 to 141)	149 (119 to 178)	611 (499 to 718)	254 (197 to 310)	358 (283 to 431)	131 (116.6 to 145.4)	118.8 (97.5 to 140.6)	140.5 (121.9 to 161)
High income	High-income Asia Pacific	Brunei Darussalam	YLDs	Age-standardized	249.6 (201.8 to 288.9)	213 (165.6 to 257.2)	303.6 (246.5 to 358.3)	212.6 (173.1 to 246.6)	170.1 (132.1 to 205.5)	272 (220.2 to 323.8)	-14.8 (-18.6 to -10.6)	-20.2 (-26 to -14.3)	-10.4 (-15.1 to -4.9)
High income	High-income Asia Pacific	Japan	Incidence	All Ages	231,765 (209,780 to 254,958)	89,987 (80,030 to 101,548)	141,778 (127,513 to 156,231)	419,343 (376,123 to 462,154)	162,728 (139,708 to 188,003)	256,614 (228,809 to 282,645)	80.9 (67.9 to 94.4)	80.8 (61 to 101.9)	81 (68.1 to 94.3)
High income	High-income Asia Pacific	Japan	Incidence	Age-standardized	144.9 (132.1 to 158.5)	102 (91.5 to 113.6)	202.3 (184.2 to 220.8)	111 (101.1 to 121.2)	71.5 (64.3 to 79.9)	158.5 (143 to 174.6)	-23.4 (-26.1 to -20.5)	-29.9 (-34 to -25.3)	-21.6 (-25 to -18.1)
High income	High-income Asia Pacific	Japan	Prevalence	All Ages	2,996,186 (2,715,338 to 3,305,788)	1,256,776 (1,129,311 to 1,393,259	1,739,410 (1,571,832 to 1,927,460)	4,789,562 (4,350,538 to 5,272,870)	1,892,153 (1,701,206 to 2,119,700	2,897,409 (2,597,070 to 3,234,313)	59.9 (50.3 to 70.3)	50.6 (39.2 to 64.4)	66.6 (55.9 to 78.7)
High income	High-income Asia Pacific	Japan	Prevalence	Age-standardized	1877 (1709.5 to 2062.5)	1426.4 (1286.7 to 1577.4)	2556.8 (2319.5 to 2818.5)	1354.2 (1226.5 to 1487.2)	1028 (915.4 to 1147.9)	1816.8 (1640.2 to 2021.8)	-27.9 (-30.5 to -24.4)	-27.9 (-32.4 to -23.1)	-28.9 (-32.4 to -24.8)
High income	High-income Asia Pacific	Japan	Deaths	All Ages	18,923 (17,175 to 20,347)	6,586 (5,371 to 7,332)	12,336 (11,459 to 13,182)	37,910 (27,825 to 50,611)	13,228 (8,759 to 19,868)	24,682 (18,850 to 31,191)	100.3 (58.6 to 157.5)	100.8 (49.1 to 187.9)	100.1 (62.9 to 143.3)
High income	High-income Asia Pacific	Japan	Deaths	Age-standardized	12.4 (11.1 to 13.4)	7.1 (5.8 to 8)	21.4 (19.7 to 23)	7.4 (5.6 to 9.8)	4 (2.8 to 5.9)	12.8 (9.8 to 16.1)	-40.5 (-51.3 to -23.2)	-44.3 (-55.7 to -20.1)	-40.5 (-50.7 to -27.9)
High income	High-income Asia Pacific	Japan	DALYs	All Ages	509,378 (462,787 to 553,626)	172,970 (150,289 to 194,326)	336,408 (305,272 to 369,029)	787,117 (656,931 to 940,623)	254,622 (202,959 to 318,474)	532,496 (446,044 to 632,075)	54.5 (37.8 to 79.6)	47.2 (27.8 to 82)	58.3 (41.6 to 79.5)
High income	High-income Asia Pacific	Japan	DALYs	Age-standardized	315 (286 to 341.8)	188.9 (164.1 to 212.6)	505.3 (460.8 to 552.5)	203.7 (173.7 to 239.5)	122.4 (102.3 to 148.4)	315.1 (265.9 to 371.5)	-35.3 (-40.6 to -26.1)	-35.2 (-40.4 to -22.8)	-37.6 (-43.2 to -30.2)
High income	High-income Asia Pacific	Japan	YLLs	All Ages	281,120 (259,298 to 301,168)	90,936 (74,873 to 101,032)	190,184 (179,171 to 203,131)	427,759 (325,097 to 565,510)	135,171 (96,680 to 199,590)	292,588 (228,986 to 371,543)	52.2 (22.8 to 96.3)	48.6 (15.1 to 116.7)	53.8 (26.9 to 88.6)
High income	High-income Asia Pacific	Japan	YLLs	Age-standardized	172.5 (158.5 to 184.9)	95 (78.2 to 105.5)	291.8 (272.4 to 311.2)	99.6 (78.7 to 131.5)	53.7 (41.1 to 79.1)	162.9 (129.4 to 206.5)	-42.2 (-51.2 to -25.5)	-43.5 (-52.7 to -18.2)	-44.2 (-53 to -32)
High income	High-income Asia Pacific	Japan	YLDs	All Ages	228,257 (184,091 to 268,638)	82,034 (63,990 to 100,553)	146,223 (115,829 to 173,014)	359,358 (286,985 to 426,040)	119,451 (92,761 to 147,482)	239,908 (187,589 to 289,725)	57.4 (46.5 to 69.6)	45.6 (31.9 to 60.9)	64.1 (51.4 to 77.7)
High income	High-income Asia Pacific	Japan	YLDs	Age-standardized	142.5 (115.2 to 167.8)	93.9 (73.7 to 114.3)	213.5 (169.9 to 253.6)	104.1 (83.5 to 122.5)	68.8 (53.6 to 84.4)	152.2 (121.2 to 183.8)	-26.9 (-29.8 to -23.2)	-26.8 (-31.1 to -21.5)	-28.7 (-32.3 to -24.3)
High income	High-income Asia Pacific	Republic of Korea	Incidence	All Ages	42,098 (39,788 to 44,403)	17,281 (16,156 to 18,589)	24,817 (23,346 to 26,246)	143,630 (137,817 to 149,402)	56,248 (52,730 to 59,733)	87,382 (83,491 to 90,978)	241.2 (226.1 to 257.6)	225.5 (203.7 to 246.5)	252.1 (234 to 272.9)
High income	High-income Asia Pacific	Republic of Korea	Incidence	Age-standardized	154.9 (146.6 to 163)	114.1 (106.9 to 122.3)	213.4 (202.5 to 223.4)	172 (165.5 to 178.2)	116.4 (110.2 to 122.9)	230.3 (222.2 to 237.9)	11 (6.2 to 16)	2 (-4.1 to 8.2)	7.9 (3.2 to 13.2)
High income	High-income Asia Pacific	Republic of Korea	Prevalence	All Ages	538,428 (498,278 to 577,133)	226,668 (207,931 to 247,370)	311,760 (288,159 to 333,791)	1,669,944 (1,582,268 to 1,761,412)	635,891 (595,276 to 680,069)	1,034,054 (975,366 to 1,095,207)	210.2 (195.5 to 227.7)	180.5 (163 to 200.4)	231.7 (212.5 to 253.2)
High income	High-income Asia Pacific	Republic of Korea	Prevalence	Age-standardized	1828.4 (1720.9 to 1946)	1367.1 (1268.7 to 1473.7)	2609.2 (2451.4 to 2761.1)	2027.3 (1921.5 to 2140.8)	1406.5 (1311.7 to 1508.1)	2853.8 (2703.7 to 3005.7)	10.9 (6.3 to 15.4)	2.9 (-2.6 to 8.3)	9.4 (3.8 to 15.3)
High income	High-income Asia Pacific	Republic of Korea	Deaths	All Ages	4,572 (3,543 to 5,215)	2,051 (1,034 to 2,647)	2,521 (2,133 to 2,923)	10,692 (8,785 to 12,326)	4,107 (2,973 to 5,073)	6,585 (5,521 to 7,716)	133.8 (90.7 to 216.3)	100.2 (42.3 to 310.3)	161.2 (118.2 to 210.1)
High income	High-income Asia Pacific	Republic of Korea	Deaths	Age-standardized	24.2 (17 to 28.3)	17.4 (8 to 22.8)	40 (32.8 to 46.5)	13.2 (10.8 to 15.1)	7.9 (5.7 to 9.7)	23.5 (19.1 to 27.2)	-45.4 (-56.1 to -21)	-54.6 (-68 to 1.1)	-41.3 (-50.6 to -29.6)
High income	High-income Asia Pacific	Republic of Korea	DALYs	All Ages	122,624 (106,554 to 135,352)	46,797 (33,510 to 55,631)	75,827 (67,057 to 84,563)	263,784 (231,186 to 295,758)	85,529 (71,556 to 101,406)	178,255 (155,424 to 201,351)	115.1 (93.4 to 154.6)	82.8 (49.4 to 177.3)	135.1 (112.6 to 161.8)
High income	High-income Asia Pacific	Republic of Korea	DALYs	Age-standardized	479.9 (402.6 to 535.8)	315.3 (209.2 to 383.5)	781.5 (687.5 to 874.2)	313.9 (275.2 to 351.3)	178.5 (149.7 to 211.4)	515.8 (448.7 to 580.8)	-34.6 (-42.3 to -19.1)	-43.4 (-54.7 to -8)	-34 (-40.6 to -25.6)
High income	High-income Asia Pacific	Republic of Korea	YLLs	All Ages	81,063 (66,683 to 90,705)	31,853 (18,964 to 39,707)	49,210 (42,767 to 56,512)	135,071 (115,284 to 158,643)	44,974 (34,662 to 58,645)	90,097 (76,660 to 106,842)	66.6 (39 to 119.5)	41.2 (2.4 to 178.6)	83.1 (54.2 to 119.2)
High income	High-income Asia Pacific	Republic of Korea	YLLs	Age-standardized	343.1 (266.7 to 388.9)	227.5 (122.9 to 288.9)	561.4 (473 to 649.1)	158.2 (134.6 to 184.6)	87.3 (67.5 to 115.4)	273.7 (230 to 321)	-53.9 (-61.7 to -35.7)	-61.6 (-72.2 to -15.9)	-51.2 (-58.7 to -42.3)
High income	High-income Asia Pacific	Republic of Korea	YLDs	All Ages	41,561 (33,505 to 48,790)	14,944 (11,419 to 18,370)	26,617 (20,867 to 31,837)	128,713 (104,822 to 149,576)	40,555 (31,359 to 49,745)	88,158 (70,775 to 104,483)	209.7 (186.4 to 234.2)	171.4 (141.4 to 202.3)	231.2 (199.8 to 264.9)
High income	High-income Asia Pacific	Republic of Korea	YLDs	Age-standardized	136.9 (111 to 159.4)	87.9 (67.7 to 106.5)	220.1 (174.2 to 259.3)	155.7 (127.3 to 181.2)	91.2 (71.1 to 111.5)	242.1 (195.8 to 285.4)	13.7 (7.4 to 20.4)	3.8 (-4.9 to 12.3)	10 (2.9 to 17)
High income	High-income Asia Pacific	Singapore	Incidence	All Ages	5,096 (4,960 to 5,234)	1,798 (1,705 to 1,904)	3,298 (3,233 to 3,362)	8,829 (8,318 to 9,352)	2,464 (2,302 to 2,644)	6,365 (5,952 to 6,771)	73.3 (64.6 to 81.8)	37.1 (28.3 to 47)	93 (79.9 to 105.5)
High income	High-income Asia Pacific	Singapore	Incidence	Age-standardized	225.5 (220.2 to 230.8)	152.8 (144.9 to 161.5)	307 (302.4 to 311.1)	115.4 (109 to 122)	63.6 (59.4 to 67.9)	166.3 (156.3 to 176.1)	-48.8 (-51.2 to -46.6)	-58.4 (-61.1 to -55.8)	-45.8 (-49 to -42.5)
High income	High-income Asia Pacific	Singapore	Prevalence	All Ages	61,138 (58,372 to 64,211)	22,403 (20,894 to 24,196)	38,735 (37,166 to 40,508)	113,408 (106,305 to 121,242)	33,901 (31,034 to 37,180)	79,507 (74,334 to 85,194)	85.5 (76.2 to 94.7)	51.3 (41.5 to 61.5)	105.3 (92 to 119.9)
High income	High-income Asia Pacific	Singapore	Prevalence	Age-standardized	2845 (2748.1 to 2948.6)	1936.7 (1820.5 to 2067.4)	3989.4 (3882.3 to 4102.3)	1499.9 (1407.5 to 1598.6)	871.6 (795.8 to 959.5)	2215.2 (2079.5 to 2361.1)	-47.3 (-49.8 to -44.7)	-55 (-58.3 to -51.5)	-44.5 (-47.9 to -41.1)
High income	High-income Asia Pacific	Singapore	Deaths	All Ages	804 (689 to 860)	212 (119 to 244)	592 (554 to 629)	635 (526 to 854)	224 (163 to 399)	411 (352 to 476)	-21.1 (-33.7 to 15.2)	5.3 (-21.4 to 185.2)	-30.6 (-40.6 to -18.8)
High income	High-income Asia Pacific	Singapore	Deaths	Age-standardized	47.3 (40.1 to 50.9)	21.9 (12.3 to 25.3)	87.9 (81.4 to 93.6)	9 (7.4 to 12.1)	5.5 (4 to 9.8)	13.8 (11.7 to 15.9)	-81 (-83.9 to -72.1)	-75 (-81.1 to -32.6)	-84.4 (-86.5 to -81.8)
High income	High-income Asia Pacific	Singapore	DALYs	All Ages	18,677 (16,605 to 20,008)	4,811 (3,245 to 5,440)	13,866 (12,947 to 14,806)	18,515 (16,192 to 21,538)	5,148 (4,207 to 7,584)	13,367 (11,670 to 15,044)	-0.9 (-11.8 to 23.4)	7 (-11.9 to 109.9)	-3.6 (-13.9 to 6.9)
High income	High-income Asia Pacific	Singapore	DALYs	Age-standardized	943.1 (833.4 to 1009.4)	441.5 (297.3 to 499.6)	1629.6 (1528.2 to 1733.9)	247.4 (216.7 to 288.3)	130.6 (107.1 to 191.4)	383.6 (336 to 429.6)	-73.8 (-76.6 to -67)	-70.4 (-75.8 to -42.2)	-76.5 (-79 to -74)
High income	High-income Asia Pacific	Singapore	YLLs	All Ages	13,909 (12,056 to 14,859)	3,362 (1,888 to 3,847)	10,547 (9,860 to 11,256)	9,441 (7,933 to 12,378)	2,934 (2,213 to 5,295)	6,507 (5,549 to 7,595)	-32.1 (-42.9 to -2.3)	-12.7 (-35.4 to 148.3)	-38.3 (-47.4 to -27.7)
High income	High-income Asia Pacific	Singapore	YLLs	Age-standardized	724.5 (625.2 to 774.9)	317.9 (178.8 to 363.7)	1290.2 (1206.3 to 1370.9)	127.8 (107.7 to 169.1)	73.3 (55.3 to 132.9)	193.3 (165.2 to 224.2)	-82.4 (-85.1 to -74.5)	-76.9 (-82.8 to -34.7)	-85 (-87.1 to -82.4)
High income	High-income Asia Pacific	Singapore	YLDs	All Ages	4,768 (3,907 to 5,554)	1,449 (1,139 to 1,777)	3,319 (2,662 to 3,933)	9,075 (7,374 to 10,708)	2,215 (1,722 to 2,725)	6,860 (5,432 to 8,195)	90.3 (77.5 to 102.3)	52.9 (37.5 to 69.3)	106.7 (89.7 to 124.3)
High income	High-income Asia Pacific	Singapore	YLDs	Age-standardized	218.6 (178.3 to 252.9)	123.6 (97 to 151.5)	339.4 (275.4 to 399.4)	119.6 (97.4 to 141.2)	57.3 (44.7 to 70.6)	190.3 (151.8 to 226.1)	-45.3 (-48.7 to -42.1)	-53.6 (-58.4 to -48.5)	-43.9 (-47.9 to -39.8)
		Australasia	Incidence	All Ages	56,745 (54,880 to 58,499)	24,833 (23,381 to 26,239)	31,912 (31,296 to 32,479)	107,884 (102,557 to 113,413)	55,315 (52,129 to 58,380)	52,569 (49,757 to 55,524)	90.1 (83.2 to 96.9)	122.7 (112.8 to 133.3)	64.7 (56.8 to 73.1)
		Australasia	Incidence	Age-standardized	247.7 (240.1 to 254.6)	205.2 (194.3 to 215.3)	301.9 (296.2 to 307.1)	230.7 (219.9 to 242.1)	223.4 (211.4 to 236.1)	239.1 (227.7 to 251.9)	-6.9 (-10 to -3.8)	8.8 (4.3 to 13.4)	-20.8 (-24.1 to -17.1)
		Australasia	Prevalence	All Ages	818,140 (783,973 to 850,593)	400,635 (377,024 to 422,390)	417,506 (402,192 to 431,802)	1,540,426 (1,462,887 to 1,624,938)	821,730 (776,510 to 872,535)	718,696 (678,880 to 765,259)	88.3 (81.2 to 95.7)	105.1 (95.2 to 114.8)	72.1 (64 to 82)
		Australasia	Prevalence	Age-standardized	3552.1 (3403.4 to 3696.8)	3169.8 (2982.7 to 3346.5)	4122.3 (3979.8 to 4256.8)	3192.8 (3027.7 to 3372.1)	3169.6 (2986.8 to 3359.8)	3227.5 (3051.5 to 3438.1)	-10.1 (-13.2 to -6.6)	0 (-4.8 to 4.5)	-21.7 (-25.2 to -17.6)
		Australasia	Deaths	All Ages	7,096 (6,617 to 7,499)	2,380 (2,104 to 2,747)	4,715 (4,431 to 4,965)	11,398 (9,581 to 12,979)	5,161 (3,720 to 6,131)	6,237 (5,328 to 7,271)	60.6 (40.8 to 82.4)	116.8 (64 to 156.3)	32.3 (13.9 to 55.7)
		Australasia	Deaths	Age-standardized	30.3 (28.2 to 32.1)	17.4 (15.4 to 20)	52.1 (48.5 to 55.2)	20.6 (17.4 to 23.3)	16.7 (12.2 to 19.6)	25.9 (22.1 to 30.2)	-32.2 (-39.8 to -23.6)	-4.1 (-25.6 to 12.4)	-50.3 (-56.9 to -41.5)
		Australasia	DALYs	All Ages	173,207 (161,240 to 184,157)	68,581 (61,474 to 75,221)	104,626 (97,288 to 111,085)	257,802 (228,535 to 285,031)	128,436 (108,874 to 144,198)	129,366 (114,126 to 145,247)	48.8 (38 to 60.9)	87.3 (66.4 to 105.3)	23.6 (11.5 to 38.4)
		Australasia	DALYs	Age-standardized	731.7 (679.4 to 778.3)	527.9 (470.8 to 580.3)	1034.6 (962.8 to 1099.3)	512.1 (455.1 to 564.8)	479.2 (411.3 to 537.4)	555.7 (490.6 to 623.3)	-30 (-34.9 to -24.6)	-9.2 (-17.7 to -1.2)	-46.3 (-51.4 to -40.1)
		Australasia	YLLs	All Ages	120,981 (113,709 to 126,850)	40,536 (35,577 to 45,481)	80,446 (76,044 to 84,407)	160,314 (139,444 to 179,031)	72,088 (54,195 to 83,457)	88,226 (76,729 to 101,322)	32.5 (18.6 to 48.3)	77.8 (44.8 to 106.3)	9.7 (-4.6 to 28.5)
		Australasia	YLLs	Age-standardized	504.9 (473.1 to 529.4)	305.3 (264.9 to 339.8)	796.8 (750.9 to 837.4)	308.6 (270.7 to 343.1)	259.2 (198.9 to 298)	370.4 (322.5 to 425.3)	-38.9 (-45.2 to -31.6)	-15.1 (-28.6 to -2.3)	-53.5 (-59.4 to -45.8)
		Australasia	YLDs	All Ages	52,226 (41,536 to 60,932)	28,045 (22,066 to 33,035)	24,181 (18,744 to 29,433)	97,488 (77,558 to 114,681)	56,348 (44,080 to 66,962)	41,140 (31,843 to 50,088)	86.7 (78.3 to 95.6)	100.9 (89.7 to 112.7)	70.1 (60.1 to 82.1)
		Australasia	YLDs	Age-standardized	226.9 (180.7 to 264.5)	222.6 (174.5 to 262.6)	237.8 (185.7 to 289.1)	203.5 (162.4 to 239.3)	220 (172.6 to 262.6)	185.3 (144.1 to 225.5)	-10.3 (-14.2 to -6.3)	-1.2 (-6.8 to 4.9)	-22.1 (-26.2 to -17.3)
High income	Australasia	Australia	Incidence	All Ages	47,059 (45,740 to 48,375)	20,102 (19,043 to 21,268)	26,957 (26,428 to 27,416)	90,112 (85,681 to 94,842)	45,930 (43,276 to 48,592)	44,182 (41,937 to 46,724)	91.5 (83.4 to 99.2)	128.5 (116.5 to 141)	63.9 (55.5 to 72.8)
High income	Australasia	Australia	Incidence	Age-standardized	246.6 (239.8 to 253)	200.2 (190.3 to 210.5)	305.5 (299.9 to 310.4)	228.4 (218 to 239.6)	219.8 (208.1 to 232.2)	238.3 (227.2 to 251)	-7.4 (-11 to -3.9)	9.8 (4.4 to 15.4)	-22 (-25.5 to -18.1)
High income	Australasia	Australia	Prevalence	All Ages	676,694 (651,315 to 703,043)	325,712 (307,726 to 344,326)	350,981 (337,868 to 363,144)	1,284,616 (1,218,823 to 1,357,765)	680,744 (642,214 to 721,372)	603,873 (570,690 to 645,147)	89.8 (81.9 to 98.4)	109 (97.7 to 120.7)	72.1 (63 to 82.2)
High income	Australasia	Australia	Prevalence	Age-standardized	3527.1 (3387.4 to 3667.6)	3101.6 (2929.1 to 3288.6)	4155.2 (4005.4 to 4292.3)	3157.3 (2989.9 to 3336.5)	3113.2 (2930.7 to 3298.8)	3215.3 (3029.1 to 3430.6)	-10.5 (-14 to -6.6)	0.4 (-5.1 to 5.9)	-22.6 (-26.6 to -18.3)
High income	Australasia	Australia	Deaths	All Ages	5,801 (5,389 to 6,150)	1,890 (1,645 to 2,174)	3,911 (3,656 to 4,155)	9,293 (7,736 to 10,688)	4,171 (2,997 to 5,071)	5,122 (4,285 to 6,073)	60.2 (38.3 to 84.7)	120.7 (66.8 to 167.6)	31 (10.6 to 58)
High income	Australasia	Australia	Deaths	Age-standardized	29.8 (27.6 to 31.7)	16.6 (14.5 to 19.1)	52.1 (48.2 to 55.6)	19.8 (16.6 to 22.7)	15.9 (11.6 to 19.1)	25.2 (21.1 to 29.9)	-33.5 (-42.1 to -23.7)	-4.3 (-25.7 to 15.2)	-51.7 (-59.2 to -41.8)
High income	Australasia	Australia	DALYs	All Ages	141,977 (131,208 to 151,583)	54,860 (48,923 to 60,487)	87,117 (80,336 to 92,870)	211,129 (186,252 to 235,273)	104,225 (87,183 to 118,059)	106,904 (93,448 to 121,857)	48.7 (36 to 62.4)	90 (69.4 to 111.1)	22.7 (9.1 to 38.9)
High income	Australasia	Australia	DALYs	Age-standardized	720.1 (665.4 to 769.3)	506.8 (450.2 to 560.6)	1034.8 (953.4 to 1103.3)	497.1 (440.4 to 553)	460.4 (390.6 to 521.5)	544.8 (478 to 618.2)	-31 (-36.6 to -24.8)	-9.2 (-17.8 to 0.5)	-47.3 (-53 to -40.6)
High income	Australasia	Australia	YLLs	All Ages	98,776 (92,650 to 104,086)	31,961 (27,914 to 35,710)	66,815 (62,648 to 70,582)	129,825 (112,685 to 146,701)	57,440 (43,364 to 68,108)	72,385 (61,681 to 84,422)	31.4 (15.1 to 49.4)	79.7 (46.5 to 114.5)	8.3 (-8.1 to 29)
High income	Australasia	Australia	YLLs	Age-standardized	494.9 (462.4 to 522.3)	288.2 (248.8 to 320.2)	795.3 (742.7 to 842.9)	296.1 (257.6 to 334.5)	244 (188.7 to 287)	360.6 (308.5 to 420.2)	-40.2 (-47.5 to -32.1)	-15.4 (-29.1 to 0.7)	-54.7 (-61.3 to -46.1)
High income	Australasia	Australia	YLDs	All Ages	43,201 (34,078 to 50,744)	22,899 (17,750 to 27,258)	20,302 (15,654 to 24,774)	81,305 (64,648 to 95,288)	46,785 (36,117 to 55,901)	34,519 (26,365 to 42,468)	88.2 (78.6 to 98.5)	104.3 (91 to 118.5)	70 (58.8 to 83.1)
High income	Australasia	Australia	YLDs	Age-standardized	225.3 (178.3 to 263.7)	218.6 (170.2 to 259.9)	239.5 (185 to 292.4)	201.1 (160.5 to 236.1)	216.4 (167.4 to 260.9)	184.3 (142.3 to 226.7)	-10.7 (-15.2 to -6.3)	-1 (-7.6 to 5.7)	-23.1 (-27.8 to -17.8)
High income	Australasia	New Zealand	Incidence	All Ages	9,686 (8,879 to 10,377)	4,731 (4,187 to 5,262)	4,955 (4,634 to 5,214)	17,772 (16,193 to 19,293)	9,385 (8,526 to 10,249)	8,387 (7,615 to 9,101)	83.5 (74.8 to 92.5)	98.4 (86.5 to 111.8)	69.3 (57 to 82.1)
High income	Australasia	New Zealand	Incidence	Age-standardized	252.8 (233.7 to 270.3)	230 (205.6 to 253.8)	284.1 (267.3 to 297.3)	242.3 (222.9 to 261.6)	242.1 (222 to 263.5)	242.9 (222.8 to 262.9)	-4.1 (-8.3 to 0.3)	5.3 (0 to 11.5)	-14.5 (-19.6 to -8.5)
High income	Australasia	New Zealand	Prevalence	All Ages	141,447 (129,989 to 151,923)	74,922 (67,555 to 82,274)	66,524 (62,110 to 70,801)	255,810 (234,240 to 278,107)	140,986 (129,277 to 154,048)	114,823 (104,462 to 125,471)	80.9 (72.7 to 90.7)	88.2 (78.8 to 99.8)	72.6 (60.5 to 87.6)
High income	Australasia	New Zealand	Prevalence	Age-standardized	3672.8 (3382.9 to 3945.2)	3504.3 (3175.9 to 3827.4)	3956.4 (3709.5 to 4198.2)	3384.3 (3116.8 to 3667.2)	3469.5 (3190.1 to 3781.9)	3295 (3009 to 3587)	-7.9 (-11.8 to -3.2)	-1 (-5.7 to 4.9)	-16.7 (-22.1 to -10.3)
High income	Australasia	New Zealand	Deaths	All Ages	1,295 (1,207 to 1,394)	490 (435 to 576)	805 (753 to 855)	2,105 (1,789 to 2,350)	990 (722 to 1,153)	1,115 (956 to 1,275)	62.5 (42.4 to 81.5)	102 (52.5 to 138.2)	38.5 (20 to 59.2)
High income	Australasia	New Zealand	Deaths	Age-standardized	32.7 (30.4 to 35.3)	21.4 (18.9 to 25)	52 (48.4 to 55.4)	24.5 (21 to 27.3)	20.9 (15.4 to 24.3)	29.6 (25.4 to 33.8)	-25.2 (-33.8 to -16.8)	-2.2 (-24.8 to 14.6)	-43.2 (-50.5 to -35)
High income	Australasia	New Zealand	DALYs	All Ages	31,230 (28,957 to 33,541)	13,721 (12,242 to 15,223)	17,509 (16,234 to 18,752)	46,673 (41,621 to 51,152)	24,211 (20,198 to 27,279)	22,462 (19,793 to 25,064)	49.4 (38.4 to 60.4)	76.4 (54.2 to 93.7)	28.3 (15.8 to 42.3)
High income	Australasia	New Zealand	DALYs	Age-standardized	789.5 (731.1 to 848.8)	632.7 (563.2 to 701.9)	1034.9 (959.8 to 1106.7)	591.7 (528.5 to 646.8)	578 (488.7 to 651.3)	614.5 (542.4 to 684.5)	-25.1 (-30.4 to -19.6)	-8.6 (-18.7 to 0.2)	-40.6 (-46.2 to -34.5)
High income	Australasia	New Zealand	YLLs	All Ages	22,205 (20,798 to 23,681)	8,575 (7,572 to 9,816)	13,630 (12,791 to 14,474)	30,490 (26,588 to 33,841)	14,649 (10,999 to 16,885)	15,841 (13,832 to 18,064)	37.3 (23 to 52.8)	70.8 (37.5 to 98.1)	16.2 (1.6 to 33)
High income	Australasia	New Zealand	YLLs	Age-standardized	554.7 (518.1 to 591.4)	390.4 (338.5 to 438)	805.2 (754.9 to 856.4)	375.2 (329.1 to 415.5)	339 (256.4 to 389.2)	423.3 (370.8 to 481.4)	-32.4 (-39 to -24.9)	-13.2 (-28.2 to 0.6)	-47.4 (-53.9 to -39.8)
High income	Australasia	New Zealand	YLDs	All Ages	9,025 (7,121 to 10,671)	5,146 (3,979 to 6,250)	3,879 (2,981 to 4,757)	16,183 (12,836 to 19,418)	9,562 (7,410 to 11,575)	6,621 (5,069 to 8,170)	79.3 (69.7 to 90.4)	85.8 (74.4 to 99.2)	70.7 (57.7 to 87.5)
High income	Australasia	New Zealand	YLDs	Age-standardized	234.8 (185.9 to 277.4)	242.3 (187.7 to 292.6)	229.7 (177.5 to 281.4)	216.5 (172 to 259.6)	239 (186.8 to 289)	191.2 (147.8 to 236.6)	-7.8 (-12.7 to -2.6)	-1.4 (-7.2 to 5.6)	-16.7 (-23 to -9.8)
		Western Europe	Incidence	All Ages	1,202,266 (1,152,107 to 1,253,191)	564,808 (532,455 to 598,852)	637,458 (615,188 to 658,531)	1,861,274 (1,783,981 to 1,934,614)	951,298 (908,612 to 990,231)	909,976 (871,798 to 946,284)	54.8 (52.6 to 57)	68.4 (63.2 to 73.1)	42.8 (39.9 to 45.4)
		Western Europe	Incidence	Age-standardized	218.7 (210.3 to 227.1)	172.3 (163.6 to 181.4)	274.7 (265.9 to 282.9)	218.5 (209.4 to 227.6)	195.8 (187.1 to 204.8)	241.9 (232.3 to 251.3)	-0.1 (-1.4 to 1.1)	13.6 (11.1 to 16)	-12 (-13.4 to -10.6)
		Western Europe	Prevalence	All Ages	16,577,913 (15,847,798 to 17,358,907	7,820,502 (7,397,905 to 8,272,056	8,757,411 (8,405,138 to 9,075,604)	26,320,873 (25,114,808 to 27,583,392	13,340,654 (12,711,875 to 14,028,753	12,980,219 (12,393,012 to 13,591,015	58.8 (56.8 to 60.8)	70.6 (67.1 to 74.1)	48.2 (45.5 to 50.9)
		Western Europe	Prevalence	Age-standardized	3002.7 (2872.9 to 3137.4)	2432.4 (2310.2 to 2567.6)	3821.4 (3678.1 to 3957.4)	3017.5 (2874.7 to 3165.8)	2724.7 (2593.9 to 2860.6)	3363.8 (3208.8 to 3527.6)	0.5 (-0.7 to 1.7)	12 (10 to 14)	-12 (-13.5 to -10.5)
		Western Europe	Deaths	All Ages	151,695 (141,500 to 163,424)	54,711 (47,986 to 65,895)	96,984 (92,420 to 100,043)	212,460 (185,963 to 233,330)	93,450 (70,460 to 105,789)	119,010 (107,158 to 130,231)	40.1 (27.6 to 51.4)	70.8 (32.7 to 91.3)	22.7 (13.8 to 33.3)
		Western Europe	Deaths	Age-standardized	25.2 (23.4 to 27.2)	14.3 (12.6 to 17.1)	45.1 (42.4 to 46.7)	19.2 (17 to 21.1)	13.9 (10.6 to 15.6)	27.2 (24.5 to 29.8)	-23.6 (-29.5 to -17.8)	-2.8 (-23.2 to 7.8)	-39.5 (-43.6 to -34.6)
		Western Europe	DALYs	All Ages	3,314,883 (3,092,068 to 3,539,729)	1,232,169 (1,108,580 to 1,394,375	2,082,714 (1,954,814 to 2,194,314)	4,306,940 (3,887,584 to 4,666,233)	1,924,926 (1,615,614 to 2,125,972	2,382,014 (2,180,239 to 2,594,181)	29.9 (23.3 to 37)	56.2 (33.9 to 67.3)	14.4 (8.4 to 21.1)
		Western Europe	DALYs	Age-standardized	569.7 (530.6 to 608.8)	359 (322.2 to 400.5)	897.9 (843.3 to 947.5)	460.8 (418.3 to 499.6)	367.3 (313.2 to 406.6)	582.2 (532.9 to 634)	-19.1 (-22.8 to -15.2)	2.3 (-10.5 to 8.6)	-35.2 (-38.5 to -31.6)
		Western Europe	YLLs	All Ages	2,360,775 (2,236,920 to 2,525,432)	779,525 (703,114 to 931,431)	1,581,250 (1,522,835 to 1,624,916)	2,762,201 (2,475,948 to 2,995,793)	1,139,610 (868,472 to 1,270,826	1,622,592 (1,481,516 to 1,760,207)	17 (8.5 to 26.1)	46.2 (14.8 to 62)	2.6 (-4.5 to 11)
		Western Europe	YLLs	Age-standardized	396.3 (375.7 to 422.9)	216.6 (196.1 to 256.7)	679.5 (652 to 698.9)	282.1 (255.3 to 304.7)	204.2 (157.1 to 225)	384.9 (353.7 to 417.3)	-28.8 (-33.4 to -23.5)	-5.7 (-23.9 to 3.7)	-43.3 (-47 to -38.9)
		Western Europe	YLDs	All Ages	954,108 (757,608 to 1,119,423)	452,644 (356,167 to 540,275)	501,464 (395,655 to 599,612)	1,544,739 (1,242,087 to 1,802,865)	785,316 (621,148 to 935,107)	759,422 (601,072 to 907,967)	61.9 (56.8 to 68.9)	73.5 (66.4 to 81.8)	51.4 (45.8 to 59)
		Western Europe	YLDs	Age-standardized	173.4 (138 to 202.9)	142.4 (112.7 to 169.5)	218.4 (172.8 to 261.1)	178.6 (143.1 to 209.5)	163.1 (128.7 to 193.9)	197.3 (156.6 to 235)	3 (0 to 6.9)	14.5 (10.3 to 19.1)	-9.7 (-12.7 to -5.7)
High income	Western Europe	Andorra	Incidence	All Ages	116 (109 to 123)	36 (33 to 40)	79 (75 to 84)	312 (294 to 329)	135 (126 to 145)	177 (166 to 187)	169.2 (159 to 179.2)	270.5 (247.6 to 294)	122.6 (112.8 to 132.5)
High income	Western Europe	Andorra	Incidence	Age-standardized	214.2 (203.3 to 225.1)	146.3 (135 to 158.7)	277.5 (264.9 to 290.9)	229.6 (216.3 to 242.7)	195 (180.8 to 211)	261.2 (246.5 to 275.5)	7.2 (3.4 to 10.9)	33.3 (25.6 to 40.9)	-5.9 (-9.7 to -2.3)
High income	Western Europe	Andorra	Prevalence	All Ages	1,632 (1,525 to 1,738)	576 (527 to 626)	1,055 (991 to 1,122)	4,441 (4,180 to 4,721)	1,992 (1,847 to 2,149)	2,450 (2,298 to 2,613)	172.2 (160.6 to 183.5)	245.7 (225.5 to 266.2)	132.1 (120.6 to 143.9)
High income	Western Europe	Andorra	Prevalence	Age-standardized	3043.1 (2856.7 to 3225.5)	2228.5 (2052.8 to 2413.7)	3871.3 (3659.3 to 4098.7)	3217 (3020.7 to 3423.5)	2817.3 (2605.9 to 3046.1)	3624.9 (3407.7 to 3858.9)	5.7 (1.7 to 9.6)	26.4 (19.9 to 32.9)	-6.4 (-10.6 to -1.8)
High income	Western Europe	Andorra	Deaths	All Ages	13 (10 to 17)	3 (2 to 4)	10 (8 to 14)	34 (26 to 42)	11 (8 to 14)	23 (17 to 29)	154.6 (81 to 242.2)	273.4 (139.4 to 488.7)	121.7 (53.5 to 201.5)
High income	Western Europe	Andorra	Deaths	Age-standardized	31 (23.9 to 39.7)	14 (9.1 to 19.8)	51.3 (39.8 to 65.1)	21.6 (16.4 to 27.3)	12.1 (8.6 to 16.1)	33.3 (25.3 to 41.8)	-30.2 (-49.4 to -7.2)	-13.5 (-44.9 to 35.7)	-35.1 (-52.7 to -13.3)
High income	Western Europe	Andorra	DALYs	All Ages	331 (271 to 411)	85 (66 to 110)	246 (199 to 309)	716 (595 to 851)	254 (207 to 306)	462 (375 to 557)	116.3 (68.4 to 167.8)	198.5 (123.6 to 289.4)	87.9 (41.2 to 138.1)
High income	Western Europe	Andorra	DALYs	Age-standardized	644.7 (531.8 to 792.8)	341.3 (265 to 439.8)	965.6 (788.1 to 1185.7)	505.4 (417.5 to 600.2)	348.4 (284.1 to 416.7)	675.8 (548 to 816.6)	-21.6 (-39 to -3.1)	2.1 (-23.1 to 32.7)	-30 (-47 to -12.6)
High income	Western Europe	Andorra	YLLs	All Ages	233 (177 to 311)	50 (33 to 73)	183 (138 to 245)	451 (333 to 579)	134 (95 to 179)	318 (236 to 408)	93.9 (34.8 to 167)	170 (68.1 to 338.1)	73.4 (17.8 to 140.1)
High income	Western Europe	Andorra	YLLs	Age-standardized	462.1 (352.3 to 603.9)	204.2 (134 to 296.9)	736.7 (566.4 to 958.8)	313 (229 to 405.2)	176.5 (124.3 to 240.2)	462.3 (344.9 to 588.7)	-32.3 (-52.5 to -7.9)	-13.6 (-46 to 39.8)	-37.2 (-56.3 to -14.3)
High income	Western Europe	Andorra	YLDs	All Ages	98 (77 to 118)	36 (27 to 44)	63 (48 to 77)	265 (208 to 313)	120 (93 to 147)	144 (111 to 177)	169.4 (154.1 to 184.8)	238.1 (211.6 to 266.9)	130.4 (114.7 to 146.2)
High income	Western Europe	Andorra	YLDs	Age-standardized	182.6 (143.5 to 217.4)	137.1 (106.1 to 167)	228.9 (177.6 to 280.6)	192.4 (150.6 to 228.4)	171.8 (132.8 to 211.5)	213.5 (165.3 to 259.6)	5.4 (0.2 to 10.3)	25.3 (16.1 to 34.3)	-6.7 (-12.4 to -0.8)
High income	Western Europe	Austria	Incidence	All Ages	22,431 (21,273 to 23,649)	11,103 (10,296 to 11,930)	11,327 (10,777 to 11,901)	34,958 (33,346 to 36,736)	17,599 (16,694 to 18,596)	17,360 (16,435 to 18,339)	55.9 (49.8 to 62.4)	58.5 (49.3 to 69.3)	53.3 (45.5 to 61.3)
High income	Western Europe	Austria	Incidence	Age-standardized	199.3 (190.6 to 208.1)	154.6 (146.2 to 163)	257.5 (245.7 to 269.2)	214 (204.7 to 224.8)	188.8 (180 to 198.7)	240.6 (228.4 to 253.7)	7.4 (3.6 to 11)	22.1 (16.3 to 27.8)	-6.5 (-11.2 to -2.2)
High income	Western Europe	Austria	Prevalence	All Ages	308,266 (294,613 to 323,825)	147,902 (139,909 to 156,999)	160,364 (152,109 to 168,382)	524,635 (499,937 to 553,157)	264,687 (251,053 to 280,803)	259,948 (245,557 to 276,598)	70.2 (64.2 to 76.5)	79 (70.8 to 88.3)	62.1 (53.3 to 71.2)
High income	Western Europe	Austria	Prevalence	Age-standardized	2773.3 (2656.5 to 2901.4)	2207.7 (2091.5 to 2332.8)	3681.3 (3502.2 to 3863.9)	3116.4 (2966.1 to 3288.3)	2816.3 (2669.3 to 2985.4)	3485.3 (3294.2 to 3700.9)	12.4 (8.7 to 16.3)	27.6 (22.1 to 33.5)	-5.3 (-10.2 to -0.2)
High income	Western Europe	Austria	Deaths	All Ages	1,991 (1,831 to 2,301)	806 (695 to 1,099)	1,185 (1,104 to 1,271)	3,113 (2,667 to 3,488)	1,346 (1,025 to 1,601)	1,767 (1,543 to 1,999)	56.4 (32.5 to 76.5)	67.1 (16.7 to 101.8)	49 (30.9 to 71.2)
High income	Western Europe	Austria	Deaths	Age-standardized	15.9 (14.6 to 18.3)	9.5 (8.3 to 13.1)	29 (26.9 to 31.1)	15.4 (13.2 to 17.1)	10.9 (8 to 12.7)	21.7 (19 to 24.6)	-3.4 (-19.1 to 8.9)	14.1 (-24.6 to 38)	-25.1 (-34.3 to -14.3)
High income	Western Europe	Austria	DALYs	All Ages	45,539 (41,533 to 50,498)	17,970 (15,490 to 22,223)	27,569 (25,310 to 30,141)	70,373 (61,776 to 77,955)	29,996 (24,184 to 34,828)	40,377 (35,719 to 45,103)	54.5 (35.7 to 67.8)	66.9 (25 to 87.6)	46.5 (33.1 to 61.6)
High income	Western Europe	Austria	DALYs	Age-standardized	385.9 (349.9 to 427.3)	243.8 (211.3 to 297.2)	629.8 (579 to 687.9)	399.1 (351.6 to 444.7)	303 (242.9 to 351.2)	520.1 (459.1 to 583)	3.4 (-9.1 to 12.2)	24.3 (-6.8 to 39.2)	-17.4 (-25.1 to -9)
High income	Western Europe	Austria	YLLs	All Ages	30,754 (28,667 to 35,148)	11,212 (9,875 to 15,372)	19,542 (18,363 to 20,710)	45,532 (39,393 to 50,333)	18,102 (12,974 to 21,081)	27,431 (24,025 to 31,094)	48.1 (23.4 to 67.6)	61.4 (2.5 to 96)	40.4 (23 to 61.7)
High income	Western Europe	Austria	YLLs	Age-standardized	252.4 (235.8 to 287.3)	142 (125.9 to 193.3)	445.7 (417.9 to 473.4)	250.3 (217.1 to 277.9)	174.5 (120.7 to 203.9)	346.2 (303.5 to 391.9)	-0.8 (-17.9 to 12.5)	22.9 (-25.2 to 48.8)	-22.3 (-31.9 to -10.7)
High income	Western Europe	Austria	YLDs	All Ages	14,786 (11,350 to 18,364)	6,758 (4,802 to 8,807)	8,028 (5,974 to 9,995)	24,840 (18,967 to 30,584)	11,894 (8,274 to 15,514)	12,946 (9,755 to 16,202)	68 (59.5 to 76.8)	76 (63.6 to 89.2)	61.3 (50.9 to 73.2)
High income	Western Europe	Austria	YLDs	Age-standardized	133.4 (103.7 to 165.5)	101.8 (74.8 to 132.8)	184.1 (137.6 to 228.8)	148.9 (114.9 to 183)	128.6 (93.1 to 166.2)	173.9 (130.8 to 216.6)	11.6 (5.9 to 17.5)	26.2 (16.5 to 36)	-5.6 (-11.2 to 0.9)
High income	Western Europe	Belgium	Incidence	All Ages	35,898 (34,926 to 36,876)	16,415 (15,546 to 17,350)	19,483 (19,232 to 19,690)	55,848 (54,368 to 57,319)	27,484 (26,350 to 28,604)	28,365 (27,656 to 28,943)	55.6 (51.1 to 59.9)	67.4 (58.6 to 76.1)	45.6 (42 to 48.7)
High income	Western Europe	Belgium	Incidence	Age-standardized	248.1 (242.3 to 253.9)	191.1 (182.3 to 200.7)	313.4 (309.7 to 316.8)	271.3 (263.8 to 278.8)	239.9 (229.3 to 250.9)	303.8 (296.2 to 310)	9.3 (6.6 to 12.1)	25.5 (19.6 to 31.5)	-3.1 (-5.5 to -1)
High income	Western Europe	Belgium	Prevalence	All Ages	493,983 (478,812 to 509,352)	229,144 (216,210 to 242,773)	264,839 (259,280 to 269,336)	853,012 (826,328 to 879,944)	432,759 (413,148 to 453,890)	420,252 (407,278 to 430,690)	72.7 (67.6 to 78.4)	88.9 (79.5 to 99.5)	58.7 (53.4 to 63.1)
High income	Western Europe	Belgium	Prevalence	Age-standardized	3373.8 (3263.9 to 3480.3)	2698.7 (2543.9 to 2855.5)	4320.7 (4224.4 to 4399.4)	3927.7 (3803.7 to 4052.9)	3588.3 (3427.3 to 3761.6)	4345.5 (4206.7 to 4456.4)	16.4 (13 to 20.1)	33 (26.3 to 40.5)	0.6 (-2.7 to 3.5)
High income	Western Europe	Belgium	Deaths	All Ages	5,324 (4,903 to 5,771)	1,681 (1,439 to 2,060)	3,643 (3,387 to 3,880)	6,693 (5,704 to 7,509)	2,759 (2,107 to 3,299)	3,934 (3,407 to 4,505)	25.7 (9.9 to 41.8)	64.1 (32.8 to 103.9)	8 (-7.4 to 26.7)
High income	Western Europe	Belgium	Deaths	Age-standardized	33.5 (30.8 to 36.2)	16.5 (14.2 to 19.8)	63.9 (59.2 to 68.5)	24.8 (21.4 to 27.5)	16.8 (12.9 to 19.8)	36.5 (31.7 to 41.8)	-26 (-34.4 to -16.9)	1.8 (-17.6 to 23.3)	-42.9 (-50.8 to -33.3)
High income	Western Europe	Belgium	DALYs	All Ages	114,475 (105,812 to 122,577)	37,516 (32,831 to 42,840)	76,959 (71,553 to 81,875)	142,937 (127,035 to 155,837)	61,779 (51,448 to 70,138)	81,158 (72,583 to 90,614)	24.9 (14.9 to 35)	64.7 (42.4 to 84.1)	5.5 (-4.8 to 18)
High income	Western Europe	Belgium	DALYs	Age-standardized	741 (684.6 to 794.8)	412.4 (360.5 to 465.5)	1239.8 (1147.9 to 1320.9)	621.9 (554.3 to 680.4)	486.9 (405.8 to 551.9)	797.8 (714.2 to 890.3)	-16.1 (-22.5 to -9.7)	18.1 (2 to 30.8)	-35.7 (-41.9 to -28.3)
High income	Western Europe	Belgium	YLLs	All Ages	84,792 (79,600 to 90,607)	23,480 (20,540 to 28,327)	61,312 (57,572 to 64,974)	92,322 (80,578 to 102,139)	35,727 (26,855 to 41,754)	56,595 (49,662 to 64,677)	8.9 (-3.4 to 21.7)	52.2 (20.2 to 82.6)	-7.7 (-19.8 to 7.6)
High income	Western Europe	Belgium	YLLs	Age-standardized	538.2 (505 to 573.9)	246.7 (218.5 to 295.9)	985 (924.5 to 1044.7)	387.7 (339.6 to 429.4)	268.8 (200 to 313)	543.4 (478.7 to 622.6)	-28 (-35.7 to -19.5)	9 (-15.5 to 29.5)	-44.8 (-52 to -36)
High income	Western Europe	Belgium	YLDs	All Ages	29,683 (23,442 to 34,961)	14,037 (10,869 to 17,012)	15,646 (12,151 to 19,069)	50,615 (40,074 to 59,210)	26,052 (20,248 to 31,552)	24,563 (19,135 to 30,104)	70.5 (63.6 to 77.3)	85.6 (74 to 98)	57 (49.3 to 64.6)
High income	Western Europe	Belgium	YLDs	Age-standardized	202.8 (160.4 to 239.2)	165.8 (128.5 to 201.4)	254.9 (197.7 to 309.6)	234.2 (185.9 to 274.4)	218.2 (169 to 263.5)	254.4 (198.2 to 309.8)	15.5 (10.7 to 20.3)	31.6 (22.6 to 40.9)	-0.2 (-4.6 to 4.1)
High income	Western Europe	Cyprus	Incidence	All Ages	1,699 (1,602 to 1,804)	706 (644 to 774)	993 (944 to 1,046)	4,116 (3,929 to 4,312)	1,743 (1,627 to 1,863)	2,373 (2,265 to 2,480)	142.3 (133.6 to 152.3)	146.9 (131.6 to 163.5)	139 (128.5 to 150.5)
High income	Western Europe	Cyprus	Incidence	Age-standardized	213.6 (203.2 to 224.3)	174.5 (162.8 to 187)	258.5 (246.9 to 270.7)	218.3 (208.8 to 228.6)	175.4 (164.9 to 186.8)	261.8 (250.3 to 273.4)	2.2 (-1 to 5.5)	0.5 (-4.7 to 5.6)	1.3 (-2.5 to 5.2)
High income	Western Europe	Cyprus	Prevalence	All Ages	21,106 (19,734 to 22,560)	9,017 (8,320 to 9,791)	12,089 (11,262 to 12,955)	51,510 (48,462 to 54,815)	21,729 (20,221 to 23,498)	29,782 (27,945 to 31,724)	144.1 (134.6 to 153.1)	141 (128.4 to 155.1)	146.4 (134 to 158.4)
High income	Western Europe	Cyprus	Prevalence	Age-standardized	2759.5 (2596.5 to 2928.7)	2283.4 (2120.5 to 2454.3)	3304.8 (3102.9 to 3521.7)	2798.8 (2637.8 to 2970.2)	2266.3 (2113.1 to 2437.6)	3394.5 (3199.6 to 3598.4)	1.4 (-2 to 4.9)	-0.7 (-5.5 to 4.2)	2.7 (-1.9 to 7.1)
High income	Western Europe	Cyprus	Deaths	All Ages	248 (202 to 292)	107 (62 to 139)	141 (118 to 166)	434 (342 to 524)	178 (107 to 222)	257 (201 to 317)	74.9 (35.4 to 114.2)	65.7 (11.5 to 197)	81.9 (42.1 to 127.7)
High income	Western Europe	Cyprus	Deaths	Age-standardized	44.8 (35.3 to 52.5)	38.2 (20.7 to 49.6)	52.2 (43.2 to 61)	26.5 (19.2 to 32.1)	20.1 (10.8 to 25.1)	34.6 (26.5 to 42.3)	-40.9 (-52.6 to -27.9)	-47.5 (-62.4 to -10.9)	-33.7 (-46.8 to -19.8)
High income	Western Europe	Cyprus	DALYs	All Ages	5,113 (4,405 to 5,861)	2,173 (1,510 to 2,676)	2,941 (2,525 to 3,370)	8,761 (7,550 to 10,063)	3,441 (2,786 to 3,985)	5,320 (4,510 to 6,201)	71.3 (46 to 97)	58.4 (25.5 to 137.7)	80.9 (50 to 112.3)
High income	Western Europe	Cyprus	DALYs	Age-standardized	729 (626.4 to 827.1)	603.5 (417.2 to 738.7)	874.7 (752.7 to 989.3)	477.9 (409.3 to 547.1)	356.9 (282.6 to 411.4)	619.6 (526.1 to 720)	-34.4 (-42.7 to -24.5)	-40.9 (-52 to -11.8)	-29.2 (-40.3 to -17.7)
High income	Western Europe	Cyprus	YLLs	All Ages	3,853 (3,189 to 4,575)	1,621 (962 to 2,096)	2,232 (1,860 to 2,644)	5,707 (4,716 to 6,839)	2,123 (1,511 to 2,598)	3,585 (2,898 to 4,412)	48.1 (17.8 to 79.3)	30.9 (-7.1 to 135.8)	60.6 (24.1 to 99.5)
High income	Western Europe	Cyprus	YLLs	Age-standardized	565 (462.6 to 659.5)	464.7 (269.6 to 592.2)	681.7 (576.3 to 794.8)	312 (256 to 372.9)	219.2 (149 to 267.5)	422.1 (335.5 to 516.2)	-44.8 (-54.5 to -33.2)	-52.8 (-65 to -18)	-38.1 (-50.9 to -24)
High income	Western Europe	Cyprus	YLDs	All Ages	1,260 (989 to 1,509)	552 (419 to 681)	709 (546 to 870)	3,054 (2,394 to 3,627)	1,318 (1,015 to 1,619)	1,736 (1,334 to 2,136)	142.3 (130.2 to 154.9)	139 (122.3 to 158.3)	144.9 (129.2 to 160.9)
High income	Western Europe	Cyprus	YLDs	Age-standardized	164 (129.4 to 194.7)	138.9 (106.6 to 170.3)	193 (149 to 234.8)	166 (130.6 to 196.7)	137.7 (106.8 to 169)	197.4 (152 to 241.6)	1.2 (-3.5 to 6.1)	-0.8 (-7.8 to 6.2)	2.3 (-3.7 to 8.5)
High income	Western Europe	Denmark	Incidence	All Ages	19,250 (18,568 to 19,879)	9,010 (8,497 to 9,498)	10,240 (9,963 to 10,495)	31,671 (30,988 to 32,211)	16,831 (16,455 to 17,116)	14,840 (14,447 to 15,184)	64.5 (59.2 to 69.9)	86.8 (76.9 to 97.5)	44.9 (40.7 to 49.5)
High income	Western Europe	Denmark	Incidence	Age-standardized	261.7 (254.1 to 268.8)	233.8 (223.3 to 243.8)	303.6 (296 to 310.9)	302.7 (296.4 to 308.2)	305.2 (299.1 to 310.4)	300.9 (292.5 to 308)	15.7 (12.5 to 18.9)	30.5 (25.2 to 36.2)	-0.9 (-3.9 to 2)
High income	Western Europe	Denmark	Prevalence	All Ages	305,492 (296,696 to 313,693)	162,494 (155,639 to 169,113)	142,998 (139,533 to 146,012)	475,839 (464,045 to 486,314)	261,928 (256,476 to 267,116)	213,910 (205,963 to 220,826)	55.8 (51.5 to 59.9)	61.2 (54.9 to 68.3)	49.6 (44.8 to 54.1)
High income	Western Europe	Denmark	Prevalence	Age-standardized	3936 (3823.9 to 4033)	3757.6 (3634.6 to 3884)	4268.1 (4160.3 to 4364.8)	4299.5 (4174.3 to 4411.8)	4378.1 (4263.1 to 4482.7)	4214 (4045.6 to 4367)	9.2 (6.3 to 12)	16.5 (12.4 to 20.7)	-1.3 (-4.6 to 1.8)
High income	Western Europe	Denmark	Deaths	All Ages	2,887 (2,655 to 3,077)	1,193 (1,021 to 1,337)	1,694 (1,572 to 1,814)	4,275 (3,364 to 4,856)	2,176 (1,294 to 2,638)	2,099 (1,804 to 2,404)	48.1 (22 to 67.3)	82.5 (22.4 to 123.5)	23.9 (6.7 to 43.8)
High income	Western Europe	Denmark	Deaths	Age-standardized	33.5 (30.6 to 35.6)	24.2 (19.5 to 26.8)	49 (45.3 to 52.5)	33.5 (26.4 to 37.9)	30.1 (17.5 to 36.2)	38.8 (33.2 to 44.6)	-0.1 (-16.9 to 12.5)	24 (-12.5 to 50.1)	-20.9 (-32.1 to -7.9)
High income	Western Europe	Denmark	DALYs	All Ages	68,826 (61,968 to 73,640)	31,212 (25,473 to 34,490)	37,614 (34,796 to 40,096)	90,486 (75,704 to 100,150)	46,905 (32,727 to 53,975)	43,580 (38,304 to 48,689)	31.5 (19 to 42.1)	50.3 (26.5 to 69)	15.9 (4.2 to 29)
High income	Western Europe	Denmark	DALYs	Age-standardized	853.4 (761.7 to 912.8)	709.6 (550.1 to 787.2)	1077.2 (993.8 to 1149.4)	771.9 (648.1 to 852.9)	741.6 (530.3 to 850.6)	817.5 (716.7 to 911.6)	-9.6 (-17.5 to -2.5)	4.5 (-7.9 to 16.8)	-24.1 (-31.6 to -15.8)
High income	Western Europe	Denmark	YLLs	All Ages	50,501 (44,324 to 53,622)	21,310 (15,879 to 23,583)	29,192 (27,297 to 31,012)	62,051 (48,395 to 69,977)	31,032 (17,582 to 37,227)	31,019 (26,899 to 35,440)	22.9 (5.8 to 37.6)	45.6 (8.9 to 72.4)	6.3 (-8.4 to 22.4)
High income	Western Europe	Denmark	YLLs	Age-standardized	617.1 (533 to 655.8)	480.1 (332.3 to 534.8)	826 (773 to 877.2)	514.4 (404.7 to 578.6)	475.1 (271 to 569.7)	570 (495.5 to 650.6)	-16.6 (-27.2 to -6.8)	-1 (-21.3 to 16.5)	-31 (-40.5 to -20.5)
High income	Western Europe	Denmark	YLDs	All Ages	18,325 (14,243 to 21,599)	9,902 (7,651 to 12,008)	8,423 (6,527 to 10,237)	28,435 (22,272 to 33,484)	15,873 (12,362 to 19,122)	12,562 (9,712 to 15,334)	55.2 (49.6 to 60.8)	60.3 (51.7 to 69.4)	49.1 (42 to 55.9)
High income	Western Europe	Denmark	YLDs	Age-standardized	236.3 (184.7 to 278.9)	229.5 (176.6 to 277.5)	251.2 (194.6 to 304.7)	257.4 (202.3 to 302.7)	266.5 (205.2 to 322.1)	247.5 (192.1 to 301)	8.9 (5 to 12.9)	16.1 (10.3 to 22.2)	-1.5 (-6.4 to 3.1)
High income	Western Europe	Finland	Incidence	All Ages	9,638 (9,198 to 10,103)	2,885 (2,712 to 3,061)	6,753 (6,409 to 7,116)	15,427 (14,417 to 16,371)	5,721 (5,292 to 6,160)	9,706 (9,010 to 10,356)	60.1 (51.3 to 67.7)	98.3 (85.3 to 111.5)	43.7 (35.5 to 53.8)
High income	Western Europe	Finland	Incidence	Age-standardized	146.8 (140.6 to 153.2)	79.1 (75.2 to 83.6)	241.3 (229.9 to 252.6)	146.8 (138.7 to 155)	102.6 (96.4 to 109)	198.3 (185.9 to 210.1)	0 (-4.3 to 3.8)	29.7 (23.3 to 36.4)	-17.8 (-21.7 to -13.5)
High income	Western Europe	Finland	Prevalence	All Ages	149,351 (142,437 to 156,800)	53,201 (50,239 to 56,486)	96,150 (91,320 to 101,283)	243,426 (229,106 to 257,920)	99,938 (93,371 to 106,798)	143,488 (133,950 to 152,623)	63 (56.2 to 70)	87.8 (77.9 to 98.4)	49.2 (40.7 to 58.4)
High income	Western Europe	Finland	Prevalence	Age-standardized	2220.7 (2115.3 to 2337.7)	1393 (1309 to 1484.7)	3559.9 (3384.7 to 3744.9)	2199.6 (2060.4 to 2341.8)	1697.3 (1570.7 to 1826.8)	2837.9 (2649.2 to 3019.2)	-1 (-5.2 to 3.2)	21.8 (15.4 to 28.8)	-20.3 (-24.7 to -15.6)
High income	Western Europe	Finland	Deaths	All Ages	1,007 (919 to 1,137)	265 (224 to 379)	742 (677 to 802)	1,602 (1,355 to 1,858)	533 (399 to 657)	1,068 (901 to 1,257)	59 (38.8 to 81.3)	101.2 (33.7 to 155.5)	43.9 (22.8 to 68.1)
High income	Western Europe	Finland	Deaths	Age-standardized	13.8 (12.6 to 15.6)	5.6 (4.8 to 7.9)	30.3 (27.5 to 32.7)	11.4 (9.7 to 13.2)	6.4 (4.7 to 7.9)	18.7 (15.8 to 21.9)	-17.3 (-28.4 to -5.7)	14.7 (-26.1 to 44.4)	-38.3 (-47.3 to -28.2)
High income	Western Europe	Finland	DALYs	All Ages	26,189 (23,707 to 28,468)	7,489 (6,533 to 8,899)	18,700 (16,840 to 20,333)	37,658 (33,213 to 42,289)	13,541 (11,016 to 15,830)	24,117 (20,810 to 27,361)	43.8 (31.6 to 57.1)	80.8 (39.9 to 106.8)	29 (15.4 to 43.8)
High income	Western Europe	Finland	DALYs	Age-standardized	370.6 (333.9 to 403.4)	181.2 (157.5 to 209.7)	694 (626.1 to 753.4)	311 (273.5 to 348.9)	209.6 (168.8 to 245.4)	443.2 (384.4 to 501.7)	-16.1 (-23.1 to -8.8)	15.7 (-8.9 to 31)	-36.1 (-42.5 to -29.3)
High income	Western Europe	Finland	YLLs	All Ages	17,492 (16,083 to 19,160)	4,247 (3,674 to 5,684)	13,245 (12,097 to 14,304)	23,505 (20,202 to 27,090)	7,530 (5,305 to 9,077)	15,975 (13,551 to 18,680)	34.4 (16.7 to 53.2)	77.3 (13.9 to 121.6)	20.6 (2.4 to 40.6)
High income	Western Europe	Finland	YLLs	Age-standardized	241.1 (221.9 to 263.2)	95.8 (83.6 to 123.8)	492.5 (450.4 to 532.2)	182.4 (157.4 to 210)	106.1 (71.5 to 127.8)	282.1 (240.5 to 328.3)	-24.3 (-34.6 to -13.8)	10.8 (-29 to 39.2)	-42.7 (-51.3 to -33.3)
High income	Western Europe	Finland	YLDs	All Ages	8,697 (6,804 to 10,445)	3,242 (2,521 to 3,955)	5,455 (4,168 to 6,780)	14,153 (11,002 to 16,958)	6,011 (4,633 to 7,307)	8,142 (6,232 to 10,002)	62.7 (53.4 to 73)	85.4 (71.7 to 101.5)	49.3 (37.6 to 61)
High income	Western Europe	Finland	YLDs	Age-standardized	129.5 (101.3 to 155.5)	85.4 (66.3 to 104.8)	201.6 (154.3 to 249.7)	128.6 (101 to 155.6)	103.5 (79.5 to 127.5)	161.1 (123.2 to 198.7)	-0.7 (-6.1 to 5.6)	21.2 (11.7 to 32.1)	-20.1 (-25.6 to -14.1)
High income	Western Europe	France	Incidence	All Ages	155,654 (149,386 to 162,132)	76,223 (71,813 to 80,761)	79,431 (75,686 to 83,580)	198,333 (188,068 to 209,024)	103,969 (97,942 to 110,865)	94,364 (88,585 to 99,951)	27.4 (21.6 to 33.7)	36.4 (27.5 to 46.5)	18.8 (10.9 to 26.7)
High income	Western Europe	France	Incidence	Age-standardized	188.9 (181.9 to 196.4)	142.1 (134.9 to 149.2)	241.7 (231.1 to 253.7)	144.6 (137.2 to 152.7)	120.8 (113.9 to 129.1)	170.3 (160.7 to 179.9)	-23.5 (-26.7 to -20)	-15 (-19.6 to -9.8)	-29.5 (-33.8 to -25.2)
High income	Western Europe	France	Prevalence	All Ages	1,932,582 (1,844,226 to 2,034,665)	864,979 (812,467 to 922,857)	1,067,603 (1,013,945 to 1,125,707)	2,543,487 (2,406,078 to 2,699,316)	1,253,147 (1,173,692 to 1,339,570	1,290,341 (1,213,532 to 1,371,837)	31.6 (26 to 37.2)	44.9 (37 to 53.3)	20.9 (13.4 to 28.1)
High income	Western Europe	France	Prevalence	Age-standardized	2406.7 (2297.1 to 2531.7)	1788.5 (1674.2 to 1906)	3255.4 (3093.1 to 3427.7)	1966.7 (1850.7 to 2089.3)	1650 (1536.1 to 1772)	2351 (2210.6 to 2502.6)	-18.3 (-21.7 to -15)	-7.7 (-12.5 to -2.9)	-27.8 (-32.1 to -23.6)
High income	Western Europe	France	Deaths	All Ages	17,611 (15,376 to 19,325)	7,239 (5,391 to 8,381)	10,372 (9,402 to 11,401)	17,570 (14,285 to 21,391)	7,701 (5,659 to 10,596)	9,869 (8,218 to 11,719)	-0.2 (-16.4 to 23.1)	6.4 (-19.5 to 48.4)	-4.8 (-21.6 to 16.7)
High income	Western Europe	France	Deaths	Age-standardized	19.6 (17 to 21.4)	11.8 (9 to 13.6)	34.7 (31.2 to 38.4)	9.8 (8.2 to 11.9)	6.5 (5 to 9.4)	15 (12.6 to 17.7)	-49.7 (-57.4 to -38.2)	-45.3 (-57.7 to -20.6)	-56.8 (-64.3 to -46.7)
High income	Western Europe	France	DALYs	All Ages	306,519 (267,873 to 341,280)	114,296 (93,015 to 132,739)	192,222 (169,960 to 215,299)	323,744 (279,313 to 373,113)	136,706 (111,350 to 174,376)	187,038 (161,537 to 214,063)	5.6 (-7.5 to 22.5)	19.6 (-0.3 to 52)	-2.7 (-15.7 to 14.6)
High income	Western Europe	France	DALYs	Age-standardized	359.7 (315.2 to 400.7)	213.4 (176.2 to 248.1)	589.8 (521.8 to 658.3)	228.3 (198.1 to 262.9)	162.1 (133.6 to 208.2)	314.7 (272.2 to 358.5)	-36.5 (-44.2 to -27)	-24 (-36.7 to -4.2)	-46.6 (-53.7 to -37.6)
High income	Western Europe	France	YLLs	All Ages	239,364 (216,578 to 257,362)	84,068 (66,957 to 95,283)	155,296 (142,384 to 168,946)	212,374 (179,942 to 258,406)	81,641 (62,683 to 118,894)	130,733 (111,321 to 152,980)	-11.3 (-24.1 to 6.7)	-2.9 (-23.6 to 33.4)	-15.8 (-29.3 to 1.3)
High income	Western Europe	France	YLLs	Age-standardized	274.9 (249.5 to 294.2)	148.8 (121.4 to 167.9)	477.2 (436.6 to 519.4)	141.3 (121.5 to 173.7)	88 (69.7 to 134.2)	211.9 (181.2 to 247.9)	-48.6 (-55.9 to -37.9)	-40.9 (-52.3 to -17.1)	-55.6 (-62.6 to -47.1)
High income	Western Europe	France	YLDs	All Ages	67,154 (35,390 to 94,797)	30,228 (15,284 to 43,855)	36,926 (18,404 to 53,873)	111,370 (82,282 to 138,511)	55,065 (39,357 to 71,187)	56,305 (40,567 to 72,225)	65.8 (30.1 to 174.8)	82.2 (36.5 to 213.9)	52.5 (16.8 to 161.6)
High income	Western Europe	France	YLDs	Age-standardized	84.9 (46.1 to 118.6)	64.7 (35 to 91.9)	112.6 (56.8 to 163.1)	87 (64.6 to 108.4)	74.2 (54.3 to 95.7)	102.8 (74.2 to 130.8)	2.6 (-18.5 to 66.1)	14.7 (-12.5 to 83.6)	-8.7 (-29.8 to 57.2)
High income	Western Europe	Germany	Incidence	All Ages	254,486 (243,465 to 266,103)	119,045 (110,604 to 127,714)	135,441 (129,861 to 140,841)	402,559 (385,401 to 422,286)	212,466 (202,040 to 223,447)	190,093 (179,459 to 202,185)	58.2 (52.4 to 64.5)	78.5 (67.3 to 91.8)	40.4 (32.7 to 48)
High income	Western Europe	Germany	Incidence	Age-standardized	213.9 (205.6 to 222.4)	160.7 (152.5 to 169.6)	285.7 (275.1 to 296)	235.6 (225.9 to 245.6)	218.6 (208.6 to 229.8)	251.6 (240.5 to 265.1)	10.1 (6.3 to 14.1)	36 (28.6 to 43.8)	-11.9 (-15.8 to -8.1)
High income	Western Europe	Germany	Prevalence	All Ages	3,596,678 (3,446,048 to 3,758,539)	1,695,273 (1,601,095 to 1,790,977	1,901,405 (1,811,901 to 1,982,005)	6,130,746 (5,844,770 to 6,420,710)	3,190,939 (3,022,653 to 3,373,500	2,939,808 (2,793,393 to 3,103,825)	70.5 (64.3 to 76.5)	88.2 (78.5 to 98.3)	54.6 (46.9 to 62.4)
High income	Western Europe	Germany	Prevalence	Age-standardized	2990 (2857.3 to 3120.4)	2349.4 (2219.6 to 2486.2)	4054.6 (3867.6 to 4223.4)	3433.4 (3263.7 to 3592.1)	3213.4 (3027.7 to 3395.7)	3683.7 (3489.6 to 3880.7)	14.8 (10.7 to 18.8)	36.8 (29.4 to 43.9)	-9.1 (-13.6 to -5.1)
High income	Western Europe	Germany	Deaths	All Ages	30,702 (28,126 to 35,852)	11,341 (9,772 to 16,040)	19,361 (18,081 to 20,649)	39,894 (34,589 to 45,760)	17,436 (13,955 to 21,313)	22,458 (19,433 to 25,853)	29.9 (16.4 to 45.7)	53.7 (21.8 to 86.9)	16 (0.4 to 34.4)
High income	Western Europe	Germany	Deaths	Age-standardized	23.1 (21.1 to 26.9)	12.7 (11 to 17.9)	44.5 (41.5 to 47.4)	18.2 (16 to 20.6)	13.4 (10.9 to 15.9)	25 (21.7 to 28.7)	-21.1 (-29.3 to -12.2)	5.2 (-19.2 to 26.3)	-43.8 (-51.1 to -35.4)
High income	Western Europe	Germany	DALYs	All Ages	726,416 (668,759 to 796,818)	279,984 (247,036 to 340,296)	446,432 (414,026 to 480,809)	973,415 (871,716 to 1,074,527)	448,386 (382,647 to 512,810)	525,030 (463,066 to 589,045)	34 (24.1 to 43.9)	60.1 (33.7 to 77.5)	17.6 (6.3 to 29.9)
High income	Western Europe	Germany	DALYs	Age-standardized	575.7 (527.6 to 632.3)	360.9 (319.7 to 427.1)	955.1 (886.2 to 1027.1)	519 (465.7 to 573.6)	432.6 (368.4 to 491.7)	625.6 (551.3 to 701.3)	-9.8 (-16.7 to -3.1)	19.8 (0.9 to 31.5)	-34.5 (-40.9 to -27.8)
High income	Western Europe	Germany	YLLs	All Ages	494,896 (460,225 to 560,095)	166,003 (146,912 to 224,970)	328,893 (309,104 to 350,196)	580,928 (515,387 to 653,110)	236,635 (193,291 to 278,396)	344,294 (301,526 to 392,828)	17.4 (4.8 to 30.5)	42.5 (8.2 to 69.6)	4.7 (-8.9 to 20)
High income	Western Europe	Germany	YLLs	Age-standardized	382.8 (356.9 to 431.6)	201.9 (180.5 to 265.4)	704.9 (664 to 749.5)	298 (266.1 to 332.7)	216.8 (174.6 to 251.4)	398.7 (348.1 to 454.4)	-22.2 (-31.1 to -12.9)	7.4 (-20.3 to 27.7)	-43.4 (-50.9 to -35)
High income	Western Europe	Germany	YLDs	All Ages	231,520 (180,920 to 275,317)	113,980 (90,221 to 137,103)	117,539 (91,617 to 144,669)	392,487 (308,349 to 464,305)	211,751 (166,409 to 255,327)	180,736 (140,206 to 221,519)	69.5 (61.4 to 77.8)	85.8 (73.6 to 98.5)	53.8 (44.4 to 63.7)
High income	Western Europe	Germany	YLDs	Age-standardized	192.8 (150.2 to 229.5)	159 (125.4 to 190.9)	250.2 (195.4 to 307.5)	221 (174.1 to 261.9)	215.8 (169.9 to 261.3)	226.9 (176.3 to 277.9)	14.6 (9 to 20.1)	35.7 (26.6 to 45.3)	-9.3 (-14.5 to -4)
High income	Western Europe	Greece	Incidence	All Ages	27,710 (26,482 to 29,061)	13,948 (13,265 to 14,681)	13,763 (12,981 to 14,511)	49,178 (47,520 to 51,094)	26,014 (25,016 to 27,069)	23,164 (22,278 to 24,255)	77.5 (71.4 to 83.8)	86.5 (78.2 to 95.9)	68.3 (59.9 to 77.6)
High income	Western Europe	Greece	Incidence	Age-standardized	190.5 (182.9 to 198.6)	173.9 (166.4 to 182.3)	208 (197.6 to 218.2)	211 (202.5 to 221)	194.1 (184.6 to 204.7)	228.2 (218.2 to 239.4)	10.8 (7 to 14.5)	11.6 (6.8 to 17)	9.7 (4.5 to 15.2)
High income	Western Europe	Greece	Prevalence	All Ages	353,147 (334,886 to 373,895)	171,646 (162,239 to 182,457)	181,501 (171,023 to 192,669)	657,517 (626,678 to 693,971)	332,936 (314,388 to 354,034)	324,581 (308,347 to 342,592)	86.2 (78.5 to 94.1)	94 (83.7 to 104.7)	78.8 (69.5 to 89.2)
High income	Western Europe	Greece	Prevalence	Age-standardized	2490.3 (2362.6 to 2631.7)	2247.1 (2122 to 2385.2)	2779.7 (2626.1 to 2949.1)	2824.2 (2694.8 to 2976.4)	2547.2 (2405.2 to 2704.3)	3136.7 (2980 to 3315)	13.4 (9.4 to 17.7)	13.4 (8.2 to 19.1)	12.8 (7 to 19.1)
High income	Western Europe	Greece	Deaths	All Ages	2,356 (2,123 to 2,553)	1,017 (851 to 1,179)	1,339 (1,216 to 1,459)	5,961 (4,640 to 6,957)	2,762 (1,540 to 3,497)	3,199 (2,623 to 3,727)	153 (102 to 197.9)	171.5 (65.2 to 250.8)	139 (96.9 to 182.7)
High income	Western Europe	Greece	Deaths	Age-standardized	16.4 (14.7 to 17.8)	12.6 (10.3 to 14.6)	21.4 (19.5 to 23.2)	18.7 (15 to 21.7)	15 (8.9 to 18.7)	23.6 (19.6 to 27.4)	14.4 (-3.6 to 33.2)	19.2 (-20.2 to 51.9)	10.2 (-8 to 28.8)
High income	Western Europe	Greece	DALYs	All Ages	61,152 (54,936 to 66,433)	27,135 (23,691 to 30,616)	34,018 (30,562 to 36,982)	119,485 (103,967 to 132,861)	55,687 (43,707 to 64,698)	63,798 (55,337 to 71,878)	95.4 (72.4 to 117.2)	105.2 (59.1 to 140.5)	87.5 (65.3 to 110.9)
High income	Western Europe	Greece	DALYs	Age-standardized	416.1 (372.7 to 452.3)	339.5 (296.6 to 382.8)	510.7 (459.4 to 555.2)	466.4 (412.7 to 517.8)	383.8 (319.9 to 440.6)	564.9 (491.1 to 635.6)	12.1 (0.5 to 23.4)	13.1 (-6.3 to 30.1)	10.6 (-2 to 23.1)
High income	Western Europe	Greece	YLLs	All Ages	34,330 (31,768 to 38,025)	13,844 (12,198 to 16,922)	20,486 (18,794 to 22,074)	67,915 (56,543 to 78,214)	29,222 (18,557 to 36,135)	38,693 (32,613 to 44,793)	97.8 (62.5 to 130.7)	111.1 (34.7 to 168.4)	88.9 (57.7 to 122.7)
High income	Western Europe	Greece	YLLs	Age-standardized	227.1 (209.4 to 249.2)	165.3 (145.5 to 201)	303.8 (279.7 to 326.8)	244.8 (210.1 to 278.4)	180.4 (127.1 to 217.3)	322.8 (275.7 to 370.5)	7.8 (-7.1 to 24.4)	9.1 (-21.6 to 35.7)	6.3 (-10.9 to 24.9)
High income	Western Europe	Greece	YLDs	All Ages	26,822 (21,653 to 30,958)	13,290 (10,556 to 15,754)	13,532 (10,525 to 16,077)	51,570 (41,917 to 60,889)	26,465 (20,869 to 32,308)	25,105 (19,642 to 30,548)	92.3 (70.4 to 125.6)	99.1 (70.7 to 140.8)	85.5 (57.3 to 121.3)
High income	Western Europe	Greece	YLDs	Age-standardized	189.1 (153.4 to 217.9)	174.2 (138.4 to 205.7)	206.9 (162.1 to 245.3)	221.6 (179.5 to 260.4)	203.4 (162.2 to 246.9)	242.1 (189 to 291.2)	17.2 (4.5 to 35.7)	16.8 (0.7 to 39)	17 (0.4 to 37.8)
High income	Western Europe	Iceland	Incidence	All Ages	606 (575 to 636)	326 (309 to 343)	280 (264 to 297)	1,161 (1,111 to 1,215)	626 (597 to 656)	535 (503 to 567)	91.6 (84.7 to 98.9)	92.2 (82.9 to 102.3)	90.9 (81.7 to 101.1)
High income	Western Europe	Iceland	Incidence	Age-standardized	215.9 (205.3 to 226.8)	214.5 (204 to 226.7)	218.1 (205.8 to 230.5)	217.7 (208.3 to 228.4)	223.7 (212.6 to 236)	211.4 (199.5 to 223.2)	0.8 (-2.5 to 4.4)	4.3 (-0.8 to 9.6)	-3.1 (-7.4 to 1.8)
High income	Western Europe	Iceland	Prevalence	All Ages	8,611 (8,164 to 9,066)	4,788 (4,530 to 5,058)	3,823 (3,599 to 4,058)	16,033 (15,245 to 16,915)	9,025 (8,529 to 9,578)	7,007 (6,610 to 7,438)	86.2 (79.9 to 93.1)	88.5 (79.6 to 97.6)	83.3 (74.8 to 92.9)
High income	Western Europe	Iceland	Prevalence	Age-standardized	3040.6 (2878.2 to 3200.5)	3119.7 (2940.1 to 3287.5)	2974.5 (2800.2 to 3158)	2938.2 (2786.1 to 3103.2)	3087.5 (2900.6 to 3284.4)	2779.1 (2621 to 2942.7)	-3.4 (-6.6 to 0.2)	-1 (-5.6 to 4.1)	-6.6 (-10.9 to -1.8)
High income	Western Europe	Iceland	Deaths	All Ages	59 (52 to 67)	31 (26 to 39)	28 (25 to 32)	99 (79 to 114)	53 (38 to 64)	46 (38 to 54)	67.4 (40.8 to 92.3)	69.8 (23.8 to 104.7)	64.6 (36.9 to 95.8)
High income	Western Europe	Iceland	Deaths	Age-standardized	19.5 (17.2 to 22.1)	17.8 (15 to 22.1)	22.1 (19.3 to 24.8)	15.5 (12.5 to 17.7)	14.3 (10.5 to 17.1)	17 (14.1 to 19.8)	-20.7 (-33.5 to -8.4)	-19.4 (-42.3 to -2.8)	-23.1 (-36 to -8.6)
High income	Western Europe	Iceland	DALYs	All Ages	1,490 (1,343 to 1,644)	826 (731 to 937)	664 (587 to 736)	2,401 (2,106 to 2,665)	1,339 (1,131 to 1,518)	1,063 (924 to 1,208)	61.2 (46.7 to 75.8)	62.1 (36.5 to 79.8)	60 (42.3 to 80)
High income	Western Europe	Iceland	DALYs	Age-standardized	515.5 (464.5 to 569.9)	524.4 (464.3 to 594.2)	513.1 (453.3 to 569.1)	426.5 (374.9 to 471.8)	445.3 (377.4 to 505.8)	408.1 (353.6 to 463.5)	-17.3 (-24.4 to -9.9)	-15.1 (-28.3 to -6.2)	-20.5 (-29.4 to -10.5)
High income	Western Europe	Iceland	YLLs	All Ages	891 (805 to 1,001)	461 (397 to 571)	430 (384 to 479)	1,293 (1,080 to 1,468)	657 (488 to 774)	637 (534 to 738)	45.2 (23 to 68)	42.6 (3.6 to 72.1)	48 (23.8 to 77.7)
High income	Western Europe	Iceland	YLLs	Age-standardized	303.7 (275.1 to 342)	285.6 (247.6 to 350.9)	331.1 (293.6 to 368.9)	222.8 (187.9 to 252.2)	209.9 (154.8 to 246.6)	239 (200.9 to 277.1)	-26.7 (-37.8 to -14.8)	-26.5 (-46.8 to -11.3)	-27.8 (-39.6 to -13.2)
High income	Western Europe	Iceland	YLDs	All Ages	599 (482 to 699)	365 (292 to 434)	234 (181 to 286)	1,108 (890 to 1,293)	682 (542 to 806)	426 (326 to 525)	84.9 (77.3 to 93)	86.8 (75.8 to 98.9)	82 (70.7 to 94.4)
High income	Western Europe	Iceland	YLDs	Age-standardized	211.8 (170.6 to 247.4)	238.8 (191.1 to 283.9)	182 (141.2 to 222.1)	203.7 (163.2 to 238.5)	235.4 (188.2 to 279.1)	169.2 (129.9 to 207.5)	-3.8 (-7.8 to 0.5)	-1.4 (-7.3 to 5.4)	-7.1 (-12.7 to -0.9)
High income	Western Europe	Ireland	Incidence	All Ages	12,096 (11,869 to 12,337)	6,362 (6,152 to 6,570)	5,734 (5,663 to 5,794)	19,537 (18,906 to 20,206)	10,176 (9,800 to 10,584)	9,360 (8,977 to 9,683)	61.5 (56.2 to 66.4)	59.9 (53.5 to 66.8)	63.3 (56.4 to 69.1)
High income	Western Europe	Ireland	Incidence	Age-standardized	302.9 (297.5 to 308.3)	293.3 (284.1 to 302)	314.8 (311 to 318)	270.5 (261.1 to 280.2)	268.6 (258.1 to 280.3)	272.6 (261.9 to 282.1)	-10.7 (-13.6 to -8)	-8.4 (-12.2 to -4.6)	-13.4 (-16.9 to -10.3)
High income	Western Europe	Ireland	Prevalence	All Ages	166,701 (161,683 to 171,505)	91,258 (87,473 to 94,740)	75,443 (73,356 to 77,280)	267,359 (254,770 to 280,102)	144,478 (137,094 to 152,715)	122,880 (115,980 to 128,977)	60.4 (53.4 to 66.9)	58.3 (50.3 to 66.5)	62.9 (53.8 to 71.2)
High income	Western Europe	Ireland	Prevalence	Age-standardized	4169 (4040.6 to 4293.9)	4107.1 (3935.4 to 4269.4)	4228.1 (4108 to 4331)	3656.1 (3483.6 to 3827)	3686.6 (3491.3 to 3905.8)	3624.3 (3427.4 to 3803.7)	-12.3 (-15.9 to -8.7)	-10.2 (-14.8 to -5.5)	-14.3 (-18.8 to -10.1)
High income	Western Europe	Ireland	Deaths	All Ages	2,052 (1,795 to 2,174)	791 (573 to 868)	1,261 (1,186 to 1,336)	2,183 (1,860 to 2,525)	1,045 (761 to 1,276)	1,138 (945 to 1,345)	6.4 (-8.9 to 22.5)	32.1 (9 to 58.6)	-9.7 (-25.2 to 8.9)
High income	Western Europe	Ireland	Deaths	Age-standardized	50.6 (44.3 to 53.6)	33.6 (24.5 to 37)	77.6 (72.7 to 82.2)	27.6 (23.6 to 31.9)	23 (16.9 to 28.1)	34.4 (28.6 to 40.5)	-45.4 (-53 to -37.4)	-31.4 (-42.9 to -18.4)	-55.7 (-63 to -47.2)
High income	Western Europe	Ireland	DALYs	All Ages	43,260 (38,591 to 46,107)	17,966 (13,979 to 19,786)	25,294 (23,599 to 27,116)	44,786 (39,741 to 49,995)	22,197 (18,581 to 25,442)	22,589 (19,760 to 25,894)	3.5 (-6.4 to 15.4)	23.5 (10.1 to 42.7)	-10.7 (-21.5 to 3.6)
High income	Western Europe	Ireland	DALYs	Age-standardized	1038.6 (926.8 to 1109)	776.5 (605.3 to 857)	1406.2 (1314.4 to 1501.5)	589.3 (523.9 to 657)	541.3 (454.7 to 621.1)	654.7 (572.9 to 749.5)	-43.3 (-48.5 to -36.9)	-30.3 (-37.6 to -19.2)	-53.4 (-58.9 to -46.4)

Chronic obstructive pulmonary disease

7 GBD super region	21 GBD region	Location	Measure	Age	1990			2019			Percent change (1990 to 2019)		
					Both	Female	Male	Both	Female	Male	Both	Female	Male
High income	Western Europe	Ireland	YLLs	All Ages	33,212 (28,999 to 35,223)	12,383 (8,677 to 13,632)	20,829 (19,518 to 22,192)	28,828 (24,953 to 33,155)	13,433 (10,210 to 16,143)	15,395 (12,865 to 18,329)	-13.2 (-25.6 to 1.6)	8.5 (-10 to 32.7)	-26.1 (-38.7 to -9.9)
High income	Western Europe	Ireland	YLLs	Age-standardized	787.5 (688.4 to 837.1)	525.3 (370.1 to 577.9)	1156.3 (1085.2 to 1228.4)	370.7 (321 to 426.3)	316.9 (244.8 to 380.8)	442.6 (370.9 to 524.9)	-52.9 (-59.5 to -44.7)	-39.7 (-49.7 to -25)	-61.7 (-68.1 to -53.5)
High income	Western Europe	Ireland	YLDs	All Ages	10,048 (7,943 to 11,873)	5,583 (4,326 to 6,779)	4,465 (3,465 to 5,447)	15,959 (12,624 to 18,847)	8,764 (6,762 to 10,692)	7,194 (5,546 to 8,772)	58.8 (50.6 to 66.4)	57 (47.4 to 66.8)	61.1 (50.5 to 71.6)
High income	Western Europe	Ireland	YLDs	Age-standardized	251.1 (199 to 296.6)	251.2 (193.7 to 305)	249.9 (194.2 to 303.9)	218.6 (172.5 to 257.8)	224.4 (172.8 to 272.7)	212.1 (164.2 to 258.1)	-12.9 (-17.3 to -8.7)	-10.7 (-16.3 to -4.9)	-15.1 (-20.5 to -9.8)
High income	Western Europe	Israel	Incidence	All Ages	8,458 (8,045 to 8,954)	4,003 (3,768 to 4,276)	4,455 (4,200 to 4,719)	18,309 (17,452 to 19,221)	9,470 (8,951 to 10,022)	8,839 (8,369 to 9,314)	116.5 (107.5 to 124.5)	136.6 (123.2 to 150.4)	98.4 (88.7 to 109.7)
High income	Western Europe	Israel	Incidence	Age-standardized	180 (172.4 to 188.8)	158 (149.9 to 167.4)	205 (194.8 to 216.4)	161.5 (153.9 to 169.7)	149 (141.2 to 157.8)	172.7 (163.6 to 181.7)	-10.3 (-13.4 to -7.2)	-5.7 (-10.1 to -0.7)	-15.8 (-19.6 to -11.6)
High income	Western Europe	Israel	Prevalence	All Ages	114,299 (108,540 to 121,163)	52,328 (49,159 to 55,892)	61,971 (58,433 to 65,693)	253,531 (240,808 to 267,767)	124,079 (117,355 to 131,947)	129,453 (122,249 to 136,904)	121.8 (114.2 to 130.3)	137.1 (125 to 150.4)	108.9 (99 to 119.2)
High income	Western Europe	Israel	Prevalence	Age-standardized	2460.3 (2342.8 to 2604.5)	2119.9 (1997.8 to 2255.3)	2864.7 (2706.4 to 3035.6)	2256.1 (2138.4 to 2389.6)	2004.2 (1888.8 to 2132.8)	2530.1 (2387.2 to 2681.3)	-8.3 (-11.3 to -5)	-5.5 (-10 to -0.5)	-11.7 (-15.8 to -7.4)
High income	Western Europe	Israel	Deaths	All Ages	1,040 (952 to 1,124)	418 (355 to 473)	623 (571 to 678)	1,720 (1,488 to 2,105)	790 (631 to 1,124)	930 (804 to 1,062)	65.3 (45 to 97.8)	89.1 (55.2 to 147.3)	49.4 (29.5 to 74.9)
High income	Western Europe	Israel	Deaths	Age-standardized	23.3 (20.9 to 25.2)	17.4 (14.7 to 19.6)	30.3 (27.8 to 32.8)	13.6 (11.9 to 16.6)	10.6 (8.5 to 15.1)	17.4 (15 to 19.8)	-41.5 (-48.4 to -29.5)	-39.2 (-50 to -18.1)	-42.7 (-50.2 to -33.1)
High income	Western Europe	Israel	DALYs	All Ages	23,265 (21,365 to 25,210)	9,541 (8,410 to 10,673)	13,724 (12,612 to 14,985)	39,412 (34,992 to 45,367)	17,465 (14,823 to 22,029)	21,947 (19,416 to 24,631)	69.4 (55.9 to 90)	83 (62.6 to 120.6)	59.9 (44.4 to 77.6)
High income	Western Europe	Israel	DALYs	Age-standardized	490.4 (449.8 to 531.5)	374.2 (327.6 to 417)	628.9 (577 to 687)	339.4 (301.6 to 388.2)	268.9 (227.8 to 337.8)	420.6 (372.3 to 472.7)	-30.8 (-36.4 to -22.3)	-28.1 (-36 to -12.6)	-33.1 (-39.4 to -25.7)
High income	Western Europe	Israel	YLLs	All Ages	16,373 (15,220 to 17,656)	6,330 (5,561 to 7,218)	10,043 (9,273 to 10,848)	24,204 (21,296 to 29,573)	9,911 (8,081 to 14,393)	14,293 (12,427 to 16,378)	47.8 (29.7 to 76.7)	56.6 (29.3 to 115.7)	42.3 (23.2 to 66.2)
High income	Western Europe	Israel	YLLs	Age-standardized	342.3 (316.4 to 368)	244.4 (211.1 to 275.9)	458.9 (424.6 to 495.5)	203.7 (179.4 to 248)	146.3 (119.4 to 215.2)	270.9 (235.6 to 310.5)	-40.5 (-47.8 to -28.1)	-40.1 (-50.9 to -15.8)	-41 (-48.9 to -31)
High income	Western Europe	Israel	YLDs	All Ages	6,892 (5,369 to 8,165)	3,211 (2,481 to 3,917)	3,681 (2,817 to 4,526)	15,209 (11,915 to 17,985)	7,554 (5,866 to 9,152)	7,655 (5,875 to 9,390)	120.7 (110.1 to 132.4)	135.2 (118.1 to 153)	108 (95 to 121.8)
High income	Western Europe	Israel	YLDs	Age-standardized	148.1 (115.6 to 174.9)	129.8 (101 to 158.1)	170 (130.2 to 208.8)	135.6 (106.4 to 160.5)	122.6 (95.6 to 149.2)	149.7 (115.1 to 184)	-8.4 (-12.8 to -3.9)	-5.5 (-12.3 to 1.4)	-12 (-17.2 to -5.9)
High income	Western Europe	Italy	Incidence	All Ages	172,032 (156,926 to 185,906)	75,291 (66,794 to 84,701)	96,741 (88,807 to 104,003)	258,698 (236,063 to 278,455)	127,467 (114,285 to 139,691)	131,231 (120,265 to 141,876)	50.4 (44.1 to 56)	69.3 (60.3 to 77.8)	35.7 (27.6 to 43.4)
High income	Western Europe	Italy	Incidence	Age-standardized	202.4 (187 to 217.2)	150 (135.3 to 166.3)	264 (244.7 to 281.6)	181.3 (166 to 195.7)	147.5 (133.9 to 161.7)	217.6 (198.8 to 235.5)	-10.4 (-13.7 to -7.4)	-1.7 (-5.1 to 2.1)	-17.6 (-21.8 to -13.4)
High income	Western Europe	Italy	Prevalence	All Ages	2,266,653 (2,084,678 to 2,452,655)	961,236 (867,037 to 1,069,621)	1,305,417 (1,199,997 to 1,413,526)	3,253,110 (2,970,227 to 3,542,194)	1,548,365 (1,404,734 to 1,705,405)	1,704,745 (1,540,786 to 1,869,930)	43.5 (36.9 to 49.6)	61.1 (53.6 to 68.7)	30.6 (22.5 to 39.3)
High income	Western Europe	Italy	Prevalence	Age-standardized	2684.6 (2474.2 to 2898.8)	1998.7 (1814.4 to 2207)	3633.2 (3349 to 3910.2)	2287.1 (2094.5 to 2491.2)	1877.8 (1709.5 to 2066.5)	2794.3 (2542.8 to 3046.5)	-14.8 (-18.1 to -11.6)	-6.1 (-9.2 to -3)	-23.1 (-27.2 to -18.4)
High income	Western Europe	Italy	Deaths	All Ages	19,633 (18,354 to 20,724)	6,227 (5,426 to 7,080)	13,406 (12,738 to 13,885)	28,080 (23,682 to 30,851)	11,544 (8,463 to 13,163)	16,536 (14,470 to 17,996)	43 (27.2 to 56)	85.4 (52.5 to 109.4)	23.3 (11.1 to 34.9)
High income	Western Europe	Italy	Deaths	Age-standardized	22 (20.3 to 23.2)	11.4 (9.8 to 12.9)	40.3 (37.5 to 42.1)	14.8 (12.6 to 16.2)	9.5 (7.3 to 10.8)	23.5 (20.6 to 25.5)	-32.8 (-38.9 to -27.1)	-16.7 (-26.2 to -7.2)	-41.8 (-46.8 to -36.6)
High income	Western Europe	Italy	DALYs	All Ages	427,450 (395,128 to 458,757)	140,865 (125,006 to 161,644)	286,585 (266,243 to 304,529)	504,846 (447,328 to 553,424)	209,634 (177,056 to 237,141)	295,213 (263,825 to 323,630)	18.1 (10 to 26)	48.8 (34.7 to 61.2)	3 (-4 to 10.6)
High income	Western Europe	Italy	DALYs	Age-standardized	481.9 (444.1 to 517.4)	275.9 (245.3 to 316.1)	789.9 (733 to 840.1)	325 (290.3 to 358.6)	232.9 (201.9 to 265.8)	452.5 (404.9 to 497.6)	-32.6 (-36.3 to -27.7)	-15.6 (-20.6 to -9.1)	-42.7 (-46.4 to -38.7)
High income	Western Europe	Italy	YLLs	All Ages	297,549 (283,521 to 318,112)	85,093 (76,844 to 102,280)	212,456 (204,207 to 219,194)	319,792 (277,771 to 352,470)	120,952 (95,494 to 138,590)	198,840 (177,906 to 216,360)	7.5 (-3.3 to 18)	42.1 (20.9 to 60.7)	-6.4 (-15.6 to 2.8)
High income	Western Europe	Italy	YLLs	Age-standardized	327.1 (310.6 to 349.4)	158.2 (142.8 to 190.9)	583.8 (558.1 to 603.5)	192 (169.6 to 214.9)	121.2 (100.2 to 150.9)	293 (263.2 to 319.4)	-41.3 (-46.4 to -35.2)	-23.3 (-31.1 to -12.7)	-49.8 (-54.2 to -44.9)
High income	Western Europe	Italy	YLDs	All Ages	129,901 (101,677 to 155,932)	55,772 (42,710 to 68,465)	74,129 (56,480 to 90,747)	185,054 (145,751 to 220,996)	88,682 (67,012 to 109,450)	96,372 (74,217 to 118,619)	42.5 (34.3 to 49.6)	59 (49.5 to 68.1)	30 (20.8 to 39.7)
High income	Western Europe	Italy	YLDs	Age-standardized	154.8 (121 to 185.2)	117.8 (90.7 to 143.8)	206.1 (157.6 to 251.4)	132.9 (105.4 to 159.3)	111.7 (85.5 to 135.8)	159.5 (123.2 to 193.8)	-14.1 (-17.7 to -10.7)	-5.2 (-8.7 to -1.5)	-22.6 (-26.9 to -17.7)
High income	Western Europe	Luxembourg	Incidence	All Ages	1,259 (1,216 to 1,304)	591 (558 to 628)	667 (648 to 685)	2,364 (2,271 to 2,467)	1,243 (1,191 to 1,300)	1,121 (1,071 to 1,178)	87.8 (81.9 to 94.1)	110.2 (99.6 to 123)	68 (60.8 to 76.4)
High income	Western Europe	Luxembourg	Incidence	Age-standardized	241.1 (233.6 to 248.7)	189.9 (180.4 to 200.3)	302.4 (293.8 to 309.7)	246.1 (236.1 to 257.7)	239.2 (227.4 to 252.8)	252.4 (241.2 to 264.6)	2.1 (-1.2 to 5.3)	26 (20.1 to 32.8)	-16.5 (-19.9 to -12.5)
High income	Western Europe	Luxembourg	Prevalence	All Ages	17,174 (16,525 to 17,875)	8,067 (7,599 to 8,570)	9,108 (8,766 to 9,422)	33,204 (31,539 to 35,080)	17,971 (16,982 to 19,119)	15,234 (14,405 to 16,136)	93.3 (86.3 to 100.4)	122.8 (111.8 to 134.4)	67.3 (59.4 to 76.2)
High income	Western Europe	Luxembourg	Prevalence	Age-standardized	3283.3 (3156.1 to 3415.5)	2651.9 (2501.2 to 2811.9)	4219.9 (4066.8 to 4359.3)	3362 (3190.1 to 3558.4)	3290.3 (3100.4 to 3518.7)	3437.6 (3256.3 to 3630.8)	2.4 (-1.3 to 5.9)	24.1 (18 to 30.3)	-18.5 (-22.3 to -14.4)
High income	Western Europe	Luxembourg	Deaths	All Ages	146 (134 to 160)	49 (42 to 63)	96 (88 to 105)	196 (162 to 229)	90 (67 to 113)	106 (87 to 128)	34.7 (13.1 to 59.3)	83.5 (36.3 to 130.2)	9.8 (-10.5 to 34.7)
High income	Western Europe	Luxembourg	Deaths	Age-standardized	26.8 (24.6 to 29.5)	14.5 (12.5 to 18.2)	50.3 (45.9 to 54.6)	17.5 (14.5 to 20.4)	13.1 (9.9 to 16.4)	24 (19.8 to 29)	-34.8 (-44.8 to -22.6)	-9.4 (-31 to 12.8)	-52.2 (-60.9 to -42)
High income	Western Europe	Luxembourg	DALYs	All Ages	3,386 (3,097 to 3,691)	1,224 (1,077 to 1,437)	2,163 (1,972 to 2,364)	4,637 (4,042 to 5,262)	2,200 (1,826 to 2,548)	2,437 (2,085 to 2,801)	36.9 (22.9 to 52.8)	79.8 (51.8 to 103.6)	12.7 (-2 to 29.8)
High income	Western Europe	Luxembourg	DALYs	Age-standardized	623.5 (568.8 to 678.1)	382.7 (336.4 to 443.1)	1014.6 (926.5 to 1107.1)	457.5 (397.8 to 520.6)	389.8 (324.1 to 453)	543.3 (465.5 to 624.1)	-26.6 (-34.1 to -18.2)	1.9 (-12.3 to 14.9)	-46.4 (-53.2 to -38.5)
High income	Western Europe	Luxembourg	YLLs	All Ages	2,354 (2,169 to 2,565)	729 (635 to 929)	1,625 (1,480 to 1,789)	2,660 (2,232 to 3,127)	1,117 (855 to 1,392)	1,542 (1,283 to 1,867)	13 (-5.7 to 34.1)	53.2 (15.5 to 89.8)	-5.1 (-23 to 17)
High income	Western Europe	Luxembourg	YLLs	Age-standardized	426.3 (392.6 to 465)	219.9 (192 to 275.2)	765.8 (697.3 to 838.5)	256.6 (215.7 to 302.2)	190.1 (146.7 to 238.2)	341.6 (284.8 to 412.9)	-39.8 (-49.6 to -28.6)	-13.6 (-33.4 to 8.4)	-55.4 (-63.6 to -45.3)
High income	Western Europe	Luxembourg	YLDs	All Ages	1,032 (809 to 1,216)	494 (383 to 602)	538 (416 to 656)	1,977 (1,560 to 2,340)	1,083 (838 to 1,310)	894 (691 to 1,106)	91.6 (83.2 to 100.7)	119.1 (106.2 to 134.3)	66.3 (56.6 to 77.7)
High income	Western Europe	Luxembourg	YLDs	Age-standardized	197.3 (155.1 to 233)	162.7 (127.2 to 198.2)	248.8 (192.8 to 301.9)	200.8 (157.6 to 238.2)	199.8 (153.6 to 242.4)	201.7 (155.6 to 249.1)	1.8 (-2.7 to 6.7)	22.8 (14.9 to 31.6)	-18.9 (-23.4 to -13.6)
High income	Western Europe	Malta	Incidence	All Ages	803 (767 to 838)	283 (265 to 301)	520 (497 to 542)	1,568 (1,490 to 1,645)	599 (556 to 642)	969 (920 to 1,019)	95.3 (87.5 to 103.4)	111.5 (98.8 to 125.1)	86.5 (76.6 to 96.9)
High income	Western Europe	Malta	Incidence	Age-standardized	194 (186.1 to 201.8)	125.3 (118.1 to 132.3)	276.7 (266.2 to 288)	177.9 (169.8 to 186)	123.6 (115.5 to 131.4)	236.4 (226.3 to 247.6)	-8.3 (-11.6 to -5.2)	-1.3 (-6.2 to 3.7)	-14.6 (-18.2 to -10.6)
High income	Western Europe	Malta	Prevalence	All Ages	10,624 (10,071 to 11,231)	3,929 (3,663 to 4,215)	6,695 (6,324 to 7,082)	20,055 (18,967 to 21,195)	7,899 (7,377 to 8,403)	12,157 (11,427 to 12,941)	88.8 (81 to 96.8)	101.1 (89.9 to 112.6)	81.6 (71.5 to 92.4)
High income	Western Europe	Malta	Prevalence	Age-standardized	2583 (2449.3 to 2728)	1749.9 (1632.3 to 1876.2)	3701.7 (3502.9 to 3903.5)	2329.1 (2193.3 to 2460)	1730.6 (1610 to 1850)	3066.7 (2893.6 to 3243.5)	-9.8 (-13.3 to -6.3)	-1.1 (-5.9 to 3.7)	-17.2 (-21.5 to -12.7)
High income	Western Europe	Malta	Deaths	All Ages	85 (77 to 94)	16 (13 to 19)	70 (62 to 77)	123 (101 to 146)	28 (21 to 37)	95 (77 to 113)	44 (17.4 to 74.6)	76.9 (33.5 to 135.9)	36.5 (9.5 to 67.7)
High income	Western Europe	Malta	Deaths	Age-standardized	21.2 (18.9 to 23.4)	6.8 (5.7 to 8.2)	43.6 (38.8 to 48.8)	11.9 (9.8 to 14.1)	4.5 (3.3 to 5.9)	22.8 (18.5 to 26.9)	-44 (-54.2 to -32.1)	-34.1 (-49.8 to -12.2)	-47.9 (-57.8 to -35.9)
High income	Western Europe	Malta	DALYs	All Ages	2,019 (1,831 to 2,197)	483 (418 to 554)	1,536 (1,381 to 1,694)	2,904 (2,513 to 3,293)	828 (675 to 976)	2,076 (1,783 to 2,364)	43.8 (25.8 to 63.4)	71.6 (50.7 to 99.6)	35.1 (16 to 55.7)
High income	Western Europe	Malta	DALYs	Age-standardized	481.2 (435.4 to 523.8)	208.1 (180.9 to 238.5)	861.4 (774.1 to 949.7)	313.7 (272.1 to 354.2)	169.4 (139.2 to 199.7)	497.6 (428.9 to 566.5)	-34.8 (-42.7 to -26.6)	-18.6 (-27.9 to -6.7)	-42.2 (-50.3 to -33.3)
High income	Western Europe	Malta	YLLs	All Ages	1,384 (1,246 to 1,521)	243 (204 to 295)	1,141 (1,017 to 1,270)	1,711 (1,400 to 2,040)	351 (262 to 462)	1,360 (1,109 to 1,614)	23.6 (1 to 50)	44.6 (9.3 to 96.2)	19.2 (-5.9 to 47)
High income	Western Europe	Malta	YLLs	Age-standardized	327 (293.8 to 359)	101.2 (85.4 to 122.3)	643.6 (574.1 to 715.8)	174.7 (143.6 to 208.3)	63.8 (48.3 to 83.6)	317.2 (258.2 to 376.8)	-46.6 (-56.5 to -35.4)	-37 (-51.9 to -14.9)	-50.7 (-60.6 to -39.3)
High income	Western Europe	Malta	YLDs	All Ages	635 (495 to 756)	240 (187 to 294)	395 (302 to 487)	1,193 (944 to 1,412)	477 (372 to 585)	715 (551 to 877)	87.9 (77.3 to 99)	98.8 (83.1 to 116)	81.2 (68.2 to 94.7)
High income	Western Europe	Malta	YLDs	Age-standardized	154.2 (120.7 to 183)	106.9 (83.5 to 131)	217.8 (167.5 to 267.6)	139 (109.5 to 164.7)	105.6 (81.7 to 129)	180.4 (139.5 to 219.7)	-9.8 (-14.4 to -5.1)	-1.3 (-8.7 to 7.7)	-17.2 (-22.5 to -12)
High income	Western Europe	Monaco	Incidence	All Ages	147 (128 to 154)	69 (65 to 73)	78 (63 to 83)	194 (185 to 203)	96 (91 to 101)	98 (93 to 103)	32.3 (25.7 to 53.7)	39 (30.4 to 48.2)	26.3 (17.2 to 61.3)
High income	Western Europe	Monaco	Incidence	Age-standardized	227.6 (204 to 238.2)	175.4 (167.4 to 183.4)	287 (242.7 to 305.1)	222.6 (211.5 to 232.1)	200.1 (188.8 to 210.7)	246.5 (234.5 to 258.5)	-2.2 (-6.3 to 11.1)	14.1 (6.7 to 20.4)	-14.1 (-19.3 to 5)
High income	Western Europe	Monaco	Prevalence	All Ages	2,089 (1,888 to 2,201)	957 (909 to 1,006)	1,132 (961 to 1,209)	2,785 (2,627 to 2,928)	1,390 (1,300 to 1,475)	1,395 (1,316 to 1,476)	33.3 (27.1 to 51)	45.2 (35.5 to 54.3)	23.3 (15.1 to 51.9)
High income	Western Europe	Monaco	Prevalence	Age-standardized	3171.4 (2941 to 3338.3)	2502.1 (2359.6 to 2642.9)	4062.1 (3558.7 to 4318.8)	3094.6 (2928 to 3256.1)	2834.1 (2647.8 to 3023.1)	3399.8 (3213.9 to 3589.7)	-2.4 (-6.4 to 7.4)	13.3 (5.9 to 20.5)	-16.3 (-21.3 to -0.7)
High income	Western Europe	Monaco	Deaths	All Ages	13 (10 to 16)	5 (3 to 7)	8 (6 to 10)	16 (13 to 19)	7 (5 to 9)	9 (8 to 11)	30 (-1.5 to 64.9)	50.1 (-8.2 to 112.4)	18.2 (-6.6 to 51)
High income	Western Europe	Monaco	Deaths	Age-standardized	15 (12 to 19)	8.8 (6.2 to 13.6)	25.6 (20.8 to 30.7)	13.7 (11.1 to 16.2)	9.8 (7.3 to 12.4)	18.9 (15.5 to 22.6)	-8.7 (-30.8 to 17.1)	12.3 (-30.9 to 61.7)	-26.1 (-41.1 to -6.7)
High income	Western Europe	Monaco	DALYs	All Ages	299 (255 to 346)	117 (95 to 154)	181 (154 to 211)	371 (318 to 418)	165 (138 to 193)	205 (177 to 235)	24.1 (5 to 43.9)	40.9 (8.9 to 69.9)	13.2 (-3 to 34.1)
High income	Western Europe	Monaco	DALYs	Age-standardized	419.7 (358.8 to 486)	285.4 (233.4 to 362.9)	612.6 (523.5 to 710.2)	383.1 (329.6 to 434.7)	313.7 (261.1 to 367)	467.3 (400.8 to 535.4)	-8.7 (-22.1 to 5.3)	9.9 (-12.8 to 31.4)	-23.7 (-34.6 to -10.5)
High income	Western Europe	Monaco	YLLs	All Ages	174 (135 to 217)	59 (41 to 93)	115 (91 to 139)	205 (164 to 243)	81 (60 to 103)	124 (101 to 148)	18.2 (-10.9 to 54.8)	38.3 (-16 to 101.1)	7.9 (-15.9 to 39.9)
High income	Western Europe	Monaco	YLLs	Age-standardized	229.2 (179 to 288.1)	131.5 (93 to 203.2)	373.5 (298.8 to 451.6)	198.3 (158.9 to 237.1)	141 (103.1 to 180.6)	268.2 (217.7 to 322.7)	-13.5 (-35.3 to 14)	7.2 (-34.4 to 56.3)	-28.2 (-43.7 to -6.1)
High income	Western Europe	Monaco	YLDs	All Ages	125 (98 to 149)	59 (46 to 71)	67 (49 to 84)	165 (131 to 196)	84 (65 to 103)	81 (63 to 100)	32.2 (25.1 to 49.3)	43.5 (32.7 to 54)	22.3 (12.7 to 51.2)
High income	Western Europe	Monaco	YLDs	Age-standardized	190.5 (148.8 to 227.3)	153.9 (119.7 to 187.8)	239.2 (179.6 to 298)	184.8 (145.7 to 220)	172.8 (133.4 to 211.2)	199 (154.4 to 242.8)	-3 (-8.1 to 7.1)	12.3 (3.8 to 21.3)	-16.8 (-23.1 to -0.8)
High income	Western Europe	Netherlands	Incidence	All Ages	48,143 (46,829 to 49,491)	22,286 (21,120 to 23,507)	25,857 (25,358 to 26,259)	87,495 (84,686 to 90,414)	45,612 (43,677 to 47,552)	41,883 (40,298 to 43,325)	81.7 (75.7 to 88.5)	104.7 (93.7 to 116.5)	62 (55.8 to 67.9)
High income	Western Europe	Netherlands	Incidence	Age-standardized	250.9 (244.5 to 257.5)	203.4 (194.3 to 213.3)	308.3 (302.8 to 312.9)	277.2 (268.2 to 286.4)	275.9 (264.6 to 288)	279.1 (268.7 to 289.3)	10.5 (7.1 to 14.1)	35.6 (29.3 to 42.6)	-9.5 (-12.8 to -6.1)
High income	Western Europe	Netherlands	Prevalence	All Ages	678,338 (653,219 to 703,348)	332,124 (314,351 to 349,435)	346,214 (333,630 to 357,018)	1,271,027 (1,220,730 to 1,321,914)	705,364 (673,370 to 738,777)	565,663 (537,129 to 596,133)	87.4 (80.6 to 95.2)	112.4 (101.6 to 124.3)	63.4 (54.4 to 72.3)
High income	Western Europe	Netherlands	Prevalence	Age-standardized	3505.6 (3364.8 to 3638.5)	3023.2 (2862.1 to 3187.4)	4228.1 (4083.2 to 4354.6)	3863.8 (3695.2 to 4023.1)	3990.2 (3801.8 to 4172.3)	3742.9 (3546.1 to 3944.6)	10.2 (6.3 to 14.6)	32 (25.2 to 39.3)	-11.5 (-15.9 to -6.9)
High income	Western Europe	Netherlands	Deaths	All Ages	6,428 (5,893 to 6,875)	1,956 (1,669 to 2,310)	4,472 (4,134 to 4,789)	9,993 (8,160 to 11,442)	4,556 (2,735 to 5,625)	5,437 (4,680 to 6,297)	55.4 (30.1 to 77.8)	132.9 (45.1 to 193)	21.6 (4.1 to 42)
High income	Western Europe	Netherlands	Deaths	Age-standardized	30.9 (28.3 to 33.1)	15.1 (12.9 to 17.8)	59.9 (54.8 to 64.2)	26.4 (21.7 to 30.1)	20.9 (12.4 to 25.5)	35.4 (30.4 to 41)	-14.5 (-28.3 to -2.4)	38.6 (-13.7 to 73.4)	-40.9 (-49.2 to -31.2)
High income	Western Europe	Netherlands	DALYs	All Ages	143,192 (132,036 to 153,054)	49,171 (43,212 to 55,608)	94,021 (87,146 to 100,793)	223,466 (192,796 to 249,805)	107,955 (79,419 to 126,169)	115,511 (101,741 to 129,587)	56.1 (38.6 to 73.6)	119.6 (64 to 155.1)	22.9 (9.7 to 39.1)
High income	Western Europe	Netherlands	DALYs	Age-standardized	708.3 (652.8 to 758.3)	425 (374.7 to 479.7)	1160.2 (1073.1 to 1241.8)	645.8 (557.6 to 721.8)	584.2 (436.7 to 679.5)	739.5 (652.5 to 830.1)	-8.8 (-18.8 to 1)	37.4 (4.2 to 59.1)	-36.3 (-42.9 to -28)
High income	Western Europe	Netherlands	YLLs	All Ages	98,316 (91,185 to 104,533)	28,431 (24,988 to 33,334)	69,885 (65,232 to 74,598)	137,748 (113,509 to 155,415)	62,691 (36,410 to 75,231)	75,057 (64,893 to 86,583)	40.1 (17.4 to 59.8)	120.5 (35.7 to 171.9)	7.4 (-7.7 to 25.1)
High income	Western Europe	Netherlands	YLLs	Age-standardized	476.4 (444.6 to 506)	235.7 (208.6 to 276.5)	865.9 (806.2 to 924.9)	385.1 (319.9 to 434.4)	327 (189 to 389.4)	472.4 (408.9 to 543.8)	-19.2 (-32.3 to -8)	38.8 (-14.4 to 69.6)	-45.4 (-53.1 to -36.6)
High income	Western Europe	Netherlands	YLDs	All Ages	44,876 (36,099 to 52,460)	20,740 (16,254 to 25,493)	24,136 (19,091 to 29,244)	85,718 (67,912 to 104,371)	45,264 (34,447 to 57,221)	40,454 (31,050 to 49,399)	91 (68.9 to 125.6)	118.2 (83.1 to 162.4)	67.6 (46.4 to 99.2)
High income	Western Europe	Netherlands	YLDs	Age-standardized	232 (187.5 to 270.5)	189.4 (149.5 to 232.3)	294.3 (232.7 to 356.1)	260.8 (206.4 to 315.7)	257.2 (197.1 to 321.5)	267.1 (206.4 to 324.5)	12.4 (0 to 30.9)	35.8 (14.3 to 61.8)	-9.3 (-20.6 to 6.4)
High income	Western Europe	Norway	Incidence	All Ages	12,456 (11,171 to 13,809)	5,808 (5,134 to 6,521)	6,648 (5,988 to 7,279)	23,455 (21,723 to 24,941)	12,382 (11,494 to 13,118)	11,073 (10,232 to 11,801)	88.3 (77.7 to 99)	113.2 (94.8 to 130.4)	66.6 (59.7 to 73.9)
High income	Western Europe	Norway	Incidence	Age-standardized	200.6 (183 to 218.4)	172.1 (156.2 to 189.7)	237 (216.8 to 257.3)	262.3 (243.2 to 279.1)	264.9 (245.9 to 282.2)	260.3 (241.3 to 276.7)	30.8 (25.1 to 36.5)	54 (44.5 to 63.1)	9.8 (6.1 to 13.9)
High income	Western Europe	Norway	Prevalence	All Ages	191,196 (174,596 to 208,571)	97,468 (88,602 to 107,176)	93,728 (85,317 to 102,120)	346,210 (320,635 to 371,915)	191,114 (177,058 to 204,836)	155,097 (142,850 to 167,141)	81.1 (73.9 to 88.3)	96.1 (85.1 to 107.2)	65.5 (60.2 to 71.2)
High income	Western Europe	Norway	Prevalence	Age-standardized	2961.5 (2728.1 to 3219.3)	2720.4 (2495.2 to 2965.5)	3345.5 (3072 to 3622.8)	3705.2 (3434.5 to 3982.7)	3796.4 (3529.7 to 4072.3)	3612.8 (3336.6 to 3890.8)	25.1 (21 to 29.4)	39.6 (33.2 to 46.5)	8 (4.9 to 11.2)
High income	Western Europe	Norway	Deaths	All Ages	1,064 (984 to 1,245)	391 (338 to 575)	674 (633 to 704)	2,477 (1,910 to 2,716)	1,195 (622 to 1,367)	1,282 (1,155 to 1,396)	132.7 (69.9 to 157.5)	205.8 (27.4 to 263.3)	90.3 (74.3 to 108.4)
High income	Western Europe	Norway	Deaths	Age-standardized	14.1 (13 to 16.3)	8.6 (7.6 to 12.1)	22.7 (21.3 to 23.8)	23 (17.7 to 25.2)	19.5 (9.6 to 22.3)	28.1 (25.2 to 30.6)	63.5 (18.6 to 80.8)	125.8 (-8 to 166.7)	23.5 (13.3 to 35.2)
High income	Western Europe	Norway	DALYs	All Ages	27,158 (24,645 to 29,651)	10,996 (9,534 to 12,892)	16,162 (14,804 to 17,468)	53,398 (43,501 to 58,789)	26,116 (17,174 to 29,931)	27,282 (24,612 to 29,740)	96.6 (63.5 to 110.2)	137.5 (48.1 to 164.8)	68.8 (59.5 to 80.6)
High income	Western Europe	Norway	DALYs	Age-standardized	396.2 (358.5 to 433.6)	290.6 (252.1 to 335.2)	548.4 (500.5 to 593.4)	548.6 (447.8 to 605.3)	501.7 (330.8 to 576.4)	610.8 (551.2 to 665.9)	38.5 (16.3 to 47.5)	72.7 (11.6 to 91.2)	11.4 (5.3 to 19.1)
High income	Western Europe	Norway	YLLs	All Ages	16,925 (15,969 to 18,674)	6,000 (5,402 to 7,729)	10,925 (10,369 to 11,391)	35,130 (26,773 to 38,283)	16,511 (7,885 to 18,858)	18,620 (17,106 to 20,220)	107.6 (56.1 to 128.3)	175.2 (17 to 220.6)	70.4 (56.8 to 87.1)
High income	Western Europe	Norway	YLLs	Age-standardized	235.8 (222.7 to 257.8)	147.9 (134.1 to 181.3)	361 (342.1 to 376.8)	350.3 (267.7 to 380.9)	306.3 (142.3 to 348.9)	408.2 (374.8 to 443.9)	48.6 (12.5 to 63.2)	107.1 (-10.1 to 139.2)	13.1 (4 to 24.2)
High income	Western Europe	Norway	YLDs	All Ages	10,233 (7,971 to 12,411)	4,996 (3,754 to 6,285)	5,237 (4,015 to 6,408)	18,268 (14,169 to 22,276)	9,605 (7,071 to 12,063)	8,663 (6,568 to 10,658)	78.5 (71.2 to 86.1)	92.3 (81.5 to 103.7)	65.4 (59.8 to 71.3)
High income	Western Europe	Norway	YLDs	Age-standardized	160.4 (124.6 to 194.2)	142.7 (108.4 to 179.7)	187.4 (143.4 to 228)	198.4 (152.8 to 241.3)	195.5 (146.5 to 245.2)	202.6 (154.9 to 247.6)	23.7 (19.2 to 28.4)	37 (29.7 to 44.4)	8.1 (4.7 to 11.6)
High income	Western Europe	Portugal	Incidence	All Ages	28,941 (27,780 to 30,154)	13,110 (12,406 to 13,828)	15,831 (15,073 to 16,566)	49,338 (47,663 to 51,102)	25,160 (24,188 to 26,201)	24,179 (23,160 to 25,199)	70.5 (64.6 to 77.1)	91.9 (82.9 to 103.1)	52.7 (45.6 to 59.3)
High income	Western Europe	Portugal	Incidence	Age-standardized	222 (214.6 to 230.1)	173.2 (165.5 to 181.6)	277.6 (266.7 to 288.1)	209.1 (201.4 to 217.7)	171.9 (164.3 to 181.2)	249.8 (239.6 to 260.6)	-5.8 (-8.9 to -2.5)	-0.8 (-5.2 to 4.2)	-10 (-13.6 to -6.3)
High income	Western Europe	Portugal	Prevalence	All Ages	387,425 (372,544 to 403,279)	166,892 (158,615 to 176,706)	220,533 (210,965 to 230,133)	630,134 (601,544 to 663,586)	297,807 (281,031 to 316,694)	332,328 (314,077 to 350,720)	62.6 (56.8 to 69.5)	78.4 (69.3 to 89.1)	50.7 (43.1 to 58.4)
High income	Western Europe	Portugal	Prevalence	Age-standardized	2994.6 (2885.3 to 3109.9)	2300.3 (2188.7 to 2426)	3912.4 (3755.6 to 4067.3)	2693 (2558.5 to 2839.4)	2118.6 (1985.1 to 2263.8)	3396.4 (3212 to 3586.4)	-10.1 (-13.6 to -6.3)	-7.9 (-12.7 to -2.4)	-13.2 (-17.5 to -8.7)
High income	Western Europe	Portugal	Deaths	All Ages	3,612 (3,336 to 4,032)	1,346 (1,151 to 1,662)	2,267 (2,085 to 2,438)	6,191 (5,221 to 7,102)	2,759 (2,012 to 3,396)	3,432 (2,886 to 4,019)	71.4 (47.3 to 99)	105 (55.1 to 160.6)	51.4 (27.9 to 79.6)
High income	Western Europe	Portugal	Deaths	Age-standardized	28.4 (25.9 to 31.3)	17.6 (14.9 to 21.1)	46.6 (42.5 to 50.5)	20.4 (17.5 to 23.4)	13.9 (10.6 to 17.2)	30.6 (25.9 to 35.6)	-28 (-37.4 to -17.5)	-20.8 (-36 to -1.8)	-34.4 (-44.4 to -22.3)
High income	Western Europe	Portugal	DALYs	All Ages	84,498 (77,184 to 92,262)	30,811 (27,269 to 36,596)	53,687 (49,035 to 58,000)	109,804 (97,283 to 123,301)	47,523 (40,143 to 55,583)	62,281 (54,841 to 70,893)	29.9 (17.4 to 43.3)	54.2 (32.8 to 78.5)	16 (2.4 to 31.9)
High income	Western Europe	Portugal	DALYs	Age-standardized	634.1 (579.1 to 687.9)	404.9 (358.7 to 473.9)	960 (880.1 to 1036.2)	428.8 (381.1 to 483.7)	304.1 (260.4 to 364)	596.1 (528.6 to 675.1)	-32.4 (-38.5 to -25.6)	-24.9 (-33.7 to -14.4)	-37.9 (-44.8 to -29.9)
High income	Western Europe	Portugal	YLLs	All Ages	59,616 (55,309 to 66,657)	19,501 (17,107 to 25,321)	40,116 (37,232 to 43,077)	72,411 (62,359 to 83,524)	28,558 (22,319 to 35,481)	43,853 (37,335 to 50,760)	21.5 (5.5 to 40.1)	46.4 (18.5 to 81.8)	9.3 (-7.3 to 28.5)
High income	Western Europe	Portugal	YLLs	Age-standardized	442 (408.9 to 489.7)	249 (219.8 to 314.1)	719.9 (667.3 to 774.1)	268.5 (232.7 to 313)	167.7 (133.2 to 229.4)	407.2 (350.8 to 470.3)	-39.2 (-47.2 to -30.2)	-32.6 (-44.9 to -17.5)	-43.4 (-51.6 to -34.1)
High income	Western Europe	Portugal	YLDs	All Ages	24,882 (19,646 to 29,541)	11,310 (8,864 to 13,713)	13,572 (10,448 to 16,506)	37,393 (29,543 to 44,655)	18,965 (14,935 to 22,943)	18,428 (13,886 to 22,732)	50.3 (31.7 to 69)	67.7 (44.3 to 94.2)	35.8 (14.6 to 58.8)
High income	Western Europe	Portugal	YLDs	Age-standardized	192.2 (151.3 to 227.6)	155.9 (121.5 to 188.2)	240.1 (185.9 to 290.7)	160.3 (125.9 to 192.9)	136.4 (107.3 to 165)	188.9 (143.3 to 232.9)	-16.6 (-26.1 to -7)	-12.5 (-24 to 1.1)	-21.3 (-32.6 to -8.4)
High income	Western Europe	San Marino	Incidence	All Ages	73 (68 to 76)	30 (28 to 32)	43 (39 to 45)	138 (131 to 142)	68 (65 to 71)	70 (66 to 73)	88.4 (80.7 to 103.6)	127.3 (116.9 to 140.2)	61.4 (53 to 86.1)
High income	Western Europe	San Marino	Incidence	Age-standardized	233.1 (219.5 to 241.8)	174.7 (166.4 to 183.3)	294.8 (268.4 to 305.6)	226 (215.3 to 233.6)	201 (191.7 to 209.7)	250.4 (236.9 to 261.6)	-3 (-7.2 to 4.4)	15 (10.6 to 21)	-15.1 (-19.4 to -4.1)
High income	Western Europe	San Marino	Prevalence	All Ages	1,021 (961 to 1,066)	421 (397 to 444)	600 (548 to 629)	1,954 (1,854 to 2,036)	969 (920 to 1,015)	985 (926 to 1,040)	91.4 (82.3 to 105.3)	130.3 (119.7 to 145.6)	64.1 (53.7 to 85.3)
High income	Western Europe	San Marino	Prevalence	Age-standardized	3226.4 (3053.4 to 3367.9)	2474.4 (2329.4 to 2612.3)	4151 (3814 to 4339.9)	3132.8 (2979.2 to 3275)	2845.3 (2688.7 to 2995.2)	3454.1 (3254.7 to 3652.6)	-2.9 (-7.3 to 3.6)	15 (9.5 to 21.7)	-16.8 (-21.9 to -6.8)
High income	Western Europe	San Marino	Deaths	All Ages	4 (4 to 5)	2 (1 to 3)	3 (2 to 3)	9 (6 to 12)	4 (3 to 6)	5 (3 to 6)	106.3 (44.5 to 189.7)	130.4 (54.9 to 239.1)	88.3 (25.5 to 166.7)
High income	Western Europe	San Marino	Deaths	Age-standardized	13.4 (11.1 to 16.2)	9.7 (7.6 to 12.9)	18.9 (15.6 to 22.9)	11.3 (7.8 to 15.5)	8.9 (5.8 to 13)	14.3 (9.9 to 19.3)	-15.4 (-41.4 to 19.4)	-7.6 (-38.9 to 40.1)	-24.2 (-49.3 to 6.7)
High income	Western Europe	San Marino	DALYs	All Ages	127 (110 to 144)	51 (43 to 62)	75 (65 to 87)	229 (183 to 280)	108 (84 to 134)	122 (96 to 150)	81.2 (49.9 to 121.7)	109.1 (72.7 to 159)	62 (29.5 to 102)
High income	Western Europe	San Marino	DALYs	Age-standardized	387.3 (338.6 to 438.6)	285.4 (240.5 to 338.8)	517.3 (446.7 to 597.6)	349 (279.2 to 425)	296.3 (232.8 to 371.6)	408.7 (321 to 501.1)	-9.9 (-25.1 to 10)	3.8 (-14 to 27.9)	-21 (-36.1 to -2.2)
High income	Western Europe	San Marino	YLLs	All Ages	65 (54 to 79)	26 (20 to 34)	40 (32 to 48)	113 (77 to 157)	49 (32 to 72)	64 (43 to 89)	72.9 (16.1 to 151.7)	90.6 (24.9 to 194)	61.5 (4.8 to 135.4)
High income	Western Europe	San Marino	YLLs	Age-standardized	194 (161.4 to 232.8)	133.5 (105.1 to 177.1)	273.3 (225 to 327.2)	161.8 (108.2 to 229.4)	122.6 (78.5 to 185.6)	206.6 (137.8 to 290.6)	-16.6 (-45.1 to 23.5)	-8.2 (-41.7 to 45.5)	-24.4 (-51.2 to 10.6)
High income	Western Europe	San Marino	YLDs	All Ages	61 (48 to 73)	26 (20 to 31)	35 (27 to 44)	116 (92 to 137)	59 (46 to 71)	57 (44 to 71)	90 (79.7 to 104.5)	127.6 (113.9 to 143.4)	62.6 (50.4 to 85)
High income	Western Europe	San Marino	YLDs	Age-standardized	193.3 (151.6 to 230)	151.9 (117.4 to 185.1)	244.1 (188.1 to 301.7)	187.2 (147 to 222.4)	173.8 (134.7 to 210.9)	202.1 (156.3 to 247.4)	-3.1 (-8.4 to 3.5)	14.4 (7.1 to 22.1)	-17.2 (-23.2 to -7.1)
High income	Western Europe	Spain	Incidence	All Ages	127,271 (123,941 to 130,486)	56,247 (53,300 to 59,040)	71,024 (69,806 to 71,963)	214,293 (208,811 to 219,868)	99,099 (95,780 to 102,971)	115,195 (112,077 to 118,002)	68.4 (64.1 to 72.7)	76.2 (68.5 to 84.7)	62.2 (57.7 to 66)
High income	Western Europe	Spain	Incidence	Age-standardized	242.9 (237 to 248.5)	180 (171 to 188.9)	311.1 (306.2 to 315.1)	233.1 (225.7 to 240.9)	174.5 (166.7 to 183.8)	293.4 (284.1 to 301.6)	-4.1 (-6.9 to -1.4)	-3.1 (-7.4 to 1.7)	-5.7 (-8.7 to -3.1)
High income	Western Europe	Spain	Prevalence	All Ages	1,656,213 (1,603,124 to 1,705,537)	677,800 (637,028 to 720,110)	978,413 (956,609 to 996,263)	2,905,818 (2,788,360 to 3,034,266)	1,257,615 (1,193,809 to 1,335,066)	1,648,203 (1,575,482 to 1,714,776)	75.4 (69.3 to 81.3)	85.5 (75.9 to 95.5)	68.5 (61.1 to 75.1)
High income	Western Europe	Spain	Prevalence	Age-standardized	3191 (3088.2 to 3282.3)	2290.4 (2154.5 to 2423.3)	4350.1 (4251.6 to 4429.2)	3081.8 (2935.3 to 3235.7)	2263.1 (2130.7 to 2412.9)	4051.1 (3855 to 4231.3)	-3.4 (-6.9 to -0.1)	-1.2 (-6.1 to 3.8)	-6.9 (-11.1 to -2.9)
High income	Western Europe	Spain	Deaths	All Ages	18,514 (16,816 to 19,749)	5,869 (4,512 to 6,767)	12,645 (11,653 to 13,522)	31,245 (25,155 to 36,629)	11,653 (7,326 to 15,404)	19,593 (16,386 to 22,484)	68.8 (44 to 96.7)	98.5 (55.5 to 155.7)	54.9 (32.3 to 80.8)
High income	Western Europe	Spain	Deaths	Age-standardized	34.5 (31.1 to 37)	17.8 (13.6 to 20.5)	61.7 (56.3 to 66.2)	24.4 (20.4 to 28.2)	13.4 (9.1 to 17.3)	41.6 (34.9 to 47.6)	-29.2 (-38.1 to -18.6)	-24.6 (-39 to -5.1)	-32.6 (-42 to -21.5)
High income	Western Europe	Spain	DALYs	All Ages	373,115 (345,015 to 399,010)	112,583 (96,818 to 125,845)	260,533 (242,013 to 278,951)	507,703 (444,859 to 567,845)	178,453 (139,434 to 212,656)	329,251 (289,401 to 369,544)	36.1 (23.7 to 50.5)	58.5 (38.5 to 85.9)	26.4 (13.4 to 40.1)
High income	Western Europe	Spain	DALYs	Age-standardized	686.7 (633.2 to 735.6)	354.5 (305.2 to 398.2)	1158 (1076 to 1240.2)	482.6 (427.1 to 536.1)	277.5 (230.7 to 320.7)	754.9 (668 to 844.9)	-29.7 (-35.1 to -23.2)	-21.7 (-30.3 to -11.5)	-34.8 (-41 to -27.8)
High income	Western Europe	Spain	YLLs	All Ages	281,191 (260,824 to 298,374)	75,926 (62,376 to 86,039)	205,265 (191,336 to 219,340)	348,086 (294,884 to 395,506)	111,470 (77,522 to 141,957)	236,616 (204,628 to 267,997)	23.8 (7.1 to 41.2)	46.8 (18.7 to 84.8)	15.3 (0.2 to 32.1)
High income	Western Europe	Spain	YLLs	Age-standardized	509.6 (472.2 to 540.8)	230 (190.9 to 260.3)	912.7 (848.9 to 977.7)	311.8 (271.2 to 351)	154.9 (119.1 to 191.3)	526.8 (458.5 to 596.5)	-38.8 (-45.5 to -30.8)	-32.6 (-44.8 to -17.2)	-42.3 (-49.4 to -34)
High income	Western Europe	Spain	YLDs	All Ages	91,924 (72,083 to 108,994)	36,657 (27,417 to 45,138)	55,268 (42,257 to 67,763)	159,618 (125,142 to 189,825)	66,983 (49,478 to 82,287)	92,635 (70,180 to 114,305)	73.6 (65.5 to 82)	82.7 (68.9 to 95.9)	67.6 (57.4 to 77.2)
High income	Western Europe	Spain	YLDs	Age-standardized	177.1 (139.1 to 209.8)	124.5 (93.7 to 153.3)	245.3 (187.5 to 299.5)	170.7 (134.6 to 203.1)	122.5 (93.2 to 149.4)	228.1 (173.9 to 281.4)	-3.6 (-8.3 to 0.7)	-1.6 (-8.5 to 5.7)	-7 (-12.5 to -1.9)
High income	Western Europe	Sweden	Incidence	All Ages	26,563 (23,595 to 29,426)	12,024 (10,563 to 13,650)	14,538 (12,916 to 16,094)	45,502 (41,269 to 49,426)	24,572 (22,315 to 26,571)	20,930 (18,874 to 22,957)	71.3 (62.3 to 81)	104.4 (84.2 to 124.1)	44 (37.5 to 52)
High income	Western Europe	Sweden	Incidence	Age-standardized	194.6 (175.7 to 213.3)	163.7 (148 to 180.6)	233.7 (210.1 to 255.6)	232.8 (212.9 to 253)	240.6 (219.9 to 261.5)	225.6 (205.1 to 246.7)	19.6 (14 to 25.1)	47 (36 to 57.8)	-3.5 (-7.5 to 1.4)
High income	Western Europe	Sweden	Prevalence	All Ages	409,237 (371,381 to 450,841)	202,128 (182,244 to 222,956)	207,109 (186,403 to 229,154)	657,306 (598,730 to 721,926)	370,760 (338,245 to 405,542)	286,546 (259,145 to 316,863)	60.6 (53.5 to 68.6)	83.4 (70.4 to 95.6)	38.4 (31.7 to 45.9)
High income	Western Europe	Sweden	Prevalence	Age-standardized	2916.1 (2663.8 to 3175.3)	2630.4 (2399.8 to 2871.8)	3344.4 (3046.4 to 3659.1)	3216.7 (2956.6 to 3508.4)	3370.7 (3088 to 3679.4)	3055 (2795.3 to 3352.7)	10.3 (5.6 to 15.3)	28.1 (20 to 36)	-8.7 (-12.9 to -3.9)
High income	Western Europe	Sweden	Deaths	All Ages	1,833 (1,664 to 2,181)	701 (598 to 1,038)	1,132 (1,040 to 1,202)	3,789 (2,955 to 4,324)	2,017 (1,153 to 2,429)	1,772 (1,520 to 2,035)	106.7 (50.7 to 140.9)	187.7 (29.9 to 263.6)	56.6 (33.9 to 82.5)
High income	Western Europe	Sweden	Deaths	Age-standardized	10.9 (9.9 to 12.9)	7.1 (6.2 to 10)	17.3 (15.9 to 18.4)	14.9 (11.6 to 17)	14.1 (7.8 to 16.9)	16.3 (14 to 18.7)	36.9 (0.2 to 58.6)	99.2 (-9.4 to 147.8)	-5.5 (-18.7 to 9.3)
High income	Western Europe	Sweden	DALYs	All Ages	53,208 (47,467 to 59,276)	24,823 (21,460 to 28,910)	28,385 (25,312 to 31,586)	90,488 (77,220 to 100,835)	51,831 (38,789 to 59,454)	38,656 (33,601 to 43,655)	70.1 (45.8 to 84.7)	108.8 (48 to 134.6)	36.2 (23.4 to 51.1)
High income	Western Europe	Sweden	DALYs	Age-standardized	358.5 (316.7 to 398.2)	306.8 (264.1 to 356.9)	436.8 (387.3 to 486.1)	418.4 (359.1 to 467.8)	451 (344.1 to 518.7)	386.9 (336.5 to 439.3)	16.7 (1.6 to 25.8)	46.1 (9.6 to 67)	-11.4 (-19.1 to -2.2)
High income	Western Europe	Sweden	YLLs	All Ages	27,918 (25,919 to 31,509)	10,457 (9,210 to 13,871)	17,461 (16,177 to 18,586)	49,674 (38,273 to 56,036)	26,014 (14,000 to 31,003)	23,659 (20,507 to 27,257)	77.9 (33.2 to 103.7)	148.8 (16.8 to 207.1)	35.5 (15.9 to 58.2)
High income	Western Europe	Sweden	YLLs	Age-standardized	176.1 (164.2 to 195.7)	118.3 (105.2 to 147.3)	259.6 (241.6 to 276.2)	216 (167.3 to 242.7)	211 (112.8 to 249.5)	225.5 (195.9 to 259.4)	22.7 (-6.4 to 39.7)	78.4 (-12.3 to 118)	-13.1 (-25.3 to 1)
High income	Western Europe	Sweden	YLDs	All Ages	25,290 (19,725 to 30,608)	14,366 (11,308 to 17,461)	10,924 (8,061 to 13,804)	40,814 (31,941 to 49,171)	25,817 (20,282 to 31,520)	14,997 (11,050 to 18,812)	61.4 (52.7 to 70.8)	79.7 (65.8 to 93.6)	37.3 (28.9 to 46.3)
High income	Western Europe	Sweden	YLDs	Age-standardized	182.4 (143.1 to 219.4)	190.4 (150.3 to 232.4)	177.2 (131.7 to 222.8)	202.4 (158.5 to 243.7)	240.1 (190.3 to 291.5)	161.5 (119.1 to 200.6)	11 (5 to 16.9)	26.1 (16.9 to 35.4)	-8.9 (-14.3 to -3)
High income	Western Europe	Switzerland	Incidence	All Ages	20,287 (19,334 to 21,283)	8,507 (7,954 to 9,095)	11,780 (11,238 to 12,319)	31,087 (29,610 to 32,594)	15,735 (14,850 to 16,638)	15,352 (14,566 to 16,201)	53.2 (47.4 to 59.1)	85 (74.7 to 95.4)	30.3 (23.9 to 37.3)
High income	Western Europe	Switzerland	Incidence	Age-standardized	205.3 (195.9 to 214.5)	144.2 (136.3 to 152.7)	278.3 (265.8 to 290.5)	186.6 (177.9 to 195)	169.6 (160.2 to 179.3)	205.2 (195.3 to 215.7)	-9.1 (-12.5 to -5.8)	17.6 (12.2 to 23.3)	-26.3 (-29.6 to -22.7)
High income	Western Europe	Switzerland	Prevalence	All Ages	286,322 (272,611 to 300,657)	121,015 (113,741 to 128,675)	165,307 (156,595 to 173,750)	427,179 (405,500 to 450,249)	221,908 (209,090 to 235,709)	205,271 (194,428 to 217,400)	49.2 (43.6 to 55.4)	83.4 (74.6 to 92.6)	24.2 (17.6 to 31.4)
High income	Western Europe	Switzerland	Prevalence	Age-standardized	2872.8 (2727.8 to 3017.4)	2135.2 (2006.2 to 2281)	3924.8 (3717.4 to 4119.6)	2553.1 (2424 to 2699.1)	2413.7 (2271.7 to 2575.2)	2736.6 (2591.3 to 2900.7)	-11.1 (-14.6 to -7.8)	13 (7.8 to 18.4)	-30.3 (-33.9 to -26.2)
High income	Western Europe	Switzerland	Deaths	All Ages	2,049 (1,853 to 2,220)	650 (539 to 756)	1,399 (1,268 to 1,521)	2,632 (2,200 to 3,012)	1,145 (842 to 1,390)	1,488 (1,245 to 1,751)	28.5 (11.7 to 47.8)	76.1 (43.6 to 117)	6.3 (-9.8 to 26.1)
High income	Western Europe	Switzerland	Deaths	Age-standardized	18.3 (16.5 to 19.8)	9.2 (7.7 to 10.6)	33.9 (30.7 to 37.1)	12.7 (10.7 to 14.4)	9.3 (6.7 to 11.2)	17.6 (14.9 to 20.7)	-30.8 (-39 to -21.1)	1.5 (-17.7 to 23.1)	-48 (-55.8 to -38.6)
High income	Western Europe	Switzerland	DALYs	All Ages	48,929 (44,614 to 52,969)	16,581 (14,458 to 18,598)	32,348 (29,343 to 35,225)	60,128 (53,078 to 66,472)	27,830 (23,007 to 32,179)	32,298 (28,664 to 36,436)	22.9 (13.4 to 33.8)	67.8 (49.5 to 86.8)	-0.2 (-9.9 to 12.1)
High income	Western Europe	Switzerland	DALYs	Age-standardized	469.1 (426.2 to 508.9)	274.6 (237.9 to 309.3)	755.8 (684.7 to 823.2)	340 (300.6 to 377.5)	287.7 (236.2 to 332.4)	408.7 (362.5 to 461.3)	-27.5 (-33 to -21.7)	4.7 (-5.9 to 15.9)	-45.9 (-50.9 to -39.6)
High income	Western Europe	Switzerland	YLLs	All Ages	31,993 (29,459 to 34,495)	9,251 (7,825 to 10,550)	22,742 (20,775 to 24,638)	34,759 (29,800 to 39,108)	14,465 (10,291 to 17,256)	20,294 (17,436 to 23,423)	8.6 (-3.9 to 23.3)	56.4 (26.5 to 89.6)	-10.8 (-23.4 to 5.4)
High income	Western Europe	Switzerland	YLLs	Age-standardized	298.8 (274.4 to 322.3)	144.5 (119.8 to 164.4)	527.9 (483.2 to 572.1)	187.4 (162.5 to 210.4)	140.6 (99 to 167)	248.4 (213.6 to 286.9)	-37.3 (-44.6 to -29)	-2.7 (-21.9 to 17)	-53 (-59.4 to -44.4)
High income	Western Europe	Switzerland	YLDs	All Ages	16,935 (13,229 to 20,024)	7,329 (5,650 to 8,924)	9,606 (7,361 to 11,686)	25,369 (19,949 to 30,042)	13,365 (10,387 to 16,440)	12,004 (9,268 to 14,767)	49.8 (42.3 to 58.5)	82.4 (70.8 to 94.7)	25 (16.7 to 34.2)
High income	Western Europe	Switzerland	YLDs	Age-standardized	170.3 (133 to 201.5)	130.1 (100.6 to 157.9)	227.9 (174.8 to 276.5)	152.6 (119.6 to 181)	147 (114.3 to 179.4)	160.3 (124.3 to 197.2)	-10.4 (-14.9 to -5.4)	13 (5.2 to 21.4)	-29.7 (-34.2 to -24.6)
High income	Western Europe	United Kingdom	Incidence	All Ages	215,250 (199,732 to 229,445)	110,029 (99,069 to 121,037)	105,221 (99,497 to 109,526)	314,108 (295,491 to 329,469)	166,202 (156,302 to 174,478)	147,906 (139,164 to 155,244)	45.9 (41.4 to 50)	51.1 (42.1 to 60.1)	40.6 (37.7 to 43.1)
High income	Western Europe	United Kingdom	Incidence	Age-standardized	256.5 (240.3 to 271.5)	233.7 (213.5 to 252.8)	287.2 (273.5 to 298.1)	273.8 (258.2 to 287.8)	271.9 (256.1 to 286.2)	276.1 (260.4 to 289.8)	6.7 (4.2 to 8.9)	16.4 (11.4 to 21.4)	-3.9 (-5.7 to -2.1)
High income	Western Europe	United Kingdom	Prevalence	All Ages	3,206,010 (2,992,311 to 3,407,355)	1,759,366 (1,613,687 to 1,903,991)	1,446,644 (1,370,786 to 1,516,110)	4,727,607 (4,443,749 to 5,006,429)	2,570,226 (2,415,150 to 2,719,711)	2,157,382 (2,027,940 to 2,284,002)	47.5 (44.3 to 50.5)	46.1 (40.5 to 52)	49.1 (46.1 to 52.3)
High income	Western Europe	United Kingdom	Prevalence	Age-standardized	3701.6 (3477 to 3923.3)	3513.5 (3267.3 to 3765.5)	4018.2 (3820.2 to 4203.1)	3902.1 (3678.5 to 4125.7)	3900.3 (3675.1 to 4121)	3903.5 (3678.7 to 4129.3)	5.4 (3.4 to 7.2)	11 (7.8 to 14.1)	-2.9 (-4.7 to -1)
High income	Western Europe	United Kingdom	Deaths	All Ages	32,896 (30,924 to 37,005)	12,562 (11,193 to 16,696)	20,334 (19,369 to 20,873)	43,945 (37,736 to 49,042)	21,580 (15,087 to 24,642)	22,364 (19,824 to 24,992)	33.6 (16.2 to 49.8)	71.8 (11 to 100.1)	10 (-0.6 to 23.8)
High income	Western Europe	United Kingdom	Deaths	Age-standardized	34.1 (31.9 to 38.5)	21.3 (19.3 to 27.8)	58.6 (55.3 to 60.3)	30.7 (26.6 to 34.1)	26.3 (18.3 to 29.8)	36.9 (32.7 to 41.2)	-10.1 (-22 to 0.3)	23.1 (-19.2 to 41.8)	-37 (-42.6 to -29.4)
High income	Western Europe	United Kingdom	DALYs	All Ages	726,174 (679,846 to 776,060)	297,248 (267,649 to 343,136)	428,926 (405,535 to 449,917)	891,037 (803,053 to 974,746)	432,909 (336,707 to 481,176)	458,128 (414,594 to 505,451)	22.7 (13.3 to 31.9)	45.6 (13 to 59.4)	6.8 (-0.4 to 16.2)
High income	Western Europe	United Kingdom	DALYs	Age-standardized	788.5 (737.4 to 842)	565.7 (510 to 635.2)	1149.1 (1087.6 to 1206.4)	696.1 (628 to 760)	622.3 (492.9 to 690.5)	788.4 (716 to 871)	-11.7 (-18.1 to -5.6)	10 (-11.8 to 18.8)	-31.4 (-35.8 to -25.6)
High income	Western Europe	United Kingdom	YLLs	All Ages	534,638 (508,173 to 576,770)	198,154 (182,453 to 245,067)	336,485 (321,818 to 344,307)	612,393 (535,112 to 676,697)	290,211 (200,762 to 325,904)	322,182 (291,794 to 358,158)	14.5 (2.8 to 26.7)	46.5 (0.2 to 66.8)	-4.3 (-12.5 to 7.3)
High income	Western Europe	United Kingdom	YLLs	Age-standardized	566 (538.5 to 609.9)	365.1 (336.5 to 432.1)	892.5 (853.8 to 914.6)	463.4 (408.3 to 510)	402 (276.2 to 448.4)	541.2 (491.1 to 601.8)	-18.1 (-26.2 to -9.7)	10.1 (-22.1 to 23.5)	-39.4 (-44.4 to -32.2)
High income	Western Europe	United Kingdom	YLDs	All Ages	191,536 (152,676 to 225,711)	99,095 (75,855 to 121,869)	92,442 (73,937 to 110,866)	278,643 (223,620 to 327,292)	142,698 (110,208 to 174,022)	135,946 (108,168 to 163,605)	45.5 (42.5 to 48.8)	44 (38.5 to 49.8)	47.1 (43.2 to 51.2)
High income	Western Europe	United Kingdom	YLDs	Age-standardized	222.5 (176.8 to 262.8)	200.5 (154 to 246.3)	256.6 (204.9 to 306.6)	232.7 (186.3 to 275.1)	220.3 (169.7 to 269.6)	247.2 (198.5 to 297)	4.6 (2.6 to 6.4)	9.9 (6.7 to 13.1)	-3.7 (-5.7 to -1.7)
		Southern Latin America	Incidence	All Ages	68,645 (65,631 to 71,703)	26,330 (24,980 to 27,697)	42,316 (40,447 to 44,243)	161,662 (154,756 to 169,069)	74,890 (71,326 to 78,924)	86,771 (82,964 to 90,677)	135.5 (129.3 to 141.7)	184.4 (174.1 to 196.4)	105.1 (97.6 to 111.7)
		Southern Latin America	Incidence	Age-standardized	151.5 (145.1 to 157.8)	107 (101.7 to 112.1)	205.6 (196.7 to 214.3)	199.2 (190.9 to 207.9)	163.3 (155.8 to 171.7)	242.2 (232.3 to 252.6)	31.5 (28.3 to 34.7)	52.6 (47.5 to 58.6)	17.8 (13.8 to 21.5)
		Southern Latin America	Prevalence	All Ages	1,047,057 (1,005,814 to 1,090,801)	430,535 (408,767 to 452,048)	616,522 (591,586 to 641,695)	2,234,237 (2,134,786 to 2,344,295)	1,072,190 (1,018,670 to 1,130,600)	1,162,047 (1,110,740 to 1,213,634)	113.4 (107.5 to 119.1)	149 (139.6 to 159)	88.5 (81.5 to 95)
		Southern Latin America	Prevalence	Age-standardized	2288.8 (2199.4 to 2384.5)	1727.4 (1640.9 to 1813.4)	3014.4 (2889.5 to 3140.1)	2741.7 (2619.3 to 2875)	2357.4 (2238.3 to 2491.5)	3264.1 (3122.4 to 3406.5)	19.8 (16.6 to 22.9)	36.5 (31.7 to 41.7)	8.3 (4.6 to 11.9)
		Southern Latin America	Deaths	All Ages	10,905 (10,056 to 12,006)	3,714 (3,192 to 4,715)	7,191 (6,712 to 7,709)	22,686 (19,735 to 25,346)	9,984 (7,594 to 11,765)	12,702 (11,215 to 14,248)	108 (83 to 135)	168.9 (96.4 to 225.4)	76.6 (53.4 to 100.2)
		Southern Latin America	Deaths	Age-standardized	25.8 (23.6 to 28.8)	15.4 (13.2 to 19.9)	40.7 (37.6 to 43.8)	26.4 (23 to 29.5)	19 (14.6 to 22.2)	37.2 (32.8 to 41.8)	2.1 (-10.5 to 15.1)	23 (-9.6 to 48.3)	-8.5 (-20.2 to 3.9)
		Southern Latin America	DALYs	All Ages	255,646 (237,577 to 274,552)	85,794 (75,909 to 100,964)	169,852 (158,333 to 182,474)	453,490 (409,533 to 500,735)	191,201 (159,941 to 216,641)	262,289 (236,576 to 291,308)	77.4 (63.3 to 94.1)	122.9 (86.8 to 152.6)	54.4 (39.5 to 71.1)
		Southern Latin America	DALYs	Age-standardized	562.2 (521.3 to 605.6)	341.7 (302.8 to 402.4)	849.3 (789.9 to 909.5)	544.8 (493 to 601.2)	403.2 (341.2 to 454.3)	733.9 (662.5 to 815)	-3.1 (-11.2 to 6.1)	18 (0.3 to 33)	-13.6 (-22.1 to -4.4)
		Southern Latin America	YLLs	All Ages	205,801 (192,896 to 220,537)	64,029 (56,168 to 77,694)	141,772 (133,162 to 150,994)	349,697 (312,468 to 386,832)	139,246 (109,420 to 160,463)	210,451 (187,030 to 235,002)	69.9 (52.7 to 89.9)	117.5 (71.9 to 155.9)	48.4 (30.9 to 67.9)
		Southern Latin America	YLLs	Age-standardized	453.6 (424.3 to 488.6)	254.4 (223.2 to 309.2)	712.9 (667.8 to 759.8)	416.6 (373.1 to 460.1)	287.1 (228.8 to 330.9)	588.5 (524.4 to 657.2)	-8.2 (-17.7 to 2.9)	12.8 (-9.2 to 32.3)	-17.4 (-27.2 to -6.7)
		Southern Latin America	YLDs	All Ages	49,845 (36,939 to 61,763)	21,765 (16,372 to 27,677)	28,080 (20,235 to 35,690)	103,793 (77,752 to 128,706)	51,955 (38,670 to 66,144)	51,838 (36,744 to 65,310)	108.2 (96.6 to 120.2)	138.7 (120.6 to 157)	84.6 (73.1 to 96)
		Southern Latin America	YLDs	Age-standardized	108.5 (80.8 to 134.5)	87.3 (65.8 to 111)	136.4 (97.3 to 172.8)	128.2 (95.5 to 158.8)	116.1 (86.1 to 146.6)	145.5 (103.3 to 183.3)	18.1 (12.7 to 23.9)	33 (24.4 to 41.5)	6.6 (0.8 to 12.5)
High income	Southern Latin America	Argentina	Incidence	All Ages	49,117 (46,737 to 51,624)	18,438 (17,357 to 19,478)	30,679 (29,179 to 32,252)	110,517 (105,543 to 115,930)	50,341 (47,850 to 53,323)	60,176 (57,211 to 63,323)	125 (117.6 to 132.6)	173 (160.4 to 186.8)	96.1 (87.6 to 104.7)
High income	Southern Latin America	Argentina	Incidence	Age-standardized	156.7 (149.7 to 163.6)	108.4 (102.5 to 114.1)	215.3 (205.4 to 225.4)	210.4 (201.4 to 220.4)	169.5 (161.6 to 179.2)	259.2 (247.4 to 271.7)	34.3 (30.2 to 38.5)	56.4 (49.6 to 64.1)	20.4 (15.4 to 25.3)
High income	Southern Latin America	Argentina	Prevalence	All Ages	760,655 (729,290 to 792,614)	308,359 (291,901 to 325,146)	452,296 (432,978 to 471,617)	1,569,490 (1,498,398 to 1,649,152)	744,174 (705,983 to 789,613)	825,316 (785,567 to 867,771)	106.3 (99.8 to 113)	141.3 (130 to 154.2)	82.5 (74.8 to 90.5)
High income	Southern Latin America	Argentina	Prevalence	Age-standardized	2400.5 (2301.2 to 2498)	1792.5 (1701.3 to 1886.6)	3188.1 (3055.3 to 3325.3)	2973.1 (2836.9 to 3123.5)	2521.4 (2390 to 2673.7)	3587.3 (3419.8 to 3769)	23.9 (20 to 27.8)	40.7 (34 to 47.4)	12.5 (7.9 to 17.1)
High income	Southern Latin America	Argentina	Deaths	All Ages	7,946 (7,269 to 9,012)	2,665 (2,227 to 3,668)	5,281 (4,828 to 5,753)	16,396 (14,191 to 18,511)	7,216 (5,668 to 8,539)	9,180 (7,897 to 10,508)	106.3 (77.3 to 136.3)	170.7 (95.6 to 236)	73.9 (48.3 to 103)
High income	Southern Latin America	Argentina	Deaths	Age-standardized	26.8 (24.3 to 30.8)	15.7 (13 to 21.6)	42.8 (38.8 to 46.8)	29.4 (25.5 to 33.2)	21 (16.5 to 24.6)	42 (35.8 to 48.3)	9.9 (-6.6 to 25.9)	33.9 (-2.7 to 65.8)	-2.1 (-17.4 to 15)
High income	Southern Latin America	Argentina	DALYs	All Ages	188,334 (173,709 to 204,094)	61,839 (54,066 to 76,141)	126,495 (116,756 to 137,295)	333,875 (299,295 to 371,566)	139,160 (117,831 to 159,198)	194,715 (172,005 to 220,104)	77.3 (60.5 to 96.6)	125 (85.3 to 159.5)	53.9 (35.8 to 73.9)
High income	Southern Latin America	Argentina	DALYs	Age-standardized	593.7 (547.3 to 645.1)	354.2 (309.1 to 434.2)	906.6 (836.1 to 982.6)	619.8 (555.1 to 690.2)	451.8 (385.3 to 514.4)	844.6 (744.5 to 954.5)	4.4 (-5.8 to 15.7)	27.5 (6.5 to 46.2)	-6.8 (-17.8 to 5.2)
High income	Southern Latin America	Argentina	YLLs	All Ages	152,174 (140,189 to 166,442)	46,267 (40,069 to 59,464)	105,907 (97,747 to 114,967)	260,774 (229,219 to 292,408)	102,958 (83,792 to 119,558)	157,815 (137,320 to 179,457)	71.4 (50.4 to 95)	122.5 (72.7 to 167.7)	49 (27.8 to 72.8)
High income	Southern Latin America	Argentina	YLLs	Age-standardized	479.8 (442.6 to 528.5)	263.5 (228.4 to 337.3)	762.3 (701.8 to 826.5)	480.5 (422 to 538.7)	327.3 (270.5 to 381.3)	684.5 (598.5 to 777.5)	0.2 (-12.6 to 13.9)	24.2 (-1.9 to 49.3)	-10.2 (-22.7 to 3.9)
High income	Southern Latin America	Argentina	YLDs	All Ages	36,160 (26,740 to 45,118)	15,572 (11,450 to 19,834)	20,588 (14,680 to 26,330)	73,102 (53,967 to 90,878)	36,202 (26,760 to 46,128)	36,900 (26,026 to 46,695)	102.2 (89.5 to 115.5)	132.5 (113.4 to 152.7)	79.2 (65.5 to 92.8)
High income	Southern Latin America	Argentina	YLDs	Age-standardized	113.9 (84.2 to 141.7)	90.7 (67 to 115.5)	144.3 (103.4 to 184.2)	139.3 (102.1 to 172.5)	124.5 (92 to 158.2)	160.1 (113 to 202.6)	22.3 (15.7 to 29.3)	37.2 (26.3 to 49)	10.9 (3.3 to 18.9)
High income	Southern Latin America	Chile	Incidence	All Ages	13,604 (13,008 to 14,208)	6,099 (5,775 to 6,402)	7,506 (7,155 to 7,866)	40,182 (38,118 to 42,343)	19,981 (18,785 to 21,260)	20,201 (19,059 to 21,323)	195.4 (183 to 207)	227.6 (209.9 to 245.1)	169.1 (155.5 to 183.6)
High income	Southern Latin America	Chile	Incidence	Age-standardized	135.1 (129.3 to 140.7)	113.1 (107.5 to 118.4)	161 (153.7 to 168.8)	172.4 (163.8 to 181.2)	153.9 (145 to 163.4)	194.7 (184.4 to 204.8)	27.7 (23.1 to 32.4)	36.1 (29.3 to 43)	20.9 (15.2 to 26.7)
High income	Southern Latin America	Chile	Prevalence	All Ages	195,431 (183,368 to 207,594)	90,673 (84,354 to 97,235)	104,758 (98,489 to 111,345)	502,110 (472,286 to 532,266)	256,050 (238,578 to 273,720)	246,061 (230,555 to 262,022)	156.9 (145.1 to 169)	182.4 (165.4 to 199.8)	134.9 (121 to 149.3)
High income	Southern Latin America	Chile	Prevalence	Age-standardized	1902.4 (1801.7 to 2008.8)	1626.9 (1523.1 to 1729.7)	2246.2 (2125.7 to 2375.2)	2162.6 (2033.8 to 2291.4)	1997.3 (1858 to 2134.8)	2387.4 (2239.2 to 2546.9)	13.7 (8.9 to 18.4)	22.8 (16.3 to 29.5)	6.3 (0.6 to 12.4)
High income	Southern Latin America	Chile	Deaths	All Ages	1,982 (1,704 to 2,132)	802 (563 to 900)	1,180 (1,070 to 1,280)	4,410 (3,607 to 5,386)	2,079 (1,273 to 2,735)	2,331 (1,900 to 2,829)	122.5 (88.2 to 167.3)	159.2 (108.4 to 228.5)	97.5 (62.6 to 143)
High income	Southern Latin America	Chile	Deaths	Age-standardized	23.6 (20 to 25.5)	17.5 (12.3 to 19.6)	31.6 (28.5 to 34.5)	18.6 (15.1 to 22.6)	14.9 (9.2 to 19.5)	23.8 (19.3 to 28.9)	-21.1 (-33.1 to -5.6)	-14.8 (-30.7 to 7.5)	-24.8 (-37.9 to -7.7)
High income	Southern Latin America	Chile	DALYs	All Ages	45,427 (40,525 to 49,197)	18,406 (14,519 to 20,658)	27,021 (24,630 to 29,211)	83,191 (72,595 to 96,438)	38,949 (30,067 to 47,381)	44,242 (37,979 to 52,032)	83.1 (61.4 to 108.9)	111.6 (80.9 to 147.7)	63.7 (40.9 to 93.8)
High income	Southern Latin America	Chile	DALYs	Age-standardized	470.1 (418.8 to 508.5)	348 (272.3 to 389.3)	625.3 (569.5 to 676.6)	352.1 (307.2 to 406.9)	293.9 (230.7 to 356.4)	429.8 (369 to 504.6)	-25.1 (-33.9 to -15.1)	-15.6 (-27.6 to -2.2)	-31.3 (-40.7 to -19)
High income	Southern Latin America	Chile	YLLs	All Ages	36,040 (31,425 to 38,659)	13,812 (10,037 to 15,346)	22,229 (20,203 to 24,140)	60,017 (50,865 to 72,078)	26,684 (17,896 to 34,515)	33,333 (27,605 to 40,688)	66.5 (41.3 to 98.4)	93.2 (56.8 to 141.8)	50 (24.1 to 84.1)
High income	Southern Latin America	Chile	YLLs	Age-standardized	380.1 (330 to 408.7)	266.9 (192 to 297.1)	523.7 (477 to 568.6)	251.7 (213.8 to 301.5)	196.8 (134.1 to 252.6)	324 (268.5 to 394.4)	-33.8 (-43.7 to -21.2)	-26.3 (-40.1 to -9.1)	-38.1 (-48.6 to -24.1)
High income	Southern Latin America	Chile	YLDs	All Ages	9,387 (6,907 to 11,687)	4,594 (3,436 to 5,880)	4,793 (3,445 to 6,088)	23,173 (17,428 to 28,843)	12,265 (8,978 to 15,638)	10,909 (7,705 to 13,990)	146.9 (126.6 to 167.3)	167 (138.6 to 196.6)	127.6 (105.5 to 154.5)
High income	Southern Latin America	Chile	YLDs	Age-standardized	90.1 (66.5 to 112.3)	81.1 (60.4 to 103.8)	101.5 (72.3 to 128.8)	100.4 (75.1 to 124.7)	97.1 (71.4 to 123.6)	105.8 (74.8 to 134.7)	11.5 (4.5 to 18.7)	19.7 (10 to 30.4)	4.1 (-4.3 to 14.2)
High income	Southern Latin America	Uruguay	Incidence	All Ages	5,921 (5,653 to 6,183)	1,792 (1,680 to 1,897)	4,129 (3,926 to 4,319)	10,954 (10,501 to 11,336)	4,564 (4,312 to 4,825)	6,391 (6,122 to 6,612)	85 (78.1 to 92.3)	154.6 (140.9 to 168.6)	54.8 (47.1 to 62.6)
High income	Southern Latin America	Uruguay	Incidence	Age-standardized	163.8 (157.4 to 170.4)	92 (87.2 to 96.8)	252.8 (242.3 to 263)	218.2 (209.9 to 225.1)	155.4 (147.3 to 163.3)	297 (285.2 to 306.4)	33.2 (28.9 to 37.5)	68.8 (61 to 76.1)	17.5 (12.2 to 22.5)
High income	Southern Latin America	Uruguay	Prevalence	All Ages	90,928 (87,468 to 94,303)	31,485 (29,774 to 33,275)	59,444 (56,971 to 61,711)	162,524 (156,155 to 168,419)	71,912 (68,029 to 75,796)	90,611 (86,756 to 93,703)	78.7 (73.2 to 84.5)	128.4 (116.7 to 140.2)	52.4 (45.8 to 59)
High income	Southern Latin America	Uruguay	Prevalence	Age-standardized	2462 (2369.3 to 2550.1)	1590.8 (1505.1 to 1678.6)	3628.4 (3486 to 3755.1)	3173.3 (3057.3 to 3286.7)	2501.6 (2363.7 to 2643)	4144.1 (3974.4 to 4278.8)	28.9 (25.2 to 33.1)	57.3 (49.5 to 65.4)	14.2 (9.3 to 18.8)
High income	Southern Latin America	Uruguay	Deaths	All Ages	976 (876 to 1,129)	246 (191 to 399)	730 (662 to 798)	1,879 (1,638 to 2,076)	689 (545 to 826)	1,190 (1,043 to 1,334)	92.5 (63.6 to 121.2)	180.2 (67.8 to 279.4)	62.9 (38.9 to 86.1)
High income	Southern Latin America	Uruguay	Deaths	Age-standardized	25 (22.5 to 29.2)	10.7 (8.4 to 17.2)	46.2 (41.9 to 50.5)	30.8 (27 to 33.9)	17.6 (14 to 20.8)	51.4 (45.2 to 57.6)	22.8 (3 to 41)	64.1 (-4.2 to 119.2)	11.3 (-4.8 to 26.9)
High income	Southern Latin America	Uruguay	DALYs	All Ages	21,875 (19,852 to 24,239)	5,546 (4,661 to 7,627)	16,329 (14,729 to 17,833)	36,401 (32,598 to 39,873)	13,082 (10,850 to 15,047)	23,319 (20,673 to 26,116)	66.4 (46.4 to 85.6)	135.9 (58.8 to 185.9)	42.8 (24.7 to 60.9)
High income	Southern Latin America	Uruguay	DALYs	Age-standardized	566.6 (514.8 to 628.1)	262.4 (223 to 353)	973.2 (879.4 to 1059.9)	679.6 (608.7 to 744.8)	424.5 (347 to 483.9)	1039.7 (922.3 to 1165)	19.9 (5.1 to 33.8)	61.8 (8.3 to 94.7)	6.8 (-6.2 to 20.6)
High income	Southern Latin America	Uruguay	YLLs	All Ages	17,579 (15,872 to 19,740)	3,948 (3,188 to 6,089)	13,631 (12,348 to 14,960)	28,888 (25,672 to 31,865)	9,597 (7,489 to 11,198)	19,292 (16,915 to 21,794)	64.3 (39.9 to 88.1)	143.1 (43.5 to 219.3)	41.5 (20.2 to 63)
High income	Southern Latin America	Uruguay	YLLs	Age-standardized	449.6 (407.2 to 504.4)	180.5 (147.4 to 273.3)	808.6 (733.9 to 885.8)	530.5 (470.6 to 585.2)	299.7 (227 to 348.4)	854.5 (747.9 to 966.1)	18 (-0.5 to 35.9)	66 (-4.8 to 114.2)	5.7 (-10.1 to 22.1)
High income	Southern Latin America	Uruguay	YLDs	All Ages	4,296 (3,170 to 5,351)	1,598 (1,186 to 2,037)	2,698 (1,914 to 3,421)	7,513 (5,561 to 9,384)	3,485 (2,559 to 4,459)	4,027 (2,800 to 5,110)	74.9 (64.7 to 85.3)	118.1 (98.5 to 136.3)	49.3 (40 to 59.2)
High income	Southern Latin America	Uruguay	YLDs	Age-standardized	117 (86.7 to 144.8)	81.9 (60.3 to 104.4)	164.6 (118.1 to 207.4)	149.1 (111.5 to 185.4)	124.8 (92.4 to 159.1)	185.2 (130.3 to 235.3)	27.4 (20.1 to 34.7)	52.5 (39.7 to 65.4)	12.5 (5.9 to 19.4)
		High-income North America	Incidence	All Ages	766,833 (711,534 to 814,556)	359,824 (325,110 to 390,692)	407,010 (382,390 to 427,240)	1,462,937 (1,386,853 to 1,529,434)	748,119 (705,932 to 784,852)	714,818 (680,242 to 744,442)	90.8 (84.8 to 98.4)	107.9 (96.4 to 122.2)	75.6 (72.1 to 80)
		High-income North America	Incidence	Age-standardized	228.8 (213.6 to 242)	200.8 (183 to 216)	272.9 (257.4 to 286.7)	249.1 (237.1 to 259.7)	241.2 (228.8 to 252)	260.5 (248.7 to 271.1)	8.8 (5.6 to 12.8)	20.1 (14.3 to 27.5)	-4.6 (-6.2 to -2.4)
		High-income North America	Prevalence	All Ages	12,245,378 (11,349,362 to 12,977,981)	6,546,077 (5,992,534 to 7,020,188)	5,699,302 (5,358,872 to 5,976,567)	21,968,671 (20,985,083 to 22,806,701)	11,943,445 (11,386,318 to 12,404,007)	10,025,225 (9,618,592 to 10,401,210)	79.4 (73.9 to 86.3)	82.5 (74.6 to 92.8)	75.9 (72.2 to 81)
		High-income North America	Prevalence	Age-standardized	3521 (3283.3 to 3725.2)	3305.5 (3048.9 to 3532.3)	3900.2 (3675.4 to 4089.7)	3558.4 (3412.9 to 3694.4)	3537.3 (3380.5 to 3666.5)	3602.9 (3462.4 to 3736.3)	1.1 (-1.9 to 4.9)	7 (2.7 to 12.3)	-7.6 (-9.5 to -5.2)
		High-income North America	Deaths	All Ages	98,505 (91,237 to 111,414)	41,726 (36,969 to 54,636)	56,779 (53,956 to 58,590)	211,434 (173,959 to 229,899)	106,385 (67,940 to 119,483)	105,049 (95,973 to 113,191)	114.6 (72.5 to 135.3)	155 (48.4 to 193.3)	85 (73.4 to 99.1)
		High-income North America	Deaths	Age-standardized	26.5 (24.6 to 30)	18.8 (16.8 to 24.2)	39.3 (37.1 to 40.7)	31.2 (25.9 to 33.8)	27.4 (17.9 to 30.5)	36.4 (33.3 to 39.3)	17.7 (-4.6 to 28.6)	46.2 (-12 to 66.4)	-7.3 (-13.2 to -0.4)
		High-income North America	DALYs	All Ages	2,884,661 (2,668,157 to 3,103,137)	1,345,467 (1,208,146 to 1,549,200)	1,539,194 (1,440,459 to 1,630,662)	5,346,347 (4,812,349 to 5,695,195)	2,728,901 (2,182,336 to 2,958,780)	2,617,446 (2,426,438 to 2,793,434)	85.3 (66.7 to 95.2)	102.8 (58.2 to 119.5)	70.1 (63.4 to 78.4)
		High-income North America	DALYs	Age-standardized	814.8 (752.3 to 875.2)	670 (601.9 to 764.3)	1034.8 (967 to 1098.2)	848.4 (765.9 to 903)	794.4 (643.3 to 860.6)	919.7 (852.5 to 982.1)	4.1 (-5.7 to 9.3)	18.6 (-5.5 to 27.6)	-11.1 (-14.4 to -6.9)
		High-income North America	YLLs	All Ages	1,722,052 (1,628,549 to 1,910,284)	719,748 (657,879 to 904,118)	1,002,303 (958,576 to 1,029,002)	3,317,412 (2,811,582 to 3,545,526)	1,623,800 (1,087,473 to 1,778,673)	1,693,612 (1,578,577 to 1,815,182)	92.6 (62.5 to 107.7)	125.6 (44.3 to 153.6)	69 (59.2 to 81.1)
		High-income North America	YLLs	Age-standardized	478.3 (452.7 to 529)	350.4 (321.8 to 436.5)	668 (638.4 to 686.7)	516.4 (439.2 to 550.4)	462.2 (314.9 to 503.9)	586.2 (546.4 to 628.4)	8 (-8.4 to 16.2)	31.9 (-13.5 to 47.2)	-12.3 (-17.3 to -6.1)
		High-income North America	YLDs	All Ages	1,162,609 (967,316 to 1,317,528)	625,719 (512,185 to 721,231)	536,891 (444,128 to 617,309)	2,028,935 (1,698,596 to 2,265,411)	1,105,101 (913,259 to 1,260,685)	923,834 (770,624 to 1,058,399)	74.5 (68.6 to 81.6)	76.6 (68.6 to 86.6)	72.1 (67.8 to 77.1)
		High-income North America	YLDs	Age-standardized	336.5 (279.6 to 381.2)	319.6 (261.6 to 369.2)	366.8 (303.2 to 422.1)	332 (277.3 to 370.8)	332.2 (272 to 377.7)	333.5 (277.7 to 381.8)	-1.3 (-4.5 to 2.7)	3.9 (-0.6 to 9.2)	-9.1 (-11.1 to -6.6)
High income	High-income North America	Canada	Incidence	All Ages	63,263 (60,787 to 65,456)	25,698 (24,088 to 27,215)	37,565 (36,014 to 38,801)	140,331 (133,759 to 147,450)	70,969 (66,871 to 75,242)	69,362 (65,766 to 73,185)	121.8 (112.8 to 132.8)	176.2 (159.9 to 194.6)	84.6 (74.8 to 95)
High income	High-income North America	Canada	Incidence	Age-standardized	196.6 (189.5 to 203.4)	147.8 (138.9 to 155.9)	263.1 (253.2 to 271.1)	209.6 (200.2 to 219.6)	199.5 (188.9 to 211)	223.1 (212.7 to 234.6)	6.6 (2.5 to 11.1)	35 (27.7 to 43.2)	-15.2 (-19.3 to -10.8)
High income	High-income North America	Canada	Prevalence	All Ages	833,885 (793,343 to 875,356)	390,211 (363,789 to 421,307)	443,674 (421,774 to 464,331)	1,818,278 (1,721,234 to 1,934,105)	986,874 (926,054 to 1,065,845)	831,404 (784,120 to 883,695)	118 (108.6 to 129)	152.9 (137.5 to 169.7)	87.4 (76.5 to 98.7)
High income	High-income North America	Canada	Prevalence	Age-standardized	2582 (2456.9 to 2710.6)	2152.7 (2001.2 to 2319.6)	3239.3 (3089.1 to 3381.2)	2646.5 (2494.6 to 2810.7)	2635.8 (2470.8 to 2842.8)	2683.9 (2525.4 to 2844.1)	2.5 (-1.9 to 7.1)	22.4 (15.3 to 30.5)	-17.1 (-21.9 to -12.2)
High income	High-income North America	Canada	Deaths	All Ages	7,999 (7,353 to 8,490)	2,760 (2,400 to 3,202)	5,239 (4,875 to 5,557)	15,578 (12,650 to 18,110)	7,362 (4,629 to 9,039)	8,216 (6,840 to 9,634)	94.8 (64.1 to 125.2)	166.8 (76.4 to 228.7)	56.8 (31.3 to 84.5)
High income	High-income North America	Canada	Deaths	Age-standardized	24.6 (22.5 to 26.2)	14.2 (12.4 to 16.5)	42.1 (38.7 to 44.7)	20.3 (16.8 to 23.4)	16.7 (11 to 20.2)	25.6 (21.2 to 30)	-17.5 (-28.9 to -5)	17.5 (-18.4 to 42.6)	-39.2 (-49 to -28.7)
High income	High-income North America	Canada	DALYs	All Ages	179,469 (165,834 to 192,015)	68,650 (61,320 to 77,705)	110,819 (102,219 to 118,869)	324,035 (285,829 to 360,471)	159,526 (129,689 to 184,557)	164,508 (144,392 to 187,509)	80.6 (65.3 to 98.5)	132.4 (88.2 to 162.3)	48.4 (31.1 to 66.1)
High income	High-income North America	Canada	DALYs	Age-standardized	546.7 (505.9 to 585.3)	370.6 (330.8 to 418.3)	810 (746.6 to 867.6)	457.3 (404.8 to 507.7)	414.9 (343.9 to 475.5)	514.7 (452 to 585.6)	-16.4 (-22.9 to -8.6)	12 (-7 to 25.8)	-36.5 (-43.6 to -29.1)
High income	High-income North America	Canada	YLLs	All Ages	128,856 (120,355 to 137,684)	44,089 (39,576 to 52,377)	84,767 (79,076 to 89,955)	216,415 (186,481 to 246,693)	99,781 (70,550 to 119,194)	116,634 (98,903 to 136,028)	68 (46.6 to 93.1)	126.3 (62.6 to 174.2)	37.6 (15.4 to 61.7)
High income	High-income North America	Canada	YLLs	Age-standardized	389.9 (363.9 to 417.4)	234.2 (210.4 to 281.3)	621 (580.5 to 658.4)	299 (258.9 to 339.5)	252.5 (185.6 to 298.8)	359.7 (304.1 to 418.6)	-23.3 (-32.6 to -12.6)	7.8 (-19.7 to 29)	-42.1 (-51.2 to -32.3)
High income	High-income North America	Canada	YLDs	All Ages	50,613 (39,781 to 60,237)	24,561 (18,897 to 30,312)	26,052 (19,940 to 31,483)	107,620 (85,651 to 127,623)	59,745 (45,521 to 74,619)	47,875 (36,857 to 57,935)	112.6 (100.1 to 125.8)	143.3 (123.8 to 164.4)	83.8 (70.9 to 97.1)
High income	High-income North America	Canada	YLDs	Age-standardized	156.8 (123.9 to 186)	136.4 (105.1 to 167.8)	189 (145.1 to 228.3)	158.3 (125.1 to 187.9)	162.4 (124.5 to 202.3)	155.1 (120.3 to 187.6)	0.9 (-4.6 to 6.9)	19 (10 to 29.2)	-18 (-23.4 to -12.4)
High income	High-income North America	Greenland	Incidence	All Ages	96 (93 to 99)	51 (49 to 52)	45 (43 to 48)	167 (160 to 173)	88 (84 to 90)	79 (74 to 84)	73.9 (68.1 to 80.1)	73 (67.9 to 77.5)	74.8 (64.2 to 87.4)
High income	High-income North America	Greenland	Incidence	Age-standardized	264.1 (254.8 to 272.4)	279.2 (269.7 to 287.7)	259.1 (247 to 269.6)	244 (233.9 to 253.5)	279.4 (268.3 to 288.8)	215.7 (203.9 to 228.3)	-7.6 (-10.3 to -5)	0.1 (-2.7 to 2.6)	-16.8 (-21 to -12.7)
High income	High-income North America	Greenland	Prevalence	All Ages	1,143 (1,087 to 1,195)	654 (625 to 684)	489 (458 to 523)	2,126 (2,041 to 2,215)	1,224 (1,173 to 1,268)	902 (841 to 961)	86.1 (79.9 to 92.6)	87.3 (81.2 to 93)	84.5 (73.2 to 97.5)
High income	High-income North America	Greenland	Prevalence	Age-standardized	3505.4 (3384.4 to 3625.9)	3895.3 (3758.3 to 4037.3)	3158.7 (2991.4 to 3321.7)	3304.6 (3167.6 to 3442.7)	3962 (3792.7 to 4104.8)	2714.3 (2540.6 to 2889.7)	-5.7 (-8.5 to -3)	1.7 (-1.3 to 4.3)	-14.1 (-18.7 to -8.9)
High income	High-income North America	Greenland	Deaths	All Ages	20 (16 to 23)	12 (9 to 15)	8 (6 to 9)	28 (21 to 33)	15 (10 to 19)	12 (10 to 15)	38.6 (12.3 to 73.9)	23.7 (-6.4 to 68.9)	63.1 (28.7 to 102.6)
High income	High-income North America	Greenland	Deaths	Age-standardized	81 (62.5 to 92.8)	89.8 (62.6 to 107.6)	70.6 (56.9 to 80.3)	49.1 (35.9 to 58.6)	54.4 (34.2 to 67)	44.6 (36 to 52.6)	-39.3 (-50.4 to -24.5)	-39.5 (-53.8 to -19)	-36.8 (-49.2 to -22.7)
High income	High-income North America	Greenland	DALYs	All Ages	507 (425 to 571)	306 (243 to 357)	200 (171 to 230)	690 (563 to 796)	383 (295 to 454)	307 (254 to 360)	36.1 (14.6 to 65.4)	25.1 (2.1 to 62.2)	52.9 (24.2 to 86.5)
High income	High-income North America	Greenland	DALYs	Age-standardized	1699.8 (1403.2 to 1915.9)	1945.1 (1515.1 to 2266.3)	1440.4 (1217.2 to 1639.1)	1070.9 (864.5 to 1235)	1247.6 (951.1 to 1485.2)	919.2 (769.2 to 1061.3)	-37 (-46.7 to -24)	-35.9 (-47.3 to -17.1)	-36.2 (-47.6 to -23.8)
High income	High-income North America	Greenland	YLLs	All Ages	415 (337 to 476)	252 (188 to 299)	163 (134 to 189)	520 (399 to 622)	282 (194 to 351)	238 (183 to 290)	25.3 (0.1 to 60.7)	12 (-15.4 to 56.5)	45.9 (11.9 to 86.2)
High income	High-income North America	Greenland	YLLs	Age-standardized	1423.5 (1131.3 to 1625.5)	1626.9 (1180.7 to 1927.3)	1203.2 (973.6 to 1385.4)	809.3 (616.8 to 967.4)	923 (622.8 to 1139.3)	714 (563.2 to 852.1)	-43.1 (-54.2 to -28.2)	-43.3 (-56.7 to -21.4)	-40.7 (-53.3 to -26.4)
High income	High-income North America	Greenland	YLDs	All Ages	92 (76 to 106)	54 (45 to 64)	38 (30 to 45)	170 (141 to 194)	101 (82 to 118)	69 (54 to 83)	84.9 (76.4 to 93.8)	85.9 (76.2 to 95.8)	83.5 (68.9 to 99.1)
High income	High-income North America	Greenland	YLDs	Age-standardized	276.3 (229.7 to 314.8)	318.3 (263.1 to 372)	237.2 (188.9 to 281.1)	261.6 (216.7 to 298.8)	324.6 (264.9 to 380.4)	205.2 (163.3 to 243.9)	-5.3 (-9 to -1.6)	2 (-2.8 to 6.4)	-13.5 (-18.9 to -7.8)
High income	High-income North America	United States of America	Incidence	All Ages	703,457 (649,067 to 750,488)	334,067 (300,002 to 364,324)	369,390 (345,030 to 389,480)	1,322,417 (1,249,460 to 1,385,858)	677,051 (637,246 to 711,790)	645,366 (612,100 to 673,577)	88 (81.4 to 95.9)	102.7 (90.4 to 117.5)	74.7 (71 to 79.5)
High income	High-income North America	United States of America	Incidence	Age-standardized	232.7 (215.7 to 247)	206.7 (187.8 to 223.1)	274.4 (257.3 to 289.3)	253.6 (240.9 to 264.6)	246 (233.1 to 257.3)	264.6 (251.8 to 275.8)	9 (5.4 to 13.3)	19 (12.9 to 26.7)	-3.6 (-5.4 to -1.1)
High income	High-income North America	United States of America	Prevalence	All Ages	11,410,071 (10,538,686 to 12,133,375)	6,155,063 (5,616,330 to 6,622,189)	5,255,009 (4,931,024 to 5,529,151)	20,147,917 (19,242,749 to 20,933,265)	10,955,158 (10,423,481 to 11,386,858)	9,192,760 (8,815,493 to 9,530,652)	76.6 (70.6 to 83.8)	78 (69.9 to 88.4)	74.9 (71 to 80.5)
High income	High-income North America	United States of America	Prevalence	Age-standardized	3622.1 (3363.7 to 3846.2)	3424.9 (3150.9 to 3670.5)	3975.9 (3734.7 to 4182.8)	3668.5 (3514.6 to 3805.4)	3644.8 (3477 to 3782.1)	3715.6 (3567.6 to 3854.4)	1.3 (-2 to 5.4)	6.4 (1.8 to 11.9)	-6.5 (-8.5 to -3.8)
High income	High-income North America	United States of America	Deaths	All Ages	90,485 (83,712 to 103,200)	38,953 (34,494 to 51,493)	51,531 (48,939 to 53,229)	195,825 (161,220 to 212,293)	99,006 (63,270 to 110,883)	96,819 (88,913 to 104,140)	116.4 (72.8 to 137.5)	154.2 (46.8 to 192.5)	87.9 (76.6 to 101.9)
High income	High-income North America	United States of America	Deaths	Age-standardized	26.7 (24.8 to 30.4)	19.2 (17.3 to 25)	39.2 (37 to 40.6)	32.5 (27 to 35.2)	28.7 (18.7 to 31.9)	37.8 (34.7 to 40.7)	21.7 (-2 to 33)	49.4 (-10.9 to 70.1)	-3.4 (-9 to 3.7)
High income	High-income North America	United States of America	DALYs	All Ages	2,704,619 (2,498,892 to 2,918,098)	1,276,479 (1,143,867 to 1,474,289)	1,428,140 (1,335,713 to 1,514,194)	5,021,538 (4,512,795 to 5,344,914)	2,568,948 (2,055,029 to 2,788,274)	2,452,590 (2,273,862 to 2,614,188)	85.7 (66.7 to 95.5)	101.3 (56.6 to 117.6)	71.7 (65.2 to 80.1)
High income	High-income North America	United States of America	DALYs	Age-standardized	843.5 (779 to 907.6)	700.8 (628.8 to 797.4)	1060.4 (989.1 to 1126.9)	896 (806.8 to 954.5)	839.8 (678.3 to 910.9)	969.9 (897.5 to 1034.4)	6.2 (-4 to 11.4)	19.8 (-4.7 to 29)	-8.5 (-11.8 to -4.1)
High income	High-income North America	United States of America	YLLs	All Ages	1,592,741 (1,505,377 to 1,778,284)	675,390 (617,348 to 851,492)	917,351 (876,664 to 941,853)	3,100,425 (2,620,307 to 3,305,632)	1,523,711 (1,018,858 to 1,667,484)	1,576,714 (1,477,202 to 1,690,486)	94.7 (63.1 to 110)	125.6 (43.3 to 153.1)	71.9 (62.2 to 84.3)
High income	High-income North America	United States of America	YLLs	Age-standardized	488.2 (462 to 544.2)	362.4 (332.8 to 452.9)	674.5 (644.5 to 693.4)	542.9 (461.2 to 577.3)	487.4 (330.1 to 530.4)	614.4 (575.8 to 659)	11.2 (-6.2 to 19.8)	34.5 (-12.4 to 49.8)	-8.9 (-14.1 to -2.3)
High income	High-income North America	United States of America	YLDs	All Ages	1,111,878 (924,354 to 1,262,675)	601,089 (490,117 to 695,474)	510,789 (422,841 to 588,086)	1,921,113 (1,606,464 to 2,147,990)	1,045,237 (858,323 to 1,191,793)	875,876 (732,512 to 1,004,946)	72.8 (66.7 to 80)	73.9 (65.6 to 84)	71.5 (67 to 76.8)
High income	High-income North America	United States of America	YLDs	Age-standardized	355.3 (294.6 to 402.7)	338.4 (276.3 to 392)	385.9 (319.9 to 445)	353.1 (294.7 to 394.7)	352.5 (288.2 to 401)	355.5 (296.4 to 406.7)	-0.6 (-3.9 to 3.5)	4.2 (-0.6 to 9.5)	-7.9 (-10 to -5.2)
		Latin America and Caribbean	Incidence	All Ages	396,361 (371,667 to 421,781)	188,471 (175,681 to 201,631)	207,890 (194,814 to 220,697)	946,454 (882,288 to 1,013,901)	464,323 (434,074 to 498,443)	482,131 (449,202 to 517,064)	138.8 (133.4 to 144.3)	146.4 (139.8 to 153.3)	131.9 (126.4 to 137.8)
		Latin America and Caribbean	Incidence	Age-standardized	165 (153.6 to 176.4)	148.6 (137.8 to 160.1)	183.4 (170.4 to 195.2)	163.2 (152.2 to 175)	147.8 (138.2 to 159)	181.3 (169.1 to 194)	-1.1 (-3.1 to 0.8)	-0.5 (-3.1 to 2.1)	-1.1 (-3.2 to 1)
		Latin America and Caribbean	Prevalence	All Ages	5,564,002 (5,261,029 to 5,883,891)	2,773,368 (2,617,206 to 2,945,049)	2,790,634 (2,638,016 to 2,948,261)	13,340,129 (12,520,217 to 14,266,644)	6,739,199 (6,322,569 to 7,225,825)	6,600,930 (6,198,837 to 7,044,520)	139.8 (134.1 to 145.2)	143 (136.7 to 149.5)	136.5 (130.9 to 142.2)
		Latin America and Caribbean	Prevalence	Age-standardized	2313.8 (2178.1 to 2462.5)	2187.6 (2059.1 to 2333.3)	2461.5 (2313.6 to 2613.1)	2281 (2140.4 to 2444.2)	2130.1 (1997.9 to 2287.1)	2467.4 (2317.7 to 2638.1)	-1.4 (-3.6 to 0.6)	-2.6 (-5.3 to -0.1)	0.2 (-1.9 to 2.4)
		Latin America and Caribbean	Deaths	All Ages	66,230 (60,899 to 69,024)	26,963 (22,715 to 28,872)	39,267 (37,007 to 40,724)	152,734 (134,633 to 169,697)	70,201 (58,597 to 80,397)	82,533 (73,897 to 91,009)	130.6 (110.7 to 150.9)	160.4 (131.3 to 188.5)	110.2 (92.1 to 130.3)
		Latin America and Caribbean	Deaths	Age-standardized	36.5 (33 to 38.3)	28.1 (23.9 to 30.4)	46.2 (42.8 to 48.2)	27.7 (24.3 to 30.7)	22.5 (18.8 to 25.7)	34.3 (30.7 to 37.9)	-24.2 (-30.4 to -17.7)	-20.1 (-28.8 to -11.8)	-25.7 (-31.8 to -18.7)
		Latin America and Caribbean	DALYs	All Ages	1,548,015 (1,441,534 to 1,633,974)	653,209 (565,923 to 702,318)	894,807 (848,449 to 937,993)	3,063,031 (2,784,977 to 3,371,539)	1,409,600 (1,239,481 to 1,589,959)	1,653,430 (1,499,966 to 1,817,987)	97.9 (83.7 to 114.4)	115.8 (95.9 to 138.5)	84.8 (71 to 99.8)
		Latin America and Caribbean	DALYs	Age-standardized	709.6 (659.8 to 746.8)	562.7 (487.2 to 603.9)	875.8 (826.6 to 916.9)	534.4 (485.2 to 588.1)	449 (394.8 to 506.4)	639.4 (580 to 702.7)	-24.7 (-29.9 to -18.7)	-20.2 (-27.2 to -12.3)	-27 (-32.4 to -21)
		Latin America and Caribbean	YLLs	All Ages	1,293,704 (1,199,792 to 1,345,245)	514,647 (426,809 to 548,377)	779,057 (746,800 to 809,782)	2,468,369 (2,233,374 to 2,733,284)	1,084,452 (940,617 to 1,245,876)	1,383,916 (1,255,791 to 1,521,586)	90.8 (73.6 to 110.1)	110.7 (85.7 to 140)	77.6 (61.8 to 94.7)
		Latin America and Caribbean	YLLs	Age-standardized	607.6 (562.2 to 631.3)	457.7 (381.8 to 488.7)	776.7 (738.4 to 804.3)	433.3 (391.5 to 479.3)	346.3 (300.6 to 397.8)	539.5 (489.5 to 592.9)	-28.7 (-34.8 to -21.8)	-24.4 (-32.9 to -14.9)	-30.5 (-36.7 to -23.7)
		Latin America and Caribbean	YLDs	All Ages	254,311 (193,148 to 313,504)	138,562 (105,664 to 170,487)	115,750 (83,116 to 148,456)	594,662 (447,529 to 737,754)	325,148 (246,768 to 405,318)	269,514 (193,158 to 349,015)	133.8 (124 to 142.8)	134.7 (123.1 to 144.9)	132.8 (122.1 to 143.3)
		Latin America and Caribbean	YLDs	Age-standardized	102 (75.6 to 126.3)	104.9 (79.6 to 130.8)	99.2 (71.1 to 127.4)	101.2 (76 to 125.3)	102.7 (78 to 127.9)	99.9 (71.5 to 128.7)	-0.8 (-3.2 to 1.6)	-2.1 (-5.2 to 0.8)	0.8 (-1.9 to 3.5)
		Caribbean	Incidence	All Ages	27,491 (26,360 to 28,652)	12,795 (12,263 to 13,345)	14,696 (14,069 to 15,339)	65,372 (62,626 to 68,325)	30,805 (29,370 to 32,248)	34,566 (33,098 to 36,130)	137.8 (132.2 to 143.6)	140.8 (133.7 to 148.3)	135.2 (129 to 141.9)
		Caribbean	Incidence	Age-standardized	98.8 (95 to 102.9)	87.5 (83.9 to 91.1)	111.4 (107.1 to 116.3)	127.5 (122.1 to 133.2)	114 (108.7 to 119.3)	143.4 (137.5 to 149.8)	29.1 (26.6 to 31.5)	30.3 (27.2 to 34)	28.7 (25.6 to 32)
		Caribbean	Prevalence	All Ages	437,591 (415,043 to 459,808)	217,486 (206,331 to 228,267)	220,105 (208,296 to 232,566)	1,050,382 (999,066 to 1,099,680)	526,325 (499,159 to 553,132)	524,057 (498,997 to 550,068)	140 (134.1 to 146.8)	142 (134.2 to 150.4)	138.1 (131.1 to 145.9)
		Caribbean	Prevalence	Age-standardized	1582.5 (1508.2 to 1660.9)	1508.4 (1436.3 to 1583.6)	1669.5 (1586.6 to 1763)	2044.2 (1944.5 to 2141)	1939.3 (1838.3 to 2036.8)	2174.5 (2070.9 to 2279.5)	29.2 (26.5 to 32)	28.6 (24.8 to 32.5)	30.2 (26.8 to 33.8)
		Caribbean	Deaths	All Ages	4,483 (3,990 to 4,967)	1,744 (1,475 to 2,156)	2,739 (2,364 to 3,053)	10,651 (8,894 to 12,381)	4,411 (3,097 to 5,336)	6,240 (5,262 to 7,240)	137.6 (98.2 to 175.1)	152.9 (79.4 to 205.9)	127.8 (94.3 to 163.9)
		Caribbean	Deaths	Age-standardized	18.7 (16.8 to 20.8)	13.9 (11.7 to 17.2)	24.4 (21.2 to 27)	20.6 (17.2 to 23.9)	15.4 (10.8 to 18.7)	26.9 (22.7 to 31.1)	9.7 (-8.4 to 26.6)	11.4 (-21.1 to 34.5)	10.2 (-5.8 to 26.7)
		Caribbean	DALYs	All Ages	109,006 (96,930 to 122,154)	45,524 (38,317 to 54,977)	63,482 (54,178 to 73,351)	237,878 (205,525 to 270,360)	103,168 (80,522 to 121,306)	134,711 (114,434 to 156,644)	118.2 (89.7 to 147.7)	126.6 (80.6 to 163.7)	112.2 (81 to 146.5)

Chronic obstructive pulmonary disease

7 GBD super region	21 GBD region	Location	Measure	Age	1990			2019			Percent change (1990 to 2019)		
					Both	Female	Male	Both	Female	Male	Both	Female	Male
		Caribbean	DALYs	Age-standardized	411.3 (368.2 to 456.7)	328.6 (277.6 to 395.2)	502.7 (434.2 to 568.2)	463.2 (400.1 to 527.7)	377.1 (294.2 to 443.8)	564 (480.4 to 654.7)	12.6 (-2.3 to 27.5)	14.8 (-8.9 to 32.8)	12.2 (-2.7 to 29.2)
		Caribbean	YLLs	All Ages	87,407 (76,165 to 99,611)	33,864 (27,682 to 42,660)	53,543 (44,822 to 63,623)	187,874 (156,472 to 221,784)	76,192 (53,705 to 93,312)	111,683 (92,596 to 132,715)	114.9 (80.4 to 151.8)	125 (62.8 to 173.9)	108.6 (73 to 150.2)
		Caribbean	YLLs	Age-standardized	334.3 (293.3 to 376.3)	249.1 (206 to 309.7)	428.1 (362.7 to 491.2)	365.7 (303.4 to 431.7)	277 (194.8 to 339.8)	468.6 (387.7 to 556.9)	9.4 (-8.4 to 27.7)	11.2 (-20 to 34.7)	9.5 (-7.6 to 29.7)
		Caribbean	YLDs	All Ages	21,599 (16,210 to 26,500)	11,660 (8,694 to 14,611)	9,939 (7,302 to 12,794)	50,004 (37,516 to 62,008)	26,976 (19,952 to 34,399)	23,028 (16,483 to 29,775)	131.5 (119.2 to 141.9)	131.4 (114.3 to 145.9)	131.7 (118.1 to 145.1)
		Caribbean	YLDs	Age-standardized	76.9 (58 to 94.9)	79.5 (58.8 to 100.2)	74.6 (54.4 to 95.2)	97.5 (73.1 to 120.7)	100.1 (74.2 to 127.1)	95.4 (68.4 to 122.8)	26.7 (21.9 to 31.1)	25.9 (19.1 to 32.3)	27.9 (22.1 to 33.7)
Latin America and Caribbean	Caribbean	Antigua and Barbuda	Incidence	All Ages	30 (28 to 32)	13 (12 to 14)	17 (16 to 18)	59 (56 to 63)	28 (26 to 30)	31 (29 to 33)	98.8 (89.4 to 108.6)	119.4 (106.9 to 133.8)	83.1 (72.6 to 93.4)
Latin America and Caribbean	Caribbean	Antigua and Barbuda	Incidence	Age-standardized	52.1 (49.2 to 55.1)	38.6 (36 to 41)	73.2 (69.1 to 77.4)	61.9 (58.4 to 65.7)	56.6 (53.1 to 60.6)	67.1 (63.1 to 71.6)	18.8 (14.7 to 22.6)	46.9 (40.4 to 53.9)	-8.3 (-12.7 to -3.7)
Latin America and Caribbean	Caribbean	Antigua and Barbuda	Prevalence	All Ages	466 (424 to 507)	222 (200 to 244)	244 (222 to 266)	1,052 (965 to 1,146)	528 (481 to 579)	524 (477 to 572)	125.6 (114.4 to 137.4)	137.4 (122 to 155.3)	114.8 (101.8 to 129.1)
Latin America and Caribbean	Caribbean	Antigua and Barbuda	Prevalence	Age-standardized	841.2 (769.6 to 912.4)	716.4 (641.8 to 790.2)	1033.8 (952.2 to 1116.5)	1057.2 (970 to 1148.1)	1006.5 (916.4 to 1099.5)	1114.6 (1019.8 to 1213.5)	25.7 (20.5 to 31.2)	40.5 (31.7 to 50.3)	7.8 (2.2 to 13.9)
Latin America and Caribbean	Caribbean	Antigua and Barbuda	Deaths	All Ages	4 (4 to 5)	1 (1 to 2)	3 (2 to 3)	7 (6 to 9)	3 (2 to 4)	4 (4 to 5)	66 (34.2 to 103.4)	93.8 (43.4 to 154.8)	51.9 (16.4 to 97.1)
Latin America and Caribbean	Caribbean	Antigua and Barbuda	Deaths	Age-standardized	7.4 (6.5 to 8.4)	3.9 (3.1 to 4.7)	14.1 (12.1 to 16.5)	8.4 (6.8 to 10.1)	5.9 (4.2 to 7.4)	11.8 (9.4 to 14.7)	13.1 (-8.9 to 38)	52.3 (12.2 to 98.2)	-16.6 (-35.9 to 6.5)
Latin America and Caribbean	Caribbean	Antigua and Barbuda	DALYs	All Ages	87 (78 to 98)	31 (27 to 37)	56 (49 to 63)	170 (143 to 199)	73 (58 to 86)	98 (80 to 119)	95.4 (65.3 to 128.9)	132.2 (90.7 to 178.2)	74.7 (42 to 117.1)
Latin America and Caribbean	Caribbean	Antigua and Barbuda	DALYs	Age-standardized	154.7 (138.3 to 173.9)	95.7 (80.9 to 112.6)	247.3 (216.3 to 280.8)	178.2 (150.5 to 207.5)	141.8 (112.2 to 167.8)	222.8 (183.3 to 269.1)	15.2 (-2.6 to 34.8)	48.3 (22.1 to 78.4)	-9.9 (-26.7 to 11.1)
Latin America and Caribbean	Caribbean	Antigua and Barbuda	YLLs	All Ages	64 (57 to 73)	19 (16 to 23)	45 (38 to 52)	119 (97 to 145)	45 (33 to 57)	74 (59 to 95)	85.5 (48.5 to 128.5)	131.9 (72.3 to 203.4)	65.3 (25.9 to 117.3)
Latin America and Caribbean	Caribbean	Antigua and Barbuda	YLLs	Age-standardized	113.5 (99.7 to 127.8)	57 (46 to 67.7)	201.4 (171.9 to 232.8)	126.9 (103.8 to 153.5)	88.8 (64.9 to 111.6)	173.3 (138.4 to 216.5)	11.7 (-10.8 to 37.5)	55.8 (14.2 to 105.8)	-13.9 (-33.7 to 11)
Latin America and Caribbean	Caribbean	Antigua and Barbuda	YLDs	All Ages	23 (17 to 29)	12 (9 to 16)	11 (8 to 14)	51 (38 to 64)	28 (21 to 36)	23 (17 to 30)	123.3 (102.7 to 144.2)	132.6 (102.8 to 165.2)	113.1 (86.1 to 140.1)
Latin America and Caribbean	Caribbean	Antigua and Barbuda	YLDs	Age-standardized	41.2 (31.2 to 52)	38.7 (28.4 to 50.3)	46 (33 to 59.7)	51.3 (38 to 64.4)	53.1 (39.4 to 68.6)	49.5 (35.7 to 63.8)	24.6 (13.9 to 35)	37.1 (19.6 to 56)	7.5 (-4.6 to 19.5)
Latin America and Caribbean	Caribbean	Bahamas	Incidence	All Ages	117 (110 to 125)	48 (44 to 52)	69 (65 to 74)	263 (249 to 279)	109 (102 to 116)	154 (145 to 165)	124.2 (113.7 to 135.1)	126.3 (112.4 to 142.5)	122.7 (111.3 to 134.5)
Latin America and Caribbean	Caribbean	Bahamas	Incidence	Age-standardized	61.9 (58.8 to 65.3)	45.7 (43 to 48.7)	82.9 (78.7 to 87.6)	68.6 (65.1 to 72.5)	53.6 (50.4 to 57)	86.1 (81.4 to 91.3)	10.8 (7.5 to 14)	17.2 (12.6 to 22.1)	3.8 (-0.3 to 8)
Latin America and Caribbean	Caribbean	Bahamas	Prevalence	All Ages	2,031 (1,846 to 2,210)	901 (802 to 998)	1,130 (1,031 to 1,232)	4,772 (4,432 to 5,132)	2,101 (1,919 to 2,296)	2,671 (2,484 to 2,872)	134.9 (123.8 to 147.4)	133.2 (118.4 to 150.3)	136.3 (122.5 to 152.1)
Latin America and Caribbean	Caribbean	Bahamas	Prevalence	Age-standardized	1076.1 (996.7 to 1155.7)	860.7 (783.6 to 937.6)	1359.9 (1266.4 to 1461.2)	1194.7 (1113.2 to 1281.7)	979.1 (896.4 to 1066.9)	1457 (1360.6 to 1560.2)	11 (7 to 14.9)	13.8 (7.8 to 20.6)	7.1 (2 to 12.5)
Latin America and Caribbean	Caribbean	Bahamas	Deaths	All Ages	13 (12 to 15)	4 (3 to 5)	9 (8 to 11)	33 (26 to 41)	10 (8 to 13)	22 (17 to 28)	143.4 (89.7 to 210.5)	143.5 (77.1 to 233.4)	143.4 (83.5 to 222.1)
Latin America and Caribbean	Caribbean	Bahamas	Deaths	Age-standardized	10.2 (8.8 to 11.7)	5.4 (4.2 to 6.6)	18.7 (15.8 to 21.9)	9.7 (7.8 to 12.1)	5.3 (4.1 to 6.9)	16.1 (12.6 to 20.3)	-4.7 (-25 to 20.9)	-1.5 (-28.6 to 35.9)	-13.7 (-34.3 to 13.2)
Latin America and Caribbean	Caribbean	Bahamas	DALYs	All Ages	364 (321 to 410)	128 (108 to 150)	236 (203 to 273)	854 (713 to 1,020)	299 (245 to 362)	555 (447 to 681)	134.6 (93.6 to 183.4)	133.3 (92.7 to 184)	135.4 (85.2 to 201.8)
Latin America and Caribbean	Caribbean	Bahamas	DALYs	Age-standardized	230.5 (202.5 to 259.3)	139.5 (117.2 to 162.9)	363.5 (313.3 to 419.9)	225.6 (188.5 to 268.2)	142.9 (117.4 to 172.9)	332.5 (269.1 to 407.5)	-2.1 (-19.4 to 18.9)	2.4 (-16.1 to 25.7)	-8.5 (-27.4 to 16)
Latin America and Caribbean	Caribbean	Bahamas	YLLs	All Ages	262 (225 to 301)	78 (63 to 95)	184 (154 to 219)	623 (494 to 777)	187 (140 to 247)	435 (337 to 556)	137.2 (81.8 to 208.9)	139.1 (74.1 to 227.8)	136.4 (74.8 to 221.3)
Latin America and Caribbean	Caribbean	Bahamas	YLLs	Age-standardized	178.2 (152.6 to 204.5)	93.2 (74.9 to 113.4)	302.7 (254.8 to 358.9)	167.9 (133.4 to 209.2)	90.9 (68.9 to 118.9)	267.7 (209.4 to 338.2)	-5.7 (-27.5 to 21.1)	-2.5 (-29.1 to 32.8)	-11.6 (-33.5 to 17.3)
Latin America and Caribbean	Caribbean	Bahamas	YLDs	All Ages	102 (79 to 129)	50 (37 to 65)	52 (38 to 67)	232 (175 to 290)	112 (82 to 143)	120 (85 to 156)	127.9 (106.1 to 150.8)	124.1 (95.6 to 158.2)	131.6 (104.7 to 161.2)
Latin America and Caribbean	Caribbean	Bahamas	YLDs	Age-standardized	52.4 (39.9 to 65.4)	46.3 (34.2 to 59.8)	60.8 (44.4 to 79.2)	57.7 (43.8 to 71.9)	52 (38.7 to 66.3)	64.8 (46.8 to 84.1)	10.2 (2 to 18.9)	12.4 (0.6 to 26.5)	6.6 (-2.8 to 17)
Latin America and Caribbean	Caribbean	Barbados	Incidence	All Ages	154 (145 to 165)	65 (61 to 70)	89 (83 to 95)	287 (269 to 305)	127 (119 to 136)	159 (149 to 171)	86.2 (77.6 to 95.7)	94.9 (83.6 to 108.2)	79.7 (68.8 to 90.7)
Latin America and Caribbean	Caribbean	Barbados	Incidence	Age-standardized	54 (50.9 to 57.1)	40.4 (37.8 to 43)	73.7 (69.6 to 77.9)	66.8 (62.8 to 70.7)	55.2 (51.7 to 58.6)	81.8 (76.8 to 87.1)	23.7 (19.6 to 28.2)	36.5 (30.5 to 43)	11 (5.9 to 16.3)
Latin America and Caribbean	Caribbean	Barbados	Prevalence	All Ages	2,394 (2,205 to 2,594)	1,125 (1,025 to 1,226)	1,268 (1,164 to 1,384)	4,459 (4,129 to 4,810)	2,138 (1,963 to 2,312)	2,320 (2,144 to 2,517)	86.3 (78.1 to 95.1)	90 (79.7 to 101.1)	83 (72 to 93.9)
Latin America and Caribbean	Caribbean	Barbados	Prevalence	Age-standardized	863.9 (793.8 to 938.3)	739.7 (668.9 to 811.6)	1053 (969.1 to 1145.8)	1039.6 (957.4 to 1124.9)	936.5 (856.4 to 1019.3)	1175.3 (1080 to 1274.3)	20.3 (15.5 to 25.2)	26.6 (19.2 to 34.2)	11.6 (5.4 to 17.8)
Latin America and Caribbean	Caribbean	Barbados	Deaths	All Ages	23 (20 to 27)	9 (7 to 11)	15 (12 to 17)	41 (33 to 50)	16 (12 to 20)	25 (20 to 31)	76.8 (39.2 to 118.3)	84.1 (35.1 to 149.8)	72.6 (30.4 to 121.8)
Latin America and Caribbean	Caribbean	Barbados	Deaths	Age-standardized	7.2 (6.3 to 8.3)	4.4 (3.5 to 5.5)	11.9 (10 to 13.9)	8.3 (6.6 to 10)	5.5 (4.1 to 7.1)	12 (9.4 to 14.7)	14.3 (-8.9 to 40.3)	25.1 (-7.5 to 68.5)	0.6 (-24.2 to 28.7)
Latin America and Caribbean	Caribbean	Barbados	DALYs	All Ages	467 (413 to 524)	183 (152 to 215)	284 (246 to 327)	844 (707 to 985)	345 (275 to 413)	499 (408 to 597)	80.5 (51.1 to 113.6)	88.5 (51.5 to 133.1)	75.3 (39.6 to 115.6)
Latin America and Caribbean	Caribbean	Barbados	DALYs	Age-standardized	154.4 (136.5 to 173.5)	107.7 (90.5 to 127.1)	225.6 (194.7 to 259.8)	177.7 (150.5 to 206.9)	135.1 (108.8 to 160.6)	232.9 (191.8 to 276.2)	15.1 (-2.3 to 34.5)	25.4 (2.7 to 53.3)	3.3 (-17.2 to 26.4)
Latin America and Caribbean	Caribbean	Barbados	YLLs	All Ages	350 (304 to 399)	123 (98 to 151)	227 (192 to 264)	631 (501 to 767)	235 (174 to 301)	396 (309 to 491)	80.2 (41 to 126.2)	91.1 (39.1 to 157.9)	74.3 (31.1 to 126.8)
Latin America and Caribbean	Caribbean	Barbados	YLLs	Age-standardized	111.8 (97.9 to 127.9)	67.6 (53.8 to 82.6)	178.2 (150.9 to 208.6)	127.1 (101.3 to 154.2)	85.4 (63.3 to 109.2)	180.6 (142.1 to 222.8)	13.7 (-10.3 to 41.4)	26.4 (-6.9 to 70.3)	1.3 (-23.8 to 32.2)
Latin America and Caribbean	Caribbean	Barbados	YLDs	All Ages	117 (88 to 148)	60 (44 to 78)	57 (41 to 75)	212 (159 to 266)	110 (80 to 141)	102 (74 to 135)	81.3 (65.8 to 98.3)	83 (61.2 to 106.3)	79.4 (59.4 to 100.9)
Latin America and Caribbean	Caribbean	Barbados	YLDs	Age-standardized	42.6 (31.8 to 53.6)	40.2 (29.8 to 52.1)	47.4 (34.3 to 62.3)	50.6 (37.7 to 63.3)	49.7 (36.8 to 63.9)	52.3 (37.9 to 68.4)	18.6 (8.9 to 29.1)	23.8 (8.3 to 39.9)	10.5 (-1.2 to 22.7)
Latin America and Caribbean	Caribbean	Belize	Incidence	All Ages	105 (99 to 110)	44 (42 to 47)	61 (57 to 64)	345 (329 to 362)	141 (134 to 148)	204 (194 to 215)	229.6 (216.8 to 243.2)	218.5 (203.7 to 234.9)	237.8 (221.4 to 254)
Latin America and Caribbean	Caribbean	Belize	Incidence	Age-standardized	89.6 (85.5 to 94.1)	71.3 (67.4 to 75.2)	108.7 (103.2 to 114.4)	106.4 (101.4 to 111.6)	83.3 (78.9 to 87.7)	129 (122.6 to 136)	18.7 (15.1 to 22.3)	16.8 (12.4 to 21.4)	18.6 (13.4 to 23.4)
Latin America and Caribbean	Caribbean	Belize	Prevalence	All Ages	1,606 (1,500 to 1,699)	725 (672 to 773)	881 (821 to 937)	5,738 (5,463 to 6,028)	2,514 (2,360 to 2,661)	3,225 (3,059 to 3,398)	257.4 (242.7 to 274.3)	246.7 (229.7 to 266)	266.1 (245.3 to 288.7)
Latin America and Caribbean	Caribbean	Belize	Prevalence	Age-standardized	1440.6 (1354.7 to 1520.9)	1276.1 (1193.2 to 1357.4)	1615.2 (1519.4 to 1716.9)	1821.7 (1729.7 to 1917.6)	1549.1 (1460.8 to 1634.8)	2087.3 (1977.4 to 2205.6)	26.5 (21.7 to 31.1)	21.4 (15.9 to 27.4)	29.2 (22.4 to 35.8)
Latin America and Caribbean	Caribbean	Belize	Deaths	All Ages	14 (12 to 16)	5 (4 to 6)	9 (7 to 11)	50 (42 to 59)	14 (11 to 17)	36 (30 to 43)	260.4 (188.5 to 355.7)	183.5 (112.6 to 271.1)	303 (209.1 to 431)
Latin America and Caribbean	Caribbean	Belize	Deaths	Age-standardized	15.5 (12.9 to 18)	10.6 (8.5 to 13.4)	21 (16.7 to 25.6)	20.6 (17.2 to 24.5)	11.4 (8.9 to 14.1)	29.9 (24.6 to 35.8)	33.2 (6.8 to 68.2)	7.6 (-19.3 to 41.8)	42.4 (9.7 to 86.7)
Latin America and Caribbean	Caribbean	Belize	DALYs	All Ages	336 (294 to 380)	129 (109 to 150)	207 (171 to 243)	1,276 (1,106 to 1,472)	420 (349 to 495)	856 (718 to 1,009)	279.7 (216.3 to 358.5)	226 (165.7 to 293.5)	313.1 (226.7 to 430.7)
Latin America and Caribbean	Caribbean	Belize	DALYs	Age-standardized	338.5 (294.3 to 384.6)	252.6 (211.3 to 299.4)	429.1 (349 to 504.5)	457.5 (394.6 to 530.4)	289.9 (240.1 to 343.7)	619.8 (520.9 to 732.6)	35.1 (12.3 to 65.3)	14.8 (-7.6 to 41.3)	44.4 (13.9 to 85.7)
Latin America and Caribbean	Caribbean	Belize	YLLs	All Ages	255 (214 to 293)	89 (72 to 109)	166 (131 to 201)	993 (828 to 1,181)	283 (224 to 349)	709 (584 to 863)	289 (208 to 402.2)	219.3 (138.4 to 318.6)	326.1 (219.4 to 473.8)
Latin America and Caribbean	Caribbean	Belize	YLLs	Age-standardized	267.8 (223 to 309.3)	184.2 (148.4 to 229.8)	355.5 (277.9 to 429.9)	369.4 (309.2 to 439.2)	208 (164.2 to 256)	526.1 (434.1 to 634.9)	37.9 (9.2 to 77.9)	12.9 (-16 to 48.8)	48 (11.6 to 99.8)
Latin America and Caribbean	Caribbean	Belize	YLDs	All Ages	81 (62 to 100)	40 (30 to 51)	41 (30 to 53)	283 (213 to 355)	137 (101 to 175)	147 (107 to 190)	250.5 (221.2 to 280.9)	240.8 (203.5 to 281.9)	259.9 (223.6 to 300)
Latin America and Caribbean	Caribbean	Belize	YLDs	Age-standardized	70.7 (52.8 to 87.2)	68.4 (50.8 to 86.7)	73.6 (53.5 to 95)	88 (66.1 to 110.3)	81.9 (61.1 to 105)	93.7 (68.5 to 122.4)	24.5 (16.3 to 33.1)	19.8 (8.6 to 31.1)	27.4 (16.9 to 39.2)
Latin America and Caribbean	Caribbean	Bermuda	Incidence	All Ages	45 (42 to 48)	17 (16 to 18)	28 (27 to 30)	101 (94 to 108)	35 (32 to 37)	66 (62 to 71)	123.9 (112.4 to 134.9)	106.5 (92.4 to 121.5)	134.1 (120.8 to 147.5)
Latin America and Caribbean	Caribbean	Bermuda	Incidence	Age-standardized	74.4 (70.4 to 78.5)	50.4 (47.4 to 53.8)	107.6 (101.8 to 113.9)	87.5 (82.4 to 92.7)	54.9 (50.9 to 58.7)	128.4 (120.9 to 136.5)	17.7 (13.4 to 22)	8.8 (3.8 to 13.9)	19.3 (13.8 to 25.5)
Latin America and Caribbean	Caribbean	Bermuda	Prevalence	All Ages	752 (695 to 813)	315 (287 to 346)	436 (405 to 470)	1,499 (1,401 to 1,603)	562 (515 to 610)	937 (879 to 1,007)	99.4 (89.7 to 109.7)	78.3 (67.2 to 90)	114.6 (101.9 to 127.7)
Latin America and Caribbean	Caribbean	Bermuda	Prevalence	Age-standardized	1194.8 (1110.3 to 1285.1)	914.9 (837.8 to 1001.4)	1588.2 (1483.4 to 1699.8)	1324 (1224.5 to 1423.8)	949 (859.9 to 1040.2)	1815.9 (1694.3 to 1949.4)	10.8 (6.4 to 15.3)	3.7 (-1.9 to 10)	14.3 (8.4 to 20.2)
Latin America and Caribbean	Caribbean	Bermuda	Deaths	All Ages	7 (6 to 8)	2 (2 to 3)	4 (4 to 5)	12 (10 to 15)	4 (3 to 5)	8 (7 to 11)	83.6 (49.5 to 130.4)	79 (35.2 to 138.5)	85.9 (43.9 to 143.6)
Latin America and Caribbean	Caribbean	Bermuda	Deaths	Age-standardized	11.8 (10.1 to 13.7)	6.4 (5.1 to 7.9)	20.7 (17.2 to 24.3)	8.6 (6.9 to 10.8)	4.4 (3.2 to 5.8)	15 (11.9 to 19.1)	-26.8 (-40.3 to -8)	-31.8 (-47.8 to -8.9)	-27.4 (-43.5 to -6.6)
Latin America and Caribbean	Caribbean	Bermuda	DALYs	All Ages	152 (133 to 173)	52 (43 to 60)	100 (84 to 117)	245 (204 to 295)	78 (63 to 94)	167 (137 to 204)	60.8 (35.7 to 91.9)	49.9 (25.4 to 83.4)	66.5 (36.8 to 107.7)
Latin America and Caribbean	Caribbean	Bermuda	DALYs	Age-standardized	249.3 (217.4 to 283.4)	149 (125.4 to 174)	396.3 (334.2 to 459.5)	194.1 (163.1 to 231.4)	113.5 (93.6 to 136)	302.2 (247.7 to 365.2)	-22.1 (-34.1 to -7.5)	-23.9 (-36.1 to -8.7)	-23.8 (-37 to -6.1)
Latin America and Caribbean	Caribbean	Bermuda	YLLs	All Ages	116 (99 to 135)	35 (28 to 42)	81 (66 to 97)	175 (141 to 219)	49 (36 to 65)	126 (100 to 161)	51.7 (21 to 93.6)	40.6 (5.8 to 89.1)	56.5 (21 to 108.1)
Latin America and Caribbean	Caribbean	Bermuda	YLLs	Age-standardized	191.3 (164.4 to 223.2)	99.7 (80.8 to 121.2)	325.5 (267.9 to 386)	130.9 (105.4 to 163.5)	62.6 (47 to 82)	222.3 (177.6 to 283)	-31.6 (-45.6 to -13)	-37.2 (-52.4 to -15.5)	-31.7 (-47.1 to -10.4)
Latin America and Caribbean	Caribbean	Bermuda	YLDs	All Ages	37 (27 to 46)	17 (12 to 22)	20 (14 to 26)	69 (51 to 88)	29 (21 to 37)	41 (29 to 53)	89.6 (70.8 to 108.6)	69 (47.6 to 91.4)	107.4 (82 to 132.3)
Latin America and Caribbean	Caribbean	Bermuda	YLDs	Age-standardized	58 (43.2 to 72.3)	49.3 (36.2 to 64)	70.9 (51.5 to 92)	63.2 (48.5 to 79.4)	50.8 (37.2 to 65.6)	79.9 (57.7 to 103.6)	9 (0.2 to 17.7)	3.1 (-9 to 15.9)	12.8 (2.5 to 23.2)
Latin America and Caribbean	Caribbean	Cuba	Incidence	All Ages	11,290 (10,831 to 11,843)	5,292 (5,040 to 5,560)	5,998 (5,701 to 6,342)	28,392 (27,025 to 29,668)	13,678 (12,900 to 14,411)	14,714 (13,958 to 15,487)	151.5 (141.9 to 162.6)	158.5 (146.3 to 172.3)	145.3 (132.6 to 158.4)
Latin America and Caribbean	Caribbean	Cuba	Incidence	Age-standardized	107.7 (103.4 to 112.7)	99.6 (94.8 to 104.5)	116.5 (111 to 122.5)	160.7 (153.4 to 167.8)	151.3 (143.1 to 159.1)	173.1 (164.6 to 181.2)	49.1 (43.9 to 54.8)	51.9 (45.1 to 59.3)	48.7 (41.5 to 55.9)
Latin America and Caribbean	Caribbean	Cuba	Prevalence	All Ages	189,823 (180,545 to 200,065)	97,549 (92,302 to 103,168)	92,274 (86,963 to 98,248)	475,795 (453,102 to 499,433)	252,004 (237,863 to 266,918)	223,791 (211,846 to 236,176)	150.7 (140.5 to 162.2)	158.3 (144.3 to 173.6)	142.5 (128.8 to 156.3)
Latin America and Caribbean	Caribbean	Cuba	Prevalence	Age-standardized	1825.2 (1740 to 1924.3)	1855.8 (1756.6 to 1963.5)	1799 (1700.1 to 1915.4)	2641.2 (2507.4 to 2766.9)	2672.5 (2523.8 to 2828)	2627.8 (2486.5 to 2773.2)	44.7 (39.2 to 50.7)	44 (36.4 to 52.3)	46.1 (38.3 to 53.8)
Latin America and Caribbean	Caribbean	Cuba	Deaths	All Ages	1,551 (1,423 to 1,663)	637 (552 to 721)	914 (843 to 983)	4,233 (3,272 to 5,201)	1,853 (1,086 to 2,376)	2,380 (1,899 to 2,936)	172.9 (112 to 233.7)	191.1 (84.3 to 280.9)	160.3 (104.6 to 221.1)
Latin America and Caribbean	Caribbean	Cuba	Deaths	Age-standardized	15.7 (14.3 to 16.9)	12.6 (10.9 to 14.3)	19 (17.5 to 20.5)	21.2 (16.3 to 26)	17.2 (9.9 to 22.1)	26.3 (20.9 to 32.3)	35.4 (4.4 to 65.1)	36.7 (-16.6 to 79.5)	38.1 (8.6 to 70.1)
Latin America and Caribbean	Caribbean	Cuba	DALYs	All Ages	36,958 (33,793 to 39,871)	17,144 (14,696 to 19,122)	19,814 (18,098 to 21,469)	93,410 (74,346 to 111,373)	44,623 (28,959 to 54,456)	48,786 (40,356 to 58,945)	152.7 (105.8 to 199.6)	160.3 (88.6 to 219.9)	146.2 (102.5 to 197.1)
Latin America and Caribbean	Caribbean	Cuba	DALYs	Age-standardized	359 (327.7 to 387.7)	328.3 (281.7 to 366.3)	392.4 (357.8 to 424.8)	497.6 (397.4 to 591.6)	453.7 (297.8 to 553.8)	552.7 (458.6 to 668.2)	38.6 (13.2 to 63.6)	38.2 (0.9 to 69.1)	40.9 (16.1 to 69.6)
Latin America and Caribbean	Caribbean	Cuba	YLLs	All Ages	27,655 (25,289 to 29,535)	11,983 (9,895 to 13,288)	15,671 (14,468 to 16,920)	71,051 (52,558 to 88,401)	31,959 (17,376 to 41,437)	39,092 (30,730 to 48,928)	156.9 (95.5 to 219.1)	166.7 (64.4 to 252.2)	149.5 (93.7 to 215.5)
Latin America and Caribbean	Caribbean	Cuba	YLLs	Age-standardized	270 (247.2 to 288.6)	230.6 (190.5 to 256)	312 (287.7 to 336.9)	371.7 (275.3 to 463.8)	316.5 (171.6 to 410.1)	438.1 (344.5 to 547.8)	37.7 (4.7 to 70.6)	37.2 (-15.8 to 82)	40.4 (9.2 to 77.4)
Latin America and Caribbean	Caribbean	Cuba	YLDs	All Ages	9,303 (6,973 to 11,566)	5,160 (3,775 to 6,615)	4,143 (3,034 to 5,341)	22,358 (16,591 to 27,924)	12,664 (9,206 to 16,210)	9,694 (6,925 to 12,666)	140.3 (121.4 to 158.6)	145.4 (120.4 to 171.1)	134 (111.8 to 157.8)
Latin America and Caribbean	Caribbean	Cuba	YLDs	Age-standardized	89 (66.5 to 110.8)	97.7 (71.4 to 125.2)	80.4 (58.6 to 103.1)	125.8 (94.7 to 156.7)	137.1 (100.4 to 175.5)	114.6 (82.2 to 149.1)	41.4 (32 to 50.4)	40.4 (27.6 to 53.2)	42.6 (30.5 to 54.8)
Latin America and Caribbean	Caribbean	Dominica	Incidence	All Ages	47 (44 to 49)	19 (18 to 20)	27 (26 to 29)	73 (69 to 78)	27 (25 to 28)	47 (44 to 50)	57.6 (51 to 64.6)	40.1 (32.3 to 47.8)	69.8 (61.3 to 79.3)
Latin America and Caribbean	Caribbean	Dominica	Incidence	Age-standardized	64.7 (61.3 to 68.2)	46.5 (43.8 to 49.2)	91 (86.2 to 96.2)	86.7 (82.2 to 91.2)	62.2 (58.5 to 66.1)	113 (107.4 to 119.3)	34 (30 to 38.1)	34 (28.3 to 39.7)	24.2 (19.1 to 29.2)
Latin America and Caribbean	Caribbean	Dominica	Prevalence	All Ages	721 (668 to 775)	322 (295 to 348)	399 (369 to 432)	1,163 (1,095 to 1,239)	458 (426 to 493)	705 (663 to 750)	61.3 (54.6 to 69)	42.4 (34.7 to 50.6)	76.6 (66.4 to 87.1)
Latin America and Caribbean	Caribbean	Dominica	Prevalence	Age-standardized	1044 (968.1 to 1127.4)	843.6 (766.6 to 922)	1337.4 (1244.1 to 1443.1)	1371.2 (1287 to 1461.3)	1077.7 (995.1 to 1166.2)	1687.7 (1590.5 to 1792.9)	31.3 (26.1 to 36.8)	27.8 (20.9 to 35.6)	26.2 (19.8 to 32.9)
Latin America and Caribbean	Caribbean	Dominica	Deaths	All Ages	11 (10 to 14)	4 (3 to 5)	8 (6 to 9)	15 (12 to 19)	5 (3 to 6)	11 (8 to 14)	34.5 (-5.6 to 72.2)	14.5 (-40 to 58.1)	45 (-3 to 90.9)
Latin America and Caribbean	Caribbean	Dominica	Deaths	Age-standardized	15.2 (13.3 to 18.1)	8.4 (6.9 to 11.6)	26.4 (22.8 to 33.5)	16.8 (13.2 to 20.6)	8.8 (5.4 to 11.2)	27.4 (21 to 34.4)	10.9 (-22.1 to 41.9)	4.2 (-45.5 to 42.6)	3.6 (-29.8 to 34.5)
Latin America and Caribbean	Caribbean	Dominica	DALYs	All Ages	224 (198 to 262)	79 (66 to 99)	145 (126 to 177)	305 (248 to 365)	91 (65 to 112)	214 (171 to 264)	35.9 (1.9 to 69.6)	15 (-27.3 to 46.3)	47.2 (5.1 to 88.6)
Latin America and Caribbean	Caribbean	Dominica	DALYs	Age-standardized	304.2 (269.6 to 356.2)	185.1 (155.2 to 231.3)	484.5 (421 to 592.2)	340.9 (277.6 to 408.5)	197 (143.6 to 242.6)	504.2 (402.5 to 620.5)	12.1 (-15.9 to 39.1)	6.4 (-30.8 to 35.1)	4 (-26.3 to 32.4)
Latin America and Caribbean	Caribbean	Dominica	YLLs	All Ages	189 (165 to 227)	62 (50 to 83)	127 (108 to 159)	250 (194 to 309)	67 (41 to 86)	183 (139 to 232)	32.2 (-7 to 72)	8.4 (-42.3 to 48.6)	43.7 (-2.8 to 91.4)
Latin America and Caribbean	Caribbean	Dominica	YLLs	Age-standardized	253.5 (221.2 to 304.3)	139.9 (113.3 to 187.3)	425 (364.5 to 534.2)	276 (213.6 to 340.8)	140.3 (85.1 to 181.2)	430.2 (327.5 to 543.4)	8.9 (-23.7 to 41.3)	0.2 (-46.9 to 37.1)	1.2 (-32 to 34.2)
Latin America and Caribbean	Caribbean	Dominica	YLDs	All Ages	35 (26 to 44)	17 (12 to 22)	18 (13 to 23)	54 (41 to 68)	24 (17 to 30)	31 (22 to 40)	56.2 (42.6 to 69.6)	39.1 (23.8 to 55.9)	72.5 (54.6 to 91.1)
Latin America and Caribbean	Caribbean	Dominica	YLDs	Age-standardized	50.6 (38.5 to 63.6)	45.2 (33.2 to 58.8)	59.5 (42.9 to 76.9)	64.9 (48.6 to 80.6)	56.7 (41.9 to 73.1)	74 (54.1 to 95.9)	28.3 (18.1 to 39.2)	25.5 (11.5 to 41.9)	24.3 (13.5 to 35.9)
Latin America and Caribbean	Caribbean	Dominican Republic	Incidence	All Ages	2,714 (2,548 to 2,883)	1,357 (1,268 to 1,453)	1,357 (1,269 to 1,447)	7,708 (7,305 to 8,130)	3,529 (3,328 to 3,738)	4,179 (3,956 to 4,434)	184 (171.2 to 197)	160 (145.1 to 174.5)	207.9 (190.4 to 226.6)
Latin America and Caribbean	Caribbean	Dominican Republic	Incidence	Age-standardized	56.8 (53.9 to 59.8)	52.5 (49.6 to 55.8)	61.2 (58 to 64.4)	80 (75.9 to 84.2)	70.3 (66.2 to 74.4)	91 (86 to 96.3)	40.8 (36.1 to 45.3)	34 (27.8 to 39.9)	48.7 (42.2 to 56)
Latin America and Caribbean	Caribbean	Dominican Republic	Prevalence	All Ages	45,803 (41,800 to 49,395)	24,380 (22,264 to 26,347)	21,424 (19,309 to 23,490)	129,279 (120,868 to 137,518)	63,692 (59,483 to 67,866)	65,587 (60,585 to 70,459)	182.2 (168.4 to 196.5)	161.2 (145.1 to 177.8)	206.1 (185.5 to 230)
Latin America and Caribbean	Caribbean	Dominican Republic	Prevalence	Age-standardized	945.2 (877.6 to 1010.6)	955.9 (884 to 1029.6)	932.4 (860.8 to 1006.5)	1316.3 (1235.1 to 1396.8)	1257.7 (1176.6 to 1341.3)	1387.9 (1293.9 to 1483.4)	39.3 (34 to 44.6)	31.6 (24.6 to 38.4)	48.9 (40.3 to 57.2)
Latin America and Caribbean	Caribbean	Dominican Republic	Deaths	All Ages	385 (329 to 453)	162 (129 to 215)	223 (187 to 264)	1,362 (1,021 to 1,764)	571 (364 to 784)	791 (591 to 1,080)	254.1 (158.5 to 372.6)	253.3 (108.4 to 403.9)	254.7 (157.4 to 401.7)
Latin America and Caribbean	Caribbean	Dominican Republic	Deaths	Age-standardized	12.9 (10.9 to 15)	10.7 (8.5 to 13.5)	15.2 (12.7 to 17.9)	16 (12.1 to 20.6)	12.6 (8 to 17.2)	19.9 (15 to 26.9)	24 (-7.7 to 65.2)	17.2 (-27.3 to 65)	31 (-4.6 to 83.9)
Latin America and Caribbean	Caribbean	Dominican Republic	DALYs	All Ages	10,126 (8,858 to 11,700)	4,617 (3,845 to 5,949)	5,508 (4,779 to 6,330)	31,322 (24,266 to 39,293)	13,236 (9,538 to 17,372)	18,086 (13,591 to 24,239)	209.3 (137.5 to 300.9)	186.7 (91.5 to 298.8)	228.3 (145.5 to 347.7)
Latin America and Caribbean	Caribbean	Dominican Republic	DALYs	Age-standardized	255.4 (223.5 to 297.8)	222.5 (184.5 to 291.2)	288.8 (250.3 to 334.1)	337.6 (262.9 to 423.9)	272.7 (197.1 to 358.2)	409.2 (309.7 to 547.9)	32.2 (1.3 to 71.8)	22.6 (-20 to 67.2)	41.7 (6.4 to 94.5)
Latin America and Caribbean	Caribbean	Dominican Republic	YLLs	All Ages	7,737 (6,555 to 9,316)	3,228 (2,552 to 4,544)	4,509 (3,828 to 5,308)	24,924 (18,221 to 33,345)	9,806 (6,247 to 13,983)	15,118 (10,652 to 21,243)	222.1 (128.9 to 346)	203.8 (74 to 359.3)	235.3 (136.6 to 384.2)
Latin America and Caribbean	Caribbean	Dominican Republic	YLLs	Age-standardized	207.8 (176.8 to 247.8)	170.3 (135.8 to 237.3)	246.2 (209.1 to 290.2)	273.1 (201.3 to 362.3)	205.7 (131.1 to 292)	347 (249.7 to 482.7)	31.5 (-5.9 to 79.8)	20.8 (-31.2 to 77.7)	41 (0.7 to 103.4)
Latin America and Caribbean	Caribbean	Dominican Republic	YLDs	All Ages	2,389 (1,812 to 2,990)	1,389 (1,019 to 1,768)	999 (726 to 1,292)	6,399 (4,837 to 7,924)	3,431 (2,554 to 4,387)	2,968 (2,112 to 3,845)	167.9 (142.1 to 196.7)	146.9 (114.3 to 183.2)	197 (158.8 to 245.1)
Latin America and Caribbean	Caribbean	Dominican Republic	YLDs	Age-standardized	47.6 (36.1 to 59)	52.2 (38.4 to 66.3)	42.6 (30.9 to 54.8)	64.4 (48.7 to 79.7)	67.1 (49.5 to 86)	62.2 (44.2 to 80.3)	35.4 (25.4 to 46.6)	28.4 (14.6 to 44)	46 (30.7 to 64.6)
Latin America and Caribbean	Caribbean	Grenada	Incidence	All Ages	58 (55 to 61)	28 (26 to 29)	31 (29 to 32)	94 (89 to 100)	47 (44 to 50)	47 (44 to 50)	61.4 (54.2 to 68.1)	70.1 (61.6 to 78.5)	53.5 (44.5 to 62.6)
Latin America and Caribbean	Caribbean	Grenada	Incidence	Age-standardized	76.7 (73 to 80.6)	63.8 (60.4 to 67.2)	94.2 (89 to 99.2)	87.7 (83.3 to 92.2)	84.2 (79.4 to 89.1)	90.9 (86.1 to 96)	14.3 (11 to 17.7)	32 (26.8 to 37.4)	-3.5 (-7.6 to 0.8)
Latin America and Caribbean	Caribbean	Grenada	Prevalence	All Ages	935 (878 to 991)	469 (440 to 498)	466 (433 to 498)	1,673 (1,570 to 1,778)	827 (773 to 878)	847 (788 to 909)	79 (72.3 to 85.9)	76.5 (68.5 to 85.1)	81.6 (72.3 to 91.5)
Latin America and Caribbean	Caribbean	Grenada	Prevalence	Age-standardized	1292.4 (1213 to 1369.8)	1169.9 (1096.5 to 1245.4)	1469.7 (1373.5 to 1562.5)	1506.3 (1412.5 to 1596.7)	1452.5 (1359.6 to 1540.6)	1554.8 (1456.4 to 1657.9)	16.6 (12.6 to 20.5)	24.2 (18.5 to 29.8)	5.8 (0.9 to 11.1)
Latin America and Caribbean	Caribbean	Grenada	Deaths	All Ages	12 (11 to 14)	4 (4 to 6)	8 (7 to 9)	14 (12 to 16)	6 (4 to 7)	9 (7 to 10)	14.5 (-4.9 to 36.6)	27.9 (-7.8 to 68.2)	7.2 (-16.2 to 34.5)
Latin America and Caribbean	Caribbean	Grenada	Deaths	Age-standardized	15.2 (13.2 to 17.4)	8.7 (7.1 to 10.9)	25.5 (21.5 to 29.7)	14.4 (12.4 to 16.5)	9.9 (7.8 to 12)	22 (18.4 to 26)	-5.2 (-20.9 to 12.5)	13.5 (-16.8 to 47.4)	-14 (-32.1 to 8.1)
Latin America and Caribbean	Caribbean	Grenada	DALYs	All Ages	244 (216 to 277)	93 (78 to 109)	152 (130 to 177)	355 (312 to 404)	142 (116 to 168)	213 (181 to 250)	45.5 (23.8 to 69.4)	53.9 (23.4 to 86)	40.4 (12.6 to 71.5)
Latin America and Caribbean	Caribbean	Grenada	DALYs	Age-standardized	325.9 (288.1 to 370.6)	216.5 (182.8 to 255.6)	486.5 (415.6 to 569.3)	326.6 (287.8 to 371.8)	248.8 (203.3 to 293.4)	431 (368.8 to 499.7)	0.2 (-15.2 to 16.6)	14.9 (-7.1 to 39.1)	-11.4 (-28.4 to 7.9)
Latin America and Caribbean	Caribbean	Grenada	YLLs	All Ages	198 (172 to 227)	68 (55 to 84)	130 (109 to 155)	274 (235 to 318)	99 (78 to 122)	175 (144 to 209)	38.3 (13.1 to 67.2)	46.6 (7.2 to 92.4)	33.9 (4.1 to 70.4)
Latin America and Caribbean	Caribbean	Grenada	YLLs	Age-standardized	262.5 (228.1 to 301.9)	153.9 (125.1 to 188.7)	420.6 (351.1 to 498.7)	253.8 (217.7 to 293.3)	172.7 (135.3 to 211.9)	361.9 (301.9 to 429.6)	-3.3 (-20.8 to 17.3)	12.2 (-17.5 to 47.6)	-14 (-33.3 to 8.8)
Latin America and Caribbean	Caribbean	Grenada	YLDs	All Ages	46 (35 to 57)	25 (18 to 31)	21 (15 to 27)	81 (61 to 101)	43 (32 to 55)	38 (27 to 49)	76.8 (63.3 to 91.3)	74 (57 to 91.2)	80.2 (60.6 to 101.1)
Latin America and Caribbean	Caribbean	Grenada	YLDs	Age-standardized	63.4 (47.6 to 78.9)	62.6 (45.9 to 79.7)	65.9 (47.5 to 85.1)	72.8 (54.5 to 90.2)	76.1 (56.7 to 96.5)	69.1 (49.2 to 88.7)	14.8 (7 to 23.4)	21.6 (9.4 to 33.8)	4.7 (-4.9 to 14.8)
Latin America and Caribbean	Caribbean	Guyana	Incidence	All Ages	304 (285 to 324)	135 (125 to 144)	169 (159 to 180)	495 (468 to 526)	209 (196 to 222)	286 (270 to 304)	62.9 (54.6 to 71.2)	55.3 (45.8 to 65.5)	69 (58.7 to 78.3)
Latin America and Caribbean	Caribbean	Guyana	Incidence	Age-standardized	61.3 (58.3 to 64.2)	49 (46.3 to 51.6)	75.2 (71.6 to 78.8)	73.2 (69.5 to 77.3)	59.4 (56 to 62.8)	88.5 (84 to 93.5)	19.4 (15.8 to 23.1)	21.3 (16.6 to 26.3)	17.6 (12.8 to 22.3)
Latin America and Caribbean	Caribbean	Guyana	Prevalence	All Ages	5,026 (4,572 to 5,507)	2,422 (2,187 to 2,665)	2,604 (2,365 to 2,859)	8,896 (8,278 to 9,525)	4,094 (3,780 to 4,413)	4,802 (4,466 to 5,151)	77 (66.6 to 88.1)	69 (57.2 to 81.1)	84.4 (70.6 to 98.3)
Latin America and Caribbean	Caribbean	Guyana	Prevalence	Age-standardized	1009.6 (938.6 to 1083.9)	908.5 (838.1 to 981.3)	1126.9 (1046.4 to 1210.1)	1302.9 (1219.9 to 1389.5)	1139 (1056.3 to 1225)	1495.2 (1403.1 to 1597.8)	29.1 (23.2 to 34.8)	25.4 (18.4 to 32.8)	32.7 (25.2 to 40.2)
Latin America and Caribbean	Caribbean	Guyana	Deaths	All Ages	47 (39 to 55)	13 (11 to 16)	33 (27 to 41)	77 (60 to 97)	24 (18 to 31)	53 (40 to 67)	65.7 (25.8 to 116.4)	79.9 (29.2 to 148.7)	60 (16.8 to 115.2)
Latin America and Caribbean	Caribbean	Guyana	Deaths	Age-standardized	15.2 (12.9 to 17.7)	8.1 (6.5 to 10)	23.6 (19.6 to 28.4)	15.1 (11.8 to 18.6)	8.5 (6.3 to 11.3)	23.5 (18 to 29.4)	-0.8 (-24.1 to 27.4)	4.9 (-24 to 43.1)	-0.6 (-26.1 to 30.8)
Latin America and Caribbean	Caribbean	Guyana	DALYs	All Ages	1,189 (1,016 to 1,381)	398 (334 to 472)	791 (654 to 946)	2,085 (1,649 to 2,539)	713 (565 to 889)	1,372 (1,062 to 1,708)	75.3 (37 to 123.1)	78.9 (40.6 to 128.9)	73.5 (31.3 to 128.8)
Latin America and Caribbean	Caribbean	Guyana	DALYs	Age-standardized	312.3 (268.4 to 359.8)	189 (156.2 to 226.4)	452.4 (375.2 to 539.5)	336.6 (268.7 to 405.8)	213 (169.7 to 263.5)	484.9 (381.2 to 595.9)	7.8 (-15 to 35.7)	12.7 (-11.3 to 46.2)	7.2 (-18.5 to 39.8)
Latin America and Caribbean	Caribbean	Guyana	YLLs	All Ages	937 (777 to 1,114)	264 (208 to 327)	672 (540 to 830)	1,652 (1,247 to 2,104)	494 (359 to 665)	1,158 (866 to 1,489)	76.4 (30.1 to 136.3)	87 (29.9 to 168.5)	72.2 (22.5 to 138.7)
Latin America and Caribbean	Caribbean	Guyana	YLLs	Age-standardized	263.5 (220.2 to 312.8)	140.8 (111.8 to 173.8)	402.6 (327.3 to 489.6)	274.2 (209.2 to 344.6)	153.1 (112.1 to 203.1)	419.2 (314.7 to 531.5)	4.1 (-22.1 to 37.5)	8.7 (-23.2 to 53.8)	4.1 (-24.5 to 40.6)
Latin America and Caribbean	Caribbean	Guyana	YLDs	All Ages	253 (193 to 318)	134 (101 to 173)	118 (86 to 154)	433 (329 to 540)	219 (162 to 284)	214 (155 to 278)	71.4 (54.3 to 90.2)	62.9 (40.3 to 90)	80.9 (57.7 to 108.7)
Latin America and Caribbean	Caribbean	Guyana	YLDs	Age-standardized	48.7 (37.4 to 60.7)	48.2 (35.6 to 61.5)	49.8 (35.7 to 64.5)	62.4 (47.3 to 77.8)	60 (44.4 to 77)	65.6 (47.7 to 84.9)	28.1 (18.3 to 39.8)	24.5 (10 to 41.5)	31.9 (19.6 to 47.9)
Latin America and Caribbean	Caribbean	Haiti	Incidence	All Ages	3,835 (3,592 to 4,105)	1,910 (1,788 to 2,049)	1,925 (1,784 to 2,076)	9,971 (9,358 to 10,615)	4,953 (4,591 to 5,323)	5,017 (4,689 to 5,353)	160 (151 to 169.1)	159.4 (146.9 to 171.9)	160.6 (149.1 to 173.3)
Latin America and Caribbean	Caribbean	Haiti	Incidence	Age-standardized	102.8 (96.5 to 109.6)	94.9 (88.2 to 102.2)	112.4 (105.6 to 120.1)	128.1 (120 to 136.6)	117.7 (109.3 to 126.8)	139.9 (131.6 to 148.6)	24.7 (20.4 to 29.1)	24 (17.2 to 30.1)	24.4 (19.3 to 30.4)
Latin America and Caribbean	Caribbean	Haiti	Prevalence	All Ages	55,627 (51,579 to 59,542)	29,360 (27,282 to 31,414)	26,268 (23,952 to 28,469)	145,127 (134,824 to 154,924)	76,382 (70,825 to 81,695)	68,745 (63,518 to 74,003)	160.9 (150.1 to 170.3)	160.2 (147.7 to 172.2)	161.7 (148.5 to 175.2)
Latin America and Caribbean	Caribbean	Haiti	Prevalence	Age-standardized	1439.9 (1342.4 to 1543.2)	1439.4 (1339.7 to 1546.7)	1449.1 (1336 to 1562.9)	1810.2 (1689.9 to 1935.8)	1771 (1640.9 to 1901.9)	1854.5 (1723.7 to 1987.4)	25.7 (21.2 to 30.4)	23 (17.1 to 28.8)	28 (22.1 to 34.7)
Latin America and Caribbean	Caribbean	Haiti	Deaths	All Ages	1,089 (668 to 1,532)	389 (212 to 760)	700 (374 to 960)	2,013 (1,263 to 2,836)	758 (411 to 1,401)	1,255 (683 to 1,787)	84.8 (41.8 to 147.3)	94.7 (29.7 to 185.6)	79.3 (34.8 to 142.4)
Latin America and Caribbean	Caribbean	Haiti	Deaths	Age-standardized	42 (25.6 to 63.2)	28 (14.7 to 59.2)	60.6 (33.1 to 81.1)	37.4 (23.4 to 53.1)	25.9 (14.2 to 49.3)	50.2 (27.8 to 70)	-11 (-33.2 to 18.6)	-7.4 (-42.2 to 38)	-17.1 (-36.9 to 10.5)
Latin America and Caribbean	Caribbean	Haiti	DALYs	All Ages	29,691 (19,262 to 40,919)	11,186 (6,348 to 19,337)	18,505 (10,338 to 27,464)	52,104 (34,340 to 70,789)	21,525 (12,975 to 35,188)	30,579 (17,479 to 43,425)	75.5 (35.5 to 131.2)	92.4 (39 to 166.9)	65.2 (21.9 to 125)
Latin America and Caribbean	Caribbean	Haiti	DALYs	Age-standardized	867.3 (558.6 to 1186.9)	616.8 (364.9 to 1121)	1164.5 (655.1 to 1581)	754.5 (500.5 to 1035.9)	571.5 (345.4 to 956.7)	959 (554.2 to 1336.6)	-13 (-31.6 to 13.5)	-7.3 (-34 to 30.5)	-17.6 (-36.8 to 10.3)
Latin America and Caribbean	Caribbean	Haiti	YLLs	All Ages	26,893 (16,337 to 38,023)	9,585 (4,723 to 17,727)	17,308 (9,141 to 26,279)	44,927 (27,407 to 63,510)	17,442 (9,180 to 30,935)	27,485 (14,443 to 40,681)	67.1 (24.1 to 128.1)	82 (23.3 to 172.4)	58.8 (14.5 to 122.3)
Latin America and Caribbean	Caribbean	Haiti	YLLs	Age-standardized	797.2 (487.6 to 1111)	541.4 (295.6 to 1035.4)	1099.8 (589.4 to 1535.2)	668 (419.5 to 940.7)	480.7 (257.5 to 876.4)	877.4 (475.7 to 1255.2)	-16.2 (-36.1 to 12.2)	-11.2 (-39.8 to 31.8)	-20.2 (-40.3 to 9)
Latin America and Caribbean	Caribbean	Haiti	YLDs	All Ages	2,798 (2,127 to 3,459)	1,601 (1,211 to 2,020)	1,197 (872 to 1,555)	7,177 (5,378 to 8,878)	4,083 (3,043 to 5,206)	3,094 (2,212 to 3,998)	156.5 (135 to 178.7)	155 (127.4 to 184.1)	158.5 (130.4 to 191.6)
Latin America and Caribbean	Caribbean	Haiti	YLDs	Age-standardized	70.1 (53.3 to 86.4)	75.4 (55.9 to 95.7)	64.7 (46.7 to 82.5)	86.5 (64.5 to 107.7)	90.8 (67 to 115.9)	81.6 (59.3 to 106.8)	23.4 (14.5 to 31.9)	20.4 (9.6 to 31.9)	26.3 (14.6 to 39.7)
Latin America and Caribbean	Caribbean	Jamaica	Incidence	All Ages	1,818 (1,728 to 1,913)	652 (612 to 690)	1,166 (1,103 to 1,228)	3,331 (3,176 to 3,492)	1,004 (946 to 1,074)	2,327 (2,217 to 2,444)	83.3 (76.9 to 89.7)	54 (46.4 to 62.7)	99.7 (90.6 to 108.3)
Latin America and Caribbean	Caribbean	Jamaica	Incidence	Age-standardized	96 (91.2 to 101)	60.5 (57.3 to 63.8)	135.5 (128.2 to 142.8)	109.5 (104.4 to 114.8)	59.2 (55.7 to 63)	160.2 (152.7 to 168.5)	14.1 (10.3 to 17.7)	-2.2 (-6.2 to 2.3)	18.2 (13.1 to 23.2)
Latin America and Caribbean	Caribbean	Jamaica	Prevalence	All Ages	29,519 (27,777 to 31,240)	11,426 (10,605 to 12,161)	18,093 (16,958 to 19,211)	55,521 (52,686 to 58,296)	17,214 (15,904 to 18,530)	38,307 (36,252 to 40,388)	88.1 (80.8 to 95.6)	50.7 (42.7 to 58.4)	111.7 (100.9 to 123.5)
Latin America and Caribbean	Caribbean	Jamaica	Prevalence	Age-standardized	1595.5 (1503.1 to 1690.8)	1145.5 (1066 to 1219.4)	2118.5 (1991.5 to 2253)	1833.3 (1738.8 to 1927.2)	1066.8 (981.3 to 1149.3)	2649 (2510.3 to 2791.1)	14.9 (10.6 to 19.3)	-6.9 (-11.7 to -2.1)	25 (18.6 to 31.6)
Latin America and Caribbean	Caribbean	Jamaica	Deaths	All Ages	249 (222 to 286)	72 (58 to 102)	177 (158 to 198)	526 (405 to 662)	115 (82 to 188)	411 (315 to 515)	111.4 (64.1 to 166.7)	60.2 (14.6 to 113)	132.2 (75.7 to 201.7)
Latin America and Caribbean	Caribbean	Jamaica	Deaths	Age-standardized	13.6 (12.1 to 15.6)	7 (5.7 to 9.9)	21.8 (19.4 to 24.4)	16.9 (13 to 21.3)	6.2 (4.4 to 10.4)	29.5 (22.5 to 36.9)	24.7 (-3.7 to 58.1)	-11.6 (-37.4 to 20.1)	35.1 (2.2 to 75.8)
Latin America and Caribbean	Caribbean	Jamaica	DALYs	All Ages	5,934 (5,382 to 6,597)	1,887 (1,601 to 2,346)	4,047 (3,648 to 4,512)	11,641 (9,288 to 14,267)	2,606 (2,065 to 3,867)	9,034 (7,209 to 11,111)	96.2 (58.1 to 143)	38.1 (8 to 80)	123.2 (75.4 to 184.2)
Latin America and Caribbean	Caribbean	Jamaica	DALYs	Age-standardized	322.6 (291.8 to 359.4)	188 (159.5 to 236.8)	481.4 (432.6 to 537.5)	390.2 (311.5 to 478.4)	161.5 (127.2 to 242.2)	639.2 (509.7 to 788.3)	20.9 (-2.3 to 50.2)	-14.1 (-33.2 to 12.3)	32.8 (4.6 to 68.7)
Latin America and Caribbean	Caribbean	Jamaica	YLLs	All Ages	4,495 (4,054 to 5,046)	1,266 (1,039 to 1,701)	3,228 (2,913 to 3,627)	9,032 (6,845 to 11,576)	1,699 (1,183 to 2,961)	7,333 (5,577 to 9,379)	100.9 (51.2 to 162.7)	34.1 (-8.6 to 96.6)	127.2 (67.9 to 201.4)
Latin America and Caribbean	Caribbean	Jamaica	YLLs	Age-standardized	245.5 (221 to 276.7)	126.4 (103.8 to 171.4)	386.1 (347.3 to 435.7)	304 (230.1 to 395.1)	104.7 (72.3 to 186.4)	521.8 (398.8 to 664.1)	23.8 (-6.9 to 61.4)	-17.1 (-44.3 to 20.7)	35.1 (0.2 to 79.1)
Latin America and Caribbean	Caribbean	Jamaica	YLDs	All Ages	1,439 (1,094 to 1,786)	621 (460 to 784)	819 (592 to 1,043)	2,609 (1,954 to 3,256)	908 (675 to 1,153)	1,701 (1,208 to 2,200)	81.3 (68 to 94.5)	46.2 (29.5 to 63.1)	107.8 (90.4 to 126.7)
Latin America and Caribbean	Caribbean	Jamaica	YLDs	Age-standardized	77.1 (58 to 95.9)	61.7 (45.6 to 78.1)	95.3 (68.8 to 122.7)	86.2 (64.7 to 107.6)	56.8 (42 to 72.6)	117.4 (84.1 to 151.8)	11.8 (4.2 to 19.3)	-7.9 (-17.6 to 2.6)	23.2 (13.9 to 33.4)
Latin America and Caribbean	Caribbean	Puerto Rico	Incidence	All Ages	4,900 (4,690 to 5,110)	2,335 (2,210 to 2,466)	2,565 (2,436 to 2,695)	9,904 (9,353 to 10,477)	5,083 (4,742 to 5,429)	4,821 (4,545 to 5,138)	102.1 (93.8 to 110.5)	117.6 (104.5 to 132.3)	88 (77.5 to 98.3)
Latin America and Caribbean	Caribbean	Puerto Rico	Incidence	Age-standardized	137.1 (131.4 to 142.5)	121 (114.9 to 127.1)	155.9 (148.2 to 163.2)	150.1 (143.1 to 157.9)	133.2 (125 to 141.3)	170.2 (161.9 to 179.9)	9.5 (6 to 13.4)	10.1 (4.5 to 16.3)	9.2 (4.2 to 14.5)
Latin America and Caribbean	Caribbean	Puerto Rico	Prevalence	All Ages	69,216 (65,785 to 72,895)	32,924 (31,047 to 34,930)	36,292 (34,396 to 38,388)	141,664 (134,603 to 149,233)	71,026 (66,788 to 74,974)	70,638 (66,780 to 75,007)	104.7 (96.5 to 113.2)	115.7 (103.6 to 128.4)	94.6 (83.9 to 104.9)
Latin America and Caribbean	Caribbean	Puerto Rico	Prevalence	Age-standardized	1944.2 (1849.7 to 2045.3)	1719.7 (1620.6 to 1824.8)	2207.1 (2093.8 to 2331.7)	2170.6 (2061.2 to 2291)	1935.7 (1820.5 to 2048.7)	2462.3 (2325.5 to 2606.9)	11.6 (7.6 to 15.9)	12.6 (7.2 to 18.2)	11.6 (6.2 to 17.3)
Latin America and Caribbean	Caribbean	Puerto Rico	Deaths	All Ages	735 (650 to 808)	319 (244 to 371)	416 (375 to 461)	1,552 (1,182 to 1,978)	770 (514 to 1,001)	781 (595 to 1,000)	111.1 (66.1 to 166.1)	141.8 (82.9 to 214.1)	87.6 (45 to 147.5)
Latin America and Caribbean	Caribbean	Puerto Rico	Deaths	Age-standardized	21.6 (18.8 to 23.8)	17 (12.9 to 19.9)	27.2 (24.2 to 30.2)	17.9 (13.8 to 22.9)	14.7 (10.2 to 18.9)	22.4 (17.1 to 28.7)	-16.9 (-34.6 to 5.7)	-14 (-35.2 to 12)	-17.7 (-36.2 to 8.8)
Latin America and Caribbean	Caribbean	Puerto Rico	DALYs	All Ages	15,037 (13,685 to 16,317)	6,490 (5,611 to 7,307)	8,547 (7,725 to 9,387)	26,948 (22,083 to 32,776)	12,852 (10,101 to 15,761)	14,096 (11,268 to 17,574)	79.2 (47.6 to 117)	98 (62 to 142.5)	64.9 (32.9 to 106.7)
Latin America and Caribbean	Caribbean	Puerto Rico	DALYs	Age-standardized	419.3 (380.3 to 455.8)	335 (289.1 to 376.7)	519.3 (470.5 to 569.8)	375.7 (305.8 to 455.2)	315.5 (252.7 to 383.3)	452.5 (362 to 563.8)	-10.4 (-25.7 to 8.1)	-5.8 (-22.8 to 13.9)	-12.8 (-29.7 to 9.2)
Latin America and Caribbean	Caribbean	Puerto Rico	YLLs	All Ages	11,720 (10,649 to 12,785)	4,786 (3,963 to 5,474)	6,934 (6,271 to 7,648)	20,410 (15,686 to 26,208)	9,359 (6,825 to 11,974)	11,051 (8,392 to 14,325)	74.1 (35.2 to 123.1)	95.6 (46.4 to 158.1)	59.4 (22 to 112)
Latin America and Caribbean	Caribbean	Puerto Rico	YLLs	Age-standardized	326.2 (295.1 to 356)	245.9 (203.6 to 281.2)	421.5 (379.9 to 464.4)	272.6 (208.7 to 349.4)	215.8 (160.6 to 277.3)	344.7 (262.1 to 448.5)	-16.4 (-35.7 to 7.3)	-12.2 (-34.4 to 14.5)	-18.2 (-38.1 to 8.8)
Latin America and Caribbean	Caribbean	Puerto Rico	YLDs	All Ages	3,317 (2,477 to 4,109)	1,704 (1,251 to 2,159)	1,613 (1,166 to 2,102)	6,538 (4,875 to 8,193)	3,493 (2,476 to 4,489)	3,045 (2,194 to 3,977)	97.1 (82.9 to 110.8)	105 (84.7 to 123.7)	88.8 (70.6 to 107)
Latin America and Caribbean	Caribbean	Puerto Rico	YLDs	Age-standardized	93 (69.5 to 115.4)	89.1 (65.2 to 112.5)	97.8 (70.7 to 126.9)	103.1 (77.5 to 129.4)	99.7 (72.4 to 127.6)	107.8 (78.5 to 140.2)	10.8 (4.5 to 17.1)	11.9 (2.8 to 21.1)	10.2 (1.5 to 19.7)
Latin America and Caribbean	Caribbean	Saint Kitts and Nevis	Incidence	All Ages	31 (29 to 32)	15 (14 to 16)	16 (15 to 17)	73 (69 to 77)	34 (32 to 35)	39 (37 to 42)	136.5 (123.4 to 148.3)	126.4 (112 to 141)	145.9 (127.3 to 164)
Latin America and Caribbean	Caribbean	Saint Kitts and Nevis	Incidence	Age-standardized	87 (83.5 to 90.4)	78 (74.9 to 81.4)	99.4 (95.1 to 104.5)	112 (107.6 to 116.6)	100.4 (96.6 to 104.8)	125.6 (119.4 to 131.7)	28.8 (24.6 to 33.4)	28.7 (23.4 to 33.9)	26.3 (20.5 to 32.3)
Latin America and Caribbean	Caribbean	Saint Kitts and Nevis	Prevalence	All Ages	517 (489 to 549)	269 (253 to 285)	249 (233 to 267)	1,228 (1,157 to 1,299)	607 (571 to 647)	621 (578 to 666)	137.4 (126 to 149.9)	125.8 (113.2 to 139)	149.8 (133.3 to 169.2)
Latin America and Caribbean	Caribbean	Saint Kitts and Nevis	Prevalence	Age-standardized	1449.9 (1378.3 to 1531.9)	1399.5 (1323.2 to 1483.2)	1532.6 (1444.8 to 1640.6)	1854.2 (1753.9 to 1956.1)	1773.6 (1673.3 to 1882.7)	1967.9 (1843.4 to 2087.4)	27.9 (23 to 33.2)	26.7 (20.4 to 33.6)	28.4 (21.4 to 36.4)
Latin America and Caribbean	Caribbean	Saint Kitts and Nevis	Deaths	All Ages	6 (5 to 7)	3 (2 to 3)	3 (3 to 4)	8 (7 to 10)	3 (2 to 4)	5 (4 to 6)	33.1 (9.4 to 63.2)	17.4 (-10.6 to 58.4)	45.3 (11.6 to 88.3)
Latin America and Caribbean	Caribbean	Saint Kitts and Nevis	Deaths	Age-standardized	16.8 (14.7 to 19.3)	12.6 (10.3 to 15.3)	23.4 (19.7 to 27.9)	15.4 (13 to 18.1)	10.7 (8.2 to 13.5)	21.9 (18.3 to 26.1)	-8.3 (-24.2 to 12)	-15.2 (-35.1 to 13.7)	-6.6 (-26.5 to 18.5)
Latin America and Caribbean	Caribbean	Saint Kitts and Nevis	DALYs	All Ages	132 (118 to 149)	61 (51 to 71)	72 (62 to 85)	219 (186 to 258)	88 (72 to 107)	131 (108 to 159)	65.9 (39.1 to 96.5)	45.3 (16.3 to 84.1)	83.2 (43.5 to 130.9)
Latin America and Caribbean	Caribbean	Saint Kitts and Nevis	DALYs	Age-standardized	349.9 (312.9 to 395.1)	289.5 (243.1 to 337.7)	438.8 (376.6 to 513.3)	350.8 (302.3 to 407.4)	268.4 (221.5 to 322.9)	450.4 (378.7 to 531.8)	0.3 (-14.5 to 17.6)	-7.3 (-24.8 to 16.2)	2.6 (-18.2 to 26.7)
Latin America and Caribbean	Caribbean	Saint Kitts and Nevis	YLLs	All Ages	107 (94 to 123)	46 (37 to 57)	61 (51 to 73)	160 (132 to 197)	56 (42 to 74)	104 (83 to 130)	49.7 (19.3 to 87)	21.4 (-10.8 to 69.8)	71.4 (26 to 126.7)
Latin America and Caribbean	Caribbean	Saint Kitts and Nevis	YLLs	Age-standardized	279.4 (244.6 to 319.9)	215.8 (173 to 261.9)	370.9 (310.7 to 443.3)	262.4 (220.1 to 316.5)	176.4 (133.7 to 227.2)	364.6 (298.9 to 443.7)	-6.1 (-23.4 to 15.6)	-18.3 (-38.9 to 12.7)	-1.7 (-24.9 to 26.8)
Latin America and Caribbean	Caribbean	Saint Kitts and Nevis	YLDs	All Ages	25 (19 to 31)	14 (10 to 18)	11 (8 to 15)	59 (44 to 74)	32 (23 to 40)	27 (19 to 36)	134.6 (115.1 to 157)	124.4 (101.8 to 149.8)	147.5 (118.4 to 178.8)
Latin America and Caribbean	Caribbean	Saint Kitts and Nevis	YLDs	Age-standardized	70.5 (53.4 to 87.5)	73.7 (54.6 to 93.3)	67.9 (48.4 to 89.3)	88.4 (66.3 to 110.9)	92 (66.8 to 117.4)	85.8 (61.1 to 111.1)	25.4 (16.6 to 35.6)	24.8 (13.4 to 37.8)	26.4 (14.5 to 39.4)
Latin America and Caribbean	Caribbean	Saint Lucia	Incidence	All Ages	98 (94 to 103)	37 (35 to 39)	61 (58 to 64)	248 (235 to 260)	93 (88 to 99)	154 (146 to 163)	151.6 (141.9 to 161.6)	150.3 (136 to 165.8)	152.5 (140.9 to 165.2)
Latin America and Caribbean	Caribbean	Saint Lucia	Incidence	Age-standardized	102.2 (97.4 to 107)	68 (64.3 to 71.8)	147.8 (140.8 to 155.5)	119.3 (113.8 to 125.2)	85.7 (81.2 to 90.5)	156.7 (149.2 to 164.7)	16.8 (13.3 to 20.6)	25.9 (20.9 to 31.7)	6 (1.6 to 10.7)
Latin America and Caribbean	Caribbean	Saint Lucia	Prevalence	All Ages	1,515 (1,430 to 1,598)	633 (584 to 679)	883 (836 to 929)	4,124 (3,914 to 4,335)	1,606 (1,509 to 1,707)	2,518 (2,373 to 2,658)	172.1 (161.9 to 184.6)	153.9 (139.9 to 169.3)	185.2 (170.9 to 199.4)
Latin America and Caribbean	Caribbean	Saint Lucia	Prevalence	Age-standardized	1594.9 (1513.1 to 1682.9)	1199 (1116 to 1283.5)	2134 (2020.8 to 2247)	1950.3 (1855.4 to 2049.5)	1465.8 (1375.3 to 1558)	2496.4 (2361.7 to 2626.9)	22.3 (18.4 to 26.8)	22.3 (16.4 to 28.3)	17 (11.7 to 22.6)
Latin America and Caribbean	Caribbean	Saint Lucia	Deaths	All Ages	19 (17 to 22)	6 (5 to 7)	14 (12 to 16)	44 (36 to 53)	12 (9 to 15)	32 (26 to 39)	128.5 (85.6 to 181)	110 (57 to 175.6)	136.3 (85.9 to 196)
Latin America and Caribbean	Caribbean	Saint Lucia	Deaths	Age-standardized	24.6 (22 to 27.6)	12.4 (10.4 to 15)	45.7 (40.2 to 52.6)	21.9 (17.7 to 26)	10.5 (7.9 to 13.1)	37.8 (30.5 to 45)	-11 (-27.4 to 9.2)	-15.6 (-37 to 10.5)	-17.3 (-34.2 to 3.3)
Latin America and Caribbean	Caribbean	Saint Lucia	DALYs	All Ages	426 (386 to 474)	134 (115 to 156)	292 (258 to 331)	981 (819 to 1,144)	280 (225 to 333)	701 (563 to 836)	130.1 (92 to 174.3)	108.5 (70.2 to 156.5)	140 (92.9 to 194.5)
Latin America and Caribbean	Caribbean	Saint Lucia	DALYs	Age-standardized	483.9 (437.7 to 537.2)	268.8 (229.4 to 313.4)	800.1 (710.1 to 901.7)	465.5 (389.4 to 541.9)	250.5 (202.2 to 297.2)	723.3 (586.7 to 853.5)	-3.8 (-19.7 to 14.1)	-6.8 (-23.9 to 14.7)	-9.6 (-26.6 to 10.8)
Latin America and Caribbean	Caribbean	Saint Lucia	YLLs	All Ages	352 (316 to 392)	100 (83 to 122)	252 (220 to 287)	786 (637 to 946)	197 (150 to 248)	589 (469 to 724)	123.3 (77 to 176)	96.8 (47.3 to 160.2)	133.8 (79.8 to 195.9)
Latin America and Caribbean	Caribbean	Saint Lucia	YLLs	Age-standardized	407.4 (363.6 to 453)	205.3 (170.3 to 248.8)	705.1 (618.3 to 803)	373.2 (303.2 to 448.1)	174.1 (132.2 to 219.4)	613 (494.1 to 746.7)	-8.4 (-26.9 to 12.7)	-15.2 (-36.4 to 12)	-13.1 (-32.2 to 9.9)
Latin America and Caribbean	Caribbean	Saint Lucia	YLDs	All Ages	74 (56 to 92)	34 (26 to 44)	40 (29 to 52)	194 (146 to 243)	83 (62 to 106)	112 (81 to 145)	162.6 (142.5 to 183.6)	142.7 (115 to 172.5)	179.7 (153 to 204.7)
Latin America and Caribbean	Caribbean	Saint Lucia	YLDs	Age-standardized	76.5 (57.7 to 95.1)	63.5 (47.6 to 81.2)	94.9 (68.5 to 121.8)	92.2 (69.5 to 115)	76.4 (56.7 to 97.1)	110.3 (79.8 to 142.6)	20.5 (13.1 to 28.1)	20.2 (9.1 to 33)	16.2 (7.4 to 25)
Latin America and Caribbean	Caribbean	Saint Vincent and the Grenadines	Incidence	All Ages	43 (40 to 46)	18 (16 to 19)	26 (24 to 27)	81 (76 to 86)	25 (23 to 26)	56 (53 to 60)	86.7 (76.6 to 97)	39.1 (30.4 to 48.5)	119.7 (105.8 to 133.8)
Latin America and Caribbean	Caribbean	Saint Vincent and the Grenadines	Incidence	Age-standardized	51.8 (49.1 to 54.8)	37.9 (35.6 to 40.4)	68.9 (65.1 to 72.9)	64.2 (60.9 to 67.7)	41 (38.5 to 43.7)	86.6 (82.2 to 91.8)	23.8 (19.8 to 28.1)	8.1 (3.2 to 13.2)	25.8 (20.4 to 31.1)
Latin America and Caribbean	Caribbean	Saint Vincent and the Grenadines	Prevalence	All Ages	703 (637 to 770)	308 (275 to 340)	395 (358 to 434)	1,483 (1,386 to 1,589)	510 (465 to 558)	973 (910 to 1,041)	111.1 (98.7 to 123.6)	66 (54.9 to 77.8)	146.2 (128.1 to 164.8)
Latin America and Caribbean	Caribbean	Saint Vincent and the Grenadines	Prevalence	Age-standardized	871.8 (800.3 to 947.5)	704.3 (636.5 to 777)	1087.3 (998.9 to 1177.4)	1144.8 (1067.8 to 1225.6)	817.8 (743.5 to 895.5)	1461.6 (1366.1 to 1562.4)	31.3 (25.6 to 37.3)	16.1 (9.6 to 23.3)	34.4 (26.7 to 42.3)
Latin America and Caribbean	Caribbean	Saint Vincent and the Grenadines	Deaths	All Ages	6 (5 to 7)	2 (2 to 3)	4 (3 to 5)	14 (12 to 16)	3 (3 to 4)	11 (9 to 12)	123.8 (76.6 to 173.9)	49.5 (5.3 to 95.5)	165 (104.8 to 235.3)
Latin America and Caribbean	Caribbean	Saint Vincent and the Grenadines	Deaths	Age-standardized	9.3 (8.1 to 10.8)	5.7 (4.5 to 7.3)	15.9 (13.5 to 18.5)	11.4 (9.6 to 13.2)	5.3 (4 to 6.6)	18.1 (15.2 to 21)	21.5 (-3.3 to 47.2)	-5.9 (-34.3 to 22.9)	14.3 (-9.4 to 41.6)
Latin America and Caribbean	Caribbean	Saint Vincent and the Grenadines	DALYs	All Ages	138 (122 to 157)	51 (43 to 61)	87 (75 to 102)	316 (270 to 363)	82 (67 to 97)	234 (197 to 273)	128.7 (88.4 to 169.3)	60.8 (24.7 to 98.6)	168.1 (116.1 to 225.3)
Latin America and Caribbean	Caribbean	Saint Vincent and the Grenadines	DALYs	Age-standardized	186.5 (165.3 to 212.1)	120.9 (101.4 to 147.8)	282.3 (242.1 to 327.5)	241.4 (206.7 to 276.7)	128.2 (105.1 to 151.7)	355.1 (300.7 to 412.8)	29.5 (7.4 to 52.7)	6.1 (-17.9 to 30.3)	25.8 (1.7 to 52.9)
Latin America and Caribbean	Caribbean	Saint Vincent and the Grenadines	YLLs	All Ages	103 (89 to 120)	34 (28 to 44)	69 (57 to 83)	245 (204 to 289)	54 (42 to 68)	191 (157 to 227)	138.1 (86.4 to 193.9)	61.1 (14.1 to 114.5)	175.7 (112 to 253.8)
Latin America and Caribbean	Caribbean	Saint Vincent and the Grenadines	YLLs	Age-standardized	144 (125.2 to 168.3)	83 (67.5 to 108.6)	233.5 (196.6 to 275)	186.9 (155.8 to 218.9)	84.5 (64.6 to 104.9)	290.2 (239.3 to 342.5)	29.8 (2.3 to 60.4)	1.9 (-27.8 to 35.7)	24.3 (-3.9 to 59.3)
Latin America and Caribbean	Caribbean	Saint Vincent and the Grenadines	YLDs	All Ages	35 (26 to 44)	17 (12 to 22)	18 (13 to 24)	70 (53 to 88)	27 (20 to 35)	43 (31 to 56)	100.9 (79.3 to 124.2)	60.3 (38 to 87.9)	138.9 (106.8 to 171.7)
Latin America and Caribbean	Caribbean	Saint Vincent and the Grenadines	YLDs	Age-standardized	42.5 (32 to 53.5)	37.9 (28.3 to 48.8)	48.8 (35.5 to 63.7)	54.5 (41 to 67.8)	43.7 (32.3 to 56.6)	64.8 (46.3 to 83.8)	28.3 (17.1 to 40.2)	15.3 (0.1 to 32.8)	33 (19.9 to 47.8)
Latin America and Caribbean	Caribbean	Suriname	Incidence	All Ages	237 (226 to 248)	115 (110 to 121)	121 (115 to 128)	520 (492 to 548)	246 (232 to 261)	273 (258 to 289)	119.4 (112.3 to 127.7)	113.2 (103.8 to 123.4)	125.3 (115.1 to 136.2)
Latin America and Caribbean	Caribbean	Suriname	Incidence	Age-standardized	79.8 (76.2 to 83.7)	72.3 (68.8 to 75.9)	89.6 (85.1 to 94.2)	87.8 (83.6 to 92.3)	78.7 (74.4 to 83.1)	98.9 (93.8 to 104.5)	10 (7.1 to 13.6)	8.9 (4.7 to 13.9)	10.5 (6 to 15.4)
Latin America and Caribbean	Caribbean	Suriname	Prevalence	All Ages	3,807 (3,564 to 4,051)	2,016 (1,882 to 2,140)	1,791 (1,652 to 1,931)	8,972 (8,466 to 9,493)	4,591 (4,335 to 4,864)	4,381 (4,079 to 4,698)	135.7 (127.1 to 146.2)	127.7 (117.6 to 140.6)	144.7 (132.2 to 158.5)
Latin America and Caribbean	Caribbean	Suriname	Prevalence	Age-standardized	1298.5 (1221.9 to 1374.4)	1316.9 (1236.7 to 1394.8)	1296.9 (1211.1 to 1383.2)	1492.7 (1413.6 to 1574.9)	1440.7 (1362.9 to 1523)	1567.8 (1468.7 to 1671.7)	15 (11.4 to 19.1)	9.4 (4.8 to 14.9)	20.9 (15.6 to 26.7)
Latin America and Caribbean	Caribbean	Suriname	Deaths	All Ages	41 (37 to 48)	18 (15 to 23)	23 (20 to 27)	85 (70 to 101)	36 (27 to 44)	49 (40 to 60)	105.1 (67.6 to 148.9)	95.8 (43.1 to 146.6)	112.3 (68.2 to 170.4)
Latin America and Caribbean	Caribbean	Suriname	Deaths	Age-standardized	17.6 (15.7 to 20.5)	13.8 (11.7 to 17.6)	22.8 (19.8 to 26.2)	15.5 (12.8 to 18.4)	11.4 (8.7 to 14.1)	21.2 (17 to 25.7)	-12.1 (-28.2 to 6.4)	-17.8 (-38.1 to 3.5)	-7.3 (-26.3 to 17.7)
Latin America and Caribbean	Caribbean	Suriname	DALYs	All Ages	955 (856 to 1,074)	445 (381 to 518)	510 (451 to 580)	2,014 (1,705 to 2,363)	889 (724 to 1,059)	1,125 (929 to 1,352)	110.9 (79.5 to 148.9)	99.9 (65.8 to 138.2)	120.5 (79 to 170.4)
Latin America and Caribbean	Caribbean	Suriname	DALYs	Age-standardized	369.1 (329.5 to 413.2)	320.6 (274.3 to 374.7)	431.6 (380.5 to 489.5)	343.8 (291.8 to 402.7)	279.5 (228.4 to 330.9)	427.3 (355.1 to 511.1)	-6.8 (-20.6 to 10.3)	-12.8 (-27.8 to 3.7)	-1 (-19.2 to 22.3)
Latin America and Caribbean	Caribbean	Suriname	YLLs	All Ages	764 (679 to 875)	336 (282 to 412)	429 (373 to 491)	1,578 (1,294 to 1,913)	649 (495 to 808)	930 (747 to 1,155)	106.6 (68.1 to 155.3)	93.3 (48.6 to 144.7)	117 (70 to 177.7)
Latin America and Caribbean	Caribbean	Suriname	YLLs	Age-standardized	305.2 (271 to 345.8)	250.6 (209.1 to 305.8)	373.4 (325.4 to 429.2)	271.5 (223.4 to 327.7)	203.7 (155.4 to 253.2)	357.8 (286.9 to 442.6)	-11.1 (-27.6 to 9.4)	-18.7 (-37 to 2.6)	-4.2 (-24.2 to 22.9)
Latin America and Caribbean	Caribbean	Suriname	YLDs	All Ages	191 (146 to 239)	109 (82 to 139)	82 (59 to 106)	436 (324 to 540)	241 (179 to 307)	195 (141 to 255)	128.2 (110.9 to 147.8)	120.1 (97.9 to 145.9)	139 (112.4 to 167.7)
Latin America and Caribbean	Caribbean	Suriname	YLDs	Age-standardized	63.9 (48.7 to 80.2)	70.1 (52.2 to 89.4)	58.2 (42.1 to 75.5)	72.3 (54.1 to 89.4)	75.7 (56.5 to 96.4)	69.5 (50.5 to 90.3)	13.2 (5.9 to 21.7)	8.1 (-1.6 to 19.1)	19.4 (8.5 to 31.4)
Latin America and Caribbean	Caribbean	Trinidad and Tobago	Incidence	All Ages	690 (659 to 723)	242 (226 to 258)	448 (427 to 470)	1,080 (1,018 to 1,149)	340 (317 to 364)	740 (694 to 789)	56.4 (49.2 to 63.7)	40.2 (32 to 48.9)	65.2 (56.4 to 74.9)
Latin America and Caribbean	Caribbean	Trinidad and Tobago	Incidence	Age-standardized	73.6 (70.3 to 77)	49 (46.1 to 51.7)	101.3 (96.5 to 106.1)	65.4 (62 to 69)	40.6 (38 to 43.5)	91.2 (86.2 to 96.5)	-11.1 (-14 to -8)	-17.1 (-20.7 to -13.4)	-9.9 (-13.7 to -5.7)
Latin America and Caribbean	Caribbean	Trinidad and Tobago	Prevalence	All Ages	11,601 (10,918 to 12,263)	4,407 (3,992 to 4,816)	7,194 (6,813 to 7,571)	20,158 (18,971 to 21,443)	6,663 (6,026 to 7,315)	13,495 (12,707 to 14,379)	73.8 (66.6 to 80.8)	51.2 (41.5 to 60.5)	87.6 (78.9 to 97.4)
Latin America and Caribbean	Caribbean	Trinidad and Tobago	Prevalence	Age-standardized	1231.1 (1161.1 to 1297.1)	890.2 (817.1 to 964.7)	1617.6 (1536.2 to 1703.8)	1166.9 (1096.3 to 1240.3)	769 (691 to 847.4)	1594.4 (1505 to 1691)	-5.2 (-8.7 to -1.7)	-13.6 (-18.7 to -8.6)	-1.4 (-5.7 to 3.3)
Latin America and Caribbean	Caribbean	Trinidad and Tobago	Deaths	All Ages	111 (102 to 121)	32 (27 to 38)	79 (72 to 86)	182 (134 to 246)	51 (36 to 86)	131 (95 to 174)	63.8 (19.5 to 117.9)	60.3 (9.1 to 138.9)	65.3 (16.8 to 123.3)
Latin America and Caribbean	Caribbean	Trinidad and Tobago	Deaths	Age-standardized	15.2 (13.9 to 16.5)	8.2 (7 to 9.7)	24.8 (22.4 to 27)	10.4 (7.6 to 14)	5.3 (3.6 to 8.7)	16.6 (12.2 to 21.7)	-31.7 (-49.6 to -9.9)	-35.4 (-56.2 to -3)	-33 (-51.2 to -11.2)
Latin America and Caribbean	Caribbean	Trinidad and Tobago	DALYs	All Ages	2,713 (2,493 to 2,941)	800 (693 to 927)	1,913 (1,752 to 2,084)	4,268 (3,268 to 5,472)	1,153 (878 to 1,776)	3,115 (2,342 to 4,026)	57.3 (20.9 to 101.6)	44.1 (10.4 to 104.7)	62.8 (22.9 to 112)
Latin America and Caribbean	Caribbean	Trinidad and Tobago	DALYs	Age-standardized	318 (292.4 to 344.2)	177 (154.5 to 205.6)	486.4 (444.6 to 527.7)	239.2 (183.7 to 303.9)	124.9 (95.9 to 186.9)	366.3 (279.1 to 467.9)	-24.8 (-41.9 to -4.2)	-29.4 (-45.6 to -2.1)	-24.7 (-42.9 to -3)
Latin America and Caribbean	Caribbean	Trinidad and Tobago	YLLs	All Ages	2,144 (1,974 to 2,338)	559 (477 to 678)	1,585 (1,441 to 1,724)	3,317 (2,361 to 4,518)	802 (543 to 1,441)	2,515 (1,787 to 3,375)	54.7 (8.3 to 111.6)	43.4 (-2.6 to 126.2)	58.7 (9.5 to 120.2)
Latin America and Caribbean	Caribbean	Trinidad and Tobago	YLLs	Age-standardized	258.8 (238.6 to 281.3)	129.4 (110.6 to 157.3)	413.9 (375.9 to 449.2)	183.7 (131.7 to 249.4)	83.7 (57 to 147.8)	295.3 (212.7 to 393.2)	-29 (-49.9 to -3.8)	-35.3 (-55.9 to 1)	-28.7 (-50.1 to -2.9)
Latin America and Caribbean	Caribbean	Trinidad and Tobago	YLDs	All Ages	569 (427 to 706)	241 (180 to 309)	328 (238 to 421)	951 (723 to 1,187)	351 (258 to 450)	600 (433 to 778)	67.2 (52.3 to 83)	45.8 (25.5 to 67.9)	83 (63.6 to 104.2)
Latin America and Caribbean	Caribbean	Trinidad and Tobago	YLDs	Age-standardized	59.2 (44.5 to 73.3)	47.6 (35.3 to 61.1)	72.5 (52 to 93.8)	55.6 (42.1 to 68.9)	41.3 (30.6 to 53.2)	71 (52.1 to 92.1)	-6.1 (-13.8 to 1.8)	-13.2 (-24.2 to -0.5)	-2 (-10.7 to 8.3)
Latin America and Caribbean	Caribbean	United States Virgin Islands	Incidence	All Ages	58 (55 to 62)	26 (24 to 28)	32 (30 to 34)	132 (123 to 143)	54 (50 to 59)	78 (72 to 85)	128.3 (114.1 to 141.5)	106.7 (93.6 to 120.7)	145.9 (128.6 to 162.8)
Latin America and Caribbean	Caribbean	United States Virgin Islands	Incidence	Age-standardized	66.1 (62.5 to 69.8)	55.6 (52.3 to 58.8)	77.9 (73.4 to 82.8)	85.7 (80.8 to 91.2)	66.7 (62.6 to 71.3)	109.2 (102.3 to 116.8)	29.8 (25.5 to 34.3)	19.8 (15.1 to 25)	40.1 (34.2 to 46.8)
Latin America and Caribbean	Caribbean	United States Virgin Islands	Prevalence	All Ages	950 (866 to 1,037)	469 (425 to 518)	481 (437 to 528)	2,196 (2,046 to 2,362)	979 (909 to 1,061)	1,218 (1,128 to 1,312)	131.3 (116.7 to 146.9)	108.6 (94 to 124.6)	153.4 (135.1 to 172.5)
Latin America and Caribbean	Caribbean	United States Virgin Islands	Prevalence	Age-standardized	1051.9 (969.1 to 1137.4)	957.8 (876.4 to 1045.8)	1170.8 (1076.6 to 1269.9)	1376.1 (1279.6 to 1484.1)	1157.6 (1063.9 to 1263.7)	1664.7 (1545.6 to 1788.8)	30.8 (25.6 to 36.4)	20.9 (14.6 to 27.3)	42.2 (34.4 to 50.6)
Latin America and Caribbean	Caribbean	United States Virgin Islands	Deaths	All Ages	8 (7 to 10)	4 (3 to 5)	4 (3 to 5)	21 (17 to 24)	8 (6 to 10)	13 (10 to 15)	155 (95.2 to 219.9)	97.2 (44.7 to 164.6)	211.7 (114 to 316.9)
Latin America and Caribbean	Caribbean	United States Virgin Islands	Deaths	Age-standardized	12.1 (10 to 14.8)	10.5 (8.1 to 13.4)	14.9 (12.1 to 18.3)	11.7 (9.5 to 13.8)	7.9 (5.9 to 10)	17.3 (13.1 to 20.5)	-2.9 (-24.6 to 19.7)	-24.5 (-44.4 to 0.4)	16.3 (-18.9 to 51.7)
Latin America and Caribbean	Caribbean	United States Virgin Islands	DALYs	All Ages	201 (172 to 236)	100 (82 to 122)	101 (84 to 122)	463 (385 to 533)	178 (144 to 216)	286 (227 to 335)	130.8 (86.6 to 180.3)	77.8 (41.7 to 125.4)	183.5 (107.8 to 261.9)
Latin America and Caribbean	Caribbean	United States Virgin Islands	DALYs	Age-standardized	249.1 (213.3 to 295.3)	225.4 (183.4 to 275.4)	283.9 (237.8 to 341.6)	259.1 (216.7 to 295.7)	184.7 (148.2 to 222)	357.8 (286.9 to 415.2)	4 (-15.9 to 24.1)	-18.1 (-34.4 to 3.9)	26 (-6 to 57.9)
Latin America and Caribbean	Caribbean	United States Virgin Islands	YLLs	All Ages	154 (128 to 189)	75 (57 to 95)	79 (63 to 99)	361 (288 to 426)	128 (95 to 165)	233 (176 to 281)	134.9 (78.7 to 197.6)	71.6 (24.5 to 137)	194.6 (103.6 to 298.3)
Latin America and Caribbean	Caribbean	United States Virgin Islands	YLLs	Age-standardized	197.7 (165.2 to 241)	174.4 (135 to 223.4)	231.7 (186 to 287.1)	193.2 (153.9 to 226.7)	123.7 (92.2 to 158.1)	285 (216.2 to 338.9)	-2.3 (-24.9 to 22.8)	-29.1 (-47.5 to -2.5)	23 (-14.6 to 62.6)
Latin America and Caribbean	Caribbean	United States Virgin Islands	YLDs	All Ages	47 (36 to 59)	25 (19 to 33)	22 (16 to 28)	103 (76 to 130)	50 (36 to 64)	53 (38 to 70)	117.6 (93.6 to 143.4)	96 (70 to 126)	142.9 (110.8 to 178.7)
Latin America and Caribbean	Caribbean	United States Virgin Islands	YLDs	Age-standardized	51.4 (38.5 to 64.6)	51 (37.8 to 65.5)	52.2 (37.8 to 68.1)	65.9 (49.6 to 82.5)	61 (43.9 to 77.6)	72.8 (52.2 to 95.8)	28.2 (18.3 to 38.6)	19.5 (6.8 to 34.4)	39.6 (26.6 to 54.3)
		Andean Latin America	Incidence	All Ages	19,568 (18,680 to 20,411)	9,557 (9,101 to 10,012)	10,011 (9,581 to 10,439)	56,022 (53,293 to 59,107)	27,115 (25,609 to 28,752)	28,906 (27,521 to 30,528)	186.3 (179 to 194.4)	183.7 (174.4 to 193.3)	188.8 (180.1 to 198.2)
		Andean Latin America	Incidence	Age-standardized	79.6 (76 to 83.5)	74.7 (71 to 78.8)	85.1 (81.4 to 89)	99.1 (94.1 to 104.8)	91.6 (86.4 to 97.2)	107.4 (102 to 113.3)	24.5 (21.8 to 27.5)	22.7 (19 to 26.5)	26.1 (22.7 to 30)
		Andean Latin America	Prevalence	All Ages	300,258 (283,720 to 314,305)	150,843 (142,173 to 158,809)	149,414 (141,369 to 156,658)	801,856 (750,171 to 851,835)	401,413 (374,281 to 428,405)	400,442 (373,804 to 425,333)	167.1 (158 to 175.8)	166.1 (155.3 to 176.5)	168 (156.5 to 178.4)
		Andean Latin America	Prevalence	Age-standardized	1174.2 (1113.9 to 1232.7)	1148.9 (1086.4 to 1209.4)	1203.1 (1138.9 to 1263)	1382.6 (1297.9 to 1464.5)	1331.7 (1242.9 to 1420.2)	1440.7 (1351.2 to 1527.1)	17.8 (14.2 to 21.3)	15.9 (11.7 to 20.1)	19.7 (15.2 to 24.1)
		Andean Latin America	Deaths	All Ages	3,682 (3,196 to 4,181)	1,654 (1,316 to 2,058)	2,028 (1,744 to 2,352)	8,060 (6,395 to 9,930)	3,715 (2,696 to 4,756)	4,345 (3,442 to 5,366)	118.9 (76.7 to 168.6)	124.6 (63.2 to 192.7)	114.2 (65.4 to 169)
		Andean Latin America	Deaths	Age-standardized	20.5 (17.7 to 23.9)	17.8 (14.5 to 23.3)	23.5 (20.2 to 27.3)	15.4 (12.2 to 18.9)	13.2 (9.6 to 16.8)	17.9 (14.2 to 22.2)	-24.9 (-40 to -7)	-25.8 (-50.1 to -3.1)	-23.7 (-41.8 to -4.4)
		Andean Latin America	DALYs	All Ages	96,207 (80,469 to 109,755)	44,814 (33,030 to 53,589)	51,394 (42,936 to 61,984)	155,790 (130,465 to 184,251)	73,406 (58,437 to 89,412)	82,384 (67,771 to 99,916)	61.9 (34.5 to 95.7)	63.8 (30.5 to 114.2)	60.3 (26.9 to 99.4)
		Andean Latin America	DALYs	Age-standardized	408.2 (351.6 to 457.4)	361.3 (286.2 to 420.5)	459.5 (396.6 to 532.3)	281.9 (236.1 to 335.1)	252.3 (200.3 to 307.7)	315.2 (258.8 to 382.2)	-31 (-41.7 to -18.3)	-30.2 (-43.5 to -12.3)	-31.4 (-44.3 to -16.8)
		Andean Latin America	YLLs	All Ages	80,895 (64,900 to 94,157)	36,433 (24,647 to 44,917)	44,462 (36,388 to 54,348)	116,609 (93,616 to 144,387)	52,207 (38,875 to 68,032)	64,402 (51,345 to 80,788)	44.1 (12.5 to 83.7)	43.3 (6.8 to 101.8)	44.8 (8.5 to 88.4)
		Andean Latin America	YLLs	Age-standardized	350.2 (295.8 to 398.9)	300 (225.7 to 358.4)	404.9 (345.6 to 475)	214.9 (172.8 to 266.4)	182.5 (135.9 to 237.8)	251.1 (199.8 to 315)	-38.6 (-50.5 to -23.8)	-39.2 (-54.4 to -19.1)	-38 (-51.8 to -21.6)
		Andean Latin America	YLDs	All Ages	15,312 (11,702 to 18,879)	8,381 (6,362 to 10,510)	6,931 (5,150 to 8,892)	39,181 (29,648 to 48,702)	21,198 (15,619 to 26,893)	17,982 (13,091 to 23,060)	155.9 (138.1 to 173.8)	152.9 (129.9 to 174.7)	159.4 (134.8 to 186.5)
		Andean Latin America	YLDs	Age-standardized	58 (43.8 to 71.6)	61.4 (46.2 to 77.4)	54.6 (39.8 to 69.9)	66.9 (50.6 to 83.5)	69.8 (51.6 to 88.6)	64.2 (47 to 82.7)	15.4 (9.6 to 21.8)	13.7 (6.3 to 21.9)	17.5 (9 to 26.9)
Latin America and Caribbean	Andean Latin America	Bolivia (Plurinational State of)	Incidence	All Ages	4,681 (4,447 to 4,926)	2,487 (2,350 to 2,641)	2,195 (2,084 to 2,309)	12,548 (11,820 to 13,344)	6,262 (5,856 to 6,716)	6,286 (5,896 to 6,714)	168.1 (158.3 to 178.2)	151.8 (140.3 to 163.3)	186.4 (172.6 to 201.6)
Latin America and Caribbean	Andean Latin America	Bolivia (Plurinational State of)	Incidence	Age-standardized	131.3 (124.2 to 138.9)	130.9 (123 to 139.5)	132 (125.3 to 139.2)	145.7 (137.6 to 154.5)	138.3 (129.5 to 148)	154.6 (145.4 to 165.2)	11 (7 to 15.1)	5.6 (0.7 to 10.5)	17.1 (11.7 to 23.1)
Latin America and Caribbean	Andean Latin America	Bolivia (Plurinational State of)	Prevalence	All Ages	64,445 (61,397 to 67,352)	34,796 (33,116 to 36,679)	29,649 (28,191 to 31,019)	167,240 (157,051 to 178,097)	87,582 (82,091 to 93,631)	79,658 (74,122 to 85,347)	159.5 (147.9 to 171.7)	151.7 (138.9 to 164.6)	168.7 (151.6 to 185.7)
Latin America and Caribbean	Andean Latin America	Bolivia (Plurinational State of)	Prevalence	Age-standardized	1714.9 (1624.9 to 1810.1)	1765.8 (1664.9 to 1880.9)	1657.4 (1567.3 to 1745.7)	1864.8 (1753.7 to 1985.7)	1864.4 (1751.1 to 1994.4)	1874.5 (1755.1 to 2002.2)	8.7 (4.2 to 13.4)	5.6 (0.5 to 11)	13.1 (6.8 to 19.8)
Latin America and Caribbean	Andean Latin America	Bolivia (Plurinational State of)	Deaths	All Ages	1,112 (866 to 1,333)	537 (351 to 676)	576 (435 to 732)	2,431 (1,869 to 3,086)	1,193 (800 to 1,556)	1,238 (905 to 1,627)	118.5 (70.3 to 179.6)	122.3 (68 to 197.7)	115 (53.9 to 195.7)
Latin America and Caribbean	Andean Latin America	Bolivia (Plurinational State of)	Deaths	Age-standardized	43.8 (35.6 to 51.6)	37.9 (26.7 to 47)	52.1 (40.3 to 65.4)	35.9 (27.7 to 45.4)	32.6 (21.9 to 42.4)	39.9 (29.3 to 52.3)	-18.1 (-36.3 to 5)	-13.9 (-35.3 to 15.9)	-23.3 (-43.7 to 2.8)
Latin America and Caribbean	Andean Latin America	Bolivia (Plurinational State of)	DALYs	All Ages	29,249 (21,598 to 36,600)	14,494 (8,557 to 19,718)	14,755 (10,791 to 19,447)	47,595 (37,950 to 58,962)	23,769 (16,972 to 30,304)	23,826 (18,068 to 30,676)	62.7 (24.7 to 110)	64 (20.7 to 130.3)	61.5 (10.8 to 124.5)
Latin America and Caribbean	Andean Latin America	Bolivia (Plurinational State of)	DALYs	Age-standardized	820.1 (653 to 966.4)	742.7 (498.2 to 919.3)	920.7 (707 to 1153)	590.7 (472.2 to 724.9)	553.6 (393.7 to 697.2)	635.5 (479.8 to 818.3)	-28 (-42.4 to -10.5)	-25.5 (-41.6 to -4.1)	-31 (-48.5 to -8.7)
Latin America and Caribbean	Andean Latin America	Bolivia (Plurinational State of)	YLLs	All Ages	26,025 (18,249 to 33,261)	12,623 (6,791 to 17,904)	13,402 (9,413 to 18,136)	39,454 (30,066 to 50,437)	19,177 (12,678 to 25,531)	20,277 (14,824 to 27,152)	51.6 (10.9 to 103.8)	51.9 (7.1 to 127.2)	51.3 (-1.3 to 119.2)
Latin America and Caribbean	Andean Latin America	Bolivia (Plurinational State of)	YLLs	Age-standardized	737.4 (564.7 to 885.4)	651.7 (410.3 to 821.3)	847.3 (640 to 1080)	502 (384.6 to 639.4)	458.2 (309.5 to 600.7)	553.6 (409.7 to 729)	-31.9 (-47.7 to -12.8)	-29.7 (-47.2 to -5.3)	-34.7 (-53.4 to -10.7)
Latin America and Caribbean	Andean Latin America	Bolivia (Plurinational State of)	YLDs	All Ages	3,224 (2,432 to 3,997)	1,871 (1,382 to 2,367)	1,353 (982 to 1,753)	8,140 (6,153 to 10,048)	4,591 (3,410 to 5,880)	3,549 (2,571 to 4,506)	152.5 (130.7 to 174.5)	145.4 (119.6 to 172)	162.3 (130.5 to 197.8)
Latin America and Caribbean	Andean Latin America	Bolivia (Plurinational State of)	YLDs	Age-standardized	82.7 (61.8 to 102.3)	91 (67.1 to 115.6)	73.4 (52.6 to 94.3)	88.7 (67.3 to 110)	95.4 (70.5 to 122.7)	81.9 (59.4 to 104)	7.2 (0.1 to 14.8)	4.9 (-4.7 to 14.6)	11.5 (0.7 to 23.7)
Latin America and Caribbean	Andean Latin America	Ecuador	Incidence	All Ages	5,761 (5,509 to 5,996)	2,601 (2,471 to 2,734)	3,160 (3,026 to 3,294)	17,149 (16,279 to 18,135)	8,014 (7,527 to 8,538)	9,135 (8,643 to 9,700)	197.7 (186.9 to 210.5)	208.1 (191.8 to 225.5)	189.1 (174.4 to 204.5)
Latin America and Caribbean	Andean Latin America	Ecuador	Incidence	Age-standardized	95.7 (91.6 to 99.6)	82.3 (78.2 to 86.6)	109.3 (104.3 to 114.2)	119.4 (113.8 to 125.9)	105.5 (99.3 to 112.1)	135 (128.1 to 142.6)	24.8 (20.3 to 30.3)	28.1 (21.7 to 35.8)	23.5 (17.4 to 30)
Latin America and Caribbean	Andean Latin America	Ecuador	Prevalence	All Ages	81,756 (77,261 to 85,742)	39,284 (36,741 to 41,552)	42,472 (40,268 to 44,625)	217,229 (202,962 to 232,141)	105,175 (97,377 to 113,504)	112,054 (104,840 to 119,731)	165.7 (152.7 to 178.5)	167.7 (151.7 to 184.6)	163.8 (149.6 to 178)
Latin America and Caribbean	Andean Latin America	Ecuador	Prevalence	Age-standardized	1260.5 (1197.5 to 1316.7)	1171 (1105.7 to 1234.3)	1350 (1282.6 to 1414.8)	1445.1 (1358 to 1540.9)	1332.7 (1239.6 to 1432.9)	1577.2 (1485 to 1677.9)	14.6 (9.5 to 19.6)	13.8 (7.7 to 20.2)	16.8 (11 to 22.8)
Latin America and Caribbean	Andean Latin America	Ecuador	Deaths	All Ages	1,126 (929 to 1,232)	486 (371 to 556)	640 (541 to 702)	2,667 (2,084 to 3,351)	1,142 (806 to 1,449)	1,525 (1,164 to 1,932)	136.8 (88.4 to 201.4)	134.9 (78.6 to 211.7)	138.3 (85.3 to 208.2)
Latin America and Caribbean	Andean Latin America	Ecuador	Deaths	Age-standardized	26.3 (21.7 to 28.8)	23.4 (17.9 to 26.8)	29.6 (24.6 to 32.6)	22.3 (17.1 to 27.8)	17.7 (12 to 22.1)	28 (20.7 to 35.3)	-15.1 (-31.5 to 7.6)	-24.3 (-42 to -1.2)	-5.5 (-26.1 to 22.7)
Latin America and Caribbean	Andean Latin America	Ecuador	DALYs	All Ages	25,112 (20,699 to 27,730)	11,347 (8,538 to 13,315)	13,764 (11,843 to 15,284)	46,294 (37,666 to 55,603)	20,375 (16,545 to 24,674)	25,919 (20,728 to 31,725)	84.4 (50.7 to 130.9)	79.6 (42 to 132.6)	88.3 (51.9 to 135.6)
Latin America and Caribbean	Andean Latin America	Ecuador	DALYs	Age-standardized	443 (377.1 to 480.9)	388.5 (307.2 to 439)	502.4 (439.4 to 546.9)	334.5 (273.5 to 403)	276.2 (219.2 to 334)	403.4 (322.3 to 493.7)	-24.5 (-37.2 to -7.2)	-28.9 (-42.1 to -10.5)	-19.7 (-35 to 0.5)
Latin America and Caribbean	Andean Latin America	Ecuador	YLLs	All Ages	20,969 (16,848 to 23,474)	9,159 (6,495 to 10,923)	11,810 (10,147 to 13,271)	35,794 (28,172 to 45,193)	14,858 (11,248 to 18,997)	20,935 (16,228 to 26,796)	70.7 (32.5 to 125)	62.2 (19.5 to 125.2)	77.3 (35.9 to 133.5)
Latin America and Caribbean	Andean Latin America	Ecuador	YLLs	Age-standardized	381.6 (317.5 to 415.5)	326.3 (247.8 to 371.6)	441.8 (384.4 to 481.9)	265.9 (209.1 to 334.7)	207.4 (154.7 to 261.6)	334.4 (258.7 to 423.7)	-30.3 (-45 to -10.6)	-36.4 (-51.2 to -15.3)	-24.3 (-41.3 to -1.1)
Latin America and Caribbean	Andean Latin America	Ecuador	YLDs	All Ages	4,143 (3,141 to 5,118)	2,188 (1,615 to 2,771)	1,955 (1,451 to 2,521)	10,500 (7,896 to 13,214)	5,517 (4,028 to 7,115)	4,984 (3,634 to 6,414)	153.5 (129.5 to 179.7)	152.1 (119.2 to 185.8)	155 (120.7 to 190.7)
Latin America and Caribbean	Andean Latin America	Ecuador	YLDs	Age-standardized	61.4 (46.5 to 76.6)	62.3 (46 to 79.1)	60.5 (43.8 to 77.3)	68.7 (51.8 to 86.2)	68.8 (50 to 88.4)	69.1 (50.2 to 89)	11.8 (3.4 to 21.2)	10.4 (-1 to 22)	14.1 (2.8 to 26.5)
Latin America and Caribbean	Andean Latin America	Peru	Incidence	All Ages	9,126 (8,615 to 9,627)	4,470 (4,174 to 4,750)	4,656 (4,407 to 4,911)	26,325 (24,855 to 28,025)	12,839 (12,053 to 13,754)	13,486 (12,700 to 14,388)	188.5 (177.1 to 201.6)	187.2 (173 to 202.9)	189.6 (176.4 to 205.6)
Latin America and Caribbean	Andean Latin America	Peru	Incidence	Age-standardized	59.7 (56.4 to 63.3)	56.8 (53.3 to 60.8)	62.8 (59.4 to 66.4)	79.8 (75.2 to 85)	74.7 (70 to 80.1)	85.2 (79.8 to 91.1)	33.6 (29.3 to 38.4)	31.6 (26 to 37.6)	35.6 (29.7 to 42.6)
Latin America and Caribbean	Andean Latin America	Peru	Prevalence	All Ages	154,056 (143,144 to 163,783)	76,763 (70,366 to 82,219)	77,293 (71,767 to 82,474)	417,387 (388,012 to 446,187)	208,657 (192,259 to 223,826)	208,731 (193,342 to 223,779)	170.9 (158.1 to 183.3)	171.8 (155.7 to 188.4)	170.1 (155.5 to 184.9)
Latin America and Caribbean	Andean Latin America	Peru	Prevalence	Age-standardized	1003.5 (939.1 to 1063.7)	979.7 (904.8 to 1047.5)	1029.5 (960.1 to 1095)	1250.7 (1164.1 to 1336.2)	1208.1 (1116.8 to 1296.7)	1297.4 (1203.1 to 1391.5)	24.6 (19.4 to 29.5)	23.3 (16.8 to 29.8)	26 (20 to 32.3)
Latin America and Caribbean	Andean Latin America	Peru	Deaths	All Ages	1,443 (1,171 to 1,888)	631 (465 to 977)	812 (638 to 1,070)	2,962 (2,140 to 3,973)	1,380 (940 to 1,939)	1,583 (1,099 to 2,162)	105.2 (43.6 to 189.3)	118.7 (31.9 to 235.3)	94.8 (28.8 to 174.6)
Latin America and Caribbean	Andean Latin America	Peru	Deaths	Age-standardized	13.1 (10.6 to 18)	11.1 (8.1 to 18.4)	15.2 (12.1 to 21.1)	9 (6.5 to 12.2)	7.8 (5.2 to 11)	10.4 (7.2 to 14.3)	-31.2 (-52.8 to -1.9)	-30.2 (-60.7 to 7.3)	-31.9 (-55.1 to -2.9)

Chronic obstructive pulmonary disease

7 GBD super region	21 GBD region	Location	Measure	Age	1990 Both	1990 Female	1990 Male	2019 Both	2019 Female	2019 Male	Percent change (1990 to 2019) Both	Percent change (1990 to 2019) Female	Percent change (1990 to 2019) Male
Latin America and Caribbean	Andean Latin America	Peru	DALYs	All Ages	41,846 (34,009 to 50,651)	18,972 (14,213 to 24,329)	22,874 (17,996 to 28,937)	61,902 (49,366 to 77,238)	29,263 (22,639 to 37,246)	32,639 (24,951 to 41,881)	47.9 (12.1 to 97.3)	54.2 (10.7 to 121)	42.7 (2.4 to 99.8)
Latin America and Caribbean	Andean Latin America	Peru	DALYs	Age-standardized	289.8 (241.3 to 345.7)	250.9 (197.3 to 330)	331.4 (265.3 to 414.2)	189.6 (150.9 to 238)	171.3 (132.3 to 218.2)	209.7 (159.5 to 270)	-34.6 (-49.5 to -14.7)	-31.7 (-50.8 to -6.1)	-36.7 (-54 to -13.6)
Latin America and Caribbean	Andean Latin America	Peru	YLLs	All Ages	33,901 (26,138 to 42,413)	14,650 (9,973 to 19,833)	19,250 (14,490 to 25,199)	41,362 (29,746 to 56,035)	18,172 (12,422 to 25,691)	23,190 (16,047 to 32,252)	22 (-16.3 to 79.1)	24 (-22.8 to 107.4)	20.5 (-22.4 to 86.3)
Latin America and Caribbean	Andean Latin America	Peru	YLLs	Age-standardized	239.5 (192.6 to 294)	197.7 (146.2 to 276.9)	284 (217.4 to 367.2)	128.3 (92.1 to 174.4)	107.2 (73.2 to 152.5)	151.2 (104.6 to 210.9)	-46.4 (-62.6 to -23.3)	-45.8 (-66.6 to -14.9)	-46.8 (-65 to -21.2)
Latin America and Caribbean	Andean Latin America	Peru	YLDs	All Ages	7,946 (6,036 to 9,892)	4,322 (3,263 to 5,515)	3,624 (2,608 to 4,723)	20,540 (15,483 to 25,631)	11,091 (8,179 to 14,188)	9,449 (6,820 to 12,400)	158.5 (133.3 to 186.3)	156.6 (123.7 to 191.6)	160.8 (126.3 to 199.8)
Latin America and Caribbean	Andean Latin America	Peru	YLDs	Age-standardized	50.3 (38.2 to 62.1)	53.2 (39.8 to 67.9)	47.4 (34.5 to 61.7)	61.3 (46.3 to 76.6)	64.1 (47.4 to 82)	58.5 (42.5 to 76.8)	21.8 (12 to 31.7)	20.5 (7.7 to 34.6)	23.4 (9.7 to 38.5)
		Central Latin America	Incidence	All Ages	134,960 (127,214 to 143,165)	64,908 (61,040 to 69,013)	70,052 (66,233 to 74,165)	388,074 (364,835 to 412,716)	196,685 (185,039 to 209,184)	191,389 (179,945 to 203,766)	187.5 (181.5 to 194.2)	203 (195.1 to 212.3)	173.2 (167.3 to 178.9)
		Central Latin America	Incidence	Age-standardized	146.4 (137.1 to 156)	133.8 (124.8 to 143.2)	160.3 (150.7 to 170.6)	166.6 (156.2 to 177.2)	156.4 (147 to 166.3)	178.1 (167.2 to 189.5)	13.8 (11.9 to 15.8)	17 (14.2 to 20)	11.1 (9.4 to 13)
		Central Latin America	Prevalence	All Ages	1,857,119 (1,739,756 to 1,969,452)	927,151 (867,299 to 989,948)	929,968 (870,698 to 986,786)	5,157,823 (4,847,688 to 5,502,692)	2,616,977 (2,451,736 to 2,800,900)	2,540,845 (2,391,120 to 2,712,980)	177.7 (172.2 to 184.3)	182.3 (175.1 to 190.4)	173.2 (167.1 to 179.8)
		Central Latin America	Prevalence	Age-standardized	1983.2 (1863.9 to 2118.5)	1883.9 (1767.7 to 2017.4)	2094.7 (1967.1 to 2235.1)	2186.5 (2052.6 to 2332.4)	2056.1 (1926.9 to 2203.3)	2339.9 (2201.8 to 2504.2)	10.3 (8.3 to 12.3)	9.1 (6.6 to 12)	11.7 (9.8 to 13.7)
		Central Latin America	Deaths	All Ages	22,412 (20,220 to 23,491)	9,598 (7,736 to 10,220)	12,814 (11,906 to 13,370)	64,353 (54,767 to 74,217)	30,281 (23,953 to 35,890)	34,072 (28,830 to 39,822)	187.1 (152.6 to 226.7)	215.5 (166.2 to 262)	165.9 (130.7 to 208.5)
		Central Latin America	Deaths	Age-standardized	33.4 (30 to 35.2)	27.4 (22.2 to 29.4)	40.1 (36.6 to 42.1)	29.2 (24.9 to 33.7)	24.7 (19.5 to 29.3)	34.9 (29.5 to 40.7)	-12.6 (-22.9 to -0.6)	-9.9 (-23.3 to 3.2)	-13.1 (-24.3 to 0.4)
		Central Latin America	DALYs	All Ages	503,240 (464,527 to 531,934)	224,362 (189,326 to 241,294)	278,877 (263,948 to 293,710)	1,214,743 (1,059,509 to 1,391,028)	570,301 (483,938 to 661,455)	644,442 (556,265 to 743,760)	141.4 (115.4 to 171.5)	154.2 (120.7 to 191.7)	131.1 (101.5 to 164.2)
		Central Latin America	DALYs	Age-standardized	614.7 (567.3 to 649.5)	518.3 (438 to 556.4)	720.9 (677.3 to 757.8)	530 (461.5 to 605.6)	456.3 (387.4 to 528.2)	618.4 (534.5 to 713.8)	-13.8 (-22.9 to -3.1)	-12 (-23.3 to 0.5)	-14.2 (-25 to -1.8)
		Central Latin America	YLLs	All Ages	410,507 (373,027 to 426,883)	173,445 (138,711 to 184,032)	237,061 (225,135 to 246,066)	970,224 (831,584 to 1,129,878)	435,747 (355,367 to 520,624)	534,477 (454,115 to 630,856)	136.3 (104 to 172.9)	151.2 (108.2 to 200.6)	125.5 (92 to 164.8)
		Central Latin America	YLLs	Age-standardized	520.1 (471.4 to 542.5)	420.1 (335.9 to 445.1)	630 (591.9 to 655.1)	427.2 (366.9 to 496.7)	351.2 (286.1 to 419.5)	518.2 (441.6 to 610.5)	-17.9 (-28.6 to -5.4)	-16.4 (-30.5 to -1.1)	-17.7 (-29.9 to -3.7)
		Central Latin America	YLDs	All Ages	92,733 (70,733 to 112,824)	50,917 (39,828 to 62,096)	41,816 (30,076 to 52,975)	244,519 (186,940 to 297,832)	134,554 (101,198 to 166,689)	109,965 (78,538 to 140,676)	163.7 (149.9 to 175.5)	164.3 (146.5 to 179.3)	163 (147.2 to 177.6)
		Central Latin America	YLDs	Age-standardized	94.6 (72.2 to 115.3)	98.2 (74.9 to 120.7)	90.9 (64.7 to 115.4)	102.7 (78.4 to 125.4)	105.1 (79.1 to 129.8)	100.2 (71.4 to 128)	8.6 (5.7 to 11.5)	7 (3.1 to 10.9)	10.2 (6.8 to 13.4)
Latin America and Caribbean	Central Latin America	Colombia	Incidence	All Ages	30,863 (29,770 to 32,103)	14,441 (13,784 to 15,078)	16,421 (15,760 to 17,214)	101,214 (96,869 to 105,293)	51,431 (48,939 to 53,712)	49,784 (47,325 to 52,120)	228 (216.7 to 240.2)	256.1 (238.1 to 275.4)	203.2 (187.3 to 217.5)
Latin America and Caribbean	Central Latin America	Colombia	Incidence	Age-standardized	158 (152.5 to 164)	141.6 (135.1 to 148.1)	175.4 (168.2 to 184.1)	191.3 (182.9 to 199.6)	176.4 (167.4 to 184.8)	208.2 (197.4 to 218.3)	21.1 (17.1 to 25.3)	24.6 (18.5 to 31.5)	18.7 (13.3 to 24.6)
Latin America and Caribbean	Central Latin America	Colombia	Prevalence	All Ages	436,335 (415,771 to 458,922)	212,679 (200,244 to 225,028)	223,656 (212,551 to 236,518)	1,319,481 (1,252,085 to 1,391,749)	655,360 (616,581 to 701,154)	664,121 (627,840 to 706,098)	202.4 (189.6 to 216.4)	208.1 (191.3 to 227)	196.9 (181.4 to 213.4)
Latin America and Caribbean	Central Latin America	Colombia	Prevalence	Age-standardized	2227 (2128.7 to 2343.2)	2082.1 (1969.5 to 2194.4)	2387.1 (2271.7 to 2527.6)	2467.5 (2338.5 to 2605.1)	2230.8 (2100.1 to 2382.5)	2748.2 (2594.9 to 2920.7)	10.8 (6.6 to 15.6)	7.1 (1.2 to 13.3)	15.1 (9.4 to 21.4)
Latin America and Caribbean	Central Latin America	Colombia	Deaths	All Ages	5,198 (4,466 to 5,580)	2,226 (1,643 to 2,465)	2,972 (2,698 to 3,212)	16,348 (12,052 to 20,722)	7,974 (5,238 to 10,328)	8,374 (6,188 to 10,754)	214.5 (141.7 to 289.9)	258.3 (168.7 to 358.1)	181.7 (113.7 to 261.2)
Latin America and Caribbean	Central Latin America	Colombia	Deaths	Age-standardized	36.3 (31.4 to 39.2)	29.9 (22.5 to 33.3)	43.3 (38.7 to 47.2)	29.2 (21.6 to 37)	25 (16.6 to 32.2)	34.6 (25.5 to 44.6)	-19.4 (-37.3 to 0.1)	-16.4 (-36.7 to 7.2)	-20.2 (-39.6 to 1.9)
Latin America and Caribbean	Central Latin America	Colombia	DALYs	All Ages	119,442 (104,166 to 128,460)	52,361 (39,859 to 57,914)	67,081 (61,642 to 72,212)	284,836 (227,823 to 347,276)	135,742 (102,655 to 167,048)	149,094 (118,454 to 184,847)	138.5 (93.8 to 189.5)	159.2 (108.3 to 220.8)	122.3 (78.1 to 177.6)
Latin America and Caribbean	Central Latin America	Colombia	DALYs	Age-standardized	707.8 (614.9 to 760.4)	595.3 (451.2 to 658.6)	829.6 (760.8 to 894.1)	532.7 (425.1 to 649.4)	457 (348.9 to 560.9)	625.3 (496 to 776.2)	-24.7 (-38.4 to -8.8)	-23.2 (-37.9 to -4.9)	-24.6 (-39.6 to -5.9)
Latin America and Caribbean	Central Latin America	Colombia	YLLs	All Ages	98,477 (83,469 to 105,331)	41,117 (28,925 to 45,371)	57,360 (52,838 to 61,505)	225,837 (171,067 to 287,238)	104,081 (73,801 to 135,162)	121,756 (91,354 to 157,420)	129.3 (76.7 to 190.4)	153.1 (89.2 to 229.7)	112.3 (61.4 to 177.5)
Latin America and Caribbean	Central Latin America	Colombia	YLLs	Age-standardized	605.5 (513.5 to 649.8)	490.5 (349.3 to 541)	729.6 (667 to 785.3)	421.8 (319.5 to 537.8)	347.8 (249.5 to 450.1)	512.3 (383.2 to 662.7)	-30.3 (-46.2 to -11.5)	-29.1 (-46.7 to -8)	-29.8 (-46.7 to -8.2)
Latin America and Caribbean	Central Latin America	Colombia	YLDs	All Ages	20,965 (15,890 to 25,935)	11,244 (8,357 to 13,936)	9,721 (6,804 to 12,699)	58,999 (44,226 to 73,235)	31,661 (22,919 to 40,301)	27,339 (18,924 to 36,317)	181.4 (155.2 to 207.3)	181.6 (147.2 to 216.5)	181.2 (149.9 to 218.7)
Latin America and Caribbean	Central Latin America	Colombia	YLDs	Age-standardized	102.3 (77.5 to 127.6)	104.7 (77.5 to 131.2)	100 (68.8 to 131.3)	110.9 (83.1 to 137.6)	109.2 (79.6 to 138.4)	113 (78.4 to 150.4)	8.4 (1.1 to 15.2)	4.2 (-4.8 to 13.9)	13.1 (4 to 22.8)
Latin America and Caribbean	Central Latin America	Costa Rica	Incidence	All Ages	3,028 (2,882 to 3,175)	1,468 (1,387 to 1,548)	1,560 (1,479 to 1,641)	8,504 (8,157 to 8,850)	4,055 (3,856 to 4,265)	4,450 (4,254 to 4,652)	180.8 (170.8 to 192)	176.2 (163 to 190.8)	185.2 (170.6 to 199.7)
Latin America and Caribbean	Central Latin America	Costa Rica	Incidence	Age-standardized	164.8 (157.6 to 172.4)	153.9 (145.8 to 162.5)	176.5 (168 to 185.1)	170 (163.1 to 176.8)	148.9 (141.3 to 156.8)	194.4 (185.9 to 202.8)	3.2 (-0.5 to 7.1)	-3.2 (-7.9 to 1.8)	10.1 (4.9 to 15.4)
Latin America and Caribbean	Central Latin America	Costa Rica	Prevalence	All Ages	37,975 (35,402 to 40,545)	18,721 (17,358 to 20,095)	19,253 (17,919 to 20,719)	106,907 (101,248 to 113,958)	51,406 (48,198 to 55,119)	55,501 (52,433 to 59,172)	181.5 (171 to 193.8)	174.6 (160.2 to 190.8)	188.3 (171.4 to 205)
Latin America and Caribbean	Central Latin America	Costa Rica	Prevalence	Age-standardized	2017.2 (1902.4 to 2139.2)	1913.6 (1790.4 to 2048.8)	2129.8 (2003 to 2271.8)	2092.9 (1981.3 to 2230.5)	1841.3 (1725.1 to 1977)	2392.2 (2259 to 2547.6)	3.8 (0.3 to 7.5)	-3.8 (-8.5 to 1.3)	12.3 (6.5 to 18.2)
Latin America and Caribbean	Central Latin America	Costa Rica	Deaths	All Ages	389 (337 to 433)	181 (148 to 212)	208 (182 to 232)	1,204 (908 to 1,515)	572 (388 to 722)	631 (483 to 795)	209.6 (147.5 to 287.9)	215.7 (141.7 to 305.5)	204.2 (135.5 to 283.6)
Latin America and Caribbean	Central Latin America	Costa Rica	Deaths	Age-standardized	24.9 (21.5 to 27.8)	22.2 (18.1 to 26)	27.8 (24.1 to 31.2)	23.2 (17.5 to 29.2)	19.2 (13.2 to 24.3)	28.3 (21.7 to 35.6)	-6.6 (-25.2 to 17.4)	-13.7 (-33.8 to 11.1)	1.9 (-21 to 28.2)
Latin America and Caribbean	Central Latin America	Costa Rica	DALYs	All Ages	7,902 (7,103 to 8,674)	3,713 (3,156 to 4,217)	4,189 (3,745 to 4,661)	20,162 (16,364 to 24,296)	9,247 (7,298 to 11,220)	10,915 (8,763 to 13,448)	155.1 (111.2 to 208.1)	149 (103.9 to 206.1)	160.6 (109.7 to 222.2)
Latin America and Caribbean	Central Latin America	Costa Rica	DALYs	Age-standardized	457 (409.1 to 500)	411.6 (348.8 to 467.1)	505.9 (449.9 to 562)	398.3 (322.3 to 480.9)	330 (261.9 to 400.7)	480.4 (385.7 to 590.3)	-12.8 (-27.6 to 5.1)	-19.8 (-33.8 to -1.3)	-5 (-23.5 to 17.2)
Latin America and Caribbean	Central Latin America	Costa Rica	YLLs	All Ages	6,132 (5,473 to 6,762)	2,759 (2,270 to 3,196)	3,373 (3,031 to 3,754)	15,411 (11,820 to 19,435)	6,758 (4,871 to 8,687)	8,653 (6,575 to 11,037)	151.3 (95.6 to 220.5)	144.9 (86.2 to 220.8)	156.5 (94.7 to 232.9)
Latin America and Caribbean	Central Latin America	Costa Rica	YLLs	Age-standardized	366.4 (325.7 to 404.9)	318 (262.2 to 370.2)	418.4 (374.9 to 467.1)	305.3 (233.2 to 384.6)	240.2 (173.7 to 309.2)	383.4 (290.6 to 485.4)	-16.7 (-35.2 to 5.9)	-24.5 (-42.7 to -1.4)	-8.4 (-30.1 to 18.6)
Latin America and Caribbean	Central Latin America	Costa Rica	YLDs	All Ages	1,770 (1,355 to 2,195)	954 (705 to 1,194)	816 (581 to 1,066)	4,751 (3,574 to 5,914)	2,489 (1,811 to 3,148)	2,262 (1,576 to 2,998)	168.4 (147 to 189.2)	160.8 (134.3 to 188.2)	177.2 (148.9 to 205.2)
Latin America and Caribbean	Central Latin America	Costa Rica	YLDs	Age-standardized	90.5 (67.4 to 112.7)	93.6 (68.8 to 118.6)	87.5 (61 to 116.1)	93 (69.6 to 115.9)	89.8 (65.3 to 113.5)	97 (67.5 to 128.8)	2.8 (-3.4 to 8.8)	-4 (-12.3 to 4.7)	10.9 (2.1 to 19.3)
Latin America and Caribbean	Central Latin America	El Salvador	Incidence	All Ages	2,922 (2,768 to 3,085)	1,527 (1,439 to 1,625)	1,395 (1,318 to 1,474)	7,596 (7,201 to 8,045)	4,219 (3,968 to 4,497)	3,377 (3,185 to 3,574)	159.9 (149.9 to 171)	176.3 (161.8 to 191.3)	142 (130.2 to 154.5)
Latin America and Caribbean	Central Latin America	El Salvador	Incidence	Age-standardized	85 (80.8 to 89.5)	82.3 (77.7 to 87.3)	88.1 (83.6 to 92.9)	120.8 (114.4 to 128)	116.9 (110 to 124.9)	126.1 (118.7 to 133.7)	42.1 (37.2 to 47)	42 (34.9 to 48.4)	43.1 (36.5 to 49.8)
Latin America and Caribbean	Central Latin America	El Salvador	Prevalence	All Ages	41,545 (38,497 to 44,518)	22,052 (20,236 to 23,740)	19,493 (17,954 to 20,872)	101,516 (95,246 to 108,231)	57,954 (53,955 to 62,312)	43,561 (40,708 to 46,500)	144.4 (133.9 to 155.9)	162.8 (149 to 179.9)	123.5 (111.5 to 136.3)
Latin America and Caribbean	Central Latin America	El Salvador	Prevalence	Age-standardized	1192.2 (1113.1 to 1270.8)	1187.3 (1100.2 to 1277.1)	1199.1 (1113.6 to 1281.1)	1630.9 (1529 to 1741.2)	1624.3 (1511.9 to 1748.4)	1641.3 (1532.6 to 1749.7)	36.8 (32.4 to 41.8)	36.8 (30.9 to 43.7)	36.9 (30.6 to 43.5)
Latin America and Caribbean	Central Latin America	El Salvador	Deaths	All Ages	603 (515 to 690)	299 (241 to 365)	304 (258 to 346)	1,200 (876 to 1,481)	657 (448 to 862)	543 (403 to 683)	99.1 (49.7 to 148.4)	119.8 (49.7 to 191.1)	78.7 (37.7 to 127.8)
Latin America and Caribbean	Central Latin America	El Salvador	Deaths	Age-standardized	21.8 (18.7 to 25.1)	19.5 (15.7 to 23.8)	24.6 (20.9 to 28)	18.1 (13.5 to 22.5)	16.6 (11.3 to 21.7)	20.2 (14.9 to 25.5)	-16.9 (-38 to 4.4)	-15 (-42.7 to 12.2)	-17.6 (-36.6 to 5.5)
Latin America and Caribbean	Central Latin America	El Salvador	DALYs	All Ages	13,156 (11,646 to 14,640)	6,555 (5,418 to 7,708)	6,601 (5,818 to 7,483)	20,881 (16,632 to 25,301)	11,337 (8,706 to 14,136)	9,544 (7,475 to 11,756)	58.7 (26.9 to 91.8)	73 (32.2 to 118)	44.6 (14.1 to 81.1)
Latin America and Caribbean	Central Latin America	El Salvador	DALYs	Age-standardized	419.9 (369.6 to 469.7)	384 (321.2 to 448.3)	462.7 (405.7 to 525.9)	336.9 (266.9 to 410)	315.3 (243.3 to 396.8)	366.4 (286.8 to 453.1)	-19.8 (-36.1 to -2.2)	-17.9 (-37.2 to 3.6)	-20.8 (-37.7 to -0.4)
Latin America and Caribbean	Central Latin America	El Salvador	YLLs	All Ages	11,110 (9,571 to 12,538)	5,369 (4,233 to 6,413)	5,741 (5,013 to 6,615)	16,194 (12,118 to 20,520)	8,463 (6,018 to 11,174)	7,731 (5,739 to 9,829)	45.8 (10.3 to 85.7)	57.6 (9.3 to 113.3)	34.7 (0.2 to 75.9)
Latin America and Caribbean	Central Latin America	El Salvador	YLLs	Age-standardized	363.2 (315.7 to 412.5)	322.5 (262.2 to 381.9)	411.6 (355.9 to 473.5)	261.4 (196.6 to 333.1)	234 (164.8 to 309.2)	298.4 (220.5 to 381.2)	-28 (-46.3 to -8.1)	-27.4 (-49.5 to -2.6)	-27.5 (-46.3 to -4.9)
Latin America and Caribbean	Central Latin America	El Salvador	YLDs	All Ages	2,046 (1,560 to 2,548)	1,186 (908 to 1,496)	860 (614 to 1,110)	4,687 (3,564 to 5,814)	2,874 (2,130 to 3,674)	1,813 (1,290 to 2,368)	129.1 (106.6 to 153.2)	142.3 (112.2 to 173.9)	110.9 (81.8 to 142.4)
Latin America and Caribbean	Central Latin America	El Salvador	YLDs	Age-standardized	56.6 (43.2 to 70.4)	61.5 (46.8 to 77.5)	51.2 (36.5 to 66.5)	75.5 (57.3 to 93.8)	81.3 (60 to 104.5)	68.1 (48 to 89.3)	33.4 (23.7 to 43.5)	32.2 (19.4 to 45.8)	33 (19.3 to 47.6)
Latin America and Caribbean	Central Latin America	Guatemala	Incidence	All Ages	3,182 (3,040 to 3,324)	1,482 (1,398 to 1,564)	1,699 (1,626 to 1,778)	10,941 (10,369 to 11,544)	5,293 (4,996 to 5,639)	5,648 (5,322 to 6,004)	243.9 (229.5 to 257.7)	257.1 (236.9 to 277)	232.4 (216 to 250.6)
Latin America and Caribbean	Central Latin America	Guatemala	Incidence	Age-standardized	74.6 (71.5 to 78.4)	66.6 (63.3 to 70.3)	83.3 (79.6 to 87.5)	92.1 (87.9 to 96.7)	80.8 (76.4 to 85.5)	107.1 (101.7 to 113.1)	23.4 (19.1 to 27.7)	21.3 (15.5 to 27.6)	28.6 (22.6 to 34.9)
Latin America and Caribbean	Central Latin America	Guatemala	Prevalence	All Ages	50,218 (47,224 to 52,993)	24,845 (23,076 to 26,586)	25,373 (23,930 to 26,752)	154,061 (143,133 to 165,670)	79,563 (73,232 to 86,554)	74,499 (69,346 to 79,746)	206.8 (190.6 to 222.6)	220.2 (199.4 to 242.8)	193.6 (174.9 to 211.6)
Latin America and Caribbean	Central Latin America	Guatemala	Prevalence	Age-standardized	1071.1 (1014.2 to 1127.4)	1023.9 (959.8 to 1088.6)	1118.3 (1061.7 to 1178)	1215.2 (1139.8 to 1296.4)	1149.6 (1070.4 to 1238)	1311.8 (1235.4 to 1400.3)	13.5 (8.9 to 18)	12.3 (6.4 to 19)	17.3 (11.3 to 23.3)
Latin America and Caribbean	Central Latin America	Guatemala	Deaths	All Ages	679 (589 to 789)	290 (236 to 357)	389 (324 to 459)	1,859 (1,458 to 2,399)	920 (698 to 1,309)	940 (723 to 1,199)	173.7 (114.2 to 249.4)	216.9 (132.4 to 325)	141.5 (82.7 to 219.1)
Latin America and Caribbean	Central Latin America	Guatemala	Deaths	Age-standardized	27.2 (23.6 to 31.6)	24.5 (20.2 to 31.3)	30.2 (25.3 to 35.4)	21.2 (16.8 to 26.9)	18.3 (14 to 25.8)	26.3 (20.8 to 32.7)	-22.1 (-37.2 to -3.6)	-25.3 (-43.8 to -3.7)	-12.8 (-33.5 to 12.6)
Latin America and Caribbean	Central Latin America	Guatemala	DALYs	All Ages	18,142 (15,696 to 20,966)	8,183 (6,397 to 9,924)	9,959 (8,265 to 11,840)	38,203 (30,919 to 47,693)	19,243 (15,376 to 26,147)	18,960 (15,160 to 23,346)	110.6 (69.3 to 165.4)	135.2 (73.8 to 236.6)	90.4 (49 to 144.2)
Latin America and Caribbean	Central Latin America	Guatemala	DALYs	Age-standardized	483 (425.4 to 552.3)	429.4 (356.2 to 515.9)	539.9 (457.5 to 630)	348.7 (282.8 to 439.6)	316.3 (251.4 to 434.1)	399 (321.1 to 489.9)	-27.8 (-41.2 to -10.8)	-26.3 (-43 to -4.5)	-26.1 (-42.3 to -5.5)
Latin America and Caribbean	Central Latin America	Guatemala	YLLs	All Ages	15,584 (13,298 to 18,184)	6,777 (5,092 to 8,463)	8,807 (7,163 to 10,640)	30,650 (23,653 to 40,048)	14,978 (11,291 to 21,923)	15,672 (12,031 to 20,149)	96.7 (48.9 to 159.6)	121 (51.5 to 242.5)	78 (34.2 to 139.1)
Latin America and Caribbean	Central Latin America	Guatemala	YLLs	Age-standardized	431.8 (374.9 to 499.9)	375.4 (305.4 to 456.5)	491.5 (409.7 to 581.6)	291.7 (226.6 to 382.1)	257.4 (195.7 to 376.4)	343.7 (267.1 to 435)	-32.4 (-47.4 to -13.1)	-31.4 (-49.8 to -7.1)	-30.1 (-47.1 to -7.6)
Latin America and Caribbean	Central Latin America	Guatemala	YLDs	All Ages	2,558 (1,935 to 3,178)	1,406 (1,044 to 1,765)	1,152 (816 to 1,505)	7,553 (5,748 to 9,385)	4,265 (3,204 to 5,395)	3,288 (2,329 to 4,322)	195.3 (164.8 to 229.8)	203.4 (162.2 to 252.7)	185.3 (142.6 to 235)
Latin America and Caribbean	Central Latin America	Guatemala	YLDs	Age-standardized	51.2 (38.9 to 63.6)	54 (40.4 to 68)	48.3 (34.5 to 63.1)	57 (43.7 to 70.4)	58.9 (44.8 to 74.2)	55.3 (39.2 to 72.5)	11.3 (2.3 to 21.3)	9.1 (-2.7 to 23)	14.3 (0.9 to 28.2)
Latin America and Caribbean	Central Latin America	Honduras	Incidence	All Ages	3,126 (2,989 to 3,276)	1,703 (1,620 to 1,798)	1,422 (1,357 to 1,493)	10,121 (9,568 to 10,754)	6,035 (5,662 to 6,483)	4,085 (3,854 to 4,337)	223.8 (213.4 to 233.6)	254.4 (239.7 to 270.8)	187.2 (175.7 to 199.1)
Latin America and Caribbean	Central Latin America	Honduras	Incidence	Age-standardized	122.2 (115.8 to 129.6)	131.6 (124.1 to 140.5)	112.3 (106.1 to 119.4)	154.3 (144.8 to 164.5)	176.5 (164.5 to 189.9)	129.5 (122.1 to 138)	26.2 (22 to 30.2)	34.1 (28.1 to 40.2)	15.3 (10.4 to 20.3)
Latin America and Caribbean	Central Latin America	Honduras	Prevalence	All Ages	45,454 (43,362 to 47,707)	24,992 (23,666 to 26,428)	20,462 (19,568 to 21,435)	147,922 (139,987 to 157,212)	87,984 (82,699 to 94,262)	59,938 (56,400 to 63,644)	225.4 (214.7 to 235.6)	252 (238.5 to 267.5)	192.9 (179 to 205.7)
Latin America and Caribbean	Central Latin America	Honduras	Prevalence	Age-standardized	1807.5 (1713.1 to 1914.2)	1976 (1861.7 to 2121.1)	1631.6 (1545.7 to 1739)	2254.9 (2123.1 to 2410.3)	2573.7 (2411.4 to 2775.4)	1899.8 (1785.4 to 2026)	24.8 (20.7 to 28.6)	30.3 (24.7 to 35.8)	16.4 (11.1 to 21.6)
Latin America and Caribbean	Central Latin America	Honduras	Deaths	All Ages	771 (573 to 931)	348 (241 to 461)	424 (314 to 533)	2,859 (1,731 to 3,671)	1,341 (719 to 1,926)	1,518 (910 to 2,182)	270.7 (170.2 to 388.6)	285.7 (128.3 to 469.4)	258.5 (150.4 to 416.9)
Latin America and Caribbean	Central Latin America	Honduras	Deaths	Age-standardized	42 (29.9 to 53.2)	35.7 (23.9 to 49)	49 (34.9 to 63.1)	57.4 (33.7 to 73.1)	50.7 (26.9 to 71.5)	64.8 (38.2 to 93.5)	36.5 (0.1 to 78.4)	42.3 (-15.4 to 109.7)	32.4 (-8.1 to 92)
Latin America and Caribbean	Central Latin America	Honduras	DALYs	All Ages	21,157 (16,868 to 25,349)	10,441 (7,297 to 14,149)	10,716 (8,428 to 13,170)	60,866 (41,210 to 77,490)	30,371 (18,508 to 43,060)	30,495 (19,959 to 43,212)	187.7 (115.3 to 276.9)	190.9 (73.1 to 324.7)	184.6 (105.3 to 296.2)
Latin America and Caribbean	Central Latin America	Honduras	DALYs	Age-standardized	864.4 (676.8 to 1022.9)	800.7 (593.5 to 1027.9)	933.9 (722.2 to 1137)	1047.7 (689.5 to 1326.8)	975.8 (587.4 to 1367)	1129.5 (721.9 to 1596.5)	21.2 (-8.4 to 58)	21.9 (-21.9 to 73.9)	20.9 (-12 to 68.7)
Latin America and Caribbean	Central Latin America	Honduras	YLLs	All Ages	18,886 (14,567 to 22,963)	9,091 (5,950 to 12,714)	9,795 (7,573 to 12,221)	53,687 (33,881 to 70,391)	25,817 (13,893 to 38,239)	27,870 (17,374 to 40,367)	184.3 (102.7 to 285.5)	184 (51.3 to 341.9)	184.5 (97.4 to 309.7)
Latin America and Caribbean	Central Latin America	Honduras	YLLs	Age-standardized	778.2 (585 to 935.2)	699.7 (493.7 to 925.9)	863.3 (650.2 to 1070.2)	942.1 (582.5 to 1226.4)	847.9 (456.5 to 1239.9)	1048.7 (641.6 to 1516)	21.1 (-11.7 to 61.8)	21.2 (-28.3 to 80.5)	21.5 (-14.3 to 73.8)
Latin America and Caribbean	Central Latin America	Honduras	YLDs	All Ages	2,271 (1,743 to 2,795)	1,350 (1,014 to 1,699)	921 (658 to 1,192)	7,179 (5,384 to 8,946)	4,554 (3,342 to 5,768)	2,625 (1,828 to 3,424)	216.1 (191.3 to 241.2)	237.3 (205.5 to 269.6)	184.9 (151.7 to 220.5)
Latin America and Caribbean	Central Latin America	Honduras	YLDs	Age-standardized	86.2 (66.3 to 106.6)	101 (75.3 to 127.5)	70.6 (50 to 92.1)	105.6 (79.9 to 131.2)	127.9 (92.4 to 161.3)	80.8 (56.1 to 105)	22.6 (14.9 to 30.7)	26.6 (17.1 to 37)	14.4 (4.1 to 26)
Latin America and Caribbean	Central Latin America	Mexico	Incidence	All Ages	76,960 (70,477 to 83,793)	37,351 (34,180 to 40,778)	39,608 (36,314 to 43,085)	199,724 (181,547 to 218,889)	101,307 (92,082 to 110,831)	98,417 (89,324 to 107,808)	159.5 (152.4 to 167.7)	171.2 (161.7 to 181.7)	148.5 (142.1 to 155.4)
Latin America and Caribbean	Central Latin America	Mexico	Incidence	Age-standardized	165.6 (150.5 to 181.9)	153.2 (138.4 to 168.4)	179.3 (163.6 to 196.4)	174.8 (158.7 to 190.9)	166 (150.6 to 181.2)	184.7 (167.9 to 202)	5.6 (3.1 to 8.1)	8.4 (4.7 to 12.6)	3 (1 to 5.1)
Latin America and Caribbean	Central Latin America	Mexico	Prevalence	All Ages	1,024,529 (940,755 to 1,109,586)	515,309 (473,082 to 559,856)	509,220 (467,044 to 552,528)	2,600,960 (2,385,776 to 2,844,272)	1,324,069 (1,212,688 to 1,452,565)	1,276,891 (1,173,908 to 1,393,364)	153.9 (147.6 to 161.2)	156.9 (148.8 to 165.9)	150.8 (143.9 to 158.9)
Latin America and Caribbean	Central Latin America	Mexico	Prevalence	Age-standardized	2181.4 (1995.8 to 2384.7)	2089.5 (1911.5 to 2299.9)	2280.5 (2083.9 to 2501.9)	2247.2 (2053.2 to 2463)	2143.3 (1960.3 to 2356.6)	2367.5 (2166.3 to 2593.8)	3 (0.5 to 5.7)	2.6 (-0.7 to 6.3)	3.8 (1.6 to 6.2)
Latin America and Caribbean	Central Latin America	Mexico	Deaths	All Ages	12,719 (11,298 to 13,309)	5,372 (4,161 to 5,719)	7,347 (6,808 to 7,654)	32,781 (27,521 to 38,201)	15,079 (12,068 to 18,334)	17,702 (14,451 to 21,296)	157.7 (122.4 to 197.8)	180.7 (132.7 to 241.9)	141 (100.1 to 189.3)
Latin America and Caribbean	Central Latin America	Mexico	Deaths	Age-standardized	38.9 (34.1 to 40.9)	32.5 (24.7 to 34.9)	45.6 (41.4 to 47.7)	31.7 (26.5 to 36.9)	26.9 (21.4 to 32.6)	37.5 (30.4 to 44.8)	-18.3 (-29 to -6)	-17.1 (-30.7 to 0)	-17.6 (-31.1 to -1.7)
Latin America and Caribbean	Central Latin America	Mexico	DALYs	All Ages	274,200 (251,037 to 289,744)	121,493 (103,471 to 130,991)	152,707 (144,034 to 160,910)	623,207 (541,012 to 715,652)	289,075 (239,805 to 348,524)	334,132 (277,422 to 395,735)	127.3 (100.3 to 160.7)	137.9 (103.6 to 185.7)	118.8 (84.1 to 159.5)
Latin America and Caribbean	Central Latin America	Mexico	DALYs	Age-standardized	669.7 (610.7 to 707.1)	567.7 (471.8 to 611.2)	780.5 (733.1 to 821.3)	559.7 (487.3 to 641)	482.6 (401.9 to 577.6)	650.6 (539.2 to 773)	-16.4 (-26.2 to -3.9)	-15 (-27.3 to 2.1)	-16.6 (-29.7 to -1.3)
Latin America and Caribbean	Central Latin America	Mexico	YLLs	All Ages	221,910 (200,721 to 230,498)	92,599 (73,994 to 98,119)	129,311 (123,243 to 133,744)	495,344 (417,057 to 582,560)	218,467 (176,018 to 274,045)	276,877 (222,999 to 339,854)	123.2 (89.1 to 164)	135.9 (92 to 199.5)	114.1 (73.7 to 162)
Latin America and Caribbean	Central Latin America	Mexico	YLLs	Age-standardized	563.4 (504.6 to 586.6)	456.6 (355 to 484.5)	679.2 (639.9 to 704.5)	450.4 (380.1 to 528.3)	369.5 (298 to 461.1)	545.6 (441.4 to 666.8)	-20.1 (-31.9 to -5.2)	-19.1 (-33.8 to 2.2)	-19.7 (-34.5 to -2.1)
Latin America and Caribbean	Central Latin America	Mexico	YLDs	All Ages	52,290 (39,531 to 64,160)	28,894 (22,195 to 35,841)	23,395 (16,836 to 29,740)	127,863 (97,289 to 157,799)	70,608 (52,862 to 88,674)	57,254 (40,564 to 73,578)	144.5 (132.4 to 155.2)	144.4 (128.9 to 158.6)	144.7 (131.5 to 156.2)
Latin America and Caribbean	Central Latin America	Mexico	YLDs	Age-standardized	106.3 (80.4 to 130.9)	111.1 (83.1 to 139.8)	101.3 (71 to 130.8)	109.3 (83.1 to 135.4)	113.1 (84.4 to 142)	105 (73.6 to 135.3)	2.8 (-0.4 to 5.7)	1.8 (-2.1 to 6)	3.6 (0.8 to 6.4)
Latin America and Caribbean	Central Latin America	Nicaragua	Incidence	All Ages	1,629 (1,522 to 1,731)	846 (786 to 901)	783 (729 to 835)	5,850 (5,543 to 6,168)	3,201 (3,013 to 3,408)	2,648 (2,504 to 2,793)	259.2 (243.6 to 276.5)	278.5 (256.5 to 302.8)	238.3 (220.8 to 257.6)
Latin America and Caribbean	Central Latin America	Nicaragua	Incidence	Age-standardized	83.3 (78.9 to 88)	76.4 (72.1 to 81.5)	92.9 (88 to 98.3)	132.6 (125.8 to 139.3)	134.8 (127.1 to 143.8)	127.5 (120.9 to 134.6)	59.2 (53.2 to 65.4)	76.5 (67.5 to 86.6)	37.2 (30.6 to 44.2)
Latin America and Caribbean	Central Latin America	Nicaragua	Prevalence	All Ages	23,369 (21,263 to 25,417)	12,576 (11,397 to 13,674)	10,793 (9,735 to 11,881)	81,712 (76,164 to 87,520)	44,274 (40,971 to 47,377)	37,439 (34,674 to 40,506)	249.7 (233.1 to 268.2)	252.1 (231.9 to 276.6)	246.9 (224.3 to 270.9)
Latin America and Caribbean	Central Latin America	Nicaragua	Prevalence	Age-standardized	1170.1 (1089.7 to 1250.3)	1151.7 (1063.6 to 1243.6)	1208.1 (1122.6 to 1298.8)	1773.4 (1668.1 to 1888.4)	1773.3 (1656.7 to 1896.5)	1763.9 (1653.5 to 1885.4)	51.6 (46.1 to 57.7)	54 (46.3 to 62.3)	46 (38.8 to 54.2)
Latin America and Caribbean	Central Latin America	Nicaragua	Deaths	All Ages	264 (231 to 307)	95 (77 to 124)	169 (147 to 198)	1,169 (872 to 1,353)	535 (322 to 638)	634 (490 to 757)	342.2 (206.8 to 424.8)	461.8 (176.1 to 622.6)	274.9 (166.1 to 375.8)
Latin America and Caribbean	Central Latin America	Nicaragua	Deaths	Age-standardized	21.2 (18.1 to 25)	12.9 (10.4 to 16.6)	34.7 (29.9 to 40.8)	35.6 (26.3 to 41.1)	28.5 (17.3 to 34)	45.7 (34.9 to 54)	68.5 (15.6 to 99.1)	121.3 (8.9 to 183.8)	31.6 (-9 to 65.1)
Latin America and Caribbean	Central Latin America	Nicaragua	DALYs	All Ages	6,010 (5,405 to 6,758)	2,444 (2,110 to 2,912)	3,566 (3,145 to 4,074)	21,927 (17,556 to 25,173)	10,176 (7,034 to 11,788)	11,751 (9,378 to 13,991)	264.8 (180.4 to 325.8)	316.4 (172.1 to 403.7)	229.5 (155 to 307.6)
Latin America and Caribbean	Central Latin America	Nicaragua	DALYs	Age-standardized	390.5 (348.9 to 440.3)	272 (234.1 to 329.2)	557.3 (489.5 to 642.1)	563.3 (445.2 to 643.4)	463.1 (313.8 to 536)	697.4 (553.7 to 826.6)	44.3 (8.6 to 68.4)	70.3 (4.4 to 107)	25.1 (-6.5 to 55.6)
Latin America and Caribbean	Central Latin America	Nicaragua	YLLs	All Ages	4,831 (4,297 to 5,469)	1,750 (1,461 to 2,194)	3,081 (2,714 to 3,575)	18,035 (13,795 to 21,239)	7,899 (4,851 to 9,467)	10,136 (7,837 to 12,262)	273.3 (169.9 to 351.3)	351.4 (145.4 to 478.7)	229 (143.5 to 323.2)
Latin America and Caribbean	Central Latin America	Nicaragua	YLLs	Age-standardized	335.1 (297.7 to 384.7)	212.4 (176.6 to 268.1)	506.1 (441.1 to 593)	482.1 (361.1 to 562.9)	375.4 (226.7 to 444.9)	624.2 (481.9 to 746.1)	43.9 (3.3 to 72.2)	76.7 (-5.2 to 126.3)	23.3 (-10.8 to 57.2)
Latin America and Caribbean	Central Latin America	Nicaragua	YLDs	All Ages	1,179 (911 to 1,479)	694 (511 to 885)	485 (343 to 649)	3,892 (2,955 to 4,804)	2,278 (1,695 to 2,852)	1,615 (1,132 to 2,135)	230.1 (196.2 to 265.4)	228.4 (185.3 to 277.9)	232.7 (185.3 to 282.9)
Latin America and Caribbean	Central Latin America	Nicaragua	YLDs	Age-standardized	55.4 (42.2 to 69.3)	59.6 (44.7 to 76)	51.2 (36.5 to 67.7)	81.2 (61.5 to 100.9)	87.7 (64.4 to 110.9)	73.2 (51 to 96.9)	46.6 (36 to 57.8)	47.2 (31.6 to 63)	43 (28.3 to 58.3)
Latin America and Caribbean	Central Latin America	Panama	Incidence	All Ages	1,838 (1,747 to 1,932)	789 (744 to 833)	1,049 (997 to 1,105)	5,047 (4,813 to 5,305)	2,429 (2,291 to 2,571)	2,619 (2,490 to 2,759)	174.6 (164.3 to 184.8)	207.9 (191.8 to 223.7)	149.6 (137.9 to 161.6)
Latin America and Caribbean	Central Latin America	Panama	Incidence	Age-standardized	115.2 (109.9 to 121)	96.1 (91.2 to 101.6)	134.2 (127.8 to 141.4)	120.1 (114.3 to 126.4)	109.4 (103 to 116.1)	131.7 (125.2 to 138.9)	4.3 (0.3 to 8.1)	13.8 (8.1 to 19.5)	-1.9 (-6.5 to 2.8)
Latin America and Caribbean	Central Latin America	Panama	Prevalence	All Ages	24,821 (23,169 to 26,629)	11,165 (10,278 to 11,991)	13,656 (12,720 to 14,694)	66,877 (62,966 to 71,327)	32,848 (30,726 to 35,233)	34,030 (31,858 to 36,532)	169.4 (158.8 to 180.9)	194.2 (178.6 to 211.2)	149.2 (136.7 to 162.8)
Latin America and Caribbean	Central Latin America	Panama	Prevalence	Age-standardized	1503.8 (1413.6 to 1598.1)	1321.2 (1230.9 to 1414.3)	1689.7 (1588.7 to 1807)	1584 (1490.8 to 1690.8)	1490.3 (1389.4 to 1600.1)	1689.6 (1581.7 to 1811.5)	5.3 (1.4 to 9.1)	12.8 (7.2 to 18.3)	0 (-4.9 to 5)
Latin America and Caribbean	Central Latin America	Panama	Deaths	All Ages	263 (230 to 300)	106 (88 to 141)	157 (138 to 177)	717 (533 to 901)	351 (255 to 459)	366 (273 to 472)	173 (114.5 to 237.7)	232.8 (157.7 to 315.1)	132.8 (77.7 to 195.8)
Latin America and Caribbean	Central Latin America	Panama	Deaths	Age-standardized	19.2 (16.6 to 22)	15.1 (12.4 to 20.3)	23.4 (20.4 to 26.4)	16.5 (12.2 to 20.8)	14.6 (10.6 to 19.2)	18.8 (14 to 24.3)	-14 (-32.3 to 6.5)	-3.4 (-25.7 to 22.1)	-19.6 (-38.4 to 2.3)
Latin America and Caribbean	Central Latin America	Panama	DALYs	All Ages	5,710 (5,168 to 6,310)	2,353 (2,037 to 2,745)	3,357 (2,983 to 3,752)	12,773 (10,256 to 15,500)	6,162 (4,845 to 7,766)	6,610 (5,207 to 8,313)	123.7 (84.2 to 170.9)	161.9 (114.8 to 216)	96.9 (55.8 to 145.9)
Latin America and Caribbean	Central Latin America	Panama	DALYs	Age-standardized	376.6 (339.4 to 418.4)	302.3 (262.7 to 361.7)	450.4 (398.7 to 504.4)	304.8 (244.1 to 370.6)	278.5 (220.3 to 352.7)	334.5 (263.6 to 421.5)	-19.1 (-33.5 to -2.5)	-7.9 (-25.3 to 11.3)	-25.7 (-41.3 to -7.5)
Latin America and Caribbean	Central Latin America	Panama	YLLs	All Ages	4,527 (4,082 to 4,995)	1,760 (1,495 to 2,138)	2,767 (2,452 to 3,134)	9,719 (7,273 to 12,515)	4,527 (3,328 to 6,036)	5,192 (3,880 to 6,825)	114.7 (64.8 to 173.4)	157.1 (95.4 to 228)	87.7 (38.3 to 146.4)
Latin America and Caribbean	Central Latin America	Panama	YLLs	Age-standardized	307.5 (275.2 to 342.1)	234.8 (199.7 to 292.4)	379.5 (335 to 430)	232.4 (174.4 to 300.2)	203.6 (149.7 to 273.7)	264.4 (197.8 to 347.7)	-24.4 (-42.2 to -4.1)	-13.3 (-34.1 to 10.5)	-30.3 (-48.4 to -9)
Latin America and Caribbean	Central Latin America	Panama	YLDs	All Ages	1,182 (902 to 1,480)	593 (448 to 748)	590 (409 to 773)	3,054 (2,329 to 3,800)	1,636 (1,206 to 2,060)	1,418 (985 to 1,877)	158.3 (137.5 to 180.1)	176 (144.1 to 210.6)	140.5 (113.9 to 167)
Latin America and Caribbean	Central Latin America	Panama	YLDs	Age-standardized	69.1 (52.4 to 86.2)	67.5 (50.4 to 85.5)	70.9 (49.5 to 93.5)	72.4 (55.2 to 90.2)	74.9 (55.1 to 94.1)	70.1 (48.3 to 92.8)	4.8 (-1.8 to 12.4)	11 (0.8 to 22.4)	-1.1 (-9.9 to 8.6)
Latin America and Caribbean	Central Latin America	Venezuela (Bolivarian Republic of)	Incidence	All Ages	11,413 (10,899 to 11,933)	5,300 (5,011 to 5,575)	6,113 (5,819 to 6,423)	39,076 (37,222 to 40,985)	18,716 (17,785 to 19,749)	20,360 (19,262 to 21,432)	242.4 (227.4 to 257.4)	253.1 (234.3 to 273.5)	233.1 (215.2 to 250.6)
Latin America and Caribbean	Central Latin America	Venezuela (Bolivarian Republic of)	Incidence	Age-standardized	99.3 (95.6 to 103.4)	85.6 (81.7 to 89.9)	115.3 (110.2 to 120.4)	137.9 (131.5 to 144.5)	122.9 (116.6 to 129.4)	154.8 (146.4 to 163.1)	38.8 (34 to 43.7)	43.5 (37 to 50.1)	34.3 (28.1 to 40.9)
Latin America and Caribbean	Central Latin America	Venezuela (Bolivarian Republic of)	Prevalence	All Ages	172,873 (160,288 to 184,266)	84,812 (78,128 to 90,692)	88,062 (81,337 to 94,821)	578,386 (546,082 to 614,104)	283,520 (266,957 to 301,365)	294,866 (276,581 to 314,826)	234.6 (218.5 to 252.3)	234.3 (215.1 to 257.4)	234.8 (214.4 to 255.3)
Latin America and Caribbean	Central Latin America	Venezuela (Bolivarian Republic of)	Prevalence	Age-standardized	1488.2 (1407.9 to 1575.9)	1370.4 (1280.4 to 1459.2)	1630.6 (1537.7 to 1735.1)	2003 (1890 to 2126.7)	1829.7 (1719.7 to 1946.4)	2208.8 (2075.2 to 2354)	34.6 (29.7 to 39.5)	33.5 (27 to 40.6)	35.5 (28.5 to 42.5)
Latin America and Caribbean	Central Latin America	Venezuela (Bolivarian Republic of)	Deaths	All Ages	1,527 (1,362 to 1,753)	682 (576 to 888)	845 (760 to 938)	6,217 (4,778 to 7,920)	2,853 (2,067 to 3,721)	3,364 (2,536 to 4,385)	307.2 (212.3 to 419.8)	318.5 (211 to 457.3)	298.1 (193.8 to 418.7)
Latin America and Caribbean	Central Latin America	Venezuela (Bolivarian Republic of)	Deaths	Age-standardized	18.8 (16.6 to 21.8)	15.6 (13 to 20.6)	22.6 (20.1 to 25.1)	23.1 (17.7 to 29.4)	18.6 (13.5 to 24.4)	28.6 (21.7 to 36.9)	22.7 (-6.1 to 55.9)	19.7 (-11.9 to 61.7)	26.4 (-5.9 to 64.2)
Latin America and Caribbean	Central Latin America	Venezuela (Bolivarian Republic of)	DALYs	All Ages	37,521 (34,211 to 41,541)	16,819 (14,912 to 19,765)	20,701 (18,765 to 22,917)	131,889 (104,357 to 164,405)	58,947 (46,062 to 73,191)	72,942 (56,380 to 94,454)	251.5 (177.5 to 341.4)	250.5 (176.2 to 349)	252.4 (169 to 355.9)
Latin America and Caribbean	Central Latin America	Venezuela (Bolivarian Republic of)	DALYs	Age-standardized	383.1 (347.2 to 427.6)	319.5 (280.3 to 385.2)	456.7 (414.9 to 504.7)	467.2 (370.4 to 578.6)	386.3 (301.9 to 482.6)	563 (438.5 to 716.6)	21.9 (-3.4 to 52.6)	20.9 (-4.9 to 55.5)	23.3 (-5.4 to 57.1)
Latin America and Caribbean	Central Latin America	Venezuela (Bolivarian Republic of)	YLLs	All Ages	29,049 (26,486 to 32,303)	12,223 (10,656 to 15,106)	16,826 (15,194 to 18,673)	105,347 (79,282 to 137,760)	44,757 (32,688 to 59,449)	60,590 (44,517 to 80,915)	262.6 (167.7 to 377.8)	266.2 (168 to 398)	260.1 (155.9 to 389)
Latin America and Caribbean	Central Latin America	Venezuela (Bolivarian Republic of)	YLLs	Age-standardized	313.8 (283.1 to 353)	248.9 (213.4 to 315.8)	388 (350.4 to 429.9)	375.8 (284.2 to 489.2)	294.7 (214.8 to 390.1)	471.6 (349.5 to 624.4)	19.8 (-10.9 to 57.3)	18.4 (-13.7 to 61.8)	21.5 (-12.3 to 62.1)
Latin America and Caribbean	Central Latin America	Venezuela (Bolivarian Republic of)	YLDs	All Ages	8,471 (6,426 to 10,676)	4,596 (3,420 to 5,811)	3,875 (2,695 to 5,131)	26,541 (20,023 to 33,195)	14,190 (10,389 to 18,102)	12,352 (8,544 to 16,407)	213.3 (182.5 to 243.7)	208.7 (169.4 to 250.8)	218.7 (176.9 to 260.4)
Latin America and Caribbean	Central Latin America	Venezuela (Bolivarian Republic of)	YLDs	Age-standardized	69.4 (52.5 to 86.6)	70.5 (52.3 to 89)	68.7 (48.3 to 90)	91.4 (69 to 114)	91.6 (67.3 to 116.5)	91.4 (64.2 to 121.4)	31.7 (22.7 to 40.6)	29.9 (16.8 to 43.2)	33.1 (19.8 to 46.8)
		Tropical Latin America	Incidence	All Ages	214,342 (196,695 to 231,273)	101,210 (92,524 to 110,122)	113,132 (103,789 to 121,784)	436,987 (399,600 to 476,916)	209,717 (192,099 to 230,082)	227,269 (206,926 to 248,790)	103.9 (95.8 to 112.2)	107.2 (97.1 to 118.3)	100.9 (92.7 to 109)
		Tropical Latin America	Incidence	Age-standardized	222 (202.8 to 239.9)	197.2 (179.9 to 215.6)	250.5 (229.2 to 268.7)	183.1 (167.9 to 199.6)	160.6 (147.3 to 176.3)	210.8 (192.5 to 230.3)	-17.5 (-20.6 to -14.5)	-18.5 (-22.6 to -14.6)	-15.9 (-19.1 to -12.7)
		Tropical Latin America	Prevalence	All Ages	2,969,035 (2,774,856 to 3,168,873)	1,477,888 (1,378,124 to 1,584,739)	1,491,147 (1,392,900 to 1,589,001)	6,330,068 (5,844,669 to 6,860,751)	3,194,483 (2,940,832 to 3,475,478)	3,135,585 (2,893,452 to 3,407,679)	113.2 (105 to 121.2)	116.2 (105.8 to 125.8)	110.3 (101.7 to 118.4)
		Tropical Latin America	Prevalence	Age-standardized	3116 (2895.1 to 3344.7)	2898.9 (2682.6 to 3128.8)	3388.3 (3144 to 3636.8)	2634.5 (2436.3 to 2860.8)	2424.8 (2234.4 to 2635.3)	2915.8 (2687.1 to 3169)	-15.5 (-18.8 to -12.5)	-16.4 (-20.4 to -12.5)	-13.9 (-17.3 to -10.7)
		Tropical Latin America	Deaths	All Ages	35,653 (32,641 to 37,392)	13,967 (11,702 to 15,237)	21,686 (20,515 to 22,596)	69,670 (62,026 to 78,843)	31,794 (27,324 to 39,295)	37,876 (34,472 to 40,860)	95.4 (79.7 to 117.1)	127.6 (103.4 to 170.8)	74.7 (61.1 to 89.1)
		Tropical Latin America	Deaths	Age-standardized	49.7 (44.7 to 52.4)	36 (30.2 to 39.6)	67.1 (62.3 to 70.4)	30.6 (27.2 to 34.7)	24 (20.7 to 29.7)	39.8 (35.8 to 43.1)	-38.4 (-43 to -31.4)	-33.2 (-39.9 to -20.9)	-40.8 (-45.1 to -36)
		Tropical Latin America	DALYs	All Ages	839,562 (783,787 to 887,661)	338,509 (289,572 to 366,803)	501,054 (474,256 to 526,495)	1,454,619 (1,339,057 to 1,596,900)	662,726 (591,393 to 778,815)	791,893 (731,803 to 853,780)	73.3 (62.9 to 88.5)	95.8 (80.4 to 123.8)	58 (47.9 to 69.2)
		Tropical Latin America	DALYs	Age-standardized	961.5 (893.4 to 1016.5)	718.8 (617.6 to 779.4)	1253.4 (1180.7 to 1313.9)	613.2 (563.9 to 674)	502.1 (447.9 to 590.1)	758 (698 to 817.7)	-36.2 (-39.9 to -30.6)	-30.1 (-35.6 to -20.1)	-39.5 (-43.4 to -35.4)
		Tropical Latin America	YLLs	All Ages	714,895 (663,646 to 745,369)	270,905 (225,510 to 292,593)	443,990 (424,053 to 461,463)	1,193,661 (1,100,824 to 1,336,790)	520,307 (463,247 to 631,019)	673,354 (623,264 to 721,100)	67 (54.7 to 85.1)	92.1 (72.7 to 127.7)	51.7 (40.7 to 63.8)
		Tropical Latin America	YLLs	Age-standardized	834.9 (768.6 to 873.7)	590.7 (491.1 to 642.2)	1127.6 (1070.9 to 1174.1)	505 (464.1 to 564.1)	393.7 (350.5 to 478.5)	648.7 (598 to 694.4)	-39.5 (-43.8 to -33.1)	-33.4 (-39.7 to -21.3)	-42.5 (-46.5 to -38)
		Tropical Latin America	YLDs	All Ages	124,667 (90,273 to 158,493)	67,604 (49,568 to 85,858)	57,064 (39,193 to 75,081)	260,958 (186,484 to 333,645)	142,419 (101,490 to 184,151)	118,539 (79,516 to 157,900)	109.3 (98.2 to 119)	110.7 (98 to 122.4)	107.7 (95.2 to 118.8)
		Tropical Latin America	YLDs	Age-standardized	126.6 (90.3 to 162)	128.1 (92.7 to 163.5)	125.8 (85.4 to 166.1)	108.2 (77.6 to 138.2)	108.4 (77.6 to 140)	109.3 (74.5 to 144.7)	-14.5 (-18 to -11.2)	-15.3 (-19.5 to -11.2)	-13.2 (-16.9 to -9.5)
Latin America and Caribbean	Tropical Latin America	Brazil	Incidence	All Ages	211,586 (193,983 to 228,466)	99,924 (91,264 to 108,773)	111,662 (102,303 to 120,325)	429,959 (392,741 to 469,636)	207,060 (189,581 to 227,429)	222,899 (202,686 to 244,296)	103.2 (95 to 111.6)	107.2 (97 to 118.5)	99.6 (91.3 to 107.8)
Latin America and Caribbean	Tropical Latin America	Brazil	Incidence	Age-standardized	224.8 (205.3 to 243.1)	199.8 (182.1 to 218.4)	253.6 (231.8 to 272.2)	184.6 (169.1 to 201.4)	162.3 (148.8 to 178.3)	212 (193.5 to 231.7)	-17.9 (-21 to -14.9)	-18.7 (-22.8 to -14.7)	-16.4 (-19.7 to -13.2)
Latin America and Caribbean	Tropical Latin America	Brazil	Prevalence	All Ages	2,929,615 (2,737,000 to 3,127,815)	1,458,086 (1,359,115 to 1,564,433)	1,471,529 (1,374,172 to 1,569,212)	6,228,274 (5,745,150 to 6,755,662)	3,152,173 (2,899,975 to 3,431,089)	3,076,101 (2,836,815 to 3,345,406)	112.6 (104.3 to 120.6)	116.2 (105.7 to 126)	109 (100.3 to 117.4)
Latin America and Caribbean	Tropical Latin America	Brazil	Prevalence	Age-standardized	3158.5 (2931.7 to 3392.1)	2937.1 (2716.2 to 3172.2)	3436.2 (3185.3 to 3690.7)	2655.5 (2453.7 to 2885.8)	2448.4 (2254.3 to 2661.9)	2933.9 (2702.4 to 3192.8)	-15.9 (-19.3 to -12.9)	-16.6 (-20.6 to -12.7)	-14.6 (-18 to -11.3)
Latin America and Caribbean	Tropical Latin America	Brazil	Deaths	All Ages	35,317 (32,304 to 37,038)	13,826 (11,560 to 15,082)	21,492 (20,340 to 22,398)	68,697 (61,265 to 77,839)	31,496 (27,023 to 38,863)	37,200 (33,812 to 40,123)	94.5 (78.7 to 116.2)	127.8 (103.8 to 171.2)	73.1 (59.2 to 87.3)
Latin America and Caribbean	Tropical Latin America	Brazil	Deaths	Age-standardized	50.7 (45.6 to 53.6)	36.8 (30.7 to 40.4)	68.7 (63.8 to 72.1)	30.9 (27.5 to 35.1)	24.3 (20.9 to 30)	40 (36 to 43.3)	-39.1 (-43.7 to -32.2)	-33.8 (-40.4 to -21.4)	-41.7 (-46.2 to -37.1)
Latin America and Caribbean	Tropical Latin America	Brazil	DALYs	All Ages	831,746 (775,724 to 880,031)	334,993 (286,009 to 363,260)	496,753 (470,183 to 521,894)	1,434,009 (1,320,385 to 1,576,595)	656,367 (585,316 to 771,675)	777,642 (718,876 to 838,468)	72.4 (62.1 to 87.7)	95.9 (80.7 to 124)	56.5 (46.2 to 67.7)
Latin America and Caribbean	Tropical Latin America	Brazil	DALYs	Age-standardized	978.8 (907.4 to 1035.1)	731 (625.6 to 793.2)	1277.3 (1201.3 to 1339.1)	618.7 (568.6 to 681.2)	508.5 (453.3 to 597.4)	762.8 (702.5 to 822.7)	-36.8 (-40.4 to -31.2)	-30.4 (-35.9 to -20.4)	-40.3 (-44.1 to -36.1)
Latin America and Caribbean	Tropical Latin America	Brazil	YLLs	All Ages	708,698 (657,223 to 739,464)	268,282 (222,819 to 289,912)	440,416 (420,285 to 458,155)	1,177,030 (1,084,003 to 1,316,624)	515,788 (456,857 to 623,428)	661,242 (610,846 to 707,345)	66.1 (53.8 to 84.4)	92.3 (73.1 to 127.6)	50.1 (39 to 62.5)
Latin America and Caribbean	Tropical Latin America	Brazil	YLLs	Age-standardized	850.4 (780 to 890)	601.2 (498.9 to 653.1)	1149.6 (1090.1 to 1197.4)	509.5 (468.3 to 569.7)	398.9 (353.7 to 483.4)	652.8 (600.9 to 699.2)	-40.1 (-44.3 to -33.6)	-33.6 (-39.9 to -21.8)	-43.2 (-47.3 to -38.6)
Latin America and Caribbean	Tropical Latin America	Brazil	YLDs	All Ages	123,048 (89,089 to 156,397)	66,711 (48,784 to 84,740)	56,337 (38,602 to 74,123)	256,979 (183,760 to 328,703)	140,579 (100,307 to 181,729)	116,400 (78,227 to 155,317)	108.8 (97.6 to 118.7)	110.7 (97.9 to 122.4)	106.6 (94.1 to 117.9)
Latin America and Caribbean	Tropical Latin America	Brazil	YLDs	Age-standardized	128.3 (91.5 to 164.1)	129.8 (93.7 to 165.8)	127.7 (86.3 to 168.8)	109.2 (78.3 to 139.7)	109.5 (78.6 to 141.4)	110.1 (74.9 to 145.7)	-14.9 (-18.4 to -11.6)	-15.6 (-19.8 to -11.4)	-13.8 (-17.5 to -10.1)
Latin America and Caribbean	Tropical Latin America	Paraguay	Incidence	All Ages	2,756 (2,637 to 2,879)	1,286 (1,230 to 1,347)	1,470 (1,392 to 1,547)	7,028 (6,676 to 7,399)	2,657 (2,489 to 2,846)	4,370 (4,149 to 4,612)	154.9 (146.1 to 163.6)	106.6 (96.1 to 118.1)	197.3 (185.5 to 210.1)
Latin America and Caribbean	Tropical Latin America	Paraguay	Incidence	Age-standardized	109.5 (104.2 to 114.6)	93.3 (88.5 to 98.4)	128.8 (121.9 to 135.4)	122.6 (116.3 to 129.4)	86.3 (80.8 to 92.7)	163.3 (154.7 to 172.3)	12 (8.2 to 15.9)	-7.6 (-12.4 to -2.3)	26.7 (21.4 to 32.5)
Latin America and Caribbean	Tropical Latin America	Paraguay	Prevalence	All Ages	39,420 (37,322 to 41,409)	19,802 (18,846 to 20,710)	19,618 (18,282 to 20,900)	101,794 (95,740 to 107,746)	42,310 (39,065 to 45,704)	59,484 (55,961 to 62,998)	158.2 (148.5 to 167.3)	113.7 (99.4 to 127.1)	203.2 (189.5 to 218.1)
Latin America and Caribbean	Tropical Latin America	Paraguay	Prevalence	Age-standardized	1540.5 (1463.8 to 1619.7)	1462.7 (1388.6 to 1535)	1650.6 (1548.7 to 1758.6)	1741.9 (1641.5 to 1846.3)	1363.6 (1261.2 to 1471.4)	2188.7 (2059.7 to 2316)	13.1 (8.9 to 17.1)	-6.8 (-12.8 to -0.7)	32.6 (26.8 to 38.6)
Latin America and Caribbean	Tropical Latin America	Paraguay	Deaths	All Ages	336 (288 to 447)	142 (117 to 187)	194 (163 to 284)	973 (748 to 1,244)	298 (209 to 433)	676 (512 to 864)	190 (114.1 to 272.3)	110.2 (39.2 to 181.6)	248.2 (136.6 to 370.3)
Latin America and Caribbean	Tropical Latin America	Paraguay	Deaths	Age-standardized	16.9 (14.3 to 22.7)	12.8 (10.5 to 17)	22.1 (18.3 to 32.5)	18.9 (14.5 to 24.3)	10.3 (7.1 to 15)	29.7 (22.4 to 37.7)	12.2 (-17.2 to 44.1)	-19.6 (-47.6 to 7.5)	34.6 (-8.9 to 81.1)
Latin America and Caribbean	Tropical Latin America	Paraguay	DALYs	All Ages	7,817 (6,896 to 9,421)	3,516 (2,996 to 4,165)	4,301 (3,712 to 5,726)	20,610 (16,499 to 25,744)	6,359 (4,870 to 8,671)	14,251 (11,085 to 17,971)	163.7 (106.7 to 229.2)	80.9 (36.9 to 135.3)	231.4 (138.9 to 334.2)
Latin America and Caribbean	Tropical Latin America	Paraguay	DALYs	Age-standardized	332.8 (291.3 to 421.1)	271.6 (232.3 to 338.2)	406.9 (348.2 to 564.8)	376.1 (299.1 to 469.9)	214.9 (163.2 to 296.5)	561.8 (435.2 to 708.8)	13 (-13 to 42.6)	-20.9 (-41.2 to 0.8)	38.1 (-2 to 81.3)
Latin America and Caribbean	Tropical Latin America	Paraguay	YLLs	All Ages	6,197 (5,423 to 7,693)	2,623 (2,157 to 3,271)	3,574 (3,036 to 4,982)	16,631 (12,710 to 21,518)	4,519 (3,172 to 6,798)	12,112 (9,054 to 15,799)	168.4 (98.4 to 250.6)	72.3 (16.2 to 144.9)	238.9 (130.2 to 365.1)
Latin America and Caribbean	Tropical Latin America	Paraguay	YLLs	Age-standardized	271.9 (236.9 to 359.6)	208.1 (173.8 to 278.7)	347.8 (292.7 to 501.2)	309.4 (235.6 to 397.2)	156.5 (109.1 to 234.4)	484.8 (361.9 to 632.5)	13.8 (-17.4 to 50.3)	-24.8 (-50.1 to 2.2)	39.4 (-6.7 to 90.2)
Latin America and Caribbean	Tropical Latin America	Paraguay	YLDs	All Ages	1,620 (1,194 to 2,051)	893 (645 to 1,147)	727 (480 to 976)	3,979 (2,857 to 5,084)	1,840 (1,323 to 2,381)	2,139 (1,408 to 2,833)	145.7 (125.5 to 167.2)	106.1 (82.9 to 131.9)	194.3 (157.4 to 232.1)
Latin America and Caribbean	Tropical Latin America	Paraguay	YLDs	Age-standardized	60.8 (44.5 to 77.7)	63.5 (45 to 82.2)	59 (38.6 to 79.9)	66.7 (47.3 to 85.4)	58.5 (41.4 to 76.1)	77 (50 to 103.1)	9.7 (2 to 18)	-7.9 (-17.4 to 2.4)	30.5 (17.5 to 43.5)
		North Africa and Middle East	Incidence	All Ages	232,496 (219,917 to 245,243)	100,164 (94,436 to 106,335)	132,332 (125,247 to 139,547)	704,341 (671,175 to 739,648)	277,525 (261,404 to 293,646)	426,816 (408,870 to 446,210)	202.9 (196.4 to 209.8)	177.1 (170.4 to 184)	222.5 (214.6 to 231.1)
		North Africa and Middle East	Incidence	Age-standardized	112.2 (105.5 to 119)	95.6 (89.3 to 102.1)	128.3 (121.4 to 135.7)	150.8 (143.1 to 158.7)	121.3 (113.9 to 128.9)	179.3 (171.1 to 187.9)	34.4 (31.5 to 37.4)	26.8 (23.8 to 29.9)	39.7 (36.3 to 43.3)
		North Africa and Middle East	Prevalence	All Ages	3,584,089 (3,404,769 to 3,768,097)	1,623,749 (1,538,103 to 1,709,707)	1,960,341 (1,865,634 to 2,060,495)	10,720,230 (10,262,918 to 11,204,177)	4,540,211 (4,315,629 to 4,780,162)	6,180,019 (5,938,386 to 6,435,666)	199.1 (194.2 to 204.6)	179.6 (174 to 185.5)	215.3 (208.8 to 222.3)
		North Africa and Middle East	Prevalence	Age-standardized	1787.6 (1690.9 to 1887.5)	1605.3 (1512 to 1704.5)	1971.1 (1867.8 to 2077)	2333.9 (2230.1 to 2443.6)	1981.2 (1876.6 to 2093.3)	2681.6 (2575.5 to 2793.8)	30.6 (28.2 to 33)	23.4 (20.8 to 26.1)	36 (33.2 to 39.3)
		North Africa and Middle East	Deaths	All Ages	43,173 (37,093 to 49,741)	16,206 (10,632 to 20,267)	26,967 (22,367 to 32,150)	89,920 (76,785 to 101,801)	32,684 (24,831 to 37,962)	57,236 (48,001 to 65,643)	108.3 (78.2 to 145.3)	101.7 (59.7 to 226.9)	112.2 (71.5 to 159.1)
		North Africa and Middle East	Deaths	Age-standardized	31.8 (26.9 to 36.7)	23.8 (16 to 29.8)	40.2 (32.9 to 48.1)	26.1 (22.2 to 29.5)	19.5 (14.7 to 22.6)	32.6 (27.5 to 37.1)	-18 (-30.9 to -2.8)	-18.2 (-35.4 to 28.7)	-18.9 (-34.3 to 0.3)
		North Africa and Middle East	DALYs	All Ages	1,315,162 (1,164,472 to 1,461,299)	530,151 (392,277 to 640,540)	785,011 (676,740 to 906,107)	2,761,836 (2,436,839 to 3,062,623)	1,073,582 (901,530 to 1,205,699)	1,688,254 (1,452,958 to 1,897,561)	110 (89.9 to 134.5)	102.5 (74.4 to 184.3)	115.1 (83.1 to 147.6)
		North Africa and Middle East	DALYs	Age-standardized	736.1 (648.7 to 818.5)	580.6 (432.2 to 691.8)	893 (765.6 to 1034.4)	649.1 (574.6 to 717.7)	509.1 (430.2 to 569.2)	786.2 (686.7 to 883)	-11.8 (-21.1 to -0.9)	-12.3 (-25 to 23.4)	-12 (-25.4 to 3.1)
		North Africa and Middle East	YLLs	All Ages	980,865 (842,403 to 1,120,521)	365,439 (225,902 to 465,937)	615,426 (512,392 to 736,176)	1,776,948 (1,502,662 to 2,033,800)	619,039 (470,445 to 727,081)	1,157,909 (944,719 to 1,345,677)	81.2 (57.6 to 112.6)	69.4 (39 to 189.8)	88.1 (50.9 to 127.8)
		North Africa and Middle East	YLLs	Age-standardized	571.7 (492.6 to 656.8)	420.5 (272.4 to 527.2)	724 (600.5 to 864.8)	437.4 (374 to 497.9)	313.4 (239.3 to 366.1)	558.3 (462.5 to 644.6)	-23.5 (-34.3 to -10.1)	-25.5 (-39.5 to 24.3)	-22.9 (-38 to -6)
		North Africa and Middle East	YLDs	All Ages	334,297 (273,977 to 387,310)	164,712 (135,753 to 188,792)	169,585 (135,966 to 200,773)	984,888 (806,904 to 1,129,850)	454,543 (375,226 to 519,060)	530,345 (425,188 to 621,602)	194.6 (187.8 to 201.6)	176 (167.5 to 184.7)	212.7 (203.7 to 221.8)
		North Africa and Middle East	YLDs	Age-standardized	164.4 (134.4 to 189.1)	160.1 (132.2 to 183.5)	169 (135.3 to 198.5)	211.7 (173.3 to 241.9)	195.7 (160.8 to 222.7)	227.8 (183.1 to 266.7)	28.8 (25.9 to 31.5)	22.3 (19 to 25.7)	34.8 (31.1 to 38.4)
		North Africa and Middle East	Incidence	All Ages	232,496 (219,917 to 245,243)	100,164 (94,436 to 106,335)	132,332 (125,247 to 139,547)	704,341 (671,175 to 739,648)	277,525 (261,404 to 293,646)	426,816 (408,870 to 446,210)	202.9 (196.4 to 209.8)	177.1 (170.4 to 184)	222.5 (214.6 to 231.1)
		North Africa and Middle East	Incidence	Age-standardized	112.2 (105.5 to 119)	95.6 (89.3 to 102.1)	128.3 (121.4 to 135.7)	150.8 (143.1 to 158.7)	121.3 (113.9 to 128.9)	179.3 (171.1 to 187.9)	34.4 (31.5 to 37.4)	26.8 (23.8 to 29.9)	39.7 (36.3 to 43.3)
		North Africa and Middle East	Prevalence	All Ages	3,584,089 (3,404,769 to 3,768,097)	1,623,749 (1,538,103 to 1,709,707)	1,960,341 (1,865,634 to 2,060,495)	10,720,230 (10,262,918 to 11,204,177)	4,540,211 (4,315,629 to 4,780,162)	6,180,019 (5,938,386 to 6,435,666)	199.1 (194.2 to 204.6)	179.6 (174 to 185.5)	215.3 (208.8 to 222.3)
		North Africa and Middle East	Prevalence	Age-standardized	1787.6 (1690.9 to 1887.5)	1605.3 (1512 to 1704.5)	1971.1 (1867.8 to 2077)	2333.9 (2230.1 to 2443.6)	1981.2 (1876.6 to 2093.3)	2681.6 (2575.5 to 2793.8)	30.6 (28.2 to 33)	23.4 (20.8 to 26.1)	36 (33.2 to 39.3)
		North Africa and Middle East	Deaths	All Ages	43,173 (37,093 to 49,741)	16,206 (10,632 to 20,267)	26,967 (22,367 to 32,150)	89,920 (76,785 to 101,801)	32,684 (24,831 to 37,962)	57,236 (48,001 to 65,643)	108.3 (78.2 to 145.3)	101.7 (59.7 to 226.9)	112.2 (71.5 to 159.1)
		North Africa and Middle East	Deaths	Age-standardized	31.8 (26.9 to 36.7)	23.8 (16 to 29.8)	40.2 (32.9 to 48.1)	26.1 (22.2 to 29.5)	19.5 (14.7 to 22.6)	32.6 (27.5 to 37.1)	-18 (-30.9 to -2.8)	-18.2 (-35.4 to 28.7)	-18.9 (-34.3 to 0.3)
		North Africa and Middle East	DALYs	All Ages	1,315,162 (1,164,472 to 1,461,299)	530,151 (392,277 to 640,540)	785,011 (676,740 to 906,107)	2,761,836 (2,436,839 to 3,062,623)	1,073,582 (901,530 to 1,205,699)	1,688,254 (1,452,958 to 1,897,561)	110 (89.9 to 134.5)	102.5 (74.4 to 184.3)	115.1 (83.1 to 147.6)
		North Africa and Middle East	DALYs	Age-standardized	736.1 (648.7 to 818.5)	580.6 (432.2 to 691.8)	893 (765.6 to 1034.4)	649.1 (574.6 to 717.7)	509.1 (430.2 to 569.2)	786.2 (686.7 to 883)	-11.8 (-21.1 to -0.9)	-12.3 (-25 to 23.4)	-12 (-25.4 to 3.1)
		North Africa and Middle East	YLLs	All Ages	980,865 (842,403 to 1,120,521)	365,439 (225,902 to 465,937)	615,426 (512,392 to 736,176)	1,776,948 (1,502,662 to 2,033,800)	619,039 (470,445 to 727,081)	1,157,909 (944,719 to 1,345,677)	81.2 (57.6 to 112.6)	69.4 (39 to 189.8)	88.1 (50.9 to 127.8)
		North Africa and Middle East	YLLs	Age-standardized	571.7 (492.6 to 656.8)	420.5 (272.4 to 527.2)	724 (600.5 to 864.8)	437.4 (374 to 497.9)	313.4 (239.3 to 366.1)	558.3 (462.5 to 644.6)	-23.5 (-34.3 to -10.1)	-25.5 (-39.5 to 24.3)	-22.9 (-38 to -6)
		North Africa and Middle East	YLDs	All Ages	334,297 (273,977 to 387,310)	164,712 (135,753 to 188,792)	169,585 (135,966 to 200,773)	984,888 (806,904 to 1,129,850)	454,543 (375,226 to 519,060)	530,345 (425,188 to 621,602)	194.6 (187.8 to 201.6)	176 (167.5 to 184.7)	212.7 (203.7 to 221.8)
		North Africa and Middle East	YLDs	Age-standardized	164.4 (134.4 to 189.1)	160.1 (132.2 to 183.5)	169 (135.3 to 198.5)	211.7 (173.3 to 241.9)	195.7 (160.8 to 222.7)	227.8 (183.1 to 266.7)	28.8 (25.9 to 31.5)	22.3 (19 to 25.7)	34.8 (31.1 to 38.4)
North Africa and Middle East	North Africa and Middle East	Afghanistan	Incidence	All Ages	9,158 (8,425 to 9,973)	4,998 (4,587 to 5,452)	4,160 (3,788 to 4,588)	27,037 (25,215 to 28,840)	14,970 (13,882 to 16,042)	12,067 (11,255 to 12,984)	195.2 (177.9 to 212)	199.5 (181.7 to 217.5)	190.1 (165.8 to 215.5)
North Africa and Middle East	North Africa and Middle East	Afghanistan	Incidence	Age-standardized	118.4 (109.3 to 128.9)	128.6 (118.4 to 139.4)	107.1 (97.7 to 118.1)	137.9 (128.9 to 147.6)	153.8 (143.1 to 165.3)	122.1 (113.6 to 131.9)	16.5 (12.1 to 20.5)	19.6 (14.4 to 25)	14 (7 to 20.5)
North Africa and Middle East	North Africa and Middle East	Afghanistan	Prevalence	All Ages	134,721 (124,810 to 145,783)	75,137 (69,174 to 81,514)	59,585 (54,696 to 65,144)	371,175 (349,917 to 393,665)	205,716 (191,824 to 219,561)	165,459 (155,088 to 176,162)	175.5 (162.5 to 188.5)	173.8 (159.8 to 188.1)	177.7 (159.2 to 195.3)
North Africa and Middle East	North Africa and Middle East	Afghanistan	Prevalence	Age-standardized	1764 (1635.3 to 1913.3)	1981.7 (1822.7 to 2153.8)	1557.9 (1434 to 1703.4)	2141.4 (1998 to 2291.4)	2413.1 (2233.2 to 2598.5)	1845 (1715.5 to 1987.7)	21.4 (16.8 to 25.9)	21.8 (16 to 27.5)	18.4 (11.7 to 25)
North Africa and Middle East	North Africa and Middle East	Afghanistan	Deaths	All Ages	2,877 (1,624 to 3,849)	1,150 (416 to 1,860)	1,727 (950 to 2,576)	3,932 (2,506 to 5,144)	1,935 (921 to 2,908)	1,997 (1,266 to 2,724)	36.7 (2.7 to 81.1)	68.2 (20.7 to 142.2)	15.7 (-13.9 to 63.3)
North Africa and Middle East	North Africa and Middle East	Afghanistan	Deaths	Age-standardized	48 (25.8 to 64.5)	40.1 (15.1 to 63.9)	54.9 (29.1 to 81)	40.4 (24.2 to 52.6)	38 (17.8 to 57.1)	42.9 (26.4 to 58.4)	-15.7 (-36.2 to 7.8)	-5.3 (-32.6 to 32.7)	-21.9 (-41.3 to 7.5)
North Africa and Middle East	North Africa and Middle East	Afghanistan	DALYs	All Ages	82,231 (55,748 to 107,273)	36,813 (17,120 to 55,609)	45,418 (28,646 to 65,723)	137,887 (100,493 to 173,594)	71,917 (44,976 to 97,362)	65,970 (47,980 to 86,641)	67.7 (31.9 to 115.5)	95.4 (48.1 to 171.1)	45.3 (11.6 to 98.2)
North Africa and Middle East	North Africa and Middle East	Afghanistan	DALYs	Age-standardized	1122.4 (745.1 to 1458.8)	1017.2 (476.3 to 1521.5)	1211.2 (752.9 to 1731.8)	964.5 (681.8 to 1203.2)	967.6 (572.1 to 1340.4)	962.5 (671.8 to 1257.3)	-14.1 (-32.8 to 7.4)	-4.9 (-28.5 to 28.8)	-20.5 (-39.4 to 5)
North Africa and Middle East	North Africa and Middle East	Afghanistan	YLLs	All Ages	69,680 (42,540 to 93,749)	29,420 (9,773 to 48,232)	40,259 (23,320 to 60,473)	103,177 (66,841 to 136,558)	51,544 (23,831 to 76,151)	51,634 (33,693 to 71,992)	48.1 (10.5 to 98.5)	75.2 (25.2 to 164.3)	28.3 (-6.1 to 83.7)
North Africa and Middle East	North Africa and Middle East	Afghanistan	YLLs	Age-standardized	959.4 (575.9 to 1299.1)	824.7 (285.6 to 1324.9)	1077 (617 to 1591.1)	768 (486.5 to 1003.9)	735 (347 to 1106.5)	804.9 (508.4 to 1105.3)	-19.9 (-41.1 to 4.3)	-10.9 (-37.1 to 31.8)	-25.3 (-44.9 to 3.7)
North Africa and Middle East	North Africa and Middle East	Afghanistan	YLDs	All Ages	12,552 (10,042 to 14,648)	7,393 (5,879 to 8,718)	5,159 (4,003 to 6,237)	34,710 (27,971 to 40,252)	20,373 (16,412 to 23,916)	14,337 (11,261 to 17,225)	176.5 (159.4 to 196.1)	175.6 (155.3 to 197.4)	177.9 (151.3 to 205.6)
North Africa and Middle East	North Africa and Middle East	Afghanistan	YLDs	Age-standardized	163 (130.2 to 189.8)	192.6 (153.3 to 226.6)	134.2 (104.5 to 161)	196.5 (157.7 to 227.8)	232.6 (187.3 to 270.9)	157.6 (124.2 to 188.7)	20.6 (14.9 to 27.4)	20.8 (13.4 to 29)	17.5 (8.6 to 26.9)
North Africa and Middle East	North Africa and Middle East	Algeria	Incidence	All Ages	13,088 (12,261 to 14,003)	5,663 (5,296 to 6,054)	7,425 (6,930 to 7,989)	38,616 (36,390 to 41,125)	15,888 (14,860 to 17,093)	22,728 (21,354 to 24,294)	195 (181.4 to 208.4)	180.6 (164.6 to 197.2)	206.1 (189.4 to 223.3)
North Africa and Middle East	North Africa and Middle East	Algeria	Incidence	Age-standardized	89.7 (84.1 to 95.8)	72.8 (67.8 to 78.3)	105.5 (98.6 to 113.1)	113.2 (106.8 to 120.5)	93.8 (87.8 to 100.6)	131.5 (123.5 to 140.6)	26.3 (21.5 to 31.1)	28.8 (22.1 to 35.5)	24.6 (18.4 to 31.2)
North Africa and Middle East	North Africa and Middle East	Algeria	Prevalence	All Ages	210,235 (196,977 to 222,964)	97,958 (91,658 to 104,198)	112,277 (104,822 to 119,452)	628,753 (590,277 to 667,208)	279,842 (262,719 to 297,406)	348,911 (326,977 to 371,769)	199.1 (188.3 to 209.9)	185.7 (172.7 to 199.2)	210.8 (196.5 to 226)
North Africa and Middle East	North Africa and Middle East	Algeria	Prevalence	Age-standardized	1442.2 (1355.9 to 1534.2)	1282.2 (1198 to 1368.2)	1587 (1484.1 to 1697.4)	1787.8 (1682 to 1899.8)	1577.5 (1483 to 1678.9)	1975.9 (1857.5 to 2103.7)	24 (19.8 to 28.1)	23 (17.7 to 28.8)	24.5 (18.6 to 30)
North Africa and Middle East	North Africa and Middle East	Algeria	Deaths	All Ages	2,164 (1,641 to 2,768)	820 (445 to 1,185)	1,344 (962 to 1,830)	4,905 (3,836 to 6,207)	1,902 (1,394 to 2,601)	3,003 (2,240 to 3,925)	126.7 (69.5 to 199.2)	132 (60 to 331)	123.5 (55.5 to 215.7)
North Africa and Middle East	North Africa and Middle East	Algeria	Deaths	Age-standardized	29.3 (22.7 to 37)	22.7 (11.9 to 32.4)	34.6 (25.5 to 45.9)	20.5 (16 to 25.7)	17.4 (12.8 to 23.7)	23.3 (17.5 to 30.4)	-29.9 (-45.6 to -8.7)	-23.4 (-46.6 to 40.5)	-32.6 (-51.3 to -7.9)
North Africa and Middle East	North Africa and Middle East	Algeria	DALYs	All Ages	68,470 (56,013 to 82,527)	29,655 (20,240 to 38,688)	38,815 (30,546 to 49,596)	148,252 (124,597 to 175,872)	63,223 (52,594 to 75,659)	85,029 (69,384 to 104,027)	116.5 (78.5 to 163)	113.2 (67.3 to 213)	119.1 (69.2 to 177.8)
North Africa and Middle East	North Africa and Middle East	Algeria	DALYs	Age-standardized	579.1 (472.6 to 697.4)	480.9 (314 to 634.1)	668.5 (525.4 to 845.3)	465.1 (390.8 to 548)	403.1 (333.1 to 492.2)	523.1 (424.8 to 642.4)	-19.7 (-34 to -1.9)	-16.2 (-34.8 to 27.6)	-21.7 (-40.1 to -1.1)
North Africa and Middle East	North Africa and Middle East	Algeria	YLLs	All Ages	48,493 (36,352 to 63,034)	19,637 (10,284 to 28,235)	28,856 (20,793 to 40,885)	89,410 (69,157 to 115,028)	35,083 (25,612 to 47,648)	54,327 (40,210 to 71,846)	84.4 (36.1 to 146.4)	78.7 (24.3 to 237.9)	88.3 (29.5 to 165.9)
North Africa and Middle East	North Africa and Middle East	Algeria	YLLs	Age-standardized	444.6 (341.1 to 566.7)	352.4 (183.5 to 502.6)	529 (385.8 to 715.2)	299.8 (234.2 to 381.1)	246.5 (185.9 to 331.3)	350.5 (259.7 to 457.9)	-32.6 (-49 to -10.7)	-30.1 (-50.8 to 28.4)	-33.7 (-53 to -8.8)
North Africa and Middle East	North Africa and Middle East	Algeria	YLDs	All Ages	19,977 (16,167 to 23,404)	10,018 (8,033 to 11,722)	9,959 (7,874 to 12,182)	58,843 (46,990 to 68,334)	28,141 (22,556 to 32,850)	30,702 (24,245 to 37,148)	194.6 (178 to 213.9)	180.9 (157.7 to 206.7)	208.3 (184.9 to 235.8)
North Africa and Middle East	North Africa and Middle East	Algeria	YLDs	Age-standardized	134.5 (108.2 to 156.2)	128.5 (103.6 to 149.1)	139.5 (110.3 to 168.5)	165.3 (132.8 to 191.4)	156.6 (127 to 182.3)	172.7 (136.5 to 206.8)	22.9 (17.1 to 29.5)	21.9 (13.6 to 30.7)	23.8 (15.6 to 32.8)
North Africa and Middle East	North Africa and Middle East	Bahrain	Incidence	All Ages	409 (384 to 436)	179 (167 to 192)	230 (214 to 247)	1,464 (1,349 to 1,581)	527 (478 to 574)	937 (861 to 1,016)	257.8 (238 to 281.3)	194.3 (173.2 to 214.8)	307.2 (278.7 to 339)
North Africa and Middle East	North Africa and Middle East	Bahrain	Incidence	Age-standardized	207.3 (196.9 to 218.9)	197.7 (185.4 to 210.1)	218.6 (207.1 to 230.4)	157.2 (147.4 to 167.2)	140.8 (129.9 to 152.3)	171.6 (161.1 to 182.2)	-24.2 (-27.1 to -21.2)	-28.8 (-33 to -25)	-21.5 (-25.3 to -17.4)
North Africa and Middle East	North Africa and Middle East	Bahrain	Prevalence	All Ages	5,335 (4,908 to 5,750)	2,401 (2,211 to 2,581)	2,934 (2,657 to 3,227)	20,177 (18,350 to 22,134)	7,924 (7,206 to 8,682)	12,253 (10,938 to 13,627)	278.2 (258.5 to 299.1)	230.1 (209.8 to 251.9)	317.6 (288.4 to 349.3)
North Africa and Middle East	North Africa and Middle East	Bahrain	Prevalence	Age-standardized	2584.5 (2430.2 to 2743.5)	2633.9 (2458.6 to 2825.2)	2589.4 (2435.9 to 2757.4)	2013.4 (1880.4 to 2151.1)	1984.2 (1825.8 to 2141.3)	2091.1 (1943.2 to 2246.8)	-22.1 (-25.2 to -18.8)	-24.7 (-29 to -20.3)	-19.2 (-23.3 to -14.6)
North Africa and Middle East	North Africa and Middle East	Bahrain	Deaths	All Ages	58 (49 to 67)	22 (16 to 27)	36 (30 to 42)	111 (88 to 142)	44 (34 to 63)	67 (53 to 87)	92 (47.8 to 161.4)	97.4 (39.6 to 226)	88.6 (42 to 155.5)
North Africa and Middle East	North Africa and Middle East	Bahrain	Deaths	Age-standardized	60.5 (50.6 to 69.2)	46.7 (31.4 to 57.3)	77.3 (65.2 to 89.4)	28.9 (23.5 to 35.7)	24.5 (19.4 to 33.2)	33.4 (26.4 to 41.4)	-52.3 (-62.3 to -37.4)	-47.5 (-61.8 to -18.3)	-56.8 (-66.5 to -44.2)
North Africa and Middle East	North Africa and Middle East	Bahrain	DALYs	All Ages	1,707 (1,503 to 1,914)	714 (581 to 828)	993 (862 to 1,132)	4,014 (3,426 to 4,797)	1,601 (1,335 to 2,081)	2,413 (2,035 to 2,913)	135.1 (97.2 to 187.6)	124.3 (80.5 to 207.9)	142.9 (100.7 to 200.9)
North Africa and Middle East	North Africa and Middle East	Bahrain	DALYs	Age-standardized	1147.5 (996.2 to 1291)	973.2 (749.9 to 1141.4)	1349.5 (1166.5 to 1546.1)	561.7 (484.6 to 662.1)	519.8 (439.4 to 659.5)	611.3 (519 to 723.5)	-51.1 (-59 to -39.5)	-46.6 (-57.7 to -25.4)	-54.7 (-63.1 to -44.2)
North Africa and Middle East	North Africa and Middle East	Bahrain	YLLs	All Ages	1,209 (1,029 to 1,413)	473 (340 to 577)	735 (623 to 872)	2,153 (1,680 to 2,864)	811 (611 to 1,260)	1,342 (1,044 to 1,758)	78.1 (33.8 to 149.7)	71.4 (19.1 to 198.3)	82.4 (35.6 to 151.4)
North Africa and Middle East	North Africa and Middle East	Bahrain	YLLs	Age-standardized	912.3 (766.5 to 1059.2)	718 (500.5 to 876.8)	1127.4 (951 to 1314.8)	380.9 (307.3 to 478.4)	327.9 (254 to 462.3)	433.6 (344 to 541.1)	-58.3 (-67.5 to -44)	-54.3 (-67.3 to -25.6)	-61.5 (-70.8 to -49.4)
North Africa and Middle East	North Africa and Middle East	Bahrain	YLDs	All Ages	499 (404 to 584)	241 (194 to 281)	258 (202 to 313)	1,862 (1,497 to 2,203)	790 (628 to 944)	1,072 (829 to 1,318)	273.3 (244.3 to 301.7)	228.3 (198.5 to 258.6)	315.3 (272.3 to 364)
North Africa and Middle East	North Africa and Middle East	Bahrain	YLDs	Age-standardized	235.2 (192 to 270.5)	255.2 (207.2 to 295.5)	222.1 (178 to 262.4)	180.8 (146.8 to 208)	192 (156.3 to 223.8)	177.7 (141.4 to 210)	-23.1 (-26.9 to -19.3)	-24.8 (-30 to -19.3)	-20 (-25.2 to -14.5)
North Africa and Middle East	North Africa and Middle East	Egypt	Incidence	All Ages	36,889 (34,700 to 39,217)	14,485 (13,590 to 15,517)	22,404 (21,071 to 23,866)	119,400 (112,594 to 126,178)	42,690 (39,697 to 45,961)	76,710 (72,249 to 81,274)	223.7 (211.8 to 236.8)	194.7 (180.7 to 210)	242.4 (224.6 to 261.2)
North Africa and Middle East	North Africa and Middle East	Egypt	Incidence	Age-standardized	102.5 (96 to 109.3)	77 (71.5 to 83)	128.1 (120 to 137.1)	165.5 (155.8 to 175.2)	122.9 (115 to 131.8)	201.2 (189.4 to 213.8)	61.5 (55.9 to 68.1)	59.7 (52.2 to 67.4)	57 (48.9 to 65.3)
North Africa and Middle East	North Africa and Middle East	Egypt	Prevalence	All Ages	588,953 (556,275 to 622,076)	254,297 (239,785 to 269,284)	334,656 (314,614 to 355,483)	1,881,259 (1,787,452 to 1,965,262)	740,196 (688,598 to 788,632)	1,141,063 (1,082,748 to 1,195,022)	219.4 (207.4 to 233.3)	191.1 (177.3 to 207.3)	241 (223 to 260.9)
North Africa and Middle East	North Africa and Middle East	Egypt	Prevalence	Age-standardized	1685.4 (1581.6 to 1790.5)	1406.5 (1319 to 1504.7)	1967.7 (1839.7 to 2103.6)	2731 (2588.1 to 2872)	2194.2 (2045.6 to 2347.1)	3135.7 (2965.6 to 3300.9)	62 (55.6 to 68.9)	56 (48.7 to 64.2)	59.4 (50.7 to 68.9)
North Africa and Middle East	North Africa and Middle East	Egypt	Deaths	All Ages	6,881 (4,992 to 8,469)	2,805 (1,785 to 3,680)	4,075 (2,703 to 5,177)	14,052 (9,238 to 18,627)	4,932 (3,268 to 7,232)	9,120 (4,617 to 12,701)	104.2 (48.9 to 176.9)	75.8 (18.8 to 213.8)	123.8 (45.4 to 219.2)
North Africa and Middle East	North Africa and Middle East	Egypt	Deaths	Age-standardized	28.9 (20.9 to 37.2)	24 (15.9 to 32.6)	33.9 (22.4 to 43.6)	28.2 (18.5 to 36.8)	25.7 (18.1 to 38)	31 (15.8 to 43.4)	-2.6 (-27.6 to 30.7)	7.2 (-26.6 to 84)	-8.4 (-39.5 to 29.1)
North Africa and Middle East	North Africa and Middle East	Egypt	DALYs	All Ages	230,577 (184,415 to 266,825)	97,204 (68,372 to 120,084)	133,373 (99,466 to 162,483)	499,452 (378,041 to 615,259)	186,097 (143,888 to 245,163)	313,355 (205,340 to 403,009)	116.6 (75.4 to 169.1)	91.5 (43.1 to 189.1)	134.9 (78.9 to 204)
North Africa and Middle East	North Africa and Middle East	Egypt	DALYs	Age-standardized	717.6 (560.9 to 836.7)	592.1 (433.6 to 729)	843.5 (618.9 to 1026.2)	776.4 (583.1 to 954.9)	649.6 (505.1 to 861)	888.3 (580.9 to 1140.7)	8.2 (-12.2 to 34.3)	9.7 (-16.2 to 64.8)	5.3 (-19.8 to 36.9)
North Africa and Middle East	North Africa and Middle East	Egypt	YLLs	All Ages	174,978 (127,923 to 210,357)	71,297 (42,560 to 93,915)	103,682 (69,365 to 131,978)	324,041 (207,945 to 435,237)	111,320 (73,038 to 166,939)	212,722 (103,929 to 298,350)	85.2 (34.4 to 150.3)	56.1 (1.5 to 185)	105.2 (31.3 to 193.2)
North Africa and Middle East	North Africa and Middle East	Egypt	YLLs	Age-standardized	560.5 (407.3 to 681.2)	450.8 (285.8 to 588.9)	669.9 (446 to 843.4)	525.5 (342.2 to 698.6)	431.7 (291.6 to 634)	613.7 (305.8 to 861.6)	-6.2 (-31.2 to 26.1)	-4.3 (-34.9 to 72.3)	-8.4 (-41.3 to 30.8)
North Africa and Middle East	North Africa and Middle East	Egypt	YLDs	All Ages	55,598 (44,465 to 65,640)	25,907 (20,743 to 30,417)	29,692 (23,437 to 35,784)	175,410 (140,113 to 203,726)	74,777 (59,829 to 87,160)	100,633 (79,159 to 120,233)	215.5 (196.5 to 236.3)	188.6 (164.1 to 213.3)	238.9 (212.8 to 267.7)
North Africa and Middle East	North Africa and Middle East	Egypt	YLDs	Age-standardized	157.2 (126.1 to 183.7)	141.2 (113.7 to 165.2)	173.6 (137 to 208.7)	250.9 (201 to 290.6)	217.9 (174.9 to 253.5)	274.7 (218.1 to 327.3)	59.6 (50.9 to 69.2)	54.3 (42.8 to 65.5)	58.2 (47.5 to 70.7)
North Africa and Middle East	North Africa and Middle East	Iran (Islamic Republic of)	Incidence	All Ages	32,052 (29,255 to 35,372)	14,421 (13,206 to 15,780)	17,630 (15,955 to 19,602)	103,602 (94,771 to 113,265)	45,891 (41,883 to 50,306)	57,711 (52,839 to 62,967)	223.2 (202.7 to 244.8)	218.2 (198.1 to 239.5)	227.3 (206.4 to 250.3)
North Africa and Middle East	North Africa and Middle East	Iran (Islamic Republic of)	Incidence	Age-standardized	99.2 (89.5 to 109.8)	90.9 (82 to 100.8)	106.9 (96.4 to 118.3)	140.6 (127.8 to 154.5)	129.8 (117.8 to 142.9)	151.7 (137.9 to 166.6)	41.7 (35 to 47.5)	42.8 (35.4 to 49.9)	41.9 (35.2 to 47.9)
North Africa and Middle East	North Africa and Middle East	Iran (Islamic Republic of)	Prevalence	All Ages	509,008 (465,525 to 555,495)	232,079 (214,369 to 251,505)	276,928 (251,490 to 304,832)	1,562,415 (1,446,402 to 1,688,536)	686,719 (633,599 to 744,997)	875,696 (806,519 to 949,356)	207 (194.3 to 222)	195.9 (183.9 to 210.2)	216.2 (200.9 to 233.4)
North Africa and Middle East	North Africa and Middle East	Iran (Islamic Republic of)	Prevalence	Age-standardized	1640 (1508.2 to 1794.9)	1520.6 (1396.7 to 1661.9)	1751.8 (1600.2 to 1933.5)	2055.5 (1891.4 to 2232.6)	1832.2 (1687.7 to 1989.4)	2282.8 (2096.2 to 2487.5)	25.3 (20.9 to 29.8)	20.5 (15.8 to 25.3)	30.3 (25.4 to 35.3)
North Africa and Middle East	North Africa and Middle East	Iran (Islamic Republic of)	Deaths	All Ages	4,230 (3,709 to 5,228)	1,641 (1,075 to 2,305)	2,588 (2,216 to 3,296)	12,557 (10,993 to 13,621)	4,855 (3,932 to 5,781)	7,701 (6,760 to 8,327)	196.9 (139.3 to 246.1)	195.9 (111.8 to 437.2)	197.5 (126 to 253.6)
North Africa and Middle East	North Africa and Middle East	Iran (Islamic Republic of)	Deaths	Age-standardized	23.4 (20.1 to 29.4)	19 (12.8 to 27.4)	28 (23.5 to 35.3)	20.3 (17.7 to 22.1)	16.4 (12.9 to 19.6)	24.2 (21.3 to 26.3)	-13 (-30.5 to 1.7)	-13.9 (-38.3 to 49.6)	-13.4 (-32.9 to 2.9)
North Africa and Middle East	North Africa and Middle East	Iran (Islamic Republic of)	DALYs	All Ages	153,371 (138,121 to 176,277)	63,198 (49,406 to 77,006)	90,173 (79,421 to 109,525)	372,569 (338,458 to 404,911)	151,368 (131,933 to 170,196)	221,200 (199,050 to 240,391)	142.9 (109 to 168)	139.5 (101.2 to 250.6)	145.3 (97.6 to 179.1)
North Africa and Middle East	North Africa and Middle East	Iran (Islamic Republic of)	DALYs	Age-standardized	555.9 (495 to 646.6)	466.8 (365.9 to 593)	641.6 (565.9 to 776.5)	517.2 (471 to 560.8)	427.5 (371.6 to 480.7)	607.8 (549.4 to 662)	-7 (-20.6 to 3.2)	-8.4 (-26.5 to 34.4)	-5.3 (-23.3 to 7.8)
North Africa and Middle East	North Africa and Middle East	Iran (Islamic Republic of)	YLLs	All Ages	105,098 (93,069 to 126,741)	39,544 (24,045 to 53,086)	65,554 (56,326 to 84,296)	227,958 (205,149 to 247,566)	83,425 (68,878 to 100,095)	144,533 (128,093 to 156,202)	116.9 (74.4 to 150.3)	111 (59.3 to 313.1)	120.5 (64 to 163.8)
North Africa and Middle East	North Africa and Middle East	Iran (Islamic Republic of)	YLLs	Age-standardized	404.4 (353.9 to 496.7)	317 (206.1 to 445)	489 (418.4 to 621.4)	329.1 (296.7 to 356.4)	248.8 (203.9 to 296.9)	409.7 (364.4 to 441.8)	-18.6 (-33.8 to -5.9)	-21.5 (-41.8 to 44.9)	-16.2 (-36.4 to -0.3)
North Africa and Middle East	North Africa and Middle East	Iran (Islamic Republic of)	YLDs	All Ages	48,273 (39,156 to 56,242)	23,654 (19,298 to 27,733)	24,619 (19,609 to 29,209)	144,611 (117,257 to 168,236)	67,944 (55,568 to 79,514)	76,667 (60,745 to 91,234)	199.6 (186.6 to 214.4)	187.2 (173.9 to 202.6)	211.4 (195.6 to 230.1)
North Africa and Middle East	North Africa and Middle East	Iran (Islamic Republic of)	YLDs	Age-standardized	151.5 (123.3 to 175.9)	149.9 (121.9 to 175)	152.6 (121.6 to 181.5)	188.1 (152.9 to 218.4)	178.7 (145 to 208.3)	198.1 (157.2 to 235.4)	24.2 (19.9 to 28.6)	19.2 (14.3 to 24.2)	29.8 (24.5 to 35.3)
North Africa and Middle East	North Africa and Middle East	Iraq	Incidence	All Ages	9,136 (8,639 to 9,656)	4,047 (3,814 to 4,287)	5,089 (4,776 to 5,427)	17,176 (16,033 to 18,467)	8,830 (8,205 to 9,535)	8,345 (7,718 to 9,020)	88 (78.9 to 97.9)	118.2 (105 to 132.3)	64 (54.3 to 74.4)
North Africa and Middle East	North Africa and Middle East	Iraq	Incidence	Age-standardized	87.8 (82.7 to 93.5)	71.2 (66.5 to 76.5)	105.9 (99.3 to 112.8)	58.8 (55.6 to 62.5)	58.4 (54.6 to 62.7)	59.1 (55.7 to 63)	-33.1 (-35.8 to -30.2)	-17.9 (-22.2 to -13.1)	-44.2 (-47.1 to -41)
North Africa and Middle East	North Africa and Middle East	Iraq	Prevalence	All Ages	144,140 (135,387 to 152,870)	69,636 (65,804 to 73,283)	74,503 (68,773 to 80,380)	308,960 (281,559 to 338,889)	166,068 (151,007 to 182,380)	142,891 (127,392 to 159,340)	114.3 (99.5 to 129.9)	138.5 (117.6 to 159.9)	91.8 (76.1 to 108.8)
North Africa and Middle East	North Africa and Middle East	Iraq	Prevalence	Age-standardized	1481.2 (1390.1 to 1578)	1373.1 (1287.9 to 1461.1)	1608.6 (1494.6 to 1733.4)	1053.5 (976.7 to 1139.4)	1109.4 (1024.4 to 1205.1)	1002.5 (921 to 1090.3)	-28.9 (-32.7 to -24.4)	-19.2 (-24.9 to -12.9)	-37.7 (-41.9 to -33.2)
North Africa and Middle East	North Africa and Middle East	Iraq	Deaths	All Ages	785 (624 to 1,000)	262 (166 to 376)	523 (395 to 757)	1,711 (1,343 to 2,143)	602 (448 to 886)	1,110 (853 to 1,396)	118 (51.6 to 196)	129.6 (53.3 to 277)	112.2 (27.1 to 215.9)
North Africa and Middle East	North Africa and Middle East	Iraq	Deaths	Age-standardized	11.7 (9.3 to 14.7)	7.3 (4.6 to 10.8)	16.8 (13 to 23.5)	9.8 (7.8 to 12.6)	6.5 (4.9 to 10.1)	13.8 (10.8 to 17.3)	-15.9 (-41 to 12.6)	-10.9 (-39.6 to 43.3)	-17.7 (-48.7 to 20.3)
North Africa and Middle East	North Africa and Middle East	Iraq	DALYs	All Ages	29,997 (25,199 to 35,564)	12,800 (10,226 to 15,316)	17,197 (13,876 to 22,964)	65,299 (54,269 to 76,574)	29,570 (24,487 to 35,483)	35,729 (29,229 to 42,619)	117.7 (72.6 to 163.8)	131 (88.9 to 191)	107.8 (41.3 to 170.6)
North Africa and Middle East	North Africa and Middle East	Iraq	DALYs	Age-standardized	345.2 (289.3 to 412.9)	271.7 (213.8 to 332.4)	427.2 (345.5 to 569.5)	265.2 (221.3 to 311.2)	221.5 (184.6 to 271)	315.7 (258.3 to 378.6)	-23.2 (-40.1 to -7)	-18.5 (-34.6 to 5.5)	-26.1 (-50 to -1.3)
North Africa and Middle East	North Africa and Middle East	Iraq	YLLs	All Ages	16,521 (12,667 to 22,065)	5,792 (3,585 to 7,936)	10,728 (7,783 to 16,253)	35,971 (27,617 to 45,322)	12,795 (9,221 to 17,196)	23,176 (17,443 to 29,615)	117.7 (42.1 to 205.6)	120.9 (41.1 to 278.2)	116 (19.5 to 232.9)
North Africa and Middle East	North Africa and Middle East	Iraq	YLLs	Age-standardized	209.1 (163.3 to 275.8)	136 (84.6 to 188.4)	289.2 (215.5 to 430.8)	166.7 (129 to 207.2)	111.3 (81.8 to 158.7)	228.5 (175.1 to 287.8)	-20.3 (-46.5 to 10.4)	-18.2 (-47.3 to 39.5)	-21 (-54.2 to 21)
North Africa and Middle East	North Africa and Middle East	Iraq	YLDs	All Ages	13,477 (10,883 to 15,752)	7,008 (5,568 to 8,186)	6,469 (5,100 to 7,748)	29,328 (23,241 to 34,967)	16,775 (13,369 to 20,015)	12,553 (9,636 to 15,516)	117.6 (97.9 to 137.9)	139.4 (112.3 to 170.7)	94.1 (71.5 to 118.2)
North Africa and Middle East	North Africa and Middle East	Iraq	YLDs	Age-standardized	136.1 (110.6 to 158.3)	135.8 (109.3 to 158.4)	138 (109.9 to 164.1)	98.5 (79.3 to 115.3)	110.2 (87.9 to 129.2)	87.2 (68.2 to 105.9)	-27.6 (-32.9 to -22.3)	-18.8 (-26.2 to -10.1)	-36.8 (-42.7 to -30.7)
North Africa and Middle East	North Africa and Middle East	Jordan	Incidence	All Ages	2,079 (1,962 to 2,197)	838 (789 to 894)	1,241 (1,163 to 1,311)	7,753 (7,312 to 8,214)	2,715 (2,517 to 2,919)	5,039 (4,762 to 5,361)	272.9 (257.1 to 288.5)	223.8 (206.1 to 241.5)	306.2 (285.5 to 328.2)
North Africa and Middle East	North Africa and Middle East	Jordan	Incidence	Age-standardized	103 (97.4 to 108.8)	80.1 (75.4 to 85.4)	125.3 (118 to 132.6)	107 (101.4 to 113.2)	75.7 (70.9 to 81)	135.2 (128 to 143.5)	3.9 (-0.6 to 8.3)	-5.5 (-10.7 to -0.5)	7.9 (2.1 to 13.7)
North Africa and Middle East	North Africa and Middle East	Jordan	Prevalence	All Ages	31,615 (29,675 to 33,533)	13,566 (12,645 to 14,533)	18,049 (16,843 to 19,199)	119,919 (110,359 to 129,523)	47,473 (42,930 to 52,111)	72,446 (67,061 to 78,341)	279.3 (258.1 to 301.6)	249.9 (223.8 to 276)	301.4 (275 to 329.4)
North Africa and Middle East	North Africa and Middle East	Jordan	Prevalence	Age-standardized	1744.3 (1641.6 to 1850.1)	1481.2 (1389.9 to 1590.6)	2004.5 (1878.5 to 2124.1)	1602.3 (1504.3 to 1706.6)	1271.9 (1173 to 1373.7)	1899.3 (1780.8 to 2029.7)	-8.1 (-12.3 to -3.6)	-14.1 (-19.3 to -8.8)	-5.2 (-10.4 to 0.7)
North Africa and Middle East	North Africa and Middle East	Jordan	Deaths	All Ages	245 (193 to 294)	90 (55 to 115)	155 (120 to 199)	555 (450 to 680)	154 (118 to 206)	402 (305 to 515)	127.1 (67.5 to 205.6)	70.9 (16.3 to 220.4)	159.7 (68.8 to 272)
North Africa and Middle East	North Africa and Middle East	Jordan	Deaths	Age-standardized	25.9 (20.7 to 31.2)	19.3 (12.3 to 24.8)	32.8 (26 to 41.4)	12.2 (9.9 to 15)	7.5 (5.8 to 10.3)	16.3 (12.4 to 21)	-53 (-64.7 to -37.4)	-61 (-73.2 to -29.3)	-50.2 (-66.8 to -29.6)
North Africa and Middle East	North Africa and Middle East	Jordan	DALYs	All Ages	8,223 (6,955 to 9,496)	3,293 (2,475 to 3,898)	4,929 (4,061 to 5,946)	22,410 (19,437 to 25,914)	7,779 (6,582 to 9,180)	14,631 (12,343 to 17,479)	172.5 (126.9 to 225)	136.2 (88.7 to 226.9)	196.8 (128.1 to 270.8)
North Africa and Middle East	North Africa and Middle East	Jordan	DALYs	Age-standardized	597.2 (502.3 to 693.8)	463.7 (337.3 to 555.2)	733.6 (602 to 896.3)	341.2 (297.1 to 392.9)	238.8 (202.8 to 284.4)	434.8 (366 to 518.8)	-42.9 (-53.4 to -30.7)	-48.5 (-59.6 to -25.5)	-40.7 (-55.6 to -24.1)
North Africa and Middle East	North Africa and Middle East	Jordan	YLLs	All Ages	5,236 (4,133 to 6,324)	1,912 (1,134 to 2,469)	3,324 (2,545 to 4,244)	11,194 (9,103 to 13,638)	2,962 (2,246 to 3,979)	8,232 (6,241 to 10,606)	113.8 (54.8 to 188.7)	54.9 (4.7 to 208.1)	147.7 (60.1 to 255.1)
North Africa and Middle East	North Africa and Middle East	Jordan	YLLs	Age-standardized	435.4 (343.9 to 524.9)	316.2 (194.7 to 405.9)	557.1 (429.4 to 712.6)	194.1 (157.2 to 236.7)	112.3 (85.9 to 150.5)	268.9 (202.6 to 344.6)	-55.4 (-67.2 to -40)	-64.5 (-75.7 to -33.6)	-51.7 (-68.6 to -31)
North Africa and Middle East	North Africa and Middle East	Jordan	YLDs	All Ages	2,987 (2,412 to 3,511)	1,381 (1,110 to 1,636)	1,605 (1,267 to 1,934)	11,216 (9,073 to 13,350)	4,817 (3,874 to 5,744)	6,399 (5,030 to 7,764)	275.6 (247.2 to 305.6)	248.8 (218.2 to 290.9)	298.6 (259.9 to 338.1)
North Africa and Middle East	North Africa and Middle East	Jordan	YLDs	Age-standardized	161.8 (130 to 187.3)	147.4 (119 to 171.4)	176.5 (139.8 to 209.1)	147.1 (119.5 to 171.4)	126.6 (103.1 to 147.7)	165.9 (131.7 to 196.6)	-9.1 (-14.5 to -3.3)	-14.1 (-21.6 to -6.2)	-6 (-12.6 to 1.1)
North Africa and Middle East	North Africa and Middle East	Kuwait	Incidence	All Ages	603 (551 to 657)	241 (218 to 263)	361 (327 to 397)	1,955 (1,789 to 2,135)	741 (668 to 820)	1,213 (1,112 to 1,322)	224.4 (204.9 to 244.6)	207.1 (182.2 to 235)	235.9 (212.1 to 260.5)
North Africa and Middle East	North Africa and Middle East	Kuwait	Incidence	Age-standardized	52.7 (49.8 to 55.9)	44.4 (41.5 to 47.5)	58.5 (55.1 to 62.1)	54.5 (51.2 to 57.9)	41.5 (38.4 to 44.6)	62.9 (59 to 67)	3.4 (-0.2 to 7)	-6.7 (-10.6 to -2.3)	7.5 (2.8 to 12.5)
North Africa and Middle East	North Africa and Middle East	Kuwait	Prevalence	All Ages	11,102 (9,849 to 12,363)	4,573 (4,053 to 5,045)	6,529 (5,714 to 7,436)	39,087 (34,844 to 43,652)	16,912 (14,885 to 19,050)	22,175 (19,672 to 25,030)	252.1 (229.8 to 273.1)	269.8 (241.6 to 299.6)	239.6 (211.3 to 266.8)
North Africa and Middle East	North Africa and Middle East	Kuwait	Prevalence	Age-standardized	995 (920.7 to 1075.8)	977 (893.1 to 1059.1)	1023.3 (942.5 to 1112.1)	1023.1 (939.3 to 1115.6)	910.8 (830.5 to 999.2)	1089.9 (998.5 to 1191.8)	2.8 (-1.4 to 6.9)	-6.8 (-12 to -1.6)	6.5 (0.7 to 12.1)
North Africa and Middle East	North Africa and Middle East	Kuwait	Deaths	All Ages	38 (32 to 43)	11 (8 to 13)	27 (23 to 32)	148 (117 to 182)	34 (25 to 46)	115 (88 to 145)	289.4 (209.7 to 385.8)	208.6 (119.8 to 358.6)	321.8 (218.7 to 452)

Chronic obstructive pulmonary disease

7 GBD super region	21 GBD region	Location	Measure	Age	1990			2019			Percent change (1990 to 2019)		
					Both	Female	Male	Both	Female	Male	Both	Female	Male
North Africa and Middle East	North Africa and Middle East	Kuwait	Deaths	Age-standardized	9.6 (8 to 11)	6 (4.4 to 7.5)	13 (10.7 to 15.4)	8.3 (6.5 to 10.3)	4.5 (3.3 to 6.1)	10.8 (8.2 to 13.8)	-12.7 (-30.8 to 8.4)	-24.5 (-47.5 to 12.1)	-16.6 (-36.8 to 8.4)
North Africa and Middle East	North Africa and Middle East	Kuwait	DALYs	All Ages	1,850 (1,594 to 2,098)	696 (585 to 806)	1,155 (974 to 1,326)	6,186 (5,236 to 7,195)	2,343 (1,915 to 2,779)	3,843 (3,210 to 4,583)	234.3 (199 to 278.1)	236.7 (192.5 to 298.1)	232.8 (184.5 to 295.6)
North Africa and Middle East	North Africa and Middle East	Kuwait	DALYs	Age-standardized	245.1 (214.3 to 272.8)	195.5 (164.9 to 225.4)	287.1 (246.6 to 327.4)	215.7 (185.7 to 249.7)	157.7 (131.9 to 186.4)	251.9 (209.8 to 301.4)	-12 (-23.7 to 1.9)	-19.4 (-32 to -1.1)	-12.2 (-27 to 6.6)
North Africa and Middle East	North Africa and Middle East	Kuwait	YLLs	All Ages	797 (683 to 913)	224 (175 to 271)	573 (472 to 679)	2,498 (1,982 to 3,097)	611 (453 to 855)	1,887 (1,430 to 2,414)	213.6 (146.2 to 301.5)	172.9 (92.4 to 320.1)	229.5 (143.4 to 344.6)
North Africa and Middle East	North Africa and Middle East	Kuwait	YLLs	Age-standardized	152.5 (129.1 to 175.3)	97 (72.5 to 120.4)	196.9 (163.5 to 231.5)	121 (95.5 to 149.3)	65.9 (48.7 to 89)	156.7 (119 to 199.7)	-20.6 (-37.9 to 0.8)	-32 (-52.6 to 6.4)	-20.4 (-40.7 to 6.1)
North Africa and Middle East	North Africa and Middle East	Kuwait	YLDs	All Ages	1,054 (823 to 1,268)	472 (370 to 576)	582 (442 to 723)	3,687 (2,907 to 4,469)	1,732 (1,329 to 2,101)	1,955 (1,514 to 2,435)	250 (218.5 to 287.9)	267 (221.5 to 320)	236.1 (194.8 to 285.7)
North Africa and Middle East	North Africa and Middle East	Kuwait	YLDs	Age-standardized	92.6 (74 to 109)	98.6 (79 to 116.7)	90.2 (70.3 to 107.9)	94.7 (76 to 111.6)	91.8 (72.7 to 108.1)	95.2 (75.4 to 114.9)	2.3 (-4.4 to 9.5)	-6.9 (-15.4 to 2.4)	5.6 (-3.5 to 15.7)
North Africa and Middle East	North Africa and Middle East	Lebanon	Incidence	All Ages	2,855 (2,676 to 3,048)	1,250 (1,175 to 1,336)	1,605 (1,496 to 1,722)	9,481 (8,888 to 10,059)	4,797 (4,487 to 5,115)	4,684 (4,377 to 4,985)	232.1 (218.5 to 246.3)	283.8 (263.9 to 306.2)	191.8 (176.6 to 208.2)
North Africa and Middle East	North Africa and Middle East	Lebanon	Incidence	Age-standardized	120.8 (113.8 to 128.4)	101.7 (95.5 to 108.4)	141.2 (132.5 to 150.4)	182.2 (170.9 to 193)	169.5 (158.8 to 180.4)	197.2 (183.9 to 209.7)	50.8 (45.9 to 55.9)	66.6 (58.9 to 75.1)	39.7 (33.2 to 46.1)
North Africa and Middle East	North Africa and Middle East	Lebanon	Prevalence	All Ages	45,238 (42,396 to 48,187)	21,350 (19,987 to 22,745)	23,888 (22,317 to 25,663)	141,237 (131,990 to 150,458)	71,969 (66,885 to 77,024)	69,268 (64,766 to 73,847)	212.2 (200.8 to 224)	237.1 (221.1 to 253.7)	190 (175.2 to 203.7)
North Africa and Middle East	North Africa and Middle East	Lebanon	Prevalence	Age-standardized	1904.5 (1788.1 to 2030.3)	1739.5 (1629.1 to 1854.1)	2085.9 (1953.4 to 2231.4)	2723.1 (2548.3 to 2901.7)	2549.9 (2371 to 2726.5)	2935.4 (2743.1 to 3130)	43 (38.1 to 47.8)	46.6 (39.9 to 53.5)	40.7 (33.9 to 46.7)
North Africa and Middle East	North Africa and Middle East	Lebanon	Deaths	All Ages	381 (294 to 484)	152 (106 to 212)	228 (164 to 301)	850 (631 to 1,107)	321 (216 to 575)	529 (332 to 705)	123.3 (71.1 to 189.6)	111.1 (46.7 to 321.2)	131.5 (53.9 to 223.1)
North Africa and Middle East	North Africa and Middle East	Lebanon	Deaths	Age-standardized	21.5 (16.7 to 27.3)	16.6 (11.7 to 23)	27.1 (19.7 to 35.9)	16.8 (12.6 to 21.8)	11.5 (7.7 to 20.6)	23.5 (14.8 to 31.4)	-21.8 (-40.2 to 2)	-30.8 (-51.2 to 38.2)	-13.2 (-42.7 to 22.2)
North Africa and Middle East	North Africa and Middle East	Lebanon	DALYs	All Ages	11,965 (10,017 to 14,161)	5,093 (4,124 to 6,339)	6,872 (5,489 to 8,384)	27,283 (22,613 to 31,817)	12,005 (9,903 to 15,879)	15,278 (11,609 to 18,603)	128 (93 to 166.8)	135.7 (89.1 to 226.2)	122.3 (74.3 to 176.3)
North Africa and Middle East	North Africa and Middle East	Lebanon	DALYs	Age-standardized	541.9 (453.3 to 639.5)	447 (362.1 to 559)	643.8 (517 to 778.9)	525.6 (436.8 to 612.6)	424.2 (350.1 to 559.8)	650.1 (495 to 791.3)	-3 (-18.7 to 13.8)	-5.1 (-23.9 to 33.6)	1 (-20.4 to 26.3)
North Africa and Middle East	North Africa and Middle East	Lebanon	YLLs	All Ages	7,573 (5,847 to 9,737)	2,954 (2,057 to 4,147)	4,620 (3,286 to 6,101)	14,092 (10,325 to 18,272)	5,131 (3,486 to 9,083)	8,960 (5,547 to 12,125)	86.1 (39 to 144.9)	73.7 (20.6 to 239.1)	94 (29.1 to 169.6)
North Africa and Middle East	North Africa and Middle East	Lebanon	YLLs	Age-standardized	358.7 (278.6 to 456.9)	274.6 (191.3 to 384.5)	448.6 (321.6 to 591.6)	271.6 (199.8 to 351)	180.6 (122.8 to 321.8)	383 (236.3 to 519.1)	-24.3 (-43.5 to -0.1)	-34.2 (-54.2 to 28.4)	-14.6 (-42.8 to 19.2)
North Africa and Middle East	North Africa and Middle East	Lebanon	YLDs	All Ages	4,392 (3,520 to 5,120)	2,139 (1,714 to 2,528)	2,252 (1,773 to 2,686)	13,191 (10,539 to 15,310)	6,873 (5,509 to 8,120)	6,318 (4,982 to 7,481)	200.4 (175.8 to 228.4)	221.3 (186 to 263)	180.5 (153.6 to 213.9)
North Africa and Middle East	North Africa and Middle East	Lebanon	YLDs	Age-standardized	183.1 (147 to 212.4)	172.4 (138.9 to 202.9)	195.1 (154 to 232.9)	254 (203.1 to 294.5)	243.6 (195.1 to 287.7)	267.2 (210.8 to 315.8)	38.7 (26.9 to 50.6)	41.3 (26.2 to 58.8)	36.9 (23.7 to 52.7)
North Africa and Middle East	North Africa and Middle East	Libya	Incidence	All Ages	2,590 (2,449 to 2,746)	1,009 (952 to 1,071)	1,581 (1,480 to 1,681)	9,229 (8,598 to 9,830)	3,554 (3,284 to 3,830)	5,675 (5,291 to 6,054)	256.3 (240.9 to 273.5)	252.1 (230.3 to 274.5)	259 (241.1 to 279.8)
North Africa and Middle East	North Africa and Middle East	Libya	Incidence	Age-standardized	108.2 (101.1 to 115.4)	84 (78.5 to 90)	130.1 (121.3 to 139.7)	157 (147.1 to 167)	121.4 (113.1 to 130.8)	190.8 (178.7 to 203.3)	45 (39.9 to 50.6)	44.6 (37.2 to 52)	46.7 (39.7 to 54.4)
North Africa and Middle East	North Africa and Middle East	Libya	Prevalence	All Ages	39,402 (37,040 to 41,923)	16,217 (15,137 to 17,289)	23,185 (21,658 to 24,810)	139,857 (131,289 to 147,932)	58,197 (54,477 to 62,120)	81,661 (76,718 to 86,665)	255 (239.9 to 274.1)	258.9 (240.2 to 282.8)	252.2 (232.6 to 275.3)
North Africa and Middle East	North Africa and Middle East	Libya	Prevalence	Age-standardized	1714.1 (1606.3 to 1832.5)	1470.7 (1375.8 to 1575.2)	1938.2 (1804.4 to 2079.3)	2461 (2306.8 to 2612.1)	2044.7 (1910.2 to 2186.2)	2867.7 (2677.7 to 3052.9)	43.6 (38.1 to 49.8)	39 (32 to 46.4)	48 (40 to 56.8)
North Africa and Middle East	North Africa and Middle East	Libya	Deaths	All Ages	286 (213 to 372)	103 (69 to 140)	183 (131 to 257)	760 (561 to 973)	265 (192 to 353)	494 (336 to 662)	165.8 (86.7 to 261.9)	158.3 (77.5 to 304.6)	170 (57.7 to 293.9)
North Africa and Middle East	North Africa and Middle East	Libya	Deaths	Age-standardized	17.8 (13.3 to 23.2)	13 (8.7 to 17.8)	22.6 (16.1 to 31.8)	17.6 (13.1 to 22.5)	12.3 (8.9 to 16.5)	22.9 (15.8 to 30.3)	-1.2 (-30.3 to 34.3)	-5 (-35.4 to 49.6)	1.4 (-38.5 to 47.5)
North Africa and Middle East	North Africa and Middle East	Libya	DALYs	All Ages	9,586 (7,969 to 11,266)	3,808 (2,918 to 4,666)	5,777 (4,670 to 7,288)	28,042 (23,234 to 33,105)	11,004 (9,115 to 13,070)	17,038 (13,186 to 20,780)	192.5 (133.7 to 257)	188.9 (134.5 to 268.8)	194.9 (104.7 to 273.6)
North Africa and Middle East	North Africa and Middle East	Libya	DALYs	Age-standardized	467.4 (383.1 to 557.4)	379.4 (290.4 to 471.3)	551.1 (434.9 to 708)	531.5 (440.8 to 630.6)	415.3 (342.9 to 493.6)	644.5 (497.9 to 789.5)	13.7 (-9.4 to 38.6)	9.5 (-12.4 to 40.8)	17 (-19.6 to 51)
North Africa and Middle East	North Africa and Middle East	Libya	YLLs	All Ages	5,894 (4,432 to 7,583)	2,165 (1,390 to 2,967)	3,730 (2,713 to 5,214)	15,127 (10,631 to 19,619)	5,210 (3,693 to 7,004)	9,917 (6,349 to 13,461)	156.6 (73.5 to 262.5)	140.7 (66.6 to 288.7)	165.9 (38.9 to 287.6)
North Africa and Middle East	North Africa and Middle East	Libya	YLLs	Age-standardized	309 (230.4 to 398.3)	232.8 (152.6 to 320.1)	381.3 (272 to 536.4)	306.7 (221.3 to 395.3)	214 (153.9 to 286.3)	396.5 (261.1 to 532)	-0.8 (-31.9 to 37.1)	-8.1 (-37.7 to 45.1)	4 (-42.5 to 54.4)
North Africa and Middle East	North Africa and Middle East	Libya	YLDs	All Ages	3,692 (2,962 to 4,308)	1,644 (1,328 to 1,927)	2,048 (1,619 to 2,462)	12,915 (10,413 to 15,008)	5,794 (4,681 to 6,759)	7,121 (5,623 to 8,522)	249.8 (228.3 to 274.6)	252.5 (223.1 to 289.9)	247.7 (220.4 to 280.1)
North Africa and Middle East	North Africa and Middle East	Libya	YLDs	Age-standardized	158.4 (127.8 to 184.1)	146.6 (119 to 171.1)	169.8 (134.9 to 201.6)	224.8 (181.9 to 259.9)	201.3 (162.8 to 233.5)	248 (196.6 to 294.9)	42 (34.9 to 49.8)	37.3 (27.7 to 49.2)	46.1 (36.7 to 57.3)
North Africa and Middle East	North Africa and Middle East	Morocco	Incidence	All Ages	13,891 (13,025 to 14,831)	6,431 (6,013 to 6,874)	7,460 (6,937 to 8,030)	37,686 (35,247 to 40,430)	14,533 (13,543 to 15,622)	23,153 (21,635 to 24,829)	171.3 (159.9 to 184.3)	126 (114 to 138.4)	210.3 (193.9 to 228.2)
North Africa and Middle East	North Africa and Middle East	Morocco	Incidence	Age-standardized	85.8 (79.9 to 92)	74.5 (69.2 to 80.3)	98.3 (91.7 to 105.4)	120.6 (113.2 to 128.9)	91.3 (85.3 to 97.9)	151.2 (141.9 to 162.1)	40.5 (35.3 to 45.8)	22.5 (16.3 to 28.8)	53.9 (46.1 to 61.7)
North Africa and Middle East	North Africa and Middle East	Morocco	Prevalence	All Ages	223,596 (208,137 to 238,935)	110,356 (102,455 to 117,517)	113,240 (104,186 to 122,585)	611,177 (573,610 to 650,326)	262,702 (246,082 to 280,430)	348,474 (326,373 to 371,268)	173.3 (160.8 to 188)	138.1 (126.3 to 152.2)	207.7 (188.8 to 230.5)
North Africa and Middle East	North Africa and Middle East	Morocco	Prevalence	Age-standardized	1365.6 (1274.9 to 1460.4)	1295.4 (1208 to 1386.1)	1444.7 (1339.9 to 1554.6)	1896.7 (1782.4 to 2018.8)	1581.7 (1483.4 to 1686.8)	2224.3 (2081.6 to 2378.6)	38.9 (33.4 to 44.9)	22.1 (16.5 to 28.5)	54 (45.9 to 62.8)
North Africa and Middle East	North Africa and Middle East	Morocco	Deaths	All Ages	2,346 (1,718 to 3,094)	921 (343 to 1,352)	1,425 (988 to 2,060)	5,935 (4,494 to 7,387)	2,129 (1,424 to 2,863)	3,807 (2,785 to 4,850)	153 (91.4 to 238.4)	131.2 (62.8 to 324)	167.1 (87.3 to 275.4)
North Africa and Middle East	North Africa and Middle East	Morocco	Deaths	Age-standardized	21.7 (15.7 to 28.7)	16.3 (6.6 to 24)	27.9 (19 to 41)	24.1 (18.6 to 29.7)	16.9 (11.4 to 22.9)	31.6 (23.3 to 40.6)	11.1 (-15.1 to 49.9)	3.6 (-26.6 to 81.1)	13.4 (-19.2 to 62.5)
North Africa and Middle East	North Africa and Middle East	Morocco	DALYs	All Ages	72,430 (58,330 to 87,827)	31,844 (19,010 to 42,698)	40,586 (31,469 to 53,611)	172,958 (141,768 to 203,952)	68,511 (52,581 to 85,679)	104,447 (83,361 to 127,007)	138.8 (95 to 189.3)	115.1 (68.5 to 201.3)	157.3 (95.1 to 227.6)
North Africa and Middle East	North Africa and Middle East	Morocco	DALYs	Age-standardized	509.3 (406.7 to 627.4)	424.2 (244.9 to 569.7)	601.1 (455 to 807.8)	577.5 (476.2 to 680.6)	442.4 (341.9 to 548.8)	718.7 (572.9 to 873.1)	13.4 (-7 to 39)	4.3 (-19.1 to 49.2)	19.6 (-9.7 to 54.6)
North Africa and Middle East	North Africa and Middle East	Morocco	YLLs	All Ages	51,168 (37,519 to 66,693)	20,629 (6,752 to 31,052)	30,539 (21,687 to 44,003)	116,058 (86,873 to 146,419)	42,185 (27,320 to 59,157)	73,873 (53,024 to 95,393)	126.8 (68.1 to 200.3)	104.5 (42 to 293.8)	141.9 (68.3 to 233.1)
North Africa and Middle East	North Africa and Middle East	Morocco	YLLs	Age-standardized	381.1 (278 to 499.7)	294.5 (104.4 to 432.5)	473.9 (332.6 to 686.1)	402.5 (303.6 to 504.4)	285.3 (190.2 to 391.3)	524.8 (381.1 to 671.6)	5.6 (-20.8 to 40)	-3.1 (-32.1 to 81.7)	10.7 (-22.4 to 56.2)
North Africa and Middle East	North Africa and Middle East	Morocco	YLDs	All Ages	21,262 (17,053 to 25,029)	11,215 (9,001 to 13,189)	10,047 (7,906 to 12,120)	56,900 (46,063 to 66,617)	26,325 (21,272 to 31,008)	30,574 (24,226 to 36,435)	167.6 (148.8 to 186.4)	134.7 (114.5 to 155.7)	204.3 (175.8 to 235.5)
North Africa and Middle East	North Africa and Middle East	Morocco	YLDs	Age-standardized	128.2 (103.1 to 149.8)	129.7 (104.9 to 150.2)	127.3 (101.2 to 152.8)	175 (141.8 to 202.9)	157.1 (127.7 to 183.6)	193.9 (153.9 to 230.3)	36.5 (28.8 to 44.6)	21.2 (12.2 to 30.6)	52.3 (40.3 to 64.5)
North Africa and Middle East	North Africa and Middle East	Palestine	Incidence	All Ages	1,168 (1,100 to 1,235)	493 (460 to 526)	675 (634 to 716)	2,874 (2,704 to 3,062)	1,284 (1,189 to 1,379)	1,591 (1,496 to 1,694)	146.1 (135.8 to 156)	160.3 (147.7 to 173.1)	135.8 (123.4 to 148.4)
North Africa and Middle East	North Africa and Middle East	Palestine	Incidence	Age-standardized	102.4 (96.5 to 108.8)	77.8 (72.6 to 83.2)	132.2 (124.7 to 140.7)	96.9 (91.6 to 102.7)	85.6 (79.7 to 91.6)	105.8 (100.2 to 112.1)	-5.4 (-8.4 to -2.1)	10 (4.7 to 15.7)	-19.9 (-23.6 to -16.3)
North Africa and Middle East	North Africa and Middle East	Palestine	Prevalence	All Ages	17,881 (16,699 to 18,965)	7,976 (7,380 to 8,521)	9,906 (9,250 to 10,539)	44,568 (40,888 to 48,163)	20,705 (18,783 to 22,594)	23,863 (21,858 to 25,822)	149.2 (134.6 to 163.2)	159.6 (143.5 to 176.8)	140.9 (123.6 to 156.6)
North Africa and Middle East	North Africa and Middle East	Palestine	Prevalence	Age-standardized	1707.3 (1607.6 to 1810.6)	1377.9 (1283 to 1471.7)	2111.8 (1982.3 to 2249.2)	1552.7 (1454.1 to 1659.8)	1390.1 (1279.8 to 1499.2)	1722.4 (1613.8 to 1839.8)	-9.1 (-12.9 to -5.2)	0.9 (-4.5 to 6.4)	-18.4 (-23 to -14)
North Africa and Middle East	North Africa and Middle East	Palestine	Deaths	All Ages	192 (146 to 273)	64 (36 to 96)	128 (94 to 223)	285 (234 to 368)	88 (65 to 147)	197 (159 to 238)	48.3 (-10.2 to 103.2)	37.2 (-7.9 to 134.5)	53.9 (-20.2 to 124)
North Africa and Middle East	North Africa and Middle East	Palestine	Deaths	Age-standardized	25.9 (19.8 to 36.8)	15.5 (8.9 to 23.7)	39.4 (29.1 to 68.3)	16.4 (13.4 to 21.4)	9.2 (6.8 to 15.6)	27.6 (22.3 to 33)	-36.5 (-61.4 to -14)	-40.6 (-60.2 to 0.4)	-30 (-63.8 to -0.9)
North Africa and Middle East	North Africa and Middle East	Palestine	DALYs	All Ages	5,537 (4,473 to 7,193)	2,101 (1,547 to 2,690)	3,436 (2,698 to 5,348)	9,932 (8,572 to 11,625)	3,742 (3,086 to 4,873)	6,190 (5,257 to 7,114)	79.4 (28 to 122.9)	78.1 (38.5 to 143.8)	80.2 (6.9 to 137.2)
North Africa and Middle East	North Africa and Middle East	Palestine	DALYs	Age-standardized	605.1 (485.7 to 801.5)	409.1 (290.1 to 533.9)	846.8 (663.8 to 1338.7)	414.3 (359.2 to 492.5)	285.3 (235.2 to 381.2)	576.9 (492 to 669.3)	-31.5 (-52.4 to -14)	-30.3 (-46 to -2.4)	-31.9 (-61.6 to -9.3)
North Africa and Middle East	North Africa and Middle East	Palestine	YLLs	All Ages	3,864 (2,881 to 5,573)	1,297 (726 to 1,894)	2,567 (1,836 to 4,391)	5,766 (4,660 to 7,348)	1,661 (1,222 to 2,684)	4,105 (3,306 to 5,003)	49.2 (-7.5 to 108)	28.1 (-15.6 to 129)	59.9 (-21.4 to 137.2)
North Africa and Middle East	North Africa and Middle East	Palestine	YLLs	Age-standardized	447.3 (336.5 to 648)	272.3 (152.2 to 400)	662.7 (477.8 to 1146.5)	271.4 (221.8 to 348.4)	148.4 (108.8 to 243.7)	428.1 (345.7 to 516.1)	-39.3 (-63 to -16.4)	-45.5 (-64.1 to -4.2)	-35.4 (-67.2 to -5.6)
North Africa and Middle East	North Africa and Middle East	Palestine	YLDs	All Ages	1,674 (1,339 to 1,958)	804 (643 to 939)	869 (675 to 1,043)	4,166 (3,325 to 4,904)	2,081 (1,656 to 2,473)	2,085 (1,648 to 2,542)	148.9 (129.6 to 167.3)	158.7 (134.2 to 185.1)	139.9 (116.7 to 164.1)
North Africa and Middle East	North Africa and Middle East	Palestine	YLDs	Age-standardized	157.8 (127.5 to 183.6)	136.8 (110.7 to 159.3)	184.1 (143.7 to 218.3)	142.8 (114.7 to 165.4)	136.9 (109.6 to 160.1)	148.8 (118.8 to 178.5)	-9.5 (-14.4 to -4.3)	0.1 (-7.6 to 8.1)	-19.2 (-25.3 to -13)
North Africa and Middle East	North Africa and Middle East	Oman	Incidence	All Ages	552 (493 to 612)	195 (173 to 217)	357 (318 to 397)	2,015 (1,795 to 2,262)	619 (551 to 687)	1,396 (1,228 to 1,572)	265.2 (241.2 to 290)	218 (190.9 to 245)	290.9 (262.5 to 321.5)
North Africa and Middle East	North Africa and Middle East	Oman	Incidence	Age-standardized	60 (55.8 to 64.7)	43.3 (39.5 to 47.5)	75.2 (69.7 to 81.3)	91 (85.3 to 97.1)	69.5 (64.4 to 75.3)	107.3 (99.7 to 114.9)	51.6 (45.6 to 57.9)	60.5 (52 to 69.6)	42.6 (35.2 to 49.9)
North Africa and Middle East	North Africa and Middle East	Oman	Prevalence	All Ages	8,852 (7,578 to 10,134)	3,284 (2,838 to 3,747)	5,568 (4,738 to 6,482)	33,346 (28,850 to 38,642)	11,359 (9,883 to 13,001)	21,987 (18,790 to 25,783)	276.7 (250.1 to 304.6)	245.9 (215.3 to 276.5)	294.9 (259.3 to 333.3)
North Africa and Middle East	North Africa and Middle East	Oman	Prevalence	Age-standardized	876.5 (784.4 to 968.7)	740.7 (654.2 to 831.3)	1043.8 (944 to 1148.6)	1281.2 (1172.5 to 1395.8)	1093.3 (987.3 to 1201.8)	1478.3 (1351.1 to 1608.8)	46.2 (39.6 to 53.6)	47.6 (37.7 to 56.6)	41.6 (33.8 to 50.1)
North Africa and Middle East	North Africa and Middle East	Oman	Deaths	All Ages	154 (114 to 207)	60 (39 to 88)	94 (65 to 151)	202 (155 to 235)	76 (46 to 99)	126 (96 to 152)	30.9 (-8.3 to 76.9)	26.7 (-20.9 to 114.8)	33.6 (-26.6 to 97.1)
North Africa and Middle East	North Africa and Middle East	Oman	Deaths	Age-standardized	34.2 (25.3 to 46.2)	26.7 (16.9 to 39.5)	44.8 (32.3 to 69.5)	23 (16.8 to 27.2)	17.7 (10.2 to 23.4)	29.1 (21 to 35.2)	-32.7 (-52.1 to -9.3)	-33.6 (-60.3 to 10.6)	-35 (-65.3 to -5.4)
North Africa and Middle East	North Africa and Middle East	Oman	DALYs	All Ages	4,386 (3,429 to 5,769)	1,631 (1,156 to 2,215)	2,755 (2,047 to 4,128)	7,193 (6,122 to 8,214)	2,656 (2,112 to 3,144)	4,537 (3,746 to 5,316)	64 (22.9 to 110.9)	62.8 (16.7 to 142.1)	64.7 (3.7 to 124.3)
North Africa and Middle East	North Africa and Middle East	Oman	DALYs	Age-standardized	673.8 (517.4 to 879.1)	533.9 (369.8 to 750.6)	836.7 (618.1 to 1270)	453.9 (363.5 to 514.4)	374.3 (265.6 to 458.7)	539.9 (428.8 to 629.8)	-32.6 (-50.4 to -12.1)	-29.9 (-52.1 to 8.3)	-35.5 (-62 to -9.9)
North Africa and Middle East	North Africa and Middle East	Oman	YLLs	All Ages	3,553 (2,611 to 4,903)	1,293 (826 to 1,858)	2,260 (1,564 to 3,604)	4,089 (3,349 to 4,747)	1,497 (1,012 to 1,956)	2,592 (1,999 to 3,146)	15.1 (-19.8 to 57)	15.8 (-27.5 to 102.2)	14.7 (-34.9 to 71.1)
North Africa and Middle East	North Africa and Middle East	Oman	YLLs	Age-standardized	593.3 (438.4 to 797.1)	459.7 (296.3 to 672.2)	746 (529.8 to 1180)	337.9 (254 to 394.7)	266.2 (160 to 347.1)	412.6 (307.6 to 496.4)	-43 (-59.8 to -22.8)	-42.1 (-64.6 to -0.7)	-44.7 (-70.3 to -18.2)
North Africa and Middle East	North Africa and Middle East	Oman	YLDs	All Ages	833 (637 to 1,013)	338 (259 to 420)	495 (375 to 631)	3,104 (2,415 to 3,814)	1,159 (902 to 1,421)	1,945 (1,484 to 2,461)	272.6 (235.3 to 314.7)	242.5 (195.5 to 297.4)	293.1 (240.3 to 351.8)
North Africa and Middle East	North Africa and Middle East	Oman	YLDs	Age-standardized	80.5 (63.8 to 95.4)	74.1 (58.1 to 89.5)	90.7 (71.7 to 109.8)	116 (94.1 to 136.5)	108 (87 to 128.3)	127.2 (101.1 to 153.3)	44.1 (34.6 to 53.9)	45.8 (32 to 60.8)	40.2 (28.6 to 52.4)
North Africa and Middle East	North Africa and Middle East	Qatar	Incidence	All Ages	256 (239 to 274)	93 (87 to 99)	163 (149 to 177)	1,635 (1,478 to 1,792)	407 (367 to 448)	1,227 (1,098 to 1,359)	537.4 (500.1 to 575.8)	336.8 (302.6 to 371.2)	651.9 (599.3 to 706.3)
North Africa and Middle East	North Africa and Middle East	Qatar	Incidence	Age-standardized	147.5 (139 to 156.5)	143.6 (134.8 to 152.8)	162.4 (153.1 to 172)	131.9 (124.1 to 140.3)	112.9 (105 to 121.7)	138.4 (129.8 to 147.6)	-10.6 (-14 to -7.2)	-21.4 (-25.4 to -17.3)	-14.7 (-18.6 to -10.7)
North Africa and Middle East	North Africa and Middle East	Qatar	Prevalence	All Ages	4,075 (3,757 to 4,378)	1,382 (1,314 to 1,448)	2,692 (2,404 to 2,950)	26,415 (23,313 to 29,791)	7,039 (6,281 to 7,709)	19,376 (16,793 to 22,349)	548.2 (497.7 to 604.3)	409.2 (356.3 to 455.2)	619.7 (551.2 to 696)
North Africa and Middle East	North Africa and Middle East	Qatar	Prevalence	Age-standardized	1981.8 (1865.8 to 2101)	2205.2 (2070.3 to 2341.6)	1987.2 (1857.8 to 2121.2)	1754.9 (1633.4 to 1886.3)	1718.7 (1581.1 to 1854.7)	1769.4 (1639.1 to 1905)	-11.4 (-15.2 to -7.7)	-22.1 (-27.1 to -17.1)	-11 (-15.2 to -6.3)
North Africa and Middle East	North Africa and Middle East	Qatar	Deaths	All Ages	15 (12 to 21)	6 (4 to 8)	9 (6 to 14)	49 (36 to 77)	14 (10 to 24)	35 (25 to 54)	233.9 (129.8 to 361.5)	138.3 (55.2 to 290.6)	299.6 (154.5 to 499.7)
North Africa and Middle East	North Africa and Middle East	Qatar	Deaths	Age-standardized	26.6 (20.9 to 38)	24.3 (17.4 to 34.6)	30.1 (22.4 to 47.1)	20.2 (15.5 to 28.6)	23.8 (17.8 to 35.9)	19.2 (14.5 to 27.9)	-24.1 (-47.2 to 1.8)	-2.3 (-35.4 to 44.2)	-36.2 (-59.8 to -7.8)
North Africa and Middle East	North Africa and Middle East	Qatar	DALYs	All Ages	774 (659 to 922)	289 (232 to 353)	485 (397 to 606)	3,730 (3,055 to 4,557)	1,085 (899 to 1,345)	2,645 (2,118 to 3,274)	382.1 (300.3 to 465.6)	275.9 (205.9 to 383.7)	445.2 (335.6 to 573.6)
North Africa and Middle East	North Africa and Middle East	Qatar	DALYs	Age-standardized	593.7 (502.9 to 768.5)	609.5 (492.5 to 784.7)	625.4 (499 to 869.7)	410.9 (345.6 to 522.7)	470.4 (385.2 to 643.7)	393.9 (326.3 to 509.3)	-30.8 (-45.7 to -15.2)	-22.8 (-40.7 to 2.5)	-37 (-54.3 to -18.7)
North Africa and Middle East	North Africa and Middle East	Qatar	YLLs	All Ages	394 (312 to 541)	148 (101 to 205)	246 (178 to 374)	1,302 (939 to 2,017)	369 (256 to 618)	933 (662 to 1,440)	230.3 (127.6 to 366.9)	149.4 (59.5 to 335.6)	279 (142.8 to 471.1)
North Africa and Middle East	North Africa and Middle East	Qatar	YLLs	Age-standardized	413.7 (325.3 to 593.8)	393.4 (281.2 to 567.1)	454.5 (334 to 705.4)	256.7 (194.7 to 375.2)	303.9 (224.8 to 476.6)	243.2 (180.7 to 358.9)	-38 (-56.3 to -15.8)	-22.8 (-48.4 to 19.4)	-46.5 (-65.1 to -21.8)
North Africa and Middle East	North Africa and Middle East	Qatar	YLDs	All Ages	380 (305 to 453)	141 (113 to 164)	239 (185 to 297)	2,429 (1,892 to 2,954)	716 (563 to 863)	1,712 (1,302 to 2,169)	539.5 (467.1 to 615.7)	409 (342.3 to 474.9)	616.3 (511.2 to 724.7)
North Africa and Middle East	North Africa and Middle East	Qatar	YLDs	Age-standardized	180 (146.2 to 206.9)	216.1 (173.2 to 250.2)	170.9 (137.6 to 201.4)	154.2 (123.7 to 180.3)	166.5 (134.9 to 194.9)	150.7 (119.8 to 180.3)	-14.3 (-19.6 to -8.8)	-22.9 (-29.3 to -16.8)	-11.8 (-17.8 to -5.2)
North Africa and Middle East	North Africa and Middle East	Saudi Arabia	Incidence	All Ages	7,388 (6,909 to 7,899)	3,278 (3,045 to 3,509)	4,111 (3,817 to 4,444)	31,297 (29,021 to 33,631)	12,014 (11,080 to 12,918)	19,283 (17,872 to 20,837)	323.6 (299.9 to 350.7)	266.6 (240.2 to 295.3)	369.1 (338 to 401)
North Africa and Middle East	North Africa and Middle East	Saudi Arabia	Incidence	Age-standardized	101.2 (95.3 to 107.9)	97.5 (91.2 to 104)	104.7 (98.3 to 111.5)	145.1 (136.6 to 154.8)	123.5 (115.3 to 132.5)	161.1 (150.6 to 172.2)	43.4 (37.7 to 49.2)	26.7 (20.4 to 32.9)	53.9 (45.7 to 61.7)
North Africa and Middle East	North Africa and Middle East	Saudi Arabia	Prevalence	All Ages	101,104 (91,334 to 111,224)	47,809 (43,109 to 52,135)	53,295 (47,142 to 60,128)	434,561 (396,012 to 473,597)	183,432 (165,952 to 199,214)	251,129 (225,638 to 276,011)	329.8 (304.3 to 354.5)	283.7 (259.5 to 310.2)	371.2 (331 to 411.7)
North Africa and Middle East	North Africa and Middle East	Saudi Arabia	Prevalence	Age-standardized	1381.3 (1285.4 to 1485)	1477.2 (1364.5 to 1585.7)	1328.8 (1226.7 to 1444)	2053 (1918.1 to 2194.3)	1975.7 (1829.1 to 2120.2)	2123.7 (1980.2 to 2283.6)	48.6 (42.5 to 54.8)	33.7 (27.5 to 40.6)	59.8 (50.3 to 68.7)
North Africa and Middle East	North Africa and Middle East	Saudi Arabia	Deaths	All Ages	1,433 (1,073 to 2,089)	600 (378 to 863)	833 (595 to 1,447)	2,119 (1,685 to 2,576)	752 (538 to 1,037)	1,368 (1,021 to 1,707)	47.9 (-5.7 to 106.7)	25.3 (-23 to 122)	64.2 (-25.8 to 147.3)
[illegible]	[illegible]	[illegible]	[illegible]	[illegible]	[illegible]	[illegible]	[illegible]	[illegible]	[illegible]	[illegible]	[illegible]	[illegible]	[illegible]

Chronic obstructive pulmonary disease

7 GBD super region	21 GBD region	Location	Measure	Age	1990			2019			Percent change (1990 to 2019)		
					Both	Female	Male	Both	Female	Male	Both	Female	Male
Sub-Saharan Africa	Central sub-Saharan Africa	Angola	Deaths	All Ages	1,325 (982 to 1,765)	583 (290 to 959)	742 (531 to 973)	2,245 (1,673 to 2,864)	1,120 (595 to 1,626)	1,125 (871 to 1,438)	69.5 (27.7 to 130.5)	92.1 (32.8 to 188.1)	51.7 (5.3 to 128.6)
Sub-Saharan Africa	Central sub-Saharan Africa	Angola	Deaths	Age-standardized	47.8 (35.3 to 65.8)	41.3 (21.8 to 69.2)	55 (40.3 to 71.6)	30.6 (23.1 to 39.2)	27.4 (14.9 to 40.6)	34.8 (27.3 to 43.4)	-36.1 (-51.5 to -14.1)	-33.7 (-54.3 to -0.7)	-36.8 (-55.3 to -6)
Sub-Saharan Africa	Central sub-Saharan Africa	Angola	DALYs	All Ages	44,820 (35,172 to 57,766)	21,323 (12,853 to 33,354)	23,498 (18,002 to 30,630)	82,532 (68,281 to 97,993)	43,295 (30,680 to 54,495)	39,237 (32,218 to 47,414)	84.1 (44.3 to 134.5)	103 (42.3 to 177.9)	67 (22.2 to 130)
Sub-Saharan Africa	Central sub-Saharan Africa	Angola	DALYs	Age-standardized	1094.2 (863 to 1392.6)	990.7 (619.3 to 1473)	1202 (918.8 to 1523.8)	729.3 (596.8 to 874.1)	689.3 (476.9 to 895.5)	780.8 (645.9 to 943.4)	-33.3 (-46.3 to -15.9)	-30.4 (-47.5 to -6.4)	-35 (-51.1 to -9.3)
Sub-Saharan Africa	Central sub-Saharan Africa	Angola	YLLs	All Ages	33,908 (24,323 to 46,278)	15,102 (6,781 to 26,830)	18,806 (13,266 to 25,520)	50,767 (37,555 to 64,790)	24,219 (12,267 to 35,073)	26,547 (19,794 to 34,804)	49.7 (7.5 to 110.5)	60.4 (2.8 to 157.3)	41.2 (-6.8 to 114.9)
Sub-Saharan Africa	Central sub-Saharan Africa	Angola	YLLs	Age-standardized	884.7 (653.5 to 1178.9)	749.7 (379.7 to 1230.1)	1026 (734.4 to 1335.2)	514.8 (384.4 to 654.3)	449.5 (238.4 to 648)	598.1 (466.4 to 759.2)	-41.8 (-56.2 to -20.8)	-40 (-58.6 to -10.3)	-41.7 (-58.9 to -12.2)
Sub-Saharan Africa	Central sub-Saharan Africa	Angola	YLDs	All Ages	10,912 (9,056 to 12,483)	6,220 (5,131 to 7,247)	4,692 (3,838 to 5,540)	31,766 (26,304 to 36,398)	19,076 (15,676 to 22,293)	12,690 (10,312 to 15,103)	191.1 (172 to 209.7)	206.7 (179.5 to 234.7)	170.5 (146.7 to 196.6)
Sub-Saharan Africa	Central sub-Saharan Africa	Angola	YLDs	Age-standardized	209.4 (174.3 to 238.7)	241 (199.3 to 280.6)	176 (144.3 to 205.2)	214.5 (177.4 to 244.3)	239.8 (196.5 to 281.5)	182.7 (149.5 to 213)	2.4 (-2.6 to 7.4)	-0.5 (-7.2 to 6.6)	3.8 (-2.7 to 11.5)
Sub-Saharan Africa	Central sub-Saharan Africa	Central African Republic	Incidence	All Ages	2,001 (1,872 to 2,135)	1,018 (940 to 1,101)	983 (922 to 1,045)	3,856 (3,607 to 4,114)	1,922 (1,783 to 2,074)	1,934 (1,802 to 2,064)	92.7 (86.3 to 99.8)	88.8 (78.6 to 99.6)	96.7 (88.5 to 105.6)
Sub-Saharan Africa	Central sub-Saharan Africa	Central African Republic	Incidence	Age-standardized	141.8 (132.1 to 151.7)	137.1 (125.6 to 149.1)	146.2 (138 to 155.7)	140.1 (131 to 149.9)	135 (124.1 to 146.8)	143.5 (134.7 to 153.1)	-1.2 (-4.7 to 2.7)	-1.5 (-7.4 to 4.8)	-1.8 (-5.5 to 1.8)
Sub-Saharan Africa	Central sub-Saharan Africa	Central African Republic	Prevalence	All Ages	27,424 (25,723 to 29,229)	14,026 (13,056 to 15,045)	13,398 (12,494 to 14,377)	52,811 (49,579 to 56,338)	26,674 (24,666 to 28,676)	26,138 (24,504 to 27,969)	92.6 (86.3 to 99.1)	90.2 (80.2 to 100.1)	95.1 (86.8 to 103.8)
Sub-Saharan Africa	Central sub-Saharan Africa	Central African Republic	Prevalence	Age-standardized	1920.3 (1789.2 to 2055.7)	1867.4 (1721.2 to 2020)	1964.4 (1832 to 2104)	1917.8 (1786.5 to 2053.6)	1846.2 (1699.9 to 1996.4)	1973.1 (1837.4 to 2109.6)	-0.1 (-3.5 to 3.6)	-1.1 (-6.6 to 4.6)	0.4 (-3.7 to 4.7)
Sub-Saharan Africa	Central sub-Saharan Africa	Central African Republic	Deaths	All Ages	532 (374 to 756)	212 (107 to 351)	320 (215 to 442)	802 (535 to 1,137)	346 (172 to 632)	456 (285 to 630)	50.8 (11.2 to 99.3)	63.4 (13.3 to 138.9)	42.4 (-2.7 to 93)
Sub-Saharan Africa	Central sub-Saharan Africa	Central African Republic	Deaths	Age-standardized	61.6 (42.3 to 91.2)	46.9 (23.9 to 80)	80.7 (54.5 to 114.4)	53.1 (35.1 to 79.3)	42.5 (21.6 to 80.7)	68.4 (44.3 to 91.6)	-13.9 (-33.7 to 9.9)	-9.5 (-36.6 to 26.7)	-15.3 (-39 to 9.1)
Sub-Saharan Africa	Central sub-Saharan Africa	Central African Republic	DALYs	All Ages	16,801 (12,747 to 22,281)	6,995 (4,159 to 10,722)	9,806 (7,032 to 13,182)	26,883 (19,794 to 35,677)	11,604 (7,452 to 18,595)	15,279 (10,324 to 20,759)	60 (24.5 to 103)	65.9 (27.9 to 119.6)	55.8 (11.6 to 105.2)
Sub-Saharan Africa	Central sub-Saharan Africa	Central African Republic	DALYs	Age-standardized	1419.6 (1055.9 to 1931.9)	1093.6 (662.3 to 1659.5)	1813.4 (1298 to 2416.2)	1233.7 (901.7 to 1651.8)	987.5 (609.8 to 1602.6)	1550 (1062.4 to 2038)	-13.1 (-31.6 to 8.7)	-9.7 (-31.6 to 19.6)	-14.5 (-37.2 to 9.5)
Sub-Saharan Africa	Central sub-Saharan Africa	Central African Republic	YLLs	All Ages	13,455 (9,531 to 18,804)	5,131 (2,317 to 8,915)	8,324 (5,594 to 11,790)	20,399 (13,494 to 29,091)	8,039 (3,896 to 14,858)	12,360 (7,368 to 17,721)	51.6 (9.4 to 105)	56.7 (7.6 to 134.1)	48.5 (-1.9 to 105.5)
Sub-Saharan Africa	Central sub-Saharan Africa	Central African Republic	YLLs	Age-standardized	1189.8 (829.9 to 1693.2)	852.1 (427.1 to 1421.5)	1599.7 (1079.1 to 2205.8)	1002.8 (669.3 to 1424.1)	747.7 (370.7 to 1361.1)	1333.9 (845.8 to 1818.2)	-15.7 (-37.2 to 10.6)	-12.2 (-39 to 28.5)	-16.6 (-41.4 to 10.5)
Sub-Saharan Africa	Central sub-Saharan Africa	Central African Republic	YLDs	All Ages	3,346 (2,781 to 3,809)	1,864 (1,542 to 2,183)	1,482 (1,213 to 1,734)	6,484 (5,389 to 7,440)	3,565 (2,937 to 4,128)	2,919 (2,400 to 3,447)	93.8 (83.5 to 105.8)	91.2 (76.5 to 107.5)	97 (82.7 to 114.4)
Sub-Saharan Africa	Central sub-Saharan Africa	Central African Republic	YLDs	Age-standardized	229.8 (192.1 to 263.1)	241.5 (199.6 to 282.9)	213.7 (175.7 to 247.5)	230.9 (192.4 to 264.1)	239.8 (200.2 to 280.1)	216 (177.4 to 252.8)	0.5 (-4.2 to 5.6)	-0.7 (-7.1 to 5.9)	1.1 (-5 to 7.9)
Sub-Saharan Africa	Central sub-Saharan Africa	Congo	Incidence	All Ages	1,854 (1,746 to 1,969)	1,059 (988 to 1,137)	795 (750 to 846)	4,455 (4,184 to 4,746)	2,653 (2,477 to 2,837)	1,802 (1,683 to 1,932)	140.3 (131 to 149.1)	150.5 (138.2 to 163)	126.7 (116.5 to 137.4)
Sub-Saharan Africa	Central sub-Saharan Africa	Congo	Incidence	Age-standardized	146.6 (137.6 to 156.5)	155.8 (145 to 168.3)	133.7 (126.2 to 141.8)	143.3 (134.7 to 152.6)	166 (154.3 to 178.4)	119.5 (113 to 127.2)	-2.2 (-5.6 to 1.1)	6.6 (1.6 to 11.8)	-10.6 (-14.2 to -6.8)
Sub-Saharan Africa	Central sub-Saharan Africa	Congo	Prevalence	All Ages	25,730 (24,214 to 27,328)	14,740 (13,793 to 15,792)	10,990 (10,286 to 11,749)	63,447 (59,272 to 67,413)	37,665 (35,230 to 40,156)	25,783 (23,658 to 27,848)	146.6 (136.3 to 155.4)	155.5 (143.3 to 167.4)	134.6 (118.9 to 149.6)
Sub-Saharan Africa	Central sub-Saharan Africa	Congo	Prevalence	Age-standardized	2047 (1917.1 to 2190.5)	2184.9 (2035.8 to 2347.4)	1854 (1732.5 to 1987.3)	2069.4 (1935.6 to 2208.9)	2429.6 (2259.2 to 2603)	1671.1 (1554.8 to 1799.5)	1.1 (-2.8 to 4.9)	11.2 (5.8 to 16.5)	-9.9 (-14.6 to -5.2)
Sub-Saharan Africa	Central sub-Saharan Africa	Congo	Deaths	All Ages	430 (305 to 576)	185 (87 to 301)	246 (171 to 315)	619 (449 to 800)	284 (145 to 427)	335 (170 to 434)	43.8 (7.2 to 86.8)	53.7 (1 to 135)	36.3 (-9.6 to 81.3)
Sub-Saharan Africa	Central sub-Saharan Africa	Congo	Deaths	Age-standardized	54.3 (38.4 to 75.4)	42.7 (20.3 to 72.8)	70.6 (50.5 to 89.8)	34.6 (26.2 to 44.2)	29.9 (15.5 to 46.8)	40.5 (21.3 to 50.9)	-36.2 (-50.4 to -18.6)	-30.1 (-53.3 to 3)	-42.6 (-62.1 to -24.2)
Sub-Saharan Africa	Central sub-Saharan Africa	Congo	DALYs	All Ages	13,270 (10,213 to 16,935)	6,101 (3,692 to 8,905)	7,169 (5,238 to 9,109)	21,129 (16,849 to 25,517)	10,770 (7,667 to 13,718)	10,360 (6,706 to 13,016)	59.2 (25.7 to 96.7)	76.5 (29.9 to 133.6)	44.5 (9.1 to 82.8)
Sub-Saharan Africa	Central sub-Saharan Africa	Congo	DALYs	Age-standardized	1242.5 (945.8 to 1588.9)	1026.9 (614.1 to 1490.8)	1526.5 (1138 to 1894.8)	832 (668.1 to 997.7)	803.2 (559 to 1050)	868.7 (537.9 to 1077.6)	-33 (-47.3 to -18)	-21.8 (-42.2 to 4.8)	-43.1 (-57.4 to -27.9)
Sub-Saharan Africa	Central sub-Saharan Africa	Congo	YLLs	All Ages	10,079 (7,106 to 13,733)	4,142 (1,818 to 6,823)	5,937 (4,052 to 7,879)	13,239 (9,162 to 17,632)	5,775 (2,826 to 8,510)	7,464 (3,580 to 10,073)	31.4 (-7.4 to 77.4)	39.4 (-10.8 to 117.3)	25.7 (-16.5 to 69.5)
Sub-Saharan Africa	Central sub-Saharan Africa	Congo	YLLs	Age-standardized	993 (702.6 to 1333.5)	743 (350.4 to 1206)	1322.1 (921.2 to 1692.8)	579.4 (420.4 to 747.9)	488.3 (248.1 to 722.5)	684.5 (349.1 to 884.8)	-41.6 (-57 to -23.8)	-34.3 (-57 to 0.8)	-48.2 (-65.4 to -31.3)
Sub-Saharan Africa	Central sub-Saharan Africa	Congo	YLDs	All Ages	3,191 (2,632 to 3,656)	1,959 (1,613 to 2,285)	1,232 (1,009 to 1,444)	7,891 (6,520 to 9,043)	4,995 (4,133 to 5,816)	2,895 (2,318 to 3,416)	147.3 (134 to 162.6)	155 (137.5 to 173.4)	135 (114.1 to 158)
Sub-Saharan Africa	Central sub-Saharan Africa	Congo	YLDs	Age-standardized	249.5 (206.5 to 285.6)	283.9 (235.4 to 331.5)	204.3 (167.4 to 238.6)	252.6 (208.9 to 288.7)	314.9 (260.4 to 368.7)	184.2 (148.8 to 214.2)	1.2 (-3.8 to 6.6)	10.9 (4.3 to 17.7)	-9.8 (-16 to -2.9)
Sub-Saharan Africa	Central sub-Saharan Africa	Democratic Republic of the Congo	Incidence	All Ages	23,915 (22,265 to 25,809)	12,769 (11,735 to 13,896)	11,146 (10,429 to 11,921)	63,303 (59,345 to 67,557)	34,141 (31,629 to 36,722)	29,162 (27,264 to 31,241)	164.7 (154 to 175.9)	167.4 (149.7 to 185.1)	161.6 (149.9 to 173.8)
Sub-Saharan Africa	Central sub-Saharan Africa	Democratic Republic of the Congo	Incidence	Age-standardized	129 (120.3 to 138.3)	131 (119.6 to 143)	126.2 (118.7 to 134.6)	146 (136.4 to 156.5)	150.1 (137.9 to 163)	139.5 (130.4 to 148.8)	13.2 (8.7 to 18.7)	14.6 (7.5 to 22.7)	10.5 (6.1 to 15.4)
Sub-Saharan Africa	Central sub-Saharan Africa	Democratic Republic of the Congo	Prevalence	All Ages	327,331 (304,148 to 351,116)	177,221 (164,303 to 191,542)	150,110 (138,268 to 162,036)	868,445 (808,523 to 929,199)	466,403 (431,651 to 501,182)	402,042 (371,743 to 432,215)	165.3 (154.8 to 176.2)	163.2 (148.7 to 179.3)	167.8 (155 to 181.8)
Sub-Saharan Africa	Central sub-Saharan Africa	Democratic Republic of the Congo	Prevalence	Age-standardized	1701.1 (1584.4 to 1833)	1764.1 (1622.9 to 1914.2)	1632.8 (1522.2 to 1757.1)	1998.8 (1865.5 to 2145.7)	2052.3 (1896.4 to 2219.9)	1905.7 (1771.2 to 2049)	17.5 (13 to 22.7)	16.3 (9.3 to 23.9)	16.7 (11.8 to 22.5)
Sub-Saharan Africa	Central sub-Saharan Africa	Democratic Republic of the Congo	Deaths	All Ages	5,775 (3,981 to 7,941)	2,444 (1,140 to 4,185)	3,330 (2,109 to 4,599)	11,865 (7,720 to 18,665)	6,156 (3,062 to 12,682)	5,709 (3,196 to 8,074)	105.5 (50.8 to 170.1)	151.9 (70.8 to 269.1)	71.4 (16.2 to 133.6)
Sub-Saharan Africa	Central sub-Saharan Africa	Democratic Republic of the Congo	Deaths	Age-standardized	53.4 (37 to 75.2)	44 (20.3 to 79.3)	63.5 (41.4 to 86.1)	46.6 (30.2 to 77.2)	41.8 (20.7 to 87.8)	53.8 (31.2 to 74.1)	-12.7 (-35.1 to 14.1)	-4.9 (-33 to 31.2)	-15.3 (-40.6 to 12.7)
Sub-Saharan Africa	Central sub-Saharan Africa	Democratic Republic of the Congo	DALYs	All Ages	178,728 (133,611 to 233,225)	83,919 (48,856 to 129,578)	94,809 (64,705 to 128,531)	364,725 (270,262 to 492,832)	185,246 (120,705 to 307,557)	179,479 (117,508 to 242,003)	104.1 (58.6 to 155.4)	120.7 (63.4 to 202.1)	89.3 (41.7 to 144.9)
Sub-Saharan Africa	Central sub-Saharan Africa	Democratic Republic of the Congo	DALYs	Age-standardized	1138.2 (849.2 to 1502.2)	983.5 (563.8 to 1546.8)	1309.1 (890.8 to 1757.7)	1042.1 (757.7 to 1493.2)	961.7 (603.6 to 1681.9)	1147.1 (740.3 to 1520.9)	-8.4 (-27.9 to 13.2)	-2.2 (-24.8 to 28.6)	-12.4 (-34.8 to 13.1)
Sub-Saharan Africa	Central sub-Saharan Africa	Democratic Republic of the Congo	YLLs	All Ages	138,562 (94,599 to 193,088)	60,410 (26,080 to 105,166)	78,152 (48,213 to 111,260)	257,781 (167,118 to 383,962)	123,315 (59,237 to 245,619)	134,466 (73,763 to 198,062)	86 (32.5 to 150.9)	104.1 (34.1 to 215.1)	72.1 (14.1 to 138.9)
Sub-Saharan Africa	Central sub-Saharan Africa	Democratic Republic of the Congo	YLLs	Age-standardized	934.6 (645.4 to 1281.4)	756.5 (350.3 to 1313.8)	1131.1 (716.6 to 1563.2)	800.6 (520.4 to 1250.7)	696.2 (340.6 to 1412)	938.2 (527 to 1320.2)	-14.3 (-37.1 to 12)	-8 (-36.5 to 31.2)	-17.1 (-43.3 to 12.6)
Sub-Saharan Africa	Central sub-Saharan Africa	Democratic Republic of the Congo	YLDs	All Ages	40,166 (33,202 to 46,240)	23,509 (19,357 to 27,659)	16,657 (13,543 to 19,661)	106,944 (89,130 to 122,971)	61,931 (50,975 to 72,563)	45,013 (36,881 to 52,317)	166.3 (149.9 to 183.3)	163.4 (142.2 to 186.6)	170.2 (148.1 to 195.3)
Sub-Saharan Africa	Central sub-Saharan Africa	Democratic Republic of the Congo	YLDs	Age-standardized	203.6 (168.9 to 233.4)	226.9 (186.4 to 267)	177.9 (144.7 to 207.1)	241.5 (201.7 to 276.3)	265.6 (219.1 to 312)	208.9 (172.2 to 242.7)	18.6 (12.7 to 25.4)	17 (8.7 to 25.3)	17.4 (10.1 to 25.9)
Sub-Saharan Africa	Central sub-Saharan Africa	Equatorial Guinea	Incidence	All Ages	294 (275 to 315)	159 (148 to 173)	135 (126 to 144)	821 (762 to 881)	483 (446 to 524)	338 (311 to 366)	179.4 (165.3 to 193.4)	203.5 (187.6 to 222.4)	150.8 (131.7 to 171.6)
Sub-Saharan Africa	Central sub-Saharan Africa	Equatorial Guinea	Incidence	Age-standardized	129.9 (121.2 to 139.2)	127.2 (116.9 to 138.7)	133.9 (125.8 to 143)	128.9 (119.8 to 138.2)	143.3 (131 to 155.6)	108.8 (102.1 to 115.9)	-0.8 (-4.9 to 3.6)	12.7 (6 to 20.3)	-18.8 (-22.3 to -14.8)
Sub-Saharan Africa	Central sub-Saharan Africa	Equatorial Guinea	Prevalence	All Ages	3,942 (3,691 to 4,210)	2,176 (2,028 to 2,343)	1,766 (1,638 to 1,895)	11,740 (10,799 to 12,645)	6,915 (6,341 to 7,504)	4,824 (4,304 to 5,316)	197.8 (180.3 to 215.4)	217.9 (200 to 236.5)	173.2 (145.4 to 201.4)
Sub-Saharan Africa	Central sub-Saharan Africa	Equatorial Guinea	Prevalence	Age-standardized	1701.8 (1589.5 to 1831.9)	1702.1 (1578 to 1842.7)	1693.9 (1575.6 to 1818.7)	1850.1 (1710.5 to 1992.4)	2067.3 (1898.1 to 2252.8)	1546 (1427.9 to 1663.3)	8.7 (4 to 13.6)	21.5 (14.8 to 28.7)	-8.7 (-14 to -2.9)
Sub-Saharan Africa	Central sub-Saharan Africa	Equatorial Guinea	Deaths	All Ages	80 (54 to 114)	32 (16 to 54)	48 (30 to 70)	101 (65 to 164)	49 (23 to 122)	52 (26 to 72)	26.2 (-20.4 to 90.8)	52.3 (-19.6 to 266.7)	8.6 (-52.6 to 78.6)
Sub-Saharan Africa	Central sub-Saharan Africa	Equatorial Guinea	Deaths	Age-standardized	52.3 (35.1 to 76.1)	38.1 (19.5 to 64.4)	72.7 (46.4 to 106.1)	30.1 (19.7 to 50.5)	24.9 (12 to 63.6)	38 (19.6 to 51.7)	-42.5 (-62.9 to -13.8)	-34.7 (-64.3 to 45.7)	-47.7 (-78.5 to -16.2)
Sub-Saharan Africa	Central sub-Saharan Africa	Equatorial Guinea	DALYs	All Ages	2,388 (1,752 to 3,170)	1,023 (638 to 1,583)	1,365 (893 to 1,944)	3,457 (2,671 to 4,636)	1,830 (1,296 to 3,147)	1,627 (1,070 to 2,158)	44.8 (5.3 to 94.5)	78.8 (17.2 to 213.1)	19.2 (-32 to 83.8)
Sub-Saharan Africa	Central sub-Saharan Africa	Equatorial Guinea	DALYs	Age-standardized	1194.8 (867.9 to 1621.9)	903.3 (556.4 to 1376.9)	1582.5 (1051.5 to 2232.5)	699.5 (522.3 to 993.7)	646.9 (437.3 to 1200.6)	780.8 (487.9 to 1020)	-41.5 (-58.1 to -19.6)	-28.4 (-52.6 to 28.3)	-50.7 (-73.9 to -24.2)
Sub-Saharan Africa	Central sub-Saharan Africa	Equatorial Guinea	YLLs	All Ages	1,906 (1,262 to 2,689)	737 (357 to 1,292)	1,169 (701 to 1,748)	1,995 (1,247 to 3,140)	911 (423 to 2,223)	1,085 (537 to 1,578)	4.7 (-36 to 61.9)	23.6 (-37.1 to 213.6)	-7.2 (-59.8 to 59.1)
Sub-Saharan Africa	Central sub-Saharan Africa	Equatorial Guinea	YLLs	Age-standardized	990.5 (661.3 to 1408.1)	684.1 (347 to 1141.1)	1398.3 (865.5 to 2029)	474.4 (302.7 to 770.7)	380.3 (179 to 930.1)	611.5 (313.5 to 844.6)	-52.1 (-69.8 to -27.8)	-44.4 (-71 to 31)	-56.3 (-81.2 to -29.6)
Sub-Saharan Africa	Central sub-Saharan Africa	Equatorial Guinea	YLDs	All Ages	483 (401 to 554)	287 (239 to 337)	196 (160 to 230)	1,462 (1,207 to 1,685)	919 (754 to 1,077)	543 (436 to 650)	203 (179.8 to 225.9)	220.3 (194.9 to 250.7)	177.5 (144.4 to 214.7)
Sub-Saharan Africa	Central sub-Saharan Africa	Equatorial Guinea	YLDs	Age-standardized	204.4 (170.7 to 234.1)	219.2 (183.1 to 257.1)	184.2 (151.4 to 214.7)	225.2 (187.6 to 258)	266.7 (219.7 to 313.4)	169.3 (137.9 to 197.3)	10.2 (4 to 16.5)	21.7 (13.7 to 31.1)	-8.1 (-15.2 to -0.2)
Sub-Saharan Africa	Central sub-Saharan Africa	Gabon	Incidence	All Ages	707 (660 to 757)	363 (337 to 393)	343 (321 to 367)	1,292 (1,213 to 1,376)	606 (564 to 652)	686 (642 to 732)	82.7 (75.9 to 89.2)	66.7 (58.2 to 75.8)	99.6 (89.8 to 109)
Sub-Saharan Africa	Central sub-Saharan Africa	Gabon	Incidence	Age-standardized	114.9 (107.7 to 123)	107.9 (100 to 116.7)	124.6 (117.3 to 132.8)	107.7 (101.3 to 114.8)	94.4 (87.8 to 102)	124.5 (117.7 to 131.9)	-6.3 (-9.1 to -3.2)	-12.6 (-16.9 to -8.1)	-0.1 (-4.1 to 4.1)
Sub-Saharan Africa	Central sub-Saharan Africa	Gabon	Prevalence	All Ages	10,174 (9,415 to 10,917)	5,262 (4,828 to 5,709)	4,912 (4,523 to 5,287)	19,732 (18,295 to 21,159)	9,507 (8,744 to 10,323)	10,225 (9,458 to 10,992)	93.9 (87 to 101.4)	80.7 (71 to 91.2)	108.2 (98.4 to 119.2)
Sub-Saharan Africa	Central sub-Saharan Africa	Gabon	Prevalence	Age-standardized	1646.9 (1535.8 to 1767.7)	1583.3 (1461.4 to 1718.8)	1726.8 (1601.9 to 1851.8)	1636.9 (1529.6 to 1752.2)	1478.2 (1372.4 to 1599.5)	1828.2 (1706.4 to 1953.2)	-0.6 (-4 to 2.7)	-6.6 (-11.2 to -1.9)	5.9 (1.2 to 10.9)
Sub-Saharan Africa	Central sub-Saharan Africa	Gabon	Deaths	All Ages	182 (124 to 227)	79 (38 to 111)	103 (78 to 128)	204 (141 to 263)	87 (40 to 120)	116 (69 to 166)	11.8 (-23.1 to 48)	10.6 (-31.3 to 70.8)	12.7 (-27.5 to 63.9)
Sub-Saharan Africa	Central sub-Saharan Africa	Gabon	Deaths	Age-standardized	39.8 (26.5 to 50.2)	29.9 (14.6 to 42.2)	55.2 (42.3 to 67.3)	25.7 (17.9 to 32.9)	19.3 (9 to 26.6)	35.1 (21.4 to 49.4)	-35.5 (-54.8 to -15.5)	-35.6 (-59.1 to -2.4)	-36.5 (-57.8 to -8.2)
Sub-Saharan Africa	Central sub-Saharan Africa	Gabon	DALYs	All Ages	5,041 (3,807 to 6,054)	2,173 (1,360 to 2,783)	2,868 (2,269 to 3,490)	6,425 (5,079 to 7,848)	2,744 (1,920 to 3,399)	3,681 (2,639 to 4,812)	27.5 (-2 to 58.2)	26.3 (-7.1 to 70.1)	28.3 (-5.7 to 69.4)
Sub-Saharan Africa	Central sub-Saharan Africa	Gabon	DALYs	Age-standardized	906.7 (684.3 to 1088.1)	701.3 (435.4 to 903.1)	1183.2 (949.6 to 1418.3)	623.8 (487.8 to 758.4)	487.8 (328.1 to 610.3)	793.7 (551.1 to 1042.9)	-31.2 (-47.5 to -15)	-30.4 (-49.1 to -6.3)	-32.9 (-50.9 to -10.5)
Sub-Saharan Africa	Central sub-Saharan Africa	Gabon	YLLs	All Ages	3,796 (2,611 to 4,768)	1,477 (693 to 2,066)	2,319 (1,727 to 2,913)	4,019 (2,696 to 5,350)	1,482 (650 to 2,097)	2,537 (1,471 to 3,667)	5.9 (-29.4 to 43.5)	0.3 (-39.1 to 58.6)	9.4 (-32.6 to 59.8)
Sub-Saharan Africa	Central sub-Saharan Africa	Gabon	YLLs	Age-standardized	707.6 (485.5 to 885.7)	494.8 (233.8 to 690.5)	993 (754.2 to 1230)	427.3 (296.5 to 559.6)	294.9 (132.1 to 410.6)	592.9 (348.4 to 843.8)	-39.6 (-58.5 to -19.4)	-40.4 (-63.5 to -6.9)	-40.3 (-61.4 to -13.7)
Sub-Saharan Africa	Central sub-Saharan Africa	Gabon	YLDs	All Ages	1,245 (1,031 to 1,429)	695 (575 to 820)	549 (449 to 650)	2,406 (1,999 to 2,768)	1,262 (1,032 to 1,480)	1,144 (930 to 1,345)	93.3 (82.1 to 104.9)	81.6 (66.8 to 97.4)	108.2 (92.8 to 125.1)
Sub-Saharan Africa	Central sub-Saharan Africa	Gabon	YLDs	Age-standardized	199.2 (165.9 to 228.4)	206.5 (170.7 to 241.9)	190.2 (156.3 to 223.5)	196.6 (163.5 to 224)	192.9 (158.8 to 224.6)	200.9 (163.9 to 233.6)	-1.3 (-6.2 to 3.7)	-6.6 (-12.9 to 0.1)	5.6 (-0.9 to 12.7)
		Eastern Sub-Saharan Africa	Incidence	All Ages	106,598 (100,598 to 113,698)	50,146 (47,241 to 53,514)	56,453 (52,992 to 60,251)	221,542 (208,433 to 235,721)	103,842 (97,574 to 110,780)	117,700 (110,528 to 125,076)	107.8 (103.9 to 111.7)	107.1 (102.6 to 111.5)	108.5 (104.2 to 112.6)
		Eastern Sub-Saharan Africa	Incidence	Age-standardized	109.3 (101.9 to 117.1)	94.3 (87.7 to 101.3)	126.4 (118 to 135.1)	99 (92.8 to 106.2)	85.2 (79.5 to 91.6)	115.8 (108.6 to 123.7)	-9.4 (-10.6 to -7.9)	-9.7 (-11.2 to -8.1)	-8.4 (-9.9 to -6.5)
		Eastern Sub-Saharan Africa	Prevalence	All Ages	1,524,659 (1,435,212 to 1,621,299)	761,049 (717,069 to 808,984)	763,610 (715,301 to 814,477)	3,292,819 (3,078,465 to 3,515,414)	1,634,862 (1,522,009 to 1,747,603)	1,657,956 (1,549,186 to 1,768,317)	116 (111 to 120.7)	114.8 (108.3 to 120.7)	117.1 (112.5 to 121.8)
		Eastern Sub-Saharan Africa	Prevalence	Age-standardized	1576.8 (1473.4 to 1690.6)	1510.4 (1408.1 to 1621.9)	1656.5 (1549 to 1773.7)	1503.5 (1408 to 1608.7)	1410.9 (1320.3 to 1512.9)	1616.4 (1509.5 to 1728.2)	-4.7 (-6.2 to -2.9)	-6.6 (-8.6 to -4.5)	-2.4 (-4.1 to -0.6)
		Eastern Sub-Saharan Africa	Deaths	All Ages	24,221 (20,003 to 30,111)	8,116 (4,879 to 11,855)	16,104 (12,542 to 21,702)	37,076 (31,613 to 43,459)	13,474 (9,645 to 18,065)	23,603 (18,999 to 28,923)	53.1 (34.7 to 73.2)	66 (34.9 to 150.1)	46.6 (16.7 to 75.7)
		Eastern Sub-Saharan Africa	Deaths	Age-standardized	40.3 (31.8 to 49.5)	25.6 (16.9 to 35.4)	56.9 (42.8 to 78.2)	29.3 (25.1 to 33.7)	19.5 (14.3 to 25.7)	41.8 (33.9 to 49.7)	-27.2 (-36.3 to -17.6)	-23.7 (-36.7 to 7.1)	-26.6 (-42.6 to -13)
		Eastern Sub-Saharan Africa	DALYs	All Ages	783,557 (666,616 to 956,135)	318,756 (207,871 to 451,556)	464,801 (383,378 to 615,233)	1,233,648 (1,081,820 to 1,417,063)	523,176 (414,176 to 650,192)	710,472 (598,670 to 863,465)	57.4 (40.9 to 76.5)	64.1 (35 to 127.7)	52.9 (26.9 to 78.7)
		Eastern Sub-Saharan Africa	DALYs	Age-standardized	955.2 (810.5 to 1150.2)	700.8 (493.9 to 941.8)	1225.6 (1001.7 to 1590.7)	707.5 (622.5 to 801.7)	539.2 (431 to 669)	906.9 (763.3 to 1077.3)	-25.9 (-33.2 to -18.4)	-23.1 (-33.9 to 1.1)	-26 (-39.3 to -14)
		Eastern Sub-Saharan Africa	YLLs	All Ages	594,228 (489,050 to 756,934)	216,062 (106,368 to 348,926)	378,166 (299,811 to 526,139)	822,290 (691,525 to 990,164)	301,032 (195,730 to 418,585)	521,258 (413,010 to 673,660)	38.4 (20 to 61.5)	39.3 (8.5 to 135.6)	37.8 (11.3 to 67.7)
		Eastern Sub-Saharan Africa	YLLs	Age-standardized	764.1 (621.9 to 953.6)	502.8 (296.5 to 744)	1040.7 (806.4 to 1406.6)	524.2 (444.4 to 616.5)	352.9 (248.6 to 476)	725.4 (582.4 to 890.1)	-31.4 (-40 to -22.2)	-29.8 (-43.1 to 5.3)	-30.3 (-44.7 to -16.6)
		Eastern Sub-Saharan Africa	YLDs	All Ages	189,329 (158,184 to 215,151)	102,694 (85,752 to 118,134)	86,634 (72,020 to 99,293)	411,358 (340,392 to 468,773)	222,143 (183,315 to 257,778)	189,214 (156,123 to 219,261)	117.3 (111.2 to 123.2)	116.3 (108.3 to 124.2)	118.4 (112.3 to 124.5)
		Eastern Sub-Saharan Africa	YLDs	Age-standardized	191.1 (159.4 to 217.3)	198 (165.8 to 228.7)	184.9 (153.9 to 211)	183.4 (153 to 208.5)	186.2 (154.8 to 216.3)	181.5 (151.6 to 207.9)	-4 (-6 to -2)	-5.9 (-8.5 to -3.4)	-1.8 (-4 to 0.4)
Sub-Saharan Africa	Eastern sub-Saharan Africa	Burundi	Incidence	All Ages	4,026 (3,826 to 4,241)	1,831 (1,732 to 1,937)	2,195 (2,084 to 2,314)	7,357 (6,952 to 7,780)	3,223 (3,041 to 3,423)	4,134 (3,886 to 4,393)	82.7 (76.3 to 89.1)	76 (68.1 to 83)	88.3 (80.3 to 97)
Sub-Saharan Africa	Eastern sub-Saharan Africa	Burundi	Incidence	Age-standardized	140.7 (133 to 148.9)	111.6 (104.6 to 119.2)	178.2 (168.7 to 188.4)	121.4 (114.7 to 128.6)	99.9 (93.7 to 106.5)	144.2 (136.4 to 153.1)	-13.8 (-16.1 to -11.3)	-10.5 (-13.9 to -7.1)	-19.1 (-22.3 to -15.9)
Sub-Saharan Africa	Eastern sub-Saharan Africa	Burundi	Prevalence	All Ages	55,035 (52,126 to 58,029)	27,095 (25,575 to 28,652)	27,940 (26,392 to 29,567)	106,070 (99,945 to 112,433)	49,009 (45,909 to 52,023)	57,061 (53,653 to 60,740)	92.7 (83.6 to 100.1)	80.9 (71.3 to 89.4)	104.2 (92.7 to 115)
Sub-Saharan Africa	Eastern sub-Saharan Africa	Burundi	Prevalence	Age-standardized	1913.6 (1791.8 to 2036.7)	1706.7 (1596.5 to 1824.1)	2179.7 (2037.4 to 2324.7)	1758.2 (1652.7 to 1871.2)	1608.7 (1507.1 to 1718.4)	1916.5 (1796.8 to 2041.7)	-8.1 (-11.7 to -5)	-5.7 (-10.3 to -1.7)	-12.1 (-16.4 to -7.4)
Sub-Saharan Africa	Eastern sub-Saharan Africa	Burundi	Deaths	All Ages	1,241 (929 to 1,577)	446 (212 to 644)	795 (561 to 1,085)	1,464 (1,100 to 1,924)	501 (311 to 756)	962 (666 to 1,371)	17.9 (-11.6 to 60.4)	12.4 (-25.2 to 86.3)	21.1 (-11.7 to 68.7)
Sub-Saharan Africa	Eastern sub-Saharan Africa	Burundi	Deaths	Age-standardized	60.4 (45.2 to 75.9)	38.7 (19.8 to 54.8)	88.4 (63.8 to 118.4)	42.5 (32.3 to 54.7)	28.8 (18.6 to 41.9)	57.7 (40.9 to 78.1)	-29.6 (-46.4 to -6.9)	-25.5 (-50.4 to 16.1)	-34.7 (-51.2 to -11.3)
Sub-Saharan Africa	Eastern sub-Saharan Africa	Burundi	DALYs	All Ages	36,935 (28,047 to 46,061)	15,405 (8,283 to 22,493)	21,530 (15,774 to 28,995)	47,998 (37,842 to 61,419)	19,029 (13,535 to 27,001)	28,968 (21,312 to 40,247)	30 (2.3 to 73)	23.5 (-10.3 to 92.5)	34.5 (2.7 to 82.8)
Sub-Saharan Africa	Eastern sub-Saharan Africa	Burundi	DALYs	Age-standardized	1419.1 (1108 to 1755.1)	1009.9 (584.1 to 1366.2)	1927.4 (1445.7 to 2546.9)	996.4 (797 to 1258.5)	753.9 (541.6 to 1027.6)	1239.2 (915.4 to 1665.5)	-29.8 (-44.8 to -8.3)	-25.3 (-45.5 to 8.9)	-35.7 (-50.4 to -14.6)
Sub-Saharan Africa	Eastern sub-Saharan Africa	Burundi	YLLs	All Ages	30,137 (21,237 to 39,327)	11,765 (4,593 to 18,844)	18,372 (12,597 to 25,923)	34,901 (25,324 to 48,141)	12,377 (7,123 to 20,613)	22,524 (15,065 to 33,586)	15.8 (-15.3 to 67.1)	5.2 (-31.6 to 99.3)	22.6 (-12.4 to 78.2)
Sub-Saharan Africa	Eastern sub-Saharan Africa	Burundi	YLLs	Age-standardized	1187.1 (876.8 to 1518.2)	785.5 (358.2 to 1140.3)	1684.2 (1195.8 to 2310.7)	784.2 (588.3 to 1033.1)	541.7 (336.1 to 815.2)	1025.4 (710.1 to 1454.7)	-33.9 (-50.8 to -8.7)	-31 (-54.1 to 17.7)	-39.1 (-55.4 to -14.9)
Sub-Saharan Africa	Eastern sub-Saharan Africa	Burundi	YLDs	All Ages	6,798 (5,665 to 7,735)	3,640 (3,002 to 4,209)	3,157 (2,564 to 3,684)	13,097 (10,804 to 15,002)	6,653 (5,413 to 7,838)	6,444 (5,252 to 7,523)	92.7 (80.4 to 105.1)	82.8 (66.8 to 98.9)	104.1 (87.5 to 121.3)
Sub-Saharan Africa	Eastern sub-Saharan Africa	Burundi	YLDs	Age-standardized	232 (192.3 to 264.4)	224.5 (184.4 to 262.8)	243.2 (198.1 to 281.8)	212.2 (176.8 to 242.1)	212.2 (174.3 to 247.4)	213.7 (175.6 to 248.7)	-8.5 (-12.9 to -4.3)	-5.4 (-11.6 to 1)	-12.1 (-17.8 to -6.5)
Sub-Saharan Africa	Eastern sub-Saharan Africa	Comoros	Incidence	All Ages	284 (270 to 301)	128 (121 to 136)	156 (148 to 165)	515 (486 to 547)	231 (217 to 247)	284 (267 to 302)	81.4 (75.8 to 87)	80.8 (73.8 to 88.4)	81.8 (74.5 to 89.4)
Sub-Saharan Africa	Eastern sub-Saharan Africa	Comoros	Incidence	Age-standardized	104.5 (98.5 to 110.6)	86.7 (81.8 to 92.4)	124.5 (117.6 to 132.1)	94 (88.6 to 99.8)	78.1 (73.3 to 83.9)	114.1 (107.4 to 121.2)	-10 (-12.6 to -7.4)	-9.9 (-13.4 to -6.2)	-8.4 (-12.2 to -4.8)
Sub-Saharan Africa	Eastern sub-Saharan Africa	Comoros	Prevalence	All Ages	4,080 (3,853 to 4,312)	1,976 (1,861 to 2,095)	2,105 (1,984 to 2,232)	8,091 (7,559 to 8,638)	3,984 (3,714 to 4,273)	4,107 (3,808 to 4,400)	98.3 (89.2 to 106.7)	101.7 (91.7 to 112.3)	95.1 (83.6 to 105.9)
Sub-Saharan Africa	Eastern sub-Saharan Africa	Comoros	Prevalence	Age-standardized	1520.1 (1432.8 to 1617.8)	1401.6 (1315.6 to 1498.4)	1654.8 (1553.3 to 1765.5)	1475 (1378.8 to 1569)	1364.4 (1273.7 to 1467.3)	1620.6 (1509 to 1728.2)	-3 (-6.6 to 0.6)	-2.7 (-7.1 to 2)	-2.1 (-7 to 2.7)
Sub-Saharan Africa	Eastern sub-Saharan Africa	Comoros	Deaths	All Ages	74 (49 to 99)	23 (12 to 37)	51 (33 to 69)	109 (84 to 141)	41 (26 to 62)	67 (51 to 86)	47.7 (12.1 to 120.3)	82.2 (28.2 to 180)	32.3 (-2.5 to 102.4)
Sub-Saharan Africa	Eastern sub-Saharan Africa	Comoros	Deaths	Age-standardized	39 (27.4 to 52.1)	22.5 (12.5 to 35.4)	58.6 (40.9 to 78.1)	26 (20.2 to 33.3)	17.4 (11.1 to 25.7)	37.6 (28.7 to 47.9)	-33.5 (-48.6 to -7.7)	-22.9 (-45 to 11.5)	-35.8 (-51.4 to -7.5)
Sub-Saharan Africa	Eastern sub-Saharan Africa	Comoros	DALYs	All Ages	2,042 (1,387 to 2,632)	774 (501 to 1,152)	1,268 (843 to 1,703)	3,098 (2,568 to 3,777)	1,346 (1,026 to 1,803)	1,752 (1,412 to 2,179)	51.8 (19.4 to 125.6)	74 (27.5 to 145.4)	38.2 (4.1 to 112.2)
Sub-Saharan Africa	Eastern sub-Saharan Africa	Comoros	DALYs	Age-standardized	880.8 (619.7 to 1131)	605.7 (392.2 to 888.4)	1187.1 (821.6 to 1564.3)	631.4 (521.1 to 768.9)	494.7 (372 to 665.2)	809.4 (653.7 to 1001.2)	-28.3 (-43 to 1.2)	-18.3 (-37.4 to 11.6)	-31.8 (-47.8 to -0.6)
Sub-Saharan Africa	Eastern sub-Saharan Africa	Comoros	YLLs	All Ages	1,534 (897 to 2,137)	506 (238 to 883)	1,028 (604 to 1,472)	2,095 (1,584 to 2,750)	811 (503 to 1,275)	1,284 (944 to 1,691)	36.6 (-1.1 to 139.1)	60.2 (1.6 to 184.7)	24.9 (-12 to 116.8)
Sub-Saharan Africa	Eastern sub-Saharan Africa	Comoros	YLLs	Age-standardized	694.9 (437.8 to 954.6)	419.9 (216.1 to 703.3)	1000.2 (636.5 to 1377.2)	450.5 (347.4 to 587.8)	313.3 (195.4 to 483.8)	626.3 (473.7 to 810.5)	-35.2 (-51.8 to 2.9)	-25.4 (-48.7 to 21.2)	-37.4 (-54.4 to -0.4)
Sub-Saharan Africa	Eastern sub-Saharan Africa	Comoros	YLDs	All Ages	507 (421 to 577)	267 (221 to 311)	240 (197 to 281)	1,003 (833 to 1,152)	535 (443 to 623)	468 (382 to 549)	97.8 (84.6 to 111.5)	100.3 (83.6 to 119.8)	94.9 (78.8 to 113.1)
Sub-Saharan Africa	Eastern sub-Saharan Africa	Comoros	YLDs	Age-standardized	186 (155.5 to 211.4)	185.8 (154.7 to 216)	186.9 (153.7 to 216.1)	180.9 (151 to 206.5)	181.4 (151 to 211.3)	183.1 (149.7 to 213.5)	-2.7 (-8 to 2.8)	-2.4 (-9.5 to 5.1)	-2 (-8.6 to 5.6)
Sub-Saharan Africa	Eastern sub-Saharan Africa	Djibouti	Incidence	All Ages	216 (202 to 230)	92 (85 to 99)	124 (116 to 132)	714 (664 to 769)	281 (259 to 305)	432 (402 to 465)	230.7 (218.1 to 243.6)	206.9 (190.7 to 223.5)	248.2 (232 to 266)
Sub-Saharan Africa	Eastern sub-Saharan Africa	Djibouti	Incidence	Age-standardized	93.1 (87.3 to 99.3)	76.8 (71.4 to 83.2)	112.8 (106 to 119.9)	91.6 (85.9 to 98)	71.3 (65.6 to 77.6)	111.3 (104.8 to 118.6)	-1.6 (-5.1 to 2.1)	-7.2 (-11.7 to -2.5)	-1.4 (-5.7 to 3)
Sub-Saharan Africa	Eastern sub-Saharan Africa	Djibouti	Prevalence	All Ages	3,221 (2,981 to 3,444)	1,429 (1,298 to 1,545)	1,793 (1,662 to 1,914)	11,476 (10,514 to 12,402)	4,920 (4,465 to 5,381)	6,556 (6,008 to 7,097)	256.3 (240 to 272.3)	244.4 (225.1 to 264.3)	265.7 (244.6 to 287.5)
Sub-Saharan Africa	Eastern sub-Saharan Africa	Djibouti	Prevalence	Age-standardized	1400 (1303.7 to 1499.7)	1284.4 (1187.7 to 1389.1)	1536.4 (1433.9 to 1649.6)	1460.3 (1357.5 to 1573)	1285.7 (1179.4 to 1402.7)	1629.5 (1513.8 to 1755.4)	4.3 (0.6 to 8.2)	0.1 (-4.7 to 4.7)	6.1 (1 to 11.6)
Sub-Saharan Africa	Eastern sub-Saharan Africa	Djibouti	Deaths	All Ages	28 (18 to 41)	9 (4 to 14)	19 (11 to 32)	86 (53 to 127)	26 (8 to 42)	60 (37 to 95)	208.7 (121.9 to 332.1)	186.7 (65.6 to 352.2)	219.5 (119 to 362.1)
Sub-Saharan Africa	Eastern sub-Saharan Africa	Djibouti	Deaths	Age-standardized	29.4 (18.5 to 39.9)	18.3 (7.6 to 28.3)	43.7 (26.7 to 68.1)	21.3 (13.5 to 30.3)	13.6 (4.3 to 21.1)	29.4 (18.9 to 45.7)	-27.8 (-46.2 to -2)	-26 (-54.6 to 9.8)	-32.6 (-51.3 to -5.9)
Sub-Saharan Africa	Eastern sub-Saharan Africa	Djibouti	DALYs	All Ages	1,140 (852 to 1,599)	450 (284 to 632)	690 (488 to 1,069)	3,402 (2,634 to 4,475)	1,271 (868 to 1,672)	2,132 (1,545 to 3,002)	198.3 (132.6 to 279)	182.1 (98 to 271)	208.9 (129.3 to 310.1)
Sub-Saharan Africa	Eastern sub-Saharan Africa	Djibouti	DALYs	Age-standardized	703.6 (507.8 to 920.9)	509.1 (310.2 to 697.5)	924.3 (629.8 to 1391.2)	549 (409.1 to 721.3)	410.1 (248.4 to 550.4)	680.5 (488.5 to 976.8)	-22 (-38 to -0.9)	-19.4 (-37.2 to 4.8)	-26.4 (-45 to -4)
Sub-Saharan Africa	Eastern sub-Saharan Africa	Djibouti	YLLs	All Ages	736 (459 to 1,183)	253 (93 to 428)	483 (286 to 869)	1,979 (1,235 to 2,982)	599 (197 to 986)	1,380 (814 to 2,254)	168.8 (80.1 to 295)	136.7 (9.8 to 308.8)	185.6 (88.1 to 331.8)
Sub-Saharan Africa	Eastern sub-Saharan Africa	Djibouti	YLLs	Age-standardized	532.4 (335.2 to 753.6)	338.1 (139 to 524.3)	749.8 (451.9 to 1228.6)	371.7 (230.8 to 541.8)	239.1 (75.8 to 378.6)	496.4 (310.8 to 788.5)	-30.2 (-49.5 to -3)	-29.3 (-57.3 to 8.9)	-33.8 (-53.7 to -5.8)
Sub-Saharan Africa	Eastern sub-Saharan Africa	Djibouti	YLDs	All Ages	404 (331 to 470)	197 (159 to 233)	207 (168 to 246)	1,423 (1,165 to 1,643)	671 (541 to 795)	752 (610 to 886)	252.2 (226.7 to 279.7)	240.4 (207.7 to 277)	263.3 (228.9 to 301.9)
Sub-Saharan Africa	Eastern sub-Saharan Africa	Djibouti	YLDs	Age-standardized	171.3 (141 to 197.3)	171 (141.1 to 201.7)	174.5 (142.3 to 204.3)	177.3 (145.9 to 202.9)	171 (141.7 to 201.2)	184.2 (150 to 214.8)	3.5 (-2.2 to 9.4)	0 (-7.2 to 8)	5.5 (-2.3 to 13.5)
Sub-Saharan Africa	Eastern sub-Saharan Africa	Eritrea	Incidence	All Ages	1,861 (1,746 to 1,974)	797 (745 to 853)	1,064 (994 to 1,134)	4,538 (4,255 to 4,835)	1,954 (1,827 to 2,097)	2,585 (2,404 to 2,765)	143.9 (136.3 to 151.7)	145.1 (134.9 to 156.4)	143 (132.5 to 154.1)
Sub-Saharan Africa	Eastern sub-Saharan Africa	Eritrea	Incidence	Age-standardized	126.2 (118.9 to 133.8)	97.4 (90.9 to 104.5)	172.1 (162 to 182.1)	116.5 (109.3 to 123.8)	94.3 (88.1 to 101.3)	146.6 (137.5 to 155.6)	-7.7 (-10.4 to -4.7)	-3.2 (-7.2 to 0.8)	-14.8 (-18.1 to -11.6)
Sub-Saharan Africa	Eastern sub-Saharan Africa	Eritrea	Prevalence	All Ages	25,679 (24,242 to 27,128)	11,801 (10,995 to 12,659)	13,878 (12,985 to 14,752)	67,089 (63,358 to 71,102)	30,831 (28,781 to 32,940)	36,259 (34,020 to 38,443)	161.3 (152.6 to 169.2)	161.3 (148.4 to 174.4)	161.3 (149.8 to 173.3)
Sub-Saharan Africa	Eastern sub-Saharan Africa	Eritrea	Prevalence	Age-standardized	1778.5 (1657.6 to 1897.6)	1479.9 (1374.6 to 1589.9)	2223.8 (2067.2 to 2384.9)	1814.4 (1697.2 to 1939.9)	1550.7 (1447.3 to 1669)	2179.7 (2024.5 to 2342.1)	2 (-1.3 to 5.3)	4.8 (0.2 to 9.8)	-2 (-6.6 to 2.6)
Sub-Saharan Africa	Eastern sub-Saharan Africa	Eritrea	Deaths	All Ages	311 (211 to 413)	120 (59 to 198)	191 (118 to 285)	637 (447 to 820)	281 (131 to 424)	356 (233 to 483)	104.7 (48.1 to 195.1)	133.2 (46.5 to 273.1)	86.7 (32.5 to 195.9)
Sub-Saharan Africa	Eastern sub-Saharan Africa	Eritrea	Deaths	Age-standardized	40.9 (27.2 to 54.4)	26.5 (13.2 to 43.6)	68.9 (42 to 104.2)	32.9 (22.7 to 41.8)	24.5 (11.6 to 36.2)	48.7 (31.8 to 65.4)	-19.6 (-41.3 to 14.3)	-7.8 (-41.6 to 47.1)	-29.4 (-49.3 to 13.7)
Sub-Saharan Africa	Eastern sub-Saharan Africa	Eritrea	DALYs	All Ages	12,239 (9,019 to 15,739)	5,149 (2,995 to 7,753)	7,090 (4,902 to 9,873)	24,248 (19,456 to 29,408)	10,763 (7,116 to 14,407)	13,485 (10,145 to 17,152)	98.1 (53.2 to 164.2)	109 (41.5 to 193.5)	90.2 (43.3 to 171.9)
Sub-Saharan Africa	Eastern sub-Saharan Africa	Eritrea	DALYs	Age-standardized	1058.9 (784.7 to 1338.3)	735.9 (460.9 to 1083.8)	1597.9 (1080 to 2275.6)	840.6 (651.8 to 1021.5)	656 (411.5 to 895.6)	1134.2 (826.8 to 1455.9)	-20.6 (-38.2 to 5)	-10.9 (-35.2 to 25)	-29 (-46.5 to 3.2)
Sub-Saharan Africa	Eastern sub-Saharan Africa	Eritrea	YLLs	All Ages	9,084 (5,884 to 12,735)	3,553 (1,433 to 6,117)	5,531 (3,365 to 8,320)	15,980 (11,273 to 20,931)	6,593 (2,947 to 9,910)	9,387 (6,039 to 13,129)	75.9 (24.5 to 162.8)	85.6 (5.5 to 211.5)	69.7 (19 to 172.9)
Sub-Saharan Africa	Eastern sub-Saharan Africa	Eritrea	YLLs	Age-standardized	844.6 (563 to 1122)	541.3 (269 to 896.2)	1351.9 (836.3 to 2025.5)	621.3 (434.7 to 798.7)	451.8 (210.6 to 677)	892.5 (584.6 to 1204)	-26.4 (-46.5 to 5.4)	-16.5 (-47.1 to 32.9)	-34 (-52.3 to 4.5)
Sub-Saharan Africa	Eastern sub-Saharan Africa	Eritrea	YLDs	All Ages	3,156 (2,631 to 3,621)	1,596 (1,309 to 1,865)	1,559 (1,267 to 1,828)	8,268 (6,756 to 9,485)	4,170 (3,404 to 4,888)	4,099 (3,322 to 4,861)	162 (146.9 to 178.1)	161.2 (138.9 to 185.5)	162.8 (142.3 to 184.1)
Sub-Saharan Africa	Eastern sub-Saharan Africa	Eritrea	YLDs	Age-standardized	214.3 (179.4 to 244)	194.5 (159.2 to 226.6)	246 (200.9 to 286.4)	219.2 (181.4 to 251.1)	204.2 (167.8 to 238.5)	241.7 (195.6 to 282.8)	2.3 (-2.6 to 7.1)	5 (-2.1 to 12.6)	-1.8 (-7.8 to 4.3)
Sub-Saharan Africa	Eastern sub-Saharan Africa	Ethiopia	Incidence	All Ages	29,441 (27,063 to 32,243)	13,119 (12,027 to 14,373)	16,322 (14,948 to 17,990)	46,277 (42,075 to 50,705)	21,312 (19,369 to 23,336)	24,965 (22,554 to 27,437)	57.2 (48 to 67.7)	62.4 (52.5 to 73.6)	53 (42.7 to 64.9)
Sub-Saharan Africa	Eastern sub-Saharan Africa	Ethiopia	Incidence	Age-standardized	112.4 (101.5 to 123.7)	91.8 (82.6 to 102.2)	136.7 (123.5 to 150.8)	80.5 (72.8 to 88.9)	69 (62.6 to 76.5)	91.4 (82.3 to 101.4)	-28.3 (-31.6 to -23.9)	-24.8 (-29 to -20.1)	-33.1 (-37.3 to -27.4)
Sub-Saharan Africa	Eastern sub-Saharan Africa	Ethiopia	Prevalence	All Ages	420,126 (386,414 to 455,091)	200,402 (184,690 to 216,346)	219,725 (201,663 to 239,379)	683,970 (612,886 to 751,405)	337,871 (303,601 to 371,839)	346,099 (309,651 to 381,569)	62.8 (51 to 75.7)	68.6 (55.4 to 81.5)	57.5 (45 to 71)
Sub-Saharan Africa	Eastern sub-Saharan Africa	Ethiopia	Prevalence	Age-standardized	1631.1 (1492.8 to 1792.5)	1519.8 (1387.6 to 1670.9)	1766.5 (1611.5 to 1945.4)	1182.2 (1068.1 to 1302.7)	1136.4 (1030.2 to 1251.6)	1225.3 (1098.9 to 1360.5)	-27.5 (-31.5 to -22.7)	-25.2 (-29.7 to -20.2)	-30.6 (-35.1 to -25.2)
Sub-Saharan Africa	Eastern sub-Saharan Africa	Ethiopia	Deaths	All Ages	7,571 (5,989 to 9,200)	2,423 (1,073 to 3,784)	5,147 (3,820 to 7,040)	9,309 (7,480 to 10,922)	3,125 (2,130 to 4,079)	6,184 (4,497 to 7,911)	23 (0.3 to 53.5)	28.9 (-10.2 to 161)	20.2 (-13.2 to 64.9)
Sub-Saharan Africa	Eastern sub-Saharan Africa	Ethiopia	Deaths	Age-standardized	46.4 (36.4 to 56.4)	30.3 (15.5 to 44.2)	63.4 (46.5 to 89.3)	28.3 (22.7 to 33.4)	19.1 (13.3 to 24.5)	37.2 (27.5 to 47)	-38.9 (-50.1 to -24.4)	-37 (-55.1 to 12.7)	-41.3 (-57.5 to -20.9)
Sub-Saharan Africa	Eastern sub-Saharan Africa	Ethiopia	DALYs	All Ages	249,815 (205,094 to 299,132)	96,764 (52,537 to 147,820)	153,051 (118,788 to 205,678)	276,051 (235,783 to 319,003)	113,655 (89,871 to 139,977)	162,396 (125,819 to 201,956)	10.5 (-6.7 to 32.6)	17.5 (-12 to 99.7)	6.1 (-19.7 to 39)
Sub-Saharan Africa	Eastern sub-Saharan Africa	Ethiopia	DALYs	Age-standardized	1139.5 (936.2 to 1350.5)	833.8 (480.4 to 1191.7)	1432.7 (1109.7 to 1915.6)	629.1 (524.5 to 724.2)	489.2 (380.4 to 596.5)	761.3 (583.6 to 938.8)	-44.8 (-53.2 to -34.3)	-41.3 (-55.5 to -4.9)	-46.9 (-59.4 to -30.5)
Sub-Saharan Africa	Eastern sub-Saharan Africa	Ethiopia	YLLs	All Ages	197,747 (155,484 to 247,675)	69,566 (25,621 to 118,596)	128,180 (93,944 to 180,537)	189,662 (150,989 to 229,253)	67,152 (44,558 to 91,883)	122,510 (87,385 to 160,773)	-4.1 (-24.2 to 21.1)	-3.5 (-32.7 to 127.2)	-4.4 (-33.1 to 32)
Sub-Saharan Africa	Eastern sub-Saharan Africa	Ethiopia	YLLs	Age-standardized	943.6 (742.9 to 1148.5)	634.5 (284 to 985.7)	1237.6 (919.4 to 1717.5)	484.1 (385.7 to 573.5)	338.1 (231.2 to 447)	622.5 (452.1 to 802.8)	-48.7 (-58.2 to -36.1)	-46.7 (-62.6 to 6.7)	-49.7 (-63.6 to -31.2)
Sub-Saharan Africa	Eastern sub-Saharan Africa	Ethiopia	YLDs	All Ages	52,068 (43,317 to 59,858)	27,198 (22,217 to 31,730)	24,870 (20,415 to 28,975)	86,388 (70,077 to 101,068)	46,503 (37,186 to 55,049)	39,885 (32,766 to 47,053)	65.9 (52.3 to 79.2)	71 (55.3 to 85.7)	60.4 (46.2 to 75.3)
Sub-Saharan Africa	Eastern sub-Saharan Africa	Ethiopia	YLDs	Age-standardized	196 (161.8 to 225.4)	199.3 (164.3 to 232.9)	195.1 (158.6 to 227.2)	145 (119.4 to 168.1)	151.1 (123.1 to 177.9)	138.7 (114.5 to 162.4)	-26 (-30.6 to -21.1)	-24.2 (-29.2 to -18.9)	-28.9 (-33.8 to -23.2)
Sub-Saharan Africa	Eastern sub-Saharan Africa	Kenya	Incidence	All Ages	12,925 (11,966 to 14,013)	6,652 (6,156 to 7,224)	6,274 (5,818 to 6,794)	33,099 (30,489 to 36,094)	15,867 (14,655 to 17,291)	17,232 (15,812 to 18,831)	156.1 (151.4 to 161.6)	138.5 (134.2 to 143.4)	174.7 (166.8 to 182.9)
Sub-Saharan Africa	Eastern sub-Saharan Africa	Kenya	Incidence	Age-standardized	115.7 (104.7 to 127)	113.7 (102.7 to 125.2)	118.2 (106.9 to 130.1)	116.6 (106 to 128.1)	104.2 (94.7 to 114.5)	133.6 (121.2 to 147)	0.8 (-0.4 to 2)	-8.3 (-9.8 to -6.1)	13 (10.9 to 15)
Sub-Saharan Africa	Eastern sub-Saharan Africa	Kenya	Prevalence	All Ages	183,853 (170,126 to 199,017)	97,542 (90,435 to 105,622)	86,310 (79,387 to 93,685)	488,588 (452,563 to 528,500)	244,895 (226,899 to 264,333)	243,693 (225,062 to 264,471)	165.7 (162.6 to 169)	151.1 (147 to 155.4)	182.3 (177.2 to 188.5)
Sub-Saharan Africa	Eastern sub-Saharan Africa	Kenya	Prevalence	Age-standardized	1718.1 (1575.3 to 1882.2)	1786.7 (1633.5 to 1960)	1649.2 (1513.3 to 1807.2)	1730.4 (1589.6 to 1892.5)	1650.6 (1520.7 to 1800.4)	1844.8 (1692.9 to 2016.5)	0.7 (-0.4 to 1.9)	-7.6 (-9.1 to -5.7)	11.9 (10 to 13.9)
Sub-Saharan Africa	Eastern sub-Saharan Africa	Kenya	Deaths	All Ages	2,138 (1,597 to 3,449)	846 (470 to 1,286)	1,292 (808 to 2,425)	5,081 (3,957 to 6,680)	1,991 (1,280 to 3,371)	3,091 (2,148 to 4,237)	137.7 (83.3 to 182.9)	135.2 (82.7 to 209.9)	139.3 (61.9 to 212.4)
Sub-Saharan Africa	Eastern sub-Saharan Africa	Kenya	Deaths	Age-standardized	32.8 (24.5 to 53.3)	24.4 (14.2 to 37)	42.1 (26.5 to 79.2)	30.8 (23.7 to 40.8)	21.7 (14 to 36.7)	44.2 (31.1 to 60.3)	-6 (-27.6 to 10.9)	-11.1 (-30.4 to 15.2)	5 (-27.8 to 34.1)
Sub-Saharan Africa	Eastern sub-Saharan Africa	Kenya	DALYs	All Ages	68,658 (55,950 to 95,972)	32,254 (22,650 to 43,166)	36,404 (26,137 to 60,008)	172,424 (143,232 to 211,306)	75,441 (57,032 to 104,847)	96,983 (74,877 to 125,007)	151.1 (109.3 to 187)	133.9 (96 to 182.4)	166.4 (96.4 to 224.4)
Sub-Saharan Africa	Eastern sub-Saharan Africa	Kenya	DALYs	Age-standardized	776.8 (626.1 to 1132)	674.9 (470.1 to 924.6)	887.1 (625.9 to 1514)	752.8 (625.2 to 933.3)	597.9 (439.9 to 860.2)	954.1 (726.3 to 1235.5)	-3.1 (-21.7 to 10.7)	-11.4 (-26 to 6.5)	7.6 (-23 to 32.9)
Sub-Saharan Africa	Eastern sub-Saharan Africa	Kenya	YLLs	All Ages	45,543 (33,852 to 72,981)	19,057 (10,040 to 29,633)	26,486 (16,232 to 50,499)	111,390 (86,236 to 147,048)	42,282 (25,838 to 71,030)	69,109 (47,139 to 96,638)	144.6 (90.3 to 199.8)	121.9 (66.1 to 207.8)	160.9 (77.3 to 247)
Sub-Saharan Africa	Eastern sub-Saharan Africa	Kenya	YLLs	Age-standardized	566.7 (421.3 to 922)	441.3 (240.8 to 685.7)	701.3 (436.4 to 1328.3)	542.3 (421.9 to 713.1)	380.8 (241.4 to 642.4)	748.4 (521.5 to 1027.1)	-4.3 (-26.5 to 15.2)	-13.7 (-33.7 to 14.3)	6.7 (-28.2 to 40.5)
Sub-Saharan Africa	Eastern sub-Saharan Africa	Kenya	YLDs	All Ages	23,115 (19,171 to 26,550)	13,197 (10,884 to 15,331)	9,918 (8,188 to 11,452)	61,034 (50,552 to 70,346)	33,160 (27,290 to 38,756)	27,874 (23,160 to 32,361)	164 (159.8 to 168.6)	151.3 (145.9 to 156.5)	181 (174.3 to 188.7)
Sub-Saharan Africa	Eastern sub-Saharan Africa	Kenya	YLDs	Age-standardized	210 (173.9 to 242.2)	233.5 (193.5 to 273.1)	185.8 (153.4 to 214.1)	210.6 (174.2 to 243.4)	217.1 (180.2 to 253.3)	205.7 (168.8 to 238.5)	0.3 (-1.2 to 1.7)	-7 (-8.8 to -5)	10.7 (8.4 to 13.1)
Sub-Saharan Africa	Eastern sub-Saharan Africa	Madagascar	Incidence	All Ages	8,757 (8,345 to 9,170)	4,249 (4,024 to 4,476)	4,508 (4,297 to 4,738)	21,284 (19,984 to 22,599)	10,380 (9,669 to 11,097)	10,904 (10,209 to 11,588)	143.1 (135.4 to 150.7)	144.3 (134.8 to 154.7)	141.9 (132.2 to 151.1)
Sub-Saharan Africa	Eastern sub-Saharan Africa	Madagascar	Incidence	Age-standardized	140.2 (133.1 to 147.9)	130.3 (123 to 137.9)	148.8 (141.2 to 157.2)	148.9 (140.4 to 158.8)	135.8 (126.8 to 145.8)	164.4 (155.4 to 174.7)	6.2 (3.4 to 9.2)	4.2 (0.4 to 8.6)	10.5 (6.6 to 14.4)
Sub-Saharan Africa	Eastern sub-Saharan Africa	Madagascar	Prevalence	All Ages	122,717 (116,293 to 129,152)	62,541 (59,115 to 66,280)	60,176 (57,004 to 63,429)	304,017 (286,418 to 321,707)	153,541 (143,543 to 163,018)	150,476 (141,545 to 159,461)	147.7 (141 to 154.7)	145.5 (136 to 155.1)	150.1 (141.5 to 158.5)
Sub-Saharan Africa	Eastern sub-Saharan Africa	Madagascar	Prevalence	Age-standardized	1951.6 (1833.7 to 2069)	1980.4 (1848.7 to 2117.4)	1922.8 (1803.6 to 2043.3)	2171 (2027.2 to 2319)	2121.2 (1967.9 to 2277.6)	2235 (2085.7 to 2386.2)	11.2 (8 to 14.5)	7.1 (2.6 to 11.7)	16.2 (11.9 to 20.6)
Sub-Saharan Africa	Eastern sub-Saharan Africa	Madagascar	Deaths	All Ages	2,014 (1,682 to 2,430)	654 (431 to 932)	1,359 (1,061 to 1,783)	3,317 (2,390 to 4,440)	1,280 (799 to 1,922)	2,037 (1,421 to 2,816)	64.7 (19.1 to 118.3)	95.5 (35.1 to 172.3)	49.9 (1.8 to 99.2)
Sub-Saharan Africa	Eastern sub-Saharan Africa	Madagascar	Deaths	Age-standardized	47.4 (37 to 57.4)	29.6 (20.9 to 41.4)	64.6 (49 to 84.3)	43 (30.9 to 56)	30.1 (18.5 to 44.7)	58.7 (42.1 to 78.5)	-9.2 (-32.7 to 17.6)	1.8 (-27.6 to 41.4)	-9.1 (-36.8 to 20.3)
Sub-Saharan Africa	Eastern sub-Saharan Africa	Madagascar	DALYs	All Ages	65,102 (55,330 to 78,709)	27,011 (18,654 to 38,562)	38,091 (31,745 to 49,853)	119,475 (95,573 to 147,713)	53,569 (39,524 to 72,709)	65,906 (49,707 to 86,068)	83.5 (41.4 to 127.2)	98.3 (38.7 to 158.5)	73 (28.5 to 120.6)
Sub-Saharan Africa	Eastern sub-Saharan Africa	Madagascar	DALYs	Age-standardized	1120.1 (970.1 to 1304.2)	861.4 (650.6 to 1138.8)	1363.4 (1102.5 to 1742)	1039.4 (817.8 to 1299.5)	847.7 (623.6 to 1139.7)	1261.7 (949 to 1631.3)	-7.2 (-27.1 to 14.7)	-1.6 (-23.2 to 24.8)	-7.5 (-31.6 to 17.7)
Sub-Saharan Africa	Eastern sub-Saharan Africa	Madagascar	YLLs	All Ages	49,887 (40,335 to 63,574)	18,599 (10,103 to 29,829)	31,289 (25,065 to 43,167)	81,550 (58,547 to 109,571)	32,805 (19,547 to 50,864)	48,746 (33,373 to 69,953)	63.5 (13.6 to 118.6)	76.4 (4.9 to 165.6)	55.8 (4.7 to 111.7)
Sub-Saharan Africa	Eastern sub-Saharan Africa	Madagascar	YLLs	Age-standardized	882.6 (735.4 to 1068.9)	601.4 (386.5 to 871.8)	1148.3 (893.3 to 1519.4)	774.7 (557.4 to 1036.7)	568.8 (354.2 to 854.3)	1010.9 (710.7 to 1393.3)	-12.2 (-36.6 to 16.5)	-5.4 (-35 to 32.2)	-12 (-40.2 to 17.5)
Sub-Saharan Africa	Eastern sub-Saharan Africa	Madagascar	YLDs	All Ages	15,215 (12,591 to 17,337)	8,413 (6,905 to 9,843)	6,802 (5,542 to 7,892)	37,925 (31,200 to 43,165)	20,764 (16,979 to 24,435)	17,161 (13,804 to 20,082)	149.3 (136.7 to 162.7)	146.8 (129.8 to 166.6)	152.3 (134.6 to 173.5)
Sub-Saharan Africa	Eastern sub-Saharan Africa	Madagascar	YLDs	Age-standardized	237.5 (196.7 to 271.4)	260 (215.2 to 303.9)	215.1 (176.1 to 249.9)	264.7 (218 to 302)	279 (228.7 to 327.2)	250.8 (203.5 to 292.6)	11.5 (6.5 to 16.5)	7.3 (1 to 14.1)	16.6 (9.8 to 24.2)
Sub-Saharan Africa	Eastern sub-Saharan Africa	Malawi	Incidence	All Ages	4,863 (4,571 to 5,169)	2,310 (2,160 to 2,462)	2,553 (2,392 to 2,724)	9,714 (9,199 to 10,249)	4,226 (3,943 to 4,512)	5,488 (5,174 to 5,800)	99.7 (93.4 to 105.9)	83 (75 to 91.3)	114.9 (105.1 to 125.5)
Sub-Saharan Africa	Eastern sub-Saharan Africa	Malawi	Incidence	Age-standardized	95.4 (89.8 to 101.4)	80.3 (75.3 to 86)	113.6 (106.8 to 121)	95.3 (90.2 to 100.9)	72.4 (68.1 to 77.3)	128.3 (121.4 to 135.3)	0 (-3.1 to 3)	-9.9 (-13.2 to -6.3)	13 (7.6 to 18.2)
Sub-Saharan Africa	Eastern sub-Saharan Africa	Malawi	Prevalence	All Ages	71,428 (66,472 to 76,365)	36,070 (33,466 to 38,675)	35,358 (32,649 to 38,074)	147,080 (137,355 to 156,496)	69,166 (63,618 to 74,578)	77,914 (72,741 to 82,782)	105.9 (98.7 to 113.3)	91.8 (80.1 to 101.5)	120.4 (108.6 to 132.4)
Sub-Saharan Africa	Eastern sub-Saharan Africa	Malawi	Prevalence	Age-standardized	1411.6 (1319.4 to 1512.4)	1323 (1228 to 1420.8)	1520.4 (1419 to 1631.8)	1492.5 (1404.8 to 1587)	1261.6 (1171.3 to 1353.5)	1817.6 (1703.2 to 1932.8)	5.7 (2.3 to 9.2)	-4.6 (-9.2 to -0.3)	19.6 (13.9 to 26.2)
Sub-Saharan Africa	Eastern sub-Saharan Africa	Malawi	Deaths	All Ages	935 (738 to 1,183)	350 (195 to 550)	585 (438 to 842)	1,450 (1,148 to 1,769)	534 (355 to 729)	916 (706 to 1,202)	55.1 (21.6 to 95.5)	52.6 (11.2 to 134.5)	56.6 (9.3 to 106.9)
Sub-Saharan Africa	Eastern sub-Saharan Africa	Malawi	Deaths	Age-standardized	31 (23.7 to 38.7)	20.7 (12.3 to 30.8)	43.8 (32.9 to 61.4)	24.8 (19.7 to 30)	15.7 (10.4 to 21.1)	39.9 (31.5 to 50.9)	-20.1 (-36.8 to -0.9)	-24.3 (-43.4 to 8.8)	-8.8 (-35.4 to 18.3)
Sub-Saharan Africa	Eastern sub-Saharan Africa	Malawi	DALYs	All Ages	31,644 (26,492 to 39,357)	13,995 (9,182 to 21,105)	17,649 (14,014 to 24,132)	49,926 (42,580 to 58,025)	20,367 (15,963 to 25,476)	29,559 (24,169 to 37,447)	57.8 (28.7 to 92.1)	45.5 (10.1 to 103.3)	67.5 (23.9 to 110.5)
Sub-Saharan Africa	Eastern sub-Saharan Africa	Malawi	DALYs	Age-standardized	743.1 (619 to 902.2)	569.5 (385.1 to 807.6)	951.6 (750.9 to 1291.6)	630.2 (535.4 to 730.8)	441.6 (341.6 to 557.1)	900.3 (739.9 to 1125.4)	-15.2 (-29.4 to 1.2)	-22.5 (-37.6 to 2.6)	-5.4 (-29.5 to 17.7)
Sub-Saharan Africa	Eastern sub-Saharan Africa	Malawi	YLLs	All Ages	22,752 (17,851 to 30,306)	9,131 (4,361 to 15,914)	13,620 (10,080 to 20,211)	31,668 (24,456 to 39,694)	10,984 (7,151 to 15,900)	20,685 (15,476 to 28,660)	39.2 (3.9 to 84.9)	20.3 (-19.5 to 112.2)	51.9 (2.2 to 106)
Sub-Saharan Africa	Eastern sub-Saharan Africa	Malawi	YLLs	Age-standardized	571 (448 to 725.2)	395.6 (218.8 to 625.7)	780.5 (585.6 to 1115.9)	448.9 (351.5 to 550.2)	274.8 (181.9 to 385.5)	696.7 (537.1 to 912.5)	-21.4 (-38.9 to -0.7)	-30.5 (-48.6 to 5.9)	-10.7 (-37.4 to 17.3)
Sub-Saharan Africa	Eastern sub-Saharan Africa	Malawi	YLDs	All Ages	8,892 (7,354 to 10,220)	4,864 (3,974 to 5,696)	4,028 (3,297 to 4,737)	18,258 (15,192 to 20,946)	9,383 (7,645 to 11,038)	8,874 (7,352 to 10,383)	105.3 (92.5 to 118.4)	92.9 (76.2 to 112.6)	120.3 (101.7 to 140.8)
Sub-Saharan Africa	Eastern sub-Saharan Africa	Malawi	YLDs	Age-standardized	172 (142.5 to 195.5)	173.9 (143.4 to 201.7)	171.1 (140.8 to 198.1)	181.3 (151.3 to 206.5)	166.8 (138 to 195.4)	203.6 (168 to 236.2)	5.4 (0.3 to 10.3)	-4.1 (-10.8 to 3)	19 (11.1 to 27.5)
Sub-Saharan Africa	Eastern sub-Saharan Africa	Mozambique	Incidence	All Ages	6,438 (6,035 to 6,873)	2,819 (2,626 to 3,019)	3,619 (3,378 to 3,867)	15,590 (14,747 to 16,434)	6,733 (6,290 to 7,194)	8,857 (8,365 to 9,317)	142.1 (134 to 150.4)	138.8 (128.9 to 149.3)	144.7 (133.8 to 157.4)
Sub-Saharan Africa	Eastern sub-Saharan Africa	Mozambique	Incidence	Age-standardized	86.2 (81.3 to 91.5)	66 (61.7 to 70.9)	110.7 (104.3 to 117.8)	97.6 (92 to 103.5)	74.1 (69.3 to 79.3)	129.6 (122.1 to 137.1)	13.1 (9.3 to 17)	12.2 (7.6 to 17.3)	17.1 (11.8 to 22.5)
Sub-Saharan Africa	Eastern sub-Saharan Africa	Mozambique	Prevalence	All Ages	95,739 (88,644 to 102,826)	45,845 (42,212 to 49,389)	49,895 (46,084 to 53,853)	234,441 (220,557 to 247,596)	107,519 (98,196 to 115,786)	126,922 (119,660 to 134,038)	144.9 (135.1 to 156)	134.5 (122.7 to 147.1)	154.4 (140 to 170.9)
Sub-Saharan Africa	Eastern sub-Saharan Africa	Mozambique	Prevalence	Age-standardized	1246.5 (1161.8 to 1339.9)	1078.2 (994.7 to 1161.8)	1443.7 (1339.8 to 1554)	1557.7 (1460 to 1651)	1268.9 (1179.5 to 1362.7)	1936.6 (1816 to 2053.6)	25 (19.9 to 30.4)	17.7 (12.3 to 23.6)	34.1 (27.2 to 42.3)
Sub-Saharan Africa	Eastern sub-Saharan Africa	Mozambique	Deaths	All Ages	1,227 (871 to 1,666)	391 (226 to 622)	836 (572 to 1,286)	2,182 (1,662 to 2,900)	675 (424 to 994)	1,507 (1,120 to 2,122)	77.9 (28 to 137.7)	72.9 (20.5 to 188.4)	80.2 (13.2 to 155.5)
Sub-Saharan Africa	Eastern sub-Saharan Africa	Mozambique	Deaths	Age-standardized	26.7 (17.8 to 35.6)	15.8 (9.1 to 23.7)	40.3 (27.4 to 61.3)	25.5 (19.6 to 33)	14.2 (8.7 to 20.5)	43.5 (33.1 to 60.3)	-4.3 (-29.8 to 27.5)	-10.6 (-37.3 to 42.8)	7.8 (-32 to 49.5)
Sub-Saharan Africa	Eastern sub-Saharan Africa	Mozambique	DALYs	All Ages	41,161 (33,562 to 52,867)	16,300 (10,864 to 24,647)	24,861 (18,699 to 36,540)	79,251 (65,153 to 95,983)	28,791 (22,759 to 36,838)	50,460 (40,477 to 66,101)	92.5 (51.2 to 138.8)	76.6 (24.4 to 154.6)	103 (40.6 to 164.3)
Sub-Saharan Africa	Eastern sub-Saharan Africa	Mozambique	DALYs	Age-standardized	636.2 (496 to 809)	427.6 (301.1 to 599)	880 (651.3 to 1286.3)	658.9 (534 to 804.1)	403.5 (306.2 to 524.4)	1007.1 (804.6 to 1323)	3.6 (-19.9 to 29)	-5.6 (-26.9 to 30.3)	14.4 (-22.5 to 49.8)
Sub-Saharan Africa	Eastern sub-Saharan Africa	Mozambique	YLLs	All Ages	29,328 (22,088 to 41,263)	10,108 (4,877 to 18,387)	19,220 (13,108 to 30,835)	50,412 (37,358 to 68,322)	14,249 (8,667 to 21,895)	36,163 (26,352 to 52,336)	71.9 (22.8 to 134.3)	41 (-15.6 to 176.3)	88.2 (18.8 to 168.3)
Sub-Saharan Africa	Eastern sub-Saharan Africa	Mozambique	YLLs	Age-standardized	485.3 (340.6 to 659.4)	285.5 (165.3 to 460.9)	718.6 (489.3 to 1113.1)	471.5 (355.3 to 632.1)	236.6 (146.9 to 356.7)	792.2 (593 to 1111.4)	-2.8 (-30.7 to 30.2)	-17.1 (-42.9 to 37.8)	10.2 (-31.1 to 56.2)
Sub-Saharan Africa	Eastern sub-Saharan Africa	Mozambique	YLDs	All Ages	11,833 (9,728 to 13,590)	6,192 (5,037 to 7,236)	5,642 (4,592 to 6,671)	28,838 (23,886 to 32,900)	14,542 (11,896 to 17,151)	14,297 (11,703 to 16,617)	143.7 (128.1 to 161.2)	134.9 (114 to 157.7)	153.4 (130.7 to 179.5)
Sub-Saharan Africa	Eastern sub-Saharan Africa	Mozambique	YLDs	Age-standardized	151 (124.9 to 171.2)	142 (116.1 to 165.5)	161.4 (132.8 to 188)	187.4 (155.5 to 213.7)	166.9 (138.6 to 195.4)	214.9 (176.6 to 248.2)	24.1 (17.3 to 31.7)	17.5 (8.9 to 26.7)	33.1 (23.4 to 44.1)
Sub-Saharan Africa	Eastern sub-Saharan Africa	Rwanda	Incidence	All Ages	5,035 (4,793 to 5,313)	2,347 (2,224 to 2,484)	2,689 (2,558 to 2,832)	8,969 (8,444 to 9,581)	4,412 (4,134 to 4,741)	4,557 (4,289 to 4,864)	78.1 (72.3 to 84.1)	88 (80.9 to 95.8)	69.5 (61.8 to 77.6)
Sub-Saharan Africa	Eastern sub-Saharan Africa	Rwanda	Incidence	Age-standardized	141.5 (133.6 to 150)	113.8 (106.9 to 121.5)	179.5 (170.1 to 190.4)	121.1 (114 to 129.2)	103.1 (96.4 to 110.9)	150.5 (142 to 159.8)	-14.4 (-16.8 to -11.5)	-9.4 (-12.8 to -5.5)	-16.1 (-19.7 to -12.2)
Sub-Saharan Africa	Eastern sub-Saharan Africa	Rwanda	Prevalence	All Ages	69,303 (65,700 to 72,836)	34,700 (32,735 to 36,632)	34,603 (32,803 to 36,423)	134,377 (126,021 to 143,261)	70,569 (65,933 to 75,795)	63,808 (59,378 to 67,965)	93.9 (84.7 to 101.7)	103.4 (93.9 to 112.3)	84.4 (73.1 to 94.6)
Sub-Saharan Africa	Eastern sub-Saharan Africa	Rwanda	Prevalence	Age-standardized	1907.4 (1794.2 to 2023.6)	1722.6 (1614.3 to 1836.4)	2162.3 (2031.4 to 2301.9)	1806.1 (1693.1 to 1937.8)	1679.8 (1565.2 to 1809.7)	2018.3 (1892 to 2160.9)	-5.3 (-8.9 to -1.8)	-2.5 (-6.6 to 1.8)	-6.7 (-11.4 to -1.7)
Sub-Saharan Africa	Eastern sub-Saharan Africa	Rwanda	Deaths	All Ages	1,499 (1,111 to 1,897)	571 (288 to 787)	928 (651 to 1,251)	1,620 (1,265 to 2,093)	708 (474 to 1,191)	911 (649 to 1,216)	8.1 (-16.3 to 51.2)	24.1 (-17.8 to 133.3)	-1.8 (-25.2 to 36.3)
Sub-Saharan Africa	Eastern sub-Saharan Africa	Rwanda	Deaths	Age-standardized	62 (46.2 to 77)	41.4 (23.2 to 56.8)	90.4 (61.7 to 121.6)	35.6 (27.7 to 45.8)	25.6 (17.1 to 42.3)	53.1 (38.5 to 69.5)	-42.6 (-54.9 to -21.7)	-38.2 (-59.1 to 5.7)	-41.3 (-54.8 to -20.5)
Sub-Saharan Africa	Eastern sub-Saharan Africa	Rwanda	DALYs	All Ages	46,528 (35,271 to 57,464)	20,145 (10,945 to 28,322)	26,383 (19,528 to 34,931)	52,643 (43,660 to 64,294)	24,970 (18,677 to 35,746)	27,673 (21,046 to 35,302)	13.1 (-10 to 50.5)	24 (-12 to 120.1)	4.9 (-19.7 to 42.6)
Sub-Saharan Africa	Eastern sub-Saharan Africa	Rwanda	DALYs	Age-standardized	1453.4 (1129.8 to 1769.4)	1071.9 (636.5 to 1407.7)	1950.1 (1423.5 to 2549.5)	849.7 (703.7 to 1037.8)	678.9 (509.1 to 985.2)	1123.4 (859.6 to 1419.8)	-41.5 (-52.8 to -23.1)	-36.7 (-54.1 to 2.7)	-42.4 (-54.5 to -24.2)
Sub-Saharan Africa	Eastern sub-Saharan Africa	Rwanda	YLLs	All Ages	37,958 (26,994 to 48,708)	15,483 (6,316 to 23,157)	22,475 (15,648 to 30,688)	35,938 (27,489 to 46,777)	15,482 (9,693 to 25,993)	20,456 (14,127 to 28,053)	-5.3 (-29.6 to 36.7)	0 (-38.6 to 127.3)	-9 (-34.1 to 32.8)
Sub-Saharan Africa	Eastern sub-Saharan Africa	Rwanda	YLLs	Age-standardized	1222.2 (898.4 to 1543.9)	845.7 (417 to 1167.3)	1709.6 (1190.6 to 2304.1)	629.4 (490 to 816.3)	457.8 (301.7 to 764.9)	898.9 (642.7 to 1194.2)	-48.5 (-60.3 to -27.4)	-45.9 (-64.6 to 4.4)	-47.4 (-60.2 to -26.8)
Sub-Saharan Africa	Eastern sub-Saharan Africa	Rwanda	YLDs	All Ages	8,569 (7,121 to 9,716)	4,662 (3,868 to 5,375)	3,908 (3,181 to 4,523)	16,705 (13,834 to 19,161)	9,488 (7,845 to 11,062)	7,217 (5,914 to 8,440)	94.9 (83.1 to 107.4)	103.5 (88.6 to 119.9)	84.7 (68.7 to 100.3)
Sub-Saharan Africa	Eastern sub-Saharan Africa	Rwanda	YLDs	Age-standardized	231.2 (192.3 to 262.5)	226.3 (187.9 to 262.6)	240.4 (195.8 to 278.3)	220.3 (183.3 to 252.2)	221.1 (184.2 to 258.9)	224.5 (184 to 260.6)	-4.7 (-9.2 to 0.1)	-2.3 (-8.7 to 4.5)	-6.6 (-12.3 to -0.4)
Sub-Saharan Africa	Eastern sub-Saharan Africa	Somalia	Incidence	All Ages	3,959 (3,721 to 4,242)	1,773 (1,655 to 1,905)	2,186 (2,051 to 2,342)	10,133 (9,487 to 10,833)	4,813 (4,461 to 5,212)	5,320 (4,967 to 5,665)	155.9 (145.3 to 166)	171.5 (157.5 to 184.6)	143.3 (131.2 to 154.6)
Sub-Saharan Africa	Eastern sub-Saharan Africa	Somalia	Incidence	Age-standardized	113.7 (106.4 to 121.4)	92.2 (85.4 to 99.2)	140.3 (131.8 to 150.2)	102.2 (95.7 to 109.9)	87.4 (80.9 to 94.8)	127.6 (120 to 136.2)	-10.1 (-13 to -6.8)	-5.2 (-9.6 to -0.8)	-9 (-12.9 to -5.2)
Sub-Saharan Africa	Eastern sub-Saharan Africa	Somalia	Prevalence	All Ages	56,230 (52,735 to 59,955)	27,091 (25,167 to 28,966)	29,139 (27,277 to 31,127)	147,261 (136,640 to 157,532)	73,891 (67,915 to 79,741)	73,370 (67,658 to 78,533)	161.9 (149.4 to 172.8)	172.8 (157.4 to 186.3)	151.8 (137 to 165.1)
Sub-Saharan Africa	Eastern sub-Saharan Africa	Somalia	Prevalence	Age-standardized	1586.2 (1482.8 to 1704.9)	1441.6 (1341.3 to 1554)	1765 (1649 to 1899)	1518.2 (1409.2 to 1627.8)	1418.2 (1311.2 to 1537.1)	1685.5 (1564.7 to 1803.8)	-4.3 (-7.9 to -0.8)	-1.6 (-6.6 to 2.9)	-4.5 (-9.1 to -0.2)
Sub-Saharan Africa	Eastern sub-Saharan Africa	Somalia	Deaths	All Ages	987 (670 to 1,599)	323 (155 to 597)	664 (437 to 1,247)	1,991 (1,263 to 3,200)	793 (411 to 1,466)	1,198 (701 to 2,274)	101.6 (49.2 to 173.7)	145.6 (68.8 to 267.7)	80.2 (29.7 to 155.9)
Sub-Saharan Africa	Eastern sub-Saharan Africa	Somalia	Deaths	Age-standardized	52.2 (36.2 to 86.1)	30.5 (15.8 to 53.2)	79.6 (53.2 to 147.9)	40 (26 to 63.3)	27.1 (14.3 to 49.6)	62.5 (38.9 to 118.5)	-23.4 (-41.7 to 2.3)	-10.9 (-38.3 to 30)	-21.6 (-41.7 to 6.8)
Sub-Saharan Africa	Eastern sub-Saharan Africa	Somalia	DALYs	All Ages	33,380 (24,450 to 51,628)	12,995 (7,077 to 22,993)	20,385 (13,813 to 35,998)	69,971 (48,897 to 107,425)	30,193 (19,334 to 49,448)	39,778 (25,793 to 69,683)	109.6 (65.8 to 174.5)	132.3 (69.7 to 224.8)	95.1 (48.8 to 164.1)
Sub-Saharan Africa	Eastern sub-Saharan Africa	Somalia	DALYs	Age-standardized	1213.6 (893.9 to 1856.5)	803.9 (479.9 to 1303.4)	1704 (1187.5 to 3018.9)	957.1 (669 to 1413.3)	704.4 (454 to 1154)	1350 (887.1 to 2426.3)	-21.1 (-37.8 to 1.5)	-12.4 (-33.6 to 17.3)	-20.8 (-39 to 6.8)
Sub-Saharan Africa	Eastern sub-Saharan Africa	Somalia	YLLs	All Ages	26,394 (17,667 to 45,102)	9,338 (3,427 to 19,216)	17,056 (10,456 to 33,084)	51,568 (31,587 to 86,630)	20,166 (9,460 to 38,799)	31,402 (17,354 to 61,583)	95.4 (41.3 to 178.5)	116 (36.6 to 253.8)	84.1 (27.5 to 168.1)
Sub-Saharan Africa	Eastern sub-Saharan Africa	Somalia	YLLs	Age-standardized	1020.9 (696.2 to 1651.2)	614.5 (297.3 to 1120.6)	1505.9 (995.6 to 2818.3)	771.4 (491 to 1219)	517.4 (268.7 to 966.7)	1160.8 (699 to 2240.9)	-24.4 (-43.7 to 2.2)	-15.8 (-42.4 to 25.1)	-22.9 (-43.6 to 8.9)
Sub-Saharan Africa	Eastern sub-Saharan Africa	Somalia	YLDs	All Ages	6,986 (5,798 to 7,989)	3,657 (3,002 to 4,255)	3,329 (2,710 to 3,911)	18,403 (15,194 to 21,312)	10,026 (8,220 to 11,888)	8,376 (6,827 to 9,834)	163.4 (144.5 to 182.1)	174.2 (148.4 to 201.6)	151.6 (126.3 to 176.9)
Sub-Saharan Africa	Eastern sub-Saharan Africa	Somalia	YLDs	Age-standardized	192.7 (160.2 to 219.8)	189.4 (156.8 to 221.2)	198.5 (161.7 to 231.8)	185.7 (153.1 to 214.1)	187 (154.9 to 219.4)	189.3 (155 to 221.1)	-3.6 (-8.5 to 1.7)	-1.3 (-8.2 to 5.9)	-4.7 (-11.5 to 1.6)
Sub-Saharan Africa	Eastern sub-Saharan Africa	South Sudan	Incidence	All Ages	3,579 (3,372 to 3,808)	1,486 (1,402 to 1,581)	2,093 (1,962 to 2,235)	5,425 (5,085 to 5,788)	2,438 (2,271 to 2,619)	2,987 (2,789 to 3,185)	51.6 (46.3 to 57.1)	64.1 (56.1 to 71.8)	42.7 (36.4 to 49.4)
Sub-Saharan Africa	Eastern sub-Saharan Africa	South Sudan	Incidence	Age-standardized	116.6 (109 to 124.1)	99.7 (92.5 to 107.6)	133.4 (124.6 to 142.3)	104.8 (98.7 to 111.8)	89.2 (83.6 to 96)	118.7 (111.3 to 126.4)	-10.1 (-12.9 to -7)	-10.5 (-14.7 to -6.6)	-11 (-14.9 to -7.3)
Sub-Saharan Africa	Eastern sub-Saharan Africa	South Sudan	Prevalence	All Ages	50,044 (46,875 to 53,197)	21,932 (20,558 to 23,397)	28,112 (26,235 to 30,032)	79,130 (73,518 to 84,549)	37,858 (35,077 to 40,721)	41,271 (38,105 to 44,214)	58.1 (52.5 to 63.8)	72.6 (64.3 to 81.3)	46.8 (40 to 53.8)
Sub-Saharan Africa	Eastern sub-Saharan Africa	South Sudan	Prevalence	Age-standardized	1631.5 (1522.9 to 1752.9)	1543.2 (1436.5 to 1668.8)	1720.3 (1603.9 to 1841)	1568.2 (1458 to 1683.7)	1470.8 (1361.7 to 1590.3)	1654.6 (1536.8 to 1774.9)	-3.9 (-7.2 to -0.4)	-4.7 (-9.1 to -0.3)	-3.8 (-8.3 to 0.7)
Sub-Saharan Africa	Eastern sub-Saharan Africa	South Sudan	Deaths	All Ages	727 (474 to 985)	185 (93 to 313)	543 (361 to 753)	757 (511 to 1,048)	189 (111 to 311)	569 (372 to 813)	4.2 (-23.3 to 42.3)	2.3 (-30.4 to 66.2)	4.8 (-27.1 to 47)
Sub-Saharan Africa	Eastern sub-Saharan Africa	South Sudan	Deaths	Age-standardized	36.2 (24 to 48.6)	18.7 (10 to 29.5)	53.8 (36.5 to 72)	26.6 (18.5 to 35.6)	13.5 (8.3 to 21.3)	38.5 (25.7 to 53.7)	-26.5 (-44.8 to -1.1)	-27.8 (-49.6 to 12.4)	-28.5 (-49.4 to -2)
Sub-Saharan Africa	Eastern sub-Saharan Africa	South Sudan	DALYs	All Ages	22,714 (16,046 to 30,122)	7,658 (4,750 to 12,454)	15,055 (10,817 to 20,588)	25,802 (20,171 to 32,891)	9,537 (7,195 to 13,149)	16,265 (12,011 to 21,588)	13.6 (-10.9 to 45.5)	24.5 (-11.5 to 77.4)	8 (-19 to 42.6)
Sub-Saharan Africa	Eastern sub-Saharan Africa	South Sudan	DALYs	Age-standardized	857.9 (625.6 to 1116.1)	556.9 (366.3 to 813.6)	1124.5 (806.3 to 1502.3)	643.9 (492.8 to 821)	433.7 (324.8 to 592.5)	828 (603.2 to 1092.3)	-24.9 (-40.4 to -4.9)	-22.1 (-38.3 to 4.7)	-26.4 (-44.5 to -3.6)
Sub-Saharan Africa	Eastern sub-Saharan Africa	South Sudan	YLLs	All Ages	16,641 (10,083 to 23,791)	4,743 (1,916 to 9,489)	11,899 (7,699 to 17,456)	16,093 (10,751 to 23,267)	4,468 (2,406 to 7,863)	11,624 (7,400 to 16,804)	-3.3 (-33.3 to 39.2)	-5.8 (-45 to 78.7)	-2.3 (-33.8 to 41.8)
Sub-Saharan Africa	Eastern sub-Saharan Africa	South Sudan	YLLs	Age-standardized	664.6 (434.7 to 910.7)	358.2 (176.6 to 612.3)	934.2 (623.9 to 1306.8)	456.4 (304.9 to 637.7)	243.4 (140.7 to 403.4)	644.4 (417.1 to 920.8)	-31.3 (-49.9 to -5.4)	-32 (-53.3 to 11)	-31 (-51.8 to -2.6)
Sub-Saharan Africa	Eastern sub-Saharan Africa	South Sudan	YLDs	All Ages	6,072 (5,019 to 6,921)	2,916 (2,389 to 3,400)	3,157 (2,597 to 3,675)	9,709 (8,066 to 11,166)	5,069 (4,175 to 5,991)	4,641 (3,808 to 5,410)	59.9 (49.7 to 70.5)	73.8 (58 to 91.4)	47 (35.5 to 59.7)
Sub-Saharan Africa	Eastern sub-Saharan Africa	South Sudan	YLDs	Age-standardized	193.3 (159.9 to 220.4)	198.7 (164.5 to 230.5)	190.3 (156 to 221)	187.5 (157 to 213.6)	190.3 (158.8 to 223.3)	183.6 (151.7 to 213)	-3 (-7.8 to 2.1)	-4.2 (-10.8 to 2.9)	-3.5 (-10 to 3)
Sub-Saharan Africa	Eastern sub-Saharan Africa	United Republic of Tanzania	Incidence	All Ages	11,572 (10,814 to 12,319)	5,819 (5,437 to 6,188)	5,753 (5,346 to 6,153)	26,848 (25,170 to 28,515)	13,522 (12,701 to 14,364)	13,326 (12,428 to 14,200)	132 (125.3 to 138.9)	132.4 (123.1 to 141.1)	131.6 (122.3 to 141.4)
Sub-Saharan Africa	Eastern sub-Saharan Africa	United Republic of Tanzania	Incidence	Age-standardized	79.3 (74.6 to 84.3)	71.7 (67.3 to 76.1)	89.1 (83.7 to 94.6)	79.3 (74.7 to 84.5)	72.4 (67.8 to 77.4)	88.2 (83.1 to 94.2)	0 (-2.8 to 2.7)	0.9 (-3 to 4.7)	-1 (-4.8 to 2.8)
Sub-Saharan Africa	Eastern sub-Saharan Africa	United Republic of Tanzania	Prevalence	All Ages	175,315 (161,450 to 187,732)	93,281 (86,127 to 100,174)	82,034 (74,763 to 89,109)	423,197 (389,806 to 453,143)	223,909 (205,886 to 240,282)	199,288 (181,101 to 215,523)	141.4 (133.6 to 149.3)	140 (129 to 150.9)	142.9 (130.8 to 155)
Sub-Saharan Africa	Eastern sub-Saharan Africa	United Republic of Tanzania	Prevalence	Age-standardized	1233.1 (1145.9 to 1316.7)	1236.2 (1147.9 to 1327.6)	1240.2 (1140.2 to 1335.1)	1299.5 (1208.3 to 1385.4)	1289 (1201.8 to 1377.4)	1321.2 (1221 to 1415.4)	5.4 (2.2 to 8.6)	4.3 (-0.1 to 8.7)	6.5 (2.1 to 11.2)
Sub-Saharan Africa	Eastern sub-Saharan Africa	United Republic of Tanzania	Deaths	All Ages	2,325 (1,829 to 2,832)	821 (535 to 1,175)	1,504 (1,102 to 2,005)	4,259 (3,417 to 5,100)	1,659 (1,142 to 2,266)	2,599 (1,960 to 3,327)	83.2 (46.4 to 128.1)	102 (52 to 187.6)	72.9 (21.1 to 126.5)
Sub-Saharan Africa	Eastern sub-Saharan Africa	United Republic of Tanzania	Deaths	Age-standardized	26.4 (20.1 to 32)	17.5 (11.5 to 24.4)	37.3 (27 to 48.5)	21.3 (17.1 to 25.4)	15.1 (10.3 to 20.4)	29.5 (22.7 to 36.7)	-19.3 (-35.4 to -0.4)	-14 (-35.3 to 18.9)	-20.9 (-44.7 to 3.1)
Sub-Saharan Africa	Eastern sub-Saharan Africa	United Republic of Tanzania	DALYs	All Ages	75,804 (64,691 to 91,836)	32,741 (23,573 to 45,431)	43,064 (34,790 to 55,896)	145,131 (124,452 to 168,085)	67,046 (52,329 to 83,325)	78,085 (63,938 to 95,809)	91.5 (60.8 to 125.9)	104.8 (61.3 to 161.2)	81.3 (37.4 to 125.9)
Sub-Saharan Africa	Eastern sub-Saharan Africa	United Republic of Tanzania	DALYs	Age-standardized	634.6 (530.2 to 749.1)	490.1 (366.2 to 646.9)	798.1 (628.3 to 1011.7)	541.2 (461.3 to 624.9)	445.6 (352.2 to 551.1)	656.4 (533.8 to 797.5)	-14.7 (-28.3 to 0.8)	-9.1 (-25.4 to 13.4)	-17.8 (-38.3 to 2.3)
Sub-Saharan Africa	Eastern sub-Saharan Africa	United Republic of Tanzania	YLLs	All Ages	53,879 (43,475 to 69,562)	20,184 (11,303 to 32,682)	33,695 (25,723 to 46,788)	91,879 (72,713 to 113,177)	36,675 (23,507 to 52,196)	55,203 (40,708 to 74,193)	70.5 (32.1 to 116.9)	81.7 (27.1 to 178.2)	63.8 (12.9 to 119.5)
Sub-Saharan Africa	Eastern sub-Saharan Africa	United Republic of Tanzania	YLLs	Age-standardized	483.2 (377.1 to 594.3)	327.7 (210.4 to 477)	657.9 (482.2 to 877.5)	380.7 (300.5 to 458.4)	275 (186.5 to 381)	506 (382.5 to 647)	-21.2 (-37.8 to -1.1)	-16.1 (-36.7 to 19.3)	-23.1 (-46.3 to 1.5)
Sub-Saharan Africa	Eastern sub-Saharan Africa	United Republic of Tanzania	YLDs	All Ages	21,925 (18,133 to 25,102)	12,556 (10,220 to 14,724)	9,369 (7,631 to 11,084)	53,252 (43,694 to 61,474)	30,371 (24,624 to 35,882)	22,882 (18,423 to 27,023)	142.9 (128.1 to 159.7)	141.9 (120.5 to 165.6)	144.2 (123.8 to 165.7)
Sub-Saharan Africa	Eastern sub-Saharan Africa	United Republic of Tanzania	YLDs	Age-standardized	151.3 (126.3 to 172.3)	162.5 (133.1 to 189.4)	140.2 (115.8 to 163.6)	160.5 (132.5 to 183.6)	170.5 (138.8 to 199.9)	150.4 (122.9 to 174.9)	6 (1.2 to 11.5)	5 (-2.4 to 12.9)	7.2 (0 to 14.4)
Sub-Saharan Africa	Eastern sub-Saharan Africa	Uganda	Incidence	All Ages	9,396 (8,840 to 10,005)	4,693 (4,398 to 5,013)	4,703 (4,401 to 5,041)	20,755 (19,454 to 22,057)	9,722 (9,023 to 10,450)	11,032 (10,336 to 11,705)	120.9 (112.9 to 129.4)	107.2 (96 to 118.5)	134.6 (123.9 to 145.8)
Sub-Saharan Africa	Eastern sub-Saharan Africa	Uganda	Incidence	Age-standardized	109.1 (102.1 to 116.8)	100.5 (93 to 108.4)	120 (112.6 to 128.1)	101.3 (94.9 to 107.7)	83.3 (77.3 to 89.8)	127.1 (120 to 134.5)	-7.2 (-10.1 to -3.9)	-17.2 (-20.9 to -13.2)	5.9 (1.1 to 10.4)
Sub-Saharan Africa	Eastern sub-Saharan Africa	Uganda	Prevalence	All Ages	131,626 (123,059 to 140,048)	68,992 (64,512 to 73,722)	62,634 (57,756 to 67,408)	303,362 (278,483 to 326,245)	151,515 (138,232 to 164,742)	151,847 (139,921 to 163,199)	130.5 (120.4 to 140.3)	119.6 (104.6 to 133.9)	142.4 (130.2 to 155.3)
Sub-Saharan Africa	Eastern sub-Saharan Africa	Uganda	Prevalence	Age-standardized	1561.9 (1455.3 to 1674.5)	1569.2 (1457.4 to 1695.9)	1566.7 (1452.3 to 1685.8)	1556.3 (1450.3 to 1661.9)	1395.6 (1287 to 1509.8)	1789 (1666.7 to 1908)	-0.4 (-3.8 to 3.4)	-11.1 (-15.9 to -6.2)	14.2 (8.9 to 19.8)
Sub-Saharan Africa	Eastern sub-Saharan Africa	Uganda	Deaths	All Ages	2,326 (1,606 to 3,594)	692 (370 to 1,090)	1,634 (1,094 to 2,804)	3,322 (2,431 to 4,316)	1,159 (756 to 1,848)	2,163 (1,419 to 2,876)	42.8 (2.2 to 89.3)	67.5 (15.9 to 163.5)	32.4 (-18.3 to 85.1)
Sub-Saharan Africa	Eastern sub-Saharan Africa	Uganda	Deaths	Age-standardized	44.1 (30.6 to 68)	24.8 (13.9 to 37.8)	67.3 (45.1 to 114)	30.2 (22.3 to 39.3)	18 (11.9 to 28.5)	49.9 (33.4 to 65.1)	-31.6 (-49.7 to -10.3)	-27.3 (-49.3 to 11.6)	-25.9 (-54.4 to 2.4)
Sub-Saharan Africa	Eastern sub-Saharan Africa	Uganda	DALYs	All Ages	69,165 (51,588 to 99,202)	25,666 (16,450 to 39,926)	43,499 (31,204 to 72,235)	111,246 (88,850 to 137,926)	45,296 (34,295 to 61,440)	65,951 (47,930 to 83,917)	60.8 (19.3 to 102.6)	76.5 (30.9 to 149.8)	51.6 (-3.8 to 100.8)
Sub-Saharan Africa	Eastern sub-Saharan Africa	Uganda	DALYs	Age-standardized	983.8 (732.4 to 1452.9)	645.2 (429.2 to 908.6)	1362.9 (967.9 to 2247.5)	726.6 (576.7 to 899.5)	497.1 (376.9 to 689.9)	1055.9 (758.2 to 1337.1)	-26.1 (-44.8 to -7.8)	-23 (-40.3 to 4.4)	-22.5 (-50.4 to 3.1)
Sub-Saharan Africa	Eastern sub-Saharan Africa	Uganda	YLLs	All Ages	52,898 (35,679 to 83,325)	16,451 (7,415 to 29,954)	36,447 (24,197 to 64,429)	73,369 (51,430 to 98,685)	24,695 (15,157 to 40,150)	48,674 (30,873 to 67,273)	38.7 (-3.8 to 90.7)	50.1 (-4.3 to 169.8)	33.5 (-19.2 to 92.1)
Sub-Saharan Africa	Eastern sub-Saharan Africa	Uganda	YLLs	Age-standardized	794.8 (545.5 to 1246.8)	441.1 (232.6 to 700.4)	1188.8 (793 to 2053.3)	537.1 (389.8 to 704.1)	312.9 (201 to 499.7)	856.2 (562.6 to 1137)	-32.4 (-52.1 to -10.3)	-29.1 (-51.5 to 12.2)	-28 (-55.6 to 0.8)
Sub-Saharan Africa	Eastern sub-Saharan Africa	Uganda	YLDs	All Ages	16,268 (13,669 to 18,641)	9,216 (7,682 to 10,746)	7,052 (5,817 to 8,317)	37,877 (30,979 to 43,760)	20,600 (16,755 to 24,409)	17,277 (14,049 to 20,441)	132.8 (117 to 149.3)	123.5 (101.1 to 148.1)	145 (123.3 to 168.7)
Sub-Saharan Africa	Eastern sub-Saharan Africa	Uganda	YLDs	Age-standardized	189 (158.7 to 216.1)	204.1 (170.1 to 239.3)	174 (142.4 to 202.4)	189.5 (157.4 to 216.1)	184.2 (153.8 to 214.7)	199.7 (163.4 to 232.6)	0.3 (-4.7 to 5.5)	-9.8 (-16.4 to -2.7)	14.7 (7.7 to 22.5)
Sub-Saharan Africa	Eastern sub-Saharan Africa	Zambia	Incidence	All Ages	4,168 (3,938 to 4,419)	1,995 (1,881 to 2,116)	2,173 (2,034 to 2,314)	10,148 (9,608 to 10,744)	4,644 (4,362 to 4,926)	5,504 (5,184 to 5,845)	143.5 (135.7 to 151.1)	132.8 (122.2 to 142.8)	153.3 (142.7 to 164.2)
Sub-Saharan Africa	Eastern sub-Saharan Africa	Zambia	Incidence	Age-standardized	104.9 (98.8 to 111.5)	92.4 (86.7 to 99.1)	115.9 (109.1 to 123.2)	105.4 (99.7 to 111.6)	87.6 (82 to 93.1)	126.7 (119.7 to 134.4)	0.5 (-2.6 to 3.7)	-5.3 (-9.1 to -1.6)	9.3 (4.7 to 13.9)
Sub-Saharan Africa	Eastern sub-Saharan Africa	Zambia	Prevalence	All Ages	59,144 (55,677 to 62,693)	29,794 (28,002 to 31,513)	29,350 (27,200 to 31,576)	152,038 (141,443 to 161,813)	74,077 (68,701 to 79,193)	77,962 (72,041 to 83,643)	157.1 (147.7 to 166.3)	148.6 (134.6 to 161.7)	165.6 (152.6 to 178.3)
Sub-Saharan Africa	Eastern sub-Saharan Africa	Zambia	Prevalence	Age-standardized	1521.9 (1427.7 to 1627.9)	1499.9 (1408 to 1604.8)	1541.6 (1435.1 to 1653.8)	1650 (1551.1 to 1755.4)	1514.1 (1418.1 to 1617.2)	1815.6 (1701.4 to 1935.9)	8.4 (4.9 to 11.9)	1 (-3.7 to 5.2)	17.8 (12.6 to 23.2)
Sub-Saharan Africa	Eastern sub-Saharan Africa	Zambia	Deaths	All Ages	801 (631 to 1,013)	256 (155 to 391)	546 (413 to 747)	1,462 (1,150 to 1,793)	500 (328 to 697)	963 (738 to 1,206)	82.5 (34.4 to 137.4)	95.5 (39.2 to 198.9)	76.5 (16 to 142.1)
Sub-Saharan Africa	Eastern sub-Saharan Africa	Zambia	Deaths	Age-standardized	35.9 (27.4 to 45.4)	22.7 (15.1 to 32.4)	47.9 (35.8 to 65.5)	28.9 (22.9 to 34.9)	18 (12 to 24.6)	42.8 (33.6 to 52.4)	-19.5 (-40.7 to 5.1)	-21 (-42.1 to 14.5)	-10.7 (-40.6 to 21.4)
Sub-Saharan Africa	Eastern sub-Saharan Africa	Zambia	DALYs	All Ages	26,655 (22,401 to 32,721)	11,215 (7,450 to 16,648)	15,440 (12,630 to 19,721)	51,995 (43,565 to 60,762)	21,483 (16,928 to 27,062)	30,512 (24,809 to 37,018)	95.1 (57.8 to 137.6)	91.6 (43.5 to 159.5)	97.6 (45.8 to 151.2)
Sub-Saharan Africa	Eastern sub-Saharan Africa	Zambia	DALYs	Age-standardized	844.5 (691.6 to 1026.7)	643.8 (464.4 to 877.2)	1018.6 (806 to 1332.8)	720 (599.7 to 842.3)	527.9 (411.3 to 671)	949.8 (772.3 to 1137.8)	-14.7 (-32.4 to 4.2)	-18 (-35.6 to 6)	-6.8 (-33.4 to 20)
Sub-Saharan Africa	Eastern sub-Saharan Africa	Zambia	YLLs	All Ages	19,274 (15,191 to 25,270)	7,166 (3,573 to 12,678)	12,107 (9,284 to 16,444)	33,147 (25,578 to 41,889)	11,452 (7,223 to 16,584)	21,695 (16,142 to 28,388)	72 (24.7 to 128.5)	59.8 (2.6 to 168.3)	79.2 (19.1 to 145.3)
Sub-Saharan Africa	Eastern sub-Saharan Africa	Zambia	YLLs	Age-standardized	659 (515.6 to 831.4)	446.2 (273.5 to 676.8)	845.3 (635.9 to 1158)	520.2 (407.1 to 638.1)	328.7 (215.1 to 462.9)	747.1 (570.7 to 930.4)	-21.1 (-41.6 to 2.5)	-26.3 (-47.7 to 11.2)	-11.6 (-42 to 21.4)
Sub-Saharan Africa	Eastern sub-Saharan Africa	Zambia	YLDs	All Ages	7,381 (6,147 to 8,394)	4,048 (3,317 to 4,740)	3,333 (2,723 to 3,892)	18,848 (15,572 to 21,698)	10,031 (8,158 to 11,773)	8,817 (7,225 to 10,448)	155.4 (137.7 to 174.2)	147.8 (124.9 to 174.2)	164.6 (140.8 to 189.5)
Sub-Saharan Africa	Eastern sub-Saharan Africa	Zambia	YLDs	Age-standardized	185.5 (155.1 to 209.9)	197.6 (163 to 230.6)	173.3 (142.9 to 201)	199.8 (166.6 to 227.1)	199.1 (164.4 to 231.6)	202.6 (165 to 234.7)	7.7 (2.3 to 13.3)	0.8 (-6.3 to 8)	16.9 (9 to 24.8)
		Southern Sub-Saharan Africa	Incidence	All Ages	54,054 (50,539 to 57,804)	24,451 (22,712 to 26,330)	29,603 (27,596 to 31,612)	94,706 (87,832 to 101,940)	42,619 (39,367 to 46,313)	52,088 (48,343 to 56,137)	75.2 (71 to 79.9)	74.3 (68.7 to 81)	76 (71.4 to 80.2)
		Southern Sub-Saharan Africa	Incidence	Age-standardized	175.3 (161.8 to 188.8)	139.7 (128.2 to 151.5)	224.7 (207.6 to 241.4)	161.3 (149.8 to 174.2)	127.3 (117.2 to 138.7)	212.2 (196.5 to 229)	-7.9 (-10.2 to -5.4)	-8.9 (-12.2 to -5.3)	-5.6 (-7.7 to -3.4)
		Southern Sub-Saharan Africa	Prevalence	All Ages	758,790 (713,993 to 806,720)	373,722 (348,913 to 398,602)	385,069 (363,000 to 408,835)	1,337,479 (1,250,978 to 1,433,369)	653,948 (606,333 to 705,450)	683,531 (640,096 to 730,993)	76.3 (71.2 to 81.2)	75 (68.6 to 81.2)	77.5 (72.5 to 82.6)
		Southern Sub-Saharan Africa	Prevalence	Age-standardized	2483.9 (2321.1 to 2658.3)	2168.6 (2009.9 to 2330.2)	2952.9 (2748.5 to 3165.2)	2243.9 (2090 to 2411.7)	1918.8 (1772.8 to 2076.4)	2770.5 (2580.3 to 2980)	-9.7 (-12.2 to -7.1)	-11.5 (-14.8 to -8.3)	-6.2 (-8.6 to -3.8)
		Southern Sub-Saharan Africa	Deaths	All Ages	8,984 (7,603 to 11,178)	3,643 (2,584 to 4,851)	5,341 (4,602 to 6,890)	14,902 (13,485 to 16,303)	6,229 (5,196 to 7,289)	8,673 (7,957 to 9,467)	65.9 (35.2 to 96.2)	71 (32.9 to 126.1)	62.4 (25.6 to 88.6)
		Southern Sub-Saharan Africa	Deaths	Age-standardized	39.1 (32.8 to 48.4)	26.8 (19 to 35.8)	58.5 (50.3 to 75.1)	32.4 (29 to 35.4)	22.2 (18.7 to 26)	50 (45.4 to 54.4)	-17.1 (-32.7 to -1.8)	-17 (-35.7 to 10)	-14.5 (-33.5 to -0.9)
		Southern Sub-Saharan Africa	DALYs	All Ages	282,777 (250,450 to 328,101)	125,820 (103,228 to 147,306)	156,957 (139,875 to 191,378)	464,451 (427,686 to 502,682)	207,082 (181,015 to 231,680)	257,369 (237,691 to 277,692)	64.2 (43.6 to 82.4)	64.6 (43.9 to 89.4)	64 (35.9 to 83.2)
		Southern Sub-Saharan Africa	DALYs	Age-standardized	1001.8 (885.9 to 1170.2)	774.5 (634.3 to 921.7)	1328.7 (1177.7 to 1628.8)	838.6 (772.6 to 907.8)	639.9 (561.1 to 715.7)	1148.6 (1060.4 to 1234.1)	-16.3 (-27.4 to -6.6)	-17.4 (-29.1 to -4)	-13.6 (-28.6 to -3.3)
		Southern Sub-Saharan Africa	YLLs	All Ages	182,580 (155,405 to 226,551)	69,401 (48,949 to 89,987)	113,179 (99,086 to 146,868)	290,321 (264,291 to 319,048)	109,937 (89,886 to 129,214)	180,384 (163,663 to 197,704)	59 (29.7 to 87.3)	58.4 (25.7 to 107.1)	59.4 (23.1 to 86.1)
		Southern Sub-Saharan Africa	YLLs	Age-standardized	680.1 (580.5 to 849)	453 (321.4 to 594.6)	999.5 (867.3 to 1289.5)	550.3 (502.7 to 603.4)	357.9 (295.4 to 417)	842.2 (771.1 to 918.2)	-19.1 (-34.3 to -4.7)	-21 (-37.9 to 3.3)	-15.7 (-35.1 to -2)
		Southern Sub-Saharan Africa	YLDs	All Ages	100,197 (83,640 to 113,849)	56,419 (46,383 to 65,241)	43,777 (36,190 to 50,346)	174,130 (145,032 to 198,138)	97,145 (80,399 to 113,134)	76,985 (63,669 to 88,749)	73.8 (68.4 to 79.1)	72.2 (65.5 to 78.5)	75.9 (70.3 to 81.4)
		Southern Sub-Saharan Africa	YLDs	Age-standardized	321.7 (269.2 to 367.7)	321.5 (265 to 373.2)	329.2 (272.6 to 379)	288.2 (239.3 to 328.9)	282 (233.8 to 329.1)	306.3 (253.3 to 352.9)	-10.4 (-13.2 to -7.6)	-12.3 (-15.8 to -9.1)	-6.9 (-9.6 to -4.4)
Sub-Saharan Africa	Southern sub-Saharan Africa	Botswana	Incidence	All Ages	1,108 (1,045 to 1,172)	436 (402 to 470)	672 (634 to 711)	2,427 (2,285 to 2,580)	1,155 (1,081 to 1,231)	1,272 (1,195 to 1,359)	119.1 (111.4 to 127.4)	165.2 (151.4 to 179.7)	89.3 (80.4 to 99.9)
Sub-Saharan Africa	Southern sub-Saharan Africa	Botswana	Incidence	Age-standardized	168.6 (159 to 178.6)	114.8 (106 to 123.9)	241.9 (229.5 to 255.7)	164.5 (156 to 174.3)	133.2 (125.3 to 142)	218.1 (206.6 to 231.1)	-2.4 (-5.7 to 1.2)	16 (9.9 to 21.9)	-9.8 (-13.6 to -5.6)
Sub-Saharan Africa	Southern sub-Saharan Africa	Botswana	Prevalence	All Ages	14,865 (13,972 to 15,774)	6,589 (6,077 to 7,106)	8,275 (7,776 to 8,781)	33,850 (31,750 to 36,001)	18,276 (17,026 to 19,467)	15,574 (14,430 to 16,796)	127.7 (119.6 to 136.9)	177.4 (163.2 to 192.7)	88.2 (75.5 to 102.4)

Chronic obstructive pulmonary disease

7 GBD super region	21 GBD region	Location	Measure	Age	1990			2019			Percent change (1990 to 2019)		
					Both	Female	Male	Both	Female	Male	Both	Female	Male
Sub-Saharan Africa	Southern sub-Saharan Africa	Botswana	Prevalence	Age-standardized	2266.1 (2136.3 to 2406.4)	1746.4 (1608.3 to 1883.4)	3014.3 (2833.6 to 3195)	2265.9 (2126.7 to 2409.3)	2112.9 (1964.7 to 2254.3)	2633.9 (2474 to 2818.1)	0 (-3.6 to 4)	21 (15.2 to 27.6)	-12.6 (-17.2 to -7.4)
Sub-Saharan Africa	Southern sub-Saharan Africa	Botswana	Deaths	All Ages	253 (172 to 387)	88 (48 to 150)	165 (118 to 261)	405 (276 to 540)	150 (76 to 218)	255 (179 to 350)	60.1 (11.6 to 122.7)	69.9 (-4.3 to 180.3)	54.9 (6.3 to 125.4)
Sub-Saharan Africa	Southern sub-Saharan Africa	Botswana	Deaths	Age-standardized	56.5 (38.5 to 85.4)	35.3 (19.4 to 59.1)	89.5 (65.4 to 138.6)	39.3 (27.2 to 51.9)	25.1 (12.8 to 35.8)	64 (46.3 to 85.9)	-30.4 (-51.2 to -3.8)	-29 (-60.5 to 15.7)	-28.4 (-50.4 to 0.6)
Sub-Saharan Africa	Southern sub-Saharan Africa	Botswana	DALYs	All Ages	7,422 (5,567 to 10,502)	2,801 (1,923 to 4,091)	4,621 (3,460 to 6,909)	13,356 (10,115 to 16,596)	5,729 (4,155 to 7,260)	7,627 (5,730 to 9,967)	79.9 (36.3 to 129.6)	104.6 (40.6 to 176)	65 (18.4 to 122.4)
Sub-Saharan Africa	Southern sub-Saharan Africa	Botswana	DALYs	Age-standardized	1304.8 (969.7 to 1854.4)	862.6 (580.6 to 1282.9)	1924.1 (1470.1 to 2875.4)	997.8 (761.3 to 1235.7)	733.1 (518.5 to 934.8)	1415.1 (1088.7 to 1841.8)	-23.5 (-42.1 to -1.8)	-15 (-43.5 to 17.1)	-26.5 (-46.9 to -0.6)
Sub-Saharan Africa	Southern sub-Saharan Africa	Botswana	YLLs	All Ages	5,477 (3,607 to 8,537)	1,799 (939 to 3,047)	3,678 (2,549 to 5,940)	8,893 (5,780 to 12,182)	3,021 (1,490 to 4,526)	5,872 (3,981 to 8,179)	62.4 (11.2 to 129.3)	67.9 (-7 to 177.2)	59.7 (6.7 to 136)
Sub-Saharan Africa	Southern sub-Saharan Africa	Botswana	YLLs	Age-standardized	1015.7 (683.8 to 1574.8)	604.7 (321.2 to 1023.4)	1586.4 (1137.8 to 2534.8)	706.3 (477.1 to 946.8)	427 (213.5 to 624.7)	1124.9 (799.5 to 1546.5)	-30.5 (-51.7 to -3.1)	-29.4 (-60.7 to 15.6)	-29.1 (-51.8 to 3)
Sub-Saharan Africa	Southern sub-Saharan Africa	Botswana	YLDs	All Ages	1,945 (1,617 to 2,220)	1,001 (826 to 1,177)	943 (772 to 1,091)	4,463 (3,711 to 5,087)	2,708 (2,238 to 3,173)	1,755 (1,439 to 2,042)	129.5 (117.5 to 142.5)	170.5 (149.9 to 190)	86 (70.5 to 103.6)
Sub-Saharan Africa	Southern sub-Saharan Africa	Botswana	YLDs	Age-standardized	289.1 (241.7 to 328.6)	257.9 (212.7 to 303)	337.7 (277.7 to 385.9)	291.5 (244.5 to 331.5)	306.1 (254 to 358.1)	290.2 (240.7 to 335.3)	0.8 (-3.8 to 6)	18.7 (10.5 to 26.5)	-14 (-19.3 to -8)
Sub-Saharan Africa	Southern sub-Saharan Africa	Lesotho	Incidence	All Ages	2,102 (1,992 to 2,215)	856 (802 to 916)	1,245 (1,182 to 1,310)	2,898 (2,749 to 3,046)	1,331 (1,240 to 1,423)	1,567 (1,492 to 1,643)	37.9 (34 to 42)	55.5 (47.9 to 62.2)	25.9 (21.5 to 30.9)
Sub-Saharan Africa	Southern sub-Saharan Africa	Lesotho	Incidence	Age-standardized	195.7 (185.6 to 206.1)	141.9 (132.3 to 152.6)	273 (260.1 to 286.4)	213.2 (202.1 to 224.2)	174.5 (163.3 to 185.9)	272.7 (259.6 to 285.8)	8.9 (5.7 to 12.3)	22.9 (17.1 to 28.3)	-0.1 (-3.4 to 3.3)
Sub-Saharan Africa	Southern sub-Saharan Africa	Lesotho	Prevalence	All Ages	27,307 (25,940 to 28,622)	12,476 (11,596 to 13,386)	14,831 (14,066 to 15,518)	38,827 (37,057 to 40,680)	19,717 (18,386 to 20,995)	19,110 (18,344 to 19,857)	42.2 (38.2 to 46.6)	58 (49.9 to 65.9)	28.9 (24.5 to 34.7)
Sub-Saharan Africa	Southern sub-Saharan Africa	Lesotho	Prevalence	Age-standardized	2532.4 (2397.9 to 2664.9)	2068.6 (1918.5 to 2228.6)	3264.3 (3090.4 to 3439.6)	2815.3 (2670.7 to 2960.6)	2500 (2329.2 to 2669.7)	3368.9 (3204.1 to 3534.1)	11.2 (7.8 to 14.7)	20.8 (14.3 to 27)	3.2 (-0.8 to 8)
Sub-Saharan Africa	Southern sub-Saharan Africa	Lesotho	Deaths	All Ages	616 (418 to 869)	268 (154 to 471)	348 (219 to 531)	712 (504 to 972)	321 (167 to 492)	392 (270 to 535)	15.7 (-21.6 to 58)	19.8 (-28.9 to 85.9)	12.6 (-29.9 to 52.2)
Sub-Saharan Africa	Southern sub-Saharan Africa	Lesotho	Deaths	Age-standardized	74.1 (50.3 to 103.9)	53.2 (30.8 to 94)	112.5 (72.4 to 168.2)	70.7 (50.2 to 95.1)	52.5 (28.4 to 79.4)	104.3 (73.2 to 139.6)	-4.6 (-33.6 to 28.5)	-1.3 (-40.2 to 50.9)	-7.3 (-39 to 21.7)
Sub-Saharan Africa	Southern sub-Saharan Africa	Lesotho	DALYs	All Ages	16,362 (12,103 to 22,410)	6,932 (4,576 to 10,876)	9,430 (6,483 to 13,950)	20,889 (15,992 to 27,111)	9,365 (6,081 to 13,346)	11,524 (8,417 to 15,259)	27.7 (-10.2 to 64.8)	35.1 (-12.2 to 89.4)	22.2 (-22.3 to 59.2)
Sub-Saharan Africa	Southern sub-Saharan Africa	Lesotho	DALYs	Age-standardized	1676.6 (1234.8 to 2265.9)	1230.2 (812.5 to 1942.2)	2372.4 (1650.2 to 3464.7)	1676.7 (1289.2 to 2156)	1279.2 (829.7 to 1808.7)	2329.8 (1725.8 to 3036.9)	0 (-28.4 to 28.5)	4 (-32.1 to 45.8)	-1.8 (-35 to 25.9)
Sub-Saharan Africa	Southern sub-Saharan Africa	Lesotho	YLLs	All Ages	12,797 (8,499 to 18,819)	5,063 (2,778 to 8,992)	7,735 (4,835 to 12,231)	15,846 (10,939 to 22,023)	6,474 (3,261 to 10,235)	9,372 (6,265 to 13,135)	23.8 (-20.2 to 71.7)	27.9 (-28.3 to 102.2)	21.2 (-29.5 to 67)
Sub-Saharan Africa	Southern sub-Saharan Africa	Lesotho	YLLs	Age-standardized	1350.2 (908.1 to 1959.2)	924.7 (515.1 to 1624.8)	2004.2 (1267.8 to 3068.2)	1315.5 (920.9 to 1802.8)	918.1 (466.6 to 1435)	1955.8 (1352.7 to 2672.6)	-2.6 (-35 to 33.6)	-0.7 (-43 to 55.5)	-2.4 (-39.4 to 30.9)
Sub-Saharan Africa	Southern sub-Saharan Africa	Lesotho	YLDs	All Ages	3,565 (2,972 to 4,051)	1,869 (1,534 to 2,194)	1,696 (1,393 to 1,959)	5,043 (4,196 to 5,717)	2,892 (2,384 to 3,380)	2,152 (1,778 to 2,465)	41.5 (35.1 to 47.9)	54.7 (44 to 65.6)	26.9 (19.7 to 35.1)
Sub-Saharan Africa	Southern sub-Saharan Africa	Lesotho	YLDs	Age-standardized	326.4 (272.3 to 372)	305.5 (251.5 to 360.8)	368.1 (302.2 to 422.9)	361.2 (300.6 to 410.3)	361.1 (298.1 to 421.6)	374 (307.1 to 427.7)	10.7 (6 to 15.3)	18.2 (10.8 to 25.7)	1.6 (-3.5 to 7.7)
Sub-Saharan Africa	Southern sub-Saharan Africa	Namibia	Incidence	All Ages	1,427 (1,353 to 1,504)	576 (542 to 613)	851 (805 to 899)	2,489 (2,364 to 2,627)	986 (921 to 1,057)	1,503 (1,424 to 1,584)	74.4 (68.4 to 80.7)	71.3 (63.4 to 79.6)	76.6 (68.7 to 84.4)
Sub-Saharan Africa	Southern sub-Saharan Africa	Namibia	Incidence	Age-standardized	181.3 (172.5 to 190.9)	133.7 (125.8 to 142.6)	241.9 (230.2 to 254.4)	162.5 (154.4 to 172.1)	111.5 (104.1 to 120.4)	233.6 (221.6 to 246.8)	-10.4 (-13.1 to -7.5)	-16.6 (-20.3 to -12.1)	-3.5 (-7.4 to 0.3)
Sub-Saharan Africa	Southern sub-Saharan Africa	Namibia	Prevalence	All Ages	18,823 (17,786 to 19,847)	8,483 (7,929 to 9,031)	10,340 (9,743 to 10,966)	33,664 (31,536 to 35,779)	14,891 (13,739 to 16,015)	18,773 (17,613 to 19,916)	78.8 (71.2 to 86.2)	75.5 (65.8 to 85.2)	81.6 (72.4 to 91)
Sub-Saharan Africa	Southern sub-Saharan Africa	Namibia	Prevalence	Age-standardized	2369.1 (2241.4 to 2510)	1947.8 (1809.8 to 2076)	2924.4 (2760.3 to 3108.4)	2191.5 (2063.4 to 2329.5)	1670 (1542.6 to 1800.1)	2981.5 (2811.7 to 3164.2)	-7.5 (-10.9 to -4.1)	-14.3 (-18.7 to -9.6)	2 (-3 to 6.7)
Sub-Saharan Africa	Southern sub-Saharan Africa	Namibia	Deaths	All Ages	386 (271 to 524)	123 (65 to 218)	263 (188 to 351)	534 (403 to 686)	191 (114 to 304)	343 (270 to 415)	38.4 (7.1 to 82.7)	55.1 (5.5 to 126.9)	30.6 (-2.6 to 82.3)
Sub-Saharan Africa	Southern sub-Saharan Africa	Namibia	Deaths	Age-standardized	62.8 (44.2 to 86.3)	36.9 (19.9 to 65.5)	99.7 (73.2 to 131.7)	45 (33.5 to 58)	26.8 (16.1 to 42.9)	74.6 (59.2 to 89.2)	-28.4 (-44 to -5.9)	-27.3 (-51 to 4.8)	-25.2 (-43.3 to 3.1)
Sub-Saharan Africa	Southern sub-Saharan Africa	Namibia	DALYs	All Ages	10,306 (7,761 to 13,254)	3,687 (2,478 to 5,567)	6,620 (4,985 to 8,637)	14,596 (11,774 to 17,719)	5,506 (4,061 to 7,567)	9,089 (7,486 to 10,768)	41.6 (14.3 to 79.8)	49.4 (11.3 to 93.8)	37.3 (6.5 to 83.5)
Sub-Saharan Africa	Southern sub-Saharan Africa	Namibia	DALYs	Age-standardized	1419.1 (1076.4 to 1845.6)	920.1 (605.4 to 1406.3)	2068.2 (1580.2 to 2689.6)	1054.8 (845.9 to 1283.7)	674.6 (487.8 to 938.4)	1621 (1341.2 to 1905.3)	-25.7 (-39.8 to -6.3)	-26.7 (-45.4 to -5.2)	-21.6 (-38.8 to 4.3)
Sub-Saharan Africa	Southern sub-Saharan Africa	Namibia	YLLs	All Ages	7,856 (5,422 to 10,791)	2,411 (1,254 to 4,225)	5,444 (3,816 to 7,416)	10,259 (7,637 to 13,323)	3,285 (1,922 to 5,315)	6,973 (5,408 to 8,639)	30.6 (-1.6 to 80.4)	36.2 (-9.9 to 106.3)	28.1 (-6.3 to 83.6)
Sub-Saharan Africa	Southern sub-Saharan Africa	Namibia	YLLs	Age-standardized	1115.9 (776.3 to 1538.2)	633.2 (330.3 to 1114.9)	1739.8 (1250.9 to 2365)	777.3 (584.6 to 1008.1)	429.8 (254.6 to 696)	1289.2 (1012.2 to 1566.9)	-30.3 (-46.6 to -5.9)	-32.1 (-54.7 to 0.1)	-25.9 (-45 to 4.7)
Sub-Saharan Africa	Southern sub-Saharan Africa	Namibia	YLDs	All Ages	2,451 (2,022 to 2,788)	1,275 (1,054 to 1,483)	1,175 (962 to 1,361)	4,337 (3,591 to 4,966)	2,221 (1,829 to 2,595)	2,116 (1,736 to 2,463)	77 (67.8 to 87)	74.1 (60.6 to 87.5)	80.1 (67.8 to 93)
Sub-Saharan Africa	Southern sub-Saharan Africa	Namibia	YLDs	Age-standardized	303.2 (250.9 to 345.1)	286.9 (236.2 to 336.3)	328.4 (269.2 to 379.5)	277.5 (231.9 to 316)	244.7 (201 to 286.4)	331.8 (274.7 to 384.8)	-8.5 (-12.5 to -4.3)	-14.7 (-20.5 to -8.8)	1 (-4.7 to 7.4)
Sub-Saharan Africa	Southern sub-Saharan Africa	South Africa	Incidence	All Ages	41,936 (38,789 to 45,114)	19,279 (17,748 to 20,891)	22,657 (20,902 to 24,399)	74,161 (68,158 to 80,446)	33,168 (30,399 to 36,371)	40,993 (37,619 to 44,625)	76.8 (71.4 to 82.8)	72 (64.8 to 80)	80.9 (75.4 to 86.3)
Sub-Saharan Africa	Southern sub-Saharan Africa	South Africa	Incidence	Age-standardized	181.5 (165.8 to 197.3)	145.6 (132.6 to 159.5)	233.2 (212 to 252.8)	163 (149.7 to 177.5)	127.8 (117 to 140.4)	215.8 (198.1 to 235.1)	-10.2 (-13 to -7.2)	-12.3 (-16.1 to -8.1)	-7.5 (-10.1 to -4.8)
Sub-Saharan Africa	Southern sub-Saharan Africa	South Africa	Prevalence	All Ages	591,669 (553,135 to 632,435)	296,903 (275,469 to 318,287)	294,767 (275,464 to 314,389)	1,046,594 (970,945 to 1,127,782)	508,448 (468,027 to 551,645)	538,146 (499,184 to 581,773)	76.9 (70.2 to 83.3)	71.3 (63.5 to 78.4)	82.6 (76.3 to 88.9)
Sub-Saharan Africa	Southern sub-Saharan Africa	South Africa	Prevalence	Age-standardized	2583.2 (2398 to 2785.9)	2271.2 (2088.8 to 2462.3)	3062.2 (2828.4 to 3305.2)	2256 (2087.8 to 2439.8)	1920.4 (1766.3 to 2088.6)	2803.1 (2591.2 to 3037.6)	-12.7 (-15.8 to -9.5)	-15.4 (-19.4 to -11.7)	-8.5 (-11.2 to -5.6)
Sub-Saharan Africa	Southern sub-Saharan Africa	South Africa	Deaths	All Ages	6,805 (5,823 to 8,274)	2,811 (2,113 to 3,695)	3,994 (3,458 to 5,045)	11,843 (10,780 to 13,332)	4,983 (4,309 to 6,199)	6,860 (6,253 to 7,577)	74 (47.3 to 107)	77.3 (40.9 to 137)	71.7 (38.2 to 100.5)
Sub-Saharan Africa	Southern sub-Saharan Africa	South Africa	Deaths	Age-standardized	37.9 (32.1 to 46.2)	26.1 (19.5 to 34.4)	56.9 (48.8 to 71.5)	31.8 (28.6 to 35.9)	21.8 (18.8 to 27.1)	49.3 (44.7 to 54.1)	-16.2 (-29.2 to 0.2)	-16.4 (-34.1 to 12.3)	-13.5 (-30.5 to 0.3)
Sub-Saharan Africa	Southern sub-Saharan Africa	South Africa	DALYs	All Ages	215,656 (193,734 to 246,068)	98,505 (83,695 to 114,251)	117,151 (104,639 to 139,456)	361,520 (330,346 to 393,541)	161,712 (142,821 to 184,291)	199,808 (183,652 to 218,720)	67.6 (52.4 to 86.3)	64.2 (46.8 to 91.3)	70.6 (47.2 to 90.6)
Sub-Saharan Africa	Southern sub-Saharan Africa	South Africa	DALYs	Age-standardized	999.1 (892 to 1151.2)	784.7 (659.8 to 924.2)	1317.2 (1168.5 to 1578.6)	825.5 (758.6 to 898.1)	632.7 (560.4 to 721.8)	1128.4 (1040.6 to 1232.1)	-17.4 (-25.9 to -7.7)	-19.4 (-28.7 to -5.6)	-14.3 (-27.1 to -4.1)
Sub-Saharan Africa	Southern sub-Saharan Africa	South Africa	YLLs	All Ages	137,509 (120,301 to 164,836)	53,770 (40,578 to 67,761)	83,739 (73,319 to 105,398)	225,719 (206,694 to 253,944)	86,346 (74,965 to 107,672)	139,373 (125,864 to 155,763)	64.1 (41.1 to 94.2)	60.6 (32.4 to 111.5)	66.4 (36.8 to 94.3)
Sub-Saharan Africa	Southern sub-Saharan Africa	South Africa	YLLs	Age-standardized	663.9 (575.5 to 802.5)	447.7 (339.3 to 575.7)	976.7 (848.8 to 1230.1)	536 (490 to 604.8)	350.1 (304.2 to 436.8)	819.1 (746.2 to 907.8)	-19.3 (-31.4 to -4.3)	-21.8 (-36.3 to 3.2)	-16.1 (-32.4 to -2.2)
Sub-Saharan Africa	Southern sub-Saharan Africa	South Africa	YLDs	All Ages	78,147 (65,130 to 89,278)	44,735 (36,675 to 51,995)	33,412 (27,713 to 38,476)	135,801 (112,436 to 155,679)	75,366 (62,394 to 88,465)	60,435 (49,988 to 70,027)	73.8 (67.1 to 80.5)	68.5 (60.2 to 76.2)	80.9 (73.8 to 88.2)
Sub-Saharan Africa	Southern sub-Saharan Africa	South Africa	YLDs	Age-standardized	335.2 (278.9 to 384.5)	337.1 (275.2 to 392.5)	340.4 (280.9 to 394.5)	289.5 (240.4 to 331.3)	282.6 (233.5 to 333.4)	309.2 (254.9 to 358.9)	-13.6 (-16.8 to -10.4)	-16.2 (-20.1 to -12.4)	-9.2 (-12.3 to -6)
Sub-Saharan Africa	Southern sub-Saharan Africa	Eswatini	Incidence	All Ages	710 (679 to 742)	309 (291 to 327)	401 (382 to 420)	1,189 (1,130 to 1,250)	494 (461 to 530)	694 (662 to 727)	67.4 (62.8 to 72.4)	60 (52.8 to 67.8)	73 (67.2 to 79.5)
Sub-Saharan Africa	Southern sub-Saharan Africa	Eswatini	Incidence	Age-standardized	195.9 (186.4 to 205.5)	146.1 (137 to 155)	269.2 (256.3 to 282.9)	179.8 (170.8 to 189.2)	132.3 (123.7 to 142)	252.3 (240.1 to 264.9)	-8.2 (-10.7 to -5.6)	-9.5 (-13.5 to -5.2)	-6.3 (-9.1 to -3.1)
Sub-Saharan Africa	Southern sub-Saharan Africa	Eswatini	Prevalence	All Ages	9,148 (8,719 to 9,553)	4,361 (4,086 to 4,632)	4,786 (4,534 to 4,995)	16,362 (15,605 to 17,119)	7,594 (7,042 to 8,138)	8,769 (8,416 to 9,102)	78.9 (73.5 to 84.8)	74.1 (65.1 to 83)	83.2 (76.8 to 90.5)
Sub-Saharan Africa	Southern sub-Saharan Africa	Eswatini	Prevalence	Age-standardized	2602.9 (2463.8 to 2735.5)	2154.7 (2005.9 to 2302.7)	3307.4 (3117.6 to 3485.3)	2476.7 (2347.7 to 2605.9)	1976.4 (1836.7 to 2121.9)	3332.7 (3162.7 to 3491.4)	-4.8 (-7.7 to -1.6)	-8.3 (-12.6 to -3.6)	0.8 (-2.9 to 5.2)
Sub-Saharan Africa	Southern sub-Saharan Africa	Eswatini	Deaths	All Ages	135 (95 to 191)	47 (24 to 80)	88 (64 to 117)	192 (143 to 253)	66 (36 to 100)	126 (98 to 160)	42.2 (-1.7 to 99)	40.1 (-18.4 to 134.9)	43.3 (-4.4 to 97.3)
Sub-Saharan Africa	Southern sub-Saharan Africa	Eswatini	Deaths	Age-standardized	58.3 (40.9 to 83.3)	34.8 (18.2 to 58.8)	99.7 (73.3 to 130.9)	42.2 (31.3 to 55)	24.6 (13.7 to 36.6)	77.7 (61.5 to 94.9)	-27.5 (-48.1 to -0.4)	-29.3 (-57.4 to 15.8)	-22 (-45 to 4.5)
Sub-Saharan Africa	Southern sub-Saharan Africa	Eswatini	DALYs	All Ages	4,180 (3,264 to 5,483)	1,610 (1,120 to 2,307)	2,570 (1,989 to 3,361)	6,411 (5,181 to 7,935)	2,409 (1,768 to 3,158)	4,001 (3,249 to 4,942)	53.4 (15.2 to 96.8)	49.6 (8.3 to 102.4)	55.7 (7.8 to 104.5)
Sub-Saharan Africa	Southern sub-Saharan Africa	Eswatini	DALYs	Age-standardized	1403.7 (1085.8 to 1866.4)	916.4 (620.3 to 1348.1)	2145.2 (1648.9 to 2752.1)	1092.7 (881.9 to 1347.7)	689.5 (496.3 to 913.9)	1787.9 (1474.3 to 2174)	-22.2 (-41.6 to 0)	-24.8 (-47 to 3.6)	-16.7 (-39.8 to 7.8)
Sub-Saharan Africa	Southern sub-Saharan Africa	Eswatini	YLLs	All Ages	2,969 (2,099 to 4,214)	946 (477 to 1,606)	2,022 (1,455 to 2,824)	4,300 (3,102 to 5,765)	1,283 (670 to 2,008)	3,016 (2,271 to 3,965)	44.8 (-2.6 to 107.1)	35.6 (-20.9 to 135.5)	49.2 (-5 to 111.9)
Sub-Saharan Africa	Southern sub-Saharan Africa	Eswatini	YLLs	Age-standardized	1068.2 (754.4 to 1515.5)	598.6 (310.3 to 1021)	1774.4 (1303.8 to 2374.6)	778.7 (572.2 to 1026.4)	403 (215.6 to 621)	1420.1 (1110.3 to 1797.5)	-27.1 (-49.5 to 2)	-32.7 (-60.5 to 13)	-20 (-46 to 9.9)
Sub-Saharan Africa	Southern sub-Saharan Africa	Eswatini	YLDs	All Ages	1,212 (1,011 to 1,376)	664 (543 to 777)	547 (454 to 627)	2,111 (1,770 to 2,395)	1,126 (926 to 1,320)	985 (816 to 1,132)	74.2 (65 to 83.4)	69.6 (57.4 to 82.5)	79.9 (68.8 to 92.6)
Sub-Saharan Africa	Southern sub-Saharan Africa	Eswatini	YLDs	Age-standardized	335.5 (278.9 to 381.9)	317.8 (259.9 to 375.7)	370.8 (307.7 to 424.4)	314 (262 to 354.5)	286.5 (236.9 to 335.7)	367.8 (305.7 to 422.2)	-6.4 (-10.3 to -2.4)	-9.8 (-15.4 to -4.1)	-0.8 (-5.9 to 4.8)
Sub-Saharan Africa	Southern sub-Saharan Africa	Zimbabwe	Incidence	All Ages	6,772 (6,407 to 7,164)	2,995 (2,805 to 3,182)	3,776 (3,573 to 4,006)	11,542 (10,890 to 12,241)	5,484 (5,112 to 5,895)	6,058 (5,704 to 6,432)	70.4 (64.5 to 76.9)	83.1 (74.6 to 91.5)	60.4 (53.2 to 68.4)
Sub-Saharan Africa	Southern sub-Saharan Africa	Zimbabwe	Incidence	Age-standardized	132.3 (125.2 to 140.1)	108.6 (101.9 to 115.7)	161.9 (153.6 to 171.7)	133.3 (125.5 to 141.2)	113 (105.2 to 121.1)	161.7 (152.6 to 170.9)	0.7 (-2.5 to 4.2)	4 (-0.9 to 8.7)	-0.1 (-3.9 to 4.7)
Sub-Saharan Africa	Southern sub-Saharan Africa	Zimbabwe	Prevalence	All Ages	96,979 (91,167 to 103,222)	44,909 (41,427 to 48,131)	52,070 (48,917 to 55,383)	168,183 (157,789 to 179,079)	85,023 (78,377 to 91,777)	83,160 (78,183 to 88,486)	73.4 (67.4 to 79.6)	89.3 (80.3 to 98.3)	59.7 (52.8 to 67.9)
Sub-Saharan Africa	Southern sub-Saharan Africa	Zimbabwe	Prevalence	Age-standardized	1944.5 (1832 to 2065.4)	1702.7 (1585.6 to 1824.4)	2238.5 (2108.2 to 2393.6)	1991.8 (1861.4 to 2134.2)	1779.2 (1641.8 to 1923.8)	2299.2 (2141.1 to 2453.5)	2.4 (-1 to 6)	4.5 (-0.5 to 9.4)	2.7 (-1.7 to 7.8)
Sub-Saharan Africa	Southern sub-Saharan Africa	Zimbabwe	Deaths	All Ages	790 (628 to 962)	306 (157 to 407)	484 (392 to 612)	1,217 (842 to 1,582)	519 (237 to 780)	698 (474 to 921)	53.9 (-10.4 to 108.4)	69.6 (-12.1 to 162.3)	44 (-17.6 to 98)
Sub-Saharan Africa	Southern sub-Saharan Africa	Zimbabwe	Deaths	Age-standardized	26.5 (20.8 to 31.7)	19.1 (9.8 to 25.3)	37.5 (31.1 to 46.3)	23.9 (16.7 to 31.1)	17.6 (8.4 to 26.3)	34.3 (25.1 to 44.1)	-9.5 (-44.5 to 20.2)	-7.7 (-52.2 to 40.1)	-8.6 (-42.9 to 20.3)
Sub-Saharan Africa	Southern sub-Saharan Africa	Zimbabwe	DALYs	All Ages	28,850 (24,994 to 33,054)	12,285 (9,262 to 14,680)	16,565 (14,226 to 19,413)	47,680 (38,959 to 56,099)	22,359 (16,657 to 28,103)	25,320 (19,819 to 30,648)	65.3 (22.4 to 97.6)	82 (38.6 to 124.6)	52.9 (6.9 to 88.7)
Sub-Saharan Africa	Southern sub-Saharan Africa	Zimbabwe	DALYs	Age-standardized	690.1 (590.6 to 792.9)	546.1 (393.9 to 660)	869.7 (749.6 to 1035)	664.1 (531.9 to 787.4)	537.1 (386.1 to 688.9)	853.9 (661.8 to 1039.9)	-3.8 (-31 to 16.8)	-1.7 (-28.7 to 24.6)	-1.8 (-32.8 to 21.9)
Sub-Saharan Africa	Southern sub-Saharan Africa	Zimbabwe	YLLs	All Ages	15,972 (12,674 to 19,924)	5,411 (2,720 to 7,259)	10,561 (8,427 to 13,616)	25,305 (16,898 to 33,012)	9,527 (4,240 to 14,684)	15,778 (10,183 to 20,903)	58.4 (-12.1 to 118.7)	76.1 (-10.3 to 185.7)	49.4 (-19.3 to 107.2)
Sub-Saharan Africa	Southern sub-Saharan Africa	Zimbabwe	YLLs	Age-standardized	437.9 (348.7 to 537.9)	293.7 (150.5 to 390.2)	615.3 (499.3 to 777.9)	404.4 (278.7 to 525)	275.5 (124.9 to 414.8)	593.7 (405.9 to 779.8)	-7.6 (-47.2 to 25)	-6.2 (-51.7 to 46.8)	-3.5 (-44.2 to 30.8)
Sub-Saharan Africa	Southern sub-Saharan Africa	Zimbabwe	YLDs	All Ages	12,878 (10,644 to 14,784)	6,874 (5,604 to 8,132)	6,004 (4,932 to 7,029)	22,375 (18,389 to 25,608)	12,832 (10,482 to 15,133)	9,543 (7,802 to 11,091)	73.7 (64 to 84)	86.7 (72.1 to 101.9)	58.9 (46.7 to 71.4)
Sub-Saharan Africa	Southern sub-Saharan Africa	Zimbabwe	YLDs	Age-standardized	252.3 (209.2 to 288)	252.5 (207.5 to 297.4)	254.4 (209.1 to 293.7)	259.8 (214 to 296.4)	261.6 (216.9 to 306)	260.2 (213.4 to 301.1)	3 (-1.9 to 8.2)	3.6 (-3.8 to 13.1)	2.2 (-4 to 9.3)
		Western Sub-Saharan Africa	Incidence	All Ages	119,637 (112,651 to 127,318)	60,590 (57,013 to 64,406)	59,046 (55,523 to 63,034)	258,354 (243,875 to 273,678)	134,805 (126,924 to 143,474)	123,549 (116,888 to 130,347)	115.9 (110.6 to 122.1)	122.5 (117 to 128.6)	109.2 (103.3 to 116.3)
		Western Sub-Saharan Africa	Incidence	Age-standardized	108.5 (100.4 to 116.5)	107.3 (99.2 to 115.6)	113.5 (105.5 to 121.7)	100.9 (94.4 to 107.9)	97.6 (90.9 to 104.7)	105 (98.3 to 111.7)	-6.9 (-8.8 to -4.2)	-9.1 (-11 to -6)	-7.5 (-9.3 to -5)
		Western Sub-Saharan Africa	Prevalence	All Ages	1,808,146 (1,713,379 to 1,917,468)	939,606 (888,171 to 997,643)	868,539 (822,181 to 920,963)	3,991,683 (3,783,437 to 4,209,533)	2,143,330 (2,028,384 to 2,267,911)	1,848,353 (1,753,378 to 1,944,984)	120.8 (114.8 to 126.7)	128.1 (121.7 to 134.7)	112.8 (106.9 to 119.2)
		Western Sub-Saharan Africa	Prevalence	Age-standardized	1695.3 (1588.4 to 1813.5)	1786.1 (1666.1 to 1917.1)	1624.7 (1526.5 to 1733.6)	1618.4 (1521.6 to 1721.3)	1664.6 (1559.2 to 1779.2)	1571.3 (1481 to 1664.6)	-4.5 (-6.5 to -2)	-6.8 (-9.1 to -4.1)	-3.3 (-5.5 to -0.4)
		Western Sub-Saharan Africa	Deaths	All Ages	21,048 (17,374 to 25,154)	7,941 (5,988 to 10,099)	13,107 (10,310 to 16,595)	35,495 (29,696 to 40,964)	12,994 (9,600 to 15,881)	22,501 (17,812 to 27,158)	68.6 (36.1 to 98)	63.6 (32.1 to 102.7)	71.7 (22.2 to 115)
		Western Sub-Saharan Africa	Deaths	Age-standardized	29.5 (24.4 to 34.8)	20.6 (15.6 to 26.1)	40.8 (32.4 to 50.6)	24.3 (20.5 to 27.8)	16.4 (12.3 to 19.9)	33.4 (26.7 to 39.9)	-17.5 (-32.3 to -3.8)	-20.1 (-35.7 to -1.2)	-18.1 (-40.4 to 0.2)
		Western Sub-Saharan Africa	DALYs	All Ages	684,909 (590,018 to 794,427)	300,827 (240,657 to 358,660)	384,082 (319,065 to 477,065)	1,256,880 (1,101,974 to 1,409,706)	562,839 (465,877 to 654,376)	694,041 (586,958 to 809,441)	83.5 (56.5 to 106.8)	87.1 (63.1 to 115.8)	80.7 (38 to 117.4)
		Western Sub-Saharan Africa	DALYs	Age-standardized	737.1 (633.9 to 857.4)	612.1 (500.7 to 727.6)	882.6 (728 to 1084.7)	619 (542.8 to 692.7)	502.8 (411.9 to 582.6)	748.6 (634.2 to 874.7)	-16 (-29 to -5.6)	-17.9 (-28.8 to -5.4)	-15.2 (-35.4 to 1.5)
		Western Sub-Saharan Africa	YLLs	All Ages	469,377 (381,822 to 571,839)	185,079 (128,382 to 241,764)	284,298 (221,250 to 375,933)	776,652 (639,678 to 918,618)	296,738 (208,102 to 373,963)	479,914 (377,933 to 594,414)	65.5 (30.5 to 97.5)	60.3 (27.7 to 105.9)	68.8 (16 to 118.9)
		Western Sub-Saharan Africa	YLLs	Age-standardized	538.2 (442 to 646.3)	396.1 (293.2 to 502.3)	698.8 (548.2 to 893.3)	427.8 (355.3 to 496.4)	300.5 (220 to 369.8)	569.3 (450.2 to 690.8)	-20.5 (-36.7 to -6.5)	-24.1 (-39.3 to -5.2)	-18.5 (-42.5 to 2.4)
		Western Sub-Saharan Africa	YLDs	All Ages	215,532 (179,785 to 243,719)	115,748 (96,273 to 132,975)	99,783 (82,410 to 114,743)	480,228 (403,110 to 542,409)	266,102 (221,897 to 304,578)	214,127 (177,107 to 245,688)	122.8 (116.1 to 129.1)	129.9 (122.3 to 137.4)	114.6 (107.2 to 121.8)
		Western Sub-Saharan Africa	YLDs	Age-standardized	198.9 (165.8 to 225.4)	216.1 (179.7 to 249.8)	183.8 (152 to 209.8)	191.2 (160.6 to 215.7)	202.4 (169.1 to 232.8)	179.3 (148.8 to 205.5)	-3.9 (-6.2 to -1.2)	-6.4 (-8.9 to -3.4)	-2.4 (-5.3 to 0.4)
Sub-Saharan Africa	Western sub-Saharan Africa	Benin	Incidence	All Ages	3,228 (3,072 to 3,387)	1,595 (1,508 to 1,683)	1,633 (1,550 to 1,718)	8,190 (7,776 to 8,618)	4,335 (4,088 to 4,579)	3,855 (3,666 to 4,069)	153.7 (147.3 to 160.5)	171.8 (162.9 to 182)	136 (127.8 to 144.2)
Sub-Saharan Africa	Western sub-Saharan Africa	Benin	Incidence	Age-standardized	126 (118.6 to 133.5)	114.1 (107 to 122)	139.6 (131.5 to 147.7)	120.6 (113.8 to 127.8)	119.6 (112 to 127.5)	124.6 (117.5 to 132.2)	-4.3 (-6.7 to -1.7)	4.8 (1.1 to 8.8)	-10.7 (-14.1 to -7.4)
Sub-Saharan Africa	Western sub-Saharan Africa	Benin	Prevalence	All Ages	46,526 (44,040 to 48,894)	24,241 (22,890 to 25,492)	22,286 (21,070 to 23,533)	122,253 (116,116 to 128,660)	66,179 (62,340 to 69,920)	56,074 (53,240 to 58,977)	162.8 (155.7 to 169.9)	173 (163.3 to 183.2)	151.6 (141.9 to 160.6)
Sub-Saharan Africa	Western sub-Saharan Africa	Benin	Prevalence	Age-standardized	1904 (1784.8 to 2020.4)	1868.6 (1744.1 to 1986.9)	1952.2 (1831 to 2077.1)	1928 (1809.2 to 2048.9)	2002.9 (1868.2 to 2133)	1856.2 (1748.9 to 1973.8)	1.3 (-1.5 to 4.2)	7.2 (3.1 to 11.6)	-4.9 (-8.7 to -1.1)
Sub-Saharan Africa	Western sub-Saharan Africa	Benin	Deaths	All Ages	664 (534 to 816)	235 (169 to 312)	430 (322 to 562)	1,016 (779 to 1,321)	402 (282 to 561)	613 (441 to 816)	52.9 (16.7 to 105.4)	71.6 (27.3 to 131.7)	42.8 (-1.4 to 109.2)
Sub-Saharan Africa	Western sub-Saharan Africa	Benin	Deaths	Age-standardized	37.7 (30.2 to 46)	24.8 (17.8 to 33.2)	52.4 (39.4 to 68.5)	25.9 (20.1 to 32.8)	18.2 (12.9 to 25)	36.8 (27.1 to 47.7)	-31.3 (-47.1 to -9.1)	-26.7 (-44.9 to -1.7)	-29.8 (-51.6 to -0.3)
Sub-Saharan Africa	Western sub-Saharan Africa	Benin	DALYs	All Ages	19,631 (16,407 to 23,274)	8,423 (6,652 to 10,363)	11,209 (8,970 to 14,407)	37,517 (31,435 to 45,489)	17,356 (13,836 to 21,488)	20,160 (15,918 to 25,537)	91.1 (56 to 135.1)	106.1 (70.2 to 150.9)	79.9 (33.8 to 142.3)
Sub-Saharan Africa	Western sub-Saharan Africa	Benin	DALYs	Age-standardized	909.7 (761.9 to 1075.4)	704.6 (558.6 to 871.4)	1135.1 (902.2 to 1448.7)	692.6 (581.3 to 841.6)	579.9 (463.2 to 725)	838.7 (659.4 to 1051.1)	-23.9 (-37.9 to -5.2)	-17.7 (-32.2 to 0.4)	-26.1 (-45.6 to -0.3)
Sub-Saharan Africa	Western sub-Saharan Africa	Benin	YLLs	All Ages	14,073 (11,011 to 17,651)	5,405 (3,748 to 7,246)	8,668 (6,418 to 11,828)	22,788 (16,740 to 30,713)	9,091 (5,955 to 13,048)	13,696 (9,547 to 19,032)	61.9 (19 to 122.8)	68.2 (19.5 to 137.9)	58 (4.8 to 138.9)
Sub-Saharan Africa	Western sub-Saharan Africa	Benin	YLLs	Age-standardized	685.1 (541.8 to 850.7)	475.6 (338.8 to 633.5)	914.7 (683.4 to 1229.8)	463.7 (352.1 to 611.5)	333.9 (229.2 to 472.5)	627.9 (451.6 to 839.4)	-32.3 (-48.8 to -7.4)	-29.8 (-48.7 to -3.5)	-31.4 (-53.8 to 1)
Sub-Saharan Africa	Western sub-Saharan Africa	Benin	YLDs	All Ages	5,558 (4,630 to 6,331)	3,018 (2,498 to 3,506)	2,540 (2,072 to 2,967)	14,729 (12,258 to 16,806)	8,265 (6,783 to 9,581)	6,464 (5,249 to 7,592)	165 (150 to 180.5)	173.9 (154.2 to 195.4)	154.4 (135.4 to 173.5)
Sub-Saharan Africa	Western sub-Saharan Africa	Benin	YLDs	Age-standardized	224.6 (186.4 to 256.3)	229.1 (189.5 to 265.5)	220.4 (180.4 to 257.1)	228.9 (191.7 to 261.3)	246 (200.5 to 285.9)	210.9 (171.8 to 244.8)	1.9 (-3.1 to 6.8)	7.4 (0.4 to 14.1)	-4.3 (-10.3 to 1.3)
Sub-Saharan Africa	Western sub-Saharan Africa	Burkina Faso	Incidence	All Ages	4,633 (4,392 to 4,898)	2,537 (2,405 to 2,684)	2,095 (1,960 to 2,235)	11,231 (10,682 to 11,812)	6,024 (5,702 to 6,368)	5,207 (4,930 to 5,504)	142.4 (135.6 to 149.7)	137.4 (128.3 to 146.4)	148.5 (138.3 to 159.4)
Sub-Saharan Africa	Western sub-Saharan Africa	Burkina Faso	Incidence	Age-standardized	82.7 (78.1 to 88)	80.5 (75.7 to 86.1)	85.1 (80.1 to 90.9)	85.2 (80.6 to 90.4)	82.7 (77.9 to 88.5)	89.5 (84.6 to 95.1)	2.9 (0 to 5.7)	2.7 (-1.3 to 6.6)	5.1 (0.6 to 9.4)
Sub-Saharan Africa	Western sub-Saharan Africa	Burkina Faso	Prevalence	All Ages	72,450 (68,515 to 76,226)	41,605 (39,546 to 43,747)	30,846 (28,494 to 33,068)	180,238 (170,496 to 189,858)	99,797 (94,007 to 105,216)	80,441 (74,946 to 85,269)	148.8 (140.8 to 156.2)	139.9 (129.6 to 149.4)	160.8 (149.1 to 174.4)
Sub-Saharan Africa	Western sub-Saharan Africa	Burkina Faso	Prevalence	Age-standardized	1344.5 (1269.6 to 1422.4)	1432 (1351.8 to 1515.4)	1246.8 (1160.4 to 1335.2)	1452 (1372.1 to 1534.4)	1490.7 (1401.9 to 1580)	1411.9 (1326.9 to 1502.3)	8 (4.8 to 10.9)	4.1 (0 to 8.1)	13.2 (8.1 to 18.3)
Sub-Saharan Africa	Western sub-Saharan Africa	Burkina Faso	Deaths	All Ages	781 (609 to 988)	316 (220 to 437)	465 (336 to 645)	1,391 (1,122 to 1,724)	576 (403 to 761)	815 (627 to 1,059)	78 (35.3 to 131)	82.1 (34.1 to 164.4)	75.2 (23.6 to 138.3)
Sub-Saharan Africa	Western sub-Saharan Africa	Burkina Faso	Deaths	Age-standardized	22.8 (18.1 to 28.8)	16 (11.2 to 22.5)	30.4 (22.5 to 40.7)	19 (15.5 to 23.2)	13.4 (9.7 to 17.8)	27 (21.3 to 33.8)	-16.3 (-35.2 to 6.2)	-16.4 (-38.3 to 18.8)	-11.1 (-37.8 to 16.3)
Sub-Saharan Africa	Western sub-Saharan Africa	Burkina Faso	DALYs	All Ages	27,063 (22,310 to 32,247)	13,594 (10,479 to 17,454)	13,469 (10,419 to 17,924)	55,148 (46,638 to 65,580)	27,665 (21,515 to 35,014)	27,482 (22,213 to 34,092)	103.8 (67.3 to 144.4)	103.5 (62.1 to 166.7)	104 (55 to 157.1)
Sub-Saharan Africa	Western sub-Saharan Africa	Burkina Faso	DALYs	Age-standardized	571.4 (475.2 to 682.6)	492.2 (390.1 to 606.4)	659.8 (515.8 to 874.5)	523.9 (448.2 to 609.8)	447.2 (358.4 to 537.5)	627.2 (509.7 to 767)	-8.3 (-24.6 to 9.8)	-9.1 (-25.3 to 14.1)	-4.9 (-28 to 21.1)
Sub-Saharan Africa	Western sub-Saharan Africa	Burkina Faso	YLLs	All Ages	18,325 (13,813 to 23,538)	8,387 (5,491 to 12,107)	9,938 (7,109 to 14,478)	33,244 (25,736 to 43,299)	15,073 (9,420 to 22,139)	18,170 (13,470 to 24,831)	81.4 (33.8 to 143.7)	79.7 (23.6 to 183.6)	82.8 (27.6 to 151.7)
Sub-Saharan Africa	Western sub-Saharan Africa	Burkina Faso	YLLs	Age-standardized	411.2 (320 to 524.1)	315.6 (219.2 to 436.1)	518.5 (375.6 to 726.4)	349.8 (281.7 to 434.3)	262.1 (180.1 to 345.2)	465.6 (357.3 to 606.1)	-14.9 (-35.7 to 10.4)	-16.9 (-39.4 to 21.4)	-10.2 (-37 to 22.9)
Sub-Saharan Africa	Western sub-Saharan Africa	Burkina Faso	YLDs	All Ages	8,739 (7,237 to 10,036)	5,208 (4,249 to 6,076)	3,531 (2,879 to 4,205)	21,904 (18,287 to 25,112)	12,592 (10,315 to 14,782)	9,312 (7,614 to 10,880)	150.7 (135.4 to 167.3)	141.8 (122.4 to 162.3)	163.7 (140.4 to 189.4)
Sub-Saharan Africa	Western sub-Saharan Africa	Burkina Faso	YLDs	Age-standardized	160.1 (133.6 to 182.5)	176.6 (143.6 to 205)	141.3 (115.5 to 165.6)	174.1 (145.6 to 198)	185.1 (151.4 to 216.1)	161.7 (134 to 187.1)	8.8 (3.4 to 14.7)	4.8 (-2.1 to 12.4)	14.4 (6.3 to 23.4)
Sub-Saharan Africa	Western sub-Saharan Africa	Cameroon	Incidence	All Ages	8,395 (7,947 to 8,854)	4,520 (4,249 to 4,774)	3,875 (3,669 to 4,093)	22,357 (21,162 to 23,592)	11,530 (10,857 to 12,238)	10,827 (10,234 to 11,460)	166.3 (158.4 to 174.5)	155.1 (144.9 to 166.2)	179.4 (170.2 to 189.7)
Sub-Saharan Africa	Western sub-Saharan Africa	Cameroon	Incidence	Age-standardized	150 (141.7 to 158.8)	152.7 (143.4 to 162.5)	148.6 (140.4 to 156.7)	141.7 (133.8 to 150.5)	140.3 (131.6 to 149.7)	145.7 (137 to 154.7)	-5.6 (-8.3 to -2.8)	-8.1 (-11.9 to -4.2)	-2 (-5.3 to 1.6)
Sub-Saharan Africa	Western sub-Saharan Africa	Cameroon	Prevalence	All Ages	120,996 (114,480 to 127,614)	66,777 (62,648 to 70,665)	54,219 (50,930 to 57,469)	333,003 (315,180 to 351,183)	174,853 (165,034 to 185,277)	158,150 (149,597 to 167,183)	175.2 (167.1 to 183.5)	161.8 (151.1 to 174.7)	191.7 (181.6 to 202.6)
Sub-Saharan Africa	Western sub-Saharan Africa	Cameroon	Prevalence	Age-standardized	2254.8 (2117.3 to 2394.6)	2433.6 (2260 to 2592)	2075.3 (1950.5 to 2211.9)	2216 (2082.3 to 2360)	2283.1 (2129.3 to 2444.4)	2154.7 (2023.7 to 2290.7)	-1.7 (-4.9 to 1.5)	-6.2 (-10.3 to -1.4)	3.8 (0.2 to 8.2)
Sub-Saharan Africa	Western sub-Saharan Africa	Cameroon	Deaths	All Ages	1,294 (990 to 1,578)	483 (306 to 633)	811 (602 to 1,039)	2,311 (1,583 to 3,034)	855 (540 to 1,185)	1,456 (895 to 1,945)	78.6 (29.6 to 143.8)	77.2 (22.5 to 146.6)	79.5 (14.6 to 161.7)
Sub-Saharan Africa	Western sub-Saharan Africa	Cameroon	Deaths	Age-standardized	37.6 (29 to 45.5)	25.9 (16.5 to 33.7)	51.3 (38.6 to 65.7)	25 (17.8 to 32.3)	17.2 (11.1 to 23.6)	35.3 (23 to 45.7)	-33.3 (-50.3 to -11.9)	-33.4 (-52.9 to -8)	-31.2 (-53.4 to -3.4)
Sub-Saharan Africa	Western sub-Saharan Africa	Cameroon	DALYs	All Ages	43,436 (35,987 to 50,519)	19,668 (15,234 to 23,666)	23,768 (18,970 to 29,184)	93,436 (73,645 to 113,581)	41,262 (32,666 to 50,312)	52,173 (37,992 to 65,682)	115.1 (75.4 to 165.1)	109.8 (69.9 to 153.6)	119.5 (62.5 to 191.2)
Sub-Saharan Africa	Western sub-Saharan Africa	Cameroon	DALYs	Age-standardized	939.6 (775.7 to 1094.3)	786.3 (602.4 to 948)	1111.1 (875.9 to 1363.5)	713.9 (564.3 to 865.5)	592.5 (467.9 to 727.2)	858.2 (625.5 to 1073.1)	-24 (-38 to -5.8)	-24.7 (-40 to -8.5)	-22.8 (-42.7 to 1.9)
Sub-Saharan Africa	Western sub-Saharan Africa	Cameroon	YLLs	All Ages	29,041 (22,274 to 35,583)	11,419 (7,267 to 15,179)	17,621 (12,994 to 22,949)	53,612 (34,746 to 72,911)	19,521 (11,839 to 27,655)	34,091 (20,042 to 47,222)	84.6 (26.5 to 161.2)	70.9 (16.6 to 144.2)	93.5 (16.8 to 190.4)
Sub-Saharan Africa	Western sub-Saharan Africa	Cameroon	YLLs	Age-standardized	675.2 (517.7 to 826.2)	490.6 (311.1 to 646.3)	878.8 (651.1 to 1130.2)	453.4 (307.3 to 598.6)	314.1 (196 to 440.5)	616.1 (378.8 to 824.4)	-32.8 (-51.5 to -7.7)	-36 (-56.2 to -10.1)	-29.9 (-55.2 to 1.9)
Sub-Saharan Africa	Western sub-Saharan Africa	Cameroon	YLDs	All Ages	14,396 (12,060 to 16,406)	8,249 (6,799 to 9,596)	6,147 (4,986 to 7,224)	39,824 (33,428 to 45,642)	21,742 (17,960 to 25,316)	18,082 (14,884 to 21,224)	176.6 (162.7 to 191.8)	163.6 (146.3 to 183.4)	194.2 (173.2 to 217.1)
Sub-Saharan Africa	Western sub-Saharan Africa	Cameroon	YLDs	Age-standardized	264.4 (220.6 to 300.3)	295.7 (244.2 to 344.6)	232.2 (189.4 to 272)	260.4 (218.9 to 297.4)	278.3 (230.1 to 325.8)	242.1 (199.3 to 281)	-1.5 (-5.8 to 3.2)	-5.9 (-11.5 to 0.6)	4.3 (-1.9 to 10.5)
Sub-Saharan Africa	Western sub-Saharan Africa	Cabo Verde	Incidence	All Ages	264 (251 to 278)	127 (119 to 135)	137 (130 to 145)	437 (414 to 459)	219 (205 to 234)	217 (206 to 229)	65.6 (59 to 72)	73.3 (65.1 to 82.4)	58.5 (50.4 to 66.8)
Sub-Saharan Africa	Western sub-Saharan Africa	Cabo Verde	Incidence	Age-standardized	103.4 (97.7 to 109.6)	86.4 (81 to 92.6)	128.6 (121.8 to 136.1)	91 (86.1 to 96.2)	84.6 (79 to 90.5)	99.5 (94.3 to 105.1)	-12 (-15 to -9.1)	-2.2 (-6.7 to 2.8)	-22.6 (-25.7 to -18.8)
Sub-Saharan Africa	Western sub-Saharan Africa	Cabo Verde	Prevalence	All Ages	4,110 (3,906 to 4,328)	2,072 (1,966 to 2,190)	2,038 (1,927 to 2,152)	7,318 (6,951 to 7,690)	3,668 (3,430 to 3,900)	3,650 (3,464 to 3,848)	78 (71.3 to 84.6)	77 (67.2 to 86.3)	79.1 (70.9 to 87.1)
Sub-Saharan Africa	Western sub-Saharan Africa	Cabo Verde	Prevalence	Age-standardized	1713.4 (1621.4 to 1808.8)	1507.1 (1427.1 to 1596.2)	2010 (1887.3 to 2133.8)	1514.2 (1436.9 to 1597.8)	1424.5 (1330.3 to 1518.1)	1614.1 (1525.4 to 1706.7)	-11.6 (-14.9 to -8.5)	-5.5 (-10.5 to -0.8)	-19.7 (-23.3 to -16.3)
Sub-Saharan Africa	Western sub-Saharan Africa	Cabo Verde	Deaths	All Ages	84 (56 to 98)	35 (21 to 45)	48 (32 to 58)	73 (58 to 100)	33 (21 to 48)	40 (34 to 55)	-13 (-31.8 to 71.4)	-7.3 (-37.4 to 84.3)	-17.2 (-36.4 to 68.8)
Sub-Saharan Africa	Western sub-Saharan Africa	Cabo Verde	Deaths	Age-standardized	34.8 (23.3 to 40.3)	25.4 (14.9 to 32.2)	48.4 (32.6 to 57.9)	17.9 (14.4 to 24.6)	13.2 (8.7 to 19.3)	25.2 (21.1 to 34.3)	-48.5 (-59.4 to 0.2)	-48.2 (-64.7 to 2.1)	-47.9 (-59.8 to 4.9)
Sub-Saharan Africa	Western sub-Saharan Africa	Cabo Verde	DALYs	All Ages	2,030 (1,510 to 2,279)	913 (652 to 1,077)	1,117 (796 to 1,314)	2,127 (1,848 to 2,566)	992 (819 to 1,220)	1,135 (982 to 1,420)	4.8 (-11.1 to 66.4)	8.7 (-13.6 to 64)	1.6 (-17.6 to 71.9)
Sub-Saharan Africa	Western sub-Saharan Africa	Cabo Verde	DALYs	Age-standardized	849.9 (634.9 to 954.5)	662.8 (471.4 to 779.5)	1115.4 (802.3 to 1306.5)	482.1 (416.7 to 585.9)	398.6 (328 to 496.9)	598.3 (517.9 to 758.5)	-43.3 (-52.1 to -9.7)	-39.9 (-52.1 to -9.3)	-46.4 (-56.6 to -8.2)
Sub-Saharan Africa	Western sub-Saharan Africa	Cabo Verde	YLLs	All Ages	1,521 (1,008 to 1,770)	628 (372 to 786)	893 (591 to 1,090)	1,227 (1,005 to 1,681)	493 (347 to 727)	733 (606 to 1,025)	-19.4 (-36.7 to 60.8)	-21.5 (-47 to 54.8)	-17.9 (-37.6 to 68.9)
Sub-Saharan Africa	Western sub-Saharan Africa	Cabo Verde	YLLs	Age-standardized	638.2 (425.6 to 738.9)	456.5 (272.6 to 568.9)	894.5 (592 to 1087.8)	296.1 (242.7 to 403.1)	205.3 (144.3 to 302.7)	421.7 (351.7 to 581.4)	-53.6 (-63.6 to -8.6)	-55 (-69.4 to -12)	-52.9 (-63.8 to -3.8)
Sub-Saharan Africa	Western sub-Saharan Africa	Cabo Verde	YLDs	All Ages	509 (418 to 578)	285 (232 to 328)	224 (182 to 261)	901 (746 to 1,015)	499 (408 to 577)	402 (324 to 467)	77 (66.7 to 87.8)	75.2 (60.9 to 89.4)	79.2 (65.8 to 94.9)
Sub-Saharan Africa	Western sub-Saharan Africa	Cabo Verde	YLDs	Age-standardized	211.7 (173.8 to 240.7)	206.3 (168.5 to 239.3)	220.9 (177.6 to 256.7)	186 (154.8 to 209.6)	193.3 (158.2 to 223.5)	176.6 (143.6 to 204.5)	-12.1 (-16.5 to -7.2)	-6.3 (-13 to 0.9)	-20.1 (-24.9 to -14.4)
Sub-Saharan Africa	Western sub-Saharan Africa	Chad	Incidence	All Ages	3,774 (3,563 to 3,997)	1,886 (1,771 to 2,008)	1,888 (1,782 to 1,996)	8,833 (8,382 to 9,287)	4,325 (4,082 to 4,581)	4,508 (4,286 to 4,769)	134 (126.9 to 141.6)	129.3 (119 to 139.8)	138.7 (130.3 to 147.5)
Sub-Saharan Africa	Western sub-Saharan Africa	Chad	Incidence	Age-standardized	109.1 (102.3 to 116.4)	100.8 (93.6 to 108.7)	119.8 (112.7 to 127.2)	108.8 (102.7 to 115.7)	105.7 (98.6 to 113.5)	112.3 (106 to 119.4)	-0.2 (-3.2 to 2.9)	4.8 (0.3 to 9.8)	-6.3 (-9.6 to -2.5)
Sub-Saharan Africa	Western sub-Saharan Africa	Chad	Prevalence	All Ages	54,923 (52,013 to 57,962)	28,948 (27,185 to 30,660)	25,975 (24,543 to 27,450)	128,055 (121,772 to 134,579)	63,851 (60,302 to 67,503)	64,204 (60,921 to 67,551)	133.2 (126.3 to 140)	120.6 (110.1 to 130)	147.2 (138.9 to 156.1)
Sub-Saharan Africa	Western sub-Saharan Africa	Chad	Prevalence	Age-standardized	1641.4 (1542.8 to 1748.6)	1645.5 (1534.9 to 1761.3)	1649.4 (1551.9 to 1751.1)	1680.4 (1579.6 to 1784.2)	1732.4 (1616 to 1853)	1645.7 (1548.4 to 1749.5)	2.4 (-0.5 to 5.3)	5.3 (0.6 to 9.6)	-0.2 (-3.7 to 3.6)
Sub-Saharan Africa	Western sub-Saharan Africa	Chad	Deaths	All Ages	909 (692 to 1,291)	346 (237 to 467)	563 (398 to 972)	1,405 (1,045 to 1,831)	470 (308 to 664)	935 (630 to 1,297)	54.6 (15.5 to 103.8)	35.8 (-2.3 to 83.5)	66.1 (9.2 to 140)
Sub-Saharan Africa	Western sub-Saharan Africa	Chad	Deaths	Age-standardized	36.2 (27.5 to 51.2)	26 (17.8 to 34.7)	48.4 (34.7 to 83.7)	31.1 (23.2 to 39.7)	21.7 (14.5 to 30.4)	40 (27.5 to 54.4)	-14.2 (-35.5 to 10.2)	-16.8 (-39.5 to 12.3)	-17.4 (-45 to 15.4)
Sub-Saharan Africa	Western sub-Saharan Africa	Chad	DALYs	All Ages	26,112 (21,082 to 34,577)	11,549 (8,747 to 14,687)	14,563 (11,054 to 23,967)	47,902 (39,157 to 58,698)	19,692 (14,966 to 25,428)	28,210 (21,095 to 37,243)	83.4 (43.5 to 126.9)	70.5 (38.1 to 109.5)	93.7 (33.9 to 161.4)
Sub-Saharan Africa	Western sub-Saharan Africa	Chad	DALYs	Age-standardized	865.7 (696.2 to 1167)	708.5 (543 to 893.2)	1041.3 (784.8 to 1709.7)	766.1 (616.5 to 947.4)	625.2 (473.2 to 806.9)	892.3 (659.9 to 1174.1)	-11.5 (-30.9 to 10.3)	-11.8 (-29.2 to 9.6)	-14.3 (-40.9 to 15.8)
Sub-Saharan Africa	Western sub-Saharan Africa	Chad	YLLs	All Ages	19,528 (14,740 to 27,655)	7,935 (5,295 to 10,940)	11,593 (8,070 to 20,921)	32,536 (24,055 to 43,439)	11,691 (7,242 to 17,365)	20,845 (13,885 to 29,771)	66.6 (20.9 to 122.5)	47.3 (4.2 to 102.3)	79.8 (15.4 to 165)
Sub-Saharan Africa	Western sub-Saharan Africa	Chad	YLLs	Age-standardized	671.2 (503.2 to 966.5)	505.9 (345.5 to 681.2)	854.4 (598.3 to 1525.4)	568.1 (417.3 to 741.1)	413 (271.1 to 590.5)	705.9 (476.3 to 985.2)	-15.4 (-37.7 to 12.4)	-18.4 (-41.1 to 11.1)	-17.4 (-46.7 to 19.1)
Sub-Saharan Africa	Western sub-Saharan Africa	Chad	YLDs	All Ages	6,584 (5,485 to 7,527)	3,614 (2,968 to 4,226)	2,970 (2,458 to 3,463)	15,366 (12,941 to 17,510)	8,001 (6,577 to 9,378)	7,366 (5,994 to 8,614)	133.4 (120.8 to 146.8)	121.4 (105.1 to 141.3)	148 (129.9 to 168.2)
Sub-Saharan Africa	Western sub-Saharan Africa	Chad	YLDs	Age-standardized	194.6 (162.6 to 222.1)	202.7 (166 to 235.1)	186.8 (154 to 217.1)	198 (166.5 to 225.2)	212.2 (174.5 to 246.8)	186.3 (153.5 to 216.1)	1.8 (-2.8 to 6.5)	4.7 (-1.6 to 11.9)	-0.3 (-6.1 to 6.2)
Sub-Saharan Africa	Western sub-Saharan Africa	Côte d'Ivoire	Incidence	All Ages	6,952 (6,581 to 7,334)	3,172 (3,006 to 3,360)	3,780 (3,572 to 3,988)	15,798 (14,946 to 16,738)	7,329 (6,923 to 7,779)	8,469 (7,993 to 8,980)	127.2 (121.4 to 132.8)	131 (122.7 to 139.2)	124 (116.8 to 131.7)
Sub-Saharan Africa	Western sub-Saharan Africa	Côte d'Ivoire	Incidence	Age-standardized	114.5 (107.5 to 121)	102 (95.4 to 109)	127.8 (120.4 to 135)	105.2 (99.2 to 111.7)	97 (90.9 to 103.6)	115.1 (108.5 to 122.1)	-8.1 (-10.4 to -5.7)	-4.8 (-8.6 to -1.2)	-9.9 (-13 to -6.7)
Sub-Saharan Africa	Western sub-Saharan Africa	Côte d'Ivoire	Prevalence	All Ages	103,099 (97,999 to 107,932)	48,158 (45,719 to 50,678)	54,941 (52,168 to 57,768)	248,534 (236,502 to 261,568)	118,421 (111,951 to 125,258)	130,114 (123,811 to 136,821)	141.1 (133.7 to 147.3)	145.9 (135.4 to 154.6)	136.8 (129.3 to 145.1)
Sub-Saharan Africa	Western sub-Saharan Africa	Côte d'Ivoire	Prevalence	Age-standardized	1777.7 (1670.5 to 1887.5)	1725.4 (1613.7 to 1841.6)	1836.7 (1724.5 to 1944.9)	1697.7 (1601.1 to 1805)	1679.9 (1576 to 1795)	1728 (1626.1 to 1836.4)	-4.5 (-7.2 to -2)	-2.6 (-6.5 to 0.9)	-5.9 (-9.2 to -2.5)
Sub-Saharan Africa	Western sub-Saharan Africa	Côte d'Ivoire	Deaths	All Ages	1,062 (804 to 1,328)	300 (204 to 394)	761 (570 to 973)	1,950 (1,472 to 2,498)	613 (419 to 844)	1,337 (959 to 1,734)	83.7 (30.7 to 152.4)	104.1 (47.4 to 176.9)	75.6 (15.9 to 152)
Sub-Saharan Africa	Western sub-Saharan Africa	Côte d'Ivoire	Deaths	Age-standardized	37.6 (29.3 to 45.6)	21.9 (15.4 to 28.5)	53.7 (40.8 to 67)	25 (19.5 to 31.2)	15.7 (11 to 21)	35.7 (26.4 to 45)	-33.3 (-50.5 to -12.4)	-28.4 (-47.4 to -4.8)	-33.5 (-53.6 to -9.4)
Sub-Saharan Africa	Western sub-Saharan Africa	Côte d'Ivoire	DALYs	All Ages	38,907 (31,503 to 47,028)	13,857 (10,736 to 16,816)	25,050 (19,642 to 31,224)	75,749 (62,556 to 91,224)	29,224 (23,578 to 35,872)	46,526 (36,996 to 57,416)	94.7 (53.3 to 143.5)	110.9 (70.7 to 150.2)	85.7 (33.1 to 145.7)
Sub-Saharan Africa	Western sub-Saharan Africa	Côte d'Ivoire	DALYs	Age-standardized	891.8 (721.8 to 1059.1)	615.6 (473.6 to 749.8)	1149.1 (903.8 to 1398.3)	647.1 (536.9 to 778.8)	489.9 (389.4 to 602.3)	807.5 (638.3 to 987.9)	-27.4 (-42.6 to -8.8)	-20.4 (-35.3 to -3.7)	-29.7 (-48.3 to -7.9)
Sub-Saharan Africa	Western sub-Saharan Africa	Côte d'Ivoire	YLLs	All Ages	26,653 (19,655 to 34,391)	7,852 (5,004 to 10,710)	18,802 (13,639 to 24,697)	45,903 (33,350 to 60,748)	14,324 (9,308 to 20,272)	31,579 (21,868 to 42,690)	72.2 (16.3 to 144.4)	82.4 (26 to 155.2)	68 (5.4 to 151.3)
Sub-Saharan Africa	Western sub-Saharan Africa	Côte d'Ivoire	YLLs	Age-standardized	685 (521 to 853.2)	406 (279.1 to 533.8)	943.8 (709.5 to 1197.7)	446.6 (335.5 to 573.9)	282.7 (191.8 to 392.7)	611.9 (440.1 to 793.4)	-34.8 (-53.7 to -11)	-30.4 (-49.9 to -4.8)	-35.2 (-56.6 to -8.3)
Sub-Saharan Africa	Western sub-Saharan Africa	Côte d'Ivoire	YLDs	All Ages	12,253 (10,261 to 13,980)	6,006 (4,904 to 7,052)	6,248 (5,113 to 7,298)	29,846 (24,830 to 34,166)	14,900 (12,213 to 17,388)	14,947 (12,238 to 17,366)	143.6 (129.1 to 158.4)	148.1 (127.7 to 169.5)	139.2 (120.9 to 158.8)
Sub-Saharan Africa	Western sub-Saharan Africa	Côte d'Ivoire	YLDs	Age-standardized	206.8 (174.2 to 236.7)	209.6 (171.3 to 245)	205.3 (170.3 to 238.3)	200.5 (168.1 to 228.3)	207.2 (171.4 to 241.3)	195.6 (161.7 to 226.2)	-3 (-7.4 to 1.7)	-1.1 (-7.4 to 5.3)	-4.7 (-10.6 to 1.1)
Sub-Saharan Africa	Western sub-Saharan Africa	Gambia	Incidence	All Ages	609 (579 to 642)	293 (279 to 308)	316 (299 to 335)	1,599 (1,520 to 1,686)	836 (791 to 884)	763 (723 to 805)	162.6 (156.1 to 169.5)	185.5 (175.5 to 195.6)	141.4 (133.8 to 150.3)
Sub-Saharan Africa	Western sub-Saharan Africa	Gambia	Incidence	Age-standardized	121.5 (114.8 to 128.8)	117.2 (110.2 to 124.8)	130.4 (123 to 138.8)	125.2 (118.2 to 132.9)	125.2 (117.3 to 133.5)	127.8 (120.8 to 135.3)	3 (0 to 6)	6.8 (2.7 to 11)	-2 (-5.5 to 1.8)
Sub-Saharan Africa	Western sub-Saharan Africa	Gambia	Prevalence	All Ages	8,944 (8,499 to 9,411)	4,407 (4,188 to 4,641)	4,537 (4,294 to 4,795)	24,182 (22,965 to 25,460)	12,999 (12,279 to 13,740)	11,183 (10,596 to 11,802)	170.4 (163.9 to 177.5)	195 (184.8 to 205.7)	146.5 (138.4 to 155.4)
Sub-Saharan Africa	Western sub-Saharan Africa	Gambia	Prevalence	Age-standardized	1899.5 (1785.4 to 2019.8)	1945.8 (1821.8 to 2078.8)	1882.7 (1765.2 to 2009.5)	2004.4 (1882.6 to 2132.9)	2118.6 (1976.4 to 2266.3)	1901.7 (1785.6 to 2022.3)	5.5 (2.7 to 8.7)	8.9 (4.8 to 13.3)	1 (-2.6 to 5.3)
Sub-Saharan Africa	Western sub-Saharan Africa	Gambia	Deaths	All Ages	95 (70 to 128)	33 (22 to 46)	62 (43 to 88)	244 (185 to 311)	95 (63 to 140)	149 (108 to 196)	156.7 (79.2 to 259)	184.9 (94.9 to 314)	141.6 (47.3 to 252.5)
Sub-Saharan Africa	Western sub-Saharan Africa	Gambia	Deaths	Age-standardized	34.3 (25.7 to 44.4)	23.4 (15.8 to 32.4)	48.8 (35.5 to 67.4)	30.4 (23.3 to 38.5)	21.8 (14.9 to 31.6)	41.7 (31 to 53.6)	-11.4 (-35.9 to 18.8)	-6.9 (-35.6 to 34.4)	-14.5 (-45.5 to 19.7)
Sub-Saharan Africa	Western sub-Saharan Africa	Gambia	DALYs	All Ages	3,253 (2,562 to 4,168)	1,314 (1,023 to 1,675)	1,939 (1,480 to 2,642)	8,039 (6,571 to 9,807)	3,546 (2,779 to 4,604)	4,493 (3,494 to 5,631)	147.2 (88 to 218.4)	169.9 (114.7 to 244.3)	131.7 (54.1 to 215.2)
Sub-Saharan Africa	Western sub-Saharan Africa	Gambia	DALYs	Age-standardized	844.5 (670.3 to 1079.5)	665.4 (511.3 to 846.9)	1043.2 (800.9 to 1411.6)	779.1 (637.9 to 943.6)	649.4 (500.6 to 855.9)	933.9 (727.4 to 1164.8)	-7.7 (-30.4 to 18.3)	-2.4 (-24.5 to 26)	-10.5 (-39.7 to 20.4)
Sub-Saharan Africa	Western sub-Saharan Africa	Gambia	YLLs	All Ages	2,178 (1,529 to 3,039)	763 (488 to 1,120)	1,415 (962 to 2,136)	5,138 (3,777 to 6,655)	1,934 (1,233 to 2,930)	3,204 (2,265 to 4,346)	135.9 (58.5 to 247.1)	153.5 (64.6 to 288.3)	126.4 (30 to 245.2)
Sub-Saharan Africa	Western sub-Saharan Africa	Gambia	YLLs	Age-standardized	619.4 (455.8 to 843.9)	426.6 (280.3 to 598.3)	828.2 (586.7 to 1193.2)	541.6 (405 to 697.1)	390.7 (257.4 to 583.7)	717.2 (515.2 to 942.4)	-12.5 (-39.6 to 23.5)	-8.4 (-38.7 to 37.1)	-13.4 (-47.1 to 26.2)
Sub-Saharan Africa	Western sub-Saharan Africa	Gambia	YLDs	All Ages	1,075 (895 to 1,226)	551 (450 to 642)	524 (428 to 612)	2,901 (2,405 to 3,312)	1,612 (1,328 to 1,868)	1,289 (1,043 to 1,501)	170 (155.8 to 185.6)	192.8 (170.9 to 217.8)	146 (127.9 to 164.7)
Sub-Saharan Africa	Western sub-Saharan Africa	Gambia	YLDs	Age-standardized	225.2 (187.8 to 258)	238.8 (196.8 to 279.1)	215 (176.8 to 250.2)	237.5 (196.9 to 271.2)	258.8 (212.7 to 300.7)	216.8 (176.9 to 251.2)	5.5 (1 to 10.3)	8.4 (2.4 to 15.5)	0.8 (-4.6 to 7.2)
Sub-Saharan Africa	Western sub-Saharan Africa	Ghana	Incidence	All Ages	7,343 (7,010 to 7,712)	4,001 (3,800 to 4,223)	3,342 (3,183 to 3,522)	21,369 (20,255 to 22,503)	10,276 (9,683 to 10,867)	11,092 (10,470 to 11,716)	191 (182.3 to 199.6)	156.8 (147.7 to 167.3)	231.9 (217.3 to 247.1)
Sub-Saharan Africa	Western sub-Saharan Africa	Ghana	Incidence	Age-standardized	82.3 (78.1 to 87)	84.3 (79.2 to 89.6)	82.2 (77.7 to 87.3)	100.6 (95.2 to 106.4)	86.5 (81.1 to 91.9)	120.8 (114.2 to 128.2)	22.2 (18.8 to 25.7)	2.6 (-1.2 to 6.9)	47 (41.1 to 52.8)
Sub-Saharan Africa	Western sub-Saharan Africa	Ghana	Prevalence	All Ages	120,121 (114,921 to 125,622)	66,303 (63,181 to 69,752)	53,818 (50,962 to 56,560)	353,063 (335,031 to 371,410)	180,233 (170,755 to 190,019)	172,829 (163,285 to 181,879)	193.9 (185.9 to 202.1)	171.8 (161.1 to 182.5)	221.1 (206.2 to 238.8)
Sub-Saharan Africa	Western sub-Saharan Africa	Ghana	Prevalence	Age-standardized	1409.3 (1341.4 to 1485.3)	1521.8 (1436.5 to 1613.7)	1295.1 (1226.6 to 1369.7)	1734.3 (1637.8 to 1833.3)	1607.1 (1509.4 to 1703.6)	1913.9 (1799.4 to 2025.3)	23.1 (19.4 to 26.6)	5.6 (1.5 to 10)	47.8 (40.8 to 54.9)
Sub-Saharan Africa	Western sub-Saharan Africa	Ghana	Deaths	All Ages	1,378 (880 to 1,755)	434 (287 to 569)	945 (517 to 1,258)	3,286 (1,973 to 4,138)	822 (555 to 1,063)	2,464 (1,285 to 3,133)	138.4 (57.7 to 220.1)	89.6 (33.8 to 174.4)	160.8 (51.8 to 274.2)
Sub-Saharan Africa	Western sub-Saharan Africa	Ghana	Deaths	Age-standardized	28.9 (18.4 to 36)	16 (10.9 to 20.8)	45.5 (25 to 58.9)	25.6 (15.6 to 31.8)	11.2 (7.5 to 14.3)	47.8 (24.3 to 60)	-11.3 (-40.8 to 17.2)	-30.2 (-50.7 to 0)	5.3 (-37.1 to 45.5)
Sub-Saharan Africa	Western sub-Saharan Africa	Ghana	DALYs	All Ages	46,511 (33,864 to 55,553)	18,516 (14,057 to 22,505)	27,995 (18,297 to 35,418)	115,490 (83,960 to 136,317)	38,234 (31,031 to 44,740)	77,256 (49,476 to 94,058)	148.3 (87.8 to 204.3)	106.5 (66.5 to 154.7)	176 (81.7 to 262.6)
Sub-Saharan Africa	Western sub-Saharan Africa	Ghana	DALYs	Age-standardized	687.2 (487.1 to 831.6)	480.3 (368 to 582.8)	932.9 (585.7 to 1185.2)	670 (473.3 to 793)	381.2 (309.4 to 447.8)	1065 (650.6 to 1290.3)	-2.5 (-27.8 to 21.2)	-20.6 (-36.2 to -0.7)	14.2 (-26.5 to 52.1)
Sub-Saharan Africa	Western sub-Saharan Africa	Ghana	YLLs	All Ages	32,565 (20,373 to 41,197)	11,057 (6,813 to 14,842)	21,508 (11,840 to 28,730)	74,523 (44,339 to 94,611)	17,987 (11,847 to 23,665)	56,536 (29,537 to 73,180)	128.8 (47.1 to 212.8)	62.7 (11.4 to 137.4)	162.9 (48.7 to 279.4)
Sub-Saharan Africa	Western sub-Saharan Africa	Ghana	YLLs	Age-standardized	525.7 (335.8 to 670.9)	311.5 (205.2 to 410.1)	778.4 (426.7 to 1032.6)	471.3 (281.4 to 592.3)	202.9 (135.5 to 262.4)	838.7 (438.1 to 1067.2)	-10.4 (-41.2 to 21.4)	-34.9 (-54.5 to -5.2)	7.7 (-37.4 to 54.5)
Sub-Saharan Africa	Western sub-Saharan Africa	Ghana	YLDs	All Ages	13,946 (11,602 to 15,799)	7,459 (6,075 to 8,653)	6,487 (5,322 to 7,557)	40,968 (33,983 to 46,466)	20,248 (16,679 to 23,504)	20,720 (16,989 to 24,063)	193.8 (177.1 to 213.1)	171.5 (148.5 to 196)	219.4 (193.2 to 249.1)
Sub-Saharan Africa	Western sub-Saharan Africa	Ghana	YLDs	Age-standardized	161.5 (134 to 181.7)	168.7 (138.1 to 193.9)	154.5 (127 to 177.7)	198.7 (164.8 to 224.3)	178.3 (147 to 206.3)	226.3 (185.7 to 260.2)	23.1 (17.4 to 29.6)	5.7 (-1.5 to 13.1)	46.5 (36.8 to 57.1)
Sub-Saharan Africa	Western sub-Saharan Africa	Guinea	Incidence	All Ages	4,448 (4,200 to 4,703)	2,296 (2,162 to 2,438)	2,152 (2,030 to 2,275)	9,030 (8,572 to 9,511)	4,506 (4,248 to 4,769)	4,525 (4,297 to 4,761)	103 (97.4 to 109.4)	96.3 (88.6 to 104.5)	110.3 (102.8 to 117.9)
Sub-Saharan Africa	Western sub-Saharan Africa	Guinea	Incidence	Age-standardized	115.9 (108.9 to 123.1)	113.3 (106.1 to 121)	118.7 (111.7 to 126)	126.8 (119.9 to 134.4)	119.8 (112.2 to 127.9)	135.2 (127.6 to 143)	9.5 (6.4 to 12.6)	5.7 (1.5 to 9.8)	13.9 (9.7 to 18.2)
Sub-Saharan Africa	Western sub-Saharan Africa	Guinea	Prevalence	All Ages	65,397 (61,747 to 69,051)	35,485 (33,436 to 37,702)	29,912 (28,197 to 31,564)	134,216 (126,884 to 141,604)	68,885 (64,887 to 72,869)	65,331 (61,877 to 69,062)	105.2 (99.9 to 111.6)	94.1 (86.8 to 102.1)	118.4 (110.6 to 127.4)
Sub-Saharan Africa	Western sub-Saharan Africa	Guinea	Prevalence	Age-standardized	1731.5 (1624.2 to 1834.9)	1835.8 (1716.4 to 1960.9)	1632.7 (1535.7 to 1725.9)	1979.4 (1858.6 to 2108.4)	1980.5 (1843.9 to 2119.8)	1989.4 (1865 to 2116.9)	14.3 (11 to 18.1)	7.9 (3.6 to 12.6)	21.8 (17 to 27.1)
Sub-Saharan Africa	Western sub-Saharan Africa	Guinea	Deaths	All Ages	1,049 (815 to 1,350)	439 (295 to 602)	610 (439 to 951)	1,478 (1,123 to 1,863)	560 (376 to 793)	918 (644 to 1,200)	40.9 (3.1 to 89.9)	27.5 (-8 to 81.7)	50.5 (-6.3 to 114.1)
Sub-Saharan Africa	Western sub-Saharan Africa	Guinea	Deaths	Age-standardized	36.7 (28.5 to 47.3)	29.2 (19.9 to 39.6)	44.7 (32.4 to 68.9)	31.1 (24 to 38.9)	22.9 (15.6 to 32)	40.4 (28.5 to 52.3)	-15.1 (-37.5 to 13.1)	-21.5 (-42.7 to 13)	-9.6 (-42 to 28.1)
Sub-Saharan Africa	Western sub-Saharan Africa	Guinea	DALYs	All Ages	30,595 (25,173 to 37,957)	14,848 (11,045 to 19,120)	15,747 (12,001 to 23,689)	48,426 (39,829 to 58,650)	21,474 (16,597 to 28,372)	26,952 (20,744 to 33,586)	58.3 (24.8 to 97.8)	44.6 (15.5 to 86.4)	71.2 (12.8 to 129.1)
Sub-Saharan Africa	Western sub-Saharan Africa	Guinea	DALYs	Age-standardized	879.7 (723.8 to 1096.8)	804.5 (604.3 to 1019.4)	957.7 (728.8 to 1428.2)	810.1 (660.5 to 977.1)	684.3 (534.4 to 879.6)	941.5 (721.9 to 1177.1)	-7.9 (-27.7 to 16.2)	-14.9 (-32.2 to 9.3)	-1.7 (-35.5 to 32)
Sub-Saharan Africa	Western sub-Saharan Africa	Guinea	YLLs	All Ages	22,700 (17,480 to 29,874)	10,422 (6,684 to 14,756)	12,279 (8,717 to 20,031)	32,228 (24,009 to 41,774)	12,824 (8,245 to 19,371)	19,404 (13,427 to 26,193)	42 (0.7 to 93.7)	23 (-14.7 to 80.9)	58 (-6 to 134.5)
Sub-Saharan Africa	Western sub-Saharan Africa	Guinea	YLLs	Age-standardized	672.2 (517.3 to 884.4)	577.6 (384.2 to 795.6)	769.5 (546.8 to 1238.7)	573.8 (432.3 to 732.9)	439.2 (292.6 to 630.8)	713.4 (497.8 to 943.7)	-14.6 (-39.3 to 16.5)	-24 (-45.8 to 9.7)	-7.3 (-44.3 to 35.8)
Sub-Saharan Africa	Western sub-Saharan Africa	Guinea	YLDs	All Ages	7,894 (6,636 to 9,001)	4,426 (3,641 to 5,137)	3,468 (2,868 to 4,049)	16,198 (13,509 to 18,503)	8,650 (7,092 to 10,099)	7,548 (6,166 to 8,852)	105.2 (94.7 to 116.4)	95.5 (82.4 to 110.6)	117.6 (101.1 to 135.3)
Sub-Saharan Africa	Western sub-Saharan Africa	Guinea	YLDs	Age-standardized	207.5 (174.3 to 236.4)	226.9 (187.9 to 263.9)	188.2 (155.4 to 218.7)	236.3 (197.1 to 271.2)	245.1 (201.8 to 287)	228.1 (186.6 to 267.5)	13.9 (8.7 to 19.2)	8 (1.6 to 15.6)	21.2 (13.3 to 29.7)
Sub-Saharan Africa	Western sub-Saharan Africa	Guinea-Bissau	Incidence	All Ages	716 (676 to 757)	339 (320 to 359)	377 (354 to 399)	1,311 (1,241 to 1,385)	671 (634 to 711)	640 (604 to 678)	83.2 (78 to 88.7)	97.8 (90.4 to 105.1)	70.1 (63.7 to 77.2)
Sub-Saharan Africa	Western sub-Saharan Africa	Guinea-Bissau	Incidence	Age-standardized	136.3 (128 to 144.9)	116.3 (108.8 to 124.5)	161.5 (152.1 to 171.5)	126.9 (119.5 to 134.6)	118.9 (111.5 to 126.7)	140.1 (132.3 to 148.4)	-6.9 (-9.3 to -4.2)	2.2 (-1.9 to 6.2)	-13.2 (-16.1 to -10.1)
Sub-Saharan Africa	Western sub-Saharan Africa	Guinea-Bissau	Prevalence	All Ages	10,332 (9,742 to 10,929)	5,123 (4,827 to 5,418)	5,209 (4,897 to 5,539)	19,764 (18,753 to 20,857)	10,394 (9,844 to 11,000)	9,369 (8,848 to 9,916)	91.3 (86.1 to 96.9)	102.9 (94.7 to 111.3)	79.9 (72.7 to 87.1)
Sub-Saharan Africa	Western sub-Saharan Africa	Guinea-Bissau	Prevalence	Age-standardized	2041.2 (1901.9 to 2182.6)	1891 (1759.4 to 2020.3)	2222.9 (2072.1 to 2385.6)	2031.8 (1908.1 to 2164.6)	1991.8 (1865 to 2131.6)	2092.1 (1956.4 to 2233.7)	-0.5 (-3.6 to 2.8)	5.3 (0.7 to 9.9)	-5.9 (-9.7 to -1.8)
Sub-Saharan Africa	Western sub-Saharan Africa	Guinea-Bissau	Deaths	All Ages	170 (130 to 216)	52 (33 to 72)	119 (84 to 157)	197 (144 to 256)	74 (49 to 105)	123 (81 to 165)	15.5 (-17.5 to 54.7)	42.6 (-4.1 to 124.2)	3.7 (-31.8 to 50.9)
Sub-Saharan Africa	Western sub-Saharan Africa	Guinea-Bissau	Deaths	Age-standardized	49.8 (38.2 to 62.3)	28.9 (19.2 to 39.8)	74.4 (54.5 to 96.2)	34.5 (25.8 to 44.3)	23.1 (15.6 to 32.4)	51.3 (35 to 67.1)	-30.8 (-49.3 to -8.6)	-20 (-45.7 to 23.2)	-31 (-52.9 to -2.9)
Sub-Saharan Africa	Western sub-Saharan Africa	Guinea-Bissau	DALYs	All Ages	5,465 (4,418 to 6,665)	2,007 (1,452 to 2,607)	3,458 (2,626 to 4,491)	7,369 (5,932 to 9,016)	3,117 (2,450 to 3,929)	4,252 (3,065 to 5,494)	34.8 (3.8 to 69.8)	55.3 (18.2 to 105.8)	23 (-12.9 to 67.7)
Sub-Saharan Africa	Western sub-Saharan Africa	Guinea-Bissau	DALYs	Age-standardized	1243.1 (999 to 1512.8)	823.8 (607.7 to 1063.2)	1702.8 (1295.7 to 2176.2)	911.2 (727.6 to 1115.3)	690.3 (537 to 881)	1201 (868.7 to 1527.3)	-26.7 (-43.3 to -7.8)	-16.2 (-36.7 to 14.2)	-29.5 (-49.7 to -4.3)
Sub-Saharan Africa	Western sub-Saharan Africa	Guinea-Bissau	YLLs	All Ages	4,232 (3,217 to 5,420)	1,368 (819 to 1,968)	2,864 (2,054 to 3,911)	4,991 (3,537 to 6,617)	1,817 (1,171 to 2,610)	3,174 (1,988 to 4,470)	17.9 (-18 to 62.3)	32.8 (-12.4 to 109.3)	10.8 (-30.3 to 64.4)
Sub-Saharan Africa	Western sub-Saharan Africa	Guinea-Bissau	YLLs	Age-standardized	1003 (758.2 to 1269.4)	591.7 (377.4 to 823)	1452.4 (1042.3 to 1923.9)	670.6 (490.7 to 872.3)	445.8 (296.7 to 633.1)	963.8 (637.4 to 1294.8)	-33.1 (-52.7 to -10.3)	-24.7 (-49.3 to 18.5)	-33.6 (-56.2 to -3.8)
Sub-Saharan Africa	Western sub-Saharan Africa	Guinea-Bissau	YLDs	All Ages	1,233 (1,025 to 1,404)	640 (520 to 748)	594 (486 to 695)	2,378 (1,972 to 2,725)	1,301 (1,073 to 1,520)	1,078 (863 to 1,267)	92.8 (82.6 to 104)	103.4 (87.8 to 120.5)	81.5 (68.1 to 95.5)
Sub-Saharan Africa	Western sub-Saharan Africa	Guinea-Bissau	YLDs	Age-standardized	240.1 (199.8 to 274)	232.1 (188.8 to 270.4)	250.4 (204.1 to 290.2)	240.6 (200.4 to 274.8)	244.5 (202.6 to 284.2)	237.2 (191.7 to 277)	0.2 (-4.1 to 5.1)	5.3 (-1.7 to 12.6)	-5.3 (-10.5 to 0.7)
Sub-Saharan Africa	Western sub-Saharan Africa	Liberia	Incidence	All Ages	1,028 (973 to 1,088)	545 (515 to 577)	483 (453 to 515)	2,679 (2,528 to 2,832)	1,492 (1,403 to 1,589)	1,188 (1,110 to 1,267)	160.6 (150.6 to 170.8)	173.7 (161.1 to 186)	145.8 (134 to 158.5)
Sub-Saharan Africa	Western sub-Saharan Africa	Liberia	Incidence	Age-standardized	78.8 (74.4 to 83.6)	82.7 (77.6 to 88.3)	75.2 (70.7 to 79.9)	91.4 (86 to 97.1)	101.3 (94.8 to 108.4)	82.8 (77.7 to 88.4)	16 (12.7 to 19.6)	22.4 (17.3 to 27.4)	10.1 (6 to 14.6)
Sub-Saharan Africa	Western sub-Saharan Africa	Liberia	Prevalence	All Ages	16,399 (15,484 to 17,293)	9,002 (8,542 to 9,476)	7,397 (6,819 to 7,958)	42,788 (40,388 to 45,276)	23,706 (22,404 to 25,090)	19,082 (17,587 to 20,571)	160.9 (153.1 to 169.3)	163.3 (152.7 to 174.5)	158 (146.2 to 170.1)
Sub-Saharan Africa	Western sub-Saharan Africa	Liberia	Prevalence	Age-standardized	1283.1 (1210.1 to 1355.5)	1448.4 (1363.5 to 1538.6)	1137.7 (1054 to 1219)	1520.4 (1430.6 to 1609.4)	1749.5 (1639.2 to 1863.9)	1306 (1212.3 to 1403.9)	18.5 (15.2 to 22.3)	20.8 (15.9 to 25.8)	14.8 (10.2 to 20.2)
Sub-Saharan Africa	Western sub-Saharan Africa	Liberia	Deaths	All Ages	207 (167 to 260)	88 (62 to 125)	119 (92 to 152)	292 (213 to 405)	132 (89 to 195)	160 (103 to 224)	41.2 (4.5 to 88.7)	49.8 (4 to 118.3)	34.8 (-11 to 88.2)
Sub-Saharan Africa	Western sub-Saharan Africa	Liberia	Deaths	Age-standardized	21.6 (17.4 to 27.2)	18.6 (13.4 to 27.7)	24.5 (19.1 to 30.6)	18.5 (13.8 to 25.3)	16.4 (11.1 to 24)	20.7 (13.8 to 28.4)	-14.3 (-34.5 to 12.9)	-12.2 (-37.9 to 25.8)	-15.4 (-42.5 to 14.2)
Sub-Saharan Africa	Western sub-Saharan Africa	Liberia	DALYs	All Ages	6,735 (5,564 to 8,267)	3,465 (2,584 to 4,765)	3,269 (2,677 to 4,023)	11,591 (9,538 to 14,466)	5,985 (4,778 to 7,719)	5,605 (4,210 to 7,309)	72.1 (36.4 to 110)	72.7 (26.8 to 131.4)	71.5 (28.9 to 119.6)
Sub-Saharan Africa	Western sub-Saharan Africa	Liberia	DALYs	Age-standardized	553.2 (466.5 to 658)	555.4 (437.5 to 721.5)	549.8 (451.3 to 670)	503.5 (408.8 to 634.7)	513.5 (404.8 to 666.8)	495.7 (365.2 to 643.5)	-9 (-25.4 to 11.7)	-7.5 (-26.8 to 19.4)	-9.8 (-32.7 to 16.1)
Sub-Saharan Africa	Western sub-Saharan Africa	Liberia	YLLs	All Ages	4,786 (3,687 to 6,250)	2,359 (1,523 to 3,610)	2,426 (1,854 to 3,171)	6,481 (4,562 to 9,216)	3,051 (1,979 to 4,657)	3,430 (2,120 to 5,105)	35.4 (-7.6 to 87.2)	29.3 (-19.8 to 105.1)	41.4 (-12 to 107.4)
Sub-Saharan Africa	Western sub-Saharan Africa	Liberia	YLLs	Age-standardized	402 (320.1 to 505.6)	379.5 (263.9 to 542.5)	420.9 (325.1 to 541.7)	324.6 (235.9 to 453.3)	301.2 (199.4 to 446.5)	348.3 (220.2 to 495.4)	-19.3 (-41.6 to 9.1)	-20.6 (-45.3 to 17.3)	-17.3 (-47.3 to 17.1)
Sub-Saharan Africa	Western sub-Saharan Africa	Liberia	YLDs	All Ages	1,949 (1,630 to 2,213)	1,106 (912 to 1,281)	843 (692 to 992)	5,110 (4,267 to 5,829)	2,935 (2,408 to 3,435)	2,175 (1,760 to 2,559)	162.2 (146.2 to 179.4)	165.3 (144 to 187.5)	158.1 (136.4 to 182.8)
Sub-Saharan Africa	Western sub-Saharan Africa	Liberia	YLDs	Age-standardized	151.2 (126.7 to 171.1)	175.9 (145 to 205.2)	128.9 (106.3 to 150.5)	178.9 (150.1 to 203.3)	212.3 (174.9 to 246.6)	147.4 (121 to 170.9)	18.3 (12.6 to 24.7)	20.7 (12.9 to 29.1)	14.4 (6.1 to 23.1)
Sub-Saharan Africa	Western sub-Saharan Africa	Mali	Incidence	All Ages	6,885 (6,472 to 7,336)	3,538 (3,317 to 3,780)	3,347 (3,139 to 3,569)	16,744 (15,793 to 17,713)	8,492 (7,962 to 9,035)	8,252 (7,794 to 8,739)	143.2 (136.6 to 149.7)	140 (131.2 to 148.6)	146.5 (137.7 to 155.2)
Sub-Saharan Africa	Western sub-Saharan Africa	Mali	Incidence	Age-standardized	134.2 (126.1 to 143.2)	132.4 (123.8 to 142.2)	137.6 (129.1 to 146.8)	144 (135.1 to 153.9)	145.9 (135.1 to 157.7)	142.3 (134.3 to 151.9)	7.3 (4.4 to 10.3)	10.2 (5.9 to 14.3)	3.4 (-0.2 to 7)
Sub-Saharan Africa	Western sub-Saharan Africa	Mali	Prevalence	All Ages	103,367 (96,538 to 110,605)	54,290 (50,596 to 57,982)	49,077 (45,777 to 52,565)	247,544 (232,568 to 261,553)	127,037 (119,248 to 134,593)	120,507 (113,172 to 127,749)	139.5 (132.6 to 146.3)	134 (124.2 to 143.3)	145.5 (135 to 155.4)
Sub-Saharan Africa	Western sub-Saharan Africa	Mali	Prevalence	Age-standardized	2121.2 (1974.8 to 2275.5)	2193.8 (2032.5 to 2359.5)	2053.6 (1909.8 to 2205.7)	2326.9 (2175.6 to 2479.6)	2439.1 (2263.3 to 2614.8)	2223.9 (2076.6 to 2376.1)	9.7 (6.3 to 13.1)	11.2 (6 to 16.1)	8.3 (3.6 to 12.8)
Sub-Saharan Africa	Western sub-Saharan Africa	Mali	Deaths	All Ages	1,355 (1,026 to 1,683)	540 (338 to 724)	815 (573 to 1,134)	2,476 (1,849 to 3,175)	916 (496 to 1,340)	1,560 (1,156 to 2,076)	82.8 (39.6 to 140.2)	69.7 (21.1 to 156.3)	91.4 (33.9 to 167.8)
Sub-Saharan Africa	Western sub-Saharan Africa	Mali	Deaths	Age-standardized	37.8 (29.5 to 46.2)	29.1 (18.8 to 38.8)	47.8 (34.5 to 63.8)	33.6 (26 to 41.9)	25.2 (14.1 to 35.6)	41.5 (31.6 to 54.7)	-11.1 (-31.2 to 14.6)	-13.2 (-37.6 to 30.3)	-13.1 (-37.7 to 17.4)
Sub-Saharan Africa	Western sub-Saharan Africa	Mali	DALYs	All Ages	45,556 (35,933 to 55,029)	20,272 (14,822 to 25,317)	25,284 (18,698 to 34,686)	88,731 (70,285 to 109,098)	38,266 (26,536 to 50,534)	50,465 (39,045 to 65,290)	94.8 (59.9 to 139.4)	88.8 (51.6 to 151.1)	99.6 (49.4 to 157.8)
Sub-Saharan Africa	Western sub-Saharan Africa	Mali	DALYs	Age-standardized	1030.9 (820.3 to 1234.8)	884.6 (630.3 to 1107.8)	1185.6 (887.2 to 1578.2)	942.2 (750 to 1149.8)	810.6 (553.4 to 1056.8)	1065.9 (834.2 to 1363.9)	-8.6 (-25.3 to 12.6)	-8.4 (-26.4 to 21)	-10.1 (-32.6 to 16)
Sub-Saharan Africa	Western sub-Saharan Africa	Mali	YLLs	All Ages	33,240 (24,213 to 42,846)	13,556 (8,295 to 18,511)	19,684 (13,052 to 28,989)	59,059 (41,107 to 79,482)	22,439 (11,741 to 34,619)	36,620 (25,758 to 50,846)	77.7 (33.7 to 139.1)	65.5 (13.3 to 160.4)	86 (28.4 to 162.1)
Sub-Saharan Africa	Western sub-Saharan Africa	Mali	YLLs	Age-standardized	780.9 (584.3 to 983.4)	616.8 (381.7 to 840.3)	953.4 (665.2 to 1346.4)	667.1 (482.5 to 866.4)	512 (272.5 to 757.8)	812.8 (592.9 to 1093.3)	-14.6 (-34.7 to 12)	-17 (-41.3 to 27.5)	-14.7 (-40.2 to 18.1)
Sub-Saharan Africa	Western sub-Saharan Africa	Mali	YLDs	All Ages	12,316 (10,190 to 14,062)	6,716 (5,554 to 7,832)	5,600 (4,552 to 6,571)	29,672 (24,863 to 33,898)	15,827 (13,091 to 18,517)	13,845 (11,253 to 16,262)	140.9 (129.7 to 153.1)	135.7 (121.2 to 152.4)	147.2 (130.8 to 165.3)
Sub-Saharan Africa	Western sub-Saharan Africa	Mali	YLDs	Age-standardized	250 (207.4 to 285.6)	267.8 (221.7 to 312.5)	232.2 (188.2 to 271.6)	275.1 (231.9 to 316.6)	298.5 (245.7 to 351.2)	253 (207 to 296.3)	10 (5.2 to 14.8)	11.5 (5 to 18.2)	9 (2.2 to 15.7)
Sub-Saharan Africa	Western sub-Saharan Africa	Mauritania	Incidence	All Ages	1,418 (1,345 to 1,492)	766 (722 to 810)	652 (617 to 689)	2,718 (2,576 to 2,875)	1,488 (1,402 to 1,581)	1,230 (1,156 to 1,306)	91.6 (85.2 to 98.3)	94.2 (86.6 to 102.2)	88.6 (78.9 to 97.3)
Sub-Saharan Africa	Western sub-Saharan Africa	Mauritania	Incidence	Age-standardized	115.7 (109.2 to 122.6)	119.3 (111.7 to 127.1)	115 (108.5 to 122)	107.5 (101.2 to 113.9)	112.4 (105.6 to 119.6)	101.4 (95.2 to 107.9)	-7.1 (-10.1 to -4.1)	-5.7 (-9.7 to -1.9)	-11.8 (-15.6 to -7.9)
Sub-Saharan Africa	Western sub-Saharan Africa	Mauritania	Prevalence	All Ages	21,593 (20,455 to 22,770)	12,079 (11,377 to 12,808)	9,514 (9,008 to 10,006)	42,163 (39,788 to 44,643)	23,902 (22,473 to 25,371)	18,261 (16,940 to 19,535)	95.3 (85.6 to 103.4)	97.9 (88.3 to 107)	91.9 (78.2 to 103.5)
Sub-Saharan Africa	Western sub-Saharan Africa	Mauritania	Prevalence	Age-standardized	1825.8 (1716.7 to 1938.4)	1983.5 (1853.4 to 2114)	1666.8 (1567.5 to 1764.5)	1715.3 (1616 to 1823.6)	1928.4 (1806.1 to 2057)	1500 (1398.3 to 1604.6)	-6.1 (-10.2 to -2.4)	-2.8 (-7.4 to 1.6)	-10 (-15.5 to -5.1)
Sub-Saharan Africa	Western sub-Saharan Africa	Mauritania	Deaths	All Ages	264 (184 to 328)	112 (51 to 148)	152 (109 to 195)	320 (246 to 405)	133 (91 to 179)	187 (143 to 243)	21.2 (-7.7 to 65.6)	18.7 (-16.8 to 123.2)	23.1 (-13.2 to 76.5)
Sub-Saharan Africa	Western sub-Saharan Africa	Mauritania	Deaths	Age-standardized	30.5 (21.6 to 38)	23.5 (10.5 to 32.7)	40.3 (28.7 to 50.7)	18.7 (14.6 to 23.3)	15.3 (10.4 to 20.4)	21.8 (16.8 to 28)	-38.8 (-52.7 to -17.9)	-34.6 (-53.9 to 20.7)	-45.8 (-60.5 to -23.7)
Sub-Saharan Africa	Western sub-Saharan Africa	Mauritania	DALYs	All Ages	8,295 (6,512 to 9,752)	3,961 (2,588 to 4,961)	4,333 (3,352 to 5,356)	11,199 (9,319 to 13,247)	5,698 (4,605 to 6,765)	5,501 (4,416 to 6,781)	35 (10.5 to 68.7)	43.8 (13.8 to 106.7)	26.9 (-3.2 to 65.9)
Sub-Saharan Africa	Western sub-Saharan Africa	Mauritania	DALYs	Age-standardized	784.7 (610.7 to 927.1)	699.3 (447.2 to 887.3)	897 (694.4 to 1099.2)	513.4 (428.1 to 607.6)	507.7 (406.7 to 604.6)	517.3 (417.4 to 632.7)	-34.6 (-46.6 to -18.3)	-27.4 (-43.1 to 6.8)	-42.3 (-55.8 to -24.1)
Sub-Saharan Africa	Western sub-Saharan Africa	Mauritania	YLLs	All Ages	5,691 (3,955 to 7,120)	2,456 (1,057 to 3,426)	3,235 (2,283 to 4,226)	6,098 (4,478 to 8,097)	2,701 (1,795 to 3,711)	3,396 (2,434 to 4,618)	7.1 (-22.3 to 51.6)	10 (-25.7 to 107.1)	5 (-29.4 to 55.5)
Sub-Saharan Africa	Western sub-Saharan Africa	Mauritania	YLLs	Age-standardized	566.8 (395.1 to 708)	454.9 (196.2 to 640.1)	706.7 (500.8 to 913.3)	308.2 (231.2 to 400.8)	269.3 (178.7 to 367.6)	345.8 (258.3 to 459.3)	-45.6 (-59.4 to -24.8)	-40.8 (-59.5 to 12.7)	-51.1 (-66 to -29.1)
Sub-Saharan Africa	Western sub-Saharan Africa	Mauritania	YLDs	All Ages	2,604 (2,164 to 2,968)	1,506 (1,248 to 1,764)	1,098 (897 to 1,283)	5,101 (4,293 to 5,789)	2,997 (2,485 to 3,489)	2,104 (1,742 to 2,462)	95.9 (83.5 to 107.5)	99 (84.1 to 113.2)	91.6 (73.5 to 109.6)
Sub-Saharan Africa	Western sub-Saharan Africa	Mauritania	YLDs	Age-standardized	218 (181.6 to 250)	244.5 (201.6 to 286.9)	190.4 (155.6 to 221.5)	205.2 (173 to 233)	238.4 (196.8 to 276.5)	171.5 (142.3 to 198.7)	-5.9 (-10.9 to -1.3)	-2.5 (-8.7 to 3.5)	-9.9 (-16.6 to -3.3)
Sub-Saharan Africa	Western sub-Saharan Africa	Niger	Incidence	All Ages	4,347 (4,102 to 4,627)	2,153 (2,022 to 2,302)	2,195 (2,068 to 2,344)	12,296 (11,593 to 12,997)	6,354 (5,961 to 6,753)	5,942 (5,603 to 6,279)	182.9 (174.3 to 191)	195.2 (183.2 to 207.3)	170.8 (160.1 to 180.9)
Sub-Saharan Africa	Western sub-Saharan Africa	Niger	Incidence	Age-standardized	109.5 (102.2 to 118)	103.7 (95.7 to 112.7)	120.3 (112.9 to 128.7)	110.6 (103.5 to 117.9)	107 (98.9 to 115.4)	114.7 (107.7 to 122.2)	1 (-2.2 to 4.5)	3.2 (-1.5 to 8.3)	-4.6 (-8.5 to -0.9)
Sub-Saharan Africa	Western sub-Saharan Africa	Niger	Prevalence	All Ages	62,840 (59,577 to 66,161)	31,805 (30,042 to 33,559)	31,035 (29,364 to 32,785)	179,661 (169,575 to 189,387)	94,640 (88,899 to 100,448)	85,021 (80,420 to 89,664)	185.9 (176.8 to 194.5)	197.6 (185.5 to 209.5)	174 (162.8 to 183.3)
Sub-Saharan Africa	Western sub-Saharan Africa	Niger	Prevalence	Age-standardized	1633.8 (1529 to 1749.5)	1678.4 (1562.4 to 1814)	1620.1 (1522.4 to 1732.1)	1718.5 (1606.7 to 1832.1)	1767.1 (1644.3 to 1895.2)	1669.9 (1565.2 to 1775.8)	5.2 (2 to 8.5)	5.3 (1.1 to 10.1)	3.1 (-1 to 7)
Sub-Saharan Africa	Western sub-Saharan Africa	Niger	Deaths	All Ages	817 (597 to 1,170)	305 (194 to 435)	512 (340 to 890)	1,790 (1,281 to 2,557)	760 (481 to 1,103)	1,031 (655 to 1,708)	119 (64.9 to 194.5)	148.6 (70.3 to 281.3)	101.4 (42.1 to 183.7)
Sub-Saharan Africa	Western sub-Saharan Africa	Niger	Deaths	Age-standardized	37.5 (27.8 to 53.1)	27 (17.8 to 37.7)	50.7 (35.6 to 84.8)	30.6 (22.4 to 43)	23.8 (15.7 to 34)	38.1 (24.7 to 61.1)	-18.4 (-35.9 to 5.5)	-11.8 (-38.6 to 32.4)	-24.7 (-45.3 to 5.5)
Sub-Saharan Africa	Western sub-Saharan Africa	Niger	DALYs	All Ages	28,841 (22,698 to 38,930)	12,929 (8,945 to 18,089)	15,912 (11,734 to 26,683)	64,941 (51,345 to 84,879)	31,468 (23,492 to 42,483)	33,473 (24,551 to 51,293)	125.2 (77.6 to 182.2)	143.4 (75.8 to 241.4)	110.4 (60 to 179.1)
Sub-Saharan Africa	Western sub-Saharan Africa	Niger	DALYs	Age-standardized	910.2 (721.2 to 1224)	745.5 (546.5 to 960.3)	1086.8 (798.4 to 1767)	751.5 (594 to 991.4)	664.5 (504.2 to 869.8)	846 (605 to 1278.3)	-17.4 (-33.4 to 3.1)	-10.9 (-31.8 to 19)	-22.2 (-40.7 to 2.8)
Sub-Saharan Africa	Western sub-Saharan Africa	Niger	YLLs	All Ages	21,250 (15,105 to 30,974)	8,923 (4,963 to 14,461)	12,326 (8,104 to 22,749)	43,149 (29,852 to 62,560)	19,537 (11,781 to 30,631)	23,612 (14,649 to 41,088)	103.1 (43.1 to 178.7)	118.9 (37.9 to 267.1)	91.6 (32.3 to 179)
Sub-Saharan Africa	Western sub-Saharan Africa	Niger	YLLs	Age-standardized	715.9 (521.8 to 1033.4)	538.3 (344.8 to 756.6)	902.2 (610.7 to 1578.8)	546.6 (389.2 to 789.6)	446.1 (283.1 to 645.1)	654.9 (418.4 to 1086.7)	-23.7 (-42.4 to 2.1)	-17.1 (-43 to 25.9)	-27.4 (-48.4 to 2.7)
Sub-Saharan Africa	Western sub-Saharan Africa	Niger	YLDs	All Ages	7,591 (6,278 to 8,675)	4,006 (3,268 to 4,670)	3,585 (2,907 to 4,204)	21,792 (18,106 to 24,915)	11,931 (9,779 to 13,864)	9,861 (8,078 to 11,598)	187.1 (169.8 to 205.3)	197.8 (173.2 to 224.3)	175 (151.2 to 201)
Sub-Saharan Africa	Western sub-Saharan Africa	Niger	YLDs	Age-standardized	194.3 (161.5 to 221.3)	207.2 (169.6 to 241)	184.6 (152.3 to 214.9)	204.9 (171.1 to 233.5)	218.3 (178.9 to 253.5)	191 (156.7 to 222.8)	5.5 (0.8 to 10.5)	5.3 (-1.3 to 12.3)	3.5 (-3.1 to 10.5)
Sub-Saharan Africa	Western sub-Saharan Africa	Nigeria	Incidence	All Ages	55,980 (51,502 to 60,923)	28,273 (26,004 to 30,733)	27,706 (25,359 to 30,342)	101,611 (93,568 to 110,326)	56,033 (51,500 to 61,325)	45,577 (41,742 to 49,344)	81.5 (72.9 to 92.2)	98.2 (88.8 to 109.6)	64.5 (54.3 to 76)
Sub-Saharan Africa	Western sub-Saharan Africa	Nigeria	Incidence	Age-standardized	104.8 (94.6 to 115.4)	106.7 (96.4 to 117.8)	109.2 (98.6 to 120.5)	85.9 (78.1 to 94.2)	86 (78.1 to 94.7)	84.9 (76.7 to 93.4)	-18 (-21.4 to -12.9)	-19.4 (-22.9 to -13.9)	-22.3 (-25.7 to -17.3)
Sub-Saharan Africa	Western sub-Saharan Africa	Nigeria	Prevalence	All Ages	855,433 (795,129 to 923,742)	438,762 (405,582 to 475,305)	416,670 (387,016 to 449,820)	1,587,743 (1,449,908 to 1,718,544)	899,508 (825,527 to 973,711)	688,236 (622,354 to 746,734)	85.6 (74.2 to 96.2)	105 (92.8 to 116.8)	65.2 (53.9 to 76.5)
Sub-Saharan Africa	Western sub-Saharan Africa	Nigeria	Prevalence	Age-standardized	1646.1 (1514.7 to 1797.3)	1765.9 (1615 to 1933.4)	1561 (1437.3 to 1696.5)	1356 (1245 to 1478.4)	1456.3 (1334.2 to 1596.2)	1241.4 (1133.8 to 1344.1)	-17.6 (-21.3 to -13)	-17.5 (-21.5 to -12.7)	-20.5 (-24.6 to -15.5)
Sub-Saharan Africa	Western sub-Saharan Africa	Nigeria	Deaths	All Ages	8,995 (7,044 to 11,435)	3,559 (2,491 to 4,950)	5,436 (3,787 to 7,479)	14,120 (10,738 to 17,549)	5,315 (3,579 to 7,043)	8,805 (6,139 to 11,979)	57 (15.8 to 107.7)	49.3 (0.3 to 108.9)	62 (2.5 to 145.9)
Sub-Saharan Africa	Western sub-Saharan Africa	Nigeria	Deaths	Age-standardized	25.1 (19.6 to 31.3)	18 (12.7 to 25.2)	35.1 (25 to 46.5)	21.8 (16.9 to 26.8)	15 (10.4 to 19.4)	29 (20.3 to 38.7)	-13.1 (-35 to 13.1)	-16.6 (-43.7 to 16)	-17.5 (-45.7 to 20.2)
Sub-Saharan Africa	Western sub-Saharan Africa	Nigeria	DALYs	All Ages	293,612 (246,938 to 352,950)	131,058 (104,581 to 163,256)	162,553 (125,565 to 211,329)	479,461 (397,882 to 566,250)	229,928 (183,379 to 278,240)	249,533 (190,337 to 321,424)	63.3 (32.8 to 97)	75.4 (40.6 to 115.6)	53.5 (9.1 to 107.9)
Sub-Saharan Africa	Western sub-Saharan Africa	Nigeria	DALYs	Age-standardized	639.9 (532.2 to 768.4)	551.1 (437.9 to 685.7)	756 (583.9 to 980.1)	523 (430.7 to 623.3)	444.4 (350.1 to 535.3)	605.1 (455.2 to 779.1)	-18.3 (-33.9 to 0.1)	-19.4 (-36.9 to 0.6)	-20 (-43.4 to 9.9)
Sub-Saharan Africa	Western sub-Saharan Africa	Nigeria	YLLs	All Ages	191,661 (148,143 to 245,457)	76,920 (52,205 to 107,176)	114,741 (77,681 to 162,138)	286,800 (216,038 to 367,679)	117,195 (75,617 to 159,373)	169,605 (111,025 to 240,145)	49.6 (6.6 to 102.6)	52.4 (-0.3 to 123.6)	47.8 (-9.8 to 129.5)
Sub-Saharan Africa	Western sub-Saharan Africa	Nigeria	YLLs	Age-standardized	447.4 (345.8 to 572.6)	337.6 (232.7 to 465.8)	580.3 (404.6 to 795.8)	362.3 (273.6 to 455.8)	266.7 (177.6 to 356.9)	463.5 (319.4 to 639.7)	-19 (-41 to 7.8)	-21 (-48.1 to 12.1)	-20.1 (-49 to 21.2)
Sub-Saharan Africa	Western sub-Saharan Africa	Nigeria	YLDs	All Ages	101,951 (83,886 to 116,667)	54,138 (44,450 to 63,216)	47,813 (39,106 to 55,364)	192,661 (158,614 to 222,305)	112,733 (91,738 to 132,894)	79,928 (65,321 to 93,049)	89 (77.1 to 100.4)	108.2 (95.3 to 120.8)	67.2 (55.3 to 78.8)
Sub-Saharan Africa	Western sub-Saharan Africa	Nigeria	YLDs	Age-standardized	192.5 (159.1 to 221.4)	213.6 (175 to 251.9)	175.6 (143.8 to 203)	160.8 (133.5 to 184.7)	177.7 (145.8 to 209.4)	141.5 (116.5 to 164)	-16.5 (-20.4 to -11.8)	-16.8 (-20.9 to -12)	-19.4 (-23.5 to -14.3)
Sub-Saharan Africa	Western sub-Saharan Africa	Sao Tome and Principe	Incidence	All Ages	125 (118 to 131)	60 (57 to 64)	64 (61 to 68)	289 (276 to 303)	134 (127 to 141)	156 (148 to 163)	132.1 (124.1 to 140.4)	121.8 (112.3 to 131.1)	141.7 (130.9 to 153.5)
Sub-Saharan Africa	Western sub-Saharan Africa	Sao Tome and Principe	Incidence	Age-standardized	179.8 (170.3 to 189.3)	161.6 (152 to 171.2)	206.1 (194.5 to 217.5)	236.5 (223.9 to 250.1)	206.1 (192.7 to 220.8)	272.2 (258.4 to 285.6)	31.5 (26.6 to 36.5)	27.5 (21.2 to 33.6)	32 (26.5 to 38.5)
Sub-Saharan Africa	Western sub-Saharan Africa	Sao Tome and Principe	Prevalence	All Ages	1,750 (1,651 to 1,851)	922 (868 to 977)	828 (777 to 885)	3,934 (3,787 to 4,087)	1,937 (1,853 to 2,021)	1,997 (1,916 to 2,071)	124.8 (114.3 to 136)	110 (99.6 to 121.5)	141.3 (127.5 to 156.1)
Sub-Saharan Africa	Western sub-Saharan Africa	Sao Tome and Principe	Prevalence	Age-standardized	2535.5 (2382.8 to 2684.5)	2532.6 (2374.5 to 2690.5)	2596.2 (2430.3 to 2770)	3392 (3221.6 to 3560)	3201.6 (3030.5 to 3387.8)	3625.7 (3436.8 to 3800.8)	33.8 (27.4 to 40.8)	26.4 (19.5 to 34.1)	39.7 (31.9 to 48.5)
Sub-Saharan Africa	Western sub-Saharan Africa	Sao Tome and Principe	Deaths	All Ages	32 (25 to 37)	13 (9 to 16)	19 (14 to 23)	48 (37 to 60)	18 (10 to 25)	30 (21 to 40)	53.1 (13.3 to 96.4)	41.9 (-12.4 to 95.5)	60.7 (7 to 123.7)
Sub-Saharan Africa	Western sub-Saharan Africa	Sao Tome and Principe	Deaths	Age-standardized	57.6 (46.6 to 67.7)	41.9 (30.8 to 53)	82.6 (65.6 to 100.1)	58.4 (44.2 to 71.6)	41.3 (22.5 to 56.7)	78.8 (56.6 to 105.7)	1.5 (-22.7 to 29.1)	-1.4 (-39.5 to 36.7)	-4.5 (-33.7 to 30.9)
Sub-Saharan Africa	Western sub-Saharan Africa	Sao Tome and Principe	DALYs	All Ages	854 (701 to 992)	383 (303 to 460)	471 (367 to 574)	1,461 (1,187 to 1,713)	610 (446 to 775)	851 (662 to 1,079)	71.1 (36.8 to 111.5)	59.4 (25.1 to 99.2)	80.6 (28.4 to 138.3)
Sub-Saharan Africa	Western sub-Saharan Africa	Sao Tome and Principe	DALYs	Age-standardized	1312.6 (1091.5 to 1518.3)	1082.8 (862.1 to 1303.5)	1627.7 (1296.7 to 1955.6)	1408.8 (1148.7 to 1656.6)	1109.3 (780.5 to 1408.8)	1750.1 (1350.4 to 2227.3)	7.3 (-14.4 to 29.9)	2.4 (-22.1 to 27.8)	7.5 (-21.8 to 39.8)
Sub-Saharan Africa	Western sub-Saharan Africa	Sao Tome and Principe	YLLs	All Ages	646 (496 to 783)	268 (194 to 348)	377 (275 to 479)	996 (738 to 1,244)	371 (210 to 527)	625 (438 to 855)	54.3 (11.3 to 105.4)	38.2 (-13.6 to 94.3)	65.7 (4.8 to 137.6)
Sub-Saharan Africa	Western sub-Saharan Africa	Sao Tome and Principe	YLLs	Age-standardized	1013.2 (789.7 to 1219.8)	771.6 (561.5 to 997.9)	1335.8 (1019 to 1656.7)	1013.9 (761 to 1260.6)	719.9 (402 to 1008.8)	1346.4 (951.6 to 1823.2)	0.1 (-26.9 to 29.3)	-6.7 (-41.7 to 29.6)	0.8 (-33.5 to 40.3)
Sub-Saharan Africa	Western sub-Saharan Africa	Sao Tome and Principe	YLDs	All Ages	209 (171 to 237)	114 (93 to 132)	94 (77 to 110)	466 (389 to 523)	239 (198 to 276)	226 (186 to 263)	123.1 (110.7 to 136.4)	109.2 (95.1 to 123.2)	140 (121.7 to 159.4)
Sub-Saharan Africa	Western sub-Saharan Africa	Sao Tome and Principe	YLDs	Age-standardized	299.3 (246 to 340.3)	311.1 (253 to 361.7)	291.9 (237.7 to 336.8)	394.8 (329.1 to 444)	389.4 (320.6 to 450.1)	403.7 (333.4 to 466.5)	31.9 (24.5 to 39.8)	25.2 (17 to 33.6)	38.3 (28.8 to 48.9)
Sub-Saharan Africa	Western sub-Saharan Africa	Senegal	Incidence	All Ages	4,734 (4,493 to 4,976)	2,167 (2,046 to 2,288)	2,567 (2,426 to 2,715)	10,332 (9,794 to 10,878)	4,887 (4,622 to 5,186)	5,445 (5,151 to 5,723)	118.2 (112.7 to 123.8)	125.5 (117.4 to 133.5)	112.1 (105.5 to 119.4)
Sub-Saharan Africa	Western sub-Saharan Africa	Senegal	Incidence	Age-standardized	114.3 (107.7 to 121.4)	96.4 (90.1 to 102.5)	133.6 (126.1 to 141.9)	110.7 (104.9 to 117.4)	97.4 (91.6 to 104.2)	126.2 (119.1 to 133.8)	-3.2 (-5.9 to -0.4)	1 (-2.8 to 5)	-5.5 (-8.8 to -1.6)

Chronic obstructive pulmonary disease

7 GBD super region	21 GBD region	Location	Measure	Age	1990			2019			Percent change (1990 to 2019)		
					Both	Female	Male	Both	Female	Male	Both	Female	Male
Sub-Saharan Africa	Western sub-Saharan Africa	Senegal	Prevalence	All Ages	69,441 (65,831 to 72,810)	33,625 (31,874 to 35,523)	35,816 (33,892 to 37,733)	159,836 (151,194 to 168,298)	80,025 (75,408 to 85,193)	79,811 (75,436 to 84,079)	130.2 (122.7 to 137)	138 (127.4 to 148.5)	122.8 (114.9 to 131.2)
Sub-Saharan Africa	Western sub-Saharan Africa	Senegal	Prevalence	Age-standardized	1732.2 (1630.1 to 1833.6)	1618.9 (1517.2 to 1727.3)	1855.6 (1746.8 to 1972.2)	1758.6 (1655.1 to 1860.8)	1686.2 (1581.3 to 1797.8)	1845.1 (1728.8 to 1951.5)	1.5 (-1.5 to 4.4)	4.2 (-0.4 to 8.5)	-0.6 (-4.1 to 3.5)
Sub-Saharan Africa	Western sub-Saharan Africa	Senegal	Deaths	All Ages	950 (735 to 1,217)	312 (221 to 410)	638 (457 to 884)	1,579 (1,240 to 1,948)	589 (421 to 767)	990 (746 to 1,304)	66.1 (26.3 to 121.8)	88.4 (36.8 to 155.9)	55.2 (11.8 to 123.6)
Sub-Saharan Africa	Western sub-Saharan Africa	Senegal	Deaths	Age-standardized	35.8 (28.3 to 45)	22.7 (16.3 to 29.6)	50.1 (37 to 69)	25.8 (20.5 to 31.4)	18 (13 to 23.3)	35.2 (26.8 to 45.1)	-27.9 (-43.9 to -5.8)	-20.9 (-42 to 7.5)	-29.9 (-47.8 to -2)
Sub-Saharan Africa	Western sub-Saharan Africa	Senegal	DALYs	All Ages	29,029 (23,666 to 35,510)	11,602 (9,000 to 14,247)	17,428 (13,469 to 23,348)	51,815 (43,596 to 61,521)	22,236 (18,149 to 26,589)	29,579 (23,710 to 36,803)	78.5 (44.1 to 123.1)	91.7 (53.6 to 137.7)	69.7 (29.5 to 126)
Sub-Saharan Africa	Western sub-Saharan Africa	Senegal	DALYs	Age-standardized	845.7 (688.2 to 1040.3)	628.8 (494.2 to 762.2)	1067 (824.6 to 1417.1)	657.4 (549.4 to 779)	528.2 (429.4 to 634.2)	803.1 (646.9 to 998.3)	-22.3 (-36.2 to -3.2)	-16 (-33 to 3)	-24.7 (-42.5 to -0.2)
Sub-Saharan Africa	Western sub-Saharan Africa	Senegal	YLLs	All Ages	20,754 (15,424 to 27,031)	7,405 (4,995 to 10,013)	13,349 (9,324 to 19,162)	32,742 (24,919 to 42,053)	12,280 (8,505 to 16,308)	20,462 (14,693 to 27,455)	57.8 (16.5 to 120.3)	65.8 (14.3 to 134.7)	53.3 (6.6 to 129.1)
Sub-Saharan Africa	Western sub-Saharan Africa	Senegal	YLLs	Age-standardized	642.5 (491.6 to 829.3)	430.2 (302.2 to 560.6)	858.4 (620.1 to 1214.3)	450.7 (349.6 to 566.9)	321.6 (226.7 to 420.1)	595.4 (445 to 793.9)	-29.9 (-46.7 to -5.6)	-25.2 (-47.1 to 2.2)	-30.6 (-50.6 to -0.3)
Sub-Saharan Africa	Western sub-Saharan Africa	Senegal	YLDs	All Ages	8,276 (6,851 to 9,431)	4,197 (3,379 to 4,923)	4,079 (3,350 to 4,772)	19,073 (15,936 to 21,762)	9,956 (8,116 to 11,609)	9,117 (7,429 to 10,659)	130.5 (116.9 to 143.4)	137.2 (117.3 to 156.6)	123.5 (107.3 to 139.2)
Sub-Saharan Africa	Western sub-Saharan Africa	Senegal	YLDs	Age-standardized	203.2 (167.9 to 232.3)	198.6 (160.3 to 232.2)	208.6 (170.5 to 242.6)	206.8 (172.7 to 235.4)	206.6 (169.2 to 240.5)	207.8 (170.3 to 241)	1.7 (-3 to 6.2)	4 (-2.9 to 11.1)	-0.4 (-6.2 to 5.6)
Sub-Saharan Africa	Western sub-Saharan Africa	Sierra Leone	Incidence	All Ages	2,501 (2,375 to 2,647)	1,176 (1,112 to 1,245)	1,325 (1,251 to 1,407)	5,639 (5,352 to 5,932)	2,961 (2,804 to 3,122)	2,678 (2,542 to 2,826)	125.5 (118.8 to 132)	151.8 (142.7 to 161.2)	102.1 (94.6 to 109.4)
Sub-Saharan Africa	Western sub-Saharan Africa	Sierra Leone	Incidence	Age-standardized	109 (102.8 to 115.8)	98.1 (92.1 to 104.9)	120.4 (113.5 to 128)	117.5 (110.8 to 124.5)	120.2 (112.8 to 127.8)	116.8 (110.4 to 124)	7.8 (4.6 to 10.8)	22.5 (17.9 to 27.3)	-3 (-6.4 to 0.9)
Sub-Saharan Africa	Western sub-Saharan Africa	Sierra Leone	Prevalence	All Ages	37,561 (35,682 to 39,399)	18,715 (17,750 to 19,699)	18,846 (17,825 to 19,904)	86,262 (81,988 to 90,499)	46,111 (43,686 to 48,556)	40,151 (38,186 to 42,144)	129.7 (123.5 to 135.9)	146.4 (138.1 to 155.3)	113 (105.4 to 120.5)
Sub-Saharan Africa	Western sub-Saharan Africa	Sierra Leone	Prevalence	Age-standardized	1672.3 (1576.6 to 1767.8)	1650.4 (1548.1 to 1756.9)	1701.7 (1598.7 to 1803)	1874.5 (1767 to 1986.5)	2023.7 (1898.7 to 2154.8)	1736.7 (1637.6 to 1844.8)	12.1 (8.8 to 15.4)	22.6 (17.8 to 27.8)	2.1 (-1.7 to 5.7)
Sub-Saharan Africa	Western sub-Saharan Africa	Sierra Leone	Deaths	All Ages	599 (459 to 753)	196 (130 to 267)	404 (292 to 530)	802 (591 to 1,054)	325 (214 to 473)	477 (323 to 664)	33.8 (1.5 to 81.4)	66.1 (18.7 to 133.5)	18.1 (-16.2 to 70.5)
Sub-Saharan Africa	Western sub-Saharan Africa	Sierra Leone	Deaths	Age-standardized	35.6 (27.3 to 44.5)	23.2 (15.8 to 31.4)	48 (35.2 to 62.5)	27.3 (20.4 to 34.9)	21 (14.2 to 30)	34.6 (23.8 to 46.4)	-23.3 (-40.8 to 0.8)	-9.7 (-34.7 to 25.4)	-28.1 (-48.3 to 1.8)
Sub-Saharan Africa	Western sub-Saharan Africa	Sierra Leone	DALYs	All Ages	17,000 (13,680 to 20,613)	6,781 (5,059 to 8,792)	10,219 (7,874 to 13,210)	28,363 (22,972 to 35,037)	13,284 (10,239 to 17,067)	15,079 (11,435 to 19,859)	66.8 (34.6 to 109.3)	95.9 (58.4 to 149.4)	47.6 (11 to 98.6)
Sub-Saharan Africa	Western sub-Saharan Africa	Sierra Leone	DALYs	Age-standardized	841.1 (678.8 to 1012.4)	643.6 (486.9 to 818.4)	1031.8 (792.9 to 1312.1)	716 (578 to 878.7)	644.7 (498.3 to 830.4)	795.9 (598.2 to 1038.5)	-14.9 (-30.5 to 7.5)	0.2 (-19.6 to 26)	-22.9 (-41.6 to 4.3)
Sub-Saharan Africa	Western sub-Saharan Africa	Sierra Leone	YLLs	All Ages	12,508 (9,329 to 16,094)	4,437 (2,779 to 6,376)	8,071 (5,777 to 11,119)	17,996 (12,734 to 24,335)	7,522 (4,603 to 11,278)	10,473 (6,856 to 15,157)	43.9 (4.9 to 102.5)	69.5 (17.6 to 152.7)	29.8 (-12.5 to 93.9)
Sub-Saharan Africa	Western sub-Saharan Africa	Sierra Leone	YLLs	Age-standardized	642.9 (486.4 to 821.7)	439.2 (287.4 to 607.9)	839.1 (606.6 to 1127.9)	493.8 (359.2 to 658)	396 (257.6 to 580.1)	598.8 (403.9 to 842.3)	-23.2 (-42.5 to 6)	-9.8 (-35.8 to 28.6)	-28.6 (-50.6 to 4.8)
Sub-Saharan Africa	Western sub-Saharan Africa	Sierra Leone	YLDs	All Ages	4,492 (3,754 to 5,076)	2,344 (1,930 to 2,709)	2,148 (1,769 to 2,497)	10,367 (8,708 to 11,739)	5,762 (4,756 to 6,648)	4,605 (3,765 to 5,364)	130.8 (118.6 to 143.7)	145.8 (127.5 to 164.6)	114.4 (98.9 to 131)
Sub-Saharan Africa	Western sub-Saharan Africa	Sierra Leone	YLDs	Age-standardized	198.2 (165.6 to 224.7)	204.4 (168.8 to 236.4)	192.7 (159.1 to 223.9)	222.3 (185.5 to 251.5)	248.6 (204 to 289.5)	197.1 (161.9 to 228.3)	12.2 (6.9 to 17.2)	21.6 (14 to 29.4)	2.3 (-3.7 to 8.5)
Sub-Saharan Africa	Western sub-Saharan Africa	Togo	Incidence	All Ages	2,253 (2,141 to 2,370)	1,145 (1,083 to 1,211)	1,108 (1,054 to 1,166)	5,886 (5,541 to 6,265)	2,910 (2,731 to 3,104)	2,975 (2,776 to 3,167)	161.2 (153.6 to 168.8)	154.2 (144.3 to 163.7)	168.4 (157.7 to 179.7)
Sub-Saharan Africa	Western sub-Saharan Africa	Togo	Incidence	Age-standardized	125.4 (118.3 to 133)	115.1 (107.9 to 122.6)	139.4 (131.8 to 148.1)	121 (114 to 128.4)	108.7 (101.8 to 116.3)	141 (132.9 to 149.3)	-3.5 (-6.2 to -0.8)	-5.6 (-9.3 to -1.8)	1.1 (-2.9 to 4.8)
Sub-Saharan Africa	Western sub-Saharan Africa	Togo	Prevalence	All Ages	32,803 (31,145 to 34,368)	17,256 (16,371 to 18,197)	15,547 (14,726 to 16,351)	91,070 (86,074 to 96,044)	47,155 (44,458 to 49,789)	43,915 (41,333 to 46,641)	177.6 (169.8 to 185.1)	173.3 (161.7 to 183.8)	182.5 (171.1 to 193.5)
Sub-Saharan Africa	Western sub-Saharan Africa	Togo	Prevalence	Age-standardized	1953.1 (1836.8 to 2073.3)	1926.8 (1801.1 to 2052.7)	1997.7 (1871.4 to 2118.6)	1969.2 (1849.7 to 2094.9)	1853 (1735.8 to 1976.4)	2137.8 (1995 to 2274.1)	0.8 (-2.2 to 3.7)	-3.8 (-8 to 0.1)	7 (2.6 to 11.3)
Sub-Saharan Africa	Western sub-Saharan Africa	Togo	Deaths	All Ages	340 (266 to 417)	141 (98 to 188)	199 (147 to 256)	717 (527 to 942)	306 (213 to 439)	411 (270 to 570)	110.6 (50.5 to 183)	116.2 (55.5 to 194.9)	106.6 (21.7 to 197.9)
Sub-Saharan Africa	Western sub-Saharan Africa	Togo	Deaths	Age-standardized	35.2 (27.6 to 42.6)	26.2 (18.7 to 34.6)	46.9 (35.1 to 58.4)	26.1 (19.7 to 33.9)	18.6 (13 to 26.9)	39.5 (27.5 to 52.5)	-25.9 (-44.2 to -2.4)	-29 (-47.9 to -4.6)	-15.7 (-46.4 to 18.5)
Sub-Saharan Africa	Western sub-Saharan Africa	Togo	DALYs	All Ages	11,962 (9,979 to 14,002)	5,677 (4,399 to 7,025)	6,285 (5,098 to 7,781)	28,098 (23,026 to 34,501)	12,792 (10,229 to 16,376)	15,306 (11,239 to 19,773)	134.9 (85.5 to 191)	125.3 (82.6 to 174.6)	143.5 (65.5 to 220.9)
Sub-Saharan Africa	Western sub-Saharan Africa	Togo	DALYs	Age-standardized	870.6 (721.5 to 1014.7)	737.1 (576.3 to 912.5)	1032.9 (820.7 to 1272.3)	713.4 (583.9 to 876.2)	567.3 (454.5 to 728.3)	938.5 (704 to 1194.1)	-18.1 (-35 to 1.3)	-23 (-37.9 to -5.5)	-9.1 (-37.6 to 20.4)
Sub-Saharan Africa	Western sub-Saharan Africa	Togo	YLLs	All Ages	8,011 (6,216 to 10,038)	3,512 (2,339 to 4,798)	4,499 (3,330 to 5,995)	17,134 (12,156 to 23,224)	6,882 (4,655 to 10,192)	10,252 (6,329 to 14,653)	113.9 (45.4 to 194.8)	95.9 (38.2 to 177)	127.9 (25.5 to 239.1)
Sub-Saharan Africa	Western sub-Saharan Africa	Togo	YLLs	Age-standardized	639.2 (499.9 to 786.2)	499.9 (344.4 to 670.8)	806.6 (597.1 to 1034.4)	479.4 (350.3 to 632.7)	338.6 (233.3 to 488.6)	696.1 (465.8 to 957.1)	-25 (-46.6 to 1.1)	-32.3 (-51.8 to -5.9)	-13.7 (-49 to 24.6)
Sub-Saharan Africa	Western sub-Saharan Africa	Togo	YLDs	All Ages	3,951 (3,259 to 4,493)	2,165 (1,775 to 2,513)	1,786 (1,467 to 2,093)	10,964 (9,134 to 12,440)	5,910 (4,834 to 6,889)	5,054 (4,106 to 5,914)	177.5 (162.8 to 192.9)	173 (151.8 to 193.7)	183 (161.8 to 204.5)
Sub-Saharan Africa	Western sub-Saharan Africa	Togo	YLDs	Age-standardized	231.4 (192.7 to 262.9)	237.1 (194.8 to 277.1)	226.3 (185.4 to 263.8)	234 (195.8 to 266.1)	228.8 (188.4 to 266)	242.5 (199.9 to 282.7)	1.1 (-3.1 to 5.5)	-3.5 (-9.6 to 2.7)	7.1 (1 to 13.9)

Pneumoconiosis

7 GBD super region	21 GBD region	Location	Measure	Age	1990			2019			Percent change (1990 to 2019)		
					Both	Female	Male	Both	Female	Male	Both	Female	Male
		Southeast Asia, East Asia, and Oceania	Incidence	All Ages	85,900 (71,918 to 103,833)	6,788 (5,725 to 7,957)	79,111 (65,644 to 96,757)	141,904 (118,022 to 168,472)	9,882 (8,207 to 11,952)	132,022 (109,037 to 156,792)	65.2 (42.1 to 87.9)	45.6 (34 to 57.1)	66.9 (41.7 to 92)
		Southeast Asia, East Asia, and Oceania	Incidence	Age-standardized	5.9 (5 to 7.1)	1 (0.9 to 1.2)	10.6 (8.9 to 12.8)	5 (4.2 to 5.8)	0.7 (0.6 to 0.9)	9.3 (7.8 to 10.9)	-15.6 (-24.4 to -7.5)	-31.2 (-35.4 to -27)	-12.7 (-22.7 to -3.6)
		Southeast Asia, East Asia, and Oceania	Prevalence	All Ages	1,356,420 (1,106,368 to 1,703,805)	78,490 (64,898 to 94,282)	1,277,930 (1,034,002 to 1,617,880)	2,654,389 (2,213,080 to 3,154,564)	129,646 (108,480 to 155,634)	2,524,743 (2,093,692 to 3,023,619)	95.7 (70.3 to 119.2)	65.2 (53.1 to 78.2)	97.6 (70.5 to 123.1)
		Southeast Asia, East Asia, and Oceania	Prevalence	Age-standardized	107.9 (88.6 to 134.5)	13.4 (11.1 to 16.2)	205.9 (168.4 to 257.3)	93.5 (78.2 to 110.7)	9.3 (7.8 to 11.2)	184 (153.5 to 218.6)	-13.4 (-24.2 to -3.9)	-30.5 (-35.3 to -25.6)	-10.6 (-22.3 to -0.2)
		Southeast Asia, East Asia, and Oceania	Deaths	All Ages	11,461 (8,066 to 14,471)	639 (449 to 1,263)	10,822 (7,435 to 13,882)	10,843 (8,689 to 14,153)	633 (456 to 831)	10,210 (7,983 to 13,480)	-5.4 (-34.5 to 60.3)	-1 (-54.9 to 57)	-5.7 (-36.3 to 65.1)
		Southeast Asia, East Asia, and Oceania	Deaths	Age-standardized	1.1 (0.8 to 1.3)	0.1 (0.1 to 0.2)	2.2 (1.5 to 2.8)	0.4 (0.3 to 0.6)	0 (0 to 0.1)	0.9 (0.7 to 1.2)	-59.4 (-71.5 to -31.6)	-58.1 (-80.4 to -35.1)	-58.7 (-71.4 to -27.9)
		Southeast Asia, East Asia, and Oceania	DALYs	All Ages	518,434 (394,067 to 641,344)	29,138 (21,545 to 46,169)	489,296 (365,670 to 609,125)	633,453 (495,964 to 806,655)	31,781 (24,461 to 41,099)	601,672 (465,915 to 768,244)	22.2 (-3.8 to 64.5)	9.1 (-36.3 to 41.5)	23 (-4.7 to 68.6)
		Southeast Asia, East Asia, and Oceania	DALYs	Age-standardized	41.1 (31.5 to 50.7)	4.6 (3.4 to 7.3)	80 (60.7 to 99.3)	22.6 (17.7 to 28.7)	2.3 (1.8 to 3)	44.8 (35.1 to 56.9)	-45 (-56.6 to -26.3)	-50.2 (-70.1 to -36.6)	-44 (-56.4 to -23.8)
		Southeast Asia, East Asia, and Oceania	YLLs	All Ages	323,254 (220,133 to 413,674)	17,717 (12,052 to 35,296)	305,537 (205,912 to 396,798)	255,296 (200,689 to 332,830)	12,789 (9,286 to 16,907)	242,508 (187,847 to 320,699)	-21 (-45.9 to 36)	-27.8 (-66.9 to 17.3)	-20.6 (-47.1 to 40.7)
		Southeast Asia, East Asia, and Oceania	YLLs	Age-standardized	25.7 (17.7 to 32.7)	2.7 (1.9 to 5.4)	50.7 (34.5 to 65.2)	9.3 (7.4 to 12.2)	0.9 (0.7 to 1.3)	18.7 (14.6 to 24.7)	-63.6 (-75 to -37.7)	-64.9 (-83.9 to -43.9)	-63 (-75.1 to -35.4)
		Southeast Asia, East Asia, and Oceania	YLDs	All Ages	195,180 (128,311 to 284,695)	11,421 (7,666 to 16,470)	183,759 (119,542 to 269,535)	378,157 (249,767 to 543,560)	18,993 (12,471 to 27,154)	359,164 (237,488 to 516,188)	93.7 (68.7 to 117.4)	66.3 (51.8 to 81.8)	95.5 (68.4 to 121.7)
		Southeast Asia, East Asia, and Oceania	YLDs	Age-standardized	15.4 (10.2 to 22.2)	1.9 (1.3 to 2.8)	29.3 (19.3 to 42.2)	13.3 (8.8 to 19.1)	1.4 (0.9 to 1.9)	26 (17.2 to 37.5)	-13.9 (-24.3 to -4.2)	-29.6 (-35.3 to -23.5)	-11.2 (-22.4 to -0.7)
		East Asia	Incidence	All Ages	84,482 (70,573 to 102,399)	6,342 (5,339 to 7,459)	78,139 (64,862 to 95,780)	138,826 (115,316 to 164,919)	9,179 (7,588 to 11,195)	129,647 (107,112 to 154,026)	64.3 (41.3 to 87.1)	44.7 (32.6 to 56.4)	65.9 (40.6 to 91.2)
		East Asia	Incidence	Age-standardized	7.6 (6.4 to 9.1)	1.3 (1.1 to 1.5)	13.6 (11.4 to 16.4)	6.6 (5.6 to 7.7)	0.9 (0.7 to 1)	12.3 (10.3 to 14.4)	-13.5 (-22.3 to -5.5)	-32.2 (-36.6 to -27.9)	-9.8 (-20 to -0.9)
		East Asia	Prevalence	All Ages	1,343,929 (1,095,621 to 1,690,162)	76,026 (62,804 to 91,198)	1,267,903 (1,025,568 to 1,608,220)	2,617,455 (2,180,162 to 3,115,778)	125,486 (104,600 to 151,253)	2,491,969 (2,066,124 to 2,989,835)	94.8 (69.5 to 118.6)	65.1 (53 to 78.2)	96.5 (69.6 to 122.3)
		East Asia	Prevalence	Age-standardized	139.3 (114.1 to 173.9)	17.1 (14.2 to 20.6)	264.2 (215.7 to 330.5)	120.4 (100.7 to 143)	11.8 (9.8 to 14.2)	235.4 (195.8 to 280.5)	-13.5 (-24.3 to -4.3)	-31.1 (-35.7 to -26.3)	-10.9 (-22.4 to -1)
		East Asia	Deaths	All Ages	11,367 (7,996 to 14,368)	618 (430 to 1,239)	10,749 (7,366 to 13,800)	10,708 (8,559 to 14,012)	595 (423 to 792)	10,113 (7,896 to 13,380)	-5.8 (-35 to 60.4)	-3.7 (-57.3 to 55.4)	-5.9 (-36.6 to 65.2)
		East Asia	Deaths	Age-standardized	1.4 (1 to 1.7)	0.1 (0.1 to 0.3)	2.9 (2 to 3.7)	0.5 (0.4 to 0.7)	0.1 (0 to 0.1)	1.2 (0.9 to 1.5)	-60 (-72 to -32.6)	-59.8 (-81.9 to -36.6)	-59.7 (-72.1 to -29.7)
		East Asia	DALYs	All Ages	514,134 (390,160 to 635,570)	28,226 (20,742 to 45,169)	485,908 (362,513 to 604,934)	624,719 (487,916 to 795,697)	30,292 (23,254 to 39,375)	594,427 (460,237 to 759,849)	21.5 (-4.5 to 64)	7.3 (-38.1 to 40.2)	22.3 (-5.2 to 68)
		East Asia	DALYs	Age-standardized	53.1 (40.6 to 65.6)	5.9 (4.4 to 9.3)	103.1 (78.2 to 127.9)	29.2 (22.9 to 37.1)	2.9 (2.2 to 3.8)	57.6 (45.1 to 73.2)	-44.9 (-56.6 to -26.1)	-51 (-71 to -37.1)	-44.2 (-56.6 to -24)
		East Asia	YLLs	All Ages	320,911 (218,347 to 411,270)	17,188 (11,579 to 34,714)	303,723 (204,672 to 394,526)	252,143 (197,759 to 329,611)	11,949 (8,490 to 16,052)	240,194 (185,734 to 318,477)	-21.4 (-46.4 to 35.3)	-30.5 (-69.1 to 14.9)	-20.9 (-47.4 to 40.2)
		East Asia	YLLs	Age-standardized	33.2 (22.8 to 42.3)	3.4 (2.4 to 7)	65.5 (44.7 to 84.3)	12.1 (9.6 to 15.9)	1.2 (0.8 to 1.6)	24.3 (18.9 to 32)	-63.5 (-75 to -37.5)	-65.8 (-84.9 to -44.3)	-63 (-75.2 to -35.2)
		East Asia	YLDs	All Ages	193,224 (126,675 to 282,402)	11,038 (7,396 to 15,880)	182,186 (118,376 to 267,469)	372,576 (246,648 to 535,056)	18,343 (12,024 to 26,244)	354,233 (233,813 to 509,326)	92.8 (67.6 to 116.6)	66.2 (51.1 to 82)	94.4 (67.5 to 120.1)
		East Asia	YLDs	Age-standardized	19.9 (13.1 to 28.7)	2.5 (1.7 to 3.5)	37.6 (24.7 to 54.2)	17.1 (11.3 to 24.5)	1.7 (1.1 to 2.5)	33.3 (22 to 47.9)	-14 (-24.4 to -4.4)	-30.1 (-36 to -23.9)	-11.4 (-22.5 to -0.9)
Southeast Asia, east Asia, and Oceania	East Asia	China	Incidence	All Ages	83,551 (69,733 to 101,410)	6,316 (5,316 to 7,436)	77,234 (64,092 to 94,837)	136,755 (113,659 to 162,518)	9,107 (7,520 to 11,114)	127,648 (105,380 to 151,758)	63.7 (40.4 to 86.7)	44.2 (32 to 55.8)	65.3 (40 to 90.7)
Southeast Asia, east Asia, and Oceania	East Asia	China	Incidence	Age-standardized	7.8 (6.6 to 9.4)	1.3 (1.1 to 1.6)	13.9 (11.6 to 16.8)	6.7 (5.7 to 7.9)	0.9 (0.7 to 1.1)	12.5 (10.5 to 14.7)	-13.9 (-22.8 to -6)	-32.6 (-37.1 to -28.4)	-10.2 (-20.3 to -1.2)
Southeast Asia, east Asia, and Oceania	East Asia	China	Prevalence	All Ages	1,330,860 (1,083,676 to 1,672,573)	75,749 (62,554 to 90,942)	1,255,111 (1,013,346 to 1,593,507)	2,587,176 (2,153,538 to 3,082,660)	124,652 (104,013 to 150,464)	2,462,525 (2,041,887 to 2,953,432)	94.4 (69.1 to 118.4)	64.6 (52.5 to 77.7)	96.2 (69.2 to 122.1)
Southeast Asia, east Asia, and Oceania	East Asia	China	Prevalence	Age-standardized	143.1 (117.1 to 178.6)	17.7 (14.7 to 21.3)	270.7 (220.7 to 339)	123.1 (103 to 146.4)	12.1 (10.2 to 14.7)	240.3 (199.8 to 286.7)	-14 (-24.7 to -4.7)	-31.4 (-36.1 to -26.6)	-11.3 (-22.7 to -1.2)
Southeast Asia, east Asia, and Oceania	East Asia	China	Deaths	All Ages	11,070 (7,744 to 14,056)	604 (422 to 1,223)	10,465 (7,107 to 13,483)	10,201 (8,050 to 13,567)	559 (383 to 754)	9,642 (7,442 to 12,995)	-7.8 (-38 to 60.1)	-7.5 (-60.6 to 49.9)	-7.9 (-39.4 to 64.9)
Southeast Asia, east Asia, and Oceania	East Asia	China	Deaths	Age-standardized	1.4 (1 to 1.7)	0.1 (0.1 to 0.3)	2.9 (2.2 to 3.7)	0.5 (0.4 to 0.7)	0.1 (0 to 0.1)	1.2 (0.9 to 1.6)	-61 (-73.3 to -33.1)	-61.3 (-83.2 to -38.5)	-60.6 (-73.5 to -29.5)
Southeast Asia, east Asia, and Oceania	East Asia	China	DALYs	All Ages	503,828 (380,498 to 624,469)	27,819 (20,444 to 44,418)	476,010 (355,425 to 594,783)	608,694 (473,558 to 779,381)	29,435 (22,555 to 38,505)	579,259 (448,047 to 744,310)	20.8 (-5.6 to 64)	5.8 (-39.7 to 38.4)	21.7 (-6.8 to 68.3)
Southeast Asia, east Asia, and Oceania	East Asia	China	DALYs	Age-standardized	54 (41.1 to 66.7)	6 (4.5 to 9.6)	104.6 (79.2 to 130.1)	29.5 (23 to 37.6)	2.9 (2.2 to 3.8)	57.9 (45 to 74)	-45.4 (-57.4 to -26.3)	-51.7 (-71.8 to -37.6)	-44.6 (-57.2 to -24)
Southeast Asia, east Asia, and Oceania	East Asia	China	YLLs	All Ages	312,499 (211,710 to 403,609)	16,825 (11,300 to 34,204)	295,674 (197,602 to 386,073)	240,450 (185,611 to 320,446)	11,224 (7,747 to 15,407)	229,227 (173,900 to 309,203)	-23.1 (-49.6 to 36.4)	-33.3 (-71.6 to 12.8)	-22.5 (-50.2 to 41.1)
Southeast Asia, east Asia, and Oceania	East Asia	China	YLLs	Age-standardized	33.6 (23 to 43)	3.5 (2.4 to 7.1)	66.1 (44.7 to 85.4)	12 (9.3 to 15.9)	1.2 (0.8 to 1.6)	23.9 (18.3 to 32.1)	-64.3 (-76.3 to -37.6)	-67.1 (-86 to -45)	-63.8 (-76.3 to -35.5)
Southeast Asia, east Asia, and Oceania	East Asia	China	YLDs	All Ages	191,329 (125,532 to 279,496)	10,994 (7,360 to 15,820)	180,335 (117,142 to 264,002)	368,243 (243,644 to 528,857)	18,211 (11,937 to 26,077)	350,032 (230,738 to 503,410)	92.5 (67 to 116.6)	65.6 (50.6 to 81.6)	94.1 (66.8 to 120)
Southeast Asia, east Asia, and Oceania	East Asia	China	YLDs	Age-standardized	20.4 (13.5 to 29.4)	2.5 (1.7 to 3.7)	38.5 (25.3 to 55.7)	17.5 (11.5 to 25.1)	1.8 (1.2 to 2.5)	34 (22.5 to 49)	-14.4 (-24.8 to -4.8)	-30.5 (-36.3 to -24.2)	-11.7 (-22.9 to -1.2)
Southeast Asia, east Asia, and Oceania	East Asia	Democratic People's Republic of Korea	Incidence	All Ages	472 (379 to 793)	17 (13 to 22)	454 (360 to 776)	1,322 (1,050 to 1,714)	34 (26 to 42)	1,288 (1,011 to 1,679)	180.4 (102.8 to 260)	92.7 (61.9 to 129.2)	183.7 (102.5 to 267.2)
Southeast Asia, east Asia, and Oceania	East Asia	Democratic People's Republic of Korea	Incidence	Age-standardized	2.4 (2 to 4)	0.2 (0.1 to 0.2)	5.4 (4.3 to 8.6)	3.9 (3.1 to 5.1)	0.2 (0.1 to 0.2)	7.9 (6.3 to 10.2)	60.2 (19 to 104.9)	7.2 (-6.6 to 23.7)	47.1 (10.2 to 85.7)
Southeast Asia, east Asia, and Oceania	East Asia	Democratic People's Republic of Korea	Prevalence	All Ages	6,928 (5,440 to 12,604)	188 (147 to 242)	6,740 (5,254 to 12,386)	20,522 (16,087 to 28,148)	409 (315 to 529)	20,113 (15,681 to 27,727)	196.2 (108.8 to 312)	117.7 (83.8 to 154.8)	198.4 (108.7 to 318.9)
Southeast Asia, east Asia, and Oceania	East Asia	Democratic People's Republic of Korea	Prevalence	Age-standardized	38.4 (30.5 to 67.6)	2 (1.6 to 2.5)	95.4 (75.2 to 166.1)	61.5 (48.3 to 83.9)	2.2 (1.7 to 2.8)	141.7 (111.3 to 191.6)	60.3 (15.1 to 119)	7 (-8.2 to 23.7)	48.6 (7.1 to 100.4)
Southeast Asia, east Asia, and Oceania	East Asia	Democratic People's Republic of Korea	Deaths	All Ages	166 (98 to 246)	12 (6 to 22)	154 (85 to 235)	274 (150 to 446)	22 (12 to 38)	253 (131 to 419)	65.5 (-2.7 to 183.1)	75.5 (-1.6 to 210.5)	64.7 (-7.1 to 188.6)
Southeast Asia, east Asia, and Oceania	East Asia	Democratic People's Republic of Korea	Deaths	Age-standardized	1 (0.6 to 1.5)	0.1 (0.1 to 0.3)	2.6 (1.4 to 3.9)	0.9 (0.5 to 1.4)	0.1 (0.1 to 0.2)	2 (1.1 to 3.4)	-16.6 (-50.4 to 39.8)	-14.9 (-50.8 to 49.3)	-23.1 (-55.8 to 34.9)
Southeast Asia, east Asia, and Oceania	East Asia	Democratic People's Republic of Korea	DALYs	All Ages	6,296 (3,931 to 9,143)	367 (200 to 617)	5,930 (3,581 to 8,758)	10,771 (6,802 to 16,310)	566 (343 to 941)	10,205 (6,337 to 15,727)	71.1 (7.9 to 173.8)	54.2 (-6.2 to 162.6)	72.1 (7.2 to 177.8)
Southeast Asia, east Asia, and Oceania	East Asia	Democratic People's Republic of Korea	DALYs	Age-standardized	34.1 (22 to 49)	3.7 (2 to 6.2)	79.4 (48.8 to 113.7)	32.5 (20.7 to 49)	3.2 (1.9 to 5.3)	70.2 (44.4 to 105.8)	-4.6 (-38.9 to 50.2)	-13.3 (-47.3 to 46.3)	-11.7 (-43.9 to 37.7)
Southeast Asia, east Asia, and Oceania	East Asia	Democratic People's Republic of Korea	YLLs	All Ages	5,280 (3,007 to 8,176)	337 (171 to 588)	4,943 (2,694 to 7,808)	7,822 (4,238 to 12,888)	501 (279 to 880)	7,321 (3,780 to 12,456)	48.2 (-17.5 to 165.6)	48.6 (-16.5 to 168.2)	48.1 (-19.2 to 170)
Southeast Asia, east Asia, and Oceania	East Asia	Democratic People's Republic of Korea	YLLs	Age-standardized	28.5 (16.8 to 42.5)	3.3 (1.7 to 5.9)	65.7 (37 to 99.8)	23.7 (13 to 38.9)	2.8 (1.6 to 4.9)	50.1 (26 to 82.7)	-16.8 (-52.3 to 46.8)	-15.3 (-51.7 to 52.9)	-23.8 (-56.7 to 34.9)
Southeast Asia, east Asia, and Oceania	East Asia	Democratic People's Republic of Korea	YLDs	All Ages	1,017 (613 to 2,005)	30 (19 to 44)	987 (593 to 1,975)	2,949 (1,819 to 4,606)	65 (40 to 98)	2,884 (1,779 to 4,522)	190 (103 to 305.3)	117.6 (83.9 to 154.4)	192.2 (102.4 to 312.4)
Southeast Asia, east Asia, and Oceania	East Asia	Democratic People's Republic of Korea	YLDs	Age-standardized	5.6 (3.4 to 10.8)	0.3 (0.2 to 0.5)	13.7 (8.6 to 26.1)	8.8 (5.5 to 13.7)	0.3 (0.2 to 0.5)	20.1 (12.7 to 31.1)	57.9 (13.2 to 119.6)	7 (-8.3 to 23.3)	46.6 (4.8 to 102.8)
Southeast Asia, east Asia, and Oceania	East Asia	Taiwan (Province of China)	Incidence	All Ages	460 (392 to 544)	9 (6 to 11)	451 (383 to 534)	750 (658 to 854)	39 (30 to 49)	711 (623 to 815)	63.2 (39.5 to 91.2)	352.3 (239.6 to 501.9)	57.7 (34 to 85)
Southeast Asia, east Asia, and Oceania	East Asia	Taiwan (Province of China)	Incidence	Age-standardized	2.6 (2.2 to 3)	0.1 (0.1 to 0.1)	4.7 (4 to 5.5)	1.9 (1.7 to 2.1)	0.2 (0.2 to 0.2)	3.8 (3.3 to 4.3)	-26.2 (-36.2 to -14.7)	87.1 (48.7 to 142.5)	-19.6 (-30.9 to -6.6)
Southeast Asia, east Asia, and Oceania	East Asia	Taiwan (Province of China)	Prevalence	All Ages	6,141 (5,164 to 7,288)	89 (68 to 116)	6,052 (5,083 to 7,196)	9,756 (8,614 to 11,037)	425 (342 to 524)	9,331 (8,185 to 10,578)	58.9 (35 to 85.4)	376.6 (281.7 to 500.5)	54.2 (30.7 to 81)
Southeast Asia, east Asia, and Oceania	East Asia	Taiwan (Province of China)	Prevalence	Age-standardized	38.1 (32.2 to 45)	1.2 (0.9 to 1.6)	71.7 (60.5 to 84.6)	24.7 (21.8 to 27.9)	2 (1.6 to 2.5)	51.1 (45 to 58.1)	-35.2 (-44.5 to -24.9)	65.4 (33.6 to 106.5)	-28.8 (-39.1 to -17.3)
Southeast Asia, east Asia, and Oceania	East Asia	Taiwan (Province of China)	Deaths	All Ages	131 (116 to 147)	1 (1 to 2)	130 (114 to 146)	232 (175 to 303)	14 (9 to 24)	218 (163 to 286)	76.8 (30.7 to 132.8)	1014.1 (600.8 to 2002.8)	67.6 (23.6 to 122.2)
Southeast Asia, east Asia, and Oceania	East Asia	Taiwan (Province of China)	Deaths	Age-standardized	0.9 (0.8 to 1)	0 (0 to 0)	1.7 (1.5 to 1.9)	0.6 (0.4 to 0.8)	0.1 (0 to 0.1)	1.2 (0.9 to 1.6)	-32.2 (-49.9 to -10.8)	214.7 (96 to 484.7)	-26.9 (-46.2 to -3.7)
Southeast Asia, east Asia, and Oceania	East Asia	Taiwan (Province of China)	DALYs	All Ages	4,010 (3,511 to 4,577)	40 (31 to 50)	3,969 (3,474 to 4,529)	5,255 (4,094 to 6,504)	291 (210 to 446)	4,963 (3,852 to 6,164)	31 (3.5 to 64.6)	619.5 (410.5 to 1093.7)	25 (-1.9 to 58.5)
Southeast Asia, east Asia, and Oceania	East Asia	Taiwan (Province of China)	DALYs	Age-standardized	24.2 (21.3 to 27.5)	0.6 (0.4 to 0.7)	45.4 (40 to 51.5)	13.4 (10.5 to 16.6)	1.4 (1 to 2.2)	27.6 (21.6 to 34.3)	-44.5 (-56 to -30.2)	150.3 (77.4 to 316.3)	-39.2 (-52 to -23)
Southeast Asia, east Asia, and Oceania	East Asia	Taiwan (Province of China)	YLLs	All Ages	3,132 (2,760 to 3,526)	26 (19 to 32)	3,106 (2,735 to 3,501)	3,870 (2,867 to 5,133)	224 (151 to 380)	3,646 (2,687 to 4,868)	23.6 (-9.2 to 66.4)	755.4 (437.9 to 1556.4)	17.4 (-14.2 to 59.2)
Southeast Asia, east Asia, and Oceania	East Asia	Taiwan (Province of China)	YLLs	Age-standardized	18.8 (16.6 to 21.1)	0.4 (0.3 to 0.4)	35.2 (31.2 to 39.3)	9.9 (7.4 to 13.2)	1.1 (0.7 to 1.8)	20.4 (15.1 to 27)	-47.2 (-61 to -29.1)	197.1 (88.2 to 475.5)	-42.2 (-57.7 to -22.2)
Southeast Asia, east Asia, and Oceania	East Asia	Taiwan (Province of China)	YLDs	All Ages	878 (563 to 1,292)	14 (9 to 22)	863 (553 to 1,273)	1,384 (925 to 1,956)	67 (43 to 99)	1,317 (885 to 1,870)	57.7 (28.4 to 98.5)	371.2 (282.4 to 489)	52.5 (23.5 to 93.5)
Southeast Asia, east Asia, and Oceania	East Asia	Taiwan (Province of China)	YLDs	Age-standardized	5.4 (3.5 to 7.9)	0.2 (0.1 to 0.3)	10.1 (6.6 to 14.7)	3.5 (2.3 to 5)	0.3 (0.2 to 0.5)	7.2 (4.8 to 10.1)	-35.2 (-46.9 to -19.9)	63.1 (32.9 to 102.5)	-29.1 (-42.2 to -12.1)
		Southeast Asia	Incidence	All Ages	1,396 (1,141 to 1,694)	439 (332 to 565)	958 (789 to 1,166)	3,012 (2,445 to 3,671)	679 (511 to 876)	2,333 (1,844 to 2,940)	115.7 (89.2 to 149.3)	54.8 (34.1 to 75.5)	143.6 (108.5 to 188.5)
		Southeast Asia	Incidence	Age-standardized	0.4 (0.3 to 0.5)	0.2 (0.2 to 0.3)	0.6 (0.5 to 0.7)	0.4 (0.4 to 0.5)	0.2 (0.1 to 0.2)	0.7 (0.6 to 0.9)	7 (-3.8 to 21.3)	-17.8 (-26.5 to -9.5)	17.2 (2.7 to 36.6)
		Southeast Asia	Prevalence	All Ages	12,352 (9,867 to 15,629)	2,451 (1,768 to 3,557)	9,901 (7,811 to 12,695)	36,460 (28,596 to 45,989)	4,118 (3,034 to 6,033)	32,342 (24,862 to 41,253)	195.2 (149.6 to 248)	68 (39.6 to 93.5)	226.7 (173.7 to 289)
		Southeast Asia	Prevalence	Age-standardized	4.2 (3.3 to 5.2)	1.5 (1.1 to 2.1)	7.3 (5.7 to 9.2)	5.6 (4.4 to 7)	1.2 (0.9 to 1.7)	10.7 (8.3 to 13.6)	33.1 (14.7 to 54.7)	-20.3 (-35 to -8.8)	47.2 (25.5 to 72.9)
		Southeast Asia	Deaths	All Ages	87 (64 to 106)	20 (12 to 26)	67 (46 to 85)	122 (101 to 154)	34 (23 to 42)	88 (70 to 121)	40.3 (8.6 to 110.7)	70.9 (22.4 to 137.6)	31.4 (-3.9 to 146.1)
		Southeast Asia	Deaths	Age-standardized	0 (0 to 0)	0 (0 to 0)	0.1 (0 to 0.1)	0 (0 to 0)	0 (0 to 0)	0 (0 to 0.1)	-42.5 (-55.3 to -14.1)	-30.8 (-51 to -2)	-45.8 (-60.5 to 1.9)
		Southeast Asia	DALYs	All Ages	4,101 (3,197 to 5,197)	863 (615 to 1,147)	3,237 (2,442 to 4,169)	8,334 (6,243 to 11,026)	1,365 (1,028 to 1,748)	6,969 (5,067 to 9,480)	103.2 (70.7 to 146.9)	58.1 (31.1 to 89.6)	115.3 (74.1 to 177.2)
		Southeast Asia	DALYs	Age-standardized	1.5 (1.1 to 1.8)	0.6 (0.4 to 0.7)	2.5 (1.9 to 3.2)	1.3 (1 to 1.7)	0.4 (0.3 to 0.5)	2.3 (1.7 to 3.1)	-12 (-26.3 to 8.1)	-27.8 (-40.9 to -13.2)	-7.7 (-25.7 to 20.9)
		Southeast Asia	YLLs	All Ages	2,165 (1,617 to 2,626)	482 (299 to 634)	1,683 (1,167 to 2,140)	2,825 (2,333 to 3,536)	722 (502 to 904)	2,102 (1,665 to 2,851)	30.4 (0.7 to 94.2)	49.8 (8.8 to 102.3)	24.9 (-10 to 124.4)
		Southeast Asia	YLLs	Age-standardized	0.8 (0.6 to 1)	0.3 (0.2 to 0.4)	1.4 (0.9 to 1.7)	0.5 (0.4 to 0.6)	0.2 (0.2 to 0.3)	0.7 (0.6 to 1)	-43.6 (-56.5 to -15)	-33.1 (-51.3 to -8.4)	-46.2 (-60.7 to -1.3)
		Southeast Asia	YLDs	All Ages	1,935 (1,221 to 2,883)	381 (219 to 619)	1,554 (982 to 2,322)	5,509 (3,491 to 8,133)	642 (390 to 1,045)	4,867 (3,081 to 7,351)	184.7 (141 to 235)	68.4 (38.8 to 95)	213.2 (160.9 to 276.5)
		Southeast Asia	YLDs	Age-standardized	0.7 (0.4 to 1)	0.2 (0.1 to 0.4)	1.1 (0.7 to 1.7)	0.8 (0.5 to 1.2)	0.2 (0.1 to 0.3)	1.6 (1 to 2.4)	27.1 (9.2 to 48.9)	-19.9 (-34.6 to -7.1)	38.9 (18 to 66.2)
Southeast Asia, east Asia, and Oceania	Southeast Asia	Cambodia	Incidence	All Ages	18 (13 to 23)	6 (3 to 8)	12 (9 to 16)	48 (36 to 60)	10 (5 to 14)	38 (29 to 49)	171.1 (120.4 to 234)	75.7 (20.7 to 143.7)	214.7 (151.7 to 290.4)
Southeast Asia, east Asia, and Oceania	Southeast Asia	Cambodia	Incidence	Age-standardized	0.3 (0.2 to 0.4)	0.2 (0.1 to 0.2)	0.5 (0.4 to 0.6)	0.3 (0.3 to 0.4)	0.1 (0.1 to 0.2)	0.6 (0.5 to 0.8)	15.1 (-3.2 to 36)	-18.9 (-40.2 to 7.1)	25.9 (4.8 to 51.6)
Southeast Asia, east Asia, and Oceania	Southeast Asia	Cambodia	Prevalence	All Ages	116 (86 to 153)	8 (6 to 12)	107 (78 to 144)	494 (363 to 652)	16 (11 to 22)	478 (348 to 634)	326 (246.1 to 433.6)	87.1 (44.9 to 138.8)	344.9 (256.9 to 465.6)
Southeast Asia, east Asia, and Oceania	Southeast Asia	Cambodia	Prevalence	Age-standardized	2.4 (1.7 to 3.1)	0.2 (0.2 to 0.3)	5.2 (3.8 to 6.9)	3.9 (2.9 to 5.1)	0.2 (0.1 to 0.3)	9 (6.6 to 11.9)	65.8 (35.8 to 106.4)	-15.4 (-31.2 to 4)	75 (42.7 to 119.8)
Southeast Asia, east Asia, and Oceania	Southeast Asia	Cambodia	Deaths	All Ages	2 (1 to 2)	0 (0 to 0)	1 (1 to 2)	3 (2 to 4)	1 (0 to 1)	2 (1 to 3)	84.1 (18.4 to 227.8)	182 (60.6 to 419.3)	63.5 (-2.3 to 224.1)
Southeast Asia, east Asia, and Oceania	Southeast Asia	Cambodia	Deaths	Age-standardized	0 (0 to 0.1)	0 (0 to 0)	0.1 (0 to 0.1)	0 (0 to 0)	0 (0 to 0)	0.1 (0 to 0.1)	-29.2 (-54.8 to 26.6)	4.9 (-39.4 to 87.2)	-33.2 (-60.6 to 36.3)
Southeast Asia, east Asia, and Oceania	Southeast Asia	Cambodia	DALYs	All Ages	57 (39 to 76)	8 (5 to 12)	49 (32 to 67)	137 (102 to 183)	18 (12 to 27)	119 (85 to 163)	140.9 (74.6 to 238.7)	124 (41 to 268.7)	143.7 (70.4 to 261.8)
Southeast Asia, east Asia, and Oceania	Southeast Asia	Cambodia	DALYs	Age-standardized	1.2 (0.8 to 1.6)	0.3 (0.2 to 0.4)	2.4 (1.6 to 3.3)	1.1 (0.8 to 1.5)	0.3 (0.2 to 0.4)	2.3 (1.7 to 3.1)	-7.1 (-32.7 to 32.5)	-7.5 (-41.4 to 54.5)	-5 (-33.6 to 42.4)
Southeast Asia, east Asia, and Oceania	Southeast Asia	Cambodia	YLLs	All Ages	39 (23 to 54)	7 (4 to 10)	32 (17 to 47)	63 (45 to 86)	16 (9 to 24)	47 (32 to 67)	62.2 (2.3 to 189.3)	131.1 (35.8 to 324.4)	47.4 (-13.4 to 188.5)
Southeast Asia, east Asia, and Oceania	Southeast Asia	Cambodia	YLLs	Age-standardized	0.8 (0.5 to 1.1)	0.3 (0.1 to 0.4)	1.6 (0.8 to 2.4)	0.5 (0.4 to 0.7)	0.2 (0.1 to 0.4)	1 (0.7 to 1.4)	-35.4 (-58.5 to 13.9)	-6.3 (-45 to 69.5)	-39.6 (-63.5 to 17.7)
Southeast Asia, east Asia, and Oceania	Southeast Asia	Cambodia	YLDs	All Ages	18 (11 to 28)	1 (1 to 2)	17 (10 to 26)	74 (45 to 115)	3 (1 to 4)	72 (43 to 111)	308 (210 to 427.9)	87.5 (45.4 to 139)	325.4 (221 to 454.4)
Southeast Asia, east Asia, and Oceania	Southeast Asia	Cambodia	YLDs	Age-standardized	0.4 (0.2 to 0.6)	0 (0 to 0.1)	0.8 (0.5 to 1.3)	0.6 (0.3 to 0.9)	0 (0 to 0.1)	1.3 (0.8 to 2.1)	56.6 (18.9 to 101.8)	-15.3 (-31 to 3.9)	63.9 (22.9 to 116.5)
Southeast Asia, east Asia, and Oceania	Southeast Asia	Indonesia	Incidence	All Ages	653 (514 to 832)	178 (130 to 244)	475 (371 to 605)	1,444 (1,178 to 1,776)	344 (259 to 453)	1,101 (876 to 1,374)	121.2 (91.9 to 155)	93.2 (61.8 to 128.7)	131.7 (93.9 to 175)
Southeast Asia, east Asia, and Oceania	Southeast Asia	Indonesia	Incidence	Age-standardized	0.5 (0.4 to 0.6)	0.2 (0.2 to 0.3)	0.7 (0.6 to 0.9)	0.6 (0.5 to 0.7)	0.3 (0.2 to 0.3)	0.9 (0.7 to 1.1)	14.8 (2.1 to 29.7)	7.8 (-10.5 to 27)	16.7 (0.5 to 35.8)
Southeast Asia, east Asia, and Oceania	Southeast Asia	Indonesia	Prevalence	All Ages	7,827 (5,933 to 10,751)	2,042 (1,382 to 3,139)	5,785 (4,370 to 7,841)	18,865 (15,028 to 24,300)	3,548 (2,511 to 5,395)	15,317 (12,063 to 19,612)	141 (98.5 to 184.9)	73.8 (40 to 103.5)	164.8 (109.9 to 221.1)
Southeast Asia, east Asia, and Oceania	Southeast Asia	Indonesia	Prevalence	Age-standardized	6.7 (5.1 to 8.9)	3.2 (2.2 to 4.9)	10.4 (8 to 13.7)	7.7 (6.2 to 9.8)	2.8 (2 to 4.2)	13.2 (10.4 to 16.7)	16.3 (-1.8 to 35.9)	-14 (-31.9 to 1.1)	27.3 (4.2 to 52.1)
Southeast Asia, east Asia, and Oceania	Southeast Asia	Indonesia	Deaths	All Ages	31 (24 to 37)	7 (5 to 10)	23 (17 to 30)	51 (39 to 70)	14 (10 to 19)	37 (26 to 56)	65 (21.2 to 155.4)	92.9 (24.5 to 196.5)	56.4 (4.3 to 205.4)
Southeast Asia, east Asia, and Oceania	Southeast Asia	Indonesia	Deaths	Age-standardized	0 (0 to 0)	0 (0 to 0)	0.1 (0 to 0.1)	0 (0 to 0)	0 (0 to 0)	0 (0 to 0.1)	-23.7 (-44 to 16.6)	-10.3 (-42.2 to 40.8)	-25.6 (-49.8 to 46.3)
Southeast Asia, east Asia, and Oceania	Southeast Asia	Indonesia	DALYs	All Ages	2,005 (1,503 to 2,708)	510 (349 to 741)	1,496 (1,114 to 2,040)	4,088 (2,940 to 5,505)	873 (633 to 1,247)	3,215 (2,267 to 4,388)	103.9 (73.4 to 142.4)	71.3 (39.5 to 107.8)	115 (74.8 to 171.9)
Southeast Asia, east Asia, and Oceania	Southeast Asia	Indonesia	DALYs	Age-standardized	1.8 (1.4 to 2.4)	0.8 (0.6 to 1.2)	2.9 (2.2 to 3.8)	1.7 (1.3 to 2.3)	0.7 (0.5 to 1)	2.9 (2.1 to 3.9)	-3.8 (-17.4 to 16)	-14.4 (-30.4 to 6)	0 (-17.9 to 29.4)
Southeast Asia, east Asia, and Oceania	Southeast Asia	Indonesia	YLLs	All Ages	782 (619 to 933)	193 (127 to 282)	589 (421 to 742)	1,197 (909 to 1,651)	320 (217 to 440)	877 (613 to 1,339)	53.2 (11.2 to 135)	66.2 (9 to 149.1)	48.9 (-3.6 to 184.1)
Southeast Asia, east Asia, and Oceania	Southeast Asia	Indonesia	YLLs	Age-standardized	0.8 (0.6 to 0.9)	0.3 (0.2 to 0.5)	1.2 (0.9 to 1.6)	0.6 (0.4 to 0.8)	0.3 (0.2 to 0.4)	0.9 (0.6 to 1.4)	-26.5 (-46.2 to 14.2)	-15.6 (-45.2 to 28.5)	-29 (-52.8 to 38.5)
Southeast Asia, east Asia, and Oceania	Southeast Asia	Indonesia	YLDs	All Ages	1,224 (744 to 1,935)	317 (175 to 530)	907 (557 to 1,432)	2,891 (1,832 to 4,275)	552 (316 to 922)	2,338 (1,448 to 3,429)	136.2 (95.2 to 181.9)	74.4 (38.4 to 107.1)	157.8 (105.9 to 215.1)
Southeast Asia, east Asia, and Oceania	Southeast Asia	Indonesia	YLDs	Age-standardized	1 (0.6 to 1.6)	0.5 (0.3 to 0.8)	1.6 (1 to 2.5)	1.2 (0.8 to 1.7)	0.4 (0.3 to 0.7)	2 (1.3 to 2.9)	13 (-4.7 to 32.9)	-13.6 (-31.9 to 2.3)	22.1 (0 to 46.8)
Southeast Asia, east Asia, and Oceania	Southeast Asia	Lao People's Democratic Republic	Incidence	All Ages	8 (6 to 10)	2 (1 to 3)	6 (4 to 7)	19 (14 to 24)	3 (2 to 5)	16 (12 to 21)	144.6 (95 to 209.6)	34.3 (-14.5 to 122.8)	186.6 (131.4 to 258)
Southeast Asia, east Asia, and Oceania	Southeast Asia	Lao People's Democratic Republic	Incidence	Age-standardized	0.3 (0.2 to 0.4)	0.1 (0.1 to 0.2)	0.5 (0.4 to 0.6)	0.3 (0.3 to 0.4)	0.1 (0.1 to 0.1)	0.6 (0.4 to 0.8)	13.7 (-6.7 to 40.4)	-34.3 (-56.8 to 0.8)	26.4 (4.6 to 53)
Southeast Asia, east Asia, and Oceania	Southeast Asia	Lao People's Democratic Republic	Prevalence	All Ages	58 (42 to 79)	4 (2 to 5)	54 (39 to 75)	211 (154 to 281)	5 (4 to 7)	206 (149 to 276)	264.7 (195.3 to 358.7)	50.8 (10.2 to 113.1)	278.6 (204.1 to 382.8)
Southeast Asia, east Asia, and Oceania	Southeast Asia	Lao People's Democratic Republic	Prevalence	Age-standardized	2.6 (1.9 to 3.5)	0.3 (0.2 to 0.3)	5.2 (3.8 to 7.1)	4.5 (3.3 to 6)	0.2 (0.1 to 0.2)	9.2 (6.7 to 12.3)	75.5 (43.6 to 121.1)	-25.5 (-43.1 to -1.4)	76.9 (44.2 to 125.2)
Southeast Asia, east Asia, and Oceania	Southeast Asia	Lao People's Democratic Republic	Deaths	All Ages	1 (1 to 2)	0 (0 to 0)	1 (1 to 1)	1 (1 to 2)	0 (0 to 1)	1 (1 to 1)	9.3 (-29.7 to 89.9)	49.6 (-19.4 to 197.9)	0.3 (-40.4 to 91.7)
Southeast Asia, east Asia, and Oceania	Southeast Asia	Lao People's Democratic Republic	Deaths	Age-standardized	0.1 (0 to 0.1)	0 (0 to 0)	0.1 (0.1 to 0.2)	0 (0 to 0.1)	0 (0 to 0)	0.1 (0 to 0.1)	-44 (-63.5 to -2.6)	-24.9 (-59.3 to 45.5)	-50.3 (-69.7 to -4.5)
Southeast Asia, east Asia, and Oceania	Southeast Asia	Lao People's Democratic Republic	DALYs	All Ages	42 (28 to 56)	7 (4 to 11)	35 (22 to 48)	64 (46 to 87)	9 (5 to 15)	55 (38 to 76)	53.8 (6.9 to 128)	34.8 (-22.4 to 155.8)	57.5 (5.4 to 145.3)
Southeast Asia, east Asia, and Oceania	Southeast Asia	Lao People's Democratic Republic	DALYs	Age-standardized	1.9 (1.3 to 2.5)	0.6 (0.3 to 0.9)	3.4 (2.1 to 4.7)	1.4 (1 to 1.9)	0.4 (0.2 to 0.6)	2.5 (1.7 to 3.4)	-26.1 (-48.4 to 10.8)	-32.2 (-61.3 to 28)	-26.9 (-51.6 to 14.1)
Southeast Asia, east Asia, and Oceania	Southeast Asia	Lao People's Democratic Republic	YLLs	All Ages	33 (20 to 46)	6 (3 to 10)	26 (14 to 40)	33 (22 to 47)	8 (5 to 14)	24 (15 to 36)	0.7 (-36.1 to 75.9)	33.4 (-27.1 to 170)	-7 (-44.9 to 74.7)
Southeast Asia, east Asia, and Oceania	Southeast Asia	Lao People's Democratic Republic	YLLs	Age-standardized	1.5 (0.9 to 2.1)	0.5 (0.3 to 0.8)	2.6 (1.4 to 3.8)	0.7 (0.5 to 1)	0.3 (0.2 to 0.6)	1.2 (0.7 to 1.7)	-50.3 (-68.1 to -14.6)	-32.8 (-63.5 to 35.9)	-55.1 (-72.8 to -16)
Southeast Asia, east Asia, and Oceania	Southeast Asia	Lao People's Democratic Republic	YLDs	All Ages	9 (5 to 14)	1 (0 to 1)	9 (5 to 13)	31 (18 to 49)	1 (0 to 1)	30 (17 to 48)	243.3 (150.4 to 373.5)	51 (10.8 to 112.6)	255.9 (156.1 to 400.7)
Southeast Asia, east Asia, and Oceania	Southeast Asia	Lao People's Democratic Republic	YLDs	Age-standardized	0.4 (0.2 to 0.6)	0 (0 to 0.1)	0.8 (0.5 to 1.3)	0.7 (0.4 to 1)	0 (0 to 0)	1.3 (0.8 to 2.1)	62.2 (16.5 to 127.3)	-25.4 (-42.6 to -1.4)	62.8 (16.2 to 129.5)
Southeast Asia, east Asia, and Oceania	Southeast Asia	Malaysia	Incidence	All Ages	25 (19 to 34)	4 (2 to 7)	21 (16 to 28)	99 (75 to 130)	9 (4 to 16)	90 (67 to 118)	289.7 (217.6 to 387.6)	121.9 (53.5 to 212)	323 (247.6 to 440.6)
Southeast Asia, east Asia, and Oceania	Southeast Asia	Malaysia	Incidence	Age-standardized	0.2 (0.2 to 0.3)	0.1 (0 to 0.1)	0.4 (0.3 to 0.5)	0.3 (0.2 to 0.4)	0.1 (0 to 0.1)	0.6 (0.4 to 0.8)	57.1 (32.4 to 89.6)	1.6 (-25.6 to 38.3)	62.4 (35.8 to 102)
Southeast Asia, east Asia, and Oceania	Southeast Asia	Malaysia	Prevalence	All Ages	256 (191 to 343)	8 (5 to 13)	248 (182 to 334)	1,337 (965 to 1,766)	20 (13 to 27)	1,318 (943 to 1,748)	421.7 (321.8 to 571.4)	132.7 (87.3 to 185.9)	431.6 (326.6 to 584.6)
Southeast Asia, east Asia, and Oceania	Southeast Asia	Malaysia	Prevalence	Age-standardized	2.6 (1.9 to 3.4)	0.1 (0.1 to 0.2)	5.1 (3.8 to 6.8)	4.8 (3.5 to 6.4)	0.1 (0.1 to 0.2)	9.6 (6.9 to 12.6)	90 (56.1 to 141.6)	0.5 (-16 to 19.1)	88 (54.2 to 139.1)
Southeast Asia, east Asia, and Oceania	Southeast Asia	Malaysia	Deaths	All Ages	1 (1 to 2)	0 (0 to 0)	1 (1 to 2)	2 (1 to 3)	0 (0 to 0)	2 (1 to 2)	33.9 (-19.6 to 143.4)	110.8 (10.8 to 288.4)	29.3 (-24.6 to 142.3)
Southeast Asia, east Asia, and Oceania	Southeast Asia	Malaysia	Deaths	Age-standardized	0 (0 to 0)	0 (0 to 0)	0 (0 to 0)	0 (0 to 0)	0 (0 to 0)	0 (0 to 0)	-55.5 (-73.1 to -19.9)	-21.3 (-57.4 to 42.4)	-58.8 (-75.8 to -23.3)
Southeast Asia, east Asia, and Oceania	Southeast Asia	Malaysia	DALYs	All Ages	71 (52 to 95)	3 (2 to 4)	68 (50 to 92)	235 (152 to 355)	6 (4 to 9)	229 (147 to 347)	231.3 (134.9 to 340.8)	105.1 (44 to 188.8)	236.9 (134.7 to 350.6)
Southeast Asia, east Asia, and Oceania	Southeast Asia	Malaysia	DALYs	Age-standardized	0.7 (0.6 to 1)	0.1 (0 to 0.1)	1.5 (1.1 to 2)	0.8 (0.6 to 1.3)	0 (0 to 0.1)	1.6 (1.1 to 2.5)	14 (-19.5 to 53.7)	-18.1 (-43.7 to 17)	12 (-22.1 to 53.2)
Southeast Asia, east Asia, and Oceania	Southeast Asia	Malaysia	YLLs	All Ages	31 (21 to 42)	2 (1 to 3)	29 (19 to 41)	40 (27 to 58)	3 (2 to 5)	37 (24 to 54)	30.3 (-22.5 to 140.4)	83.2 (-2.3 to 234.8)	27.3 (-26.5 to 142.7)
Southeast Asia, east Asia, and Oceania	Southeast Asia	Malaysia	YLLs	Age-standardized	0.3 (0.2 to 0.5)	0 (0 to 0.1)	0.7 (0.4 to 0.9)	0.1 (0.1 to 0.2)	0 (0 to 0)	0.3 (0.2 to 0.4)	-57 (-74.3 to -21)	-29.3 (-62.5 to 28.9)	-59.7 (-76.7 to -22.2)
Southeast Asia, east Asia, and Oceania	Southeast Asia	Malaysia	YLDs	All Ages	40 (24 to 62)	1 (1 to 2)	39 (23 to 61)	195 (116 to 314)	3 (2 to 5)	192 (113 to 312)	384.1 (252 to 556.3)	132.7 (87.5 to 185.8)	392.7 (255.6 to 572.6)
Southeast Asia, east Asia, and Oceania	Southeast Asia	Malaysia	YLDs	Age-standardized	0.4 (0.2 to 0.6)	0 (0 to 0)	0.8 (0.5 to 1.2)	0.7 (0.4 to 1.1)	0 (0 to 0)	1.4 (0.8 to 2.2)	74.4 (28.2 to 136.2)	0.5 (-15.9 to 18.9)	72.1 (25.1 to 135)
Southeast Asia, east Asia, and Oceania	Southeast Asia	Maldives	Incidence	All Ages	1 (1 to 2)	1 (0 to 1)	1 (1 to 1)	2 (1 to 3)	0 (0 to 0)	2 (1 to 2)	47.6 (5 to 105.1)	-51.2 (-71.7 to -21.7)	110.3 (53.8 to 187)
Southeast Asia, east Asia, and Oceania	Southeast Asia	Maldives	Incidence	Age-standardized	0.9 (0.7 to 1.2)	0.8 (0.5 to 1)	1.1 (0.9 to 1.4)	0.4 (0.3 to 0.5)	0.1 (0.1 to 0.2)	0.6 (0.5 to 0.8)	-56.2 (-66.7 to -42.2)	-82.1 (-88.7 to -73.9)	-44.1 (-57.7 to -26.5)
Southeast Asia, east Asia, and Oceania	Southeast Asia	Maldives	Prevalence	All Ages	4 (3 to 6)	1 (1 to 1)	4 (3 to 5)	19 (14 to 25)	0 (0 to 1)	18 (14 to 25)	318.7 (224.6 to 429.5)	-49.5 (-66.7 to -23.9)	395.4 (292 to 524.5)
Southeast Asia, east Asia, and Oceania	Southeast Asia	Maldives	Prevalence	Age-standardized	4.2 (3.3 to 5.4)	1.2 (0.9 to 1.6)	6.3 (4.8 to 8.4)	5.4 (4 to 7.1)	0.2 (0.1 to 0.3)	9.6 (7 to 12.8)	29.2 (3.8 to 58.2)	-82 (-87.2 to -74.9)	53.7 (24.4 to 89)
Southeast Asia, east Asia, and Oceania	Southeast Asia	Maldives	Deaths	All Ages	0 (0 to 0)	0 (0 to 0)	0 (0 to 0)	0 (0 to 0)	0 (0 to 0)	0 (0 to 0)	-48.1 (-71.3 to -1.3)	95.7 (3.6 to 294.8)	-50.5 (-73.1 to -4.3)
Southeast Asia, east Asia, and Oceania	Southeast Asia	Maldives	Deaths	Age-standardized	0.1 (0.1 to 0.1)	0 (0 to 0)	0.1 (0.1 to 0.2)	0 (0 to 0)	0 (0 to 0)	0 (0 to 0)	-84.3 (-91 to -71.8)	-55.5 (-76.7 to -8.5)	-84.7 (-91.4 to -71.5)
Southeast Asia, east Asia, and Oceania	Southeast Asia	Maldives	DALYs	All Ages	4 (2 to 5)	0 (0 to 0)	4 (2 to 5)	4 (3 to 6)	0 (0 to 0)	4 (3 to 6)	16.2 (-29.7 to 86.7)	-26.3 (-47.4 to 7.8)	18.2 (-30.3 to 96.9)
Southeast Asia, east Asia, and Oceania	Southeast Asia	Maldives	DALYs	Age-standardized	3.2 (2.1 to 4.6)	0.3 (0.2 to 0.4)	5.5 (3.5 to 8)	1.2 (0.8 to 1.6)	0.1 (0.1 to 0.1)	2 (1.4 to 2.9)	-64.1 (-77.8 to -43.1)	-74.5 (-82.1 to -63.5)	-63.2 (-78 to -40.3)
Southeast Asia, east Asia, and Oceania	Southeast Asia	Maldives	YLLs	All Ages	3 (2 to 5)	0 (0 to 0)	3 (2 to 5)	2 (1 to 2)	0 (0 to 0)	1 (1 to 2)	-50 (-73.4 to -3.1)	37.4 (-27.2 to 171.1)	-51.3 (-74.4 to -3.4)
Southeast Asia, east Asia, and Oceania	Southeast Asia	Maldives	YLLs	Age-standardized	2.6 (1.5 to 4)	0.1 (0.1 to 0.2)	4.5 (2.6 to 7)	0.4 (0.3 to 0.5)	0 (0 to 0.1)	0.6 (0.4 to 0.9)	-85.5 (-92.1 to -72.7)	-61.4 (-79 to -25.5)	-86.2 (-92.5 to -73.5)
Southeast Asia, east Asia, and Oceania	Southeast Asia	Maldives	YLDs	All Ages	1 (0 to 1)	0 (0 to 0)	1 (0 to 1)	3 (2 to 4)	0 (0 to 0)	3 (2 to 4)	299.2 (186.5 to 436.7)	-49.4 (-66.6 to -23.8)	371.6 (245.8 to 527.9)
Southeast Asia, east Asia, and Oceania	Southeast Asia	Maldives	YLDs	Age-standardized	0.7 (0.4 to 1)	0.2 (0.1 to 0.3)	1 (0.6 to 1.5)	0.8 (0.4 to 1.2)	0 (0 to 0.1)	1.4 (0.8 to 2.2)	20.4 (-13.7 to 62.6)	-82 (-87.2 to -74.8)	42.5 (1.3 to 93.8)
Southeast Asia, east Asia, and Oceania	Southeast Asia	Mauritius	Incidence	All Ages	2 (1 to 2)	0 (0 to 1)	1 (1 to 2)	5 (4 to 7)	0 (0 to 1)	5 (3 to 6)	198.5 (129.5 to 279.6)	27.4 (-16.8 to 97.2)	233.5 (159.4 to 323.1)
Southeast Asia, east Asia, and Oceania	Southeast Asia	Mauritius	Incidence	Age-standardized	0.2 (0.1 to 0.2)	0.1 (0.1 to 0.1)	0.3 (0.3 to 0.4)	0.3 (0.2 to 0.4)	0 (0 to 0.1)	0.6 (0.4 to 0.7)	60.4 (30.7 to 98.4)	-18.3 (-43.8 to 17.1)	72.9 (42.1 to 112.3)
Southeast Asia, east Asia, and Oceania	Southeast Asia	Mauritius	Prevalence	All Ages	18 (14 to 24)	1 (0 to 1)	18 (13 to 23)	79 (56 to 106)	1 (1 to 1)	78 (55 to 105)	331.6 (232.3 to 440.2)	52.1 (17 to 100.7)	340.4 (237.1 to 455.8)
Southeast Asia, east Asia, and Oceania	Southeast Asia	Mauritius	Prevalence	Age-standardized	2.3 (1.7 to 3)	0.1 (0.1 to 0.2)	4.9 (3.7 to 6.5)	4.4 (3.2 to 5.9)	0.1 (0.1 to 0.1)	9.5 (6.8 to 12.7)	91.6 (51.2 to 138.4)	-12.8 (-29.8 to 7)	91.5 (52.1 to 139.1)
Southeast Asia, east Asia, and Oceania	Southeast Asia	Mauritius	Deaths	All Ages	0 (0 to 0)	0 (0 to 0)	0 (0 to 0)	0 (0 to 0)	0 (0 to 0)	0 (0 to 0)	-44.5 (-58.4 to -24.9)	45.9 (-7.7 to 168)	-56.9 (-67.9 to -41.3)
Southeast Asia, east Asia, and Oceania	Southeast Asia	Mauritius	Deaths	Age-standardized	0 (0 to 0)	0 (0 to 0)	0 (0 to 0.1)	0 (0 to 0)	0 (0 to 0)	0 (0 to 0)	-78.5 (-83.7 to -71.1)	-45.2 (-65.7 to -0.2)	-84.4 (-88 to -79.5)
Southeast Asia, east Asia, and Oceania	Southeast Asia	Mauritius	DALYs	All Ages	6 (5 to 8)	1 (0 to 1)	5 (4 to 7)	13 (8 to 20)	0 (0 to 1)	12 (7 to 20)	117.7 (50.6 to 195.6)	-6.5 (-34.6 to 51.5)	130 (57 to 215.7)
Southeast Asia, east Asia, and Oceania	Southeast Asia	Mauritius	DALYs	Age-standardized	0.8 (0.6 to 1)	0.1 (0.1 to 0.1)	1.6 (1.3 to 2.1)	0.7 (0.4 to 1.1)	0.1 (0 to 0.1)	1.5 (0.9 to 2.4)	-7.3 (-34.7 to 24.6)	-47.9 (-63.3 to -16.2)	-8.4 (-36.6 to 24.7)
Southeast Asia, east Asia, and Oceania	Southeast Asia	Mauritius	YLLs	All Ages	3 (3 to 3)	0 (0 to 1)	3 (2 to 3)	1 (1 to 2)	0 (0 to 1)	1 (1 to 1)	-52.1 (-65 to -33.8)	-18.1 (-48.9 to 50.1)	-57.9 (-69.6 to -41)
Southeast Asia, east Asia, and Oceania	Southeast Asia	Mauritius	YLLs	Age-standardized	0.4 (0.4 to 0.5)	0.1 (0.1 to 0.1)	0.9 (0.7 to 1)	0.1 (0.1 to 0.1)	0 (0 to 0.1)	0.1 (0.1 to 0.2)	-79.4 (-84.8 to -71.7)	-54.7 (-71.2 to -17.3)	-83.9 (-88.1 to -77.8)
Southeast Asia, east Asia, and Oceania	Southeast Asia	Mauritius	YLDs	All Ages	3 (2 to 4)	0 (0 to 0)	3 (2 to 4)	11 (6 to 19)	0 (0 to 0)	11 (6 to 19)	298.5 (181.5 to 447.2)	52.2 (17.4 to 100.7)	306.4 (184.6 to 459.8)
Southeast Asia, east Asia, and Oceania	Southeast Asia	Mauritius	YLDs	Age-standardized	0.4 (0.2 to 0.6)	0 (0 to 0)	0.8 (0.5 to 1.2)	0.6 (0.4 to 1)	0 (0 to 0)	1.4 (0.8 to 2.2)	76.5 (28.1 to 137.5)	-12.8 (-29.7 to 6.8)	75.2 (27.2 to 135.6)
Southeast Asia, east Asia, and Oceania	Southeast Asia	Myanmar	Incidence	All Ages	229 (171 to 299)	97 (67 to 136)	132 (100 to 170)	280 (218 to 347)	99 (67 to 136)	180 (141 to 226)	21.8 (-2.2 to 55.7)	2.2 (-25.4 to 39.1)	36.3 (4.6 to 75.9)
Southeast Asia, east Asia, and Oceania	Southeast Asia	Myanmar	Incidence	Age-standardized	0.7 (0.6 to 0.9)	0.6 (0.4 to 0.8)	0.9 (0.7 to 1.1)	0.5 (0.4 to 0.6)	0.3 (0.2 to 0.5)	0.7 (0.6 to 0.9)	-27.9 (-40.3 to -11.3)	-40.9 (-56 to -22.8)	-16 (-32.4 to 5.4)
Southeast Asia, east Asia, and Oceania	Southeast Asia	Myanmar	Prevalence	All Ages	837 (659 to 1,066)	148 (102 to 201)	689 (531 to 903)	2,124 (1,614 to 2,747)	153 (112 to 202)	1,971 (1,457 to 2,615)	153.7 (104.6 to 216.1)	3.4 (-19.9 to 36.3)	186.1 (130.7 to 260.6)
Southeast Asia, east Asia, and Oceania	Southeast Asia	Myanmar	Prevalence	Age-standardized	3.1 (2.5 to 4)	0.9 (0.7 to 1.2)	5.7 (4.4 to 7.5)	4.3 (3.3 to 5.6)	0.5 (0.4 to 0.7)	9.2 (6.9 to 12.1)	37.4 (12.9 to 68.9)	-41.4 (-53.7 to -25.7)	62.3 (31.6 to 103.2)
Southeast Asia, east Asia, and Oceania	Southeast Asia	Myanmar	Deaths	All Ages	18 (11 to 26)	4 (1 to 6)	14 (8 to 22)	21 (15 to 28)	6 (2 to 9)	15 (10 to 21)	15.9 (-26.2 to 84.9)	55.9 (-11 to 180.7)	5.3 (-37.4 to 91.6)
Southeast Asia, east Asia, and Oceania	Southeast Asia	Myanmar	Deaths	Age-standardized	0.1 (0.1 to 0.1)	0 (0 to 0.1)	0.2 (0.1 to 0.2)	0.1 (0 to 0.1)	0 (0 to 0)	0.1 (0.1 to 0.1)	-41.2 (-61.5 to -5.4)	-26 (-57.5 to 32.7)	-42.4 (-65.5 to 5.6)
Southeast Asia, east Asia, and Oceania	Southeast Asia	Myanmar	DALYs	All Ages	594 (405 to 821)	122 (61 to 185)	473 (313 to 669)	778 (570 to 1,038)	147 (77 to 215)	631 (463 to 864)	31 (-10.8 to 88.7)	20.7 (-24.8 to 95.9)	33.6 (-13.4 to 110.4)
Southeast Asia, east Asia, and Oceania	Southeast Asia	Myanmar	DALYs	Age-standardized	2.4 (1.6 to 3.3)	0.9 (0.4 to 1.4)	4.2 (2.8 to 5.9)	1.7 (1.2 to 2.2)	0.6 (0.3 to 0.8)	3.1 (2.3 to 4.2)	-31.6 (-52.4 to -0.9)	-36.2 (-60.2 to 3.4)	-26.9 (-51.4 to 13.7)
Southeast Asia, east Asia, and Oceania	Southeast Asia	Myanmar	YLLs	All Ages	462 (291 to 681)	98 (36 to 161)	364 (215 to 551)	458 (317 to 636)	123 (55 to 191)	335 (225 to 476)	-1 (-38.4 to 63.2)	24.8 (-30 to 126.9)	-7.9 (-46.8 to 73.1)
Southeast Asia, east Asia, and Oceania	Southeast Asia	Myanmar	YLLs	Age-standardized	1.9 (1.2 to 2.8)	0.7 (0.3 to 1.2)	3.3 (2 to 5)	1 (0.7 to 1.4)	0.5 (0.2 to 0.7)	1.7 (1.2 to 2.4)	-47.5 (-66.6 to -16.5)	-35.2 (-62.8 to 17.2)	-48.2 (-69.2 to -6.6)
Southeast Asia, east Asia, and Oceania	Southeast Asia	Myanmar	YLDs	All Ages	132 (81 to 197)	23 (14 to 37)	108 (67 to 164)	320 (193 to 495)	24 (15 to 38)	296 (173 to 466)	143 (84 to 221.2)	3.6 (-19.5 to 36.6)	173.1 (105.4 to 268.4)
Southeast Asia, east Asia, and Oceania	Southeast Asia	Myanmar	YLDs	Age-standardized	0.5 (0.3 to 0.7)	0.1 (0.1 to 0.2)	0.9 (0.6 to 1.4)	0.6 (0.4 to 1)	0.1 (0.1 to 0.1)	1.4 (0.8 to 2.2)	29.9 (-1.1 to 73.6)	-41.3 (-53.5 to -25.7)	52.2 (13.5 to 106.6)
Southeast Asia, east Asia, and Oceania	Southeast Asia	Philippines	Incidence	All Ages	104 (80 to 130)	38 (24 to 55)	66 (51 to 83)	326 (257 to 409)	90 (60 to 125)	236 (180 to 308)	214 (175.1 to 261.8)	135.2 (105.5 to 176.2)	260 (209.1 to 325.3)
Southeast Asia, east Asia, and Oceania	Southeast Asia	Philippines	Incidence	Age-standardized	0.2 (0.2 to 0.3)	0.2 (0.1 to 0.2)	0.3 (0.3 to 0.4)	0.3 (0.3 to 0.4)	0.2 (0.1 to 0.3)	0.5 (0.4 to 0.7)	37.2 (23 to 56)	8.2 (-2.5 to 23.7)	53.6 (34.3 to 79.3)
Southeast Asia, east Asia, and Oceania	Southeast Asia	Philippines	Prevalence	All Ages	830 (641 to 1,074)	59 (42 to 80)	771 (587 to 1,013)	3,170 (2,373 to 4,217)	142 (104 to 185)	3,028 (2,227 to 4,082)	281.8 (226.9 to 358.4)	139.9 (115.5 to 172.3)	292.7 (234.4 to 375.1)
Southeast Asia, east Asia, and Oceania	Southeast Asia	Philippines	Prevalence	Age-standardized	2.5 (1.9 to 3.3)	0.3 (0.2 to 0.4)	4.9 (3.7 to 6.5)	3.7 (2.8 to 5)	0.3 (0.2 to 0.4)	7.7 (5.8 to 10.4)	49.4 (29.4 to 77.5)	5 (-3.3 to 17.2)	58.8 (36.3 to 88.8)
Southeast Asia, east Asia, and Oceania	Southeast Asia	Philippines	Deaths	All Ages	11 (9 to 12)	4 (3 to 5)	7 (5 to 8)	14 (12 to 18)	5 (4 to 8)	9 (7 to 12)	35.5 (4.9 to 90.1)	40.8 (-1.6 to 110.5)	32.6 (-3.2 to 114.3)
Southeast Asia, east Asia, and Oceania	Southeast Asia	Philippines	Deaths	Age-standardized	0 (0 to 0.1)	0 (0 to 0)	0.1 (0.1 to 0.1)	0 (0 to 0)	0 (0 to 0)	0 (0 to 0)	-55.4 (-64.7 to -37.6)	-54.2 (-67.6 to -31.5)	-52.9 (-64.8 to -25.7)
Southeast Asia, east Asia, and Oceania	Southeast Asia	Philippines	DALYs	All Ages	390 (322 to 469)	103 (82 to 133)	286 (223 to 355)	835 (646 to 1,100)	153 (116 to 203)	682 (498 to 937)	114.4 (77.6 to 159.4)	47.8 (6.6 to 104.2)	138.4 (92.9 to 201)
Southeast Asia, east Asia, and Oceania	Southeast Asia	Philippines	DALYs	Age-standardized	1.3 (1 to 1.5)	0.6 (0.5 to 0.8)	1.9 (1.5 to 2.3)	1 (0.8 to 1.3)	0.3 (0.3 to 0.5)	1.8 (1.3 to 2.4)	-20.3 (-34 to -2.1)	-45.6 (-60.9 to -24.2)	-7.6 (-24.9 to 18.4)
Southeast Asia, east Asia, and Oceania	Southeast Asia	Philippines	YLLs	All Ages	259 (218 to 303)	94 (74 to 123)	165 (129 to 194)	351 (281 to 435)	130 (96 to 179)	221 (166 to 295)	35.5 (4.5 to 83.8)	38.6 (-5 to 100.1)	33.8 (-3.5 to 101.1)
Southeast Asia, east Asia, and Oceania	Southeast Asia	Philippines	YLLs	Age-standardized	0.9 (0.7 to 1)	0.6 (0.5 to 0.8)	1.1 (0.8 to 1.3)	0.4 (0.4 to 0.6)	0.3 (0.2 to 0.4)	0.6 (0.5 to 0.8)	-49.3 (-60.7 to -30.1)	-49.4 (-65 to -25.9)	-47 (-61.3 to -15.5)
Southeast Asia, east Asia, and Oceania	Southeast Asia	Philippines	YLDs	All Ages	131 (81 to 199)	9 (5 to 15)	121 (74 to 187)	484 (298 to 742)	22 (14 to 35)	462 (283 to 710)	270.8 (217.1 to 344.2)	139.9 (115.7 to 172.2)	280.9 (222.8 to 361.3)
Southeast Asia, east Asia, and Oceania	Southeast Asia	Philippines	YLDs	Age-standardized	0.4 (0.2 to 0.6)	0 (0 to 0.1)	0.8 (0.5 to 1.2)	0.6 (0.4 to 0.9)	0 (0 to 0.1)	1.2 (0.7 to 1.8)	43.1 (22.9 to 70.2)	4.8 (-3.4 to 16.9)	50.9 (28.8 to 81.3)
Southeast Asia, east Asia, and Oceania	Southeast Asia	Seychelles	Incidence	All Ages	0 (0 to 0)	0 (0 to 0)	0 (0 to 0)	0 (0 to 1)	0 (0 to 0)	0 (0 to 0)	182 (115.6 to 259.5)	19.3 (-22.2 to 72.6)	232.6 (157.9 to 326.1)
Southeast Asia, east Asia, and Oceania	Southeast Asia	Seychelles	Incidence	Age-standardized	0.2 (0.2 to 0.3)	0.1 (0.1 to 0.2)	0.4 (0.3 to 0.5)	0.3 (0.3 to 0.4)	0.1 (0 to 0.1)	0.6 (0.4 to 0.7)	37.5 (11 to 70.3)	-28.1 (-51 to 1.6)	47.1 (19.4 to 84.2)
Southeast Asia, east Asia, and Oceania	Southeast Asia	Seychelles	Prevalence	All Ages	1 (1 to 2)	0 (0 to 0)	1 (1 to 2)	5 (4 to 7)	0 (0 to 0)	5 (4 to 7)	295.5 (211.2 to 405.4)	33.3 (-0.2 to 70.1)	307.3 (221.1 to 425.6)
Southeast Asia, east Asia, and Oceania	Southeast Asia	Seychelles	Prevalence	Age-standardized	2.4 (1.8 to 3.1)	0.2 (0.1 to 0.2)	5.1 (3.7 to 6.7)	4.5 (3.2 to 6)	0.1 (0.1 to 0.2)	9.3 (6.7 to 12.4)	90.7 (52.5 to 144.1)	-21.4 (-39.7 to -1.2)	83.6 (46.6 to 133.9)
Southeast Asia, east Asia, and Oceania	Southeast Asia	Seychelles	Deaths	All Ages	0 (0 to 0)	0 (0 to 0)	0 (0 to 0)	0 (0 to 0)	0 (0 to 0)	0 (0 to 0)	17.9 (-15.8 to 73.4)	66.8 (0.1 to 175.9)	3.3 (-35.3 to 63.7)
Southeast Asia, east Asia, and Oceania	Southeast Asia	Seychelles	Deaths	Age-standardized	0 (0 to 0)	0 (0 to 0)	0 (0 to 0.1)	0 (0 to 0)	0 (0 to 0)	0 (0 to 0.1)	-32 (-51.9 to -0.5)	5.3 (-36.9 to 74.2)	-45.1 (-65.9 to -9)
Southeast Asia, east Asia, and Oceania	Southeast Asia	Seychelles	DALYs	All Ages	0 (0 to 1)	0 (0 to 0)	0 (0 to 1)	1 (1 to 2)	0 (0 to 0)	1 (1 to 2)	131.3 (72.6 to 204.1)	57.7 (2.5 to 142.5)	142.9 (75 to 231.1)
Southeast Asia, east Asia, and Oceania	Southeast Asia	Seychelles	DALYs	Age-standardized	0.8 (0.6 to 1.2)	0.2 (0.1 to 0.3)	1.7 (1.2 to 2.7)	0.9 (0.6 to 1.4)	0.2 (0.1 to 0.3)	1.8 (1.1 to 2.8)	13.6 (-15.1 to 48)	-5.6 (-38.1 to 44.4)	7.7 (-21.4 to 44.6)
Southeast Asia, east Asia, and Oceania	Southeast Asia	Seychelles	YLLs	All Ages	0 (0 to 0)	0 (0 to 0)	0 (0 to 0)	0 (0 to 1)	0 (0 to 0)	0 (0 to 1)	21.4 (-15.3 to 75.8)	61.8 (-1.3 to 168.5)	10.6 (-29.8 to 72)
Southeast Asia, east Asia, and Oceania	Southeast Asia	Seychelles	YLLs	Age-standardized	0.5 (0.3 to 0.8)	0.2 (0.1 to 0.3)	0.9 (0.5 to 1.8)	0.3 (0.2 to 0.7)	0.2 (0.1 to 0.3)	0.4 (0.2 to 1.2)	-36.4 (-55.5 to -9.5)	-3 (-39.4 to 58.6)	-47.6 (-67.5 to -16.7)
Southeast Asia, east Asia, and Oceania	Southeast Asia	Seychelles	YLDs	All Ages	0 (0 to 0)	0 (0 to 0)	0 (0 to 0)	1 (0 to 1)	0 (0 to 0)	1 (0 to 1)	268.8 (164.2 to 411.7)	33.2 (0.1 to 70)	279.5 (170.5 to 428.1)
Southeast Asia, east Asia, and Oceania	Southeast Asia	Seychelles	YLDs	Age-standardized	0.4 (0.2 to 0.6)	0 (0 to 0)	0.8 (0.5 to 1.2)	0.7 (0.4 to 1.1)	0 (0 to 0)	1.3 (0.8 to 2.2)	75.5 (27.3 to 145.3)	-21.4 (-39.5 to -1.3)	67.4 (20.8 to 133.6)
Southeast Asia, east Asia, and Oceania	Southeast Asia	Sri Lanka	Incidence	All Ages	59 (44 to 76)	20 (13 to 29)	39 (30 to 50)	108 (84 to 135)	28 (19 to 39)	80 (60 to 104)	84.3 (46 to 128.2)	42.8 (-1.5 to 93.4)	105.4 (60.3 to 162.5)
Southeast Asia, east Asia, and Oceania	Southeast Asia	Sri Lanka	Incidence	Age-standardized	0.4 (0.3 to 0.5)	0.3 (0.2 to 0.4)	0.6 (0.4 to 0.7)	0.4 (0.3 to 0.5)	0.2 (0.1 to 0.3)	0.7 (0.5 to 0.9)	3.6 (-14.4 to 23.9)	-20.3 (-43.9 to 2.4)	21.2 (-3.3 to 49.6)
Southeast Asia, east Asia, and Oceania	Southeast Asia	Sri Lanka	Prevalence	All Ages	346 (264 to 454)	30 (21 to 41)	316 (233 to 422)	1,171 (850 to 1,567)	45 (33 to 59)	1,126 (802 to 1,525)	238.4 (167.8 to 323.7)	51.6 (12.7 to 93.4)	256 (181.1 to 350.4)
Southeast Asia, east Asia, and Oceania	Southeast Asia	Sri Lanka	Prevalence	Age-standardized	2.9 (2.2 to 3.8)	0.4 (0.3 to 0.6)	5.4 (4 to 7.2)	4.5 (3.3 to 6)	0.3 (0.2 to 0.4)	9.7 (7 to 13)	54.1 (23.2 to 90.5)	-18.6 (-37.9 to 1.1)	79 (42.2 to 125)
Southeast Asia, east Asia, and Oceania	Southeast Asia	Sri Lanka	Deaths	All Ages	1 (1 to 1)	0 (0 to 0)	1 (0 to 1)	1 (1 to 2)	0 (0 to 0)	1 (1 to 1)	32.5 (-19.7 to 130.7)	32.3 (-27.3 to 140.6)	32.6 (-26.6 to 163.2)
Southeast Asia, east Asia, and Oceania	Southeast Asia	Sri Lanka	Deaths	Age-standardized	0 (0 to 0)	0 (0 to 0)	0 (0 to 0)	0 (0 to 0)	0 (0 to 0)	0 (0 to 0)	-45.5 (-66.5 to -4)	-47.9 (-71.7 to -7)	-38.5 (-65.3 to 25.5)
Southeast Asia, east Asia, and Oceania	Southeast Asia	Sri Lanka	DALYs	All Ages	76 (55 to 107)	11 (7 to 15)	66 (45 to 94)	194 (121 to 288)	12 (8 to 16)	182 (111 to 275)	154.1 (89.7 to 228)	12 (-22 to 55.2)	176.9 (103.7 to 261.3)
Southeast Asia, east Asia, and Oceania	Southeast Asia	Sri Lanka	DALYs	Age-standardized	0.7 (0.5 to 0.9)	0.2 (0.1 to 0.2)	1.1 (0.8 to 1.6)	0.7 (0.5 to 1.1)	0.1 (0.1 to 0.1)	1.6 (1 to 2.3)	14.4 (-12.9 to 44.8)	-42.7 (-58.5 to -21.5)	36.1 (1.2 to 75.7)
Southeast Asia, east Asia, and Oceania	Southeast Asia	Sri Lanka	YLLs	All Ages	22 (16 to 29)	6 (4 to 10)	16 (11 to 22)	25 (16 to 36)	5 (3 to 7)	20 (12 to 31)	11.6 (-32.7 to 95.8)	-19.4 (-56.4 to 54.6)	22.9 (-34.5 to 140.3)
Southeast Asia, east Asia, and Oceania	Southeast Asia	Sri Lanka	YLLs	Age-standardized	0.2 (0.1 to 0.3)	0.1 (0.1 to 0.1)	0.3 (0.2 to 0.4)	0.1 (0.1 to 0.1)	0 (0 to 0.1)	0.2 (0.1 to 0.3)	-50.6 (-70.2 to -13)	-60 (-77 to -25.6)	-42.4 (-68.2 to 14.5)
Southeast Asia, east Asia, and Oceania	Southeast Asia	Sri Lanka	YLDs	All Ages	54 (34 to 83)	5 (3 to 7)	50 (31 to 77)	170 (97 to 265)	7 (4 to 11)	162 (92 to 256)	212.1 (123.5 to 327.8)	51.7 (12.8 to 93.4)	227.3 (130.4 to 353.8)
Southeast Asia, east Asia, and Oceania	Southeast Asia	Sri Lanka	YLDs	Age-standardized	0.5 (0.3 to 0.7)	0.1 (0 to 0.1)	0.8 (0.5 to 1.3)	0.7 (0.4 to 1)	0.1 (0 to 0.1)	1.4 (0.8 to 2.2)	41.9 (3.6 to 89.3)	-18.6 (-37.8 to 1.1)	63.4 (17.4 to 123.6)
Southeast Asia, east Asia, and Oceania	Southeast Asia	Thailand	Incidence	All Ages	194 (151 to 248)	69 (47 to 96)	126 (97 to 158)	342 (257 to 438)	51 (31 to 75)	291 (211 to 382)	76.2 (32.8 to 131.4)	-26 (-51.8 to 13.2)	131.9 (76.2 to 202.6)
Southeast Asia, east Asia, and Oceania	Southeast Asia	Thailand	Incidence	Age-standardized	0.4 (0.3 to 0.5)	0.3 (0.2 to 0.4)	0.5 (0.4 to 0.7)	0.3 (0.3 to 0.4)	0.1 (0.1 to 0.2)	0.6 (0.5 to 0.8)	-13.7 (-32 to 8.3)	-60.8 (-74.3 to -42.9)	11.8 (-10.8 to 40.4)
Southeast Asia, east Asia, and Oceania	Southeast Asia	Thailand	Prevalence	All Ages	1,106 (844 to 1,467)	103 (73 to 139)	1,003 (748 to 1,368)	4,828 (3,563 to 6,472)	95 (68 to 126)	4,733 (3,487 to 6,374)	336.3 (249.2 to 450.5)	-7.9 (-32.8 to 24.6)	371.7 (273.5 to 499.1)
Southeast Asia, east Asia, and Oceania	Southeast Asia	Thailand	Prevalence	Age-standardized	2.8 (2.1 to 3.6)	0.4 (0.3 to 0.5)	5.5 (4.1 to 7.4)	4.7 (3.5 to 6.2)	0.2 (0.1 to 0.3)	9.9 (7.3 to 13.2)	69.1 (37 to 111.5)	-54.1 (-65.5 to -39.7)	81.4 (46.4 to 127.9)
Southeast Asia, east Asia, and Oceania	Southeast Asia	Thailand	Deaths	All Ages	8 (6 to 12)	1 (1 to 2)	7 (5 to 10)	8 (6 to 12)	1 (1 to 2)	7 (5 to 10)	1.5 (-40 to 70.6)	29.3 (-38 to 174.3)	-2.4 (-44.2 to 79.5)
Southeast Asia, east Asia, and Oceania	Southeast Asia	Thailand	Deaths	Age-standardized	0 (0 to 0)	0 (0 to 0)	0 (0 to 0.1)	0 (0 to 0)	0 (0 to 0)	0 (0 to 0)	-65.4 (-78.9 to -42.4)	-62.6 (-82.4 to -19.6)	-66.7 (-80.4 to -41.2)
Southeast Asia, east Asia, and Oceania	Southeast Asia	Thailand	DALYs	All Ages	406 (297 to 541)	37 (25 to 52)	369 (262 to 499)	944 (629 to 1,373)	38 (26 to 53)	907 (597 to 1,328)	132.5 (64.8 to 224.3)	2.6 (-35.4 to 62.9)	145.4 (69.5 to 246.1)
Southeast Asia, east Asia, and Oceania	Southeast Asia	Thailand	DALYs	Age-standardized	1 (0.8 to 1.3)	0.2 (0.1 to 0.3)	2 (1.4 to 2.6)	0.9 (0.6 to 1.3)	0.1 (0.1 to 0.1)	1.9 (1.2 to 2.8)	-10.9 (-37 to 21.7)	-60.6 (-75.8 to -36.6)	-5.2 (-33.9 to 32)
Southeast Asia, east Asia, and Oceania	Southeast Asia	Thailand	YLLs	All Ages	232 (157 to 337)	20 (11 to 33)	212 (135 to 316)	236 (154 to 345)	22 (13 to 36)	214 (136 to 319)	1.8 (-44.5 to 84.3)	10.3 (-49.2 to 136.2)	1 (-46.2 to 91.1)
Southeast Asia, east Asia, and Oceania	Southeast Asia	Thailand	YLLs	Age-standardized	0.6 (0.4 to 0.8)	0.1 (0.1 to 0.2)	1.1 (0.7 to 1.6)	0.2 (0.1 to 0.3)	0 (0 to 0.1)	0.4 (0.3 to 0.6)	-61.5 (-78.4 to -31.6)	-64.5 (-83.3 to -23.9)	-61.6 (-79 to -29)
Southeast Asia, east Asia, and Oceania	Southeast Asia	Thailand	YLDs	All Ages	174 (107 to 274)	16 (10 to 25)	158 (97 to 252)	708 (408 to 1,126)	15 (9 to 23)	693 (400 to 1,111)	306.8 (204.1 to 454.8)	-7.1 (-31.8 to 25.5)	339.1 (225.8 to 503.1)
Southeast Asia, east Asia, and Oceania	Southeast Asia	Thailand	YLDs	Age-standardized	0.4 (0.3 to 0.7)	0.1 (0 to 0.1)	0.9 (0.5 to 1.4)	0.7 (0.4 to 1.1)	0 (0 to 0)	1.4 (0.8 to 2.3)	57.3 (18.4 to 109.8)	-53.8 (-65.2 to -39.3)	67.9 (25.8 to 128.4)
Southeast Asia, east Asia, and Oceania	Southeast Asia	Timor-Leste	Incidence	All Ages	1 (1 to 1)	0 (0 to 0)	1 (1 to 1)	3 (2 to 4)	0 (0 to 1)	3 (2 to 3)	195.9 (143.7 to 261.6)	127.1 (44.6 to 292)	210 (150.5 to 280.4)
Southeast Asia, east Asia, and Oceania	Southeast Asia	Timor-Leste	Incidence	Age-standardized	0.2 (0.2 to 0.3)	0.1 (0 to 0.1)	0.4 (0.3 to 0.5)	0.3 (0.3 to 0.4)	0.1 (0 to 0.1)	0.6 (0.4 to 0.7)	42.2 (22.3 to 67.7)	15.4 (-18.5 to 78.9)	47.1 (26.1 to 76)
Southeast Asia, east Asia, and Oceania	Southeast Asia	Timor-Leste	Prevalence	All Ages	9 (6 to 12)	0 (0 to 0)	8 (6 to 12)	39 (28 to 51)	1 (0 to 1)	38 (28 to 50)	338.4 (250.1 to 457.2)	128.5 (60.9 to 223.3)	346 (258.6 to 475)
Southeast Asia, east Asia, and Oceania	Southeast Asia	Timor-Leste	Prevalence	Age-standardized	2.7 (2 to 3.6)	0.1 (0.1 to 0.2)	5.1 (3.7 to 6.9)	4.7 (3.4 to 6.1)	0.1 (0.1 to 0.2)	9.2 (6.7 to 12)	75.6 (46.8 to 117.6)	11.2 (-13.8 to 42.4)	79.7 (49.5 to 123.8)
Southeast Asia, east Asia, and Oceania	Southeast Asia	Timor-Leste	Deaths	All Ages	0 (0 to 0)	0 (0 to 0)	0 (0 to 0)	0 (0 to 0)	0 (0 to 0)	0 (0 to 0)	153.8 (57.4 to 328.7)	177.9 (49.4 to 404.5)	146.4 (38.2 to 440.8)
Southeast Asia, east Asia, and Oceania	Southeast Asia	Timor-Leste	Deaths	Age-standardized	0 (0 to 0.1)	0 (0 to 0)	0.1 (0 to 0.1)	0 (0 to 0.1)	0 (0 to 0)	0.1 (0 to 0.1)	-15.8 (-47.7 to 43)	-7.4 (-49 to 63)	-20.9 (-56.4 to 70.5)
Southeast Asia, east Asia, and Oceania	Southeast Asia	Timor-Leste	DALYs	All Ages	4 (3 to 5)	1 (0 to 1)	3 (2 to 5)	11 (8 to 15)	2 (1 to 3)	10 (6 to 13)	179.8 (98.2 to 299.9)	128.3 (24.8 to 313.4)	190.9 (104.6 to 354.8)
Southeast Asia, east Asia, and Oceania	Southeast Asia	Timor-Leste	DALYs	Age-standardized	1.3 (0.8 to 1.7)	0.4 (0.2 to 0.7)	2.1 (1.1 to 3)	1.4 (1 to 1.8)	0.4 (0.2 to 0.6)	2.3 (1.6 to 3.3)	5.8 (-25.6 to 52.1)	-12.6 (-52.2 to 51.5)	9.6 (-24.4 to 71.4)
Southeast Asia, east Asia, and Oceania	Southeast Asia	Timor-Leste	YLLs	All Ages	3 (1 to 4)	1 (0 to 1)	2 (1 to 3)	5 (3 to 8)	1 (1 to 3)	4 (2 to 6)	112.1 (28.6 to 258.1)	128.2 (19.3 to 323.4)	106.6 (16.8 to 351.4)
Southeast Asia, east Asia, and Oceania	Southeast Asia	Timor-Leste	YLLs	Age-standardized	0.9 (0.5 to 1.3)	0.4 (0.2 to 0.7)	1.3 (0.4 to 2)	0.7 (0.4 to 1)	0.4 (0.2 to 0.6)	1 (0.5 to 1.6)	-21.3 (-50.7 to 32.6)	-13.8 (-54.2 to 54.4)	-23.8 (-57.4 to 63.6)
Southeast Asia, east Asia, and Oceania	Southeast Asia	Timor-Leste	YLDs	All Ages	1 (1 to 2)	0 (0 to 0)	1 (1 to 2)	6 (3 to 9)	0 (0 to 0)	6 (3 to 9)	306.8 (195.7 to 445.5)	129.3 (61.9 to 223.1)	313.3 (197.9 to 460.9)
Southeast Asia, east Asia, and Oceania	Southeast Asia	Timor-Leste	YLDs	Age-standardized	0.4 (0.3 to 0.7)	0 (0 to 0)	0.8 (0.5 to 1.3)	0.7 (0.4 to 1.1)	0 (0 to 0)	1.3 (0.8 to 2.1)	62.1 (21.1 to 115.9)	11 (-13.5 to 41.6)	65.2 (21.5 to 121.5)
Southeast Asia, east Asia, and Oceania	Southeast Asia	Viet Nam	Incidence	All Ages	100 (76 to 130)	23 (13 to 38)	77 (59 to 99)	331 (249 to 423)	43 (24 to 66)	289 (214 to 378)	230 (164.4 to 306.7)	84.1 (22.9 to 193.2)	273.6 (198.8 to 367.4)
Southeast Asia, east Asia, and Oceania	Southeast Asia	Viet Nam	Incidence	Age-standardized	0.2 (0.2 to 0.3)	0.1 (0 to 0.1)	0.4 (0.3 to 0.5)	0.3 (0.2 to 0.4)	0.1 (0 to 0.1)	0.6 (0.4 to 0.7)	44.2 (20.5 to 72.4)	-4 (-32.8 to 47.2)	49.4 (23.2 to 80.3)
Southeast Asia, east Asia, and Oceania	Southeast Asia	Viet Nam	Prevalence	All Ages	927 (694 to 1,245)	44 (30 to 62)	883 (652 to 1,204)	4,071 (2,986 to 5,444)	86 (61 to 115)	3,985 (2,917 to 5,356)	339.3 (256.1 to 451.9)	96.6 (53.4 to 158.9)	351.4 (264.7 to 477.3)
Southeast Asia, east Asia, and Oceania	Southeast Asia	Viet Nam	Prevalence	Age-standardized	2.2 (1.7 to 3)	0.2 (0.1 to 0.2)	5 (3.8 to 6.8)	4.1 (3 to 5.4)	0.2 (0.1 to 0.2)	9.3 (6.8 to 12.3)	83.9 (49.5 to 131.7)	-2.6 (-21.9 to 23.9)	83.8 (49 to 132)
Southeast Asia, east Asia, and Oceania	Southeast Asia	Viet Nam	Deaths	All Ages	14 (4 to 21)	3 (1 to 5)	11 (3 to 18)	20 (7 to 31)	5 (1 to 10)	15 (4 to 25)	44.3 (-14.7 to 189.5)	82.6 (-23.4 to 275.3)	33.7 (-28 to 277.1)
Southeast Asia, east Asia, and Oceania	Southeast Asia	Viet Nam	Deaths	Age-standardized	0 (0 to 0.1)	0 (0 to 0)	0.1 (0 to 0.1)	0 (0 to 0)	0 (0 to 0)	0 (0 to 0.1)	-34.5 (-61 to 29.1)	-16.6 (-65.2 to 73.1)	-40.8 (-68.5 to 69.7)
Southeast Asia, east Asia, and Oceania	Southeast Asia	Viet Nam	DALYs	All Ages	440 (222 to 623)	61 (24 to 101)	379 (191 to 556)	1,018 (646 to 1,434)	105 (41 to 172)	913 (577 to 1,320)	131.1 (56.2 to 269.5)	72.3 (-16.4 to 218.8)	140.6 (56.4 to 321.8)
Southeast Asia, east Asia, and Oceania	Southeast Asia	Viet Nam	DALYs	Age-standardized	1.1 (0.5 to 1.5)	0.3 (0.1 to 0.4)	2.3 (1.1 to 3.3)	1.1 (0.7 to 1.5)	0.2 (0.1 to 0.3)	2.2 (1.4 to 3.2)	-2.3 (-33 to 53.3)	-19.8 (-61.7 to 49.9)	-3.4 (-37.5 to 73.1)
Southeast Asia, east Asia, and Oceania	Southeast Asia	Viet Nam	YLLs	All Ages	295 (94 to 454)	54 (17 to 93)	241 (70 to 393)	409 (140 to 638)	91 (28 to 155)	318 (98 to 549)	39 (-18.8 to 198.5)	69.2 (-26.9 to 241.7)	32.2 (-28.9 to 261.6)
Southeast Asia, east Asia, and Oceania	Southeast Asia	Viet Nam	YLLs	Age-standardized	0.7 (0.2 to 1.1)	0.2 (0.1 to 0.4)	1.5 (0.4 to 2.4)	0.5 (0.2 to 0.7)	0.2 (0.1 to 0.3)	0.8 (0.3 to 1.5)	-38.1 (-63.2 to 29.2)	-21.8 (-66.4 to 58.4)	-43 (-69.3 to 58.1)
Southeast Asia, east Asia, and Oceania	Southeast Asia	Viet Nam	YLDs	All Ages	146 (89 to 230)	7 (4 to 12)	139 (85 to 221)	608 (356 to 982)	14 (8 to 21)	595 (344 to 967)	317.5 (214.7 to 463.5)	96.5 (53.7 to 158.1)	328.6 (221.1 to 483.3)
Southeast Asia, east Asia, and Oceania	Southeast Asia	Viet Nam	YLDs	Age-standardized	0.3 (0.2 to 0.6)	0 (0 to 0)	0.8 (0.5 to 1.3)	0.6 (0.3 to 1)	0 (0 to 0)	1.4 (0.8 to 2.2)	72.6 (29 to 133.6)	-2.7 (-21.9 to 23.2)	70.8 (26 to 133.8)
		Oceania	Incidence	All Ages	21 (17 to 26)	8 (5 to 10)	14 (11 to 17)	65 (53 to 79)	24 (17 to 32)	41 (33 to 51)	205.1 (177 to 234.9)	217.7 (173.7 to 268.2)	198.3 (164 to 236.5)
		Oceania	Incidence	Age-standardized	0.6 (0.5 to 0.8)	0.5 (0.3 to 0.6)	0.8 (0.7 to 1)	0.8 (0.7 to 0.9)	0.6 (0.5 to 0.8)	1 (0.8 to 1.2)	25.6 (16.8 to 35.5)	32.6 (19.4 to 49.3)	20.9 (10.2 to 34.9)
		Oceania	Prevalence	All Ages	139 (110 to 174)	13 (10 to 17)	126 (98 to 163)	474 (369 to 601)	43 (33 to 55)	432 (327 to 558)	241.9 (200.1 to 288.7)	232 (194.7 to 280.1)	242.9 (195.7 to 294.3)
		Oceania	Prevalence	Age-standardized	4.6 (3.7 to 5.7)	0.9 (0.7 to 1.1)	8.2 (6.5 to 10.4)	6.7 (5.2 to 8.3)	1.3 (1 to 1.6)	11.9 (9.1 to 15)	44.9 (28.3 to 63.2)	41.4 (26.7 to 59.9)	44.6 (26.9 to 64.9)
		Oceania	Deaths	All Ages	7 (4 to 10)	2 (1 to 3)	5 (2 to 8)	13 (8 to 19)	4 (3 to 7)	9 (4 to 14)	88.7 (38.4 to 157)	155 (58.2 to 298)	66.7 (16.2 to 139.5)
		Oceania	Deaths	Age-standardized	0.4 (0.2 to 0.5)	0.2 (0.1 to 0.3)	0.6 (0.2 to 1)	0.3 (0.1 to 0.4)	0.2 (0.1 to 0.3)	0.4 (0.2 to 0.6)	-23.3 (-44.4 to 2.4)	3 (-37.5 to 67.9)	-32.7 (-54.4 to -2.9)
		Oceania	DALYs	All Ages	199 (122 to 278)	49 (30 to 76)	150 (74 to 231)	400 (268 to 563)	125 (78 to 203)	275 (155 to 405)	100.8 (49.9 to 175)	156.4 (60.7 to 304.3)	82.9 (32.9 to 160.5)
		Oceania	DALYs	Age-standardized	7 (4 to 9.9)	3.3 (2.1 to 5)	10.9 (5 to 16.8)	5.8 (3.7 to 7.9)	3.4 (2.2 to 5.2)	8.2 (4.3 to 11.9)	-17.2 (-37.4 to 9)	4.9 (-33 to 59.5)	-24.7 (-44.9 to 3.1)
		Oceania	YLLs	All Ages	178 (102 to 258)	47 (29 to 74)	131 (55 to 208)	329 (202 to 500)	118 (71 to 194)	211 (98 to 345)	85.1 (33.1 to 167)	153.2 (56.2 to 307.6)	60.9 (10.6 to 142.2)
		Oceania	YLLs	Age-standardized	6.3 (3.3 to 9.1)	3.1 (1.9 to 4.8)	9.6 (3.7 to 15.4)	4.8 (2.8 to 7)	3.2 (2 to 5)	6.5 (2.7 to 10.4)	-23.6 (-44.5 to 4.4)	3.2 (-35.8 to 60.4)	-33 (-52.8 to -4.8)
		Oceania	YLDs	All Ages	21 (14 to 32)	2 (1 to 3)	19 (12 to 29)	71 (45 to 106)	7 (4 to 10)	64 (40 to 96)	231.5 (175.2 to 299.8)	230.7 (194 to 277.1)	231.6 (170.3 to 308)
		Oceania	YLDs	Age-standardized	0.7 (0.5 to 1)	0.1 (0.1 to 0.2)	1.2 (0.8 to 1.9)	1 (0.6 to 1.5)	0.2 (0.1 to 0.3)	1.7 (1.1 to 2.6)	40.1 (16.9 to 69.4)	40.4 (26 to 58.1)	39.4 (14.8 to 71.9)
Southeast Asia, east Asia, and Oceania	Oceania	American Samoa	Incidence	All Ages	0 (0 to 0)	0 (0 to 0)	0 (0 to 0)	0 (0 to 0)	0 (0 to 0)	0 (0 to 0)	173.9 (139 to 216.1)	203 (144.5 to 286.3)	158.3 (117.2 to 206.8)
Southeast Asia, east Asia, and Oceania	Oceania	American Samoa	Incidence	Age-standardized	0.5 (0.4 to 0.6)	0.4 (0.3 to 0.5)	0.6 (0.5 to 0.8)	0.7 (0.6 to 0.9)	0.6 (0.4 to 0.7)	0.9 (0.7 to 1.2)	45.6 (30.4 to 64.1)	51.3 (25.1 to 87.1)	44.7 (25.7 to 67.9)
Southeast Asia, east Asia, and Oceania	Oceania	American Samoa	Prevalence	All Ages	1 (1 to 1)	0 (0 to 0)	1 (1 to 1)	3 (2 to 4)	0 (0 to 0)	3 (2 to 4)	221.7 (167.7 to 283.9)	228 (172.9 to 301.3)	221.1 (163.1 to 289)
Southeast Asia, east Asia, and Oceania	Oceania	American Samoa	Prevalence	Age-standardized	4 (3.2 to 5.1)	0.7 (0.6 to 0.9)	7.2 (5.6 to 9.3)	6.3 (4.9 to 8)	1.1 (0.8 to 1.4)	12 (9 to 15.6)	58.7 (35.6 to 88.2)	52.2 (30.1 to 80.1)	66.1 (39.3 to 100.4)
Southeast Asia, east Asia, and Oceania	Oceania	American Samoa	Deaths	All Ages	0 (0 to 0)	0 (0 to 0)	0 (0 to 0)	0 (0 to 0)	0 (0 to 0)	0 (0 to 0)	-0.1 (-32.2 to 45.6)	42.3 (-19.2 to 140.6)	-24 (-51.9 to 21.6)
Southeast Asia, east Asia, and Oceania	Oceania	American Samoa	Deaths	Age-standardized	0.1 (0.1 to 0.1)	0.1 (0 to 0.1)	0.1 (0.1 to 0.1)	0 (0 to 0)	0 (0 to 0)	0 (0 to 0)	-62.3 (-75.2 to -42.5)	-54.8 (-75.8 to -18.2)	-67.1 (-80.7 to -42.3)
Southeast Asia, east Asia, and Oceania	Oceania	American Samoa	DALYs	All Ages	0 (0 to 0)	0 (0 to 0)	0 (0 to 0)	1 (0 to 1)	0 (0 to 0)	0 (0 to 1)	75 (32.2 to 125.2)	51.6 (-2.7 to 127.2)	81.9 (32.2 to 140.9)
Southeast Asia, east Asia, and Oceania	Oceania	American Samoa	DALYs	Age-standardized	1.7 (1.3 to 2.1)	0.8 (0.6 to 1.2)	2.5 (1.9 to 3.2)	1.3 (0.9 to 1.8)	0.5 (0.4 to 0.7)	2.2 (1.4 to 3.1)	-20.9 (-40.4 to 2.8)	-37 (-59.9 to -4.4)	-13.1 (-37.1 to 17)

Pneumoconiosis

7 GBD super region	21 GBD region	Location	Measure	Age	1990 Both	1990 Female	1990 Male	2019 Both	2019 Female	2019 Male	Percent change (1990 to 2019) Both	Percent change (1990 to 2019) Female	Percent change (1990 to 2019) Male
Southeast Asia, east Asia, and Oceania	Oceania	American Samoa	YLLs	All Ages	0 (0 to 0)	0 (0 to 0)	0 (0 to 0)	0 (0 to 0)	0 (0 to 0)	0 (0 to 0)	-19.1 (-45 to 21)	17.9 (-33.4 to 104.4)	-37.2 (-60.5 to -2.3)
Southeast Asia, east Asia, and Oceania	Oceania	American Samoa	YLLs	Age-standardized	1 (0.8 to 1.3)	0.7 (0.5 to 1.1)	1.4 (1 to 1.9)	0.4 (0.3 to 0.5)	0.4 (0.2 to 0.5)	0.4 (0.3 to 0.6)	-62.5 (-74.5 to -44.5)	-50.9 (-72.1 to -17.3)	-68.4 (-79.6 to -50.9)
Southeast Asia, east Asia, and Oceania	Oceania	American Samoa	YLDs	All Ages	0 (0 to 0)	0 (0 to 0)	0 (0 to 0)	0 (0 to 1)	0 (0 to 0)	0 (0 to 1)	205 (133.8 to 291.7)	227.1 (173.3 to 298.6)	202.9 (126.1 to 296.4)
Southeast Asia, east Asia, and Oceania	Oceania	American Samoa	YLDs	Age-standardized	0.6 (0.4 to 0.9)	0.1 (0.1 to 0.2)	1.1 (0.7 to 1.7)	0.9 (0.6 to 1.4)	0.2 (0.1 to 0.3)	1.7 (1 to 2.6)	50.5 (16 to 95.1)	50.8 (29 to 77.3)	56.9 (17.5 to 108)
Southeast Asia, east Asia, and Oceania	Oceania	Cook Islands	Incidence	All Ages	0 (0 to 0)	0 (0 to 0)	0 (0 to 0)	0 (0 to 0)	0 (0 to 0)	0 (0 to 0)	134 (101.1 to 169.5)	148.6 (95.6 to 218.3)	128.1 (91.6 to 169.8)
Southeast Asia, east Asia, and Oceania	Oceania	Cook Islands	Incidence	Age-standardized	0.4 (0.4 to 0.5)	0.3 (0.2 to 0.4)	0.6 (0.5 to 0.7)	0.6 (0.5 to 0.7)	0.4 (0.3 to 0.5)	0.8 (0.6 to 1)	33.8 (18.6 to 51.6)	32.1 (7.5 to 62.5)	40.1 (21.4 to 62.8)
Southeast Asia, east Asia, and Oceania	Oceania	Cook Islands	Prevalence	All Ages	1 (0 to 1)	0 (0 to 0)	0 (0 to 1)	2 (1 to 2)	0 (0 to 0)	1 (1 to 2)	189.3 (144.3 to 245.8)	175.6 (125.5 to 254.8)	190.2 (141.6 to 252.3)
Southeast Asia, east Asia, and Oceania	Oceania	Cook Islands	Prevalence	Age-standardized	4.1 (3.2 to 5.3)	0.6 (0.4 to 0.7)	7.4 (5.6 to 9.7)	6.1 (4.7 to 7.8)	0.7 (0.6 to 1)	11.7 (8.8 to 15.2)	48.8 (26.7 to 76.7)	33 (11.7 to 66.2)	59.3 (34.9 to 91.7)
Southeast Asia, east Asia, and Oceania	Oceania	Cook Islands	Deaths	All Ages	0 (0 to 0)	0 (0 to 0)	0 (0 to 0)	0 (0 to 0)	0 (0 to 0)	0 (0 to 0)	7.8 (-27.6 to 65.1)	41.4 (-25.2 to 153.8)	-13.9 (-48.5 to 50)
Southeast Asia, east Asia, and Oceania	Oceania	Cook Islands	Deaths	Age-standardized	0.1 (0.1 to 0.2)	0.1 (0.1 to 0.2)	0.2 (0.1 to 0.2)	0.1 (0 to 0.1)	0.1 (0 to 0.1)	0.1 (0 to 0.1)	-51.8 (-68.4 to -24.3)	-40.7 (-68.4 to 10)	-61.4 (-77.9 to -29.4)
Southeast Asia, east Asia, and Oceania	Oceania	Cook Islands	DALYs	All Ages	0 (0 to 0)	0 (0 to 0)	0 (0 to 0)	0 (0 to 1)	0 (0 to 0)	0 (0 to 0)	28.9 (-4.2 to 70.4)	8.7 (-34.2 to 87)	38.4 (-0.2 to 87.2)
Southeast Asia, east Asia, and Oceania	Oceania	Cook Islands	DALYs	Age-standardized	2.7 (2.2 to 3.4)	1.8 (1.2 to 2.6)	3.6 (2.7 to 4.6)	1.8 (1.3 to 2.4)	1 (0.6 to 1.5)	2.6 (1.8 to 3.6)	-33.8 (-51.1 to -12.7)	-44.9 (-67 to -5.2)	-27.4 (-48.3 to -1.7)
Southeast Asia, east Asia, and Oceania	Oceania	Cook Islands	YLLs	All Ages	0 (0 to 0)	0 (0 to 0)	0 (0 to 0)	0 (0 to 0)	0 (0 to 0)	0 (0 to 0)	-19.3 (-46.7 to 23.2)	0 (-43.8 to 78.7)	-32.6 (-58.1 to 14.3)
Southeast Asia, east Asia, and Oceania	Oceania	Cook Islands	YLLs	Age-standardized	2.1 (1.6 to 2.6)	1.7 (1.1 to 2.5)	2.5 (1.8 to 3.4)	0.9 (0.6 to 1.3)	0.9 (0.5 to 1.4)	0.9 (0.6 to 1.4)	-56.7 (-71.3 to -33.6)	-49 (-71.3 to -7.5)	-63 (-76.7 to -37.9)
Southeast Asia, east Asia, and Oceania	Oceania	Cook Islands	YLDs	All Ages	0 (0 to 0)	0 (0 to 0)	0 (0 to 0)	0 (0 to 0)	0 (0 to 0)	0 (0 to 0)	173.3 (110 to 256.8)	175 (125.9 to 253.2)	173.2 (106.7 to 262.2)
Southeast Asia, east Asia, and Oceania	Oceania	Cook Islands	YLDs	Age-standardized	0.6 (0.4 to 1)	0.1 (0.1 to 0.1)	1.1 (0.7 to 1.7)	0.9 (0.6 to 1.3)	0.1 (0.1 to 0.2)	1.7 (1 to 2.6)	41.2 (9.2 to 82.4)	32.1 (11.1 to 64.6)	50.9 (14.3 to 97)
Southeast Asia, east Asia, and Oceania	Oceania	Fiji	Incidence	All Ages	2 (2 to 2)	1 (0 to 1)	1 (1 to 2)	5 (4 to 6)	1 (1 to 2)	3 (3 to 4)	143.1 (109.7 to 180.6)	118.9 (66.3 to 178.7)	153.8 (113.5 to 203.6)
Southeast Asia, east Asia, and Oceania	Oceania	Fiji	Incidence	Age-standardized	0.5 (0.4 to 0.6)	0.3 (0.2 to 0.4)	0.6 (0.5 to 0.8)	0.6 (0.5 to 0.7)	0.3 (0.2 to 0.4)	0.9 (0.7 to 1.1)	28.3 (13.8 to 43.9)	18.7 (-2.9 to 45.6)	34.5 (16.3 to 53.8)
Southeast Asia, east Asia, and Oceania	Oceania	Fiji	Prevalence	All Ages	15 (11 to 19)	1 (1 to 1)	14 (10 to 18)	44 (33 to 57)	2 (2 to 3)	42 (31 to 55)	198.3 (151.4 to 260.6)	129.2 (87.7 to 184.4)	203.3 (153.3 to 269.6)
Southeast Asia, east Asia, and Oceania	Oceania	Fiji	Prevalence	Age-standardized	4 (3.1 to 5.2)	0.5 (0.4 to 0.7)	7.6 (5.9 to 10)	5.8 (4.4 to 7.4)	0.7 (0.5 to 0.8)	11.8 (8.9 to 15.3)	45.1 (25.4 to 71.9)	19.7 (0.8 to 47.2)	56.4 (34.4 to 86.2)
Southeast Asia, east Asia, and Oceania	Oceania	Fiji	Deaths	All Ages	0 (0 to 0)	0 (0 to 0)	0 (0 to 0)	0 (0 to 0)	0 (0 to 0)	0 (0 to 0)	24 (-21.4 to 89.5)	29.2 (-35 to 124)	20.7 (-27.5 to 110.4)
Southeast Asia, east Asia, and Oceania	Oceania	Fiji	Deaths	Age-standardized	0 (0 to 0)	0 (0 to 0)	0 (0 to 0)	0 (0 to 0)	0 (0 to 0)	0 (0 to 0)	-37 (-60.1 to -5.3)	-34.8 (-67 to 14.4)	-34.1 (-62.1 to 19.1)
Southeast Asia, east Asia, and Oceania	Oceania	Fiji	DALYs	All Ages	4 (3 to 5)	1 (1 to 1)	3 (2 to 4)	8 (5 to 11)	1 (1 to 1)	7 (4 to 10)	121.3 (72 to 179.5)	33.8 (-22.3 to 112.7)	144.7 (87.6 to 214.3)
Southeast Asia, east Asia, and Oceania	Oceania	Fiji	DALYs	Age-standardized	1 (0.7 to 1.3)	0.4 (0.3 to 0.5)	1.6 (1.2 to 2.3)	1.1 (0.7 to 1.5)	0.3 (0.2 to 0.4)	2 (1.3 to 2.9)	8.9 (-14.9 to 36.7)	-23.5 (-53.3 to 14.9)	22.7 (-5.9 to 55.8)
Southeast Asia, east Asia, and Oceania	Oceania	Fiji	YLLs	All Ages	1 (1 to 2)	1 (0 to 1)	1 (0 to 1)	2 (1 to 2)	1 (0 to 1)	1 (1 to 1)	12.9 (-30.4 to 73.3)	8.5 (-47.2 to 104.4)	16.4 (-31.4 to 106.8)
Southeast Asia, east Asia, and Oceania	Oceania	Fiji	YLLs	Age-standardized	0.4 (0.3 to 0.5)	0.3 (0.2 to 0.4)	0.5 (0.3 to 0.7)	0.2 (0.2 to 0.3)	0.2 (0.1 to 0.3)	0.3 (0.2 to 0.4)	-40.2 (-61.8 to -9.3)	-36.9 (-68.4 to 14.1)	-38.8 (-62.4 to 3.9)
Southeast Asia, east Asia, and Oceania	Oceania	Fiji	YLDs	All Ages	2 (1 to 3)	0 (0 to 0)	2 (1 to 3)	6 (4 to 10)	0 (0 to 1)	6 (4 to 9)	186.4 (124.6 to 268.2)	129.1 (87.8 to 184.1)	190.8 (123.9 to 282.1)
Southeast Asia, east Asia, and Oceania	Oceania	Fiji	YLDs	Age-standardized	0.6 (0.4 to 0.9)	0.1 (0.1 to 0.1)	1.1 (0.7 to 1.8)	0.8 (0.5 to 1.3)	0.1 (0.1 to 0.2)	1.7 (1 to 2.6)	39.8 (9.1 to 80.3)	19.6 (0.6 to 46.8)	49.7 (14.6 to 95.5)
Southeast Asia, east Asia, and Oceania	Oceania	Guam	Incidence	All Ages	0 (0 to 0)	0 (0 to 0)	0 (0 to 0)	1 (1 to 1)	0 (0 to 0)	1 (1 to 1)	195.7 (149.8 to 252.6)	220.3 (141 to 355.5)	188.8 (137.7 to 251.5)
Southeast Asia, east Asia, and Oceania	Oceania	Guam	Incidence	Age-standardized	0.4 (0.3 to 0.5)	0.2 (0.1 to 0.3)	0.6 (0.5 to 0.7)	0.5 (0.4 to 0.7)	0.3 (0.2 to 0.3)	0.8 (0.7 to 1.1)	38.7 (22.5 to 58.5)	26 (0.6 to 64.1)	44.2 (24.5 to 69.1)
Southeast Asia, east Asia, and Oceania	Oceania	Guam	Prevalence	All Ages	3 (2 to 4)	0 (0 to 0)	3 (2 to 4)	12 (9 to 15)	1 (0 to 1)	11 (8 to 14)	263.3 (197 to 351.2)	269.5 (197.1 to 384)	262.9 (193.8 to 356.5)
Southeast Asia, east Asia, and Oceania	Oceania	Guam	Prevalence	Age-standardized	4 (3.2 to 5.1)	0.5 (0.4 to 0.6)	7.5 (6 to 9.7)	6.1 (4.6 to 7.7)	0.6 (0.4 to 0.8)	11.9 (9 to 15.4)	51.7 (27.1 to 85.7)	14.2 (-3.1 to 41.9)	57.9 (31.7 to 93.3)
Southeast Asia, east Asia, and Oceania	Oceania	Guam	Deaths	All Ages	0 (0 to 0)	0 (0 to 0)	0 (0 to 0)	0 (0 to 0)	0 (0 to 0)	0 (0 to 0)	-4.6 (-37.8 to 47.8)	-0.7 (-43.2 to 83.5)	-5.3 (-41.2 to 53.8)
Southeast Asia, east Asia, and Oceania	Oceania	Guam	Deaths	Age-standardized	0.1 (0 to 0.1)	0 (0 to 0)	0.1 (0.1 to 0.2)	0 (0 to 0)	0 (0 to 0)	0 (0 to 0)	-78.9 (-86.3 to -67.3)	-85.2 (-91.6 to -70.5)	-76.6 (-85.7 to -60.3)
Southeast Asia, east Asia, and Oceania	Oceania	Guam	DALYs	All Ages	1 (1 to 1)	0 (0 to 0)	1 (1 to 1)	2 (1 to 3)	0 (0 to 0)	2 (1 to 3)	103.4 (48.1 to 167.2)	44.4 (1.2 to 101.2)	110.1 (49.7 to 179.6)
Southeast Asia, east Asia, and Oceania	Oceania	Guam	DALYs	Age-standardized	1.5 (1.2 to 1.8)	0.4 (0.2 to 0.5)	2.7 (2.1 to 3.4)	1.1 (0.7 to 1.6)	0.1 (0.1 to 0.2)	2.1 (1.4 to 3.1)	-25.8 (-46.2 to -1.2)	-58.2 (-71 to -40.2)	-21.2 (-44.6 to 6.5)
Southeast Asia, east Asia, and Oceania	Oceania	Guam	YLLs	All Ages	1 (0 to 1)	0 (0 to 0)	0 (0 to 1)	0 (0 to 1)	0 (0 to 0)	0 (0 to 0)	-27.8 (-51.5 to 9.6)	-27.9 (-55.6 to 18.2)	-27.8 (-54 to 15.6)
Southeast Asia, east Asia, and Oceania	Oceania	Guam	YLLs	Age-standardized	0.9 (0.7 to 1.1)	0.3 (0.2 to 0.4)	1.5 (1.1 to 2.1)	0.2 (0.2 to 0.3)	0.1 (0 to 0.1)	0.4 (0.3 to 0.5)	-76.2 (-83.7 to -64.7)	-79 (-87 to -65.5)	-75.5 (-84.1 to -60.6)
Southeast Asia, east Asia, and Oceania	Oceania	Guam	YLDs	All Ages	0 (0 to 1)	0 (0 to 0)	0 (0 to 1)	2 (1 to 3)	0 (0 to 0)	2 (1 to 3)	247.8 (164.1 to 360.5)	270.6 (198.2 to 384.3)	246.6 (159.8 to 366.4)
Southeast Asia, east Asia, and Oceania	Oceania	Guam	YLDs	Age-standardized	0.6 (0.4 to 0.9)	0.1 (0.1 to 0.1)	1.1 (0.7 to 1.7)	0.9 (0.5 to 1.4)	0.1 (0.1 to 0.1)	1.7 (1 to 2.7)	46.9 (11.9 to 95.9)	14 (-3.1 to 41.2)	53 (13.8 to 107.4)
Southeast Asia, east Asia, and Oceania	Oceania	Kiribati	Incidence	All Ages	0 (0 to 0)	0 (0 to 0)	0 (0 to 0)	1 (1 to 1)	0 (0 to 0)	1 (0 to 1)	150.2 (122.7 to 185.9)	176.4 (107.9 to 260.2)	144 (112.6 to 184.7)
Southeast Asia, east Asia, and Oceania	Oceania	Kiribati	Incidence	Age-standardized	0.6 (0.5 to 0.8)	0.2 (0.2 to 0.3)	1.2 (1 to 1.4)	0.9 (0.7 to 1.1)	0.4 (0.3 to 0.5)	1.5 (1.2 to 1.9)	33.9 (21.9 to 48.9)	50.5 (22.6 to 87.5)	30.9 (18.7 to 47.7)
Southeast Asia, east Asia, and Oceania	Oceania	Kiribati	Prevalence	All Ages	2 (2 to 3)	0 (0 to 0)	2 (1 to 3)	5 (4 to 7)	0 (0 to 0)	5 (4 to 7)	168.2 (130.9 to 224.3)	180.6 (127.1 to 244.7)	167.6 (128.4 to 227.4)
Southeast Asia, east Asia, and Oceania	Oceania	Kiribati	Prevalence	Age-standardized	5.4 (4.2 to 7.1)	0.6 (0.4 to 0.7)	11.7 (9.1 to 15.5)	7.8 (6.1 to 10.5)	0.9 (0.7 to 1.2)	17.8 (13.6 to 23.9)	46.4 (28.3 to 73.4)	60.9 (32.3 to 95.9)	52.3 (33.1 to 81.9)
Southeast Asia, east Asia, and Oceania	Oceania	Kiribati	Deaths	All Ages	0 (0 to 0)	0 (0 to 0)	0 (0 to 0)	0 (0 to 1)	0 (0 to 0)	0 (0 to 1)	45.3 (-5.8 to 128.7)	67.9 (-9.7 to 200.9)	40.2 (-16.2 to 143.9)
Southeast Asia, east Asia, and Oceania	Oceania	Kiribati	Deaths	Age-standardized	1.2 (0.9 to 1.5)	0.3 (0.2 to 0.6)	2.5 (1.8 to 3.4)	1 (0.7 to 1.4)	0.4 (0.2 to 0.6)	2.2 (1.5 to 3.3)	-10.9 (-41.8 to 37.1)	3.4 (-42.4 to 87.5)	-9 (-46.3 to 56.1)
Southeast Asia, east Asia, and Oceania	Oceania	Kiribati	DALYs	All Ages	10 (7 to 14)	2 (1 to 3)	8 (5 to 12)	13 (9 to 19)	3 (2 to 4)	11 (7 to 17)	40.9 (-14.7 to 128.9)	56.1 (-20 to 189.6)	37.5 (-23.9 to 145)
Southeast Asia, east Asia, and Oceania	Oceania	Kiribati	DALYs	Age-standardized	23.9 (18 to 30.7)	7.6 (4.8 to 11.9)	46.4 (34 to 61.6)	19.6 (14.3 to 26.4)	7 (4.3 to 10.9)	38.7 (27.3 to 55.5)	-18 (-44.6 to 23.7)	-9.1 (-50.5 to 60.2)	-16.6 (-47.5 to 35.6)
Southeast Asia, east Asia, and Oceania	Oceania	Kiribati	YLLs	All Ages	9 (7 to 13)	2 (1 to 3)	8 (5 to 12)	13 (9 to 19)	3 (2 to 4)	10 (6 to 16)	37 (-18.9 to 127)	55 (-21 to 189.4)	32.9 (-28.2 to 141.4)
Southeast Asia, east Asia, and Oceania	Oceania	Kiribati	YLLs	Age-standardized	23.1 (17.3 to 29.9)	7.6 (4.7 to 11.8)	44.6 (32 to 60.2)	18.5 (13.2 to 25.4)	6.8 (4.1 to 10.7)	36.1 (24.7 to 53.1)	-20.1 (-47 to 23.9)	-10 (-51.5 to 60.1)	-19 (-50.9 to 34.7)
Southeast Asia, east Asia, and Oceania	Oceania	Kiribati	YLDs	All Ages	0 (0 to 0)	0 (0 to 0)	0 (0 to 0)	1 (0 to 1)	0 (0 to 0)	1 (0 to 1)	158.3 (97.1 to 247.9)	178.9 (127.2 to 241)	157.2 (94.3 to 251.5)
Southeast Asia, east Asia, and Oceania	Oceania	Kiribati	YLDs	Age-standardized	0.8 (0.5 to 1.3)	0.1 (0.1 to 0.1)	1.8 (1.1 to 2.7)	1.1 (0.7 to 1.8)	0.2 (0.1 to 0.2)	2.6 (1.6 to 4)	40 (8.3 to 82.5)	59.8 (32.1 to 93.8)	44.8 (10.9 to 91)
Southeast Asia, east Asia, and Oceania	Oceania	Marshall Islands	Incidence	All Ages	0 (0 to 0)	0 (0 to 0)	0 (0 to 0)	0 (0 to 0)	0 (0 to 0)	0 (0 to 0)	182.3 (146.8 to 224.5)	210.8 (148.9 to 301.5)	168.3 (129.9 to 211.7)
Southeast Asia, east Asia, and Oceania	Oceania	Marshall Islands	Incidence	Age-standardized	0.6 (0.5 to 0.7)	0.4 (0.3 to 0.5)	0.8 (0.6 to 0.9)	0.8 (0.6 to 0.9)	0.6 (0.4 to 0.8)	0.9 (0.8 to 1.1)	34.5 (22 to 49.6)	51.6 (27.1 to 87.1)	23.7 (10.3 to 39.9)
Southeast Asia, east Asia, and Oceania	Oceania	Marshall Islands	Prevalence	All Ages	1 (1 to 1)	0 (0 to 0)	1 (0 to 1)	2 (2 to 3)	0 (0 to 0)	2 (2 to 3)	211.6 (161.9 to 270.1)	195.3 (144.9 to 265.1)	213.2 (159.7 to 278.1)
Southeast Asia, east Asia, and Oceania	Oceania	Marshall Islands	Prevalence	Age-standardized	4.2 (3.3 to 5.2)	0.8 (0.6 to 0.9)	7.8 (6.1 to 10.1)	6.4 (5 to 8.1)	1.2 (0.9 to 1.5)	11.4 (8.7 to 14.7)	54.9 (32.5 to 80.9)	54.5 (33.1 to 83.6)	45 (23.1 to 71.4)
Southeast Asia, east Asia, and Oceania	Oceania	Marshall Islands	Deaths	All Ages	0 (0 to 0)	0 (0 to 0)	0 (0 to 0)	0 (0 to 0)	0 (0 to 0)	0 (0 to 0)	19 (-23.3 to 87.6)	73.2 (-6 to 219.2)	1.2 (-39.8 to 76.3)
Southeast Asia, east Asia, and Oceania	Oceania	Marshall Islands	Deaths	Age-standardized	0.3 (0.1 to 0.4)	0.1 (0.1 to 0.2)	0.5 (0.2 to 0.9)	0.2 (0.1 to 0.3)	0.1 (0.1 to 0.2)	0.3 (0.1 to 0.5)	-28.2 (-53.2 to 12.8)	-1.8 (-45.4 to 73.2)	-48.1 (-69.8 to -6.3)
Southeast Asia, east Asia, and Oceania	Oceania	Marshall Islands	DALYs	All Ages	1 (1 to 2)	0 (0 to 0)	1 (0 to 1)	2 (1 to 2)	1 (0 to 1)	1 (1 to 2)	43.6 (-7.5 to 129.3)	96.3 (1.9 to 284.7)	26.8 (-23.6 to 112)
Southeast Asia, east Asia, and Oceania	Oceania	Marshall Islands	DALYs	Age-standardized	6.4 (3.6 to 9.2)	3 (1.6 to 5.1)	10.6 (5.4 to 16.4)	4.8 (3.2 to 6.9)	3 (1.5 to 5)	6.5 (4.2 to 9.9)	-25.3 (-48.6 to 11)	-0.4 (-42.9 to 74.6)	-39.1 (-59.9 to -0.9)
Southeast Asia, east Asia, and Oceania	Oceania	Marshall Islands	YLLs	All Ages	1 (1 to 2)	0 (0 to 0)	1 (0 to 1)	1 (1 to 2)	1 (0 to 1)	1 (0 to 1)	27.2 (-23.5 to 116.1)	92.9 (-1.7 to 288.2)	4.5 (-43 to 90.7)
Southeast Asia, east Asia, and Oceania	Oceania	Marshall Islands	YLLs	Age-standardized	5.8 (2.9 to 8.6)	2.9 (1.4 to 5)	9.4 (4.2 to 15.1)	3.9 (2.2 to 6)	2.8 (1.3 to 4.8)	4.8 (2.6 to 8.1)	-33.4 (-56.8 to 4.7)	-2.6 (-46.4 to 75.9)	-49 (-69.5 to -12.2)
Southeast Asia, east Asia, and Oceania	Oceania	Marshall Islands	YLDs	All Ages	0 (0 to 0)	0 (0 to 0)	0 (0 to 0)	0 (0 to 0)	0 (0 to 0)	0 (0 to 0)	203.2 (127.6 to 298.6)	192.6 (142.8 to 261.2)	204.3 (121.8 to 311.7)
Southeast Asia, east Asia, and Oceania	Oceania	Marshall Islands	YLDs	Age-standardized	0.6 (0.4 to 1)	0.1 (0.1 to 0.2)	1.2 (0.7 to 1.8)	0.9 (0.6 to 1.4)	0.2 (0.1 to 0.3)	1.7 (1 to 2.6)	49.4 (13.8 to 92.6)	53.1 (32.3 to 81.2)	40.1 (3.4 to 85.3)
Southeast Asia, east Asia, and Oceania	Oceania	Micronesia (Federated States of)	Incidence	All Ages	0 (0 to 0)	0 (0 to 0)	0 (0 to 0)	1 (0 to 1)	0 (0 to 0)	0 (0 to 0)	86.2 (63.9 to 111.1)	97 (63.8 to 138.1)	79.2 (50.9 to 109.1)
Southeast Asia, east Asia, and Oceania	Oceania	Micronesia (Federated States of)	Incidence	Age-standardized	0.7 (0.5 to 0.8)	0.5 (0.4 to 0.7)	0.8 (0.7 to 1)	0.8 (0.7 to 0.9)	0.7 (0.5 to 0.9)	0.9 (0.8 to 1.2)	22.5 (11.9 to 35.5)	28.4 (9.9 to 51.1)	19.1 (5.3 to 34.5)
Southeast Asia, east Asia, and Oceania	Oceania	Micronesia (Federated States of)	Prevalence	All Ages	2 (2 to 2)	0 (0 to 0)	2 (1 to 2)	4 (3 to 6)	0 (0 to 1)	4 (3 to 5)	117.5 (84.4 to 160.2)	106.1 (77.3 to 144.4)	119 (81.2 to 166.9)
Southeast Asia, east Asia, and Oceania	Oceania	Micronesia (Federated States of)	Prevalence	Age-standardized	4.2 (3.4 to 5.2)	0.9 (0.7 to 1.2)	7.6 (6 to 9.7)	6 (4.7 to 7.5)	1.3 (1 to 1.7)	11.5 (8.8 to 14.9)	43.3 (24 to 67.1)	39.1 (20.9 to 62.3)	51.7 (29.7 to 79.5)
Southeast Asia, east Asia, and Oceania	Oceania	Micronesia (Federated States of)	Deaths	All Ages	0 (0 to 0)	0 (0 to 0)	0 (0 to 0)	0 (0 to 0)	0 (0 to 0)	0 (0 to 0)	-11.2 (-43.4 to 42.3)	28.2 (-38.7 to 141)	-24.3 (-54.6 to 43.2)
Southeast Asia, east Asia, and Oceania	Oceania	Micronesia (Federated States of)	Deaths	Age-standardized	0.3 (0.2 to 0.5)	0.2 (0.1 to 0.3)	0.6 (0.3 to 0.9)	0.2 (0.1 to 0.3)	0.1 (0.1 to 0.2)	0.3 (0.2 to 0.6)	-34.9 (-57.7 to 0.2)	-9.8 (-55.2 to 73)	-43.3 (-66.9 to 3)
Southeast Asia, east Asia, and Oceania	Oceania	Micronesia (Federated States of)	DALYs	All Ages	4 (2 to 5)	1 (0 to 1)	3 (1 to 4)	3 (2 to 5)	1 (1 to 2)	2 (1 to 4)	-4 (-39.9 to 53.5)	23 (-39.5 to 138.8)	-12.4 (-46.1 to 56.7)
Southeast Asia, east Asia, and Oceania	Oceania	Micronesia (Federated States of)	DALYs	Age-standardized	7.1 (4.5 to 10.2)	3.4 (2 to 5.3)	11.3 (6.3 to 17.7)	4.8 (3.3 to 6.9)	2.8 (1.7 to 4.7)	7.2 (4.8 to 11.1)	-31.7 (-54.2 to 3.6)	-15.3 (-56.7 to 56)	-35.9 (-58 to 9.3)
Southeast Asia, east Asia, and Oceania	Oceania	Micronesia (Federated States of)	YLLs	All Ages	3 (2 to 5)	1 (0 to 1)	2 (1 to 4)	3 (2 to 4)	1 (1 to 2)	2 (1 to 3)	-15 (-51.4 to 45.5)	19.4 (-45.5 to 142.9)	-26.4 (-60.8 to 48.6)
Southeast Asia, east Asia, and Oceania	Oceania	Micronesia (Federated States of)	YLLs	Age-standardized	6.4 (3.9 to 9.5)	3.2 (1.9 to 5.2)	10.1 (5.3 to 16.5)	3.9 (2.5 to 6.1)	2.6 (1.5 to 4.5)	5.5 (3.3 to 9.4)	-38.7 (-60.9 to -2.3)	-17.8 (-60.9 to 56.8)	-45.2 (-66.5 to 3.6)
Southeast Asia, east Asia, and Oceania	Oceania	Micronesia (Federated States of)	YLDs	All Ages	0 (0 to 0)	0 (0 to 0)	0 (0 to 0)	1 (0 to 1)	0 (0 to 0)	1 (0 to 1)	110.5 (65.1 to 171.9)	105.1 (76.8 to 142.9)	111.2 (59.1 to 180.4)
Southeast Asia, east Asia, and Oceania	Oceania	Micronesia (Federated States of)	YLDs	Age-standardized	0.6 (0.4 to 1)	0.2 (0.1 to 0.2)	1.2 (0.7 to 1.7)	0.9 (0.6 to 1.3)	0.2 (0.1 to 0.3)	1.7 (1 to 2.6)	38.2 (10 to 74.6)	38.3 (20.5 to 60.7)	45.5 (12.7 to 89.7)
Southeast Asia, east Asia, and Oceania	Oceania	Nauru	Incidence	All Ages	0 (0 to 0)	0 (0 to 0)	0 (0 to 0)	0 (0 to 0)	0 (0 to 0)	0 (0 to 0)	49.8 (30.3 to 74.5)	62.1 (23.8 to 109)	45.1 (22 to 73.4)
Southeast Asia, east Asia, and Oceania	Oceania	Nauru	Incidence	Age-standardized	0.4 (0.4 to 0.5)	0.3 (0.2 to 0.4)	0.6 (0.5 to 0.7)	0.6 (0.5 to 0.7)	0.3 (0.3 to 0.5)	0.8 (0.6 to 1)	30.3 (16.7 to 46.3)	31.1 (8.4 to 59.5)	38.6 (20.7 to 59.6)
Southeast Asia, east Asia, and Oceania	Oceania	Nauru	Prevalence	All Ages	0 (0 to 0)	0 (0 to 0)	0 (0 to 0)	0 (0 to 0)	0 (0 to 0)	0 (0 to 0)	47.8 (22.1 to 79.3)	54.2 (24.9 to 89.2)	47.4 (20.4 to 81.4)
Southeast Asia, east Asia, and Oceania	Oceania	Nauru	Prevalence	Age-standardized	4 (3.2 to 5.2)	0.5 (0.4 to 0.7)	7.2 (5.6 to 9.6)	5.6 (4.4 to 7.1)	0.7 (0.5 to 0.9)	11.4 (8.7 to 14.6)	40.8 (21 to 65.5)	32.8 (12.1 to 59.1)	57.9 (33.7 to 87.3)
Southeast Asia, east Asia, and Oceania	Oceania	Nauru	Deaths	All Ages	0 (0 to 0)	0 (0 to 0)	0 (0 to 0)	0 (0 to 0)	0 (0 to 0)	0 (0 to 0)	-31.9 (-58.1 to 8.5)	-0.4 (-54.2 to 91.8)	-42.9 (-68.8 to 6.3)
Southeast Asia, east Asia, and Oceania	Oceania	Nauru	Deaths	Age-standardized	0.3 (0.2 to 0.4)	0.1 (0.1 to 0.2)	0.4 (0.2 to 0.6)	0.2 (0.1 to 0.3)	0.1 (0.1 to 0.2)	0.2 (0.2 to 0.4)	-34.8 (-59.4 to 7.3)	-10.9 (-55.9 to 60.6)	-39.6 (-67.2 to 14.9)
Southeast Asia, east Asia, and Oceania	Oceania	Nauru	DALYs	All Ages	0 (0 to 0)	0 (0 to 0)	0 (0 to 0)	0 (0 to 0)	0 (0 to 0)	0 (0 to 0)	-17.2 (-48.6 to 34.1)	10.1 (-50.7 to 122.1)	-26.2 (-58.7 to 32.9)
Southeast Asia, east Asia, and Oceania	Oceania	Nauru	DALYs	Age-standardized	5.7 (4.1 to 8)	2.8 (1.7 to 4.5)	8.3 (5.5 to 12.3)	4.1 (2.9 to 5.5)	2.5 (1.2 to 4.1)	6 (4.1 to 8.5)	-27.4 (-51.1 to 6.3)	-10.9 (-53 to 61.2)	-27.4 (-53.9 to 15.1)
Southeast Asia, east Asia, and Oceania	Oceania	Nauru	YLLs	All Ages	0 (0 to 0)	0 (0 to 0)	0 (0 to 0)	0 (0 to 0)	0 (0 to 0)	0 (0 to 0)	-24 (-56.3 to 31.8)	9 (-52.8 to 124.5)	-36.2 (-67.7 to 29.1)
Southeast Asia, east Asia, and Oceania	Oceania	Nauru	YLLs	Age-standardized	5.1 (3.5 to 7.3)	2.7 (1.6 to 4.3)	7.2 (4.5 to 11)	3.3 (2.2 to 4.6)	2.4 (1.1 to 3.9)	4.4 (2.6 to 6.8)	-34.9 (-59.6 to 1.2)	-12.3 (-55.7 to 62)	-39.3 (-66.3 to 7.4)
Southeast Asia, east Asia, and Oceania	Oceania	Nauru	YLDs	All Ages	0 (0 to 0)	0 (0 to 0)	0 (0 to 0)	0 (0 to 0)	0 (0 to 0)	0 (0 to 0)	44.1 (14.2 to 82.6)	53.1 (24.5 to 87.3)	43.5 (11.9 to 84.8)
Southeast Asia, east Asia, and Oceania	Oceania	Nauru	YLDs	Age-standardized	0.6 (0.4 to 0.9)	0.1 (0.1 to 0.1)	1.1 (0.7 to 1.7)	0.8 (0.5 to 1.2)	0.1 (0.1 to 0.2)	1.7 (1 to 2.6)	34.7 (6.7 to 69.2)	31.9 (11.9 to 57.3)	50.4 (14.7 to 94.1)
Southeast Asia, east Asia, and Oceania	Oceania	Niue	Incidence	All Ages	0 (0 to 0)	0 (0 to 0)	0 (0 to 0)	0 (0 to 0)	0 (0 to 0)	0 (0 to 0)	37.1 (20.7 to 57.2)	18 (-3 to 43.1)	48.7 (25.2 to 76)
Southeast Asia, east Asia, and Oceania	Oceania	Niue	Incidence	Age-standardized	0.4 (0.3 to 0.5)	0.3 (0.2 to 0.4)	0.6 (0.5 to 0.7)	0.6 (0.5 to 0.7)	0.4 (0.3 to 0.5)	0.8 (0.6 to 1)	39.4 (23.9 to 58.5)	32.4 (8.5 to 63.2)	39 (21 to 61.8)
Southeast Asia, east Asia, and Oceania	Oceania	Niue	Prevalence	All Ages	0 (0 to 0)	0 (0 to 0)	0 (0 to 0)	0 (0 to 0)	0 (0 to 0)	0 (0 to 0)	64.6 (40.7 to 94.9)	13.7 (-6.9 to 41.2)	70.4 (44.4 to 104.3)
Southeast Asia, east Asia, and Oceania	Oceania	Niue	Prevalence	Age-standardized	3.5 (2.7 to 4.5)	0.6 (0.4 to 0.7)	7.3 (5.6 to 9.5)	5.7 (4.4 to 7.2)	0.8 (0.6 to 1)	11.6 (8.7 to 15)	63 (39.5 to 92.6)	35.3 (11.9 to 65.5)	59.6 (36 to 90.1)
Southeast Asia, east Asia, and Oceania	Oceania	Niue	Deaths	All Ages	0 (0 to 0)	0 (0 to 0)	0 (0 to 0)	0 (0 to 0)	0 (0 to 0)	0 (0 to 0)	-46.8 (-65.2 to -19.2)	-40.5 (-69.4 to 8)	-50.9 (-70.7 to -19.9)
Southeast Asia, east Asia, and Oceania	Oceania	Niue	Deaths	Age-standardized	0.2 (0.1 to 0.2)	0.1 (0.1 to 0.2)	0.3 (0.2 to 0.4)	0.1 (0.1 to 0.2)	0.1 (0 to 0.1)	0.1 (0.1 to 0.2)	-37.1 (-58.4 to -4.7)	-22.9 (-59 to 37.1)	-49.9 (-70.4 to -17.5)
Southeast Asia, east Asia, and Oceania	Oceania	Niue	DALYs	All Ages	0 (0 to 0)	0 (0 to 0)	0 (0 to 0)	0 (0 to 0)	0 (0 to 0)	0 (0 to 0)	-31 (-50.4 to -4)	-33.2 (-62.5 to 16.6)	-29.9 (-51.6 to 0.6)
Southeast Asia, east Asia, and Oceania	Oceania	Niue	DALYs	Age-standardized	3.5 (2.6 to 4.6)	1.9 (1.2 to 3.1)	5.8 (4.1 to 8)	2.6 (1.9 to 3.4)	1.5 (0.8 to 2.5)	3.9 (2.8 to 5.5)	-26.9 (-48 to 1)	-22.5 (-56.6 to 31.9)	-32.6 (-53.1 to -2.1)
Southeast Asia, east Asia, and Oceania	Oceania	Niue	YLLs	All Ages	0 (0 to 0)	0 (0 to 0)	0 (0 to 0)	0 (0 to 0)	0 (0 to 0)	0 (0 to 0)	-46.4 (-64.8 to -18)	-35.6 (-66 to 17)	-52.4 (-70.7 to -22.1)
Southeast Asia, east Asia, and Oceania	Oceania	Niue	YLLs	Age-standardized	3 (2 to 4)	1.9 (1.1 to 3)	4.7 (3 to 6.8)	1.7 (1.1 to 2.5)	1.4 (0.7 to 2.4)	2.2 (1.5 to 3.4)	-41.8 (-62.3 to -11)	-25.2 (-60.1 to 31.4)	-52.4 (-70.5 to -22)
Southeast Asia, east Asia, and Oceania	Oceania	Niue	YLDs	All Ages	0 (0 to 0)	0 (0 to 0)	0 (0 to 0)	0 (0 to 0)	0 (0 to 0)	0 (0 to 0)	56.5 (20.6 to 101.2)	12.2 (-7.8 to 38.7)	61.9 (21.7 to 113.5)
Southeast Asia, east Asia, and Oceania	Oceania	Niue	YLDs	Age-standardized	0.5 (0.3 to 0.8)	0.1 (0.1 to 0.1)	1.1 (0.7 to 1.7)	0.8 (0.5 to 1.3)	0.1 (0.1 to 0.2)	1.7 (1 to 2.6)	54.6 (20.9 to 97)	34.1 (11.6 to 63.3)	50.9 (14.9 to 97.6)
Southeast Asia, east Asia, and Oceania	Oceania	Northern Mariana Islands	Incidence	All Ages	0 (0 to 0)	0 (0 to 0)	0 (0 to 0)	0 (0 to 0)	0 (0 to 0)	0 (0 to 0)	148.9 (98.4 to 214.1)	98.3 (30 to 192.5)	161.6 (95.5 to 238)
Southeast Asia, east Asia, and Oceania	Oceania	Northern Mariana Islands	Incidence	Age-standardized	0.4 (0.3 to 0.5)	0.2 (0.1 to 0.3)	0.6 (0.5 to 0.7)	0.5 (0.4 to 0.6)	0.2 (0.1 to 0.3)	0.8 (0.6 to 1)	17.3 (2.8 to 37.4)	-8.5 (-29.7 to 17.6)	35.8 (16.5 to 61.3)
Southeast Asia, east Asia, and Oceania	Oceania	Northern Mariana Islands	Prevalence	All Ages	1 (1 to 1)	0 (0 to 0)	1 (1 to 1)	3 (2 to 4)	0 (0 to 0)	3 (2 to 4)	265.8 (185.9 to 358)	159.6 (88.5 to 254.8)	270.6 (186 to 370.5)
Southeast Asia, east Asia, and Oceania	Oceania	Northern Mariana Islands	Prevalence	Age-standardized	4.4 (3.4 to 5.6)	0.5 (0.4 to 0.6)	7.2 (5.6 to 9.3)	6.1 (4.6 to 7.8)	0.5 (0.4 to 0.7)	11.5 (8.7 to 15)	39 (16.5 to 67)	10.9 (-10.8 to 42.9)	59.8 (33.1 to 93.4)
Southeast Asia, east Asia, and Oceania	Oceania	Northern Mariana Islands	Deaths	All Ages	0 (0 to 0)	0 (0 to 0)	0 (0 to 0)	0 (0 to 0)	0 (0 to 0)	0 (0 to 0)	-36.7 (-57.8 to -4.5)	-32.5 (-62.5 to 21.8)	-37.3 (-59.6 to -3.8)
Southeast Asia, east Asia, and Oceania	Oceania	Northern Mariana Islands	Deaths	Age-standardized	0.1 (0.1 to 0.2)	0 (0 to 0.1)	0.3 (0.2 to 0.4)	0 (0 to 0)	0 (0 to 0)	0.1 (0 to 0.1)	-79 (-86.5 to -67.9)	-80.1 (-89.1 to -62.3)	-77 (-85.9 to -62.6)
Southeast Asia, east Asia, and Oceania	Oceania	Northern Mariana Islands	DALYs	All Ages	1 (0 to 1)	0 (0 to 0)	0 (0 to 1)	1 (0 to 1)	0 (0 to 0)	1 (0 to 1)	24.5 (-14.6 to 76)	-29.4 (-56.8 to 13.7)	30.7 (-12.2 to 85.8)
Southeast Asia, east Asia, and Oceania	Oceania	Northern Mariana Islands	DALYs	Age-standardized	3.1 (2.4 to 3.9)	0.8 (0.5 to 1.1)	5 (3.8 to 6.5)	1.4 (1 to 1.9)	0.2 (0.2 to 0.3)	2.6 (1.9 to 3.6)	-53.6 (-67.7 to -35.5)	-72.2 (-83 to -55)	-46.8 (-63.4 to -24)
Southeast Asia, east Asia, and Oceania	Oceania	Northern Mariana Islands	YLLs	All Ages	0 (0 to 1)	0 (0 to 0)	0 (0 to 1)	0 (0 to 0)	0 (0 to 0)	0 (0 to 0)	-50.6 (-68.7 to -21.2)	-52.4 (-73.3 to -15.9)	-50.3 (-69.2 to -18.7)
Southeast Asia, east Asia, and Oceania	Oceania	Northern Mariana Islands	YLLs	Age-standardized	2.4 (1.7 to 3.2)	0.7 (0.4 to 1)	3.9 (2.8 to 5.2)	0.5 (0.4 to 0.7)	0.1 (0.1 to 0.2)	1 (0.7 to 1.4)	-77.8 (-85.4 to -65.3)	-81.9 (-89.8 to -67.7)	-75.1 (-83.9 to -60.2)
Southeast Asia, east Asia, and Oceania	Oceania	Northern Mariana Islands	YLDs	All Ages	0 (0 to 0)	0 (0 to 0)	0 (0 to 0)	0 (0 to 1)	0 (0 to 0)	0 (0 to 1)	249.2 (160.6 to 363.3)	161.4 (91.3 to 256.5)	253.2 (160.5 to 372.6)
Southeast Asia, east Asia, and Oceania	Oceania	Northern Mariana Islands	YLDs	Age-standardized	0.7 (0.4 to 1)	0.1 (0.1 to 0.1)	1.1 (0.7 to 1.6)	0.9 (0.6 to 1.4)	0.1 (0.1 to 0.1)	1.7 (1 to 2.6)	32.4 (2.1 to 72.6)	10.7 (-10.7 to 41.9)	52.1 (13.8 to 101.8)
Southeast Asia, east Asia, and Oceania	Oceania	Palau	Incidence	All Ages	0 (0 to 0)	0 (0 to 0)	0 (0 to 0)	0 (0 to 0)	0 (0 to 0)	0 (0 to 0)	169 (143.6 to 198.4)	171.8 (133.3 to 216.5)	168.1 (138.1 to 204.7)
Southeast Asia, east Asia, and Oceania	Oceania	Palau	Incidence	Age-standardized	1.1 (0.9 to 1.2)	0.6 (0.4 to 0.7)	1.6 (1.3 to 1.8)	1.4 (1.2 to 1.7)	0.8 (0.6 to 1)	2 (1.7 to 2.4)	34.6 (25.4 to 44.4)	31.7 (16.6 to 49.1)	30.9 (20.9 to 42.1)
Southeast Asia, east Asia, and Oceania	Oceania	Palau	Prevalence	All Ages	1 (1 to 1)	0 (0 to 0)	1 (1 to 1)	3 (3 to 4)	0 (0 to 0)	3 (2 to 4)	217.5 (182.1 to 255.9)	188.1 (143.3 to 241.9)	219.7 (181.5 to 259.9)
Southeast Asia, east Asia, and Oceania	Oceania	Palau	Prevalence	Age-standardized	10.3 (8.3 to 12.6)	1.8 (1.4 to 2.3)	19.1 (15.2 to 23.6)	15 (12 to 18.4)	2.6 (2 to 3.3)	27.4 (21.6 to 33.9)	46 (32.6 to 62)	47.3 (25.9 to 75.9)	43.5 (29.6 to 60.6)
Southeast Asia, east Asia, and Oceania	Oceania	Palau	Deaths	All Ages	0 (0 to 1)	0 (0 to 0)	0 (0 to 0)	1 (0 to 1)	0 (0 to 0)	0 (0 to 1)	34.3 (-12.6 to 104.9)	44.8 (-26.8 to 175.6)	29.7 (-18.8 to 104.5)
Southeast Asia, east Asia, and Oceania	Oceania	Palau	Deaths	Age-standardized	6.2 (4.3 to 8.6)	3.9 (2.3 to 6.7)	8.5 (5.8 to 11.8)	4.5 (3.3 to 6)	3.1 (1.9 to 4.8)	6.1 (4.3 to 8.3)	-26.7 (-52 to 13.7)	-21.2 (-60.8 to 52.4)	-28.2 (-54.6 to 21.6)
Southeast Asia, east Asia, and Oceania	Oceania	Palau	DALYs	All Ages	11 (8 to 16)	3 (2 to 5)	8 (6 to 12)	15 (11 to 20)	4 (3 to 7)	10 (7 to 15)	32 (-16.1 to 104.8)	42.7 (-27.7 to 167.4)	28 (-21.6 to 108.1)
Southeast Asia, east Asia, and Oceania	Oceania	Palau	DALYs	Age-standardized	111.5 (80.9 to 154.4)	65 (38.6 to 103.4)	159.3 (111 to 220.4)	80.1 (58.3 to 107.5)	48.9 (30.6 to 72.3)	110.9 (79.5 to 150.7)	-28.1 (-52.9 to 7.3)	-24.8 (-60.7 to 38.2)	-30.4 (-55.6 to 10.5)
Southeast Asia, east Asia, and Oceania	Oceania	Palau	YLLs	All Ages	11 (8 to 16)	3 (2 to 5)	8 (5 to 11)	14 (10 to 19)	4 (3 to 7)	10 (7 to 14)	29.6 (-18.9 to 102.2)	42.1 (-28.2 to 167.3)	24.7 (-24.7 to 106.2)
Southeast Asia, east Asia, and Oceania	Oceania	Palau	YLLs	Age-standardized	109.9 (79.4 to 152.9)	64.7 (38.4 to 103.1)	156.4 (108.1 to 217.3)	77.9 (56.3 to 105.4)	48.5 (30.1 to 71.9)	106.9 (75.5 to 146.5)	-29.1 (-53.8 to 7.1)	-25.1 (-60.9 to 38.3)	-31.6 (-56.9 to 9.9)
Southeast Asia, east Asia, and Oceania	Oceania	Palau	YLDs	All Ages	0 (0 to 0)	0 (0 to 0)	0 (0 to 0)	0 (0 to 1)	0 (0 to 0)	0 (0 to 1)	204.7 (136.1 to 299.5)	186.7 (143.3 to 239.8)	206.2 (131.8 to 309.2)
Southeast Asia, east Asia, and Oceania	Oceania	Palau	YLDs	Age-standardized	1.6 (1 to 2.3)	0.3 (0.2 to 0.5)	2.9 (1.8 to 4.3)	2.2 (1.4 to 3.3)	0.4 (0.3 to 0.7)	4 (2.6 to 6)	40.5 (13.6 to 74.4)	46.3 (24.8 to 74)	37.2 (9.3 to 72.8)
Southeast Asia, east Asia, and Oceania	Oceania	Papua New Guinea	Incidence	All Ages	15 (12 to 18)	5 (4 to 7)	9 (7 to 11)	48 (39 to 58)	18 (13 to 25)	30 (24 to 36)	228 (194.2 to 266.3)	240.1 (183.7 to 303.7)	220.9 (179.2 to 269)
Southeast Asia, east Asia, and Oceania	Oceania	Papua New Guinea	Incidence	Age-standardized	0.7 (0.6 to 0.8)	0.5 (0.4 to 0.7)	0.9 (0.7 to 1)	0.9 (0.7 to 1)	0.7 (0.5 to 0.9)	1 (0.8 to 1.2)	22.3 (12.1 to 33.5)	30.8 (14.9 to 51.1)	15.7 (4 to 30.8)
Southeast Asia, east Asia, and Oceania	Oceania	Papua New Guinea	Prevalence	All Ages	90 (71 to 113)	9 (7 to 12)	81 (62 to 103)	329 (256 to 416)	33 (25 to 43)	297 (225 to 383)	266.6 (213.1 to 328.1)	255.4 (207.5 to 316.9)	267.9 (209.2 to 337.4)
Southeast Asia, east Asia, and Oceania	Oceania	Papua New Guinea	Prevalence	Age-standardized	4.8 (3.9 to 5.9)	1.1 (0.9 to 1.4)	8.4 (6.6 to 10.5)	6.9 (5.5 to 8.6)	1.5 (1.2 to 2)	11.8 (9.1 to 15)	43.5 (24.1 to 64.5)	42.2 (23.5 to 66.3)	40.2 (19.3 to 62.9)
Southeast Asia, east Asia, and Oceania	Oceania	Papua New Guinea	Deaths	All Ages	5 (2 to 8)	1 (1 to 2)	4 (1 to 6)	10 (5 to 16)	4 (2 to 6)	7 (2 to 11)	108 (41.3 to 217.5)	184.4 (60.7 to 393.8)	81.9 (17.1 to 190.8)
Southeast Asia, east Asia, and Oceania	Oceania	Papua New Guinea	Deaths	Age-standardized	0.4 (0.2 to 0.7)	0.2 (0.1 to 0.4)	0.7 (0.2 to 1.3)	0.3 (0.1 to 0.5)	0.2 (0.1 to 0.4)	0.5 (0.1 to 0.8)	-20.2 (-46.8 to 17.5)	15.1 (-34.4 to 107.9)	-32.2 (-58.4 to 9.1)
Southeast Asia, east Asia, and Oceania	Oceania	Papua New Guinea	DALYs	All Ages	135 (71 to 204)	34 (19 to 57)	101 (36 to 170)	299 (179 to 446)	99 (57 to 168)	200 (92 to 318)	120.9 (53.3 to 236.3)	186 (59.3 to 410.3)	98.6 (33.4 to 220.3)
Southeast Asia, east Asia, and Oceania	Oceania	Papua New Guinea	DALYs	Age-standardized	7.9 (3.8 to 12.1)	3.8 (2.2 to 6.2)	12.2 (4.2 to 20)	6.6 (3.7 to 9.5)	4.2 (2.5 to 6.7)	9 (3.7 to 14)	-16 (-40.7 to 21.2)	10.9 (-34.9 to 86)	-25.9 (-49.7 to 11.5)
Southeast Asia, east Asia, and Oceania	Oceania	Papua New Guinea	YLLs	All Ages	121 (57 to 189)	33 (18 to 56)	88 (24 to 156)	249 (131 to 405)	93 (52 to 163)	156 (51 to 280)	105.4 (35 to 231.1)	183 (51.7 to 421.5)	76.5 (11.1 to 206.4)
Southeast Asia, east Asia, and Oceania	Oceania	Papua New Guinea	YLLs	Age-standardized	7.2 (3.2 to 11.3)	3.6 (2.1 to 6)	10.9 (2.9 to 18.8)	5.6 (2.8 to 8.6)	4 (2.2 to 6.4)	7.3 (2 to 12.5)	-21.7 (-46.6 to 17)	9.4 (-37.3 to 89.5)	-33.1 (-56.5 to 4.9)
Southeast Asia, east Asia, and Oceania	Oceania	Papua New Guinea	YLDs	All Ages	14 (9 to 21)	1 (1 to 2)	12 (8 to 19)	49 (31 to 75)	5 (3 to 8)	44 (27 to 68)	256.3 (182.2 to 351.3)	253.8 (206.4 to 313.5)	256.6 (175.2 to 362.9)
Southeast Asia, east Asia, and Oceania	Oceania	Papua New Guinea	YLDs	Age-standardized	0.7 (0.5 to 1.1)	0.2 (0.1 to 0.3)	1.3 (0.8 to 1.9)	1 (0.6 to 1.5)	0.2 (0.2 to 0.4)	1.7 (1.1 to 2.6)	39 (10.1 to 78.1)	41.1 (22.8 to 64.3)	35.6 (4.4 to 78.2)
Southeast Asia, east Asia, and Oceania	Oceania	Samoa	Incidence	All Ages	0 (0 to 0)	0 (0 to 0)	0 (0 to 0)	1 (1 to 1)	0 (0 to 0)	1 (1 to 1)	123.8 (96.1 to 155.5)	121.8 (74.8 to 180.4)	124.5 (91 to 161.4)
Southeast Asia, east Asia, and Oceania	Oceania	Samoa	Incidence	Age-standardized	0.4 (0.4 to 0.5)	0.2 (0.2 to 0.3)	0.6 (0.5 to 0.8)	0.6 (0.5 to 0.7)	0.3 (0.2 to 0.4)	0.8 (0.7 to 1)	33.4 (19 to 49.6)	36.2 (11.9 to 69.7)	29.7 (14.1 to 48)
Southeast Asia, east Asia, and Oceania	Oceania	Samoa	Prevalence	All Ages	3 (3 to 4)	0 (0 to 0)	3 (2 to 4)	9 (7 to 11)	1 (0 to 1)	8 (6 to 11)	152 (114.2 to 198.4)	128.8 (88.6 to 180.4)	153.6 (114.1 to 204.9)
Southeast Asia, east Asia, and Oceania	Oceania	Samoa	Prevalence	Age-standardized	3.9 (3.1 to 5)	0.5 (0.4 to 0.7)	7.6 (6 to 9.9)	5.9 (4.5 to 7.6)	0.7 (0.6 to 0.9)	11.4 (8.5 to 14.8)	51 (29 to 77.1)	38.8 (14.9 to 68.9)	49.1 (27 to 75.9)
Southeast Asia, east Asia, and Oceania	Oceania	Samoa	Deaths	All Ages	0 (0 to 0)	0 (0 to 0)	0 (0 to 0)	0 (0 to 0)	0 (0 to 0)	0 (0 to 0)	10.1 (-25.9 to 68.4)	56.8 (-28.8 to 213.3)	-8.4 (-40.9 to 52.7)
Southeast Asia, east Asia, and Oceania	Oceania	Samoa	Deaths	Age-standardized	0.2 (0.2 to 0.3)	0.1 (0.1 to 0.2)	0.4 (0.2 to 0.7)	0.1 (0.1 to 0.2)	0.1 (0.1 to 0.2)	0.2 (0.1 to 0.3)	-38.2 (-60.2 to -3.7)	-12.3 (-61.9 to 76.9)	-53.3 (-72.2 to -19.6)
Southeast Asia, east Asia, and Oceania	Oceania	Samoa	DALYs	All Ages	4 (3 to 5)	1 (1 to 2)	3 (2 to 4)	5 (3 to 6)	2 (1 to 2)	3 (2 to 4)	18.9 (-18.4 to 74.2)	52.2 (-31 to 189.1)	7.1 (-28 to 62.2)
Southeast Asia, east Asia, and Oceania	Oceania	Samoa	DALYs	Age-standardized	4.6 (3.2 to 6.4)	2.3 (1.3 to 4.2)	7.5 (4.7 to 11.2)	3.2 (2.4 to 4.2)	2.1 (1.3 to 3.2)	4.5 (3.3 to 6.1)	-29.7 (-50.2 to 1.2)	-9.5 (-58.6 to 70.8)	-39.5 (-58.6 to -8.1)
Southeast Asia, east Asia, and Oceania	Oceania	Samoa	YLLs	All Ages	3 (2 to 5)	1 (1 to 2)	2 (1 to 4)	3 (2 to 5)	1 (1 to 2)	2 (1 to 3)	-0.7 (-36.3 to 56.8)	49.5 (-34.6 to 190.7)	-21.4 (-50.3 to 31.7)
Southeast Asia, east Asia, and Oceania	Oceania	Samoa	YLLs	Age-standardized	4 (2.6 to 5.8)	2.2 (1.2 to 4.1)	6.4 (3.5 to 10.1)	2.4 (1.7 to 3.2)	2 (1.2 to 3.1)	2.9 (1.8 to 4.2)	-41.1 (-61 to -8)	-11.3 (-60.2 to 73.4)	-54.8 (-71.2 to -25)
Southeast Asia, east Asia, and Oceania	Oceania	Samoa	YLDs	All Ages	1 (0 to 1)	0 (0 to 0)	0 (0 to 1)	1 (1 to 2)	0 (0 to 0)	1 (1 to 2)	144.4 (90.8 to 214.8)	127.7 (88.8 to 177.3)	145.6 (87.4 to 220.9)
Southeast Asia, east Asia, and Oceania	Oceania	Samoa	YLDs	Age-standardized	0.6 (0.4 to 0.9)	0.1 (0.1 to 0.1)	1.2 (0.7 to 1.8)	0.9 (0.5 to 1.3)	0.1 (0.1 to 0.2)	1.7 (1 to 2.5)	46.1 (13.2 to 89.1)	37.8 (14.6 to 67.4)	44.2 (9.7 to 89.7)
Southeast Asia, east Asia, and Oceania	Oceania	Solomon Islands	Incidence	All Ages	1 (1 to 1)	0 (0 to 0)	1 (0 to 1)	3 (2 to 3)	1 (1 to 1)	2 (1 to 2)	184.3 (150.7 to 222)	210.5 (148.8 to 284.2)	171 (132.3 to 216.2)
Southeast Asia, east Asia, and Oceania	Oceania	Solomon Islands	Incidence	Age-standardized	0.6 (0.5 to 0.7)	0.4 (0.3 to 0.6)	0.7 (0.6 to 0.9)	0.7 (0.6 to 0.9)	0.6 (0.4 to 0.7)	0.9 (0.7 to 1.1)	19.4 (7.9 to 31.7)	26.7 (7.9 to 48.9)	20.1 (5.6 to 35.5)
Southeast Asia, east Asia, and Oceania	Oceania	Solomon Islands	Prevalence	All Ages	6 (5 to 8)	0 (0 to 1)	6 (4 to 7)	19 (15 to 24)	2 (1 to 2)	17 (13 to 23)	208.9 (159.5 to 269.8)	222.5 (177.4 to 281.1)	207.7 (157.6 to 273.6)
Southeast Asia, east Asia, and Oceania	Oceania	Solomon Islands	Prevalence	Age-standardized	4.6 (3.7 to 5.8)	0.8 (0.6 to 1)	7.5 (5.9 to 9.7)	6.3 (5 to 7.9)	1.1 (0.9 to 1.4)	11.2 (8.7 to 14.4)	36.8 (18.1 to 59.9)	38.2 (20.2 to 61.8)	49.1 (27.4 to 76.4)
Southeast Asia, east Asia, and Oceania	Oceania	Solomon Islands	Deaths	All Ages	0 (0 to 1)	0 (0 to 0)	0 (0 to 0)	1 (0 to 1)	0 (0 to 0)	0 (0 to 1)	83.3 (15.8 to 200.6)	187.1 (49.8 to 441.5)	61.4 (-0.2 to 192.9)
Southeast Asia, east Asia, and Oceania	Oceania	Solomon Islands	Deaths	Age-standardized	0.4 (0.2 to 0.6)	0.1 (0.1 to 0.2)	0.6 (0.2 to 1)	0.3 (0.2 to 0.5)	0.1 (0.1 to 0.3)	0.5 (0.2 to 0.8)	-17.2 (-46.2 to 37)	18.1 (-35 to 116.7)	-19.4 (-51.9 to 39.7)
Southeast Asia, east Asia, and Oceania	Oceania	Solomon Islands	DALYs	All Ages	11 (6 to 17)	2 (1 to 4)	9 (4 to 15)	21 (14 to 31)	6 (3 to 12)	15 (9 to 25)	94.9 (24.5 to 208.4)	178.4 (41 to 441.5)	75.2 (9.6 to 216)
Southeast Asia, east Asia, and Oceania	Oceania	Solomon Islands	DALYs	Age-standardized	7.9 (4.5 to 12.2)	2.9 (1.5 to 5.5)	11.9 (5.5 to 19.4)	6.7 (4.4 to 9.7)	3.2 (1.8 to 5.9)	9.9 (5.8 to 15.6)	-16.1 (-44 to 28.5)	10.7 (-38 to 99.3)	-16.5 (-45.3 to 38.5)
Southeast Asia, east Asia, and Oceania	Oceania	Solomon Islands	YLLs	All Ages	10 (6 to 16)	2 (1 to 4)	8 (3 to 14)	18 (11 to 29)	6 (3 to 12)	13 (7 to 22)	84.7 (12.2 to 209.5)	176.7 (35.9 to 455.1)	61.6 (-4.9 to 216)
Southeast Asia, east Asia, and Oceania	Oceania	Solomon Islands	YLLs	Age-standardized	7.2 (3.8 to 11.5)	2.8 (1.4 to 5.4)	10.7 (4.3 to 18)	5.7 (3.5 to 8.7)	3 (1.6 to 5.7)	8.2 (4.3 to 13.8)	-20.9 (-49 to 26.1)	9.4 (-40.8 to 102.9)	-23 (-52.1 to 34.8)
Southeast Asia, east Asia, and Oceania	Oceania	Solomon Islands	YLDs	All Ages	1 (1 to 1)	0 (0 to 0)	1 (1 to 1)	3 (2 to 4)	0 (0 to 0)	3 (2 to 4)	201 (132.2 to 286.8)	222.1 (177.4 to 280.1)	199.1 (125.4 to 290.2)
Southeast Asia, east Asia, and Oceania	Oceania	Solomon Islands	YLDs	Age-standardized	0.7 (0.4 to 1)	0.1 (0.1 to 0.2)	1.1 (0.7 to 1.7)	0.9 (0.6 to 1.4)	0.2 (0.1 to 0.3)	1.7 (1 to 2.6)	33.1 (0.3 to 73.6)	37.5 (20.2 to 60.1)	44.3 (6.6 to 92.9)
Southeast Asia, east Asia, and Oceania	Oceania	Tokelau	Incidence	All Ages	0 (0 to 0)	0 (0 to 0)	0 (0 to 0)	0 (0 to 0)	0 (0 to 0)	0 (0 to 0)	41.3 (23.7 to 62.1)	22.2 (-0.1 to 53.5)	51.1 (28.3 to 79)
Southeast Asia, east Asia, and Oceania	Oceania	Tokelau	Incidence	Age-standardized	0.4 (0.3 to 0.5)	0.3 (0.2 to 0.4)	0.6 (0.5 to 0.8)	0.6 (0.5 to 0.7)	0.3 (0.2 to 0.5)	0.8 (0.6 to 1)	37.9 (22.8 to 56.8)	28.9 (7 to 59.4)	33.6 (15.9 to 55.5)
Southeast Asia, east Asia, and Oceania	Oceania	Tokelau	Prevalence	All Ages	0 (0 to 0)	0 (0 to 0)	0 (0 to 0)	0 (0 to 0)	0 (0 to 0)	0 (0 to 0)	71.5 (43 to 103)	24.7 (5.1 to 52.2)	75.3 (45.3 to 110.2)
Southeast Asia, east Asia, and Oceania	Oceania	Tokelau	Prevalence	Age-standardized	3.5 (2.8 to 4.5)	0.5 (0.4 to 0.7)	7.1 (5.5 to 9.3)	6.1 (4.7 to 7.9)	0.7 (0.5 to 0.9)	11.4 (8.6 to 15)	73.6 (46.2 to 105.8)	34.3 (12.5 to 64.7)	60.1 (33.2 to 92.1)
Southeast Asia, east Asia, and Oceania	Oceania	Tokelau	Deaths	All Ages	0 (0 to 0)	0 (0 to 0)	0 (0 to 0)	0 (0 to 0)	0 (0 to 0)	0 (0 to 0)	-46.5 (-66.4 to -13)	-23.4 (-62.2 to 42.5)	-58.4 (-76.1 to -20)
Southeast Asia, east Asia, and Oceania	Oceania	Tokelau	Deaths	Age-standardized	0.2 (0.1 to 0.3)	0.1 (0.1 to 0.2)	0.3 (0.1 to 0.5)	0.1 (0.1 to 0.2)	0.1 (0.1 to 0.2)	0.1 (0.1 to 0.2)	-47.4 (-65.9 to -16.3)	-25.9 (-63.1 to 35.4)	-59.9 (-77.6 to -23.9)
Southeast Asia, east Asia, and Oceania	Oceania	Tokelau	DALYs	All Ages	0 (0 to 0)	0 (0 to 0)	0 (0 to 0)	0 (0 to 0)	0 (0 to 0)	0 (0 to 0)	-35.3 (-55.9 to -4.1)	-28.4 (-64.3 to 32.8)	-39.1 (-60.4 to -0.7)
Southeast Asia, east Asia, and Oceania	Oceania	Tokelau	DALYs	Age-standardized	4.4 (2.9 to 6)	3 (1.8 to 4.8)	6 (3.3 to 9)	2.8 (2.1 to 3.8)	2.1 (1.2 to 3.4)	3.5 (2.5 to 4.7)	-35.5 (-55.8 to -4.6)	-29.8 (-63.7 to 29.8)	-41.3 (-60.7 to -4.5)
Southeast Asia, east Asia, and Oceania	Oceania	Tokelau	YLLs	All Ages	0 (0 to 0)	0 (0 to 0)	0 (0 to 0)	0 (0 to 0)	0 (0 to 0)	0 (0 to 0)	-50.1 (-69.7 to -17.6)	-30 (-66.8 to 33.6)	-63 (-77.5 to -32.5)
Southeast Asia, east Asia, and Oceania	Oceania	Tokelau	YLLs	Age-standardized	3.8 (2.4 to 5.4)	2.9 (1.8 to 4.7)	4.9 (2.3 to 7.9)	1.9 (1.3 to 2.8)	2 (1.1 to 3.3)	1.9 (1.2 to 2.8)	-49.8 (-68.8 to -19.1)	-31.7 (-66.3 to 29.8)	-62.2 (-76.9 to -30.9)
Southeast Asia, east Asia, and Oceania	Oceania	Tokelau	YLDs	All Ages	0 (0 to 0)	0 (0 to 0)	0 (0 to 0)	0 (0 to 0)	0 (0 to 0)	0 (0 to 0)	63.6 (26.8 to 109.5)	24 (4.8 to 51.1)	67.1 (27.2 to 118.4)
Southeast Asia, east Asia, and Oceania	Oceania	Tokelau	YLDs	Age-standardized	0.5 (0.3 to 0.8)	0.1 (0.1 to 0.1)	1.1 (0.7 to 1.7)	0.9 (0.6 to 1.4)	0.1 (0.1 to 0.2)	1.7 (1 to 2.6)	65 (29.9 to 109.4)	33.4 (12.1 to 62.6)	51.7 (17.8 to 94.5)
Southeast Asia, east Asia, and Oceania	Oceania	Tonga	Incidence	All Ages	0 (0 to 0)	0 (0 to 0)	0 (0 to 0)	0 (0 to 1)	0 (0 to 0)	0 (0 to 0)	76.1 (55.7 to 99.4)	73.7 (42.3 to 117.9)	77.1 (53.4 to 104.5)
Southeast Asia, east Asia, and Oceania	Oceania	Tonga	Incidence	Age-standardized	0.4 (0.4 to 0.5)	0.3 (0.2 to 0.4)	0.6 (0.5 to 0.8)	0.6 (0.5 to 0.7)	0.3 (0.2 to 0.4)	0.8 (0.7 to 1)	24.4 (10.8 to 38.7)	16.6 (-2.3 to 40.4)	27.7 (12 to 44.8)
Southeast Asia, east Asia, and Oceania	Oceania	Tonga	Prevalence	All Ages	2 (2 to 3)	0 (0 to 0)	2 (2 to 3)	5 (4 to 6)	0 (0 to 0)	4 (3 to 5)	105.7 (76.4 to 144.6)	113.9 (74.5 to 164.5)	105.1 (73.8 to 147.3)
Southeast Asia, east Asia, and Oceania	Oceania	Tonga	Prevalence	Age-standardized	4 (3.2 to 5.1)	0.6 (0.4 to 0.7)	7.7 (6 to 10.1)	5.7 (4.5 to 7.2)	0.7 (0.6 to 0.9)	11.5 (8.8 to 14.7)	43.2 (24.1 to 68.2)	28 (5.4 to 54.7)	48.7 (27.3 to 75.2)
Southeast Asia, east Asia, and Oceania	Oceania	Tonga	Deaths	All Ages	0 (0 to 0)	0 (0 to 0)	0 (0 to 0)	0 (0 to 0)	0 (0 to 0)	0 (0 to 0)	17.8 (-21.9 to 78.4)	88 (-16 to 271.7)	2.6 (-37.9 to 76.8)
Southeast Asia, east Asia, and Oceania	Oceania	Tonga	Deaths	Age-standardized	0.2 (0.1 to 0.2)	0.1 (0 to 0.1)	0.3 (0.2 to 0.5)	0.1 (0.1 to 0.2)	0 (0 to 0.1)	0.2 (0.1 to 0.3)	-35.8 (-58 to -0.1)	-9.3 (-59.3 to 80.3)	-40.2 (-65.3 to 8.1)
Southeast Asia, east Asia, and Oceania	Oceania	Tonga	DALYs	All Ages	2 (1 to 2)	0 (0 to 0)	1 (1 to 2)	2 (1 to 3)	0 (0 to 1)	2 (1 to 2)	20.8 (-11.9 to 65.3)	44.1 (-26 to 166.3)	15.7 (-19.6 to 73.8)
Southeast Asia, east Asia, and Oceania	Oceania	Tonga	DALYs	Age-standardized	3.2 (2.1 to 4.2)	1 (0.6 to 1.7)	5.8 (3.4 to 8.3)	2.5 (1.8 to 3.4)	0.9 (0.6 to 1.5)	4.5 (3.1 to 6.4)	-21.8 (-43.4 to 6.9)	-9.7 (-53.5 to 65.3)	-22.8 (-47 to 14.9)
Southeast Asia, east Asia, and Oceania	Oceania	Tonga	YLLs	All Ages	1 (1 to 2)	0 (0 to 0)	1 (0 to 2)	1 (1 to 2)	0 (0 to 1)	1 (1 to 1)	-0.1 (-33.4 to 52.8)	37.5 (-35.9 to 176.5)	-9.9 (-43.8 to 54.3)
Southeast Asia, east Asia, and Oceania	Oceania	Tonga	YLLs	Age-standardized	2.6 (1.6 to 3.6)	0.9 (0.5 to 1.6)	4.6 (2.3 to 7)	1.6 (1.1 to 2.4)	0.8 (0.5 to 1.3)	2.8 (1.7 to 4.3)	-36.3 (-57.3 to -4.2)	-13.3 (-60.2 to 72.3)	-39.9 (-63.1 to 1.3)
Southeast Asia, east Asia, and Oceania	Oceania	Tonga	YLDs	All Ages	0 (0 to 1)	0 (0 to 0)	0 (0 to 0)	1 (0 to 1)	0 (0 to 0)	1 (0 to 1)	99.6 (52.4 to 161)	113.8 (75.7 to 162.8)	98.4 (47.9 to 165.8)
Southeast Asia, east Asia, and Oceania	Oceania	Tonga	YLDs	Age-standardized	0.6 (0.4 to 0.9)	0.1 (0.1 to 0.1)	1.2 (0.7 to 1.8)	0.8 (0.5 to 1.3)	0.1 (0.1 to 0.2)	1.7 (1 to 2.7)	39.1 (4.6 to 81.3)	26.9 (5.2 to 52.4)	44 (7.3 to 90.9)
Southeast Asia, east Asia, and Oceania	Oceania	Tuvalu	Incidence	All Ages	0 (0 to 0)	0 (0 to 0)	0 (0 to 0)	0 (0 to 0)	0 (0 to 0)	0 (0 to 0)	108.7 (84.3 to 139)	77.4 (40.6 to 123.7)	126 (94 to 166.9)
Southeast Asia, east Asia, and Oceania	Oceania	Tuvalu	Incidence	Age-standardized	0.4 (0.3 to 0.5)	0.3 (0.2 to 0.4)	0.6 (0.5 to 0.8)	0.6 (0.5 to 0.7)	0.4 (0.3 to 0.5)	0.8 (0.6 to 1)	40.3 (24.6 to 59)	30.9 (7.4 to 59.9)	34.9 (16.5 to 56.9)
Southeast Asia, east Asia, and Oceania	Oceania	Tuvalu	Prevalence	All Ages	0 (0 to 0)	0 (0 to 0)	0 (0 to 0)	1 (0 to 1)	0 (0 to 0)	1 (0 to 1)	153.3 (113.4 to 202.9)	86 (55.3 to 128.6)	159.3 (116.3 to 212.9)
Southeast Asia, east Asia, and Oceania	Oceania	Tuvalu	Prevalence	Age-standardized	3.2 (2.6 to 4.2)	0.5 (0.4 to 0.7)	7.1 (5.5 to 9.3)	5.6 (4.4 to 7.3)	0.7 (0.5 to 0.9)	11.2 (8.6 to 14.7)	73.4 (47.6 to 105.8)	35.3 (12.6 to 64.2)	59 (33.8 to 90)
Southeast Asia, east Asia, and Oceania	Oceania	Tuvalu	Deaths	All Ages	0 (0 to 0)	0 (0 to 0)	0 (0 to 0)	0 (0 to 0)	0 (0 to 0)	0 (0 to 0)	-13.5 (-45.5 to 37.3)	2.9 (-49.1 to 107.1)	-21.4 (-55 to 42.6)
Southeast Asia, east Asia, and Oceania	Oceania	Tuvalu	Deaths	Age-standardized	0.3 (0.2 to 0.4)	0.2 (0.1 to 0.3)	0.6 (0.2 to 1)	0.2 (0.1 to 0.3)	0.1 (0.1 to 0.2)	0.2 (0.1 to 0.4)	-42.7 (-64.8 to -10.4)	-23.9 (-64.7 to 56.5)	-59.6 (-76.6 to -24)
Southeast Asia, east Asia, and Oceania	Oceania	Tuvalu	DALYs	All Ages	0 (0 to 1)	0 (0 to 0)	0 (0 to 0)	0 (0 to 1)	0 (0 to 0)	0 (0 to 0)	-13.4 (-41.7 to 34.7)	-10.8 (-55.1 to 77.3)	-14.5 (-47.4 to 49.1)
Southeast Asia, east Asia, and Oceania	Oceania	Tuvalu	DALYs	Age-standardized	6.3 (4 to 9)	3.4 (2 to 5.8)	10.7 (5.5 to 17.4)	3.7 (2.6 to 5.3)	2.4 (1.4 to 3.9)	5.3 (3.6 to 8.1)	-40.1 (-59 to -9.5)	-30.2 (-63.9 to 37.3)	-50.4 (-68.4 to -12.1)
Southeast Asia, east Asia, and Oceania	Oceania	Tuvalu	YLLs	All Ages	0 (0 to 1)	0 (0 to 0)	0 (0 to 0)	0 (0 to 0)	0 (0 to 0)	0 (0 to 0)	-27.4 (-54.9 to 20.2)	-13.1 (-57.2 to 77.1)	-34.6 (-63.5 to 22.1)
Southeast Asia, east Asia, and Oceania	Oceania	Tuvalu	YLLs	Age-standardized	5.7 (3.5 to 8.6)	3.3 (2 to 5.8)	9.7 (4.5 to 16.5)	2.9 (1.9 to 4.5)	2.2 (1.3 to 3.8)	3.7 (2.1 to 6.3)	-49.3 (-67.8 to -19.6)	-31.9 (-66.1 to 37.4)	-61.8 (-77.4 to -30.7)
Southeast Asia, east Asia, and Oceania	Oceania	Tuvalu	YLDs	All Ages	0 (0 to 0)	0 (0 to 0)	0 (0 to 0)	0 (0 to 0)	0 (0 to 0)	0 (0 to 0)	138.7 (83.7 to 211.4)	85.4 (55.2 to 127)	143.6 (83.9 to 224)
Southeast Asia, east Asia, and Oceania	Oceania	Tuvalu	YLDs	Age-standardized	0.5 (0.3 to 0.8)	0.1 (0.1 to 0.1)	1.1 (0.7 to 1.7)	0.8 (0.5 to 1.3)	0.1 (0.1 to 0.2)	1.6 (1 to 2.5)	63.6 (26.7 to 111.8)	34.5 (12.1 to 62.4)	49.6 (12.9 to 98.1)
Southeast Asia, east Asia, and Oceania	Oceania	Vanuatu	Incidence	All Ages	0 (0 to 1)	0 (0 to 0)	0 (0 to 0)	1 (1 to 2)	1 (0 to 1)	1 (1 to 1)	234.5 (195.8 to 283.4)	278.3 (200 to 378.1)	212.8 (172.8 to 264.7)
Southeast Asia, east Asia, and Oceania	Oceania	Vanuatu	Incidence	Age-standardized	0.6 (0.5 to 0.7)	0.4 (0.3 to 0.5)	0.7 (0.6 to 0.9)	0.8 (0.6 to 0.9)	0.6 (0.4 to 0.7)	0.9 (0.7 to 1.1)	31.3 (19 to 47.2)	43.8 (20.2 to 74.2)	27.9 (13.8 to 46.1)
Southeast Asia, east Asia, and Oceania	Oceania	Vanuatu	Prevalence	All Ages	3 (2 to 4)	0 (0 to 0)	3 (2 to 4)	11 (8 to 14)	1 (1 to 1)	10 (8 to 13)	272.8 (217.8 to 341)	285.9 (215 to 363.2)	271.7 (210.8 to 345.5)
Southeast Asia, east Asia, and Oceania	Oceania	Vanuatu	Prevalence	Age-standardized	4.4 (3.5 to 5.6)	0.7 (0.6 to 0.9)	7.4 (5.7 to 9.6)	6.3 (5 to 8.1)	1 (0.8 to 1.3)	11.1 (8.6 to 14.6)	43.4 (22.3 to 68.1)	44.3 (23.4 to 68.4)	50.9 (27 to 78.8)
Southeast Asia, east Asia, and Oceania	Oceania	Vanuatu	Deaths	All Ages	0 (0 to 0)	0 (0 to 0)	0 (0 to 0)	0 (0 to 0)	0 (0 to 0)	0 (0 to 0)	82.2 (19 to 194.6)	162.1 (37.8 to 389.1)	65.3 (3.6 to 184.8)
Southeast Asia, east Asia, and Oceania	Oceania	Vanuatu	Deaths	Age-standardized	0.3 (0.1 to 0.6)	0.1 (0.1 to 0.3)	0.5 (0.2 to 0.9)	0.2 (0.1 to 0.4)	0.1 (0.1 to 0.2)	0.3 (0.1 to 0.6)	-33.5 (-56.1 to 5.5)	-7.9 (-49.9 to 73)	-35.8 (-61.3 to 9.2)
Southeast Asia, east Asia, and Oceania	Oceania	Vanuatu	DALYs	All Ages	4 (2 to 7)	1 (0 to 2)	4 (2 to 6)	9 (5 to 13)	2 (1 to 4)	7 (4 to 11)	99.2 (27.5 to 215.4)	163.5 (40.9 to 407.1)	85 (14.9 to 211.2)
Southeast Asia, east Asia, and Oceania	Oceania	Vanuatu	DALYs	Age-standardized	6.6 (3.5 to 10.4)	2.4 (1.3 to 4.7)	10 (4.1 to 17.2)	5 (3.1 to 7.5)	2.4 (1.4 to 4.1)	7.4 (3.9 to 12.1)	-24 (-47.9 to 16)	-2.5 (-45.5 to 72.5)	-25.6 (-51.4 to 21)
Southeast Asia, east Asia, and Oceania	Oceania	Vanuatu	YLLs	All Ages	4 (2 to 7)	1 (0 to 1)	3 (1 to 6)	7 (4 to 12)	2 (1 to 4)	5 (2 to 10)	81 (10.9 to 199.4)	157.7 (32.5 to 418.3)	62.7 (-6.5 to 191.8)
Southeast Asia, east Asia, and Oceania	Oceania	Vanuatu	YLLs	Age-standardized	5.9 (2.9 to 9.8)	2.3 (1.2 to 4.5)	8.9 (3.1 to 16.1)	4.1 (2.3 to 6.7)	2.2 (1.2 to 3.9)	5.8 (2.4 to 10.6)	-31 (-54.5 to 12.1)	-4.8 (-48.2 to 74.2)	-34.5 (-59.1 to 11.2)
Southeast Asia, east Asia, and Oceania	Oceania	Vanuatu	YLDs	All Ages	0 (0 to 1)	0 (0 to 0)	0 (0 to 1)	2 (1 to 2)	0 (0 to 0)	1 (1 to 2)	256 (175.9 to 351.1)	284.2 (214.3 to 360.6)	253.6 (167.6 to 356.6)
Southeast Asia, east Asia, and Oceania	Oceania	Vanuatu	YLDs	Age-standardized	0.7 (0.4 to 1)	0.1 (0.1 to 0.2)	1.1 (0.7 to 1.7)	0.9 (0.6 to 1.4)	0.2 (0.1 to 0.2)	1.6 (1 to 2.5)	36.8 (6.7 to 70.5)	43 (22.6 to 66.8)	43.3 (9.3 to 81.7)
		Central Europe, Eastern Europe, and Central Asia	Incidence	All Ages	4,017 (3,442 to 4,681)	651 (525 to 792)	3,366 (2,850 to 3,963)	4,439 (3,781 to 5,166)	985 (782 to 1,220)	3,454 (2,929 to 4,025)	10.5 (1.2 to 18.8)	51.4 (40.6 to 62.9)	2.6 (-6.9 to 11)
		Central Europe, Eastern Europe, and Central Asia	Incidence	Age-standardized	0.9 (0.7 to 1)	0.2 (0.2 to 0.3)	1.6 (1.4 to 1.9)	0.7 (0.6 to 0.9)	0.3 (0.2 to 0.3)	1.3 (1.2 to 1.5)	-12.1 (-19.1 to -6.4)	16.6 (9.7 to 23.4)	-18.6 (-25.4 to -13)
		Central Europe, Eastern Europe, and Central Asia	Prevalence	All Ages	48,287 (40,237 to 58,410)	5,687 (4,496 to 7,179)	42,600 (35,325 to 52,131)	56,322 (47,504 to 66,653)	9,057 (7,010 to 11,542)	47,265 (39,332 to 56,312)	16.6 (5 to 26.1)	59.3 (45.1 to 72.4)	11 (-0.8 to 20.4)
		Central Europe, Eastern Europe, and Central Asia	Prevalence	Age-standardized	10.2 (8.5 to 12.3)	2 (1.6 to 2.5)	23.1 (19.2 to 28.1)	9.1 (7.8 to 10.8)	2.3 (1.8 to 2.9)	18.9 (15.8 to 22.5)	-10.5 (-19 to -4)	15.8 (6.9 to 24)	-18.4 (-26.9 to -12.2)
		Central Europe, Eastern Europe, and Central Asia	Deaths	All Ages	1,405 (1,262 to 1,578)	146 (93 to 173)	1,259 (1,124 to 1,428)	625 (559 to 711)	163 (134 to 231)	461 (412 to 519)	-55.5 (-62.9 to -46.8)	12.2 (-13.2 to 93.5)	-63.4 (-69.6 to -55.4)
		Central Europe, Eastern Europe, and Central Asia	Deaths	Age-standardized	0.3 (0.3 to 0.3)	0.1 (0 to 0.1)	0.8 (0.7 to 0.9)	0.1 (0.1 to 0.1)	0 (0 to 0.1)	0.2 (0.2 to 0.2)	-66.9 (-72.3 to -60.5)	-18.4 (-36.6 to 40.6)	-74.4 (-78.7 to -69.2)
		Central Europe, Eastern Europe, and Central Asia	DALYs	All Ages	38,494 (33,934 to 43,509)	3,680 (2,675 to 4,323)	34,814 (30,661 to 39,602)	20,546 (17,500 to 24,145)	4,198 (3,448 to 5,423)	16,348 (13,793 to 19,543)	-46.6 (-55.1 to -37.5)	14.1 (-6.3 to 69.3)	-53 (-60.9 to -43.6)
		Central Europe, Eastern Europe, and Central Asia	DALYs	Age-standardized	8 (7 to 9)	1.3 (0.9 to 1.5)	19 (16.8 to 21.6)	3.3 (2.8 to 3.9)	1.1 (0.9 to 1.4)	6.5 (5.5 to 7.8)	-58.3 (-64.7 to -51.3)	-11.9 (-27.5 to 31)	-65.8 (-71.4 to -59.3)
		Central Europe, Eastern Europe, and Central Asia	YLLs	All Ages	31,504 (28,085 to 35,648)	2,802 (1,764 to 3,304)	28,702 (25,421 to 32,725)	12,356 (11,087 to 13,984)	2,825 (2,328 to 4,045)	9,532 (8,477 to 10,694)	-60.8 (-67.2 to -53.1)	0.8 (-22.1 to 73.8)	-66.8 (-72.5 to -59.6)
		Central Europe, Eastern Europe, and Central Asia	YLLs	Age-standardized	6.5 (5.8 to 7.3)	1 (0.6 to 1.1)	15.8 (14.1 to 17.9)	2 (1.8 to 2.3)	0.8 (0.6 to 1.1)	3.8 (3.4 to 4.3)	-69.3 (-74.4 to -63.6)	-20.2 (-38.4 to 36.6)	-75.8 (-79.9 to -70.8)
		Central Europe, Eastern Europe, and Central Asia	YLDs	All Ages	6,990 (4,605 to 10,043)	878 (566 to 1,283)	6,111 (3,964 to 8,767)	8,190 (5,507 to 11,708)	1,373 (882 to 1,997)	6,816 (4,553 to 9,632)	17.2 (4.6 to 28.3)	56.4 (40.9 to 71.2)	11.5 (-1.5 to 23.6)
		Central Europe, Eastern Europe, and Central Asia	YLDs	Age-standardized	1.5 (1 to 2.1)	0.3 (0.2 to 0.4)	3.3 (2.2 to 4.7)	1.3 (0.9 to 1.9)	0.3 (0.2 to 0.5)	2.7 (1.8 to 3.8)	-9.8 (-18.9 to -1.8)	14.4 (4.4 to 23.9)	-17.6 (-26.5 to -9.3)
		Central Asia	Incidence	All Ages	212 (177 to 253)	55 (44 to 69)	157 (130 to 189)	402 (336 to 485)	91 (71 to 114)	311 (259 to 378)	89.1 (73.8 to 105.8)	64.3 (47.9 to 85.1)	97.8 (78.6 to 115)
		Central Asia	Incidence	Age-standardized	0.4 (0.4 to 0.5)	0.2 (0.2 to 0.2)	0.7 (0.6 to 0.9)	0.5 (0.4 to 0.6)	0.2 (0.2 to 0.3)	0.9 (0.7 to 1)	18.9 (9.6 to 28)	12.9 (1.1 to 27.5)	17.3 (7.7 to 26.2)
		Central Asia	Prevalence	All Ages	1,448 (1,190 to 1,761)	360 (283 to 453)	1,088 (886 to 1,335)	3,245 (2,643 to 3,978)	589 (453 to 776)	2,656 (2,168 to 3,299)	124.1 (108.3 to 144.3)	63.8 (45.2 to 90.9)	144.1 (123.9 to 166.9)
		Central Asia	Prevalence	Age-standardized	3.2 (2.6 to 3.9)	1.3 (1 to 1.7)	6.3 (5.1 to 7.7)	4.8 (3.9 to 6)	1.6 (1.2 to 2.1)	9.5 (7.8 to 11.8)	51 (40.7 to 64.6)	21.2 (7.3 to 41.5)	51.6 (40.1 to 64.6)
		Central Asia	Deaths	All Ages	81 (63 to 99)	26 (17 to 35)	55 (41 to 71)	96 (75 to 119)	34 (23 to 47)	62 (47 to 81)	18.4 (-16.4 to 67.2)	29.8 (-15.7 to 99.9)	13 (-24.7 to 79.1)
		Central Asia	Deaths	Age-standardized	0.2 (0.1 to 0.2)	0.1 (0.1 to 0.1)	0.3 (0.2 to 0.4)	0.2 (0.1 to 0.2)	0.1 (0.1 to 0.1)	0.2 (0.2 to 0.3)	-12.4 (-37.1 to 25.9)	0.3 (-36.4 to 55.1)	-18.8 (-46.3 to 31.7)
		Central Asia	DALYs	All Ages	2,234 (1,747 to 2,745)	624 (437 to 811)	1,610 (1,211 to 2,091)	2,707 (2,177 to 3,324)	808 (596 to 1,075)	1,899 (1,511 to 2,422)	21.2 (-10.3 to 63.6)	29.5 (-9.1 to 87.6)	17.9 (-18.7 to 73.4)
		Central Asia	DALYs	Age-standardized	4.6 (3.6 to 5.6)	2.2 (1.6 to 2.9)	7.9 (6.1 to 10)	3.8 (3.1 to 4.6)	2.1 (1.5 to 2.8)	6.2 (4.9 to 7.8)	-17.1 (-37.7 to 11.7)	-7.4 (-35.3 to 34.2)	-21.7 (-44.4 to 14.3)
		Central Asia	YLLs	All Ages	2,013 (1,555 to 2,536)	567 (381 to 749)	1,446 (1,063 to 1,930)	2,220 (1,740 to 2,768)	715 (511 to 977)	1,505 (1,160 to 1,990)	10.3 (-22 to 56.2)	26.2 (-15.4 to 90.8)	4.1 (-31.9 to 63.1)
		Central Asia	YLLs	Age-standardized	4.1 (3.2 to 5.1)	2 (1.4 to 2.7)	7 (5.2 to 9)	3.1 (2.4 to 3.8)	1.8 (1.3 to 2.5)	4.8 (3.7 to 6.2)	-24.8 (-46.6 to 7.3)	-10.3 (-39.5 to 34.5)	-31 (-54.1 to 8.6)
		Central Asia	YLDs	All Ages	221 (145 to 317)	57 (37 to 84)	164 (106 to 240)	487 (318 to 708)	93 (58 to 140)	394 (257 to 574)	120.2 (92.7 to 150.4)	63.1 (44.7 to 89.9)	140.1 (102.2 to 182.4)
		Central Asia	YLDs	Age-standardized	0.5 (0.3 to 0.7)	0.2 (0.1 to 0.3)	0.9 (0.6 to 1.3)	0.7 (0.5 to 1)	0.3 (0.2 to 0.4)	1.4 (0.9 to 2)	47.1 (29.4 to 66.6)	20.8 (7.1 to 41)	47.1 (25.3 to 71.6)
Central Europe, eastern Europe, and central Asia	Central Asia	Armenia	Incidence	All Ages	25 (20 to 31)	7 (5 to 9)	18 (14 to 22)	32 (26 to 39)	10 (7 to 13)	22 (18 to 27)	28.2 (11.3 to 48)	41.4 (15.2 to 74.9)	23.1 (5.5 to 45.6)
Central Europe, eastern Europe, and central Asia	Central Asia	Armenia	Incidence	Age-standardized	0.8 (0.7 to 1)	0.4 (0.3 to 0.6)	1.3 (1.1 to 1.6)	0.8 (0.6 to 1)	0.5 (0.3 to 0.6)	1.2 (1 to 1.5)	-3.5 (-14.5 to 8.1)	7.1 (-10.4 to 29.5)	-7.6 (-19.3 to 6.4)
Central Europe, eastern Europe, and central Asia	Central Asia	Armenia	Prevalence	All Ages	100 (83 to 120)	20 (16 to 25)	79 (65 to 97)	204 (165 to 256)	37 (29 to 48)	166 (133 to 215)	104.3 (79.4 to 144.7)	84.6 (50 to 125.2)	109.4 (80.5 to 164)
Central Europe, eastern Europe, and central Asia	Central Asia	Armenia	Prevalence	Age-standardized	3.7 (3.1 to 4.5)	1.3 (1 to 1.7)	7.3 (6 to 8.8)	4.9 (4 to 6.2)	1.6 (1.3 to 2.1)	9.5 (7.7 to 12.2)	30.9 (16.4 to 57.5)	20.7 (-0.7 to 46.5)	30.3 (14.1 to 63.7)
Central Europe, eastern Europe, and central Asia	Central Asia	Armenia	Deaths	All Ages	2 (1 to 3)	0 (0 to 0)	2 (1 to 3)	2 (1 to 2)	0 (0 to 0)	2 (1 to 2)	-18.5 (-44.3 to 17.7)	100.3 (12.1 to 275.5)	-20.3 (-45.8 to 16.1)
Central Europe, eastern Europe, and central Asia	Central Asia	Armenia	Deaths	Age-standardized	0.1 (0.1 to 0.1)	0 (0 to 0)	0.2 (0.2 to 0.3)	0 (0 to 0.1)	0 (0 to 0)	0.1 (0.1 to 0.1)	-51.4 (-66.9 to -30.7)	4.4 (-39.3 to 88.6)	-52.9 (-68.1 to -32.2)
Central Europe, eastern Europe, and central Asia	Central Asia	Armenia	DALYs	All Ages	59 (45 to 74)	4 (3 to 5)	55 (42 to 70)	58 (46 to 75)	7 (4 to 9)	52 (41 to 66)	-0.5 (-26 to 32.6)	75.8 (45.4 to 111.5)	-5.8 (-30.9 to 27.3)
Central Europe, eastern Europe, and central Asia	Central Asia	Armenia	DALYs	Age-standardized	2.2 (1.7 to 2.7)	0.3 (0.2 to 0.4)	5 (3.8 to 6.3)	1.4 (1.1 to 1.8)	0.3 (0.2 to 0.4)	3 (2.4 to 3.8)	-35.3 (-51.4 to -15.8)	13.8 (-5.3 to 35.6)	-40.1 (-55.8 to -20.8)
Central Europe, eastern Europe, and central Asia	Central Asia	Armenia	YLLs	All Ages	43 (31 to 58)	1 (0 to 1)	42 (30 to 57)	28 (21 to 36)	1 (0 to 1)	27 (20 to 35)	-35.6 (-56.6 to -3.7)	30.9 (-20.4 to 133.7)	-36.6 (-57.5 to -4.9)
Central Europe, eastern Europe, and central Asia	Central Asia	Armenia	YLLs	Age-standardized	1.6 (1.1 to 2.1)	0 (0 to 0.1)	3.8 (2.8 to 5)	0.7 (0.5 to 0.8)	0 (0 to 0)	1.6 (1.2 to 2)	-57.8 (-70.8 to -39.6)	-22 (-51.5 to 31.4)	-58.8 (-71.6 to -40.8)
Central Europe, eastern Europe, and central Asia	Central Asia	Armenia	YLDs	All Ages	16 (10 to 23)	3 (2 to 5)	12 (8 to 18)	31 (20 to 46)	6 (4 to 9)	25 (16 to 37)	95.8 (66.8 to 136.7)	84.5 (50 to 125.2)	98.7 (63 to 150.7)
Central Europe, eastern Europe, and central Asia	Central Asia	Armenia	YLDs	Age-standardized	0.6 (0.4 to 0.8)	0.2 (0.1 to 0.3)	1.1 (0.7 to 1.6)	0.7 (0.5 to 1.1)	0.3 (0.2 to 0.4)	1.4 (0.9 to 2.1)	25.1 (7.5 to 51)	20.6 (-0.7 to 46.4)	22.5 (-0.6 to 55.9)
Central Europe, eastern Europe, and central Asia	Central Asia	Azerbaijan	Incidence	All Ages	13 (10 to 16)	3 (2 to 5)	9 (7 to 12)	34 (27 to 44)	7 (5 to 9)	28 (22 to 36)	170.4 (135.6 to 214.1)	107.8 (66.5 to 160.1)	192.3 (154 to 246.2)
Central Europe, eastern Europe, and central Asia	Central Asia	Azerbaijan	Incidence	Age-standardized	0.2 (0.2 to 0.3)	0.1 (0.1 to 0.1)	0.4 (0.3 to 0.5)	0.3 (0.3 to 0.4)	0.1 (0.1 to 0.2)	0.6 (0.4 to 0.7)	40.5 (26.3 to 57.7)	23.7 (0.9 to 54.3)	37.8 (21.9 to 56.7)
Central Europe, eastern Europe, and central Asia	Central Asia	Azerbaijan	Prevalence	All Ages	99 (76 to 126)	25 (18 to 33)	73 (56 to 96)	300 (235 to 381)	51 (36 to 68)	250 (193 to 320)	204.6 (169.7 to 255.3)	100.3 (57.4 to 151.4)	240.5 (193.3 to 302.4)
Central Europe, eastern Europe, and central Asia	Central Asia	Azerbaijan	Prevalence	Age-standardized	2.1 (1.6 to 2.7)	0.9 (0.6 to 1.2)	4.1 (3.1 to 5.4)	3.5 (2.8 to 4.5)	1.1 (0.8 to 1.5)	6.7 (5.2 to 8.8)	69.9 (50.4 to 94.1)	28.7 (1.5 to 61.1)	63.8 (43.1 to 91.1)

Pneumoconiosis

7 GBD super region	21 GBD region	Location	Measure	Age	1990			2019			Percent change (1990 to 2019)		
					Both	Female	Male	Both	Female	Male	Both	Female	Male
Central Europe, eastern Europe, and central Asia	Central Asia	Azerbaijan	Deaths	All Ages	1 (1 to 2)	0 (0 to 1)	1 (0 to 1)	1 (1 to 2)	1 (0 to 1)	1 (0 to 1)	22.4 (-32.9 to 114.5)	52.8 (-45.5 to 284.7)	3.5 (-43.3 to 95)
Central Europe, eastern Europe, and central Asia	Central Asia	Azerbaijan	Deaths	Age-standardized	0 (0 to 0)	0 (0 to 0)	0 (0 to 0.1)	0 (0 to 0)	0 (0 to 0)	0 (0 to 0)	-20.9 (-56.9 to 40.3)	-4.7 (-64.4 to 140.2)	-38 (-66.2 to 23.2)
Central Europe, eastern Europe, and central Asia	Central Asia	Azerbaijan	DALYs	All Ages	46 (34 to 62)	12 (8 to 21)	34 (24 to 47)	77 (55 to 106)	20 (12 to 33)	58 (40 to 80)	68.4 (18 to 129.3)	58.8 (-19.1 to 195)	72 (18.3 to 138.6)
Central Europe, eastern Europe, and central Asia	Central Asia	Azerbaijan	DALYs	Age-standardized	0.9 (0.6 to 1.1)	0.4 (0.3 to 0.7)	1.5 (1.1 to 2)	0.9 (0.6 to 1.2)	0.4 (0.3 to 0.6)	1.5 (1 to 2)	2.9 (-24.9 to 35.7)	-3.1 (-48.2 to 70.5)	-3.7 (-31.2 to 29.6)
Central Europe, eastern Europe, and central Asia	Central Asia	Azerbaijan	YLLs	All Ages	30 (20 to 44)	8 (4 to 16)	22 (14 to 34)	31 (19 to 49)	12 (5 to 24)	20 (11 to 34)	2.8 (-42.4 to 82.6)	39.4 (-51.8 to 256.2)	-11.1 (-54.6 to 73.2)
Central Europe, eastern Europe, and central Asia	Central Asia	Azerbaijan	YLLs	Age-standardized	0.5 (0.4 to 0.7)	0.3 (0.1 to 0.5)	0.9 (0.6 to 1.3)	0.3 (0.2 to 0.5)	0.2 (0.1 to 0.4)	0.5 (0.3 to 0.8)	-34.7 (-62.5 to 13.1)	-19 (-70.2 to 102.9)	-46.3 (-71.1 to -2.5)
Central Europe, eastern Europe, and central Asia	Central Asia	Azerbaijan	YLDs	All Ages	15 (10 to 23)	4 (2 to 6)	11 (7 to 17)	46 (29 to 67)	8 (5 to 12)	38 (23 to 56)	197.6 (156.4 to 252.6)	99.9 (57.4 to 151.1)	231.4 (178.3 to 301.8)
Central Europe, eastern Europe, and central Asia	Central Asia	Azerbaijan	YLDs	Age-standardized	0.3 (0.2 to 0.5)	0.1 (0.1 to 0.2)	0.6 (0.4 to 0.9)	0.5 (0.3 to 0.8)	0.2 (0.1 to 0.3)	1 (0.6 to 1.6)	63.7 (37.8 to 96.7)	28.5 (1.4 to 61)	56.1 (25.6 to 96)
Central Europe, eastern Europe, and central Asia	Central Asia	Georgia	Incidence	All Ages	14 (11 to 18)	4 (3 to 5)	10 (8 to 13)	32 (27 to 39)	9 (7 to 12)	23 (19 to 27)	132.2 (95.4 to 180.5)	157.7 (89 to 255.9)	123.1 (86.3 to 174.1)
Central Europe, eastern Europe, and central Asia	Central Asia	Georgia	Incidence	Age-standardized	0.2 (0.2 to 0.3)	0.1 (0.1 to 0.1)	0.4 (0.3 to 0.5)	0.6 (0.5 to 0.7)	0.3 (0.2 to 0.4)	0.9 (0.8 to 1.1)	167.8 (124.6 to 221.5)	187.3 (112.8 to 290.9)	149.8 (108.6 to 201.4)
Central Europe, eastern Europe, and central Asia	Central Asia	Georgia	Prevalence	All Ages	126 (98 to 163)	33 (23 to 44)	94 (71 to 123)	242 (197 to 298)	53 (40 to 69)	189 (153 to 235)	91.9 (67.2 to 123.8)	62.2 (24.8 to 109.7)	102.3 (73.7 to 142)
Central Europe, eastern Europe, and central Asia	Central Asia	Georgia	Prevalence	Age-standardized	2.1 (1.6 to 2.6)	0.9 (0.7 to 1.2)	4.1 (3.2 to 5.4)	4 (3.3 to 4.9)	1.4 (1.1 to 1.8)	7.9 (6.4 to 9.7)	92.6 (67.7 to 123.4)	57.6 (22.3 to 104)	90.4 (64.6 to 124)
Central Europe, eastern Europe, and central Asia	Central Asia	Georgia	Deaths	All Ages	2 (1 to 2)	0 (0 to 1)	1 (1 to 1)	3 (2 to 4)	1 (1 to 2)	2 (1 to 3)	93.4 (41.5 to 158.9)	132.4 (22.6 to 286.5)	78.4 (24.4 to 146.9)
Central Europe, eastern Europe, and central Asia	Central Asia	Georgia	Deaths	Age-standardized	0 (0 to 0)	0 (0 to 0)	0.1 (0 to 0.1)	0 (0 to 0.1)	0 (0 to 0)	0.1 (0.1 to 0.1)	71.5 (27.3 to 129.3)	83.1 (1.1 to 199.6)	49.7 (8.9 to 104)
Central Europe, eastern Europe, and central Asia	Central Asia	Georgia	DALYs	All Ages	50 (41 to 62)	12 (8 to 17)	38 (30 to 48)	88 (69 to 109)	22 (15 to 33)	66 (50 to 85)	74.9 (42.6 to 114.1)	85.9 (31.8 to 153.9)	71.5 (35.1 to 113.1)
Central Europe, eastern Europe, and central Asia	Central Asia	Georgia	DALYs	Age-standardized	0.8 (0.7 to 1)	0.3 (0.2 to 0.5)	1.6 (1.3 to 2.1)	1.5 (1.1 to 1.8)	0.6 (0.4 to 0.8)	2.7 (2.1 to 3.5)	79 (47.9 to 117.6)	74.9 (26.2 to 134.7)	66.6 (33.1 to 105.8)
Central Europe, eastern Europe, and central Asia	Central Asia	Georgia	YLLs	All Ages	30 (24 to 39)	7 (4 to 11)	24 (18 to 31)	51 (39 to 66)	14 (8 to 24)	37 (27 to 51)	68.4 (22.6 to 131.4)	104.1 (11.1 to 236)	58.3 (8.5 to 126.3)
Central Europe, eastern Europe, and central Asia	Central Asia	Georgia	YLLs	Age-standardized	0.5 (0.4 to 0.6)	0.2 (0.1 to 0.3)	1 (0.8 to 1.3)	0.9 (0.6 to 1.1)	0.3 (0.2 to 0.6)	1.6 (1.2 to 2.1)	74.5 (27.6 to 137.3)	88.4 (6.4 to 206.1)	57.4 (10.7 to 118.2)
Central Europe, eastern Europe, and central Asia	Central Asia	Georgia	YLDs	All Ages	20 (13 to 29)	5 (3 to 8)	15 (9 to 22)	37 (24 to 52)	8 (5 to 13)	28 (18 to 41)	84.9 (56.8 to 122.5)	62.1 (24.9 to 109.5)	92.9 (58.9 to 143.7)
Central Europe, eastern Europe, and central Asia	Central Asia	Georgia	YLDs	Age-standardized	0.3 (0.2 to 0.5)	0.1 (0.1 to 0.2)	0.6 (0.4 to 1)	0.6 (0.4 to 0.9)	0.2 (0.1 to 0.3)	1.2 (0.7 to 1.7)	85.8 (57.3 to 123.8)	57.5 (22.3 to 103.7)	80.7 (47 to 127.4)
Central Europe, eastern Europe, and central Asia	Central Asia	Kazakhstan	Incidence	All Ages	98 (80 to 119)	25 (19 to 32)	73 (59 to 90)	136 (112 to 167)	31 (24 to 42)	105 (86 to 131)	39.5 (23.4 to 57)	26 (5 to 57.2)	44.1 (24.6 to 62.7)
Central Europe, eastern Europe, and central Asia	Central Asia	Kazakhstan	Incidence	Age-standardized	0.7 (0.6 to 0.9)	0.3 (0.2 to 0.4)	1.3 (1.1 to 1.6)	0.7 (0.6 to 0.9)	0.3 (0.2 to 0.4)	1.3 (1.1 to 1.6)	1.1 (-10.2 to 13)	-2.5 (-18.4 to 22.2)	-1 (-12.8 to 10.8)
Central Europe, eastern Europe, and central Asia	Central Asia	Kazakhstan	Prevalence	All Ages	647 (527 to 794)	160 (123 to 206)	488 (392 to 607)	1,152 (934 to 1,463)	214 (160 to 295)	938 (753 to 1,208)	77.9 (60.2 to 100)	33.6 (10.1 to 75.4)	92.4 (69.7 to 116.5)
Central Europe, eastern Europe, and central Asia	Central Asia	Kazakhstan	Prevalence	Age-standardized	5.3 (4.3 to 6.5)	2.1 (1.6 to 2.7)	11.3 (9 to 14.1)	7 (5.6 to 8.8)	2.2 (1.7 to 3.1)	15 (12 to 18.9)	32.3 (20 to 48.3)	6.8 (-11.7 to 40.4)	32.6 (18.8 to 47.6)
Central Europe, eastern Europe, and central Asia	Central Asia	Kazakhstan	Deaths	All Ages	67 (50 to 85)	22 (14 to 31)	45 (31 to 60)	72 (51 to 96)	24 (13 to 37)	47 (33 to 66)	7.8 (-27.5 to 63.4)	10.6 (-37.6 to 83.8)	6.4 (-33.8 to 82.6)
Central Europe, eastern Europe, and central Asia	Central Asia	Kazakhstan	Deaths	Age-standardized	0.5 (0.4 to 0.7)	0.3 (0.2 to 0.4)	1 (0.7 to 1.3)	0.5 (0.3 to 0.6)	0.3 (0.1 to 0.4)	0.8 (0.6 to 1.1)	-13.8 (-41.8 to 28.4)	-9.5 (-49.2 to 49.4)	-16.4 (-48.4 to 45.3)
Central Europe, eastern Europe, and central Asia	Central Asia	Kazakhstan	DALYs	All Ages	1,772 (1,318 to 2,287)	503 (335 to 684)	1,269 (889 to 1,740)	1,773 (1,292 to 2,328)	516 (314 to 764)	1,256 (891 to 1,718)	0 (-32.6 to 48.2)	2.6 (-35.2 to 60.5)	-1 (-37.4 to 61.5)
Central Europe, eastern Europe, and central Asia	Central Asia	Kazakhstan	DALYs	Age-standardized	13.2 (10 to 16.7)	6.3 (4.3 to 8.6)	23.9 (17.2 to 31.7)	10.3 (7.7 to 13.5)	5.2 (3.2 to 7.8)	18.2 (13.2 to 24.6)	-21.7 (-46.4 to 14.2)	-17.6 (-48 to 29)	-23.9 (-50.2 to 23.8)
Central Europe, eastern Europe, and central Asia	Central Asia	Kazakhstan	YLLs	All Ages	1,676 (1,225 to 2,189)	478 (309 to 660)	1,198 (820 to 1,666)	1,604 (1,148 to 2,141)	482 (284 to 725)	1,121 (774 to 1,572)	-4.3 (-36.7 to 46.6)	1 (-38.2 to 62.4)	-6.4 (-43.5 to 60.6)
Central Europe, eastern Europe, and central Asia	Central Asia	Kazakhstan	YLLs	Age-standardized	12.4 (9.3 to 16)	6 (3.9 to 8.3)	22.3 (15.8 to 30.1)	9.3 (6.7 to 12.4)	4.9 (2.8 to 7.5)	16.1 (11.2 to 22.3)	-24.9 (-50.4 to 13.5)	-18.9 (-50.7 to 31.5)	-27.9 (-55.1 to 22.3)
Central Europe, eastern Europe, and central Asia	Central Asia	Kazakhstan	YLDs	All Ages	96 (61 to 140)	26 (16 to 38)	71 (43 to 108)	169 (106 to 251)	34 (21 to 52)	135 (82 to 202)	75.1 (33.8 to 128.9)	32.9 (9.9 to 73.9)	90.3 (36.6 to 165.6)
Central Europe, eastern Europe, and central Asia	Central Asia	Kazakhstan	YLDs	Age-standardized	0.8 (0.5 to 1.1)	0.3 (0.2 to 0.5)	1.6 (1 to 2.4)	1 (0.7 to 1.5)	0.4 (0.2 to 0.5)	2.1 (1.3 to 3.1)	29.8 (2.6 to 66.8)	6.2 (-11.9 to 39.5)	30.9 (-3.7 to 80.3)
Central Europe, eastern Europe, and central Asia	Central Asia	Kyrgyzstan	Incidence	All Ages	8 (6 to 10)	2 (1 to 3)	6 (4 to 7)	17 (13 to 21)	4 (3 to 5)	13 (10 to 17)	116.8 (92.6 to 145.4)	79.7 (40 to 130)	129.7 (102.5 to 164.3)
Central Europe, eastern Europe, and central Asia	Central Asia	Kyrgyzstan	Incidence	Age-standardized	0.2 (0.2 to 0.3)	0.1 (0.1 to 0.1)	0.4 (0.3 to 0.6)	0.3 (0.3 to 0.4)	0.1 (0.1 to 0.2)	0.6 (0.5 to 0.7)	39.6 (24.4 to 56.3)	26.7 (-0.2 to 62.7)	35.4 (20.6 to 52.9)
Central Europe, eastern Europe, and central Asia	Central Asia	Kyrgyzstan	Prevalence	All Ages	62 (49 to 80)	16 (12 to 22)	46 (35 to 60)	147 (116 to 188)	27 (20 to 36)	120 (94 to 155)	135.7 (107.5 to 172.4)	65.7 (22.8 to 110.9)	160.7 (124.1 to 207.4)
Central Europe, eastern Europe, and central Asia	Central Asia	Kyrgyzstan	Prevalence	Age-standardized	2.1 (1.6 to 2.7)	0.9 (0.6 to 1.2)	4.2 (3.2 to 5.5)	3.5 (2.7 to 4.5)	1.1 (0.8 to 1.5)	6.9 (5.4 to 9)	67 (47.9 to 92.2)	28.9 (-6.1 to 64.2)	63.6 (42.1 to 91)
Central Europe, eastern Europe, and central Asia	Central Asia	Kyrgyzstan	Deaths	All Ages	0 (0 to 0)	0 (0 to 0)	0 (0 to 0)	0 (0 to 0)	0 (0 to 0)	0 (0 to 0)	-10.4 (-31.7 to 20.5)	75 (10.6 to 319.1)	-34.2 (-51.1 to -9.4)
Central Europe, eastern Europe, and central Asia	Central Asia	Kyrgyzstan	Deaths	Age-standardized	0 (0 to 0)	0 (0 to 0)	0 (0 to 0)	0 (0 to 0)	0 (0 to 0)	0 (0 to 0)	-38.2 (-53.2 to -17.4)	33.1 (-14.3 to 212.2)	-61.4 (-71.1 to -48.5)
Central Europe, eastern Europe, and central Asia	Central Asia	Kyrgyzstan	DALYs	All Ages	17 (13 to 22)	4 (3 to 6)	13 (10 to 17)	29 (21 to 41)	7 (5 to 10)	22 (16 to 32)	74.7 (44.5 to 107.3)	70.8 (33.7 to 148.1)	75.9 (39 to 116.8)
Central Europe, eastern Europe, and central Asia	Central Asia	Kyrgyzstan	DALYs	Age-standardized	0.6 (0.4 to 0.7)	0.2 (0.2 to 0.3)	1.1 (0.8 to 1.5)	0.7 (0.5 to 0.9)	0.3 (0.2 to 0.4)	1.2 (0.8 to 1.8)	20.7 (-0.9 to 44.1)	27.5 (0.4 to 78.8)	8.8 (-15 to 35.4)
Central Europe, eastern Europe, and central Asia	Central Asia	Kyrgyzstan	YLLs	All Ages	7 (6 to 9)	2 (1 to 2)	6 (4 to 7)	7 (6 to 9)	3 (2 to 5)	4 (3 to 5)	-1.8 (-25.8 to 38.4)	79.5 (13.8 to 341.5)	-24.1 (-44.2 to 5.8)
Central Europe, eastern Europe, and central Asia	Central Asia	Kyrgyzstan	YLLs	Age-standardized	0.2 (0.2 to 0.3)	0.1 (0 to 0.1)	0.5 (0.4 to 0.6)	0.1 (0.1 to 0.2)	0.1 (0.1 to 0.2)	0.2 (0.2 to 0.2)	-38.4 (-53.2 to -16.1)	25.1 (-20 to 200)	-58.6 (-69.3 to -43.5)
Central Europe, eastern Europe, and central Asia	Central Asia	Kyrgyzstan	YLDs	All Ages	10 (6 to 14)	3 (2 to 4)	7 (5 to 11)	22 (14 to 34)	4 (3 to 7)	18 (11 to 28)	130 (94.2 to 170.2)	65.7 (22.8 to 110.8)	153 (108.2 to 207.4)
Central Europe, eastern Europe, and central Asia	Central Asia	Kyrgyzstan	YLDs	Age-standardized	0.3 (0.2 to 0.5)	0.1 (0.1 to 0.2)	0.7 (0.4 to 1)	0.5 (0.3 to 0.8)	0.2 (0.1 to 0.3)	1 (0.6 to 1.6)	61.5 (33.8 to 92.9)	28.9 (-6.1 to 64.1)	56.3 (24.4 to 96.5)
Central Europe, eastern Europe, and central Asia	Central Asia	Mongolia	Incidence	All Ages	9 (7 to 11)	2 (2 to 3)	7 (5 to 8)	12 (10 to 14)	2 (2 to 3)	9 (8 to 12)	28.6 (6.2 to 58)	1.9 (-29 to 46.1)	37.5 (11.8 to 70)
Central Europe, eastern Europe, and central Asia	Central Asia	Mongolia	Incidence	Age-standardized	0.7 (0.6 to 0.9)	0.3 (0.2 to 0.4)	1.2 (1 to 1.5)	0.5 (0.4 to 0.6)	0.2 (0.1 to 0.2)	0.8 (0.7 to 1)	-38 (-47.1 to -26.8)	-48.9 (-62.9 to -29.8)	-31.4 (-41.6 to -19.5)
Central Europe, eastern Europe, and central Asia	Central Asia	Mongolia	Prevalence	All Ages	35 (28 to 44)	7 (5 to 9)	28 (22 to 35)	84 (68 to 104)	13 (10 to 18)	71 (57 to 88)	141.7 (112.5 to 175.1)	88.8 (52.9 to 133.7)	155.4 (120.3 to 197)
Central Europe, eastern Europe, and central Asia	Central Asia	Mongolia	Prevalence	Age-standardized	3.3 (2.6 to 4.1)	1.2 (0.9 to 1.5)	6.1 (4.8 to 7.8)	4.1 (3.3 to 5.1)	1.2 (0.9 to 1.6)	8.4 (6.8 to 10.5)	24.9 (11.2 to 39.1)	-0.7 (-20 to 24.2)	37.4 (20.3 to 56)
Central Europe, eastern Europe, and central Asia	Central Asia	Mongolia	Deaths	All Ages	2 (1 to 3)	0 (0 to 0)	2 (1 to 3)	2 (1 to 3)	0 (0 to 0)	2 (1 to 3)	-3.9 (-47 to 109.1)	-1 (-56.5 to 125.9)	-4 (-47.4 to 115.2)
Central Europe, eastern Europe, and central Asia	Central Asia	Mongolia	Deaths	Age-standardized	0.3 (0.1 to 0.4)	0 (0 to 0)	0.6 (0.4 to 1)	0.1 (0.1 to 0.2)	0 (0 to 0)	0.3 (0.2 to 0.5)	-49.6 (-72.2 to 9.8)	-61.5 (-81.8 to -18.2)	-47.8 (-71.7 to 15.3)
Central Europe, eastern Europe, and central Asia	Central Asia	Mongolia	DALYs	All Ages	48 (30 to 70)	3 (2 to 5)	45 (28 to 66)	57 (40 to 83)	4 (3 to 6)	53 (37 to 79)	18.6 (-28.3 to 126.8)	32.2 (-19.7 to 118.8)	17.8 (-31.1 to 134)
Central Europe, eastern Europe, and central Asia	Central Asia	Mongolia	DALYs	Age-standardized	4.9 (3.1 to 7.1)	0.5 (0.3 to 0.8)	11.3 (6.8 to 16.4)	2.8 (2 to 4.1)	0.3 (0.2 to 0.4)	6.7 (4.7 to 9.6)	-42.9 (-65.4 to 8.9)	-40.7 (-63.9 to -6.6)	-41.2 (-64.8 to 17.6)
Central Europe, eastern Europe, and central Asia	Central Asia	Mongolia	YLLs	All Ages	43 (25 to 63)	2 (1 to 3)	41 (23 to 62)	44 (28 to 70)	2 (1 to 4)	42 (27 to 68)	3.3 (-44.6 to 120.9)	-2.8 (-60.5 to 141.3)	3.6 (-45.3 to 131)
Central Europe, eastern Europe, and central Asia	Central Asia	Mongolia	YLLs	Age-standardized	4.4 (2.6 to 6.5)	0.3 (0.2 to 0.6)	10.4 (6 to 15.4)	2.2 (1.4 to 3.4)	0.1 (0.1 to 0.2)	5.4 (3.6 to 8.2)	-50.5 (-72.6 to 7)	-65.6 (-85.5 to -16.7)	-48.1 (-71.2 to 13.2)
Central Europe, eastern Europe, and central Asia	Central Asia	Mongolia	YLDs	All Ages	5 (3 to 8)	1 (1 to 2)	4 (3 to 6)	13 (8 to 19)	2 (1 to 3)	11 (7 to 16)	141.1 (97.5 to 194.2)	88.8 (53 to 133.7)	155 (100.2 to 222.8)
Central Europe, eastern Europe, and central Asia	Central Asia	Mongolia	YLDs	Age-standardized	0.5 (0.3 to 0.7)	0.2 (0.1 to 0.3)	0.9 (0.6 to 1.4)	0.6 (0.4 to 0.9)	0.2 (0.1 to 0.3)	1.3 (0.8 to 1.9)	23.6 (0.3 to 51.7)	-0.8 (-20 to 24.1)	35.8 (3.8 to 75.7)
Central Europe, eastern Europe, and central Asia	Central Asia	Tajikistan	Incidence	All Ages	9 (7 to 11)	2 (1 to 3)	7 (5 to 9)	23 (19 to 29)	5 (3 to 7)	19 (15 to 24)	165.2 (135.7 to 204.7)	118.3 (70.2 to 172.1)	180.4 (145.4 to 226.6)
Central Europe, eastern Europe, and central Asia	Central Asia	Tajikistan	Incidence	Age-standardized	0.3 (0.2 to 0.4)	0.1 (0.1 to 0.2)	0.5 (0.4 to 0.6)	0.4 (0.3 to 0.5)	0.2 (0.1 to 0.2)	0.7 (0.5 to 0.8)	38.4 (23.6 to 56.1)	27.6 (1.8 to 57.7)	33.4 (19.2 to 51.7)
Central Europe, eastern Europe, and central Asia	Central Asia	Tajikistan	Prevalence	All Ages	63 (49 to 80)	15 (11 to 19)	49 (37 to 63)	168 (131 to 213)	26 (19 to 34)	142 (109 to 181)	165.9 (133 to 208.4)	76.4 (40.8 to 118.1)	192.7 (150.3 to 247.2)
Central Europe, eastern Europe, and central Asia	Central Asia	Tajikistan	Prevalence	Age-standardized	2.3 (1.8 to 3)	0.9 (0.7 to 1.2)	4.3 (3.2 to 5.6)	3.8 (3 to 4.9)	1.2 (0.8 to 1.6)	6.9 (5.3 to 8.9)	65.9 (45.6 to 92.2)	26 (0.7 to 59.9)	61.7 (39.3 to 89.3)
Central Europe, eastern Europe, and central Asia	Central Asia	Tajikistan	Deaths	All Ages	0 (0 to 0)	0 (0 to 0)	0 (0 to 0)	0 (0 to 0)	0 (0 to 0)	0 (0 to 0)	8.5 (-36 to 85.4)	2.9 (-46.9 to 139.9)	10.4 (-39.5 to 115.4)
Central Europe, eastern Europe, and central Asia	Central Asia	Tajikistan	Deaths	Age-standardized	0 (0 to 0)	0 (0 to 0)	0 (0 to 0)	0 (0 to 0)	0 (0 to 0)	0 (0 to 0)	-9.8 (-46.5 to 54.2)	4 (-47.2 to 132)	-17.8 (-53.9 to 65.7)
Central Europe, eastern Europe, and central Asia	Central Asia	Tajikistan	DALYs	All Ages	13 (9 to 18)	3 (2 to 4)	10 (7 to 14)	30 (20 to 42)	5 (3 to 7)	25 (16 to 36)	123.1 (83.1 to 169.3)	59.1 (24.8 to 101.9)	142.2 (91.9 to 200.9)
Central Europe, eastern Europe, and central Asia	Central Asia	Tajikistan	DALYs	Age-standardized	0.5 (0.3 to 0.7)	0.2 (0.1 to 0.3)	0.9 (0.6 to 1.2)	0.7 (0.4 to 1)	0.2 (0.1 to 0.3)	1.2 (0.8 to 1.7)	39.2 (12.6 to 70.9)	16.6 (-8.5 to 49.1)	33.3 (3.6 to 68.4)
Central Europe, eastern Europe, and central Asia	Central Asia	Tajikistan	YLLs	All Ages	3 (2 to 5)	1 (0 to 1)	3 (1 to 4)	4 (3 to 6)	1 (1 to 1)	3 (2 to 5)	15.6 (-32.5 to 93.9)	8.4 (-39.8 to 140.7)	17.7 (-35.8 to 124.3)
Central Europe, eastern Europe, and central Asia	Central Asia	Tajikistan	YLLs	Age-standardized	0.1 (0.1 to 0.2)	0 (0 to 0.1)	0.2 (0.1 to 0.4)	0.1 (0.1 to 0.1)	0 (0 to 0.1)	0.2 (0.1 to 0.2)	-21 (-53.8 to 37.8)	-11.9 (-54.7 to 100.5)	-28.3 (-60 to 36.4)
Central Europe, eastern Europe, and central Asia	Central Asia	Tajikistan	YLDs	All Ages	10 (6 to 15)	2 (1 to 3)	8 (5 to 11)	26 (16 to 38)	4 (2 to 6)	22 (13 to 33)	160.4 (120.5 to 206.3)	76.4 (40.8 to 118.1)	185.5 (135.2 to 242.2)
Central Europe, eastern Europe, and central Asia	Central Asia	Tajikistan	YLDs	Age-standardized	0.4 (0.2 to 0.5)	0.1 (0.1 to 0.2)	0.7 (0.4 to 1)	0.6 (0.4 to 0.9)	0.2 (0.1 to 0.3)	1 (0.6 to 1.5)	59.6 (32.6 to 94.8)	26 (0.6 to 59.9)	54.1 (22.9 to 92.4)
Central Europe, eastern Europe, and central Asia	Central Asia	Turkmenistan	Incidence	All Ages	5 (4 to 7)	1 (1 to 2)	4 (3 to 5)	14 (11 to 17)	3 (2 to 4)	11 (8 to 13)	161.1 (126.7 to 202.1)	107.7 (64.9 to 154.8)	181.1 (138.1 to 231.2)
Central Europe, eastern Europe, and central Asia	Central Asia	Turkmenistan	Incidence	Age-standardized	0.2 (0.2 to 0.3)	0.1 (0.1 to 0.2)	0.4 (0.3 to 0.5)	0.3 (0.3 to 0.4)	0.1 (0.1 to 0.2)	0.5 (0.4 to 0.7)	30.6 (15.4 to 47.8)	16.5 (-4.9 to 40.7)	30.3 (13.2 to 50.4)
Central Europe, eastern Europe, and central Asia	Central Asia	Turkmenistan	Prevalence	All Ages	38 (29 to 49)	10 (7 to 13)	28 (21 to 37)	120 (95 to 152)	22 (16 to 30)	98 (77 to 125)	217.8 (172.2 to 269.5)	130 (82.6 to 185.7)	248 (190.6 to 314.5)
Central Europe, eastern Europe, and central Asia	Central Asia	Turkmenistan	Prevalence	Age-standardized	2.1 (1.6 to 2.7)	0.9 (0.7 to 1.2)	4 (3 to 5.2)	3.4 (2.7 to 4.2)	1.1 (0.8 to 1.5)	6.5 (5.1 to 8.2)	61.6 (39.9 to 85.5)	25.1 (-0.6 to 59)	62.2 (38.1 to 91.2)
Central Europe, eastern Europe, and central Asia	Central Asia	Turkmenistan	Deaths	All Ages	1 (0 to 1)	0 (0 to 0)	0 (0 to 1)	0 (0 to 1)	0 (0 to 0)	0 (0 to 0)	-19.9 (-48.4 to 41.6)	-13.8 (-58 to 198.8)	-22.6 (-51.5 to 28.3)
Central Europe, eastern Europe, and central Asia	Central Asia	Turkmenistan	Deaths	Age-standardized	0 (0 to 0)	0 (0 to 0)	0.1 (0 to 0.1)	0 (0 to 0)	0 (0 to 0)	0 (0 to 0)	-61 (-75 to -30.5)	-56.4 (-78.9 to 55.9)	-63.6 (-76.7 to -41.8)
Central Europe, eastern Europe, and central Asia	Central Asia	Turkmenistan	DALYs	All Ages	20 (15 to 26)	5 (4 to 8)	14 (11 to 20)	30 (22 to 41)	7 (5 to 14)	23 (16 to 32)	53.6 (11.7 to 107.9)	33.3 (-15.2 to 189.5)	61.1 (13 to 122.8)
Central Europe, eastern Europe, and central Asia	Central Asia	Turkmenistan	DALYs	Age-standardized	1 (0.8 to 1.3)	0.5 (0.3 to 0.7)	1.8 (1.4 to 2.5)	0.8 (0.6 to 1.1)	0.3 (0.2 to 0.7)	1.4 (1 to 2)	-22.4 (-43.3 to 4.9)	-28.6 (-54.1 to 52.4)	-23.4 (-45.2 to 5.4)
Central Europe, eastern Europe, and central Asia	Central Asia	Turkmenistan	YLLs	All Ages	14 (10 to 19)	4 (2 to 6)	10 (7 to 15)	12 (8 to 18)	4 (2 to 10)	8 (5 to 12)	-13.9 (-44.1 to 50)	-4.9 (-52.8 to 216)	-17.3 (-48.7 to 40.8)
Central Europe, eastern Europe, and central Asia	Central Asia	Turkmenistan	YLLs	Age-standardized	0.7 (0.5 to 1)	0.3 (0.2 to 0.5)	1.2 (0.8 to 1.8)	0.3 (0.2 to 0.4)	0.2 (0.1 to 0.5)	0.4 (0.3 to 0.6)	-59 (-73.4 to -28.3)	-51.2 (-76.2 to 64.2)	-63.6 (-77.1 to -40.2)
Central Europe, eastern Europe, and central Asia	Central Asia	Turkmenistan	YLDs	All Ages	6 (4 to 9)	2 (1 to 2)	4 (3 to 7)	18 (12 to 27)	4 (2 to 5)	15 (9 to 22)	210.2 (158.2 to 269.1)	129.6 (82.6 to 185.6)	238.1 (171.7 to 321)
Central Europe, eastern Europe, and central Asia	Central Asia	Turkmenistan	YLDs	Age-standardized	0.3 (0.2 to 0.5)	0.1 (0.1 to 0.2)	0.6 (0.4 to 0.9)	0.5 (0.3 to 0.8)	0.2 (0.1 to 0.3)	1 (0.6 to 1.5)	56.2 (28.9 to 90.4)	24.9 (-0.6 to 58.9)	55 (22 to 95.7)
Central Europe, eastern Europe, and central Asia	Central Asia	Uzbekistan	Incidence	All Ages	32 (26 to 40)	9 (6 to 12)	23 (19 to 30)	101 (80 to 126)	20 (14 to 27)	81 (63 to 102)	214.3 (167.3 to 262.3)	128.4 (73.7 to 191)	246.1 (191.9 to 305.1)
Central Europe, eastern Europe, and central Asia	Central Asia	Uzbekistan	Incidence	Age-standardized	0.3 (0.2 to 0.3)	0.1 (0.1 to 0.2)	0.4 (0.4 to 0.5)	0.4 (0.3 to 0.5)	0.2 (0.1 to 0.2)	0.7 (0.6 to 0.9)	62.3 (42.2 to 83.3)	33.8 (4.5 to 69.1)	66.7 (44.3 to 90.7)
Central Europe, eastern Europe, and central Asia	Central Asia	Uzbekistan	Prevalence	All Ages	278 (224 to 343)	74 (54 to 97)	204 (161 to 257)	828 (655 to 1,045)	146 (104 to 202)	682 (529 to 874)	197.9 (153 to 246.6)	97.4 (51.2 to 155)	234.3 (174.9 to 300.4)
Central Europe, eastern Europe, and central Asia	Central Asia	Uzbekistan	Prevalence	Age-standardized	2.6 (2 to 3.1)	1.1 (0.8 to 1.5)	4.8 (3.8 to 5.9)	4.8 (3.8 to 5.9)	1.7 (1.2 to 2.3)	9.1 (7.2 to 11.4)	86.9 (62.1 to 113.6)	44.4 (9.4 to 85.2)	91.4 (62 to 123.8)
Central Europe, eastern Europe, and central Asia	Central Asia	Uzbekistan	Deaths	All Ages	6 (4 to 8)	3 (1 to 4)	4 (2 to 5)	15 (11 to 20)	7 (5 to 12)	8 (6 to 10)	138.9 (57.4 to 291.1)	176.7 (56.6 to 630.9)	112 (23.3 to 287.1)
Central Europe, eastern Europe, and central Asia	Central Asia	Uzbekistan	Deaths	Age-standardized	0.1 (0 to 0.1)	0 (0 to 0.1)	0.1 (0 to 0.1)	0.1 (0.1 to 0.1)	0.1 (0 to 0.1)	0.1 (0.1 to 0.1)	52.3 (-2.5 to 151)	79 (-2.1 to 422)	31.4 (-21.5 to 137.7)
Central Europe, eastern Europe, and central Asia	Central Asia	Uzbekistan	DALYs	All Ages	209 (152 to 264)	77 (45 to 104)	132 (93 to 180)	564 (446 to 709)	220 (156 to 348)	344 (273 to 435)	169.5 (94.1 to 284.2)	186.2 (80.1 to 532.4)	159.8 (71.7 to 295.7)
Central Europe, eastern Europe, and central Asia	Central Asia	Uzbekistan	DALYs	Age-standardized	1.8 (1.3 to 2.3)	1.2 (0.7 to 1.6)	2.6 (1.8 to 3.6)	2.6 (2.1 to 3.4)	1.9 (1.4 to 3.1)	3.6 (2.8 to 4.6)	46.2 (6.2 to 107.9)	59.6 (-0.4 to 268.1)	36.9 (-7.6 to 103.8)
Central Europe, eastern Europe, and central Asia	Central Asia	Uzbekistan	YLLs	All Ages	167 (111 to 220)	65 (34 to 92)	101 (65 to 148)	439 (336 to 580)	197 (135 to 327)	242 (182 to 316)	163.6 (77.8 to 314.8)	202.1 (78.7 to 649.8)	138.7 (41 to 319.2)
Central Europe, eastern Europe, and central Asia	Central Asia	Uzbekistan	YLLs	Age-standardized	1.4 (0.9 to 1.9)	1 (0.5 to 1.4)	1.9 (1.2 to 2.8)	1.9 (1.5 to 2.6)	1.6 (1.1 to 2.9)	2.3 (1.8 to 2.9)	36.7 (-8.2 to 116.2)	62.4 (-6.6 to 326.3)	19.3 (-28.2 to 108.8)
Central Europe, eastern Europe, and central Asia	Central Asia	Uzbekistan	YLDs	All Ages	43 (28 to 62)	12 (7 to 18)	31 (20 to 46)	125 (78 to 184)	23 (14 to 35)	102 (62 to 155)	192.8 (132.6 to 265)	97.1 (51.2 to 154.7)	228.6 (148.7 to 330.5)
Central Europe, eastern Europe, and central Asia	Central Asia	Uzbekistan	YLDs	Age-standardized	0.4 (0.3 to 0.6)	0.2 (0.1 to 0.3)	0.7 (0.5 to 1.1)	0.7 (0.4 to 1)	0.3 (0.2 to 0.4)	1.3 (0.8 to 2)	80.2 (41.9 to 125.4)	44.1 (9.4 to 84.8)	83 (35.2 to 148.9)
		Central Europe	Incidence	All Ages	1,609 (1,363 to 1,909)	243 (199 to 299)	1,367 (1,141 to 1,647)	1,802 (1,545 to 2,099)	384 (308 to 474)	1,419 (1,221 to 1,649)	12 (-0.7 to 24)	58.1 (44.6 to 73.1)	3.8 (-8.9 to 15.4)
		Central Europe	Incidence	Age-standardized	1.1 (0.9 to 1.3)	0.3 (0.2 to 0.4)	2 (1.7 to 2.4)	1 (0.8 to 1.1)	0.4 (0.3 to 0.4)	1.6 (1.4 to 1.9)	-13.4 (-22.3 to -5.8)	15.2 (8.2 to 22.6)	-18.6 (-27.9 to -11)
		Central Europe	Prevalence	All Ages	22,052 (18,277 to 27,025)	2,394 (1,897 to 3,066)	19,658 (16,113 to 24,440)	24,659 (21,002 to 28,896)	3,777 (2,932 to 4,849)	20,882 (17,653 to 24,472)	11.8 (-2 to 24)	57.8 (44.7 to 70.8)	6.2 (-7.6 to 18.4)
		Central Europe	Prevalence	Age-standardized	14.9 (12.4 to 18.2)	2.9 (2.3 to 3.7)	30.2 (24.9 to 37.5)	12.1 (10.4 to 14.1)	3.1 (2.4 to 3.9)	23.3 (19.8 to 27.3)	-18.5 (-28.4 to -10.4)	6.3 (0 to 12.3)	-22.7 (-32.6 to -14.6)
		Central Europe	Deaths	All Ages	809 (760 to 860)	50 (35 to 62)	759 (713 to 806)	250 (212 to 289)	40 (32 to 51)	210 (179 to 245)	-69.1 (-73.7 to -63.8)	-20.6 (-41.2 to 16.2)	-72.3 (-76.5 to -67.3)
		Central Europe	Deaths	Age-standardized	0.5 (0.5 to 0.6)	0.1 (0 to 0.1)	1.2 (1.2 to 1.3)	0.1 (0.1 to 0.1)	0 (0 to 0)	0.2 (0.2 to 0.3)	-79.5 (-82.5 to -76)	-51.4 (-63.3 to -31.4)	-81.4 (-84.1 to -78.1)
		Central Europe	DALYs	All Ages	21,251 (19,519 to 23,039)	1,327 (993 to 1,595)	19,924 (18,347 to 21,652)	8,191 (6,808 to 9,777)	1,206 (955 to 1,540)	6,986 (5,818 to 8,416)	-61.5 (-66.9 to -55.4)	-9.2 (-27.2 to 12.4)	-64.9 (-70.1 to -59.1)
		Central Europe	DALYs	Age-standardized	14 (12.9 to 15.2)	1.6 (1.2 to 1.9)	30.5 (28.1 to 33)	3.9 (3.2 to 4.7)	1 (0.8 to 1.3)	7.7 (6.4 to 9.2)	-72 (-76 to -67.5)	-37.7 (-49.8 to -23.5)	-74.8 (-78.5 to -70.7)
		Central Europe	YLLs	All Ages	18,090 (16,892 to 19,347)	955 (663 to 1,167)	17,135 (15,957 to 18,352)	4,626 (3,934 to 5,412)	627 (506 to 789)	4,000 (3,387 to 4,736)	-74.4 (-78.4 to -69.8)	-34.4 (-50.4 to -7.1)	-76.7 (-80.4 to -72.2)
		Central Europe	YLLs	Age-standardized	11.9 (11.1 to 12.7)	1.1 (0.8 to 1.4)	26.2 (24.6 to 27.9)	2.2 (1.8 to 2.5)	0.5 (0.4 to 0.6)	4.3 (3.7 to 5.1)	-81.8 (-84.6 to -78.5)	-54.8 (-65.6 to -37.1)	-83.5 (-86.1 to -80.3)
		Central Europe	YLDs	All Ages	3,162 (2,094 to 4,601)	373 (240 to 547)	2,789 (1,814 to 4,041)	3,565 (2,408 to 5,027)	579 (370 to 863)	2,986 (2,001 to 4,191)	12.8 (-1.8 to 27.1)	55.5 (42.2 to 70)	7.1 (-7.8 to 22.1)
		Central Europe	YLDs	Age-standardized	2.1 (1.4 to 3.1)	0.5 (0.3 to 0.7)	4.3 (2.8 to 6.2)	1.8 (1.2 to 2.5)	0.5 (0.3 to 0.7)	3.4 (2.2 to 4.7)	-17.2 (-27.4 to -7.6)	5.5 (-1.9 to 12.4)	-21.6 (-31.8 to -11.7)
Central Europe, eastern Europe, and central Asia	Central Europe	Albania	Incidence	All Ages	24 (19 to 29)	2 (1 to 2)	22 (18 to 27)	42 (33 to 53)	3 (2 to 4)	38 (30 to 50)	76.8 (51.7 to 117.9)	98.9 (54.2 to 150.9)	75.1 (48.4 to 120.3)
Central Europe, eastern Europe, and central Asia	Central Europe	Albania	Incidence	Age-standardized	1 (0.8 to 1.2)	0.1 (0.1 to 0.2)	1.8 (1.5 to 2.2)	1 (0.8 to 1.3)	0.2 (0.1 to 0.2)	2 (1.6 to 2.5)	6.2 (-6.1 to 28.3)	9.5 (-10.6 to 32.6)	11.3 (-1.5 to 36.8)
Central Europe, eastern Europe, and central Asia	Central Europe	Albania	Prevalence	All Ages	310 (252 to 387)	17 (13 to 23)	292 (235 to 366)	700 (553 to 897)	41 (29 to 56)	659 (519 to 853)	126.1 (96.2 to 182.9)	138.4 (87.8 to 200.3)	125.4 (94.3 to 185)
Central Europe, eastern Europe, and central Asia	Central Europe	Albania	Prevalence	Age-standardized	14.3 (11.7 to 17.5)	1.6 (1.2 to 2.2)	27.9 (22.6 to 34.2)	16.4 (13.1 to 20.9)	1.8 (1.3 to 2.4)	32.6 (25.9 to 41.9)	15.1 (2 to 43)	12.3 (-10.4 to 40.4)	17.1 (3.2 to 45.9)
Central Europe, eastern Europe, and central Asia	Central Europe	Albania	Deaths	All Ages	18 (14 to 22)	0 (0 to 1)	18 (14 to 21)	10 (7 to 16)	1 (0 to 1)	9 (6 to 15)	-44.7 (-63.3 to -8)	91.3 (1.2 to 251.9)	-48.2 (-66.9 to -11.6)
Central Europe, eastern Europe, and central Asia	Central Europe	Albania	Deaths	Age-standardized	0.9 (0.7 to 1.1)	0 (0 to 0.1)	2 (1.5 to 2.4)	0.2 (0.2 to 0.4)	0 (0 to 0.1)	0.5 (0.3 to 0.7)	-74.4 (-82.9 to -56.9)	-17.3 (-57 to 52.1)	-76.8 (-85.1 to -60)
Central Europe, eastern Europe, and central Asia	Central Europe	Albania	DALYs	All Ages	495 (410 to 581)	10 (7 to 13)	485 (401 to 572)	304 (212 to 425)	17 (12 to 25)	287 (196 to 407)	-38.6 (-57.3 to -8.9)	77.9 (17.6 to 168.9)	-40.9 (-59.6 to -11.4)
Central Europe, eastern Europe, and central Asia	Central Europe	Albania	DALYs	Age-standardized	22.9 (18.7 to 26.9)	0.9 (0.7 to 1.3)	46.5 (37.7 to 54.8)	7.2 (5 to 10)	0.7 (0.5 to 1.1)	14.2 (9.8 to 20.2)	-68.5 (-78.3 to -53.2)	-15.6 (-45.4 to 26)	-69.4 (-78.9 to -53.9)
Central Europe, eastern Europe, and central Asia	Central Europe	Albania	YLLs	All Ages	450 (362 to 537)	7 (5 to 11)	443 (352 to 530)	205 (131 to 328)	11 (6 to 17)	194 (121 to 315)	-54.5 (-71.2 to -22.6)	53.9 (-16.7 to 174.7)	-56.1 (-72.9 to -24.8)
Central Europe, eastern Europe, and central Asia	Central Europe	Albania	YLLs	Age-standardized	20.8 (16.7 to 25)	0.6 (0.4 to 1)	42.5 (33.7 to 50.7)	4.9 (3 to 7.7)	0.5 (0.3 to 0.8)	9.7 (5.9 to 15.5)	-76.6 (-85.4 to -59.8)	-27 (-61.4 to 29.7)	-77.3 (-85.8 to -61)
Central Europe, eastern Europe, and central Asia	Central Europe	Albania	YLDs	All Ages	45 (28 to 67)	3 (2 to 4)	42 (26 to 64)	99 (64 to 149)	6 (4 to 10)	92 (59 to 141)	120.3 (64.7 to 202.1)	138.4 (87.8 to 200.1)	119.2 (60.9 to 206.1)
Central Europe, eastern Europe, and central Asia	Central Europe	Albania	YLDs	Age-standardized	2.1 (1.3 to 3)	0.3 (0.2 to 0.4)	4 (2.5 to 5.8)	2.3 (1.5 to 3.5)	0.3 (0.2 to 0.4)	4.6 (2.9 to 6.9)	13.6 (-13.4 to 51.3)	12.3 (-10.4 to 40.1)	15.9 (-11.2 to 56.4)
Central Europe, eastern Europe, and central Asia	Central Europe	Bosnia and Herzegovina	Incidence	All Ages	18 (14 to 22)	3 (2 to 4)	15 (12 to 19)	34 (27 to 42)	5 (4 to 8)	28 (22 to 35)	88.7 (62.4 to 117.8)	87.2 (36.7 to 162.7)	89 (61.1 to 120.6)
Central Europe, eastern Europe, and central Asia	Central Europe	Bosnia and Herzegovina	Incidence	Age-standardized	0.4 (0.3 to 0.5)	0.1 (0.1 to 0.2)	0.7 (0.6 to 0.9)	0.6 (0.5 to 0.8)	0.2 (0.1 to 0.2)	1.1 (0.9 to 1.4)	60.5 (42 to 81.5)	38.8 (6.7 to 84.6)	59.8 (40.5 to 81.9)
Central Europe, eastern Europe, and central Asia	Central Europe	Bosnia and Herzegovina	Prevalence	All Ages	231 (184 to 289)	31 (22 to 42)	200 (156 to 252)	541 (428 to 674)	65 (46 to 91)	476 (370 to 599)	134 (98.6 to 174.3)	109.6 (54.2 to 190.3)	137.8 (98.9 to 183.2)
Central Europe, eastern Europe, and central Asia	Central Europe	Bosnia and Herzegovina	Prevalence	Age-standardized	5.5 (4.5 to 6.8)	1.4 (1 to 1.9)	10.8 (8.5 to 13.4)	9.5 (7.6 to 11.8)	2 (1.4 to 2.7)	18.5 (14.6 to 23.2)	71.9 (48.7 to 98.5)	38.2 (3.5 to 85.6)	71.5 (47.1 to 100.1)
Central Europe, eastern Europe, and central Asia	Central Europe	Bosnia and Herzegovina	Deaths	All Ages	1 (0 to 1)	0 (0 to 0)	1 (0 to 1)	3 (2 to 4)	1 (1 to 2)	2 (1 to 2)	243.6 (113 to 512.4)	353.1 (113.2 to 868.9)	198.9 (56.9 to 624.5)
Central Europe, eastern Europe, and central Asia	Central Europe	Bosnia and Herzegovina	Deaths	Age-standardized	0 (0 to 0)	0 (0 to 0)	0 (0 to 0.1)	0 (0 to 0.1)	0 (0 to 0.1)	0.1 (0 to 0.1)	81.2 (13.5 to 211.1)	99 (-7 to 331)	66.3 (-9.6 to 315.8)
Central Europe, eastern Europe, and central Asia	Central Europe	Bosnia and Herzegovina	DALYs	All Ages	50 (36 to 67)	8 (5 to 11)	43 (29 to 59)	115 (84 to 158)	20 (13 to 28)	95 (67 to 134)	129.4 (84.2 to 193.7)	161.8 (84.9 to 284.7)	123.6 (73.2 to 199.3)
Central Europe, eastern Europe, and central Asia	Central Europe	Bosnia and Herzegovina	DALYs	Age-standardized	1.2 (0.9 to 1.7)	0.4 (0.3 to 0.6)	2.4 (1.6 to 3.2)	2 (1.5 to 2.7)	0.6 (0.4 to 0.9)	3.7 (2.6 to 5.1)	60.3 (29.3 to 103.2)	53.6 (6.7 to 125.8)	55.3 (21 to 107.6)
Central Europe, eastern Europe, and central Asia	Central Europe	Bosnia and Herzegovina	YLLs	All Ages	14 (8 to 21)	3 (2 to 4)	12 (5 to 18)	37 (26 to 50)	10 (5 to 16)	27 (18 to 39)	156.3 (52.6 to 382.5)	259.3 (72.7 to 645.2)	132.5 (21.8 to 446)
Central Europe, eastern Europe, and central Asia	Central Europe	Bosnia and Herzegovina	YLLs	Age-standardized	0.4 (0.2 to 0.6)	0.2 (0.1 to 0.3)	0.7 (0.3 to 1.1)	0.6 (0.4 to 0.9)	0.3 (0.2 to 0.5)	1 (0.7 to 1.5)	55.3 (-5.7 to 181.7)	76.2 (-14.3 to 259.5)	42.9 (-23.9 to 245.9)
Central Europe, eastern Europe, and central Asia	Central Europe	Bosnia and Herzegovina	YLDs	All Ages	36 (23 to 53)	5 (3 to 8)	31 (19 to 47)	78 (49 to 117)	10 (6 to 15)	68 (42 to 104)	118.6 (74.8 to 172.1)	107.9 (52.1 to 187.4)	120.3 (69.9 to 181.8)
Central Europe, eastern Europe, and central Asia	Central Europe	Bosnia and Herzegovina	YLDs	Age-standardized	0.8 (0.5 to 1.2)	0.2 (0.1 to 0.3)	1.6 (1.1 to 2.4)	1.4 (0.9 to 2.1)	0.3 (0.2 to 0.5)	2.6 (1.7 to 4)	62.6 (31 to 99)	36.3 (1.3 to 84.9)	60.8 (25.3 to 100.9)
Central Europe, eastern Europe, and central Asia	Central Europe	Bulgaria	Incidence	All Ages	139 (115 to 167)	8 (6 to 11)	131 (106 to 159)	116 (95 to 141)	22 (16 to 35)	93 (77 to 113)	-16.6 (-30.3 to 1.2)	174.8 (102.1 to 336)	-28.5 (-40 to -15.3)
Central Europe, eastern Europe, and central Asia	Central Europe	Bulgaria	Incidence	Age-standardized	1.1 (0.9 to 1.3)	0.1 (0.1 to 0.2)	2.2 (1.8 to 2.6)	0.9 (0.8 to 1.1)	0.3 (0.2 to 0.4)	1.7 (1.4 to 2)	-16.9 (-28.6 to -3.6)	105.5 (59.5 to 206.9)	-24.2 (-35 to -12.4)
Central Europe, eastern Europe, and central Asia	Central Europe	Bulgaria	Prevalence	All Ages	2,128 (1,761 to 2,588)	96 (66 to 131)	2,033 (1,670 to 2,482)	1,833 (1,499 to 2,211)	262 (181 to 389)	1,571 (1,278 to 1,915)	-13.9 (-25.7 to 0.9)	174.3 (104.9 to 304)	-22.7 (-33.3 to -10)
Central Europe, eastern Europe, and central Asia	Central Europe	Bulgaria	Prevalence	Age-standardized	16.7 (13.8 to 20.2)	1.5 (1.1 to 2)	34.6 (28.6 to 42.1)	13.1 (10.8 to 15.9)	2.9 (2 to 4.1)	26.1 (21.2 to 31.8)	-21.3 (-31.7 to -8.3)	94.1 (50 to 178.5)	-24.5 (-34.6 to -12.3)
Central Europe, eastern Europe, and central Asia	Central Europe	Bulgaria	Deaths	All Ages	110 (93 to 127)	2 (1 to 2)	108 (92 to 126)	32 (24 to 41)	10 (7 to 15)	22 (16 to 29)	-70.9 (-79.2 to -60.5)	538.1 (297.3 to 950.6)	-79.8 (-85.7 to -71.4)
Central Europe, eastern Europe, and central Asia	Central Europe	Bulgaria	Deaths	Age-standardized	0.9 (0.7 to 1)	0 (0 to 0)	1.9 (1.6 to 2.1)	0.2 (0.2 to 0.3)	0.1 (0.1 to 0.2)	0.3 (0.3 to 0.5)	-75.3 (-82.2 to -66.6)	312.9 (163.5 to 565.8)	-81.3 (-86.5 to -73.8)
Central Europe, eastern Europe, and central Asia	Central Europe	Bulgaria	DALYs	All Ages	2,837 (2,409 to 3,273)	45 (35 to 57)	2,792 (2,360 to 3,225)	845 (654 to 1,053)	196 (139 to 272)	650 (499 to 831)	-70.2 (-77.6 to -61)	336.2 (197.8 to 547.2)	-76.7 (-82.8 to -68.8)
Central Europe, eastern Europe, and central Asia	Central Europe	Bulgaria	DALYs	Age-standardized	21.4 (18.2 to 24.5)	0.7 (0.5 to 0.9)	45.4 (38.8 to 52.1)	6 (4.7 to 7.4)	2.3 (1.7 to 3.2)	10.6 (8.1 to 13.5)	-72.1 (-78.8 to -63.8)	234.5 (131 to 384)	-76.7 (-82.6 to -69)
Central Europe, eastern Europe, and central Asia	Central Europe	Bulgaria	YLLs	All Ages	2,539 (2,132 to 2,993)	30 (22 to 38)	2,509 (2,092 to 2,963)	587 (436 to 773)	157 (104 to 230)	430 (310 to 589)	-76.9 (-83.8 to -67.6)	426 (229.3 to 751.8)	-82.9 (-88.2 to -75.1)
Central Europe, eastern Europe, and central Asia	Central Europe	Bulgaria	YLLs	Age-standardized	19 (16 to 22.2)	0.5 (0.3 to 0.6)	40.6 (34.2 to 47.2)	4.1 (3 to 5.4)	1.9 (1.3 to 2.8)	6.9 (5 to 9.4)	-78.5 (-84.8 to -70.3)	311 (159 to 557.4)	-83 (-88.2 to -75.5)
Central Europe, eastern Europe, and central Asia	Central Europe	Bulgaria	YLDs	All Ages	298 (196 to 444)	15 (9 to 23)	283 (185 to 423)	258 (168 to 372)	39 (23 to 65)	219 (140 to 318)	-13.2 (-34.5 to 14.3)	159 (79.8 to 284.6)	-22.4 (-42.1 to 2.4)
Central Europe, eastern Europe, and central Asia	Central Europe	Bulgaria	YLDs	Age-standardized	2.3 (1.5 to 3.5)	0.2 (0.1 to 0.4)	4.8 (3.2 to 7.1)	1.9 (1.2 to 2.7)	0.4 (0.3 to 0.7)	3.7 (2.4 to 5.3)	-19.6 (-38.3 to 3.6)	84.3 (33.9 to 162.8)	-23.4 (-41 to -0.4)
Central Europe, eastern Europe, and central Asia	Central Europe	Croatia	Incidence	All Ages	40 (33 to 47)	8 (6 to 10)	32 (26 to 38)	60 (49 to 72)	11 (8 to 15)	49 (40 to 59)	51 (32.6 to 71)	40.7 (15.9 to 71.6)	53.6 (33.4 to 77.9)
Central Europe, eastern Europe, and central Asia	Central Europe	Croatia	Incidence	Age-standardized	0.6 (0.5 to 0.8)	0.2 (0.2 to 0.3)	1.1 (0.9 to 1.3)	0.8 (0.7 to 1)	0.3 (0.2 to 0.3)	1.5 (1.2 to 1.8)	31 (17.9 to 45.7)	13.8 (-3 to 34.5)	31.7 (17.2 to 48.1)
Central Europe, eastern Europe, and central Asia	Central Europe	Croatia	Prevalence	All Ages	596 (490 to 726)	108 (79 to 144)	489 (391 to 605)	915 (742 to 1,104)	157 (114 to 210)	757 (600 to 929)	53.4 (36.5 to 70.2)	46.2 (22 to 78.3)	55 (36.1 to 74.5)
Central Europe, eastern Europe, and central Asia	Central Europe	Croatia	Prevalence	Age-standardized	9.4 (7.8 to 11.4)	2.9 (2.2 to 3.9)	18.2 (14.7 to 22.3)	11.5 (9.4 to 13.9)	3.1 (2.3 to 4.1)	21.6 (17.4 to 26.5)	22.1 (8.8 to 35.8)	8.1 (-9 to 29.9)	18.4 (4.4 to 32.3)
Central Europe, eastern Europe, and central Asia	Central Europe	Croatia	Deaths	All Ages	3 (3 to 4)	0 (0 to 0)	3 (3 to 4)	11 (9 to 14)	2 (1 to 3)	9 (7 to 12)	228.7 (138.1 to 344.6)	750.6 (413.1 to 1245.9)	192.3 (110 to 301.5)
Central Europe, eastern Europe, and central Asia	Central Europe	Croatia	Deaths	Age-standardized	0.1 (0 to 0.1)	0 (0 to 0)	0.1 (0.1 to 0.2)	0.1 (0.1 to 0.2)	0 (0 to 0)	0.2 (0.2 to 0.3)	116.5 (58.9 to 186.9)	434.6 (231.1 to 726.8)	77.6 (29.1 to 140)
Central Europe, eastern Europe, and central Asia	Central Europe	Croatia	DALYs	All Ages	158 (123 to 202)	21 (14 to 30)	137 (107 to 176)	323 (254 to 405)	51 (36 to 69)	272 (211 to 343)	104.5 (65.7 to 158.2)	145.8 (83.2 to 240.8)	98.3 (56.6 to 153.2)
Central Europe, eastern Europe, and central Asia	Central Europe	Croatia	DALYs	Age-standardized	2.5 (1.9 to 3.2)	0.6 (0.4 to 0.8)	5.2 (4.1 to 6.6)	3.8 (3 to 4.8)	1 (0.7 to 1.3)	7.5 (5.8 to 9.4)	56.4 (28.7 to 95.7)	76.4 (33.9 to 140.1)	43.8 (16.5 to 80.5)
Central Europe, eastern Europe, and central Asia	Central Europe	Croatia	YLLs	All Ages	69 (60 to 80)	4 (3 to 5)	65 (56 to 77)	192 (146 to 249)	27 (17 to 39)	165 (123 to 216)	177.6 (105.6 to 277.4)	607 (337.8 to 1005.4)	152.5 (82.6 to 247.5)
Central Europe, eastern Europe, and central Asia	Central Europe	Croatia	YLLs	Age-standardized	1.1 (0.9 to 1.2)	0.1 (0.1 to 0.1)	2.5 (2.2 to 3)	2.2 (1.7 to 2.8)	0.5 (0.3 to 0.7)	4.4 (3.3 to 5.8)	105.6 (52.3 to 175.9)	400.2 (215 to 668.7)	72.9 (24.9 to 134.4)
Central Europe, eastern Europe, and central Asia	Central Europe	Croatia	YLDs	All Ages	89 (56 to 133)	17 (11 to 25)	72 (44 to 109)	131 (85 to 195)	24 (14 to 37)	107 (68 to 158)	47.7 (17.4 to 88.5)	41.7 (14 to 75.4)	49.2 (14.1 to 98.4)
Central Europe, eastern Europe, and central Asia	Central Europe	Croatia	YLDs	Age-standardized	1.4 (0.9 to 2.1)	0.5 (0.3 to 0.7)	2.7 (1.6 to 4)	1.7 (1.1 to 2.4)	0.5 (0.3 to 0.7)	3.1 (2 to 4.5)	19 (-3.1 to 49.8)	5.7 (-12.8 to 27.9)	15.8 (-9.6 to 50.6)
Central Europe, eastern Europe, and central Asia	Central Europe	Czechia	Incidence	All Ages	64 (53 to 84)	2 (1 to 3)	62 (51 to 82)	100 (82 to 121)	2 (1 to 4)	98 (80 to 118)	55.6 (24.8 to 77.5)	24.3 (-5.5 to 58.2)	56.6 (24.8 to 78.5)
Central Europe, eastern Europe, and central Asia	Central Europe	Czechia	Incidence	Age-standardized	0.5 (0.4 to 0.6)	0 (0 to 0.1)	1.1 (0.9 to 1.4)	0.6 (0.5 to 0.7)	0 (0 to 0.1)	1.2 (1 to 1.4)	18.2 (-5 to 34)	1 (-30.7 to 24.8)	8.5 (-11.5 to 23.1)
Central Europe, eastern Europe, and central Asia	Central Europe	Czechia	Prevalence	All Ages	804 (633 to 1,063)	6 (4 to 8)	798 (628 to 1,056)	1,711 (1,372 to 2,094)	9 (6 to 12)	1,702 (1,365 to 2,085)	112.9 (68.6 to 148.7)	43.7 (20.7 to 70.9)	113.4 (69 to 149.5)
Central Europe, eastern Europe, and central Asia	Central Europe	Czechia	Prevalence	Age-standardized	5.9 (4.7 to 7.8)	0.1 (0.1 to 0.1)	14 (11.1 to 18.5)	8.8 (7 to 11)	0.1 (0.1 to 0.1)	19.2 (15.4 to 23.7)	48.5 (19.6 to 75.3)	3.8 (-13.2 to 19.2)	37.2 (10.2 to 60.6)
Central Europe, eastern Europe, and central Asia	Central Europe	Czechia	Deaths	All Ages	185 (167 to 205)	4 (3 to 6)	181 (162 to 202)	52 (40 to 66)	3 (2 to 4)	49 (37 to 62)	-71.8 (-78.7 to -63)	-21.4 (-53.7 to 22)	-72.8 (-79.6 to -64.3)
Central Europe, eastern Europe, and central Asia	Central Europe	Czechia	Deaths	Age-standardized	1.3 (1.2 to 1.5)	0 (0 to 0.1)	3.5 (3.1 to 3.8)	0.2 (0.2 to 0.3)	0 (0 to 0)	0.5 (0.4 to 0.7)	-82.3 (-86.7 to -76.9)	-49.3 (-70.2 to -22.5)	-84.3 (-88.3 to -79.5)
Central Europe, eastern Europe, and central Asia	Central Europe	Czechia	DALYs	All Ages	3,852 (3,437 to 4,323)	66 (52 to 100)	3,786 (3,364 to 4,258)	1,137 (893 to 1,404)	48 (32 to 66)	1,089 (856 to 1,351)	-70.5 (-77.3 to -62)	-27.2 (-57.8 to 10)	-71.2 (-77.9 to -62.7)
Central Europe, eastern Europe, and central Asia	Central Europe	Czechia	DALYs	Age-standardized	27.5 (24.6 to 30.8)	0.8 (0.6 to 1.2)	67.1 (60.2 to 74.9)	5.4 (4.2 to 6.7)	0.4 (0.3 to 0.5)	11.8 (9.3 to 14.7)	-80.4 (-84.9 to -74.8)	-50.7 (-70.9 to -25.8)	-82.4 (-86.4 to -77.1)
Central Europe, eastern Europe, and central Asia	Central Europe	Czechia	YLLs	All Ages	3,733 (3,332 to 4,203)	65 (52 to 99)	3,668 (3,251 to 4,136)	895 (671 to 1,153)	47 (30 to 65)	849 (635 to 1,100)	-76 (-82.1 to -68.1)	-28.3 (-59.1 to 9.3)	-76.9 (-82.9 to -68.8)
Central Europe, eastern Europe, and central Asia	Central Europe	Czechia	YLLs	Age-standardized	26.6 (23.7 to 30)	0.8 (0.6 to 1.2)	65 (58.2 to 72.9)	4.1 (3.1 to 5.3)	0.4 (0.2 to 0.5)	9.1 (6.8 to 11.8)	-84.5 (-88.5 to -79.2)	-51.7 (-71.9 to -26.5)	-86 (-89.6 to -81.2)
Central Europe, eastern Europe, and central Asia	Central Europe	Czechia	YLDs	All Ages	119 (74 to 180)	1 (1 to 2)	118 (73 to 179)	242 (154 to 357)	1 (1 to 2)	240 (153 to 354)	103.8 (47.3 to 167.6)	44 (21.7 to 70.2)	104.3 (47.5 to 168.5)
Central Europe, eastern Europe, and central Asia	Central Europe	Czechia	YLDs	Age-standardized	0.9 (0.5 to 1.3)	0 (0 to 0)	2.1 (1.3 to 3.1)	1.3 (0.8 to 1.9)	0 (0 to 0)	2.7 (1.7 to 4)	43.3 (6.1 to 87.4)	3.5 (-13.6 to 18)	32.2 (-3.1 to 72.8)
Central Europe, eastern Europe, and central Asia	Central Europe	Hungary	Incidence	All Ages	91 (75 to 110)	10 (7 to 14)	81 (66 to 98)	135 (112 to 164)	15 (11 to 21)	119 (97 to 148)	48 (35.6 to 61.3)	53.2 (19.2 to 92.1)	47.3 (34.5 to 61.7)
Central Europe, eastern Europe, and central Asia	Central Europe	Hungary	Incidence	Age-standardized	0.7 (0.5 to 0.8)	0.1 (0.1 to 0.2)	1.3 (1.1 to 1.6)	0.8 (0.7 to 1)	0.2 (0.1 to 0.2)	1.7 (1.4 to 2)	28.3 (17.7 to 39.3)	18.9 (-4 to 45.4)	26.7 (16.5 to 37.8)
Central Europe, eastern Europe, and central Asia	Central Europe	Hungary	Prevalence	All Ages	1,380 (1,114 to 1,681)	127 (92 to 171)	1,253 (1,011 to 1,550)	2,283 (1,846 to 2,807)	210 (149 to 288)	2,073 (1,657 to 2,556)	65.4 (48.5 to 81.6)	64.7 (25.1 to 104.9)	65.5 (48 to 82.9)
Central Europe, eastern Europe, and central Asia	Central Europe	Hungary	Prevalence	Age-standardized	9.5 (7.7 to 11.5)	1.5 (1.1 to 2)	20.5 (16.7 to 25.3)	12.6 (10.2 to 15.5)	1.8 (1.3 to 2.4)	27.3 (21.7 to 33.7)	32.7 (18.9 to 45.4)	19.4 (-7.4 to 47.8)	33 (19.1 to 46.3)
Central Europe, eastern Europe, and central Asia	Central Europe	Hungary	Deaths	All Ages	54 (49 to 60)	4 (3 to 5)	50 (45 to 56)	36 (28 to 43)	6 (4 to 8)	30 (23 to 37)	-34.2 (-49.1 to -15.8)	51.7 (1.3 to 110.4)	-40.7 (-54.8 to -23.3)
Central Europe, eastern Europe, and central Asia	Central Europe	Hungary	Deaths	Age-standardized	0.4 (0.3 to 0.4)	0 (0 to 0.1)	0.9 (0.8 to 1)	0.2 (0.1 to 0.2)	0 (0 to 0.1)	0.4 (0.3 to 0.5)	-52.2 (-62.9 to -39.5)	4.2 (-30.2 to 42.7)	-54.9 (-65.2 to -42.2)
Central Europe, eastern Europe, and central Asia	Central Europe	Hungary	DALYs	All Ages	1,415 (1,265 to 1,580)	103 (86 to 130)	1,312 (1,168 to 1,462)	979 (789 to 1,200)	136 (99 to 176)	843 (673 to 1,047)	-30.8 (-44.3 to -14.6)	32.1 (-4.2 to 71.9)	-35.7 (-48.7 to -20.5)
Central Europe, eastern Europe, and central Asia	Central Europe	Hungary	DALYs	Age-standardized	9.7 (8.7 to 10.8)	1.3 (1.1 to 1.6)	21.4 (19.1 to 23.8)	5.3 (4.2 to 6.5)	1.3 (0.9 to 1.7)	11 (8.8 to 13.5)	-45.5 (-56.2 to -33)	1.7 (-29.3 to 34.9)	-48.7 (-58.6 to -37.1)
Central Europe, eastern Europe, and central Asia	Central Europe	Hungary	YLLs	All Ages	1,217 (1,095 to 1,365)	83 (69 to 110)	1,134 (1,014 to 1,279)	658 (516 to 807)	103 (71 to 135)	555 (430 to 692)	-45.9 (-58.2 to -30.4)	24.2 (-20 to 75.3)	-51.1 (-62.7 to -36.5)
Central Europe, eastern Europe, and central Asia	Central Europe	Hungary	YLLs	Age-standardized	8.3 (7.5 to 9.3)	1 (0.9 to 1.4)	18.5 (16.7 to 20.8)	3.5 (2.7 to 4.3)	1 (0.7 to 1.4)	7.2 (5.6 to 8.9)	-58 (-67.9 to -46)	-2.3 (-39.6 to 38.9)	-61.1 (-70.3 to -49.6)
Central Europe, eastern Europe, and central Asia	Central Europe	Hungary	YLDs	All Ages	198 (125 to 295)	20 (12 to 31)	178 (111 to 268)	321 (207 to 467)	33 (19 to 52)	288 (183 to 425)	62.1 (26.2 to 107.5)	64.7 (25.1 to 104.7)	61.8 (22.3 to 112.6)
Central Europe, eastern Europe, and central Asia	Central Europe	Hungary	YLDs	Age-standardized	1.4 (0.9 to 2)	0.2 (0.1 to 0.4)	2.9 (1.9 to 4.3)	1.8 (1.1 to 2.6)	0.3 (0.2 to 0.4)	3.8 (2.4 to 5.6)	30.9 (2 to 66.5)	19.4 (-7.5 to 47.6)	30.8 (0.6 to 69.4)
Central Europe, eastern Europe, and central Asia	Central Europe	North Macedonia	Incidence	All Ages	12 (10 to 17)	1 (1 to 2)	11 (9 to 15)	19 (15 to 23)	2 (2 to 3)	16 (13 to 20)	52.4 (13.8 to 73.7)	85.6 (38 to 148.6)	48.8 (8.7 to 71)
Central Europe, eastern Europe, and central Asia	Central Europe	North Macedonia	Incidence	Age-standardized	0.6 (0.5 to 0.8)	0.1 (0.1 to 0.2)	1.1 (0.9 to 1.5)	0.6 (0.5 to 0.7)	0.1 (0.1 to 0.2)	1.1 (0.9 to 1.3)	0.6 (-24 to 13.4)	17.4 (-6.4 to 49)	-3.4 (-28.9 to 10.2)
Central Europe, eastern Europe, and central Asia	Central Europe	North Macedonia	Prevalence	All Ages	175 (140 to 243)	13 (9 to 18)	162 (128 to 230)	298 (239 to 369)	27 (19 to 37)	271 (215 to 338)	70.1 (26.3 to 96.1)	104.4 (52.4 to 176.4)	67.4 (21.6 to 95.6)
Central Europe, eastern Europe, and central Asia	Central Europe	North Macedonia	Prevalence	Age-standardized	9.2 (7.4 to 12.6)	1.4 (1 to 1.9)	17.6 (14 to 24.8)	9.4 (7.6 to 11.6)	1.7 (1.2 to 2.3)	17.9 (14.3 to 22.1)	3.1 (-23.1 to 17.9)	18.7 (-10.6 to 57.1)	1.5 (-25.6 to 17.6)
Central Europe, eastern Europe, and central Asia	Central Europe	North Macedonia	Deaths	All Ages	4 (3 to 6)	0 (0 to 0)	4 (3 to 6)	0 (0 to 1)	0 (0 to 0)	0 (0 to 1)	-89.2 (-92.9 to -83.5)	28.6 (-29 to 112.5)	-90 (-93.6 to -84.5)
Central Europe, eastern Europe, and central Asia	Central Europe	North Macedonia	Deaths	Age-standardized	0.2 (0.2 to 0.3)	0 (0 to 0)	0.5 (0.4 to 0.6)	0 (0 to 0)	0 (0 to 0)	0 (0 to 0)	-93.5 (-95.5 to -90.2)	-31.6 (-61.8 to 12.7)	-93.9 (-95.9 to -90.7)
Central Europe, eastern Europe, and central Asia	Central Europe	North Macedonia	DALYs	All Ages	130 (102 to 162)	3 (2 to 4)	127 (99 to 159)	54 (38 to 76)	5 (3 to 7)	49 (34 to 69)	-58.6 (-71.1 to -41.4)	89.3 (43.6 to 146.6)	-61.6 (-73.4 to -45.3)
Central Europe, eastern Europe, and central Asia	Central Europe	North Macedonia	DALYs	Age-standardized	6.6 (5.2 to 8.2)	0.3 (0.2 to 0.4)	13.6 (10.8 to 17)	1.7 (1.2 to 2.4)	0.3 (0.2 to 0.5)	3.2 (2.2 to 4.5)	-74.4 (-81.9 to -64.2)	9 (-16.1 to 38)	-76.6 (-83.7 to -67)
Central Europe, eastern Europe, and central Asia	Central Europe	North Macedonia	YLLs	All Ages	104 (78 to 133)	1 (0 to 1)	103 (77 to 133)	10 (7 to 14)	1 (0 to 1)	9 (7 to 13)	-90.5 (-93.8 to -85)	31.1 (-24 to 115.8)	-91.1 (-94.4 to -85.7)
Central Europe, eastern Europe, and central Asia	Central Europe	North Macedonia	YLLs	Age-standardized	5.2 (4 to 6.7)	0.1 (0 to 0.1)	11 (8.4 to 14.1)	0.3 (0.2 to 0.4)	0 (0 to 0.1)	0.6 (0.4 to 0.8)	-94.3 (-96.3 to -91.3)	-26.4 (-56.3 to 17)	-94.7 (-96.6 to -91.7)
Central Europe, eastern Europe, and central Asia	Central Europe	North Macedonia	YLDs	All Ages	26 (16 to 41)	2 (1 to 3)	24 (14 to 38)	44 (28 to 65)	4 (2 to 7)	39 (25 to 60)	69.1 (19.2 to 120)	104.3 (52.3 to 176.4)	66 (14.1 to 119.2)
Central Europe, eastern Europe, and central Asia	Central Europe	North Macedonia	YLDs	Age-standardized	1.3 (0.8 to 2.1)	0.2 (0.1 to 0.3)	2.6 (1.6 to 4.1)	1.4 (0.9 to 2.1)	0.3 (0.2 to 0.4)	2.6 (1.7 to 3.9)	2.9 (-25.5 to 30.5)	18.7 (-10.6 to 57)	0.8 (-28.5 to 30.9)
Central Europe, eastern Europe, and central Asia	Central Europe	Montenegro	Incidence	All Ages	3 (2 to 3)	1 (1 to 1)	2 (2 to 3)	6 (5 to 7)	1 (1 to 1)	5 (4 to 6)	106.1 (80.9 to 134.4)	59.1 (32.8 to 91.3)	122.1 (91.6 to 158.4)
Central Europe, eastern Europe, and central Asia	Central Europe	Montenegro	Incidence	Age-standardized	0.5 (0.4 to 0.5)	0.2 (0.2 to 0.3)	0.7 (0.6 to 0.9)	0.7 (0.5 to 0.8)	0.2 (0.2 to 0.3)	1.1 (0.9 to 1.4)	44.9 (29 to 62.9)	4.9 (-10.4 to 23.3)	55.6 (35.8 to 77.8)
Central Europe, eastern Europe, and central Asia	Central Europe	Montenegro	Prevalence	All Ages	37 (31 to 46)	6 (4 to 8)	31 (25 to 39)	90 (72 to 110)	11 (8 to 15)	79 (62 to 98)	142.8 (110.6 to 182.1)	78.3 (38.7 to 119.5)	155.6 (118.5 to 202.2)
Central Europe, eastern Europe, and central Asia	Central Europe	Montenegro	Prevalence	Age-standardized	5.9 (4.9 to 7.3)	1.8 (1.3 to 2.3)	11.1 (9.1 to 13.8)	9.4 (7.6 to 11.5)	2 (1.5 to 2.7)	18.3 (14.5 to 22.6)	59.1 (38.9 to 83.3)	13.1 (-10.1 to 38.5)	64.3 (41.6 to 92.7)
Central Europe, eastern Europe, and central Asia	Central Europe	Montenegro	Deaths	All Ages	1 (1 to 2)	1 (0 to 1)	1 (0 to 1)	2 (1 to 3)	1 (1 to 2)	1 (1 to 1)	58.9 (6.8 to 129.1)	77 (-0.6 to 195.8)	35.7 (-11.8 to 106.7)
Central Europe, eastern Europe, and central Asia	Central Europe	Montenegro	Deaths	Age-standardized	0.2 (0.2 to 0.3)	0.2 (0.1 to 0.3)	0.2 (0.2 to 0.3)	0.2 (0.1 to 0.3)	0.2 (0.1 to 0.3)	0.2 (0.1 to 0.2)	-3.4 (-35.3 to 39.1)	9.9 (-37.9 to 83.1)	-21.3 (-48 to 17.9)
Central Europe, eastern Europe, and central Asia	Central Europe	Montenegro	DALYs	All Ages	30 (23 to 38)	14 (10 to 21)	16 (12 to 20)	49 (39 to 61)	23 (16 to 32)	25 (19 to 33)	63 (18 to 119.2)	63.7 (-1.9 to 160.4)	62.4 (19.6 to 117.9)
Central Europe, eastern Europe, and central Asia	Central Europe	Montenegro	DALYs	Age-standardized	4.8 (3.8 to 6.2)	4.1 (2.8 to 6.1)	5.8 (4.4 to 7.3)	5 (4 to 6.2)	4.4 (3.1 to 6)	5.8 (4.4 to 7.4)	3.9 (-25.4 to 39.1)	6.2 (-35.3 to 65)	0 (-25.9 to 32.6)
Central Europe, eastern Europe, and central Asia	Central Europe	Montenegro	YLLs	All Ages	24 (18 to 32)	13 (9 to 20)	11 (8 to 15)	35 (27 to 47)	21 (15 to 31)	14 (10 to 19)	46.7 (-0.3 to 114.7)	62.7 (-5.4 to 168.6)	27.4 (-18.9 to 97.6)
Central Europe, eastern Europe, and central Asia	Central Europe	Montenegro	YLLs	Age-standardized	3.9 (2.9 to 5.2)	3.8 (2.5 to 5.8)	4.1 (2.9 to 5.5)	3.6 (2.7 to 4.7)	4 (2.8 to 5.7)	3.1 (2.2 to 4.2)	-7.6 (-37.1 to 35.4)	5.7 (-37.3 to 70.4)	-23.8 (-50.3 to 16.3)
Central Europe, eastern Europe, and central Asia	Central Europe	Montenegro	YLDs	All Ages	6 (4 to 8)	1 (1 to 2)	5 (3 to 7)	13 (9 to 20)	2 (1 to 3)	12 (7 to 17)	132 (88.1 to 187.4)	77.6 (38.9 to 118.2)	143.2 (90.7 to 210.5)
Central Europe, eastern Europe, and central Asia	Central Europe	Montenegro	YLDs	Age-standardized	0.9 (0.6 to 1.3)	0.3 (0.2 to 0.4)	1.7 (1.1 to 2.4)	1.4 (0.9 to 2.1)	0.3 (0.2 to 0.5)	2.7 (1.7 to 4)	52.8 (25.6 to 88.7)	12.4 (-10.3 to 37.9)	57 (25.1 to 96.1)
Central Europe, eastern Europe, and central Asia	Central Europe	Poland	Incidence	All Ages	860 (716 to 1,051)	135 (108 to 168)	725 (596 to 904)	868 (740 to 1,015)	235 (188 to 289)	633 (548 to 732)	0.9 (-17.1 to 18.5)	74.1 (56.3 to 93.7)	-12.7 (-30.3 to 5.1)
Central Europe, eastern Europe, and central Asia	Central Europe	Poland	Incidence	Age-standardized	2 (1.7 to 2.5)	0.6 (0.5 to 0.7)	3.7 (3.1 to 4.7)	1.4 (1.2 to 1.6)	0.7 (0.5 to 0.8)	2.2 (1.9 to 2.5)	-32.6 (-44.9 to -21.6)	16 (5.4 to 28.2)	-40.4 (-52.1 to -28.9)
Central Europe, eastern Europe, and central Asia	Central Europe	Poland	Prevalence	All Ages	11,240 (9,073 to 14,610)	1,144 (918 to 1,444)	10,096 (7,979 to 13,444)	8,970 (7,678 to 10,485)	1,802 (1,417 to 2,284)	7,168 (6,138 to 8,360)	-20.2 (-38.9 to -2.8)	57.4 (44.8 to 69.5)	-29 (-46.6 to -11.3)
Central Europe, eastern Europe, and central Asia	Central Europe	Poland	Prevalence	Age-standardized	25.7 (20.7 to 33.5)	4.5 (3.6 to 5.7)	54 (42.8 to 71.5)	13.4 (11.6 to 15.6)	4.5 (3.6 to 5.7)	24.8 (21.3 to 28.8)	-47.8 (-60 to -36.3)	-0.4 (-6.9 to 5.8)	-54.1 (-65.5 to -43.3)
Central Europe, eastern Europe, and central Asia	Central Europe	Poland	Deaths	All Ages	238 (223 to 253)	7 (6 to 8)	231 (217 to 246)	52 (42 to 65)	6 (5 to 10)	45 (36 to 58)	-78.2 (-82.4 to -72.2)	-3.5 (-26.6 to 33.5)	-80.4 (-84.5 to -74.5)
Central Europe, eastern Europe, and central Asia	Central Europe	Poland	Deaths	Age-standardized	0.5 (0.5 to 0.6)	0 (0 to 0)	1.3 (1.2 to 1.4)	0.1 (0.1 to 0.1)	0 (0 to 0)	0.2 (0.1 to 0.2)	-86.6 (-89.1 to -82.8)	-48.3 (-60.6 to -29.4)	-87.8 (-90.4 to -84.2)
Central Europe, eastern Europe, and central Asia	Central Europe	Poland	DALYs	All Ages	7,145 (6,438 to 7,976)	299 (228 to 393)	6,846 (6,187 to 7,630)	2,311 (1,811 to 2,921)	370 (269 to 513)	1,941 (1,522 to 2,446)	-67.7 (-73.6 to -61.3)	24 (10.2 to 38.4)	-71.7 (-77 to -65.7)
Central Europe, eastern Europe, and central Asia	Central Europe	Poland	DALYs	Age-standardized	16 (14.4 to 17.9)	1.2 (0.9 to 1.5)	36.4 (33 to 40.5)	3.4 (2.7 to 4.4)	0.9 (0.7 to 1.3)	6.7 (5.2 to 8.4)	-78.6 (-82.5 to -74.3)	-21.3 (-29.8 to -12.6)	-81.7 (-85.1 to -77.8)
Central Europe, eastern Europe, and central Asia	Central Europe	Poland	YLLs	All Ages	5,548 (5,204 to 5,938)	120 (99 to 141)	5,428 (5,083 to 5,812)	989 (789 to 1,257)	93 (70 to 135)	896 (703 to 1,157)	-82.2 (-85.8 to -76.6)	-22 (-41.6 to 8.2)	-83.5 (-87.3 to -78)
Central Europe, eastern Europe, and central Asia	Central Europe	Poland	YLLs	Age-standardized	12.3 (11.6 to 13.2)	0.5 (0.4 to 0.5)	28.8 (27.1 to 30.8)	1.4 (1.1 to 1.8)	0.2 (0.2 to 0.3)	3.1 (2.4 to 3.9)	-88.4 (-90.8 to -84.7)	-51.9 (-64.2 to -32.9)	-89.4 (-91.8 to -85.9)
Central Europe, eastern Europe, and central Asia	Central Europe	Poland	YLDs	All Ages	1,597 (1,039 to 2,388)	179 (116 to 262)	1,418 (912 to 2,126)	1,322 (889 to 1,855)	277 (176 to 408)	1,045 (701 to 1,477)	-17.2 (-35.9 to 1.2)	54.7 (43.1 to 67.1)	-26.3 (-44.8 to -8.1)
Central Europe, eastern Europe, and central Asia	Central Europe	Poland	YLDs	Age-standardized	3.7 (2.4 to 5.4)	0.7 (0.5 to 1)	7.5 (4.9 to 11.3)	2 (1.3 to 2.8)	0.7 (0.4 to 1)	3.6 (2.4 to 5.1)	-45.5 (-57.8 to -33.7)	-1.2 (-7.5 to 5.1)	-52 (-63.7 to -40.2)
Central Europe, eastern Europe, and central Asia	Central Europe	Romania	Incidence	All Ages	245 (204 to 291)	44 (33 to 59)	201 (165 to 241)	228 (183 to 279)	43 (30 to 58)	186 (146 to 228)	-6.7 (-17.8 to 4.9)	-2.9 (-25.5 to 19.7)	-7.5 (-19 to 5.4)
Central Europe, eastern Europe, and central Asia	Central Europe	Romania	Incidence	Age-standardized	0.9 (0.7 to 1)	0.3 (0.2 to 0.4)	1.5 (1.3 to 1.8)	0.7 (0.6 to 0.9)	0.2 (0.2 to 0.3)	1.3 (1 to 1.6)	-14.3 (-23.6 to -5)	-21 (-36.6 to -6.4)	-13.4 (-23.5 to -2.7)
Central Europe, eastern Europe, and central Asia	Central Europe	Romania	Prevalence	All Ages	3,587 (2,989 to 4,332)	511 (375 to 691)	3,076 (2,546 to 3,764)	4,059 (3,255 to 4,959)	629 (446 to 859)	3,430 (2,706 to 4,283)	13.2 (-0.1 to 27)	23.1 (-3.7 to 52.8)	11.5 (-3.2 to 27)
Central Europe, eastern Europe, and central Asia	Central Europe	Romania	Prevalence	Age-standardized	12.7 (10.7 to 15.2)	3.4 (2.5 to 4.5)	24.2 (20.1 to 29.3)	11.6 (9.4 to 14.3)	2.9 (2.1 to 3.8)	22.4 (17.7 to 28)	-8.6 (-18.8 to 2.2)	-14 (-31.7 to 4.2)	-7.1 (-18.6 to 5.1)
Central Europe, eastern Europe, and central Asia	Central Europe	Romania	Deaths	All Ages	158 (125 to 199)	30 (14 to 42)	128 (97 to 164)	33 (25 to 41)	7 (5 to 10)	26 (20 to 33)	-79.4 (-86.2 to -70.2)	-76.5 (-85.9 to -43.4)	-80 (-87 to -69.6)
Central Europe, eastern Europe, and central Asia	Central Europe	Romania	Deaths	Age-standardized	0.6 (0.5 to 0.7)	0.2 (0.1 to 0.3)	1 (0.8 to 1.3)	0.1 (0.1 to 0.1)	0 (0 to 0)	0.2 (0.1 to 0.2)	-85 (-89.8 to -78.4)	-85.3 (-91.1 to -64.9)	-84.6 (-89.7 to -76.9)
Central Europe, eastern Europe, and central Asia	Central Europe	Romania	DALYs	All Ages	4,148 (3,270 to 5,166)	656 (367 to 865)	3,493 (2,693 to 4,498)	1,251 (972 to 1,574)	214 (157 to 292)	1,036 (785 to 1,325)	-69.9 (-78.3 to -57.9)	-67.3 (-78.6 to -38.1)	-70.3 (-79.4 to -57.1)
Central Europe, eastern Europe, and central Asia	Central Europe	Romania	DALYs	Age-standardized	14.4 (11.4 to 17.7)	4.2 (2.4 to 5.6)	26.7 (20.9 to 33.7)	3.5 (2.7 to 4.5)	1 (0.7 to 1.4)	6.7 (5.1 to 8.5)	-75.3 (-82.1 to -65.7)	-76.3 (-84.3 to -55.3)	-75.1 (-82.4 to -64.3)
Central Europe, eastern Europe, and central Asia	Central Europe	Romania	YLLs	All Ages	3,631 (2,802 to 4,607)	578 (287 to 786)	3,053 (2,261 to 3,987)	664 (506 to 848)	118 (83 to 169)	546 (407 to 720)	-81.7 (-88 to -72.6)	-79.6 (-87.7 to -54.5)	-82.1 (-88.7 to -71.9)
Central Europe, eastern Europe, and central Asia	Central Europe	Romania	YLLs	Age-standardized	12.5 (9.8 to 15.7)	3.7 (1.9 to 5)	23.3 (17.6 to 29.8)	1.8 (1.4 to 2.4)	0.6 (0.4 to 0.8)	3.4 (2.6 to 4.5)	-85.3 (-90.2 to -78)	-85.1 (-91 to -66.3)	-85.2 (-90.4 to -77)
Central Europe, eastern Europe, and central Asia	Central Europe	Romania	YLDs	All Ages	518 (323 to 760)	78 (48 to 121)	440 (271 to 661)	587 (367 to 868)	97 (57 to 148)	490 (305 to 745)	13.3 (-11.3 to 47.8)	23.7 (-7.4 to 58.1)	11.5 (-16.6 to 51.4)
Central Europe, eastern Europe, and central Asia	Central Europe	Romania	YLDs	Age-standardized	1.8 (1.2 to 2.7)	0.5 (0.3 to 0.8)	3.4 (2.1 to 5.2)	1.7 (1.1 to 2.5)	0.4 (0.3 to 0.7)	3.2 (2 to 4.9)	-7.5 (-27.2 to 19.4)	-12.4 (-33.3 to 11.1)	-6.3 (-28 to 24.9)
Central Europe, eastern Europe, and central Asia	Central Europe	Serbia	Incidence	All Ages	41 (33 to 52)	11 (8 to 15)	30 (24 to 38)	90 (72 to 112)	18 (13 to 25)	72 (57 to 91)	117.8 (85.9 to 157.1)	63.4 (26.3 to 111.7)	138.1 (98.8 to 187.9)
Central Europe, eastern Europe, and central Asia	Central Europe	Serbia	Incidence	Age-standardized	0.4 (0.3 to 0.4)	0.2 (0.1 to 0.2)	0.5 (0.4 to 0.7)	0.7 (0.5 to 0.8)	0.2 (0.2 to 0.3)	1.1 (0.9 to 1.4)	86.9 (62.3 to 120.8)	25.8 (3.7 to 53.4)	107 (75.3 to 147.8)
Central Europe, eastern Europe, and central Asia	Central Europe	Serbia	Prevalence	All Ages	556 (450 to 684)	145 (106 to 199)	411 (329 to 506)	1,561 (1,255 to 1,923)	263 (187 to 356)	1,298 (1,030 to 1,626)	181 (137.9 to 246.7)	81.8 (42.5 to 129.6)	216 (161.6 to 293.3)
Central Europe, eastern Europe, and central Asia	Central Europe	Serbia	Prevalence	Age-standardized	4.9 (4 to 6)	2.4 (1.8 to 3.3)	7.9 (6.4 to 9.6)	10.4 (8.4 to 12.7)	3.1 (2.2 to 4.1)	18.8 (15 to 23.4)	110.6 (80.3 to 155.8)	26 (0.5 to 54.8)	138.2 (99 to 197.3)
Central Europe, eastern Europe, and central Asia	Central Europe	Serbia	Deaths	All Ages	8 (6 to 11)	1 (0 to 1)	8 (5 to 10)	6 (4 to 8)	1 (1 to 2)	5 (3 to 6)	-29.3 (-52.4 to 7.8)	70.2 (-2.1 to 192.1)	-38.7 (-60.5 to -1.3)
Central Europe, eastern Europe, and central Asia	Central Europe	Serbia	Deaths	Age-standardized	0.1 (0.1 to 0.1)	0 (0 to 0)	0.2 (0.1 to 0.2)	0 (0 to 0)	0 (0 to 0)	0.1 (0 to 0.1)	-53.5 (-68.4 to -30)	1.2 (-39.9 to 71.6)	-59.5 (-73.6 to -36.3)
Central Europe, eastern Europe, and central Asia	Central Europe	Serbia	DALYs	All Ages	281 (215 to 358)	37 (26 to 50)	244 (183 to 316)	341 (252 to 454)	61 (43 to 85)	280 (204 to 381)	21.6 (-9.9 to 64.5)	65.4 (26.8 to 113.7)	15 (-18.8 to 63)
Central Europe, eastern Europe, and central Asia	Central Europe	Serbia	DALYs	Age-standardized	2.4 (1.9 to 3.1)	0.6 (0.4 to 0.9)	4.6 (3.5 to 5.9)	2.2 (1.6 to 3)	0.7 (0.5 to 1)	4 (2.9 to 5.4)	-8.8 (-31.9 to 21.8)	10.5 (-13.5 to 40.5)	-13.2 (-37.4 to 21.8)
Central Europe, eastern Europe, and central Asia	Central Europe	Serbia	YLLs	All Ages	194 (135 to 264)	14 (9 to 21)	180 (121 to 247)	114 (85 to 155)	20 (13 to 29)	94 (68 to 129)	-40.9 (-62.1 to -8.6)	43.5 (-16.6 to 143)	-47.5 (-66.7 to -15.8)
Central Europe, eastern Europe, and central Asia	Central Europe	Serbia	YLLs	Age-standardized	1.7 (1.2 to 2.2)	0.2 (0.2 to 0.4)	3.4 (2.3 to 4.5)	0.7 (0.5 to 1)	0.2 (0.1 to 0.3)	1.3 (0.9 to 1.7)	-57.6 (-72.3 to -35.6)	-9.7 (-45.4 to 51.8)	-62 (-75.7 to -40)
Central Europe, eastern Europe, and central Asia	Central Europe	Serbia	YLDs	All Ages	87 (57 to 127)	23 (14 to 35)	64 (42 to 93)	227 (146 to 339)	41 (25 to 63)	186 (117 to 284)	160.9 (107.2 to 235.5)	78.8 (36.6 to 128.5)	189.9 (119.5 to 284.5)
Central Europe, eastern Europe, and central Asia	Central Europe	Serbia	YLDs	Age-standardized	0.8 (0.5 to 1.1)	0.4 (0.2 to 0.6)	1.2 (0.8 to 1.8)	1.5 (1 to 2.2)	0.5 (0.3 to 0.7)	2.7 (1.7 to 4.1)	96.8 (57.9 to 151.8)	23.3 (-2.3 to 55.9)	119.8 (71.8 to 187.5)
Central Europe, eastern Europe, and central Asia	Central Europe	Slovakia	Incidence	All Ages	50 (41 to 72)	15 (12 to 19)	35 (27 to 56)	74 (60 to 92)	20 (15 to 25)	55 (43 to 68)	47.4 (13.9 to 70.2)	27.3 (5.8 to 50.5)	56.3 (11.3 to 87.3)
Central Europe, eastern Europe, and central Asia	Central Europe	Slovakia	Incidence	Age-standardized	0.9 (0.7 to 1.2)	0.5 (0.4 to 0.6)	1.3 (1 to 2.1)	0.9 (0.7 to 1.1)	0.4 (0.3 to 0.5)	1.4 (1.1 to 1.7)	2.6 (-20.2 to 18.2)	-12.7 (-26.7 to 1.6)	6 (-24.5 to 26.3)
Central Europe, eastern Europe, and central Asia	Central Europe	Slovakia	Prevalence	All Ages	678 (538 to 993)	159 (121 to 211)	519 (400 to 813)	1,141 (907 to 1,409)	240 (175 to 321)	901 (699 to 1,149)	68.3 (28.5 to 95.9)	51 (24.1 to 77.4)	73.6 (24.4 to 107.9)
Central Europe, eastern Europe, and central Asia	Central Europe	Slovakia	Prevalence	Age-standardized	11.4 (9.1 to 16.4)	4.7 (3.6 to 6.2)	20.4 (15.8 to 31.5)	12.7 (10.2 to 15.7)	4.6 (3.3 to 6.1)	23.2 (18.2 to 29.5)	11.7 (-14 to 30.3)	-2.2 (-18.8 to 15.6)	14 (-18 to 35.6)
Central Europe, eastern Europe, and central Asia	Central Europe	Slovakia	Deaths	All Ages	23 (18 to 33)	2 (1 to 2)	21 (16 to 32)	7 (5 to 9)	1 (0 to 1)	6 (4 to 8)	-70.5 (-79.4 to -58.8)	-65.3 (-77.3 to -46.2)	-71 (-80 to -58.3)
Central Europe, eastern Europe, and central Asia	Central Europe	Slovakia	Deaths	Age-standardized	0.4 (0.3 to 0.5)	0 (0 to 0.1)	0.9 (0.7 to 1.3)	0.1 (0.1 to 0.1)	0 (0 to 0)	0.2 (0.1 to 0.2)	-81.3 (-86.8 to -73.8)	-78.3 (-85.8 to -67)	-80.8 (-86.5 to -72.9)
Central Europe, eastern Europe, and central Asia	Central Europe	Slovakia	DALYs	All Ages	565 (457 to 770)	59 (45 to 79)	506 (400 to 709)	291 (218 to 380)	46 (31 to 68)	245 (182 to 323)	-48.4 (-63 to -31.8)	-21.8 (-43.9 to 4.8)	-51.5 (-66.4 to -34.6)
Central Europe, eastern Europe, and central Asia	Central Europe	Slovakia	DALYs	Age-standardized	9.4 (7.6 to 12.6)	1.8 (1.3 to 2.3)	19.8 (15.9 to 27.8)	3.2 (2.4 to 4.1)	0.9 (0.6 to 1.3)	6.3 (4.6 to 8.2)	-65.9 (-75.7 to -55.4)	-49.2 (-62.9 to -33.6)	-68.4 (-77.8 to -57.6)
Central Europe, eastern Europe, and central Asia	Central Europe	Slovakia	YLLs	All Ages	467 (369 to 673)	35 (24 to 47)	432 (336 to 637)	128 (91 to 174)	11 (7 to 15)	117 (82 to 162)	-72.6 (-81.3 to -60.7)	-69.2 (-79.8 to -52.8)	-72.9 (-82 to -60.6)
Central Europe, eastern Europe, and central Asia	Central Europe	Slovakia	YLLs	Age-standardized	7.7 (6.1 to 11.1)	1.1 (0.7 to 1.4)	17 (13.3 to 25.4)	1.4 (1 to 1.8)	0.2 (0.1 to 0.3)	3 (2.1 to 4.1)	-82.4 (-87.9 to -75.1)	-79.6 (-86.6 to -68.7)	-82.5 (-88 to -74.8)
Central Europe, eastern Europe, and central Asia	Central Europe	Slovakia	YLDs	All Ages	98 (62 to 153)	24 (15 to 38)	73 (45 to 124)	163 (102 to 240)	36 (21 to 56)	128 (77 to 194)	67.4 (23.6 to 117.5)	46.4 (10.6 to 89.4)	74.3 (18.9 to 142.3)
Central Europe, eastern Europe, and central Asia	Central Europe	Slovakia	YLDs	Age-standardized	1.6 (1 to 2.6)	0.7 (0.4 to 1.1)	2.9 (1.8 to 4.8)	1.8 (1.2 to 2.7)	0.7 (0.4 to 1.1)	3.3 (2 to 5)	11.7 (-17.3 to 42.4)	-4.4 (-25.9 to 20.7)	14.8 (-21.7 to 55.9)

Pneumoconiosis

7 GBD super region	21 GBD region	Location	Measure	Age	1990			2019			Percent change (1990 to 2019)		
					Both	Female	Male	Both	Female	Male	Both	Female	Male
Central Europe, eastern Europe, and central Asia	Central Europe	Slovenia	Incidence	All Ages	22 (18 to 29)	2 (2 to 3)	20 (16 to 27)	31 (25 to 38)	4 (3 to 6)	27 (21 to 33)	39.8 (7.2 to 60.4)	82.6 (45.4 to 129.9)	34.7 (1.8 to 56.9)
Central Europe, eastern Europe, and central Asia	Central Europe	Slovenia	Incidence	Age-standardized	0.9 (0.8 to 1.2)	0.2 (0.1 to 0.2)	1.8 (1.5 to 2.4)	0.9 (0.7 to 1.1)	0.2 (0.1 to 0.3)	1.6 (1.3 to 2)	-0.2 (-21.7 to 12.1)	18.9 (-3 to 46)	-9.8 (-30.8 to 2.4)
Central Europe, eastern Europe, and central Asia	Central Europe	Slovenia	Prevalence	All Ages	331 (267 to 421)	31 (23 to 42)	300 (240 to 388)	559 (451 to 692)	62 (44 to 84)	498 (394 to 621)	69.2 (41.8 to 90.6)	100.4 (54.4 to 151.5)	65.9 (37.4 to 89.8)
Central Europe, eastern Europe, and central Asia	Central Europe	Slovenia	Prevalence	Age-standardized	13.6 (11 to 17.2)	2.1 (1.6 to 2.8)	30.2 (24.2 to 39.1)	14.6 (11.7 to 17.9)	2.5 (1.8 to 3.4)	28.2 (22.4 to 34.9)	7.1 (-9.5 to 21.3)	17.3 (-8.5 to 47)	-6.5 (-23.9 to 7.3)
Central Europe, eastern Europe, and central Asia	Central Europe	Slovenia	Deaths	All Ages	5 (4 to 7)	0 (0 to 0)	5 (4 to 7)	7 (5 to 9)	1 (0 to 1)	6 (5 to 8)	32.5 (-10.6 to 94.1)	199.3 (81.3 to 393.8)	25.1 (-15.3 to 85.3)
Central Europe, eastern Europe, and central Asia	Central Europe	Slovenia	Deaths	Age-standardized	0.2 (0.2 to 0.3)	0 (0 to 0)	0.6 (0.4 to 0.7)	0.1 (0.1 to 0.2)	0 (0 to 0)	0.3 (0.2 to 0.4)	-32.5 (-54.5 to -1.1)	40.4 (-15.8 to 125.8)	-41.7 (-60.1 to -13.5)
Central Europe, eastern Europe, and central Asia	Central Europe	Slovenia	DALYs	All Ages	147 (115 to 186)	8 (6 to 12)	139 (108 to 178)	192 (148 to 242)	18 (13 to 24)	174 (133 to 222)	30.2 (-0.6 to 71.8)	115 (58.9 to 178.7)	25.1 (-5.9 to 67.1)
Central Europe, eastern Europe, and central Asia	Central Europe	Slovenia	DALYs	Age-standardized	6 (4.7 to 7.7)	0.6 (0.4 to 0.8)	14.6 (11.4 to 18.6)	4.6 (3.6 to 6)	0.7 (0.5 to 1)	9.4 (7.2 to 12.1)	-23 (-41.4 to 0.9)	24.9 (-6.7 to 60.9)	-35.4 (-51.5 to -13.9)
Central Europe, eastern Europe, and central Asia	Central Europe	Slovenia	YLLs	All Ages	100 (74 to 132)	4 (3 to 5)	97 (71 to 129)	112 (81 to 150)	9 (5 to 13)	103 (74 to 139)	11.5 (-26.8 to 66.6)	140 (44.3 to 291.3)	6.7 (-30.5 to 62.4)
Central Europe, eastern Europe, and central Asia	Central Europe	Slovenia	YLLs	Age-standardized	4.1 (3 to 5.4)	0.2 (0.2 to 0.3)	10.4 (7.7 to 13.6)	2.5 (1.8 to 3.4)	0.3 (0.2 to 0.5)	5.4 (3.9 to 7.3)	-38.1 (-59.6 to -7.8)	36.7 (-18.7 to 117.5)	-48.1 (-65.9 to -21.8)
Central Europe, eastern Europe, and central Asia	Central Europe	Slovenia	YLDs	All Ages	47 (29 to 68)	5 (3 to 7)	42 (26 to 62)	80 (51 to 119)	9 (6 to 15)	70 (44 to 106)	70.3 (27.4 to 118.9)	96.3 (50 to 151)	67.3 (20 to 121.8)
Central Europe, eastern Europe, and central Asia	Central Europe	Slovenia	YLDs	Age-standardized	1.9 (1.2 to 2.8)	0.3 (0.2 to 0.5)	4.2 (2.6 to 6.1)	2.1 (1.3 to 3.1)	0.4 (0.2 to 0.6)	4 (2.5 to 6)	9 (-16.7 to 41.2)	16.1 (-9 to 45.4)	-4.2 (-29 to 27.2)
		Eastern Europe	Incidence	All Ages	2,196 (1,880 to 2,589)	353 (269 to 447)	1,842 (1,556 to 2,192)	2,236 (1,873 to 2,637)	511 (389 to 664)	1,724 (1,433 to 2,043)	1.8 (-7.2 to 10.8)	44.7 (31 to 59.7)	-6.4 (-15.7 to 2.6)
		Eastern Europe	Incidence	Age-standardized	0.8 (0.7 to 0.9)	0.2 (0.2 to 0.3)	1.6 (1.4 to 1.9)	0.7 (0.6 to 0.8)	0.2 (0.2 to 0.3)	1.3 (1.1 to 1.5)	-11.1 (-18.3 to -4.5)	18.8 (8.3 to 29)	-19.2 (-26.3 to -12.6)
		Eastern Europe	Prevalence	All Ages	24,787 (20,583 to 30,090)	2,933 (2,223 to 3,821)	21,854 (17,810 to 26,909)	28,418 (23,528 to 34,452)	4,690 (3,474 to 6,150)	23,728 (19,087 to 29,233)	14.6 (2.9 to 24.6)	59.9 (38.5 to 80.7)	8.6 (-3.6 to 18.8)
		Eastern Europe	Prevalence	Age-standardized	8.9 (7.5 to 10.8)	1.6 (1.3 to 2.1)	21.9 (18.1 to 26.9)	8.5 (7.1 to 10.3)	2 (1.5 to 2.6)	18.4 (14.9 to 22.6)	-4.7 (-14.2 to 3.1)	21.5 (6.6 to 34.9)	-15.9 (-25.4 to -8.3)
		Eastern Europe	Deaths	All Ages	515 (410 to 664)	70 (40 to 90)	446 (343 to 592)	279 (239 to 340)	90 (69 to 144)	189 (160 to 224)	-45.8 (-60.5 to -27.7)	29.1 (-9.2 to 183.7)	-57.5 (-69.9 to -39.4)
		Eastern Europe	Deaths	Age-standardized	0.2 (0.1 to 0.2)	0 (0 to 0)	0.5 (0.4 to 0.7)	0.1 (0.1 to 0.1)	0 (0 to 0.1)	0.2 (0.1 to 0.2)	-56.9 (-68.3 to -42.7)	-1.3 (-29.6 to 113.6)	-69.9 (-78.6 to -57.7)
		Eastern Europe	DALYs	All Ages	15,008 (12,092 to 18,634)	1,729 (1,150 to 2,120)	13,279 (10,476 to 16,904)	9,648 (7,989 to 11,645)	2,184 (1,721 to 3,147)	7,464 (6,101 to 9,203)	-35.7 (-50.3 to -19.3)	26.3 (-2.5 to 123.4)	-43.8 (-58.3 to -27.1)
		Eastern Europe	DALYs	Age-standardized	5.3 (4.3 to 6.5)	1 (0.7 to 1.2)	13.5 (10.8 to 17.1)	2.9 (2.4 to 3.5)	1 (0.8 to 1.5)	5.8 (4.7 to 7.1)	-45.7 (-57.8 to -32.4)	3.5 (-20.2 to 85.7)	-57.3 (-67.8 to -44.4)
		Eastern Europe	YLLs	All Ages	11,401 (8,947 to 14,796)	1,280 (727 to 1,615)	10,121 (7,711 to 13,518)	5,510 (4,692 to 6,737)	1,483 (1,142 to 2,442)	4,027 (3,390 to 4,807)	-51.7 (-64.9 to -34.5)	15.8 (-17.4 to 161.4)	-60.2 (-72.2 to -43.1)
		Eastern Europe	YLLs	Age-standardized	4 (3.2 to 5.1)	0.7 (0.4 to 0.9)	10.4 (8 to 13.9)	1.6 (1.4 to 2)	0.7 (0.5 to 1.2)	3.1 (2.6 to 3.7)	-59.2 (-70.2 to -45.3)	-2.1 (-30.2 to 122.5)	-70 (-78.8 to -57.3)
		Eastern Europe	YLDs	All Ages	3,607 (2,318 to 5,172)	449 (274 to 677)	3,158 (2,020 to 4,631)	4,138 (2,727 to 5,967)	701 (435 to 1,044)	3,437 (2,235 to 5,007)	14.7 (0.4 to 29.3)	56.3 (32.6 to 78.9)	8.8 (-6.2 to 24.1)
		Eastern Europe	YLDs	Age-standardized	1.3 (0.8 to 1.9)	0.2 (0.2 to 0.4)	3.1 (2 to 4.5)	1.2 (0.8 to 1.8)	0.3 (0.2 to 0.4)	2.7 (1.7 to 3.9)	-4.1 (-16 to 7.4)	19.8 (3.3 to 35.9)	-14.9 (-26.8 to -3)
Central Europe, eastern Europe, and central Asia	Eastern Europe	Belarus	Incidence	All Ages	60 (50 to 71)	12 (9 to 15)	48 (40 to 58)	82 (67 to 99)	16 (11 to 22)	67 (54 to 80)	37.6 (23.9 to 52.4)	32.5 (7.6 to 64)	38.8 (23.2 to 55.3)
Central Europe, eastern Europe, and central Asia	Eastern Europe	Belarus	Incidence	Age-standardized	0.5 (0.4 to 0.6)	0.1 (0.1 to 0.2)	0.9 (0.8 to 1.1)	0.6 (0.5 to 0.7)	0.2 (0.1 to 0.2)	1.1 (0.9 to 1.3)	19.5 (8.7 to 31.9)	7.8 (-11.4 to 30.3)	18.5 (6.9 to 31.1)
Central Europe, eastern Europe, and central Asia	Eastern Europe	Belarus	Prevalence	All Ages	768 (624 to 938)	99 (72 to 133)	668 (533 to 834)	1,177 (944 to 1,444)	153 (108 to 214)	1,024 (810 to 1,274)	53.3 (35.7 to 73.7)	54.3 (23.9 to 102.9)	53.2 (33.6 to 75.8)
Central Europe, eastern Europe, and central Asia	Eastern Europe	Belarus	Prevalence	Age-standardized	6 (4.9 to 7.3)	1.2 (0.9 to 1.6)	13.8 (11.2 to 17)	7.7 (6.2 to 9.5)	1.4 (1 to 1.9)	17.3 (13.9 to 21.4)	27.4 (12.8 to 44.9)	15.8 (-6.8 to 49.2)	25.3 (10.1 to 43.1)
Central Europe, eastern Europe, and central Asia	Eastern Europe	Belarus	Deaths	All Ages	12 (8 to 17)	4 (2 to 6)	8 (5 to 13)	7 (4 to 10)	2 (1 to 5)	4 (3 to 7)	-44.2 (-65.1 to -7.3)	-44.4 (-72.4 to 122.3)	-44.1 (-66.6 to -4.8)
Central Europe, eastern Europe, and central Asia	Eastern Europe	Belarus	Deaths	Age-standardized	0.1 (0.1 to 0.1)	0 (0 to 0.1)	0.2 (0.1 to 0.3)	0 (0 to 0.1)	0 (0 to 0)	0.1 (0 to 0.1)	-56.8 (-72.9 to -28.6)	-59.1 (-79.5 to 63.8)	-57.3 (-74.2 to -27.7)
Central Europe, eastern Europe, and central Asia	Eastern Europe	Belarus	DALYs	All Ages	346 (257 to 469)	79 (43 to 116)	268 (181 to 386)	294 (215 to 397)	56 (36 to 98)	238 (167 to 335)	-15 (-39.2 to 14.6)	-28.8 (-55.2 to 84.6)	-11 (-39.2 to 25.4)
Central Europe, eastern Europe, and central Asia	Eastern Europe	Belarus	DALYs	Age-standardized	2.7 (2 to 3.6)	0.9 (0.5 to 1.4)	5.7 (3.9 to 8.1)	1.9 (1.4 to 2.6)	0.5 (0.3 to 0.9)	4 (2.9 to 5.6)	-29.3 (-49.1 to -5.2)	-44.3 (-64.8 to 47.9)	-28.6 (-50.8 to -0.6)
Central Europe, eastern Europe, and central Asia	Eastern Europe	Belarus	YLLs	All Ages	232 (162 to 342)	63 (27 to 99)	169 (98 to 281)	123 (76 to 184)	32 (17 to 72)	91 (52 to 144)	-47 (-66.7 to -14.7)	-48.6 (-73.7 to 94.5)	-46.4 (-67.1 to -3.8)
Central Europe, eastern Europe, and central Asia	Eastern Europe	Belarus	YLLs	Age-standardized	1.8 (1.3 to 2.6)	0.8 (0.3 to 1.2)	3.6 (2.1 to 6)	0.8 (0.5 to 1.2)	0.3 (0.2 to 0.7)	1.6 (0.9 to 2.4)	-56.4 (-72.2 to -31)	-58.4 (-78.4 to 58.6)	-57.3 (-73.8 to -25.5)
Central Europe, eastern Europe, and central Asia	Eastern Europe	Belarus	YLDs	All Ages	114 (73 to 170)	16 (10 to 24)	99 (62 to 150)	171 (107 to 258)	24 (14 to 38)	148 (91 to 229)	49.9 (14.6 to 90.5)	51.3 (20.2 to 100)	49.6 (9.3 to 96.8)
Central Europe, eastern Europe, and central Asia	Eastern Europe	Belarus	YLDs	Age-standardized	0.9 (0.6 to 1.3)	0.2 (0.1 to 0.3)	2 (1.3 to 3)	1.1 (0.7 to 1.7)	0.2 (0.1 to 0.3)	2.5 (1.5 to 3.8)	24.8 (-1.5 to 55.4)	13.9 (-8.5 to 47.5)	22.9 (-6.4 to 58.3)
Central Europe, eastern Europe, and central Asia	Eastern Europe	Estonia	Incidence	All Ages	8 (6 to 9)	1 (1 to 2)	6 (5 to 8)	12 (9 to 14)	3 (2 to 4)	9 (7 to 11)	48.5 (32.3 to 68.6)	77.2 (38.8 to 136.8)	42 (24.6 to 62.3)
Central Europe, eastern Europe, and central Asia	Eastern Europe	Estonia	Incidence	Age-standardized	0.4 (0.3 to 0.5)	0.1 (0.1 to 0.2)	0.8 (0.7 to 1)	0.6 (0.5 to 0.7)	0.1 (0.1 to 0.2)	1 (0.8 to 1.3)	37.2 (23.3 to 53.6)	31.3 (5.4 to 67.4)	29.8 (15.5 to 46.8)
Central Europe, eastern Europe, and central Asia	Eastern Europe	Estonia	Prevalence	All Ages	106 (85 to 131)	13 (9 to 18)	93 (73 to 117)	181 (143 to 225)	26 (18 to 38)	154 (121 to 197)	71 (50.6 to 95.7)	101.9 (52.7 to 170.8)	66.6 (44.1 to 94)
Central Europe, eastern Europe, and central Asia	Eastern Europe	Estonia	Prevalence	Age-standardized	5.3 (4.3 to 6.6)	1 (0.7 to 1.3)	12.2 (9.7 to 15.3)	7.6 (6 to 9.6)	1.3 (0.9 to 1.8)	16.4 (12.8 to 20.9)	43.4 (25.7 to 62.8)	34 (1.6 to 72.1)	34.2 (17.1 to 53.8)
Central Europe, eastern Europe, and central Asia	Eastern Europe	Estonia	Deaths	All Ages	1 (1 to 1)	0 (0 to 0)	1 (1 to 1)	0 (0 to 0)	0 (0 to 0)	0 (0 to 0)	-81.7 (-86.8 to -74.5)	-57.3 (-73.6 to -29.9)	-84.3 (-89.1 to -78)
Central Europe, eastern Europe, and central Asia	Eastern Europe	Estonia	Deaths	Age-standardized	0 (0 to 0)	0 (0 to 0)	0.1 (0.1 to 0.1)	0 (0 to 0)	0 (0 to 0)	0 (0 to 0)	-86.9 (-90.6 to -82)	-72.4 (-82.3 to -55.5)	-89.1 (-92.2 to -84.8)
Central Europe, eastern Europe, and central Asia	Eastern Europe	Estonia	DALYs	All Ages	33 (26 to 41)	3 (2 to 5)	29 (23 to 37)	29 (19 to 42)	4 (3 to 7)	24 (15 to 36)	-12.3 (-37.1 to 14.4)	38.3 (-2.9 to 82.4)	-17.9 (-43 to 11.9)
Central Europe, eastern Europe, and central Asia	Eastern Europe	Estonia	DALYs	Age-standardized	1.6 (1.3 to 2)	0.2 (0.2 to 0.3)	3.9 (3.1 to 4.9)	1.2 (0.8 to 1.8)	0.2 (0.1 to 0.3)	2.6 (1.6 to 3.8)	-24.4 (-45.5 to -1.9)	-4.9 (-30.1 to 19.8)	-34.4 (-53.8 to -12.4)
Central Europe, eastern Europe, and central Asia	Eastern Europe	Estonia	YLLs	All Ages	17 (13 to 21)	1 (1 to 2)	16 (12 to 20)	2 (2 to 3)	0 (0 to 1)	2 (1 to 3)	-85.3 (-90.1 to -79.1)	-63.5 (-77.1 to -39.4)	-87 (-91.5 to -81)
Central Europe, eastern Europe, and central Asia	Eastern Europe	Estonia	YLLs	Age-standardized	0.8 (0.6 to 1)	0.1 (0.1 to 0.1)	2.1 (1.7 to 2.7)	0.1 (0.1 to 0.1)	0 (0 to 0)	0.2 (0.1 to 0.3)	-88.1 (-92.1 to -83)	-69 (-80.2 to -52.3)	-90.2 (-93.5 to -85.9)
Central Europe, eastern Europe, and central Asia	Eastern Europe	Estonia	YLDs	All Ages	16 (10 to 23)	2 (1 to 3)	14 (9 to 20)	26 (16 to 39)	4 (2 to 7)	22 (13 to 34)	65.1 (25.4 to 115.6)	97.5 (46.6 to 170.5)	60.3 (15.9 to 119.1)
Central Europe, eastern Europe, and central Asia	Eastern Europe	Estonia	YLDs	Age-standardized	0.8 (0.5 to 1.2)	0.2 (0.1 to 0.2)	1.8 (1.1 to 2.6)	1.1 (0.7 to 1.7)	0.2 (0.1 to 0.3)	2.4 (1.4 to 3.6)	39.7 (8.4 to 79.2)	31.6 (-2.5 to 71.1)	30.7 (-2.2 to 76.6)
Central Europe, eastern Europe, and central Asia	Eastern Europe	Latvia	Incidence	All Ages	19 (15 to 26)	2 (1 to 2)	18 (14 to 25)	15 (12 to 18)	3 (2 to 4)	12 (10 to 15)	-24.2 (-42.8 to -11.6)	63.4 (26.8 to 114.3)	-32.2 (-49.6 to -21.6)
Central Europe, eastern Europe, and central Asia	Eastern Europe	Latvia	Incidence	Age-standardized	0.6 (0.5 to 0.8)	0.1 (0.1 to 0.1)	1.2 (1 to 1.7)	0.5 (0.4 to 0.6)	0.1 (0.1 to 0.1)	1 (0.8 to 1.2)	-14.5 (-34.2 to -2.5)	33.5 (8 to 70.6)	-23.2 (-41.6 to -12.2)
Central Europe, eastern Europe, and central Asia	Eastern Europe	Latvia	Prevalence	All Ages	290 (225 to 388)	13 (9 to 18)	277 (212 to 374)	258 (205 to 322)	25 (17 to 36)	234 (180 to 293)	-10.8 (-30.1 to 3.3)	90 (47.4 to 148.5)	-15.5 (-34.2 to -2)
Central Europe, eastern Europe, and central Asia	Eastern Europe	Latvia	Prevalence	Age-standardized	8.2 (6.4 to 11)	0.5 (0.4 to 0.8)	20.9 (16.2 to 28.2)	7.4 (5.8 to 9.4)	0.8 (0.6 to 1.1)	17.1 (13.1 to 21.4)	-9.4 (-28.3 to 5.1)	43.2 (13.4 to 84.8)	-18.4 (-36.8 to -5)
Central Europe, eastern Europe, and central Asia	Eastern Europe	Latvia	Deaths	All Ages	5 (4 to 6)	0 (0 to 0)	5 (4 to 6)	1 (0 to 1)	0 (0 to 0)	0 (0 to 1)	-88.2 (-91.7 to -83.2)	0.1 (-38.8 to 68.7)	-89.3 (-92.5 to -84.5)
Central Europe, eastern Europe, and central Asia	Eastern Europe	Latvia	Deaths	Age-standardized	0.1 (0.1 to 0.2)	0 (0 to 0)	0.4 (0.3 to 0.5)	0 (0 to 0)	0 (0 to 0)	0 (0 to 0)	-90.4 (-93.2 to -86.5)	-25.6 (-54.2 to 21.7)	-91 (-93.6 to -87)
Central Europe, eastern Europe, and central Asia	Eastern Europe	Latvia	DALYs	All Ages	138 (111 to 171)	3 (2 to 4)	135 (108 to 167)	47 (33 to 67)	5 (3 to 7)	42 (29 to 62)	-66 (-75.8 to -53.8)	52.9 (18.1 to 94.7)	-68.7 (-77.9 to -56.5)
Central Europe, eastern Europe, and central Asia	Eastern Europe	Latvia	DALYs	Age-standardized	3.8 (3.1 to 4.8)	0.1 (0.1 to 0.2)	10.4 (8.5 to 12.8)	1.3 (0.9 to 1.9)	0.2 (0.1 to 0.2)	3.1 (2.1 to 4.5)	-65.5 (-75.5 to -52.7)	16.3 (-7.8 to 46.5)	-70.5 (-78.9 to -59.8)
Central Europe, eastern Europe, and central Asia	Eastern Europe	Latvia	YLLs	All Ages	97 (76 to 122)	1 (1 to 1)	96 (75 to 121)	9 (7 to 12)	1 (0 to 1)	8 (6 to 11)	-90.8 (-93.6 to -86.4)	-24.4 (-53.8 to 31.1)	-91.4 (-94.2 to -87.2)
Central Europe, eastern Europe, and central Asia	Eastern Europe	Latvia	YLLs	Age-standardized	2.7 (2.1 to 3.4)	0 (0 to 0.1)	7.4 (6 to 9.3)	0.2 (0.2 to 0.3)	0 (0 to 0)	0.6 (0.4 to 0.8)	-91.6 (-94.2 to -87.7)	-35.7 (-60.6 to 15.8)	-92.3 (-94.7 to -88.8)
Central Europe, eastern Europe, and central Asia	Eastern Europe	Latvia	YLDs	All Ages	41 (24 to 65)	2 (1 to 3)	39 (23 to 63)	38 (23 to 58)	4 (2 to 6)	34 (21 to 53)	-8 (-35.4 to 30.4)	90.2 (47.3 to 148.3)	-13.1 (-39.6 to 25.9)
Central Europe, eastern Europe, and central Asia	Eastern Europe	Latvia	YLDs	Age-standardized	1.2 (0.7 to 1.8)	0.1 (0.1 to 0.1)	2.9 (1.7 to 4.6)	1.1 (0.7 to 1.7)	0.1 (0.1 to 0.2)	2.5 (1.5 to 3.9)	-6.2 (-32.6 to 30.6)	43.2 (13.4 to 84.6)	-15.2 (-39.4 to 19.2)
Central Europe, eastern Europe, and central Asia	Eastern Europe	Lithuania	Incidence	All Ages	37 (31 to 43)	11 (9 to 14)	26 (22 to 31)	39 (33 to 46)	12 (10 to 16)	27 (22 to 32)	5.6 (-6.1 to 18.3)	12.9 (-9.5 to 39.1)	2.5 (-8.8 to 15.1)
Central Europe, eastern Europe, and central Asia	Eastern Europe	Lithuania	Incidence	Age-standardized	0.9 (0.7 to 1)	0.4 (0.3 to 0.6)	1.4 (1.2 to 1.7)	0.8 (0.7 to 1)	0.4 (0.3 to 0.5)	1.4 (1.2 to 1.6)	-4.3 (-14.1 to 6.9)	-13 (-31.7 to 8)	-3.8 (-13.7 to 7.1)
Central Europe, eastern Europe, and central Asia	Eastern Europe	Lithuania	Prevalence	All Ages	355 (297 to 421)	82 (66 to 101)	273 (221 to 331)	492 (406 to 596)	127 (97 to 161)	366 (295 to 447)	38.8 (24.4 to 52.9)	54.3 (30.8 to 78.6)	34.2 (18.7 to 50.8)
Central Europe, eastern Europe, and central Asia	Eastern Europe	Lithuania	Prevalence	Age-standardized	8 (6.7 to 9.5)	3 (2.4 to 3.7)	15.5 (12.7 to 18.7)	9.1 (7.5 to 10.9)	3.3 (2.6 to 4)	17.8 (14.4 to 21.9)	13.9 (2.4 to 25.6)	8.2 (-5.7 to 23.8)	14.7 (1.4 to 28.5)
Central Europe, eastern Europe, and central Asia	Eastern Europe	Lithuania	Deaths	All Ages	2 (2 to 2)	0 (0 to 0)	2 (1 to 2)	1 (0 to 1)	0 (0 to 0)	0 (0 to 1)	-73.5 (-81 to -64)	-25.7 (-53.9 to 61.7)	-77.1 (-83.8 to -67.3)
Central Europe, eastern Europe, and central Asia	Eastern Europe	Lithuania	Deaths	Age-standardized	0 (0 to 0.1)	0 (0 to 0)	0.1 (0.1 to 0.1)	0 (0 to 0)	0 (0 to 0)	0 (0 to 0)	-80.8 (-86.2 to -73.7)	-51.7 (-69.2 to 6.3)	-82.6 (-87.8 to -75.3)
Central Europe, eastern Europe, and central Asia	Eastern Europe	Lithuania	DALYs	All Ages	98 (76 to 124)	15 (10 to 21)	83 (64 to 105)	81 (56 to 114)	20 (13 to 30)	61 (42 to 87)	-17.1 (-34.7 to 1.6)	32.2 (2.3 to 67.4)	-26.1 (-44 to -5.5)
Central Europe, eastern Europe, and central Asia	Eastern Europe	Lithuania	DALYs	Age-standardized	2.2 (1.8 to 2.8)	0.6 (0.4 to 0.8)	4.8 (3.8 to 6.1)	1.5 (1.1 to 2.1)	0.5 (0.3 to 0.8)	3 (2.1 to 4.2)	-32.2 (-46.3 to -17.3)	-4.5 (-20.8 to 14.6)	-38 (-53.2 to -20.8)
Central Europe, eastern Europe, and central Asia	Eastern Europe	Lithuania	YLLs	All Ages	45 (36 to 56)	2 (1 to 3)	43 (33 to 54)	10 (7 to 12)	1 (1 to 2)	8 (6 to 10)	-78.6 (-84.7 to -70.4)	-40.2 (-61.7 to 37.4)	-80.8 (-86.5 to -72.5)
Central Europe, eastern Europe, and central Asia	Eastern Europe	Lithuania	YLLs	Age-standardized	1.1 (0.8 to 1.3)	0.1 (0.1 to 0.1)	2.6 (2 to 3.2)	0.2 (0.1 to 0.2)	0 (0 to 0.1)	0.4 (0.3 to 0.5)	-82.6 (-87.4 to -76)	-53.3 (-69.3 to 8.5)	-84.1 (-88.6 to -77.2)
Central Europe, eastern Europe, and central Asia	Eastern Europe	Lithuania	YLDs	All Ages	53 (35 to 78)	13 (8 to 18)	40 (26 to 60)	71 (47 to 103)	19 (11 to 29)	53 (34 to 78)	35.4 (12.2 to 63.4)	45.9 (12.1 to 86)	32 (2.9 to 67.9)
Central Europe, eastern Europe, and central Asia	Eastern Europe	Lithuania	YLDs	Age-standardized	1.2 (0.8 to 1.7)	0.5 (0.3 to 0.7)	2.3 (1.5 to 3.4)	1.3 (0.9 to 2)	0.5 (0.3 to 0.7)	2.6 (1.7 to 3.8)	12.5 (-6.3 to 34.2)	4.6 (-13.8 to 25.8)	14 (-10.8 to 44.3)
Central Europe, eastern Europe, and central Asia	Eastern Europe	Republic of Moldova	Incidence	All Ages	17 (14 to 21)	3 (2 to 4)	15 (12 to 18)	29 (23 to 36)	5 (4 to 7)	24 (19 to 30)	68.3 (48.4 to 91.1)	81.7 (41.4 to 132.1)	65.7 (45 to 91.4)
Central Europe, eastern Europe, and central Asia	Eastern Europe	Republic of Moldova	Incidence	Age-standardized	0.4 (0.3 to 0.5)	0.1 (0.1 to 0.2)	0.7 (0.6 to 0.9)	0.5 (0.4 to 0.7)	0.1 (0.1 to 0.2)	1 (0.8 to 1.3)	40.8 (26 to 58)	32.1 (6.3 to 64.9)	39.1 (22.1 to 59.2)
Central Europe, eastern Europe, and central Asia	Eastern Europe	Republic of Moldova	Prevalence	All Ages	230 (182 to 290)	22 (16 to 32)	208 (162 to 266)	432 (343 to 547)	47 (32 to 66)	385 (298 to 497)	87.7 (63.5 to 116.4)	110.5 (59.4 to 177.1)	85.2 (60.4 to 116.3)
Central Europe, eastern Europe, and central Asia	Eastern Europe	Republic of Moldova	Prevalence	Age-standardized	5.2 (4.2 to 6.5)	1 (0.7 to 1.4)	11.2 (8.9 to 14.2)	7.7 (6.1 to 9.8)	1.3 (0.9 to 1.8)	16.5 (12.9 to 21.2)	47.1 (29 to 68.3)	34.9 (3.4 to 74.7)	47 (28.4 to 69.6)
Central Europe, eastern Europe, and central Asia	Eastern Europe	Republic of Moldova	Deaths	All Ages	1 (1 to 1)	0 (0 to 0)	1 (0 to 1)	0 (0 to 0)	0 (0 to 0)	0 (0 to 0)	-60.9 (-71.5 to -46.7)	-2.9 (-39.3 to 122.1)	-65.8 (-75.8 to -50.6)
Central Europe, eastern Europe, and central Asia	Eastern Europe	Republic of Moldova	Deaths	Age-standardized	0 (0 to 0)	0 (0 to 0)	0 (0 to 0)	0 (0 to 0)	0 (0 to 0)	0 (0 to 0)	-72.8 (-79.9 to -64)	-36.9 (-60.3 to 44.3)	-76.6 (-83.1 to -67.5)
Central Europe, eastern Europe, and central Asia	Eastern Europe	Republic of Moldova	DALYs	All Ages	49 (36 to 65)	5 (3 to 7)	44 (32 to 59)	68 (44 to 99)	8 (5 to 13)	60 (38 to 89)	37.9 (7.5 to 73.7)	70.7 (28.7 to 123.8)	34.5 (0.8 to 73.5)
Central Europe, eastern Europe, and central Asia	Eastern Europe	Republic of Moldova	DALYs	Age-standardized	1.1 (0.8 to 1.5)	0.2 (0.1 to 0.3)	2.4 (1.8 to 3.2)	1.2 (0.8 to 1.8)	0.2 (0.1 to 0.4)	2.6 (1.6 to 3.8)	8.9 (-14 to 35.7)	13.4 (-12.1 to 45.7)	5.6 (-19.8 to 34.8)
Central Europe, eastern Europe, and central Asia	Eastern Europe	Republic of Moldova	YLLs	All Ages	14 (11 to 18)	1 (1 to 2)	13 (10 to 17)	5 (4 to 6)	1 (1 to 1)	4 (3 to 5)	-66.1 (-75.9 to -52.4)	-33.3 (-58.4 to 53.3)	-69.1 (-78.5 to -54.6)
Central Europe, eastern Europe, and central Asia	Eastern Europe	Republic of Moldova	YLLs	Age-standardized	0.3 (0.3 to 0.4)	0 (0 to 0.1)	0.7 (0.6 to 0.9)	0.1 (0.1 to 0.1)	0 (0 to 0)	0.2 (0.1 to 0.2)	-74.1 (-81.2 to -64.8)	-47 (-66.7 to 25.3)	-77.1 (-83.8 to -67.3)
Central Europe, eastern Europe, and central Asia	Eastern Europe	Republic of Moldova	YLDs	All Ages	35 (22 to 50)	3 (2 to 6)	31 (19 to 46)	63 (39 to 94)	7 (4 to 12)	56 (33 to 85)	80.5 (42.8 to 129.5)	106.3 (54.4 to 176.7)	77.6 (36.3 to 131.9)
Central Europe, eastern Europe, and central Asia	Eastern Europe	Republic of Moldova	YLDs	Age-standardized	0.8 (0.5 to 1.1)	0.1 (0.1 to 0.2)	1.7 (1 to 2.4)	1.1 (0.7 to 1.7)	0.2 (0.1 to 0.3)	2.4 (1.5 to 3.6)	42.4 (14.7 to 76.9)	32.5 (1.5 to 70.7)	41.9 (10.6 to 84)
Central Europe, eastern Europe, and central Asia	Eastern Europe	Russian Federation	Incidence	All Ages	911 (766 to 1,079)	171 (127 to 223)	740 (612 to 887)	1,539 (1,293 to 1,813)	390 (299 to 504)	1,149 (956 to 1,362)	68.9 (52.8 to 85.9)	127.5 (95.7 to 176.5)	55.4 (40 to 72.5)
Central Europe, eastern Europe, and central Asia	Eastern Europe	Russian Federation	Incidence	Age-standardized	0.5 (0.4 to 0.6)	0.2 (0.1 to 0.2)	1 (0.9 to 1.2)	0.7 (0.6 to 0.8)	0.3 (0.2 to 0.3)	1.3 (1.1 to 1.5)	36.7 (24.7 to 48.3)	73 (50.7 to 104.9)	22.1 (11.3 to 33.3)
Central Europe, eastern Europe, and central Asia	Eastern Europe	Russian Federation	Prevalence	All Ages	9,361 (7,623 to 11,552)	1,367 (1,002 to 1,840)	7,995 (6,376 to 10,132)	19,273 (15,919 to 23,343)	3,534 (2,616 to 4,584)	15,739 (12,587 to 19,393)	105.9 (88.8 to 124.2)	158.6 (115 to 217.9)	96.9 (79.2 to 114.8)
Central Europe, eastern Europe, and central Asia	Eastern Europe	Russian Federation	Prevalence	Age-standardized	5.3 (4.3 to 6.5)	1.2 (0.9 to 1.6)	12.2 (9.9 to 15.3)	8.5 (7 to 10.3)	2.2 (1.7 to 2.9)	17.9 (14.4 to 22)	60.3 (48 to 73.1)	85.4 (55.9 to 123.3)	46.3 (34.4 to 58.5)
Central Europe, eastern Europe, and central Asia	Eastern Europe	Russian Federation	Deaths	All Ages	99 (93 to 107)	25 (17 to 28)	75 (69 to 81)	188 (159 to 234)	78 (59 to 120)	110 (90 to 134)	89.8 (59.1 to 132.8)	218.6 (141.5 to 461.6)	47.4 (19.7 to 83.2)
Central Europe, eastern Europe, and central Asia	Eastern Europe	Russian Federation	Deaths	Age-standardized	0.1 (0.1 to 0.1)	0 (0 to 0)	0.1 (0.1 to 0.2)	0.1 (0.1 to 0.1)	0 (0 to 0.1)	0.1 (0.1 to 0.2)	39 (16.4 to 71)	126.9 (71.4 to 304.4)	-3.1 (-20.7 to 19.4)
Central Europe, eastern Europe, and central Asia	Eastern Europe	Russian Federation	DALYs	All Ages	3,733 (3,176 to 4,452)	670 (524 to 796)	3,063 (2,570 to 3,674)	6,390 (5,212 to 7,846)	1,790 (1,413 to 2,530)	4,599 (3,667 to 5,705)	71.2 (52.7 to 93.3)	167.4 (114.9 to 313.7)	50.2 (31.6 to 70.6)
Central Europe, eastern Europe, and central Asia	Eastern Europe	Russian Federation	DALYs	Age-standardized	2.1 (1.8 to 2.5)	0.6 (0.5 to 0.7)	4.7 (4 to 5.7)	2.8 (2.3 to 3.4)	1.2 (1 to 1.7)	5.3 (4.2 to 6.5)	34.9 (20.7 to 52.7)	106.5 (66 to 223.5)	10.9 (-2.3 to 25.2)
Central Europe, eastern Europe, and central Asia	Eastern Europe	Russian Federation	YLLs	All Ages	2,297 (2,124 to 2,480)	453 (312 to 503)	1,843 (1,671 to 2,024)	3,592 (2,997 to 4,447)	1,268 (965 to 2,004)	2,324 (1,878 to 2,850)	56.4 (29.9 to 90.5)	179.6 (108.6 to 418.2)	26.1 (1.9 to 58.5)
Central Europe, eastern Europe, and central Asia	Eastern Europe	Russian Federation	YLLs	Age-standardized	1.3 (1.2 to 1.4)	0.4 (0.3 to 0.4)	2.9 (2.7 to 3.2)	1.6 (1.3 to 1.9)	0.9 (0.7 to 1.4)	2.7 (2.2 to 3.3)	23.5 (3 to 50.2)	121.7 (63.7 to 312.2)	-7.5 (-24.8 to 15.4)
Central Europe, eastern Europe, and central Asia	Eastern Europe	Russian Federation	YLDs	All Ages	1,436 (922 to 2,089)	216 (131 to 336)	1,220 (770 to 1,817)	2,798 (1,854 to 4,002)	523 (325 to 772)	2,275 (1,469 to 3,298)	94.8 (76.9 to 113.5)	141.7 (100.6 to 194.6)	86.5 (68.5 to 106.3)
Central Europe, eastern Europe, and central Asia	Eastern Europe	Russian Federation	YLDs	Age-standardized	0.8 (0.5 to 1.2)	0.2 (0.1 to 0.3)	1.8 (1.2 to 2.7)	1.2 (0.8 to 1.8)	0.3 (0.2 to 0.5)	2.6 (1.7 to 3.7)	52.6 (39.8 to 66)	74.5 (46.7 to 109.3)	39.9 (27.7 to 53.4)
Central Europe, eastern Europe, and central Asia	Eastern Europe	Ukraine	Incidence	All Ages	1,143 (952 to 1,405)	153 (114 to 202)	990 (811 to 1,231)	519 (435 to 613)	83 (60 to 115)	436 (362 to 518)	-54.6 (-62.2 to -48.5)	-45.8 (-57.8 to -32.2)	-55.9 (-63.5 to -49.8)
Central Europe, eastern Europe, and central Asia	Eastern Europe	Ukraine	Incidence	Age-standardized	1.6 (1.4 to 2)	0.3 (0.3 to 0.4)	3.4 (2.8 to 4.1)	0.8 (0.6 to 0.9)	0.2 (0.1 to 0.2)	1.5 (1.3 to 1.8)	-53.4 (-60.7 to -47.7)	-45 (-56.6 to -31.6)	-54.4 (-61.5 to -48.6)
Central Europe, eastern Europe, and central Asia	Eastern Europe	Ukraine	Prevalence	All Ages	13,678 (11,147 to 17,414)	1,337 (945 to 1,823)	12,341 (9,946 to 15,971)	6,605 (5,407 to 8,069)	779 (558 to 1,083)	5,825 (4,727 to 7,182)	-51.7 (-59.8 to -44.8)	-41.7 (-56.5 to -24.1)	-52.8 (-61.5 to -45.3)
Central Europe, eastern Europe, and central Asia	Eastern Europe	Ukraine	Prevalence	Age-standardized	19.1 (15.6 to 24.2)	2.9 (2.1 to 3.9)	47.4 (38.6 to 60.7)	9 (7.3 to 11)	1.5 (1.1 to 2.1)	20.7 (16.9 to 25.4)	-52.8 (-60.9 to -46.2)	-46.8 (-58.9 to -31.7)	-56.4 (-64.1 to -49.4)
Central Europe, eastern Europe, and central Asia	Eastern Europe	Ukraine	Deaths	All Ages	396 (294 to 537)	41 (18 to 60)	355 (260 to 499)	83 (64 to 107)	9 (6 to 21)	74 (54 to 97)	-79.1 (-86.7 to -67.8)	-76.9 (-87.9 to -13.9)	-79.3 (-87.2 to -66.3)
Central Europe, eastern Europe, and central Asia	Eastern Europe	Ukraine	Deaths	Age-standardized	0.5 (0.4 to 0.7)	0.1 (0 to 0.1)	1.5 (1.1 to 2.1)	0.1 (0.1 to 0.1)	0 (0 to 0)	0.3 (0.2 to 0.3)	-80.6 (-87.5 to -70.8)	-78.2 (-88.3 to -19.7)	-82.7 (-89.2 to -72.2)
Central Europe, eastern Europe, and central Asia	Eastern Europe	Ukraine	DALYs	All Ages	10,612 (8,000 to 13,975)	955 (544 to 1,274)	9,657 (7,135 to 13,005)	2,740 (2,159 to 3,481)	301 (209 to 527)	2,439 (1,886 to 3,123)	-74.2 (-82 to -62.9)	-68.5 (-80.6 to -19.6)	-74.7 (-82.8 to -61.8)
Central Europe, eastern Europe, and central Asia	Eastern Europe	Ukraine	DALYs	Age-standardized	14.5 (11.1 to 19)	2.1 (1.2 to 2.8)	37.2 (27.8 to 49.9)	3.7 (2.9 to 4.7)	0.7 (0.5 to 1.2)	8.5 (6.6 to 10.9)	-74.7 (-82.2 to -63.7)	-68.9 (-80.8 to -18.7)	-77.2 (-84.5 to -66.1)
Central Europe, eastern Europe, and central Asia	Eastern Europe	Ukraine	YLLs	All Ages	8,700 (6,310 to 11,915)	758 (361 to 1,069)	7,942 (5,621 to 11,183)	1,769 (1,336 to 2,342)	179 (118 to 403)	1,590 (1,161 to 2,155)	-79.7 (-87.1 to -68.4)	-76.3 (-87.2 to -13.2)	-80 (-87.7 to -67.1)
Central Europe, eastern Europe, and central Asia	Eastern Europe	Ukraine	YLLs	Age-standardized	11.9 (8.7 to 16.1)	1.7 (0.8 to 2.4)	30.7 (22.1 to 43.1)	2.4 (1.8 to 3.1)	0.4 (0.3 to 1)	5.5 (4 to 7.4)	-80.2 (-87.4 to -69.5)	-75.2 (-86.7 to -9.8)	-82.2 (-89 to -70.8)
Central Europe, eastern Europe, and central Asia	Eastern Europe	Ukraine	YLDs	All Ages	1,912 (1,221 to 2,855)	196 (115 to 308)	1,715 (1,095 to 2,586)	970 (624 to 1,439)	121 (72 to 188)	849 (546 to 1,282)	-49.2 (-61.6 to -34.2)	-38.3 (-56.2 to -15.1)	-50.5 (-63.7 to -34)
Central Europe, eastern Europe, and central Asia	Eastern Europe	Ukraine	YLDs	Age-standardized	2.7 (1.7 to 4)	0.4 (0.2 to 0.6)	6.5 (4.2 to 9.6)	1.3 (0.9 to 2)	0.2 (0.1 to 0.4)	3 (1.9 to 4.5)	-50.2 (-62.1 to -36.2)	-43.4 (-59.1 to -23.7)	-53.9 (-65.7 to -40.1)
		High-income	Incidence	All Ages	21,157 (17,726 to 24,660)	3,170 (2,352 to 4,056)	17,987 (15,190 to 21,151)	23,775 (20,130 to 27,535)	3,261 (2,557 to 4,014)	20,514 (17,421 to 23,660)	12.4 (1.4 to 25.6)	2.8 (-10.7 to 19.5)	14.1 (1.3 to 28.7)
		High-income	Incidence	Age-standardized	1.8 (1.5 to 2.1)	0.5 (0.4 to 0.7)	3.5 (2.9 to 4)	1.1 (1 to 1.3)	0.3 (0.3 to 0.4)	2.1 (1.8 to 2.4)	-36.3 (-43 to -29.4)	-36.5 (-45.1 to -26.1)	-38.4 (-44.9 to -31.2)
		High-income	Prevalence	All Ages	175,746 (146,391 to 215,269)	14,377 (12,092 to 17,316)	161,369 (133,737 to 198,960)	157,468 (140,410 to 177,902)	16,722 (14,355 to 19,466)	140,746 (125,121 to 159,497)	-10.4 (-24.8 to 1.7)	16.3 (6.1 to 26.4)	-12.8 (-27.9 to 0.1)
		High-income	Prevalence	Age-standardized	14.3 (11.9 to 17.5)	2.1 (1.8 to 2.6)	31.9 (26.5 to 39.3)	7.1 (6.4 to 8)	1.5 (1.3 to 1.8)	14.3 (12.8 to 16.1)	-49.9 (-58.1 to -43.3)	-28.8 (-35.1 to -21.9)	-55.2 (-62.5 to -48.8)
		High-income	Deaths	All Ages	6,937 (6,597 to 7,209)	205 (178 to 248)	6,732 (6,412 to 7,006)	6,067 (5,351 to 6,810)	380 (279 to 457)	5,687 (4,999 to 6,378)	-12.5 (-21.2 to -2.7)	85.1 (52.1 to 117.2)	-15.5 (-24 to -5.4)
		High-income	Deaths	Age-standardized	0.6 (0.5 to 0.6)	0 (0 to 0)	1.4 (1.3 to 1.4)	0.2 (0.2 to 0.3)	0 (0 to 0)	0.6 (0.5 to 0.6)	-55.9 (-60 to -51.1)	-3.9 (-18.7 to 11.9)	-59.5 (-63.3 to -54.8)
		High-income	DALYs	All Ages	155,272 (145,486 to 165,897)	5,792 (4,858 to 6,962)	149,480 (139,951 to 159,253)	106,158 (94,482 to 117,961)	7,782 (6,302 to 9,223)	98,376 (87,878 to 109,314)	-31.6 (-37.2 to -25.1)	34.3 (20.7 to 49.9)	-34.2 (-39.6 to -27.5)
		High-income	DALYs	Age-standardized	12.7 (11.9 to 13.6)	0.9 (0.7 to 1)	29.3 (27.4 to 31.2)	4.7 (4.2 to 5.3)	0.7 (0.6 to 0.8)	9.9 (8.9 to 11)	-62.8 (-65.7 to -59.3)	-18.8 (-25.1 to -10.2)	-66.1 (-68.8 to -62.7)
		High-income	YLLs	All Ages	130,577 (124,798 to 135,852)	3,560 (3,169 to 4,226)	127,017 (121,337 to 132,282)	83,534 (74,678 to 93,207)	5,159 (4,096 to 6,146)	78,375 (70,156 to 87,833)	-36 (-41.7 to -28.6)	44.9 (24 to 68.3)	-38.3 (-43.9 to -30.7)
		High-income	YLLs	Age-standardized	10.7 (10.2 to 11.1)	0.5 (0.5 to 0.6)	24.8 (23.7 to 25.8)	3.7 (3.3 to 4.1)	0.5 (0.4 to 0.5)	7.9 (7.1 to 8.8)	-65.5 (-68.4 to -61.6)	-12.9 (-22.6 to -0.2)	-68.3 (-71 to -64.5)
		High-income	YLDs	All Ages	24,695 (16,403 to 35,798)	2,232 (1,477 to 3,226)	22,463 (14,826 to 32,516)	22,624 (15,692 to 31,460)	2,623 (1,755 to 3,720)	20,001 (13,912 to 27,748)	-8.4 (-22.9 to 3.9)	17.5 (6.8 to 28.2)	-11 (-26 to 2.6)
		High-income	YLDs	Age-standardized	2 (1.3 to 2.9)	0.3 (0.2 to 0.5)	4.4 (2.9 to 6.4)	1 (0.7 to 1.4)	0.2 (0.2 to 0.3)	2 (1.4 to 2.8)	-48.5 (-56.8 to -41.6)	-28.1 (-34.6 to -21.4)	-53.9 (-61.4 to -47.3)
		High-income Asia Pacific	Incidence	All Ages	6,466 (5,375 to 7,809)	402 (305 to 512)	6,063 (4,988 to 7,406)	6,622 (5,694 to 7,638)	548 (420 to 694)	6,074 (5,210 to 6,993)	2.4 (-11.7 to 18.7)	36.1 (10 to 67)	0.2 (-14.2 to 17.3)
		High-income Asia Pacific	Incidence	Age-standardized	3.1 (2.6 to 3.8)	0.4 (0.3 to 0.5)	6.7 (5.6 to 8.1)	1.5 (1.3 to 1.7)	0.2 (0.2 to 0.3)	2.9 (2.5 to 3.4)	-53.3 (-58.7 to -46.9)	-42.2 (-51.7 to -32.8)	-56.2 (-61.5 to -49.8)
		High-income Asia Pacific	Prevalence	All Ages	63,288 (51,216 to 80,082)	1,890 (1,474 to 2,425)	61,398 (49,483 to 78,066)	59,303 (49,820 to 70,231)	3,368 (2,635 to 4,333)	55,935 (47,042 to 66,269)	-6.3 (-23 to 8.7)	78.2 (52.5 to 103.3)	-8.9 (-25.6 to 6)
		High-income Asia Pacific	Prevalence	Age-standardized	32 (26 to 40.2)	1.7 (1.4 to 2.2)	77.5 (62.8 to 97.2)	11.6 (9.8 to 13.8)	1 (0.8 to 1.3)	25.5 (21.5 to 30.3)	-63.7 (-69.7 to -58.4)	-40.1 (-47.3 to -33)	-67 (-72.1 to -62.1)
		High-income Asia Pacific	Deaths	All Ages	1,218 (1,105 to 1,333)	45 (35 to 51)	1,173 (1,060 to 1,284)	1,565 (1,249 to 1,938)	99 (64 to 139)	1,466 (1,179 to 1,825)	28.5 (4 to 60.8)	117.9 (62 to 212.2)	25 (1.2 to 56)
		High-income Asia Pacific	Deaths	Age-standardized	0.6 (0.6 to 0.7)	0 (0 to 0)	1.4 (1.3 to 1.6)	0.3 (0.2 to 0.4)	0 (0 to 0)	0.6 (0.5 to 0.8)	-53.4 (-61.9 to -41.2)	-35.7 (-49.5 to -6.4)	-54.5 (-62.8 to -43.4)
		High-income Asia Pacific	DALYs	All Ages	35,168 (30,854 to 39,523)	1,087 (896 to 1,262)	34,081 (29,918 to 38,381)	30,113 (25,019 to 36,034)	1,638 (1,263 to 2,109)	28,475 (23,626 to 34,231)	-14.4 (-26.9 to 3.3)	50.7 (26.8 to 98.9)	-16.4 (-28.6 to 1.6)
		High-income Asia Pacific	DALYs	Age-standardized	17.2 (15.1 to 19.4)	1 (0.8 to 1.1)	39.3 (34.5 to 44.3)	6.1 (5.1 to 7.3)	0.6 (0.4 to 0.7)	13.2 (10.9 to 15.8)	-64.7 (-69.6 to -57.1)	-42.9 (-50.8 to -23.1)	-66.5 (-71.1 to -59.4)
		High-income Asia Pacific	YLLs	All Ages	26,412 (23,588 to 29,182)	791 (612 to 889)	25,621 (22,871 to 28,352)	21,721 (17,709 to 27,139)	1,109 (793 to 1,547)	20,611 (16,787 to 25,912)	-17.8 (-33.6 to 5.6)	40.2 (8.5 to 106.6)	-19.6 (-35 to 3.8)
		High-income Asia Pacific	YLLs	Age-standardized	12.8 (11.5 to 14.1)	0.7 (0.5 to 0.8)	28.7 (25.7 to 31.6)	4.4 (3.6 to 5.6)	0.4 (0.3 to 0.5)	9.5 (7.8 to 12)	-65.5 (-71.8 to -55.4)	-44.2 (-54.5 to -15.1)	-66.7 (-72.8 to -57.3)
		High-income Asia Pacific	YLDs	All Ages	8,756 (5,758 to 12,572)	296 (191 to 443)	8,460 (5,562 to 12,203)	8,392 (5,742 to 11,821)	529 (334 to 775)	7,864 (5,393 to 11,047)	-4.2 (-21.4 to 11.7)	78.8 (52.7 to 104.2)	-7.1 (-24.2 to 8.8)
		High-income Asia Pacific	YLDs	Age-standardized	4.4 (2.9 to 6.3)	0.3 (0.2 to 0.4)	10.6 (7 to 15.2)	1.7 (1.1 to 2.4)	0.2 (0.1 to 0.2)	3.6 (2.5 to 5.1)	-62.3 (-68.5 to -56.5)	-39.7 (-47.2 to -32.6)	-65.8 (-71.2 to -60.4)
High income	High-income Asia Pacific	Brunei Darussalam	Incidence	All Ages	1 (0 to 1)	0 (0 to 0)	0 (0 to 1)	2 (1 to 2)	0 (0 to 0)	1 (1 to 2)	187.4 (157.2 to 214.9)	116.9 (67.8 to 185.7)	203.3 (174.7 to 233.2)
High income	High-income Asia Pacific	Brunei Darussalam	Incidence	Age-standardized	0.6 (0.5 to 0.7)	0.2 (0.1 to 0.2)	1.1 (0.9 to 1.3)	0.6 (0.5 to 0.7)	0.2 (0.1 to 0.2)	1.2 (1 to 1.4)	1.8 (-5.5 to 9.6)	-12.9 (-27.7 to 6.8)	7.3 (-0.9 to 16.4)
High income	High-income Asia Pacific	Brunei Darussalam	Prevalence	All Ages	3 (2 to 4)	0 (0 to 0)	3 (2 to 4)	9 (8 to 12)	1 (0 to 1)	9 (7 to 12)	211.1 (179.2 to 246)	118.5 (82.1 to 167.5)	219.3 (186.8 to 256.8)
High income	High-income Asia Pacific	Brunei Darussalam	Prevalence	Age-standardized	3.7 (3 to 4.6)	0.5 (0.4 to 0.7)	8.6 (6.9 to 10.8)	4.2 (3.3 to 5.3)	0.4 (0.3 to 0.5)	10 (8 to 12.6)	12.1 (1.8 to 23.1)	-18.3 (-31.9 to -0.2)	16.7 (6.1 to 28.8)
High income	High-income Asia Pacific	Brunei Darussalam	Deaths	All Ages	0 (0 to 0)	0 (0 to 0)	0 (0 to 0)	0 (0 to 0)	0 (0 to 0)	0 (0 to 0)	47.6 (-8.1 to 151.5)	64.1 (-7.7 to 228.9)	3.2 (-40.8 to 81.2)
High income	High-income Asia Pacific	Brunei Darussalam	Deaths	Age-standardized	0.1 (0.1 to 0.1)	0.1 (0.1 to 0.2)	0 (0 to 0.1)	0 (0 to 0.1)	0.1 (0 to 0.1)	0 (0 to 0)	-44.7 (-65.5 to -7.3)	-41.2 (-66.2 to 11.7)	-60.9 (-76.6 to -33.1)
High income	High-income Asia Pacific	Brunei Darussalam	DALYs	All Ages	2 (1 to 2)	1 (1 to 2)	1 (1 to 1)	3 (2 to 4)	2 (1 to 2)	2 (1 to 2)	77.3 (24.3 to 158.8)	49.6 (-12 to 198.1)	113.9 (58.2 to 178)
High income	High-income Asia Pacific	Brunei Darussalam	DALYs	Age-standardized	2.1 (1.5 to 2.7)	2.1 (1.2 to 3.2)	2.1 (1.5 to 2.8)	1.3 (1 to 1.7)	1.2 (0.8 to 1.7)	1.7 (1.2 to 2.4)	-34.7 (-53.6 to -7.6)	-46.3 (-68.6 to 4.5)	-18.8 (-38.9 to 5)
High income	High-income Asia Pacific	Brunei Darussalam	YLLs	All Ages	1 (1 to 2)	1 (1 to 1)	0 (0 to 1)	2 (1 to 3)	1 (1 to 2)	0 (0 to 1)	34.8 (-15.7 to 137)	46.9 (-16.4 to 202.6)	2.8 (-40.6 to 81.2)
High income	High-income Asia Pacific	Brunei Darussalam	YLLs	Age-standardized	1.5 (1 to 2.1)	2.1 (1.1 to 3.2)	0.9 (0.6 to 1.3)	0.8 (0.5 to 1)	1.1 (0.7 to 1.6)	0.3 (0.2 to 0.4)	-50.3 (-68.8 to -16.1)	-47.4 (-69.6 to 5.4)	-64.1 (-78.5 to -38.9)
High income	High-income Asia Pacific	Brunei Darussalam	YLDs	All Ages	0 (0 to 1)	0 (0 to 0)	0 (0 to 1)	1 (1 to 2)	0 (0 to 0)	1 (1 to 2)	208.2 (149.5 to 290.2)	118.5 (82.5 to 166.7)	216.8 (153.6 to 304.7)
High income	High-income Asia Pacific	Brunei Darussalam	YLDs	Age-standardized	0.5 (0.3 to 0.8)	0.1 (0.1 to 0.1)	1.2 (0.8 to 1.8)	0.6 (0.4 to 0.9)	0.1 (0 to 0.1)	1.4 (0.9 to 2)	9.9 (-12.3 to 41.1)	-18.4 (-31.9 to -0.6)	14.7 (-9.9 to 48.5)
High income	High-income Asia Pacific	Japan	Incidence	All Ages	6,167 (5,098 to 7,481)	386 (292 to 490)	5,781 (4,738 to 7,086)	5,832 (4,990 to 6,772)	513 (391 to 654)	5,319 (4,537 to 6,156)	-5.4 (-19.2 to 10.6)	32.8 (6.8 to 63.9)	-8 (-21.9 to 9.2)
High income	High-income Asia Pacific	Japan	Incidence	Age-standardized	3.6 (3 to 4.3)	0.4 (0.3 to 0.6)	7.5 (6.2 to 9.1)	1.7 (1.4 to 1.9)	0.3 (0.2 to 0.3)	3.3 (2.8 to 3.8)	-53.3 (-58.9 to -46.8)	-39.8 (-49.8 to -28.8)	-56.3 (-61.7 to -49.6)
High income	High-income Asia Pacific	Japan	Prevalence	All Ages	60,886 (48,912 to 77,310)	1,840 (1,427 to 2,375)	59,046 (47,257 to 75,216)	52,224 (44,054 to 61,549)	3,226 (2,512 to 4,176)	48,997 (41,192 to 57,978)	-14.2 (-30 to -0.1)	75.3 (49.6 to 100.7)	-17 (-32.7 to -3.1)
High income	High-income Asia Pacific	Japan	Prevalence	Age-standardized	35.8 (28.9 to 45.2)	2 (1.5 to 2.5)	85.2 (68.7 to 107.5)	12.8 (10.8 to 15.2)	1.2 (1 to 1.6)	27.7 (23.3 to 32.8)	-64.2 (-70.4 to -58.8)	-37.1 (-44.7 to -29.7)	-67.5 (-72.8 to -62.5)
High income	High-income Asia Pacific	Japan	Deaths	All Ages	826 (773 to 887)	38 (30 to 44)	788 (738 to 847)	1,138 (866 to 1,436)	81 (51 to 118)	1,057 (807 to 1,335)	37.8 (10.4 to 67.6)	111 (53.5 to 194.5)	34.2 (7.9 to 63.7)
High income	High-income Asia Pacific	Japan	Deaths	Age-standardized	0.5 (0.5 to 0.5)	0 (0 to 0)	1.1 (1.1 to 1.2)	0.2 (0.2 to 0.3)	0 (0 to 0)	0.5 (0.4 to 0.7)	-51.4 (-60.4 to -40.8)	-37.2 (-50.7 to -12.9)	-52.6 (-61.4 to -42.3)
High income	High-income Asia Pacific	Japan	DALYs	All Ages	24,341 (21,326 to 28,140)	945 (770 to 1,114)	23,396 (20,474 to 27,041)	21,566 (17,362 to 26,501)	1,367 (1,023 to 1,779)	20,199 (16,276 to 24,856)	-11.4 (-23.9 to 3.6)	44.6 (22 to 81.4)	-13.7 (-26.3 to 1.4)
High income	High-income Asia Pacific	Japan	DALYs	Age-standardized	14.1 (12.4 to 16.3)	1 (0.8 to 1.2)	32.4 (28.3 to 37.6)	5.2 (4.2 to 6.4)	0.6 (0.5 to 0.8)	11.2 (9 to 13.7)	-63.3 (-68.1 to -57.3)	-41.9 (-49 to -24.3)	-65.5 (-70.2 to -59.7)
High income	High-income Asia Pacific	Japan	YLLs	All Ages	15,935 (15,013 to 17,148)	658 (506 to 742)	15,277 (14,374 to 16,461)	14,211 (10,990 to 18,018)	861 (579 to 1,243)	13,350 (10,347 to 17,005)	-10.8 (-27.8 to 9.4)	31 (0.4 to 85.9)	-12.6 (-29.3 to 7.4)
High income	High-income Asia Pacific	Japan	YLLs	Age-standardized	9.2 (8.7 to 9.9)	0.7 (0.5 to 0.8)	20.8 (19.5 to 22.3)	3.4 (2.7 to 4.3)	0.4 (0.3 to 0.6)	7.3 (5.7 to 9.2)	-63.5 (-69.8 to -55.2)	-44.2 (-53.7 to -18.7)	-65 (-71.2 to -57.1)
High income	High-income Asia Pacific	Japan	YLDs	All Ages	8,407 (5,527 to 12,067)	288 (186 to 430)	8,119 (5,337 to 11,698)	7,355 (5,020 to 10,367)	506 (319 to 745)	6,849 (4,666 to 9,629)	-12.5 (-28.4 to 2)	75.8 (49.7 to 102)	-15.6 (-31.6 to -0.8)
High income	High-income Asia Pacific	Japan	YLDs	Age-standardized	4.9 (3.3 to 7.1)	0.3 (0.2 to 0.5)	11.6 (7.7 to 16.7)	1.8 (1.2 to 2.6)	0.2 (0.1 to 0.3)	3.9 (2.7 to 5.5)	-62.9 (-69.2 to -57)	-36.7 (-44.6 to -29)	-66.4 (-71.8 to -60.9)
High income	High-income Asia Pacific	Republic of Korea	Incidence	All Ages	282 (241 to 333)	13 (8 to 21)	269 (229 to 319)	739 (620 to 884)	31 (21 to 41)	708 (594 to 849)	161.4 (126.7 to 205.3)	132.5 (73.2 to 218.1)	162.9 (127 to 208.2)
High income	High-income Asia Pacific	Republic of Korea	Incidence	Age-standardized	0.9 (0.8 to 1)	0.1 (0 to 0.1)	2.1 (1.8 to 2.4)	0.8 (0.7 to 1)	0.1 (0 to 0.1)	1.7 (1.4 to 2)	-7.8 (-18.1 to 5.9)	-1 (-16.1 to 16.7)	-18 (-26.9 to -6)
High income	High-income Asia Pacific	Republic of Korea	Prevalence	All Ages	2,317 (1,953 to 2,800)	44 (34 to 56)	2,273 (1,910 to 2,750)	6,701 (5,537 to 8,273)	130 (103 to 162)	6,571 (5,413 to 8,127)	189.2 (141.5 to 247.8)	196.9 (145.4 to 257.7)	189.1 (140.5 to 249.2)
High income	High-income Asia Pacific	Republic of Korea	Prevalence	Age-standardized	8.3 (7 to 10)	0.3 (0.2 to 0.4)	22 (18.6 to 26.2)	7.5 (6.2 to 9.3)	0.3 (0.2 to 0.3)	17.7 (14.7 to 21.7)	-9.8 (-23.1 to 6.9)	-3.7 (-15.5 to 10.8)	-19.7 (-30.4 to -5.2)
High income	High-income Asia Pacific	Republic of Korea	Deaths	All Ages	391 (301 to 482)	7 (4 to 10)	384 (295 to 474)	426 (345 to 532)	18 (12 to 26)	409 (329 to 514)	9 (-18.9 to 57.4)	156.9 (47.9 to 414.8)	6.3 (-21.5 to 54.3)
High income	High-income Asia Pacific	Republic of Korea	Deaths	Age-standardized	1.3 (1 to 1.6)	0 (0 to 0.1)	3.2 (2.4 to 3.9)	0.5 (0.4 to 0.6)	0 (0 to 0)	1.1 (0.9 to 1.4)	-63.3 (-72.6 to -47.4)	-31.6 (-59.9 to 44.8)	-65.1 (-74.3 to -49.7)
High income	High-income Asia Pacific	Republic of Korea	DALYs	All Ages	10,796 (8,311 to 13,326)	138 (87 to 188)	10,657 (8,171 to 13,178)	8,479 (6,923 to 10,566)	266 (189 to 380)	8,213 (6,671 to 10,273)	-21.5 (-42 to 13.1)	92.7 (21.5 to 256.3)	-22.9 (-43.1 to 11.4)
High income	High-income Asia Pacific	Republic of Korea	DALYs	Age-standardized	33.2 (25.8 to 40.6)	0.9 (0.5 to 1.2)	77.4 (60 to 94.8)	9.4 (7.7 to 11.6)	0.5 (0.4 to 0.7)	20.8 (17.2 to 25.9)	-71.7 (-78.9 to -59.7)	-40.3 (-62.8 to 16.6)	-73.1 (-79.7 to -62.3)
High income	High-income Asia Pacific	Republic of Korea	YLLs	All Ages	10,459 (8,002 to 13,025)	131 (80 to 180)	10,328 (7,873 to 12,905)	7,496 (6,035 to 9,506)	245 (169 to 357)	7,250 (5,825 to 9,245)	-28.3 (-47.2 to 6.1)	87 (13.2 to 258.4)	-29.8 (-48.7 to 4.6)
High income	High-income Asia Pacific	Republic of Korea	YLLs	Age-standardized	32 (24.6 to 39.5)	0.8 (0.5 to 1.1)	74.3 (56.8 to 91.5)	8.3 (6.7 to 10.5)	0.5 (0.3 to 0.7)	18.3 (14.8 to 23)	-74.1 (-81 to -61.9)	-42.3 (-65.5 to 18.5)	-75.4 (-81.7 to -64.3)
High income	High-income Asia Pacific	Republic of Korea	YLDs	All Ages	337 (216 to 488)	7 (4 to 11)	330 (211 to 480)	983 (639 to 1,430)	21 (14 to 31)	962 (625 to 1,401)	191.7 (124.1 to 285.1)	198.2 (148.3 to 257.2)	191.6 (122.6 to 287.2)
High income	High-income Asia Pacific	Republic of Korea	YLDs	Age-standardized	1.2 (0.8 to 1.7)	0 (0 to 0.1)	3.1 (2 to 4.4)	1.1 (0.7 to 1.6)	0 (0 to 0.1)	2.6 (1.7 to 3.7)	-7.7 (-28.4 to 21.1)	-3.5 (-15.2 to 10.6)	-17.7 (-36.2 to 7.4)
High income	High-income Asia Pacific	Singapore	Incidence	All Ages	16 (13 to 19)	3 (2 to 5)	13 (10 to 15)	50 (41 to 60)	4 (3 to 6)	45 (36 to 56)	212 (176.5 to 255.2)	26.1 (-12.2 to 83.3)	262.9 (221.2 to 308.1)
High income	High-income Asia Pacific	Singapore	Incidence	Age-standardized	0.7 (0.6 to 0.9)	0.3 (0.2 to 0.3)	1.3 (1.1 to 1.5)	0.6 (0.5 to 0.8)	0.1 (0.1 to 0.2)	1.2 (1 to 1.5)	-11.2 (-20.5 to -0.9)	-54.4 (-67.3 to -37.6)	-6.8 (-16.5 to 4)
High income	High-income Asia Pacific	Singapore	Prevalence	All Ages	82 (68 to 101)	6 (5 to 8)	76 (62 to 95)	369 (294 to 463)	11 (9 to 14)	358 (284 to 451)	347 (304.2 to 398.2)	80.7 (45.1 to 129.8)	368.3 (322.7 to 423.7)
High income	High-income Asia Pacific	Singapore	Prevalence	Age-standardized	4.2 (3.4 to 5.1)	0.5 (0.4 to 0.6)	9.3 (7.6 to 11.5)	5 (4 to 6.3)	0.3 (0.2 to 0.4)	10.9 (8.7 to 13.7)	19.9 (9.2 to 32.7)	-40.7 (-51.5 to -27.2)	16.7 (6.5 to 29.5)
High income	High-income Asia Pacific	Singapore	Deaths	All Ages	1 (1 to 1)	0 (0 to 0)	1 (1 to 1)	1 (1 to 1)	0 (0 to 0)	1 (0 to 1)	-24.7 (-40.7 to -2.2)	23.4 (-18.4 to 243)	-28.5 (-45.1 to -6.3)
High income	High-income Asia Pacific	Singapore	Deaths	Age-standardized	0 (0 to 0)	0 (0 to 0)	0.1 (0.1 to 0.1)	0 (0 to 0)	0 (0 to 0)	0 (0 to 0)	-80.4 (-84.5 to -74.9)	-64.2 (-76.2 to -0.9)	-82.8 (-86.5 to -78)
High income	High-income Asia Pacific	Singapore	DALYs	All Ages	30 (25 to 36)	2 (2 to 3)	27 (22 to 33)	65 (46 to 90)	3 (2 to 4)	62 (44 to 87)	117.6 (67.1 to 173.1)	15.5 (-10.4 to 93.5)	127 (71.6 to 186.1)
High income	High-income Asia Pacific	Singapore	DALYs	Age-standardized	1.4 (1.2 to 1.7)	0.2 (0.1 to 0.2)	3 (2.4 to 3.7)	0.9 (0.6 to 1.2)	0.1 (0.1 to 0.1)	1.8 (1.3 to 2.6)	-39.1 (-53.3 to -23.9)	-59.9 (-68.6 to -32.4)	-39.9 (-54.1 to -24.7)
High income	High-income Asia Pacific	Singapore	YLLs	All Ages	18 (15 to 20)	2 (1 to 2)	16 (14 to 19)	12 (10 to 15)	1 (1 to 2)	11 (8 to 14)	-32.8 (-48 to -11.4)	-25.2 (-51 to 103.9)	-33.6 (-49.5 to -11.5)
High income	High-income Asia Pacific	Singapore	YLLs	Age-standardized	0.8 (0.7 to 0.9)	0.1 (0.1 to 0.1)	1.7 (1.4 to 1.9)	0.2 (0.1 to 0.2)	0 (0 to 0.1)	0.3 (0.2 to 0.3)	-81.4 (-85.7 to -75.6)	-72.7 (-81.8 to -23.9)	-83.3 (-87.3 to -78.2)
High income	High-income Asia Pacific	Singapore	YLDs	All Ages	12 (8 to 18)	1 (1 to 1)	11 (7 to 16)	53 (35 to 79)	2 (1 to 3)	51 (33 to 76)	336.7 (245.4 to 452.3)	80.8 (45.2 to 129.8)	358.8 (256.1 to 491)
High income	High-income Asia Pacific	Singapore	YLDs	Age-standardized	0.6 (0.4 to 0.9)	0.1 (0 to 0.1)	1.3 (0.8 to 2)	0.7 (0.5 to 1.1)	0 (0 to 0.1)	1.5 (1 to 2.3)	17.6 (-8.6 to 51.1)	-40.7 (-51.5 to -27.2)	14.8 (-12.1 to 49.2)
		Australasia	Incidence	All Ages	157 (126 to 190)	30 (21 to 41)	127 (102 to 156)	435 (332 to 564)	45 (31 to 59)	390 (296 to 512)	177.3 (143.4 to 261.1)	49 (20.5 to 85.2)	207.7 (168.6 to 316.2)
		Australasia	Incidence	Age-standardized	0.6 (0.5 to 0.8)	0.2 (0.2 to 0.3)	1.2 (1 to 1.4)	0.9 (0.7 to 1.1)	0.2 (0.1 to 0.3)	1.6 (1.2 to 2.1)	33.2 (17.7 to 71.1)	-19.5 (-36.7 to 3)	36.4 (20.4 to 81.8)
		Australasia	Prevalence	All Ages	666 (561 to 783)	54 (42 to 69)	611 (509 to 730)	1,659 (1,356 to 2,152)	85 (67 to 107)	1,573 (1,277 to 2,059)	149.1 (112.9 to 214.1)	56.8 (35.1 to 84.7)	157.3 (118 to 230.2)
		Australasia	Prevalence	Age-standardized	2.8 (2.3 to 3.2)	0.4 (0.3 to 0.6)	6.3 (5.3 to 7.4)	3.1 (2.6 to 4.1)	0.4 (0.3 to 0.5)	6.5 (5.3 to 8.4)	13.9 (-1.9 to 41.8)	-17.2 (-29.9 to -1.9)	3.2 (-11.4 to 30.7)
		Australasia	Deaths	All Ages	47 (42 to 52)	1 (1 to 1)	46 (41 to 51)	180 (147 to 216)	5 (3 to 7)	175 (143 to 212)	284.5 (206.3 to 373.8)	690.6 (411.9 to 1025.2)	278.5 (199.2 to 369.5)
		Australasia	Deaths	Age-standardized	0.2 (0.2 to 0.2)	0 (0 to 0)	0.5 (0.4 to 0.5)	0.3 (0.3 to 0.4)	0 (0 to 0)	0.7 (0.6 to 0.9)	66.2 (33.8 to 105.2)	261.4 (140.3 to 410.7)	47 (17.9 to 82.8)
		Australasia	DALYs	All Ages	929 (835 to 1,031)	20 (16 to 26)	909 (817 to 1,011)	2,708 (2,286 to 3,186)	90 (64 to 116)	2,618 (2,204 to 3,092)	191.5 (141.8 to 250.9)	353.9 (234.3 to 505.4)	188 (136.9 to 250.3)
		Australasia	DALYs	Age-standardized	3.8 (3.4 to 4.2)	0.2 (0.1 to 0.2)	8.8 (7.9 to 9.7)	5.1 (4.3 to 6)	0.3 (0.3 to 0.4)	10.8 (9.1 to 12.7)	33.6 (11.4 to 60.3)	123.7 (70.8 to 195.8)	22.3 (1.2 to 48.1)
		Australasia	YLLs	All Ages	828 (741 to 922)	11 (9 to 14)	817 (730 to 910)	2,455 (2,053 to 2,930)	76 (50 to 100)	2,378 (1,979 to 2,856)	196.5 (139 to 265)	589.2 (371.4 to 860.5)	191.2 (133.8 to 261.6)
		Australasia	YLLs	Age-standardized	3.4 (3.1 to 3.8)	0.1 (0.1 to 0.1)	7.9 (7.1 to 8.7)	4.6 (3.9 to 5.5)	0.3 (0.2 to 0.4)	9.8 (8.2 to 11.7)	35.8 (10.7 to 66.8)	245.6 (145.8 to 372.9)	24.4 (0.8 to 53.3)
		Australasia	YLDs	All Ages	101 (69 to 146)	9 (6 to 13)	93 (62 to 133)	254 (166 to 385)	14 (9 to 20)	240 (156 to 366)	150.5 (107 to 221.6)	56.8 (35.7 to 83.2)	159.4 (111.1 to 236.9)
		Australasia	YLDs	Age-standardized	0.4 (0.3 to 0.6)	0.1 (0.1 to 0.1)	0.9 (0.6 to 1.3)	0.5 (0.3 to 0.7)	0.1 (0.1 to 0.1)	1 (0.6 to 1.5)	15.1 (-3.8 to 46.1)	-17.5 (-29.8 to -2.6)	5.2 (-14.1 to 34.9)
High income	Australasia	Australia	Incidence	All Ages	108 (86 to 132)	24 (16 to 32)	84 (67 to 104)	334 (250 to 454)	40 (27 to 53)	294 (219 to 406)	210 (162.4 to 334)	68.1 (35.9 to 117.9)	250.3 (194.7 to 414.5)
High income	Australasia	Australia	Incidence	Age-standardized	0.5 (0.4 to 0.7)	0.2 (0.2 to 0.3)	0.9 (0.8 to 1.2)	0.8 (0.6 to 1)	0.2 (0.1 to 0.3)	1.4 (1.1 to 2)	47.1 (26.1 to 101.9)	-10.4 (-29.2 to 17)	52.1 (28.9 to 120.2)
High income	Australasia	Australia	Prevalence	All Ages	404 (334 to 485)	36 (27 to 47)	368 (302 to 448)	1,164 (926 to 1,596)	62 (46 to 80)	1,101 (866 to 1,528)	187.9 (137.4 to 290)	71.3 (41.5 to 109.2)	199.5 (143.2 to 313)
High income	Australasia	Australia	Prevalence	Age-standardized	2 (1.7 to 2.4)	0.4 (0.3 to 0.5)	4.6 (3.8 to 5.6)	2.6 (2.1 to 3.5)	0.3 (0.2 to 0.4)	5.3 (4.2 to 7.4)	28.2 (6.5 to 71.9)	-10 (-27 to 13.4)	14.9 (-5.3 to 57.1)
High income	Australasia	Australia	Deaths	All Ages	43 (38 to 49)	1 (0 to 1)	43 (38 to 48)	164 (133 to 199)	5 (3 to 7)	159 (129 to 195)	278.2 (198.7 to 376)	676.1 (397.1 to 1031.3)	272.4 (191.3 to 372.5)
High income	Australasia	Australia	Deaths	Age-standardized	0.2 (0.2 to 0.2)	0 (0 to 0)	0.5 (0.5 to 0.6)	0.3 (0.3 to 0.4)	0 (0 to 0)	0.8 (0.6 to 0.9)	60.4 (27.3 to 101)	249.8 (129.7 to 404.8)	42.1 (12.4 to 80.1)
High income	Australasia	Australia	DALYs	All Ages	831 (743 to 927)	16 (13 to 21)	815 (729 to 910)	2,396 (2,000 to 2,848)	81 (56 to 105)	2,315 (1,920 to 2,770)	188.2 (134.6 to 254.9)	395.7 (260.5 to 587.2)	184 (130.1 to 253.1)
High income	Australasia	Australia	DALYs	Age-standardized	4.1 (3.7 to 4.6)	0.2 (0.1 to 0.2)	9.5 (8.5 to 10.5)	5.3 (4.5 to 6.4)	0.4 (0.3 to 0.5)	11.3 (9.4 to 13.5)	30 (6.3 to 59)	142.9 (79.4 to 227.6)	19 (-3.2 to 46.8)
High income	Australasia	Australia	YLLs	All Ages	768 (683 to 862)	10 (8 to 13)	758 (674 to 852)	2,214 (1,824 to 2,668)	71 (46 to 95)	2,143 (1,754 to 2,592)	188.1 (129.2 to 261.2)	581.2 (360.9 to 873.7)	182.7 (123.7 to 257.6)
High income	Australasia	Australia	YLLs	Age-standardized	3.8 (3.4 to 4.2)	0.1 (0.1 to 0.1)	8.8 (7.9 to 9.8)	4.9 (4.1 to 5.9)	0.3 (0.2 to 0.4)	10.5 (8.6 to 12.6)	30 (4.1 to 62.2)	238 (136.9 to 374.5)	19.2 (-4.8 to 49.8)
High income	Australasia	Australia	YLDs	All Ages	63 (42 to 92)	6 (4 to 9)	57 (37 to 84)	182 (115 to 283)	10 (6 to 15)	172 (108 to 267)	188.5 (122.3 to 303.8)	70.8 (41.6 to 108.1)	200.8 (127.3 to 327.7)
High income	Australasia	Australia	YLDs	Age-standardized	0.3 (0.2 to 0.5)	0.1 (0 to 0.1)	0.7 (0.5 to 1)	0.4 (0.3 to 0.6)	0.1 (0 to 0.1)	0.8 (0.5 to 1.3)	29.3 (1.2 to 78.7)	-10.7 (-27 to 11.9)	16.9 (-11.4 to 63.8)
High income	Australasia	New Zealand	Incidence	All Ages	49 (39 to 60)	6 (4 to 10)	43 (34 to 52)	102 (80 to 124)	5 (3 to 7)	97 (76 to 119)	106 (81.2 to 133.1)	-22.3 (-54.9 to 34.6)	124.9 (100 to 153.7)
High income	Australasia	New Zealand	Incidence	Age-standardized	1.2 (1 to 1.5)	0.3 (0.2 to 0.5)	2.4 (1.9 to 2.9)	1.3 (1 to 1.6)	0.1 (0.1 to 0.2)	2.6 (2.1 to 3.2)	5.2 (-7.2 to 18.8)	-53.9 (-74.2 to -20.4)	8 (-2.9 to 20.8)
High income	Australasia	New Zealand	Prevalence	All Ages	262 (214 to 317)	18 (14 to 23)	244 (198 to 298)	495 (418 to 583)	23 (18 to 29)	472 (395 to 560)	89.2 (65.2 to 116.6)	27.6 (-1.6 to 60.5)	93.7 (67.1 to 124.2)
High income	Australasia	New Zealand	Prevalence	Age-standardized	6.5 (5.3 to 7.8)	0.9 (0.7 to 1.2)	14.4 (11.8 to 17.5)	6.2 (5.3 to 7.2)	0.6 (0.5 to 0.8)	12.7 (10.7 to 14.9)	-4.8 (-16.2 to 8.4)	-29.8 (-46 to -11.7)	-12 (-23.3 to 0.7)
High income	Australasia	New Zealand	Deaths	All Ages	3 (3 to 4)	0 (0 to 0)	3 (3 to 4)	16 (13 to 19)	0 (0 to 1)	16 (13 to 19)	362.3 (261.4 to 479.4)	910 (515.5 to 1416.6)	355.7 (255.1 to 474.8)
High income	Australasia	New Zealand	Deaths	Age-standardized	0.1 (0.1 to 0.1)	0 (0 to 0)	0.2 (0.2 to 0.2)	0.2 (0.2 to 0.2)	0 (0 to 0)	0.4 (0.3 to 0.5)	122.9 (74.3 to 176.8)	391 (207.1 to 627.3)	93.5 (52 to 142)
High income	Australasia	New Zealand	DALYs	All Ages	98 (83 to 116)	4 (2 to 5)	94 (80 to 112)	313 (261 to 368)	9 (7 to 12)	303 (252 to 359)	220.1 (166.1 to 289)	160.3 (87.3 to 262.7)	222.3 (165.9 to 294)
High income	Australasia	New Zealand	DALYs	Age-standardized	2.4 (2 to 2.8)	0.2 (0.1 to 0.2)	5.4 (4.6 to 6.4)	3.8 (3.2 to 4.5)	0.2 (0.2 to 0.3)	8 (6.6 to 9.4)	59.6 (33.3 to 93.8)	34 (-4.2 to 86.9)	46.2 (22 to 78)
High income	Australasia	New Zealand	YLLs	All Ages	59 (51 to 68)	1 (1 to 1)	59 (51 to 67)	241 (196 to 291)	6 (4 to 8)	235 (191 to 286)	305.5 (218.8 to 407.2)	709.7 (440.9 to 1072.2)	300.8 (213.7 to 401.7)
High income	Australasia	New Zealand	YLLs	Age-standardized	1.4 (1.2 to 1.6)	0 (0 to 0)	3.4 (2.9 to 3.8)	2.9 (2.4 to 3.5)	0.1 (0.1 to 0.2)	6.1 (5 to 7.4)	102.5 (59.8 to 152.5)	324.1 (189.2 to 508.3)	81.9 (43.9 to 126.7)
High income	Australasia	New Zealand	YLDs	All Ages	38 (25 to 56)	3 (2 to 4)	36 (23 to 52)	72 (46 to 104)	4 (2 to 5)	68 (44 to 100)	88 (46.4 to 140.6)	27.7 (-1.4 to 60.2)	92.8 (48 to 150)
High income	Australasia	New Zealand	YLDs	Age-standardized	1 (0.6 to 1.4)	0.1 (0.1 to 0.2)	2.1 (1.4 to 3.1)	0.9 (0.6 to 1.3)	0.1 (0.1 to 0.1)	1.8 (1.2 to 2.7)	-5 (-24.8 to 20.2)	-29.9 (-46 to -11.8)	-11.4 (-31.2 to 13.1)
		Western Europe	Incidence	All Ages	6,223 (5,165 to 7,500)	524 (420 to 642)	5,699 (4,680 to 6,955)	4,662 (4,012 to 5,388)	475 (380 to 572)	4,187 (3,599 to 4,862)	-25.1 (-36.3 to -10.1)	-9.5 (-19.5 to 1.7)	-26.5 (-38.4 to -10.1)
		Western Europe	Incidence	Age-standardized	1.1 (0.9 to 1.4)	0.2 (0.1 to 0.2)	2.3 (1.9 to 2.8)	0.5 (0.5 to 0.6)	0.1 (0.1 to 0.1)	1 (0.9 to 1.2)	-53 (-60.3 to -44)	-36.4 (-43.5 to -28.4)	-55.5 (-62.4 to -46.4)
		Western Europe	Prevalence	All Ages	69,186 (55,030 to 89,801)	3,765 (3,112 to 4,663)	65,421 (51,511 to 85,410)	38,197 (33,391 to 44,339)	3,442 (2,913 to 4,060)	34,755 (30,414 to 40,303)	-44.8 (-54.8 to -34.3)	-8.6 (-17.5 to 0.9)	-46.9 (-57 to -35.9)
		Western Europe	Prevalence	Age-standardized	11.6 (9.2 to 15.1)	1.1 (0.9 to 1.4)	27 (21.4 to 34.9)	4.1 (3.6 to 4.7)	0.7 (0.6 to 0.8)	8.3 (7.3 to 9.6)	-64.8 (-71.7 to -58.1)	-37.9 (-43.6 to -32.4)	-69.2 (-75 to -63.1)
		Western Europe	Deaths	All Ages	4,248 (4,025 to 4,452)	119 (103 to 150)	4,129 (3,909 to 4,329)	2,891 (2,551 to 3,210)	186 (135 to 233)	2,705 (2,394 to 3,016)	-31.9 (-39.3 to -23.7)	56.2 (19.1 to 92.9)	-34.5 (-41.7 to -26.5)
		Western Europe	Deaths	Age-standardized	0.7 (0.7 to 0.7)	0 (0 to 0)	1.8 (1.7 to 1.8)	0.3 (0.2 to 0.3)	0 (0 to 0)	0.6 (0.5 to 0.7)	-62.4 (-66.2 to -58)	-8.5 (-27.4 to 11.8)	-65.8 (-69.5 to -61.7)
		Western Europe	DALYs	All Ages	88,084 (82,704 to 93,344)	2,578 (2,269 to 3,080)	85,506 (80,387 to 90,721)	43,522 (39,213 to 47,790)	2,981 (2,411 to 3,647)	40,541 (36,522 to 44,545)	-50.6 (-55.2 to -45.3)	15.6 (-4.3 to 36.8)	-52.6 (-57 to -47.1)
		Western Europe	DALYs	Age-standardized	14.9 (14 to 15.8)	0.8 (0.7 to 0.9)	35 (32.9 to 37.1)	4.4 (4 to 4.8)	0.6 (0.5 to 0.7)	9.4 (8.4 to 10.3)	-70.4 (-73 to -67.3)	-23.1 (-33.9 to -9.8)	-73.3 (-75.6 to -70.3)
		Western Europe	YLLs	All Ages	78,514 (74,564 to 82,302)	2,009 (1,748 to 2,493)	76,504 (72,682 to 80,249)	38,007 (34,034 to 41,919)	2,449 (1,906 to 3,106)	35,558 (31,902 to 39,337)	-51.6 (-56.6 to -46)	21.9 (-3.8 to 49)	-53.5 (-58.4 to -47.8)
		Western Europe	YLLs	Age-standardized	13.2 (12.6 to 13.9)	0.6 (0.5 to 0.7)	31.3 (29.8 to 32.8)	3.8 (3.4 to 4.2)	0.5 (0.4 to 0.6)	8.2 (7.3 to 9)	-71.3 (-74.1 to -68.1)	-19.3 (-33 to -2)	-74 (-76.6 to -70.8)
		Western Europe	YLDs	All Ages	9,570 (6,134 to 13,907)	568 (366 to 841)	9,002 (5,738 to 13,123)	5,515 (3,776 to 7,752)	531 (346 to 772)	4,983 (3,418 to 6,996)	-42.4 (-53.1 to -31.3)	-6.5 (-16.1 to 3.7)	-44.6 (-55.4 to -33.2)
		Western Europe	YLDs	Age-standardized	1.6 (1 to 2.3)	0.2 (0.1 to 0.3)	3.7 (2.4 to 5.4)	0.6 (0.4 to 0.8)	0.1 (0.1 to 0.2)	1.2 (0.8 to 1.7)	-63 (-70.2 to -55.9)	-36.5 (-42.6 to -30.4)	-67.6 (-73.7 to -61.2)
High income	Western Europe	Andorra	Incidence	All Ages	0 (0 to 0)	0 (0 to 0)	0 (0 to 0)	0 (0 to 0)	0 (0 to 0)	0 (0 to 0)	102.4 (57.3 to 158.9)	76.5 (23.3 to 172.3)	108.9 (58 to 172.4)
High income	Western Europe	Andorra	Incidence	Age-standardized	0.1 (0 to 0.1)	0 (0 to 0)	0.1 (0.1 to 0.2)	0.1 (0 to 0.1)	0 (0 to 0)	0.1 (0.1 to 0.1)	-8.6 (-27.4 to 14.6)	-1.8 (-33.8 to 63)	-9.7 (-30.4 to 13.2)
High income	Western Europe	Andorra	Prevalence	All Ages	0 (0 to 0)	0 (0 to 0)	0 (0 to 0)	0 (0 to 0)	0 (0 to 0)	0 (0 to 0)	102.6 (70.5 to 143.5)	90.7 (45.2 to 155.5)	105.2 (70.5 to 151.2)
High income	Western Europe	Andorra	Prevalence	Age-standardized	0.2 (0.1 to 0.2)	0.1 (0 to 0.1)	0.3 (0.2 to 0.3)	0.1 (0.1 to 0.2)	0 (0 to 0.1)	0.2 (0.2 to 0.3)	-19.4 (-30.8 to -4.8)	-3.6 (-26.9 to 33)	-22.3 (-34.6 to -7.1)
High income	Western Europe	Andorra	Deaths	All Ages	0 (0 to 0)	0 (0 to 0)	0 (0 to 0)	0 (0 to 1)	0 (0 to 0)	0 (0 to 1)	86.8 (9.9 to 210.1)	119.9 (11.4 to 342.1)	86.4 (8.5 to 210.9)
High income	Western Europe	Andorra	Deaths	Age-standardized	0.4 (0.2 to 0.7)	0 (0 to 0)	0.9 (0.4 to 1.5)	0.2 (0.1 to 0.4)	0 (0 to 0)	0.5 (0.2 to 0.9)	-40.7 (-65.2 to -1.1)	-38.9 (-68.7 to 24.2)	-39.3 (-64.4 to 1.3)
High income	Western Europe	Andorra	DALYs	All Ages	4 (2 to 6)	0 (0 to 0)	3 (2 to 6)	5 (2 to 8)	0 (0 to 0)	5 (2 to 8)	45.2 (-13.9 to 137.6)	60 (-11.8 to 200.3)	45 (-14.6 to 139.1)
High income	Western Europe	Andorra	DALYs	Age-standardized	6.6 (3 to 11.3)	0.2 (0.1 to 0.3)	13.3 (6.1 to 22.6)	3.6 (1.7 to 5.9)	0.1 (0.1 to 0.2)	7.4 (3.4 to 12)	-45.4 (-67.9 to -10.7)	-35.6 (-64.5 to 16.8)	-44.4 (-66.9 to -8.9)
High income	Western Europe	Andorra	YLLs	All Ages	4 (2 to 6)	0 (0 to 0)	3 (2 to 6)	5 (2 to 8)	0 (0 to 0)	5 (2 to 8)	44.9 (-14.3 to 137.8)	58.4 (-15.3 to 207)	44.8 (-14.8 to 139.3)
High income	Western Europe	Andorra	YLLs	Age-standardized	6.6 (3 to 11.3)	0.2 (0.1 to 0.3)	13.3 (6 to 22.6)	3.6 (1.7 to 5.9)	0.1 (0.1 to 0.2)	7.4 (3.4 to 12)	-45.5 (-68.1 to -10.6)	-37.1 (-66.7 to 17.9)	-44.4 (-67 to -8.8)
High income	Western Europe	Andorra	YLDs	All Ages	0 (0 to 0)	0 (0 to 0)	0 (0 to 0)	0 (0 to 0)	0 (0 to 0)	0 (0 to 0)	103.7 (72.1 to 144)	91.8 (46.8 to 154.8)	106.1 (71.4 to 151)
High income	Western Europe	Andorra	YLDs	Age-standardized	0 (0 to 0)	0 (0 to 0)	0 (0 to 0.1)	0 (0 to 0)	0 (0 to 0)	0 (0 to 0.1)	-19.8 (-31.2 to -5.4)	-4 (-26.6 to 31.2)	-22.5 (-34.8 to -7.8)
High income	Western Europe	Austria	Incidence	All Ages	8 (6 to 10)	2 (2 to 3)	5 (4 to 7)	17 (13 to 21)	5 (3 to 7)	12 (9 to 15)	119.6 (83.6 to 173.6)	99 (47.6 to 183.3)	129.2 (86.3 to 193.1)
High income	Western Europe	Austria	Incidence	Age-standardized	0.1 (0.1 to 0.1)	0 (0 to 0.1)	0.1 (0.1 to 0.1)	0.1 (0.1 to 0.1)	0.1 (0 to 0.1)	0.2 (0.1 to 0.2)	47.7 (24.2 to 83.1)	46.6 (12.1 to 108.9)	33.5 (10.5 to 68.1)
High income	Western Europe	Austria	Prevalence	All Ages	66 (53 to 81)	20 (15 to 25)	46 (36 to 58)	140 (110 to 178)	37 (28 to 47)	104 (78 to 134)	113.7 (77.9 to 165.3)	88.2 (56.8 to 132.6)	124.5 (78 to 191.4)
High income	Western Europe	Austria	Prevalence	Age-standardized	0.6 (0.4 to 0.7)	0.3 (0.2 to 0.4)	1.1 (0.8 to 1.3)	0.8 (0.6 to 1)	0.4 (0.3 to 0.5)	1.3 (1 to 1.7)	40.6 (18.5 to 73.5)	38.7 (18.1 to 71.2)	21.9 (-2.2 to 56.5)
High income	Western Europe	Austria	Deaths	All Ages	21 (18 to 23)	1 (0 to 1)	20 (18 to 23)	20 (17 to 24)	0 (0 to 1)	20 (16 to 24)	-1.9 (-20.6 to 22.4)	-7.7 (-41 to 35.1)	-1.7 (-21 to 23.1)
High income	Western Europe	Austria	Deaths	Age-standardized	0.2 (0.1 to 0.2)	0 (0 to 0)	0.5 (0.4 to 0.5)	0.1 (0.1 to 0.1)	0 (0 to 0)	0.2 (0.2 to 0.3)	-38 (-49.6 to -23.2)	-36.3 (-59.1 to -8.7)	-47.3 (-57.5 to -34.4)
High income	Western Europe	Austria	DALYs	All Ages	403 (355 to 453)	11 (8 to 14)	393 (344 to 442)	343 (290 to 405)	12 (9 to 16)	331 (278 to 393)	-14.9 (-30.5 to 5.2)	12.1 (-18.6 to 44.8)	-15.7 (-31.6 to 4.8)
High income	Western Europe	Austria	DALYs	Age-standardized	3.4 (3 to 3.8)	0.1 (0.1 to 0.2)	8.5 (7.5 to 9.6)	1.9 (1.6 to 2.2)	0.1 (0.1 to 0.2)	4.1 (3.5 to 4.9)	-44.2 (-54.8 to -31.4)	-11.7 (-35 to 10.8)	-52 (-61.1 to -40.8)

Pneumoconiosis

7 GBD super region	21 GBD region	Location	Measure	Age	1990			2019			Percent change (1990 to 2019)		
					Both	Female	Male	Both	Female	Male	Both	Female	Male
High income	Western Europe	Austria	YLLs	All Ages	393 (344 to 443)	8 (6 to 10)	385 (336 to 434)	320 (268 to 381)	6 (4 to 8)	314 (262 to 375)	-18.4 (-34.2 to 1.7)	-18.8 (-48.5 to 18)	-18.4 (-34.3 to 2)
High income	Western Europe	Austria	YLLs	Age-standardized	3.3 (2.9 to 3.7)	0.1 (0.1 to 0.1)	8.4 (7.4 to 9.4)	1.8 (1.5 to 2.1)	0.1 (0 to 0.1)	3.9 (3.3 to 4.6)	-46.4 (-56.8 to -33.2)	-37 (-60.2 to -12.1)	-53.5 (-62.5 to -42.2)
High income	Western Europe	Austria	YLDs	All Ages	11 (7 to 16)	3 (2 to 5)	8 (5 to 11)	22 (14 to 33)	6 (4 to 9)	17 (11 to 25)	111.4 (76.6 to 162)	87.2 (56.2 to 131.3)	121.3 (76.1 to 186.2)
High income	Western Europe	Austria	YLDs	Age-standardized	0.1 (0.1 to 0.1)	0 (0 to 0.1)	0.2 (0.1 to 0.3)	0.1 (0.1 to 0.2)	0.1 (0 to 0.1)	0.2 (0.1 to 0.3)	39 (17.3 to 71.7)	38 (17.6 to 69.8)	20.1 (-3.4 to 54.1)
High income	Western Europe	Belgium	Incidence	All Ages	46 (39 to 54)	2 (1 to 3)	44 (37 to 51)	25 (20 to 31)	3 (1 to 4)	23 (19 to 28)	-44.7 (-55.7 to -32)	27.6 (-6.4 to 66.3)	-48.2 (-59 to -35.8)
High income	Western Europe	Belgium	Incidence	Age-standardized	0.3 (0.3 to 0.4)	0 (0 to 0.1)	0.7 (0.6 to 0.8)	0.1 (0.1 to 0.1)	0 (0 to 0.1)	0.2 (0.2 to 0.3)	-63.5 (-71.5 to -55.4)	1.5 (-28.3 to 34.2)	-67.1 (-73.7 to -59.8)
High income	Western Europe	Belgium	Prevalence	All Ages	355 (300 to 416)	5 (3 to 7)	350 (295 to 411)	110 (90 to 134)	6 (4 to 8)	104 (85 to 127)	-69.1 (-75.7 to -60.6)	19.7 (-1.1 to 40.5)	-70.4 (-77 to -62.1)
High income	Western Europe	Belgium	Prevalence	Age-standardized	2.2 (1.9 to 2.6)	0.1 (0 to 0.1)	5.3 (4.5 to 6.2)	0.4 (0.4 to 0.5)	0.1 (0 to 0.1)	0.9 (0.8 to 1.2)	-80.9 (-84.9 to -75.8)	-5.5 (-24.6 to 9.9)	-82.3 (-86.1 to -77.5)
High income	Western Europe	Belgium	Deaths	All Ages	487 (437 to 539)	5 (3 to 6)	482 (433 to 535)	96 (78 to 116)	3 (2 to 5)	93 (75 to 113)	-80.2 (-84.2 to -74.9)	-25.1 (-51.1 to 9.5)	-80.8 (-84.7 to -75.5)
High income	Western Europe	Belgium	Deaths	Age-standardized	3 (2.7 to 3.4)	0 (0 to 0.1)	7.6 (6.8 to 8.4)	0.4 (0.3 to 0.4)	0 (0 to 0)	0.8 (0.7 to 1)	-88.4 (-90.7 to -85.5)	-52.2 (-68.8 to -32.3)	-89 (-91.3 to -86.1)
High income	Western Europe	Belgium	DALYs	All Ages	9,411 (8,400 to 10,474)	68 (52 to 89)	9,343 (8,333 to 10,407)	1,285 (1,064 to 1,537)	45 (31 to 59)	1,240 (1,026 to 1,492)	-86.3 (-89 to -83.1)	-34.1 (-56 to -7)	-86.7 (-89.4 to -83.5)
High income	Western Europe	Belgium	DALYs	Age-standardized	59.6 (53.2 to 66.6)	0.7 (0.5 to 0.9)	140.7 (126.1 to 156.7)	5.1 (4.3 to 6.1)	0.3 (0.2 to 0.4)	11.5 (9.5 to 13.8)	-91.4 (-93 to -89.4)	-52.4 (-67.7 to -33.9)	-91.8 (-93.4 to -89.9)
High income	Western Europe	Belgium	YLLs	All Ages	9,350 (8,345 to 10,417)	67 (51 to 88)	9,283 (8,279 to 10,352)	1,266 (1,044 to 1,517)	44 (30 to 58)	1,222 (1,007 to 1,474)	-86.5 (-89.2 to -83.2)	-34.8 (-57 to -7.2)	-86.8 (-89.5 to -83.6)
High income	Western Europe	Belgium	YLLs	Age-standardized	59.2 (52.8 to 66.2)	0.7 (0.5 to 0.9)	139.8 (125.3 to 155.6)	5 (4.2 to 6)	0.3 (0.2 to 0.4)	11.3 (9.3 to 13.6)	-91.5 (-93.1 to -89.5)	-53.2 (-68.4 to -34.4)	-91.9 (-93.5 to -90)
High income	Western Europe	Belgium	YLDs	All Ages	61 (41 to 85)	1 (1 to 1)	60 (40 to 84)	19 (12 to 27)	1 (1 to 2)	18 (11 to 26)	-68.9 (-75.7 to -60.6)	19.8 (-1.6 to 40.4)	-70.2 (-76.8 to -62)
High income	Western Europe	Belgium	YLDs	Age-standardized	0.4 (0.3 to 0.5)	0 (0 to 0)	0.9 (0.6 to 1.3)	0.1 (0 to 0.1)	0 (0 to 0)	0.2 (0.1 to 0.2)	-80.9 (-84.8 to -75.8)	-5.9 (-24.8 to 9.6)	-82.2 (-86.1 to -77.4)
High income	Western Europe	Cyprus	Incidence	All Ages	0 (0 to 1)	0 (0 to 0)	0 (0 to 0)	1 (0 to 1)	0 (0 to 0)	0 (0 to 1)	82.4 (53.7 to 133.5)	97.1 (47.5 to 221.4)	75.3 (39.4 to 125.7)
High income	Western Europe	Cyprus	Incidence	Age-standardized	0 (0 to 0.1)	0 (0 to 0)	0.1 (0 to 0.1)	0 (0 to 0.1)	0 (0 to 0)	0.1 (0 to 0.1)	-19 (-34.3 to -4.1)	-2.9 (-27.4 to 47.4)	-18.4 (-36.2 to -0.1)
High income	Western Europe	Cyprus	Prevalence	All Ages	1 (1 to 1)	0 (0 to 0)	1 (0 to 1)	1 (1 to 2)	0 (0 to 1)	1 (1 to 1)	81.8 (56.1 to 114.1)	100.1 (66.2 to 168.3)	75.5 (45.7 to 115.3)
High income	Western Europe	Cyprus	Prevalence	Age-standardized	0.1 (0.1 to 0.2)	0.1 (0 to 0.1)	0.2 (0.2 to 0.2)	0.1 (0.1 to 0.1)	0 (0 to 0.1)	0.2 (0.1 to 0.2)	-26.2 (-37.3 to -15)	-6.2 (-22.9 to 21.2)	-18.2 (-32.5 to -1)
High income	Western Europe	Cyprus	Deaths	All Ages	2 (1 to 3)	0 (0 to 0)	2 (1 to 2)	1 (1 to 2)	0 (0 to 0)	1 (1 to 1)	-29.2 (-53.9 to 6.9)	74.6 (2.5 to 207.3)	-36.7 (-61.1 to -0.6)
High income	Western Europe	Cyprus	Deaths	Age-standardized	0.4 (0.3 to 0.5)	0 (0 to 0.1)	0.7 (0.5 to 1)	0.1 (0.1 to 0.1)	0 (0 to 0)	0.2 (0.1 to 0.2)	-79.7 (-86.8 to -70.7)	-35.3 (-60.5 to 11.2)	-78 (-86.4 to -67.6)
High income	Western Europe	Cyprus	DALYs	All Ages	34 (24 to 45)	3 (2 to 4)	31 (22 to 41)	20 (15 to 26)	4 (3 to 6)	16 (12 to 20)	-40.9 (-59.8 to -14.8)	34 (-16.7 to 127)	-48.2 (-66.2 to -22.9)
High income	Western Europe	Cyprus	DALYs	Age-standardized	5.4 (3.9 to 7.1)	0.8 (0.5 to 1.2)	10.2 (7.2 to 13.6)	1.2 (1 to 1.5)	0.5 (0.4 to 0.8)	2.2 (1.6 to 2.8)	-77.5 (-84.7 to -68.5)	-33.8 (-60.1 to 17.8)	-78.9 (-86.2 to -69.8)
High income	Western Europe	Cyprus	YLLs	All Ages	34 (24 to 44)	3 (2 to 4)	31 (21 to 41)	20 (15 to 25)	4 (3 to 6)	16 (12 to 20)	-41.4 (-60.2 to -15.3)	33.3 (-17.4 to 127)	-48.6 (-66.6 to -23.2)
High income	Western Europe	Cyprus	YLLs	Age-standardized	5.4 (3.9 to 7.1)	0.8 (0.5 to 1.2)	10.2 (7.2 to 13.6)	1.2 (0.9 to 1.5)	0.5 (0.4 to 0.8)	2.1 (1.6 to 2.7)	-77.7 (-84.9 to -68.7)	-34 (-60.5 to 18.1)	-79.1 (-86.4 to -70)
High income	Western Europe	Cyprus	YLDs	All Ages	0 (0 to 0)	0 (0 to 0)	0 (0 to 0)	0 (0 to 0)	0 (0 to 0)	0 (0 to 0)	81.6 (56 to 113.3)	100 (66.6 to 164.9)	75.5 (45.3 to 114.3)
High income	Western Europe	Cyprus	YLDs	Age-standardized	0 (0 to 0)	0 (0 to 0)	0 (0 to 0)	0 (0 to 0)	0 (0 to 0)	0 (0 to 0)	-27 (-37.8 to -15.7)	-6.7 (-23 to 19.2)	-18.7 (-32.6 to -1.8)
High income	Western Europe	Denmark	Incidence	All Ages	7 (5 to 9)	1 (0 to 2)	6 (4 to 8)	6 (4 to 9)	1 (1 to 2)	5 (4 to 8)	-4.3 (-26.5 to 18.7)	17.3 (-18.8 to 55.5)	-7.7 (-31.7 to 21.1)
High income	Western Europe	Denmark	Incidence	Age-standardized	0.1 (0.1 to 0.1)	0 (0 to 0)	0.2 (0.1 to 0.2)	0.1 (0 to 0.1)	0 (0 to 0)	0.1 (0.1 to 0.1)	-28.6 (-46.1 to -12.7)	-0.2 (-26.5 to 43.1)	-37.7 (-53.6 to -19.9)
High income	Western Europe	Denmark	Prevalence	All Ages	15 (12 to 18)	2 (1 to 3)	13 (10 to 16)	11 (8 to 14)	2 (2 to 3)	8 (6 to 11)	-28.1 (-42.2 to -11.9)	26.3 (3.6 to 53.5)	-36.4 (-50.7 to -19.3)
High income	Western Europe	Denmark	Prevalence	Age-standardized	0.2 (0.1 to 0.2)	0.1 (0 to 0.1)	0.4 (0.3 to 0.4)	0.1 (0.1 to 0.1)	0.1 (0 to 0.1)	0.2 (0.1 to 0.2)	-43.1 (-54.8 to -31.5)	2.9 (-15.7 to 32.9)	-55.5 (-65.6 to -44.7)
High income	Western Europe	Denmark	Deaths	All Ages	23 (19 to 26)	1 (1 to 1)	22 (18 to 26)	15 (12 to 18)	2 (1 to 2)	13 (11 to 16)	-33.9 (-48.4 to -14.8)	102.3 (-4.4 to 230.3)	-38.6 (-53.2 to -19.5)
High income	Western Europe	Denmark	Deaths	Age-standardized	0.3 (0.2 to 0.3)	0 (0 to 0)	0.6 (0.5 to 0.7)	0.1 (0.1 to 0.1)	0 (0 to 0)	0.2 (0.2 to 0.3)	-54.4 (-64.4 to -41.5)	50 (-26.2 to 138.8)	-61.1 (-70.5 to -48.9)
High income	Western Europe	Denmark	DALYs	All Ages	393 (335 to 462)	12 (9 to 16)	381 (323 to 452)	226 (185 to 274)	22 (11 to 30)	204 (164 to 251)	-42.6 (-55.5 to -26.5)	85.5 (-2.2 to 187.3)	-46.6 (-59.9 to -29.9)
High income	Western Europe	Denmark	DALYs	Age-standardized	4.7 (4 to 5.5)	0.2 (0.2 to 0.3)	10.7 (9.1 to 12.6)	1.9 (1.5 to 2.3)	0.3 (0.2 to 0.5)	3.7 (3 to 4.5)	-60.5 (-69.3 to -49.6)	40.4 (-20.7 to 113.3)	-65.8 (-74 to -55.4)
High income	Western Europe	Denmark	YLLs	All Ages	391 (332 to 460)	12 (9 to 16)	379 (321 to 450)	224 (183 to 272)	22 (11 to 30)	202 (162 to 249)	-42.7 (-55.6 to -26.5)	87.1 (-2.9 to 192.9)	-46.6 (-60 to -29.9)
High income	Western Europe	Denmark	YLLs	Age-standardized	4.7 (4 to 5.5)	0.2 (0.2 to 0.3)	10.7 (9.1 to 12.5)	1.8 (1.5 to 2.2)	0.3 (0.2 to 0.5)	3.6 (2.9 to 4.5)	-60.6 (-69.4 to -49.7)	41.8 (-21.5 to 117.8)	-65.9 (-74.1 to -55.4)
High income	Western Europe	Denmark	YLDs	All Ages	2 (2 to 4)	0 (0 to 1)	2 (1 to 3)	2 (1 to 3)	0 (0 to 1)	1 (1 to 2)	-28.3 (-42.3 to -12.3)	26.2 (4 to 52.1)	-36.3 (-50.6 to -19.3)
High income	Western Europe	Denmark	YLDs	Age-standardized	0 (0 to 0)	0 (0 to 0)	0.1 (0 to 0.1)	0 (0 to 0)	0 (0 to 0)	0 (0 to 0)	-43.7 (-55.1 to -32.3)	2.3 (-15.9 to 31.3)	-55.7 (-65.6 to -45.1)
High income	Western Europe	Finland	Incidence	All Ages	6 (5 to 8)	1 (1 to 2)	5 (4 to 7)	10 (7 to 13)	2 (1 to 2)	8 (6 to 11)	52.7 (25.4 to 84.7)	23.7 (-0.5 to 57)	60.2 (27.1 to 98)
High income	Western Europe	Finland	Incidence	Age-standardized	0.1 (0.1 to 0.1)	0 (0 to 0.1)	0.2 (0.1 to 0.2)	0.1 (0.1 to 0.1)	0 (0 to 0.1)	0.1 (0.1 to 0.2)	-7 (-21.4 to 9)	-3.7 (-23.8 to 18.5)	-18.8 (-33.1 to -1.2)
High income	Western Europe	Finland	Prevalence	All Ages	20 (16 to 24)	7 (5 to 10)	12 (10 to 15)	30 (24 to 37)	11 (8 to 14)	19 (16 to 23)	51.4 (33 to 71.4)	47.1 (29 to 65.9)	54 (30.3 to 81.8)
High income	Western Europe	Finland	Prevalence	Age-standardized	0.3 (0.2 to 0.3)	0.2 (0.1 to 0.2)	0.5 (0.4 to 0.6)	0.3 (0.2 to 0.3)	0.2 (0.1 to 0.2)	0.4 (0.3 to 0.4)	-10.6 (-20.1 to -1.3)	-1.5 (-12.5 to 9.5)	-25.3 (-36.2 to -13.9)
High income	Western Europe	Finland	Deaths	All Ages	20 (16 to 24)	1 (0 to 1)	19 (16 to 23)	31 (25 to 39)	0 (0 to 0)	31 (25 to 39)	57.2 (15.7 to 110.9)	-51.5 (-74.8 to -16.9)	60.9 (17.6 to 118.2)
High income	Western Europe	Finland	Deaths	Age-standardized	0.3 (0.2 to 0.3)	0 (0 to 0)	0.8 (0.6 to 0.9)	0.2 (0.2 to 0.3)	0 (0 to 0)	0.5 (0.4 to 0.7)	-16.5 (-38.6 to 11.6)	-69.1 (-84.2 to -47.1)	-29.8 (-48.4 to -5.2)
High income	Western Europe	Finland	DALYs	All Ages	357 (292 to 431)	11 (7 to 17)	346 (280 to 421)	456 (367 to 569)	6 (4 to 8)	450 (361 to 562)	27.6 (-6.4 to 71.9)	-44.9 (-67.9 to -11.8)	30 (-5.1 to 75.5)
High income	Western Europe	Finland	DALYs	Age-standardized	4.8 (3.9 to 5.8)	0.2 (0.2 to 0.3)	12.7 (10.4 to 15.3)	3.4 (2.7 to 4.2)	0.1 (0.1 to 0.1)	7.7 (6.2 to 9.5)	-30 (-48.7 to -6)	-61.3 (-76.7 to -39.7)	-39.6 (-55.9 to -18.7)
High income	Western Europe	Finland	YLLs	All Ages	354 (288 to 428)	10 (6 to 15)	344 (278 to 419)	451 (362 to 563)	4 (3 to 6)	446 (358 to 559)	27.4 (-6.8 to 72.1)	-55.6 (-77.3 to -23.6)	29.8 (-5.4 to 75.9)
High income	Western Europe	Finland	YLLs	Age-standardized	4.8 (3.9 to 5.8)	0.2 (0.1 to 0.3)	12.6 (10.3 to 15.2)	3.3 (2.7 to 4.1)	0.1 (0 to 0.1)	7.6 (6.1 to 9.5)	-30.2 (-49.1 to -6)	-69.9 (-84.4 to -48.8)	-39.7 (-56 to -18.6)
High income	Western Europe	Finland	YLDs	All Ages	3 (2 to 5)	1 (1 to 2)	2 (1 to 3)	5 (3 to 7)	2 (1 to 3)	3 (2 to 5)	50.9 (32.7 to 70.8)	46.7 (28.6 to 65.4)	53.2 (29.4 to 80.6)
High income	Western Europe	Finland	YLDs	Age-standardized	0 (0 to 0.1)	0 (0 to 0)	0.1 (0.1 to 0.1)	0 (0 to 0.1)	0 (0 to 0)	0.1 (0 to 0.1)	-11.2 (-20.7 to -1.7)	-1.7 (-12.7 to 9)	-26 (-36.8 to -14.6)
High income	Western Europe	France	Incidence	All Ages	115 (95 to 136)	13 (7 to 20)	102 (85 to 120)	157 (122 to 205)	32 (21 to 49)	125 (96 to 159)	36.4 (9.6 to 73)	150.1 (68.1 to 314.2)	22.4 (-3.4 to 56.5)
High income	Western Europe	France	Incidence	Age-standardized	0.1 (0.1 to 0.2)	0 (0 to 0.1)	0.3 (0.2 to 0.4)	0.1 (0.1 to 0.1)	0 (0 to 0.1)	0.2 (0.2 to 0.3)	-20.5 (-35.6 to -1.5)	35.1 (-10.5 to 107.3)	-30.3 (-44.4 to -12.8)
High income	Western Europe	France	Prevalence	All Ages	797 (666 to 959)	32 (22 to 50)	765 (638 to 926)	563 (455 to 693)	59 (44 to 85)	504 (402 to 625)	-29.3 (-45.1 to -8.5)	85.3 (40.2 to 159)	-34.1 (-49.4 to -12.7)
High income	Western Europe	France	Prevalence	Age-standardized	0.9 (0.8 to 1.1)	0.1 (0 to 0.1)	2.2 (1.8 to 2.7)	0.4 (0.3 to 0.4)	0.1 (0.1 to 0.1)	0.8 (0.6 to 1)	-60.8 (-69.3 to -49.8)	9 (-18 to 41.1)	-65.1 (-73 to -54.2)
High income	Western Europe	France	Deaths	All Ages	815 (735 to 901)	25 (19 to 31)	790 (712 to 875)	657 (530 to 816)	75 (49 to 113)	582 (467 to 737)	-19.4 (-36.3 to 2.1)	204.7 (102.1 to 362.9)	-26.4 (-42.2 to -4)
High income	Western Europe	France	Deaths	Age-standardized	0.9 (0.9 to 1.1)	0 (0 to 0.1)	2.4 (2.1 to 2.6)	0.4 (0.3 to 0.5)	0.1 (0 to 0.1)	0.9 (0.7 to 1.1)	-58.9 (-67 to -48.6)	68.8 (17.7 to 145)	-63 (-70.8 to -52.1)
High income	Western Europe	France	DALYs	All Ages	15,287 (13,849 to 16,904)	367 (297 to 457)	14,919 (13,479 to 16,492)	8,534 (6,983 to 10,360)	930 (641 to 1,354)	7,604 (6,234 to 9,483)	-44.2 (-54.9 to -30.8)	153.1 (78.5 to 260.5)	-49 (-59.2 to -35.3)
High income	Western Europe	France	DALYs	Age-standardized	18.5 (16.7 to 20.5)	0.7 (0.6 to 0.9)	43.2 (38.8 to 47.8)	5.7 (4.7 to 6.9)	1.1 (0.8 to 1.7)	11.9 (9.8 to 14.8)	-69.3 (-75.1 to -61.8)	57.2 (14.3 to 117.2)	-72.4 (-77.8 to -65.2)
High income	Western Europe	France	YLLs	All Ages	15,149 (13,683 to 16,746)	362 (292 to 453)	14,787 (13,327 to 16,367)	8,437 (6,892 to 10,259)	920 (631 to 1,346)	7,517 (6,133 to 9,401)	-44.3 (-55 to -30.7)	154.1 (78.6 to 263.2)	-49.2 (-59.3 to -35.3)
High income	Western Europe	France	YLLs	Age-standardized	18.4 (16.5 to 20.3)	0.7 (0.6 to 0.9)	42.8 (38.5 to 47.3)	5.6 (4.6 to 6.8)	1.1 (0.8 to 1.6)	11.8 (9.6 to 14.7)	-69.4 (-75.2 to -61.8)	58.1 (14.2 to 119.3)	-72.5 (-77.8 to -65.2)
High income	Western Europe	France	YLDs	All Ages	137 (91 to 196)	5 (3 to 9)	132 (87 to 189)	97 (64 to 141)	10 (6 to 16)	87 (57 to 126)	-29.4 (-45.1 to -8.3)	86 (41.6 to 160.2)	-34.2 (-49.5 to -12.5)
High income	Western Europe	France	YLDs	Age-standardized	0.2 (0.1 to 0.2)	0 (0 to 0)	0.4 (0.3 to 0.5)	0.1 (0 to 0.1)	0 (0 to 0)	0.1 (0.1 to 0.2)	-61 (-69.4 to -50)	8.7 (-17.5 to 40.9)	-65.2 (-73.1 to -54.2)
High income	Western Europe	Germany	Incidence	All Ages	133 (104 to 167)	18 (10 to 28)	115 (91 to 145)	170 (129 to 214)	21 (12 to 31)	149 (114 to 190)	27.7 (1.8 to 57.5)	12.7 (-9.7 to 50)	30.1 (0.4 to 64)
High income	Western Europe	Germany	Incidence	Age-standardized	0.1 (0.1 to 0.1)	0 (0 to 0.1)	0.2 (0.2 to 0.3)	0.1 (0.1 to 0.1)	0 (0 to 0.1)	0.2 (0.1 to 0.2)	-20.3 (-37.4 to -3.2)	-1.8 (-19.6 to 27.4)	-32.3 (-46.8 to -16.4)
High income	Western Europe	Germany	Prevalence	All Ages	623 (500 to 775)	38 (25 to 52)	585 (467 to 733)	609 (479 to 770)	46 (32 to 61)	563 (436 to 717)	-2.2 (-29.7 to 30.8)	22.6 (6.2 to 46.9)	-3.8 (-32.2 to 31.5)
High income	Western Europe	Germany	Prevalence	Age-standardized	0.5 (0.4 to 0.6)	0.1 (0 to 0.1)	1.3 (1 to 1.6)	0.3 (0.2 to 0.3)	0.1 (0 to 0.1)	0.6 (0.5 to 0.7)	-42.2 (-56.6 to -24.9)	3.3 (-8.5 to 23.9)	-54.1 (-66.9 to -37.9)
High income	Western Europe	Germany	Deaths	All Ages	804 (679 to 941)	27 (18 to 40)	777 (653 to 913)	578 (472 to 704)	25 (18 to 35)	553 (449 to 672)	-28 (-44 to -4.9)	-5.2 (-38.9 to 50.6)	-28.8 (-45.3 to -5)
High income	Western Europe	Germany	Deaths	Age-standardized	0.6 (0.5 to 0.7)	0 (0 to 0)	1.7 (1.4 to 2)	0.2 (0.2 to 0.3)	0 (0 to 0)	0.6 (0.5 to 0.7)	-59.3 (-68.2 to -47.1)	-35.3 (-55.7 to -1.8)	-66.5 (-74 to -55.9)
High income	Western Europe	Germany	DALYs	All Ages	14,587 (12,376 to 17,100)	448 (322 to 618)	14,139 (11,967 to 16,646)	7,796 (6,498 to 9,269)	357 (261 to 477)	7,439 (6,157 to 8,945)	-46.6 (-58.1 to -30)	-20.4 (-45.3 to 19.1)	-47.4 (-59.3 to -30.7)
High income	Western Europe	Germany	DALYs	Age-standardized	11.3 (9.6 to 13.2)	0.6 (0.4 to 0.8)	29.8 (25.3 to 34.9)	3.6 (3 to 4.2)	0.4 (0.3 to 0.5)	7.9 (6.5 to 9.4)	-68.2 (-75 to -58.4)	-40.4 (-56.6 to -15.2)	-73.6 (-79.4 to -65)
High income	Western Europe	Germany	YLLs	All Ages	14,481 (12,263 to 16,953)	442 (317 to 612)	14,039 (11,841 to 16,543)	7,691 (6,421 to 9,159)	349 (254 to 469)	7,342 (6,087 to 8,829)	-46.9 (-58.6 to -30.3)	-21 (-45.9 to 19)	-47.7 (-59.6 to -30.8)
High income	Western Europe	Germany	YLLs	Age-standardized	11.2 (9.5 to 13.1)	0.6 (0.4 to 0.8)	29.6 (25 to 34.7)	3.5 (3 to 4.2)	0.3 (0.3 to 0.4)	7.8 (6.4 to 9.3)	-68.4 (-75.3 to -58.5)	-41.2 (-57.5 to -15.8)	-73.7 (-79.6 to -65.2)
High income	Western Europe	Germany	YLDs	All Ages	106 (70 to 151)	6 (4 to 10)	100 (65 to 143)	105 (68 to 152)	8 (5 to 12)	97 (62 to 143)	-1.7 (-28.5 to 31.3)	22.4 (6 to 46.5)	-3.2 (-31.4 to 31.8)
High income	Western Europe	Germany	YLDs	Age-standardized	0.1 (0.1 to 0.1)	0 (0 to 0)	0.2 (0.1 to 0.3)	0 (0 to 0.1)	0 (0 to 0)	0.1 (0.1 to 0.2)	-42.2 (-56.5 to -25)	2.7 (-9 to 22.7)	-54 (-66.9 to -38)
High income	Western Europe	Greece	Incidence	All Ages	3 (2 to 6)	2 (1 to 3)	2 (1 to 3)	4 (2 to 6)	2 (1 to 3)	2 (1 to 4)	19.7 (-1.8 to 50.8)	15.8 (-8.4 to 55.3)	23.1 (-11.6 to 66.5)
High income	Western Europe	Greece	Incidence	Age-standardized	0 (0 to 0)	0 (0 to 0)	0 (0 to 0.1)	0 (0 to 0)	0 (0 to 0)	0 (0 to 0.1)	1.6 (-20.7 to 18.8)	2.1 (-23.8 to 42.1)	0.8 (-26 to 31.5)
High income	Western Europe	Greece	Prevalence	All Ages	9 (6 to 12)	4 (2 to 5)	5 (4 to 7)	11 (8 to 15)	4 (3 to 6)	7 (5 to 9)	24.1 (9 to 43.9)	24.2 (6.9 to 45.6)	24.1 (3.6 to 47.3)
High income	Western Europe	Greece	Prevalence	Age-standardized	0.1 (0 to 0.1)	0.1 (0 to 0.1)	0.1 (0.1 to 0.1)	0.1 (0 to 0.1)	0.1 (0 to 0.1)	0.1 (0.1 to 0.1)	-5.5 (-18.7 to 5.6)	0 (-15.9 to 23.6)	-10.2 (-25.6 to 3)
High income	Western Europe	Greece	Deaths	All Ages	5 (4 to 5)	0 (0 to 0)	4 (4 to 5)	4 (3 to 4)	0 (0 to 1)	3 (3 to 4)	-20.1 (-37.1 to 0.9)	57.2 (-4.5 to 131.6)	-25.5 (-43.1 to -3.2)
High income	Western Europe	Greece	Deaths	Age-standardized	0 (0 to 0)	0 (0 to 0)	0.1 (0.1 to 0.1)	0 (0 to 0)	0 (0 to 0)	0 (0 to 0)	-51.4 (-61.4 to -38.8)	-13.5 (-41.6 to 21.1)	-55.5 (-65.6 to -42.6)
High income	Western Europe	Greece	DALYs	All Ages	84 (73 to 97)	6 (5 to 7)	79 (68 to 91)	63 (52 to 75)	7 (5 to 10)	55 (45 to 68)	-25.5 (-41.7 to -6)	28.2 (-10.4 to 72.8)	-29.5 (-45.7 to -8.3)
High income	Western Europe	Greece	DALYs	Age-standardized	0.6 (0.5 to 0.6)	0.1 (0.1 to 0.1)	1.1 (1 to 1.3)	0.3 (0.3 to 0.4)	0.1 (0.1 to 0.1)	0.6 (0.5 to 0.7)	-43.9 (-55.5 to -29)	-9.4 (-32.9 to 17)	-48.1 (-59.9 to -32.6)
High income	Western Europe	Greece	YLLs	All Ages	83 (72 to 95)	5 (4 to 7)	78 (67 to 90)	61 (50 to 73)	7 (5 to 9)	54 (43 to 67)	-26.4 (-42.5 to -6.4)	28.6 (-13.4 to 77.7)	-30.1 (-46.3 to -8.6)
High income	Western Europe	Greece	YLLs	Age-standardized	0.5 (0.5 to 0.6)	0.1 (0.1 to 0.1)	1.1 (1 to 1.3)	0.3 (0.2 to 0.4)	0.1 (0.1 to 0.1)	0.6 (0.5 to 0.7)	-44.7 (-56.5 to -29.5)	-10.6 (-36.7 to 19.1)	-48.6 (-60.4 to -32.9)
High income	Western Europe	Greece	YLDs	All Ages	1 (1 to 2)	1 (0 to 1)	1 (1 to 1)	2 (1 to 3)	1 (0 to 1)	1 (1 to 2)	24.2 (9.4 to 43.4)	24.4 (7.4 to 45.2)	24.1 (3.7 to 47)
High income	Western Europe	Greece	YLDs	Age-standardized	0 (0 to 0)	0 (0 to 0)	0 (0 to 0)	0 (0 to 0)	0 (0 to 0)	0 (0 to 0)	-6.2 (-19.3 to 4.6)	-0.4 (-16 to 23)	-11 (-25.8 to 2.2)
High income	Western Europe	Iceland	Incidence	All Ages	0 (0 to 0)	0 (0 to 0)	0 (0 to 0)	0 (0 to 0)	0 (0 to 0)	0 (0 to 0)	39.5 (9.4 to 73.1)	45.5 (-10.6 to 85.9)	36.9 (2.8 to 79.9)
High income	Western Europe	Iceland	Incidence	Age-standardized	0 (0 to 0.1)	0 (0 to 0)	0.1 (0 to 0.1)	0 (0 to 0.1)	0 (0 to 0)	0 (0 to 0.1)	-13.4 (-34.5 to 5)	-1.6 (-41.1 to 26.6)	-22 (-42.1 to 0.3)
High income	Western Europe	Iceland	Prevalence	All Ages	0 (0 to 0)	0 (0 to 0)	0 (0 to 0)	0 (0 to 0)	0 (0 to 0)	0 (0 to 0)	42.3 (19.6 to 67.3)	52.7 (10 to 85.2)	37.1 (11.2 to 69.4)
High income	Western Europe	Iceland	Prevalence	Age-standardized	0.1 (0 to 0.1)	0 (0 to 0.1)	0.1 (0.1 to 0.1)	0.1 (0 to 0.1)	0 (0 to 0.1)	0.1 (0 to 0.1)	-13.6 (-29.5 to 1.6)	-2 (-32.5 to 18.2)	-22.3 (-38.3 to -2.7)
High income	Western Europe	Iceland	Deaths	All Ages	0 (0 to 0)	0 (0 to 0)	0 (0 to 0)	0 (0 to 0)	0 (0 to 0)	0 (0 to 0)	-39.2 (-52.9 to -20.6)	78.9 (14.2 to 153.5)	-41.3 (-54.9 to -23)
High income	Western Europe	Iceland	Deaths	Age-standardized	0.1 (0.1 to 0.1)	0 (0 to 0)	0.2 (0.1 to 0.2)	0 (0 to 0)	0 (0 to 0)	0 (0 to 0.1)	-69.2 (-76 to -60.2)	-10.7 (-43.5 to 24.9)	-71.6 (-78.1 to -63.1)
High income	Western Europe	Iceland	DALYs	All Ages	4 (3 to 4)	0 (0 to 0)	4 (3 to 4)	2 (2 to 3)	0 (0 to 0)	2 (2 to 2)	-42.4 (-55.7 to -25)	54.4 (8.8 to 105.8)	-44.2 (-57.5 to -27)
High income	Western Europe	Iceland	DALYs	Age-standardized	1.3 (1.1 to 1.5)	0 (0 to 0.1)	2.8 (2.3 to 3.3)	0.4 (0.3 to 0.5)	0 (0 to 0.1)	0.8 (0.6 to 0.9)	-69.8 (-76.7 to -60.6)	-11.5 (-36.6 to 16.3)	-72.3 (-78.9 to -63.7)
High income	Western Europe	Iceland	YLLs	All Ages	4 (3 to 4)	0 (0 to 0)	4 (3 to 4)	2 (2 to 3)	0 (0 to 0)	2 (2 to 2)	-43.1 (-56.6 to -25.5)	54.7 (0.3 to 114.4)	-44.7 (-57.9 to -27.3)
High income	Western Europe	Iceland	YLLs	Age-standardized	1.3 (1 to 1.5)	0 (0 to 0.1)	2.7 (2.3 to 3.3)	0.4 (0.3 to 0.5)	0 (0 to 0)	0.8 (0.6 to 0.9)	-70.3 (-77.3 to -61.2)	-13.3 (-43.1 to 17.4)	-72.6 (-79.1 to -63.9)
High income	Western Europe	Iceland	YLDs	All Ages	0 (0 to 0)	0 (0 to 0)	0 (0 to 0)	0 (0 to 0)	0 (0 to 0)	0 (0 to 0)	42 (19.5 to 66.1)	52.5 (10.9 to 83.9)	37 (11 to 69.9)
High income	Western Europe	Iceland	YLDs	Age-standardized	0 (0 to 0)	0 (0 to 0)	0 (0 to 0)	0 (0 to 0)	0 (0 to 0)	0 (0 to 0)	-14.3 (-29.5 to 0.3)	-2.5 (-32.4 to 17.3)	-23 (-38.6 to -3.2)
High income	Western Europe	Ireland	Incidence	All Ages	2 (1 to 2)	0 (0 to 1)	1 (1 to 2)	3 (2 to 5)	1 (0 to 1)	2 (1 to 3)	100.5 (55.1 to 163)	56.1 (2.4 to 115.3)	121.7 (57.4 to 208)
High income	Western Europe	Ireland	Incidence	Age-standardized	0 (0 to 0.1)	0 (0 to 0)	0.1 (0 to 0.1)	0 (0 to 0.1)	0 (0 to 0)	0.1 (0 to 0.1)	17.6 (-7.2 to 48.5)	0.7 (-33.8 to 38.2)	20.6 (-10.8 to 59.3)
High income	Western Europe	Ireland	Prevalence	All Ages	3 (2 to 5)	1 (1 to 2)	2 (2 to 3)	6 (4 to 8)	2 (1 to 2)	4 (3 to 5)	64.9 (34.9 to 95.3)	62.5 (30.6 to 95.9)	65.9 (25.7 to 108.7)
High income	Western Europe	Ireland	Prevalence	Age-standardized	0.1 (0.1 to 0.1)	0.1 (0 to 0.1)	0.1 (0.1 to 0.2)	0.1 (0.1 to 0.1)	0.1 (0 to 0.1)	0.1 (0.1 to 0.2)	-4.2 (-21.3 to 12.3)	-0.5 (-22.1 to 22.9)	-9.9 (-30.2 to 10.5)
High income	Western Europe	Ireland	Deaths	All Ages	3 (3 to 4)	0 (0 to 0)	3 (3 to 4)	5 (4 to 6)	0 (0 to 0)	5 (4 to 6)	60.9 (23.3 to 109.1)	230.7 (124.9 to 370.8)	57.8 (19.4 to 105.9)
High income	Western Europe	Ireland	Deaths	Age-standardized	0.1 (0.1 to 0.1)	0 (0 to 0)	0.2 (0.2 to 0.2)	0.1 (0.1 to 0.1)	0 (0 to 0)	0.1 (0.1 to 0.2)	-11.2 (-31.6 to 15)	85 (29.1 to 162.6)	-19.2 (-38.5 to 4.7)
High income	Western Europe	Ireland	DALYs	All Ages	62 (53 to 72)	1 (1 to 2)	61 (52 to 71)	89 (70 to 111)	4 (3 to 5)	85 (66 to 108)	44.1 (10 to 88.3)	208.7 (119.1 to 345.6)	41 (5.9 to 85.5)
High income	Western Europe	Ireland	DALYs	Age-standardized	1.5 (1.3 to 1.7)	0.1 (0 to 0.1)	3.2 (2.8 to 3.7)	1.2 (0.9 to 1.5)	0.1 (0.1 to 0.1)	2.4 (1.9 to 3)	-18.8 (-38.1 to 6.2)	88.4 (35.9 to 174.5)	-25.2 (-43 to -1.5)
High income	Western Europe	Ireland	YLLs	All Ages	61 (52 to 71)	1 (1 to 1)	60 (51 to 70)	88 (69 to 111)	3 (2 to 4)	85 (66 to 107)	44 (9.2 to 88.3)	233.3 (127.9 to 399.5)	40.8 (5.6 to 85.5)
High income	Western Europe	Ireland	YLLs	Age-standardized	1.4 (1.2 to 1.7)	0 (0 to 0.1)	3.2 (2.7 to 3.7)	1.2 (0.9 to 1.5)	0.1 (0.1 to 0.1)	2.4 (1.8 to 3)	-18.9 (-38.3 to 6.3)	106.2 (43.8 to 211.4)	-25.3 (-43.2 to -1.5)
High income	Western Europe	Ireland	YLDs	All Ages	1 (0 to 1)	0 (0 to 0)	0 (0 to 1)	1 (1 to 1)	0 (0 to 0)	1 (0 to 1)	65 (35.2 to 96.2)	61.3 (30.4 to 93.3)	66.6 (26.5 to 109.4)
High income	Western Europe	Ireland	YLDs	Age-standardized	0 (0 to 0)	0 (0 to 0)	0 (0 to 0)	0 (0 to 0)	0 (0 to 0)	0 (0 to 0)	-4.3 (-21.6 to 12)	-1.2 (-22.3 to 21.3)	-9.9 (-30.1 to 10.4)
High income	Western Europe	Israel	Incidence	All Ages	1 (1 to 2)	1 (0 to 1)	1 (0 to 1)	3 (1 to 4)	1 (1 to 2)	1 (1 to 2)	97 (59.1 to 150.9)	101.2 (28 to 206.3)	93.6 (51.9 to 142.2)
High income	Western Europe	Israel	Incidence	Age-standardized	0 (0 to 0)	0 (0 to 0)	0 (0 to 0.1)	0 (0 to 0)	0 (0 to 0)	0 (0 to 0.1)	-4.2 (-24.1 to 21.3)	1.4 (-37.5 to 61.8)	-8.6 (-29.4 to 16.2)
High income	Western Europe	Israel	Prevalence	All Ages	3 (2 to 5)	1 (1 to 2)	2 (1 to 3)	6 (4 to 9)	3 (2 to 4)	4 (2 to 5)	87.4 (59 to 116.2)	108.1 (69.9 to 177.7)	74.4 (41.7 to 101.5)
High income	Western Europe	Israel	Prevalence	Age-standardized	0.1 (0 to 0.1)	0.1 (0 to 0.1)	0.1 (0.1 to 0.1)	0.1 (0 to 0.1)	0.1 (0 to 0.1)	0.1 (0 to 0.1)	-13.7 (-27.5 to -0.4)	-0.7 (-21.7 to 37.7)	-21.6 (-37.3 to -9.2)
High income	Western Europe	Israel	Deaths	All Ages	2 (2 to 2)	0 (0 to 0)	2 (2 to 2)	3 (2 to 3)	0 (0 to 0)	3 (2 to 3)	33.9 (6.1 to 70)	48.4 (5.7 to 124.5)	33 (4.1 to 71.3)
High income	Western Europe	Israel	Deaths	Age-standardized	0 (0 to 0.1)	0 (0 to 0)	0.1 (0.1 to 0.1)	0 (0 to 0)	0 (0 to 0)	0.1 (0 to 0.1)	-45.7 (-56.8 to -31.3)	-44.7 (-59.8 to -16.2)	-45.4 (-56.9 to -30.1)
High income	Western Europe	Israel	DALYs	All Ages	51 (43 to 59)	3 (2 to 4)	48 (40 to 57)	66 (54 to 80)	4 (3 to 5)	62 (51 to 76)	28.6 (1.2 to 64.7)	33.9 (-0.1 to 92.9)	28.3 (0.4 to 67.1)
High income	Western Europe	Israel	DALYs	Age-standardized	1.1 (0.9 to 1.3)	0.1 (0.1 to 0.1)	2.2 (1.9 to 2.6)	0.6 (0.5 to 0.8)	0.1 (0.1 to 0.1)	1.3 (1 to 1.5)	-42.4 (-55.1 to -26.1)	-39.1 (-54 to -12.4)	-43.7 (-56.4 to -26.8)
High income	Western Europe	Israel	YLLs	All Ages	51 (42 to 59)	3 (2 to 3)	48 (40 to 56)	65 (53 to 79)	3 (2 to 5)	61 (50 to 75)	27.9 (0.4 to 64.6)	27.9 (-7.1 to 92.8)	27.9 (-0.1 to 67)
High income	Western Europe	Israel	YLLs	Age-standardized	1.1 (0.9 to 1.3)	0.1 (0.1 to 0.1)	2.2 (1.8 to 2.6)	0.6 (0.5 to 0.8)	0.1 (0.1 to 0.1)	1.2 (1 to 1.5)	-42.7 (-55.4 to -26.3)	-42.3 (-57.7 to -14)	-43.9 (-56.6 to -26.9)
High income	Western Europe	Israel	YLDs	All Ages	1 (0 to 1)	0 (0 to 0)	0 (0 to 1)	1 (1 to 2)	0 (0 to 1)	1 (0 to 1)	86.5 (58.3 to 114)	107.6 (70.3 to 175.4)	73.6 (41.3 to 99.6)
High income	Western Europe	Israel	YLDs	Age-standardized	0 (0 to 0)	0 (0 to 0)	0 (0 to 0)	0 (0 to 0)	0 (0 to 0)	0 (0 to 0)	-14.5 (-28.3 to -1.7)	-1.3 (-21.6 to 36.3)	-22.3 (-38 to -9.8)
High income	Western Europe	Italy	Incidence	All Ages	4,791 (3,844 to 6,024)	289 (238 to 354)	4,502 (3,597 to 5,689)	2,626 (2,196 to 3,135)	281 (231 to 333)	2,345 (1,949 to 2,805)	-45.2 (-54.3 to -31.1)	-2.7 (-12 to 6.7)	-47.9 (-57 to -33.3)
High income	Western Europe	Italy	Incidence	Age-standardized	5.6 (4.5 to 7)	0.6 (0.5 to 0.8)	11.5 (9.2 to 14.4)	2.1 (1.7 to 2.3)	0.4 (0.4 to 0.5)	3.8 (3.3 to 4.5)	-64.2 (-69.9 to -56.4)	-29.6 (-36.6 to -22.8)	-66.6 (-72 to -58.7)
High income	Western Europe	Italy	Prevalence	All Ages	58,800 (45,508 to 78,288)	2,528 (2,069 to 3,229)	56,272 (43,314 to 75,172)	26,559 (22,575 to 31,730)	2,593 (2,157 to 3,114)	23,966 (20,328 to 28,693)	-54.8 (-64 to -44.6)	2.6 (-8 to 12.4)	-57.4 (-66.4 to -47.1)
High income	Western Europe	Italy	Prevalence	Age-standardized	63.6 (49.3 to 85)	5 (4.1 to 6.2)	146.5 (113.3 to 193.2)	18.1 (15.4 to 21.2)	3.3 (2.8 to 3.9)	37 (31.6 to 43.8)	-71.6 (-77.6 to -65.4)	-34 (-40.6 to -27.9)	-74.7 (-79.9 to -69.1)
High income	Western Europe	Italy	Deaths	All Ages	1,051 (1,005 to 1,095)	27 (24 to 35)	1,024 (978 to 1,064)	498 (431 to 552)	35 (25 to 42)	463 (402 to 515)	-52.6 (-58.4 to -47.2)	28.2 (-1.3 to 53.3)	-54.8 (-60.4 to -49.3)
High income	Western Europe	Italy	Deaths	Age-standardized	1.1 (1.1 to 1.2)	0.1 (0 to 0.1)	2.8 (2.6 to 2.9)	0.3 (0.2 to 0.3)	0 (0 to 0)	0.6 (0.6 to 0.7)	-76.2 (-78.9 to -73.5)	-31 (-44.2 to -18.2)	-76.9 (-79.6 to -74.2)
High income	Western Europe	Italy	DALYs	All Ages	27,176 (24,183 to 31,110)	891 (735 to 1,120)	26,285 (23,418 to 30,043)	9,787 (8,412 to 11,353)	891 (708 to 1,112)	8,896 (7,659 to 10,311)	-64 (-67.5 to -60.2)	0 (-11.3 to 12.2)	-66.2 (-69.6 to -62.5)
High income	Western Europe	Italy	DALYs	Age-standardized	29.5 (26.2 to 33.8)	1.9 (1.5 to 2.3)	68 (60.6 to 77.7)	6.3 (5.4 to 7.4)	1.2 (1 to 1.5)	13.2 (11.3 to 15.3)	-78.7 (-80.8 to -76.3)	-34.2 (-41.5 to -24.9)	-80.6 (-82.5 to -78.4)
High income	Western Europe	Italy	YLLs	All Ages	19,199 (18,440 to 19,955)	514 (459 to 657)	18,685 (17,939 to 19,411)	6,028 (5,278 to 6,722)	495 (380 to 632)	5,533 (4,853 to 6,156)	-68.6 (-72.2 to -64.8)	-3.6 (-21.8 to 15)	-70.4 (-73.8 to -66.6)
High income	Western Europe	Italy	YLLs	Age-standardized	20.8 (20 to 21.6)	1.1 (1 to 1.4)	48.3 (46.3 to 50.1)	3.7 (3.3 to 4.1)	0.7 (0.6 to 1)	7.9 (7 to 8.8)	-82.3 (-84.2 to -80.2)	-35.5 (-46.4 to -20.5)	-83.6 (-85.3 to -81.5)
High income	Western Europe	Italy	YLDs	All Ages	7,977 (5,033 to 11,796)	377 (243 to 555)	7,599 (4,784 to 11,274)	3,758 (2,556 to 5,314)	396 (256 to 576)	3,363 (2,295 to 4,733)	-52.9 (-62.5 to -42.3)	4.9 (-6.5 to 17.4)	-55.8 (-65.3 to -45.3)
High income	Western Europe	Italy	YLDs	Age-standardized	8.7 (5.4 to 12.8)	0.7 (0.5 to 1.1)	19.7 (12.5 to 29.1)	2.6 (1.8 to 3.7)	0.5 (0.3 to 0.7)	5.3 (3.6 to 7.4)	-70 (-76.4 to -63.3)	-32.1 (-39.1 to -25.1)	-73.4 (-78.9 to -67.4)
High income	Western Europe	Luxembourg	Incidence	All Ages	0 (0 to 0)	0 (0 to 0)	0 (0 to 0)	0 (0 to 0)	0 (0 to 0)	0 (0 to 0)	45.4 (18.3 to 76.4)	88 (48.1 to 153.2)	30.8 (0.1 to 70.5)
High income	Western Europe	Luxembourg	Incidence	Age-standardized	0.1 (0 to 0.1)	0 (0 to 0)	0.1 (0.1 to 0.1)	0 (0 to 0.1)	0 (0 to 0.1)	0.1 (0 to 0.1)	-16.6 (-33.1 to 0.3)	9.2 (-17.8 to 42.5)	-31.7 (-48.1 to -13)
High income	Western Europe	Luxembourg	Prevalence	All Ages	2 (1 to 2)	0 (0 to 0)	1 (1 to 2)	1 (1 to 2)	0 (0 to 0)	1 (1 to 1)	-13.3 (-33.2 to 11.8)	70 (47.1 to 99.8)	-24.3 (-44.3 to 1.2)
High income	Western Europe	Luxembourg	Prevalence	Age-standardized	0.3 (0.2 to 0.3)	0.1 (0 to 0.1)	0.6 (0.5 to 0.8)	0.1 (0.1 to 0.2)	0.1 (0 to 0.1)	0.2 (0.2 to 0.3)	-50.9 (-62.1 to -37.6)	-0.9 (-16.4 to 17.9)	-63.1 (-72.5 to -50.6)
High income	Western Europe	Luxembourg	Deaths	All Ages	2 (1 to 2)	0 (0 to 0)	2 (1 to 2)	1 (1 to 1)	0 (0 to 0)	1 (1 to 1)	-30.7 (-48.1 to -10.5)	100.5 (18.9 to 204.4)	-39.9 (-56 to -21.2)
High income	Western Europe	Luxembourg	Deaths	Age-standardized	0.3 (0.3 to 0.3)	0 (0 to 0)	0.7 (0.6 to 0.9)	0.1 (0.1 to 0.1)	0 (0 to 0)	0.2 (0.2 to 0.3)	-62.9 (-72.1 to -51.9)	13.6 (-30.3 to 68.1)	-72.2 (-79.4 to -63.6)
High income	Western Europe	Luxembourg	DALYs	All Ages	31 (27 to 37)	2 (1 to 2)	30 (25 to 35)	19 (15 to 24)	3 (2 to 4)	16 (12 to 20)	-39.3 (-54.3 to -20.6)	81.6 (15.2 to 163.2)	-46.6 (-61.2 to -28.5)
High income	Western Europe	Luxembourg	DALYs	Age-standardized	5.6 (4.8 to 6.6)	0.5 (0.4 to 0.7)	13.3 (11.2 to 15.7)	1.9 (1.5 to 2.4)	0.6 (0.4 to 0.8)	3.4 (2.6 to 4.4)	-66 (-74.4 to -55.4)	13.3 (-25.8 to 63.4)	-74.1 (-81 to -65.4)
High income	Western Europe	Luxembourg	YLLs	All Ages	31 (26 to 37)	2 (1 to 2)	29 (25 to 35)	19 (15 to 24)	3 (2 to 4)	16 (12 to 20)	-39.5 (-54.8 to -20.6)	81.8 (14.4 to 164.7)	-46.8 (-61.4 to -28.7)
High income	Western Europe	Luxembourg	YLLs	Age-standardized	5.6 (4.8 to 6.6)	0.5 (0.4 to 0.7)	13.2 (11.1 to 15.6)	1.9 (1.5 to 2.4)	0.6 (0.4 to 0.8)	3.4 (2.6 to 4.3)	-66.1 (-74.6 to -55.4)	13.6 (-26 to 64.6)	-74.2 (-81.1 to -65.4)
High income	Western Europe	Luxembourg	YLDs	All Ages	0 (0 to 0)	0 (0 to 0)	0 (0 to 0)	0 (0 to 0)	0 (0 to 0)	0 (0 to 0)	-13.9 (-33.9 to 11.2)	69.9 (47.4 to 98.5)	-24.7 (-44.7 to 0.9)
High income	Western Europe	Luxembourg	YLDs	Age-standardized	0 (0 to 0.1)	0 (0 to 0)	0.1 (0.1 to 0.2)	0 (0 to 0)	0 (0 to 0)	0 (0 to 0.1)	-51.4 (-62.6 to -38.1)	-1.2 (-16.2 to 17.1)	-63.3 (-72.5 to -51)
High income	Western Europe	Malta	Incidence	All Ages	0 (0 to 0)	0 (0 to 0)	0 (0 to 0)	0 (0 to 1)	0 (0 to 0)	0 (0 to 1)	73.2 (36.8 to 117.9)	57.1 (12.5 to 136.3)	77.1 (35.3 to 125.2)
High income	Western Europe	Malta	Incidence	Age-standardized	0.1 (0 to 0.1)	0 (0 to 0)	0.1 (0.1 to 0.2)	0.1 (0 to 0.1)	0 (0 to 0)	0.1 (0.1 to 0.1)	-10 (-25.6 to 6.1)	6.7 (-19.1 to 59.1)	-17.9 (-34.8 to -0.2)
High income	Western Europe	Malta	Prevalence	All Ages	0 (0 to 1)	0 (0 to 0)	0 (0 to 0)	1 (1 to 1)	0 (0 to 0)	1 (0 to 1)	52.6 (29.4 to 80.1)	60.6 (28.7 to 101.2)	50 (20.9 to 81.5)
High income	Western Europe	Malta	Prevalence	Age-standardized	0.1 (0.1 to 0.2)	0.1 (0 to 0.1)	0.2 (0.2 to 0.3)	0.1 (0.1 to 0.1)	0.1 (0 to 0.1)	0.1 (0.1 to 0.2)	-19 (-31.1 to -8.3)	0.8 (-16.9 to 25.3)	-28.4 (-40.7 to -16.4)
High income	Western Europe	Malta	Deaths	All Ages	1 (1 to 1)	0 (0 to 0)	1 (1 to 1)	1 (1 to 1)	0 (0 to 0)	1 (1 to 1)	-7.2 (-28.7 to 19.2)	191.7 (84.5 to 346.5)	-16.4 (-36.9 to 10.4)
High income	Western Europe	Malta	Deaths	Age-standardized	0.2 (0.2 to 0.2)	0 (0 to 0)	0.5 (0.4 to 0.5)	0.1 (0.1 to 0.1)	0 (0 to 0)	0.2 (0.1 to 0.2)	-61.2 (-69.8 to -50.1)	19.5 (-23.4 to 80.5)	-66.5 (-74.3 to -56.3)
High income	Western Europe	Malta	DALYs	All Ages	15 (13 to 18)	1 (0 to 1)	15 (12 to 17)	12 (10 to 15)	2 (1 to 2)	11 (9 to 14)	-18.3 (-37.3 to 5.4)	142.5 (57.4 to 266)	-25.2 (-43.2 to -2.4)
High income	Western Europe	Malta	DALYs	Age-standardized	3.5 (3 to 4.1)	0.3 (0.2 to 0.4)	7.8 (6.7 to 9.1)	1.3 (1 to 1.6)	0.3 (0.2 to 0.4)	2.5 (2 to 3)	-63.1 (-71.5 to -52.7)	16.8 (-21.8 to 72)	-68.3 (-75.8 to -58.8)
High income	Western Europe	Malta	YLLs	All Ages	15 (13 to 18)	1 (0 to 1)	15 (12 to 17)	12 (10 to 15)	2 (1 to 2)	11 (9 to 13)	-18.7 (-37.7 to 5.1)	145 (57.4 to 273.1)	-25.6 (-43.5 to -2.6)
High income	Western Europe	Malta	YLLs	Age-standardized	3.5 (3 to 4.1)	0.3 (0.2 to 0.3)	7.8 (6.6 to 9.1)	1.3 (1 to 1.6)	0.3 (0.2 to 0.4)	2.4 (2 to 3)	-63.4 (-71.8 to -52.8)	17.3 (-22.4 to 75.3)	-68.5 (-76 to -58.9)
High income	Western Europe	Malta	YLDs	All Ages	0 (0 to 0)	0 (0 to 0)	0 (0 to 0)	0 (0 to 0)	0 (0 to 0)	0 (0 to 0)	53.7 (30 to 81.6)	62.8 (30.9 to 102.3)	51 (21.2 to 83.3)
High income	Western Europe	Malta	YLDs	Age-standardized	0 (0 to 0)	0 (0 to 0)	0 (0 to 0.1)	0 (0 to 0)	0 (0 to 0)	0 (0 to 0)	-19.3 (-31.5 to -8.4)	0.8 (-16.3 to 23.7)	-28.6 (-41 to -16.6)
High income	Western Europe	Monaco	Incidence	All Ages	0 (0 to 0)	0 (0 to 0)	0 (0 to 0)	0 (0 to 0)	0 (0 to 0)	0 (0 to 0)	39.8 (21.9 to 58.2)	30.2 (6.8 to 65.5)	44.2 (21.8 to 69)
High income	Western Europe	Monaco	Incidence	Age-standardized	0.1 (0.1 to 0.1)	0.1 (0 to 0.1)	0.2 (0.1 to 0.2)	0.1 (0.1 to 0.1)	0.1 (0 to 0.1)	0.1 (0.1 to 0.2)	3.4 (-9.9 to 16.9)	5.6 (-19.1 to 37.4)	-1.7 (-16.3 to 15.4)
High income	Western Europe	Monaco	Prevalence	All Ages	0 (0 to 0)	0 (0 to 0)	0 (0 to 0)	0 (0 to 1)	0 (0 to 0)	0 (0 to 0)	40 (16.7 to 67.1)	29.4 (10.1 to 53.1)	41.2 (15 to 71.3)
High income	Western Europe	Monaco	Prevalence	Age-standardized	0.4 (0.3 to 0.5)	0.1 (0.1 to 0.1)	0.8 (0.7 to 1)	0.4 (0.3 to 0.5)	0.1 (0.1 to 0.1)	0.8 (0.6 to 1)	2.7 (-14.4 to 21.4)	4.8 (-14.7 to 28.5)	-7 (-24.2 to 12.1)
High income	Western Europe	Monaco	Deaths	All Ages	0 (0 to 1)	0 (0 to 0)	0 (0 to 0)	1 (0 to 1)	0 (0 to 0)	0 (0 to 0)	7.9 (-27.6 to 65.3)	32.7 (-28 to 157.4)	-0.8 (-38.8 to 67.2)
High income	Western Europe	Monaco	Deaths	Age-standardized	0.6 (0.4 to 0.8)	0.2 (0.2 to 0.4)	1.1 (0.8 to 1.5)	0.5 (0.3 to 0.6)	0.3 (0.2 to 0.4)	0.7 (0.5 to 1)	-21.4 (-47.1 to 18.1)	5 (-41.8 to 95.1)	-34.4 (-58.4 to 6.9)
High income	Western Europe	Monaco	DALYs	All Ages	8 (5 to 10)	2 (1 to 3)	6 (4 to 8)	8 (5 to 10)	2 (1 to 3)	5 (4 to 7)	-0.9 (-33.8 to 51.7)	26.4 (-31.8 to 138.3)	-9.1 (-41.7 to 48.7)
High income	Western Europe	Monaco	DALYs	Age-standardized	10.8 (7.7 to 14.6)	4.5 (2.9 to 7)	19.2 (13.3 to 26.8)	7.9 (5.7 to 10.4)	4.6 (2.8 to 6.8)	11.8 (8.1 to 16.2)	-26.4 (-50.3 to 10.3)	1.8 (-43.3 to 80.5)	-38.3 (-59.1 to -1.7)
High income	Western Europe	Monaco	YLLs	All Ages	8 (5 to 10)	2 (1 to 3)	6 (4 to 8)	8 (5 to 10)	2 (1 to 3)	5 (4 to 7)	-1.1 (-34.3 to 52)	26.4 (-31.9 to 138.7)	-9.5 (-42.3 to 48.5)
High income	Western Europe	Monaco	YLLs	Age-standardized	10.7 (7.6 to 14.6)	4.5 (2.9 to 7)	19 (13.2 to 26.7)	7.9 (5.6 to 10.3)	4.6 (2.8 to 6.8)	11.7 (8 to 16.1)	-26.6 (-50.5 to 10.4)	1.8 (-43.4 to 80.7)	-38.5 (-59.3 to -1.6)
High income	Western Europe	Monaco	YLDs	All Ages	0 (0 to 0)	0 (0 to 0)	0 (0 to 0)	0 (0 to 0)	0 (0 to 0)	0 (0 to 0)	38.9 (15.7 to 64.3)	29.1 (9.9 to 52.7)	40 (14.2 to 68.5)
High income	Western Europe	Monaco	YLDs	Age-standardized	0.1 (0 to 0.1)	0 (0 to 0)	0.1 (0.1 to 0.2)	0.1 (0 to 0.1)	0 (0 to 0)	0.1 (0.1 to 0.2)	2 (-14.9 to 19.7)	4.2 (-15 to 27.7)	-7.8 (-24.7 to 10.3)
High income	Western Europe	Netherlands	Incidence	All Ages	17 (13 to 22)	2 (1 to 4)	15 (12 to 19)	14 (9 to 20)	3 (1 to 5)	11 (8 to 16)	-19.7 (-41.9 to 12.3)	19.1 (-14.2 to 83.3)	-26.1 (-49.1 to 9)
High income	Western Europe	Netherlands	Incidence	Age-standardized	0.1 (0.1 to 0.1)	0 (0 to 0)	0.2 (0.1 to 0.2)	0 (0 to 0.1)	0 (0 to 0)	0.1 (0.1 to 0.1)	-44.4 (-60.9 to -25.2)	-0.2 (-32.5 to 68.7)	-54.8 (-68.4 to -36.6)
High income	Western Europe	Netherlands	Prevalence	All Ages	118 (93 to 150)	6 (3 to 8)	113 (88 to 144)	31 (24 to 40)	7 (4 to 9)	24 (19 to 31)	-74 (-81 to -63.7)	23 (3.4 to 52.4)	-78.8 (-84.9 to -69.5)
High income	Western Europe	Netherlands	Prevalence	Age-standardized	0.6 (0.4 to 0.7)	0.1 (0 to 0.1)	1.4 (1.1 to 1.8)	0.1 (0.1 to 0.1)	0.1 (0 to 0.1)	0.2 (0.1 to 0.2)	-81.7 (-87 to -74.4)	-4.7 (-23.7 to 26)	-87.8 (-91.2 to -82.6)
High income	Western Europe	Netherlands	Deaths	All Ages	98 (87 to 108)	1 (1 to 2)	96 (85 to 107)	28 (23 to 33)	1 (1 to 2)	27 (22 to 32)	-71.7 (-77.2 to -64.9)	-17.8 (-54.4 to 21.6)	-72.5 (-78 to -65.7)
High income	Western Europe	Netherlands	Deaths	Age-standardized	0.5 (0.4 to 0.5)	0 (0 to 0)	1.2 (1.1 to 1.3)	0.1 (0.1 to 0.1)	0 (0 to 0)	0.2 (0.1 to 0.2)	-84.1 (-87.2 to -80.4)	-49.5 (-71.6 to -26.5)	-86.3 (-89 to -83.1)
High income	Western Europe	Netherlands	DALYs	All Ages	1,644 (1,465 to 1,826)	22 (17 to 28)	1,622 (1,445 to 1,805)	404 (333 to 479)	17 (10 to 23)	387 (316 to 463)	-75.4 (-80.2 to -69.8)	-23.2 (-55.9 to 9.7)	-76.2 (-80.9 to -70.4)
High income	Western Europe	Netherlands	DALYs	Age-standardized	7.8 (7 to 8.7)	0.2 (0.1 to 0.2)	19.4 (17.4 to 21.4)	1.1 (0.9 to 1.3)	0.1 (0.1 to 0.1)	2.4 (1.9 to 2.8)	-85.9 (-88.6 to -82.6)	-48.7 (-68.4 to -29.6)	-87.8 (-90.2 to -85)
High income	Western Europe	Netherlands	YLLs	All Ages	1,624 (1,448 to 1,807)	21 (16 to 27)	1,603 (1,428 to 1,784)	399 (327 to 474)	16 (9 to 22)	383 (311 to 457)	-75.5 (-80.3 to -69.7)	-25.1 (-58.1 to 9.1)	-76.1 (-81 to -70.2)
High income	Western Europe	Netherlands	YLLs	Age-standardized	7.8 (6.9 to 8.6)	0.2 (0.1 to 0.2)	19.2 (17.1 to 21.3)	1.1 (0.9 to 1.3)	0.1 (0.1 to 0.1)	2.3 (1.9 to 2.8)	-85.9 (-88.7 to -82.6)	-51.1 (-71.3 to -30.7)	-87.8 (-90.2 to -84.9)
High income	Western Europe	Netherlands	YLDs	All Ages	20 (13 to 28)	1 (1 to 1)	19 (12 to 27)	5 (3 to 8)	1 (1 to 2)	4 (3 to 6)	-73.7 (-80.6 to -63.4)	22.9 (4 to 51.7)	-78.3 (-84.3 to -69.1)
High income	Western Europe	Netherlands	YLDs	Age-standardized	0.1 (0.1 to 0.1)	0 (0 to 0)	0.2 (0.1 to 0.3)	0 (0 to 0)	0 (0 to 0)	0 (0 to 0)	-81.7 (-86.9 to -74.7)	-5.3 (-23.8 to 24.5)	-87.7 (-91 to -82.5)
High income	Western Europe	Norway	Incidence	All Ages	60 (49 to 73)	11 (8 to 13)	49 (39 to 62)	53 (45 to 63)	14 (11 to 17)	39 (32 to 48)	-11.2 (-23.2 to 1.2)	33.6 (24 to 44.4)	-20.9 (-33.1 to -7.7)
High income	Western Europe	Norway	Incidence	Age-standardized	1 (0.8 to 1.2)	0.4 (0.3 to 0.5)	1.7 (1.4 to 2.1)	0.7 (0.6 to 0.8)	0.4 (0.3 to 0.5)	1 (0.8 to 1.2)	-31.4 (-39.4 to -23.2)	2.9 (-3.4 to 10.4)	-42.6 (-50.8 to -33.8)
High income	Western Europe	Norway	Prevalence	All Ages	728 (587 to 895)	61 (48 to 76)	667 (530 to 831)	699 (571 to 861)	85 (67 to 107)	614 (496 to 772)	-3.9 (-17.6 to 12)	39.8 (30.4 to 53.4)	-7.9 (-22.5 to 9.2)
High income	Western Europe	Norway	Prevalence	Age-standardized	10.7 (8.7 to 13)	1.8 (1.4 to 2.2)	22.5 (18.1 to 27.8)	8.1 (6.6 to 10.1)	1.9 (1.5 to 2.3)	14.7 (12 to 18.4)	-24.3 (-35.1 to -12.3)	4.5 (-0.7 to 12)	-34.6 (-44.8 to -22.6)
High income	Western Europe	Norway	Deaths	All Ages	9 (9 to 10)	0 (0 to 0)	9 (8 to 10)	11 (10 to 13)	0 (0 to 0)	11 (9 to 12)	20.7 (5.3 to 39.7)	79.4 (-18 to 124.8)	19.3 (3.3 to 39.7)
High income	Western Europe	Norway	Deaths	Age-standardized	0.1 (0.1 to 0.1)	0 (0 to 0)	0.3 (0.3 to 0.3)	0.1 (0.1 to 0.1)	0 (0 to 0)	0.2 (0.2 to 0.3)	-12.4 (-23.9 to 1.5)	24.7 (-41.3 to 53.9)	-20.2 (-30.9 to -6.9)
High income	Western Europe	Norway	DALYs	All Ages	255 (220 to 300)	13 (9 to 18)	242 (210 to 282)	267 (227 to 318)	18 (13 to 26)	249 (211 to 294)	4.7 (-6.5 to 17.1)	43.9 (19.2 to 57.5)	2.6 (-9.2 to 15.6)
High income	Western Europe	Norway	DALYs	Age-standardized	3.6 (3.1 to 4.3)	0.4 (0.3 to 0.5)	8 (6.9 to 9.4)	2.9 (2.4 to 3.4)	0.4 (0.3 to 0.5)	5.6 (4.7 to 6.7)	-21.5 (-30.2 to -12.6)	7.6 (-8.8 to 16.3)	-29.6 (-37.6 to -20.9)
High income	Western Europe	Norway	YLLs	All Ages	153 (143 to 163)	3 (3 to 4)	150 (140 to 160)	164 (145 to 186)	5 (3 to 6)	159 (140 to 181)	6.7 (-8.4 to 24.6)	56.1 (-23.5 to 91.2)	5.7 (-9.3 to 24.3)
High income	Western Europe	Norway	YLLs	Age-standardized	2.1 (2 to 2.2)	0.1 (0.1 to 0.1)	4.9 (4.5 to 5.2)	1.6 (1.5 to 1.9)	0.1 (0.1 to 0.1)	3.5 (3 to 3.9)	-22.3 (-33.2 to -9.4)	17.2 (-37.7 to 41.4)	-29 (-39.1 to -16.5)
High income	Western Europe	Norway	YLDs	All Ages	102 (67 to 145)	10 (6 to 14)	92 (61 to 132)	103 (68 to 149)	13 (9 to 20)	90 (58 to 131)	1.6 (-12.9 to 18.4)	39.7 (30.3 to 53.3)	-2.3 (-18.1 to 15.6)
High income	Western Europe	Norway	YLDs	Age-standardized	1.5 (1 to 2.2)	0.3 (0.2 to 0.4)	3.1 (2.1 to 4.5)	1.2 (0.8 to 1.7)	0.3 (0.2 to 0.4)	2.2 (1.4 to 3.1)	-20.3 (-31.7 to -7.5)	4.4 (-0.8 to 11.9)	-30.6 (-41.6 to -17.5)
High income	Western Europe	Portugal	Incidence	All Ages	16 (13 to 19)	2 (1 to 3)	14 (11 to 17)	15 (12 to 19)	2 (1 to 4)	13 (10 to 16)	-6.7 (-23.5 to 15.5)	23.4 (-7.9 to 80)	-10.8 (-29 to 12.7)
High income	Western Europe	Portugal	Incidence	Age-standardized	0.1 (0.1 to 0.1)	0 (0 to 0)	0.2 (0.2 to 0.3)	0.1 (0.1 to 0.1)	0 (0 to 0)	0.1 (0.1 to 0.2)	-36.9 (-48.9 to -23)	-6.3 (-29.9 to 29.7)	-40.7 (-52 to -26)
High income	Western Europe	Portugal	Prevalence	All Ages	182 (145 to 225)	6 (4 to 8)	175 (139 to 218)	144 (112 to 186)	6 (4 to 11)	138 (106 to 180)	-20.5 (-37.7 to 3.5)	2.2 (-20.3 to 69.3)	-21.3 (-39.2 to 3.9)
High income	Western Europe	Portugal	Prevalence	Age-standardized	1.2 (1 to 1.5)	0.1 (0.1 to 0.1)	2.8 (2.2 to 3.5)	0.6 (0.5 to 0.8)	0.1 (0 to 0.1)	1.3 (1 to 1.7)	-52.7 (-62.7 to -38.7)	-28.6 (-45.1 to -6)	-53.8 (-63.9 to -39.7)
High income	Western Europe	Portugal	Deaths	All Ages	159 (142 to 177)	8 (6 to 10)	151 (134 to 169)	102 (85 to 123)	6 (4 to 9)	96 (78 to 116)	-35.7 (-47.9 to -20.2)	-18.5 (-43.4 to 14.6)	-36.6 (-49 to -20.4)
High income	Western Europe	Portugal	Deaths	Age-standardized	1.1 (1 to 1.2)	0.1 (0.1 to 0.1)	2.6 (2.3 to 2.9)	0.4 (0.3 to 0.5)	0 (0 to 0.1)	0.9 (0.7 to 1.1)	-65.2 (-71.5 to -57.3)	-58.7 (-71.1 to -43.7)	-65.7 (-72.3 to -57.8)
High income	Western Europe	Portugal	DALYs	All Ages	3,431 (3,053 to 3,823)	146 (112 to 198)	3,285 (2,913 to 3,677)	1,717 (1,444 to 2,044)	97 (62 to 147)	1,620 (1,354 to 1,962)	-50 (-59.3 to -38.5)	-33.8 (-53.4 to -8.1)	-50.7 (-60.5 to -39)
High income	Western Europe	Portugal	DALYs	Age-standardized	24.4 (21.8 to 27)	1.8 (1.4 to 2.5)	53.5 (47.6 to 59.3)	7.7 (6.4 to 9.3)	0.7 (0.5 to 1.2)	16.5 (13.7 to 19.9)	-68.5 (-74.5 to -61)	-59.9 (-71.5 to -43.5)	-69.2 (-75.3 to -61.9)
High income	Western Europe	Portugal	YLLs	All Ages	3,400 (3,028 to 3,793)	145 (111 to 197)	3,255 (2,886 to 3,631)	1,692 (1,419 to 2,022)	96 (60 to 145)	1,596 (1,328 to 1,941)	-50.2 (-59.7 to -38.6)	-34 (-53.8 to -8.4)	-51 (-60.8 to -39)
High income	Western Europe	Portugal	YLLs	Age-standardized	24.2 (21.6 to 26.8)	1.8 (1.4 to 2.5)	53 (47.2 to 58.8)	7.6 (6.3 to 9.2)	0.7 (0.5 to 1.2)	16.2 (13.5 to 19.7)	-68.7 (-74.7 to -61.1)	-60.2 (-71.9 to -43.7)	-69.4 (-75.5 to -62)
High income	Western Europe	Portugal	YLDs	All Ages	31 (20 to 45)	1 (1 to 2)	30 (19 to 43)	25 (16 to 37)	1 (1 to 2)	24 (15 to 36)	-19.8 (-37.2 to 3.9)	1.7 (-20.8 to 70.6)	-20.5 (-38.5 to 4.6)
High income	Western Europe	Portugal	YLDs	Age-standardized	0.2 (0.1 to 0.3)	0 (0 to 0)	0.5 (0.3 to 0.7)	0.1 (0.1 to 0.2)	0 (0 to 0)	0.2 (0.1 to 0.3)	-52.4 (-62.6 to -38.7)	-29.8 (-45.8 to -7.1)	-53.4 (-63.6 to -39.2)
High income	Western Europe	San Marino	Incidence	All Ages	0 (0 to 0)	0 (0 to 0)	0 (0 to 0)	0 (0 to 0)	0 (0 to 0)	0 (0 to 0)	76.7 (48.3 to 115.3)	99.1 (52.8 to 158)	56.2 (26.5 to 93.7)
High income	Western Europe	San Marino	Incidence	Age-standardized	0 (0 to 0.1)	0 (0 to 0.1)	0 (0 to 0.1)	0 (0 to 0.1)	0 (0 to 0.1)	0 (0 to 0.1)	-3.4 (-15.7 to 13.3)	-0.7 (-26.1 to 23.1)	-7 (-23.8 to 18.7)
High income	Western Europe	San Marino	Prevalence	All Ages	0 (0 to 0)	0 (0 to 0)	0 (0 to 0)	0 (0 to 0)	0 (0 to 0)	0 (0 to 0)	80.3 (55 to 106.5)	111 (76.4 to 152.1)	63.3 (34.5 to 95.7)
High income	Western Europe	San Marino	Prevalence	Age-standardized	0.1 (0.1 to 0.2)	0.1 (0.1 to 0.1)	0.2 (0.1 to 0.2)	0.1 (0.1 to 0.1)	0.1 (0.1 to 0.1)	0.1 (0.1 to 0.2)	-14.3 (-23.6 to -5.7)	-7.9 (-21.6 to 5.2)	-20.7 (-33.3 to -8.4)
High income	Western Europe	San Marino	Deaths	All Ages	0 (0 to 0)	0 (0 to 0)	0 (0 to 0)	0 (0 to 0)	0 (0 to 0)	0 (0 to 0)	85 (6.9 to 212)	113.3 (11.4 to 298)	56.3 (-16 to 184.3)
High income	Western Europe	San Marino	Deaths	Age-standardized	0.2 (0.1 to 0.3)	0.2 (0.1 to 0.3)	0.2 (0.2 to 0.3)	0.2 (0.1 to 0.3)	0.2 (0.1 to 0.3)	0.2 (0.1 to 0.3)	-18.2 (-52.3 to 39)	-5.2 (-50.6 to 79.5)	-30.9 (-62.4 to 22.3)
High income	Western Europe	San Marino	DALYs	All Ages	1 (1 to 1)	1 (0 to 1)	1 (0 to 1)	2 (1 to 3)	1 (1 to 2)	1 (0 to 1)	56.4 (-8.7 to 164.9)	84 (-2.8 to 253.2)	32.4 (-27.7 to 143.5)
High income	Western Europe	San Marino	DALYs	Age-standardized	3.3 (2.5 to 4.3)	2.8 (1.8 to 4)	4 (2.8 to 5.5)	2.7 (1.6 to 4.2)	2.7 (1.5 to 4.6)	2.6 (1.5 to 4.1)	-19.8 (-53.5 to 37.1)	-2.3 (-49.2 to 87.2)	-34.4 (-64 to 18.9)
High income	Western Europe	San Marino	YLLs	All Ages	1 (1 to 1)	1 (0 to 1)	1 (0 to 1)	2 (1 to 3)	1 (1 to 2)	1 (0 to 1)	56.2 (-9.3 to 165.5)	83.9 (-3 to 253.9)	32.2 (-28.1 to 144.6)
High income	Western Europe	San Marino	YLLs	Age-standardized	3.3 (2.5 to 4.3)	2.7 (1.8 to 4)	3.9 (2.8 to 5.4)	2.6 (1.6 to 4.1)	2.7 (1.4 to 4.6)	2.6 (1.5 to 4.1)	-19.8 (-53.7 to 37.5)	-2.3 (-49.4 to 87.7)	-34.5 (-64.5 to 19.2)
High income	Western Europe	San Marino	YLDs	All Ages	0 (0 to 0)	0 (0 to 0)	0 (0 to 0)	0 (0 to 0)	0 (0 to 0)	0 (0 to 0)	80.6 (54.5 to 107.2)	111.5 (78.1 to 152.5)	63.5 (34.6 to 95)
High income	Western Europe	San Marino	YLDs	Age-standardized	0 (0 to 0)	0 (0 to 0)	0 (0 to 0)	0 (0 to 0)	0 (0 to 0)	0 (0 to 0)	-14.9 (-24.1 to -6.4)	-8.4 (-21.6 to 3.7)	-21.3 (-33.6 to -9.2)
High income	Western Europe	Spain	Incidence	All Ages	54 (42 to 66)	6 (3 to 10)	48 (37 to 58)	76 (58 to 96)	12 (7 to 18)	63 (50 to 80)	41 (7.7 to 85.6)	100.8 (36.2 to 207.9)	33.3 (-1.6 to 79.8)
High income	Western Europe	Spain	Incidence	Age-standardized	0.1 (0.1 to 0.1)	0 (0 to 0)	0.2 (0.2 to 0.2)	0.1 (0.1 to 0.1)	0 (0 to 0.1)	0.1 (0.1 to 0.2)	-21.3 (-40 to -0.1)	21.7 (-9.5 to 82.3)	-24.2 (-43.9 to -0.7)
High income	Western Europe	Spain	Prevalence	All Ages	543 (424 to 691)	14 (9 to 20)	529 (411 to 676)	350 (279 to 433)	28 (20 to 38)	322 (252 to 402)	-35.5 (-54 to -9)	98.6 (58.6 to 153.9)	-39.1 (-57.3 to -13.3)
High income	Western Europe	Spain	Prevalence	Age-standardized	1 (0.7 to 1.2)	0.1 (0 to 0.1)	2.2 (1.7 to 2.8)	0.3 (0.3 to 0.4)	0.1 (0 to 0.1)	0.7 (0.5 to 0.9)	-67.1 (-75.9 to -54.9)	16.5 (-3.8 to 46.7)	-68.5 (-77.5 to -55.9)
High income	Western Europe	Spain	Deaths	All Ages	347 (314 to 381)	3 (2 to 4)	344 (311 to 378)	299 (243 to 363)	25 (15 to 36)	274 (221 to 335)	-13.9 (-30.9 to 6.3)	697.4 (445 to 1106)	-20.4 (-36.5 to -1.1)
High income	Western Europe	Spain	Deaths	Age-standardized	0.6 (0.6 to 0.7)	0 (0 to 0)	1.5 (1.3 to 1.6)	0.3 (0.2 to 0.3)	0 (0 to 0)	0.6 (0.5 to 0.7)	-59.1 (-66.8 to -50.1)	251.4 (142.8 to 415.2)	-60.8 (-68.5 to -51.7)
High income	Western Europe	Spain	DALYs	All Ages	7,354 (6,618 to 8,097)	52 (42 to 68)	7,301 (6,563 to 8,046)	3,994 (3,337 to 4,749)	304 (204 to 417)	3,690 (3,083 to 4,417)	-45.7 (-55.9 to -34.3)	481.6 (307.9 to 737.8)	-49.5 (-59.2 to -38.1)
High income	Western Europe	Spain	DALYs	Age-standardized	13.3 (12 to 14.7)	0.2 (0.1 to 0.2)	30.1 (27.3 to 33.1)	3.8 (3.3 to 4.6)	0.5 (0.4 to 0.7)	8.3 (6.9 to 9.9)	-71.2 (-76.5 to -65)	202.1 (117.8 to 323.6)	-72.6 (-77.7 to -66.6)
High income	Western Europe	Spain	YLLs	All Ages	7,262 (6,536 to 8,017)	50 (40 to 65)	7,212 (6,488 to 7,966)	3,934 (3,285 to 4,686)	299 (199 to 412)	3,635 (3,027 to 4,362)	-45.8 (-56.2 to -34.2)	499.6 (314.2 to 776.9)	-49.6 (-59.3 to -38.2)
High income	Western Europe	Spain	YLLs	Age-standardized	13.2 (11.9 to 14.5)	0.2 (0.1 to 0.2)	29.8 (27 to 32.8)	3.8 (3.2 to 4.5)	0.5 (0.4 to 0.7)	8.1 (6.8 to 9.7)	-71.3 (-76.6 to -64.9)	213 (122.9 to 347.3)	-72.6 (-77.8 to -66.7)
High income	Western Europe	Spain	YLDs	All Ages	92 (59 to 137)	2 (1 to 4)	90 (57 to 134)	60 (39 to 87)	5 (3 to 7)	55 (36 to 80)	-34.6 (-53 to -9)	100.8 (60.1 to 154.4)	-38.2 (-56.9 to -12.9)
High income	Western Europe	Spain	YLDs	Age-standardized	0.2 (0.1 to 0.2)	0 (0 to 0)	0.4 (0.2 to 0.6)	0.1 (0 to 0.1)	0 (0 to 0)	0.1 (0.1 to 0.2)	-66.8 (-75.7 to -54.8)	16.7 (-2.9 to 45.5)	-68.1 (-77.3 to -55.9)
High income	Western Europe	Sweden	Incidence	All Ages	28 (21 to 45)	6 (4 to 9)	22 (16 to 38)	27 (19 to 36)	8 (5 to 11)	19 (14 to 27)	-4.1 (-36.2 to 22.4)	22.2 (-4.2 to 52.6)	-11.9 (-47.1 to 18.7)
High income	Western Europe	Sweden	Incidence	Age-standardized	0.2 (0.1 to 0.3)	0.1 (0.1 to 0.2)	0.3 (0.2 to 0.5)	0.1 (0.1 to 0.2)	0.1 (0.1 to 0.2)	0.2 (0.1 to 0.3)	-23.9 (-48.3 to -6.6)	-2.8 (-25.3 to 23.5)	-38.5 (-61.8 to -20.1)
High income	Western Europe	Sweden	Prevalence	All Ages	126 (92 to 250)	13 (10 to 16)	113 (80 to 236)	70 (56 to 90)	17 (13 to 21)	53 (41 to 72)	-44.4 (-68.3 to -24.5)	32.6 (14.7 to 50.8)	-53.1 (-73.6 to -32.5)
High income	Western Europe	Sweden	Prevalence	Age-standardized	0.8 (0.6 to 1.4)	0.2 (0.1 to 0.3)	1.7 (1.2 to 3.5)	0.3 (0.3 to 0.4)	0.2 (0.1 to 0.3)	0.5 (0.4 to 0.7)	-54.6 (-74.2 to -40)	1.3 (-14.6 to 19.4)	-68.9 (-82.8 to -55.9)
High income	Western Europe	Sweden	Deaths	All Ages	24 (21 to 27)	0 (0 to 1)	23 (21 to 26)	22 (18 to 26)	1 (1 to 2)	21 (17 to 25)	-8.5 (-27.9 to 14.6)	180.5 (35.4 to 280.7)	-12.4 (-31.7 to 11)
High income	Western Europe	Sweden	Deaths	Age-standardized	0.1 (0.1 to 0.2)	0 (0 to 0)	0.3 (0.3 to 0.4)	0.1 (0.1 to 0.1)	0 (0 to 0)	0.2 (0.2 to 0.2)	-35.4 (-48.2 to -19.9)	148.2 (33.2 to 242.1)	-45.2 (-56.8 to -31.3)
High income	Western Europe	Sweden	DALYs	All Ages	385 (344 to 433)	10 (9 to 13)	375 (334 to 422)	310 (262 to 364)	30 (20 to 38)	279 (230 to 335)	-19.6 (-35.5 to -1.4)	190.9 (76.5 to 285.2)	-25.5 (-40.9 to -7.4)
High income	Western Europe	Sweden	DALYs	Age-standardized	2.4 (2.1 to 2.6)	0.1 (0.1 to 0.2)	5.5 (4.9 to 6.1)	1.4 (1.2 to 1.7)	0.4 (0.3 to 0.6)	2.7 (2.2 to 3.2)	-39 (-49.4 to -26.4)	195.5 (90 to 299.9)	-50.8 (-60.6 to -39.3)
High income	Western Europe	Sweden	YLLs	All Ages	365 (324 to 408)	8 (7 to 11)	356 (316 to 399)	298 (248 to 352)	28 (17 to 35)	270 (221 to 327)	-18.3 (-34.5 to 0.5)	229.9 (87.5 to 357.2)	-24.1 (-40.4 to -5.4)
High income	Western Europe	Sweden	YLLs	Age-standardized	2.2 (2 to 2.5)	0.1 (0.1 to 0.1)	5.2 (4.6 to 5.8)	1.4 (1.2 to 1.6)	0.4 (0.2 to 0.5)	2.6 (2.1 to 3.1)	-38.1 (-49 to -24.4)	251.7 (116.6 to 389.8)	-49.9 (-60.1 to -37.8)
High income	Western Europe	Sweden	YLDs	All Ages	20 (12 to 43)	2 (1 to 3)	18 (11 to 41)	12 (8 to 17)	3 (2 to 4)	9 (6 to 13)	-43.4 (-66.7 to -23.6)	32.9 (15.6 to 50.6)	-52 (-71.8 to -31.4)
High income	Western Europe	Sweden	YLDs	Age-standardized	0.1 (0.1 to 0.2)	0 (0 to 0)	0.3 (0.2 to 0.6)	0.1 (0 to 0.1)	0 (0 to 0.1)	0.1 (0.1 to 0.1)	-54 (-73 to -39.6)	1.3 (-14.2 to 19)	-68.3 (-81.7 to -55.7)
High income	Western Europe	Switzerland	Incidence	All Ages	8 (7 to 11)	2 (1 to 3)	7 (5 to 8)	8 (6 to 11)	2 (1 to 3)	6 (5 to 8)	-3.4 (-20.7 to 17.2)	29.2 (-3.6 to 65.9)	-11.1 (-30.1 to 12.3)
High income	Western Europe	Switzerland	Incidence	Age-standardized	0.1 (0.1 to 0.1)	0 (0 to 0.1)	0.2 (0.1 to 0.2)	0.1 (0 to 0.1)	0 (0 to 0.1)	0.1 (0.1 to 0.1)	-36 (-48.8 to -23.3)	-7 (-29.1 to 16.8)	-47.4 (-59.2 to -34.7)
High income	Western Europe	Switzerland	Prevalence	All Ages	59 (49 to 72)	6 (4 to 8)	54 (43 to 65)	43 (34 to 54)	9 (6 to 11)	35 (27 to 44)	-26.8 (-41.1 to -10)	46 (28.1 to 65.2)	-34.8 (-48.8 to -17.6)
High income	Western Europe	Switzerland	Prevalence	Age-standardized	0.5 (0.4 to 0.6)	0.1 (0.1 to 0.1)	1.2 (1 to 1.5)	0.2 (0.2 to 0.3)	0.1 (0.1 to 0.1)	0.4 (0.3 to 0.5)	-53.8 (-62.5 to -44)	-3.6 (-14.2 to 7.9)	-64.8 (-72.3 to -55.7)

Pneumoconiosis

7 GBD super region	21 GBD region	Location	Measure	Age	1990			2019			Percent change (1990 to 2019)		
					Both	Female	Male	Both	Female	Male	Both	Female	Male
High income	Western Europe	Switzerland	Deaths	All Ages	36 (29 to 43)	1 (1 to 1)	35 (28 to 42)	14 (11 to 17)	1 (1 to 1)	13 (10 to 16)	-61.2 (-70.7 to -47.4)	-13.3 (-42.5 to 33.2)	-62.5 (-71.8 to -48.2)
High income	Western Europe	Switzerland	Deaths	Age-standardized	0.3 (0.3 to 0.4)	0 (0 to 0)	0.8 (0.7 to 1)	0.1 (0.1 to 0.1)	0 (0 to 0)	0.2 (0.1 to 0.2)	-78 (-83.3 to -70.2)	-46.8 (-63.3 to -21.5)	-81 (-85.8 to -74)
High income	Western Europe	Switzerland	DALYs	All Ages	573 (467 to 686)	21 (15 to 27)	553 (447 to 663)	191 (157 to 231)	14 (11 to 19)	176 (144 to 217)	-66.7 (-74.5 to -55.7)	-29.9 (-51.5 to 1.7)	-68.1 (-75.8 to -57.2)
High income	Western Europe	Switzerland	DALYs	Age-standardized	5.2 (4.3 to 6.2)	0.4 (0.3 to 0.6)	12.5 (10.2 to 14.9)	1 (0.9 to 1.2)	0.2 (0.2 to 0.3)	2.1 (1.7 to 2.6)	-80.1 (-84.6 to -73.9)	-50.9 (-66.9 to -22.4)	-83.1 (-87.1 to -77.4)
High income	Western Europe	Switzerland	YLLs	All Ages	564 (457 to 674)	20 (14 to 26)	544 (439 to 654)	184 (151 to 224)	13 (9 to 17)	171 (138 to 210)	-67.4 (-75.1 to -56.4)	-33.5 (-54.6 to -1)	-68.6 (-76.4 to -57.5)
High income	Western Europe	Switzerland	YLLs	Age-standardized	5.1 (4.2 to 6.1)	0.4 (0.3 to 0.6)	12.3 (10 to 14.7)	1 (0.8 to 1.2)	0.2 (0.1 to 0.3)	2 (1.7 to 2.5)	-80.6 (-85 to -74.4)	-52.9 (-68.9 to -23.4)	-83.4 (-87.5 to -77.7)
High income	Western Europe	Switzerland	YLDs	All Ages	10 (6 to 14)	1 (1 to 1)	9 (6 to 13)	7 (5 to 10)	1 (1 to 2)	6 (4 to 8)	-27.6 (-41.9 to -11)	44.9 (27.6 to 62.9)	-35.4 (-49.4 to -18.5)
High income	Western Europe	Switzerland	YLDs	Age-standardized	0.1 (0.1 to 0.1)	0 (0 to 0)	0.2 (0.1 to 0.3)	0 (0 to 0.1)	0 (0 to 0)	0.1 (0 to 0.1)	-54.4 (-63 to -44.7)	-4.3 (-14.7 to 6.9)	-65.2 (-72.4 to -56.2)
High income	Western Europe	United Kingdom	Incidence	All Ages	922 (779 to 1,071)	165 (118 to 220)	756 (648 to 871)	1,442 (1,176 to 1,731)	85 (61 to 113)	1,357 (1,103 to 1,634)	56.5 (37.4 to 78.7)	-48.5 (-61.2 to -30.7)	79.5 (58 to 103.9)
High income	Western Europe	United Kingdom	Incidence	Age-standardized	1.1 (0.9 to 1.3)	0.4 (0.3 to 0.6)	2 (1.7 to 2.3)	1.1 (0.9 to 1.4)	0.1 (0.1 to 0.2)	2.3 (1.9 to 2.7)	5.7 (-8.2 to 21.4)	-65.1 (-75 to -50.9)	14.3 (0.8 to 28.5)
High income	Western Europe	United Kingdom	Prevalence	All Ages	6,679 (5,735 to 7,803)	1,019 (788 to 1,341)	5,660 (4,843 to 6,583)	8,777 (7,587 to 10,089)	523 (416 to 661)	8,254 (7,112 to 9,503)	31.4 (17.3 to 46.8)	-48.7 (-60.5 to -34.3)	45.8 (28.8 to 63.5)
High income	Western Europe	United Kingdom	Prevalence	Age-standardized	7.3 (6.3 to 8.5)	2.1 (1.6 to 2.7)	14.9 (12.8 to 17.2)	6.8 (5.9 to 7.7)	0.8 (0.7 to 1)	13.8 (12 to 15.8)	-6.5 (-16.1 to 4.4)	-60.7 (-69.1 to -49.9)	-7.2 (-17.3 to 3.7)
High income	Western Europe	United Kingdom	Deaths	All Ages	337 (320 to 350)	19 (16 to 25)	318 (302 to 331)	501 (446 to 554)	8 (5 to 9)	493 (439 to 547)	48.8 (34.9 to 65.1)	-59.1 (-73.8 to -50.7)	55.3 (40.2 to 73.9)
High income	Western Europe	United Kingdom	Deaths	Age-standardized	0.3 (0.3 to 0.4)	0 (0 to 0)	0.9 (0.8 to 0.9)	0.3 (0.3 to 0.4)	0 (0 to 0)	0.8 (0.7 to 0.9)	0.9 (-8.1 to 11.9)	-68.3 (-78.9 to -62.3)	-11.5 (-19.5 to -1.2)
High income	Western Europe	United Kingdom	DALYs	All Ages	6,460 (6,044 to 6,903)	486 (405 to 596)	5,974 (5,599 to 6,351)	7,891 (7,102 to 8,697)	208 (159 to 256)	7,683 (6,917 to 8,474)	22.1 (12.4 to 34)	-57.2 (-65.6 to -50)	28.6 (18.2 to 42.7)
High income	Western Europe	United Kingdom	DALYs	Age-standardized	6.8 (6.3 to 7.2)	1 (0.8 to 1.2)	15.7 (14.7 to 16.7)	5.8 (5.2 to 6.4)	0.3 (0.3 to 0.4)	12.5 (11.3 to 13.8)	-14.3 (-20.9 to -6.3)	-65.4 (-71 to -59.8)	-20.1 (-26.5 to -12)
High income	Western Europe	United Kingdom	YLLs	All Ages	5,474 (5,222 to 5,668)	331 (281 to 413)	5,143 (4,898 to 5,335)	6,605 (5,949 to 7,281)	125 (82 to 147)	6,480 (5,827 to 7,153)	20.7 (9.8 to 34.1)	-62 (-73.4 to -55)	26 (14.4 to 41.1)
High income	Western Europe	United Kingdom	YLLs	Age-standardized	5.7 (5.4 to 5.9)	0.7 (0.6 to 0.8)	13.5 (12.8 to 14)	4.8 (4.3 to 5.3)	0.2 (0.1 to 0.2)	10.5 (9.4 to 11.6)	-15.6 (-22.9 to -6.3)	-68.1 (-75.9 to -62.5)	-22.2 (-29 to -13.1)
High income	Western Europe	United Kingdom	YLDs	All Ages	986 (652 to 1,383)	156 (97 to 234)	831 (547 to 1,160)	1,285 (866 to 1,795)	82 (52 to 122)	1,203 (811 to 1,682)	30.3 (17.3 to 45.1)	-47 (-59.2 to -32.3)	44.7 (29 to 62.5)
High income	Western Europe	United Kingdom	YLDs	Age-standardized	1.1 (0.7 to 1.5)	0.3 (0.2 to 0.5)	2.2 (1.4 to 3)	1 (0.7 to 1.4)	0.1 (0.1 to 0.2)	2 (1.4 to 2.8)	-7.2 (-16.3 to 2.9)	-59.6 (-68.3 to -48.5)	-7.5 (-16.8 to 2.8)
		Southern Latin America	Incidence	All Ages	238 (197 to 285)	46 (32 to 60)	192 (157 to 236)	688 (569 to 841)	86 (63 to 111)	602 (486 to 755)	189 (159 to 222)	89.3 (58.9 to 128.3)	212.6 (177.4 to 251.2)
		Southern Latin America	Incidence	Age-standardized	0.5 (0.4 to 0.6)	0.2 (0.1 to 0.2)	0.9 (0.7 to 1.1)	0.8 (0.7 to 1)	0.2 (0.1 to 0.3)	1.6 (1.3 to 2)	65.6 (49.2 to 84.4)	13.6 (-6.3 to 37)	77.7 (58.3 to 99.3)
		Southern Latin America	Prevalence	All Ages	1,952 (1,588 to 2,427)	116 (95 to 142)	1,836 (1,475 to 2,309)	6,291 (5,049 to 7,905)	236 (193 to 288)	6,055 (4,799 to 7,667)	222.2 (180.6 to 266.9)	102.9 (79.2 to 132.5)	229.8 (185.5 to 277.3)
		Southern Latin America	Prevalence	Age-standardized	4.2 (3.4 to 5.2)	0.5 (0.4 to 0.6)	9.3 (7.5 to 11.7)	7.3 (5.9 to 9.2)	0.5 (0.4 to 0.6)	16.8 (13.3 to 21.3)	73.1 (51.9 to 96)	11.2 (-0.6 to 25.2)	79.9 (57.3 to 105.1)
		Southern Latin America	Deaths	All Ages	142 (127 to 157)	5 (4 to 7)	137 (122 to 153)	167 (143 to 200)	12 (8 to 15)	155 (133 to 188)	17.5 (-1.6 to 41.9)	129.9 (44.5 to 229.7)	13.3 (-6.1 to 39.3)
		Southern Latin America	Deaths	Age-standardized	0.3 (0.3 to 0.3)	0 (0 to 0)	0.7 (0.6 to 0.8)	0.2 (0.2 to 0.2)	0 (0 to 0)	0.4 (0.4 to 0.5)	-38.4 (-48.1 to -25.5)	11.2 (-29.4 to 58.5)	-38.1 (-48.5 to -23.6)
		Southern Latin America	DALYs	All Ages	3,345 (2,980 to 3,692)	114 (91 to 141)	3,230 (2,879 to 3,576)	3,773 (3,264 to 4,346)	219 (167 to 262)	3,554 (3,065 to 4,116)	12.8 (-4.4 to 33.6)	91.5 (42.1 to 152.3)	10 (-7.6 to 31.6)
		Southern Latin America	DALYs	Age-standardized	7.2 (6.4 to 7.9)	0.5 (0.4 to 0.6)	15.7 (14 to 17.4)	4.5 (3.9 to 5.2)	0.5 (0.4 to 0.6)	9.9 (8.5 to 11.4)	-37.5 (-46.7 to -25.9)	3.7 (-21.9 to 35.3)	-37.4 (-47.1 to -25.2)
		Southern Latin America	YLLs	All Ages	3,063 (2,728 to 3,411)	96 (74 to 124)	2,968 (2,629 to 3,319)	2,902 (2,512 to 3,450)	181 (131 to 224)	2,721 (2,336 to 3,278)	-5.3 (-19.9 to 14.5)	89.4 (32 to 166.2)	-8.3 (-23.5 to 12.3)
		Southern Latin America	YLLs	Age-standardized	6.6 (5.9 to 7.3)	0.4 (0.3 to 0.5)	14.4 (12.8 to 16)	3.5 (3 to 4.1)	0.4 (0.3 to 0.5)	7.6 (6.5 to 9.1)	-47.1 (-55.2 to -36.4)	2.2 (-27.3 to 42.2)	-47.5 (-56.2 to -36)
		Southern Latin America	YLDs	All Ages	281 (180 to 415)	19 (12 to 27)	263 (166 to 388)	871 (568 to 1,273)	38 (24 to 56)	833 (538 to 1,222)	209.4 (148.7 to 286.5)	102.5 (79.2 to 131.3)	217 (151.7 to 301.9)
		Southern Latin America	YLDs	Age-standardized	0.6 (0.4 to 0.9)	0.1 (0 to 0.1)	1.3 (0.8 to 2)	1 (0.7 to 1.5)	0.1 (0.1 to 0.1)	2.3 (1.5 to 3.4)	66.6 (34.2 to 106.5)	10.8 (-0.9 to 24.6)	72.9 (37.9 to 115.7)
High income	Southern Latin America	Argentina	Incidence	All Ages	102 (81 to 125)	27 (18 to 37)	75 (59 to 95)	279 (225 to 334)	65 (46 to 85)	213 (169 to 270)	172 (138.4 to 212.4)	140.5 (85.3 to 223.4)	183.3 (143.5 to 230.2)
High income	Southern Latin America	Argentina	Incidence	Age-standardized	0.3 (0.2 to 0.4)	0.2 (0.1 to 0.2)	0.5 (0.4 to 0.6)	0.5 (0.4 to 0.6)	0.2 (0.2 to 0.3)	0.9 (0.7 to 1.1)	68 (47.5 to 92.7)	48.6 (13.2 to 102.2)	74.2 (51.2 to 101.7)
High income	Southern Latin America	Argentina	Prevalence	All Ages	720 (559 to 918)	74 (59 to 90)	646 (489 to 841)	2,077 (1,626 to 2,683)	160 (130 to 193)	1,917 (1,474 to 2,529)	188.6 (150.5 to 233)	117.4 (84.1 to 160.1)	196.7 (153.1 to 248.9)
High income	Southern Latin America	Argentina	Prevalence	Age-standardized	2.2 (1.7 to 2.8)	0.4 (0.3 to 0.5)	4.7 (3.6 to 6.2)	3.7 (2.9 to 4.8)	0.5 (0.4 to 0.7)	8.2 (6.3 to 10.8)	67.3 (46 to 92.8)	26.8 (7.6 to 52.2)	73.2 (48.9 to 101.6)
High income	Southern Latin America	Argentina	Deaths	All Ages	29 (24 to 34)	3 (2 to 4)	26 (22 to 31)	36 (30 to 42)	8 (5 to 10)	28 (23 to 34)	24.4 (-3.9 to 60.2)	165.9 (46.1 to 353.4)	8.9 (-18.1 to 41.1)
High income	Southern Latin America	Argentina	Deaths	Age-standardized	0.1 (0.1 to 0.1)	0 (0 to 0)	0.2 (0.2 to 0.2)	0.1 (0.1 to 0.1)	0 (0 to 0)	0.1 (0.1 to 0.1)	-27.6 (-43.6 to -6.9)	36.9 (-23.2 to 130.3)	-34 (-49.9 to -14.6)
High income	Southern Latin America	Argentina	DALYs	All Ages	846 (717 to 993)	67 (48 to 92)	779 (654 to 923)	1,056 (880 to 1,276)	142 (109 to 177)	914 (756 to 1,122)	24.8 (-0.7 to 55.9)	112.9 (42.1 to 216.7)	17.3 (-8.2 to 48.3)
High income	Southern Latin America	Argentina	DALYs	Age-standardized	2.6 (2.2 to 3.1)	0.4 (0.3 to 0.5)	5.3 (4.5 to 6.3)	2 (1.7 to 2.4)	0.5 (0.4 to 0.6)	3.9 (3.2 to 4.8)	-24.2 (-39.4 to -5.7)	22.1 (-17.1 to 79.1)	-26.7 (-42.2 to -7.9)
High income	Southern Latin America	Argentina	YLLs	All Ages	739 (622 to 873)	55 (37 to 82)	684 (567 to 815)	765 (640 to 903)	117 (85 to 152)	648 (536 to 779)	3.5 (-20.2 to 33.4)	112.2 (29.6 to 244.2)	-5.2 (-28.2 to 22.7)
High income	Southern Latin America	Argentina	YLLs	Age-standardized	2.3 (1.9 to 2.7)	0.3 (0.2 to 0.5)	4.6 (3.8 to 5.5)	1.5 (1.2 to 1.7)	0.4 (0.3 to 0.5)	2.8 (2.3 to 3.3)	-36.1 (-50.6 to -18)	21.3 (-23.1 to 93.3)	-40.3 (-54.7 to -23.1)
High income	Southern Latin America	Argentina	YLDs	All Ages	107 (63 to 171)	12 (8 to 17)	95 (54 to 155)	291 (169 to 451)	25 (16 to 37)	265 (151 to 417)	172.3 (84.1 to 294)	116.5 (83.9 to 157.9)	179.2 (80.8 to 316.4)
High income	Southern Latin America	Argentina	YLDs	Age-standardized	0.3 (0.2 to 0.5)	0.1 (0 to 0.1)	0.7 (0.4 to 1.1)	0.5 (0.3 to 0.8)	0.1 (0.1 to 0.1)	1.1 (0.6 to 1.8)	58.7 (8.6 to 126.5)	26.2 (7 to 50.6)	63.7 (6.7 to 146.3)
High income	Southern Latin America	Chile	Incidence	All Ages	126 (104 to 152)	16 (11 to 22)	109 (90 to 135)	389 (313 to 491)	18 (13 to 25)	371 (294 to 474)	209.7 (164.5 to 263.7)	11.1 (-18.5 to 54.4)	239.4 (189.6 to 300.8)
High income	Southern Latin America	Chile	Incidence	Age-standardized	1.2 (1 to 1.5)	0.3 (0.2 to 0.3)	2.3 (1.9 to 2.9)	1.6 (1.3 to 2)	0.2 (0.1 to 0.2)	3.3 (2.6 to 4.2)	33.2 (14.5 to 55.7)	-41.8 (-56.1 to -21.7)	42.4 (21.5 to 67.4)
High income	Southern Latin America	Chile	Prevalence	All Ages	1,155 (938 to 1,427)	36 (29 to 47)	1,118 (904 to 1,388)	4,018 (3,168 to 5,129)	66 (50 to 100)	3,952 (3,105 to 5,061)	248 (187.7 to 317.2)	81.6 (43.2 to 137.2)	253.4 (191.4 to 326.8)
High income	Southern Latin America	Chile	Prevalence	Age-standardized	12 (9.8 to 14.8)	0.6 (0.5 to 0.8)	26.5 (21.4 to 32.9)	16.6 (13.1 to 21.2)	0.5 (0.4 to 0.8)	37.7 (29.6 to 48.2)	38.7 (15.7 to 65.5)	-20.1 (-35.6 to -1.8)	42 (18.4 to 69.7)
High income	Southern Latin America	Chile	Deaths	All Ages	112 (97 to 127)	2 (1 to 3)	110 (95 to 124)	130 (107 to 161)	4 (2 to 5)	126 (103 to 157)	15.7 (-7.8 to 47.8)	79.9 (18 to 163.2)	14.5 (-9.3 to 46.5)
High income	Southern Latin America	Chile	Deaths	Age-standardized	1.2 (1.1 to 1.4)	0 (0 to 0.1)	2.7 (2.4 to 3.1)	0.5 (0.4 to 0.7)	0 (0 to 0)	1.2 (1 to 1.5)	-55.7 (-64.5 to -43.7)	-35.8 (-57.1 to -7)	-54.7 (-64 to -42.1)
High income	Southern Latin America	Chile	DALYs	All Ages	2,453 (2,150 to 2,771)	42 (33 to 52)	2,410 (2,108 to 2,728)	2,642 (2,217 to 3,173)	67 (45 to 90)	2,575 (2,154 to 3,111)	7.7 (-13 to 34.9)	58.2 (13.1 to 116.2)	6.8 (-14.3 to 33.9)
High income	Southern Latin America	Chile	DALYs	Age-standardized	24.9 (21.8 to 28.1)	0.8 (0.6 to 1)	55 (48.2 to 62)	10.9 (9.2 to 13.1)	0.5 (0.3 to 0.7)	24.4 (20.5 to 29.4)	-56 (-64.4 to -45.3)	-35.8 (-52.9 to -13.3)	-55.6 (-64.2 to -44.6)
High income	Southern Latin America	Chile	YLLs	All Ages	2,289 (1,989 to 2,622)	37 (27 to 46)	2,253 (1,955 to 2,585)	2,089 (1,713 to 2,620)	57 (35 to 78)	2,033 (1,658 to 2,564)	-8.8 (-27.6 to 17.2)	54.4 (3.1 to 121.4)	-9.8 (-28.7 to 16.9)
High income	Southern Latin America	Chile	YLLs	Age-standardized	23.2 (20.2 to 26.5)	0.7 (0.5 to 0.9)	51.2 (44.6 to 58.4)	8.7 (7.1 to 10.8)	0.4 (0.3 to 0.6)	19.2 (15.7 to 24.2)	-62.7 (-70.2 to -52.3)	-38.2 (-57.1 to -12.2)	-62.5 (-70.2 to -51.7)
High income	Southern Latin America	Chile	YLDs	All Ages	163 (103 to 236)	6 (4 to 9)	157 (98 to 228)	553 (359 to 797)	11 (7 to 18)	543 (352 to 786)	238.8 (151.8 to 354.1)	82.3 (44.5 to 137)	244.6 (153.9 to 367.6)
High income	Southern Latin America	Chile	YLDs	Age-standardized	1.7 (1.1 to 2.5)	0.1 (0.1 to 0.2)	3.7 (2.3 to 5.4)	2.3 (1.5 to 3.3)	0.1 (0.1 to 0.1)	5.2 (3.4 to 7.5)	35 (1.1 to 79.2)	-20.2 (-35.5 to -2.1)	38.1 (3.4 to 83.7)
High income	Southern Latin America	Uruguay	Incidence	All Ages	10 (8 to 13)	2 (1 to 3)	8 (6 to 10)	20 (16 to 26)	3 (2 to 4)	17 (13 to 23)	101.8 (78.6 to 133)	36.3 (9.1 to 71.6)	119.9 (92.4 to 156.8)
High income	Southern Latin America	Uruguay	Incidence	Age-standardized	0.3 (0.2 to 0.3)	0.1 (0.1 to 0.2)	0.4 (0.3 to 0.6)	0.4 (0.3 to 0.5)	0.1 (0.1 to 0.2)	0.8 (0.6 to 1)	54.1 (36 to 78.5)	-1.9 (-23.4 to 25.1)	72.5 (51.8 to 99.7)
High income	Southern Latin America	Uruguay	Prevalence	All Ages	78 (58 to 104)	6 (5 to 8)	72 (52 to 97)	196 (146 to 263)	10 (8 to 12)	186 (136 to 253)	151 (117.8 to 194.9)	57.1 (35 to 87)	159.3 (122.7 to 208.5)
High income	Southern Latin America	Uruguay	Prevalence	Age-standardized	1.9 (1.5 to 2.5)	0.3 (0.2 to 0.4)	4.2 (3.1 to 5.7)	3.4 (2.5 to 4.6)	0.3 (0.3 to 0.4)	8 (5.8 to 10.8)	76.1 (53.4 to 106.4)	3.1 (-9.3 to 19.1)	87.7 (62.3 to 121.8)
High income	Southern Latin America	Uruguay	Deaths	All Ages	2 (1 to 2)	0 (0 to 0)	1 (1 to 2)	2 (2 to 2)	0 (0 to 1)	1 (1 to 2)	16.9 (-6.7 to 45.9)	155.2 (47.8 to 292.3)	0.3 (-21.5 to 27.8)
High income	Southern Latin America	Uruguay	Deaths	Age-standardized	0 (0 to 0)	0 (0 to 0)	0.1 (0.1 to 0.1)	0 (0 to 0)	0 (0 to 0)	0.1 (0.1 to 0.1)	-7.9 (-26.7 to 14.4)	56.5 (-11.2 to 130.4)	-20 (-37.6 to 1)
High income	Southern Latin America	Uruguay	DALYs	All Ages	46 (38 to 55)	5 (4 to 7)	41 (34 to 49)	75 (60 to 92)	10 (7 to 12)	65 (51 to 82)	62.2 (31 to 97.8)	88 (26.2 to 165.2)	59 (23.8 to 100.3)
High income	Southern Latin America	Uruguay	DALYs	Age-standardized	1.2 (1 to 1.4)	0.3 (0.2 to 0.4)	2.4 (2 to 2.9)	1.6 (1.3 to 2)	0.4 (0.3 to 0.5)	3.2 (2.5 to 3.9)	35.2 (8.7 to 65.5)	46.4 (-5.8 to 122.6)	31.2 (2.6 to 63.6)
High income	Southern Latin America	Uruguay	YLLs	All Ages	35 (29 to 41)	4 (3 to 6)	31 (26 to 36)	48 (40 to 56)	8 (5 to 11)	40 (33 to 48)	38.2 (8.5 to 72.2)	95.9 (20.9 to 198.3)	30.5 (-0.8 to 65.6)
High income	Southern Latin America	Uruguay	YLLs	Age-standardized	0.9 (0.8 to 1.1)	0.2 (0.2 to 0.3)	1.8 (1.5 to 2.1)	1.1 (0.9 to 1.4)	0.3 (0.2 to 0.5)	2.1 (1.7 to 2.5)	25.7 (-3.1 to 57.8)	56.3 (-7 to 151.1)	15.5 (-12.5 to 47.3)
High income	Southern Latin America	Uruguay	YLDs	All Ages	11 (7 to 18)	1 (1 to 2)	10 (6 to 16)	27 (15 to 43)	2 (1 to 2)	25 (14 to 41)	135.3 (58.3 to 241.1)	56.5 (34.8 to 85.5)	143.1 (58.5 to 262.7)
High income	Southern Latin America	Uruguay	YLDs	Age-standardized	0.3 (0.2 to 0.4)	0 (0 to 0.1)	0.6 (0.4 to 1)	0.5 (0.3 to 0.7)	0.1 (0 to 0.1)	1.1 (0.6 to 1.7)	66.1 (12.8 to 140.6)	2.6 (-10 to 18.4)	77 (17.5 to 165.1)
		High-income North America	Incidence	All Ages	8,073 (6,251 to 10,051)	2,168 (1,493 to 2,932)	5,905 (4,626 to 7,249)	11,368 (9,054 to 13,806)	2,107 (1,584 to 2,676)	9,261 (7,424 to 11,197)	40.8 (27.2 to 56.9)	-2.8 (-18.6 to 17.6)	56.8 (41.4 to 73.9)
		High-income North America	Incidence	Age-standardized	2.3 (1.8 to 2.9)	1.2 (0.8 to 1.6)	3.9 (3.1 to 4.8)	1.8 (1.5 to 2.2)	0.7 (0.5 to 0.8)	3.2 (2.6 to 3.9)	-22.5 (-30.2 to -13.2)	-42.9 (-53.2 to -29.8)	-18.2 (-26 to -9.6)
		High-income North America	Prevalence	All Ages	40,654 (34,460 to 48,023)	8,552 (6,972 to 10,557)	32,102 (27,012 to 37,875)	52,018 (45,759 to 59,068)	9,591 (8,104 to 11,274)	42,427 (37,249 to 48,157)	28 (14.2 to 41)	12.2 (-2.1 to 26.5)	32.2 (16.3 to 47.7)
		High-income North America	Prevalence	Age-standardized	11.5 (9.8 to 13.6)	4.5 (3.6 to 5.5)	21.3 (18 to 25.1)	8.3 (7.4 to 9.4)	3.1 (2.6 to 3.6)	14.8 (13.1 to 16.8)	-27.3 (-35.4 to -19.6)	-30.5 (-39.6 to -21.2)	-30.3 (-38.5 to -22.3)
		High-income North America	Deaths	All Ages	1,282 (1,211 to 1,345)	35 (31 to 47)	1,247 (1,176 to 1,310)	1,263 (1,135 to 1,354)	78 (49 to 90)	1,185 (1,070 to 1,274)	-1.5 (-8.1 to 4.9)	122.7 (26.1 to 162.3)	-5 (-11.9 to 1.6)
		High-income North America	Deaths	Age-standardized	0.3 (0.3 to 0.4)	0 (0 to 0)	0.9 (0.8 to 0.9)	0.2 (0.2 to 0.2)	0 (0 to 0)	0.4 (0.4 to 0.4)	-45.1 (-48.7 to -41.6)	34.4 (-19.8 to 56.4)	-51.8 (-55.2 to -48.6)
		High-income North America	DALYs	All Ages	27,746 (25,447 to 30,260)	1,993 (1,502 to 2,660)	25,753 (23,857 to 27,726)	26,041 (23,136 to 28,928)	2,853 (2,208 to 3,573)	23,188 (20,767 to 25,660)	-6.1 (-11.7 to -1.3)	43.2 (17.9 to 63.3)	-10 (-15.9 to -4.7)
		High-income North America	DALYs	Age-standardized	7.6 (7 to 8.3)	1 (0.8 to 1.4)	17.1 (15.8 to 18.4)	4.1 (3.6 to 4.6)	0.9 (0.7 to 1.2)	8.1 (7.2 to 8.9)	-46.3 (-49.4 to -43.5)	-10.1 (-24.8 to 2.4)	-52.7 (-55.8 to -49.9)
		High-income North America	YLLs	All Ages	21,760 (20,705 to 22,734)	653 (588 to 837)	21,107 (20,074 to 22,084)	18,449 (16,980 to 19,692)	1,343 (906 to 1,516)	17,107 (15,734 to 18,339)	-15.2 (-20.7 to -9.8)	105.7 (34.5 to 135.9)	-19 (-24.7 to -13.5)
		High-income North America	YLLs	Age-standardized	5.9 (5.6 to 6.2)	0.3 (0.3 to 0.4)	14 (13.3 to 14.6)	2.9 (2.6 to 3.1)	0.4 (0.3 to 0.5)	6 (5.5 to 6.4)	-51.6 (-54.5 to -48.5)	32.1 (-8.2 to 50.6)	-57.5 (-60.3 to -54.7)
		High-income North America	YLDs	All Ages	5,986 (4,030 to 8,508)	1,340 (869 to 1,965)	4,646 (3,131 to 6,599)	7,592 (5,250 to 10,557)	1,511 (1,011 to 2,167)	6,081 (4,218 to 8,464)	26.8 (13.1 to 40)	12.7 (-1.1 to 27)	30.9 (14.8 to 46.9)
		High-income North America	YLDs	Age-standardized	1.7 (1.1 to 2.4)	0.7 (0.5 to 1)	3.1 (2.1 to 4.4)	1.2 (0.8 to 1.7)	0.5 (0.3 to 0.7)	2.1 (1.5 to 3)	-28.1 (-36 to -20.5)	-30.2 (-39.3 to -20.5)	-31 (-39.4 to -22.7)
High income	High-income North America	Canada	Incidence	All Ages	770 (576 to 974)	275 (206 to 349)	495 (368 to 634)	1,352 (1,027 to 1,695)	431 (313 to 561)	921 (703 to 1,165)	75.5 (61.5 to 91.3)	56.4 (39.3 to 72.7)	86.1 (63.5 to 111.6)
High income	High-income North America	Canada	Incidence	Age-standardized	2.4 (1.8 to 3)	1.6 (1.2 to 2)	3.4 (2.6 to 4.3)	2 (1.6 to 2.5)	1.4 (1 to 1.7)	2.8 (2.2 to 3.6)	-13.8 (-20.4 to -6.5)	-15.4 (-24.4 to -6.9)	-16.1 (-26 to -5.7)
High income	High-income North America	Canada	Prevalence	All Ages	3,069 (2,538 to 3,723)	867 (679 to 1,113)	2,202 (1,832 to 2,658)	5,705 (4,761 to 6,855)	1,367 (1,086 to 1,748)	4,338 (3,610 to 5,178)	85.9 (70.1 to 103.9)	57.7 (43.8 to 72.3)	97 (77 to 121.3)
High income	High-income North America	Canada	Prevalence	Age-standardized	9.3 (7.7 to 11.2)	4.8 (3.8 to 6.1)	15.5 (13 to 18.5)	8.3 (7 to 9.9)	4 (3.2 to 5)	13.4 (11.3 to 16.1)	-10.6 (-17.8 to -2.8)	-16.5 (-23.1 to -8.8)	-13.4 (-22.3 to -3)
High income	High-income North America	Canada	Deaths	All Ages	54 (47 to 60)	1 (1 to 1)	53 (46 to 59)	130 (106 to 159)	4 (3 to 5)	127 (103 to 155)	143.4 (93.9 to 201.9)	284 (168.6 to 427.6)	140.9 (90.2 to 200.8)
High income	High-income North America	Canada	Deaths	Age-standardized	0.2 (0.1 to 0.2)	0 (0 to 0)	0.4 (0.4 to 0.5)	0.2 (0.1 to 0.2)	0 (0 to 0)	0.4 (0.3 to 0.5)	7.8 (-13.7 to 33.3)	116.6 (58.4 to 193.3)	-2.2 (-22.3 to 21.8)
High income	High-income North America	Canada	DALYs	All Ages	1,427 (1,225 to 1,672)	161 (110 to 231)	1,266 (1,103 to 1,452)	2,754 (2,307 to 3,291)	305 (226 to 410)	2,449 (2,050 to 2,921)	92.9 (65.3 to 125.1)	88.9 (66.4 to 118.9)	93.4 (62.8 to 130.4)
High income	High-income North America	Canada	DALYs	Age-standardized	4.3 (3.7 to 5)	0.9 (0.6 to 1.3)	8.9 (7.8 to 10.2)	4 (3.3 to 4.7)	1 (0.7 to 1.3)	7.6 (6.4 to 9)	-7.9 (-20.4 to 7)	9.7 (-5.2 to 31.1)	-15.1 (-28.2 to 0.4)
High income	High-income North America	Canada	YLLs	All Ages	966 (858 to 1,086)	25 (20 to 32)	941 (832 to 1,062)	1,909 (1,578 to 2,271)	90 (64 to 116)	1,819 (1,493 to 2,191)	97.6 (58.6 to 145.7)	259.5 (156.9 to 394.3)	93.3 (53.5 to 140.8)
High income	High-income North America	Canada	YLLs	Age-standardized	2.9 (2.6 to 3.3)	0.2 (0.1 to 0.2)	6.7 (5.9 to 7.5)	2.7 (2.3 to 3.2)	0.4 (0.3 to 0.5)	5.6 (4.7 to 6.8)	-6.1 (-24 to 15.6)	140.7 (71.7 to 237.1)	-15.3 (-32.1 to 4.3)
High income	High-income North America	Canada	YLDs	All Ages	462 (302 to 681)	136 (86 to 206)	325 (210 to 480)	845 (564 to 1,224)	215 (139 to 318)	630 (407 to 917)	83.1 (55.7 to 121.2)	57.6 (43.9 to 72.3)	93.8 (53.3 to 147.4)
High income	High-income North America	Canada	YLDs	Age-standardized	1.4 (0.9 to 2.1)	0.8 (0.5 to 1.1)	2.3 (1.5 to 3.4)	1.2 (0.8 to 1.8)	0.6 (0.4 to 0.9)	2 (1.3 to 2.8)	-11.7 (-24.5 to 5.6)	-16.5 (-23.1 to -8.9)	-14.5 (-31.3 to 8.5)
High income	High-income North America	Greenland	Incidence	All Ages	1 (1 to 1)	0 (0 to 1)	1 (0 to 1)	2 (1 to 2)	1 (0 to 1)	1 (1 to 1)	56.5 (40.5 to 71.8)	39.1 (23.2 to 55.7)	69.6 (48.1 to 91.3)
High income	High-income North America	Greenland	Incidence	Age-standardized	3.2 (2.4 to 4)	2.4 (1.8 to 3)	4.2 (3.1 to 5.5)	2.5 (1.9 to 3.1)	2.1 (1.6 to 2.6)	2.9 (2.1 to 3.7)	-20.8 (-27.2 to -14.3)	-14.4 (-21 to -7)	-31.1 (-39.9 to -23)
High income	High-income North America	Greenland	Prevalence	All Ages	3 (3 to 4)	1 (1 to 2)	2 (2 to 3)	6 (4 to 7)	2 (1 to 2)	4 (3 to 5)	63.7 (49.3 to 79.4)	46.1 (31.2 to 62.3)	74.6 (53 to 96.7)
High income	High-income North America	Greenland	Prevalence	Age-standardized	10.9 (8.5 to 13.9)	7.3 (5.5 to 9.5)	16 (12.4 to 20.3)	8.5 (6.7 to 10.7)	6.1 (4.7 to 7.9)	10.8 (8.4 to 13.7)	-22.2 (-29 to -15.2)	-16.6 (-24 to -7.3)	-32.5 (-40.9 to -23.5)
High income	High-income North America	Greenland	Deaths	All Ages	0 (0 to 0)	0 (0 to 0)	0 (0 to 0)	0 (0 to 0)	0 (0 to 0)	0 (0 to 0)	-2.1 (-34.1 to 41.1)	164.1 (61.6 to 339.6)	-5.3 (-37.1 to 38)
High income	High-income North America	Greenland	Deaths	Age-standardized	0.1 (0.1 to 0.2)	0 (0 to 0)	0.4 (0.3 to 0.5)	0.1 (0 to 0.1)	0 (0 to 0)	0.1 (0.1 to 0.2)	-57.8 (-71.4 to -39.3)	19.9 (-25.5 to 98)	-64.8 (-75.8 to -48.8)
High income	High-income North America	Greenland	DALYs	All Ages	1 (1 to 1)	0 (0 to 0)	1 (1 to 1)	1 (1 to 2)	0 (0 to 0)	1 (1 to 1)	20.3 (-4.7 to 47.6)	53.7 (24.9 to 89.9)	12.9 (-14.5 to 44.1)
High income	High-income North America	Greenland	DALYs	Age-standardized	3.9 (3.1 to 4.9)	1.2 (0.7 to 1.7)	7.7 (6 to 9.6)	2.2 (1.7 to 2.8)	1 (0.7 to 1.5)	3.3 (2.6 to 4.2)	-44.7 (-56.8 to -31.6)	-12 (-31 to 13.6)	-56.5 (-67.3 to -44.1)
High income	High-income North America	Greenland	YLLs	All Ages	1 (0 to 1)	0 (0 to 0)	1 (0 to 1)	1 (0 to 1)	0 (0 to 0)	1 (0 to 1)	-12 (-41.6 to 26.3)	192 (81.8 to 358.1)	-16.1 (-44.8 to 21.4)
High income	High-income North America	Greenland	YLLs	Age-standardized	2.4 (1.8 to 3.1)	0.1 (0.1 to 0.1)	5.4 (4.1 to 7.1)	1 (0.7 to 1.2)	0.1 (0.1 to 0.2)	1.8 (1.4 to 2.3)	-59.6 (-73 to -42.1)	50.9 (-4.9 to 139.3)	-66.7 (-77.7 to -51.5)
High income	High-income North America	Greenland	YLDs	All Ages	0 (0 to 1)	0 (0 to 0)	0 (0 to 0)	1 (1 to 1)	0 (0 to 0)	1 (0 to 1)	62.3 (35.3 to 94.4)	44.7 (15.6 to 82)	73.6 (34.1 to 127.1)
High income	High-income North America	Greenland	YLDs	Age-standardized	1.6 (1 to 2.3)	1.1 (0.7 to 1.7)	2.2 (1.4 to 3.3)	1.2 (0.8 to 1.8)	0.9 (0.6 to 1.4)	1.5 (1 to 2.3)	-21.9 (-36.2 to -3.4)	-16.8 (-36.3 to 9.7)	-31.7 (-48.5 to -7.5)
High income	High-income North America	United States of America	Incidence	All Ages	7,302 (5,679 to 9,084)	1,892 (1,285 to 2,586)	5,410 (4,264 to 6,645)	10,014 (7,999 to 12,101)	1,676 (1,272 to 2,146)	8,339 (6,692 to 10,121)	37.2 (22.8 to 54.8)	-11.4 (-28.4 to 11.5)	54.1 (38 to 71.8)
High income	High-income North America	United States of America	Incidence	Age-standardized	2.3 (1.8 to 2.9)	1.1 (0.8 to 1.6)	4 (3.1 to 4.8)	1.8 (1.4 to 2.1)	0.6 (0.5 to 0.7)	3.2 (2.6 to 3.9)	-23.6 (-32 to -13.3)	-47.8 (-59 to -32.8)	-18.3 (-26.6 to -8.9)
High income	High-income North America	United States of America	Prevalence	All Ages	37,580 (31,804 to 44,364)	7,683 (6,244 to 9,547)	29,897 (25,165 to 35,319)	46,307 (40,897 to 52,301)	8,222 (6,962 to 9,777)	38,085 (33,553 to 43,057)	23.2 (9 to 37.1)	7 (-7.7 to 23.3)	27.4 (11.3 to 43.6)
High income	High-income North America	United States of America	Prevalence	Age-standardized	11.7 (9.9 to 13.8)	4.4 (3.6 to 5.5)	21.9 (18.5 to 25.9)	8.3 (7.4 to 9.4)	3 (2.5 to 3.5)	15 (13.2 to 16.9)	-28.8 (-37.1 to -20.7)	-32.6 (-42.4 to -21.7)	-31.5 (-39.9 to -22.9)
High income	High-income North America	United States of America	Deaths	All Ages	1,229 (1,158 to 1,291)	34 (30 to 46)	1,194 (1,124 to 1,256)	1,133 (1,026 to 1,217)	75 (46 to 86)	1,058 (959 to 1,137)	-7.8 (-13.9 to -1.9)	118.3 (22.2 to 157.3)	-11.4 (-17.6 to -5.1)
High income	High-income North America	United States of America	Deaths	Age-standardized	0.4 (0.3 to 0.4)	0 (0 to 0)	0.9 (0.9 to 1)	0.2 (0.2 to 0.2)	0 (0 to 0)	0.4 (0.4 to 0.4)	-47.3 (-50.7 to -44)	33.8 (-21.6 to 56)	-54 (-57.1 to -50.8)
High income	High-income North America	United States of America	DALYs	All Ages	26,317 (24,189 to 28,616)	1,832 (1,381 to 2,420)	24,486 (22,717 to 26,388)	23,286 (20,620 to 25,983)	2,548 (1,991 to 3,159)	20,738 (18,548 to 22,978)	-11.5 (-17 to -6.7)	39.1 (12.7 to 59.9)	-15.3 (-21 to -10.4)
High income	High-income North America	United States of America	DALYs	Age-standardized	8 (7.3 to 8.7)	1 (0.8 to 1.4)	17.9 (16.6 to 19.3)	4.1 (3.6 to 4.6)	0.9 (0.7 to 1.1)	8.1 (7.3 to 9)	-48.4 (-51.6 to -45.6)	-11.9 (-27.8 to 0.9)	-54.5 (-57.6 to -51.9)
High income	High-income North America	United States of America	YLLs	All Ages	20,793 (19,762 to 21,759)	628 (566 to 806)	20,165 (19,173 to 21,109)	16,540 (15,297 to 17,643)	1,252 (832 to 1,409)	15,287 (14,125 to 16,350)	-20.5 (-25.6 to -15.3)	99.5 (27.3 to 128.9)	-24.2 (-29.6 to -19.1)
High income	High-income North America	United States of America	YLLs	Age-standardized	6.2 (5.9 to 6.5)	0.4 (0.3 to 0.4)	14.8 (14 to 15.4)	2.9 (2.7 to 3.1)	0.5 (0.3 to 0.5)	6 (5.5 to 6.4)	-53.6 (-56.5 to -50.7)	28.1 (-13 to 45.2)	-59.4 (-62.2 to -56.6)
High income	High-income North America	United States of America	YLDs	All Ages	5,524 (3,717 to 7,850)	1,204 (773 to 1,786)	4,320 (2,919 to 6,136)	6,746 (4,681 to 9,341)	1,296 (867 to 1,861)	5,450 (3,808 to 7,563)	22.1 (8 to 36)	7.6 (-7.1 to 23.9)	26.2 (9.8 to 42.7)
High income	High-income North America	United States of America	YLDs	Age-standardized	1.7 (1.2 to 2.5)	0.7 (0.4 to 1)	3.2 (2.1 to 4.5)	1.2 (0.8 to 1.7)	0.5 (0.3 to 0.7)	2.2 (1.5 to 3)	-29.5 (-37.7 to -21.5)	-32.2 (-42.1 to -21.3)	-32.2 (-40.9 to -23.5)
		Latin America and Caribbean	Incidence	All Ages	2,944 (2,580 to 3,398)	558 (473 to 657)	2,386 (2,073 to 2,789)	5,729 (5,003 to 6,587)	1,375 (1,165 to 1,627)	4,353 (3,781 to 5,056)	94.6 (83.9 to 105.4)	146.5 (128.5 to 163.1)	82.4 (72.4 to 92.8)
		Latin America and Caribbean	Incidence	Age-standardized	1.1 (0.9 to 1.2)	0.4 (0.3 to 0.5)	1.7 (1.5 to 2)	0.9 (0.8 to 1.1)	0.4 (0.4 to 0.5)	1.5 (1.3 to 1.7)	-10.9 (-14.9 to -6.8)	5.7 (1 to 10.7)	-13.9 (-18 to -9.6)
		Latin America and Caribbean	Prevalence	All Ages	38,473 (32,906 to 45,704)	5,871 (4,971 to 6,856)	32,601 (27,485 to 39,422)	83,330 (71,324 to 98,168)	16,001 (13,545 to 18,897)	67,329 (56,719 to 80,503)	116.6 (104.7 to 128.2)	172.5 (159.1 to 187.1)	106.5 (94.1 to 118.3)
		Latin America and Caribbean	Prevalence	Age-standardized	15.6 (13.3 to 18.6)	4.8 (4 to 5.6)	27.4 (23.1 to 32.9)	13.9 (12 to 16.3)	5 (4.3 to 6)	24.1 (20.3 to 28.6)	-11 (-15.5 to -6.7)	5.9 (1.6 to 10.1)	-12.4 (-17.4 to -7.7)
		Latin America and Caribbean	Deaths	All Ages	564 (528 to 599)	77 (65 to 90)	487 (455 to 518)	905 (818 to 991)	190 (155 to 226)	715 (647 to 787)	60.5 (46.1 to 77.1)	146.4 (108.1 to 190.8)	46.9 (32.5 to 62.8)
		Latin America and Caribbean	Deaths	Age-standardized	0.3 (0.2 to 0.3)	0.1 (0.1 to 0.1)	0.5 (0.4 to 0.5)	0.2 (0.1 to 0.2)	0.1 (0 to 0.1)	0.3 (0.2 to 0.3)	-39.9 (-45.3 to -33.8)	-16.9 (-29.7 to -2.2)	-42.3 (-48 to -36.4)
		Latin America and Caribbean	DALYs	All Ages	21,256 (19,144 to 23,872)	2,811 (2,372 to 3,314)	18,445 (16,635 to 20,696)	33,559 (29,040 to 39,022)	6,073 (5,060 to 7,254)	27,486 (23,907 to 31,934)	57.9 (45.9 to 70.7)	116 (93.5 to 142.4)	49 (36.9 to 61.7)
		Latin America and Caribbean	DALYs	Age-standardized	8.5 (7.7 to 9.5)	2.2 (1.9 to 2.6)	15.3 (13.9 to 17.1)	5.6 (4.9 to 6.5)	1.9 (1.6 to 2.3)	9.8 (8.6 to 11.4)	-34 (-39 to -28.7)	-13.4 (-22.1 to -3.2)	-35.9 (-41.1 to -30.5)
		Latin America and Caribbean	YLLs	All Ages	15,595 (14,582 to 16,518)	1,898 (1,630 to 2,209)	13,697 (12,822 to 14,555)	21,289 (19,383 to 23,246)	3,619 (3,087 to 4,282)	17,670 (16,006 to 19,390)	36.5 (23.2 to 51.3)	90.7 (61.3 to 127.8)	29 (15.5 to 43.3)
		Latin America and Caribbean	YLLs	Age-standardized	6.2 (5.8 to 6.6)	1.5 (1.3 to 1.7)	11.4 (10.7 to 12.1)	3.6 (3.2 to 3.9)	1.1 (1 to 1.4)	6.3 (5.7 to 6.9)	-42.7 (-48.2 to -36.7)	-22.5 (-34.4 to -7.7)	-44.4 (-49.9 to -38.4)
		Latin America and Caribbean	YLDs	All Ages	5,661 (3,724 to 8,100)	913 (607 to 1,307)	4,748 (3,110 to 6,794)	12,269 (8,248 to 17,408)	2,453 (1,629 to 3,520)	9,816 (6,513 to 14,017)	116.7 (102.5 to 132.4)	168.8 (154.8 to 184.4)	106.7 (90.7 to 123.2)
		Latin America and Caribbean	YLDs	Age-standardized	2.3 (1.5 to 3.2)	0.7 (0.5 to 1.1)	4 (2.6 to 5.7)	2 (1.4 to 2.9)	0.8 (0.5 to 1.1)	3.5 (2.3 to 5)	-10.3 (-15.9 to -4.5)	4.8 (0 to 9.5)	-11.6 (-17.9 to -5)
		Caribbean	Incidence	All Ages	31 (23 to 41)	15 (11 to 21)	15 (12 to 20)	58 (44 to 74)	30 (22 to 39)	28 (21 to 36)	87.3 (69.5 to 107.6)	93.4 (70.4 to 123.5)	81.2 (61.6 to 101.8)
		Caribbean	Incidence	Age-standardized	0.1 (0.1 to 0.1)	0.1 (0.1 to 0.1)	0.1 (0.1 to 0.1)	0.1 (0.1 to 0.1)	0.1 (0.1 to 0.2)	0.1 (0.1 to 0.1)	8.1 (0.3 to 16.8)	13 (0.9 to 26.8)	4.1 (-4.4 to 12.7)
		Caribbean	Prevalence	All Ages	167 (135 to 206)	61 (49 to 76)	105 (83 to 134)	351 (281 to 440)	129 (101 to 163)	223 (174 to 286)	110.9 (93.9 to 128)	109.8 (89.1 to 132.6)	111.6 (92.3 to 132.2)
		Caribbean	Prevalence	Age-standardized	0.6 (0.5 to 0.8)	0.4 (0.4 to 0.6)	0.8 (0.6 to 1)	0.7 (0.5 to 0.9)	0.5 (0.4 to 0.6)	0.9 (0.7 to 1.2)	10.3 (2.6 to 18.9)	6.4 (-3.3 to 16.6)	14.2 (4.1 to 24.9)
		Caribbean	Deaths	All Ages	18 (12 to 23)	5 (3 to 6)	13 (7 to 19)	21 (14 to 31)	3 (2 to 5)	18 (10 to 28)	18.5 (-9.7 to 55.3)	-26.3 (-51 to 19.4)	34 (-1.9 to 77.8)
		Caribbean	Deaths	Age-standardized	0.1 (0 to 0.1)	0 (0 to 0)	0.1 (0.1 to 0.2)	0 (0 to 0.1)	0 (0 to 0)	0.1 (0 to 0.1)	-41.7 (-55.8 to -23.6)	-65.3 (-76.9 to -43)	-31.1 (-49.1 to -9.5)
		Caribbean	DALYs	All Ages	458 (302 to 611)	111 (84 to 144)	348 (188 to 503)	563 (375 to 821)	108 (75 to 163)	455 (267 to 721)	22.8 (-5.6 to 61.4)	-2.3 (-28.9 to 40.5)	30.8 (-4.2 to 78.3)
		Caribbean	DALYs	Age-standardized	1.6 (1.1 to 2.2)	0.8 (0.6 to 1)	2.5 (1.4 to 3.6)	1.1 (0.7 to 1.6)	0.4 (0.3 to 0.6)	1.9 (1.1 to 3)	-32.1 (-47.8 to -9.8)	-47.5 (-62.1 to -25.4)	-26 (-45.5 to 0.3)
		Caribbean	YLLs	All Ages	432 (277 to 585)	101 (75 to 136)	331 (173 to 486)	507 (321 to 773)	88 (58 to 141)	419 (230 to 684)	17.4 (-12.9 to 57.3)	-13.1 (-41.4 to 32.7)	26.7 (-9.2 to 76.6)
		Caribbean	YLLs	Age-standardized	1.5 (1 to 2.1)	0.7 (0.5 to 1)	2.4 (1.3 to 3.5)	1 (0.6 to 1.5)	0.3 (0.2 to 0.5)	1.7 (1 to 2.8)	-34.8 (-51.4 to -11.8)	-52.8 (-68.4 to -28.6)	-28.1 (-47.9 to -0.5)
		Caribbean	YLDs	All Ages	26 (17 to 39)	10 (6 to 15)	17 (11 to 25)	56 (36 to 84)	20 (13 to 31)	35 (22 to 54)	110.9 (93.9 to 127.7)	109.7 (89.1 to 132.1)	111.6 (92.4 to 131.9)
		Caribbean	YLDs	Age-standardized	0.1 (0.1 to 0.1)	0.1 (0 to 0.1)	0.1 (0.1 to 0.2)	0.1 (0.1 to 0.2)	0.1 (0 to 0.1)	0.1 (0.1 to 0.2)	10.2 (2.6 to 18.7)	6.2 (-3.5 to 16.2)	14.1 (4 to 24.6)
Latin America and Caribbean	Caribbean	Antigua and Barbuda	Incidence	All Ages	0 (0 to 0)	0 (0 to 0)	0 (0 to 0)	0 (0 to 0)	0 (0 to 0)	0 (0 to 0)	39.9 (18.7 to 62.6)	42.4 (9 to 91.9)	38.6 (13.6 to 67.7)
Latin America and Caribbean	Caribbean	Antigua and Barbuda	Incidence	Age-standardized	0.1 (0.1 to 0.2)	0.1 (0.1 to 0.1)	0.2 (0.2 to 0.3)	0.1 (0.1 to 0.1)	0.1 (0.1 to 0.1)	0.2 (0.1 to 0.2)	-20.2 (-32 to -8.3)	-10.9 (-31.6 to 16)	-27.4 (-40.1 to -12.8)
Latin America and Caribbean	Caribbean	Antigua and Barbuda	Prevalence	All Ages	1 (0 to 1)	0 (0 to 0)	0 (0 to 1)	1 (1 to 1)	0 (0 to 0)	1 (0 to 1)	42.6 (20.2 to 70)	67.5 (36.7 to 102.6)	35.9 (9.5 to 70.1)
Latin America and Caribbean	Caribbean	Antigua and Barbuda	Prevalence	Age-standardized	1.1 (0.8 to 1.3)	0.4 (0.3 to 0.5)	2 (1.5 to 2.5)	0.9 (0.7 to 1.1)	0.4 (0.3 to 0.5)	1.4 (1.1 to 1.8)	-16.8 (-29.4 to -1.1)	5.6 (-13.8 to 26.4)	-28.1 (-41 to -10.7)
Latin America and Caribbean	Caribbean	Antigua and Barbuda	Deaths	All Ages	0 (0 to 0)	0 (0 to 0)	0 (0 to 0)	0 (0 to 0)	0 (0 to 0)	0 (0 to 0)	-7.9 (-31.4 to 24.8)	-40.6 (-63.3 to -6.4)	-1.2 (-29.3 to 37.4)
Latin America and Caribbean	Caribbean	Antigua and Barbuda	Deaths	Age-standardized	0.1 (0.1 to 0.2)	0 (0 to 0.1)	0.3 (0.3 to 0.4)	0.1 (0.1 to 0.1)	0 (0 to 0)	0.2 (0.1 to 0.2)	-39.8 (-55.2 to -18.7)	-57.6 (-73.2 to -33.7)	-45.1 (-60.8 to -23.5)
Latin America and Caribbean	Caribbean	Antigua and Barbuda	DALYs	All Ages	2 (1 to 2)	0 (0 to 0)	1 (1 to 2)	2 (1 to 2)	0 (0 to 0)	1 (1 to 2)	0.3 (-24 to 31.9)	-23.6 (-47.5 to 10.7)	5 (-23 to 42.6)
Latin America and Caribbean	Caribbean	Antigua and Barbuda	DALYs	Age-standardized	2.8 (2.3 to 3.3)	0.8 (0.6 to 1)	5.6 (4.6 to 6.7)	1.6 (1.3 to 2)	0.4 (0.3 to 0.5)	3.1 (2.4 to 3.9)	-42 (-55.6 to -23.3)	-54.6 (-68.9 to -35.2)	-44.7 (-59.2 to -25.7)
Latin America and Caribbean	Caribbean	Antigua and Barbuda	YLLs	All Ages	1 (1 to 2)	0 (0 to 0)	1 (1 to 1)	1 (1 to 2)	0 (0 to 0)	1 (1 to 2)	-2.5 (-27.8 to 31.1)	-31.5 (-55.7 to 3.9)	3.1 (-26.3 to 41.7)
Latin America and Caribbean	Caribbean	Antigua and Barbuda	YLLs	Age-standardized	2.6 (2.2 to 3.1)	0.7 (0.6 to 1)	5.2 (4.3 to 6.3)	1.5 (1.2 to 1.8)	0.3 (0.2 to 0.4)	2.8 (2.2 to 3.7)	-43.6 (-57.9 to -23.7)	-59.6 (-74 to -39.7)	-45.8 (-60.8 to -25.9)
Latin America and Caribbean	Caribbean	Antigua and Barbuda	YLDs	All Ages	0 (0 to 0)	0 (0 to 0)	0 (0 to 0)	0 (0 to 0)	0 (0 to 0)	0 (0 to 0)	42.8 (20.6 to 70.1)	67.1 (36.6 to 101.5)	36.2 (10.3 to 69.5)
Latin America and Caribbean	Caribbean	Antigua and Barbuda	YLDs	Age-standardized	0.2 (0.1 to 0.2)	0.1 (0 to 0.1)	0.3 (0.2 to 0.5)	0.1 (0.1 to 0.2)	0.1 (0 to 0.1)	0.2 (0.1 to 0.3)	-16.6 (-28.8 to -1.2)	5.5 (-13.9 to 25.9)	-27.8 (-40.4 to -10.8)
Latin America and Caribbean	Caribbean	Bahamas	Incidence	All Ages	0 (0 to 0)	0 (0 to 0)	0 (0 to 0)	0 (0 to 1)	0 (0 to 0)	0 (0 to 0)	108.5 (74.6 to 154.5)	111.2 (59.1 to 187.3)	105.7 (68.5 to 150.7)
Latin America and Caribbean	Caribbean	Bahamas	Incidence	Age-standardized	0.1 (0.1 to 0.2)	0.1 (0.1 to 0.2)	0.1 (0.1 to 0.2)	0.1 (0.1 to 0.2)	0.1 (0.1 to 0.2)	0.1 (0.1 to 0.2)	2.1 (-10.7 to 18.3)	6.8 (-17.1 to 35.8)	-4.7 (-19.3 to 11.1)
Latin America and Caribbean	Caribbean	Bahamas	Prevalence	All Ages	1 (1 to 1)	0 (0 to 1)	1 (1 to 1)	3 (2 to 3)	1 (1 to 1)	2 (1 to 2)	135.8 (108.1 to 165.7)	128.7 (90.4 to 171.5)	139.9 (99.2 to 181.6)
Latin America and Caribbean	Caribbean	Bahamas	Prevalence	Age-standardized	0.7 (0.5 to 0.9)	0.4 (0.3 to 0.6)	1 (0.8 to 1.3)	0.7 (0.6 to 0.9)	0.5 (0.4 to 0.6)	1 (0.8 to 1.3)	2.4 (-9.3 to 14.8)	2.5 (-15.8 to 22)	-0.8 (-17.1 to 18.1)
Latin America and Caribbean	Caribbean	Bahamas	Deaths	All Ages	0 (0 to 0)	0 (0 to 0)	0 (0 to 0)	0 (0 to 0)	0 (0 to 0)	0 (0 to 0)	55 (12.6 to 110)	-35.4 (-56.3 to 0)	87.2 (32.2 to 167.1)
Latin America and Caribbean	Caribbean	Bahamas	Deaths	Age-standardized	0.1 (0.1 to 0.1)	0 (0 to 0.1)	0.2 (0.1 to 0.2)	0.1 (0 to 0.1)	0 (0 to 0)	0.1 (0.1 to 0.2)	-35.2 (-52.7 to -13.3)	-72.3 (-81.2 to -56.3)	-28.2 (-48.6 to 0.3)
Latin America and Caribbean	Caribbean	Bahamas	DALYs	All Ages	4 (3 to 4)	1 (1 to 1)	3 (2 to 3)	6 (4 to 7)	1 (1 to 1)	5 (4 to 6)	50.1 (14 to 106.6)	-26 (-47 to 5.5)	75.4 (26.6 to 149.3)
Latin America and Caribbean	Caribbean	Bahamas	DALYs	Age-standardized	2 (1.7 to 2.4)	0.9 (0.7 to 1.2)	3.5 (2.9 to 4.3)	1.4 (1.1 to 1.8)	0.3 (0.2 to 0.4)	2.7 (2.1 to 3.5)	-30.9 (-47.6 to -7)	-66.2 (-75.7 to -52.3)	-22.4 (-44.1 to 8.1)
Latin America and Caribbean	Caribbean	Bahamas	YLLs	All Ages	3 (3 to 4)	1 (1 to 1)	3 (2 to 3)	5 (4 to 7)	1 (0 to 1)	5 (3 to 6)	45.8 (8.3 to 105.6)	-37.9 (-58.5 to -5.9)	72.7 (22.6 to 148.9)
Latin America and Caribbean	Caribbean	Bahamas	YLLs	Age-standardized	1.9 (1.6 to 2.3)	0.9 (0.6 to 1.1)	3.3 (2.7 to 4.1)	1.3 (1 to 1.7)	0.2 (0.2 to 0.3)	2.6 (1.9 to 3.3)	-32.8 (-50.4 to -7.4)	-71.8 (-81 to -57.2)	-23.5 (-45.4 to 8.5)
Latin America and Caribbean	Caribbean	Bahamas	YLDs	All Ages	0 (0 to 0)	0 (0 to 0)	0 (0 to 0)	0 (0 to 1)	0 (0 to 0)	0 (0 to 0)	135.8 (108.4 to 165.6)	128.6 (90.5 to 171.2)	140 (99.5 to 181.3)
Latin America and Caribbean	Caribbean	Bahamas	YLDs	Age-standardized	0.1 (0.1 to 0.2)	0.1 (0 to 0.1)	0.2 (0.1 to 0.2)	0.1 (0.1 to 0.2)	0.1 (0 to 0.1)	0.2 (0.1 to 0.2)	2.3 (-9.3 to 14.7)	2.4 (-15.7 to 21.9)	-0.9 (-17.1 to 17.7)
Latin America and Caribbean	Caribbean	Barbados	Incidence	All Ages	0 (0 to 0)	0 (0 to 0)	0 (0 to 0)	0 (0 to 1)	0 (0 to 0)	0 (0 to 0)	64.5 (39.1 to 96.7)	57.2 (28.1 to 95.6)	73.3 (37.6 to 120.4)
Latin America and Caribbean	Caribbean	Barbados	Incidence	Age-standardized	0.1 (0.1 to 0.1)	0.1 (0.1 to 0.1)	0.1 (0.1 to 0.1)	0.1 (0.1 to 0.1)	0.1 (0.1 to 0.1)	0.1 (0.1 to 0.1)	10.7 (-2.5 to 28.7)	10.8 (-6.4 to 38.8)	10.1 (-8.6 to 34.8)
Latin America and Caribbean	Caribbean	Barbados	Prevalence	All Ages	2 (1 to 2)	1 (0 to 1)	1 (1 to 1)	3 (2 to 4)	1 (1 to 1)	2 (1 to 2)	86.4 (62.5 to 115.6)	66.5 (35.7 to 101.3)	100.8 (64.1 to 141.3)
Latin America and Caribbean	Caribbean	Barbados	Prevalence	Age-standardized	0.5 (0.4 to 0.7)	0.4 (0.3 to 0.5)	0.7 (0.6 to 1)	0.6 (0.5 to 0.8)	0.4 (0.3 to 0.6)	0.9 (0.7 to 1.2)	16.8 (4.2 to 32.9)	11.6 (-6.7 to 31.4)	17.7 (-0.7 to 40.2)
Latin America and Caribbean	Caribbean	Barbados	Deaths	All Ages	0 (0 to 0)	0 (0 to 0)	0 (0 to 0)	0 (0 to 0)	0 (0 to 0)	0 (0 to 0)	2.4 (-26.4 to 42.5)	-44.7 (-65.2 to -5.1)	41.9 (-0.7 to 101.1)
Latin America and Caribbean	Caribbean	Barbados	Deaths	Age-standardized	0 (0 to 0)	0 (0 to 0)	0 (0 to 0)	0 (0 to 0)	0 (0 to 0)	0 (0 to 0)	-33.9 (-52.3 to -9)	-60.9 (-74.9 to -34.4)	-17.8 (-42.5 to 14.3)
Latin America and Caribbean	Caribbean	Barbados	DALYs	All Ages	1 (1 to 1)	0 (0 to 1)	1 (1 to 1)	1 (1 to 2)	0 (0 to 1)	1 (1 to 1)	19.1 (-6.5 to 50.3)	-20.2 (-42 to 13.9)	49.2 (12.1 to 92.9)
Latin America and Caribbean	Caribbean	Barbados	DALYs	Age-standardized	0.4 (0.3 to 0.5)	0.3 (0.2 to 0.4)	0.5 (0.4 to 0.6)	0.3 (0.2 to 0.4)	0.2 (0.1 to 0.2)	0.5 (0.4 to 0.6)	-23.6 (-39.9 to -4.1)	-46 (-60.3 to -23.7)	-11.1 (-33.1 to 15)
Latin America and Caribbean	Caribbean	Barbados	YLLs	All Ages	1 (1 to 1)	0 (0 to 1)	1 (0 to 1)	1 (1 to 1)	0 (0 to 0)	1 (1 to 1)	0.6 (-27 to 37.8)	-43 (-62.4 to -7.7)	34.6 (-6.1 to 89.9)
Latin America and Caribbean	Caribbean	Barbados	YLLs	Age-standardized	0.3 (0.2 to 0.4)	0.2 (0.2 to 0.3)	0.4 (0.3 to 0.5)	0.2 (0.2 to 0.2)	0.1 (0.1 to 0.1)	0.3 (0.2 to 0.4)	-34.9 (-52.6 to -11.9)	-60.9 (-74 to -37.6)	-19.4 (-43.7 to 11.9)
Latin America and Caribbean	Caribbean	Barbados	YLDs	All Ages	0 (0 to 0)	0 (0 to 0)	0 (0 to 0)	0 (0 to 1)	0 (0 to 0)	0 (0 to 0)	86.2 (62.5 to 115.1)	66.3 (35.6 to 101)	100.6 (64.2 to 140.8)
Latin America and Caribbean	Caribbean	Barbados	YLDs	Age-standardized	0.1 (0.1 to 0.1)	0.1 (0 to 0.1)	0.1 (0.1 to 0.2)	0.1 (0.1 to 0.2)	0.1 (0 to 0.1)	0.1 (0.1 to 0.2)	16.8 (4.1 to 32.7)	11.5 (-6.7 to 31.2)	17.6 (-0.7 to 39.8)
Latin America and Caribbean	Caribbean	Belize	Incidence	All Ages	0 (0 to 0)	0 (0 to 0)	0 (0 to 0)	1 (1 to 1)	0 (0 to 1)	0 (0 to 0)	332.5 (228.7 to 450.1)	512.1 (300.1 to 869.1)	200.5 (134.3 to 287.4)
Latin America and Caribbean	Caribbean	Belize	Incidence	Age-standardized	0.2 (0.1 to 0.2)	0.1 (0.1 to 0.2)	0.2 (0.2 to 0.2)	0.2 (0.2 to 0.3)	0.2 (0.2 to 0.3)	0.2 (0.1 to 0.2)	41.8 (14.8 to 72.3)	105.9 (44.8 to 195.9)	-2 (-20.8 to 21.9)
Latin America and Caribbean	Caribbean	Belize	Prevalence	All Ages	1 (1 to 1)	0 (0 to 0)	0 (0 to 1)	3 (2 to 3)	1 (1 to 1)	2 (1 to 2)	247.8 (207.5 to 294.4)	302.3 (227.2 to 395.8)	218.5 (172.7 to 268.9)
Latin America and Caribbean	Caribbean	Belize	Prevalence	Age-standardized	0.7 (0.6 to 0.9)	0.5 (0.4 to 0.6)	0.9 (0.7 to 1.2)	0.8 (0.7 to 1)	0.7 (0.5 to 0.8)	1 (0.8 to 1.3)	16.8 (3.8 to 31.9)	33.4 (11.9 to 61.3)	8.2 (-8.9 to 26.6)
Latin America and Caribbean	Caribbean	Belize	Deaths	All Ages	0 (0 to 0)	0 (0 to 0)	0 (0 to 0)	0 (0 to 1)	0 (0 to 0)	0 (0 to 1)	198.2 (105.8 to 333.9)	-3.4 (-36 to 56.2)	226.3 (120.1 to 386.6)
Latin America and Caribbean	Caribbean	Belize	Deaths	Age-standardized	0.2 (0.1 to 0.2)	0 (0 to 0.1)	0.3 (0.2 to 0.4)	0.2 (0.1 to 0.2)	0 (0 to 0)	0.3 (0.3 to 0.4)	4.9 (-28.5 to 55)	-66.6 (-78.5 to -45.6)	11.1 (-26.3 to 68.2)
Latin America and Caribbean	Caribbean	Belize	DALYs	All Ages	4 (3 to 6)	0 (0 to 1)	4 (3 to 5)	14 (12 to 18)	1 (1 to 1)	14 (11 to 17)	238.2 (141.6 to 387.7)	47.4 (-1 to 128)	262.7 (152.9 to 431.2)
Latin America and Caribbean	Caribbean	Belize	DALYs	Age-standardized	3.6 (2.7 to 4.7)	0.9 (0.6 to 1.2)	6.3 (4.7 to 8.6)	4.2 (3.4 to 5.1)	0.4 (0.3 to 0.5)	8 (6.4 to 9.8)	17 (-16.7 to 67.2)	-53 (-67.8 to -28)	26.1 (-13.2 to 84.3)
Latin America and Caribbean	Caribbean	Belize	YLLs	All Ages	4 (3 to 5)	0 (0 to 1)	4 (3 to 5)	14 (11 to 17)	1 (0 to 1)	13 (11 to 17)	238 (139 to 391.9)	24.4 (-19.3 to 108)	263.7 (151.9 to 436.5)
Latin America and Caribbean	Caribbean	Belize	YLLs	Age-standardized	3.5 (2.6 to 4.6)	0.8 (0.6 to 1.1)	6.2 (4.5 to 8.4)	4.1 (3.3 to 5)	0.3 (0.2 to 0.4)	7.8 (6.3 to 9.6)	17 (-17.6 to 69)	-61.2 (-74.6 to -36.5)	26.6 (-13.6 to 86.8)
Latin America and Caribbean	Caribbean	Belize	YLDs	All Ages	0 (0 to 0)	0 (0 to 0)	0 (0 to 0)	0 (0 to 1)	0 (0 to 0)	0 (0 to 0)	245.5 (206.3 to 290.4)	300.4 (226.5 to 391.9)	216.4 (171.3 to 265.4)
Latin America and Caribbean	Caribbean	Belize	YLDs	Age-standardized	0.1 (0.1 to 0.2)	0.1 (0 to 0.1)	0.2 (0.1 to 0.2)	0.1 (0.1 to 0.2)	0.1 (0.1 to 0.2)	0.2 (0.1 to 0.2)	16.1 (3.2 to 30.9)	32.9 (11.7 to 60.4)	7.4 (-9 to 25.5)
Latin America and Caribbean	Caribbean	Bermuda	Incidence	All Ages	0 (0 to 0)	0 (0 to 0)	0 (0 to 0)	0 (0 to 0)	0 (0 to 0)	0 (0 to 0)	-42.7 (-56.6 to -25.8)	-51.1 (-65.5 to -31.2)	-28.5 (-46.1 to -3.2)
Latin America and Caribbean	Caribbean	Bermuda	Incidence	Age-standardized	0.4 (0.3 to 0.5)	0.5 (0.3 to 0.6)	0.4 (0.3 to 0.4)	0.1 (0.1 to 0.2)	0.2 (0.1 to 0.2)	0.1 (0.1 to 0.2)	-64.4 (-72.8 to -54.4)	-68.5 (-77.6 to -55.9)	-57.9 (-67.5 to -44.2)
Latin America and Caribbean	Caribbean	Bermuda	Prevalence	All Ages	1 (1 to 1)	0 (0 to 0)	0 (0 to 0)	1 (1 to 1)	0 (0 to 0)	1 (0 to 1)	26 (4.2 to 51.1)	-2.2 (-24.8 to 26.5)	52.6 (25.8 to 85.2)
Latin America and Caribbean	Caribbean	Bermuda	Prevalence	Age-standardized	1.2 (1 to 1.4)	1 (0.8 to 1.3)	1.4 (1.2 to 1.7)	0.8 (0.6 to 1)	0.5 (0.4 to 0.7)	1.1 (0.9 to 1.4)	-34.3 (-44.4 to -23.2)	-48.9 (-59.5 to -35.4)	-23.2 (-35.1 to -8.9)
Latin America and Caribbean	Caribbean	Bermuda	Deaths	All Ages	0 (0 to 0)	0 (0 to 0)	0 (0 to 0)	0 (0 to 0)	0 (0 to 0)	0 (0 to 0)	-28.9 (-48.5 to -0.9)	-65.9 (-79.5 to -41.6)	-21.3 (-45.3 to 16.7)
Latin America and Caribbean	Caribbean	Bermuda	Deaths	Age-standardized	0.2 (0.1 to 0.2)	0 (0 to 0.1)	0.3 (0.3 to 0.4)	0 (0 to 0.1)	0 (0 to 0)	0.1 (0.1 to 0.1)	-69.5 (-77.5 to -57.3)	-85.9 (-91.3 to -76.1)	-67.4 (-76.9 to -52.2)
Latin America and Caribbean	Caribbean	Bermuda	DALYs	All Ages	2 (2 to 2)	0 (0 to 0)	2 (1 to 2)	1 (1 to 1)	0 (0 to 0)	1 (1 to 1)	-41.2 (-56.1 to -19.9)	-61.4 (-72.1 to -43.2)	-37.2 (-55.3 to -10.1)
Latin America and Caribbean	Caribbean	Bermuda	DALYs	Age-standardized	3.3 (2.6 to 4)	1 (0.7 to 1.2)	6.4 (5 to 8.1)	1 (0.8 to 1.3)	0.2 (0.1 to 0.3)	2.1 (1.7 to 2.6)	-68.6 (-76.4 to -57.2)	-80 (-85.5 to -70.9)	-67.5 (-76.3 to -53.2)
Latin America and Caribbean	Caribbean	Bermuda	YLLs	All Ages	2 (2 to 2)	0 (0 to 0)	2 (1 to 2)	1 (1 to 1)	0 (0 to 0)	1 (1 to 1)	-45.4 (-60.6 to -23.2)	-73.7 (-83 to -56.2)	-40.5 (-58.5 to -13)
Latin America and Caribbean	Caribbean	Bermuda	YLLs	Age-standardized	3.1 (2.5 to 3.8)	0.8 (0.5 to 1.1)	6.2 (4.7 to 7.8)	0.9 (0.7 to 1.2)	0.1 (0.1 to 0.2)	1.9 (1.5 to 2.4)	-70.7 (-78.6 to -58.9)	-86.3 (-91 to -77.5)	-69.1 (-77.8 to -54.7)
Latin America and Caribbean	Caribbean	Bermuda	YLDs	All Ages	0 (0 to 0)	0 (0 to 0)	0 (0 to 0)	0 (0 to 0)	0 (0 to 0)	0 (0 to 0)	26.8 (5.6 to 51.5)	-1.5 (-24 to 27.1)	53.3 (26.4 to 85.3)
Latin America and Caribbean	Caribbean	Bermuda	YLDs	Age-standardized	0.2 (0.1 to 0.3)	0.2 (0.1 to 0.2)	0.2 (0.2 to 0.3)	0.1 (0.1 to 0.2)	0.1 (0.1 to 0.1)	0.2 (0.1 to 0.3)	-34.1 (-44.1 to -23.1)	-48.6 (-59.2 to -35.2)	-23.3 (-34.9 to -9.4)
Latin America and Caribbean	Caribbean	Cuba	Incidence	All Ages	8 (6 to 11)	3 (2 to 5)	5 (3 to 6)	13 (10 to 18)	6 (4 to 8)	7 (5 to 10)	68.7 (35.9 to 105.2)	72.9 (29.8 to 134.8)	65.4 (28.7 to 103.9)
Latin America and Caribbean	Caribbean	Cuba	Incidence	Age-standardized	0.1 (0.1 to 0.1)	0.1 (0 to 0.1)	0.1 (0.1 to 0.1)	0.1 (0.1 to 0.1)	0.1 (0 to 0.1)	0.1 (0.1 to 0.1)	12.6 (-3.3 to 29.9)	14.1 (-12.1 to 46.5)	12.6 (-6.4 to 32.3)
Latin America and Caribbean	Caribbean	Cuba	Prevalence	All Ages	58 (45 to 73)	19 (14 to 24)	39 (30 to 51)	119 (92 to 152)	41 (31 to 54)	78 (58 to 103)	104.3 (74.6 to 137.8)	115.7 (71.8 to 172.9)	98.8 (65.8 to 137.8)
Latin America and Caribbean	Caribbean	Cuba	Prevalence	Age-standardized	0.6 (0.4 to 0.7)	0.4 (0.3 to 0.5)	0.8 (0.6 to 1)	0.7 (0.5 to 0.8)	0.4 (0.3 to 0.5)	0.9 (0.7 to 1.2)	17.3 (2.8 to 35.4)	16.1 (-5.5 to 43.4)	20.4 (2.6 to 41.8)
Latin America and Caribbean	Caribbean	Cuba	Deaths	All Ages	1 (1 to 2)	0 (0 to 0)	1 (1 to 2)	3 (2 to 4)	0 (0 to 0)	2 (2 to 3)	86.6 (34.7 to 150.6)	221.8 (92.4 to 406.3)	78.3 (25.8 to 141.6)
Latin America and Caribbean	Caribbean	Cuba	Deaths	Age-standardized	0 (0 to 0)	0 (0 to 0)	0 (0 to 0)	0 (0 to 0)	0 (0 to 0)	0 (0 to 0)	-6.8 (-33.2 to 24.7)	50.1 (-9.7 to 133.2)	-4.6 (-32.9 to 30.1)
Latin America and Caribbean	Caribbean	Cuba	DALYs	All Ages	37 (31 to 43)	5 (3 to 6)	32 (27 to 37)	67 (52 to 85)	12 (9 to 16)	55 (42 to 72)	83.4 (40.8 to 130.4)	158.7 (106.8 to 227)	72.4 (28.5 to 122.6)
Latin America and Caribbean	Caribbean	Cuba	DALYs	Age-standardized	0.4 (0.3 to 0.4)	0.1 (0.1 to 0.1)	0.6 (0.5 to 0.7)	0.4 (0.3 to 0.5)	0.1 (0.1 to 0.2)	0.6 (0.5 to 0.8)	1.3 (-22.4 to 27.2)	42.6 (16.1 to 80.2)	-0.7 (-26.4 to 28.1)
Latin America and Caribbean	Caribbean	Cuba	YLLs	All Ages	27 (23 to 32)	2 (1 to 2)	26 (21 to 30)	48 (36 to 63)	6 (3 to 8)	43 (31 to 57)	76.4 (23.9 to 136.8)	232.9 (113.8 to 420)	66.1 (14.2 to 128.8)
Latin America and Caribbean	Caribbean	Cuba	YLLs	Age-standardized	0.3 (0.2 to 0.3)	0 (0 to 0)	0.5 (0.4 to 0.6)	0.3 (0.2 to 0.3)	0.1 (0 to 0.1)	0.5 (0.4 to 0.6)	-3.9 (-32.6 to 29.6)	88.6 (25.8 to 195.4)	-5.7 (-35 to 29.4)
Latin America and Caribbean	Caribbean	Cuba	YLDs	All Ages	9 (6 to 14)	3 (2 to 4)	6 (4 to 9)	19 (12 to 29)	6 (4 to 10)	12 (8 to 19)	104.3 (74.8 to 137.6)	116 (71.9 to 172.8)	98.8 (65.8 to 137.4)
Latin America and Caribbean	Caribbean	Cuba	YLDs	Age-standardized	0.1 (0.1 to 0.1)	0.1 (0 to 0.1)	0.1 (0.1 to 0.2)	0.1 (0.1 to 0.2)	0.1 (0 to 0.1)	0.1 (0.1 to 0.2)	17.3 (2.6 to 35.2)	16.2 (-5.6 to 43.3)	20.3 (2.5 to 41.4)
Latin America and Caribbean	Caribbean	Dominica	Incidence	All Ages	0 (0 to 0)	0 (0 to 0)	0 (0 to 0)	0 (0 to 0)	0 (0 to 0)	0 (0 to 0)	46.5 (24 to 74.7)	33.6 (4.5 to 67.5)	60.3 (26.3 to 106.7)
Latin America and Caribbean	Caribbean	Dominica	Incidence	Age-standardized	0.1 (0.1 to 0.1)	0.1 (0.1 to 0.1)	0.1 (0.1 to 0.1)	0.1 (0.1 to 0.1)	0.1 (0.1 to 0.1)	0.1 (0.1 to 0.1)	22.6 (4.6 to 45.7)	24.1 (-4.3 to 58.6)	19.6 (-1.8 to 46.5)
Latin America and Caribbean	Caribbean	Dominica	Prevalence	All Ages	0 (0 to 0)	0 (0 to 0)	0 (0 to 0)	1 (0 to 1)	0 (0 to 0)	0 (0 to 1)	50.5 (29.9 to 75.2)	26.5 (4.6 to 56.8)	66.5 (34.4 to 100.9)
Latin America and Caribbean	Caribbean	Dominica	Prevalence	Age-standardized	0.5 (0.4 to 0.7)	0.4 (0.3 to 0.5)	0.8 (0.6 to 1)	0.7 (0.5 to 0.8)	0.4 (0.3 to 0.6)	0.9 (0.7 to 1.2)	21.1 (6.4 to 40.3)	15 (-3.4 to 39.3)	16.4 (-4.7 to 39.6)
Latin America and Caribbean	Caribbean	Dominica	Deaths	All Ages	0 (0 to 0)	0 (0 to 0)	0 (0 to 0)	0 (0 to 0)	0 (0 to 0)	0 (0 to 0)	-14.8 (-44.9 to 28.5)	-59.6 (-79.9 to -26.6)	24.7 (-26.1 to 115.9)
Latin America and Caribbean	Caribbean	Dominica	Deaths	Age-standardized	0 (0 to 0)	0 (0 to 0)	0.1 (0 to 0.1)	0 (0 to 0)	0 (0 to 0)	0 (0 to 0.1)	-29.7 (-54.2 to 4.5)	-63 (-80.6 to -36.8)	-8.9 (-45.9 to 57.8)
Latin America and Caribbean	Caribbean	Dominica	DALYs	All Ages	1 (0 to 1)	0 (0 to 0)	0 (0 to 0)	1 (0 to 1)	0 (0 to 0)	0 (0 to 1)	-10.6 (-38.3 to 25.8)	-52.7 (-71.5 to -28.9)	21 (-22.6 to 87)
Latin America and Caribbean	Caribbean	Dominica	DALYs	Age-standardized	0.9 (0.7 to 1.1)	0.6 (0.5 to 0.9)	1.1 (0.8 to 1.5)	0.6 (0.5 to 0.8)	0.3 (0.2 to 0.4)	1 (0.8 to 1.4)	-25 (-48.2 to 5.8)	-57 (-73.9 to -35.4)	-8.4 (-40.6 to 39.3)
Latin America and Caribbean	Caribbean	Dominica	YLLs	All Ages	1 (0 to 1)	0 (0 to 0)	0 (0 to 0)	0 (0 to 1)	0 (0 to 0)	0 (0 to 0)	-17.3 (-45.6 to 22)	-60.7 (-78.9 to -35.2)	15.8 (-30.1 to 89.4)
Latin America and Caribbean	Caribbean	Dominica	YLLs	Age-standardized	0.8 (0.6 to 1)	0.6 (0.4 to 0.8)	1 (0.7 to 1.4)	0.5 (0.4 to 0.7)	0.2 (0.1 to 0.3)	0.9 (0.6 to 1.2)	-30.2 (-54 to 3.3)	-64.1 (-80.6 to -40.7)	-11.5 (-45.7 to 43.9)
Latin America and Caribbean	Caribbean	Dominica	YLDs	All Ages	0 (0 to 0)	0 (0 to 0)	0 (0 to 0)	0 (0 to 0)	0 (0 to 0)	0 (0 to 0)	50.3 (29.8 to 74.7)	26.4 (4.6 to 56.5)	66.2 (34.4 to 100.5)
Latin America and Caribbean	Caribbean	Dominica	YLDs	Age-standardized	0.1 (0.1 to 0.1)	0.1 (0 to 0.1)	0.1 (0.1 to 0.2)	0.1 (0.1 to 0.2)	0.1 (0 to 0.1)	0.1 (0.1 to 0.2)	21 (6.4 to 40)	15 (-3.4 to 39)	16.3 (-4.8 to 39.2)
Latin America and Caribbean	Caribbean	Dominican Republic	Incidence	All Ages	4 (2 to 5)	2 (1 to 3)	2 (1 to 3)	8 (5 to 11)	3 (2 to 5)	4 (3 to 6)	120.5 (86.5 to 165.8)	127 (80.9 to 196.6)	115.6 (77.8 to 169.8)
Latin America and Caribbean	Caribbean	Dominican Republic	Incidence	Age-standardized	0.1 (0.1 to 0.1)	0.1 (0 to 0.1)	0.1 (0.1 to 0.1)	0.1 (0.1 to 0.1)	0.1 (0 to 0.1)	0.1 (0.1 to 0.1)	6.1 (-6.5 to 24.3)	14.7 (-3.5 to 39)	0.7 (-14.9 to 20.8)
Latin America and Caribbean	Caribbean	Dominican Republic	Prevalence	All Ages	22 (17 to 28)	7 (5 to 9)	15 (11 to 20)	59 (46 to 74)	19 (15 to 26)	39 (30 to 52)	168.9 (138.3 to 207.6)	177 (135.2 to 232.1)	165.1 (125.1 to 208)
Latin America and Caribbean	Caribbean	Dominican Republic	Prevalence	Age-standardized	0.5 (0.4 to 0.7)	0.3 (0.3 to 0.5)	0.7 (0.5 to 1)	0.6 (0.5 to 0.8)	0.4 (0.3 to 0.5)	0.9 (0.7 to 1.1)	16.6 (3 to 34.2)	17.1 (-0.4 to 39.3)	17.4 (-1.4 to 38.8)
Latin America and Caribbean	Caribbean	Dominican Republic	Deaths	All Ages	1 (1 to 1)	0 (0 to 0)	1 (1 to 1)	1 (1 to 2)	0 (0 to 0)	1 (1 to 2)	45.3 (-4.4 to 131.3)	199.5 (62.7 to 425.1)	30.7 (-17.2 to 122)
Latin America and Caribbean	Caribbean	Dominican Republic	Deaths	Age-standardized	0 (0 to 0)	0 (0 to 0)	0.1 (0 to 0.1)	0 (0 to 0)	0 (0 to 0)	0 (0 to 0)	-47 (-65.5 to -16.4)	7.9 (-42.3 to 97.8)	-50.8 (-69 to -16.5)
Latin America and Caribbean	Caribbean	Dominican Republic	DALYs	All Ages	27 (21 to 34)	3 (3 to 4)	24 (18 to 31)	43 (31 to 63)	8 (6 to 12)	35 (24 to 54)	59.7 (8.2 to 138.6)	143 (64.4 to 250.9)	47.5 (-3.3 to 140.1)
Latin America and Caribbean	Caribbean	Dominican Republic	DALYs	Age-standardized	0.7 (0.5 to 0.9)	0.2 (0.1 to 0.2)	1.2 (0.9 to 1.6)	0.5 (0.3 to 0.6)	0.2 (0.1 to 0.2)	0.8 (0.5 to 1.2)	-32.8 (-53.4 to 0.3)	11 (-22.4 to 57)	-37.7 (-58.4 to 0.9)
Latin America and Caribbean	Caribbean	Dominican Republic	YLLs	All Ages	24 (18 to 31)	2 (2 to 3)	21 (16 to 28)	34 (23 to 53)	5 (3 to 8)	29 (19 to 48)	43.8 (-10.5 to 136.2)	127 (27 to 279.6)	34.6 (-18.2 to 136.3)
Latin America and Caribbean	Caribbean	Dominican Republic	YLLs	Age-standardized	0.6 (0.4 to 0.8)	0.1 (0.1 to 0.1)	1.1 (0.8 to 1.4)	0.4 (0.2 to 0.6)	0.1 (0.1 to 0.2)	0.6 (0.4 to 1)	-39.8 (-61.6 to -2.9)	7.7 (-40.6 to 84.8)	-43.5 (-64.5 to -0.6)
Latin America and Caribbean	Caribbean	Dominican Republic	YLDs	All Ages	3 (2 to 5)	1 (1 to 2)	2 (1 to 4)	9 (6 to 14)	3 (2 to 5)	6 (4 to 10)	168.9 (138.3 to 207.3)	177.2 (135.3 to 232.1)	165 (125.2 to 207.6)
Latin America and Caribbean	Caribbean	Dominican Republic	YLDs	Age-standardized	0.1 (0.1 to 0.1)	0.1 (0 to 0.1)	0.1 (0.1 to 0.2)	0.1 (0.1 to 0.2)	0.1 (0 to 0.1)	0.1 (0.1 to 0.2)	16.5 (2.9 to 33.9)	17.1 (-0.4 to 39.2)	17.2 (-1.4 to 38.5)
Latin America and Caribbean	Caribbean	Grenada	Incidence	All Ages	0 (0 to 0)	0 (0 to 0)	0 (0 to 0)	0 (0 to 0)	0 (0 to 0)	0 (0 to 0)	-18.9 (-35.3 to 2.6)	-27.4 (-48.9 to 0.8)	-9.4 (-27.9 to 15.9)
Latin America and Caribbean	Caribbean	Grenada	Incidence	Age-standardized	0.3 (0.2 to 0.4)	0.3 (0.2 to 0.4)	0.3 (0.3 to 0.4)	0.2 (0.1 to 0.2)	0.1 (0.1 to 0.2)	0.2 (0.2 to 0.2)	-44 (-55 to -30.8)	-48.3 (-64.1 to -28.9)	-38.2 (-49.4 to -24)
Latin America and Caribbean	Caribbean	Grenada	Prevalence	All Ages	1 (1 to 1)	0 (0 to 1)	0 (0 to 0)	1 (1 to 1)	0 (0 to 0)	1 (0 to 1)	34.2 (15.8 to 54.4)	-6.6 (-25.5 to 11.9)	71.2 (41.5 to 107.6)
Latin America and Caribbean	Caribbean	Grenada	Prevalence	Age-standardized	0.9 (0.8 to 1.1)	0.7 (0.6 to 0.9)	1.1 (0.9 to 1.4)	0.8 (0.7 to 1)	0.5 (0.4 to 0.7)	1.2 (1 to 1.5)	-8.6 (-19.5 to 3.4)	-29.1 (-42.9 to -15.8)	7.7 (-11.2 to 28.3)
Latin America and Caribbean	Caribbean	Grenada	Deaths	All Ages	0 (0 to 0)	0 (0 to 0)	0 (0 to 0)	0 (0 to 0)	0 (0 to 0)	0 (0 to 0)	-42.4 (-60 to -16.8)	-72.5 (-82.4 to -56.5)	-38.8 (-58.7 to -9.8)
Latin America and Caribbean	Caribbean	Grenada	Deaths	Age-standardized	0.3 (0.2 to 0.4)	0.1 (0 to 0.1)	0.7 (0.5 to 1)	0.1 (0.1 to 0.2)	0 (0 to 0)	0.4 (0.3 to 0.4)	-55.8 (-68.8 to -36.7)	-76.7 (-84.7 to -63.1)	-51.4 (-66.5 to -28.2)
Latin America and Caribbean	Caribbean	Grenada	DALYs	All Ages	5 (4 to 7)	1 (0 to 1)	5 (4 to 6)	3 (3 to 4)	0 (0 to 0)	3 (3 to 4)	-34.1 (-51.8 to -8.9)	-64.5 (-75.3 to -49.1)	-30.4 (-50.7 to -1.7)
Latin America and Caribbean	Caribbean	Grenada	DALYs	Age-standardized	7.2 (5.7 to 9)	1.4 (1 to 1.8)	14.8 (11.3 to 19.1)	3.1 (2.6 to 3.9)	0.4 (0.3 to 0.4)	6.6 (5.4 to 8.1)	-56.4 (-67.7 to -40.3)	-74.6 (-82.2 to -63.7)	-55.5 (-67.9 to -36.9)
Latin America and Caribbean	Caribbean	Grenada	YLLs	All Ages	5 (4 to 6)	1 (0 to 1)	5 (4 to 6)	3 (3 to 4)	0 (0 to 0)	3 (2 to 4)	-35.5 (-53.3 to -9.7)	-70.2 (-80.4 to -55.1)	-31.6 (-52 to -2.9)
Latin America and Caribbean	Caribbean	Grenada	YLLs	Age-standardized	7 (5.5 to 8.8)	1.3 (0.9 to 1.7)	14.6 (11.1 to 18.8)	3 (2.4 to 3.7)	0.3 (0.2 to 0.4)	6.4 (5.2 to 7.9)	-57.4 (-68.9 to -40.9)	-78.8 (-86 to -68.1)	-56.3 (-68.7 to -37.7)
Latin America and Caribbean	Caribbean	Grenada	YLDs	All Ages	0 (0 to 0)	0 (0 to 0)	0 (0 to 0)	0 (0 to 0)	0 (0 to 0)	0 (0 to 0)	33.8 (15.7 to 53.9)	-6.5 (-25.4 to 11.8)	69.9 (40.9 to 105.2)
Latin America and Caribbean	Caribbean	Grenada	YLDs	Age-standardized	0.1 (0.1 to 0.2)	0.1 (0.1 to 0.2)	0.2 (0.1 to 0.3)	0.1 (0.1 to 0.2)	0.1 (0.1 to 0.1)	0.2 (0.1 to 0.3)	-8.7 (-19.5 to 3)	-28.9 (-42.7 to -15.7)	7 (-11.2 to 26.9)

Pneumoconiosis

7 GBD super region	21 GBD region	Location	Measure	Age	1990 Both	1990 Female	1990 Male	2019 Both	2019 Female	2019 Male	Percent change (1990 to 2019) Both	Percent change (1990 to 2019) Female	Percent change (1990 to 2019) Male
Latin America and Caribbean	Caribbean	Guyana	Incidence	All Ages	1 (1 to 1)	0 (0 to 1)	0 (0 to 0)	1 (1 to 2)	1 (0 to 1)	0 (0 to 1)	46.3 (20.9 to 81.6)	55.6 (19.3 to 122.2)	34.8 (4.3 to 69.2)
Latin America and Caribbean	Caribbean	Guyana	Incidence	Age-standardized	0.2 (0.1 to 0.2)	0.2 (0.1 to 0.2)	0.2 (0.1 to 0.2)	0.2 (0.1 to 0.2)	0.2 (0.1 to 0.3)	0.2 (0.1 to 0.2)	9.3 (-5.2 to 29)	18.7 (-6.8 to 56.2)	-1.1 (-21 to 21.5)
Latin America and Caribbean	Caribbean	Guyana	Prevalence	All Ages	3 (2 to 4)	1 (1 to 1)	2 (1 to 2)	5 (4 to 6)	2 (1 to 2)	3 (2 to 4)	54.6 (37.3 to 76.7)	65.5 (41.3 to 96.8)	48.2 (25 to 77.1)
Latin America and Caribbean	Caribbean	Guyana	Prevalence	Age-standardized	0.7 (0.6 to 0.9)	0.5 (0.4 to 0.6)	0.9 (0.7 to 1.2)	0.7 (0.6 to 0.9)	0.5 (0.4 to 0.7)	1 (0.7 to 1.2)	5 (-7.9 to 20.6)	14.2 (-1.1 to 32.6)	1.2 (-14.5 to 22.7)
Latin America and Caribbean	Caribbean	Guyana	Deaths	All Ages	0 (0 to 0)	0 (0 to 0)	0 (0 to 0)	0 (0 to 0)	0 (0 to 0)	0 (0 to 0)	0 (-30.4 to 43)	-76.8 (-85.4 to -61.3)	26.9 (-13.6 to 85)
Latin America and Caribbean	Caribbean	Guyana	Deaths	Age-standardized	0.1 (0 to 0.1)	0 (0 to 0)	0.1 (0.1 to 0.1)	0 (0 to 0)	0 (0 to 0)	0.1 (0.1 to 0.1)	-39.5 (-57.7 to -13.9)	-85.8 (-91.2 to -75.7)	-19.7 (-45.3 to 15.8)
Latin America and Caribbean	Caribbean	Guyana	DALYs	All Ages	6 (5 to 7)	2 (1 to 2)	4 (3 to 5)	7 (5 to 9)	1 (0 to 1)	6 (4 to 8)	14.8 (-16 to 60.6)	-59.6 (-71.5 to -40.6)	41.4 (1.1 to 101.5)
Latin America and Caribbean	Caribbean	Guyana	DALYs	Age-standardized	1.3 (1.1 to 1.6)	0.7 (0.5 to 0.9)	2 (1.6 to 2.5)	1 (0.7 to 1.2)	0.2 (0.1 to 0.2)	1.8 (1.4 to 2.4)	-26.5 (-46.2 to 2.1)	-73.4 (-81.3 to -59.9)	-8.3 (-34.8 to 30)
Latin America and Caribbean	Caribbean	Guyana	YLLs	All Ages	5 (4 to 6)	1 (1 to 2)	4 (3 to 5)	6 (4 to 8)	0 (0 to 0)	6 (4 to 7)	11.3 (-21.2 to 61.8)	-75.9 (-84.6 to -59.9)	40.9 (-3 to 105.6)
Latin America and Caribbean	Caribbean	Guyana	YLLs	Age-standardized	1.2 (1 to 1.4)	0.6 (0.4 to 0.8)	1.9 (1.5 to 2.3)	0.8 (0.6 to 1.1)	0.1 (0.1 to 0.1)	1.7 (1.2 to 2.2)	-29.4 (-50.5 to 2.4)	-84.5 (-90.2 to -74.7)	-9 (-37 to 32.3)
Latin America and Caribbean	Caribbean	Guyana	YLDs	All Ages	0 (0 to 1)	0 (0 to 0)	0 (0 to 0)	1 (0 to 1)	0 (0 to 0)	0 (0 to 1)	54.5 (37.3 to 76.7)	65.3 (41.3 to 96.7)	48.2 (25.1 to 76.9)
Latin America and Caribbean	Caribbean	Guyana	YLDs	Age-standardized	0.1 (0.1 to 0.2)	0.1 (0 to 0.1)	0.1 (0.1 to 0.2)	0.1 (0.1 to 0.2)	0.1 (0.1 to 0.1)	0.2 (0.1 to 0.2)	4.9 (-7.9 to 20.4)	14.1 (-1.1 to 32.6)	1.1 (-14.5 to 22.4)
Latin America and Caribbean	Caribbean	Haiti	Incidence	All Ages	8 (6 to 11)	5 (3 to 7)	3 (3 to 5)	18 (14 to 25)	11 (8 to 16)	7 (5 to 10)	122.3 (87.1 to 169.8)	133 (75.8 to 206.1)	107.6 (70.3 to 150.8)
Latin America and Caribbean	Caribbean	Haiti	Incidence	Age-standardized	0.2 (0.2 to 0.3)	0.2 (0.2 to 0.3)	0.2 (0.2 to 0.3)	0.2 (0.2 to 0.3)	0.2 (0.2 to 0.3)	0.2 (0.1 to 0.2)	0.4 (-11.6 to 17.2)	3.9 (-15.3 to 31.9)	-6 (-19.9 to 10.4)
Latin America and Caribbean	Caribbean	Haiti	Prevalence	All Ages	25 (20 to 31)	11 (8 to 14)	14 (11 to 19)	62 (50 to 77)	26 (20 to 34)	36 (28 to 46)	146.9 (123.5 to 175.6)	146.1 (110.7 to 193.3)	147.5 (118.9 to 193.4)
Latin America and Caribbean	Caribbean	Haiti	Prevalence	Age-standardized	0.7 (0.6 to 0.9)	0.6 (0.4 to 0.7)	0.9 (0.7 to 1.1)	0.8 (0.7 to 1)	0.6 (0.5 to 0.8)	1 (0.8 to 1.3)	16.6 (4.8 to 31.2)	13.9 (-1.9 to 34.5)	18.3 (2 to 40.9)
Latin America and Caribbean	Caribbean	Haiti	Deaths	All Ages	8 (3 to 13)	1 (1 to 2)	7 (2 to 12)	11 (4 to 21)	2 (1 to 4)	9 (2 to 19)	38.9 (-11.7 to 117.9)	54 (-9.6 to 169)	36.3 (-17.7 to 125.2)
Latin America and Caribbean	Caribbean	Haiti	Deaths	Age-standardized	0.3 (0.1 to 0.5)	0.1 (0 to 0.2)	0.5 (0.1 to 1)	0.2 (0.1 to 0.4)	0.1 (0 to 0.1)	0.3 (0.1 to 0.7)	-32 (-57.7 to 7.2)	-29.8 (-60.3 to 24.5)	-36.1 (-63 to 5)
Latin America and Caribbean	Caribbean	Haiti	DALYs	All Ages	236 (99 to 377)	39 (22 to 70)	197 (52 to 342)	313 (132 to 566)	60 (32 to 112)	253 (71 to 512)	32.8 (-13.5 to 111.5)	54.7 (-6.9 to 161.5)	28.5 (-20.9 to 111.8)
Latin America and Caribbean	Caribbean	Haiti	DALYs	Age-standardized	6.3 (2.6 to 10.2)	1.9 (1 to 3.7)	11.3 (3 to 19.6)	4 (1.6 to 7.3)	1.4 (0.7 to 2.6)	7 (1.8 to 14)	-36.5 (-58.7 to -1.6)	-29.9 (-57.2 to 19.7)	-38.3 (-61.8 to 0.8)
Latin America and Caribbean	Caribbean	Haiti	YLLs	All Ages	232 (95 to 372)	37 (20 to 68)	195 (50 to 340)	303 (121 to 557)	56 (27 to 107)	247 (61 to 505)	30.8 (-16.7 to 111)	50.6 (-11.3 to 163.5)	27.1 (-23.1 to 111.5)
Latin America and Caribbean	Caribbean	Haiti	YLLs	Age-standardized	6.2 (2.5 to 10.1)	1.8 (0.9 to 3.6)	11.2 (2.8 to 19.5)	3.9 (1.5 to 7.2)	1.3 (0.6 to 2.5)	6.8 (1.7 to 13.9)	-37.5 (-60 to -2.3)	-32 (-59.8 to 20.4)	-39 (-63 to 0.5)
Latin America and Caribbean	Caribbean	Haiti	YLDs	All Ages	4 (3 to 6)	2 (1 to 3)	2 (1 to 4)	10 (6 to 15)	4 (3 to 6)	6 (4 to 9)	146.8 (123.7 to 174.9)	146.3 (110.8 to 192.8)	147.2 (118.8 to 192.3)
Latin America and Caribbean	Caribbean	Haiti	YLDs	Age-standardized	0.1 (0.1 to 0.2)	0.1 (0.1 to 0.1)	0.1 (0.1 to 0.2)	0.1 (0.1 to 0.2)	0.1 (0.1 to 0.1)	0.2 (0.1 to 0.3)	16.5 (4.9 to 30.9)	14 (-1.9 to 34)	17.9 (2.1 to 39.9)
Latin America and Caribbean	Caribbean	Jamaica	Incidence	All Ages	3 (2 to 3)	2 (1 to 2)	1 (1 to 1)	3 (2 to 4)	1 (1 to 2)	1 (1 to 2)	8.5 (-11.8 to 34.6)	-13.2 (-37.2 to 20)	42.9 (17.8 to 71.1)
Latin America and Caribbean	Caribbean	Jamaica	Incidence	Age-standardized	0.1 (0.1 to 0.2)	0.2 (0.1 to 0.2)	0.1 (0.1 to 0.1)	0.1 (0.1 to 0.1)	0.1 (0.1 to 0.1)	0.1 (0.1 to 0.1)	-32.1 (-45 to -15.5)	-43.7 (-60.1 to -22.5)	-12.6 (-27.6 to 4.4)
Latin America and Caribbean	Caribbean	Jamaica	Prevalence	All Ages	16 (13 to 20)	8 (7 to 11)	8 (6 to 10)	19 (15 to 25)	7 (5 to 9)	12 (9 to 16)	18.3 (0.5 to 39.3)	-17.8 (-37.5 to 2.4)	57.5 (32.7 to 88.4)
Latin America and Caribbean	Caribbean	Jamaica	Prevalence	Age-standardized	0.9 (0.7 to 1.1)	0.8 (0.6 to 1)	0.9 (0.7 to 1.2)	0.6 (0.5 to 0.8)	0.4 (0.3 to 0.6)	0.9 (0.6 to 1.1)	-28.2 (-38.8 to -15.9)	-48.4 (-60.3 to -35.9)	-7.1 (-22 to 11)
Latin America and Caribbean	Caribbean	Jamaica	Deaths	All Ages	3 (2 to 4)	2 (1 to 3)	1 (1 to 1)	1 (0 to 1)	0 (0 to 0)	1 (0 to 1)	-78.6 (-85.4 to -65.8)	-96.3 (-97.8 to -93.9)	-25.1 (-48.4 to 7.1)
Latin America and Caribbean	Caribbean	Jamaica	Deaths	Age-standardized	0.2 (0.1 to 0.2)	0.2 (0.1 to 0.3)	0.1 (0.1 to 0.1)	0 (0 to 0)	0 (0 to 0)	0 (0 to 0)	-86.9 (-91.2 to -78.8)	-97.9 (-98.8 to -96.4)	-54.1 (-68.5 to -34.4)
Latin America and Caribbean	Caribbean	Jamaica	DALYs	All Ages	52 (40 to 68)	37 (25 to 52)	15 (12 to 19)	14 (11 to 19)	2 (2 to 3)	12 (9 to 16)	-72.3 (-80.4 to -58.8)	-93.5 (-95.8 to -89.7)	-22.2 (-44.1 to 8.5)
Latin America and Caribbean	Caribbean	Jamaica	DALYs	Age-standardized	2.9 (2.2 to 3.7)	3.9 (2.6 to 5.4)	1.7 (1.3 to 2.1)	0.5 (0.4 to 0.6)	0.1 (0.1 to 0.2)	0.9 (0.6 to 1.1)	-83.1 (-88.1 to -74.3)	-96.2 (-97.6 to -94.1)	-49.6 (-63.5 to -30.2)
Latin America and Caribbean	Caribbean	Jamaica	YLLs	All Ages	49 (37 to 66)	35 (23 to 50)	14 (11 to 18)	11 (8 to 15)	1 (1 to 2)	10 (7 to 13)	-77.1 (-84.4 to -64.3)	-96.4 (-97.8 to -94.1)	-29.1 (-51.9 to 3.2)
Latin America and Caribbean	Caribbean	Jamaica	YLLs	Age-standardized	2.7 (2 to 3.6)	3.7 (2.4 to 5.3)	1.5 (1.2 to 1.9)	0.4 (0.3 to 0.5)	0.1 (0.1 to 0.1)	0.7 (0.5 to 1)	-85.9 (-90.5 to -77.8)	-97.9 (-98.8 to -96.6)	-53.6 (-68.4 to -32.8)
Latin America and Caribbean	Caribbean	Jamaica	YLDs	All Ages	3 (2 to 4)	1 (1 to 2)	1 (1 to 2)	3 (2 to 5)	1 (1 to 2)	2 (1 to 3)	18.6 (1 to 39.3)	-16.9 (-36.3 to 2.9)	57.5 (32.7 to 88.1)
Latin America and Caribbean	Caribbean	Jamaica	YLDs	Age-standardized	0.1 (0.1 to 0.2)	0.1 (0.1 to 0.2)	0.1 (0.1 to 0.2)	0.1 (0.1 to 0.2)	0.1 (0 to 0.1)	0.1 (0.1 to 0.2)	-28 (-38.5 to -15.9)	-47.8 (-59.9 to -35.6)	-7.1 (-21.9 to 10.7)
Latin America and Caribbean	Caribbean	Puerto Rico	Incidence	All Ages	3 (2 to 5)	2 (1 to 2)	2 (1 to 2)	6 (5 to 8)	3 (2 to 5)	3 (2 to 4)	86.6 (50.2 to 129)	107.8 (49 to 182.8)	67.5 (36.4 to 109.3)
Latin America and Caribbean	Caribbean	Puerto Rico	Incidence	Age-standardized	0.1 (0.1 to 0.1)	0.1 (0.1 to 0.1)	0.1 (0.1 to 0.1)	0.1 (0.1 to 0.2)	0.1 (0.1 to 0.2)	0.1 (0.1 to 0.2)	17.2 (-1.7 to 40.9)	31.3 (-3.1 to 78.4)	4.7 (-13.6 to 25.2)
Latin America and Caribbean	Caribbean	Puerto Rico	Prevalence	All Ages	20 (16 to 26)	7 (5 to 10)	13 (10 to 17)	43 (33 to 57)	17 (13 to 24)	25 (19 to 34)	115.3 (82.9 to 153.9)	142.3 (96.2 to 200.6)	100 (58.5 to 149.7)
Latin America and Caribbean	Caribbean	Puerto Rico	Prevalence	Age-standardized	0.6 (0.4 to 0.7)	0.4 (0.3 to 0.5)	0.8 (0.6 to 1)	0.6 (0.5 to 0.8)	0.5 (0.4 to 0.6)	0.9 (0.7 to 1.2)	17.1 (2.2 to 32.6)	22.6 (3.6 to 47)	15.1 (-5.4 to 38.7)
Latin America and Caribbean	Caribbean	Puerto Rico	Deaths	All Ages	1 (1 to 1)	0 (0 to 0)	1 (1 to 1)	1 (1 to 2)	0 (0 to 0)	1 (1 to 1)	26 (-7.3 to 73.8)	175.2 (69.4 to 310.1)	8.6 (-21.3 to 54.1)
Latin America and Caribbean	Caribbean	Puerto Rico	Deaths	Age-standardized	0 (0 to 0)	0 (0 to 0)	0.1 (0 to 0.1)	0 (0 to 0)	0 (0 to 0)	0 (0 to 0)	-45.4 (-59.5 to -24.8)	29.9 (-18.3 to 92.3)	-51.9 (-64.9 to -33)
Latin America and Caribbean	Caribbean	Puerto Rico	DALYs	All Ages	21 (18 to 25)	3 (2 to 4)	18 (16 to 21)	26 (20 to 32)	8 (6 to 10)	18 (14 to 23)	21.1 (-5.1 to 54.3)	148.3 (82.5 to 223.1)	-0.6 (-24.4 to 30.3)
Latin America and Caribbean	Caribbean	Puerto Rico	DALYs	Age-standardized	0.6 (0.5 to 0.7)	0.2 (0.1 to 0.2)	1.1 (0.9 to 1.3)	0.4 (0.3 to 0.5)	0.2 (0.2 to 0.3)	0.6 (0.5 to 0.8)	-30.9 (-46.3 to -11.1)	41.8 (4.8 to 89.7)	-42.3 (-56.1 to -24.3)
Latin America and Caribbean	Caribbean	Puerto Rico	YLLs	All Ages	18 (15 to 21)	2 (2 to 3)	16 (14 to 19)	19 (14 to 24)	5 (3 to 7)	14 (10 to 18)	4.7 (-23 to 43.1)	151.8 (55.5 to 272.1)	-13.2 (-38.2 to 21.1)
Latin America and Caribbean	Caribbean	Puerto Rico	YLLs	Age-standardized	0.5 (0.4 to 0.6)	0.1 (0.1 to 0.1)	1 (0.8 to 1.1)	0.3 (0.2 to 0.4)	0.2 (0.1 to 0.2)	0.5 (0.4 to 0.7)	-39.3 (-55.7 to -16.4)	53.1 (-3.9 to 126.2)	-49.4 (-63.9 to -29.6)
Latin America and Caribbean	Caribbean	Puerto Rico	YLDs	All Ages	3 (2 to 5)	1 (1 to 2)	2 (1 to 3)	7 (4 to 10)	3 (2 to 4)	4 (2 to 6)	115.2 (82.9 to 153.4)	142.3 (96.2 to 200.1)	100 (58.9 to 149.1)
Latin America and Caribbean	Caribbean	Puerto Rico	YLDs	Age-standardized	0.1 (0.1 to 0.1)	0.1 (0 to 0.1)	0.1 (0.1 to 0.2)	0.1 (0.1 to 0.2)	0.1 (0 to 0.1)	0.1 (0.1 to 0.2)	16.9 (2.1 to 32.2)	22.5 (3.6 to 46.7)	14.9 (-5.4 to 38.2)
Latin America and Caribbean	Caribbean	Saint Kitts and Nevis	Incidence	All Ages	0 (0 to 0)	0 (0 to 0)	0 (0 to 0)	0 (0 to 0)	0 (0 to 0)	0 (0 to 0)	66.1 (33.2 to 103.4)	75.8 (27.3 to 123.3)	58.2 (21 to 101.3)
Latin America and Caribbean	Caribbean	Saint Kitts and Nevis	Incidence	Age-standardized	0.1 (0.1 to 0.1)	0.1 (0.1 to 0.1)	0.1 (0.1 to 0.2)	0.1 (0.1 to 0.1)	0.1 (0.1 to 0.1)	0.1 (0.1 to 0.1)	-3.6 (-19.6 to 12)	6.8 (-22.1 to 36.4)	-14.2 (-29.6 to 1.4)
Latin America and Caribbean	Caribbean	Saint Kitts and Nevis	Prevalence	All Ages	0 (0 to 0)	0 (0 to 0)	0 (0 to 0)	0 (0 to 1)	0 (0 to 0)	0 (0 to 0)	71 (41.8 to 103)	63.3 (35 to 97.3)	75 (37.6 to 121.7)
Latin America and Caribbean	Caribbean	Saint Kitts and Nevis	Prevalence	Age-standardized	0.7 (0.5 to 0.9)	0.4 (0.3 to 0.5)	1 (0.8 to 1.4)	0.7 (0.5 to 0.9)	0.4 (0.3 to 0.6)	1 (0.7 to 1.2)	-0.1 (-15 to 13.2)	3.2 (-12 to 23.8)	-6.1 (-24.7 to 11.7)
Latin America and Caribbean	Caribbean	Saint Kitts and Nevis	Deaths	All Ages	0 (0 to 0)	0 (0 to 0)	0 (0 to 0)	0 (0 to 0)	0 (0 to 0)	0 (0 to 0)	-24.4 (-45.3 to 3.7)	-74.1 (-84.3 to -56.7)	-3.7 (-33.4 to 36.2)
Latin America and Caribbean	Caribbean	Saint Kitts and Nevis	Deaths	Age-standardized	0.1 (0.1 to 0.1)	0 (0 to 0)	0.1 (0.1 to 0.1)	0 (0 to 0)	0 (0 to 0)	0.1 (0 to 0.1)	-53.6 (-65.1 to -38.7)	-81.8 (-88.7 to -69.9)	-43.9 (-59.7 to -23.3)
Latin America and Caribbean	Caribbean	Saint Kitts and Nevis	DALYs	All Ages	0 (0 to 1)	0 (0 to 0)	0 (0 to 0)	0 (0 to 1)	0 (0 to 0)	0 (0 to 1)	-4 (-28.9 to 29.3)	-59.4 (-73.1 to -35.9)	18.4 (-16.1 to 63.1)
Latin America and Caribbean	Caribbean	Saint Kitts and Nevis	DALYs	Age-standardized	1.3 (1.1 to 1.6)	0.7 (0.5 to 1)	2.2 (1.8 to 2.7)	0.7 (0.6 to 0.9)	0.2 (0.1 to 0.2)	1.4 (1.1 to 1.7)	-45.8 (-58.9 to -28.7)	-75.1 (-83.4 to -61.3)	-37.4 (-54.1 to -16.2)
Latin America and Caribbean	Caribbean	Saint Kitts and Nevis	YLLs	All Ages	0 (0 to 1)	0 (0 to 0)	0 (0 to 0)	0 (0 to 1)	0 (0 to 0)	0 (0 to 0)	-10.6 (-38.1 to 24.8)	-72.3 (-82.9 to -53.6)	13.8 (-22.7 to 62)
Latin America and Caribbean	Caribbean	Saint Kitts and Nevis	YLLs	Age-standardized	1.2 (1 to 1.5)	0.7 (0.4 to 0.9)	2 (1.6 to 2.5)	0.6 (0.5 to 0.8)	0.1 (0.1 to 0.2)	1.2 (0.9 to 1.6)	-49.8 (-63.6 to -31.1)	-83.1 (-89.4 to -72.5)	-39.9 (-57.6 to -16.4)
Latin America and Caribbean	Caribbean	Saint Kitts and Nevis	YLDs	All Ages	0 (0 to 0)	0 (0 to 0)	0 (0 to 0)	0 (0 to 0)	0 (0 to 0)	0 (0 to 0)	70.6 (41.6 to 102.4)	63.1 (35 to 96.6)	74.5 (37.5 to 120.7)
Latin America and Caribbean	Caribbean	Saint Kitts and Nevis	YLDs	Age-standardized	0.1 (0.1 to 0.2)	0.1 (0 to 0.1)	0.2 (0.1 to 0.3)	0.1 (0.1 to 0.2)	0.1 (0 to 0.1)	0.2 (0.1 to 0.2)	-0.1 (-14.9 to 13)	3.1 (-12 to 23.7)	-6.1 (-24.6 to 11.5)
Latin America and Caribbean	Caribbean	Saint Lucia	Incidence	All Ages	0 (0 to 0)	0 (0 to 0)	0 (0 to 0)	0 (0 to 0)	0 (0 to 0)	0 (0 to 0)	61.5 (35 to 92.2)	53.1 (18.4 to 95.7)	72.5 (36.3 to 119.9)
Latin America and Caribbean	Caribbean	Saint Lucia	Incidence	Age-standardized	0.1 (0.1 to 0.2)	0.2 (0.1 to 0.2)	0.1 (0.1 to 0.2)	0.1 (0.1 to 0.1)	0.1 (0.1 to 0.2)	0.1 (0.1 to 0.1)	-25 (-36.2 to -13)	-25.1 (-41.7 to -5.8)	-26.3 (-40.9 to -8.7)
Latin America and Caribbean	Caribbean	Saint Lucia	Prevalence	All Ages	1 (1 to 1)	0 (0 to 0)	0 (0 to 1)	1 (1 to 2)	1 (0 to 1)	1 (1 to 1)	98 (67.8 to 131.3)	67 (31.7 to 102.2)	125.7 (82.4 to 178.5)
Latin America and Caribbean	Caribbean	Saint Lucia	Prevalence	Age-standardized	0.9 (0.7 to 1.1)	0.7 (0.6 to 0.9)	1 (0.8 to 1.4)	0.7 (0.6 to 0.9)	0.5 (0.4 to 0.7)	0.9 (0.7 to 1.2)	-17.6 (-30.5 to -4.2)	-26.9 (-41.3 to -11.5)	-12.1 (-30 to 7.4)
Latin America and Caribbean	Caribbean	Saint Lucia	Deaths	All Ages	0 (0 to 0)	0 (0 to 0)	0 (0 to 0)	0 (0 to 0)	0 (0 to 0)	0 (0 to 0)	-28.4 (-47.2 to 0.5)	-51.2 (-67.6 to -23)	38.8 (2.9 to 91.4)
Latin America and Caribbean	Caribbean	Saint Lucia	Deaths	Age-standardized	0.1 (0.1 to 0.2)	0.2 (0.1 to 0.2)	0.1 (0.1 to 0.1)	0 (0 to 0.1)	0 (0 to 0)	0 (0 to 0.1)	-72.2 (-79.6 to -60.9)	-79.9 (-86.7 to -68.5)	-51 (-64.1 to -33.3)
Latin America and Caribbean	Caribbean	Saint Lucia	DALYs	All Ages	3 (2 to 3)	2 (1 to 2)	1 (1 to 1)	2 (2 to 2)	1 (1 to 1)	1 (1 to 1)	-24.9 (-42.7 to 2.2)	-49.7 (-64.9 to -25)	40.3 (7.3 to 84.1)
Latin America and Caribbean	Caribbean	Saint Lucia	DALYs	Age-standardized	2.9 (2.2 to 3.5)	3.8 (2.6 to 4.9)	1.8 (1.5 to 2.1)	0.9 (0.8 to 1.2)	0.9 (0.6 to 1.1)	1.1 (0.8 to 1.3)	-67.2 (-75.1 to -56)	-77.4 (-84.3 to -66.1)	-41.5 (-54.7 to -23.5)
Latin America and Caribbean	Caribbean	Saint Lucia	YLLs	All Ages	3 (2 to 3)	2 (1 to 2)	1 (1 to 1)	2 (1 to 2)	1 (1 to 1)	1 (1 to 1)	-30.8 (-48.9 to -1.6)	-53.3 (-68.3 to -28.4)	32.1 (-2.8 to 81.5)
Latin America and Caribbean	Caribbean	Saint Lucia	YLLs	Age-standardized	2.7 (2.1 to 3.4)	3.7 (2.5 to 4.8)	1.6 (1.4 to 1.9)	0.8 (0.6 to 1.1)	0.8 (0.6 to 1.1)	0.9 (0.7 to 1.2)	-69.7 (-77.3 to -58)	-79 (-85.8 to -67.6)	-44.4 (-58.4 to -25.1)
Latin America and Caribbean	Caribbean	Saint Lucia	YLDs	All Ages	0 (0 to 0)	0 (0 to 0)	0 (0 to 0)	0 (0 to 0)	0 (0 to 0)	0 (0 to 0)	98 (68.3 to 130.7)	67.4 (33 to 102.3)	125.8 (82.7 to 178.3)
Latin America and Caribbean	Caribbean	Saint Lucia	YLDs	Age-standardized	0.1 (0.1 to 0.2)	0.1 (0.1 to 0.2)	0.2 (0.1 to 0.3)	0.1 (0.1 to 0.2)	0.1 (0.1 to 0.1)	0.1 (0.1 to 0.2)	-17.6 (-30.3 to -4.4)	-26.7 (-41 to -11.6)	-12.1 (-29.9 to 7.2)
Latin America and Caribbean	Caribbean	Saint Vincent and the Grenadines	Incidence	All Ages	0 (0 to 0)	0 (0 to 0)	0 (0 to 0)	0 (0 to 0)	0 (0 to 0)	0 (0 to 0)	82.5 (55.2 to 114.6)	47.3 (14.3 to 90.8)	104.8 (71.1 to 143.1)
Latin America and Caribbean	Caribbean	Saint Vincent and the Grenadines	Incidence	Age-standardized	0.1 (0.1 to 0.2)	0.1 (0.1 to 0.1)	0.2 (0.1 to 0.2)	0.2 (0.1 to 0.2)	0.1 (0.1 to 0.1)	0.2 (0.2 to 0.3)	12.7 (0.5 to 27.4)	3 (-17.6 to 31.2)	8 (-6.8 to 22.8)
Latin America and Caribbean	Caribbean	Saint Vincent and the Grenadines	Prevalence	All Ages	1 (1 to 1)	0 (0 to 0)	0 (0 to 1)	1 (1 to 2)	0 (0 to 0)	1 (1 to 1)	113.1 (85.3 to 144)	66.5 (38.4 to 93.5)	129.6 (93.1 to 174.2)
Latin America and Caribbean	Caribbean	Saint Vincent and the Grenadines	Prevalence	Age-standardized	0.9 (0.7 to 1.1)	0.4 (0.3 to 0.5)	1.5 (1.2 to 1.9)	1.1 (0.9 to 1.4)	0.4 (0.3 to 0.6)	1.7 (1.3 to 2.2)	21.4 (5.7 to 37)	7.2 (-9.4 to 23.9)	12.3 (-4.7 to 31.7)
Latin America and Caribbean	Caribbean	Saint Vincent and the Grenadines	Deaths	All Ages	0 (0 to 0)	0 (0 to 0)	0 (0 to 0)	0 (0 to 0)	0 (0 to 0)	0 (0 to 0)	109.6 (59.4 to 171.4)	-17.3 (-48.9 to 33.3)	138.5 (76.6 to 215.1)
Latin America and Caribbean	Caribbean	Saint Vincent and the Grenadines	Deaths	Age-standardized	0.1 (0.1 to 0.1)	0 (0 to 0.1)	0.3 (0.2 to 0.3)	0.1 (0.1 to 0.2)	0 (0 to 0)	0.3 (0.2 to 0.3)	19.9 (-8.9 to 55.4)	-48.1 (-68 to -16.8)	10.9 (-17.4 to 45.2)
Latin America and Caribbean	Caribbean	Saint Vincent and the Grenadines	DALYs	All Ages	2 (2 to 3)	0 (0 to 0)	2 (1 to 2)	4 (3 to 5)	0 (0 to 0)	4 (3 to 5)	92.3 (47.3 to 146.1)	-10.4 (-41.2 to 30.4)	114.1 (60.1 to 180)
Latin America and Caribbean	Caribbean	Saint Vincent and the Grenadines	DALYs	Age-standardized	2.7 (2.3 to 3.2)	0.9 (0.7 to 1.2)	5 (4.2 to 6.1)	3.2 (2.6 to 3.8)	0.5 (0.4 to 0.7)	5.9 (4.8 to 7.1)	18.9 (-8.8 to 52.2)	-42.7 (-62.6 to -16.4)	16.3 (-13 to 49.8)
Latin America and Caribbean	Caribbean	Saint Vincent and the Grenadines	YLLs	All Ages	2 (2 to 2)	0 (0 to 0)	2 (1 to 2)	4 (3 to 5)	0 (0 to 0)	4 (3 to 4)	91.3 (44.2 to 147.8)	-16.5 (-47.5 to 27.4)	113.4 (57.6 to 180.8)
Latin America and Caribbean	Caribbean	Saint Vincent and the Grenadines	YLLs	Age-standardized	2.5 (2.1 to 3)	0.8 (0.6 to 1.1)	4.8 (3.9 to 5.8)	3 (2.4 to 3.7)	0.5 (0.3 to 0.6)	5.6 (4.5 to 6.8)	18.8 (-10.2 to 54.1)	-46.5 (-66.4 to -18.3)	16.5 (-13.9 to 52.2)
Latin America and Caribbean	Caribbean	Saint Vincent and the Grenadines	YLDs	All Ages	0 (0 to 0)	0 (0 to 0)	0 (0 to 0)	0 (0 to 0)	0 (0 to 0)	0 (0 to 0)	112.4 (85.4 to 142.9)	66 (38.2 to 93)	128.5 (92.6 to 172)
Latin America and Caribbean	Caribbean	Saint Vincent and the Grenadines	YLDs	Age-standardized	0.1 (0.1 to 0.2)	0.1 (0 to 0.1)	0.2 (0.2 to 0.4)	0.2 (0.1 to 0.3)	0.1 (0 to 0.1)	0.3 (0.2 to 0.4)	21 (5.6 to 36.3)	6.9 (-9.5 to 23.5)	11.6 (-4.9 to 30.6)
Latin America and Caribbean	Caribbean	Suriname	Incidence	All Ages	1 (0 to 1)	0 (0 to 0)	0 (0 to 0)	1 (1 to 1)	0 (0 to 1)	1 (0 to 1)	65.5 (41.1 to 94.9)	94.8 (50.8 to 149)	48.8 (22.6 to 79.1)
Latin America and Caribbean	Caribbean	Suriname	Incidence	Age-standardized	0.2 (0.2 to 0.3)	0.2 (0.1 to 0.2)	0.3 (0.2 to 0.4)	0.2 (0.1 to 0.2)	0.1 (0.1 to 0.2)	0.2 (0.2 to 0.3)	-22.8 (-34 to -8.5)	-10.2 (-30.3 to 15.5)	-27.9 (-39.8 to -14.4)
Latin America and Caribbean	Caribbean	Suriname	Prevalence	All Ages	4 (3 to 5)	1 (1 to 2)	3 (2 to 3)	6 (5 to 8)	2 (2 to 3)	4 (3 to 5)	51.1 (27.8 to 83.9)	68.4 (35.6 to 125.1)	43 (12.6 to 80.9)
Latin America and Caribbean	Caribbean	Suriname	Prevalence	Age-standardized	1.6 (1.3 to 2)	0.9 (0.7 to 1.2)	2.3 (1.8 to 2.9)	1.1 (0.8 to 1.4)	0.7 (0.5 to 0.9)	1.5 (1.1 to 2)	-33.9 (-44.1 to -18)	-27.9 (-43 to -2.5)	-34.7 (-48.8 to -17.6)
Latin America and Caribbean	Caribbean	Suriname	Deaths	All Ages	1 (1 to 2)	0 (0 to 1)	1 (1 to 1)	1 (1 to 2)	0 (0 to 0)	1 (1 to 1)	-9.5 (-35.8 to 27.1)	-32.5 (-58.3 to 10)	3.4 (-30.6 to 54.6)
Latin America and Caribbean	Caribbean	Suriname	Deaths	Age-standardized	0.5 (0.4 to 0.6)	0.3 (0.2 to 0.5)	0.7 (0.6 to 1)	0.2 (0.2 to 0.3)	0.1 (0.1 to 0.1)	0.4 (0.3 to 0.5)	-59.1 (-71.1 to -42.2)	-70.2 (-81.6 to -50.9)	-51.8 (-68 to -27.2)
Latin America and Caribbean	Caribbean	Suriname	DALYs	All Ages	33 (26 to 42)	12 (9 to 16)	21 (16 to 28)	30 (22 to 38)	8 (6 to 11)	21 (15 to 29)	-11.2 (-36.8 to 25.9)	-31.9 (-57.2 to 7.6)	0.6 (-32.3 to 52.7)
Latin America and Caribbean	Caribbean	Suriname	DALYs	Age-standardized	11.5 (9.3 to 14.3)	8 (5.6 to 10.8)	15.4 (12.1 to 20)	5 (3.8 to 6.5)	2.6 (1.8 to 3.6)	7.9 (5.7 to 10.4)	-56.3 (-69 to -38.9)	-67.2 (-79.4 to -48.6)	-48.9 (-65.4 to -24.9)
Latin America and Caribbean	Caribbean	Suriname	YLLs	All Ages	33 (26 to 41)	12 (8 to 16)	21 (16 to 28)	29 (21 to 37)	8 (5 to 11)	21 (15 to 28)	-12.4 (-38.4 to 25.5)	-33.6 (-58.9 to 6.7)	-0.3 (-33.4 to 52.4)
Latin America and Caribbean	Caribbean	Suriname	YLLs	Age-standardized	11.2 (9 to 14)	7.8 (5.5 to 10.6)	15.1 (11.8 to 19.7)	4.8 (3.6 to 6.3)	2.5 (1.7 to 3.5)	7.6 (5.4 to 10.2)	-56.9 (-69.6 to -38.8)	-68 (-79.9 to -49.2)	-49.3 (-66 to -25)
Latin America and Caribbean	Caribbean	Suriname	YLDs	All Ages	1 (0 to 1)	0 (0 to 0)	0 (0 to 1)	1 (1 to 1)	0 (0 to 1)	1 (0 to 1)	53 (29.9 to 85.3)	70.4 (38.3 to 125.4)	44.9 (15.3 to 82.1)
Latin America and Caribbean	Caribbean	Suriname	YLDs	Age-standardized	0.3 (0.2 to 0.4)	0.2 (0.1 to 0.2)	0.4 (0.2 to 0.5)	0.2 (0.1 to 0.3)	0.1 (0.1 to 0.2)	0.2 (0.2 to 0.4)	-33 (-43.1 to -17.4)	-27.1 (-41.6 to -2.2)	-33.8 (-47.4 to -17)
Latin America and Caribbean	Caribbean	Trinidad and Tobago	Incidence	All Ages	1 (1 to 1)	0 (0 to 1)	0 (0 to 1)	2 (1 to 2)	1 (1 to 1)	1 (1 to 1)	99 (59.2 to 144.1)	105.2 (44.4 to 186.8)	93.3 (55.6 to 137.5)
Latin America and Caribbean	Caribbean	Trinidad and Tobago	Incidence	Age-standardized	0.1 (0.1 to 0.1)	0.1 (0 to 0.1)	0.1 (0.1 to 0.1)	0.1 (0.1 to 0.1)	0.1 (0.1 to 0.1)	0.1 (0.1 to 0.1)	15.1 (-1.2 to 33.5)	21.2 (-11.9 to 59.5)	9 (-7 to 26.6)
Latin America and Caribbean	Caribbean	Trinidad and Tobago	Prevalence	All Ages	5 (4 to 6)	2 (1 to 2)	3 (2 to 4)	11 (9 to 14)	4 (3 to 5)	7 (5 to 10)	136.4 (103.8 to 170.8)	139.8 (100 to 190)	134.5 (90.5 to 176.4)
Latin America and Caribbean	Caribbean	Trinidad and Tobago	Prevalence	Age-standardized	0.5 (0.4 to 0.7)	0.4 (0.3 to 0.5)	0.7 (0.5 to 0.9)	0.6 (0.5 to 0.8)	0.4 (0.3 to 0.5)	0.8 (0.6 to 1.1)	18.4 (4.6 to 33.8)	19.1 (0 to 41.2)	17.5 (-2.8 to 38)
Latin America and Caribbean	Caribbean	Trinidad and Tobago	Deaths	All Ages	0 (0 to 0)	0 (0 to 0)	0 (0 to 0)	0 (0 to 0)	0 (0 to 0)	0 (0 to 0)	23 (-15.6 to 72.7)	52.7 (-7.3 to 147.8)	19.6 (-20 to 75.3)
Latin America and Caribbean	Caribbean	Trinidad and Tobago	Deaths	Age-standardized	0 (0 to 0)	0 (0 to 0)	0 (0 to 0)	0 (0 to 0)	0 (0 to 0)	0 (0 to 0)	-43.1 (-60.1 to -21)	-36.3 (-60.6 to 1.5)	-47.1 (-63.3 to -25.1)
Latin America and Caribbean	Caribbean	Trinidad and Tobago	DALYs	All Ages	5 (4 to 6)	1 (0 to 1)	4 (3 to 5)	6 (5 to 8)	1 (1 to 1)	5 (4 to 7)	30.8 (-2.2 to 76.2)	82.2 (43.1 to 126.8)	24.3 (-10.6 to 73.2)
Latin America and Caribbean	Caribbean	Trinidad and Tobago	DALYs	Age-standardized	0.5 (0.4 to 0.6)	0.1 (0.1 to 0.2)	0.9 (0.8 to 1.1)	0.4 (0.3 to 0.5)	0.1 (0.1 to 0.1)	0.6 (0.5 to 0.9)	-25.8 (-44.6 to -1)	-8.8 (-27.7 to 12.6)	-30.3 (-49.5 to -4.7)
Latin America and Caribbean	Caribbean	Trinidad and Tobago	YLLs	All Ages	4 (3 to 5)	0 (0 to 0)	4 (3 to 5)	4 (3 to 6)	0 (0 to 1)	4 (3 to 6)	11.4 (-25.5 to 61.7)	29.3 (-16.9 to 100.6)	10.1 (-27.7 to 61.7)
Latin America and Caribbean	Caribbean	Trinidad and Tobago	YLLs	Age-standardized	0.4 (0.4 to 0.5)	0.1 (0 to 0.1)	0.8 (0.7 to 1)	0.3 (0.2 to 0.4)	0 (0 to 0.1)	0.5 (0.4 to 0.7)	-34.6 (-55.9 to -6.2)	-33.6 (-57 to 1.2)	-36.8 (-57.7 to -8.1)
Latin America and Caribbean	Caribbean	Trinidad and Tobago	YLDs	All Ages	1 (0 to 1)	0 (0 to 0)	0 (0 to 1)	2 (1 to 3)	1 (0 to 1)	1 (1 to 2)	136.4 (104 to 170.6)	139.8 (100.1 to 189.8)	134.6 (90.6 to 176.2)
Latin America and Caribbean	Caribbean	Trinidad and Tobago	YLDs	Age-standardized	0.1 (0.1 to 0.1)	0.1 (0 to 0.1)	0.1 (0.1 to 0.2)	0.1 (0.1 to 0.2)	0.1 (0 to 0.1)	0.1 (0.1 to 0.2)	18.4 (4.6 to 33.6)	19 (0 to 41.1)	17.5 (-2.8 to 37.6)
Latin America and Caribbean	Caribbean	United States Virgin Islands	Incidence	All Ages	0 (0 to 0)	0 (0 to 0)	0 (0 to 0)	1 (0 to 1)	0 (0 to 0)	0 (0 to 0)	135.6 (91.9 to 197.5)	150.7 (85.5 to 248.8)	117.4 (70.2 to 175.4)
Latin America and Caribbean	Caribbean	United States Virgin Islands	Incidence	Age-standardized	0.2 (0.2 to 0.3)	0.2 (0.2 to 0.3)	0.3 (0.2 to 0.3)	0.4 (0.3 to 0.5)	0.4 (0.3 to 0.5)	0.3 (0.2 to 0.4)	48 (23.5 to 79.8)	68.7 (27.1 to 135.8)	24.7 (2.8 to 50.9)
Latin America and Caribbean	Caribbean	United States Virgin Islands	Prevalence	All Ages	1 (1 to 1)	0 (0 to 0)	0 (0 to 1)	2 (1 to 2)	1 (1 to 1)	1 (1 to 1)	122.5 (94.7 to 158.6)	147.4 (102.9 to 201.5)	104 (72.6 to 148.1)
Latin America and Caribbean	Caribbean	United States Virgin Islands	Prevalence	Age-standardized	0.9 (0.7 to 1.1)	0.7 (0.5 to 0.9)	1.1 (0.9 to 1.4)	1 (0.8 to 1.3)	0.9 (0.7 to 1.2)	1.2 (0.9 to 1.5)	19 (4.9 to 35.1)	34 (10.5 to 61.5)	8.8 (-7.4 to 28.8)
Latin America and Caribbean	Caribbean	United States Virgin Islands	Deaths	All Ages	0 (0 to 0)	0 (0 to 0)	0 (0 to 0)	0 (0 to 0)	0 (0 to 0)	0 (0 to 0)	36.8 (-5 to 106.3)	-15.6 (-50.4 to 39.1)	59.8 (4.2 to 169)
Latin America and Caribbean	Caribbean	United States Virgin Islands	Deaths	Age-standardized	0.1 (0.1 to 0.2)	0.1 (0.1 to 0.1)	0.2 (0.2 to 0.3)	0.1 (0.1 to 0.1)	0 (0 to 0)	0.2 (0.1 to 0.2)	-40.3 (-58.6 to -11)	-62.9 (-77.8 to -38.7)	-29.4 (-53.8 to 18.1)
Latin America and Caribbean	Caribbean	United States Virgin Islands	DALYs	All Ages	3 (2 to 4)	1 (1 to 1)	2 (1 to 3)	3 (2 to 4)	1 (0 to 1)	3 (2 to 3)	11.8 (-22.5 to 70.3)	-25.1 (-49.8 to 15.7)	27 (-18.4 to 114.5)
Latin America and Caribbean	Caribbean	United States Virgin Islands	DALYs	Age-standardized	3.2 (2.4 to 4.1)	1.7 (1.2 to 2.3)	5 (3.6 to 6.8)	1.9 (1.5 to 2.5)	0.7 (0.5 to 1)	3.5 (2.5 to 4.7)	-40.6 (-57.7 to -11.5)	-59.1 (-72.8 to -38.4)	-31.4 (-53.7 to 11.6)
Latin America and Caribbean	Caribbean	United States Virgin Islands	YLLs	All Ages	3 (2 to 4)	1 (1 to 1)	2 (1 to 3)	3 (2 to 4)	0 (0 to 1)	2 (2 to 3)	7.1 (-27 to 67.2)	-35.9 (-60.4 to 6)	24.3 (-21.2 to 115.9)
Latin America and Caribbean	Caribbean	United States Virgin Islands	YLLs	Age-standardized	3.1 (2.3 to 4)	1.6 (1.1 to 2.2)	4.9 (3.5 to 6.6)	1.7 (1.3 to 2.3)	0.6 (0.4 to 0.8)	3.3 (2.4 to 4.5)	-43.2 (-60.6 to -12.9)	-65.3 (-78.5 to -44.1)	-32.8 (-55.3 to 11.7)
Latin America and Caribbean	Caribbean	United States Virgin Islands	YLDs	All Ages	0 (0 to 0)	0 (0 to 0)	0 (0 to 0)	0 (0 to 0)	0 (0 to 0)	0 (0 to 0)	122.6 (95.2 to 158.8)	147 (102.9 to 201.1)	104.4 (73.3 to 147.8)
Latin America and Caribbean	Caribbean	United States Virgin Islands	YLDs	Age-standardized	0.1 (0.1 to 0.2)	0.1 (0.1 to 0.2)	0.2 (0.1 to 0.3)	0.2 (0.1 to 0.2)	0.1 (0.1 to 0.2)	0.2 (0.1 to 0.3)	18.7 (4.9 to 34.6)	33.5 (10.4 to 60.8)	8.7 (-7.1 to 28.5)
		Andean Latin America	Incidence	All Ages	123 (101 to 148)	33 (26 to 42)	90 (73 to 110)	293 (244 to 352)	103 (81 to 129)	190 (160 to 224)	139.4 (106.5 to 177.8)	214.1 (156.6 to 274.7)	111.9 (79.7 to 149.6)
		Andean Latin America	Incidence	Age-standardized	0.5 (0.4 to 0.6)	0.3 (0.2 to 0.3)	0.7 (0.6 to 0.9)	0.5 (0.4 to 0.6)	0.4 (0.3 to 0.4)	0.7 (0.6 to 0.8)	2 (-9.9 to 15.6)	28 (9.9 to 47.3)	-7.7 (-20.5 to 6)
		Andean Latin America	Prevalence	All Ages	681 (561 to 880)	189 (149 to 238)	492 (404 to 672)	2,730 (2,201 to 3,344)	919 (688 to 1,214)	1,811 (1,492 to 2,188)	300.5 (232 to 348.3)	385.8 (323.3 to 456.5)	267.8 (195.5 to 319.4)
		Andean Latin America	Prevalence	Age-standardized	3.3 (2.7 to 4.3)	1.9 (1.5 to 2.4)	4.9 (4 to 6.7)	5 (4 to 6.2)	3.2 (2.4 to 4.3)	6.9 (5.7 to 8.3)	50.5 (24.3 to 66.9)	73.6 (53.2 to 97.7)	42.1 (13.4 to 61.7)
		Andean Latin America	Deaths	All Ages	95 (72 to 117)	23 (16 to 31)	72 (54 to 92)	121 (88 to 161)	41 (27 to 58)	81 (56 to 109)	27.7 (-11.4 to 81.5)	80.4 (11.5 to 180.9)	11.2 (-28.3 to 66.4)
		Andean Latin America	Deaths	Age-standardized	0.5 (0.4 to 0.6)	0.2 (0.2 to 0.3)	0.7 (0.5 to 0.9)	0.2 (0.2 to 0.3)	0.1 (0.1 to 0.2)	0.3 (0.2 to 0.4)	-52.1 (-67.1 to -32)	-33.2 (-59.3 to 6)	-57.7 (-72.9 to -36.2)
		Andean Latin America	DALYs	All Ages	2,788 (2,175 to 3,461)	675 (484 to 900)	2,113 (1,582 to 2,704)	3,027 (2,311 to 3,950)	938 (666 to 1,288)	2,089 (1,531 to 2,793)	8.6 (-23.8 to 51.7)	39 (-8.2 to 108.6)	-1.1 (-34.2 to 43.1)
		Andean Latin America	DALYs	Age-standardized	11.8 (9.2 to 14.6)	5.4 (3.9 to 7.3)	18.5 (13.9 to 23.6)	5.3 (4.1 to 7)	3.2 (2.3 to 4.4)	7.6 (5.6 to 10.2)	-54.8 (-67.9 to -37.1)	-40.9 (-60.8 to -12.3)	-58.8 (-72.3 to -41)
		Andean Latin America	YLLs	All Ages	2,678 (2,060 to 3,353)	644 (456 to 867)	2,034 (1,504 to 2,627)	2,609 (1,890 to 3,506)	800 (530 to 1,137)	1,809 (1,263 to 2,495)	-2.6 (-34.3 to 40.8)	24.2 (-22.9 to 95.3)	-11.1 (-43.8 to 33.8)
		Andean Latin America	YLLs	Age-standardized	11.3 (8.6 to 14.2)	5.1 (3.7 to 7.1)	17.8 (13.1 to 22.8)	4.6 (3.3 to 6.1)	2.7 (1.8 to 3.9)	6.6 (4.6 to 9.1)	-59.5 (-72.5 to -41.5)	-46.9 (-66.8 to -18.1)	-63 (-76.5 to -44.8)
		Andean Latin America	YLDs	All Ages	109 (72 to 160)	30 (20 to 44)	79 (51 to 120)	418 (278 to 598)	138 (86 to 207)	281 (184 to 409)	282.3 (219.1 to 335.2)	352.3 (275 to 443.9)	255.3 (184.9 to 316.8)
		Andean Latin America	YLDs	Age-standardized	0.5 (0.4 to 0.8)	0.3 (0.2 to 0.4)	0.8 (0.5 to 1.2)	0.8 (0.5 to 1.1)	0.5 (0.3 to 0.7)	1.1 (0.7 to 1.6)	43.1 (18.7 to 63)	61.3 (33.5 to 95.1)	36.7 (8.4 to 60.5)
Latin America and Caribbean	Andean Latin America	Bolivia (Plurinational State of)	Incidence	All Ages	18 (14 to 22)	5 (4 to 6)	13 (10 to 17)	59 (48 to 72)	19 (15 to 24)	40 (32 to 51)	234.5 (191.9 to 285.5)	290.1 (209.6 to 380.1)	213.3 (169.8 to 274.5)
Latin America and Caribbean	Andean Latin America	Bolivia (Plurinational State of)	Incidence	Age-standardized	0.5 (0.4 to 0.6)	0.3 (0.2 to 0.3)	0.7 (0.6 to 0.9)	0.6 (0.5 to 0.7)	0.4 (0.3 to 0.5)	0.9 (0.7 to 1.1)	32.7 (19 to 50.1)	49.7 (25.6 to 77)	24.1 (9.7 to 47)
Latin America and Caribbean	Andean Latin America	Bolivia (Plurinational State of)	Prevalence	All Ages	89 (71 to 122)	24 (18 to 33)	65 (51 to 98)	388 (307 to 495)	118 (83 to 163)	270 (214 to 365)	333.9 (270.4 to 410)	380.3 (299.7 to 476.4)	316.4 (243.7 to 417.4)
Latin America and Caribbean	Andean Latin America	Bolivia (Plurinational State of)	Prevalence	Age-standardized	2.8 (2.2 to 3.8)	1.5 (1.1 to 2.1)	4.2 (3.3 to 6.3)	4.6 (3.7 to 5.9)	2.8 (2 to 3.8)	6.6 (5.2 to 8.9)	66.2 (41.5 to 94.6)	81.4 (52.6 to 116.3)	59.3 (31.2 to 98.5)
Latin America and Caribbean	Andean Latin America	Bolivia (Plurinational State of)	Deaths	All Ages	15 (10 to 21)	3 (2 to 5)	12 (7 to 18)	26 (18 to 36)	8 (5 to 13)	18 (11 to 26)	74 (13.8 to 158.4)	146.8 (39.5 to 322.6)	54.1 (-6.8 to 151.4)
Latin America and Caribbean	Andean Latin America	Bolivia (Plurinational State of)	Deaths	Age-standardized	0.5 (0.3 to 0.7)	0.2 (0.1 to 0.3)	0.8 (0.5 to 1.2)	0.3 (0.2 to 0.4)	0.2 (0.1 to 0.3)	0.5 (0.3 to 0.7)	-33.6 (-56.2 to -2.4)	-5.3 (-45.5 to 62.8)	-42.6 (-65 to -4.5)
Latin America and Caribbean	Andean Latin America	Bolivia (Plurinational State of)	DALYs	All Ages	423 (287 to 596)	87 (56 to 131)	336 (209 to 501)	647 (454 to 880)	186 (114 to 283)	461 (302 to 660)	53.2 (0.1 to 129.1)	114.3 (30.3 to 255.1)	37.3 (-16.4 to 122.3)
Latin America and Caribbean	Andean Latin America	Bolivia (Plurinational State of)	DALYs	Age-standardized	12 (8.2 to 16.6)	4.7 (3 to 7.1)	20.2 (12.7 to 29.8)	7.2 (5.1 to 9.8)	4 (2.5 to 6.1)	10.7 (7.1 to 15.3)	-39.4 (-59.9 to -10.7)	-13.4 (-47.4 to 43.8)	-46.7 (-66.8 to -14.1)
Latin America and Caribbean	Andean Latin America	Bolivia (Plurinational State of)	YLLs	All Ages	409 (273 to 581)	83 (52 to 127)	325 (199 to 492)	588 (399 to 829)	169 (98 to 261)	419 (262 to 620)	43.9 (-9.1 to 119.7)	102.8 (17.9 to 249)	28.8 (-23.9 to 114.3)
Latin America and Caribbean	Andean Latin America	Bolivia (Plurinational State of)	YLLs	Age-standardized	11.5 (7.8 to 16.2)	4.4 (2.8 to 6.8)	19.5 (12 to 29.2)	6.5 (4.4 to 9.1)	3.6 (2.1 to 5.6)	9.7 (6.2 to 14.4)	-43.2 (-63.7 to -14.3)	-18 (-52.3 to 41)	-50.2 (-70.3 to -17.2)
Latin America and Caribbean	Andean Latin America	Bolivia (Plurinational State of)	YLDs	All Ages	14 (9 to 22)	4 (2 to 6)	10 (6 to 17)	59 (38 to 89)	18 (11 to 28)	42 (27 to 63)	322.1 (255.2 to 405.9)	362 (263.5 to 473)	307.1 (230.7 to 413.4)
Latin America and Caribbean	Andean Latin America	Bolivia (Plurinational State of)	YLDs	Age-standardized	0.4 (0.3 to 0.7)	0.2 (0.1 to 0.4)	0.7 (0.4 to 1.1)	0.7 (0.4 to 1)	0.4 (0.2 to 0.7)	1 (0.6 to 1.5)	60.2 (34 to 92.8)	72.2 (33.9 to 118.4)	54.6 (24.1 to 95.6)
Latin America and Caribbean	Andean Latin America	Ecuador	Incidence	All Ages	13 (10 to 16)	4 (3 to 5)	9 (8 to 12)	55 (45 to 66)	16 (12 to 21)	38 (32 to 47)	321.7 (254.1 to 397.2)	366.1 (262.2 to 513.3)	305.2 (234.7 to 391.3)
Latin America and Caribbean	Andean Latin America	Ecuador	Incidence	Age-standardized	0.2 (0.2 to 0.3)	0.1 (0.1 to 0.2)	0.3 (0.3 to 0.4)	0.4 (0.3 to 0.4)	0.2 (0.2 to 0.3)	0.5 (0.4 to 0.6)	62.3 (39.4 to 88.3)	77.5 (41.4 to 127.7)	58.4 (31.9 to 90.7)
Latin America and Caribbean	Andean Latin America	Ecuador	Prevalence	All Ages	113 (92 to 143)	24 (19 to 32)	89 (70 to 118)	510 (415 to 624)	139 (100 to 187)	371 (301 to 459)	349.5 (280.8 to 430.8)	472.5 (343.4 to 634.8)	316 (243.9 to 403.6)
Latin America and Caribbean	Andean Latin America	Ecuador	Prevalence	Age-standardized	2.1 (1.7 to 2.6)	0.9 (0.7 to 1.2)	3.3 (2.6 to 4.4)	3.4 (2.8 to 4.2)	1.8 (1.3 to 2.5)	5.2 (4.2 to 6.4)	64.1 (38.2 to 93.6)	104.5 (58.2 to 165.2)	55.5 (27.7 to 88.8)
Latin America and Caribbean	Andean Latin America	Ecuador	Deaths	All Ages	7 (5 to 8)	1 (1 to 2)	5 (4 to 7)	12 (9 to 16)	3 (2 to 4)	9 (6 to 12)	75.6 (21.6 to 157.4)	115.4 (35.8 to 240.3)	64.7 (8.8 to 147.9)
Latin America and Caribbean	Andean Latin America	Ecuador	Deaths	Age-standardized	0.1 (0.1 to 0.2)	0.1 (0 to 0.1)	0.2 (0.2 to 0.3)	0.1 (0.1 to 0.1)	0 (0 to 0.1)	0.1 (0.1 to 0.2)	-35.2 (-54.8 to -7)	-25.3 (-52.6 to 17.1)	-35.5 (-56.8 to -5.3)
Latin America and Caribbean	Andean Latin America	Ecuador	DALYs	All Ages	199 (163 to 242)	38 (29 to 49)	161 (130 to 200)	349 (269 to 465)	82 (62 to 110)	267 (200 to 357)	75.5 (25.2 to 145.5)	118.4 (49.7 to 217.1)	65.5 (13 to 138.4)
Latin America and Caribbean	Andean Latin America	Ecuador	DALYs	Age-standardized	3.4 (2.8 to 4.1)	1.3 (1 to 1.7)	5.5 (4.4 to 6.8)	2.3 (1.8 to 3)	1.1 (0.8 to 1.4)	3.6 (2.7 to 4.8)	-32.2 (-51.6 to -6.1)	-18.5 (-44 to 17.9)	-34.1 (-54 to -5.7)
Latin America and Caribbean	Andean Latin America	Ecuador	YLLs	All Ages	181 (147 to 224)	34 (25 to 45)	147 (116 to 186)	269 (198 to 373)	61 (42 to 87)	209 (147 to 300)	48.6 (-1.2 to 122.7)	79.1 (14.3 to 184.6)	41.6 (-8.6 to 118.7)
Latin America and Caribbean	Andean Latin America	Ecuador	YLLs	Age-standardized	3 (2.5 to 3.8)	1.2 (0.9 to 1.5)	4.9 (3.9 to 6.3)	1.7 (1.3 to 2.4)	0.8 (0.5 to 1.1)	2.8 (2 to 4)	-42.6 (-61.8 to -13.8)	-33.1 (-57.6 to 5.6)	-43.6 (-63.7 to -13.3)
Latin America and Caribbean	Andean Latin America	Ecuador	YLDs	All Ages	18 (12 to 26)	4 (2 to 6)	14 (9 to 21)	80 (52 to 116)	22 (13 to 33)	58 (38 to 84)	347.5 (276.8 to 428.1)	465.5 (337.7 to 623.1)	315.3 (243.2 to 402.8)
Latin America and Caribbean	Andean Latin America	Ecuador	YLDs	Age-standardized	0.3 (0.2 to 0.5)	0.1 (0.1 to 0.2)	0.5 (0.3 to 0.8)	0.5 (0.4 to 0.8)	0.3 (0.2 to 0.4)	0.8 (0.5 to 1.2)	63 (37.2 to 93.2)	101 (54 to 161.7)	55.1 (27.6 to 87.9)
Latin America and Caribbean	Andean Latin America	Peru	Incidence	All Ages	92 (74 to 113)	24 (19 to 31)	67 (53 to 85)	179 (148 to 219)	68 (52 to 87)	112 (91 to 133)	95.3 (60.4 to 138.6)	177.2 (116.5 to 251)	65.6 (31.8 to 105.4)
Latin America and Caribbean	Andean Latin America	Peru	Incidence	Age-standardized	0.6 (0.5 to 0.8)	0.4 (0.3 to 0.5)	0.9 (0.8 to 1.2)	0.6 (0.5 to 0.7)	0.4 (0.3 to 0.5)	0.7 (0.6 to 0.9)	-12.7 (-26.8 to 3.3)	17.6 (-3.9 to 41.6)	-24 (-38 to -8.1)
Latin America and Caribbean	Andean Latin America	Peru	Prevalence	All Ages	479 (390 to 615)	140 (109 to 180)	338 (274 to 469)	1,832 (1,450 to 2,275)	662 (494 to 883)	1,170 (932 to 1,419)	282.7 (206.1 to 338.3)	371.8 (299.6 to 455.2)	245.8 (166.8 to 306.4)
Latin America and Caribbean	Andean Latin America	Peru	Prevalence	Age-standardized	4 (3.3 to 5.2)	2.4 (1.8 to 3.1)	5.7 (4.6 to 8)	5.8 (4.6 to 7.2)	4 (3 to 5.4)	7.7 (6.2 to 9.4)	44.3 (15.1 to 64.2)	67.5 (42.9 to 97.3)	35 (2.5 to 59.1)
Latin America and Caribbean	Andean Latin America	Peru	Deaths	All Ages	73 (53 to 96)	18 (12 to 26)	55 (39 to 74)	84 (57 to 118)	30 (19 to 45)	54 (34 to 79)	13.9 (-26.7 to 73)	65.7 (-6.9 to 183.3)	-2.9 (-43.2 to 54.9)
Latin America and Caribbean	Andean Latin America	Peru	Deaths	Age-standardized	0.6 (0.4 to 0.8)	0.3 (0.2 to 0.4)	1 (0.7 to 1.3)	0.3 (0.2 to 0.4)	0.2 (0.1 to 0.3)	0.4 (0.2 to 0.5)	-57 (-72.7 to -33.9)	-38.4 (-66 to 9.1)	-62.9 (-78.1 to -40.6)
Latin America and Caribbean	Andean Latin America	Peru	DALYs	All Ages	2,166 (1,593 to 2,799)	550 (387 to 765)	1,616 (1,159 to 2,154)	2,031 (1,461 to 2,799)	669 (459 to 970)	1,361 (925 to 1,926)	-6.2 (-37.5 to 38.6)	21.7 (-24.5 to 98.7)	-15.7 (-47.6 to 31.2)
Latin America and Caribbean	Andean Latin America	Peru	DALYs	Age-standardized	15.6 (11.3 to 20.3)	7.5 (5.3 to 10.6)	24.1 (17 to 32.1)	6.3 (4.5 to 8.7)	4 (2.7 to 5.8)	8.7 (6 to 12.3)	-59.7 (-72.9 to -40.7)	-46.6 (-66.9 to -12.5)	-63.8 (-77.5 to -43.4)
Latin America and Caribbean	Andean Latin America	Peru	YLLs	All Ages	2,089 (1,524 to 2,722)	527 (367 to 746)	1,561 (1,103 to 2,092)	1,752 (1,182 to 2,510)	571 (359 to 853)	1,181 (756 to 1,725)	-16.1 (-46.7 to 30.3)	8.3 (-37 to 86.7)	-24.4 (-55.8 to 22.5)
Latin America and Caribbean	Andean Latin America	Peru	YLLs	Age-standardized	15 (10.7 to 19.5)	7.1 (4.9 to 10.2)	23.2 (16.2 to 31.2)	5.4 (3.6 to 7.8)	3.4 (2.1 to 5.1)	7.5 (4.8 to 11)	-63.9 (-77 to -44.6)	-52.1 (-72.3 to -16.5)	-67.5 (-80.9 to -47.4)
Latin America and Caribbean	Andean Latin America	Peru	YLDs	All Ages	77 (51 to 115)	23 (15 to 33)	55 (35 to 83)	279 (183 to 404)	98 (61 to 152)	181 (116 to 263)	260 (187.2 to 328.2)	331.7 (238.2 to 450.7)	230.2 (149.5 to 311.1)
Latin America and Caribbean	Andean Latin America	Peru	YLDs	Age-standardized	0.7 (0.4 to 1)	0.4 (0.2 to 0.6)	0.9 (0.6 to 1.4)	0.9 (0.6 to 1.3)	0.6 (0.4 to 0.9)	1.2 (0.8 to 1.7)	35.3 (7.9 to 60.9)	53.6 (19.1 to 97.8)	28.2 (-4 to 60.5)
		Central Latin America	Incidence	All Ages	1,262 (1,082 to 1,473)	195 (161 to 235)	1,067 (899 to 1,266)	2,187 (1,897 to 2,556)	422 (348 to 508)	1,766 (1,519 to 2,091)	73.4 (61 to 86)	116.1 (98.1 to 134.6)	65.6 (52.6 to 77.5)
		Central Latin America	Incidence	Age-standardized	1.3 (1.1 to 1.5)	0.4 (0.3 to 0.4)	2.2 (1.9 to 2.7)	0.9 (0.8 to 1)	0.3 (0.3 to 0.4)	1.5 (1.3 to 1.8)	-31.1 (-35.9 to -25.9)	-12.1 (-17.6 to -6.7)	-32.7 (-37.8 to -27.2)
		Central Latin America	Prevalence	All Ages	15,062 (12,646 to 17,695)	1,925 (1,636 to 2,266)	13,137 (10,839 to 15,690)	29,621 (25,295 to 34,775)	4,823 (4,044 to 5,757)	24,798 (21,044 to 29,414)	96.7 (81.7 to 111.9)	150.6 (136.3 to 167.1)	88.8 (72.5 to 104.9)
		Central Latin America	Prevalence	Age-standardized	17.3 (14.4 to 20.6)	4.1 (3.5 to 4.9)	31.5 (25.9 to 38.2)	12.3 (10.5 to 14.5)	3.7 (3.1 to 4.5)	22.2 (18.8 to 26.5)	-28.7 (-34.5 to -22.8)	-9.4 (-14.3 to -4.1)	-29.4 (-35.8 to -22.6)
		Central Latin America	Deaths	All Ages	246 (230 to 260)	30 (23 to 34)	216 (203 to 228)	260 (220 to 307)	36 (28 to 44)	225 (188 to 268)	5.7 (-9.9 to 23)	19 (-4.4 to 50.5)	3.8 (-12.6 to 22.1)
		Central Latin America	Deaths	Age-standardized	0.3 (0.3 to 0.3)	0.1 (0.1 to 0.1)	0.6 (0.5 to 0.6)	0.1 (0.1 to 0.1)	0 (0 to 0)	0.2 (0.2 to 0.3)	-64 (-69.3 to -58.3)	-62.2 (-69.7 to -52.2)	-62.7 (-68.5 to -56.1)
		Central Latin America	DALYs	All Ages	8,178 (7,352 to 9,106)	1,007 (824 to 1,171)	7,171 (6,464 to 7,977)	9,975 (8,231 to 12,006)	1,452 (1,147 to 1,805)	8,523 (6,999 to 10,364)	22 (7.6 to 37.1)	44.2 (23.5 to 69.2)	18.9 (4 to 34.6)
		Central Latin America	DALYs	Age-standardized	9.2 (8.3 to 10.2)	2.1 (1.7 to 2.5)	16.7 (15.1 to 18.6)	4.2 (3.4 to 5)	1.1 (0.9 to 1.4)	7.7 (6.3 to 9.3)	-54.5 (-59.7 to -48.7)	-46.7 (-54.3 to -37.2)	-54.2 (-59.7 to -48.2)
		Central Latin America	YLLs	All Ages	6,007 (5,636 to 6,321)	707 (544 to 796)	5,300 (4,993 to 5,587)	5,615 (4,744 to 6,626)	700 (567 to 868)	4,915 (4,063 to 5,866)	-6.5 (-20.7 to 10.3)	-1 (-20 to 26.2)	-7.3 (-23.4 to 10.3)
		Central Latin America	YLLs	Age-standardized	6.7 (6.3 to 7)	1.5 (1.1 to 1.7)	12.3 (11.5 to 13)	2.4 (2 to 2.8)	0.5 (0.4 to 0.7)	4.5 (3.7 to 5.3)	-64.8 (-70.1 to -58.5)	-63 (-70.2 to -52.8)	-63.8 (-70 to -57)
		Central Latin America	YLDs	All Ages	2,171 (1,453 to 3,037)	300 (199 to 428)	1,871 (1,248 to 2,612)	4,360 (2,890 to 6,125)	752 (499 to 1,080)	3,608 (2,401 to 5,084)	100.8 (83.4 to 118.5)	150.5 (134.9 to 167.3)	92.9 (74.2 to 112.6)
		Central Latin America	YLDs	Age-standardized	2.5 (1.6 to 3.4)	0.6 (0.4 to 0.9)	4.4 (2.9 to 6.2)	1.8 (1.2 to 2.5)	0.6 (0.4 to 0.8)	3.2 (2.1 to 4.5)	-26.6 (-33.1 to -19.7)	-9.3 (-14.8 to -3.7)	-27.3 (-34.6 to -19.7)
Latin America and Caribbean	Central Latin America	Colombia	Incidence	All Ages	93 (79 to 110)	26 (19 to 33)	68 (57 to 79)	210 (172 to 263)	50 (39 to 64)	160 (129 to 206)	125.3 (95.8 to 166.7)	94.9 (51.6 to 146.9)	137 (102.6 to 192.8)
Latin America and Caribbean	Central Latin America	Colombia	Incidence	Age-standardized	0.5 (0.4 to 0.5)	0.2 (0.2 to 0.3)	0.7 (0.6 to 0.8)	0.4 (0.3 to 0.5)	0.2 (0.1 to 0.2)	0.7 (0.5 to 0.8)	-12.8 (-23.3 to 3)	-28.7 (-44.1 to -11.5)	-3.1 (-16.1 to 20)
Latin America and Caribbean	Central Latin America	Colombia	Prevalence	All Ages	940 (779 to 1,126)	215 (164 to 277)	725 (589 to 878)	2,737 (2,231 to 3,398)	577 (436 to 762)	2,160 (1,710 to 2,725)	191.2 (149.6 to 243)	168.4 (114.6 to 236.8)	198 (147.5 to 266.8)
Latin America and Caribbean	Central Latin America	Colombia	Prevalence	Age-standardized	5.1 (4.3 to 6.1)	2.4 (1.8 to 3)	8 (6.6 to 9.6)	5.2 (4.2 to 6.4)	2 (1.5 to 2.6)	9 (7.1 to 11.4)	1.5 (-12.8 to 19.3)	-15.9 (-33.8 to 6.7)	12.2 (-6.1 to 36.1)
Latin America and Caribbean	Central Latin America	Colombia	Deaths	All Ages	62 (55 to 69)	15 (11 to 19)	47 (41 to 52)	72 (53 to 97)	9 (6 to 14)	63 (46 to 84)	15.6 (-14.7 to 56.6)	-39.2 (-59 to -7.5)	33.7 (-3.3 to 85.3)
Latin America and Caribbean	Central Latin America	Colombia	Deaths	Age-standardized	0.4 (0.3 to 0.4)	0.2 (0.1 to 0.2)	0.6 (0.5 to 0.6)	0.1 (0.1 to 0.2)	0 (0 to 0)	0.3 (0.2 to 0.4)	-64.5 (-74 to -52.6)	-84 (-89.3 to -75.8)	-54.8 (-67.3 to -38.1)
Latin America and Caribbean	Central Latin America	Colombia	DALYs	All Ages	1,694 (1,510 to 1,874)	364 (266 to 433)	1,329 (1,184 to 1,481)	1,994 (1,522 to 2,558)	258 (190 to 341)	1,736 (1,316 to 2,259)	17.7 (-9.1 to 51.2)	-29.3 (-48.1 to -4)	30.6 (-0.9 to 71.5)
Latin America and Caribbean	Central Latin America	Colombia	DALYs	Age-standardized	9 (8 to 10)	4 (2.9 to 4.8)	14.3 (12.7 to 15.9)	3.8 (2.9 to 4.9)	0.9 (0.7 to 1.2)	7.2 (5.5 to 9.3)	-57.8 (-67.4 to -45.9)	-77.7 (-83.8 to -69.3)	-49.5 (-61.1 to -33.1)
Latin America and Caribbean	Central Latin America	Colombia	YLLs	All Ages	1,545 (1,370 to 1,718)	331 (235 to 394)	1,215 (1,076 to 1,361)	1,570 (1,139 to 2,092)	167 (113 to 238)	1,403 (1,011 to 1,893)	1.6 (-25.9 to 38.3)	-49.6 (-65.8 to -21.9)	15.5 (-16.7 to 60)
Latin America and Caribbean	Central Latin America	Colombia	YLLs	Age-standardized	8.2 (7.2 to 9.1)	3.6 (2.5 to 4.4)	13 (11.4 to 14.6)	3 (2.2 to 4)	0.6 (0.4 to 0.8)	5.8 (4.2 to 7.9)	-63.4 (-73.4 to -50.1)	-84.1 (-89.2 to -75.2)	-55.2 (-67.5 to -37.7)
Latin America and Caribbean	Central Latin America	Colombia	YLDs	All Ages	148 (96 to 211)	34 (21 to 50)	114 (73 to 163)	424 (272 to 615)	91 (55 to 136)	333 (212 to 497)	185.6 (144.3 to 239.6)	168.3 (114.8 to 236.1)	190.7 (139.6 to 259.3)
Latin America and Caribbean	Central Latin America	Colombia	YLDs	Age-standardized	0.8 (0.5 to 1.1)	0.4 (0.2 to 0.6)	1.3 (0.8 to 1.8)	0.8 (0.5 to 1.2)	0.3 (0.2 to 0.5)	1.4 (0.9 to 2.1)	-0.5 (-15 to 17.4)	-16 (-33.8 to 6.4)	9.3 (-9.4 to 35)
Latin America and Caribbean	Central Latin America	Costa Rica	Incidence	All Ages	5 (4 to 6)	1 (1 to 2)	4 (3 to 5)	15 (12 to 19)	4 (3 to 5)	11 (9 to 14)	207.2 (162.3 to 260.3)	197.8 (132.1 to 276.9)	210.8 (160.4 to 267.8)
Latin America and Caribbean	Central Latin America	Costa Rica	Incidence	Age-standardized	0.2 (0.2 to 0.3)	0.1 (0.1 to 0.2)	0.3 (0.3 to 0.4)	0.3 (0.2 to 0.4)	0.1 (0.1 to 0.2)	0.5 (0.4 to 0.6)	26.9 (12.2 to 45.7)	17.9 (-4.2 to 49.2)	34.1 (15 to 57.1)
Latin America and Caribbean	Central Latin America	Costa Rica	Prevalence	All Ages	62 (50 to 78)	14 (10 to 18)	48 (37 to 62)	221 (179 to 277)	51 (38 to 67)	170 (135 to 215)	256.2 (205.4 to 326.2)	273.1 (198.3 to 368.4)	251.5 (191.6 to 327.3)
Latin America and Caribbean	Central Latin America	Costa Rica	Prevalence	Age-standardized	3.2 (2.6 to 4.1)	1.4 (1 to 1.9)	5.2 (4 to 6.5)	4.3 (3.5 to 5.4)	1.8 (1.4 to 2.4)	7.1 (5.7 to 9)	32.2 (14.4 to 56)	27.9 (1.7 to 61.8)	38.5 (15.6 to 68.9)
Latin America and Caribbean	Central Latin America	Costa Rica	Deaths	All Ages	0 (0 to 0)	0 (0 to 0)	0 (0 to 0)	1 (1 to 2)	0 (0 to 0)	1 (1 to 2)	478.3 (294.3 to 728.1)	173.2 (82.6 to 302.1)	536.6 (325.3 to 836.4)
Latin America and Caribbean	Central Latin America	Costa Rica	Deaths	Age-standardized	0 (0 to 0)	0 (0 to 0)	0 (0 to 0)	0 (0 to 0)	0 (0 to 0)	0.1 (0 to 0.1)	96.5 (33.9 to 182.3)	5.4 (-28.9 to 58.4)	118.2 (46.7 to 218.5)
Latin America and Caribbean	Central Latin America	Costa Rica	DALYs	All Ages	16 (13 to 21)	4 (3 to 5)	13 (10 to 17)	76 (59 to 98)	11 (8 to 15)	65 (50 to 84)	371.7 (279.6 to 502.3)	201.8 (141.8 to 275.1)	420.7 (304.7 to 585)
Latin America and Caribbean	Central Latin America	Costa Rica	DALYs	Age-standardized	0.8 (0.7 to 1.1)	0.3 (0.2 to 0.5)	1.4 (1.1 to 1.8)	1.5 (1.1 to 1.9)	0.4 (0.3 to 0.5)	2.7 (2.1 to 3.5)	76.5 (41.5 to 123.9)	18.1 (-3.3 to 45.5)	97.6 (55.4 to 156.1)
Latin America and Caribbean	Central Latin America	Costa Rica	YLLs	All Ages	6 (5 to 8)	1 (1 to 2)	5 (4 to 6)	42 (30 to 58)	3 (2 to 4)	39 (27 to 54)	552.6 (338.6 to 851.9)	100.1 (29.8 to 201.1)	688 (416.3 to 1084)
Latin America and Caribbean	Central Latin America	Costa Rica	YLLs	Age-standardized	0.3 (0.3 to 0.4)	0.1 (0.1 to 0.1)	0.6 (0.5 to 0.7)	0.8 (0.6 to 1.1)	0.1 (0.1 to 0.2)	1.6 (1.1 to 2.2)	147.7 (64.9 to 265)	-1 (-34.5 to 47.3)	187 (86.7 to 334.3)
Latin America and Caribbean	Central Latin America	Costa Rica	YLDs	All Ages	10 (6 to 14)	2 (1 to 3)	8 (5 to 11)	34 (22 to 50)	8 (5 to 12)	26 (17 to 38)	251.7 (203.1 to 318.6)	272.7 (198.2 to 368.2)	245.9 (185.8 to 328.5)
Latin America and Caribbean	Central Latin America	Costa Rica	YLDs	Age-standardized	0.5 (0.3 to 0.7)	0.2 (0.1 to 0.3)	0.8 (0.5 to 1.2)	0.7 (0.4 to 1)	0.3 (0.2 to 0.4)	1.1 (0.7 to 1.6)	30.4 (12.3 to 54.4)	27.8 (1.7 to 61.7)	36 (12.9 to 67)
Latin America and Caribbean	Central Latin America	El Salvador	Incidence	All Ages	9 (7 to 11)	2 (2 to 3)	6 (5 to 8)	17 (13 to 21)	5 (4 to 7)	12 (9 to 15)	95.1 (71.7 to 122.1)	122.3 (78.4 to 177)	85.1 (60.4 to 114.9)
Latin America and Caribbean	Central Latin America	El Salvador	Incidence	Age-standardized	0.3 (0.2 to 0.3)	0.1 (0.1 to 0.2)	0.4 (0.3 to 0.5)	0.3 (0.2 to 0.4)	0.2 (0.1 to 0.2)	0.5 (0.4 to 0.6)	10.8 (-1.5 to 24)	18.4 (-4.5 to 45.2)	13.9 (-0.5 to 31.7)
Latin America and Caribbean	Central Latin America	El Salvador	Prevalence	All Ages	100 (80 to 125)	23 (18 to 30)	77 (60 to 97)	236 (190 to 298)	62 (46 to 82)	174 (139 to 223)	135 (104.6 to 176.9)	168.3 (108.1 to 237.5)	124.9 (94.5 to 170.7)
Latin America and Caribbean	Central Latin America	El Salvador	Prevalence	Age-standardized	3.2 (2.5 to 4)	1.4 (1.1 to 1.9)	5.3 (4.1 to 6.6)	3.9 (3.2 to 5)	1.8 (1.3 to 2.4)	6.8 (5.5 to 8.8)	23.1 (7.2 to 42.8)	27.6 (0.6 to 62.6)	29.7 (11.9 to 54.6)
Latin America and Caribbean	Central Latin America	El Salvador	Deaths	All Ages	1 (0 to 1)	0 (0 to 0)	0 (0 to 1)	1 (0 to 1)	0 (0 to 0)	0 (0 to 1)	-4.7 (-35.2 to 44.9)	163.4 (53.7 to 349.8)	-19.9 (-46.7 to 32.2)
Latin America and Caribbean	Central Latin America	El Salvador	Deaths	Age-standardized	0 (0 to 0)	0 (0 to 0)	0 (0 to 0)	0 (0 to 0)	0 (0 to 0)	0 (0 to 0)	-55.3 (-69.7 to -31.9)	13 (-34.5 to 96.2)	-58.9 (-73 to -32.3)
Latin America and Caribbean	Central Latin America	El Salvador	DALYs	All Ages	28 (22 to 36)	5 (3 to 7)	23 (18 to 30)	48 (34 to 65)	12 (8 to 17)	36 (26 to 49)	71.1 (41.9 to 107.3)	156.3 (107.8 to 215.3)	54.1 (23.6 to 93.3)
Latin America and Caribbean	Central Latin America	El Salvador	DALYs	Age-standardized	0.9 (0.7 to 1.2)	0.3 (0.2 to 0.4)	1.6 (1.3 to 2.1)	0.8 (0.6 to 1.1)	0.3 (0.2 to 0.5)	1.4 (1 to 1.9)	-11 (-26.6 to 6.7)	23.1 (0.4 to 53.1)	-12.3 (-29.9 to 9.7)
Latin America and Caribbean	Central Latin America	El Salvador	YLLs	All Ages	12 (9 to 15)	1 (1 to 1)	11 (8 to 14)	11 (8 to 16)	2 (1 to 3)	9 (6 to 13)	-7.4 (-37 to 44.8)	111.9 (27.9 to 252.6)	-18 (-46.6 to 36.4)
Latin America and Caribbean	Central Latin America	El Salvador	YLLs	Age-standardized	0.4 (0.3 to 0.5)	0.1 (0 to 0.1)	0.8 (0.6 to 1)	0.2 (0.1 to 0.3)	0.1 (0 to 0.1)	0.4 (0.3 to 0.5)	-52.1 (-67.6 to -24.2)	5.5 (-36.9 to 78.4)	-53.7 (-69.7 to -22.4)
Latin America and Caribbean	Central Latin America	El Salvador	YLDs	All Ages	16 (10 to 23)	4 (2 to 6)	12 (8 to 18)	37 (23 to 54)	10 (6 to 15)	27 (17 to 40)	131.9 (100.5 to 171.8)	168.4 (108.1 to 237.5)	120.9 (88.6 to 166.3)
Latin America and Caribbean	Central Latin America	El Salvador	YLDs	Age-standardized	0.5 (0.3 to 0.7)	0.2 (0.1 to 0.3)	0.8 (0.5 to 1.2)	0.6 (0.4 to 0.9)	0.3 (0.2 to 0.4)	1.1 (0.7 to 1.6)	21.6 (5.4 to 42)	27.7 (0.6 to 62.5)	27.5 (9.1 to 53.6)
Latin America and Caribbean	Central Latin America	Guatemala	Incidence	All Ages	15 (13 to 19)	4 (3 to 5)	12 (10 to 14)	39 (31 to 49)	10 (8 to 14)	29 (23 to 36)	153.6 (123.7 to 182.9)	171 (126.7 to 225.5)	147.8 (114.2 to 180.9)
Latin America and Caribbean	Central Latin America	Guatemala	Incidence	Age-standardized	0.4 (0.3 to 0.4)	0.2 (0.1 to 0.2)	0.5 (0.5 to 0.7)	0.3 (0.2 to 0.4)	0.1 (0.1 to 0.2)	0.5 (0.4 to 0.6)	-15.7 (-26.1 to -6.5)	-17.2 (-33.1 to -0.5)	-10 (-22.2 to 2.2)
Latin America and Caribbean	Central Latin America	Guatemala	Prevalence	All Ages	153 (122 to 188)	35 (27 to 46)	118 (92 to 146)	478 (384 to 590)	115 (88 to 151)	363 (285 to 456)	212.8 (169.8 to 254.7)	228.3 (171 to 301.4)	208.2 (159.1 to 254.8)
Latin America and Caribbean	Central Latin America	Guatemala	Prevalence	Age-standardized	3.8 (3.1 to 4.6)	1.8 (1.4 to 2.4)	5.7 (4.6 to 7.1)	4 (3.2 to 5)	1.8 (1.4 to 2.4)	6.7 (5.3 to 8.4)	7.3 (-5.9 to 20.5)	-0.6 (-17.7 to 22.7)	17.3 (-0.2 to 35)
Latin America and Caribbean	Central Latin America	Guatemala	Deaths	All Ages	9 (6 to 11)	2 (1 to 3)	6 (5 to 8)	1 (1 to 2)	0 (0 to 0)	1 (1 to 1)	-85.8 (-89.9 to -79.5)	-92.4 (-95.3 to -86.6)	-83.3 (-88.4 to -76)
Latin America and Caribbean	Central Latin America	Guatemala	Deaths	Age-standardized	0.2 (0.2 to 0.3)	0.1 (0.1 to 0.2)	0.3 (0.2 to 0.4)	0 (0 to 0)	0 (0 to 0)	0 (0 to 0)	-94.9 (-96.3 to -92.7)	-97.6 (-98.5 to -95.9)	-92.5 (-94.6 to -89.5)
Latin America and Caribbean	Central Latin America	Guatemala	DALYs	All Ages	348 (262 to 431)	83 (51 to 114)	265 (202 to 342)	107 (79 to 142)	22 (15 to 31)	86 (62 to 113)	-69.1 (-78.4 to -54.1)	-73.8 (-84.1 to -53.1)	-67.7 (-77.8 to -53.3)
Latin America and Caribbean	Central Latin America	Guatemala	DALYs	Age-standardized	6.9 (5.3 to 8.4)	3.6 (2.3 to 4.9)	10.4 (8 to 13)	0.9 (0.6 to 1.2)	0.3 (0.2 to 0.5)	1.5 (1.1 to 2.1)	-87.3 (-91 to -81.5)	-90.5 (-94.2 to -83.3)	-85.1 (-89.6 to -78.7)
Latin America and Caribbean	Central Latin America	Guatemala	YLLs	All Ages	324 (238 to 408)	78 (45 to 107)	246 (185 to 321)	32 (24 to 43)	4 (3 to 6)	29 (21 to 38)	-90 (-93 to -84.7)	-95.2 (-97.2 to -91.3)	-88.4 (-92.2 to -82.5)
Latin America and Caribbean	Central Latin America	Guatemala	YLLs	Age-standardized	6.3 (4.7 to 7.8)	3.4 (2 to 4.6)	9.5 (7.3 to 12.2)	0.3 (0.2 to 0.3)	0.1 (0 to 0.1)	0.5 (0.4 to 0.7)	-96.1 (-97.2 to -94.1)	-98.2 (-98.9 to -97)	-94.9 (-96.5 to -92.4)
Latin America and Caribbean	Central Latin America	Guatemala	YLDs	All Ages	24 (16 to 34)	6 (3 to 8)	19 (12 to 27)	75 (48 to 108)	18 (11 to 27)	57 (37 to 82)	212.7 (169.7 to 253.7)	228.6 (171.3 to 301.5)	208 (159.4 to 255)
Latin America and Caribbean	Central Latin America	Guatemala	YLDs	Age-standardized	0.6 (0.4 to 0.8)	0.3 (0.2 to 0.4)	0.9 (0.6 to 1.3)	0.6 (0.4 to 0.9)	0.3 (0.2 to 0.4)	1.1 (0.7 to 1.5)	7.2 (-5.8 to 20.3)	-0.5 (-17.6 to 22.7)	17.2 (-0.1 to 35)
Latin America and Caribbean	Central Latin America	Honduras	Incidence	All Ages	8 (7 to 10)	2 (2 to 3)	6 (5 to 7)	26 (21 to 32)	8 (6 to 10)	18 (15 to 22)	221.4 (187.9 to 259.8)	267.2 (199.8 to 358.3)	205.8 (167.1 to 248.4)
Latin America and Caribbean	Central Latin America	Honduras	Incidence	Age-standardized	0.3 (0.3 to 0.4)	0.2 (0.1 to 0.2)	0.5 (0.4 to 0.6)	0.4 (0.3 to 0.4)	0.2 (0.2 to 0.3)	0.6 (0.5 to 0.7)	13.7 (1.9 to 26.3)	27.6 (4.3 to 54.4)	11.9 (-1.7 to 27.5)
Latin America and Caribbean	Central Latin America	Honduras	Prevalence	All Ages	83 (68 to 102)	17 (13 to 23)	65 (52 to 82)	284 (228 to 349)	66 (50 to 87)	218 (172 to 272)	242.8 (201.2 to 286.3)	282.5 (213.8 to 364.4)	232.3 (184.8 to 286.8)
Latin America and Caribbean	Central Latin America	Honduras	Prevalence	Age-standardized	3.7 (3 to 4.5)	1.5 (1.2 to 2)	5.9 (4.8 to 7.4)	4.4 (3.6 to 5.5)	2 (1.5 to 2.6)	7.2 (5.7 to 8.9)	20.8 (7 to 36.4)	29.8 (3.5 to 60.9)	22 (4.4 to 40.6)
Latin America and Caribbean	Central Latin America	Honduras	Deaths	All Ages	6 (3 to 8)	1 (0 to 1)	5 (2 to 7)	14 (7 to 21)	3 (1 to 5)	11 (5 to 18)	142.9 (63.9 to 321.3)	241.8 (70 to 508)	127.3 (42.8 to 377.5)
Latin America and Caribbean	Central Latin America	Honduras	Deaths	Age-standardized	0.3 (0.1 to 0.4)	0.1 (0 to 0.1)	0.5 (0.2 to 0.7)	0.2 (0.1 to 0.4)	0.1 (0 to 0.2)	0.4 (0.2 to 0.7)	-10.9 (-40.8 to 63.9)	31.8 (-35.2 to 139.7)	-14.6 (-47.7 to 90.1)
Latin America and Caribbean	Central Latin America	Honduras	DALYs	All Ages	178 (99 to 250)	29 (19 to 43)	149 (72 to 217)	365 (220 to 534)	77 (42 to 131)	287 (155 to 448)	104.5 (43.7 to 220.2)	165 (45.5 to 339.1)	92.6 (28.7 to 242.2)
Latin America and Caribbean	Central Latin America	Honduras	DALYs	Age-standardized	7.2 (3.8 to 10.2)	2 (1.3 to 3)	12.7 (5.8 to 18.6)	5.8 (3.4 to 8.5)	2.2 (1.2 to 3.7)	9.8 (5.3 to 15.4)	-20.5 (-44.2 to 28.3)	8.8 (-38.3 to 78.7)	-22.9 (-48.9 to 43.1)
Latin America and Caribbean	Central Latin America	Honduras	YLLs	All Ages	165 (87 to 238)	27 (16 to 40)	139 (62 to 207)	320 (172 to 495)	67 (32 to 119)	253 (122 to 417)	93.6 (31.2 to 217.8)	153 (23.7 to 348.3)	82.2 (17.4 to 242.4)
Latin America and Caribbean	Central Latin America	Honduras	YLLs	Age-standardized	6.7 (3.2 to 9.6)	1.8 (1.1 to 2.8)	11.8 (4.8 to 17.6)	5.1 (2.7 to 7.8)	1.9 (0.9 to 3.3)	8.6 (4.2 to 14.3)	-24.1 (-48.8 to 28.3)	6 (-48 to 87.6)	-26.5 (-53.4 to 45.7)
Latin America and Caribbean	Central Latin America	Honduras	YLDs	All Ages	13 (8 to 19)	3 (2 to 4)	10 (7 to 15)	45 (29 to 65)	10 (6 to 16)	34 (22 to 50)	242.6 (201.1 to 285.8)	282.2 (213.8 to 363.6)	232.2 (185 to 286.3)
Latin America and Caribbean	Central Latin America	Honduras	YLDs	Age-standardized	0.6 (0.4 to 0.8)	0.2 (0.2 to 0.4)	0.9 (0.6 to 1.4)	0.7 (0.5 to 1)	0.3 (0.2 to 0.5)	1.1 (0.7 to 1.7)	20.7 (7.1 to 36.2)	29.6 (3.5 to 60.6)	21.9 (4.7 to 40.5)
Latin America and Caribbean	Central Latin America	Mexico	Incidence	All Ages	1,087 (920 to 1,283)	148 (123 to 179)	940 (784 to 1,127)	1,762 (1,520 to 2,094)	311 (256 to 378)	1,451 (1,234 to 1,741)	62.1 (48.4 to 74.6)	110.6 (92 to 129.5)	54.4 (40.1 to 66.8)
Latin America and Caribbean	Central Latin America	Mexico	Incidence	Age-standardized	2.2 (1.8 to 2.6)	0.5 (0.4 to 0.6)	3.9 (3.2 to 4.7)	1.4 (1.2 to 1.7)	0.5 (0.4 to 0.6)	2.4 (2.1 to 2.9)	-34.6 (-40 to -29)	-10.6 (-16.2 to -4.6)	-37 (-43 to -31.3)
Latin America and Caribbean	Central Latin America	Mexico	Prevalence	All Ages	13,241 (11,018 to 15,758)	1,511 (1,285 to 1,773)	11,731 (9,577 to 14,124)	24,062 (20,355 to 28,617)	3,558 (3,002 to 4,246)	20,504 (17,083 to 24,713)	81.7 (64.6 to 97.5)	135.5 (122.7 to 148.9)	74.8 (56.1 to 91.9)
Latin America and Caribbean	Central Latin America	Mexico	Prevalence	Age-standardized	29.7 (24.6 to 36)	6.3 (5.3 to 7.4)	55.1 (44.7 to 67.9)	20 (16.9 to 23.9)	5.6 (4.7 to 6.7)	36.4 (30.4 to 44.2)	-32.8 (-39.3 to -26.3)	-11.3 (-16.1 to -6.5)	-33.9 (-41 to -27)
Latin America and Caribbean	Central Latin America	Mexico	Deaths	All Ages	162 (153 to 170)	10 (8 to 11)	152 (144 to 160)	161 (132 to 194)	19 (15 to 25)	141 (114 to 174)	-0.8 (-17.7 to 19.1)	101.1 (60.4 to 152.1)	-7.2 (-24.2 to 13.9)
Latin America and Caribbean	Central Latin America	Mexico	Deaths	Age-standardized	0.4 (0.4 to 0.4)	0 (0 to 0.1)	0.8 (0.8 to 0.9)	0.1 (0.1 to 0.2)	0 (0 to 0)	0.3 (0.2 to 0.3)	-65.1 (-71 to -58.2)	-31.9 (-45.5 to -14.7)	-65.7 (-71.8 to -58.2)
Latin America and Caribbean	Central Latin America	Mexico	DALYs	All Ages	5,675 (5,008 to 6,421)	476 (390 to 581)	5,199 (4,612 to 5,885)	6,918 (5,577 to 8,527)	952 (734 to 1,203)	5,966 (4,773 to 7,423)	21.9 (7 to 37.3)	100 (78.1 to 124.8)	14.8 (-0.7 to 30.7)
Latin America and Caribbean	Central Latin America	Mexico	DALYs	Age-standardized	12.6 (11.2 to 14.3)	1.9 (1.5 to 2.3)	24.2 (21.5 to 27.4)	5.8 (4.7 to 7.1)	1.5 (1.2 to 1.9)	10.7 (8.6 to 13.3)	-54 (-59.7 to -48.2)	-21 (-29.5 to -11.4)	-55.7 (-61.6 to -49.7)

Pneumoconiosis

7 GBD super region	21 GBD region	Location	Measure	Age	1990			2019			Percent change (1990 to 2019)		
					Both	Female	Male	Both	Female	Male	Both	Female	Male
Latin America and Caribbean	Central Latin America	Mexico	YLLs	All Ages	3,790 (3,602 to 3,966)	241 (201 to 265)	3,549 (3,366 to 3,724)	3,423 (2,793 to 4,160)	399 (313 to 518)	3,024 (2,409 to 3,745)	-9.7 (-26.3 to 9.3)	65.6 (30.7 to 110.2)	-14.8 (-31.6 to 5.4)
Latin America and Caribbean	Central Latin America	Mexico	YLLs	Age-standardized	8.4 (8 to 8.8)	0.9 (0.8 to 1)	16.5 (15.7 to 17.4)	2.9 (2.4 to 3.5)	0.6 (0.5 to 0.8)	5.5 (4.4 to 6.8)	-65.5 (-71.8 to -58.4)	-31.4 (-45.3 to -12.7)	-66.6 (-73.1 to -58.8)
Latin America and Caribbean	Central Latin America	Mexico	YLDs	All Ages	1,884 (1,260 to 2,632)	235 (156 to 338)	1,649 (1,107 to 2,308)	3,495 (2,344 to 4,903)	553 (366 to 798)	2,942 (1,952 to 4,155)	85.5 (65.8 to 104.8)	135.3 (121.4 to 149.6)	78.4 (57.2 to 99.9)
Latin America and Caribbean	Central Latin America	Mexico	YLDs	Age-standardized	4.2 (2.8 to 5.8)	1 (0.7 to 1.4)	7.7 (5.1 to 10.7)	2.9 (1.9 to 4.1)	0.9 (0.6 to 1.2)	5.2 (3.5 to 7.3)	-30.9 (-38.4 to -23.1)	-11.1 (-16.2 to -5.7)	-32.1 (-40.3 to -23.7)
Latin America and Caribbean	Central Latin America	Nicaragua	Incidence	All Ages	5 (4 to 7)	1 (1 to 2)	4 (3 to 5)	15 (12 to 18)	4 (3 to 5)	11 (8 to 13)	174.8 (137.3 to 216.6)	207.8 (141.9 to 287.6)	164.6 (119.9 to 215)
Latin America and Caribbean	Central Latin America	Nicaragua	Incidence	Age-standardized	0.3 (0.2 to 0.3)	0.1 (0.1 to 0.2)	0.4 (0.4 to 0.5)	0.3 (0.2 to 0.4)	0.1 (0.1 to 0.2)	0.4 (0.3 to 0.6)	3.6 (-10.5 to 19.3)	21.4 (-5 to 51.6)	0.8 (-16 to 19.9)
Latin America and Caribbean	Central Latin America	Nicaragua	Prevalence	All Ages	59 (48 to 73)	12 (9 to 16)	47 (37 to 59)	192 (152 to 242)	45 (34 to 61)	147 (113 to 187)	222.6 (176.8 to 278.8)	264.1 (193.7 to 354.5)	211.7 (155.6 to 280.9)
Latin America and Caribbean	Central Latin America	Nicaragua	Prevalence	Age-standardized	3.5 (2.8 to 4.3)	1.4 (1 to 1.9)	5.9 (4.7 to 7.3)	4.1 (3.3 to 5.2)	1.8 (1.4 to 2.4)	6.9 (5.4 to 8.8)	17 (-0.2 to 35)	29.9 (2.8 to 64.7)	16 (-5.5 to 39.4)
Latin America and Caribbean	Central Latin America	Nicaragua	Deaths	All Ages	2 (1 to 2)	0 (0 to 0)	2 (1 to 2)	1 (1 to 2)	0 (0 to 0)	1 (1 to 1)	-20.4 (-48.6 to 26.4)	874 (476 to 1530.4)	-35.1 (-59.5 to 6.2)
Latin America and Caribbean	Central Latin America	Nicaragua	Deaths	Age-standardized	0.1 (0.1 to 0.2)	0 (0 to 0)	0.3 (0.2 to 0.4)	0 (0 to 0)	0 (0 to 0)	0.1 (0 to 0.1)	-71.1 (-81.4 to -53.6)	276.1 (119.4 to 540.7)	-77 (-85.9 to -62.3)
Latin America and Caribbean	Central Latin America	Nicaragua	DALYs	All Ages	42 (33 to 55)	2 (2 to 4)	40 (31 to 52)	54 (40 to 69)	11 (8 to 15)	42 (32 to 55)	26.4 (-11.2 to 71.6)	351 (261.5 to 476.7)	6.3 (-26.4 to 48.8)
Latin America and Caribbean	Central Latin America	Nicaragua	DALYs	Age-standardized	2.7 (2.1 to 3.5)	0.3 (0.2 to 0.4)	5.6 (4.3 to 7.3)	1.2 (0.9 to 1.5)	0.5 (0.3 to 0.6)	2.1 (1.6 to 2.7)	-55.7 (-68.7 to -39.2)	68.7 (34.3 to 119.3)	-62.5 (-74.1 to -46.6)
Latin America and Caribbean	Central Latin America	Nicaragua	YLLs	All Ages	33 (25 to 45)	1 (0 to 1)	33 (24 to 44)	24 (18 to 31)	4 (3 to 6)	20 (14 to 26)	-28.3 (-53.2 to 11.9)	672.3 (374.5 to 1165.9)	-39.7 (-62.2 to -3)
Latin America and Caribbean	Central Latin America	Nicaragua	YLLs	Age-standardized	2.2 (1.6 to 2.9)	0.1 (0 to 0.1)	4.7 (3.4 to 6.3)	0.6 (0.4 to 0.7)	0.2 (0.1 to 0.3)	1.1 (0.8 to 1.4)	-73.8 (-83 to -58.9)	216.2 (90.1 to 428.3)	-77.6 (-86 to -63.7)
Latin America and Caribbean	Central Latin America	Nicaragua	YLDs	All Ages	9 (6 to 14)	2 (1 to 3)	7 (5 to 11)	30 (19 to 44)	7 (4 to 11)	23 (14 to 35)	220.1 (173.2 to 275.5)	263.7 (193.7 to 354.3)	208.7 (151.9 to 279.4)
Latin America and Caribbean	Central Latin America	Nicaragua	YLDs	Age-standardized	0.6 (0.4 to 0.8)	0.2 (0.1 to 0.3)	0.9 (0.6 to 1.4)	0.6 (0.4 to 0.9)	0.3 (0.2 to 0.4)	1.1 (0.7 to 1.6)	15.6 (-1.5 to 34.4)	29.8 (2.8 to 64.6)	14 (-7.5 to 38.7)
Latin America and Caribbean	Central Latin America	Panama	Incidence	All Ages	4 (3 to 5)	1 (1 to 2)	3 (3 to 4)	12 (10 to 15)	3 (2 to 4)	9 (7 to 11)	182.7 (142.9 to 230)	178.8 (123.6 to 254.5)	184.1 (136.6 to 238.1)
Latin America and Caribbean	Central Latin America	Panama	Incidence	Age-standardized	0.2 (0.2 to 0.3)	0.1 (0.1 to 0.2)	0.4 (0.3 to 0.5)	0.3 (0.2 to 0.4)	0.1 (0.1 to 0.2)	0.4 (0.4 to 0.6)	19.7 (4.6 to 37.2)	15 (-7.4 to 45.1)	23.3 (3.8 to 45.1)
Latin America and Caribbean	Central Latin America	Panama	Prevalence	All Ages	53 (43 to 66)	11 (8 to 15)	42 (33 to 52)	176 (141 to 222)	39 (29 to 52)	137 (108 to 175)	232.1 (185 to 294.3)	255.3 (184 to 349.9)	226 (170.1 to 293.8)
Latin America and Caribbean	Central Latin America	Panama	Prevalence	Age-standardized	3.3 (2.6 to 4.1)	1.4 (1 to 1.9)	5.2 (4.1 to 6.4)	4.2 (3.4 to 5.4)	1.8 (1.4 to 2.4)	6.8 (5.3 to 8.6)	28 (10.1 to 49.3)	28.7 (0.9 to 65)	31 (8.1 to 55.7)
Latin America and Caribbean	Central Latin America	Panama	Deaths	All Ages	0 (0 to 0)	0 (0 to 0)	0 (0 to 0)	1 (0 to 1)	0 (0 to 0)	0 (0 to 1)	121 (52.3 to 200.2)	242.9 (109.4 to 448.8)	101.6 (33.1 to 183.3)
Latin America and Caribbean	Central Latin America	Panama	Deaths	Age-standardized	0 (0 to 0)	0 (0 to 0)	0 (0 to 0)	0 (0 to 0)	0 (0 to 0)	0 (0 to 0)	-24 (-48 to 3.5)	13.3 (-31.5 to 84.3)	-26.8 (-51.3 to 2.5)
Latin America and Caribbean	Central Latin America	Panama	DALYs	All Ages	14 (10 to 18)	3 (2 to 4)	11 (8 to 14)	38 (27 to 51)	8 (6 to 11)	29 (21 to 41)	174.8 (130.8 to 223.8)	219.1 (159 to 293.5)	164.6 (116.4 to 217.8)
Latin America and Caribbean	Central Latin America	Panama	DALYs	Age-standardized	0.9 (0.7 to 1.1)	0.3 (0.2 to 0.4)	1.4 (1.1 to 1.8)	0.9 (0.7 to 1.2)	0.4 (0.3 to 0.5)	1.5 (1.1 to 2)	5 (-12.1 to 23.3)	21.2 (-1 to 50.7)	4.1 (-15.3 to 25.6)
Latin America and Caribbean	Central Latin America	Panama	YLLs	All Ages	5 (4 to 6)	1 (1 to 1)	5 (4 to 6)	10 (8 to 14)	2 (1 to 3)	8 (6 to 11)	91.7 (28.7 to 165.7)	142.2 (54.7 to 276.1)	82.6 (19.4 to 162.8)
Latin America and Caribbean	Central Latin America	Panama	YLLs	Age-standardized	0.3 (0.3 to 0.4)	0.1 (0.1 to 0.1)	0.6 (0.5 to 0.7)	0.2 (0.2 to 0.3)	0.1 (0.1 to 0.1)	0.4 (0.3 to 0.6)	-27.5 (-51.5 to 2)	2.5 (-33.7 to 54.9)	-29.9 (-54.3 to 0.7)
Latin America and Caribbean	Central Latin America	Panama	YLDs	All Ages	8 (5 to 12)	2 (1 to 3)	7 (4 to 10)	27 (18 to 40)	6 (4 to 9)	21 (14 to 32)	228.1 (179.1 to 285.3)	255.2 (184 to 349.7)	221 (165.4 to 287.2)
Latin America and Caribbean	Central Latin America	Panama	YLDs	Age-standardized	0.5 (0.3 to 0.8)	0.2 (0.1 to 0.3)	0.8 (0.5 to 1.2)	0.7 (0.4 to 1)	0.3 (0.2 to 0.4)	1 (0.7 to 1.6)	26.5 (7.8 to 46.6)	28.7 (0.9 to 64.9)	28.7 (6.5 to 53.9)
Latin America and Caribbean	Central Latin America	Venezuela (Bolivarian Republic of)	Incidence	All Ages	34 (28 to 42)	10 (7 to 13)	24 (20 to 30)	91 (73 to 113)	26 (19 to 34)	65 (52 to 81)	165.7 (127.4 to 210.8)	170.9 (116.8 to 244.3)	163.6 (121.7 to 216.8)
Latin America and Caribbean	Central Latin America	Venezuela (Bolivarian Republic of)	Incidence	Age-standardized	0.3 (0.2 to 0.3)	0.2 (0.1 to 0.2)	0.4 (0.3 to 0.5)	0.3 (0.2 to 0.4)	0.2 (0.1 to 0.2)	0.5 (0.4 to 0.6)	9.3 (-4.5 to 26.3)	8.9 (-10.8 to 35.7)	10.2 (-5.7 to 30.1)
Latin America and Caribbean	Central Latin America	Venezuela (Bolivarian Republic of)	Prevalence	All Ages	370 (300 to 455)	86 (66 to 114)	284 (224 to 349)	1,235 (1,008 to 1,517)	309 (233 to 405)	926 (742 to 1,165)	233.6 (184.1 to 291.9)	257.4 (183.4 to 351)	226.4 (173.8 to 295.9)
Latin America and Caribbean	Central Latin America	Venezuela (Bolivarian Republic of)	Prevalence	Age-standardized	3.4 (2.8 to 4.2)	1.6 (1.2 to 2.1)	5.4 (4.3 to 6.6)	4.2 (3.5 to 5.2)	2 (1.5 to 2.6)	6.8 (5.4 to 8.5)	24.2 (7.5 to 45.1)	25.3 (0 to 58.4)	25.1 (5.6 to 50.6)
Latin America and Caribbean	Central Latin America	Venezuela (Bolivarian Republic of)	Deaths	All Ages	5 (4 to 6)	2 (1 to 2)	4 (3 to 4)	9 (6 to 12)	3 (2 to 5)	6 (4 to 8)	70.4 (17.8 to 147.2)	122.5 (33.8 to 268.3)	48.3 (-1.9 to 123.7)
Latin America and Caribbean	Central Latin America	Venezuela (Bolivarian Republic of)	Deaths	Age-standardized	0.1 (0 to 0.1)	0 (0 to 0.1)	0.1 (0.1 to 0.1)	0 (0 to 0)	0 (0 to 0)	0 (0 to 0.1)	-45.3 (-62.6 to -20.3)	-35.6 (-62.3 to 5.9)	-50.2 (-66.5 to -25.7)
Latin America and Caribbean	Central Latin America	Venezuela (Bolivarian Republic of)	DALYs	All Ages	183 (148 to 214)	41 (31 to 53)	142 (115 to 167)	376 (286 to 485)	101 (75 to 135)	275 (204 to 359)	105.8 (58.5 to 160.7)	147.7 (79.7 to 234.6)	93.8 (46.5 to 153.4)
Latin America and Caribbean	Central Latin America	Venezuela (Bolivarian Republic of)	DALYs	Age-standardized	1.7 (1.4 to 2)	0.8 (0.6 to 1)	2.8 (2.3 to 3.3)	1.3 (1 to 1.7)	0.7 (0.5 to 0.9)	2 (1.5 to 2.6)	-25.9 (-42.5 to -6.3)	-15 (-39.6 to 17)	-28.7 (-45.8 to -7.5)
Latin America and Caribbean	Central Latin America	Venezuela (Bolivarian Republic of)	YLLs	All Ages	125 (98 to 149)	27 (19 to 38)	97 (76 to 117)	183 (130 to 254)	52 (35 to 77)	130 (90 to 187)	46.7 (-2.5 to 115.8)	92.9 (20.2 to 204.2)	33.8 (-12.6 to 104.1)
Latin America and Caribbean	Central Latin America	Venezuela (Bolivarian Republic of)	YLLs	Age-standardized	1.2 (1 to 1.5)	0.5 (0.4 to 0.8)	1.9 (1.5 to 2.3)	0.6 (0.4 to 0.9)	0.4 (0.2 to 0.5)	0.9 (0.6 to 1.3)	-47.7 (-64.9 to -23.8)	-33.8 (-59.6 to 8.3)	-51.9 (-68.4 to -28.1)
Latin America and Caribbean	Central Latin America	Venezuela (Bolivarian Republic of)	YLDs	All Ages	58 (38 to 84)	14 (8 to 20)	45 (29 to 65)	193 (124 to 286)	49 (30 to 74)	145 (92 to 217)	232.5 (183.6 to 289.3)	257 (183.4 to 350.6)	225 (171.7 to 291.6)
Latin America and Caribbean	Central Latin America	Venezuela (Bolivarian Republic of)	YLDs	Age-standardized	0.5 (0.4 to 0.8)	0.3 (0.2 to 0.4)	0.9 (0.6 to 1.2)	0.7 (0.4 to 1)	0.3 (0.2 to 0.5)	1.1 (0.7 to 1.6)	23.6 (6.7 to 43.8)	25.1 (-0.1 to 58.1)	24.3 (4.7 to 48.3)
		Tropical Latin America	Incidence	All Ages	1,529 (1,320 to 1,825)	315 (269 to 374)	1,215 (1,035 to 1,486)	3,190 (2,757 to 3,755)	820 (695 to 965)	2,370 (1,990 to 2,861)	108.6 (93.6 to 123.2)	160.8 (140.3 to 182.9)	95.1 (80.1 to 111.5)
		Tropical Latin America	Incidence	Age-standardized	1.2 (1.1 to 1.5)	0.6 (0.5 to 0.7)	2 (1.7 to 2.4)	1.3 (1.1 to 1.5)	0.6 (0.5 to 0.7)	2 (1.7 to 2.4)	3.1 (-1.7 to 8.1)	10.8 (4.6 to 17.3)	1.9 (-3.9 to 7.6)
		Tropical Latin America	Prevalence	All Ages	22,563 (19,098 to 28,051)	3,696 (3,111 to 4,385)	18,867 (15,498 to 24,027)	50,628 (42,560 to 61,458)	10,131 (8,559 to 12,072)	40,498 (33,114 to 50,833)	124.4 (106.5 to 142.3)	174.1 (157.4 to 191.6)	114.7 (95.2 to 134.6)
		Tropical Latin America	Prevalence	Age-standardized	21.2 (17.9 to 26.3)	7.2 (6.1 to 8.5)	36.5 (30.2 to 46.5)	20.5 (17.2 to 24.8)	7.7 (6.5 to 9.1)	35.1 (28.9 to 44)	-3.6 (-10.1 to 2.5)	6.3 (1.1 to 11.8)	-3.8 (-11.4 to 3.4)
		Tropical Latin America	Deaths	All Ages	205 (191 to 219)	20 (16 to 26)	185 (172 to 198)	502 (460 to 546)	110 (87 to 138)	392 (360 to 425)	145.4 (124.6 to 168.8)	455.3 (349.6 to 615.4)	112.2 (91.6 to 135.3)
		Tropical Latin America	Deaths	Age-standardized	0.2 (0.2 to 0.2)	0 (0 to 0.1)	0.4 (0.4 to 0.4)	0.2 (0.2 to 0.2)	0.1 (0.1 to 0.1)	0.4 (0.3 to 0.4)	-4.2 (-12 to 5.4)	66.8 (33.2 to 115.7)	-11.6 (-19.7 to -2.5)
		Tropical Latin America	DALYs	All Ages	9,832 (8,605 to 11,411)	1,018 (797 to 1,296)	8,814 (7,749 to 10,190)	19,994 (17,322 to 23,421)	3,574 (2,910 to 4,349)	16,419 (14,202 to 19,310)	103.4 (89 to 118.5)	251.1 (214.1 to 306.4)	86.3 (71.5 to 102.3)
		Tropical Latin America	DALYs	Age-standardized	9.1 (8 to 10.6)	2 (1.6 to 2.5)	16.8 (14.9 to 19.5)	8.1 (7 to 9.4)	2.7 (2.2 to 3.3)	14.2 (12.3 to 16.7)	-11.1 (-17 to -4.6)	35.2 (21.2 to 56.3)	-15.5 (-21.8 to -8.7)
		Tropical Latin America	YLLs	All Ages	6,478 (6,025 to 6,958)	446 (359 to 572)	6,032 (5,581 to 6,473)	12,558 (11,627 to 13,550)	2,032 (1,708 to 2,524)	10,527 (9,657 to 11,446)	93.9 (75.7 to 114.7)	355.8 (276.1 to 481.1)	74.5 (56.1 to 95.3)
		Tropical Latin America	YLLs	Age-standardized	6 (5.6 to 6.4)	0.9 (0.7 to 1.2)	11.5 (10.7 to 12.3)	5.1 (4.7 to 5.5)	1.5 (1.3 to 1.9)	9.1 (8.4 to 9.9)	-14.8 (-22.5 to -6.1)	72.5 (41.9 to 119.9)	-20.6 (-28.6 to -11.5)
		Tropical Latin America	YLDs	All Ages	3,354 (2,161 to 4,956)	572 (376 to 824)	2,782 (1,789 to 4,148)	7,435 (4,912 to 10,748)	1,543 (1,014 to 2,207)	5,892 (3,849 to 8,571)	121.7 (101.2 to 144.9)	169.6 (152.5 to 188.5)	111.8 (88.6 to 138)
		Tropical Latin America	YLDs	Age-standardized	3.1 (2 to 4.6)	1.1 (0.7 to 1.6)	5.3 (3.4 to 7.9)	3 (2 to 4.3)	1.2 (0.8 to 1.7)	5.1 (3.3 to 7.4)	-4.1 (-11.7 to 4.2)	5.1 (-0.9 to 11.3)	-4.4 (-13.7 to 5.8)
Latin America and Caribbean	Tropical Latin America	Brazil	Incidence	All Ages	1,508 (1,302 to 1,801)	311 (266 to 370)	1,197 (1,020 to 1,466)	3,139 (2,709 to 3,695)	811 (687 to 953)	2,328 (1,953 to 2,810)	108.2 (93.2 to 122.9)	160.9 (140.4 to 183.3)	94.5 (79.1 to 110.9)
Latin America and Caribbean	Tropical Latin America	Brazil	Incidence	Age-standardized	1.3 (1.1 to 1.5)	0.6 (0.5 to 0.7)	2 (1.7 to 2.4)	1.3 (1.1 to 1.5)	0.6 (0.5 to 0.7)	2 (1.7 to 2.4)	3.1 (-1.8 to 8.1)	10.8 (4.6 to 17.3)	1.9 (-3.8 to 7.6)
Latin America and Caribbean	Tropical Latin America	Brazil	Prevalence	All Ages	22,221 (18,797 to 27,665)	3,649 (3,070 to 4,325)	18,572 (15,263 to 23,648)	49,744 (41,788 to 60,362)	10,010 (8,456 to 11,920)	39,734 (32,483 to 49,915)	123.9 (105.9 to 141.6)	174.3 (157.5 to 192.1)	113.9 (94.6 to 134)
Latin America and Caribbean	Tropical Latin America	Brazil	Prevalence	Age-standardized	21.4 (18 to 26.6)	7.3 (6.2 to 8.6)	36.8 (30.4 to 47)	20.6 (17.3 to 24.9)	7.7 (6.6 to 9.2)	35.4 (29.1 to 44.3)	-3.8 (-10.3 to 2.2)	6.1 (0.8 to 11.7)	-4 (-11.6 to 3.2)
Latin America and Caribbean	Tropical Latin America	Brazil	Deaths	All Ages	194 (181 to 208)	20 (16 to 25)	174 (162 to 187)	487 (445 to 530)	110 (87 to 138)	377 (345 to 410)	150.9 (128.3 to 176.6)	456.3 (350.2 to 617.8)	116.4 (94.3 to 140.9)
Latin America and Caribbean	Tropical Latin America	Brazil	Deaths	Age-standardized	0.2 (0.2 to 0.2)	0.1 (0 to 0.1)	0.4 (0.4 to 0.4)	0.2 (0.2 to 0.2)	0.1 (0.1 to 0.1)	0.4 (0.3 to 0.4)	-2.8 (-10.8 to 6.7)	65.3 (31.6 to 113.6)	-10.3 (-18.7 to -0.5)
Latin America and Caribbean	Tropical Latin America	Brazil	DALYs	All Ages	9,411 (8,203 to 10,978)	1,009 (791 to 1,282)	8,402 (7,368 to 9,770)	19,338 (16,724 to 22,690)	3,554 (2,898 to 4,320)	15,785 (13,608 to 18,624)	105.5 (91 to 121.6)	252.1 (214.8 to 308.1)	87.9 (72.2 to 105.1)
Latin America and Caribbean	Tropical Latin America	Brazil	DALYs	Age-standardized	8.9 (7.8 to 10.4)	2 (1.6 to 2.6)	16.5 (14.5 to 19.2)	8 (6.9 to 9.4)	2.8 (2.3 to 3.4)	14 (12.1 to 16.5)	-10.3 (-16.3 to -3.7)	35.1 (21.1 to 56.3)	-14.7 (-21.4 to -7.5)
Latin America and Caribbean	Tropical Latin America	Brazil	YLLs	All Ages	6,109 (5,675 to 6,562)	444 (358 to 570)	5,665 (5,229 to 6,104)	12,035 (11,095 to 13,058)	2,029 (1,707 to 2,522)	10,006 (9,159 to 10,886)	97 (77.8 to 118.3)	356.7 (276.8 to 482.5)	76.6 (56.9 to 98.9)
Latin America and Caribbean	Tropical Latin America	Brazil	YLLs	Age-standardized	5.8 (5.4 to 6.2)	0.9 (0.7 to 1.2)	11.1 (10.3 to 11.9)	5 (4.6 to 5.4)	1.6 (1.3 to 2)	8.9 (8.2 to 9.7)	-13.6 (-22.1 to -4.5)	72.1 (41.5 to 119.9)	-19.6 (-28.1 to -10.2)
Latin America and Caribbean	Tropical Latin America	Brazil	YLDs	All Ages	3,303 (2,125 to 4,875)	565 (371 to 813)	2,737 (1,759 to 4,087)	7,303 (4,816 to 10,564)	1,524 (1,002 to 2,180)	5,779 (3,777 to 8,431)	121.1 (100.7 to 144.8)	169.8 (152.1 to 188.8)	111.1 (87.6 to 137.4)
Latin America and Caribbean	Tropical Latin America	Brazil	YLDs	Age-standardized	3.2 (2 to 4.6)	1.1 (0.7 to 1.6)	5.4 (3.4 to 8)	3 (2 to 4.3)	1.2 (0.8 to 1.7)	5.1 (3.4 to 7.5)	-4.3 (-11.8 to 4.2)	4.9 (-1 to 11.2)	-4.6 (-13.7 to 5.6)
Latin America and Caribbean	Tropical Latin America	Paraguay	Incidence	All Ages	22 (18 to 29)	4 (3 to 5)	18 (15 to 25)	51 (44 to 61)	9 (7 to 12)	42 (35 to 50)	137.2 (83.2 to 170.5)	150.4 (112.9 to 197.9)	134.6 (73.5 to 172.9)
Latin America and Caribbean	Tropical Latin America	Paraguay	Incidence	Age-standardized	0.8 (0.6 to 1)	0.3 (0.2 to 0.3)	1.2 (1 to 1.8)	0.8 (0.7 to 0.9)	0.3 (0.2 to 0.4)	1.3 (1.1 to 1.5)	5.3 (-19.3 to 19.5)	8.6 (-7.7 to 28.2)	4.2 (-24 to 21)
Latin America and Caribbean	Tropical Latin America	Paraguay	Prevalence	All Ages	341 (281 to 453)	47 (36 to 61)	294 (237 to 409)	884 (740 to 1,061)	120 (93 to 155)	764 (622 to 934)	158.9 (97.6 to 203.5)	155.8 (115 to 205.6)	159.4 (89.9 to 210.8)
Latin America and Caribbean	Tropical Latin America	Paraguay	Prevalence	Age-standardized	13.6 (11.3 to 18.6)	3.8 (2.9 to 4.8)	24 (19.4 to 34)	14.9 (12.5 to 17.7)	4 (3.1 to 5.2)	26.3 (21.5 to 31.9)	9.1 (-18.3 to 27.2)	6.2 (-11.4 to 27.5)	9.7 (-21.4 to 30.5)
Latin America and Caribbean	Tropical Latin America	Paraguay	Deaths	All Ages	10 (8 to 14)	0 (0 to 0)	10 (8 to 14)	15 (10 to 21)	0 (0 to 0)	15 (10 to 20)	43.4 (-9 to 135.4)	112.5 (20.9 to 252.9)	43 (-9.3 to 136)
Latin America and Caribbean	Tropical Latin America	Paraguay	Deaths	Age-standardized	0.4 (0.3 to 0.6)	0 (0 to 0)	0.9 (0.7 to 1.2)	0.2 (0.2 to 0.3)	0 (0 to 0)	0.5 (0.3 to 0.7)	-41.2 (-62.9 to -3.6)	-14.4 (-51.3 to 43.8)	-41.8 (-63.3 to -4.2)
Latin America and Caribbean	Tropical Latin America	Paraguay	DALYs	All Ages	421 (324 to 531)	9 (6 to 12)	412 (316 to 524)	655 (477 to 860)	21 (14 to 30)	634 (456 to 840)	55.8 (7.1 to 141.6)	139.5 (102.5 to 186.2)	54 (5.3 to 141)
Latin America and Caribbean	Tropical Latin America	Paraguay	DALYs	Age-standardized	15.8 (12.2 to 20.4)	0.7 (0.5 to 1)	31.3 (24.1 to 40.8)	10.5 (7.6 to 13.7)	0.7 (0.5 to 1)	20.4 (14.7 to 26.8)	-33.9 (-54.7 to 1.5)	0.9 (-15.2 to 20.6)	-34.9 (-55.6 to 1.1)
Latin America and Caribbean	Tropical Latin America	Paraguay	YLLs	All Ages	369 (275 to 483)	1 (1 to 2)	367 (273 to 482)	523 (359 to 710)	2 (1 to 3)	521 (357 to 708)	41.9 (-8.5 to 139.1)	72.8 (-3.7 to 187.4)	41.8 (-8.6 to 139.2)
Latin America and Caribbean	Tropical Latin America	Paraguay	YLLs	Age-standardized	13.8 (10.3 to 18.5)	0.1 (0.1 to 0.1)	27.7 (20.7 to 37.5)	8.3 (5.7 to 11.2)	0.1 (0 to 0.1)	16.5 (11.3 to 22.4)	-40.1 (-61.5 to 0.2)	-21.6 (-56.1 to 28.3)	-40.5 (-61.6 to -0.4)
Latin America and Caribbean	Tropical Latin America	Paraguay	YLDs	All Ages	52 (34 to 78)	7 (5 to 11)	45 (29 to 68)	132 (84 to 189)	19 (12 to 28)	113 (72 to 164)	154.2 (89.9 to 220.4)	151.8 (110.1 to 199.8)	154.6 (82.9 to 231.5)
Latin America and Caribbean	Tropical Latin America	Paraguay	YLDs	Age-standardized	2.1 (1.3 to 3.1)	0.6 (0.4 to 0.9)	3.6 (2.3 to 5.5)	2.2 (1.4 to 3.2)	0.6 (0.4 to 0.9)	3.9 (2.5 to 5.6)	7.4 (-20.7 to 36.7)	4.5 (-14.2 to 24.8)	8 (-23.2 to 42.2)
		North Africa and Middle East	Incidence	All Ages	1,506 (1,135 to 1,938)	1,183 (886 to 1,539)	323 (236 to 424)	4,859 (3,756 to 6,075)	4,396 (3,375 to 5,553)	463 (367 to 574)	222.6 (196.3 to 254.3)	271.7 (238.5 to 312.3)	43.2 (19.8 to 73)
		North Africa and Middle East	Incidence	Age-standardized	0.6 (0.5 to 0.8)	1 (0.7 to 1.2)	0.3 (0.2 to 0.4)	0.9 (0.7 to 1.1)	1.7 (1.3 to 2.1)	0.2 (0.2 to 0.2)	43.2 (34.8 to 53.2)	70.3 (60.4 to 81.9)	-32.8 (-42.2 to -21.5)
		North Africa and Middle East	Prevalence	All Ages	3,343 (2,572 to 4,263)	2,502 (1,816 to 3,320)	841 (695 to 1,009)	12,474 (10,005 to 15,275)	10,294 (7,883 to 12,996)	2,180 (1,834 to 2,631)	273.2 (241.8 to 309.2)	311.5 (270.2 to 359.3)	159.3 (124.6 to 197.7)
		North Africa and Middle East	Prevalence	Age-standardized	1.6 (1.3 to 2)	2.2 (1.7 to 2.9)	0.9 (0.8 to 1.1)	2.6 (2.1 to 3.1)	4.1 (3.2 to 5.1)	1.1 (1 to 1.4)	62.8 (52.3 to 75.7)	82.9 (69.7 to 101.1)	22.4 (8.3 to 37.5)
		North Africa and Middle East	Deaths	All Ages	147 (103 to 187)	17 (12 to 23)	130 (85 to 170)	289 (244 to 344)	70 (56 to 88)	219 (180 to 266)	96.2 (43.5 to 215)	309.5 (204.8 to 479.3)	68.2 (17.8 to 198.3)
		North Africa and Middle East	Deaths	Age-standardized	0.1 (0.1 to 0.1)	0 (0 to 0)	0.2 (0.1 to 0.2)	0.1 (0.1 to 0.1)	0 (0 to 0)	0.1 (0.1 to 0.1)	-15.1 (-36.8 to 36.3)	56.9 (14.7 to 119.9)	-27.5 (-49 to 34.2)
		North Africa and Middle East	DALYs	All Ages	5,135 (3,771 to 6,554)	830 (627 to 1,084)	4,305 (2,975 to 5,696)	9,437 (7,982 to 11,095)	3,337 (2,604 to 4,281)	6,100 (4,984 to 7,400)	83.8 (35.3 to 167.5)	302 (242.9 to 389)	41.7 (-4.2 to 132.4)
		North Africa and Middle East	DALYs	Age-standardized	2.4 (1.8 to 3)	0.8 (0.6 to 1.1)	4 (2.7 to 5.2)	2 (1.7 to 2.3)	1.4 (1.1 to 1.8)	2.6 (2.1 to 3.1)	-17.6 (-37.3 to 22.4)	70.3 (43.2 to 108.6)	-35.3 (-54.1 to 9.6)
		North Africa and Middle East	YLLs	All Ages	4,607 (3,292 to 6,002)	436 (298 to 580)	4,171 (2,849 to 5,573)	7,497 (6,187 to 8,968)	1,745 (1,389 to 2,235)	5,753 (4,651 to 7,031)	62.7 (15.3 to 152.3)	299.9 (199.3 to 480.4)	37.9 (-7.7 to 131.3)
		North Africa and Middle East	YLLs	Age-standardized	2.2 (1.5 to 2.8)	0.5 (0.3 to 0.6)	3.8 (2.6 to 5)	1.6 (1.3 to 1.9)	0.8 (0.6 to 1)	2.4 (1.9 to 2.9)	-26.4 (-46.3 to 15.7)	64.5 (23.5 to 132.6)	-37.5 (-56.4 to 8.9)
		North Africa and Middle East	YLDs	All Ages	528 (331 to 804)	394 (237 to 625)	134 (86 to 195)	1,940 (1,249 to 2,858)	1,593 (1,013 to 2,388)	347 (227 to 503)	267.4 (236.3 to 303.3)	304.4 (264 to 352.8)	158.7 (124.5 to 196.3)
		North Africa and Middle East	YLDs	Age-standardized	0.2 (0.2 to 0.4)	0.4 (0.2 to 0.5)	0.1 (0.1 to 0.2)	0.4 (0.3 to 0.6)	0.6 (0.4 to 0.9)	0.2 (0.1 to 0.3)	59 (47.8 to 71.6)	78 (63.2 to 96.3)	21.8 (7.9 to 36.4)
		North Africa and Middle East	Incidence	All Ages	1,506 (1,135 to 1,938)	1,183 (886 to 1,539)	323 (236 to 424)	4,859 (3,756 to 6,075)	4,396 (3,375 to 5,553)	463 (367 to 574)	222.6 (196.3 to 254.3)	271.7 (238.5 to 312.3)	43.2 (19.8 to 73)
		North Africa and Middle East	Incidence	Age-standardized	0.6 (0.5 to 0.8)	1 (0.7 to 1.2)	0.3 (0.2 to 0.4)	0.9 (0.7 to 1.1)	1.7 (1.3 to 2.1)	0.2 (0.2 to 0.2)	43.2 (34.8 to 53.2)	70.3 (60.4 to 81.9)	-32.8 (-42.2 to -21.5)
		North Africa and Middle East	Prevalence	All Ages	3,343 (2,572 to 4,263)	2,502 (1,816 to 3,320)	841 (695 to 1,009)	12,474 (10,005 to 15,275)	10,294 (7,883 to 12,996)	2,180 (1,834 to 2,631)	273.2 (241.8 to 309.2)	311.5 (270.2 to 359.3)	159.3 (124.6 to 197.7)
		North Africa and Middle East	Prevalence	Age-standardized	1.6 (1.3 to 2)	2.2 (1.7 to 2.9)	0.9 (0.8 to 1.1)	2.6 (2.1 to 3.1)	4.1 (3.2 to 5.1)	1.1 (1 to 1.4)	62.8 (52.3 to 75.7)	82.9 (69.7 to 101.1)	22.4 (8.3 to 37.5)
		North Africa and Middle East	Deaths	All Ages	147 (103 to 187)	17 (12 to 23)	130 (85 to 170)	289 (244 to 344)	70 (56 to 88)	219 (180 to 266)	96.2 (43.5 to 215)	309.5 (204.8 to 479.3)	68.2 (17.8 to 198.3)
		North Africa and Middle East	Deaths	Age-standardized	0.1 (0.1 to 0.1)	0 (0 to 0)	0.2 (0.1 to 0.2)	0.1 (0.1 to 0.1)	0 (0 to 0)	0.1 (0.1 to 0.1)	-15.1 (-36.8 to 36.3)	56.9 (14.7 to 119.9)	-27.5 (-49 to 34.2)
		North Africa and Middle East	DALYs	All Ages	5,135 (3,771 to 6,554)	830 (627 to 1,084)	4,305 (2,975 to 5,696)	9,437 (7,982 to 11,095)	3,337 (2,604 to 4,281)	6,100 (4,984 to 7,400)	83.8 (35.3 to 167.5)	302 (242.9 to 389)	41.7 (-4.2 to 132.4)
		North Africa and Middle East	DALYs	Age-standardized	2.4 (1.8 to 3)	0.8 (0.6 to 1.1)	4 (2.7 to 5.2)	2 (1.7 to 2.3)	1.4 (1.1 to 1.8)	2.6 (2.1 to 3.1)	-17.6 (-37.3 to 22.4)	70.3 (43.2 to 108.6)	-35.3 (-54.1 to 9.6)
		North Africa and Middle East	YLLs	All Ages	4,607 (3,292 to 6,002)	436 (298 to 580)	4,171 (2,849 to 5,573)	7,497 (6,187 to 8,968)	1,745 (1,389 to 2,235)	5,753 (4,651 to 7,031)	62.7 (15.3 to 152.3)	299.9 (199.3 to 480.4)	37.9 (-7.7 to 131.3)
		North Africa and Middle East	YLLs	Age-standardized	2.2 (1.5 to 2.8)	0.5 (0.3 to 0.6)	3.8 (2.6 to 5)	1.6 (1.3 to 1.9)	0.8 (0.6 to 1)	2.4 (1.9 to 2.9)	-26.4 (-46.3 to 15.7)	64.5 (23.5 to 132.6)	-37.5 (-56.4 to 8.9)
		North Africa and Middle East	YLDs	All Ages	528 (331 to 804)	394 (237 to 625)	134 (86 to 195)	1,940 (1,249 to 2,858)	1,593 (1,013 to 2,388)	347 (227 to 503)	267.4 (236.3 to 303.3)	304.4 (264 to 352.8)	158.7 (124.5 to 196.3)
		North Africa and Middle East	YLDs	Age-standardized	0.2 (0.2 to 0.4)	0.4 (0.2 to 0.5)	0.1 (0.1 to 0.2)	0.4 (0.3 to 0.6)	0.6 (0.4 to 0.9)	0.2 (0.1 to 0.3)	59 (47.8 to 71.6)	78 (63.2 to 96.3)	21.8 (7.9 to 36.4)
North Africa and Middle East	North Africa and Middle East	Afghanistan	Incidence	All Ages	60 (45 to 77)	48 (35 to 64)	12 (8 to 16)	291 (212 to 382)	273 (197 to 362)	18 (11 to 27)	382 (301.7 to 475.4)	463.9 (369 to 586.8)	51.8 (1.9 to 112.6)
North Africa and Middle East	North Africa and Middle East	Afghanistan	Incidence	Age-standardized	0.8 (0.6 to 1)	1.2 (0.9 to 1.5)	0.3 (0.2 to 0.4)	1.2 (1 to 1.6)	2.3 (1.7 to 2.9)	0.2 (0.2 to 0.3)	62.5 (42 to 87.4)	95.1 (69.2 to 126.1)	-35.9 (-52.2 to -17.6)
North Africa and Middle East	North Africa and Middle East	Afghanistan	Prevalence	All Ages	106 (84 to 132)	79 (59 to 102)	27 (21 to 33)	592 (442 to 778)	546 (401 to 725)	47 (36 to 59)	459.9 (368.8 to 559.1)	590.8 (480.7 to 733.7)	74.3 (38 to 111.5)
North Africa and Middle East	North Africa and Middle East	Afghanistan	Prevalence	Age-standardized	1.4 (1.1 to 1.7)	2 (1.6 to 2.6)	0.8 (0.6 to 0.9)	2.9 (2.3 to 3.6)	5 (3.8 to 6.3)	0.8 (0.6 to 0.9)	104.3 (78.7 to 130.5)	150.6 (118.3 to 186.6)	-2.4 (-20 to 16.6)
North Africa and Middle East	North Africa and Middle East	Afghanistan	Deaths	All Ages	6 (2 to 11)	1 (0 to 1)	6 (1 to 10)	13 (7 to 21)	4 (2 to 6)	10 (3 to 16)	105.9 (24.3 to 256.1)	352.8 (153.2 to 666.8)	71.1 (-1.8 to 219.2)
North Africa and Middle East	North Africa and Middle East	Afghanistan	Deaths	Age-standardized	0.1 (0 to 0.2)	0 (0 to 0)	0.2 (0 to 0.3)	0.1 (0.1 to 0.2)	0.1 (0 to 0.1)	0.2 (0.1 to 0.3)	10.9 (-30.9 to 83.4)	123.7 (24.7 to 282)	1.1 (-41.2 to 80.8)
North Africa and Middle East	North Africa and Middle East	Afghanistan	DALYs	All Ages	190 (87 to 315)	38 (23 to 58)	152 (46 to 276)	546 (328 to 813)	215 (141 to 306)	332 (129 to 573)	187.4 (79.3 to 372.5)	466.2 (277.7 to 710)	117.9 (22.1 to 298.5)
North Africa and Middle East	North Africa and Middle East	Afghanistan	DALYs	Age-standardized	2.5 (1.1 to 4.1)	1 (0.6 to 1.5)	3.9 (1.2 to 7)	3 (1.7 to 4.5)	2.1 (1.4 to 3)	4 (1.5 to 6.7)	21.7 (-22.2 to 90.3)	123.2 (50.9 to 212.9)	3.7 (-39.7 to 78.3)
North Africa and Middle East	North Africa and Middle East	Afghanistan	YLLs	All Ages	173 (68 to 298)	25 (13 to 45)	148 (42 to 272)	453 (239 to 715)	129 (68 to 213)	324 (121 to 563)	161.3 (56.1 to 352.6)	405.6 (180.1 to 776.1)	119.2 (21.4 to 310.1)
North Africa and Middle East	North Africa and Middle East	Afghanistan	YLLs	Age-standardized	2.3 (0.9 to 3.9)	0.6 (0.3 to 1.1)	3.8 (1 to 6.9)	2.6 (1.3 to 4)	1.3 (0.7 to 2.2)	3.9 (1.4 to 6.6)	13.6 (-30.3 to 87)	109.8 (21.8 to 255.1)	3.9 (-40.2 to 83.6)
North Africa and Middle East	North Africa and Middle East	Afghanistan	YLDs	All Ages	17 (11 to 25)	12 (8 to 19)	4 (3 to 6)	93 (57 to 146)	86 (52 to 136)	7 (5 to 11)	458.4 (367.6 to 557.5)	590.8 (480.3 to 733.2)	74.4 (38.5 to 111)
North Africa and Middle East	North Africa and Middle East	Afghanistan	YLDs	Age-standardized	0.2 (0.1 to 0.3)	0.3 (0.2 to 0.5)	0.1 (0.1 to 0.2)	0.5 (0.3 to 0.7)	0.8 (0.5 to 1.2)	0.1 (0.1 to 0.2)	103.8 (78.2 to 129.3)	150.5 (118.1 to 186.4)	-2.3 (-19.7 to 16.4)
North Africa and Middle East	North Africa and Middle East	Algeria	Incidence	All Ages	19 (13 to 27)	13 (8 to 20)	6 (4 to 9)	96 (71 to 125)	79 (56 to 104)	17 (13 to 24)	398.9 (259.2 to 604)	494.8 (298.3 to 827.7)	189.1 (114.4 to 300.9)
North Africa and Middle East	North Africa and Middle East	Algeria	Incidence	Age-standardized	0.1 (0.1 to 0.2)	0.2 (0.1 to 0.2)	0.1 (0.1 to 0.1)	0.3 (0.2 to 0.3)	0.4 (0.3 to 0.6)	0.1 (0.1 to 0.1)	110.7 (63.2 to 176.8)	155.3 (83.7 to 263.9)	24.9 (-0.7 to 61.5)
North Africa and Middle East	North Africa and Middle East	Algeria	Prevalence	All Ages	52 (41 to 66)	22 (16 to 31)	29 (22 to 37)	231 (192 to 280)	121 (91 to 156)	110 (87 to 139)	346.8 (267.4 to 443.6)	437.1 (289.5 to 635.8)	277.3 (210.6 to 369.1)
North Africa and Middle East	North Africa and Middle East	Algeria	Prevalence	Age-standardized	0.4 (0.3 to 0.5)	0.3 (0.2 to 0.4)	0.5 (0.4 to 0.6)	0.7 (0.6 to 0.9)	0.7 (0.5 to 0.9)	0.7 (0.6 to 1)	76.3 (48.7 to 108.7)	118.6 (71.9 to 176)	48.5 (20.8 to 84.5)
North Africa and Middle East	North Africa and Middle East	Algeria	Deaths	All Ages	4 (3 to 6)	1 (0 to 1)	3 (2 to 5)	12 (9 to 16)	4 (3 to 6)	8 (5 to 11)	192.4 (88.5 to 355.1)	397.5 (185 to 839.8)	141.2 (42.6 to 320.1)
North Africa and Middle East	North Africa and Middle East	Algeria	Deaths	Age-standardized	0 (0 to 0.1)	0 (0 to 0)	0.1 (0 to 0.1)	0 (0 to 0.1)	0 (0 to 0.1)	0.1 (0 to 0.1)	-4.2 (-38.1 to 48)	82.9 (-0.3 to 249)	-23.3 (-53.3 to 31.8)
North Africa and Middle East	North Africa and Middle East	Algeria	DALYs	All Ages	117 (85 to 164)	30 (17 to 46)	87 (59 to 128)	323 (243 to 418)	115 (83 to 162)	207 (147 to 287)	175.1 (84.3 to 320.1)	284.4 (126.1 to 589.4)	137.5 (45.5 to 289.2)
North Africa and Middle East	North Africa and Middle East	Algeria	DALYs	Age-standardized	0.9 (0.7 to 1.3)	0.4 (0.2 to 0.6)	1.4 (1 to 2.1)	1 (0.7 to 1.2)	0.7 (0.5 to 1)	1.2 (0.9 to 1.7)	3.7 (-29.2 to 54)	68.3 (4.3 to 185.2)	-14.9 (-47.2 to 37.8)
North Africa and Middle East	North Africa and Middle East	Algeria	YLLs	All Ages	109 (76 to 155)	26 (13 to 43)	83 (54 to 124)	286 (210 to 379)	96 (65 to 143)	190 (130 to 270)	162.4 (68.5 to 310.6)	264.3 (99.3 to 619.1)	129.7 (35.3 to 285.3)
North Africa and Middle East	North Africa and Middle East	Algeria	YLLs	Age-standardized	0.9 (0.6 to 1.2)	0.4 (0.2 to 0.6)	1.3 (0.9 to 2)	0.8 (0.6 to 1.1)	0.6 (0.4 to 0.9)	1.1 (0.7 to 1.5)	-1.7 (-35.7 to 49.9)	61.9 (-5 to 201.7)	-18.6 (-51.2 to 34.4)
North Africa and Middle East	North Africa and Middle East	Algeria	YLDs	All Ages	8 (5 to 12)	4 (2 to 6)	5 (3 to 7)	37 (24 to 53)	19 (12 to 29)	17 (11 to 26)	344.4 (266.6 to 439.6)	434 (288.7 to 628.4)	275.9 (210.5 to 367.1)
North Africa and Middle East	North Africa and Middle East	Algeria	YLDs	Age-standardized	0.1 (0 to 0.1)	0 (0 to 0.1)	0.1 (0.1 to 0.1)	0.1 (0.1 to 0.2)	0.1 (0.1 to 0.2)	0.1 (0.1 to 0.2)	75.2 (48.1 to 107)	117 (71.2 to 173.3)	47.7 (20.5 to 83.2)
North Africa and Middle East	North Africa and Middle East	Bahrain	Incidence	All Ages	2 (1 to 2)	1 (1 to 2)	0 (0 to 1)	7 (5 to 10)	6 (4 to 9)	1 (1 to 2)	321.8 (245.7 to 418.6)	391.6 (291.1 to 523.9)	119.9 (34.8 to 278.5)
North Africa and Middle East	North Africa and Middle East	Bahrain	Incidence	Age-standardized	0.5 (0.4 to 0.6)	0.8 (0.6 to 1.1)	0.3 (0.2 to 0.4)	0.6 (0.4 to 0.7)	1.2 (0.9 to 1.6)	0.2 (0.1 to 0.2)	12.8 (-1.2 to 29.9)	41.4 (23.2 to 64.2)	-34.6 (-50.6 to -15)
North Africa and Middle East	North Africa and Middle East	Bahrain	Prevalence	All Ages	3 (2 to 4)	2 (1 to 3)	1 (1 to 1)	14 (11 to 18)	11 (8 to 14)	3 (3 to 4)	373.9 (308 to 455.5)	428 (336.4 to 543.8)	260.8 (169.8 to 370.9)
North Africa and Middle East	North Africa and Middle East	Bahrain	Prevalence	Age-standardized	1 (0.8 to 1.2)	1.4 (1.1 to 1.8)	0.8 (0.6 to 0.9)	1.3 (1 to 1.5)	2.1 (1.6 to 2.6)	0.8 (0.6 to 1)	24.7 (13.4 to 38)	49.3 (31.7 to 69.1)	0.2 (-16 to 19.7)
North Africa and Middle East	North Africa and Middle East	Bahrain	Deaths	All Ages	0 (0 to 0)	0 (0 to 0)	0 (0 to 0)	0 (0 to 1)	0 (0 to 0)	0 (0 to 1)	245.4 (132.3 to 413.5)	65 (2.7 to 176.8)	280.1 (150.4 to 482.4)
North Africa and Middle East	North Africa and Middle East	Bahrain	Deaths	Age-standardized	0.1 (0.1 to 0.1)	0 (0 to 0)	0.2 (0.1 to 0.2)	0.1 (0.1 to 0.1)	0 (0 to 0)	0.2 (0.1 to 0.3)	2.5 (-30.4 to 45.6)	-55.2 (-71 to -27.8)	-0.3 (-34.3 to 44.2)
North Africa and Middle East	North Africa and Middle East	Bahrain	DALYs	All Ages	4 (3 to 5)	1 (1 to 2)	3 (2 to 4)	14 (10 to 19)	3 (2 to 4)	11 (8 to 15)	219.6 (123.7 to 366.3)	142.5 (69.9 to 242.5)	250.3 (129.2 to 441.6)
North Africa and Middle East	North Africa and Middle East	Bahrain	DALYs	Age-standardized	2 (1.6 to 2.4)	0.8 (0.6 to 1.1)	3.2 (2.5 to 4)	1.6 (1.3 to 2.1)	0.6 (0.4 to 0.8)	2.7 (2 to 3.5)	-15.7 (-39.5 to 17.4)	-26.1 (-46.1 to 0.8)	-16.8 (-42.7 to 19.4)
North Africa and Middle East	North Africa and Middle East	Bahrain	YLLs	All Ages	4 (3 to 5)	1 (1 to 1)	3 (2 to 4)	11 (8 to 16)	1 (1 to 2)	10 (7 to 15)	200.7 (100.5 to 366.2)	42.4 (-14.9 to 145.7)	249.7 (125.3 to 455.7)
North Africa and Middle East	North Africa and Middle East	Bahrain	YLLs	Age-standardized	1.8 (1.4 to 2.2)	0.6 (0.4 to 0.8)	3.1 (2.4 to 3.9)	1.4 (1.1 to 2)	0.3 (0.2 to 0.4)	2.5 (1.9 to 3.4)	-19.2 (-44 to 16.2)	-55.1 (-72.2 to -26)	-17.5 (-44.1 to 20.3)
North Africa and Middle East	North Africa and Middle East	Bahrain	YLDs	All Ages	0 (0 to 1)	0 (0 to 0)	0 (0 to 0)	2 (1 to 3)	2 (1 to 3)	1 (0 to 1)	372.6 (307.9 to 453.3)	427 (336.2 to 542.9)	260.8 (171.9 to 369.2)
North Africa and Middle East	North Africa and Middle East	Bahrain	YLDs	Age-standardized	0.2 (0.1 to 0.2)	0.2 (0.1 to 0.3)	0.1 (0.1 to 0.2)	0.2 (0.1 to 0.3)	0.3 (0.2 to 0.5)	0.1 (0.1 to 0.2)	24.2 (13 to 37.1)	49.1 (31.5 to 68.9)	-0.5 (-16.3 to 18.4)
North Africa and Middle East	North Africa and Middle East	Egypt	Incidence	All Ages	65 (45 to 90)	48 (32 to 68)	17 (10 to 25)	294 (217 to 378)	254 (182 to 329)	40 (29 to 56)	352.6 (231 to 528.7)	424.7 (265.9 to 690.3)	141.7 (84.9 to 222.7)
North Africa and Middle East	North Africa and Middle East	Egypt	Incidence	Age-standardized	0.2 (0.1 to 0.2)	0.3 (0.2 to 0.3)	0.1 (0.1 to 0.1)	0.4 (0.3 to 0.5)	0.7 (0.5 to 0.8)	0.1 (0.1 to 0.1)	110.5 (65.5 to 173.3)	161.9 (95.9 to 266.5)	17.4 (-8.5 to 48.7)
North Africa and Middle East	North Africa and Middle East	Egypt	Prevalence	All Ages	148 (117 to 183)	76 (54 to 102)	71 (55 to 91)	616 (494 to 746)	381 (286 to 493)	235 (186 to 296)	316.5 (238.4 to 400.9)	397.5 (267.8 to 583.2)	229.8 (172.2 to 307.1)
North Africa and Middle East	North Africa and Middle East	Egypt	Prevalence	Age-standardized	0.5 (0.4 to 0.6)	0.4 (0.3 to 0.5)	0.5 (0.4 to 0.6)	1 (0.8 to 1.1)	1.1 (0.9 to 1.3)	0.8 (0.6 to 1)	105.2 (72.9 to 140.7)	148.5 (96.3 to 220.1)	59.8 (31 to 99.1)
North Africa and Middle East	North Africa and Middle East	Egypt	Deaths	All Ages	15 (10 to 19)	3 (2 to 4)	12 (8 to 17)	46 (31 to 71)	11 (7 to 17)	35 (21 to 59)	216.6 (97.3 to 495.1)	321.8 (133.3 to 679.8)	194 (65.4 to 533.6)
North Africa and Middle East	North Africa and Middle East	Egypt	Deaths	Age-standardized	0.1 (0 to 0.1)	0 (0 to 0)	0.1 (0.1 to 0.1)	0.1 (0.1 to 0.1)	0.1 (0 to 0.1)	0.1 (0.1 to 0.2)	43.5 (-7.9 to 165.4)	148.8 (43.4 to 353.5)	17 (-31.3 to 151.1)
North Africa and Middle East	North Africa and Middle East	Egypt	DALYs	All Ages	420 (300 to 550)	85 (55 to 123)	335 (225 to 463)	1,380 (930 to 2,072)	353 (229 to 535)	1,027 (627 to 1,668)	228.5 (108.9 to 490.3)	316.1 (156.3 to 592.4)	206.3 (79.7 to 541.1)
North Africa and Middle East	North Africa and Middle East	Egypt	DALYs	Age-standardized	1.3 (1 to 1.7)	0.5 (0.3 to 0.7)	2.1 (1.4 to 2.9)	2 (1.4 to 2.9)	1.2 (0.8 to 1.7)	2.7 (1.7 to 4.3)	49.4 (-2.8 to 167.9)	127.2 (40.6 to 282.1)	26.8 (-25.2 to 164.3)
North Africa and Middle East	North Africa and Middle East	Egypt	YLLs	All Ages	396 (274 to 522)	73 (46 to 110)	324 (212 to 452)	1,282 (828 to 1,965)	293 (177 to 476)	989 (588 to 1,626)	223.4 (99.9 to 508.3)	303 (126.1 to 644.5)	205.5 (74.2 to 561.5)
North Africa and Middle East	North Africa and Middle East	Egypt	YLLs	Age-standardized	1.2 (0.9 to 1.6)	0.4 (0.3 to 0.7)	2 (1.3 to 2.9)	1.8 (1.2 to 2.8)	1 (0.6 to 1.6)	2.6 (1.6 to 4.2)	46.2 (-9 to 175.1)	124.2 (29.9 to 306.9)	25.6 (-28.2 to 168.5)
North Africa and Middle East	North Africa and Middle East	Egypt	YLDs	All Ages	24 (15 to 34)	12 (7 to 19)	11 (7 to 17)	97 (64 to 143)	60 (38 to 91)	37 (24 to 56)	314.2 (237.3 to 395.8)	394.9 (266.1 to 580.2)	228.6 (172 to 304)
North Africa and Middle East	North Africa and Middle East	Egypt	YLDs	Age-standardized	0.1 (0 to 0.1)	0.1 (0 to 0.1)	0.1 (0.1 to 0.1)	0.2 (0.1 to 0.2)	0.2 (0.1 to 0.3)	0.1 (0.1 to 0.2)	103.8 (72.1 to 139.2)	146.8 (95.6 to 216.5)	58.9 (30.7 to 97.6)
North Africa and Middle East	North Africa and Middle East	Iran (Islamic Republic of)	Incidence	All Ages	290 (218 to 370)	262 (194 to 340)	28 (21 to 37)	2,027 (1,562 to 2,584)	1,949 (1,486 to 2,497)	78 (64 to 95)	600.1 (508.8 to 716.8)	645.4 (541.4 to 781.9)	177.8 (120.2 to 250.5)
North Africa and Middle East	North Africa and Middle East	Iran (Islamic Republic of)	Incidence	Age-standardized	0.8 (0.6 to 0.9)	1.3 (1 to 1.7)	0.2 (0.1 to 0.2)	2.4 (1.9 to 3)	4.6 (3.5 to 5.7)	0.2 (0.2 to 0.3)	212.8 (178.5 to 251.2)	238.6 (201.3 to 282.3)	20.9 (0.2 to 45.3)
North Africa and Middle East	North Africa and Middle East	Iran (Islamic Republic of)	Prevalence	All Ages	575 (457 to 711)	459 (352 to 583)	117 (97 to 141)	5,749 (4,497 to 7,201)	5,152 (3,866 to 6,583)	597 (489 to 722)	899.3 (773 to 1025.5)	1023.6 (866.4 to 1189.8)	411.2 (339.4 to 501)
North Africa and Middle East	North Africa and Middle East	Iran (Islamic Republic of)	Prevalence	Age-standardized	1.7 (1.5 to 2.1)	2.5 (2 to 3.2)	1 (0.8 to 1.2)	6.9 (5.6 to 8.5)	12.1 (9.4 to 15.2)	1.8 (1.5 to 2.2)	296 (254.6 to 335.7)	376.4 (327.5 to 429.6)	83 (60.6 to 111.5)
North Africa and Middle East	North Africa and Middle East	Iran (Islamic Republic of)	Deaths	All Ages	12 (9 to 16)	2 (1 to 3)	10 (7 to 13)	68 (59 to 77)	17 (13 to 20)	51 (43 to 59)	452 (310.1 to 725)	631.7 (392.4 to 1106.6)	410.8 (256.3 to 774.4)
North Africa and Middle East	North Africa and Middle East	Iran (Islamic Republic of)	Deaths	Age-standardized	0.1 (0 to 0.1)	0 (0 to 0)	0.1 (0.1 to 0.1)	0.1 (0.1 to 0.1)	0.1 (0 to 0.1)	0.2 (0.1 to 0.2)	89.6 (41.9 to 174.8)	128.3 (46.9 to 265.4)	77.6 (24.5 to 207.6)
North Africa and Middle East	North Africa and Middle East	Iran (Islamic Republic of)	DALYs	All Ages	443 (342 to 546)	141 (104 to 189)	303 (208 to 400)	2,422 (2,010 to 2,890)	1,176 (875 to 1,575)	1,246 (1,056 to 1,429)	446.6 (320.8 to 635.9)	736.4 (551.6 to 970.1)	311.9 (189.8 to 556.4)
North Africa and Middle East	North Africa and Middle East	Iran (Islamic Republic of)	DALYs	Age-standardized	1.4 (1.1 to 1.8)	0.9 (0.6 to 1.1)	2 (1.4 to 2.6)	3.1 (2.6 to 3.7)	2.9 (2.2 to 3.8)	3.4 (2.9 to 3.9)	117.3 (68.1 to 191.5)	237.9 (166.8 to 331.6)	69.8 (21.3 to 171)
North Africa and Middle East	North Africa and Middle East	Iran (Islamic Republic of)	YLLs	All Ages	352 (255 to 450)	68 (43 to 98)	284 (190 to 379)	1,534 (1,329 to 1,743)	383 (310 to 480)	1,151 (972 to 1,327)	335.8 (217.3 to 547.3)	460.3 (258.9 to 886.6)	305.8 (178.2 to 573.7)
North Africa and Middle East	North Africa and Middle East	Iran (Islamic Republic of)	YLLs	Age-standardized	1.2 (0.9 to 1.5)	0.5 (0.3 to 0.6)	1.8 (1.2 to 2.4)	2.1 (1.8 to 2.3)	1 (0.8 to 1.3)	3.1 (2.6 to 3.5)	77.6 (31.9 to 162.6)	128.8 (52.4 to 278.2)	68.9 (16.6 to 183.9)
North Africa and Middle East	North Africa and Middle East	Iran (Islamic Republic of)	YLDs	All Ages	91 (58 to 134)	72 (45 to 109)	19 (12 to 28)	888 (570 to 1,316)	793 (502 to 1,191)	95 (61 to 140)	874.9 (752.9 to 1000.5)	997.7 (846.4 to 1160.2)	404.7 (335.1 to 489.2)
North Africa and Middle East	North Africa and Middle East	Iran (Islamic Republic of)	YLDs	Age-standardized	0.3 (0.2 to 0.4)	0.4 (0.3 to 0.6)	0.2 (0.1 to 0.2)	1.1 (0.7 to 1.6)	1.9 (1.2 to 2.7)	0.3 (0.2 to 0.4)	283 (242.6 to 321.7)	361.2 (310.3 to 412.1)	80.6 (58.7 to 107.9)
North Africa and Middle East	North Africa and Middle East	Iraq	Incidence	All Ages	63 (47 to 82)	49 (36 to 65)	14 (10 to 18)	108 (80 to 143)	91 (64 to 124)	17 (12 to 24)	72.4 (43.6 to 108.6)	84.7 (47.6 to 133.9)	27.8 (-8.9 to 76.2)
North Africa and Middle East	North Africa and Middle East	Iraq	Incidence	Age-standardized	0.6 (0.4 to 0.7)	0.9 (0.6 to 1.1)	0.3 (0.2 to 0.4)	0.4 (0.3 to 0.5)	0.6 (0.4 to 0.7)	0.1 (0.1 to 0.2)	-37.8 (-46.2 to -26.9)	-33.5 (-45 to -19.2)	-52.1 (-63.5 to -37.5)
North Africa and Middle East	North Africa and Middle East	Iraq	Prevalence	All Ages	121 (98 to 147)	79 (60 to 102)	42 (34 to 53)	249 (202 to 300)	150 (113 to 191)	99 (80 to 126)	105.8 (79.6 to 138.5)	89.1 (55.2 to 130.6)	137.2 (102.5 to 174.8)
North Africa and Middle East	North Africa and Middle East	Iraq	Prevalence	Age-standardized	1.3 (1.1 to 1.5)	1.5 (1.2 to 1.9)	1.1 (0.9 to 1.4)	1.1 (0.9 to 1.3)	1.1 (0.9 to 1.3)	1.1 (0.8 to 1.4)	-18.9 (-27.7 to -8.7)	-29.1 (-39.6 to -16.6)	-3.7 (-17 to 11.8)
North Africa and Middle East	North Africa and Middle East	Iraq	Deaths	All Ages	10 (7 to 14)	4 (2 to 6)	6 (4 to 10)	27 (20 to 36)	14 (9 to 21)	13 (9 to 18)	179.3 (73.6 to 348.9)	288.1 (118.4 to 615.6)	116.6 (16.8 to 307.8)
North Africa and Middle East	North Africa and Middle East	Iraq	Deaths	Age-standardized	0.1 (0.1 to 0.2)	0.1 (0.1 to 0.2)	0.2 (0.1 to 0.3)	0.1 (0.1 to 0.2)	0.1 (0.1 to 0.2)	0.1 (0.1 to 0.2)	0.1 (-36.7 to 59.6)	39 (-18.4 to 159.7)	-22.2 (-58.9 to 48)
North Africa and Middle East	North Africa and Middle East	Iraq	DALYs	All Ages	252 (179 to 353)	104 (63 to 156)	148 (91 to 229)	731 (521 to 994)	390 (259 to 568)	341 (237 to 459)	190.3 (82.9 to 368.5)	276.6 (114.5 to 573.1)	130 (26.1 to 317.1)
North Africa and Middle East	North Africa and Middle East	Iraq	DALYs	Age-standardized	3 (2.1 to 4.3)	2.3 (1.4 to 3.6)	3.8 (2.4 to 5.9)	2.9 (2.2 to 3.9)	2.9 (2 to 4.3)	3 (2.1 to 4)	-3.6 (-38.4 to 53.7)	24.9 (-29.4 to 123.8)	-22.1 (-56.3 to 43.7)
North Africa and Middle East	North Africa and Middle East	Iraq	YLLs	All Ages	232 (160 to 332)	91 (52 to 145)	141 (84 to 222)	691 (488 to 952)	366 (234 to 542)	325 (221 to 442)	197.3 (82.4 to 397.1)	302.4 (116.3 to 696.1)	129.6 (21.5 to 331.1)
North Africa and Middle East	North Africa and Middle East	Iraq	YLLs	Age-standardized	2.8 (2 to 4.1)	2.1 (1.2 to 3.3)	3.6 (2.2 to 5.7)	2.8 (2 to 3.7)	2.8 (1.8 to 4.1)	2.8 (2 to 3.8)	-2.5 (-39.4 to 61)	31 (-29.3 to 155.9)	-23 (-58.3 to 45)
North Africa and Middle East	North Africa and Middle East	Iraq	YLDs	All Ages	19 (12 to 28)	13 (8 to 19)	7 (4 to 10)	40 (26 to 57)	24 (15 to 36)	16 (10 to 24)	106.3 (80.5 to 138.6)	89.9 (56.1 to 131.3)	137 (102.9 to 174.4)
North Africa and Middle East	North Africa and Middle East	Iraq	YLDs	Age-standardized	0.2 (0.1 to 0.3)	0.2 (0.2 to 0.4)	0.2 (0.1 to 0.3)	0.2 (0.1 to 0.2)	0.2 (0.1 to 0.2)	0.2 (0.1 to 0.3)	-18.7 (-27.3 to -8.7)	-28.7 (-39.2 to -16.5)	-4.2 (-17.2 to 10.8)
North Africa and Middle East	North Africa and Middle East	Jordan	Incidence	All Ages	12 (9 to 16)	10 (7 to 14)	2 (1 to 3)	32 (23 to 41)	28 (20 to 37)	3 (2 to 5)	161.1 (116.4 to 211.6)	183.3 (130.6 to 248.6)	59 (3.1 to 135.7)
North Africa and Middle East	North Africa and Middle East	Jordan	Incidence	Age-standardized	0.5 (0.4 to 0.7)	0.9 (0.6 to 1.1)	0.2 (0.2 to 0.3)	0.4 (0.3 to 0.4)	0.7 (0.5 to 0.8)	0.1 (0.1 to 0.1)	-34.5 (-43.2 to -25.7)	-24.3 (-35.9 to -11.8)	-62.4 (-74.7 to -47.9)
North Africa and Middle East	North Africa and Middle East	Jordan	Prevalence	All Ages	20 (15 to 26)	15 (11 to 20)	5 (4 to 6)	59 (47 to 73)	42 (31 to 54)	17 (13 to 22)	195.4 (152.7 to 242.4)	177.8 (128.2 to 230.6)	248.5 (179.9 to 330.9)
North Africa and Middle East	North Africa and Middle East	Jordan	Prevalence	Age-standardized	1 (0.8 to 1.2)	1.4 (1.1 to 1.8)	0.7 (0.5 to 0.8)	0.8 (0.6 to 0.9)	1 (0.8 to 1.3)	0.6 (0.5 to 0.7)	-23.3 (-32.1 to -14.5)	-27.3 (-37.1 to -17.1)	-12.5 (-28.6 to 5.9)
North Africa and Middle East	North Africa and Middle East	Jordan	Deaths	All Ages	0 (0 to 0)	0 (0 to 0)	0 (0 to 0)	0 (0 to 0)	0 (0 to 0)	0 (0 to 0)	145.3 (51.4 to 283.3)	283.9 (135 to 651.1)	120.4 (30.6 to 269.8)
North Africa and Middle East	North Africa and Middle East	Jordan	Deaths	Age-standardized	0 (0 to 0)	0 (0 to 0)	0 (0 to 0)	0 (0 to 0)	0 (0 to 0)	0 (0 to 0)	-47.5 (-67 to -18)	-9.8 (-43.2 to 63.7)	-55.3 (-73.1 to -26)
North Africa and Middle East	North Africa and Middle East	Jordan	DALYs	All Ages	7 (6 to 10)	3 (2 to 5)	4 (3 to 5)	20 (16 to 25)	10 (7 to 15)	10 (7 to 13)	171 (109.1 to 248.2)	205.8 (136.8 to 298.9)	142.3 (57.9 to 257.7)
North Africa and Middle East	North Africa and Middle East	Jordan	DALYs	Age-standardized	0.4 (0.3 to 0.6)	0.3 (0.2 to 0.4)	0.6 (0.4 to 0.8)	0.3 (0.2 to 0.3)	0.2 (0.2 to 0.3)	0.3 (0.2 to 0.4)	-38.5 (-53.5 to -20)	-20.2 (-33 to -1.4)	-48.8 (-66.1 to -23.1)
North Africa and Middle East	North Africa and Middle East	Jordan	YLLs	All Ages	4 (3 to 6)	1 (1 to 2)	3 (2 to 5)	11 (8 to 14)	4 (2 to 6)	7 (5 to 9)	152.5 (58.4 to 289.6)	276 (99.2 to 715.6)	116.5 (27.7 to 262.2)
North Africa and Middle East	North Africa and Middle East	Jordan	YLLs	Age-standardized	0.3 (0.2 to 0.4)	0.1 (0 to 0.1)	0.5 (0.3 to 0.6)	0.1 (0.1 to 0.2)	0.1 (0.1 to 0.1)	0.2 (0.1 to 0.3)	-47.7 (-67.9 to -18.6)	0.3 (-38.1 to 97.5)	-57 (-74.7 to -26.9)
North Africa and Middle East	North Africa and Middle East	Jordan	YLDs	All Ages	3 (2 to 5)	2 (1 to 4)	1 (1 to 1)	9 (6 to 14)	7 (4 to 10)	3 (2 to 4)	195.5 (153 to 242.5)	177.8 (128.3 to 230.6)	248.6 (180.5 to 331.2)
North Africa and Middle East	North Africa and Middle East	Jordan	YLDs	Age-standardized	0.2 (0.1 to 0.2)	0.2 (0.1 to 0.3)	0.1 (0.1 to 0.2)	0.1 (0.1 to 0.2)	0.2 (0.1 to 0.2)	0.1 (0.1 to 0.1)	-23.2 (-32.1 to -14.5)	-27.3 (-37.1 to -17.1)	-12.4 (-28.5 to 5.8)
North Africa and Middle East	North Africa and Middle East	Kuwait	Incidence	All Ages	3 (2 to 4)	2 (1 to 3)	1 (0 to 1)	18 (12 to 24)	16 (10 to 22)	2 (1 to 2)	565.2 (380.2 to 850.4)	747.8 (479.6 to 1203)	114.1 (37.8 to 232.7)
North Africa and Middle East	North Africa and Middle East	Kuwait	Incidence	Age-standardized	0.2 (0.2 to 0.3)	0.4 (0.3 to 0.5)	0.1 (0.1 to 0.2)	0.4 (0.3 to 0.5)	0.8 (0.6 to 1)	0.1 (0.1 to 0.1)	89.3 (51.5 to 138.9)	124.8 (73.8 to 201.4)	-23.4 (-43.9 to 2.9)
North Africa and Middle East	North Africa and Middle East	Kuwait	Prevalence	All Ages	5 (4 to 7)	3 (2 to 4)	2 (2 to 3)	33 (25 to 41)	24 (18 to 33)	8 (6 to 10)	527.9 (412.1 to 685.1)	772.9 (543.6 to 1158.1)	241.8 (172.9 to 323)
North Africa and Middle East	North Africa and Middle East	Kuwait	Prevalence	Age-standardized	0.6 (0.4 to 0.7)	0.6 (0.5 to 0.8)	0.5 (0.4 to 0.7)	0.9 (0.7 to 1.1)	1.3 (1 to 1.7)	0.6 (0.4 to 0.7)	65.2 (43.2 to 91.2)	122.9 (82.1 to 178.8)	7.2 (-12.5 to 30.8)
North Africa and Middle East	North Africa and Middle East	Kuwait	Deaths	All Ages	0 (0 to 0)	0 (0 to 0)	0 (0 to 0)	0 (0 to 0)	0 (0 to 0)	0 (0 to 0)	-15.4 (-41.1 to 23.2)	75.8 (12 to 189.9)	-19.7 (-45.4 to 20)
North Africa and Middle East	North Africa and Middle East	Kuwait	Deaths	Age-standardized	0 (0 to 0)	0 (0 to 0)	0 (0 to 0.1)	0 (0 to 0)	0 (0 to 0)	0 (0 to 0)	-77.4 (-84 to -67.9)	-56.5 (-73.1 to -28)	-78.6 (-85 to -69.5)
North Africa and Middle East	North Africa and Middle East	Kuwait	DALYs	All Ages	5 (4 to 6)	1 (0 to 1)	5 (3 to 6)	8 (6 to 11)	4 (3 to 6)	4 (3 to 5)	57.4 (13.4 to 116.8)	580.4 (419.1 to 807.7)	-11.6 (-38.1 to 25.1)
North Africa and Middle East	North Africa and Middle East	Kuwait	DALYs	Age-standardized	0.8 (0.6 to 1)	0.2 (0.1 to 0.2)	1.1 (0.9 to 1.5)	0.3 (0.2 to 0.3)	0.2 (0.2 to 0.3)	0.3 (0.2 to 0.4)	-65.3 (-74.7 to -52.7)	50 (18.6 to 90)	-75.3 (-82.4 to -65.3)
North Africa and Middle East	North Africa and Middle East	Kuwait	YLLs	All Ages	4 (3 to 6)	0 (0 to 0)	4 (3 to 6)	3 (2 to 4)	0 (0 to 0)	3 (2 to 4)	-31.6 (-53.2 to 5.1)	49.5 (-2.6 to 146.7)	-34.7 (-56.4 to 2.1)
North Africa and Middle East	North Africa and Middle East	Kuwait	YLLs	Age-standardized	0.7 (0.5 to 0.9)	0.1 (0 to 0.1)	1 (0.8 to 1.4)	0.1 (0.1 to 0.2)	0 (0 to 0)	0.2 (0.1 to 0.3)	-81.9 (-87.6 to -73)	-61.7 (-75.6 to -33.6)	-81.9 (-87.9 to -72.6)
North Africa and Middle East	North Africa and Middle East	Kuwait	YLDs	All Ages	1 (1 to 1)	0 (0 to 1)	0 (0 to 1)	5 (3 to 8)	4 (2 to 6)	1 (1 to 2)	526.8 (411.6 to 684.2)	771.7 (543.5 to 1157.6)	242.1 (173.7 to 322.9)
North Africa and Middle East	North Africa and Middle East	Kuwait	YLDs	Age-standardized	0.1 (0.1 to 0.1)	0.1 (0.1 to 0.1)	0.1 (0.1 to 0.1)	0.1 (0.1 to 0.2)	0.2 (0.1 to 0.3)	0.1 (0.1 to 0.1)	65 (43.1 to 90.8)	122.6 (82 to 178.5)	7.2 (-12.3 to 30.6)
North Africa and Middle East	North Africa and Middle East	Lebanon	Incidence	All Ages	5 (4 to 7)	4 (3 to 6)	1 (1 to 2)	12 (9 to 15)	10 (7 to 13)	3 (2 to 4)	132.8 (96.1 to 182.8)	135.1 (93.1 to 196.7)	125 (65.7 to 219.9)
North Africa and Middle East	North Africa and Middle East	Lebanon	Incidence	Age-standardized	0.2 (0.2 to 0.3)	0.3 (0.2 to 0.4)	0.1 (0.1 to 0.1)	0.2 (0.2 to 0.3)	0.3 (0.2 to 0.5)	0.1 (0.1 to 0.1)	10.8 (-5 to 31)	9.3 (-9.6 to 37.2)	10 (-17.6 to 49.8)
North Africa and Middle East	North Africa and Middle East	Lebanon	Prevalence	All Ages	12 (10 to 15)	6 (5 to 9)	6 (4 to 7)	36 (30 to 43)	16 (12 to 20)	20 (16 to 25)	198 (156.3 to 254.7)	146.5 (109.2 to 199.6)	258.7 (183.8 to 355)
North Africa and Middle East	North Africa and Middle East	Lebanon	Prevalence	Age-standardized	0.5 (0.4 to 0.6)	0.5 (0.4 to 0.7)	0.5 (0.4 to 0.7)	0.7 (0.6 to 0.8)	0.6 (0.4 to 0.7)	0.9 (0.7 to 1.1)	31.2 (14.2 to 53.8)	9.8 (-5.8 to 28.9)	60.7 (28.9 to 103.7)
North Africa and Middle East	North Africa and Middle East	Lebanon	Deaths	All Ages	1 (0 to 1)	0 (0 to 0)	1 (0 to 1)	2 (1 to 4)	1 (0 to 1)	1 (1 to 3)	181.1 (72.9 to 428.5)	357.7 (174 to 752.2)	138.1 (24.8 to 458.7)
North Africa and Middle East	North Africa and Middle East	Lebanon	Deaths	Age-standardized	0 (0 to 0.1)	0 (0 to 0)	0.1 (0 to 0.1)	0 (0 to 0.1)	0 (0 to 0)	0.1 (0 to 0.1)	11.8 (-30.5 to 113.9)	61.2 (-2.4 to 200.6)	2.6 (-46.7 to 145.5)
North Africa and Middle East	North Africa and Middle East	Lebanon	DALYs	All Ages	19 (13 to 27)	4 (3 to 6)	15 (9 to 23)	48 (32 to 82)	15 (10 to 20)	33 (19 to 67)	153.1 (60.4 to 355.3)	234.9 (119.2 to 425.7)	128.3 (23.9 to 390.6)
North Africa and Middle East	North Africa and Middle East	Lebanon	DALYs	Age-standardized	0.8 (0.6 to 1.2)	0.4 (0.3 to 0.5)	1.3 (0.8 to 1.9)	0.9 (0.6 to 1.6)	0.5 (0.4 to 0.7)	1.4 (0.8 to 2.9)	15 (-26.7 to 106.7)	44.8 (-5.4 to 126.2)	11.6 (-39.5 to 140.2)
North Africa and Middle East	North Africa and Middle East	Lebanon	YLLs	All Ages	17 (11 to 25)	3 (2 to 5)	14 (8 to 22)	43 (27 to 76)	12 (8 to 18)	30 (16 to 64)	148.2 (48.8 to 370.2)	261.3 (114.6 to 535.4)	120.1 (11.3 to 393.6)
North Africa and Middle East	North Africa and Middle East	Lebanon	YLLs	Age-standardized	0.7 (0.5 to 1.1)	0.3 (0.2 to 0.4)	1.2 (0.7 to 1.8)	0.8 (0.5 to 1.5)	0.4 (0.3 to 0.6)	1.3 (0.7 to 2.7)	13.2 (-31.9 to 114.1)	54.9 (-8.3 to 177.2)	8.2 (-45.2 to 145.4)
North Africa and Middle East	North Africa and Middle East	Lebanon	YLDs	All Ages	2 (1 to 3)	1 (1 to 2)	1 (1 to 1)	6 (4 to 8)	3 (2 to 4)	3 (2 to 5)	197.6 (156 to 253.5)	147 (109.8 to 199.7)	257.1 (183.1 to 351.8)
North Africa and Middle East	North Africa and Middle East	Lebanon	YLDs	Age-standardized	0.1 (0.1 to 0.1)	0.1 (0.1 to 0.1)	0.1 (0.1 to 0.1)	0.1 (0.1 to 0.2)	0.1 (0.1 to 0.1)	0.1 (0.1 to 0.2)	31 (14 to 53.2)	9.9 (-5.7 to 28.7)	59.9 (28.7 to 102.5)
North Africa and Middle East	North Africa and Middle East	Libya	Incidence	All Ages	5 (3 to 6)	3 (2 to 5)	1 (1 to 2)	30 (22 to 39)	27 (20 to 36)	3 (2 to 5)	564.7 (398.9 to 812.1)	713.2 (474.1 to 1112.1)	163.3 (89.9 to 260.2)
North Africa and Middle East	North Africa and Middle East	Libya	Incidence	Age-standardized	0.2 (0.1 to 0.2)	0.3 (0.2 to 0.4)	0.1 (0.1 to 0.1)	0.5 (0.4 to 0.6)	0.8 (0.6 to 1.1)	0.1 (0.1 to 0.2)	166.3 (113.6 to 242.3)	211.8 (133.7 to 322.1)	22.6 (-5.6 to 56.8)
North Africa and Middle East	North Africa and Middle East	Libya	Prevalence	All Ages	10 (8 to 13)	5 (4 to 7)	5 (4 to 7)	61 (49 to 74)	42 (32 to 55)	18 (14 to 22)	480.5 (380.8 to 608.1)	714.1 (517.2 to 1003.8)	247 (190.3 to 331.4)
North Africa and Middle East	North Africa and Middle East	Libya	Prevalence	Age-standardized	0.5 (0.4 to 0.6)	0.5 (0.3 to 0.6)	0.5 (0.4 to 0.7)	1.1 (0.9 to 1.3)	1.4 (1.1 to 1.7)	0.8 (0.6 to 1)	119.1 (87.1 to 158.6)	198.6 (139.4 to 275.3)	50.5 (22.6 to 88)
North Africa and Middle East	North Africa and Middle East	Libya	Deaths	All Ages	1 (0 to 1)	0 (0 to 0)	0 (0 to 1)	2 (1 to 3)	1 (0 to 1)	2 (1 to 2)	322.4 (136.5 to 679)	504.3 (250.9 to 1026.8)	275.8 (80.6 to 725.6)
North Africa and Middle East	North Africa and Middle East	Libya	Deaths	Age-standardized	0 (0 to 0)	0 (0 to 0)	0 (0 to 0.1)	0 (0 to 0.1)	0 (0 to 0)	0.1 (0 to 0.1)	54.3 (-13.4 to 182.9)	122.5 (25.2 to 320.1)	39.4 (-32.6 to 207.1)
North Africa and Middle East	North Africa and Middle East	Libya	DALYs	All Ages	14 (10 to 20)	4 (2 to 5)	11 (7 to 16)	65 (44 to 90)	22 (15 to 32)	42 (26 to 67)	353.5 (168.2 to 683.7)	516.1 (296.8 to 880.8)	298.5 (98.1 to 727.9)
North Africa and Middle East	North Africa and Middle East	Libya	DALYs	Age-standardized	0.7 (0.5 to 1)	0.3 (0.2 to 0.5)	1 (0.6 to 1.5)	1.1 (0.8 to 1.6)	0.8 (0.5 to 1.1)	1.5 (0.9 to 2.3)	66.7 (-2.2 to 186.8)	133.3 (53.1 to 278.1)	49.7 (-23.6 to 205.9)
North Africa and Middle East	North Africa and Middle East	Libya	YLLs	All Ages	13 (8 to 19)	3 (2 to 4)	10 (6 to 15)	55 (36 to 79)	15 (9 to 25)	40 (23 to 64)	337.2 (137.4 to 742)	458.3 (200.8 to 980.3)	303 (91.7 to 791.1)
North Africa and Middle East	North Africa and Middle East	Libya	YLLs	Age-standardized	0.6 (0.4 to 0.9)	0.3 (0.1 to 0.4)	0.9 (0.6 to 1.5)	1 (0.6 to 1.4)	0.5 (0.3 to 0.9)	1.4 (0.8 to 2.2)	60.2 (-13.1 to 199.9)	115.3 (15.9 to 310.8)	49.7 (-29.3 to 229.5)
North Africa and Middle East	North Africa and Middle East	Libya	YLDs	All Ages	2 (1 to 2)	1 (0 to 1)	1 (1 to 1)	10 (6 to 14)	7 (4 to 10)	3 (2 to 4)	476.8 (379 to 599.6)	710.9 (515.5 to 994.3)	245.4 (190 to 328.8)
North Africa and Middle East	North Africa and Middle East	Libya	YLDs	Age-standardized	0.1 (0.1 to 0.1)	0.1 (0 to 0.1)	0.1 (0.1 to 0.1)	0.2 (0.1 to 0.2)	0.2 (0.1 to 0.3)	0.1 (0.1 to 0.2)	117.4 (85.9 to 155.7)	196.8 (138.4 to 272)	49.6 (22.4 to 86.1)
North Africa and Middle East	North Africa and Middle East	Morocco	Incidence	All Ages	28 (19 to 38)	20 (13 to 29)	7 (4 to 11)	120 (91 to 156)	105 (76 to 139)	16 (11 to 21)	336.6 (229.5 to 508.3)	412 (257.7 to 672.1)	119.9 (67.4 to 217.4)
North Africa and Middle East	North Africa and Middle East	Morocco	Incidence	Age-standardized	0.2 (0.1 to 0.2)	0.2 (0.2 to 0.3)	0.1 (0.1 to 0.1)	0.4 (0.3 to 0.4)	0.6 (0.4 to 0.8)	0.1 (0.1 to 0.1)	123.4 (78 to 186.7)	170 (100 to 280.3)	14.2 (-8.5 to 51.2)
North Africa and Middle East	North Africa and Middle East	Morocco	Prevalence	All Ages	65 (51 to 81)	33 (23 to 44)	32 (25 to 41)	254 (206 to 308)	160 (122 to 204)	94 (74 to 119)	291.1 (230.9 to 373.6)	387.3 (270.6 to 558.1)	192.9 (147.3 to 259.3)
North Africa and Middle East	North Africa and Middle East	Morocco	Prevalence	Age-standardized	0.4 (0.4 to 0.5)	0.4 (0.3 to 0.5)	0.5 (0.4 to 0.6)	0.8 (0.7 to 1)	1 (0.7 to 1.2)	0.7 (0.6 to 0.9)	90.4 (64.1 to 124.7)	146.7 (96.5 to 214.1)	47.2 (22.5 to 79.1)
North Africa and Middle East	North Africa and Middle East	Morocco	Deaths	All Ages	5 (2 to 7)	1 (0 to 2)	4 (2 to 7)	17 (11 to 23)	5 (3 to 7)	12 (7 to 18)	235.9 (120.8 to 489)	410.9 (213.5 to 850.3)	197.8 (81.9 to 489.9)
North Africa and Middle East	North Africa and Middle East	Morocco	Deaths	Age-standardized	0 (0 to 0.1)	0 (0 to 0)	0.1 (0 to 0.1)	0.1 (0 to 0.1)	0 (0 to 0.1)	0.1 (0.1 to 0.1)	49.9 (-0.2 to 164.8)	140.7 (46.7 to 335.8)	27.4 (-19.9 to 153)
North Africa and Middle East	North Africa and Middle East	Morocco	DALYs	All Ages	136 (73 to 194)	30 (15 to 47)	105 (49 to 162)	446 (303 to 614)	137 (89 to 204)	309 (191 to 458)	228.7 (122.2 to 440.7)	350.2 (194.4 to 642)	193.6 (81.5 to 447.7)
North Africa and Middle East	North Africa and Middle East	Morocco	DALYs	Age-standardized	0.9 (0.5 to 1.3)	0.4 (0.2 to 0.6)	1.5 (0.7 to 2.3)	1.4 (1 to 1.9)	0.8 (0.6 to 1.2)	2 (1.2 to 2.9)	52 (2.9 to 151.8)	129.1 (50.1 to 271.3)	31.6 (-17.8 to 145)
North Africa and Middle East	North Africa and Middle East	Morocco	YLLs	All Ages	125 (61 to 184)	25 (9 to 42)	100 (44 to 157)	406 (263 to 575)	112 (66 to 178)	294 (175 to 443)	223.7 (111.6 to 467.3)	343 (169 to 744.6)	193.7 (76.9 to 474.6)
North Africa and Middle East	North Africa and Middle East	Morocco	YLLs	Age-standardized	0.9 (0.4 to 1.3)	0.3 (0.1 to 0.5)	1.4 (0.6 to 2.2)	1.3 (0.8 to 1.8)	0.7 (0.4 to 1.1)	1.8 (1.1 to 2.8)	49 (-2 to 160.1)	125.9 (39.2 to 312)	30.8 (-19.8 to 159.8)
North Africa and Middle East	North Africa and Middle East	Morocco	YLDs	All Ages	10 (7 to 15)	5 (3 to 8)	5 (3 to 8)	40 (26 to 58)	25 (16 to 38)	15 (10 to 23)	288.7 (229.8 to 369.4)	384.9 (270.2 to 554.4)	191.5 (147.2 to 256.2)
North Africa and Middle East	North Africa and Middle East	Morocco	YLDs	Age-standardized	0.1 (0 to 0.1)	0.1 (0 to 0.1)	0.1 (0.1 to 0.1)	0.1 (0.1 to 0.2)	0.2 (0.1 to 0.2)	0.1 (0.1 to 0.2)	89 (63.3 to 122.4)	145.1 (95.9 to 212.1)	46.2 (22.1 to 77.3)
North Africa and Middle East	North Africa and Middle East	Palestine	Incidence	All Ages	2 (2 to 3)	2 (1 to 3)	1 (0 to 1)	7 (5 to 9)	6 (4 to 8)	1 (1 to 2)	174.3 (120.7 to 248.9)	190.4 (125.8 to 290.2)	111.5 (32.8 to 193.6)
North Africa and Middle East	North Africa and Middle East	Palestine	Incidence	Age-standardized	0.2 (0.2 to 0.3)	0.3 (0.2 to 0.4)	0.1 (0.1 to 0.1)	0.2 (0.2 to 0.3)	0.3 (0.2 to 0.4)	0.1 (0.1 to 0.1)	0.4 (-16 to 21.6)	8.4 (-12.8 to 35.6)	-20.9 (-42.4 to 4)
North Africa and Middle East	North Africa and Middle East	Palestine	Prevalence	All Ages	5 (4 to 6)	3 (2 to 4)	2 (2 to 3)	14 (11 to 18)	9 (6 to 12)	5 (4 to 7)	182.8 (146 to 225.1)	188.2 (138.5 to 259.9)	174.6 (126.5 to 219.8)
North Africa and Middle East	North Africa and Middle East	Palestine	Prevalence	Age-standardized	0.5 (0.4 to 0.6)	0.5 (0.4 to 0.6)	0.5 (0.4 to 0.6)	0.5 (0.4 to 0.7)	0.6 (0.4 to 0.7)	0.6 (0.4 to 0.7)	9.6 (-2.9 to 23.5)	8.7 (-8.2 to 28.7)	15.2 (-5.2 to 37.4)
North Africa and Middle East	North Africa and Middle East	Palestine	Deaths	All Ages	0 (0 to 0)	0 (0 to 0)	0 (0 to 0)	0 (0 to 0)	0 (0 to 0)	0 (0 to 0)	82 (19 to 196.6)	132.6 (31.5 to 326.7)	27.4 (-29.9 to 149.8)
North Africa and Middle East	North Africa and Middle East	Palestine	Deaths	Age-standardized	0 (0 to 0)	0 (0 to 0)	0 (0 to 0)	0 (0 to 0)	0 (0 to 0)	0 (0 to 0)	-18.2 (-47 to 32.1)	5.4 (-39.9 to 96)	-41.6 (-68.9 to 23.6)
North Africa and Middle East	North Africa and Middle East	Palestine	DALYs	All Ages	6 (4 to 8)	3 (2 to 5)	3 (2 to 4)	11 (8 to 14)	7 (5 to 9)	4 (3 to 5)	82.6 (23 to 169.9)	107.9 (22.1 to 251.1)	50.7 (-10.5 to 149.8)
North Africa and Middle East	North Africa and Middle East	Palestine	DALYs	Age-standardized	0.6 (0.4 to 0.8)	0.6 (0.3 to 0.9)	0.6 (0.4 to 0.9)	0.4 (0.3 to 0.5)	0.5 (0.3 to 0.7)	0.3 (0.3 to 0.4)	-25.9 (-49.2 to 9.9)	-11.7 (-47.7 to 46.8)	-40.7 (-64.2 to 0.2)
North Africa and Middle East	North Africa and Middle East	Palestine	YLLs	All Ages	5 (3 to 8)	3 (2 to 5)	2 (1 to 4)	9 (7 to 11)	6 (4 to 8)	3 (2 to 4)	67.5 (5.3 to 165.6)	94.6 (4.2 to 264.4)	34.1 (-26.1 to 144.8)
North Africa and Middle East	North Africa and Middle East	Palestine	YLLs	Age-standardized	0.5 (0.3 to 0.7)	0.5 (0.3 to 0.8)	0.5 (0.3 to 0.8)	0.3 (0.2 to 0.4)	0.4 (0.3 to 0.6)	0.3 (0.2 to 0.3)	-31.6 (-55.8 to 10.3)	-15.1 (-53.4 to 57.2)	-49.3 (-72.2 to -4.3)
North Africa and Middle East	North Africa and Middle East	Palestine	YLDs	All Ages	1 (1 to 1)	0 (0 to 1)	0 (0 to 0)	2 (1 to 3)	1 (1 to 2)	1 (1 to 1)	182.4 (146 to 224.3)	187.8 (138.6 to 258.4)	174.2 (126.6 to 219.5)
North Africa and Middle East	North Africa and Middle East	Palestine	YLDs	Age-standardized	0.1 (0.1 to 0.1)	0.1 (0.1 to 0.1)	0.1 (0.1 to 0.1)	0.1 (0.1 to 0.1)	0.1 (0.1 to 0.1)	0.1 (0.1 to 0.1)	9.4 (-2.9 to 23.2)	8.6 (-8.2 to 28.2)	15 (-5.2 to 37.2)
North Africa and Middle East	North Africa and Middle East	Oman	Incidence	All Ages	3 (2 to 4)	2 (1 to 3)	1 (0 to 1)	9 (7 to 13)	8 (5 to 11)	2 (1 to 3)	247.8 (170.3 to 362.5)	295.8 (202.8 to 447.1)	125.3 (40 to 251.6)
North Africa and Middle East	North Africa and Middle East	Oman	Incidence	Age-standardized	0.2 (0.2 to 0.3)	0.4 (0.3 to 0.6)	0.1 (0.1 to 0.2)	0.3 (0.2 to 0.4)	0.6 (0.4 to 0.8)	0.1 (0.1 to 0.1)	20.8 (2.4 to 43.3)	44.3 (18.4 to 80.6)	-10.8 (-33.7 to 21.2)
North Africa and Middle East	North Africa and Middle East	Oman	Prevalence	All Ages	5 (4 to 7)	3 (2 to 4)	2 (2 to 3)	18 (14 to 23)	11 (8 to 16)	7 (5 to 9)	253 (200.2 to 320.3)	296.1 (214.7 to 412.8)	197.8 (136.9 to 262.1)
North Africa and Middle East	North Africa and Middle East	Oman	Prevalence	Age-standardized	0.6 (0.5 to 0.7)	0.7 (0.5 to 0.9)	0.5 (0.4 to 0.7)	0.8 (0.7 to 0.9)	1 (0.8 to 1.2)	0.8 (0.6 to 1)	38.3 (21.2 to 58.6)	44.1 (21.4 to 73.4)	42.5 (16.3 to 79.6)

Pneumoconiosis

7 GBD super region	21 GBD region	Location	Measure	Age	1990			2019			Percent change (1990 to 2019)		
					Both	Female	Male	Both	Female	Male	Both	Female	Male
North Africa and Middle East	North Africa and Middle East	Oman	Deaths	All Ages	0 (0 to 1)	0 (0 to 0)	0 (0 to 1)	1 (1 to 1)	0 (0 to 0)	1 (0 to 1)	68.1 (-8.2 to 236.5)	194.7 (47.9 to 488.2)	41.2 (-34.5 to 246.1)
North Africa and Middle East	North Africa and Middle East	Oman	Deaths	Age-standardized	0.1 (0 to 0.1)	0 (0 to 0.1)	0.1 (0.1 to 0.2)	0.1 (0 to 0.1)	0 (0 to 0.1)	0.1 (0 to 0.1)	-18.2 (-55.3 to 67.5)	51.8 (-25.1 to 208.7)	-38.9 (-71.7 to 77.1)
North Africa and Middle East	North Africa and Middle East	Oman	DALYs	All Ages	15 (9 to 23)	3 (2 to 5)	12 (6 to 20)	27 (19 to 37)	9 (6 to 13)	18 (12 to 27)	77.7 (1.1 to 228.8)	191.8 (65.3 to 426.2)	49.9 (-27.8 to 238.3)
North Africa and Middle East	North Africa and Middle East	Oman	DALYs	Age-standardized	1.8 (1.1 to 2.9)	0.8 (0.4 to 1.3)	2.8 (1.4 to 4.7)	1.3 (0.9 to 1.8)	1 (0.7 to 1.5)	1.6 (1 to 2.4)	-28.9 (-60 to 38.3)	27.3 (-28.8 to 129.3)	-42.9 (-71.9 to 39.9)
North Africa and Middle East	North Africa and Middle East	Oman	YLLs	All Ages	14 (8 to 23)	3 (1 to 4)	12 (6 to 19)	24 (16 to 33)	7 (4 to 11)	17 (11 to 25)	67.6 (-9.9 to 225.1)	172.8 (36.5 to 470.2)	45.4 (-31.8 to 240.8)
North Africa and Middle East	North Africa and Middle East	Oman	YLLs	Age-standardized	1.8 (1 to 2.8)	0.7 (0.3 to 1.2)	2.7 (1.3 to 4.6)	1.2 (0.8 to 1.6)	0.8 (0.5 to 1.3)	1.5 (0.9 to 2.3)	-32.4 (-63.4 to 39.1)	24.7 (-36.4 to 152.5)	-45.5 (-74 to 38.7)
North Africa and Middle East	North Africa and Middle East	Oman	YLDs	All Ages	1 (1 to 1)	0 (0 to 1)	0 (0 to 1)	3 (2 to 4)	2 (1 to 3)	1 (1 to 2)	252.6 (200.3 to 319.5)	295.7 (214.6 to 411.8)	198 (138.6 to 261.9)
North Africa and Middle East	North Africa and Middle East	Oman	YLDs	Age-standardized	0.1 (0.1 to 0.1)	0.1 (0.1 to 0.2)	0.1 (0.1 to 0.1)	0.1 (0.1 to 0.2)	0.2 (0.1 to 0.2)	0.1 (0.1 to 0.2)	37.8 (21 to 57.9)	43.9 (21.4 to 73)	41.5 (15.8 to 78.1)
North Africa and Middle East	North Africa and Middle East	Qatar	Incidence	All Ages	0 (0 to 1)	0 (0 to 0)	0 (0 to 0)	4 (3 to 6)	3 (2 to 4)	1 (1 to 2)	890.3 (620.4 to 1330.4)	1139.4 (747.9 to 1887.3)	570.5 (298.7 to 980.5)
North Africa and Middle East	North Africa and Middle East	Qatar	Incidence	Age-standardized	0.1 (0.1 to 0.2)	0.3 (0.2 to 0.3)	0.1 (0.1 to 0.1)	0.2 (0.1 to 0.2)	0.5 (0.4 to 0.7)	0.1 (0.1 to 0.1)	23.5 (1.7 to 53.2)	102.8 (57 to 172.1)	-9.3 (-31.6 to 17.7)
North Africa and Middle East	North Africa and Middle East	Qatar	Prevalence	All Ages	1 (1 to 1)	0 (0 to 0)	1 (0 to 1)	8 (6 to 11)	4 (3 to 6)	4 (3 to 6)	832.2 (678.3 to 1053.9)	1079.3 (753.1 to 1606.3)	677.1 (524.5 to 841.2)
North Africa and Middle East	North Africa and Middle East	Qatar	Prevalence	Age-standardized	0.4 (0.4 to 0.5)	0.4 (0.3 to 0.5)	0.5 (0.4 to 0.6)	0.6 (0.5 to 0.8)	0.8 (0.6 to 1)	0.6 (0.4 to 0.7)	42.4 (22.6 to 66.6)	91.8 (57.2 to 138.5)	18.7 (-2.4 to 44.9)
North Africa and Middle East	North Africa and Middle East	Qatar	Deaths	All Ages	0 (0 to 0)	0 (0 to 0)	0 (0 to 0)	0 (0 to 0)	0 (0 to 0)	0 (0 to 0)	210.2 (84 to 402.8)	144.7 (29.6 to 363.2)	240 (79.5 to 530.3)
North Africa and Middle East	North Africa and Middle East	Qatar	Deaths	Age-standardized	0 (0 to 0)	0 (0 to 0)	0 (0 to 0.1)	0 (0 to 0)	0 (0 to 0.1)	0 (0 to 0)	-40.8 (-64.8 to -2.5)	8.4 (-45.1 to 114.4)	-48.3 (-72.3 to -3.1)
North Africa and Middle East	North Africa and Middle East	Qatar	DALYs	All Ages	1 (0 to 1)	0 (0 to 0)	0 (0 to 1)	3 (2 to 4)	1 (1 to 1)	2 (1 to 3)	357.4 (211.9 to 550.2)	471.5 (276.8 to 763.5)	313.6 (151.8 to 543.9)
North Africa and Middle East	North Africa and Middle East	Qatar	DALYs	Age-standardized	0.6 (0.4 to 0.8)	0.5 (0.3 to 0.7)	0.7 (0.4 to 1.1)	0.4 (0.3 to 0.5)	0.5 (0.3 to 0.7)	0.3 (0.2 to 0.5)	-37.3 (-58.6 to -6)	9.9 (-34.8 to 85.2)	-47.7 (-68.5 to -11.7)
North Africa and Middle East	North Africa and Middle East	Qatar	YLLs	All Ages	0 (0 to 1)	0 (0 to 0)	0 (0 to 1)	1 (1 to 2)	0 (0 to 1)	1 (1 to 2)	215.4 (83 to 416.2)	186.4 (58.2 to 423.7)	224.9 (71.5 to 496.8)
North Africa and Middle East	North Africa and Middle East	Qatar	YLLs	Age-standardized	0.5 (0.4 to 0.8)	0.4 (0.2 to 0.6)	0.6 (0.4 to 1)	0.3 (0.2 to 0.4)	0.4 (0.2 to 0.6)	0.3 (0.2 to 0.4)	-47.8 (-68.7 to -15.8)	-4.5 (-49.5 to 84.4)	-56.4 (-75.9 to -18.6)
North Africa and Middle East	North Africa and Middle East	Qatar	YLDs	All Ages	0 (0 to 0)	0 (0 to 0)	0 (0 to 0)	1 (1 to 2)	1 (0 to 1)	1 (0 to 1)	831.3 (678.5 to 1050.9)	1078 (752.8 to 1601.6)	677 (526.2 to 840.4)
North Africa and Middle East	North Africa and Middle East	Qatar	YLDs	Age-standardized	0.1 (0 to 0.1)	0.1 (0 to 0.1)	0.1 (0 to 0.1)	0.1 (0.1 to 0.1)	0.1 (0.1 to 0.2)	0.1 (0.1 to 0.1)	41.9 (21.9 to 65.4)	91 (56.7 to 137)	18.5 (-2.4 to 44.4)
North Africa and Middle East	North Africa and Middle East	Saudi Arabia	Incidence	All Ages	11 (7 to 16)	7 (4 to 10)	4 (2 to 7)	60 (42 to 83)	47 (31 to 68)	13 (8 to 19)	460.4 (308.4 to 714.2)	611.5 (370.5 to 1024.9)	212.6 (133.4 to 342.3)
North Africa and Middle East	North Africa and Middle East	Saudi Arabia	Incidence	Age-standardized	0.1 (0.1 to 0.2)	0.2 (0.1 to 0.2)	0.1 (0.1 to 0.1)	0.2 (0.2 to 0.3)	0.4 (0.3 to 0.5)	0.1 (0.1 to 0.1)	83.9 (46.3 to 131.7)	129.4 (67.2 to 214.5)	24.6 (0.7 to 60.6)
North Africa and Middle East	North Africa and Middle East	Saudi Arabia	Prevalence	All Ages	29 (22 to 36)	11 (8 to 15)	17 (14 to 22)	130 (102 to 164)	71 (50 to 97)	59 (46 to 76)	355.5 (280.8 to 460.2)	535.7 (367.1 to 805.7)	240.3 (190.6 to 299)
North Africa and Middle East	North Africa and Middle East	Saudi Arabia	Prevalence	Age-standardized	0.4 (0.3 to 0.5)	0.3 (0.2 to 0.4)	0.5 (0.4 to 0.6)	0.7 (0.6 to 0.8)	0.6 (0.5 to 0.8)	0.7 (0.6 to 0.9)	63.7 (40.6 to 90.8)	96.1 (57.4 to 145)	45.9 (19.1 to 78.6)
North Africa and Middle East	North Africa and Middle East	Saudi Arabia	Deaths	All Ages	1 (1 to 2)	0 (0 to 1)	1 (0 to 2)	3 (2 to 4)	1 (1 to 2)	2 (1 to 3)	130.5 (17.1 to 394.7)	164.8 (35.7 to 453.1)	116 (-4.2 to 557)
North Africa and Middle East	North Africa and Middle East	Saudi Arabia	Deaths	Age-standardized	0 (0 to 0)	0 (0 to 0)	0 (0 to 0.1)	0 (0 to 0)	0 (0 to 0)	0 (0 to 0)	-15.8 (-57.1 to 75.9)	-5.4 (-51.8 to 92.4)	-23.3 (-65.1 to 125.3)
North Africa and Middle East	North Africa and Middle East	Saudi Arabia	DALYs	All Ages	34 (21 to 52)	12 (7 to 19)	22 (11 to 38)	97 (72 to 129)	41 (28 to 59)	56 (40 to 79)	189.2 (61.7 to 451.5)	245.6 (88.8 to 522.1)	158.5 (27.6 to 548.9)
North Africa and Middle East	North Africa and Middle East	Saudi Arabia	DALYs	Age-standardized	0.6 (0.3 to 0.9)	0.4 (0.2 to 0.7)	0.7 (0.4 to 1.2)	0.5 (0.4 to 0.7)	0.4 (0.3 to 0.6)	0.6 (0.4 to 0.8)	-8.3 (-48.5 to 73.8)	3.9 (-42.6 to 94.7)	-16.4 (-58.7 to 107.8)
North Africa and Middle East	North Africa and Middle East	Saudi Arabia	YLLs	All Ages	29 (17 to 48)	10 (6 to 17)	19 (8 to 35)	77 (54 to 109)	30 (18 to 48)	47 (31 to 71)	163.6 (33.8 to 471.5)	195.4 (45.1 to 525.1)	146.8 (6.9 to 659.4)
North Africa and Middle East	North Africa and Middle East	Saudi Arabia	YLLs	Age-standardized	0.5 (0.3 to 0.8)	0.4 (0.2 to 0.6)	0.6 (0.3 to 1.1)	0.4 (0.3 to 0.6)	0.3 (0.2 to 0.5)	0.5 (0.3 to 0.7)	-17.6 (-57.5 to 75.8)	-9.2 (-56.5 to 92.4)	-24.1 (-66.3 to 128.4)
North Africa and Middle East	North Africa and Middle East	Saudi Arabia	YLDs	All Ages	5 (3 to 7)	2 (1 to 3)	3 (2 to 4)	21 (13 to 30)	11 (6 to 18)	9 (6 to 14)	354.2 (280.5 to 458.3)	532.8 (366.7 to 800)	239.7 (190.6 to 298.6)
North Africa and Middle East	North Africa and Middle East	Saudi Arabia	YLDs	Age-standardized	0.1 (0 to 0.1)	0.1 (0 to 0.1)	0.1 (0.1 to 0.1)	0.1 (0.1 to 0.2)	0.1 (0.1 to 0.1)	0.1 (0.1 to 0.2)	63 (40.4 to 90.1)	95 (56.9 to 143.3)	45.3 (19.1 to 77.8)
North Africa and Middle East	North Africa and Middle East	Sudan	Incidence	All Ages	17 (11 to 24)	12 (8 to 18)	5 (3 to 8)	75 (54 to 101)	64 (44 to 88)	11 (7 to 16)	344.6 (218.9 to 529.4)	438.8 (261.8 to 731.4)	122.3 (69.9 to 202.3)
North Africa and Middle East	North Africa and Middle East	Sudan	Incidence	Age-standardized	0.1 (0.1 to 0.2)	0.2 (0.1 to 0.3)	0.1 (0.1 to 0.1)	0.3 (0.2 to 0.3)	0.5 (0.3 to 0.6)	0.1 (0.1 to 0.1)	95.5 (51.3 to 158)	143.9 (74.6 to 242.4)	9 (-12.3 to 39.6)
North Africa and Middle East	North Africa and Middle East	Sudan	Prevalence	All Ages	43 (33 to 55)	19 (14 to 27)	23 (18 to 30)	156 (123 to 194)	95 (67 to 126)	62 (49 to 78)	266.7 (202.8 to 351.1)	386.2 (254.8 to 569.6)	166.4 (125.6 to 218.1)
North Africa and Middle East	North Africa and Middle East	Sudan	Prevalence	Age-standardized	0.4 (0.3 to 0.5)	0.3 (0.3 to 0.4)	0.5 (0.4 to 0.6)	0.7 (0.6 to 0.9)	0.7 (0.6 to 0.9)	0.7 (0.5 to 0.9)	72.7 (48.1 to 104.1)	116 (70.7 to 174.2)	40.9 (17.2 to 69)
North Africa and Middle East	North Africa and Middle East	Sudan	Deaths	All Ages	8 (3 to 14)	1 (0 to 1)	7 (2 to 13)	15 (8 to 25)	3 (2 to 5)	12 (5 to 23)	90.1 (13 to 284)	262.4 (118.5 to 523.2)	69.8 (-3 to 278.2)
North Africa and Middle East	North Africa and Middle East	Sudan	Deaths	Age-standardized	0.1 (0 to 0.2)	0 (0 to 0)	0.2 (0 to 0.3)	0.1 (0 to 0.1)	0 (0 to 0.1)	0.1 (0 to 0.2)	-7.9 (-44 to 80.7)	85.6 (14 to 222.1)	-21.5 (-55.1 to 73.3)
North Africa and Middle East	North Africa and Middle East	Sudan	DALYs	All Ages	213 (80 to 361)	29 (16 to 47)	184 (45 to 335)	450 (235 to 776)	104 (63 to 171)	346 (142 to 664)	111.2 (29.5 to 291.4)	260.4 (117.1 to 529.4)	87.9 (7.9 to 301.3)
North Africa and Middle East	North Africa and Middle East	Sudan	DALYs	Age-standardized	2.1 (0.8 to 3.6)	0.5 (0.3 to 0.8)	3.5 (0.9 to 6.4)	2 (1 to 3.4)	0.9 (0.5 to 1.5)	2.9 (1.2 to 5.6)	-4.8 (-42.4 to 79.6)	75.7 (12.5 to 185.7)	-16.7 (-51.8 to 75.9)
North Africa and Middle East	North Africa and Middle East	Sudan	YLLs	All Ages	206 (73 to 354)	26 (13 to 44)	181 (42 to 331)	425 (209 to 752)	89 (48 to 155)	336 (132 to 655)	106.2 (23.8 to 296)	245.7 (93.3 to 551.7)	86.4 (5.6 to 309.7)
North Africa and Middle East	North Africa and Middle East	Sudan	YLLs	Age-standardized	2 (0.7 to 3.5)	0.4 (0.2 to 0.8)	3.4 (0.8 to 6.3)	1.9 (0.9 to 3.3)	0.8 (0.4 to 1.3)	2.8 (1.1 to 5.5)	-7.3 (-45.3 to 80.8)	71 (3 to 201.6)	-18 (-53.4 to 79.9)
North Africa and Middle East	North Africa and Middle East	Sudan	YLDs	All Ages	7 (4 to 10)	3 (2 to 5)	4 (2 to 6)	25 (16 to 36)	15 (9 to 23)	10 (6 to 15)	263.6 (201.2 to 345.9)	383.6 (254.1 to 565.7)	164.7 (125.2 to 213.4)
North Africa and Middle East	North Africa and Middle East	Sudan	YLDs	Age-standardized	0.1 (0 to 0.1)	0.1 (0 to 0.1)	0.1 (0.1 to 0.1)	0.1 (0.1 to 0.2)	0.1 (0.1 to 0.2)	0.1 (0.1 to 0.2)	71.1 (47.3 to 101.2)	114.6 (70.1 to 172.4)	39.6 (16.9 to 66.8)
North Africa and Middle East	North Africa and Middle East	Syrian Arab Republic	Incidence	All Ages	21 (14 to 29)	17 (11 to 24)	4 (3 to 6)	43 (32 to 56)	38 (27 to 50)	5 (4 to 7)	105.7 (62.3 to 158.8)	127 (73.8 to 196.7)	20 (-16 to 73.3)
North Africa and Middle East	North Africa and Middle East	Syrian Arab Republic	Incidence	Age-standardized	0.3 (0.2 to 0.3)	0.4 (0.3 to 0.6)	0.1 (0.1 to 0.2)	0.3 (0.2 to 0.4)	0.6 (0.4 to 0.7)	0.1 (0.1 to 0.1)	18.3 (-1.6 to 40.5)	28 (5.1 to 54.9)	-31 (-50.1 to -7.2)
North Africa and Middle East	North Africa and Middle East	Syrian Arab Republic	Prevalence	All Ages	39 (30 to 49)	24 (17 to 33)	15 (12 to 18)	87 (70 to 106)	56 (42 to 72)	31 (24 to 40)	123.2 (88.6 to 161.7)	128.6 (81 to 183.1)	114.2 (76.5 to 157.1)
North Africa and Middle East	North Africa and Middle East	Syrian Arab Republic	Prevalence	Age-standardized	0.6 (0.5 to 0.7)	0.7 (0.5 to 0.9)	0.5 (0.4 to 0.7)	0.7 (0.6 to 0.9)	0.9 (0.7 to 1.1)	0.6 (0.5 to 0.7)	23.6 (8.9 to 39.8)	26.7 (8.3 to 49.4)	12 (-7.1 to 34.2)
North Africa and Middle East	North Africa and Middle East	Syrian Arab Republic	Deaths	All Ages	1 (0 to 1)	0 (0 to 0)	0 (0 to 1)	1 (1 to 1)	0 (0 to 0)	1 (1 to 1)	82.1 (-1.8 to 287.5)	91.9 (7.7 to 289.2)	80.8 (-7.7 to 307.3)
North Africa and Middle East	North Africa and Middle East	Syrian Arab Republic	Deaths	Age-standardized	0 (0 to 0)	0 (0 to 0)	0 (0 to 0)	0 (0 to 0)	0 (0 to 0)	0 (0 to 0)	-13.8 (-53.1 to 76.5)	11 (-39.5 to 130.3)	-18.9 (-58.1 to 79.8)
North Africa and Middle East	North Africa and Middle East	Syrian Arab Republic	DALYs	All Ages	18 (12 to 24)	6 (4 to 8)	12 (8 to 18)	35 (26 to 47)	12 (8 to 16)	23 (16 to 34)	92.5 (25.6 to 211.6)	109 (62.7 to 174.5)	85.1 (5.3 to 260.7)
North Africa and Middle East	North Africa and Middle East	Syrian Arab Republic	DALYs	Age-standardized	0.3 (0.2 to 0.4)	0.2 (0.1 to 0.2)	0.5 (0.3 to 0.7)	0.3 (0.2 to 0.4)	0.2 (0.1 to 0.3)	0.4 (0.3 to 0.6)	-7.8 (-40.3 to 48.3)	16.5 (-6.9 to 47.7)	-16.3 (-51.3 to 60.3)
North Africa and Middle East	North Africa and Middle East	Syrian Arab Republic	YLLs	All Ages	12 (7 to 18)	2 (1 to 3)	10 (5 to 16)	21 (13 to 32)	3 (2 to 5)	18 (11 to 29)	76.7 (-5.5 to 270.3)	66 (-13.1 to 266.5)	78.6 (-9.4 to 316.3)
North Africa and Middle East	North Africa and Middle East	Syrian Arab Republic	YLLs	Age-standardized	0.2 (0.1 to 0.3)	0.1 (0 to 0.1)	0.4 (0.2 to 0.6)	0.2 (0.1 to 0.3)	0.1 (0 to 0.1)	0.3 (0.2 to 0.5)	-20.9 (-57.7 to 67.7)	-3.5 (-45.3 to 101.4)	-22.4 (-60.1 to 79.1)
North Africa and Middle East	North Africa and Middle East	Syrian Arab Republic	YLDs	All Ages	6 (4 to 9)	4 (2 to 6)	2 (1 to 3)	14 (9 to 20)	9 (5 to 13)	5 (3 to 8)	123 (88.7 to 161.6)	128.5 (81 to 183.1)	114 (76.7 to 156.7)
North Africa and Middle East	North Africa and Middle East	Syrian Arab Republic	YLDs	Age-standardized	0.1 (0.1 to 0.1)	0.1 (0.1 to 0.2)	0.1 (0.1 to 0.1)	0.1 (0.1 to 0.2)	0.1 (0.1 to 0.2)	0.1 (0.1 to 0.1)	23.4 (8.9 to 39.7)	26.6 (8.2 to 49.4)	11.8 (-7.1 to 34.1)
North Africa and Middle East	North Africa and Middle East	Tunisia	Incidence	All Ages	7 (4 to 9)	4 (3 to 6)	2 (1 to 3)	30 (22 to 39)	24 (17 to 32)	6 (5 to 8)	364.9 (231 to 548.9)	469.3 (286.5 to 767.4)	170.1 (111.1 to 272)
North Africa and Middle East	North Africa and Middle East	Tunisia	Incidence	Age-standardized	0.1 (0.1 to 0.1)	0.1 (0.1 to 0.2)	0.1 (0.1 to 0.1)	0.2 (0.2 to 0.3)	0.4 (0.3 to 0.5)	0.1 (0.1 to 0.1)	124 (72.8 to 195.5)	169.3 (91.9 to 282.4)	36 (12.6 to 77.6)
North Africa and Middle East	North Africa and Middle East	Tunisia	Prevalence	All Ages	20 (16 to 25)	8 (5 to 10)	12 (9 to 16)	80 (67 to 97)	39 (30 to 50)	42 (33 to 53)	301.5 (235.4 to 389.7)	407.1 (276.5 to 567.5)	236.4 (173.2 to 326.5)
North Africa and Middle East	North Africa and Middle East	Tunisia	Prevalence	Age-standardized	0.4 (0.3 to 0.5)	0.3 (0.2 to 0.4)	0.5 (0.4 to 0.6)	0.7 (0.6 to 0.8)	0.6 (0.5 to 0.8)	0.8 (0.6 to 1)	73.3 (47.4 to 105.8)	120.5 (72.8 to 178.8)	54.9 (26.5 to 96.2)
North Africa and Middle East	North Africa and Middle East	Tunisia	Deaths	All Ages	1 (1 to 2)	0 (0 to 0)	1 (1 to 2)	4 (3 to 7)	1 (1 to 2)	3 (2 to 5)	232.5 (100.6 to 527.7)	473.3 (223.2 to 895.5)	181.7 (59.6 to 520.7)
North Africa and Middle East	North Africa and Middle East	Tunisia	Deaths	Age-standardized	0 (0 to 0)	0 (0 to 0)	0.1 (0 to 0.1)	0 (0 to 0.1)	0 (0 to 0)	0.1 (0 to 0.1)	24.3 (-23.7 to 132.7)	95.2 (8.7 to 235.4)	10.2 (-37.5 to 147.7)
North Africa and Middle East	North Africa and Middle East	Tunisia	DALYs	All Ages	35 (24 to 48)	8 (5 to 12)	27 (17 to 41)	109 (72 to 159)	33 (22 to 49)	76 (47 to 119)	212.7 (99.7 to 466.7)	336.2 (171 to 595.1)	178.7 (64.1 to 469)
North Africa and Middle East	North Africa and Middle East	Tunisia	DALYs	Age-standardized	0.7 (0.5 to 0.9)	0.3 (0.2 to 0.4)	1.1 (0.7 to 1.6)	0.9 (0.6 to 1.3)	0.5 (0.4 to 0.8)	1.3 (0.8 to 2)	31.7 (-15.8 to 133.9)	89.7 (16 to 200)	20.8 (-27.3 to 142.7)
North Africa and Middle East	North Africa and Middle East	Tunisia	YLLs	All Ages	32 (21 to 45)	6 (4 to 10)	25 (15 to 39)	96 (60 to 147)	27 (16 to 43)	69 (42 to 113)	203.9 (83.8 to 483.9)	323.1 (137.2 to 638.1)	174.3 (54.2 to 502.7)
North Africa and Middle East	North Africa and Middle East	Tunisia	YLLs	Age-standardized	0.6 (0.4 to 0.9)	0.2 (0.1 to 0.4)	1 (0.6 to 1.5)	0.8 (0.5 to 1.2)	0.4 (0.3 to 0.7)	1.1 (0.7 to 1.8)	27.6 (-22.7 to 142)	84.2 (4.2 to 216.2)	18 (-32.8 to 153.7)
North Africa and Middle East	North Africa and Middle East	Tunisia	YLDs	All Ages	3 (2 to 5)	1 (1 to 2)	2 (1 to 3)	13 (8 to 19)	6 (4 to 9)	7 (4 to 10)	299.5 (234.2 to 386.1)	403.9 (275.9 to 561.2)	235 (172.6 to 322.6)
North Africa and Middle East	North Africa and Middle East	Tunisia	YLDs	Age-standardized	0.1 (0 to 0.1)	0 (0 to 0.1)	0.1 (0.1 to 0.1)	0.1 (0.1 to 0.2)	0.1 (0.1 to 0.1)	0.1 (0.1 to 0.2)	72.1 (46.8 to 103.8)	118.5 (71.7 to 176)	54.1 (26.2 to 94.6)
North Africa and Middle East	North Africa and Middle East	Turkey	Incidence	All Ages	879 (645 to 1,158)	666 (476 to 885)	213 (151 to 291)	1,473 (1,123 to 1,851)	1,262 (950 to 1,619)	211 (161 to 268)	67.6 (48.7 to 90.4)	89.6 (65.6 to 116.1)	-1.2 (-24.7 to 30.3)
North Africa and Middle East	North Africa and Middle East	Turkey	Incidence	Age-standardized	1.8 (1.4 to 2.4)	2.7 (2 to 3.6)	1 (0.7 to 1.3)	1.6 (1.2 to 2)	2.7 (2.1 to 3.5)	0.5 (0.4 to 0.6)	-11.8 (-18.8 to -3.4)	1.6 (-6.8 to 10.6)	-49.6 (-59.7 to -36.3)
North Africa and Middle East	North Africa and Middle East	Turkey	Prevalence	All Ages	2,051 (1,469 to 2,760)	1,634 (1,131 to 2,272)	417 (322 to 526)	3,859 (3,022 to 4,822)	3,202 (2,434 to 4,134)	657 (536 to 836)	88.2 (63.4 to 118.7)	96 (66.5 to 130.7)	57.5 (26.4 to 98.9)
North Africa and Middle East	North Africa and Middle East	Turkey	Prevalence	Age-standardized	4.6 (3.4 to 6)	7 (5 to 9.5)	2.1 (1.7 to 2.7)	4.3 (3.4 to 5.4)	6.9 (5.3 to 8.9)	1.7 (1.4 to 2.1)	-5.9 (-15.1 to 5.2)	-1 (-12.1 to 12.8)	-21.7 (-35.2 to -4.8)
North Africa and Middle East	North Africa and Middle East	Turkey	Deaths	All Ages	78 (52 to 107)	4 (2 to 6)	74 (49 to 103)	61 (45 to 81)	6 (4 to 10)	55 (40 to 73)	-22.3 (-50.3 to 28.2)	67.5 (-6.3 to 191.6)	-26.8 (-54.1 to 24.8)
North Africa and Middle East	North Africa and Middle East	Turkey	Deaths	Age-standardized	0.2 (0.1 to 0.3)	0 (0 to 0)	0.4 (0.2 to 0.5)	0.1 (0.1 to 0.1)	0 (0 to 0)	0.1 (0.1 to 0.2)	-64.5 (-77.5 to -40.2)	-42.3 (-67.5 to 1.6)	-65.1 (-78.5 to -38.4)
North Africa and Middle East	North Africa and Middle East	Turkey	DALYs	All Ages	3,085 (2,117 to 4,307)	311 (197 to 474)	2,773 (1,807 to 3,998)	2,132 (1,659 to 2,699)	573 (375 to 833)	1,559 (1,156 to 2,044)	-30.9 (-54.1 to 5.9)	84.1 (53.7 to 119)	-43.8 (-64.7 to -6.8)
North Africa and Middle East	North Africa and Middle East	Turkey	DALYs	Age-standardized	6.6 (4.6 to 9.1)	1.4 (0.9 to 2.1)	12 (8 to 16.8)	2.4 (1.9 to 3)	1.2 (0.8 to 1.8)	3.6 (2.7 to 4.7)	-64.1 (-75.3 to -45.6)	-13 (-27.5 to 1.2)	-69.8 (-80.6 to -50.3)
North Africa and Middle East	North Africa and Middle East	Turkey	YLLs	All Ages	2,761 (1,803 to 4,011)	54 (33 to 87)	2,707 (1,734 to 3,944)	1,533 (1,128 to 2,037)	78 (49 to 126)	1,454 (1,059 to 1,943)	-44.5 (-65.5 to -7.2)	44.1 (-16.4 to 144.1)	-46.3 (-66.7 to -9.9)
North Africa and Middle East	North Africa and Middle East	Turkey	YLLs	Age-standardized	5.9 (4 to 8.3)	0.3 (0.2 to 0.5)	11.7 (7.7 to 16.4)	1.7 (1.3 to 2.3)	0.2 (0.1 to 0.3)	3.4 (2.5 to 4.5)	-71 (-81.6 to -52.5)	-47.1 (-69.5 to -9.7)	-71.2 (-82 to -51.4)
North Africa and Middle East	North Africa and Middle East	Turkey	YLDs	All Ages	323 (192 to 510)	257 (147 to 414)	66 (42 to 100)	599 (371 to 881)	495 (301 to 743)	104 (67 to 154)	85.3 (60.5 to 117.5)	92.5 (62.3 to 131)	57.5 (26.8 to 98.5)
North Africa and Middle East	North Africa and Middle East	Turkey	YLDs	Age-standardized	0.7 (0.4 to 1.1)	1.1 (0.7 to 1.8)	0.3 (0.2 to 0.5)	0.7 (0.4 to 1)	1.1 (0.7 to 1.6)	0.3 (0.2 to 0.4)	-7.5 (-18.4 to 4.6)	-2.8 (-15.1 to 11.9)	-21.9 (-35.1 to -5.4)
North Africa and Middle East	North Africa and Middle East	United Arab Emirates	Incidence	All Ages	2 (1 to 2)	1 (0 to 1)	1 (0 to 1)	18 (12 to 26)	13 (8 to 19)	5 (3 to 8)	1058.4 (703.3 to 1725.5)	1437.9 (834 to 2508)	626.9 (376.4 to 1175.3)
North Africa and Middle East	North Africa and Middle East	United Arab Emirates	Incidence	Age-standardized	0.1 (0.1 to 0.2)	0.2 (0.2 to 0.3)	0.1 (0.1 to 0.1)	0.3 (0.2 to 0.3)	0.6 (0.4 to 0.8)	0.1 (0.1 to 0.2)	86.1 (52.3 to 132.2)	163.2 (97.9 to 249.4)	29.8 (4.1 to 72.7)
North Africa and Middle East	North Africa and Middle East	United Arab Emirates	Prevalence	All Ages	4 (3 to 5)	1 (1 to 2)	2 (2 to 3)	38 (29 to 48)	18 (13 to 26)	19 (14 to 26)	968.2 (755.8 to 1276.6)	1351.3 (875.2 to 2141.6)	754.1 (595.6 to 977.1)
North Africa and Middle East	North Africa and Middle East	United Arab Emirates	Prevalence	Age-standardized	0.5 (0.4 to 0.6)	0.4 (0.3 to 0.5)	0.6 (0.4 to 0.7)	0.9 (0.8 to 1.1)	1 (0.8 to 1.2)	0.9 (0.7 to 1.1)	85.8 (59.4 to 120.5)	137.6 (93.8 to 187.6)	60.7 (30.8 to 100.1)
North Africa and Middle East	North Africa and Middle East	United Arab Emirates	Deaths	All Ages	0 (0 to 1)	0 (0 to 0)	0 (0 to 1)	5 (3 to 13)	0 (0 to 1)	5 (2 to 12)	1136.8 (447.2 to 2600.6)	877 (398.9 to 2109.7)	1163.1 (413.1 to 2883.6)
North Africa and Middle East	North Africa and Middle East	United Arab Emirates	Deaths	Age-standardized	0.1 (0 to 0.2)	0 (0 to 0.1)	0.1 (0 to 0.2)	0.1 (0 to 0.2)	0 (0 to 0.1)	0.1 (0 to 0.3)	11.4 (-40.9 to 113.9)	35.2 (-26.4 to 170.9)	-3.4 (-53.6 to 110.5)
North Africa and Middle East	North Africa and Middle East	United Arab Emirates	DALYs	All Ages	17 (7 to 31)	2 (1 to 2)	15 (6 to 29)	215 (109 to 505)	18 (11 to 28)	197 (94 to 489)	1158.7 (446.1 to 2662.9)	999.1 (508.3 to 2066.6)	1175.3 (424.8 to 2907.2)
North Africa and Middle East	North Africa and Middle East	United Arab Emirates	DALYs	Age-standardized	2 (0.9 to 3.8)	0.7 (0.3 to 1.2)	2.7 (1 to 5.4)	2.5 (1.3 to 6)	1.1 (0.7 to 1.6)	2.9 (1.5 to 7.8)	23.4 (-35.7 to 140.2)	51.3 (-14.6 to 184.6)	9.5 (-48.1 to 133.3)
North Africa and Middle East	North Africa and Middle East	United Arab Emirates	YLLs	All Ages	17 (7 to 30)	1 (1 to 2)	15 (6 to 29)	209 (104 to 498)	15 (8 to 25)	194 (91 to 486)	1165.3 (433.6 to 2738.8)	950.5 (427.5 to 2382.1)	1185.3 (417.8 to 2997.9)
North Africa and Middle East	North Africa and Middle East	United Arab Emirates	YLLs	Age-standardized	1.9 (0.8 to 3.8)	0.6 (0.3 to 1.2)	2.6 (0.9 to 5.3)	2.3 (1.2 to 5.9)	0.9 (0.5 to 1.4)	2.8 (1.4 to 7.7)	20.9 (-39 to 142.3)	42.7 (-23.2 to 193.8)	7.8 (-50 to 136.2)
North Africa and Middle East	North Africa and Middle East	United Arab Emirates	YLDs	All Ages	1 (0 to 1)	0 (0 to 0)	0 (0 to 1)	6 (4 to 9)	3 (2 to 5)	3 (2 to 5)	965.1 (757 to 1265.4)	1341.8 (873 to 2124.4)	758.3 (604.1 to 979.4)
North Africa and Middle East	North Africa and Middle East	United Arab Emirates	YLDs	Age-standardized	0.1 (0.1 to 0.1)	0.1 (0 to 0.1)	0.1 (0.1 to 0.1)	0.1 (0.1 to 0.2)	0.2 (0.1 to 0.2)	0.1 (0.1 to 0.2)	84.1 (58.4 to 117.9)	135.3 (92 to 184.6)	59.3 (30.5 to 97.8)
North Africa and Middle East	North Africa and Middle East	Yemen	Incidence	All Ages	13 (9 to 18)	10 (7 to 15)	3 (2 to 5)	98 (70 to 130)	90 (62 to 119)	9 (6 to 13)	646 (450.8 to 906.7)	774.3 (514.9 to 1142.5)	199.4 (132.2 to 318.6)
North Africa and Middle East	North Africa and Middle East	Yemen	Incidence	Age-standardized	0.2 (0.1 to 0.2)	0.3 (0.2 to 0.4)	0.1 (0.1 to 0.1)	0.5 (0.4 to 0.6)	0.8 (0.6 to 1.1)	0.1 (0.1 to 0.2)	144.9 (95.8 to 205.4)	200.2 (128.2 to 294.7)	11.6 (-8.1 to 40.1)
North Africa and Middle East	North Africa and Middle East	Yemen	Prevalence	All Ages	27 (21 to 34)	16 (11 to 21)	11 (9 to 15)	176 (136 to 220)	134 (97 to 175)	42 (33 to 53)	549.3 (423.5 to 689.9)	754 (545.9 to 1027.3)	267 (207.4 to 343.2)
North Africa and Middle East	North Africa and Middle East	Yemen	Prevalence	Age-standardized	0.5 (0.4 to 0.6)	0.5 (0.4 to 0.6)	0.5 (0.4 to 0.6)	1 (0.8 to 1.2)	1.3 (1 to 1.7)	0.7 (0.6 to 0.9)	119.4 (86.2 to 157.4)	190.6 (132.4 to 259.3)	42.4 (17.7 to 72.4)
North Africa and Middle East	North Africa and Middle East	Yemen	Deaths	All Ages	3 (1 to 6)	0 (0 to 1)	3 (1 to 5)	11 (6 to 17)	3 (2 to 4)	8 (4 to 14)	213.1 (68.1 to 498.6)	443.8 (172 to 958.7)	176.9 (39.3 to 529.8)
North Africa and Middle East	North Africa and Middle East	Yemen	Deaths	Age-standardized	0.1 (0 to 0.1)	0 (0 to 0)	0.1 (0 to 0.3)	0.1 (0 to 0.1)	0 (0 to 0.1)	0.1 (0.1 to 0.2)	14 (-36.6 to 113.3)	102.8 (2.6 to 307.9)	-10.7 (-54.6 to 102.7)
North Africa and Middle East	North Africa and Middle East	Yemen	DALYs	All Ages	101 (49 to 173)	16 (9 to 27)	85 (28 to 154)	348 (218 to 528)	98 (64 to 149)	250 (127 to 414)	243.7 (94.2 to 542.8)	510.2 (229.1 to 973.5)	193.6 (52.7 to 551.4)
North Africa and Middle East	North Africa and Middle East	Yemen	DALYs	Age-standardized	1.8 (0.9 to 3.2)	0.5 (0.3 to 0.9)	3.3 (1 to 6)	2.1 (1.3 to 3.2)	1.1 (0.7 to 1.6)	3.2 (1.6 to 5.2)	16.5 (-35.4 to 114.8)	111.5 (12.8 to 264.9)	-3.6 (-49.9 to 114.2)
North Africa and Middle East	North Africa and Middle East	Yemen	YLLs	All Ages	97 (44 to 170)	14 (7 to 24)	83 (27 to 152)	320 (186 to 501)	77 (43 to 126)	243 (121 to 406)	230.4 (81 to 537.5)	466.1 (170.8 to 1046.8)	192 (50 to 566.6)
North Africa and Middle East	North Africa and Middle East	Yemen	YLLs	Age-standardized	1.7 (0.8 to 3.1)	0.4 (0.2 to 0.8)	3.2 (0.9 to 5.9)	2 (1.1 to 3)	0.9 (0.5 to 1.4)	3.1 (1.5 to 5.1)	12.2 (-39.4 to 114.1)	98.9 (-2.9 to 288.6)	-4.7 (-51.5 to 116.3)
North Africa and Middle East	North Africa and Middle East	Yemen	YLDs	All Ages	4 (3 to 6)	2 (1 to 4)	2 (1 to 3)	28 (18 to 41)	21 (13 to 32)	7 (4 to 10)	544.9 (421.4 to 682)	751.3 (544.4 to 1021.3)	264.8 (206.5 to 338.8)
North Africa and Middle East	North Africa and Middle East	Yemen	YLDs	Age-standardized	0.1 (0 to 0.1)	0.1 (0 to 0.1)	0.1 (0.1 to 0.1)	0.2 (0.1 to 0.2)	0.2 (0.1 to 0.3)	0.1 (0.1 to 0.2)	117.5 (85 to 154.3)	189.2 (131.6 to 256.6)	41.2 (17.3 to 71.1)
		South Asia	Incidence	All Ages	5,057 (4,308 to 5,897)	2,190 (1,767 to 2,694)	2,867 (2,391 to 3,402)	12,955 (10,894 to 15,150)	7,700 (6,112 to 9,447)	5,255 (4,446 to 6,194)	156.2 (135.4 to 179.3)	251.6 (211.4 to 299.9)	83.3 (70.7 to 95)
		South Asia	Incidence	Age-standardized	0.8 (0.7 to 0.9)	0.6 (0.5 to 0.8)	0.9 (0.8 to 1.1)	0.8 (0.7 to 1)	1 (0.8 to 1.2)	0.7 (0.6 to 0.9)	9.2 (1.2 to 18.2)	53.9 (38.8 to 72)	-19.5 (-23.6 to -16)
		South Asia	Prevalence	All Ages	39,831 (32,941 to 47,883)	15,120 (12,064 to 18,995)	24,711 (19,759 to 30,302)	88,996 (75,029 to 107,476)	38,531 (31,724 to 47,804)	50,465 (41,358 to 61,185)	123.4 (109.9 to 138.2)	154.8 (130.6 to 181.5)	104.2 (89.3 to 119.5)
		South Asia	Prevalence	Age-standardized	7 (5.8 to 8.4)	4.9 (3.9 to 6.1)	9 (7.3 to 10.9)	6.3 (5.3 to 7.6)	5.1 (4.3 to 6.4)	7.6 (6.2 to 9.1)	-9.9 (-15 to -4.8)	5.9 (-3.2 to 15.5)	-15.6 (-21.1 to -10.5)
		South Asia	Deaths	All Ages	2,598 (844 to 3,830)	166 (93 to 247)	2,432 (668 to 3,671)	3,389 (1,941 to 4,784)	387 (256 to 528)	3,002 (1,565 to 4,422)	30.4 (-2.2 to 151.4)	132.8 (67 to 228.1)	23.4 (-9.4 to 165.7)
		South Asia	Deaths	Age-standardized	0.5 (0.2 to 0.8)	0.1 (0 to 0.1)	1 (0.3 to 1.4)	0.3 (0.2 to 0.4)	0.1 (0 to 0.1)	0.5 (0.3 to 0.7)	-50.3 (-62.6 to -5.5)	-22.3 (-45.5 to 11.3)	-49.2 (-62.6 to 11.9)
		South Asia	DALYs	All Ages	70,482 (26,453 to 101,897)	6,196 (4,345 to 8,339)	64,286 (20,200 to 96,290)	88,706 (55,603 to 122,516)	13,492 (10,065 to 17,357)	75,214 (42,209 to 108,612)	25.9 (-4.6 to 124.3)	117.8 (77.2 to 166.8)	17 (-13.4 to 131.7)
		South Asia	DALYs	Age-standardized	12.4 (4.7 to 17.7)	2.3 (1.5 to 3.1)	21.6 (6.9 to 32)	6.3 (4 to 8.7)	1.9 (1.4 to 2.5)	10.9 (6.2 to 15.6)	-48.9 (-61.2 to -9.2)	-16 (-32.9 to 4.4)	-49.3 (-62.5 to 0.6)
		South Asia	YLLs	All Ages	64,498 (20,569 to 95,884)	3,820 (2,261 to 5,581)	60,677 (16,680 to 92,779)	75,115 (41,734 to 108,985)	7,436 (4,972 to 10,257)	67,679 (34,653 to 101,337)	16.5 (-13.8 to 122.8)	94.6 (40.5 to 171.4)	11.5 (-18.7 to 135.5)
		South Asia	YLLs	Age-standardized	11.3 (3.7 to 16.8)	1.5 (0.8 to 2.2)	20.3 (5.6 to 30.6)	5.4 (3 to 7.7)	1.1 (0.7 to 1.5)	9.8 (5.1 to 14.6)	-52.7 (-64.6 to -10.1)	-27.2 (-48 to 2.8)	-51.6 (-64.6 to 3.5)
		South Asia	YLDs	All Ages	5,984 (3,895 to 8,590)	2,375 (1,516 to 3,556)	3,609 (2,322 to 5,222)	13,591 (8,859 to 19,128)	6,056 (3,953 to 8,986)	7,535 (4,893 to 10,938)	127.1 (111.3 to 143.7)	154.9 (130.7 to 181.6)	108.8 (90 to 127.9)
		South Asia	YLDs	Age-standardized	1 (0.7 to 1.5)	0.8 (0.5 to 1.1)	1.3 (0.8 to 1.9)	1 (0.6 to 1.4)	0.8 (0.5 to 1.2)	1.1 (0.7 to 1.6)	-7.7 (-13.8 to -1.5)	6 (-3.1 to 15.6)	-13.1 (-20.3 to -6.1)
		South Asia	Incidence	All Ages	5,057 (4,308 to 5,897)	2,190 (1,767 to 2,694)	2,867 (2,391 to 3,402)	12,955 (10,894 to 15,150)	7,700 (6,112 to 9,447)	5,255 (4,446 to 6,194)	156.2 (135.4 to 179.3)	251.6 (211.4 to 299.9)	83.3 (70.7 to 95)
		South Asia	Incidence	Age-standardized	0.8 (0.7 to 0.9)	0.6 (0.5 to 0.8)	0.9 (0.8 to 1.1)	0.8 (0.7 to 1)	1 (0.8 to 1.2)	0.7 (0.6 to 0.9)	9.2 (1.2 to 18.2)	53.9 (38.8 to 72)	-19.5 (-23.6 to -16)
		South Asia	Prevalence	All Ages	39,831 (32,941 to 47,883)	15,120 (12,064 to 18,995)	24,711 (19,759 to 30,302)	88,996 (75,029 to 107,476)	38,531 (31,724 to 47,804)	50,465 (41,358 to 61,185)	123.4 (109.9 to 138.2)	154.8 (130.6 to 181.5)	104.2 (89.3 to 119.5)
		South Asia	Prevalence	Age-standardized	7 (5.8 to 8.4)	4.9 (3.9 to 6.1)	9 (7.3 to 10.9)	6.3 (5.3 to 7.6)	5.1 (4.3 to 6.4)	7.6 (6.2 to 9.1)	-9.9 (-15 to -4.8)	5.9 (-3.2 to 15.5)	-15.6 (-21.1 to -10.5)
		South Asia	Deaths	All Ages	2,598 (844 to 3,830)	166 (93 to 247)	2,432 (668 to 3,671)	3,389 (1,941 to 4,784)	387 (256 to 528)	3,002 (1,565 to 4,422)	30.4 (-2.2 to 151.4)	132.8 (67 to 228.1)	23.4 (-9.4 to 165.7)
		South Asia	Deaths	Age-standardized	0.5 (0.2 to 0.8)	0.1 (0 to 0.1)	1 (0.3 to 1.4)	0.3 (0.2 to 0.4)	0.1 (0 to 0.1)	0.5 (0.3 to 0.7)	-50.3 (-62.6 to -5.5)	-22.3 (-45.5 to 11.3)	-49.2 (-62.6 to 11.9)
		South Asia	DALYs	All Ages	70,482 (26,453 to 101,897)	6,196 (4,345 to 8,339)	64,286 (20,200 to 96,290)	88,706 (55,603 to 122,516)	13,492 (10,065 to 17,357)	75,214 (42,209 to 108,612)	25.9 (-4.6 to 124.3)	117.8 (77.2 to 166.8)	17 (-13.4 to 131.7)
		South Asia	DALYs	Age-standardized	12.4 (4.7 to 17.7)	2.3 (1.5 to 3.1)	21.6 (6.9 to 32)	6.3 (4 to 8.7)	1.9 (1.4 to 2.5)	10.9 (6.2 to 15.6)	-48.9 (-61.2 to -9.2)	-16 (-32.9 to 4.4)	-49.3 (-62.5 to 0.6)
		South Asia	YLLs	All Ages	64,498 (20,569 to 95,884)	3,820 (2,261 to 5,581)	60,677 (16,680 to 92,779)	75,115 (41,734 to 108,985)	7,436 (4,972 to 10,257)	67,679 (34,653 to 101,337)	16.5 (-13.8 to 122.8)	94.6 (40.5 to 171.4)	11.5 (-18.7 to 135.5)
		South Asia	YLLs	Age-standardized	11.3 (3.7 to 16.8)	1.5 (0.8 to 2.2)	20.3 (5.6 to 30.6)	5.4 (3 to 7.7)	1.1 (0.7 to 1.5)	9.8 (5.1 to 14.6)	-52.7 (-64.6 to -10.1)	-27.2 (-48 to 2.8)	-51.6 (-64.6 to 3.5)
		South Asia	YLDs	All Ages	5,984 (3,895 to 8,590)	2,375 (1,516 to 3,556)	3,609 (2,322 to 5,222)	13,591 (8,859 to 19,128)	6,056 (3,953 to 8,986)	7,535 (4,893 to 10,938)	127.1 (111.3 to 143.7)	154.9 (130.7 to 181.6)	108.8 (90 to 127.9)
		South Asia	YLDs	Age-standardized	1 (0.7 to 1.5)	0.8 (0.5 to 1.1)	1.3 (0.8 to 1.9)	1 (0.6 to 1.4)	0.8 (0.5 to 1.2)	1.1 (0.7 to 1.6)	-7.7 (-13.8 to -1.5)	6 (-3.1 to 15.6)	-13.1 (-20.3 to -6.1)
South Asia	South Asia	Bangladesh	Incidence	All Ages	283 (231 to 340)	107 (73 to 149)	176 (146 to 209)	580 (474 to 697)	128 (87 to 179)	452 (361 to 551)	105 (72.1 to 141.1)	19.9 (-16.9 to 81)	156.7 (118.2 to 200.1)
South Asia	South Asia	Bangladesh	Incidence	Age-standardized	0.5 (0.4 to 0.6)	0.4 (0.3 to 0.5)	0.6 (0.5 to 0.8)	0.4 (0.4 to 0.5)	0.2 (0.1 to 0.3)	0.6 (0.5 to 0.8)	-18 (-28.6 to -7.3)	-48.3 (-61.9 to -29.9)	0.1 (-13 to 14)
South Asia	South Asia	Bangladesh	Prevalence	All Ages	1,612 (1,330 to 1,954)	210 (162 to 263)	1,402 (1,131 to 1,718)	5,475 (4,381 to 6,748)	428 (336 to 542)	5,047 (3,969 to 6,298)	239.6 (198.9 to 286.4)	104.1 (59.9 to 154.7)	259.9 (213.8 to 310.2)
South Asia	South Asia	Bangladesh	Prevalence	Age-standardized	3.5 (2.9 to 4.3)	0.8 (0.7 to 1.1)	5.6 (4.6 to 6.9)	4.4 (3.5 to 5.4)	0.7 (0.6 to 0.9)	7.7 (6 to 9.6)	24.9 (10.2 to 40.4)	-15.5 (-30.8 to 0.5)	36.3 (19.5 to 54.3)
South Asia	South Asia	Bangladesh	Deaths	All Ages	253 (63 to 408)	10 (5 to 18)	243 (53 to 398)	185 (61 to 307)	16 (8 to 32)	169 (48 to 288)	-26.8 (-55.8 to 37)	55.6 (-16.6 to 191.4)	-30.3 (-58.6 to 33.8)
South Asia	South Asia	Bangladesh	Deaths	Age-standardized	0.6 (0.1 to 0.9)	0.1 (0 to 0.1)	1 (0.2 to 1.6)	0.2 (0.1 to 0.3)	0 (0 to 0.1)	0.3 (0.1 to 0.4)	-73.7 (-83.8 to -50.6)	-47.1 (-71.9 to -3.6)	-73.7 (-84.3 to -49.4)
South Asia	South Asia	Bangladesh	DALYs	All Ages	6,586 (1,873 to 10,886)	287 (168 to 461)	6,299 (1,586 to 10,526)	4,848 (2,121 to 7,712)	392 (228 to 699)	4,456 (1,794 to 7,192)	-26.4 (-55.5 to 35.9)	36.5 (-19.6 to 136.6)	-29.3 (-57.3 to 35.3)
South Asia	South Asia	Bangladesh	DALYs	Age-standardized	13.8 (3.8 to 22.5)	1.3 (0.7 to 2.1)	24 (6 to 39.6)	3.7 (1.7 to 5.8)	0.6 (0.4 to 1.1)	6.6 (2.7 to 10.6)	-72.9 (-83.5 to -49.5)	-49.1 (-69.8 to -14.3)	-72.6 (-83.4 to -47.6)
South Asia	South Asia	Bangladesh	YLLs	All Ages	6,341 (1,603 to 10,620)	254 (137 to 427)	6,087 (1,343 to 10,345)	4,029 (1,312 to 6,861)	325 (162 to 636)	3,705 (1,034 to 6,463)	-36.5 (-62.3 to 19)	27.7 (-33 to 178.6)	-39.1 (-64.5 to 18.4)
South Asia	South Asia	Bangladesh	YLLs	Age-standardized	13.3 (3.3 to 21.8)	1.1 (0.6 to 2)	23.2 (5.1 to 38.8)	3.1 (1 to 5.2)	0.5 (0.3 to 1)	5.4 (1.5 to 9.4)	-76.8 (-86.3 to -55.9)	-53.1 (-74.7 to -13.6)	-76.5 (-86.3 to -54.4)
South Asia	South Asia	Bangladesh	YLDs	All Ages	245 (154 to 364)	33 (21 to 49)	212 (131 to 319)	818 (516 to 1,212)	68 (44 to 104)	751 (467 to 1,115)	233.5 (161.2 to 333.3)	104.4 (60.4 to 155)	253.6 (169.4 to 372.1)
South Asia	South Asia	Bangladesh	YLDs	Age-standardized	0.5 (0.3 to 0.8)	0.1 (0.1 to 0.2)	0.9 (0.5 to 1.3)	0.7 (0.4 to 1)	0.1 (0.1 to 0.2)	1.1 (0.7 to 1.7)	22.7 (-4.4 to 60.2)	-15.4 (-30.7 to 0.5)	33.9 (1.6 to 79.4)
South Asia	South Asia	Bhutan	Incidence	All Ages	1 (1 to 2)	0 (0 to 1)	1 (1 to 1)	3 (2 to 3)	1 (1 to 1)	2 (2 to 2)	110.8 (86.3 to 138)	81.1 (46.5 to 128.5)	128.5 (98.8 to 160.3)
South Asia	South Asia	Bhutan	Incidence	Age-standardized	0.5 (0.4 to 0.5)	0.3 (0.2 to 0.4)	0.6 (0.5 to 0.8)	0.5 (0.4 to 0.5)	0.3 (0.2 to 0.4)	0.6 (0.5 to 0.8)	0.2 (-9 to 9.8)	-4.8 (-19.4 to 16.8)	-1.3 (-11.1 to 9.3)
South Asia	South Asia	Bhutan	Prevalence	All Ages	6 (5 to 8)	1 (1 to 1)	5 (4 to 7)	21 (17 to 26)	2 (2 to 3)	19 (15 to 23)	226.4 (187.1 to 266.7)	129.2 (97.8 to 167.9)	245 (200.6 to 295.9)
South Asia	South Asia	Bhutan	Prevalence	Age-standardized	2.7 (2.2 to 3.3)	0.8 (0.6 to 0.9)	4.9 (3.9 to 6.2)	4 (3.2 to 4.8)	0.9 (0.7 to 1.1)	6.9 (5.6 to 8.5)	47.5 (31.9 to 65)	14.5 (-0.3 to 31.3)	41 (25.1 to 59.3)
South Asia	South Asia	Bhutan	Deaths	All Ages	1 (0 to 1)	0 (0 to 0)	1 (0 to 1)	1 (0 to 2)	0 (0 to 0)	1 (0 to 2)	35.6 (-19.8 to 175.2)	109 (14.1 to 340.2)	27.3 (-27.7 to 202.7)
South Asia	South Asia	Bhutan	Deaths	Age-standardized	0.4 (0.1 to 0.7)	0.1 (0 to 0.1)	0.7 (0.1 to 1.5)	0.2 (0.1 to 0.4)	0.1 (0 to 0.1)	0.4 (0.1 to 0.7)	-40.8 (-63.8 to 12.9)	-7.3 (-50.9 to 94.2)	-51 (-72.1 to 15.7)
South Asia	South Asia	Bhutan	DALYs	All Ages	21 (6 to 41)	2 (1 to 3)	19 (3 to 39)	24 (10 to 43)	3 (2 to 5)	21 (6 to 39)	12.4 (-32.1 to 128.9)	64.8 (-4.5 to 204.2)	6.8 (-37.7 to 148.6)
South Asia	South Asia	Bhutan	DALYs	Age-standardized	8.4 (2.2 to 15.8)	1.6 (0.9 to 2.7)	15.8 (2.8 to 31)	4.4 (1.8 to 7.9)	1.3 (0.8 to 2)	7.4 (2.4 to 14)	-46.9 (-67.4 to 4.8)	-18.8 (-52.6 to 52)	-52.9 (-72.1 to 6.9)
South Asia	South Asia	Bhutan	YLLs	All Ages	20 (5 to 40)	2 (1 to 3)	18 (3 to 38)	21 (7 to 40)	3 (2 to 5)	18 (4 to 36)	2.5 (-40.9 to 107.8)	59.3 (-11.8 to 213.4)	-3.4 (-46.9 to 118.7)
South Asia	South Asia	Bhutan	YLLs	Age-standardized	7.9 (1.8 to 15.3)	1.5 (0.7 to 2.5)	15 (2.1 to 30.3)	3.9 (1.2 to 7.3)	1.2 (0.7 to 1.9)	6.4 (1.4 to 12.8)	-51.5 (-71.6 to -1.5)	-21.5 (-57.7 to 59.3)	-57.3 (-76.4 to 1.5)
South Asia	South Asia	Bhutan	YLDs	All Ages	1 (1 to 1)	0 (0 to 0)	1 (1 to 1)	3 (2 to 5)	0 (0 to 1)	3 (2 to 4)	212.6 (159.3 to 278.5)	129.1 (98.1 to 167.6)	228.7 (166 to 309.9)
South Asia	South Asia	Bhutan	YLDs	Age-standardized	0.4 (0.3 to 0.6)	0.1 (0.1 to 0.2)	0.8 (0.5 to 1.1)	0.6 (0.4 to 0.9)	0.1 (0.1 to 0.2)	1 (0.6 to 1.5)	40.8 (15.5 to 71.3)	14.2 (-0.3 to 30.7)	34.2 (6.4 to 67.5)
South Asia	South Asia	India	Incidence	All Ages	4,473 (3,803 to 5,241)	2,015 (1,638 to 2,472)	2,457 (2,047 to 2,937)	11,669 (9,773 to 13,696)	7,286 (5,775 to 8,934)	4,383 (3,708 to 5,180)	160.9 (137.4 to 188.4)	261.5 (217.6 to 311.8)	78.4 (65.3 to 91)
South Asia	South Asia	India	Incidence	Age-standardized	0.9 (0.7 to 1)	0.7 (0.6 to 0.9)	1 (0.8 to 1.2)	0.9 (0.8 to 1.1)	1.1 (0.9 to 1.4)	0.8 (0.6 to 0.9)	10.1 (1.2 to 20.2)	57.8 (41 to 77.1)	-23.1 (-27.1 to -19.2)
South Asia	South Asia	India	Prevalence	All Ages	35,898 (29,665 to 43,411)	14,708 (11,678 to 18,517)	21,190 (16,880 to 26,051)	79,358 (66,736 to 96,189)	37,464 (30,709 to 46,743)	41,894 (34,263 to 50,722)	121.1 (106.3 to 137)	154.7 (130 to 182)	97.7 (81 to 114.5)
South Asia	South Asia	India	Prevalence	Age-standardized	8 (6.6 to 9.6)	6 (4.9 to 7.5)	10 (8.1 to 12.2)	6.9 (5.8 to 8.3)	6.2 (5.1 to 7.7)	7.7 (6.4 to 9.3)	-13.9 (-19.3 to -8.6)	3.3 (-5.3 to 12.6)	-22.4 (-27.9 to -17.1)
South Asia	South Asia	India	Deaths	All Ages	2,070 (705 to 3,154)	142 (78 to 216)	1,928 (554 to 3,015)	2,863 (1,688 to 4,121)	343 (216 to 475)	2,520 (1,348 to 3,757)	38.3 (0.9 to 169.3)	141.2 (69 to 247.1)	30.8 (-7.4 to 185.1)
South Asia	South Asia	India	Deaths	Age-standardized	0.6 (0.2 to 0.8)	0.1 (0.1 to 0.1)	1 (0.3 to 1.6)	0.3 (0.2 to 0.4)	0.1 (0 to 0.1)	0.5 (0.3 to 0.7)	-50.3 (-63.3 to -7.6)	-27.3 (-49.8 to 6.1)	-49.2 (-63.7 to 10)
South Asia	South Asia	India	DALYs	All Ages	57,133 (22,305 to 85,664)	5,572 (3,910 to 7,462)	51,561 (16,591 to 79,722)	75,204 (47,642 to 105,400)	12,390 (9,055 to 16,142)	62,814 (35,791 to 92,518)	31.6 (-3.1 to 135.5)	122.4 (80.9 to 175)	21.8 (-13.3 to 144.9)
South Asia	South Asia	India	DALYs	Age-standardized	12.7 (5 to 18.8)	2.6 (1.8 to 3.5)	22.2 (7.3 to 34)	6.6 (4.2 to 9.2)	2.1 (1.6 to 2.8)	11.4 (6.6 to 16.6)	-48 (-61.2 to -8.5)	-17.5 (-34.8 to 3)	-48.8 (-63.4 to 1.3)
South Asia	South Asia	India	YLLs	All Ages	51,749 (17,177 to 79,604)	3,262 (1,862 to 4,815)	48,488 (13,852 to 76,442)	63,079 (35,721 to 93,636)	6,503 (4,191 to 8,988)	56,577 (29,635 to 86,234)	21.9 (-12.4 to 138.3)	99.4 (40.6 to 184.4)	16.7 (-18.4 to 153.4)
South Asia	South Asia	India	YLLs	Age-standardized	11.5 (4 to 17.5)	1.7 (0.9 to 2.5)	20.8 (6 to 32.4)	5.6 (3.2 to 8.2)	1.2 (0.7 to 1.6)	10.2 (5.4 to 15.4)	-51.8 (-65.1 to -8.8)	-29.4 (-50.7 to 2.3)	-50.8 (-65.5 to 5.9)
South Asia	South Asia	India	YLDs	All Ages	5,383 (3,509 to 7,757)	2,310 (1,471 to 3,468)	3,073 (1,990 to 4,457)	12,125 (7,898 to 17,150)	5,887 (3,837 to 8,756)	6,237 (4,047 to 9,055)	125.2 (108.6 to 143.3)	154.8 (130.2 to 182)	103 (82.4 to 124.1)
South Asia	South Asia	India	YLDs	Age-standardized	1.2 (0.8 to 1.7)	0.9 (0.6 to 1.4)	1.4 (0.9 to 2)	1 (0.7 to 1.5)	1 (0.6 to 1.4)	1.1 (0.8 to 1.7)	-11.4 (-17.3 to -4.9)	3.4 (-5.2 to 12.6)	-19.5 (-26.8 to -12.1)
South Asia	South Asia	Nepal	Incidence	All Ages	48 (40 to 57)	15 (10 to 20)	33 (27 to 40)	123 (102 to 146)	46 (32 to 61)	77 (64 to 93)	158 (121.6 to 196.5)	215.4 (127.2 to 337.7)	132.7 (104.7 to 163)
South Asia	South Asia	Nepal	Incidence	Age-standardized	0.5 (0.4 to 0.5)	0.3 (0.2 to 0.3)	0.6 (0.5 to 0.8)	0.5 (0.4 to 0.6)	0.3 (0.2 to 0.5)	0.7 (0.6 to 0.8)	11.6 (-1 to 24.9)	34.2 (2.4 to 72.8)	6.8 (-4.9 to 18.4)
South Asia	South Asia	Nepal	Prevalence	All Ages	306 (244 to 381)	30 (24 to 39)	276 (214 to 352)	890 (728 to 1,086)	102 (80 to 130)	788 (631 to 978)	190.7 (151.7 to 227.9)	237.6 (170.4 to 310)	185.6 (144.6 to 226.8)
South Asia	South Asia	Nepal	Prevalence	Age-standardized	3.4 (2.8 to 4.2)	0.6 (0.5 to 0.8)	6.1 (4.9 to 7.6)	4.2 (3.5 to 5.1)	0.9 (0.7 to 1.1)	7.9 (6.4 to 9.8)	23.2 (8.1 to 38.1)	38.6 (15.9 to 64.8)	29.6 (12.4 to 46.4)
South Asia	South Asia	Nepal	Deaths	All Ages	60 (13 to 103)	5 (2 to 8)	55 (9 to 97)	99 (32 to 165)	12 (7 to 21)	86 (22 to 149)	65.5 (2.4 to 199.8)	156.1 (43.2 to 354)	57.5 (-6.9 to 214.5)
South Asia	South Asia	Nepal	Deaths	Age-standardized	0.7 (0.2 to 1.2)	0.1 (0.1 to 0.2)	1.3 (0.2 to 2.3)	0.5 (0.2 to 0.8)	0.1 (0.1 to 0.2)	0.9 (0.2 to 1.6)	-31 (-57.3 to 23.8)	-1.2 (-41.9 to 68.9)	-30.7 (-58.8 to 37.1)
South Asia	South Asia	Nepal	DALYs	All Ages	1,531 (372 to 2,616)	122 (61 to 209)	1,409 (266 to 2,479)	2,251 (816 to 3,758)	266 (150 to 435)	1,985 (568 to 3,446)	47.1 (-8.1 to 162.4)	117.9 (25.4 to 283.6)	40.9 (-16 to 175.4)
South Asia	South Asia	Nepal	DALYs	Age-standardized	16.1 (4 to 27.4)	2.7 (1.3 to 4.6)	29 (5.4 to 50.7)	10.3 (3.8 to 16.9)	2.4 (1.4 to 3.8)	19 (5.5 to 32.5)	-36.2 (-59.9 to 12.7)	-11.1 (-48 to 52.8)	-34.5 (-60.3 to 26.3)
South Asia	South Asia	Nepal	YLLs	All Ages	1,484 (328 to 2,571)	117 (56 to 203)	1,367 (224 to 2,439)	2,120 (675 to 3,636)	250 (136 to 417)	1,870 (445 to 3,335)	42.8 (-12.8 to 161.4)	113.1 (18.5 to 287.2)	36.8 (-20.5 to 176.1)
South Asia	South Asia	Nepal	YLLs	Age-standardized	15.6 (3.5 to 27)	2.6 (1.2 to 4.5)	28.1 (4.5 to 49.5)	9.7 (3.1 to 16.3)	2.2 (1.2 to 3.7)	17.8 (4.4 to 31.4)	-38.1 (-61.7 to 11.1)	-13 (-50.6 to 53.4)	-36.4 (-62.6 to 26)
South Asia	South Asia	Nepal	YLDs	All Ages	47 (29 to 69)	5 (3 to 7)	42 (26 to 62)	131 (81 to 193)	16 (10 to 24)	115 (70 to 173)	181.4 (117.9 to 265.3)	236.9 (170.4 to 308.3)	175.1 (104.8 to 270.5)
South Asia	South Asia	Nepal	YLDs	Age-standardized	0.5 (0.3 to 0.8)	0.1 (0.1 to 0.2)	0.9 (0.6 to 1.4)	0.6 (0.4 to 0.9)	0.1 (0.1 to 0.2)	1.1 (0.7 to 1.7)	19.9 (-7.1 to 56.2)	38.1 (15.6 to 63.6)	25.5 (-5.8 to 67.5)
South Asia	South Asia	Pakistan	Incidence	All Ages	252 (210 to 302)	52 (35 to 73)	199 (167 to 238)	579 (476 to 699)	239 (174 to 320)	340 (280 to 408)	129.9 (100.9 to 162.2)	354.9 (233.9 to 515.2)	70.7 (54.9 to 87.9)
South Asia	South Asia	Pakistan	Incidence	Age-standardized	0.4 (0.3 to 0.5)	0.2 (0.1 to 0.2)	0.6 (0.5 to 0.7)	0.5 (0.4 to 0.5)	0.3 (0.3 to 0.4)	0.6 (0.5 to 0.7)	11.5 (0.8 to 23.8)	102.5 (55.7 to 165.3)	-8.1 (-16 to 0.2)
South Asia	South Asia	Pakistan	Prevalence	All Ages	2,008 (1,634 to 2,434)	171 (140 to 212)	1,837 (1,475 to 2,256)	3,251 (2,681 to 3,897)	535 (436 to 659)	2,717 (2,183 to 3,339)	61.9 (45.3 to 82.1)	212.7 (165.8 to 272.8)	47.9 (30.9 to 68.4)
South Asia	South Asia	Pakistan	Prevalence	Age-standardized	3.6 (2.9 to 4.4)	0.7 (0.5 to 0.8)	5.9 (4.8 to 7.3)	3 (2.5 to 3.6)	0.9 (0.8 to 1.1)	4.9 (4 to 6.1)	-16.4 (-24.7 to -7)	43.2 (25.3 to 66.6)	-16.6 (-26.3 to -6.3)
South Asia	South Asia	Pakistan	Deaths	All Ages	216 (57 to 341)	9 (5 to 15)	207 (46 to 333)	241 (120 to 366)	16 (11 to 23)	226 (105 to 351)	12 (-26.5 to 135.4)	74.8 (0.2 to 208.6)	9.3 (-29.3 to 155.7)
South Asia	South Asia	Pakistan	Deaths	Age-standardized	0.4 (0.1 to 0.7)	0 (0 to 0.1)	0.7 (0.2 to 1.1)	0.3 (0.1 to 0.4)	0 (0 to 0.1)	0.5 (0.2 to 0.7)	-37.1 (-58.2 to 29.8)	-7.3 (-46.5 to 65)	-33.4 (-56.2 to 58.1)
South Asia	South Asia	Pakistan	DALYs	All Ages	5,211 (1,627 to 8,074)	213 (132 to 329)	4,998 (1,377 to 7,865)	6,379 (3,380 to 9,593)	440 (320 to 614)	5,939 (2,984 to 9,162)	22.4 (-17.8 to 130.8)	106.8 (28.3 to 226.2)	18.8 (-22.2 to 139.5)
South Asia	South Asia	Pakistan	DALYs	Age-standardized	9.1 (2.8 to 14.2)	0.8 (0.5 to 1.3)	15.9 (4.3 to 24.9)	5.8 (3.1 to 8.5)	0.8 (0.6 to 1.1)	10.4 (5.2 to 15.7)	-36.9 (-57.3 to 20.6)	-1.1 (-38.4 to 55.2)	-34.4 (-56.7 to 37.1)
South Asia	South Asia	Pakistan	YLLs	All Ages	4,903 (1,330 to 7,772)	186 (104 to 301)	4,717 (1,098 to 7,594)	5,866 (2,892 to 9,108)	356 (248 to 530)	5,510 (2,546 to 8,751)	19.6 (-22.2 to 143.2)	91.6 (10.5 to 226)	16.8 (-25.6 to 158.9)
South Asia	South Asia	Pakistan	YLLs	Age-standardized	8.6 (2.3 to 13.6)	0.7 (0.4 to 1.2)	15 (3.4 to 24)	5.3 (2.6 to 8.1)	0.7 (0.5 to 1)	9.6 (4.5 to 15)	-38.4 (-59.4 to 28.6)	-7.3 (-47.1 to 58.1)	-35.7 (-58.1 to 48.1)
South Asia	South Asia	Pakistan	YLDs	All Ages	308 (199 to 451)	27 (17 to 41)	281 (179 to 415)	513 (332 to 744)	84 (54 to 124)	429 (275 to 623)	66.7 (45.9 to 93.1)	211.7 (165 to 271.7)	52.7 (31.1 to 79.3)
South Asia	South Asia	Pakistan	YLDs	Age-standardized	0.5 (0.4 to 0.8)	0.1 (0.1 to 0.2)	0.9 (0.6 to 1.3)	0.5 (0.3 to 0.7)	0.1 (0.1 to 0.2)	0.8 (0.5 to 1.1)	-13.7 (-24.6 to -0.1)	42.8 (25.1 to 66)	-13.7 (-25.9 to 0.3)
		Sub-Saharan Africa	Incidence	All Ages	2,691 (2,164 to 3,253)	817 (650 to 989)	1,874 (1,506 to 2,268)	5,464 (4,417 to 6,582)	2,675 (2,111 to 3,276)	2,790 (2,290 to 3,342)	103.1 (95.7 to 110.6)	227.5 (212.3 to 242.6)	48.9 (40.3 to 57.9)
		Sub-Saharan Africa	Incidence	Age-standardized	1.1 (0.9 to 1.4)	0.7 (0.6 to 0.8)	1.6 (1.3 to 1.9)	1 (0.9 to 1.3)	0.9 (0.8 to 1.2)	1.2 (1 to 1.4)	-6.9 (-10 to -3.8)	38.3 (31.3 to 44.8)	-26.5 (-30.4 to -22.8)
		Sub-Saharan Africa	Prevalence	All Ages	8,752 (7,584 to 10,044)	2,564 (2,168 to 3,003)	6,188 (5,335 to 7,179)	19,572 (17,073 to 22,461)	8,240 (6,901 to 9,830)	11,332 (9,774 to 13,137)	123.6 (116.7 to 130)	221.4 (205.3 to 237.7)	83.1 (74.5 to 91.8)
		Sub-Saharan Africa	Prevalence	Age-standardized	4.2 (3.6 to 4.8)	2.5 (2.1 to 2.9)	5.9 (5.1 to 6.9)	4.4 (3.8 to 5)	3.4 (2.8 to 4)	5.5 (4.7 to 6.4)	3.6 (0.4 to 6.7)	34.6 (27.3 to 42.1)	-8 (-11.8 to -3.9)
		Sub-Saharan Africa	Deaths	All Ages	607 (320 to 822)	125 (89 to 177)	482 (181 to 693)	897 (551 to 1,201)	219 (155 to 344)	678 (323 to 1,015)	47.7 (15.4 to 87.3)	75.2 (42.5 to 119.6)	40.6 (4.7 to 96.4)
		Sub-Saharan Africa	Deaths	Age-standardized	0.3 (0.2 to 0.4)	0.1 (0.1 to 0.2)	0.5 (0.2 to 0.7)	0.2 (0.1 to 0.3)	0.1 (0.1 to 0.2)	0.3 (0.2 to 0.5)	-31.7 (-45.3 to -13.9)	-22.8 (-37.1 to -4.9)	-32.5 (-48.4 to -4.4)
		Sub-Saharan Africa	DALYs	All Ages	17,593 (10,074 to 23,241)	3,744 (2,847 to 4,993)	13,849 (5,970 to 19,485)	27,219 (17,648 to 36,080)	7,065 (5,296 to 10,192)	20,154 (10,392 to 29,614)	54.7 (20.5 to 91.1)	88.7 (53.4 to 136.9)	45.5 (8.3 to 89.9)
		Sub-Saharan Africa	DALYs	Age-standardized	7.7 (4.3 to 10.2)	3.1 (2.3 to 4.3)	12.4 (5.2 to 17.3)	5.4 (3.5 to 7)	2.6 (1.9 to 3.7)	8.6 (4.5 to 12.5)	-29.9 (-44.5 to -13.1)	-17.8 (-31.3 to 0.5)	-30.9 (-48 to -8.3)
		Sub-Saharan Africa	YLLs	All Ages	16,249 (8,724 to 21,929)	3,359 (2,489 to 4,594)	12,889 (5,037 to 18,587)	24,252 (14,984 to 32,981)	5,847 (4,183 to 9,007)	18,405 (8,744 to 27,932)	49.3 (14.2 to 88.4)	74.1 (37.9 to 127.1)	42.8 (4.2 to 90.1)
		Sub-Saharan Africa	YLLs	Age-standardized	7 (3.7 to 9.5)	2.7 (2 to 3.9)	11.5 (4.4 to 16.5)	4.7 (2.9 to 6.4)	2.1 (1.5 to 3.2)	7.7 (3.7 to 11.6)	-32.9 (-48 to -14.8)	-24.6 (-38.9 to -3.9)	-32.7 (-50.2 to -8.4)
		Sub-Saharan Africa	YLDs	All Ages	1,345 (913 to 1,889)	385 (258 to 556)	960 (647 to 1,359)	2,967 (2,006 to 4,139)	1,218 (811 to 1,751)	1,750 (1,168 to 2,441)	120.7 (113 to 129.5)	216.2 (194 to 240.4)	82.3 (72.1 to 92.8)
		Sub-Saharan Africa	YLDs	Age-standardized	0.6 (0.4 to 0.9)	0.4 (0.3 to 0.5)	0.9 (0.6 to 1.3)	0.7 (0.4 to 0.9)	0.5 (0.3 to 0.7)	0.8 (0.6 to 1.2)	2.4 (-1.7 to 6.4)	32.6 (23 to 43.2)	-8.4 (-13.2 to -3.4)
		Central Sub-Saharan Africa	Incidence	All Ages	201 (165 to 244)	49 (37 to 61)	152 (124 to 185)	526 (426 to 644)	163 (126 to 204)	363 (290 to 448)	162 (144.4 to 177.6)	235.2 (193.2 to 285)	138.7 (121.2 to 155.8)
		Central Sub-Saharan Africa	Incidence	Age-standardized	0.9 (0.7 to 1)	0.4 (0.4 to 0.5)	1.3 (1.1 to 1.6)	1 (0.8 to 1.2)	0.6 (0.5 to 0.7)	1.4 (1.2 to 1.7)	12.7 (7 to 19.1)	31.2 (18.8 to 46)	7.4 (1.1 to 14.5)
		Central Sub-Saharan Africa	Prevalence	All Ages	678 (578 to 794)	151 (124 to 184)	527 (441 to 622)	1,827 (1,564 to 2,120)	495 (410 to 590)	1,332 (1,106 to 1,586)	169.3 (152.8 to 187.8)	226.7 (198.4 to 259.5)	152.8 (133.6 to 176.1)
		Central Sub-Saharan Africa	Prevalence	Age-standardized	3.4 (2.9 to 3.9)	1.6 (1.3 to 1.9)	5.4 (4.5 to 6.3)	4 (3.4 to 4.7)	2.1 (1.7 to 2.5)	6.6 (5.5 to 7.8)	18.9 (11.5 to 26.8)	27.9 (18.1 to 39.5)	23.8 (14 to 35.3)
		Central Sub-Saharan Africa	Deaths	All Ages	101 (56 to 151)	25 (12 to 46)	77 (23 to 124)	169 (90 to 274)	51 (26 to 119)	118 (36 to 226)	66.7 (5.1 to 164.7)	108.4 (23.4 to 241.5)	53.4 (-11.5 to 161.5)
		Central Sub-Saharan Africa	Deaths	Age-standardized	0.5 (0.3 to 0.8)	0.3 (0.1 to 0.5)	0.8 (0.2 to 1.3)	0.4 (0.2 to 0.6)	0.2 (0.1 to 0.5)	0.6 (0.2 to 1.1)	-28.2 (-53.9 to 12.7)	-17.5 (-49.6 to 33)	-29.2 (-58.1 to 19.5)
		Central Sub-Saharan Africa	DALYs	All Ages	2,852 (1,607 to 4,207)	664 (364 to 1,190)	2,188 (753 to 3,455)	4,782 (2,518 to 7,710)	1,257 (710 to 2,588)	3,525 (1,234 to 6,611)	67.7 (9.1 to 156.1)	89.1 (19.4 to 199.2)	61.1 (-4.1 to 165.3)
		Central Sub-Saharan Africa	DALYs	Age-standardized	11.9 (6.9 to 17.3)	5.5 (3 to 9.9)	19.1 (6.3 to 30)	8.5 (4.8 to 13.5)	4.4 (2.4 to 9.6)	13.5 (4.8 to 24.9)	-28 (-52.5 to 10.5)	-19.6 (-49.7 to 25.7)	-29.2 (-57.6 to 16.2)
		Central Sub-Saharan Africa	YLLs	All Ages	2,744 (1,508 to 4,087)	640 (339 to 1,167)	2,104 (671 to 3,376)	4,495 (2,272 to 7,386)	1,178 (631 to 2,506)	3,316 (1,035 to 6,332)	63.8 (4.2 to 155.9)	84 (13.3 to 197.2)	57.6 (-9 to 166.6)
		Central Sub-Saharan Africa	YLLs	Age-standardized	11.3 (6.3 to 16.8)	5.2 (2.7 to 9.7)	18.2 (5.4 to 29.1)	7.9 (4.2 to 12.8)	4.1 (2.1 to 9.2)	12.5 (3.7 to 23.9)	-30.1 (-55.2 to 10.3)	-21.8 (-53 to 25.6)	-31.5 (-60 to 16)
		Central Sub-Saharan Africa	YLDs	All Ages	108 (71 to 153)	24 (15 to 35)	84 (54 to 120)	287 (189 to 407)	78 (51 to 116)	208 (135 to 299)	165.6 (147.9 to 185.8)	225.6 (197.9 to 257.9)	148.4 (127.4 to 174.1)
		Central Sub-Saharan Africa	YLDs	Age-standardized	0.5 (0.3 to 0.8)	0.3 (0.2 to 0.4)	0.9 (0.6 to 1.2)	0.6 (0.4 to 0.9)	0.3 (0.2 to 0.5)	1 (0.7 to 1.5)	16.5 (8.2 to 26.1)	27.4 (17.8 to 38.8)	20 (8.5 to 34)
Sub-Saharan Africa	Central sub-Saharan Africa	Angola	Incidence	All Ages	37 (30 to 45)	8 (6 to 11)	29 (23 to 36)	106 (84 to 131)	33 (24 to 42)	73 (58 to 91)	187.8 (157.6 to 217.3)	301.3 (232.3 to 384.1)	155.6 (123.7 to 190.5)
Sub-Saharan Africa	Central sub-Saharan Africa	Angola	Incidence	Age-standardized	0.9 (0.7 to 1.1)	0.5 (0.4 to 0.6)	1.4 (1.1 to 1.6)	0.9 (0.7 to 1.1)	0.6 (0.4 to 0.7)	1.4 (1.1 to 1.7)	1.6 (-6.7 to 10)	22.5 (6.2 to 40.7)	0.1 (-9.9 to 10.5)
Sub-Saharan Africa	Central sub-Saharan Africa	Angola	Prevalence	All Ages	115 (97 to 135)	23 (18 to 28)	92 (76 to 110)	350 (294 to 410)	87 (71 to 106)	263 (215 to 314)	204.3 (179.4 to 230.7)	282.5 (238.2 to 335.4)	184.9 (155.6 to 215.6)
Sub-Saharan Africa	Central sub-Saharan Africa	Angola	Prevalence	Age-standardized	3.3 (2.8 to 3.9)	1.4 (1.2 to 1.8)	5.3 (4.4 to 6.2)	3.7 (3.1 to 4.3)	1.8 (1.4 to 2.1)	6.2 (5.1 to 7.4)	11.8 (3 to 20.9)	22.5 (8.9 to 38.8)	17.9 (6.1 to 29.7)
Sub-Saharan Africa	Central sub-Saharan Africa	Angola	Deaths	All Ages	16 (9 to 26)	4 (2 to 7)	12 (4 to 21)	21 (14 to 29)	7 (4 to 12)	14 (7 to 20)	30 (-18.6 to 115.3)	89.6 (2.8 to 270.2)	11.6 (-35 to 128.3)
Sub-Saharan Africa	Central sub-Saharan Africa	Angola	Deaths	Age-standardized	0.5 (0.3 to 0.8)	0.2 (0.1 to 0.4)	0.7 (0.2 to 1.2)	0.2 (0.2 to 0.3)	0.2 (0.1 to 0.3)	0.3 (0.2 to 0.5)	-50.9 (-68.6 to -18.7)	-35.8 (-64.4 to 31.8)	-54.3 (-73.3 to -2.7)
Sub-Saharan Africa	Central sub-Saharan Africa	Angola	DALYs	All Ages	475 (262 to 753)	106 (58 to 185)	369 (137 to 632)	616 (422 to 839)	191 (121 to 289)	425 (246 to 613)	29.7 (-18 to 112.1)	79.5 (4.2 to 220.8)	15.3 (-31.8 to 128.8)
Sub-Saharan Africa	Central sub-Saharan Africa	Angola	DALYs	Age-standardized	11.1 (6.3 to 17.7)	5.2 (2.6 to 9.2)	17.1 (6 to 29)	5.3 (3.7 to 7.2)	3.2 (2 to 5)	7.9 (4.5 to 11.3)	-52.4 (-69.4 to -23)	-38.4 (-65 to 16)	-53.9 (-72.5 to -9.3)
Sub-Saharan Africa	Central sub-Saharan Africa	Angola	YLLs	All Ages	457 (245 to 733)	103 (54 to 181)	354 (121 to 618)	561 (368 to 784)	177 (108 to 274)	384 (207 to 577)	22.8 (-23.2 to 107.7)	72.4 (-2.8 to 218.2)	8.5 (-37.6 to 124.7)
Sub-Saharan Africa	Central sub-Saharan Africa	Angola	YLLs	Age-standardized	10.6 (5.9 to 17)	4.9 (2.4 to 8.9)	16.3 (5.2 to 28.2)	4.7 (3.2 to 6.6)	2.9 (1.7 to 4.7)	6.9 (3.7 to 10.3)	-55.4 (-71.9 to -25.5)	-41.2 (-67.9 to 15.8)	-57.4 (-75.4 to -11.6)
Sub-Saharan Africa	Central sub-Saharan Africa	Angola	YLDs	All Ages	18 (12 to 26)	4 (2 to 5)	15 (10 to 21)	55 (35 to 79)	14 (9 to 21)	41 (27 to 59)	200.2 (172.2 to 230.3)	281.4 (237.4 to 333.9)	180.1 (148.4 to 216.6)
Sub-Saharan Africa	Central sub-Saharan Africa	Angola	YLDs	Age-standardized	0.5 (0.3 to 0.7)	0.2 (0.1 to 0.3)	0.8 (0.5 to 1.2)	0.6 (0.4 to 0.8)	0.3 (0.2 to 0.4)	1 (0.6 to 1.4)	9.4 (-1.6 to 21.5)	22.1 (8.7 to 37.7)	14.3 (0.1 to 31.6)
Sub-Saharan Africa	Central sub-Saharan Africa	Central African Republic	Incidence	All Ages	13 (10 to 16)	3 (2 to 4)	10 (8 to 12)	27 (21 to 33)	8 (6 to 11)	18 (15 to 23)	102.2 (85.6 to 121.4)	161.9 (118.7 to 222.8)	83.3 (64.4 to 103.5)
Sub-Saharan Africa	Central sub-Saharan Africa	Central African Republic	Incidence	Age-standardized	1.1 (0.9 to 1.3)	0.5 (0.4 to 0.7)	1.7 (1.4 to 2)	1.2 (1 to 1.4)	0.7 (0.6 to 0.9)	1.7 (1.4 to 2.1)	9.8 (2.1 to 17.4)	32.7 (15.9 to 53.6)	2.7 (-5.7 to 11.3)
Sub-Saharan Africa	Central sub-Saharan Africa	Central African Republic	Prevalence	All Ages	39 (33 to 45)	8 (7 to 10)	31 (25 to 36)	74 (63 to 87)	19 (16 to 23)	55 (46 to 66)	92 (77.5 to 107)	136.7 (108.9 to 171.1)	80 (62.9 to 97.4)
Sub-Saharan Africa	Central sub-Saharan Africa	Central African Republic	Prevalence	Age-standardized	3.7 (3.1 to 4.2)	1.6 (1.3 to 1.9)	6.2 (5.1 to 7.3)	3.9 (3.4 to 4.6)	2 (1.6 to 2.4)	6.7 (5.6 to 7.9)	7.9 (0.9 to 15.3)	23.3 (10.7 to 39.2)	8.1 (-0.8 to 17.6)
Sub-Saharan Africa	Central sub-Saharan Africa	Central African Republic	Deaths	All Ages	8 (4 to 14)	2 (1 to 3)	7 (2 to 12)	12 (5 to 20)	2 (1 to 5)	10 (2 to 19)	42.7 (-13.8 to 132.4)	48.7 (-19.2 to 168.2)	41.3 (-19.2 to 140.5)
Sub-Saharan Africa	Central sub-Saharan Africa	Central African Republic	Deaths	Age-standardized	0.8 (0.4 to 1.3)	0.3 (0.1 to 0.7)	1.3 (0.3 to 2.3)	0.6 (0.3 to 1)	0.3 (0.1 to 0.6)	1.1 (0.2 to 2)	-21.6 (-50.1 to 20.8)	-18.5 (-54.2 to 43.1)	-19.8 (-51.5 to 30.2)
Sub-Saharan Africa	Central sub-Saharan Africa	Central African Republic	DALYs	All Ages	244 (117 to 405)	42 (22 to 77)	202 (63 to 365)	362 (153 to 630)	62 (32 to 127)	300 (83 to 577)	48.8 (-9.6 to 139.8)	48 (-16.1 to 162)	48.9 (-15.2 to 152.4)
Sub-Saharan Africa	Central sub-Saharan Africa	Central African Republic	DALYs	Age-standardized	18.9 (9.3 to 31.1)	6.6 (3.3 to 12.6)	33.1 (10 to 58.1)	14.8 (6.6 to 24.9)	5.3 (2.6 to 11.2)	26.3 (7.1 to 50.3)	-21.5 (-50.9 to 23.9)	-19.7 (-53.4 to 37.9)	-20.5 (-52.2 to 30.3)
Sub-Saharan Africa	Central sub-Saharan Africa	Central African Republic	YLLs	All Ages	237 (111 to 399)	41 (21 to 76)	197 (58 to 360)	351 (142 to 618)	59 (29 to 123)	292 (75 to 568)	47.7 (-11.9 to 141)	45.2 (-20.6 to 162.6)	48.2 (-17.6 to 154.9)
Sub-Saharan Africa	Central sub-Saharan Africa	Central African Republic	YLLs	Age-standardized	18.3 (8.9 to 30.5)	6.3 (3.1 to 12.4)	32.1 (9.1 to 57.2)	14.2 (6 to 24.1)	5 (2.3 to 11)	25.3 (6.1 to 49.2)	-22.4 (-52.7 to 24.7)	-21.4 (-56.1 to 38.7)	-21.3 (-54.3 to 31.2)
Sub-Saharan Africa	Central sub-Saharan Africa	Central African Republic	YLDs	All Ages	6 (4 to 9)	1 (1 to 2)	5 (3 to 7)	12 (8 to 17)	3 (2 to 5)	9 (6 to 13)	90.3 (75.3 to 106)	136.1 (108.7 to 170.2)	78.1 (60.6 to 96.5)
Sub-Saharan Africa	Central sub-Saharan Africa	Central African Republic	YLDs	Age-standardized	0.6 (0.4 to 0.8)	0.3 (0.2 to 0.4)	1 (0.6 to 1.4)	0.6 (0.4 to 0.9)	0.3 (0.2 to 0.5)	1 (0.7 to 1.5)	6.4 (-1.7 to 16)	22.9 (10.6 to 38.9)	5.6 (-6.2 to 19.7)
Sub-Saharan Africa	Central sub-Saharan Africa	Congo	Incidence	All Ages	15 (12 to 19)	4 (3 to 5)	11 (9 to 14)	36 (28 to 44)	11 (9 to 15)	24 (19 to 30)	136.1 (112.7 to 159.7)	169.7 (134.3 to 212.9)	123.1 (95 to 152.2)
Sub-Saharan Africa	Central sub-Saharan Africa	Congo	Incidence	Age-standardized	1.3 (1 to 1.6)	0.7 (0.5 to 0.9)	2 (1.6 to 2.5)	1.3 (1 to 1.5)	0.8 (0.6 to 1)	1.7 (1.4 to 2.1)	-1 (-8.7 to 7)	16.4 (4 to 29.5)	-12.7 (-21 to -3.5)
Sub-Saharan Africa	Central sub-Saharan Africa	Congo	Prevalence	All Ages	40 (34 to 47)	10 (8 to 12)	30 (26 to 35)	103 (88 to 120)	28 (23 to 33)	75 (63 to 89)	155 (136.9 to 175.2)	172.6 (143.3 to 210.8)	149 (127.9 to 173)
Sub-Saharan Africa	Central sub-Saharan Africa	Congo	Prevalence	Age-standardized	3.9 (3.4 to 4.5)	1.9 (1.6 to 2.3)	6.4 (5.5 to 7.5)	4.4 (3.8 to 5.1)	2.4 (1.9 to 2.8)	6.7 (5.7 to 8)	12.9 (5.2 to 21.7)	22 (9.9 to 38.1)	4.5 (-4.2 to 14.4)
Sub-Saharan Africa	Central sub-Saharan Africa	Congo	Deaths	All Ages	5 (3 to 7)	1 (1 to 2)	4 (1 to 6)	6 (4 to 8)	2 (1 to 3)	4 (2 to 6)	14 (-28.6 to 87)	44.3 (-23.2 to 172)	2.8 (-42.1 to 90.6)
Sub-Saharan Africa	Central sub-Saharan Africa	Congo	Deaths	Age-standardized	0.5 (0.3 to 0.7)	0.3 (0.1 to 0.5)	0.8 (0.3 to 1.3)	0.3 (0.2 to 0.4)	0.2 (0.1 to 0.3)	0.4 (0.2 to 0.5)	-49.2 (-67.3 to -19)	-34.7 (-63.9 to 24.5)	-57.6 (-74.9 to -22.4)
Sub-Saharan Africa	Central sub-Saharan Africa	Congo	DALYs	All Ages	143 (87 to 202)	35 (19 to 58)	108 (49 to 170)	163 (106 to 227)	50 (31 to 79)	113 (60 to 169)	14.2 (-29.3 to 84.7)	44.1 (-20.5 to 158.6)	4.6 (-41.6 to 87.3)
Sub-Saharan Africa	Central sub-Saharan Africa	Congo	DALYs	Age-standardized	12.5 (7.8 to 17.6)	5.8 (3.2 to 10.3)	20.8 (9.1 to 32.2)	6.1 (4.1 to 8.4)	3.8 (2.3 to 6)	8.6 (4.8 to 12.7)	-51.2 (-68.7 to -22.8)	-34.9 (-62.8 to 15.9)	-58.9 (-75.5 to -27.6)
Sub-Saharan Africa	Central sub-Saharan Africa	Congo	YLLs	All Ages	136 (80 to 196)	33 (18 to 57)	103 (45 to 165)	147 (90 to 210)	46 (27 to 74)	101 (47 to 158)	7.7 (-36.2 to 81.4)	37.9 (-26.8 to 157.7)	-2 (-47.5 to 82.2)
Sub-Saharan Africa	Central sub-Saharan Africa	Congo	YLLs	Age-standardized	11.9 (7.2 to 17)	5.5 (2.9 to 10.1)	19.8 (8.2 to 31.5)	5.4 (3.4 to 7.7)	3.4 (2 to 5.7)	7.5 (3.6 to 11.6)	-54.5 (-71.7 to -25.3)	-38.1 (-66.4 to 15.5)	-62.1 (-78.4 to -30.2)
Sub-Saharan Africa	Central sub-Saharan Africa	Congo	YLDs	All Ages	6 (4 to 9)	2 (1 to 2)	5 (3 to 7)	16 (11 to 23)	4 (3 to 6)	12 (8 to 17)	153.5 (135.4 to 173.8)	171.6 (142.8 to 209)	147.4 (124.5 to 172.9)
Sub-Saharan Africa	Central sub-Saharan Africa	Congo	YLDs	Age-standardized	0.6 (0.4 to 0.9)	0.3 (0.2 to 0.5)	1 (0.7 to 1.5)	0.7 (0.5 to 1)	0.4 (0.2 to 0.5)	1.1 (0.7 to 1.5)	11.9 (3.9 to 21)	21.5 (9.7 to 37.4)	3.2 (-6.6 to 14)

Pneumoconiosis

7 GBD super region	21 GBD region	Location	Measure	Age	1990			2019			Percent change (1990 to 2019)		
					Both	Female	Male	Both	Female	Male	Both	Female	Male
Sub-Saharan Africa	Central sub-Saharan Africa	Democratic Republic of the Congo	Incidence	All Ages	128 (104 to 156)	31 (23 to 40)	97 (78 to 118)	342 (277 to 418)	106 (81 to 134)	236 (187 to 293)	168 (143.6 to 191.9)	241.6 (187.1 to 313.5)	144.4 (120.3 to 169.7)
Sub-Saharan Africa	Central sub-Saharan Africa	Democratic Republic of the Congo	Incidence	Age-standardized	0.8 (0.7 to 1)	0.4 (0.3 to 0.5)	1.2 (1 to 1.5)	1 (0.8 to 1.1)	0.6 (0.4 to 0.7)	1.4 (1.2 to 1.7)	18.8 (10.8 to 27.5)	37.2 (21 to 59.2)	13.5 (4.6 to 23.6)
Sub-Saharan Africa	Central sub-Saharan Africa	Democratic Republic of the Congo	Prevalence	All Ages	461 (390 to 541)	104 (84 to 128)	356 (296 to 423)	1,249 (1,064 to 1,452)	347 (288 to 419)	902 (748 to 1,080)	171.1 (148.9 to 195.6)	232.4 (193.7 to 280.6)	153.1 (127 to 185.4)
Sub-Saharan Africa	Central sub-Saharan Africa	Democratic Republic of the Congo	Prevalence	Age-standardized	3.3 (2.8 to 3.9)	1.6 (1.3 to 2)	5.2 (4.4 to 6.2)	4.1 (3.5 to 4.8)	2.1 (1.7 to 2.6)	6.8 (5.6 to 8.1)	21.8 (12 to 33.2)	29.8 (15.8 to 45.1)	29.1 (16 to 45.1)
Sub-Saharan Africa	Central sub-Saharan Africa	Democratic Republic of the Congo	Deaths	All Ages	69 (35 to 108)	17 (8 to 36)	52 (13 to 90)	128 (60 to 224)	39 (17 to 100)	89 (21 to 191)	85.7 (7.4 to 213)	127.3 (17.2 to 328.1)	72 (-11.6 to 215.9)
Sub-Saharan Africa	Central sub-Saharan Africa	Democratic Republic of the Congo	Deaths	Age-standardized	0.5 (0.3 to 0.8)	0.3 (0.1 to 0.6)	0.8 (0.2 to 1.3)	0.4 (0.2 to 0.7)	0.2 (0.1 to 0.7)	0.6 (0.1 to 1.4)	-19.8 (-53.7 to 30.4)	-11.3 (-54 to 57.4)	-18.3 (-57.2 to 45.3)
Sub-Saharan Africa	Central sub-Saharan Africa	Democratic Republic of the Congo	DALYs	All Ages	1,909 (1,002 to 3,009)	463 (232 to 904)	1,446 (424 to 2,501)	3,573 (1,725 to 6,228)	933 (462 to 2,139)	2,640 (735 to 5,384)	87.1 (9.9 to 210)	101.4 (13.2 to 260.6)	82.6 (-3.4 to 227.5)
Sub-Saharan Africa	Central sub-Saharan Africa	Democratic Republic of the Congo	DALYs	Age-standardized	11.6 (6.2 to 17.8)	5.5 (2.7 to 11.1)	18.5 (5.2 to 31.3)	9.5 (4.7 to 16.3)	4.9 (2.3 to 11.8)	15.2 (4.3 to 30.9)	-17.8 (-50.5 to 33)	-12.4 (-51.3 to 54)	-17.8 (-55.1 to 45.2)
Sub-Saharan Africa	Central sub-Saharan Africa	Democratic Republic of the Congo	YLLs	All Ages	1,836 (918 to 2,927)	447 (214 to 890)	1,389 (362 to 2,454)	3,377 (1,522 to 6,021)	878 (413 to 2,081)	2,499 (597 to 5,239)	84 (3.6 to 211.1)	96.6 (5.5 to 261.2)	79.9 (-6.9 to 233.4)
Sub-Saharan Africa	Central sub-Saharan Africa	Democratic Republic of the Congo	YLLs	Age-standardized	11 (5.6 to 17.3)	5.3 (2.5 to 10.8)	17.7 (4.5 to 30.5)	8.9 (4.1 to 15.7)	4.5 (2 to 11.4)	14.2 (3.3 to 30.1)	-19.6 (-53.2 to 34.4)	-14.5 (-54.2 to 55.6)	-19.8 (-58 to 46.1)
Sub-Saharan Africa	Central sub-Saharan Africa	Democratic Republic of the Congo	YLDs	All Ages	73 (48 to 105)	17 (11 to 24)	57 (37 to 83)	196 (128 to 279)	55 (35 to 81)	141 (92 to 204)	166.9 (142.9 to 193.8)	231.2 (193 to 279)	148.1 (120.7 to 182.5)
Sub-Saharan Africa	Central sub-Saharan Africa	Democratic Republic of the Congo	YLDs	Age-standardized	0.5 (0.3 to 0.8)	0.3 (0.2 to 0.4)	0.8 (0.5 to 1.2)	0.6 (0.4 to 0.9)	0.3 (0.2 to 0.5)	1 (0.7 to 1.5)	19.2 (8.2 to 32.3)	29.3 (15.6 to 44.5)	24.8 (8.7 to 44.2)
Sub-Saharan Africa	Central sub-Saharan Africa	Equatorial Guinea	Incidence	All Ages	2 (2 to 3)	1 (0 to 1)	2 (1 to 2)	4 (3 to 5)	1 (1 to 2)	3 (2 to 3)	84.1 (62.9 to 104.9)	136.6 (100.6 to 180.2)	65.1 (41.8 to 89.2)
Sub-Saharan Africa	Central sub-Saharan Africa	Equatorial Guinea	Incidence	Age-standardized	1.1 (0.9 to 1.3)	0.5 (0.4 to 0.7)	1.7 (1.4 to 2)	0.8 (0.6 to 1)	0.5 (0.4 to 0.6)	1.2 (1 to 1.4)	-25.4 (-32.3 to -19.1)	-9.1 (-20.4 to 3.2)	-30 (-37.5 to -22.7)
Sub-Saharan Africa	Central sub-Saharan Africa	Equatorial Guinea	Prevalence	All Ages	6 (5 to 7)	1 (1 to 2)	5 (4 to 6)	14 (12 to 17)	4 (3 to 5)	10 (8 to 12)	129.1 (108.2 to 151.3)	180.7 (146.3 to 216.5)	113.1 (88.5 to 137.2)
Sub-Saharan Africa	Central sub-Saharan Africa	Equatorial Guinea	Prevalence	Age-standardized	3.4 (3 to 4)	1.6 (1.3 to 1.9)	5.9 (4.9 to 6.9)	3.4 (2.9 to 4)	1.8 (1.5 to 2.2)	5.8 (4.8 to 7.1)	0.3 (-8.4 to 9.1)	14.5 (1.5 to 28.3)	-0.7 (-11.6 to 10.6)
Sub-Saharan Africa	Central sub-Saharan Africa	Equatorial Guinea	Deaths	All Ages	1 (1 to 2)	0 (0 to 0)	1 (0 to 2)	1 (0 to 1)	0 (0 to 1)	0 (0 to 1)	-37.1 (-69.1 to 50.2)	34.2 (-38.6 to 189.2)	-54.2 (-79.7 to 54.4)
Sub-Saharan Africa	Central sub-Saharan Africa	Equatorial Guinea	Deaths	Age-standardized	0.7 (0.3 to 1.1)	0.3 (0.1 to 0.5)	1.2 (0.3 to 2.1)	0.2 (0.1 to 0.3)	0.1 (0.1 to 0.3)	0.3 (0.1 to 0.4)	-70.5 (-85.5 to -31.5)	-43.8 (-74.1 to 25.6)	-77.9 (-90.3 to -20.7)
Sub-Saharan Africa	Central sub-Saharan Africa	Equatorial Guinea	DALYs	All Ages	34 (16 to 57)	6 (3 to 11)	28 (8 to 51)	20 (12 to 32)	7 (4 to 14)	13 (7 to 21)	-39.5 (-69.3 to 35.9)	25.2 (-40.8 to 170.6)	-53.2 (-78.7 to 42.3)
Sub-Saharan Africa	Central sub-Saharan Africa	Equatorial Guinea	DALYs	Age-standardized	15.8 (7.8 to 26.5)	5.3 (2.5 to 10.2)	29.1 (8.3 to 51.9)	4.2 (2.6 to 6.6)	2.7 (1.3 to 5.5)	6.2 (3.4 to 9.9)	-73.7 (-86.5 to -41.7)	-48.4 (-75.4 to 8.1)	-78.9 (-89.8 to -33.7)
Sub-Saharan Africa	Central sub-Saharan Africa	Equatorial Guinea	YLLs	All Ages	33 (15 to 56)	6 (3 to 11)	27 (7 to 50)	18 (10 to 30)	7 (3 to 13)	11 (5 to 20)	-44.6 (-73.2 to 31.3)	18.7 (-48.5 to 170.2)	-57.9 (-81.7 to 35.3)
Sub-Saharan Africa	Central sub-Saharan Africa	Equatorial Guinea	YLLs	Age-standardized	15.3 (7.2 to 26.1)	5.1 (2.3 to 9.9)	28.2 (7.2 to 51.2)	3.6 (2.1 to 6)	2.5 (1 to 5.2)	5.3 (2.5 to 8.8)	-76.3 (-88.5 to -44)	-51.4 (-78.5 to 7.6)	-81.4 (-91.8 to -37.6)
Sub-Saharan Africa	Central sub-Saharan Africa	Equatorial Guinea	YLDs	All Ages	1 (1 to 1)	0 (0 to 0)	1 (0 to 1)	2 (1 to 3)	1 (0 to 1)	2 (1 to 2)	127 (105.3 to 150.3)	180.1 (146.1 to 215.8)	110.4 (85.5 to 136.9)
Sub-Saharan Africa	Central sub-Saharan Africa	Equatorial Guinea	YLDs	Age-standardized	0.5 (0.4 to 0.8)	0.2 (0.2 to 0.4)	0.9 (0.6 to 1.3)	0.5 (0.4 to 0.8)	0.3 (0.2 to 0.4)	0.9 (0.6 to 1.3)	-1.2 (-10.2 to 9.1)	14.1 (1.1 to 27.9)	-3 (-15.1 to 10.4)
Sub-Saharan Africa	Central sub-Saharan Africa	Gabon	Incidence	All Ages	6 (5 to 7)	2 (1 to 2)	4 (3 to 5)	11 (9 to 14)	3 (3 to 4)	8 (6 to 10)	99.7 (81.9 to 118.8)	124.4 (90.1 to 163.4)	90.6 (70.1 to 110.5)
Sub-Saharan Africa	Central sub-Saharan Africa	Gabon	Incidence	Age-standardized	1 (0.8 to 1.2)	0.5 (0.4 to 0.6)	1.6 (1.2 to 1.9)	1.1 (0.8 to 1.3)	0.6 (0.5 to 0.8)	1.5 (1.2 to 1.9)	7 (-1.5 to 15.8)	22.3 (5.4 to 40)	-2.5 (-11.4 to 6.6)
Sub-Saharan Africa	Central sub-Saharan Africa	Gabon	Prevalence	All Ages	17 (15 to 20)	4 (4 to 6)	13 (11 to 15)	37 (31 to 43)	10 (8 to 12)	27 (23 to 33)	111.7 (94.8 to 129.4)	113.6 (88.4 to 139.5)	111.1 (89.9 to 133.3)
Sub-Saharan Africa	Central sub-Saharan Africa	Gabon	Prevalence	Age-standardized	3.2 (2.7 to 3.7)	1.6 (1.3 to 1.9)	5.3 (4.5 to 6.3)	3.8 (3.2 to 4.5)	1.9 (1.5 to 2.3)	6.2 (5.2 to 7.4)	19.8 (10.6 to 29.6)	19.8 (5.5 to 33.1)	17.5 (6.8 to 29.6)
Sub-Saharan Africa	Central sub-Saharan Africa	Gabon	Deaths	All Ages	2 (1 to 3)	1 (0 to 1)	1 (1 to 2)	2 (1 to 2)	1 (0 to 1)	1 (1 to 2)	-6.1 (-42.8 to 67.9)	7.1 (-49.9 to 115.9)	-11.7 (-53.8 to 105.1)
Sub-Saharan Africa	Central sub-Saharan Africa	Gabon	Deaths	Age-standardized	0.4 (0.2 to 0.5)	0.2 (0.1 to 0.3)	0.6 (0.3 to 0.9)	0.2 (0.1 to 0.3)	0.1 (0.1 to 0.2)	0.3 (0.2 to 0.4)	-46.4 (-67.2 to -7.9)	-38.4 (-70.1 to 24.1)	-51.9 (-74.5 to 9.1)
Sub-Saharan Africa	Central sub-Saharan Africa	Gabon	DALYs	All Ages	48 (30 to 72)	12 (7 to 19)	36 (19 to 58)	47 (32 to 67)	13 (8 to 20)	34 (21 to 53)	-2.3 (-40.4 to 70.9)	7 (-46 to 98.7)	-5.6 (-48 to 97.7)
Sub-Saharan Africa	Central sub-Saharan Africa	Gabon	DALYs	Age-standardized	8.3 (5.2 to 12.4)	4 (2.3 to 6.2)	13.6 (7.2 to 21.8)	4.4 (3.1 to 6.2)	2.4 (1.4 to 3.7)	6.7 (4.4 to 10.4)	-46.5 (-66.7 to -8.3)	-39 (-69.2 to 12.7)	-50.6 (-72.4 to 2.7)
Sub-Saharan Africa	Central sub-Saharan Africa	Gabon	YLLs	All Ages	45 (27 to 70)	11 (6 to 18)	34 (17 to 56)	41 (26 to 62)	11 (6 to 18)	29 (17 to 50)	-9.3 (-47.3 to 67.9)	0.5 (-53 to 97.2)	-12.6 (-55.5 to 97.6)
Sub-Saharan Africa	Central sub-Saharan Africa	Gabon	YLLs	Age-standardized	7.8 (4.7 to 11.9)	3.7 (2 to 5.9)	12.8 (6.4 to 20.9)	3.8 (2.6 to 5.6)	2.1 (1.1 to 3.4)	5.7 (3.5 to 9.5)	-50.7 (-70.2 to -11.8)	-42.9 (-73 to 11.3)	-55.1 (-76.7 to 1.1)
Sub-Saharan Africa	Central sub-Saharan Africa	Gabon	YLDs	All Ages	3 (2 to 4)	1 (0 to 1)	2 (1 to 3)	6 (4 to 8)	2 (1 to 2)	4 (3 to 6)	111.3 (94.3 to 128.9)	113 (88.3 to 138.6)	110.7 (89.7 to 132.8)
Sub-Saharan Africa	Central sub-Saharan Africa	Gabon	YLDs	Age-standardized	0.5 (0.3 to 0.7)	0.2 (0.2 to 0.4)	0.8 (0.5 to 1.2)	0.6 (0.4 to 0.9)	0.3 (0.2 to 0.4)	1 (0.6 to 1.4)	19.5 (10.3 to 29.2)	19.5 (5.4 to 32.6)	17.2 (6.5 to 29.2)
		Eastern Sub-Saharan Africa	Incidence	All Ages	845 (675 to 1,024)	138 (105 to 175)	707 (566 to 858)	1,387 (1,117 to 1,680)	462 (352 to 581)	925 (751 to 1,116)	64.1 (52.4 to 76.2)	235.2 (205.8 to 269.1)	30.7 (20.2 to 42)
		Eastern Sub-Saharan Africa	Incidence	Age-standardized	1 (0.8 to 1.2)	0.3 (0.3 to 0.4)	1.7 (1.3 to 2)	0.8 (0.6 to 0.9)	0.5 (0.4 to 0.6)	1.1 (0.9 to 1.4)	-21.3 (-26.5 to -16.3)	42.5 (31.3 to 54)	-32.7 (-37.9 to -27.9)
		Eastern Sub-Saharan Africa	Prevalence	All Ages	2,511 (2,157 to 2,919)	385 (324 to 460)	2,125 (1,793 to 2,501)	4,906 (4,167 to 5,746)	1,129 (947 to 1,351)	3,777 (3,151 to 4,528)	95.4 (85 to 105.4)	193 (176.4 to 212.4)	77.7 (64.4 to 89.9)
		Eastern Sub-Saharan Africa	Prevalence	Age-standardized	3.4 (2.9 to 4)	1 (0.9 to 1.2)	5.8 (4.9 to 6.9)	3.2 (2.7 to 3.8)	1.3 (1.1 to 1.6)	5.4 (4.5 to 6.5)	-5.7 (-10.3 to -1.3)	25.6 (18.9 to 33.3)	-7.8 (-13.6 to -2.1)
		Eastern Sub-Saharan Africa	Deaths	All Ages	320 (112 to 483)	53 (34 to 87)	267 (59 to 443)	417 (166 to 625)	84 (50 to 144)	333 (85 to 559)	30.3 (-4 to 69.3)	57.7 (20.1 to 113.6)	24.8 (-13 to 72.2)
		Eastern Sub-Saharan Africa	Deaths	Age-standardized	0.5 (0.2 to 0.7)	0.2 (0.1 to 0.3)	0.8 (0.2 to 1.3)	0.3 (0.1 to 0.4)	0.1 (0.1 to 0.2)	0.5 (0.1 to 0.8)	-39.1 (-54.1 to -20.6)	-29 (-45.2 to -5.2)	-38.9 (-55.9 to -16)
		Eastern Sub-Saharan Africa	DALYs	All Ages	9,106 (3,691 to 13,630)	1,569 (1,072 to 2,374)	7,537 (2,050 to 12,300)	11,955 (5,434 to 17,802)	2,374 (1,557 to 3,962)	9,582 (3,102 to 15,874)	31.3 (-3.7 to 67.6)	51.3 (14.9 to 116.2)	27.1 (-11.8 to 72.7)
		Eastern Sub-Saharan Africa	DALYs	Age-standardized	11.1 (4.3 to 16.6)	3.6 (2.3 to 5.7)	18.8 (4.9 to 30.5)	6.7 (3 to 9.9)	2.5 (1.6 to 4.1)	11.4 (3.6 to 18.6)	-39.4 (-55.1 to -22.4)	-30.5 (-46.3 to -7.3)	-39.3 (-57.1 to -17.4)
		Eastern Sub-Saharan Africa	YLLs	All Ages	8,699 (3,322 to 13,181)	1,508 (1,009 to 2,313)	7,191 (1,724 to 12,009)	11,162 (4,631 to 17,066)	2,195 (1,370 to 3,783)	8,967 (2,495 to 15,236)	28.3 (-7.8 to 65.6)	45.6 (8.5 to 112.3)	24.7 (-15.4 to 72.3)
		Eastern Sub-Saharan Africa	YLLs	Age-standardized	10.6 (3.7 to 16)	3.4 (2.2 to 5.5)	17.8 (4 to 29.7)	6.2 (2.4 to 9.4)	2.3 (1.4 to 3.9)	10.5 (2.7 to 17.7)	-41.2 (-57 to -23.6)	-33.2 (-49.3 to -8.9)	-41 (-58.9 to -19.5)
		Eastern Sub-Saharan Africa	YLDs	All Ages	407 (272 to 574)	61 (40 to 90)	346 (229 to 492)	794 (527 to 1,133)	179 (119 to 261)	615 (403 to 882)	95 (85 to 104.7)	192.3 (175.9 to 211.4)	77.8 (65.3 to 89.7)
		Eastern Sub-Saharan Africa	YLDs	Age-standardized	0.6 (0.4 to 0.8)	0.2 (0.1 to 0.2)	0.9 (0.6 to 1.3)	0.5 (0.3 to 0.7)	0.2 (0.1 to 0.3)	0.9 (0.6 to 1.2)	-6.3 (-10.6 to -2)	25.3 (18.7 to 32.8)	-8.3 (-13.8 to -2.9)
Sub-Saharan Africa	Eastern sub-Saharan Africa	Burundi	Incidence	All Ages	32 (26 to 39)	8 (6 to 10)	24 (19 to 30)	43 (34 to 52)	12 (9 to 16)	31 (24 to 38)	32 (15.9 to 48.2)	50 (24.3 to 83.8)	26.1 (7.1 to 44.1)
Sub-Saharan Africa	Eastern sub-Saharan Africa	Burundi	Incidence	Age-standardized	1.2 (1 to 1.5)	0.6 (0.4 to 0.7)	2.1 (1.6 to 2.5)	0.9 (0.7 to 1.1)	0.5 (0.4 to 0.6)	1.3 (1 to 1.5)	-28.2 (-35.3 to -21.2)	-13.6 (-23.9 to -0.2)	-38.7 (-46.1 to -31.1)
Sub-Saharan Africa	Eastern sub-Saharan Africa	Burundi	Prevalence	All Ages	91 (77 to 106)	18 (15 to 22)	73 (61 to 87)	154 (127 to 185)	28 (23 to 35)	126 (101 to 156)	69 (49.5 to 88.5)	58.7 (40.8 to 82.3)	71.5 (48.2 to 95.9)
Sub-Saharan Africa	Eastern sub-Saharan Africa	Burundi	Prevalence	Age-standardized	3.9 (3.3 to 4.5)	1.4 (1.1 to 1.7)	6.9 (5.8 to 8.1)	3.8 (3.1 to 4.6)	1.3 (1.1 to 1.7)	6.2 (5 to 7.7)	-1 (-11.9 to 10.1)	-1.8 (-12.5 to 11.7)	-9.6 (-20.8 to 2.1)
Sub-Saharan Africa	Eastern sub-Saharan Africa	Burundi	Deaths	All Ages	14 (6 to 24)	3 (1 to 5)	11 (3 to 21)	18 (6 to 32)	3 (1 to 6)	15 (3 to 30)	27.6 (-21.2 to 105.9)	13.7 (-38.6 to 101.5)	30.7 (-28.5 to 119.3)
Sub-Saharan Africa	Eastern sub-Saharan Africa	Burundi	Deaths	Age-standardized	0.6 (0.2 to 1)	0.2 (0.1 to 0.4)	1.1 (0.3 to 2.1)	0.4 (0.1 to 0.8)	0.1 (0.1 to 0.3)	0.7 (0.1 to 1.4)	-31.2 (-57.2 to 8.4)	-29.2 (-63.2 to 29.5)	-37.9 (-65.7 to 0.6)
Sub-Saharan Africa	Eastern sub-Saharan Africa	Burundi	DALYs	All Ages	385 (177 to 646)	74 (40 to 129)	311 (96 to 580)	515 (187 to 925)	86 (47 to 169)	429 (99 to 860)	33.9 (-18 to 108.9)	17.1 (-35.4 to 116.8)	37.9 (-20.8 to 131)
Sub-Saharan Africa	Eastern sub-Saharan Africa	Burundi	DALYs	Age-standardized	15.1 (6.5 to 25.7)	5 (2.7 to 8.8)	27.4 (7.9 to 50.9)	10.2 (3.7 to 18.1)	3.4 (1.7 to 6.6)	16.6 (3.8 to 32.6)	-32.3 (-57.3 to 6.3)	-32.1 (-62.3 to 20.5)	-39.5 (-64.2 to -0.5)
Sub-Saharan Africa	Eastern sub-Saharan Africa	Burundi	YLLs	All Ages	370 (164 to 632)	71 (37 to 127)	299 (86 to 570)	491 (164 to 904)	82 (42 to 164)	409 (80 to 837)	32.6 (-21.7 to 110.7)	15.4 (-38.2 to 119.6)	36.6 (-25.5 to 132.5)
Sub-Saharan Africa	Eastern sub-Saharan Africa	Burundi	YLLs	Age-standardized	14.5 (6 to 25)	4.7 (2.5 to 8.6)	26.3 (6.9 to 49.9)	9.6 (3 to 17.6)	3.2 (1.5 to 6.3)	15.6 (2.8 to 31.6)	-33.6 (-60.1 to 6.7)	-33.5 (-64.6 to 22.2)	-40.6 (-68.4 to 0)
Sub-Saharan Africa	Eastern sub-Saharan Africa	Burundi	YLDs	All Ages	15 (10 to 21)	3 (2 to 4)	12 (8 to 17)	25 (16 to 36)	4 (3 to 7)	20 (13 to 29)	67.6 (47.7 to 88.6)	58.7 (40.8 to 82.3)	69.7 (46.5 to 93.9)
Sub-Saharan Africa	Eastern sub-Saharan Africa	Burundi	YLDs	Age-standardized	0.6 (0.4 to 0.9)	0.2 (0.1 to 0.3)	1.1 (0.7 to 1.6)	0.6 (0.4 to 0.9)	0.2 (0.1 to 0.3)	1 (0.6 to 1.4)	-3.1 (-15 to 9.5)	-1.8 (-12.5 to 11.6)	-12.2 (-25.2 to 1.3)
Sub-Saharan Africa	Eastern sub-Saharan Africa	Comoros	Incidence	All Ages	2 (2 to 2)	0 (0 to 0)	2 (1 to 2)	4 (3 to 5)	1 (1 to 2)	3 (2 to 3)	103 (83.9 to 122.7)	229.3 (166.9 to 306.7)	71.6 (53.7 to 90.7)
Sub-Saharan Africa	Eastern sub-Saharan Africa	Comoros	Incidence	Age-standardized	0.8 (0.7 to 1)	0.3 (0.2 to 0.4)	1.4 (1.1 to 1.7)	0.8 (0.6 to 0.9)	0.5 (0.4 to 0.6)	1.2 (0.9 to 1.4)	-5.6 (-13.9 to 3.2)	45.9 (21.8 to 75.3)	-14.6 (-23 to -5.6)
Sub-Saharan Africa	Eastern sub-Saharan Africa	Comoros	Prevalence	All Ages	6 (6 to 8)	1 (1 to 1)	5 (5 to 6)	15 (13 to 18)	3 (3 to 4)	12 (10 to 14)	130.6 (110.6 to 150.2)	205.4 (166.2 to 250.4)	115.6 (93 to 139.2)
Sub-Saharan Africa	Eastern sub-Saharan Africa	Comoros	Prevalence	Age-standardized	3 (2.5 to 3.5)	1 (0.8 to 1.2)	5.1 (4.3 to 6.1)	3.2 (2.7 to 3.8)	1.3 (1.1 to 1.6)	5.7 (4.7 to 6.9)	8.5 (-0.7 to 17.6)	33.7 (17.7 to 52.2)	10.8 (-1.5 to 23.1)
Sub-Saharan Africa	Eastern sub-Saharan Africa	Comoros	Deaths	All Ages	1 (0 to 1)	0 (0 to 0)	1 (0 to 1)	1 (0 to 2)	0 (0 to 0)	1 (0 to 2)	32.3 (-21.4 to 158.3)	74.1 (-8.3 to 248.1)	22.3 (-34.1 to 160)
Sub-Saharan Africa	Eastern sub-Saharan Africa	Comoros	Deaths	Age-standardized	0.4 (0.2 to 0.7)	0.2 (0.1 to 0.3)	0.7 (0.2 to 1.2)	0.2 (0.1 to 0.4)	0.1 (0.1 to 0.2)	0.4 (0.1 to 0.8)	-39.9 (-63.9 to 12.4)	-25.4 (-60.5 to 44.2)	-40.5 (-67.5 to 16.5)
Sub-Saharan Africa	Eastern sub-Saharan Africa	Comoros	DALYs	All Ages	21 (9 to 35)	4 (2 to 7)	17 (5 to 29)	29 (13 to 47)	7 (4 to 12)	22 (7 to 41)	33.8 (-19.2 to 177.4)	66.7 (-11.3 to 262)	25.8 (-30.9 to 163.7)
Sub-Saharan Africa	Eastern sub-Saharan Africa	Comoros	DALYs	Age-standardized	9.3 (3.7 to 14.9)	3.4 (1.7 to 5.9)	15.6 (4.5 to 26.6)	5.8 (2.5 to 9.4)	2.6 (1.6 to 4.3)	9.6 (3.1 to 17.7)	-38.1 (-63 to 20.4)	-23.7 (-57.8 to 51.5)	-38.7 (-66.1 to 23.1)
Sub-Saharan Africa	Eastern sub-Saharan Africa	Comoros	YLLs	All Ages	20 (8 to 34)	4 (2 to 7)	16 (4 to 28)	26 (10 to 45)	6 (4 to 11)	20 (5 to 39)	29 (-24.8 to 182.7)	60.8 (-17.7 to 265.3)	21.2 (-36.2 to 173.2)
Sub-Saharan Africa	Eastern sub-Saharan Africa	Comoros	YLLs	Age-standardized	8.8 (3.3 to 14.5)	3.2 (1.5 to 5.7)	14.8 (3.6 to 25.8)	5.2 (2 to 8.8)	2.4 (1.4 to 4)	8.7 (2.3 to 17)	-40.6 (-65.1 to 21.3)	-26.4 (-61.9 to 53.2)	-41.4 (-68.6 to 26.5)
Sub-Saharan Africa	Eastern sub-Saharan Africa	Comoros	YLDs	All Ages	1 (1 to 2)	0 (0 to 0)	1 (1 to 1)	2 (2 to 3)	1 (0 to 1)	2 (1 to 3)	126.5 (107.1 to 149.8)	204.2 (165.3 to 248.6)	111.4 (89.9 to 137.3)
Sub-Saharan Africa	Eastern sub-Saharan Africa	Comoros	YLDs	Age-standardized	0.5 (0.3 to 0.7)	0.2 (0.1 to 0.2)	0.8 (0.6 to 1.2)	0.5 (0.3 to 0.7)	0.2 (0.1 to 0.3)	0.9 (0.6 to 1.3)	6.3 (-3.4 to 17.4)	33.1 (17.3 to 51.6)	8.1 (-3.9 to 22.6)
Sub-Saharan Africa	Eastern sub-Saharan Africa	Djibouti	Incidence	All Ages	1 (1 to 2)	0 (0 to 0)	1 (1 to 1)	5 (4 to 7)	1 (1 to 2)	4 (3 to 5)	341.6 (292.2 to 393.1)	561.6 (375.9 to 799.1)	294.7 (243.6 to 349.4)
Sub-Saharan Africa	Eastern sub-Saharan Africa	Djibouti	Incidence	Age-standardized	0.8 (0.6 to 0.9)	0.3 (0.2 to 0.3)	1.2 (1 to 1.5)	0.8 (0.7 to 1)	0.5 (0.3 to 0.6)	1.2 (0.9 to 1.4)	11.4 (2.4 to 21.5)	71.6 (38.1 to 114.1)	-6.3 (-14.6 to 3.2)
Sub-Saharan Africa	Eastern sub-Saharan Africa	Djibouti	Prevalence	All Ages	4 (3 to 4)	1 (0 to 1)	3 (3 to 4)	19 (16 to 23)	3 (3 to 4)	16 (13 to 19)	402.3 (357.1 to 452.5)	462.2 (377.3 to 556.8)	391.1 (337.5 to 448.8)
Sub-Saharan Africa	Eastern sub-Saharan Africa	Djibouti	Prevalence	Age-standardized	2.9 (2.5 to 3.5)	0.9 (0.7 to 1.1)	5.1 (4.2 to 6.1)	3.6 (3 to 4.3)	1.3 (1.1 to 1.5)	5.8 (4.7 to 7.1)	24.2 (13.5 to 35.1)	43.2 (24.7 to 62.2)	13.7 (2.5 to 25)
Sub-Saharan Africa	Eastern sub-Saharan Africa	Djibouti	Deaths	All Ages	0 (0 to 1)	0 (0 to 0)	0 (0 to 1)	1 (0 to 2)	0 (0 to 0)	1 (0 to 1)	175 (57.9 to 374.9)	186.8 (34.9 to 462.2)	172.1 (46.1 to 412.9)
Sub-Saharan Africa	Eastern sub-Saharan Africa	Djibouti	Deaths	Age-standardized	0.3 (0.1 to 0.5)	0.1 (0.1 to 0.2)	0.5 (0.2 to 0.9)	0.2 (0.1 to 0.3)	0.1 (0 to 0.2)	0.3 (0.1 to 0.5)	-34.6 (-61.4 to 11.8)	-25.4 (-64.9 to 44.6)	-40.9 (-67.8 to 14.2)
Sub-Saharan Africa	Eastern sub-Saharan Africa	Djibouti	DALYs	All Ages	11 (6 to 18)	2 (1 to 4)	9 (4 to 16)	30 (16 to 49)	6 (2 to 11)	24 (13 to 43)	166.7 (56.6 to 347)	165.5 (33.6 to 417.3)	167 (48 to 382.7)
Sub-Saharan Africa	Eastern sub-Saharan Africa	Djibouti	DALYs	Age-standardized	7.1 (3.4 to 11.3)	2.8 (1.5 to 4.6)	11.4 (4.8 to 19.2)	4.8 (2.6 to 7.8)	2.1 (0.8 to 3.8)	7.2 (3.8 to 12)	-32.4 (-59 to 11.3)	-25.2 (-60.9 to 38.4)	-37.4 (-64 to 11.3)
Sub-Saharan Africa	Eastern sub-Saharan Africa	Djibouti	YLLs	All Ages	11 (5 to 18)	2 (1 to 4)	9 (3 to 15)	27 (13 to 46)	5 (2 to 10)	22 (10 to 40)	153.3 (43.8 to 344.5)	152.3 (11 to 413)	153.5 (33.5 to 380.1)
Sub-Saharan Africa	Eastern sub-Saharan Africa	Djibouti	YLLs	Age-standardized	6.6 (2.9 to 10.9)	2.6 (1.3 to 4.4)	10.6 (4 to 18.4)	4.2 (2.1 to 7.1)	1.9 (0.6 to 3.6)	6.2 (2.9 to 11.1)	-36.3 (-63.1 to 10.1)	-28.8 (-66.7 to 37.9)	-41.2 (-67.8 to 11.8)
Sub-Saharan Africa	Eastern sub-Saharan Africa	Djibouti	YLDs	All Ages	1 (0 to 1)	0 (0 to 0)	1 (0 to 1)	3 (2 to 4)	1 (0 to 1)	3 (2 to 4)	397.6 (352.4 to 447.4)	460.2 (376.4 to 552.4)	386.3 (334.5 to 443.6)
Sub-Saharan Africa	Eastern sub-Saharan Africa	Djibouti	YLDs	Age-standardized	0.5 (0.3 to 0.7)	0.1 (0.1 to 0.2)	0.8 (0.6 to 1.2)	0.6 (0.4 to 0.8)	0.2 (0.1 to 0.3)	0.9 (0.6 to 1.3)	22 (10.7 to 34.1)	42.5 (24.3 to 61.6)	11.2 (-1.8 to 25.1)
Sub-Saharan Africa	Eastern sub-Saharan Africa	Eritrea	Incidence	All Ages	15 (11 to 18)	4 (3 to 5)	11 (8 to 14)	35 (27 to 43)	14 (10 to 18)	20 (16 to 25)	133.4 (107.6 to 160.5)	258.4 (197 to 350.9)	88.2 (59.1 to 117.7)
Sub-Saharan Africa	Eastern sub-Saharan Africa	Eritrea	Incidence	Age-standardized	1.2 (1 to 1.5)	0.6 (0.5 to 0.7)	2.1 (1.6 to 2.5)	1.1 (0.9 to 1.3)	0.8 (0.6 to 1)	1.6 (1.2 to 1.9)	-7.9 (-15.4 to -0.5)	32 (15.5 to 53.9)	-24.1 (-31.1 to -16.4)
Sub-Saharan Africa	Eastern sub-Saharan Africa	Eritrea	Prevalence	All Ages	33 (28 to 39)	8 (6 to 10)	26 (21 to 31)	89 (75 to 105)	28 (22 to 34)	61 (51 to 73)	166.3 (142.1 to 190.3)	259.6 (215.3 to 316.3)	138.5 (111.1 to 168.5)
Sub-Saharan Africa	Eastern sub-Saharan Africa	Eritrea	Prevalence	Age-standardized	3.2 (2.8 to 3.8)	1.3 (1.1 to 1.6)	6.4 (5.4 to 7.6)	3.5 (2.9 to 4.1)	1.8 (1.4 to 2.1)	6.3 (5.2 to 7.6)	7.6 (-0.3 to 16.8)	34.8 (20.9 to 51)	-0.3 (-10.4 to 10.3)
Sub-Saharan Africa	Eastern sub-Saharan Africa	Eritrea	Deaths	All Ages	5 (2 to 8)	1 (0 to 2)	4 (1 to 7)	8 (4 to 14)	2 (1 to 3)	6 (2 to 12)	62.2 (1.3 to 186.5)	123.9 (17.2 to 368.5)	49 (-14.8 to 188.7)
Sub-Saharan Africa	Eastern sub-Saharan Africa	Eritrea	Deaths	Age-standardized	0.5 (0.2 to 0.8)	0.2 (0.1 to 0.4)	1 (0.3 to 1.9)	0.3 (0.1 to 0.5)	0.1 (0.1 to 0.3)	0.6 (0.2 to 1.1)	-37.3 (-60.9 to 6.2)	-14.4 (-56.1 to 83.6)	-44.3 (-67.7 to 7.6)
Sub-Saharan Africa	Eastern sub-Saharan Africa	Eritrea	DALYs	All Ages	153 (66 to 261)	26 (13 to 49)	126 (42 to 238)	241 (125 to 424)	55 (29 to 98)	187 (81 to 364)	58 (-2.4 to 176.4)	107.7 (14.9 to 296.3)	47.6 (-14.3 to 191.1)
Sub-Saharan Africa	Eastern sub-Saharan Africa	Eritrea	DALYs	Age-standardized	12.8 (5.3 to 21.4)	4 (2 to 8.1)	25.5 (8 to 46.8)	7.8 (4.1 to 13.5)	3.3 (1.8 to 5.9)	14.1 (6 to 26.6)	-39 (-61.4 to 3.2)	-17 (-54.1 to 64.8)	-44.8 (-67.4 to 2.1)
Sub-Saharan Africa	Eastern sub-Saharan Africa	Eritrea	YLLs	All Ages	147 (61 to 255)	25 (12 to 48)	122 (38 to 233)	227 (109 to 411)	50 (25 to 93)	177 (71 to 355)	54 (-6.5 to 176.8)	100.4 (5.3 to 299.2)	44.5 (-18.1 to 194.8)
Sub-Saharan Africa	Eastern sub-Saharan Africa	Eritrea	YLLs	Age-standardized	12.3 (4.8 to 20.9)	3.8 (1.8 to 7.9)	24.5 (6.8 to 45.7)	7.3 (3.5 to 12.9)	3 (1.5 to 5.7)	13.1 (5.1 to 25.8)	-40.9 (-63.4 to 3.3)	-19.8 (-58.1 to 67.2)	-46.6 (-69.3 to 2.2)
Sub-Saharan Africa	Eastern sub-Saharan Africa	Eritrea	YLDs	All Ages	5 (4 to 8)	1 (1 to 2)	4 (3 to 6)	14 (9 to 20)	4 (3 to 6)	10 (7 to 14)	165.7 (142.1 to 190.5)	259 (215 to 315.5)	138.5 (111.1 to 169.4)
Sub-Saharan Africa	Eastern sub-Saharan Africa	Eritrea	YLDs	Age-standardized	0.5 (0.3 to 0.7)	0.2 (0.1 to 0.3)	1 (0.7 to 1.5)	0.6 (0.4 to 0.8)	0.3 (0.2 to 0.4)	1 (0.7 to 1.5)	6.6 (-1.8 to 16)	34.6 (20.6 to 50.5)	-1.9 (-12.9 to 10.4)
Sub-Saharan Africa	Eastern sub-Saharan Africa	Ethiopia	Incidence	All Ages	375 (292 to 462)	41 (30 to 52)	334 (260 to 414)	361 (291 to 442)	105 (78 to 135)	256 (207 to 312)	-3.7 (-15.4 to 9.6)	158.2 (129.6 to 193.1)	-23.4 (-33.7 to -12)
Sub-Saharan Africa	Eastern sub-Saharan Africa	Ethiopia	Incidence	Age-standardized	1.5 (1.2 to 1.9)	0.4 (0.3 to 0.5)	2.7 (2.1 to 3.3)	0.8 (0.6 to 1)	0.4 (0.3 to 0.5)	1.1 (0.9 to 1.4)	-48.9 (-54.6 to -42.9)	15.2 (6.2 to 25.4)	-57.7 (-62.7 to -52.3)
Sub-Saharan Africa	Eastern sub-Saharan Africa	Ethiopia	Prevalence	All Ages	895 (758 to 1,056)	102 (84 to 125)	793 (667 to 939)	1,180 (1,002 to 1,399)	253 (209 to 308)	927 (774 to 1,118)	31.9 (16.7 to 46.9)	148 (131.6 to 167.8)	16.9 (2.1 to 32.6)
Sub-Saharan Africa	Eastern sub-Saharan Africa	Ethiopia	Prevalence	Age-standardized	4.2 (3.5 to 4.9)	1 (0.9 to 1.3)	7 (6 to 8.3)	2.9 (2.4 to 3.4)	1.2 (1 to 1.4)	4.5 (3.7 to 5.4)	-30.6 (-37.3 to -23.7)	12.2 (5.5 to 20.3)	-36.4 (-43.6 to -29)
Sub-Saharan Africa	Eastern sub-Saharan Africa	Ethiopia	Deaths	All Ages	109 (33 to 197)	14 (8 to 26)	95 (17 to 187)	85 (24 to 141)	15 (9 to 28)	70 (11 to 131)	-22.1 (-47.3 to 19)	8 (-32.9 to 93.3)	-26.5 (-54.3 to 17.8)
Sub-Saharan Africa	Eastern sub-Saharan Africa	Ethiopia	Deaths	Age-standardized	0.6 (0.2 to 1)	0.2 (0.1 to 0.3)	1 (0.2 to 1.9)	0.2 (0.1 to 0.4)	0.1 (0 to 0.2)	0.4 (0.1 to 0.7)	-59.7 (-72.5 to -39.3)	-47.2 (-67 to -11.3)	-61.9 (-76.1 to -39.1)
Sub-Saharan Africa	Eastern sub-Saharan Africa	Ethiopia	DALYs	All Ages	3,208 (1,103 to 5,689)	439 (251 to 765)	2,769 (632 to 5,286)	2,268 (802 to 3,628)	418 (268 to 732)	1,849 (429 to 3,324)	-29.3 (-51.4 to 5.1)	-4.8 (-39 to 83.7)	-33.2 (-56.7 to 4.7)
Sub-Saharan Africa	Eastern sub-Saharan Africa	Ethiopia	DALYs	Age-standardized	14.5 (4.8 to 25.5)	3.9 (2.3 to 7.1)	24.1 (5.2 to 45.8)	5.2 (1.8 to 8.4)	1.9 (1.2 to 3.3)	8.4 (1.9 to 15.1)	-63.7 (-75.1 to -46.3)	-51.9 (-68.9 to -16.6)	-65 (-77.4 to -45.7)
Sub-Saharan Africa	Eastern sub-Saharan Africa	Ethiopia	YLLs	All Ages	3,064 (976 to 5,550)	423 (233 to 753)	2,641 (502 to 5,156)	2,075 (612 to 3,491)	378 (226 to 694)	1,697 (288 to 3,170)	-32.3 (-54.2 to 2.9)	-10.6 (-45.3 to 80.7)	-35.8 (-60.4 to 3)
Sub-Saharan Africa	Eastern sub-Saharan Africa	Ethiopia	YLLs	Age-standardized	13.8 (4.2 to 24.9)	3.7 (2.1 to 6.9)	23 (4.1 to 44.8)	4.8 (1.3 to 8)	1.7 (1 to 3.1)	7.7 (1.2 to 14.4)	-65.4 (-76.8 to -47.4)	-54.7 (-71.7 to -18)	-66.5 (-79.3 to -46.6)
Sub-Saharan Africa	Eastern sub-Saharan Africa	Ethiopia	YLDs	All Ages	143 (96 to 203)	16 (10 to 24)	127 (84 to 181)	192 (127 to 271)	40 (26 to 59)	152 (100 to 219)	34 (19.2 to 48.6)	148.1 (131.8 to 167.8)	19.5 (5 to 34.7)
Sub-Saharan Africa	Eastern sub-Saharan Africa	Ethiopia	YLDs	Age-standardized	0.7 (0.4 to 0.9)	0.2 (0.1 to 0.2)	1.1 (0.8 to 1.6)	0.5 (0.3 to 0.7)	0.2 (0.1 to 0.3)	0.7 (0.5 to 1.1)	-29.6 (-36.2 to -22.8)	12.3 (5.5 to 20.3)	-35.1 (-42.3 to -27.7)
Sub-Saharan Africa	Eastern sub-Saharan Africa	Kenya	Incidence	All Ages	76 (62 to 91)	17 (13 to 21)	60 (49 to 71)	221 (179 to 265)	96 (74 to 118)	125 (103 to 149)	189 (177 to 201)	470.7 (394.3 to 557.3)	109.9 (98.3 to 121.6)
Sub-Saharan Africa	Eastern sub-Saharan Africa	Kenya	Incidence	Age-standardized	0.8 (0.7 to 1)	0.3 (0.3 to 0.4)	1.3 (1.1 to 1.6)	0.9 (0.7 to 1.1)	0.7 (0.5 to 0.8)	1.1 (0.9 to 1.3)	7.1 (3.3 to 10.8)	98.6 (74.9 to 124.7)	-16.3 (-20 to -13)
Sub-Saharan Africa	Eastern sub-Saharan Africa	Kenya	Prevalence	All Ages	297 (255 to 348)	55 (45 to 65)	243 (205 to 289)	770 (659 to 901)	216 (181 to 259)	553 (462 to 661)	158.9 (150.7 to 168)	295.9 (258.8 to 342.8)	128.1 (118.5 to 136.8)
Sub-Saharan Africa	Eastern sub-Saharan Africa	Kenya	Prevalence	Age-standardized	3.8 (3.2 to 4.4)	1.3 (1.1 to 1.6)	6.3 (5.3 to 7.6)	3.6 (3.1 to 4.3)	1.8 (1.5 to 2.1)	5.9 (4.9 to 7.1)	-3.3 (-6.1 to -0.3)	34 (22.4 to 49.4)	-6.3 (-9.3 to -3.3)
Sub-Saharan Africa	Eastern sub-Saharan Africa	Kenya	Deaths	All Ages	20 (10 to 28)	5 (3 to 10)	14 (3 to 23)	47 (26 to 68)	12 (6 to 24)	35 (14 to 57)	139.9 (77.7 to 233)	124.1 (59.8 to 211)	145.5 (66.3 to 357.1)
Sub-Saharan Africa	Eastern sub-Saharan Africa	Kenya	Deaths	Age-standardized	0.3 (0.1 to 0.4)	0.1 (0.1 to 0.3)	0.4 (0.1 to 0.6)	0.2 (0.1 to 0.3)	0.1 (0.1 to 0.2)	0.4 (0.1 to 0.6)	-9.9 (-32.2 to 23)	-16.4 (-39.4 to 12.9)	-2.2 (-31.8 to 79.3)
Sub-Saharan Africa	Eastern sub-Saharan Africa	Kenya	DALYs	All Ages	542 (301 to 754)	141 (85 to 259)	400 (127 to 624)	1,371 (828 to 1,968)	327 (192 to 625)	1,044 (472 to 1,651)	153.1 (89.7 to 243.5)	131.9 (66 to 222.4)	160.6 (82.2 to 319.2)
Sub-Saharan Africa	Eastern sub-Saharan Africa	Kenya	DALYs	Age-standardized	6.2 (3.4 to 8.7)	3 (1.8 to 5.4)	9.5 (3 to 14.7)	5.8 (3.4 to 8.2)	2.6 (1.5 to 4.9)	9.4 (4.2 to 14.7)	-6.8 (-29.3 to 25.6)	-14.5 (-37.6 to 16.9)	-1.4 (-30 to 55.9)
Sub-Saharan Africa	Eastern sub-Saharan Africa	Kenya	YLLs	All Ages	494 (254 to 703)	132 (75 to 250)	361 (88 to 586)	1,248 (696 to 1,834)	293 (162 to 593)	955 (387 to 1,554)	152.7 (83.8 to 251)	121.3 (53 to 215.5)	164.2 (77.3 to 387)
Sub-Saharan Africa	Eastern sub-Saharan Africa	Kenya	YLLs	Age-standardized	5.6 (2.7 to 8.1)	2.8 (1.6 to 5.3)	8.5 (2 to 13.8)	5.2 (2.8 to 7.6)	2.3 (1.3 to 4.7)	8.4 (3.2 to 13.6)	-7.1 (-31.6 to 29.7)	-18.1 (-42.1 to 15.4)	-0.8 (-32.2 to 81.9)
Sub-Saharan Africa	Eastern sub-Saharan Africa	Kenya	YLDs	All Ages	48 (32 to 67)	9 (6 to 13)	39 (26 to 56)	123 (82 to 174)	34 (23 to 49)	89 (58 to 127)	157.3 (148.7 to 166.5)	293.5 (257.2 to 339.2)	127.1 (117.3 to 136.5)
Sub-Saharan Africa	Eastern sub-Saharan Africa	Kenya	YLDs	Age-standardized	0.6 (0.4 to 0.9)	0.2 (0.1 to 0.3)	1 (0.7 to 1.5)	0.6 (0.4 to 0.8)	0.3 (0.2 to 0.4)	0.9 (0.6 to 1.3)	-3.9 (-7.1 to -0.8)	33.3 (21.6 to 48.3)	-7.2 (-10.6 to -3.9)
Sub-Saharan Africa	Eastern sub-Saharan Africa	Madagascar	Incidence	All Ages	42 (34 to 51)	7 (5 to 10)	35 (28 to 42)	86 (69 to 105)	23 (17 to 31)	63 (50 to 77)	107.2 (85.8 to 133.2)	224.1 (152.8 to 313)	83 (62 to 105.3)
Sub-Saharan Africa	Eastern sub-Saharan Africa	Madagascar	Incidence	Age-standardized	0.8 (0.7 to 1)	0.3 (0.2 to 0.3)	1.3 (1 to 1.6)	0.8 (0.6 to 0.9)	0.4 (0.3 to 0.5)	1.2 (1 to 1.4)	-1 (-9.5 to 8.1)	46.3 (21.8 to 76.6)	-6.9 (-15.6 to 1.7)
Sub-Saharan Africa	Eastern sub-Saharan Africa	Madagascar	Prevalence	All Ages	162 (134 to 193)	24 (19 to 30)	138 (111 to 167)	346 (282 to 420)	66 (53 to 83)	280 (221 to 351)	113.8 (92.8 to 135.3)	175.3 (135.7 to 221.5)	103.1 (80.1 to 125.6)
Sub-Saharan Africa	Eastern sub-Saharan Africa	Madagascar	Prevalence	Age-standardized	3.4 (2.8 to 4.1)	1 (0.8 to 1.3)	5.6 (4.6 to 6.8)	3.8 (3.1 to 4.6)	1.3 (1.1 to 1.7)	6.6 (5.3 to 8.2)	11.8 (1.1 to 22.1)	32.1 (14.8 to 54.3)	16.7 (3.2 to 29.6)
Sub-Saharan Africa	Eastern sub-Saharan Africa	Madagascar	Deaths	All Ages	25 (10 to 34)	5 (3 to 9)	19 (5 to 29)	42 (16 to 71)	10 (5 to 19)	32 (8 to 61)	70.1 (10.4 to 157.6)	89 (7.6 to 222.2)	64.8 (-0.2 to 169.3)
Sub-Saharan Africa	Eastern sub-Saharan Africa	Madagascar	Deaths	Age-standardized	0.5 (0.2 to 0.7)	0.2 (0.1 to 0.4)	0.8 (0.2 to 1.2)	0.4 (0.2 to 0.7)	0.2 (0.1 to 0.4)	0.7 (0.2 to 1.3)	-14.8 (-43.9 to 27.3)	-5.5 (-47.4 to 61.8)	-12.7 (-45.7 to 39.9)
Sub-Saharan Africa	Eastern sub-Saharan Africa	Madagascar	DALYs	All Ages	737 (372 to 1,001)	185 (114 to 286)	552 (182 to 824)	1,296 (564 to 2,117)	333 (185 to 590)	963 (282 to 1,826)	75.8 (13.1 to 167.5)	79.5 (1.4 to 199.8)	74.5 (5.9 to 180)
Sub-Saharan Africa	Eastern sub-Saharan Africa	Madagascar	DALYs	Age-standardized	12.3 (5.5 to 17.1)	5.4 (3.3 to 9.2)	18.8 (5.6 to 28)	10.3 (4.1 to 16.9)	4.8 (2.6 to 8.4)	16.2 (4.6 to 30.2)	-16.3 (-44.4 to 24.4)	-12.5 (-49.3 to 44.2)	-14.2 (-46.3 to 36.7)
Sub-Saharan Africa	Eastern sub-Saharan Africa	Madagascar	YLLs	All Ages	711 (349 to 979)	182 (111 to 282)	529 (160 to 802)	1,240 (513 to 2,076)	322 (176 to 577)	918 (241 to 1,778)	74.5 (9 to 171)	77.5 (-2.3 to 200.2)	73.5 (1.9 to 183.2)
Sub-Saharan Africa	Eastern sub-Saharan Africa	Madagascar	YLLs	Age-standardized	11.7 (5 to 16.4)	5.3 (3.2 to 9)	17.9 (4.7 to 27)	9.7 (3.6 to 16.4)	4.5 (2.4 to 8.1)	15.1 (3.7 to 29.2)	-17.5 (-46.6 to 25.1)	-13.9 (-51 to 44.8)	-15.6 (-49 to 37.6)
Sub-Saharan Africa	Eastern sub-Saharan Africa	Madagascar	YLDs	All Ages	26 (17 to 38)	4 (2 to 6)	23 (15 to 33)	56 (36 to 81)	10 (7 to 16)	45 (29 to 66)	110.7 (88.7 to 134.9)	174.8 (135.5 to 219.9)	99.9 (75.7 to 125.9)
Sub-Saharan Africa	Eastern sub-Saharan Africa	Madagascar	YLDs	Age-standardized	0.6 (0.4 to 0.8)	0.2 (0.1 to 0.2)	0.9 (0.6 to 1.3)	0.6 (0.4 to 0.9)	0.2 (0.1 to 0.3)	1 (0.6 to 1.5)	8.9 (-3.9 to 23.5)	31.8 (14.5 to 53.2)	13 (-2.9 to 31.2)
Sub-Saharan Africa	Eastern sub-Saharan Africa	Malawi	Incidence	All Ages	26 (21 to 32)	5 (4 to 7)	21 (17 to 25)	53 (43 to 64)	17 (12 to 22)	36 (29 to 44)	101.5 (80.9 to 125.4)	210.3 (145.8 to 298)	73.4 (53.9 to 95.4)
Sub-Saharan Africa	Eastern sub-Saharan Africa	Malawi	Incidence	Age-standardized	0.7 (0.5 to 0.8)	0.2 (0.2 to 0.3)	1.1 (0.9 to 1.3)	0.7 (0.6 to 0.8)	0.4 (0.3 to 0.5)	1.1 (0.9 to 1.3)	6.6 (-2.6 to 16)	52 (27.2 to 86)	-0.8 (-10.3 to 8.8)
Sub-Saharan Africa	Eastern sub-Saharan Africa	Malawi	Prevalence	All Ages	97 (81 to 114)	19 (16 to 23)	78 (64 to 94)	216 (180 to 256)	49 (41 to 60)	166 (134 to 204)	123.4 (100.8 to 147.1)	160 (127.9 to 200.4)	114.5 (87.4 to 143.4)
Sub-Saharan Africa	Eastern sub-Saharan Africa	Malawi	Prevalence	Age-standardized	2.7 (2.2 to 3.1)	1 (0.8 to 1.2)	4.7 (3.9 to 5.7)	3.2 (2.6 to 3.8)	1.2 (1 to 1.5)	6 (4.8 to 7.3)	18.7 (6.9 to 31.4)	27 (12.3 to 46.4)	27.3 (10.5 to 45)
Sub-Saharan Africa	Eastern sub-Saharan Africa	Malawi	Deaths	All Ages	13 (4 to 19)	3 (1 to 5)	10 (2 to 17)	18 (7 to 30)	4 (2 to 6)	15 (4 to 27)	45.8 (-4.4 to 120.3)	38.7 (-22.6 to 145.2)	47.7 (-9.7 to 141.2)
Sub-Saharan Africa	Eastern sub-Saharan Africa	Malawi	Deaths	Age-standardized	0.4 (0.1 to 0.6)	0.1 (0.1 to 0.3)	0.6 (0.1 to 1)	0.3 (0.1 to 0.4)	0.1 (0 to 0.2)	0.5 (0.1 to 0.9)	-24.8 (-49.5 to 11.4)	-31.1 (-62.1 to 20.5)	-16.5 (-47.6 to 33.7)
Sub-Saharan Africa	Eastern sub-Saharan Africa	Malawi	DALYs	All Ages	347 (139 to 536)	73 (44 to 119)	274 (72 to 455)	514 (218 to 855)	94 (51 to 162)	420 (138 to 767)	47.9 (-2.8 to 119.8)	27.9 (-26 to 120.7)	53.2 (-6 to 141.7)
Sub-Saharan Africa	Eastern sub-Saharan Africa	Malawi	DALYs	Age-standardized	8.4 (3.2 to 12.7)	3.2 (1.9 to 5.4)	14.3 (3.6 to 23.4)	6.6 (2.7 to 10.8)	2.2 (1.2 to 3.7)	12.2 (3.9 to 21.6)	-21.8 (-47.4 to 14.8)	-32.8 (-60.7 to 14.3)	-15 (-46.2 to 31.6)
Sub-Saharan Africa	Eastern sub-Saharan Africa	Malawi	YLLs	All Ages	331 (122 to 516)	70 (41 to 116)	261 (61 to 441)	479 (184 to 821)	86 (44 to 153)	393 (112 to 737)	44.5 (-7.6 to 120.5)	22.3 (-32.9 to 118.9)	50.4 (-9.6 to 145.3)
Sub-Saharan Africa	Eastern sub-Saharan Africa	Malawi	YLLs	Age-standardized	7.9 (2.8 to 12.3)	3.1 (1.7 to 5.3)	13.5 (2.9 to 22.6)	6 (2.2 to 10.2)	2 (1 to 3.5)	11.2 (2.9 to 20.5)	-23.9 (-50.5 to 15.1)	-35.8 (-64 to 13.1)	-17.2 (-49.2 to 32.9)
Sub-Saharan Africa	Eastern sub-Saharan Africa	Malawi	YLDs	All Ages	16 (11 to 23)	3 (2 to 4)	13 (8 to 19)	35 (23 to 50)	8 (5 to 12)	27 (17 to 39)	119.1 (97.5 to 143.4)	158.8 (127.1 to 199)	109.8 (83.8 to 138.1)
Sub-Saharan Africa	Eastern sub-Saharan Africa	Malawi	YLDs	Age-standardized	0.4 (0.3 to 0.6)	0.2 (0.1 to 0.2)	0.8 (0.5 to 1.1)	0.5 (0.3 to 0.7)	0.2 (0.1 to 0.3)	1 (0.6 to 1.4)	15.8 (5 to 29.2)	26.4 (11.7 to 45.5)	23.1 (8.2 to 41.8)
Sub-Saharan Africa	Eastern sub-Saharan Africa	Mozambique	Incidence	All Ages	45 (36 to 56)	9 (6 to 12)	37 (29 to 45)	92 (73 to 113)	30 (22 to 39)	63 (49 to 78)	102.4 (81.9 to 126.8)	230.1 (156.3 to 322.7)	71.1 (52.4 to 93.5)
Sub-Saharan Africa	Eastern sub-Saharan Africa	Mozambique	Incidence	Age-standardized	0.7 (0.6 to 0.9)	0.3 (0.2 to 0.4)	1.2 (1 to 1.5)	0.8 (0.6 to 1)	0.4 (0.3 to 0.5)	1.2 (1 to 1.5)	7.9 (-1.3 to 18.1)	57.4 (26.6 to 91)	-0.3 (-9.6 to 11.3)
Sub-Saharan Africa	Eastern sub-Saharan Africa	Mozambique	Prevalence	All Ages	153 (128 to 181)	26 (21 to 32)	128 (104 to 154)	343 (278 to 427)	73 (60 to 89)	270 (211 to 350)	123.8 (95.7 to 159.7)	184.2 (143.8 to 232.8)	111.7 (81.6 to 154.5)
Sub-Saharan Africa	Eastern sub-Saharan Africa	Mozambique	Prevalence	Age-standardized	2.7 (2.3 to 3.2)	0.9 (0.7 to 1.1)	4.9 (4 to 5.9)	3.4 (2.7 to 4.2)	1.2 (1 to 1.5)	6.5 (5.1 to 8.4)	24.5 (9.5 to 44.1)	40.7 (21.8 to 62.8)	33.3 (14.4 to 60.1)
Sub-Saharan Africa	Eastern sub-Saharan Africa	Mozambique	Deaths	All Ages	20 (4 to 35)	3 (1 to 5)	17 (2 to 32)	31 (12 to 53)	5 (2 to 9)	27 (8 to 49)	58 (-2 to 225.3)	69.7 (-5.9 to 223.1)	56.1 (-10.2 to 286.2)
Sub-Saharan Africa	Eastern sub-Saharan Africa	Mozambique	Deaths	Age-standardized	0.4 (0.1 to 0.6)	0.1 (0.1 to 0.2)	0.7 (0.1 to 1.2)	0.3 (0.1 to 0.5)	0.1 (0 to 0.2)	0.6 (0.2 to 1.1)	-17 (-49 to 61.4)	-16 (-55.4 to 58.6)	-10.4 (-47.5 to 109.5)
Sub-Saharan Africa	Eastern sub-Saharan Africa	Mozambique	DALYs	All Ages	537 (138 to 949)	74 (39 to 138)	462 (87 to 866)	915 (396 to 1,546)	129 (72 to 234)	786 (284 to 1,430)	70.5 (5.2 to 243.7)	73.7 (3.9 to 203.3)	70 (2.5 to 286.5)
Sub-Saharan Africa	Eastern sub-Saharan Africa	Mozambique	DALYs	Age-standardized	8.5 (2.2 to 14.8)	2.2 (1.2 to 4.2)	15.5 (2.9 to 28.7)	7.6 (3.2 to 12.6)	1.9 (1 to 3.5)	14.8 (5 to 26)	-10.9 (-43.3 to 72.3)	-14 (-48.6 to 50.4)	-5.1 (-42.9 to 103.1)
Sub-Saharan Africa	Eastern sub-Saharan Africa	Mozambique	YLLs	All Ages	511 (113 to 919)	70 (35 to 134)	441 (65 to 847)	859 (346 to 1,488)	117 (60 to 222)	742 (233 to 1,389)	68.1 (1.5 to 263.7)	67.3 (-5 to 204.8)	68.2 (-1.6 to 342.2)
Sub-Saharan Africa	Eastern sub-Saharan Africa	Mozambique	YLLs	Age-standardized	8.1 (1.8 to 14.3)	2.1 (1 to 4.1)	14.7 (2 to 28.2)	7.1 (2.7 to 12.2)	1.7 (0.9 to 3.3)	13.7 (4 to 25.1)	-12.7 (-45.7 to 80.4)	-17.5 (-54.2 to 52.3)	-7 (-45.6 to 125.8)
Sub-Saharan Africa	Eastern sub-Saharan Africa	Mozambique	YLDs	All Ages	25 (17 to 36)	4 (3 to 6)	21 (14 to 31)	56 (36 to 81)	12 (7 to 17)	44 (28 to 66)	120.3 (93.5 to 153.8)	182.9 (143 to 231.2)	108.2 (78.7 to 148.6)
Sub-Saharan Africa	Eastern sub-Saharan Africa	Mozambique	YLDs	Age-standardized	0.4 (0.3 to 0.6)	0.1 (0.1 to 0.2)	0.8 (0.5 to 1.2)	0.5 (0.4 to 0.8)	0.2 (0.1 to 0.3)	1 (0.7 to 1.5)	21.9 (7.4 to 41.1)	40 (21.6 to 61.5)	29.6 (10.4 to 56.1)
Sub-Saharan Africa	Eastern sub-Saharan Africa	Rwanda	Incidence	All Ages	34 (27 to 41)	8 (6 to 10)	26 (21 to 32)	47 (37 to 57)	16 (12 to 21)	31 (24 to 39)	39.3 (21.4 to 57.5)	107.4 (76.4 to 148.7)	19.3 (0.3 to 39.6)
Sub-Saharan Africa	Eastern sub-Saharan Africa	Rwanda	Incidence	Age-standardized	1.1 (0.8 to 1.3)	0.4 (0.3 to 0.6)	1.8 (1.5 to 2.2)	0.7 (0.6 to 0.9)	0.4 (0.3 to 0.6)	1.2 (0.9 to 1.5)	-29.9 (-37.5 to -22.5)	-2.4 (-15.4 to 12.6)	-35.2 (-43.5 to -25.7)
Sub-Saharan Africa	Eastern sub-Saharan Africa	Rwanda	Prevalence	All Ages	100 (82 to 119)	17 (14 to 21)	82 (66 to 101)	177 (144 to 228)	39 (32 to 48)	138 (107 to 186)	77.6 (53.5 to 101.9)	127.7 (101.4 to 158.1)	67.1 (39.2 to 96.2)
Sub-Saharan Africa	Eastern sub-Saharan Africa	Rwanda	Prevalence	Age-standardized	3.5 (2.9 to 4.2)	1.1 (0.9 to 1.3)	6.6 (5.2 to 8)	3.3 (2.6 to 4.2)	1.2 (1 to 1.4)	6.5 (5 to 8.8)	-7.1 (-20.1 to 5.8)	9.4 (-3.9 to 24.2)	-1.2 (-18.3 to 17.4)
Sub-Saharan Africa	Eastern sub-Saharan Africa	Rwanda	Deaths	All Ages	16 (7 to 26)	3 (2 to 6)	13 (4 to 23)	15 (6 to 23)	4 (2 to 8)	11 (3 to 20)	-10 (-43.4 to 43.3)	13 (-38.6 to 130.8)	-15.9 (-50.1 to 40.6)
Sub-Saharan Africa	Eastern sub-Saharan Africa	Rwanda	Deaths	Age-standardized	0.6 (0.2 to 0.9)	0.2 (0.1 to 0.4)	1.1 (0.3 to 1.9)	0.3 (0.1 to 0.4)	0.1 (0.1 to 0.3)	0.5 (0.1 to 0.9)	-54.1 (-70.4 to -26.4)	-43.8 (-69.5 to 8.3)	-53.3 (-71.8 to -23.6)
Sub-Saharan Africa	Eastern sub-Saharan Africa	Rwanda	DALYs	All Ages	469 (220 to 748)	101 (52 to 174)	368 (127 to 645)	415 (191 to 646)	100 (49 to 210)	315 (97 to 559)	-11.5 (-44.1 to 39.2)	-0.4 (-46.1 to 113.6)	-14.5 (-49.7 to 39.7)
Sub-Saharan Africa	Eastern sub-Saharan Africa	Rwanda	DALYs	Age-standardized	14.5 (6.5 to 23)	5.3 (2.7 to 9.7)	26 (8.4 to 45.5)	6.4 (2.9 to 9.7)	2.7 (1.3 to 5.4)	11.6 (3.7 to 19.8)	-55.9 (-71.8 to -32)	-49.2 (-71.5 to 3.8)	-55.3 (-73.1 to -29.2)
Sub-Saharan Africa	Eastern sub-Saharan Africa	Rwanda	YLLs	All Ages	453 (206 to 734)	98 (50 to 172)	355 (114 to 632)	387 (164 to 612)	94 (43 to 204)	293 (75 to 535)	-14.6 (-47.7 to 37.9)	-3.9 (-49.3 to 112.9)	-17.5 (-52.5 to 39.2)
Sub-Saharan Africa	Eastern sub-Saharan Africa	Rwanda	YLLs	Age-standardized	14 (5.9 to 22.5)	5.1 (2.5 to 9.5)	25 (7.4 to 44.5)	5.9 (2.4 to 9.2)	2.5 (1.1 to 5.3)	10.6 (2.6 to 18.9)	-57.8 (-73.5 to -32.9)	-51.2 (-73.6 to 3.1)	-57.4 (-75.1 to -29.8)
Sub-Saharan Africa	Eastern sub-Saharan Africa	Rwanda	YLDs	All Ages	16 (11 to 23)	3 (2 to 4)	13 (9 to 19)	28 (18 to 41)	6 (4 to 9)	22 (14 to 33)	74.8 (50.8 to 101.6)	127.6 (101.5 to 157.5)	64.1 (36.6 to 96)
Sub-Saharan Africa	Eastern sub-Saharan Africa	Rwanda	YLDs	Age-standardized	0.6 (0.4 to 0.8)	0.2 (0.1 to 0.3)	1.1 (0.7 to 1.5)	0.5 (0.3 to 0.8)	0.2 (0.1 to 0.3)	1 (0.6 to 1.5)	-9.6 (-23 to 5.9)	9.3 (-3.8 to 23.9)	-5 (-22.7 to 16.8)
Sub-Saharan Africa	Eastern sub-Saharan Africa	Somalia	Incidence	All Ages	27 (21 to 32)	5 (4 to 7)	21 (17 to 26)	68 (53 to 83)	25 (18 to 32)	43 (34 to 54)	153.7 (123 to 186.5)	350.1 (237.7 to 481.3)	103.2 (76.2 to 133.8)
Sub-Saharan Africa	Eastern sub-Saharan Africa	Somalia	Incidence	Age-standardized	1 (0.8 to 1.2)	0.4 (0.3 to 0.5)	1.7 (1.3 to 2)	0.9 (0.7 to 1.1)	0.6 (0.4 to 0.7)	1.4 (1.2 to 1.7)	-5.1 (-13.4 to 3.4)	47.9 (20 to 80.1)	-13 (-20.7 to -4.5)
Sub-Saharan Africa	Eastern sub-Saharan Africa	Somalia	Prevalence	All Ages	80 (66 to 99)	14 (11 to 17)	66 (53 to 85)	217 (176 to 268)	54 (43 to 67)	163 (128 to 211)	172.1 (147.8 to 196.7)	293.6 (234.4 to 368.3)	146.8 (120.3 to 173.1)
Sub-Saharan Africa	Eastern sub-Saharan Africa	Somalia	Prevalence	Age-standardized	3.5 (2.9 to 4.3)	1.1 (0.9 to 1.4)	6.3 (5 to 8.2)	3.5 (2.9 to 4.4)	1.5 (1.2 to 1.8)	6.8 (5.3 to 8.9)	1.5 (-7.1 to 10.2)	33.1 (15.1 to 55.3)	7.8 (-3 to 19.3)
Sub-Saharan Africa	Eastern sub-Saharan Africa	Somalia	Deaths	All Ages	20 (6 to 37)	3 (1 to 5)	17 (3 to 35)	42 (10 to 90)	6 (3 to 13)	36 (4 to 87)	113.3 (28.8 to 233.6)	132.3 (20.4 to 319.6)	110.5 (11.4 to 239.1)
Sub-Saharan Africa	Eastern sub-Saharan Africa	Somalia	Deaths	Age-standardized	0.9 (0.2 to 1.6)	0.2 (0.1 to 0.4)	1.6 (0.2 to 3.3)	0.7 (0.2 to 1.4)	0.2 (0.1 to 0.4)	1.4 (0.1 to 3.3)	-22 (-51.9 to 20.7)	-16.6 (-56.6 to 49.5)	-13 (-52.2 to 39.2)
Sub-Saharan Africa	Eastern sub-Saharan Africa	Somalia	DALYs	All Ages	595 (186 to 1,111)	79 (39 to 152)	517 (98 to 1,033)	1,286 (344 to 2,754)	174 (82 to 353)	1,112 (150 to 2,610)	116 (30.3 to 237.8)	121.7 (25.5 to 288.5)	115.2 (17.3 to 242.7)
Sub-Saharan Africa	Eastern sub-Saharan Africa	Somalia	DALYs	Age-standardized	20.9 (6.2 to 38.5)	5.1 (2.5 to 10.1)	39 (6.6 to 76.6)	16.7 (4.5 to 35)	4.1 (1.9 to 8.6)	34 (4.3 to 78.1)	-20.1 (-50.6 to 22.9)	-18.5 (-55.1 to 43.2)	-12.8 (-51.9 to 38.5)
Sub-Saharan Africa	Eastern sub-Saharan Africa	Somalia	YLLs	All Ages	582 (173 to 1,094)	76 (37 to 150)	506 (87 to 1,019)	1,250 (304 to 2,722)	166 (73 to 348)	1,085 (126 to 2,587)	114.8 (26.6 to 238.7)	116.9 (18.7 to 288.9)	114.5 (13.4 to 244.9)
Sub-Saharan Africa	Eastern sub-Saharan Africa	Somalia	YLLs	Age-standardized	20.4 (5.7 to 37.9)	4.9 (2.4 to 9.9)	37.9 (5.6 to 75.6)	16.2 (3.9 to 34.4)	3.9 (1.7 to 8.3)	32.9 (3.2 to 77.1)	-20.7 (-52.1 to 23.4)	-20.3 (-58 to 43.9)	-13.3 (-54.4 to 39.4)
Sub-Saharan Africa	Eastern sub-Saharan Africa	Somalia	YLDs	All Ages	13 (9 to 20)	2 (1 to 3)	11 (7 to 16)	35 (23 to 52)	9 (6 to 13)	27 (17 to 41)	169.9 (145.8 to 194.6)	292.3 (233.7 to 365.9)	145.6 (120.4 to 171.4)
Sub-Saharan Africa	Eastern sub-Saharan Africa	Somalia	YLDs	Age-standardized	0.6 (0.4 to 0.8)	0.2 (0.1 to 0.3)	1 (0.7 to 1.5)	0.6 (0.4 to 0.8)	0.2 (0.2 to 0.3)	1.1 (0.7 to 1.7)	0.2 (-8.4 to 9.4)	32.7 (14.8 to 54.4)	6.3 (-4.9 to 18.2)
[truncated: 3,686,362 more chars]
